# Composition and organization of active centromere sequences in complex genomes

**DOI:** 10.1186/1471-2164-13-324

**Published:** 2012-07-20

**Authors:** Karen E Hayden, Huntington F Willard

**Affiliations:** 1 Genome Biology Group, Duke Institute for Genome Sciences & Policy, Duke University, Durham, NC, USA; 2 Present address: Center for Biomolecular Science and Engineering, University of California, 501 Engineering 2 Building, Mailstop CBSE/ITI, UC Santa Cruz, 1156 High Street, Santa Cruz, CA, 95064, USA

**Keywords:** Centromere, Satellite DNAs, CENP-A, Centromere protein A, *Canis familiaris* (dog)

## Abstract

**Background:**

Centromeres are sites of chromosomal spindle attachment during mitosis and meiosis. While the sequence basis for centromere identity remains a subject of considerable debate, one approach is to examine the genomic organization at these active sites that are correlated with epigenetic marks of centromere function.

**Results:**

We have developed an approach to characterize both satellite and non-satellite centromeric sequences that are missing from current assemblies in complex genomes, using the dog genome as an example. Combining this genomic reference with an epigenetic dataset corresponding to sequences associated with the histone H3 variant centromere protein A (CENP-A), we identify active satellite sequence domains that appear to be both functionally and spatially distinct within the overall definition of satellite families.

**Conclusions:**

These findings establish a genomic and epigenetic foundation for exploring the functional role of centromeric sequences in the previously sequenced dog genome and provide a model for similar studies within the context of less-characterized genomes.

## Background

Centromeres are genomic sites of spindle attachment that are essential for ensuring proper chromosome segregation during cell division. Despite their recognized functional importance, centromeres are not well defined at a sequence level in most genomes [1-4]. This has greatly limited efforts to understand in detail the nature and determinants of the synergistic relationship between genome sequence and epigenetics that is generally believed to underlie centromere identity and function [5,6].

The relatively poor state of sequence assembly and annotation in centromeric regions is due to the presence and abundance of identical or near-identical satellite DNA sequences that confound attempts to generate a reliable reference sequence [2,3,7]. As a result, efforts to study the interaction of centromere proteins with the underlying genome sequence are largely incapable of distinguishing sequences that are ‘functional’ from those that are ‘non-functional’. This remains a fundamental roadblock for sequence-based studies of centromere identity, variation and function in virtually all complex genomes.

Robust genomic studies of centromeric sequences and their variation are not straightforward, as generating comprehensive and high-confidence inventories of satellite DNA families requires substantial manual curation [5,8-12]. This level of genomic resolution requires both long- and short-range sequence information capable of capturing sequence variation and spatial organization within a single satellite array, between satellites occupying distinct chromosomal domains, and within a given population [13-17]. As a result, few detailed studies have been reported to date, largely limited to well characterized and intensely studied genomes [3,5,8,18-22]. Further, satellite-rich regions are known to turn over rapidly over short evolutionary periods, thus restricting comparative efforts to closely related species [23-26]. To address the biological questions of centromere identity, evolution and function, therefore, there is a need to improve upon the current rate of sequence exploration in satellite-rich domains, thereby enabling detailed studies at the intersection of genomics and epigenetics.

The functional identification and annotation of centromeres depends on the availability of two comprehensive and complementary sequence datasets: (i) a reference sequence database that describes all centromeric sequence variation and its underlying organization, and (ii) a functional sequence database that highlights the genomic features associated with centromere identity and function. Current attempts to construct such databases have focused on centromere-associated sequences, as functional sequences can be readily identified epigenetically through association with centromere-specific proteins, such as the histone H3 variant centromere protein A (CENP-A) [27-29]. This specialized component of centromeric nucleosomes is believed to be the fundamental epigenetic mark for defining kinetochore localization and is observed at discrete sites within satellite DNA domains in many genomes [4,29,30]. Following this approach, inventories of sequences bound to CENP-A have been reported for several species to define, at least at the level of consensus sequences, the genomic content of centromeres in those genomes [11,20,24,31-33].

While adequate for identifying particular classes of satellite DNA associated with centromere function, chromatin immunoprecipitation sequencing (ChIP-seq) projects alone, however, provide only a broad view of centromere sequence organization that is largely incapable of distinguishing between sequences on different chromosomes and/or between closely related sequences, only a subset of which may be actually involved in centromere function. As an alternative and complementary approach, efforts to work from the bottom up to generate high-resolution genomic libraries of centromeric sequences have been taxed by the level and precision of experimental effort needed to derive linear sequence predictions through long spans of near-identical repeats in these regions. These limitations and difficulties notwithstanding, the value of paired genomic and epigenetic centromere datasets has been amply demonstrated in studies from human, plant, and Drosophila genomes that have been valuable for defining current models of centromere specification, identity and function [9,11,20,24,31,33]. To extend this to many other genomes that are less completely studied, however, will require new approaches to facilitate development and analysis of centromere datasets.

To address this gap in current knowledge, we present a strategy to produce a reference sequence database for satellite domains of less-characterized genomes in order to promote broader comparative studies on function and centromere sequence organization. Rather than characterizing satellite domains by extending linear maps from the assembled euchromatic chromosome arms [3,7,10,34,35], we apply a computational approach to generate a preassembled satellite sequence database, resulting in a comprehensive list of satellite domain features as well as adjacent non-satellite sequences. When paired with an equivalent epigenetic dataset of CENP-A-associated sequences, this enables one to functionally annotate satellite and non-satellite sequence variation, as well as describe the short- and long-range sequence organization associated with active centromeres.

To implement this strategy, we focus on the dog genome as an example, as it offers a high-quality whole-genome Sanger sequences (WGS) and assembly [36]. Further, unlike the situation for many other high-quality genomes, at least some information about satellite DNAs is available [37,38]. These potential advantages are balanced, however, by limitations of linear assembly across canine satellite DNA arrays and the enrichment of segmental duplications found at centric transitions [39]. Thus, the current understanding of centromeric sequence organization in the dog genome is summarized only by a small number of satellite family consensus sequences and marginal representation directly adjacent to centromere gaps [36,40,41]**.**

Here we describe an initial canine reference satellite domain database, utilizing both previously assembled and unassembled sequences, providing genome-wide descriptions of satellite families and annotation of all sequences physically linked to centromeric domains. To annotate the database, we then extracted a library of informative satellite domain sequence features that include polymorphisms and junctions with interspersed repeats found within or adjacent to satellite arrays. Finally, to relate the genomic dataset to centromere function, we then developed a complementary dataset of CENP-A-associated sequences in the dog genome and determined the census of sequence features that occupy functional centromeres. This combined genomic, epigenetic and functional approach reveals domains of satellite sequences that are not only distinguishable functionally and spatially, but also by sequence. This approach should be generally applicable to any sequenced genome, with hopes of expanding our understanding of the genomic and functional definition of centromeres in complex genomes.

## Results and discussion

**Figure 1:**
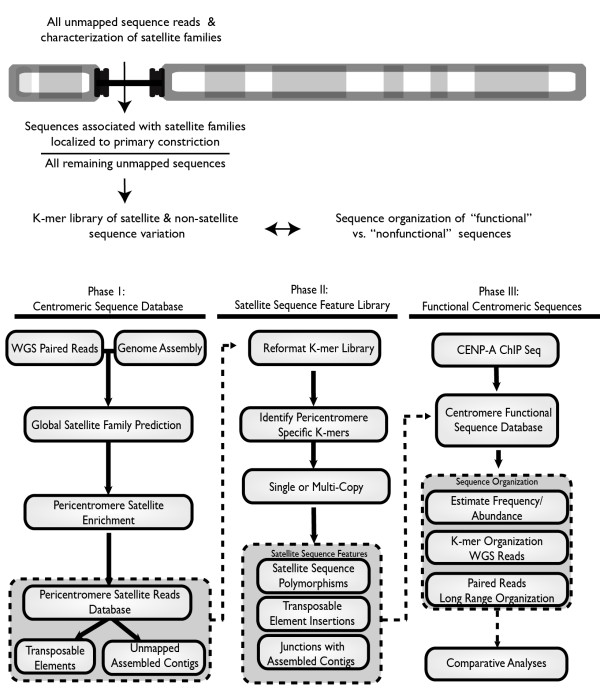
**General strategy for informatic and functional analysis of centromere satellite domains in complex genomes.** The diagram and underlying flow chart highlights three phases involved in the sequence processing and centromeric database construction. The first phase defines the sequences that are unassigned to a specific chromosome in the current genome reference assembly (all reads in that are unassembled as well as constitute the assembled unmapped contigs; or canFam2.0 chrUn). Of the tandemly repeated satellite sequence families within this database, seven were enriched in centromeric regions, resulting in an inventory of all satellites and any adjacent non-satellite sequences. Phase II reformats the read database from Phase I into a list of unique k-mers demonstrated to be specific to the pericentromere and each determined to be single-copy or multi-copy based on observed sequence frequency in the genome. These k-mers result in a library describing all inherent sequence variation in centromeric regions and are useful for investigating enrichment trends using next gen sequence datasets in Phase III, such as CENP-A ChIP sequence reads. Comparative analyses result in a list of functional k-mers that define the genomic context of the centromere. K-mers are mapped back to the read and paired read dataset to study regional sequence organization.

Our approach utilizes all sequence reads from the dog WGS project, including those that are reported in the canine genome assembly [36] and those that are missing from the assembly and fall within centromere “gaps”. The strategy consists of three phases, outlined in Figure 1: creating a database of sequence reads in and adjacent to the centromere “gaps” in the assembly, including satellite DNAs known to localize to centromeric regions (Phase I); characterizing unique variants in that database to create a library of informative sequence features (Phase II); and developing a database of functional centromere sequences and sequence features associated with CENP-A (Phase III).

### A comprehensive centromeric sequence database

Sequences in centromeric regions of complex genomes are generally of two types: those showing very limited variation, which remain unassembled and largely uncharacterized within centromeric “gaps” in the chromosome assemblies; and those adjacent to the gaps that have sufficient variation to allow standard assembly. In the current dog reference genome sequence (canFam2.0), 2,618,899 sequence reads (comprising ~7.9% of the canine genome) are currently unassigned to specific chromosomes and thus are candidates for sequences that map to the centromeric gaps. Consistent with what has been found in other complex genomes, a significant proportion of these unassembled and unassigned sequences consist of tandemly repeated satellite DNAs (Additional file 1: Figure S1; Additional file 2: Table S1, Additional file 3: Tables S2). Notably, two centromeric satellite families, Carnivore Satellite 1 (CarSat1) and/or Satellite 1 Canis Familiaris (SAT1CF) – shown previously to hybridize to primary constrictions of dog chromosomes [37,42] – are significantly enriched in the unmapped canFam2.0 scaffolds and are also enriched in regions of the genome assembly directly adjacent to the centromere gaps. These two centromere-associated satellite families account for reads containing 327.5 Mb and 212.5 Mb (for CarSat1 and SAT1CF, respectively) and thus comprise a large proportion of the sequence content of canine centromere gaps (Table 1).

**Table 1:** Summary of Canine Centromeric Sequence Database

	**Content of Satellite DNA Sequence Database (Mb) [Phase i]**			**Satellite DNA Unique k-mers (x 10**^**6**^**)**	
**Centromeric Satellite Family**	**Total satellite DNA contained in reads (7.5x coverage)**	**Total satellite DNA contained in reads (~1x coverage)**	**Other DNA contained in reads**	**Total Unique [Phase II]**	**Unique CENP-A-associated [Phase III]**
CarSat1	327.5	43.6	2.8	5.1	1.5
Sat1CF	212.5	28.3	1.4	12.9	0.9
Sat2CF	15.4	2.1	1.8	0.7	0.05
Sat3CF	14.7	2.0	0.2	1.4	0.2
CarSat2	5.9	0.8	0.3	0.3	0.05
Sat4CF	1.5	0.2	0.1	0.1	0.02
Sat6CF	1.4	0.2	0.1	0.2	0.05

Because of their prior association with centromeric regions [35,36], we used these two satellite families to nucleate the centromeric sequence database. By surveying for satellite DNAs on all assembled and unassembled sequences (see Methods), we established that the dog genome contains, in addition to CarSat1 and SAT1CF, only nine satellite families estimated to account for more than 100 kb (Additional file 2: Table S1). Notably, members of five of these satellite families can be networked to CarSat 1 and/or SAT1CF by paired-read frequency and by proximity to centromere gaps and unmapped scaffolds in canFam2.0 (Additional file 1: Figure S1, Additional file 2: Table S1, Additional file 3: Table S2, Additional file 4: Table S3, Additional file 5: Table S4).

Overall, the network of sequences that are anchored to the centromeric regions by their read linkages to CarSat1 and SAT1CF includes some 83.9 Mb (Table 1) and 720,357 reads, together accounting for 27.5% of all unassigned sequences in canFam2.0. Therefore, this approach yields a database (“Centromeric Assembly Gap Satellite Reads Database”, Figure 1, Phase I) that contains a significant number of previously unassembled and uncharacterized sequences, suitable for exploring satellite family sequence variation and for identifying potential non-satellite sequences that might also be localized to centromeric regions in this genome.

While the vast majority of sequences in this database correspond to members of the seven satellite families (Table 1), the 720,357 reads also include members of other repetitive DNA families, as well as non-repetitive DNA (as defined by RepeatMasker). About 7% of sequences in the database correspond to transposable elements embedded within reads otherwise consisting of satellite DNA (Additional file 6: Table S5). Most of the transposable element families are underrepresented in canine satellite regions relative to the rest of the genome (Additional file 7: Table S6), as expected given the nature of tandemly repeated satellite DNAs and their modes of homogenization. However, at least some transposable element families appear enriched in reads containing specific satellite DNAs. For example, CfERV1a, a canine-specific LTR family [43], was found to be 2.2-fold enriched in reads containing CarSat1 sequence (p < 0.001). Similarly, specific LINE subfamilies (L1 Canis1 and Canid) were enriched 2.5- and 2.8-fold respectively in the Sat2CF satellite family. This enrichment notwithstanding, it should be emphasized that these embedded transposable elements represent only a small proportion of the overall content of satellite DNAs in the centromeric read database, and it is unclear what role, if any, they might play in the maintenance or evolution of these regions of the genome. Nonetheless, as demonstrated for other genomes [44,45], the elements described here should be useful as genomic landmarks within satellite domains.

We also uncovered small amounts of non-repetitive sequences within the centromeric satellite read database. Within the unmapped centromeric contigs in the canFam2.0 genome assembly, 1.8 Mb correspond to contiguous sequences ≥100 bp that remain unmasked by the RepeatMasker or satellite family libraries. Using available annotation, we identified 34.7 kb of highly conserved sequences as reported by PhastCons predictions [46]. We also found 106 unmapped, centromere-linked contigs that contain sequences homologous to provisional, overlapping protein-coding and non-coding genes in organisms other than dog, taken from the reference sequences collection (RefSeq) (Additional file 5: Table S4). Many of these sequences, as expected [37], correspond to segmental duplications in the dog genome. Together, these results indicate that analysis of satellite DNA families, once annotated in a genomic context, can provide information on the sequence content and description of previously unmapped regions of the genome.

### A sequence feature library for satellite domains

To explore sequence variation within the centromeric read database and as a prerequisite for searching for specific sequence features that distinguish functional centromeres from related (but non-functional) sequences, we reformatted the entire canine unmapped database and the remaining unassigned “gap” reads into a library of k-mers (see Methods) (Figure 1, Phase II). While enrichment can be detected over a range of k-mer values, we have used k-mers 50 bp in length for all analyses described here, as this length maximizes the sequence-based information within our enrichment search while limiting edge effects observed when mapping exact matches in our short-read ChIP-seq database (see next section). The library of 50-mers represents the frequency of all sequence signatures found within the reference database and allows one to annotate high- and low-frequency events, insertions and/or deletions within the highly repetitive sequences that dominate centromeric regions [47]. We excluded 50-mers that also mapped to identical sequences in the canFam2.0 genome assembly itself, leaving ~20.7 million different 50-mers that are specific to the unassembled gap regions of the genome (Table 1). By normalizing to single-copy 50-mer depth estimates (see Methods), we could use this library to predict the abundance of individual sites within satellite domains, thus defining satellite sequence polymorphisms, transposable element insertions, and boundaries between satellite and non-satellite sequences within these regions.

### Identifying sequence features of functional centromeres

**Figure 2:**
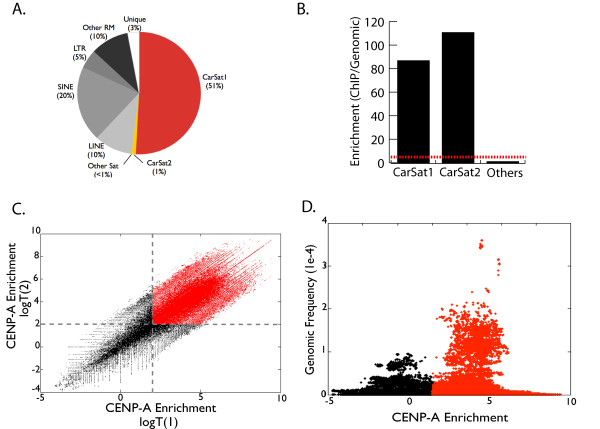
**Characterizing functional satellite sequence features. Centromere sequence features associated with CENP-A ChIP sequences.** (**A**) Reads were initially mapped to canFam2.0 and characterized relative to sequence classification, as indicated in pie graph. (**B**) Both CarSat1 and CarSat2 are highly enriched in the CENP-A ChIPseq dataset (p < 0.01) relative to genomic background estimates (as demonstrated by red dotted line). Other satellite families showed no evidence of enrichment and are combined into one data point. (**C**) CarSat satellite families (CarSat1 and CarSat2) show enrichment of select sequences in the CENP-A ChIP dataset on an xy-plot of two replicate enrichment estimates (log transformed relative enrichment scores), highlighting in red in the upper right quadrant those k-mers that are enriched in both comparisons as delineated with grey dotted lines. (**D**) CarSat k-mers that are enriched (red) compared to those that are not enriched (black), as a function of their observed frequency in the genome. Both high-copy and low-copy number k-mers are enriched in both satellite families.

In the third phase of the strategy, we generated a library of sequences associated with canine CENP-A-containing nucleosomes, providing a functional context for the satellite DNA domain database (Figure 1, Phase III). To identify all sequences associated with CENP-A in canine cells, we performed CENP-A ChIP-seq, generating 34.6 million 72 bp Illumina sequence reads (see Methods) (Figure 2A).

Using these sequences, we surveyed the complete canine WGS database to identify sites of enrichment throughout both the current chromosome reference assembly, as well as the unmapped regions of the dog genome. Based on non-repetitive (as defined by the absence of RepeatMasker annotation) alignments to canFam2.0 using standard ChIP-seq mapping (bwa) and enrichment detection software (QuEST), we found no evidence for significant CENP-A enrichment outside of the centromeric regions (see Methods) [48,49].

Next, to identify CENP-A-associated sequences within the unmapped gap regions, we compared the ChIP-seq reads to the unmapped 50-mer-based sequence feature set from Phase II. From this analysis, we identified 406,487 WGS reads that align with CENP-A ChIP-seq reads; notably, 70.5% of these WGS reads are associated with the CarSat1 and/or CarSat2 satellite families (together, CarSat1/2), indicating significant enrichment (Figure 2B). CarSat1 and CarSat 2 are related satellite families, of lengths 738 bp and 1466 bp, respectively (see Methods). Smaller enrichment sets were identified for several other satellite families; however, in sharp contrast to the CarSat1/2 datasets, these reads constitute <2% of the total number of reads that define each respective satellite family and thus are of uncertain significance (data not shown).

To further subdivide the CarSat1/2 sequences, we focused on the 50-mers that are found in the ChIP-seq database (Table 1) and used these to distinguish specific sequence features of CENP-A-associated versus non-associated copies of CarSat1/2 satellite repeats. By this analysis, 60% of CarSat1/2 reads contain a minimum threshold of continuous bases of 50-mers that were >2-fold enriched (Figure 2C), while 40% of reads contained no such enriched sequences. This supports the hypothesis that only particular CarSat1/2 sequences are interacting with CENP-A, suggesting the presence of definable subtypes within the overall satellite domains at canine centromeres.

The majority of enriched 50-mers appear to be multi-copy, high-frequency satellite sequences in the CarSat1/2 arrays, thereby providing evidence for functional CENP-A domains that are predominantly found associated with near-identical satellite repeat units (Figure 2D). Although not all multi-copy sites within the arrays are enriched, it is clear that high-frequency 50-mers (those represented greater than an estimated 1000 copies) are most likely to be associated with CENP-A. This may indicate that CENP-A is associated preferentially with specific, highly conserved positions within the majority of monomer units at the centromere, reflecting the sequence homogenization that is a common feature of satellite arrays [3,5,16]. Alternatively, the CarSat1/2 satellite families might be divided into distinct monomer types that are associated or not associated with CENP-A, suggesting the existence of different subtypes or subfamilies around the genome, similar to what is observed, for example, in primate alpha satellite [50,51].

In Phases I and II of this strategy, we defined various non-satellite sequences embedded within satellite domains (see above). To determine if any of these sequences are enriched for CENP-A, we focused on 50-mers that provide junction information between satellite repeats and these non-satellite sequences. Notably, only transposable element junctions embedded in CarSat1/2 arrays appear to be enriched among CENP-A-associated 50-mers (Additional file 1: Figure S3). Most of the detected CENP-A enrichment was observed at transposable element sequence junctions predicted (on the basis of 50-mer frequency, as above) to be single-copy within the domain; only LINE elements in the CarSat1 array appear to have an enrichment signature associated with homogenized repeat units (Additional file 1: Figure S3). However, we caution against any functional inference, since these enrichment patterns could simply reflect the presence of CENP-A over a region of the CarSat1 array that happens to contain amplified LINE sequence in the particular canine genome tested here.

When we mapped CENP-A-enriched 50-mers, we also found enrichment over 323 kb of centromere-linked contigs identified in Phase I. The majority of these sequences appear both by alignment and 50-mer frequency to be multi-copy. It is notable, however, that we find overlap with sites of conserved sequence elements identified within the centromeric read database. While these may correspond to segmental duplications in the region [37], their significance remains to be determined.

### Investigating the unassembled sequences for functional centromere sequence variation and organization

**Figure 3:**
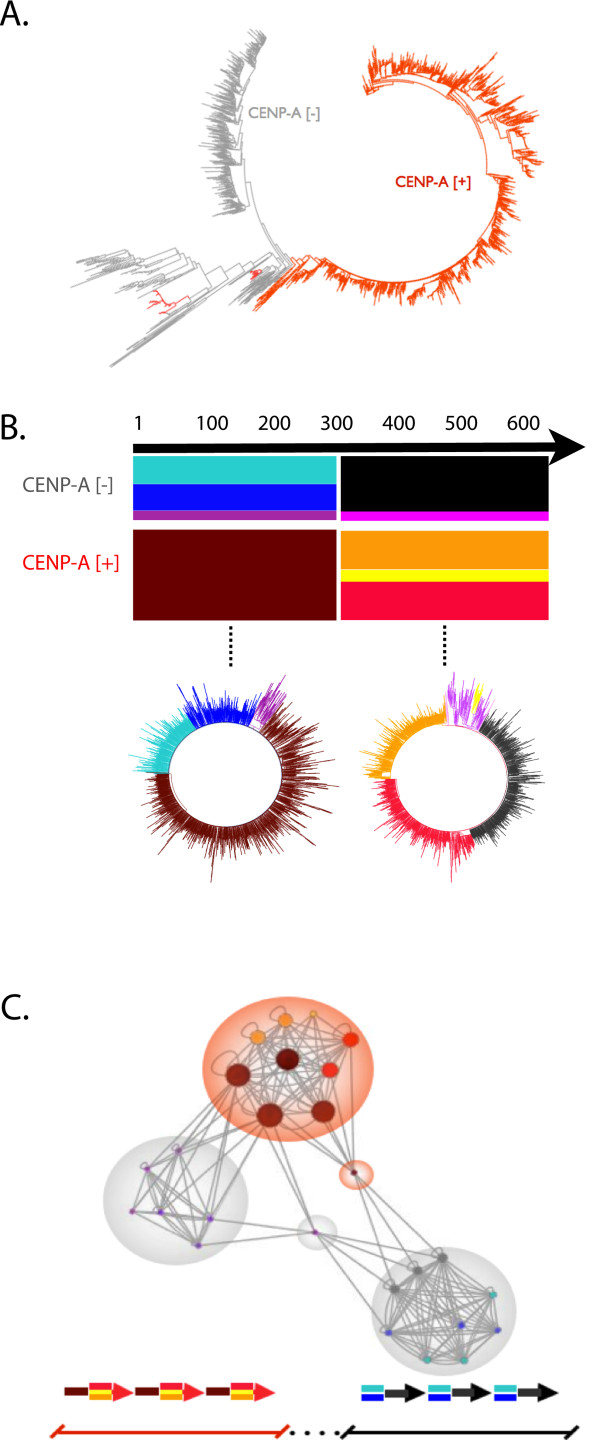
**CarSat satellite family contains functional sequence subtypes.** (**A**) Phylogenetic analysis of reads containing a full-length CarSat1 monomer illustrate largely distinct clades of reads associated with CENP-A (CENP-A[+]; red) or not associated with CENP-A (CENP-A[−]; gray). (**B**) The subset of reads containing full-length monomers was further characterized by sliding window 200 bp clustering approach (see Methods) and assigned to distinct sequence subgroups, as indicated by different colors. CENP-A[−] reads are highly similar in the 3’ end of the monomer but divide into definable major subgroups in the 5’ end; CENP-A[+] reads appear to have the inverted similarity pattern. Phylogenetic analysis of the 5’ end of CarSat1 reads shows distinct clades that distinguish CENP-A[+] from CENP-A[−] sequences. A similar analysis of the 3’ end of CarSat1 reads. Overall, [+] and [−] reads could be classified into four predominant monomer types, shown as turquoise-black and blue-black for CENP-A[−], and maroon-red and maroon-yellow for CENP-A[+]. There are smaller subfamilies, one in CENP-A[−] (pink-purple) and one in CENP-A[+] (maroon-yellow) that are far less abundant and appear to clade together. (**C**) Paired read frequency patterns between monomer cluster types predict that the CENP-A-containing satellites (CENP-A[+]] are spatially distinct from the non-CENP-A-containing satellites (CENP-A[−]) at dog centromeres. Relative node sizes represent read depth for each of the 200 bp windows, while lines represent a minimum threshold for paired-read connectivity. Three sequence groups are identified: CENP-A[+] array, highlighted in red, and two CENP-A[−] arrays in grey. CENP-A[−] arrays can be further divided into two groups, both minimally connected to CENP-A[+] domain through transitional monomer clusters. Model of predicted genomic organization at dog centromeres, indicating the two major types (CENP-A [+] and [−]) and predicted transition monomers at bottom.

To further study sequence organization of centromere sequence features, we investigated enrichment patterns in the WGS reads comprising the reference satellite read database (Phase I). Focusing on the most abundant CarSat1 family, we divided the CarSat1 read database into those that contain CENP-A-associated k-mers (CENP-A[+]) and those that lack entirely any association with CENP-A (CENP-A[−]). Notably, the full-length CENP-A[+] CarSat1 monomers are phylogenetically distinct from CENP-A[−] monomers (Figure 3A). Therefore, CENP-A appears to be associated largely with a distinct subset of satellite sequences, suggesting that the functional component of the array can be delineated by local sequence variation.

To extend this observation, we clustered reads and focused on sequence patterns that distinguish the functionally distinct subtypes. We reformatted CarSat1 reads to evaluate sequence variation within overlapping 200 bp windows (with a 100 bp slide, Additional file 1: Figure S4). This revealed six major monomer types, three associated with CENP-A and three not (Figure 3B). Different regions within these monomer types showed different patterns, with significant sequence differences between the left and right halves of the monomer (Figure 3B) (Additional file 1: Figure S4). Interestingly, these sequence groups could be readily distinguished by phylogenetic analysis (Figure 3B).

While the above analysis distinguishes subfamilies of CarSat1 monomers by sequence and functional attributes, it does not reveal how those subfamilies are organized in a genomic context. To address this and to study the long-range, regional organization of CENP-A-enriched sequences, we next investigated paired-read frequencies between the predicted clusters. High levels of ‘self-pairing’ between clusters of CENP-A[+] monomers or CENP-A[−] monomers provides evidence for homogenized functional satellite domains, suggesting that these reads are not only similar in sequence but are also spatially close to one another in the genome, with only limited spatial proximity between the CENP-A[+] and CENP-A[−] domains (Figure 3C). As expected, there is also evidence for a limited number of ‘intermediate’ monomers – CENP-A[+] monomers that mate-pair with CENP-A[−] monomers. Overall, these data are consistent with models that indicate that CENP-A-associated centromere sequences are clustered within centromeric domains in both human and other genomes [52].

## Conclusions

We have presented here a generally applicable strategy, outlined in Figure 1, to construct a comprehensive database of all sequences that occupy centromeric satellite DNA domains in less characterized genomes with localized centromeres. This strategy provides a comprehensive description of satellite sequence variation and organization, revealing in addition both embedded transposable element insertion sites and adjacent non-satellite sequences that are often missing from current genome assemblies. Global satellite sequence inventories -- defined both by WGS read characterization and by k-mer libraries of satellite sequence signature features – provide a platform to explore sequence variation within these domains, which has been masked previously by collapsed assembly efforts.

The ability to characterize the relative abundance and frequency of each genomic feature associated with centromere regions should promote studies to expand functionally annotated mapping efforts in these domains, as well as provide resources for exploring sequence evolution. Comprehensive databases of this sort could serve as a “reference database” for centromere regions in complex genomes, replacing the featureless gaps that exist now and providing features of satellite sequence and organization that can be used to explore trends in genome biology and function.

The results here provide an initial sequence definition of canine centromeres, while presenting a complete genomic reference database to further studies aimed to address centromere plasticity. It remains to be tested, for example, how stable CENP-A sequence enrichment patterns are within the context of the same cell type, among different cell types of the same individual, or among different individuals or related species. Such questions can now be addressed using not only the broad classification of functional repeat subfamilies, but also extending such analyses to additional centromere sequence features, including precise single-copy junctions and rare sequence polymorphisms within these centromeric domains.

## Methods

### Satellite network database construction

All 31.5 million WGS reads (~7.5-fold coverage) and 2.385 Gb of assembled sequences (canFam2.0) for the domestic dog (*Canis familiaris*; female boxer) were downloaded from the published sequencing project [36]. Previously described canine satellite DNA families were obtained from RepBase (version 15.10) [43] and GenBank (AY339973-80) [41]. We identified all remaining, previously uncharacterized satellite DNAs using Tandem Repeat Finder [53] (match probability = 75, match indel = 20; maximum period size (2000 bp), with match, mismatch, and delta of 2, 7, 7 respectively) on the assembled canFam2.0 genome after removing all sites defined by RepeatMasker (http://repeatmasker. genome.washington.edu). Identified tandem repeat consensus sequences were then clustered to provide a non-redundant list of 185 satellite families, using pairwise alignments and grouping those satellite sequences that overlapped with >95% identity for a minimum of 100 bp. Reads containing satellite DNA were identified using RepeatMasker, using both complete (RepBase 15.10) and canine-specific satellite libraries to define sequences that corresponded to non-satellite repeats, to satellite DNA, and to potentially unique, non-repetitive sequences. Satellite sequence coverage (in the context of the overall 7.5x WGS sequence coverage) was used to estimate array sizes. Similarly, the downloaded canFam2.0 sequences (including chromosome Un, chrUn) were screened by RepeatMasker with the comprehensive canine satellite library to report both location and abundance of the respective families. Visualization of satellite family locations in canFam2.0 was illustrated using Circos software package [54].

Centromeric satellite enrichment was determined by evaluating the -fold base pair enrichment of each satellite family in the 2 Mb directly adjacent to the centromere clone gap (defined as the end of the chromosomes for all autosomes; and 2 Mb on both arms adjacent to the X centromeric gap) relative to all remaining non-centromeric 2 Mb windows of the canFam2.0 genome (omitting chrUn). Paired-read frequencies were reported for the sequences containing satellite families localized to centromeric regions, providing information on the number of paired reads with intra- or inter-satellite sequence representation and supplementing the existing database with paired reads containing centromere gap linked, but non-satellite DNA. All non-satellite, non-RepeatMasked high-quality sequences ≥100 bp were aligned using a Burrows-Wheeler Aligner for designed for long reads (bwa-sw) [49,55], to the canFam2.0 assembly to identify all assembled pericentromere-linked assembled chrUn contigs. Unmapped contig annotations were obtained from the UCSC browser [56].

The centromeric satellite sequence reads were reformatted as k-mers, with a 50 bp window, 1 bp slide thereby maximizing the linear information of satellite informative bases to report both sequence and frequency in the pericentromere satellite reads database in the context of short-read (72-bp) ChIP-seq data. For this study, 50-mers proved to be most informative, as they provided the maximum linear sequence information to reference the short-read ChIP-seq library with minimum edge effects. K-mers not specific to the centromeric database, as demonstrated by an exact match to reads outside of the defined read database, were eliminated. Additionally, all centromere-linked assembled contigs were reformatted to 50-mers and evaluated against all non-centromere-linked reads. To identify single-copy and multi-copy 50-mers, sequence frequencies were compared between each pericentromere satellite sequence feature and a list of 50-mers collected from both simulated single-copy 50-mers with 7.5x read coverage and observed 50-mer frequencies from the single-copy canine *XIST* locus (canFam2 chrX:60374223–60411096). Multi-copy sites were defined as those >2 standard deviations from the single-copy mean.

### Tissue culture

The Madin-Darby canine cell line (MDCK; ATCC CCL-34) is derived from a kidney of a normal adult female cocker spaniel. Cells were cultured in Eagle’s minimum essential medium with 2 mM L-glutamine and Earle’s BSS (MEME, Sigma 4655) adjusted to contain 1.5 g/litre sodium bicarbonate (Gibco 25080–094), 0.1 mM non-essential amino acids (Gibco 11140–050), 1.0 mM sodium pyruvate (Gibco 11360–070 90%), fetal bovine serum 10% (Hyclone SH30071.03) and 1% (v/v) penicillin and streptomycin, and were grown at 37 °C in a 5% CO_2_ environment.

### Fluorescence *in situ* hybridization

Preparation of mitotic chromosomes was carried out using standard methods [57]. Exponentially growing MDCK cells were obtained after a 1–2 hr colcemid/karyomax (Gibco) treatment followed by 10-min incubation in a hypotonic solution (equal volume 0.0075 M KCl, 0.8% NaCitrate, and dH_2_O) and dropped in high humidity. Slides were rehydrated by immersion in a 2x SSC, at 37 °C, followed by EtOH dehydration cycle. Chromosomes were denatured briefly (70% Formamide, 2x SSC at 72 °C) before repeating EtOH cycle. CarSat1 and SAT1CF satellite nick-translated probes were produced as satellite amplicon sequences (CarSat1: AACCTTTCCCTGCCACTAAC/CTCACCCTCAGTCCTTCACA; Sat1CF: GAACAAAGTCACCAGGACTG/CCTGGACATGATGACAGTGG), and hybridized for 16–18 hrs at 60 °C, corresponding to high stringency. Post-hybridization washes consisted of two 8-min washes in 50% formamide/2x SSC (pH 7.0) at 42 °C, followed by one 8-min wash in 2x SSC at 37 °C. Slides were briefly rinsed in reagent-grade water before being counterstained with 4,6-diaminidino-2-phenylindole in Vectashield (Vector Laboratories). Slides were analyzed under a Zeiss Axiovert 200 M microscope fitted with a Hamamatsu ORCA-ER camera. Images were captured with OpenLab (Improvision) and processed with Adobe Photoshop.

### CENP-A immunoblotting and immunolocalization

To avoid potential pitfalls associated with overexpression of tagged CENP-A constructs, we demonstrated that endogenous canine CENP-A protein could be readily detected by both western blot immunoassay and by indirect immunofluorescence localization by a primary antibody (Additional file 1: Figures S1, S2). Whole-cell protein samples (10^7^ cells resuspended in 3x protein sample buffer; 2x Laemmli buffer with 15% BME) were prepared from MDCK cells. Proteins were separated (40 min, 200 V, 0.08A) using BioRad’s precast gels and MiniProtean set-up (BioRad Ready Gel Tris–HCl Gel, 12% resolving gel, 4% stacking gel; 161–1102), using 10 μl of the Kaleidoscope marker (BioRad 161 0324). Standard buffers were prepared for both running (10X; Tris-Cl (30 g); Glycine (144 g); SDS (10 g)) and transfer buffer (10x; Tris-Cl (30 g); Glycine (144 g)). Gel was equilibrated in transfer buffer for 10 min before transferring to PVDF membrane (Biorad) at 30 V, 4 centigrade (C) for 18 hrs. Transferred membrane was washed in 0.1% PBS-Tween for 20 minutes. CENP-A was detected by incubation in 5% non-fat dairy milk (NFDM) with a 1:500 dilution of mouse anti-CENP-A monoclonal antibody designed for human CENP-A (a.a. 3–19); (Stressgen; KAM-CC006). Membrane was washed in 0.1% PBS-Tween for 20 min before incubation in 5% NFDM with a 1:2000 dilution of the secondary antibody for 30 min, followed by washing in 0.1% PBS-Tween for 20 min. Immunodetection was reported with exposure time of 3 minutes, as described in ECL protocol (Amersham).

Immunostaining on metaphase chromosomes was carried out using minor modifications to procedures described previously [57]. Slides were prepared by cytospinning (10 min, 1900 rpm) and fixed in 4% formaldehyde-PBS-triton (0.1%) solution for 10 min. Slides were then washed twice in PBS for two minutes before the addition of antibodies. Slides were blocked in 3% BSA-PBS-tween (0.1%; 60 min), followed by a PBS wash. To detect centromeric regions, we incubated slides with 1:100 dilution of primary mouse anti-human (a.a. 3–19) centromere protein A (CENP-A) monoclonal antibody (Stressgen; KAM-CC006), and a 1:200 dilution of secondary antibody anti-mouse IgG (Jackson Laboratories Cat. No. 711-165-152) in 1% BSA-PBS-Tween (0.1%) solution.

### CENP-A ChIP-seq analysis

Native chromatin immunoprecipitation (N-ChIP) analysis was performed as described [58], using moderate salt buffers (300 mM NaCl) previously shown to be adequate for CENP-A immunopurification [59]. Chromatin was prepared by micrococcal nuclease (30U; Worthington) digestion of MDCK cell nuclei to predominantly mono- and di- nucleosomes (Additional file 1: Figure S5). Immunoprecipitation was carried out using 5 micrograms of antibodies against human CENP-A (Stressgen; mouse monoclonal), and normal mouse IgG (Upstate) to control for non-specific binding. One-tenth of starting material was reserved as input DNA control. After extraction with phenol/chloroform and precipitated with ethanol, immunoprecipitated DNA was resuspended in 10 mM Tris/1 mM EDTA, pH 8.0, supplemented with 10 μg/ml RNase A. Sequencing was performed at the Duke IGSP Genome Sequencing and Analysis Core Facility (Illumina GAII, 72 bp single-end reads; 34.6 million reads with library fragments of ~250 bp (insert plus adaptor and ChIP sequences)).

Canine CENP-A ChIP-seq reads were aligned to the assembled canFam2.0 genome using Burrows-Wheeler Aligner (bwa) [49]. Relative enrichment values were determined against a genomic background simulated dataset provided by random draws from the WGS database (with estimates based on ten independent replicates). This simulation assumes a uniform recovery of chromatin in the IP sample. While we have not systematically explored the potential bias of micrococcal nuclease digestion in our protocol, we have limited our study to address satellite family enrichment, in which context we believe that such a simulated dataset provides a conservative basis for estimating enrichment. Enrichment peaks were identified using the QuEST software package [48]. Genomic coordinates of enriched domains with canFam2.0 annotation allowed for those sites that overlap with specific satellite families, as well as potential sites of non-repetitive regions, to be identified. CENP-A-enriched centromeric sequence features (50-mer database) were determined by taking the log transformed normalized ratio of the frequency within the CENP-A relative to the genomic database. Assignment of identical matches to CENP-A-enriched 50-mers was determined and mapped in both assembled contigs and unassembled reads.

### Sequence analysis of CarSat monomers

CarSat (CarSat1 and CarSat2) monomer repeat unit consensus sequences are defined as GC-rich sequences (previously estimated at 51%), with no detectable internal direct or inverted repeat structure [38]. Surveys of repeat unit length within the canFam2.0 assembly provided evidence for satellite monomer lengths (CarSat1: 738 bp; CarSat2: 1466 bp) that are larger than expected within the average WGS read; therefore, complete units are rarely observed. To evaluate each sequence, we reformatted each read into 200 bp windows (100 bp overlap) standardized to each respective consensus sequence. Pairwise alignments of all sequences represented in each 200 bp-window (MUSCLE) [60] were used to perform unsupervised clustering predictions. K-means clustering (MATLAB, 2009b, The MathWorks; squared euclidean distance measure) was implemented for a range of k clusters (k = 2-20). The optimal “k” was determined as the highest average measure of cluster proximity, or mean silhouette values (MATLAB, silhouette plot). Phylogenetic trees were constructed using the PHYLIP 3.65 package (http://evolution.genetics.washington.edu/phylip.html). A DNA distance matrix was calculated using the F84 method, and trees were constructed by UPGMA (Unweighted Pair Group Method with Arithmetic Mean) and neighbor-joining methods [60,61]. Bootstrap replicates (100) were performed to assess internal support for nodes.

## Additional files

The following additional data are available with the online version of this paper:Six supplemental tables are provided as follows: Supplemental Table 1 is a table listing global satellite descriptions and relative abundance and location in the canFam2.0 assembly. Supplemental Table 2 is the bed file of 11 satellites families mapped to canFam2.0 assembly. Supplemental Table 3 provides the paired read data for abundant satellite families. Supplemental Table 4 provides a list of all centromeric assembled contig and relevant annotations. Supplemental Table 5 lists the repeat content within the centromeric satellite domain. Supplemental Table 6 lists all estimated enrichment of non-satellite repeats associated with canine centromeric satellite families.Five supplemental figures are provided as follows: Supplemental Figure 1 describes the characterization of canine centromeric satellite families. Supplemental Figure 2 provides the CENP-A antibody immunoblotting results. Supplemental Figure 3 provides enrichment information for transposable element junctions in CarSat1. Supplemental Figure 4 provides k-means clustering information. Supplemental Figure 5 provides evidence for mono- and dinucleosomes in the MNase-digested chromatin used for the ChIP-seq experiments.Sequencing data used in our analysis are available through GEO Accession number GSE38079.

## Abbreviations

CENP-A, (centromere protein A); CarSat, (Carnivore Satellites 1,2); SAT1CF, (Satellite family 1, Canis familiaris); CFA, (Canis familiaris); HSA, (Homo sapiens); WGS, (whole-genome sequence).

## Competing interests

The authors have no competing interests.

## Authors’ contributions

Experimental, genome informatics, and data analysis/presentation were performed by KEH. Study design, manuscript preparation and figure design were carried out by KEH and HFW. Both authors read and approved the final manuscript.

## Funding

Supported by funds from the Duke Institute for Genome Sciences & Policy and from the Howard Hughes Medical Institute Professors Program.

## Supplementary Material

Additional file 1 **Figure S1. Characterization of canine pericentromeric satellite families.** (a) Locations of the eleven largest satellite families in the assembly are highlighted relative to 39 canine chromosomes, using the color code indicated in the figure. Each tile represents 10 kb of satellite sequence. Pericentromeric regions (defined as 2 Mb proximal to each centromere gap) are shown in gray. Open arrowheads indicating sites of pericentromere satellite enrichment, closed arrowheads indicate sites of CarSat1 and/or Sat1CF enrichment. (b) Satellite families in pericentromeric regions of the assembly are extensively represented in unmapped contigs (chrUn). Each tile equals a 100 kb bin of satellite sequence. (c) CarSat1 (red signals) and Sat1CF (blue signals) sequence hybridization to canine (MDCK) chromosome spreads show primary pericentromeric localization of both satellite families. Overlap of the two colors at some centromeres appears as a white signal. Two chromosomes (the X chromosomes, indicated by arrows) do not contain detectable CarSat1 or Sat1CF. (d) The physical sequence distance, or relative frequency of paired-reads connections, between the eleven largest satellite families are indicated, using the color code indicated in the figure. Size of each ball corresponds to the relative representation of each family in the genome. Lines represent at least 10 paired reads; bold lines represent >1000 paired reads. Additional file 1: Figure S2: CENP-A antibody to MDCK cells. Canine CENP-A was detected using mouse anti-centromere protein A (CENP-A) monoclonal antibody designed for human CENP-A (a.a. 3–19); (Stressgen; KAM-CC006) by immunoblotting (a), with canine CENP-A (XP_532899.2; ~16kD) shown relative to human CENP-A (NP_001800; ~17kD) compared to loading controls. CENP-A antibody is shown by immunofluorescence (FITC/green) to localize to dog (MDCK) centromeres and colocalize with centromeric satellite family CarSat1 (RHOD/red) (b). Figure S3: Identifying enrichment patterns in satellite transposable element junctions in CarSat1 satellite families. Relative enrichment scores of satellite-transposable element junction sequences are shown in a xy plot from two comparisons with genomic background. Those enrichment patterns that fall below log transformed enrichment value of 2 are shown in shaded box. Remaining single copy (shown as stars) and multi-copy (boxes) transposable element junctions for SINE (red), LINE (blue), and LTR (black) are provided. Additional file 1: Figure S4. Read Subtype assignments by k-means clustering of 200 bp sliding window. All CarSat1 reads reformatted relative to identified consensus sequence (737 bp; as determined from consensus bases from all assembled CarSat1 monomers (canFam2.0)). Reads were further divided into six 200 bp windows with 100 bp overlap/slide. Sequence windows were assigned to clusters using k-means (see Methods) and reads were relabeled as ordered clusters and sorted accordingly. Reads containing minimally four windows are shown above; demonstrating the clustering subgroups defined in paper Figure 3. Additional file 1: Figure S5. MNase digestion for Chromatin IP protocol, demonstrating that mono- and di- nucleosomes are enriched within this study. Lane 1 contains size markers, with appropriate bands (bp) and predicted sites of nucleosome-sized DNA indicated. Lane 2 contains MNase-digested input DNA used in this study. Click here for file

Additional file 2 Table S1. Global satellite descriptions and relative abundance and location in the canFam2.0 assembly. Click here for file

Additional file 3 **Table S2. Satellite genomic distribution assignments in the canFam2.0 assembly.** Column header information is defined as follows: chr, CanFam2.0 chromosome; chrS, chromosome start position; chrE, chromosome end position; bp_span, the length of the repeat unit (chrE-chrS); satellite name, the canine satellite name either assigned by RepBase, GenBank, or this study; tile_color, color assignments for each family as illustrate in Circos image (Additional file1: Figure S1a,b); type, either pericentromeric, or located within a 2 Mb window of a chromosome centromere gap, or ‘na’ if found within the chromosome arms or and unmapped assembled contig (chrUn). Click here for file

Additional file 4 Table S3. Paired read data between abundant (estimated ≥100 kb) satellite families. Click here for file

Additional file 5 Table S4. Annotation of centromeric associated unmapped contigs. Click here for file

Additional file 6 **Table S5. Distribution of centromeric transposable elements.** Repeat element representation for each centromeric satellite family, describing relative proportions of each repeat family and overall contribution to array. (PDF 37 kb) Click here for file

Additional file 7 Table S6. Centromeric satellite family repeat class enrichment estimates. Click here for file
