# Supplementary material for: Composition and organization of active centromere sequences in complex genomes
Source: BMC Genomics. 2012 Jul 20;13:324. doi: 10.1186/1471-2164-13-324 (PMC3422206; doi:10.1186/1471-2164-13-324)
Supplement: Additional file 1 — Figure S1. Characterization of canine pericentromeric satellite families. (a) Locations of the eleven largest satellite families in the assembly are highlighted relative to 39 canine chromosomes, using the color code indicated in the figure. Each tile represents 10 kb of satellite sequence. Pericentromeric regions (defined as 2 Mb proximal to each centromere gap) are shown in gray. Open arrowheads indicating sites of pericentromere satellite enrichment, closed arrowheads indicate sites of CarSat1 and/or Sat1CF enrichment. (b) Satellite families in pericentromeric regions of the assembly are extensively represented in unmapped contigs (chrUn). Each tile equals a 100 kb bin of satellite sequence. (c) CarSat1 (red signals) and Sat1CF (blue signals) sequence hybridization to canine (MDCK) chromosome spreads show primary pericentromeric localization of both satellite families. Overlap of the two colors at some centromeres appears as a white signal. Two chromosomes (the X chromosomes, indicated by arrows) do not contain detectable CarSat1 or Sat1CF. (d) The physical sequence distance, or relative frequency of paired-reads connections, between the eleven largest satellite families are indicated, using the color code indicated in the figure. Size of each ball corresponds to the relative representation of each family in the genome. Lines represent at least 10 paired reads; bold lines represent >1000 paired reads. Additional file 1: Figure S2: CENP-A antibody to MDCK cells. Canine CENP-A was detected using mouse anti-centromere protein A (CENP-A) monoclonal antibody designed for human CENP-A (a.a. 3–19); (Stressgen; KAM-CC006) by immunoblotting (a), with canine CENP-A (XP_532899.2; ~16kD) shown relative to human CENP-A (NP_001800; ~17kD) compared to loading controls. CENP-A antibody is shown by immunofluorescence (FITC/green) to localize to dog (MDCK) centromeres and colocalize with centromeric satellite family CarSat1 (RHOD/red) (b). Figure S3: Identifying enrichment pattern [file 1471-2164-13-324-S1.docx]

**Supplemental Figures and Table Legends**

**Composition and organization of active centromere sequences**

**in complex genomes**

**Karen E. Hayden^1@^ and Huntington F. Willard^1^**

^1^ Genome Biology Group, Duke Institute for Genome Sciences & Policy, Duke University, Durham, North Carolina, United States of America

^@^ Present address: Center for Biomolecular Science and Engineering, University of California, Santa Cruz, CA 95064, USA.

Karen E. Hayden (karen.hayden@soe.ucsc.edu)

Huntington F. Willard (hunt.willard@duke.edu)

**
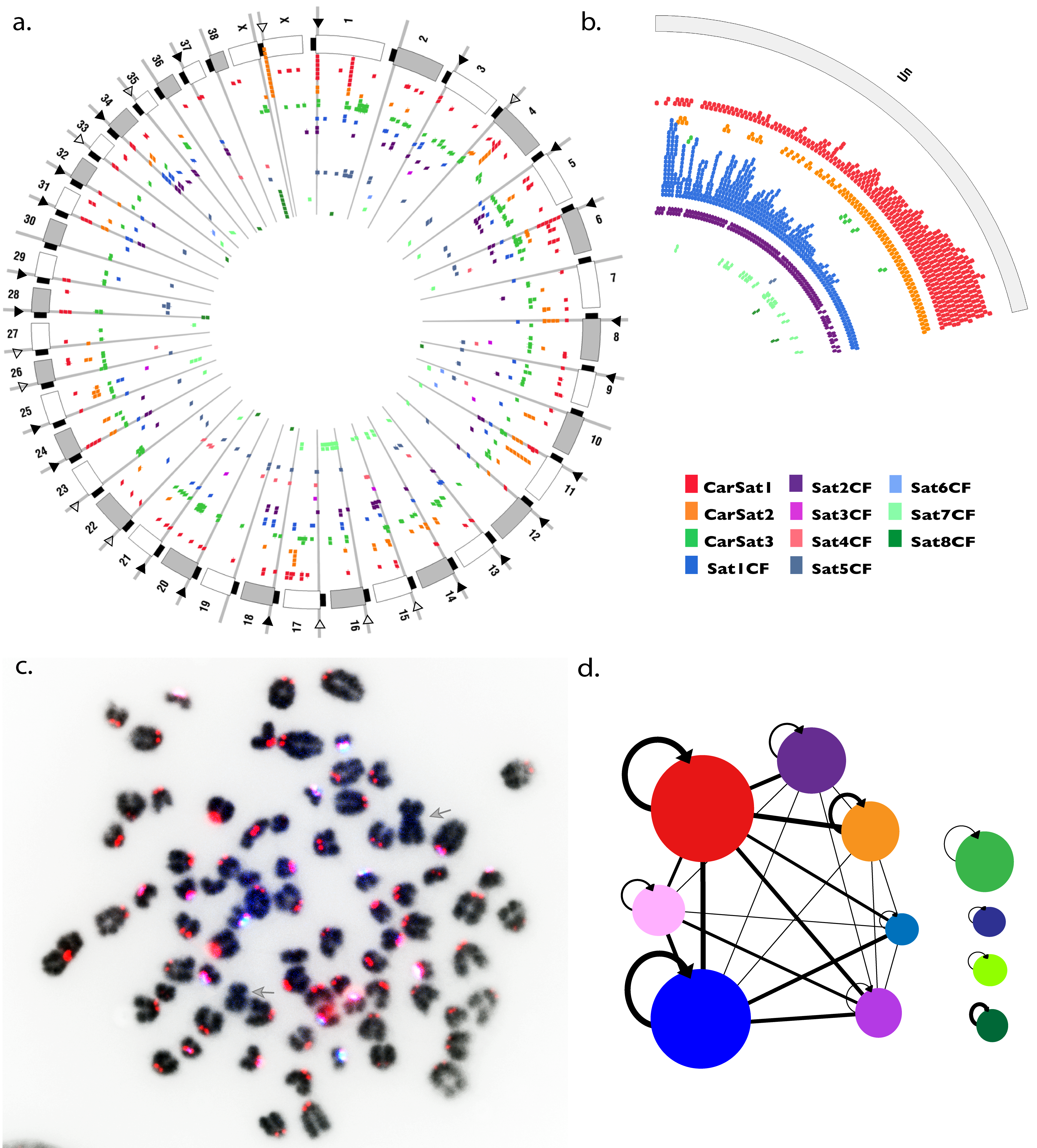
**

**Supplemental Figure 1: Characterization of canine pericentromeric satellite families.**

**(a)** Locations of the eleven largest satellite families in the assembly are highlighted relative to 39 canine chromosomes, using the color code indicated in the figure. Each tile represents 10 kb of satellite sequence. Pericentromeric regions (defined as 2Mb proximal to each centromere gap) are shown in gray. Open arrowheads indicating sites of pericentromere satellite enrichment, closed arrowheads indicate sites of CarSat1 and/or Sat1CF enrichment. **(b)** Satellite families in pericentromeric regions of the assembly are extensively represented in unmapped contigs (chrUn). Each tile equals a 100 kb bin of satellite sequence. **(c)** CarSat1 (red signals) and Sat1CF (blue signals) sequence hybridization to canine (MDCK) chromosome spreads show primary pericentromeric localization of both satellite families. Overlap of the two colors at some centromeres appears as a white signal. Two chromosomes (the X chromosomes, indicated by arrows) do not contain detectable CarSat1 or Sat1CF. **(d)** The physical sequence distance, or relative frequency of paired-reads connections, between the eleven largest satellite families are indicated, using the color code indicated in the figure. Size of each ball corresponds to the relative representation of each family in the genome. Lines represent at least 10 paired reads; bold lines represent >1000 paired reads.

**
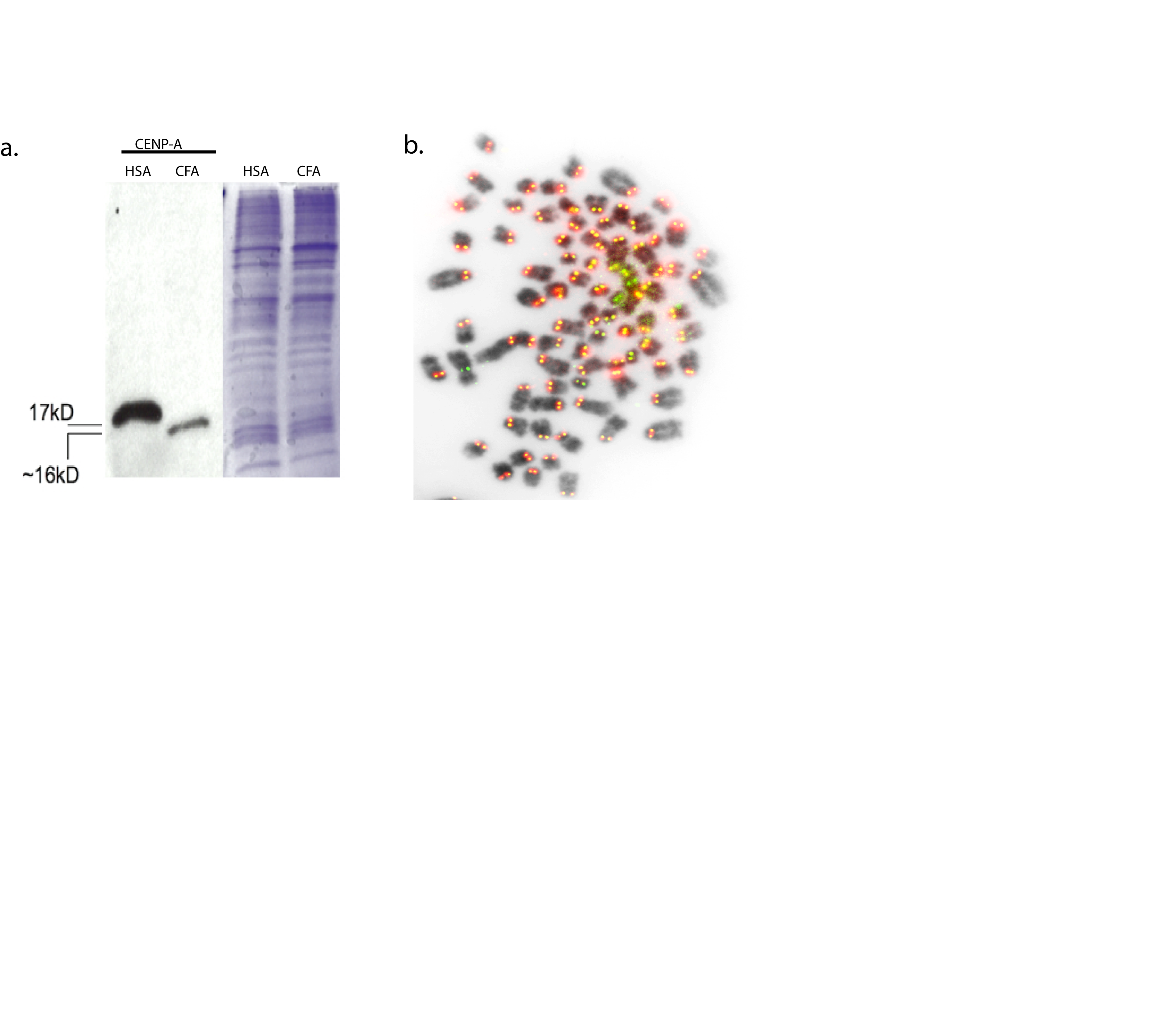
**

**Supplemental Figure 2: CENP-A antibody to MDCK cells**

Canine CENP-A was detected using mouse anti-centromere protein A (CENP-A) monoclonal antibody designed for human CENP-A (a.a. 3-19); (Stressgen; KAM-CC006) by immunoblotting (a), with canine CENP-A (XP_532899.2; ~16kD) shown relative to human CENP-A (NP_001800; ~17kD) compared to loading controls. CENP-A antibody is shown by immunofluorescence (FITC/green) to localize to dog (MDCK) centromeres and colocalize with centromeric satellite family CarSat1 (RHOD/red) (b).


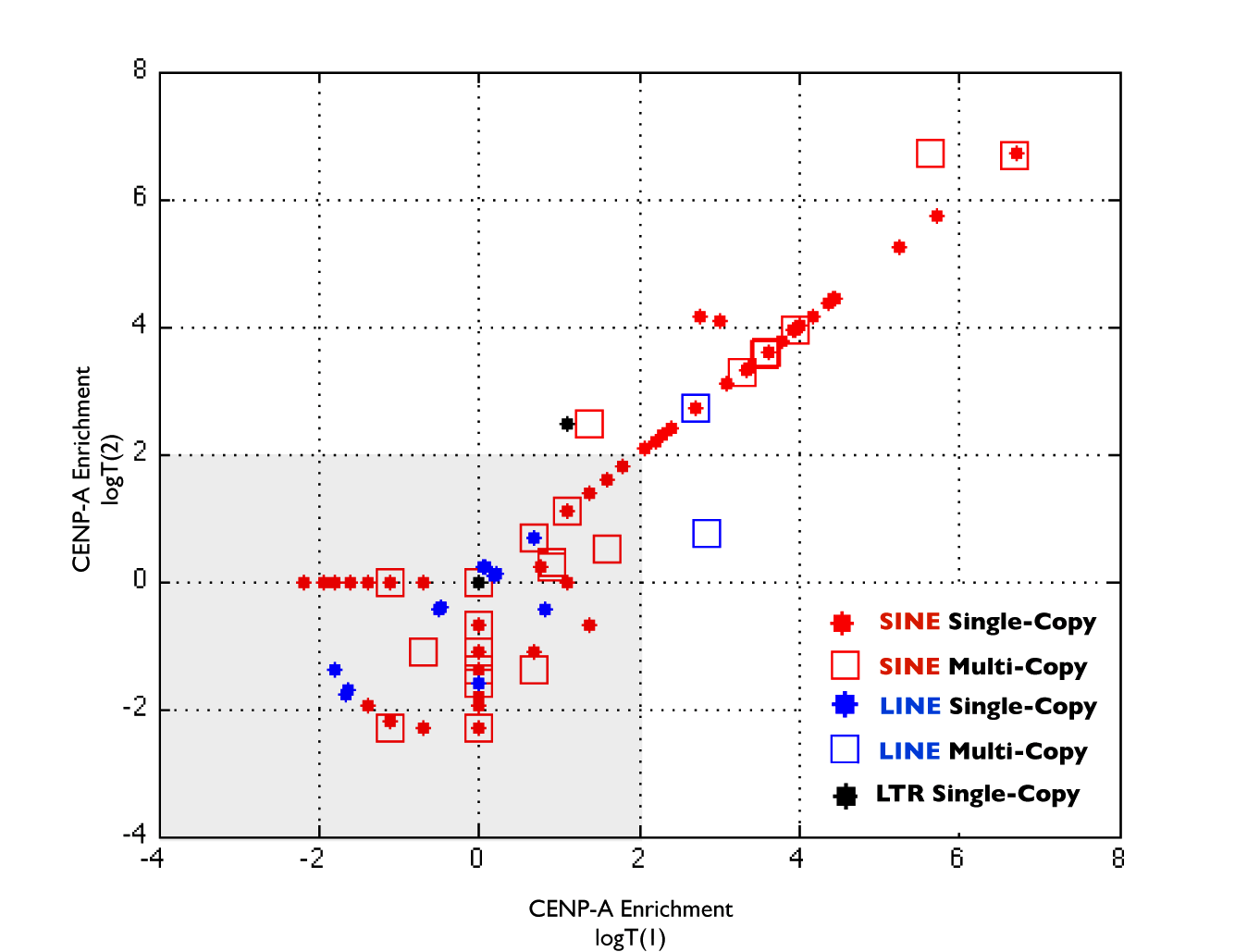


Supplemental Figure 3: Identifying enrichment patterns in satellite transposable element junctions in CarSat1 satellite families

Relative enrichment scores of satellite-transposable element junction sequences are shown in a xy plot from two comparisons with genomic background. Those enrichment patterns that fall below log transformed enrichment value of 2 are shown in shaded box. Remaining single copy (shown as stars) and multi-copy (boxes) transposable element junctions for SINE (red), LINE (blue), and LTR (black) are provided.

**
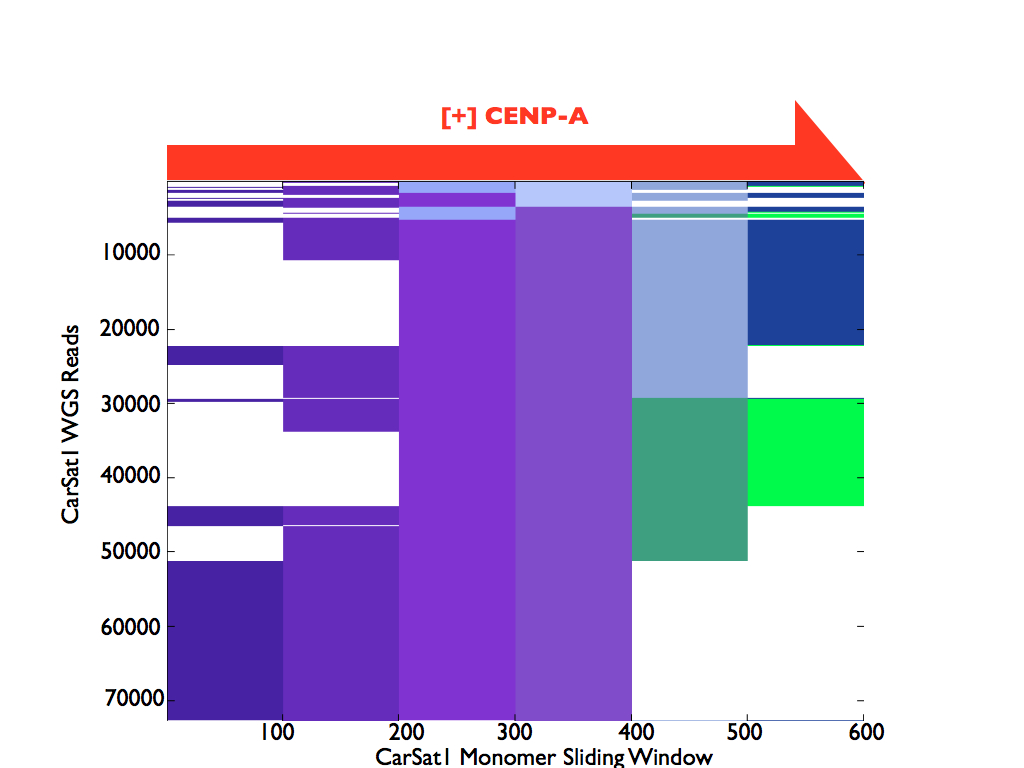
**

**
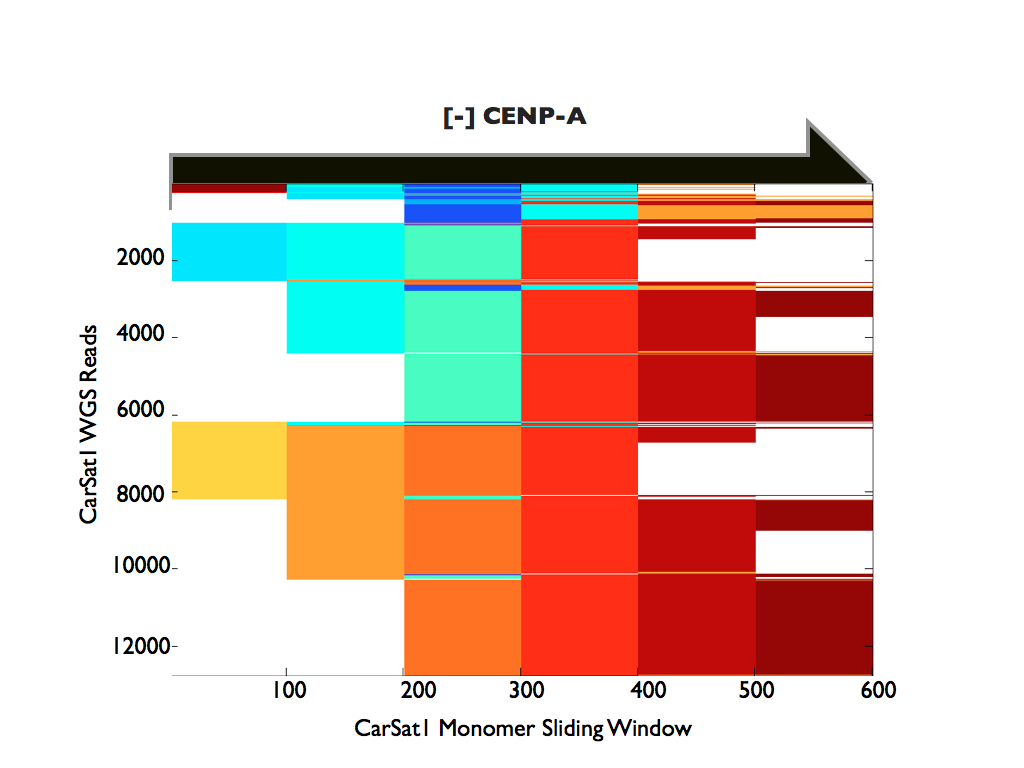
**

**Supplemental Figure 4.** Read Subtype assignments by k-means clustering of 200bp sliding window. All CarSat1 reads reformatted relative to identified consensus sequence (737bp; as determined from consensus bases from all assembled CarSat1 monomers (canFam2.0)). Reads were further divided into six 200bp windows with 100bp overlap/slide. Sequence windows were assigned to clusters using k-means (see Methods) and reads were relabeled as ordered clusters and sorted accordingly. Reads containing minimally four windows are shown above; demonstrating the clustering subgroups defined in paper Figure 3.


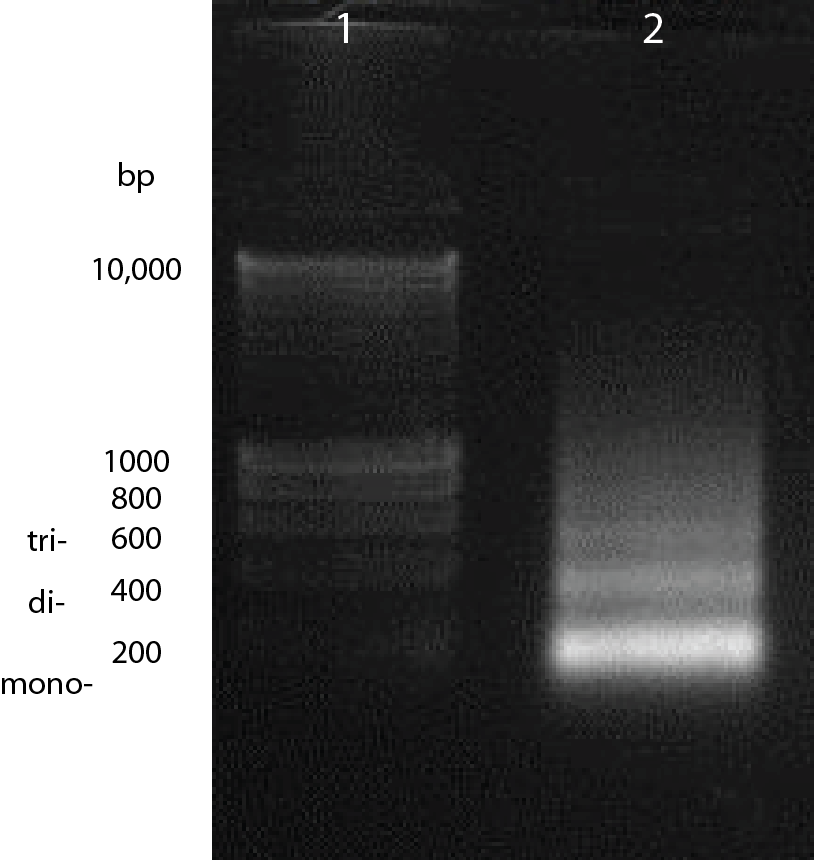


**Supplemental Figure 5.**  MNase digestion for Chromatin IP protocol, demonstrating that mono- and di- nucleosomes are enriched within this study. Lane 1 contains size markers, with appropriate bands (bp) and predicted sites of nucleosome-sized DNA indicated. Lane 2 contains MNase-digested input DNA used in this study.

**Supplemental Table Legends**

**Supplemental Table 1.** Global satellite descriptions and relative abundance and location in the canFam2.0 assembly.

**Supplemental Table 2.** Satellite genomic distribution assignments in the canFam2.0 assembly. Column header information is defined as follows: chr, CanFam2.0 chromosome; chrS, chromosome start position; chrE, chromosome end position; bp_span, the length of the repeat unit (chrE-chrS); satellite name, the canine satellite name either assigned by RepBase, GenBank, or this study; tile_color, color assignments for each family as illustrate in Circos image (Supplemental Figure 1a,b); type, either pericentromeric, or located within a 2Mb window of a chromosome centromere gap, or ‘na’ if found within the chromosome arms or and unmapped assembled contig (chrUn).

**Supplemental Table 3.** Paired read data between abundant (estimated ≥100kb) satellite families.

**Supplemental Table 4.** Annotation of centromeric associated unmapped contigs.

**Supplemental Table 5.**  Distribution of centromeric transposable elements.

Repeat element representation for each centromeric satellite family, describing relative proportions of each repeat family and overall contribution to array.

**Supplemental Table 6.** Centromeric satellite family repeat class enrichment estimates.
