# Supplementary material for: Composition and organization of active centromere sequences in complex genomes
Source: BMC Genomics. 2012 Jul 20;13:324. doi: 10.1186/1471-2164-13-324 (PMC3422206; doi:10.1186/1471-2164-13-324)
Supplement: Additional file 6 — Table S5. Distribution of centromeric transposable elements. Repeat element representation for each centromeric satellite family, describing relative proportions of each repeat family and overall contribution to array. (PDF 37 kb) [file 1471-2164-13-324-S6.pdf]

Supplemental Table 5. Distribution of Centromeric Transposable Elements within Satellite Reads

Repeat element representation embedded within each centromeric satellite family, describing relative proportions of each repeat family and overall contribution to array.

Satellite DNAs		RepeatMasker-Defined Non-Satellite Sequence Content (bp)				
Family	Satellite DNA Content (bp)[1]	Total	SINE elements	LINE elements	LTR elements	DNA family elements
CarSat1	327,474,573	3,135,468	1,589,720	1,080,702	441,032	24,014
Sat1CF	212,545,017	2,331,986	990,648	1,255,325	59,159	26,854
Sat2CF	15,405,544	1,244,757	107,663	1,066,787	67,585	2722
Sat3CF	14,733,077	118,491	47,433	64,803	4206	2049
CarSat2	5,881,357	396,805	158,137	226,013	10,088	2567
Sat4CF	1,559,626	49,300	23,103	19,290	5790	1117
Sat6CF	1,406,032	71,994	14,003	54,215	3017	759

[1] From Supplemental Table 1
