# Supplementary material for: Composition and organization of active centromere sequences in complex genomes
Source: BMC Genomics. 2012 Jul 20;13:324. doi: 10.1186/1471-2164-13-324 (PMC3422206; doi:10.1186/1471-2164-13-324)
Supplement: Additional file 3 — Table S2. Satellite genomic distribution assignments in the canFam2.0 assembly. Column header information is defined as follows: chr, CanFam2.0 chromosome; chrS, chromosome start position; chrE, chromosome end position; bp_span, the length of the repeat unit (chrE-chrS); satellite name, the canine satellite name either assigned by RepBase, GenBank, or this study; tile_color, color assignments for each family as illustrate in Circos image (Additional file1: Figure S1a,b); type, either pericentromeric, or located within a 2 Mb window of a chromosome centromere gap, or ‘na’ if found within the chromosome arms or and unmapped assembled contig (chrUn). [file 1471-2164-13-324-S3.pdf]

Supplemental Table 2: CanFam2 satellite bed file

chr	chrS	chrE	bp_span	satelliteName	tile_color	type
chr1	3000005	3001054	1050	CarSat1	red	pericen
chr1	3001070	3002770	1701	CarSat1	red	pericen
chr1	3002797	3003508	712	CarSat1	red	pericen
chr1	3003535	3004246	712	CarSat1	red	pericen
chr1	3004273	3005479	1207	CarSat1	red	pericen
chr1	3005495	3007933	2439	CarSat1	red	pericen
chr1	3007954	3009890	1937	CarSat1	red	pericen
chr1	3009902	3010870	969	CarSat1	red	pericen
chr1	3010887	3011497	611	CarSat1	red	pericen
chr1	3011694	3012393	700	CarSat1	red	pericen
chr1	3021112	3022045	934	CarSat1	red	pericen
chr1	3022058	3022982	925	CarSat1	red	pericen
chr1	3022996	3024025	1030	SAT1_CF	blue	pericen
chr1	3027678	3028314	637	CarSat1	red	pericen
chr1	3028341	3031750	3410	CarSat1	red	pericen
chr1	3031764	3032648	885	CarSat1	red	pericen
chr1	3032845	3032943	99	CarSat1	red	pericen
chr1	3032957	3033555	599	CarSat1	red	pericen
chr1	3037515	3038618	1104	Sat2CF	purple	pericen
chr1	3037580	3038566	987	Sat2CF	purple	pericen
chr1	3038812	3038947	136	SAT1_CF	blue	pericen
chr1	3038979	3040041	1063	SAT1_CF	blue	pericen
chr1	3040070	3040472	403	CarSat1	red	pericen
chr1	3040491	3042272	1782	CarSat1	red	pericen
chr1	3042306	3042713	408	CarSat1	red	pericen
chr1	3051996	3052741	746	CarSat1	red	pericen
chr1	3052762	3054354	1593	CarSat1	red	pericen
chr1	3054375	3054515	141	CarSat1	red	pericen
chr1	3056986	3058237	1252	Sat2CF	purple	pericen
chr1	3073626	3074523	898	Sat2CF	purple	pericen
chr1	3169146	3169999	854	CarSat1	red	pericen
chr1	3170021	3170842	822	CarSat1	red	pericen
chr1	3171028	3171376	349	CarSat1	red	pericen
chr1	3192099	3192869	771	Sat2CF	purple	pericen
chr1	3192188	3192854	667	Sat2CF	purple	pericen
chr1	3212314	3213463	1150	Sat78CF	na	pericen
chr1	3437128	3437263	136	Carsat2	orange	pericen
chr1	3437150	3439750	2601	CarSat1	red	pericen
chr1	3440928	3443291	2364	CarSat1	red	pericen
chr1	3443477	3444367	891	CarSat1	red	pericen

chr1	3449097	3450615	1519	CarSat1	red	pericen
chr1	3450632	3451597	966	CarSat1	red	pericen
chr1	3451610	3452836	1227	CarSat1	red	pericen
chr1	3452868	3453572	705	CarSat1	red	pericen
chr1	3453748	3454615	868	CarSat1	red	pericen
chr1	3454802	3455407	606	CarSat1	red	pericen
chr1	3455420	3456659	1240	CarSat1	red	pericen
chr1	3456852	3457474	623	CarSat1	red	pericen
chr1	3458263	3458780	518	CarSat1	red	pericen
chr1	3458997	3459126	130	Carsat2	orange	pericen
chr1	3459016	3461492	2477	CarSat1	red	pericen
chr1	3461504	3463589	2086	CarSat1	red	pericen
chr1	3465217	3465384	168	CarSat1	red	pericen
chr1	3465572	3466728	1157	CarSat1	red	pericen
chr1	3596351	3596490	140	Sat18CF	na	pericen
chr1	3966489	3966602	114	Sat49CF	na	pericen
chr1	3988513	3988600	88	Sat43CF	na	pericen
chr1	4071468	4072830	1363	Sat140CF	na	pericen
chr1	4072562	4072861	300	Sat140CF	na	pericen
chr1	4266622	4266748	127	Sat5CF	stalk	pericen
chr1	4512957	4513039	83	Sat13CF	na	pericen
chr1	4584372	4584631	260	Sat91CF	na	pericen
chr1	4607419	4607647	229	Sat170CF	na	pericen
chr1	4636446	4636574	129	Sat5CF	stalk	pericen
chr1	5093351	5093421	71	Sat5CF	stalk	na
chr1	5916203	5916475	273	Sat120CF	na	na
chr1	5942342	5942547	206	Sat65CF	na	na
chr1	5942397	5942624	228	Sat86CF	na	na
chr1	5942425	5942719	295	Sat65CF	na	na
chr1	5964203	5964311	109	Carsat3	green	na
chr1	6038235	6038335	101	Carsat3	green	na
chr1	6099492	6099695	204	Sat102CF	na	na
chr1	6222953	6223187	235	Sat5CF	stalk	na
chr1	6223000	6223181	182	Sat93CF	na	na
chr1	6441256	6441325	70	Sat63CF	na	na
chr1	7905859	7906036	178	Sat65CF	na	na
chr1	17884491	17884768	278	Sat17CF	na	na
chr1	20354668	20354711	44	Sat40CF	na	na
chr1	21129007	21129061	55	Sat22CF	na	na
chr1	21956958	21957016	59	Sat88CF	na	na
chr1	23049267	23049440	174	SAT1_CF	blue	na
chr1	23755927	23757348	1422	Sat132CF	na	na

chr1	26880011	26881850	1840	Sat55CF	na	na
chr1	26881923	26881952	30	Sat39CF	na	na
chr1	26882759	26884222	1464	Sat39CF	na	na
chr1	26884292	26884543	252	Sat39CF	na	na
chr1	26884296	26884620	325	Sat38CF	na	na
chr1	26893527	26895268	1742	Sat86CF	na	na
chr1	27657904	27658007	104	Sat169CF	na	na
chr1	27657918	27659065	1148	Sat169CF	na	na
chr1	27659022	27659070	49	Sat169CF	na	na
chr1	27709453	27709630	178	Sat37CF	na	na
chr1	27710265	27710420	156	Sat37CF	na	na
chr1	27738445	27738760	316	Sat34CF	na	na
chr1	30048883	30048937	55	Sat21CF	na	na
chr1	32497340	32497378	39	Sat5CF	stalk	na
chr1	33626633	33626837	205	Sat17CF	na	na
chr1	34751290	34751365	76	Sat46CF	na	na
chr1	40712618	40712659	42	Sat36CF	na	na
chr1	41651010	41651057	48	Sat27CF	na	na
chr1	48803534	48803580	47	Sat41CF	na	na
chr1	49611233	49611277	45	Sat76CF	na	na
chr1	50694257	50694307	51	CarSat1	red	na
chr1	54297071	54297113	43	Sat140CF	na	na
chr1	55040309	55040395	87	Sat102CF	na	na
chr1	56773850	56773977	128	Sat141CF	na	na
chr1	56998588	56998717	130	Sat37CF	na	na
chr1	57064994	57065061	68	Sat18CF	na	na
chr1	57070447	57070639	193	Sat47CF	na	na
chr1	57365368	57365550	183	Sat93CF	na	na
chr1	57399798	57399894	97	Sat181CF	na	na
chr1	57399895	57400956	1062	Sat181CF	na	na
chr1	57400957	57401015	59	Sat181CF	na	na
chr1	57401168	57401373	206	Sat181CF	na	na
chr1	58076304	58076568	265	Sat47CF	na	na
chr1	58076309	58076541	233	Sat85CF	na	na
chr1	58378757	58378912	156	Sat79CF	na	na
chr1	58679694	58679920	227	Sat171CF	na	na
chr1	59738934	59739091	158	Sat146CF	na	na
chr1	70048687	70048745	59	Sat107CF	na	na
chr1	70516005	70516154	150	Sat112CF	na	na
chr1	71383054	71383215	162	Sat5CF	stalk	na
chr1	71646925	71647684	760	Sat96CF	na	na
chr1	72082906	72083073	168	Sat16CF	na	na

chr1	72131928	72132085	158	Sat150CF	na	na
chr1	72319446	72319491	46	Sat52CF	na	na
chr1	72442735	72442912	178	Sat130CF	na	na
chr1	72598755	72601699	2945	SAT1_CF	blue	na
chr1	72601744	72602478	735	SAT1_CF	blue	na
chr1	72602491	72603795	1305	CarSat1	red	na
chr1	72610096	72610224	129	CarSat1	red	na
chr1	72643333	72644823	1491	CarSat1	red	na
chr1	72644844	72646701	1858	CarSat1	red	na
chr1	72646731	72648530	1800	CarSat1	red	na
chr1	72648551	72648792	242	CarSat1	red	na
chr1	72648973	72649532	560	CarSat1	red	na
chr1	72649546	72649749	204	CarSat1	red	na
chr1	72649933	72651773	1841	CarSat1	red	na
chr1	72651808	72654910	3103	CarSat1	red	na
chr1	72654968	72656243	1276	CarSat1	red	na
chr1	72656275	72656983	709	CarSat1	red	na
chr1	72657018	72657708	691	CarSat1	red	na
chr1	72657719	72658444	726	CarSat1	red	na
chr1	72658456	72659185	730	CarSat1	red	na
chr1	72659198	72660142	945	CarSat1	red	na
chr1	72666424	72667248	825	CarSat1	red	na
chr1	72667274	72669679	2406	CarSat1	red	na
chr1	72669692	72670928	1237	CarSat1	red	na
chr1	72671101	72674244	3144	CarSat1	red	na
chr1	72674266	72674855	590	CarSat1	red	na
chr1	72675017	72675410	394	CarSat1	red	na
chr1	72678438	72678565	128	Carsat2	orange	na
chr1	72678454	72680037	1584	CarSat1	red	na
chr1	72692981	72694139	1159	Carsat2	orange	na
chr1	72694139	72694255	117	CarSat1	red	na
chr1	72694435	72694792	358	CarSat1	red	na
chr1	72694825	72696407	1583	Carsat2	orange	na
chr1	72703532	72706848	3317	Carsat2	orange	na
chr1	72743751	72743873	123	Carsat3	green	na
chr1	72744048	72744198	151	Carsat3	green	na
chr1	72744218	72746321	2104	Carsat3	green	na
chr1	72746737	72746817	81	Carsat3	green	na
chr1	72790024	72790065	42	Sat91CF	na	na
chr1	73016153	73016221	69	Sat33CF	na	na
chr1	73161879	73162014	136	Carsat3	green	na
chr1	73162228	73163545	1318	Carsat3	green	na

chr1	73231371	73232918	1548	Carsat3	green	na
chr1	73535114	73535220	107	Carsat3	green	na
chr1	73674731	73675016	286	Sat47CF	na	na
chr1	74394507	74394864	358	Sat8CF	dgreen	na
chr1	74396636	74396755	120	Sat8CF	dgreen	na
chr1	74396815	74397000	186	Sat8CF	dgreen	na
chr1	75386635	75386671	37	Sat45CF	na	na
chr1	77185775	77185867	93	Sat63CF	na	na
chr1	77796299	77796348	50	Sat18CF	na	na
chr1	79634562	79634797	236	Carsat3	green	na
chr1	80117048	80117092	45	Sat22CF	na	na
chr1	80493201	80493295	95	Sat88CF	na	na
chr1	92117334	92117495	162	SUBTEL_sa	na	na
chr1	96683677	96683897	221	Sat17CF	na	na
chr1	97057527	97057806	280	Carsat3	green	na
chr1	97057821	97058776	956	Carsat3	green	na
chr1	97280789	97280871	83	Sat16CF	na	na
chr1	98434580	98434971	392	Sat30CF	na	na
chr1	99600496	99600683	188	Sat5CF	stalk	na
chr1	99600579	99600682	104	Sat5CF	stalk	na
chr1	99919695	99919846	152	Sat57CF	na	na
chr1	100047620	100049392	1773	Sat89CF	na	na
chr1	100049249	100049418	170	Sat89CF	na	na
chr1	100207475	100207526	52	Sat43CF	na	na
chr1	100524675	100524749	75	Sat27CF	na	na
chr1	100880304	100880453	150	Carsat3	green	na
chr1	100880625	100880873	249	Carsat3	green	na
chr1	100880965	100881348	384	Carsat3	green	na
chr1	101335915	101336167	253	Sat106CF	na	na
chr1	101467699	101467868	170	Sat89CF	na	na
chr1	101467725	101468696	972	Sat89CF	na	na
chr1	101471252	101472064	813	Sat89CF	na	na
chr1	102233191	102235026	1836	Carsat3	green	na
chr1	102239651	102239776	126	Carsat3	green	na
chr1	102246856	102247453	598	Carsat3	green	na
chr1	102247502	102247745	244	Carsat3	green	na
chr1	102376651	102376883	233	SAT1_CF	blue	na
chr1	102382742	102382939	198	Sat2CF	purple	na
chr1	102382749	102382992	244	Sat2CF	purple	na
chr1	102391074	102391300	227	Sat2CF	purple	na
chr1	102391114	102391342	229	Sat2CF	purple	na
chr1	102418460	102420754	2295	Carsat2	orange	na

chr1	102433732	102433872	141	Carsat3	green	na
chr1	102433975	102434525	551	Carsat3	green	na
chr1	102434555	102435022	468	Carsat3	green	na
chr1	102464634	102464807	174	Carsat3	green	na
chr1	102465498	102465639	142	Carsat3	green	na
chr1	102519001	102519105	105	Carsat3	green	na
chr1	102519134	102519770	637	Carsat3	green	na
chr1	102536788	102536858	71	Carsat3	green	na
chr1	102537026	102537121	96	Carsat3	green	na
chr1	102537347	102537772	426	Carsat3	green	na
chr1	102537998	102538104	107	Carsat3	green	na
chr1	102538683	102538745	63	Carsat3	green	na
chr1	102538936	102539033	98	Carsat3	green	na
chr1	102553452	102554494	1043	Carsat3	green	na
chr1	102557322	102557457	136	Carsat3	green	na
chr1	102565393	102566808	1416	Carsat3	green	na
chr1	102588169	102588841	673	Carsat3	green	na
chr1	102605129	102605401	273	Carsat3	green	na
chr1	102605563	102606137	575	Carsat3	green	na
chr1	102613033	102613133	101	Carsat3	green	na
chr1	102645136	102645252	117	Carsat3	green	na
chr1	102645407	102645620	214	Carsat3	green	na
chr1	102645739	102645902	164	Carsat3	green	na
chr1	102655904	102656031	128	Carsat3	green	na
chr1	102681200	102681875	676	Carsat3	green	na
chr1	102691219	102691523	305	Carsat3	green	na
chr1	102691575	102691804	230	Carsat3	green	na
chr1	102698564	102698950	387	Carsat3	green	na
chr1	102698988	102699548	561	Carsat3	green	na
chr1	102699638	102699971	334	Carsat3	green	na
chr1	102701175	102701218	44	Sat70CF	na	na
chr1	102714023	102714487	465	Carsat3	green	na
chr1	102737929	102739177	1249	Carsat3	green	na
chr1	102753046	102753437	392	Carsat3	green	na
chr1	102753455	102753628	174	Carsat3	green	na
chr1	102765623	102766132	510	Carsat3	green	na
chr1	102796122	102796377	256	Carsat3	green	na
chr1	102796618	102796759	142	Carsat3	green	na
chr1	102831788	102832914	1127	Carsat3	green	na
chr1	102858904	102859241	338	Carsat3	green	na
chr1	102859355	102859519	165	Carsat3	green	na
chr1	102888914	102890247	1334	Carsat3	green	na

chr1	102943490	102943679	190	Sat17CF	na	na
chr1	102945781	102946437	657	Carsat3	green	na
chr1	102983645	102983780	136	Carsat3	green	na
chr1	102983810	102985102	1293	Carsat3	green	na
chr1	102985237	102985329	93	Carsat3	green	na
chr1	103030735	103030874	140	Carsat3	green	na
chr1	103031052	103032169	1118	Carsat3	green	na
chr1	103048570	103048690	121	Carsat3	green	na
chr1	103048745	103049246	502	Carsat3	green	na
chr1	103049422	103049713	292	Carsat3	green	na
chr1	103079392	103080266	875	Carsat3	green	na
chr1	103106719	103108355	1637	Carsat3	green	na
chr1	103127658	103127802	145	Carsat3	green	na
chr1	103127962	103128243	282	Carsat3	green	na
chr1	103128433	103129842	1410	Carsat3	green	na
chr1	103146550	103146852	303	Carsat3	green	na
chr1	103147020	103149031	2012	Carsat3	green	na
chr1	103168534	103168900	367	Carsat3	green	na
chr1	103168919	103171732	2814	Carsat3	green	na
chr1	103178055	103178184	130	Carsat3	green	na
chr1	103178843	103179035	193	Carsat3	green	na
chr1	103179611	103179820	210	Carsat3	green	na
chr1	103230705	103232460	1756	Carsat3	green	na
chr1	103261494	103261722	229	Carsat3	green	na
chr1	103261733	103263770	2038	Carsat3	green	na
chr1	103325141	103325945	805	Carsat3	green	na
chr1	103350690	103351694	1005	Carsat3	green	na
chr1	103386279	103387076	798	Carsat3	green	na
chr1	103423671	103424454	784	Carsat3	green	na
chr1	103424465	103424570	106	Carsat3	green	na
chr1	103450354	103451095	742	Carsat3	green	na
chr1	103451108	103451400	293	Carsat3	green	na
chr1	103476495	103476666	172	Carsat3	green	na
chr1	103491894	103492764	871	Carsat3	green	na
chr1	103492802	103493078	277	Carsat3	green	na
chr1	103510977	103512407	1431	Carsat3	green	na
chr1	103534677	103534953	277	Carsat3	green	na
chr1	103534991	103535864	874	Carsat3	green	na
chr1	103551664	103551835	172	Carsat3	green	na
chr1	103573903	103574832	930	Carsat3	green	na
chr1	103591260	103591988	729	Carsat3	green	na
chr1	103592123	103592219	97	Carsat3	green	na

chr1	103620380	103621453	1074	Carsat3	green	na
chr1	103638168	103638559	392	Carsat3	green	na
chr1	103638586	103639646	1061	Carsat3	green	na
chr1	103675479	103675949	471	Carsat3	green	na
chr1	103675985	103676161	177	Carsat3	green	na
chr1	103693347	103694383	1037	Carsat3	green	na
chr1	103715165	103715862	698	Carsat3	green	na
chr1	103745472	103746411	940	Carsat3	green	na
chr1	103754112	103754391	280	Carsat3	green	na
chr1	103754503	103754662	160	Carsat3	green	na
chr1	103754735	103754879	145	Carsat3	green	na
chr1	103771815	103771912	98	Carsat3	green	na
chr1	103772063	103773279	1217	Carsat3	green	na
chr1	103795382	103796414	1033	Carsat3	green	na
chr1	103813664	103814957	1294	Carsat3	green	na
chr1	103835981	103836075	95	Carsat3	green	na
chr1	103836087	103836267	181	Carsat3	green	na
chr1	103836301	103836420	120	Carsat3	green	na
chr1	103836461	103836640	180	Carsat3	green	na
chr1	103855873	103857323	1451	Carsat3	green	na
chr1	103883785	103883860	76	Carsat3	green	na
chr1	103883977	103885207	1231	Carsat3	green	na
chr1	103922932	103924141	1210	Carsat3	green	na
chr1	103979895	103980429	535	Carsat3	green	na
chr1	103980807	103981125	319	Carsat3	green	na
chr1	103982219	103982514	296	Carsat3	green	na
chr1	104192627	104193383	757	Carsat3	green	na
chr1	104194093	104194222	130	Carsat3	green	na
chr1	104194965	104195064	100	Carsat3	green	na
chr1	104195190	104195266	77	Carsat3	green	na
chr1	104195317	104195446	130	Carsat3	green	na
chr1	104195464	104195587	124	Carsat3	green	na
chr1	104227011	104227560	550	Carsat3	green	na
chr1	104227691	104227908	218	Carsat3	green	na
chr1	104256760	104257017	258	Carsat3	green	na
chr1	104257109	104257324	216	Carsat3	green	na
chr1	104269197	104269291	95	Carsat3	green	na
chr1	104269335	104270043	709	Carsat3	green	na
chr1	104433860	104434084	225	Carsat3	green	na
chr1	104434439	104434658	220	Carsat3	green	na
chr1	104434691	104435107	417	Carsat3	green	na
chr1	104472334	104472486	153	Carsat3	green	na

chr1	104472617	104472723	107	Carsat3	green	na
chr1	104472784	104472954	171	Carsat3	green	na
chr1	104480766	104481868	1103	Carsat3	green	na
chr1	104510235	104511672	1438	Carsat3	green	na
chr1	104531478	104532754	1277	Carsat3	green	na
chr1	104559866	104560554	689	Carsat3	green	na
chr1	104560569	104561406	838	Carsat3	green	na
chr1	104587857	104588524	668	Carsat3	green	na
chr1	104588712	104588937	226	Carsat3	green	na
chr1	104610615	104610766	152	Carsat3	green	na
chr1	104610848	104611162	315	Carsat3	green	na
chr1	104611220	104611514	295	Carsat3	green	na
chr1	104625686	104626741	1056	Carsat3	green	na
chr1	104649929	104650065	137	Sat5CF	stalk	na
chr1	104663841	104664863	1023	Carsat3	green	na
chr1	104680420	104681304	885	Carsat3	green	na
chr1	104681317	104681837	521	Carsat3	green	na
chr1	104784903	104785016	114	Carsat3	green	na
chr1	104785076	104785180	105	Carsat3	green	na
chr1	105263952	105264195	244	Sat18CF	na	na
chr1	106318691	106319363	673	Sat74CF	na	na
chr1	106318835	106319369	535	Sat74CF	na	na
chr1	106592996	106594082	1087	Carsat3	green	na
chr1	106668358	106668520	163	Carsat3	green	na
chr1	106668806	106668916	111	Carsat3	green	na
chr1	106697037	106697166	130	Carsat3	green	na
chr1	106697510	106697967	458	Carsat3	green	na
chr1	106698090	106698283	194	Carsat3	green	na
chr1	106811166	106811400	235	Carsat3	green	na
chr1	106811466	106812848	1383	Carsat3	green	na
chr1	106830192	106830408	217	Carsat3	green	na
chr1	106869827	106869989	163	Carsat3	green	na
chr1	106870252	106870438	187	Carsat3	green	na
chr1	106889640	106891010	1371	Carsat3	green	na
chr1	106922533	106922672	140	Carsat3	green	na
chr1	106922964	106923061	98	Carsat3	green	na
chr1	106942876	106944608	1733	Carsat3	green	na
chr1	106944637	106944746	110	Carsat3	green	na
chr1	106989896	106991448	1553	Carsat3	green	na
chr1	107006305	107006512	208	Carsat3	green	na
chr1	107006557	107006849	293	Carsat3	green	na
chr1	107007103	107007242	140	Carsat3	green	na

chr1	107028213	107028335	123	Carsat3	green	na
chr1	107041461	107043557	2097	Carsat3	green	na
chr1	107045190	107045316	127	Carsat3	green	na
chr1	107055599	107055806	208	Carsat3	green	na
chr1	107055820	107056221	402	Carsat3	green	na
chr1	107056399	107056538	140	Carsat3	green	na
chr1	107086669	107086793	125	Carsat3	green	na
chr1	107086987	107088208	1222	Carsat3	green	na
chr1	107101972	107102179	208	Carsat3	green	na
chr1	107102217	107102463	247	Carsat3	green	na
chr1	107124384	107124502	119	Carsat3	green	na
chr1	107124876	107125726	851	Carsat3	green	na
chr1	107172259	107172521	263	Carsat3	green	na
chr1	107172576	107174003	1428	Carsat3	green	na
chr1	107174030	107174156	127	Carsat3	green	na
chr1	107187349	107187469	121	Carsat3	green	na
chr1	107187562	107187802	241	Carsat3	green	na
chr1	107188022	107188129	108	Carsat3	green	na
chr1	107213945	107214076	132	Carsat3	green	na
chr1	107225331	107225564	234	Carsat3	green	na
chr1	107225733	107227169	1437	Carsat3	green	na
chr1	107235103	107235311	209	Carsat3	green	na
chr1	107235349	107235649	301	Carsat3	green	na
chr1	107274956	107276954	1999	Carsat3	green	na
chr1	107291449	107291656	208	Carsat3	green	na
chr1	107291694	107291954	261	Carsat3	green	na
chr1	107314168	107314401	234	Carsat3	green	na
chr1	107314486	107315624	1139	Carsat3	green	na
chr1	107327321	107327529	209	Carsat3	green	na
chr1	107327567	107327790	224	Carsat3	green	na
chr1	107354860	107355697	838	Carsat3	green	na
chr1	107400897	107401080	184	Carsat3	green	na
chr1	107401351	107401445	95	Carsat3	green	na
chr1	107401488	107403003	1516	Carsat3	green	na
chr1	107403125	107403251	127	Carsat3	green	na
chr1	107415959	107416491	533	Carsat3	green	na
chr1	107449809	107449885	77	Carsat3	green	na
chr1	107450185	107450971	787	Carsat3	green	na
chr1	107450998	107451133	136	Carsat3	green	na
chr1	107550116	107550209	94	Carsat3	green	na
chr1	107550278	107550985	708	Carsat3	green	na
chr1	107572059	107572145	87	Carsat3	green	na

chr1	107574643	107575903	1261	Carsat3	green	na
chr1	107683502	107684796	1295	Carsat3	green	na
chr1	107684883	107685048	166	Carsat3	green	na
chr1	107685276	107685342	67	Carsat3	green	na
chr1	107703730	107703840	111	Carsat3	green	na
chr1	107704016	107704910	895	Carsat3	green	na
chr1	107704925	107705822	898	Carsat3	green	na
chr1	107726186	107726313	128	Carsat3	green	na
chr1	107750926	107752306	1381	Carsat3	green	na
chr1	107791936	107792192	257	Carsat3	green	na
chr1	107792205	107792895	691	Carsat3	green	na
chr1	108001915	108002083	169	Carsat3	green	na
chr1	108002098	108002978	881	Carsat3	green	na
chr1	108019574	108019780	207	Carsat3	green	na
chr1	108019994	108021603	1610	Carsat3	green	na
chr1	108038555	108038649	95	Carsat3	green	na
chr1	108038759	108038832	74	Carsat3	green	na
chr1	108039223	108040448	1226	Carsat3	green	na
chr1	108059867	108060009	143	Carsat3	green	na
chr1	108060062	108061535	1474	Carsat3	green	na
chr1	108077906	108078771	866	Carsat3	green	na
chr1	108090274	108090394	121	Carsat3	green	na
chr1	108090570	108090758	189	Carsat3	green	na
chr1	108090919	108092000	1082	Carsat3	green	na
chr1	108115456	108115580	125	Carsat3	green	na
chr1	108115789	108116829	1041	Carsat3	green	na
chr1	108131616	108133049	1434	Carsat3	green	na
chr1	108161999	108163014	1016	Carsat3	green	na
chr1	108163248	108163372	125	Carsat3	green	na
chr1	108179364	108180611	1248	Carsat3	green	na
chr1	108180622	108180998	377	Carsat3	green	na
chr1	108181447	108181591	145	Carsat3	green	na
chr1	108202748	108204514	1767	Carsat3	green	na
chr1	108212720	108213045	326	Carsat3	green	na
chr1	108213056	108213185	130	Carsat3	green	na
chr1	108213362	108214002	641	Carsat3	green	na
chr1	108214174	108214324	151	Carsat3	green	na
chr1	108324771	108324849	79	Sat43CF	na	na
chr1	108339240	108339318	79	Sat43CF	na	na
chr1	108383905	108385325	1421	Carsat3	green	na
chr1	108511623	108511707	85	Sat42CF	na	na
chr1	108882454	108882835	382	Sat55CF	na	na

chr1	108882639	108882867	229	Sat55CF	na	na
chr1	109173980	109174112	133	Sat98CF	na	na
chr1	109421299	109422768	1470	Carsat3	green	na
chr1	109439204	109440146	943	Carsat3	green	na
chr1	109440171	109440652	482	Carsat3	green	na
chr1	109440739	109441229	491	Carsat3	green	na
chr1	109648845	109648888	44	Sat13CF	na	na
chr1	109786651	109786703	53	Sat32CF	na	na
chr1	109974224	109974279	56	Sat40CF	na	na
chr1	110832645	110832689	45	Carsat3	green	na
chr1	110833640	110833931	292	Carsat3	green	na
chr1	110834061	110834171	111	Carsat3	green	na
chr1	113526245	113526729	485	Sat12CF	na	na
chr1	113545997	113546073	77	Sat12CF	na	na
chr1	113546300	113546458	159	Sat12CF	na	na
chr1	113546391	113546469	79	Sat12CF	na	na
chr1	113546681	113546879	199	Sat12CF	na	na
chr1	113738757	113738965	209	Carsat3	green	na
chr1	113739015	113740130	1116	Carsat3	green	na
chr1	113777747	113777960	214	Carsat3	green	na
chr1	113778085	113778903	819	Carsat3	green	na
chr1	113809000	113809298	299	Carsat3	green	na
chr1	113809419	113810007	589	Carsat3	green	na
chr1	113871185	113872655	1471	Carsat3	green	na
chr1	113872686	113872915	230	Carsat3	green	na
chr1	113925627	113926924	1298	Carsat3	green	na
chr1	113975036	113975757	722	Carsat3	green	na
chr1	113975808	113975906	99	Carsat3	green	na
chr1	113987078	113988699	1622	Carsat3	green	na
chr1	114023566	114025194	1629	Carsat3	green	na
chr1	114046572	114046866	295	Carsat3	green	na
chr1	114046881	114048126	1246	Carsat3	green	na
chr1	114075345	114075952	608	Carsat3	green	na
chr1	114076056	114076451	396	Carsat3	green	na
chr1	114113572	114115079	1508	Carsat3	green	na
chr1	114176659	114177931	1273	Carsat3	green	na
chr1	114195488	114195671	184	Carsat3	green	na
chr1	114195686	114195906	221	Carsat3	green	na
chr1	114196024	114197448	1425	Carsat3	green	na
chr1	115828546	115828628	83	Sat18CF	na	na
chr1	116433804	116436461	2658	Carsat3	green	na
chr1	116812428	116812599	172	Sat5CF	stalk	na

chr1	117983451	117983968	518	Carsat3	green	na
chr1	117983984	117984666	683	Carsat3	green	na
chr1	117998020	118000197	2178	Carsat3	green	na
chr1	118032880	118034248	1369	Carsat3	green	na
chr1	118095854	118097883	2030	Carsat3	green	na
chr1	118158183	118159153	971	Carsat3	green	na
chr1	118171043	118171173	131	Carsat3	green	na
chr1	118214845	118215511	667	Carsat3	green	na
chr1	118215781	118216084	304	Carsat3	green	na
chr1	118257219	118257306	88	Carsat3	green	na
chr1	118257387	118258504	1118	Carsat3	green	na
chr1	118323848	118325476	1629	Carsat3	green	na
chr1	118403824	118404770	947	Carsat3	green	na
chr1	118423789	118425486	1698	Carsat3	green	na
chr1	118498930	118499279	350	Carsat3	green	na
chr1	118499295	118500370	1076	Carsat3	green	na
chr1	118534075	118534299	225	Carsat3	green	na
chr1	118534482	118534495	14	Carsat3	green	na
chr1	118534754	118535263	510	Carsat3	green	na
chr1	118535365	118535429	65	Carsat3	green	na
chr1	118535511	118535639	129	Carsat3	green	na
chr1	118551998	118553323	1326	Carsat3	green	na
chr1	118585108	118586397	1290	Carsat3	green	na
chr1	118629924	118631293	1370	Carsat3	green	na
chr1	118654760	118656173	1414	Carsat3	green	na
chr1	118696699	118697816	1118	Carsat3	green	na
chr1	118763467	118764025	559	Carsat3	green	na
chr1	118783715	118784600	886	Carsat3	green	na
chr1	118840666	118841956	1291	Carsat3	green	na
chr1	118880176	118881402	1227	Carsat3	green	na
chr1	118905438	118905521	84	Carsat3	green	na
chr1	118906367	118906455	89	Carsat3	green	na
chr1	118906584	118907702	1119	Carsat3	green	na
chr1	118947249	118948373	1125	Carsat3	green	na
chr1	119003207	119004077	871	Carsat3	green	na
chr1	119051704	119053158	1455	Carsat3	green	na
chr1	119099000	119099916	917	Carsat3	green	na
chr1	119100051	119100212	162	Carsat3	green	na
chr1	119186058	119186183	126	Carsat3	green	na
chr1	119186196	119187071	876	Carsat3	green	na
chr1	119213165	119214235	1071	Carsat3	green	na
chr1	119290289	119291607	1319	Carsat3	green	na

chr1	119308949	119309884	936	Carsat3	green	na
chr1	119309903	119310089	187	Carsat3	green	na
chr1	119319552	119320256	705	Carsat3	green	na
chr1	119368295	119369496	1202	Carsat3	green	na
chr1	119369536	119369769	234	Carsat3	green	na
chr1	119422069	119423174	1106	Carsat3	green	na
chr1	119643509	119643769	261	Sat21CF	na	na
chr1	120041826	120043074	1249	Sat126CF	na	na
chr1	120041960	120043722	1763	Sat126CF	na	na
chr1	120042141	120043938	1798	Sat126CF	na	na
chr1	120043275	120044017	743	Sat126CF	na	na
chr1	120332658	120333178	521	CarSat1	red	na
chr1	120494109	120495101	993	Carsat3	green	na
chr1	120495181	120495267	87	Carsat3	green	na
chr1	120503623	120504799	1177	Carsat3	green	na
chr1	120550589	120551757	1169	Carsat3	green	na
chr1	120632909	120633063	155	Carsat3	green	na
chr1	120633087	120634433	1347	Carsat3	green	na
chr1	120680264	120680912	649	Carsat3	green	na
chr1	120839130	120841069	1940	Sat77CF	na	na
chr1	120839341	120841435	2095	Sat77CF	na	na
chr1	120839737	120841855	2119	Sat77CF	na	na
chr1	120840105	120842231	2127	Sat77CF	na	na
chr1	120840476	120842574	2099	Sat77CF	na	na
chr1	120840898	120842958	2061	Sat77CF	na	na
chr1	120842775	120843205	431	Sat77CF	na	na
chr1	120843441	120843532	92	Sat77CF	na	na
chr1	121351289	121351317	29	Sat5CF	stalk	na
chr1	121351300	121351317	18	Sat5CF	stalk	na
chr1	121920304	121920471	168	SUBTEL_sa	na	na
chr1	121920512	121920838	327	SUBTEL_sa	na	na
chr1	121920885	121921032	148	SUBTEL_sa	na	na
chr1	121981697	121981800	104	Sat107CF	na	na
chr1	122337364	122337401	38	Sat72CF	na	na
chr1	122337895	122337967	73	Sat72CF	na	na
chr1	123352689	123352892	204	Sat37CF	na	na
chr1	123875374	123875822	449	Sat57CF	na	na
chr1	123875404	123875756	353	Sat57CF	na	na
chr1	124008473	124009972	1500	Sat14CF	na	na
chr1	124156855	124157343	489	Sat147CF	na	na
chr1	124872228	124872531	304	Sat53CF	na	na
chr1	124920462	124920569	108	Sat18CF	na	na

chr1	124921880	124922469	590	Sat34CF	na	na
chr1	124921959	124922469	511	Sat34CF	na	na
chr1	124922041	124922519	479	Sat34CF	na	na
chr1	125091305	125091480	176	Sat52CF	na	na
chr1	125429159	125429464	306	Sat106CF	na	na
chr1	125429208	125429458	251	Sat57CF	na	na
chr10	4838249	4838338	90	Sat62CF	na	pericen
chr10	8466362	8466949	588	CarSat1	red	na
chr10	10460678	10460774	97	SUBTEL_sa	na	na
chr10	12103412	12103452	41	Sat76CF	na	na
chr10	13163894	13164009	116	Carsat3	green	na
chr10	13821784	13822035	252	Sat6CF	select	na
chr10	13821814	13822664	851	Sat6CF	select	na
chr10	14173241	14173313	73	Sat86CF	na	na
chr10	15196626	15196739	114	Sat21CF	na	na
chr10	15744650	15744768	119	Sat27CF	na	na
chr10	16096201	16096656	456	Sat18CF	na	na
chr10	16344157	16344245	89	Sat136CF	na	na
chr10	16344169	16345308	1140	Sat136CF	na	na
chr10	16345188	16345322	135	Sat136CF	na	na
chr10	16770319	16770363	45	Sat60CF	na	na
chr10	17277015	17277215	201	Sat178CF	na	na
chr10	17944026	17944247	222	Sat17CF	na	na
chr10	18412858	18413660	803	Sat163CF	na	na
chr10	18431449	18432042	594	Sat28CF	na	na
chr10	18431528	18432075	548	Sat28CF	na	na
chr10	18979638	18980334	697	Sat79CF	na	na
chr10	19396023	19396393	371	Sat7CF	lime	na
chr10	19721305	19722034	730	Sat53CF	na	na
chr10	20489596	20489680	85	Sat27CF	na	na
chr10	20489608	20490138	531	Sat18CF	na	na
chr10	20489687	20495352	5666	Sat27CF	na	na
chr10	20611930	20612362	433	Sat19CF	na	na
chr10	20613963	20613991	29	Sat52CF	na	na
chr10	20738021	20738279	259	SUBTEL_sa	na	na
chr10	20738285	20738403	119	Sat5CF	stalk	na
chr10	20738296	20738426	131	Sat5CF	stalk	na
chr10	20913306	20913728	423	Sat91CF	na	na
chr10	20913439	20913763	325	Sat39CF	na	na
chr10	21007751	21007769	19	Sat38CF	na	na
chr10	21134616	21135078	463	Sat104CF	na	na
chr10	21326916	21327115	200	Sat52CF	na	na

chr10	21327182	21327276	95	Sat52CF	na	na
chr10	21605904	21606114	211	Sat50CF	na	na
chr10	21764832	21765264	433	Sat4CF	lred	na
chr10	21870720	21871282	563	Sat124CF	na	na
chr10	21958637	21959612	976	Sat97CF	na	na
chr10	22061615	22061778	164	Sat5CF	stalk	na
chr10	23619507	23619776	270	Sat28CF	na	na
chr10	23869701	23869782	82	Sat88CF	na	na
chr10	24003733	24003790	58	Sat7CF	lime	na
chr10	24215061	24215405	345	Sat30CF	na	na
chr10	28424682	28424860	179	Sat95CF	na	na
chr10	28770729	28770822	94	Sat125CF	na	na
chr10	30500661	30500764	104	Sat13CF	na	na
chr10	31795115	31796167	1053	Sat99CF	na	na
chr10	37150427	37150585	159	Carsat2	orange	na
chr10	37150798	37151371	574	Carsat2	orange	na
chr10	37153666	37155454	1789	Carsat2	orange	na
chr10	37155489	37156478	990	Carsat2	orange	na
chr10	37156922	37157313	392	Carsat2	orange	na
chr10	37157340	37159280	1941	Carsat2	orange	na
chr10	37161896	37162224	329	Carsat2	orange	na
chr10	37181202	37181691	490	SAT1_CF	blue	na
chr10	37195616	37197906	2291	CarSat1	red	na
chr10	37198082	37198909	828	CarSat1	red	na
chr10	38313875	38313941	67	Sat45CF	na	na
chr10	38426662	38426692	31	Sat58CF	na	na
chr10	38828455	38828517	63	Sat26CF	na	na
chr10	40040466	40040518	53	Sat27CF	na	na
chr10	40918746	40918852	107	Sat95CF	na	na
chr10	42380155	42380282	128	Sat27CF	na	na
chr10	42957621	42957693	73	Sat154CF	na	na
chr10	43412297	43412344	48	Sat95CF	na	na
chr10	43737403	43737473	71	Sat98CF	na	na
chr10	45015113	45015386	274	Sat19CF	na	na
chr10	45015184	45015411	228	Sat19CF	na	na
chr10	45630565	45630614	50	Sat164CF	na	na
chr10	47379462	47379514	53	Sat98CF	na	na
chr10	48852810	48852859	50	Sat12CF	na	na
chr10	48852829	48852900	72	Sat12CF	na	na
chr10	49776716	49776793	78	Sat83CF	na	na
chr10	51630151	51630209	59	Sat95CF	na	na
chr10	57014876	57015388	513	Sat3CF	optpurple	na

chr10	57014965	57015388	424	Sat3CF	optpurple	na
chr10	57314841	57314882	42	Sat36CF	na	na
chr10	58967197	58967286	90	Sat153CF	na	na
chr10	59281460	59281548	89	Sat17CF	na	na
chr10	59282433	59282520	88	Sat17CF	na	na
chr10	63745703	63745780	78	Carsat3	green	na
chr10	67538181	67538571	391	Sat97CF	na	na
chr10	67658713	67659291	579	Sat19CF	na	na
chr10	68062999	68063073	75	Sat173CF	na	na
chr10	70331386	70331494	109	Sat24CF	na	na
chr10	70456777	70457116	340	Sat81CF	na	na
chr10	71212045	71212189	145	Sat50CF	na	na
chr10	71493837	71494401	565	Sat74CF	na	na
chr10	71731869	71732114	246	Sat79CF	na	na
chr10	71731915	71732143	229	Sat57CF	na	na
chr10	71872754	71873818	1065	Sat6CF	select	na
chr10	72015728	72016116	389	Sat52CF	na	na
chr10	72417808	72417858	51	Sat12CF	na	na
chr10	72473586	72473750	165	Carsat2	orange	na
chr10	72476204	72476285	82	Sat2CF	purple	na
chr10	72476220	72476287	68	Sat2CF	purple	na
chr11	3000259	3000664	406	CarSat1	red	pericen
chr11	3000845	3001233	389	CarSat1	red	pericen
chr11	3001254	3002275	1022	CarSat1	red	pericen
chr11	3002289	3002835	547	CarSat1	red	pericen
chr11	3003159	3003800	642	CarSat1	red	pericen
chr11	3003846	3004612	767	CarSat1	red	pericen
chr11	3004771	3005192	422	CarSat1	red	pericen
chr11	3013115	3013705	591	CarSat1	red	pericen
chr11	3013892	3013991	100	CarSat1	red	pericen
chr11	3014009	3015429	1421	CarSat1	red	pericen
chr11	3021038	3021200	163	Sat2CF	purple	pericen
chr11	3021065	3022121	1057	Sat2CF	purple	pericen
chr11	3022348	3023756	1409	SAT1_CF	blue	pericen
chr11	3023785	3024929	1145	CarSat1	red	pericen
chr11	3024950	3025687	738	CarSat1	red	pericen
chr11	3056872	3057038	167	Sat2CF	purple	pericen
chr11	3057294	3057782	489	Sat2CF	purple	pericen
chr11	3057784	3059187	1404	CarSat1	red	pericen
chr11	3059355	3059967	613	CarSat1	red	pericen
chr11	3060000	3060417	418	CarSat1	red	pericen
chr11	3061009	3062720	1712	Carsat2	orange	pericen

chr11	3062735	3063824	1090	Carsat2	orange	pericen
chr11	3063835	3064953	1119	Carsat2	orange	pericen
chr11	3064965	3067687	2723	Carsat2	orange	pericen
chr11	3067799	3068346	548	Carsat2	orange	pericen
chr11	3068938	3069194	257	Carsat2	orange	pericen
chr11	3081770	3082518	749	Carsat2	orange	pericen
chr11	3082561	3083887	1327	Carsat2	orange	pericen
chr11	3083925	3085026	1102	Carsat2	orange	pericen
chr11	3176796	3179007	2212	Carsat3	green	pericen
chr11	4837346	4837431	86	SUBTEL_sa	na	pericen
chr11	5273788	5273878	91	Sat89CF	na	na
chr11	5282966	5283211	246	CarSat1	red	na
chr11	5522751	5523861	1111	Carsat3	green	na
chr11	5546129	5547369	1241	Carsat3	green	na
chr11	5602210	5602725	516	Carsat3	green	na
chr11	5602814	5602943	130	Carsat3	green	na
chr11	5603120	5603295	176	Carsat3	green	na
chr11	5610151	5611252	1102	Carsat3	green	na
chr11	5637177	5638273	1097	Carsat3	green	na
chr11	5684442	5685666	1225	Carsat3	green	na
chr11	5919716	5919869	154	Carsat3	green	na
chr11	5919884	5921197	1314	Carsat3	green	na
chr11	9097016	9098050	1035	Sat174CF	na	na
chr11	9097957	9098062	106	Sat174CF	na	na
chr11	9799559	9799611	53	Sat7CF	lime	na
chr11	10764671	10764699	29	Sat156CF	na	na
chr11	17021209	17022290	1082	Sat148CF	na	na
chr11	17255787	17256042	256	Sat17CF	na	na
chr11	25374550	25374666	117	SAT1_CF	blue	na
chr11	28750097	28750137	41	Sat24CF	na	na
chr11	29816096	29816590	495	Carsat2	orange	na
chr11	29816955	29817353	399	Carsat2	orange	na
chr11	29838113	29839239	1127	Carsat2	orange	na
chr11	29839264	29839575	312	Carsat2	orange	na
chr11	29839770	29840032	263	Carsat2	orange	na
chr11	29840044	29841502	1459	Carsat2	orange	na
chr11	29841797	29842402	606	Carsat2	orange	na
chr11	29842434	29845639	3206	Carsat2	orange	na
chr11	29845796	29846667	872	Carsat2	orange	na
chr11	29846878	29847337	460	Carsat2	orange	na
chr11	29847528	29847687	160	Carsat2	orange	na
chr11	29847999	29848144	146	Carsat2	orange	na

chr11	29848349	29848637	289	Carsat2	orange	na
chr11	29848839	29848903	65	Carsat2	orange	na
chr11	29851482	29852138	657	Carsat2	orange	na
chr11	29852362	29854617	2256	Carsat2	orange	na
chr11	29854802	29855692	891	Carsat2	orange	na
chr11	29855992	29856647	656	Carsat2	orange	na
chr11	29856798	29857206	409	Carsat2	orange	na
chr11	29857826	29858268	443	Carsat2	orange	na
chr11	29859174	29860049	876	Carsat2	orange	na
chr11	29860128	29860459	332	Carsat2	orange	na
chr11	29864056	29866130	2075	Carsat2	orange	na
chr11	29866235	29868295	2061	Carsat2	orange	na
chr11	29868492	29869583	1092	Carsat2	orange	na
chr11	29869812	29871540	1729	Carsat2	orange	na
chr11	29871567	29872679	1113	Carsat2	orange	na
chr11	29872698	29873705	1008	Carsat2	orange	na
chr11	33707410	33707463	54	Sat45CF	na	na
chr11	34037161	34037270	110	Carsat3	green	na
chr11	34286400	34286526	127	Carsat3	green	na
chr11	36850653	36850729	77	Sat154CF	na	na
chr11	39488633	39488689	57	Sat88CF	na	na
chr11	40327916	40328190	275	Sat2CF	purple	na
chr11	41981950	41982017	68	Sat67CF	na	na
chr11	43577888	43578326	439	Sat75CF	na	na
chr11	43577904	43578091	188	Sat75CF	na	na
chr11	43577941	43579386	1446	Sat75CF	na	na
chr11	43578179	43579522	1344	Sat75CF	na	na
chr11	43725458	43726044	587	Sat75CF	na	na
chr11	43726203	43726458	256	Sat75CF	na	na
chr11	43976944	43976995	52	Sat33CF	na	na
chr11	47148638	47149649	1012	Sat185CF	na	na
chr11	48031402	48031478	77	Sat27CF	na	na
chr11	49136157	49136221	65	Sat107CF	na	na
chr11	50678043	50678088	46	Sat89CF	na	na
chr11	55234857	55234916	60	Sat19CF	na	na
chr11	55415393	55415674	282	Sat86CF	na	na
chr11	55415578	55415703	126	Sat86CF	na	na
chr11	61168411	61169812	1402	Carsat3	green	na
chr11	63939363	63939425	63	Sat116CF	na	na
chr11	64503727	64503793	67	SUBTEL_sa	na	na
chr11	66603051	66603366	316	Carsat2	orange	na
chr11	66937441	66937485	45	Sat155CF	na	na

chr11	69205315	69205454	140	Carsat3	green	na
chr11	69205545	69206586	1042	Carsat3	green	na
chr11	70426276	70427364	1089	Carsat3	green	na
chr11	70445975	70447089	1115	Carsat3	green	na
chr11	70472429	70473558	1130	Carsat3	green	na
chr11	70552255	70552399	145	Sat98CF	na	na
chr11	75815793	75815975	183	Sat52CF	na	na
chr11	76954939	76956270	1332	Sat81CF	na	na
chr11	76955980	76956306	327	Sat81CF	na	na
chr11	76963548	76963739	192	Sat69CF	na	na
chr12	3011881	3012190	310	Sat2CF	purple	pericen
chr12	3012537	3012887	351	Sat2CF	purple	pericen
chr12	3012541	3012850	310	Sat2CF	purple	pericen
chr12	3017337	3017835	499	SAT1_CF	blue	pericen
chr12	3020063	3021082	1020	CarSat1	red	pericen
chr12	3021114	3022050	937	CarSat1	red	pericen
chr12	3285634	3286151	518	Carsat2	orange	pericen
chr12	4191218	4191275	58	Sat30CF	na	pericen
chr12	4648661	4648881	221	Sat7CF	lime	pericen
chr12	5094937	5094983	47	Sat165CF	na	na
chr12	5713057	5713139	83	Sat179CF	na	na
chr12	5914290	5914384	95	Carsat3	green	na
chr12	6856555	6856674	120	Sat5CF	stalk	na
chr12	8116195	8116239	45	Sat10CF	na	na
chr12	8886180	8886297	118	Sat12CF	na	na
chr12	8886204	8886345	142	Sat12CF	na	na
chr12	8908056	8908122	67	Sat18CF	na	na
chr12	9567893	9567967	75	Sat12CF	na	na
chr12	9740747	9740845	99	Sat12CF	na	na
chr12	9740758	9740868	111	Sat12CF	na	na
chr12	9741005	9741130	126	Sat12CF	na	na
chr12	9741023	9741160	138	Sat12CF	na	na
chr12	10172826	10172887	62	Sat35CF	na	na
chr12	11100525	11100697	173	Sat12CF	na	na
chr12	11979641	11979693	53	Sat76CF	na	na
chr12	12050064	12050357	294	Sat12CF	na	na
chr12	12109700	12109794	95	Sat12CF	na	na
chr12	12265303	12265402	100	Sat12CF	na	na
chr12	12265460	12265680	221	Sat12CF	na	na
chr12	12265736	12265879	144	Sat12CF	na	na
chr12	12266243	12266441	199	Sat12CF	na	na
chr12	12468917	12469021	105	Sat50CF	na	na

chr12	12477056	12477264	209	Sat12CF	na	na
chr12	12534237	12534476	240	Sat12CF	na	na
chr12	12960525	12960712	188	Sat12CF	na	na
chr12	12960528	12960825	298	Sat12CF	na	na
chr12	13249122	13249163	42	Sat86CF	na	na
chr12	13639338	13639417	80	Sat38CF	na	na
chr12	14312328	14312528	201	Sat12CF	na	na
chr12	14312587	14312707	121	Sat12CF	na	na
chr12	14313461	14313623	163	Sat12CF	na	na
chr12	15163503	15163597	95	Sat63CF	na	na
chr12	15239596	15239687	92	Sat12CF	na	na
chr12	15265323	15265422	100	Sat12CF	na	na
chr12	15265326	15265440	115	Sat12CF	na	na
chr12	15373513	15373640	128	Sat12CF	na	na
chr12	15424128	15424305	178	Sat12CF	na	na
chr12	15424138	15424282	145	Sat12CF	na	na
chr12	15474999	15475193	195	Sat12CF	na	na
chr12	15475035	15475194	160	Sat12CF	na	na
chr12	15475389	15475501	113	Sat12CF	na	na
chr12	15493736	15493916	181	Sat12CF	na	na
chr12	15493738	15493986	249	Sat12CF	na	na
chr12	15494991	15495195	205	Sat12CF	na	na
chr12	15495002	15495225	224	Sat12CF	na	na
chr12	16057802	16057875	74	Sat27CF	na	na
chr12	20534833	20535037	205	Sat21CF	na	na
chr12	21567779	21567848	70	Sat57CF	na	na
chr12	23210809	23210894	86	Sat107CF	na	na
chr12	23931205	23931244	40	Sat36CF	na	na
chr12	27072415	27072528	114	SUBTEL_sa	na	na
chr12	30738598	30738678	81	Sat78CF	na	na
chr12	46185531	46185619	89	Sat88CF	na	na
chr12	46541069	46541126	58	Sat45CF	na	na
chr12	61561186	61561261	76	Sat18CF	na	na
chr12	68268120	68268438	319	Sat38CF	na	na
chr12	68325437	68325824	388	Sat24CF	na	na
chr12	69413527	69413627	101	Carsat3	green	na
chr12	71658036	71658207	172	Sat86CF	na	na
chr12	73048818	73049016	199	Sat182CF	na	na
chr12	73048819	73049046	228	Sat182CF	na	na
chr12	73060424	73061059	636	Sat38CF	na	na
chr12	73472399	73473547	1149	Sat124CF	na	na
chr12	73687256	73687720	465	Sat87CF	na	na

chr12	74490364	74490426	63	Sat14CF	na	na
chr12	74501888	74502015	128	Sat16CF	na	na
chr12	74616607	74616978	372	Sat44CF	na	na
chr12	75078509	75078844	336	Sat64CF	na	na
chr12	75078573	75080590	2018	Sat64CF	na	na
chr12	75080542	75080602	61	Sat64CF	na	na
chr12	75263979	75264142	164	Sat28CF	na	na
chr12	75414071	75414426	356	Sat43CF	na	na
chr12	75515042	75515260	219	Sat2CF	purple	na
chr13	3004284	3005170	887	Sat2CF	purple	pericen
chr13	3004839	3005207	369	Sat2CF	purple	pericen
chr13	3015092	3016101	1010	SAT1_CF	blue	pericen
chr13	3016120	3016665	546	SAT1_CF	blue	pericen
chr13	3016699	3019171	2473	SAT1_CF	blue	pericen
chr13	3019189	3020021	833	CarSat1	red	pericen
chr13	3027679	3030138	2460	CarSat1	red	pericen
chr13	3030151	3030338	188	CarSat1	red	pericen
chr13	3030527	3030983	457	CarSat1	red	pericen
chr13	4074234	4074280	47	CarSat1	red	pericen
chr13	8733409	8733609	201	SAT1_CF	blue	na
chr13	17119036	17119917	882	Sat7CF	lime	na
chr13	18230229	18230306	78	Sat27CF	na	na
chr13	19827874	19828111	238	CarSat1	red	na
chr13	23942011	23942157	147	Carsat3	green	na
chr13	23942261	23942563	303	Carsat3	green	na
chr13	24581498	24581768	271	Carsat3	green	na
chr13	24581944	24582036	93	Carsat3	green	na
chr13	26019581	26020484	904	Carsat3	green	na
chr13	26020513	26020693	181	Carsat3	green	na
chr13	32103319	32103378	60	Sat165CF	na	na
chr13	32103326	32103378	53	Sat165CF	na	na
chr13	32620227	32620281	55	Sat66CF	na	na
chr13	33487150	33487366	217	Sat4CF	lred	na
chr13	35039228	35039286	59	Sat177CF	na	na
chr13	36558518	36558560	43	Sat15CF	na	na
chr13	37527726	37527794	69	Sat5CF	stalk	na
chr13	37561181	37561404	224	Sat99CF	na	na
chr13	37561221	37561404	184	Sat99CF	na	na
chr13	38521030	38521152	123	Carsat3	green	na
chr13	38836141	38836322	182	Sat57CF	na	na
chr13	38836214	38836358	145	Sat57CF	na	na
chr13	38859032	38859181	150	Sat52CF	na	na

chr13	38886335	38886643	309	Sat153CF	na	na
chr13	38886514	38886668	155	Sat153CF	na	na
chr13	39081745	39082054	310	Sat30CF	na	na
chr13	39081855	39082054	200	Sat30CF	na	na
chr13	39081865	39082095	231	Sat30CF	na	na
chr13	39679102	39679279	178	Sat38CF	na	na
chr13	39679151	39679279	129	Sat38CF	na	na
chr13	40044455	40044702	248	Carsat3	green	na
chr13	40060578	40060968	391	Carsat3	green	na
chr13	40079345	40079844	500	Carsat3	green	na
chr13	40318673	40319828	1156	Carsat3	green	na
chr13	41048147	41048289	143	Sat72CF	na	na
chr13	41076529	41076859	331	Carsat3	green	na
chr13	41076873	41077314	442	Carsat3	green	na
chr13	41096821	41097733	913	Carsat3	green	na
chr13	41097763	41098194	432	Carsat3	green	na
chr13	41119471	41119657	187	Carsat3	green	na
chr13	41120029	41120241	213	Carsat3	green	na
chr13	41147622	41148564	943	Carsat3	green	na
chr13	41161207	41161370	164	Carsat3	green	na
chr13	41161544	41161790	247	Carsat3	green	na
chr13	41161964	41162279	316	Carsat3	green	na
chr13	41193174	41194591	1418	Carsat3	green	na
chr13	41226049	41227573	1525	Carsat3	green	na
chr13	41227630	41227921	292	Carsat3	green	na
chr13	41862795	41863139	345	Sat18CF	na	na
chr13	41911320	41911462	143	SUBTEL_sa	na	na
chr13	42352824	42352906	83	SUBTEL_sa	na	na
chr13	42602537	42602612	76	Sat93CF	na	na
chr13	42602706	42602752	47	Sat93CF	na	na
chr13	43056775	43056943	169	Sat93CF	na	na
chr13	43056798	43056943	146	Sat93CF	na	na
chr13	46672473	46672514	42	Sat36CF	na	na
chr13	46905448	46906081	634	Sat16CF	na	na
chr13	50587200	50587242	43	Sat10CF	na	na
chr13	50811321	50811436	116	Carsat3	green	na
chr13	50811449	50811664	216	Carsat3	green	na
chr13	50812480	50812628	149	Carsat3	green	na
chr13	50812659	50812874	216	Carsat3	green	na
chr13	53853132	53853180	49	Sat5CF	stalk	na
chr13	62159329	62159581	253	Sat17CF	na	na
chr13	62902929	62902999	71	Sat78CF	na	na

chr13	65090231	65090289	59	Sat81CF	na	na
chr13	66071633	66071908	276	Carsat2	orange	na
chr13	66147457	66147741	285	Carsat2	orange	na
chr13	66154177	66157164	2988	Carsat2	orange	na
chr13	66158884	66160057	1174	Carsat2	orange	na
chr13	66160090	66161163	1074	Carsat2	orange	na
chr13	66161199	66162962	1764	Carsat2	orange	na
chr13	66162993	66164326	1334	Carsat2	orange	na
chr13	66169359	66169920	562	Carsat2	orange	na
chr13	66170145	66171819	1675	Carsat2	orange	na
chr14	3299081	3300623	1543	CarSat1	red	pericen
chr14	3317694	3318009	316	SAT1_CF	blue	pericen
chr14	3894407	3894432	26	Sat52CF	na	pericen
chr14	4076949	4077198	250	Carsat2	orange	pericen
chr14	4112314	4112925	612	Carsat2	orange	pericen
chr14	4127934	4129078	1145	Carsat2	orange	pericen
chr14	4129250	4129864	615	Carsat2	orange	pericen
chr14	4504529	4504672	144	Sat2CF	purple	pericen
chr14	5225161	5225986	826	CarSat1	red	na
chr14	10978894	10978961	68	Sat9CF	na	na
chr14	14975885	14977754	1870	Carsat2	orange	na
chr14	14977767	14979241	1475	Carsat2	orange	na
chr14	14979282	14980153	872	Carsat2	orange	na
chr14	15102161	15102258	98	Sat7CF	lime	na
chr14	15102771	15103890	1120	Carsat3	green	na
chr14	15354672	15354724	53	Sat65CF	na	na
chr14	21335038	21335068	31	Sat16CF	na	na
chr14	23365883	23365974	92	Carsat3	green	na
chr14	25267318	25268098	781	Sat10CF	na	na
chr14	25267508	25268265	758	Sat10CF	na	na
chr14	25267783	25268899	1117	Sat10CF	na	na
chr14	25267799	25268141	343	Sat10CF	na	na
chr14	25267907	25269126	1220	Sat10CF	na	na
chr14	25268591	25269761	1171	Sat10CF	na	na
chr14	25268832	25269876	1045	Sat10CF	na	na
chr14	25269128	25270319	1192	Sat10CF	na	na
chr14	25269981	25270695	715	Sat10CF	na	na
chr14	25272440	25272502	63	Sat10CF	na	na
chr14	35403252	35403294	43	Sat27CF	na	na
chr14	37167383	37167418	36	Sat23CF	na	na
chr14	38767320	38767533	214	Sat17CF	na	na
chr14	39299267	39299545	279	Carsat2	orange	na

chr14	40578247	40578328	82	Sat27CF	na	na
chr14	40900865	40904124	3260	Sat7CF	lime	na
chr14	40903358	40904236	879	Sat7CF	lime	na
chr14	43269123	43269196	74	SUBTEL_sa	na	na
chr14	45573499	45573542	44	Sat104CF	na	na
chr14	49534481	49534544	64	Sat63CF	na	na
chr14	50015556	50015654	99	Carsat3	green	na
chr14	50164890	50164988	99	Sat72CF	na	na
chr14	51233291	51233529	239	Sat18CF	na	na
chr14	52572816	52572887	72	Sat17CF	na	na
chr14	52831830	52831923	94	Carsat3	green	na
chr14	52832457	52832597	141	Carsat3	green	na
chr14	54947531	54947682	152	Sat175CF	na	na
chr14	54947554	54948660	1107	Sat175CF	na	na
chr14	54948529	54948681	153	Sat175CF	na	na
chr14	59874306	59875091	786	Sat119CF	na	na
chr14	61890010	61890261	252	Sat87CF	na	na
chr14	61890015	61890388	374	Sat13CF	na	na
chr14	61936230	61937393	1164	Sat167CF	na	na
chr14	62813741	62814535	795	Sat47CF	na	na
chr14	62868764	62869481	718	Sat177CF	na	na
chr14	62868845	62869847	1003	Sat177CF	na	na
chr14	62909796	62910011	216	Sat153CF	na	na
chr14	63038368	63038599	232	Sat111CF	na	na
chr15	3013850	3014222	373	Sat2CF	purple	pericen
chr15	3157667	3158384	718	Sat7CF	lime	pericen
chr15	3519801	3519981	181	Carsat3	green	pericen
chr15	3520155	3520398	244	Carsat3	green	pericen
chr15	4304150	4304213	64	Sat21CF	na	pericen
chr15	4703456	4703737	282	Sat3CF	optpurple	pericen
chr15	5428139	5428252	114	Carsat3	green	na
chr15	5443377	5443509	133	Carsat3	green	na
chr15	5443557	5443900	344	Carsat3	green	na
chr15	5460542	5461344	803	Carsat3	green	na
chr15	5481261	5482052	792	Carsat3	green	na
chr15	5497476	5497613	138	Carsat3	green	na
chr15	5516317	5516495	179	Carsat3	green	na
chr15	5516510	5517085	576	Carsat3	green	na
chr15	5540282	5541058	777	Carsat3	green	na
chr15	5843784	5843847	64	Sat15CF	na	na
chr15	8428774	8428858	85	Sat41CF	na	na
chr15	8432900	8433719	820	Sat42CF	na	na

chr15	8746160	8746235	76	Sat12CF	na	na
chr15	8768916	8769328	413	Sat18CF	na	na
chr15	8768965	8769328	364	Sat18CF	na	na
chr15	9111522	9111733	212	Sat171CF	na	na
chr15	9458634	9458703	70	Sat12CF	na	na
chr15	9618697	9619096	400	Carsat3	green	na
chr15	9619276	9619844	569	Carsat3	green	na
chr15	9656681	9656836	156	Sat17CF	na	na
chr15	10208886	10209278	393	Sat4CF	lred	na
chr15	10209831	10210003	173	Sat4CF	lred	na
chr15	10733410	10734033	624	Sat86CF	na	na
chr15	10810543	10810788	246	Sat120CF	na	na
chr15	10837776	10838265	490	Sat176CF	na	na
chr15	11029946	11030364	419	Sat47CF	na	na
chr15	11551804	11552135	332	Sat2CF	purple	na
chr15	11551926	11552122	197	Sat2CF	purple	na
chr15	11555987	11556073	87	SAT1_CF	blue	na
chr15	11577028	11577344	317	Carsat2	orange	na
chr15	11583177	11584028	852	CarSat1	red	na
chr15	11592289	11592422	134	CarSat1	red	na
chr15	11603798	11604195	398	Carsat2	orange	na
chr15	11614634	11614767	134	CarSat1	red	na
chr15	14830389	14830434	46	Carsat2	orange	na
chr15	14935970	14936033	64	Sat65CF	na	na
chr15	15299794	15300227	434	Sat49CF	na	na
chr15	15347654	15347748	95	Sat88CF	na	na
chr15	15861747	15861809	63	Sat4CF	lred	na
chr15	15861810	15862918	1109	Sat4CF	lred	na
chr15	16089668	16089761	94	Sat105CF	na	na
chr15	16863840	16863935	96	Sat179CF	na	na
chr15	18229806	18229862	57	Sat66CF	na	na
chr15	19117206	19117434	229	Sat17CF	na	na
chr15	20036504	20036850	347	Sat2CF	purple	na
chr15	20036521	20036827	307	Sat2CF	purple	na
chr15	20036592	20036786	195	Sat2CF	purple	na
chr15	20036608	20036810	203	Sat2CF	purple	na
chr15	20061982	20062256	275	Sat2CF	purple	na
chr15	20062017	20062325	309	Sat2CF	purple	na
chr15	20062124	20062364	241	Sat2CF	purple	na
chr15	20170497	20171370	874	Sat7CF	lime	na
chr15	21224262	21224422	161	Sat38CF	na	na
chr15	21224281	21224451	171	Sat44CF	na	na

chr15	25081138	25081471	334	Sat39CF	na	na
chr15	39669668	39669857	190	Sat17CF	na	na
chr15	41177645	41177755	111	Sat123CF	na	na
chr15	42767664	42767767	104	SUBTEL_sa	na	na
chr15	43555751	43555826	76	Sat143CF	na	na
chr15	43555759	43557906	2148	Sat143CF	na	na
chr15	45680456	45680720	265	SAT1_CF	blue	na
chr15	45701178	45702302	1125	Carsat2	orange	na
chr15	45702387	45703165	779	Carsat2	orange	na
chr15	45712720	45713301	582	Carsat2	orange	na
chr15	45713042	45713579	538	CarSat1	red	na
chr15	45713612	45716213	2602	CarSat1	red	na
chr15	45723672	45725271	1600	Carsat2	orange	na
chr15	45728380	45728942	563	Carsat2	orange	na
chr15	45729125	45729283	159	Carsat2	orange	na
chr15	48509523	48509562	40	Sat26CF	na	na
chr15	58985248	58985319	72	Sat42CF	na	na
chr15	64231612	64231654	43	Sat139CF	na	na
chr15	65801491	65801564	74	Sat47CF	na	na
chr15	66460358	66460719	362	Sat69CF	na	na
chr15	66481193	66481308	116	Sat21CF	na	na
chr15	66799958	66800308	351	Sat122CF	na	na
chr15	66809823	66810146	324	Sat19CF	na	na
chr15	66860536	66860589	54	Sat14CF	na	na
chr15	66994773	66994963	191	Sat74CF	na	na
chr15	66994873	66994971	99	Sat74CF	na	na
chr16	3017589	3018523	935	Sat2CF	purple	pericen
chr16	3018075	3018582	508	Sat2CF	purple	pericen
chr16	3062366	3063369	1004	Sat29CF	na	pericen
chr16	3063160	3063395	236	Sat29CF	na	pericen
chr16	3075629	3075835	207	Sat23CF	na	pericen
chr16	3119784	3120160	377	Sat79CF	na	pericen
chr16	3424229	3424336	108	Sat5CF	stalk	pericen
chr16	3701985	3702068	84	Sat49CF	na	pericen
chr16	3730256	3730372	117	Sat24CF	na	pericen
chr16	4202958	4203089	132	Sat14CF	na	pericen
chr16	4277035	4277575	541	Sat7CF	lime	pericen
chr16	4277043	4277680	638	Sat7CF	lime	pericen
chr16	4477112	4477480	369	Sat7CF	lime	pericen
chr16	4684997	4686102	1106	Sat7CF	lime	pericen
chr16	4714538	4715189	652	Sat7CF	lime	pericen
chr16	4760402	4760906	505	Sat7CF	lime	pericen

chr16	4760507	4760903	397	Sat7CF	lime	pericen
chr16	4760820	4760919	100	Sat7CF	lime	pericen
chr16	5117879	5117918	40	Sat130CF	na	na
chr16	5527425	5527472	48	Sat145CF	na	na
chr16	5638036	5638125	90	Sat36CF	na	na
chr16	10511119	10511929	811	Sat41CF	na	na
chr16	13552378	13552747	370	Sat7CF	lime	na
chr16	13578319	13578689	371	Sat7CF	lime	na
chr16	13629402	13629539	138	Sat7CF	lime	na
chr16	15335825	15335894	70	Sat12CF	na	na
chr16	16408200	16408460	261	Sat7CF	lime	na
chr16	16408200	16408460	261	Sat7CF	lime	na
chr16	16841656	16842761	1106	Sat7CF	lime	na
chr16	16857395	16858348	954	Sat7CF	lime	na
chr16	16894931	16896038	1108	Sat7CF	lime	na
chr16	17121746	17121882	137	Carsat3	green	na
chr16	17122329	17122554	226	Carsat3	green	na
chr16	17486251	17486413	163	Carsat3	green	na
chr16	17638182	17638388	207	Carsat3	green	na
chr16	17659198	17659415	218	Carsat3	green	na
chr16	18772439	18772582	144	Sat14CF	na	na
chr16	20000734	20000829	96	Sat109CF	na	na
chr16	20046091	20046148	58	Sat95CF	na	na
chr16	20383689	20384897	1209	Sat108CF	na	na
chr16	20384667	20384944	278	Sat97CF	na	na
chr16	21028759	21028828	70	Sat48CF	na	na
chr16	21717670	21717860	191	Sat86CF	na	na
chr16	22522817	22522962	146	Sat96CF	na	na
chr16	22522829	22523009	181	Sat96CF	na	na
chr16	22820060	22820218	159	Sat120CF	na	na
chr16	23205471	23205785	315	Sat53CF	na	na
chr16	23205562	23205774	213	Sat31CF	na	na
chr16	23205598	23205774	177	Sat31CF	na	na
chr16	23205635	23205815	181	Sat31CF	na	na
chr16	23369100	23369228	129	Sat16CF	na	na
chr16	23503338	23503595	258	Sat14CF	na	na
chr16	23503391	23503609	219	Sat14CF	na	na
chr16	23913511	23913567	57	Sat67CF	na	na
chr16	24049095	24050014	920	Sat7CF	lime	na
chr16	24050209	24053043	2835	Sat7CF	lime	na
chr16	24167885	24169613	1729	Carsat2	orange	na
chr16	24173082	24173828	747	Carsat2	orange	na

chr16	24175684	24176537	854	Carsat2	orange	na
chr16	24177548	24178859	1312	Sat2CF	purple	na
chr16	24177794	24179152	1359	Sat2CF	purple	na
chr16	24178341	24179699	1359	Sat2CF	purple	na
chr16	24179387	24180721	1335	Sat2CF	purple	na
chr16	24179726	24181060	1335	Sat2CF	purple	na
chr16	24179865	24181194	1330	Sat2CF	purple	na
chr16	24180476	24181279	804	Sat2CF	purple	na
chr16	24201006	24201046	41	SAT1_CF	blue	na
chr16	24201397	24201672	276	SAT1_CF	blue	na
chr16	24228259	24229226	968	CarSat1	red	na
chr16	24229248	24229963	716	CarSat1	red	na
chr16	24229982	24231021	1040	CarSat1	red	na
chr16	32420444	32420480	37	Sat33CF	na	na
chr16	33136981	33137670	690	Sat7CF	lime	na
chr16	33137457	33137700	244	Sat7CF	lime	na
chr16	41562684	41563035	352	Sat7CF	lime	na
chr16	42947608	42947959	352	Sat7CF	lime	na
chr16	43014376	43014727	352	Sat7CF	lime	na
chr16	43017374	43017650	277	Sat7CF	lime	na
chr16	44351001	44351393	393	Sat7CF	lime	na
chr16	44351042	44351380	339	Sat7CF	lime	na
chr16	44378224	44378606	383	Sat7CF	lime	na
chr16	50263496	50264172	677	Sat7CF	lime	na
chr16	50273389	50273785	397	Sat7CF	lime	na
chr16	50273582	50273823	242	Sat7CF	lime	na
chr16	50291215	50291892	678	Sat7CF	lime	na
chr16	53666080	53666639	560	Sat7CF	lime	na
chr16	53998626	53999187	562	Sat7CF	lime	na
chr16	53998687	53999188	502	Sat7CF	lime	na
chr16	54540257	54540311	55	Sat63CF	na	na
chr16	54605418	54606089	672	Sat7CF	lime	na
chr16	54618679	54619353	675	Sat7CF	lime	na
chr16	55239587	55240256	670	Sat7CF	lime	na
chr16	55239602	55240161	560	Sat7CF	lime	na
chr16	56344376	56344579	204	Sat168CF	na	na
chr16	56545950	56546304	355	Sat7CF	lime	na
chr16	56546100	56546343	244	Sat7CF	lime	na
chr16	56948284	56948817	534	Carsat3	green	na
chr16	56948854	56948986	133	Carsat3	green	na
chr16	56984092	56984629	538	Carsat3	green	na
chr16	56984660	56984769	110	Carsat3	green	na

chr16	57002788	57003141	354	Sat7CF	lime	na
chr16	57002937	57003180	244	Sat7CF	lime	na
chr16	57181927	57182171	245	Sat94CF	na	na
chr16	57182102	57182191	90	Sat94CF	na	na
chr16	57402178	57402959	782	Sat34CF	na	na
chr16	57402294	57403040	747	Sat34CF	na	na
chr16	57762538	57762595	58	Sat98CF	na	na
chr16	58181555	58181718	164	Sat57CF	na	na
chr16	58181632	58181787	156	Sat57CF	na	na
chr16	58384593	58386112	1520	Sat107CF	na	na
chr16	59343139	59343345	207	Sat157CF	na	na
chr16	60118331	60118397	67	Sat152CF	na	na
chr16	61767620	61768712	1093	Sat141CF	na	na
chr16	61990969	61991273	305	Sat7CF	lime	na
chr16	61991067	61991304	238	Sat7CF	lime	na
chr16	62037819	62038488	670	Sat7CF	lime	na
chr16	62256782	62257451	670	Sat7CF	lime	na
chr16	62330595	62330880	286	Sat7CF	lime	na
chr16	62330674	62330911	238	Sat7CF	lime	na
chr16	62349442	62350105	664	Sat7CF	lime	na
chr16	62411334	62411638	305	Sat7CF	lime	na
chr16	62411432	62411669	238	Sat7CF	lime	na
chr16	62431597	62431970	374	Sat7CF	lime	na
chr16	62431636	62431979	344	Sat7CF	lime	na
chr17	3025673	3025870	198	Sat2CF	purple	pericen
chr17	3127500	3127797	298	Sat44CF	na	pericen
chr17	3259360	3259501	142	Sat132CF	na	pericen
chr17	3299643	3299804	162	Sat16CF	na	pericen
chr17	3300035	3300281	247	Sat16CF	na	pericen
chr17	3319755	3320069	315	Sat49CF	na	pericen
chr17	3326552	3326870	319	Sat19CF	na	pericen
chr17	3336110	3336503	394	Sat24CF	na	pericen
chr17	3378970	3379655	686	Sat52CF	na	pericen
chr17	3443251	3443297	47	Sat19CF	na	pericen
chr17	3465792	3466005	214	Sat84CF	na	pericen
chr17	3499126	3499752	627	Sat42CF	na	pericen
chr17	3614753	3614892	140	Sat53CF	na	pericen
chr17	3614768	3616073	1306	Sat53CF	na	pericen
chr17	3699469	3699510	42	Sat5CF	stalk	pericen
chr17	3819169	3819273	105	Sat13CF	na	pericen
chr17	3840653	3841021	369	Sat60CF	na	pericen
chr17	3924979	3925911	933	Sat34CF	na	pericen

chr17	4151311	4151808	498	Sat42CF	na	pericen
chr17	4419125	4419294	170	Sat5CF	stalk	pericen
chr17	4459732	4459899	168	Sat30CF	na	pericen
chr17	4523195	4523328	134	Sat57CF	na	pericen
chr17	4523330	4524533	1204	Sat57CF	na	pericen
chr17	4524534	4524575	42	Sat57CF	na	pericen
chr17	4597257	4597779	523	Sat49CF	na	pericen
chr17	4597486	4597894	409	Sat49CF	na	pericen
chr17	4668911	4669410	500	Sat28CF	na	pericen
chr17	5572118	5572305	188	Sat17CF	na	na
chr17	5830795	5831038	244	Sat72CF	na	na
chr17	5831788	5832103	316	Sat72CF	na	na
chr17	5953254	5953409	156	Sat5CF	stalk	na
chr17	6255239	6255390	152	Sat5CF	stalk	na
chr17	6746208	6746323	116	Sat83CF	na	na
chr17	6746212	6746391	180	Sat106CF	na	na
chr17	7496823	7496897	75	Sat42CF	na	na
chr17	8091742	8091904	163	Sat50CF	na	na
chr17	8561302	8561397	96	Sat72CF	na	na
chr17	8623152	8623234	83	Sat118CF	na	na
chr17	8782738	8783328	591	Sat4CF	lred	na
chr17	10124011	10124045	35	Sat74CF	na	na
chr17	10281805	10281868	64	Sat3CF	optpurple	na
chr17	10897165	10897237	73	Sat72CF	na	na
chr17	11228269	11228375	107	Sat83CF	na	na
chr17	13962264	13962626	363	Sat30CF	na	na
chr17	14693425	14693690	266	SAT1_CF	blue	na
chr17	17484532	17484597	66	Carsat3	green	na
chr17	18210504	18210547	44	Sat50CF	na	na
chr17	18608153	18608491	339	SAT1_CF	blue	na
chr17	21025833	21025923	91	Sat19CF	na	na
chr17	23570083	23570125	43	Sat103CF	na	na
chr17	24069586	24069625	40	Sat99CF	na	na
chr17	24487130	24488163	1034	Sat36CF	na	na
chr17	24595126	24595997	872	Sat36CF	na	na
chr17	24606139	24607997	1859	Sat36CF	na	na
chr17	24607998	24608130	133	Sat36CF	na	na
chr17	24608349	24608600	252	Sat36CF	na	na
chr17	24608365	24608578	214	Sat36CF	na	na
chr17	24724337	24724838	502	Sat36CF	na	na
chr17	25445728	25445990	263	CarSat1	red	na
chr17	26655510	26655598	89	Sat46CF	na	na

chr17	27741323	27741468	146	Sat57CF	na	na
chr17	31982984	31983033	50	Sat36CF	na	na
chr17	33340360	33340769	410	Carsat3	green	na
chr17	33483589	33483638	50	Sat12CF	na	na
chr17	33868993	33869049	57	Sat169CF	na	na
chr17	36337317	36337440	124	Carsat3	green	na
chr17	37049077	37049157	81	Sat43CF	na	na
chr17	37102356	37102448	93	CarSat1	red	na
chr17	37962463	37963432	970	Carsat3	green	na
chr17	37978502	37979244	743	Carsat3	green	na
chr17	37994277	37995078	802	Carsat3	green	na
chr17	38024200	38025246	1047	Carsat3	green	na
chr17	38025284	38025749	466	Carsat3	green	na
chr17	38562867	38562983	117	Sat55CF	na	na
chr17	40811729	40811812	84	Sat139CF	na	na
chr17	42521877	42521950	74	Sat32CF	na	na
chr17	43523214	43523386	173	Carsat3	green	na
chr17	47587423	47587983	561	Carsat3	green	na
chr17	48096251	48096363	113	Sat17CF	na	na
chr17	52516013	52517835	1823	Sat26CF	na	na
chr17	52516212	52519239	3028	Sat26CF	na	na
chr17	52516592	52519521	2930	Sat26CF	na	na
chr17	52516932	52519944	3013	Sat26CF	na	na
chr17	52517340	52520367	3028	Sat26CF	na	na
chr17	52517763	52520790	3028	Sat26CF	na	na
chr17	52518183	52521219	3037	Sat26CF	na	na
chr17	52518603	52521642	3040	Sat26CF	na	na
chr17	52520436	52521783	1348	Sat26CF	na	na
chr17	52921791	52921952	162	Sat80CF	na	na
chr17	53786194	53786306	113	Sat12CF	na	na
chr17	53921033	53921111	79	Sat12CF	na	na
chr17	54074411	54074586	176	Sat12CF	na	na
chr17	54550079	54550984	906	Carsat2	orange	na
chr17	54571571	54577418	5848	Carsat2	orange	na
chr17	54577853	54579865	2013	Carsat2	orange	na
chr17	54579914	54580232	319	Carsat2	orange	na
chr17	54580300	54581776	1477	Carsat2	orange	na
chr17	54582041	54582259	219	Carsat2	orange	na
chr17	54582304	54583288	985	Carsat2	orange	na
chr17	54593796	54594363	568	Carsat2	orange	na
chr17	54752922	54753038	117	CarSat1	red	na
chr17	54753237	54753899	663	CarSat1	red	na

chr17	55172526	55172672	147	CarSat1	red	na
chr17	55989829	55990217	389	Sat108CF	na	na
chr17	55989833	55990271	439	Sat108CF	na	na
chr17	58952463	58952509	47	CarSat1	red	na
chr17	62251548	62251735	188	SAT1_CF	blue	na
chr17	63360979	63361059	81	Sat137CF	na	na
chr17	63644793	63644944	152	Sat179CF	na	na
chr17	63803000	63803048	49	Sat73CF	na	na
chr17	64173589	64173750	162	Sat133CF	na	na
chr17	64173611	64173843	233	Sat133CF	na	na
chr17	64173803	64175156	1354	Sat133CF	na	na
chr17	64270384	64270700	317	Sat46CF	na	na
chr17	64270417	64272412	1996	Sat46CF	na	na
chr17	64270670	64272995	2326	Sat46CF	na	na
chr17	64271256	64273570	2315	Sat46CF	na	na
chr17	64273493	64273792	300	Sat46CF	na	na
chr17	64296035	64296227	193	Sat22CF	na	na
chr17	64296062	64301646	5585	Sat22CF	na	na
chr17	64296860	64303293	6434	Sat22CF	na	na
chr17	64298507	64304026	5520	Sat22CF	na	na
chr17	64303247	64304133	887	Sat22CF	na	na
chr17	64392059	64392149	91	Sat22CF	na	na
chr17	64392082	64392381	300	Sat66CF	na	na
chr17	64392129	64397547	5419	Sat66CF	na	na
chr17	64397663	64398989	1327	Sat46CF	na	na
chr17	65113188	65113551	364	Carsat2	orange	na
chr17	65113602	65113796	195	Carsat2	orange	na
chr17	65114038	65114142	105	Carsat2	orange	na
chr17	65123546	65123868	323	Carsat2	orange	na
chr17	65127180	65127284	105	Carsat2	orange	na
chr17	65127526	65127720	195	Carsat2	orange	na
chr17	65127761	65128283	523	Carsat2	orange	na
chr17	65526682	65526744	63	Sat88CF	na	na
chr17	66288161	66288278	118	Sat27CF	na	na
chr17	66617264	66617500	237	Sat21CF	na	na
chr17	66759009	66759054	46	Sat46CF	na	na
chr17	67105637	67105673	37	CarSat1	red	na
chr17	67105880	67107084	1205	CarSat1	red	na
chr17	67107118	67107178	61	CarSat1	red	na
chr17	67107361	67109024	1664	CarSat1	red	na
chr17	67109053	67110025	973	CarSat1	red	na
chr17	67110222	67112366	2145	CarSat1	red	na

chr17	67112785	67112810	26	CarSat1	red	na
chr17	67167062	67167945	884	Sat7CF	lime	na
chr17	67249212	67250085	874	Sat7CF	lime	na
chr18	3260802	3260986	185	Sat42CF	na	pericen
chr18	3677981	3678050	70	Sat26CF	na	pericen
chr18	3686680	3686883	204	Sat17CF	na	pericen
chr18	3784109	3784307	199	Sat30CF	na	pericen
chr18	4136273	4136497	225	Sat4CF	lred	pericen
chr18	4136307	4136507	201	Sat4CF	lred	pericen
chr18	4136791	4136927	137	Sat4CF	lred	pericen
chr18	4879606	4879850	245	CarSat1	red	pericen
chr18	4884752	4884925	174	Sat166CF	na	pericen
chr18	5074253	5074305	53	Sat5CF	stalk	na
chr18	5074259	5074315	57	Sat5CF	stalk	na
chr18	5393240	5393392	153	Sat74CF	na	na
chr18	5393249	5393410	162	Sat74CF	na	na
chr18	5581044	5583739	2696	Sat60CF	na	na
chr18	5585845	5586225	381	Sat97CF	na	na
chr18	5586438	5586527	90	Sat60CF	na	na
chr18	5839581	5840108	528	Sat51CF	na	na
chr18	6605109	6605202	94	Carsat3	green	na
chr18	6605241	6605365	125	Carsat3	green	na
chr18	6792733	6792776	44	Sat26CF	na	na
chr18	7348661	7348705	45	Sat94CF	na	na
chr18	8048528	8048582	55	Sat19CF	na	na
chr18	8048534	8048593	60	Sat19CF	na	na
chr18	8500639	8500995	357	Sat14CF	na	na
chr18	8500730	8501036	307	Sat14CF	na	na
chr18	8504438	8504729	292	Sat14CF	na	na
chr18	8504545	8504729	185	Sat14CF	na	na
chr18	8505070	8505942	873	Sat14CF	na	na
chr18	8507909	8508093	185	Sat14CF	na	na
chr18	8507978	8508265	288	Sat14CF	na	na
chr18	8508381	8508705	325	Sat14CF	na	na
chr18	8509641	8509884	244	Sat14CF	na	na
chr18	8509750	8509977	228	Sat14CF	na	na
chr18	12564738	12564778	41	Sat118CF	na	na
chr18	13115218	13115446	229	Sat34CF	na	na
chr18	14365394	14365525	132	Sat55CF	na	na
chr18	15128735	15128825	91	Sat66CF	na	na
chr18	15927614	15927911	298	Sat178CF	na	na
chr18	17501262	17501319	58	Sat18CF	na	na

chr18	18124703	18124753	51	Sat139CF	na	na
chr18	18633707	18634050	344	Sat28CF	na	na
chr18	18684816	18685022	207	Carsat3	green	na
chr18	18913003	18913098	96	Sat39CF	na	na
chr18	19262064	19262117	54	Sat154CF	na	na
chr18	19587081	19587402	322	Carsat3	green	na
chr18	26861632	26861748	117	Sat128CF	na	na
chr18	28202993	28203030	38	CarSat1	red	na
chr18	28205692	28206452	761	CarSat1	red	na
chr18	28247525	28248708	1184	SAT1_CF	blue	na
chr18	28250925	28251354	430	Sat2CF	purple	na
chr18	28250983	28251375	393	Sat2CF	purple	na
chr18	28251051	28251450	400	Sat2CF	purple	na
chr18	28295856	28296417	562	CarSat1	red	na
chr18	28296446	28297494	1049	SAT1_CF	blue	na
chr18	28297537	28299570	2034	SAT1_CF	blue	na
chr18	28299615	28300658	1044	SAT1_CF	blue	na
chr18	28303739	28303934	196	CarSat1	red	na
chr18	28305260	28305611	352	Sat2CF	purple	na
chr18	28310054	28310483	430	SAT1_CF	blue	na
chr18	28532977	28533594	618	Sat102CF	na	na
chr18	28558306	28558574	269	Sat39CF	na	na
chr18	28558336	28558570	235	Sat42CF	na	na
chr18	28622360	28622833	474	SAT1_CF	blue	na
chr18	28641528	28641968	441	Sat74CF	na	na
chr18	28651815	28652600	786	Sat179CF	na	na
chr18	29467122	29467439	318	Sat18CF	na	na
chr18	31190656	31190691	36	Sat108CF	na	na
chr18	35405478	35405514	37	Sat8CF	dgreen	na
chr18	36928025	36928144	120	Carsat2	orange	na
chr18	48287405	48288641	1237	Sat80CF	na	na
chr18	48288620	48288697	78	Sat80CF	na	na
chr18	48401736	48402682	947	Sat158CF	na	na
chr18	48402700	48403638	939	Sat158CF	na	na
chr18	48512981	48513295	315	Sat56CF	na	na
chr18	48513770	48514043	274	Sat56CF	na	na
chr18	48514256	48514556	301	Sat56CF	na	na
chr18	48515655	48516112	458	Sat73CF	na	na
chr18	48516297	48516383	87	Sat73CF	na	na
chr18	48516362	48516719	358	Sat56CF	na	na
chr18	48516824	48517065	242	Sat73CF	na	na
chr18	48517530	48518035	506	Sat73CF	na	na

chr18	48518285	48518642	358	Sat56CF	na	na
chr18	48557535	48557995	461	Sat56CF	na	na
chr18	48558202	48558541	340	Sat56CF	na	na
chr18	48558957	48559292	336	Sat56CF	na	na
chr18	48559385	48559721	337	Sat56CF	na	na
chr18	48559802	48560397	596	Sat56CF	na	na
chr18	48559944	48564076	4133	Sat56CF	na	na
chr18	48563672	48565441	1770	Sat56CF	na	na
chr18	48803963	48804124	162	Sat131CF	na	na
chr18	49005547	49005795	249	Sat49CF	na	na
chr18	49118425	49118646	222	Sat39CF	na	na
chr18	49138090	49139393	1304	Sat12CF	na	na
chr18	49138237	49140004	1768	Sat12CF	na	na
chr18	49138481	49140177	1697	Sat12CF	na	na
chr18	49231893	49232704	812	Sat59CF	na	na
chr18	49232247	49236655	4409	Sat59CF	na	na
chr18	49235780	49236930	1151	Sat59CF	na	na
chr18	49236609	49236970	362	Sat59CF	na	na
chr18	49352524	49352649	126	Sat86CF	na	na
chr18	49712073	49713167	1095	Sat145CF	na	na
chr18	49712198	49713315	1118	Sat145CF	na	na
chr18	49712322	49713440	1119	Sat145CF	na	na
chr18	49712966	49713511	546	Sat145CF	na	na
chr18	50193219	50193279	61	SAT1_CF	blue	na
chr18	50216103	50216163	61	SAT1_CF	blue	na
chr18	50270772	50271881	1110	Sat4CF	lred	na
chr18	50324636	50324709	74	Sat156CF	na	na
chr18	51902319	51902380	62	Sat2CF	purple	na
chr18	51987446	51987733	288	Sat5CF	stalk	na
chr18	54236006	54236099	94	Sat21CF	na	na
chr18	54276143	54276358	216	Sat49CF	na	na
chr18	54276166	54276358	193	Sat49CF	na	na
chr18	54583490	54583544	55	Sat130CF	na	na
chr18	54821088	54821154	67	Sat101CF	na	na
chr18	55413907	55413949	43	Sat172CF	na	na
chr18	56755483	56755565	83	Sat103CF	na	na
chr18	57109208	57109312	105	Sat52CF	na	na
chr18	57126008	57127809	1802	Sat25CF	na	na
chr18	57126930	57128786	1857	Sat25CF	na	na
chr18	57128787	57130899	2113	Sat25CF	na	na
chr18	57129986	57131172	1187	Sat25CF	na	na
chr18	57131196	57133243	2048	Sat25CF	na	na

chr18	57133248	57133329	82	Sat25CF	na	na
chr18	57133334	57134984	1651	Sat25CF	na	na
chr18	57134082	57135974	1893	Sat25CF	na	na
chr18	57135840	57137729	1890	Sat25CF	na	na
chr18	57137730	57138963	1234	Sat25CF	na	na
chr18	57139244	57139309	66	Sat25CF	na	na
chr18	57139253	57139309	57	Sat25CF	na	na
chr18	57139259	57139593	335	Sat25CF	na	na
chr18	57139517	57139870	354	Sat25CF	na	na
chr18	57140568	57140610	43	Sat25CF	na	na
chr18	57608800	57608878	79	Sat94CF	na	na
chr18	57927175	57927208	34	Sat56CF	na	na
chr18	58597350	58597393	44	Sat46CF	na	na
chr18	58805798	58807585	1788	SAT1_CF	blue	na
chr18	58853261	58853689	429	SAT1_CF	blue	na
chr18	58858173	58858960	788	Sat2CF	purple	na
chr18	58858641	58859019	379	Sat2CF	purple	na
chr19	3577151	3577645	495	Sat65CF	na	pericen
chr19	4285599	4285658	60	Sat69CF	na	pericen
chr19	4398499	4398938	440	Sat13CF	na	pericen
chr19	5050734	5050795	62	Sat5CF	stalk	na
chr19	5947085	5947123	39	Sat52CF	na	na
chr19	6197701	6199159	1459	Sat114CF	na	na
chr19	6874395	6874481	87	Sat164CF	na	na
chr19	6972715	6972759	45	Sat26CF	na	na
chr19	10879435	10880263	829	Sat52CF	na	na
chr19	10879680	10880277	598	Sat52CF	na	na
chr19	14924864	14924924	61	Sat4CF	lred	na
chr19	15739303	15739668	366	CarSat1	red	na
chr19	16800579	16800622	44	Sat26CF	na	na
chr19	22324596	22324702	107	Carsat3	green	na
chr19	26542660	26542787	128	Sat37CF	na	na
chr19	26855850	26855900	51	Sat38CF	na	na
chr19	31843478	31843699	222	Sat17CF	na	na
chr19	38530554	38530611	58	Sat27CF	na	na
chr19	41512190	41512237	48	Sat35CF	na	na
chr19	42148352	42148611	260	Sat94CF	na	na
chr19	44158108	44158165	58	Sat33CF	na	na
chr19	53319043	53319962	920	Sat106CF	na	na
chr19	55022194	55022242	49	Sat18CF	na	na
chr2	5060246	5060371	126	Sat42CF	na	na
chr2	7567404	7567660	257	Sat15CF	na	na

chr2	7587487	7587848	362	Sat15CF	na	na
chr2	9367110	9367439	330	Sat15CF	na	na
chr2	9367542	9367822	281	Sat15CF	na	na
chr2	12567322	12567405	84	Sat17CF	na	na
chr2	12567465	12567586	122	Sat17CF	na	na
chr2	12904247	12904326	80	Sat72CF	na	na
chr2	12904251	12904320	70	Sat72CF	na	na
chr2	17193740	17193827	88	Sat15CF	na	na
chr2	17194303	17194595	293	Sat15CF	na	na
chr2	17194949	17195555	607	Sat15CF	na	na
chr2	17217586	17217911	326	Sat15CF	na	na
chr2	17219404	17219986	583	Sat15CF	na	na
chr2	17225858	17226229	372	Sat15CF	na	na
chr2	18804846	18805412	567	Sat21CF	na	na
chr2	18804952	18805389	438	Sat21CF	na	na
chr2	22056588	22056717	130	Carsat2	orange	na
chr2	22883753	22884384	632	Sat41CF	na	na
chr2	22883819	22884433	615	Sat83CF	na	na
chr2	24847784	24847848	65	Sat39CF	na	na
chr2	27754897	27755164	268	SAT1_CF	blue	na
chr2	28360327	28361335	1009	Sat135CF	na	na
chr2	28360347	28360736	390	Sat135CF	na	na
chr2	28360437	28361491	1055	Sat135CF	na	na
chr2	31528672	31528859	188	Sat74CF	na	na
chr2	31598405	31598461	57	Sat86CF	na	na
chr2	32031905	32032482	578	Sat94CF	na	na
chr2	32031964	32032136	173	Sat94CF	na	na
chr2	32032008	32032222	215	Sat94CF	na	na
chr2	32032123	32032491	369	Sat94CF	na	na
chr2	32697445	32697604	160	SAT1_CF	blue	na
chr2	32756137	32756193	57	Sat27CF	na	na
chr2	33088298	33088460	163	Sat23CF	na	na
chr2	33960643	33960710	68	Sat36CF	na	na
chr2	34298767	34298823	57	Sat67CF	na	na
chr2	34356917	34357023	107	Carsat3	green	na
chr2	34378160	34378395	236	Sat39CF	na	na
chr2	35027358	35028056	699	Sat21CF	na	na
chr2	35589453	35589818	366	HSATII	na	na
chr2	36408934	36409032	99	Sat72CF	na	na
chr2	36668888	36669025	138	Sat5CF	stalk	na
chr2	36740006	36740207	202	Sat5CF	stalk	na
chr2	37170043	37170146	104	Sat5CF	stalk	na

chr2	37310865	37311052	188	Sat176CF	na	na
chr2	37515234	37515361	128	Sat2CF	purple	na
chr2	37515245	37515300	56	Sat2CF	purple	na
chr2	37820455	37820536	82	Sat12CF	na	na
chr2	38743097	38743383	287	Carsat3	green	na
chr2	38743429	38743637	209	Carsat3	green	na
chr2	38743753	38743884	132	Carsat3	green	na
chr2	38744082	38744219	138	Carsat3	green	na
chr2	39113022	39113150	129	Carsat3	green	na
chr2	39113571	39113708	138	Carsat3	green	na
chr2	39113977	39114111	135	Carsat3	green	na
chr2	39114219	39114348	130	Carsat3	green	na
chr2	39137368	39137708	341	CarSat1	red	na
chr2	43484353	43484413	61	CarSat1	red	na
chr2	50037464	50037643	180	Carsat3	green	na
chr2	50715784	50715840	57	Sat32CF	na	na
chr2	52964828	52965169	342	Sat85CF	na	na
chr2	52964855	52965056	202	Sat85CF	na	na
chr2	57425570	57425993	424	Sat19CF	na	na
chr2	58978327	58978783	457	Sat106CF	na	na
chr2	59007031	59007292	262	Sat43CF	na	na
chr2	59079411	59079727	317	Sat30CF	na	na
chr2	59079454	59079727	274	Sat30CF	na	na
chr2	59230559	59230622	64	Sat50CF	na	na
chr2	59726318	59726790	473	Sat50CF	na	na
chr2	59726329	59726724	396	Sat79CF	na	na
chr2	59803830	59804352	523	Sat52CF	na	na
chr2	59884152	59884634	483	Sat6CF	select	na
chr2	60176622	60176753	132	Sat123CF	na	na
chr2	60273097	60273437	341	Carsat2	orange	na
chr2	60288554	60289670	1117	SAT1_CF	blue	na
chr2	60295105	60295284	180	Sat2CF	purple	na
chr2	60295125	60295284	160	Sat2CF	purple	na
chr2	60471546	60471686	141	Sat2CF	purple	na
chr2	60471627	60471704	78	Sat2CF	purple	na
chr2	60471707	60472711	1005	SAT1_CF	blue	na
chr2	60586155	60586496	342	Sat2CF	purple	na
chr2	60586194	60586481	288	Sat2CF	purple	na
chr2	60606577	60606872	296	Sat2CF	purple	na
chr2	60606600	60606797	198	Sat2CF	purple	na
chr2	60672499	60672786	288	Sat7CF	lime	na
chr2	60672505	60672890	386	Sat7CF	lime	na

chr2	60761176	60762049	874	Sat7CF	lime	na
chr2	60812884	60814534	1651	CarSat1	red	na
chr2	60814829	60815224	396	CarSat1	red	na
chr2	60815516	60815705	190	CarSat1	red	na
chr2	60815888	60816695	808	CarSat1	red	na
chr2	60816892	60817217	326	CarSat1	red	na
chr2	60818031	60818323	293	CarSat1	red	na
chr2	60818352	60819345	994	CarSat1	red	na
chr2	61401180	61401218	39	Sat97CF	na	na
chr2	63983939	63984013	75	Sat91CF	na	na
chr2	64619081	64619115	35	Sat32CF	na	na
chr2	67142776	67142833	58	Carsat3	green	na
chr2	67830933	67831094	162	Sat12CF	na	na
chr2	68758750	68758907	158	Sat93CF	na	na
chr2	69148600	69148662	63	Sat169CF	na	na
chr2	69208364	69208552	189	Sat30CF	na	na
chr2	70828903	70829421	519	Carsat3	green	na
chr2	70829582	70829939	358	Carsat3	green	na
chr2	71866498	71866694	197	Sat12CF	na	na
chr2	71866534	71866672	139	Sat12CF	na	na
chr2	72640423	72640489	67	Sat51CF	na	na
chr2	72901141	72901230	90	Sat132CF	na	na
chr2	73138718	73138932	215	CarSat1	red	na
chr2	75571102	75571220	119	Sat12CF	na	na
chr2	75602161	75602232	72	Sat12CF	na	na
chr2	75619867	75619936	70	Sat12CF	na	na
chr2	76036300	76036359	60	Sat86CF	na	na
chr2	76119475	76119619	145	Sat12CF	na	na
chr2	78377054	78377871	818	Sat12CF	na	na
chr2	78377535	78377910	376	Sat12CF	na	na
chr2	78392543	78392612	70	Sat12CF	na	na
chr2	78886317	78886414	98	Sat12CF	na	na
chr2	78947384	78948366	983	Carsat3	green	na
chr2	79026427	79026572	146	Carsat3	green	na
chr2	79271728	79271905	178	Sat17CF	na	na
chr2	79440859	79440982	124	Sat12CF	na	na
chr2	80424861	80425036	176	Sat13CF	na	na
chr2	81580066	81580270	205	Sat41CF	na	na
chr2	82773284	82773342	59	Sat21CF	na	na
chr2	82776087	82776142	56	Sat18CF	na	na
chr2	82871828	82872177	350	Sat12CF	na	na
chr2	83033206	83033574	369	Sat83CF	na	na

chr2	83033282	83033694	413	Sat83CF	na	na
chr2	83033602	83034025	424	Sat41CF	na	na
chr2	83033921	83034037	117	Sat55CF	na	na
chr2	83554978	83555156	179	Sat12CF	na	na
chr2	83589187	83589364	178	Sat12CF	na	na
chr2	83985994	83986351	358	Sat120CF	na	na
chr2	84524165	84524277	113	Sat12CF	na	na
chr2	84524194	84524278	85	Sat12CF	na	na
chr2	84839691	84839725	35	Sat17CF	na	na
chr2	84839718	84839841	124	Sat17CF	na	na
chr2	85310164	85310716	553	Sat64CF	na	na
chr2	85900864	85901366	503	Sat19CF	na	na
chr2	86461849	86461935	87	Sat28CF	na	na
chr2	86506654	86506735	82	Sat16CF	na	na
chr2	87495373	87495831	459	Sat21CF	na	na
chr2	88392293	88392734	442	CarSat1	red	na
chr20	3010613	3012045	1433	CarSat1	red	pericen
chr20	3027577	3028493	917	Carsat3	green	pericen
chr20	3047775	3049348	1574	Carsat3	green	pericen
chr20	3049360	3049608	249	Carsat3	green	pericen
chr20	3074009	3074127	119	Carsat3	green	pericen
chr20	3087038	3087916	879	Carsat3	green	pericen
chr20	3747432	3747520	89	SUBTEL_sa	na	pericen
chr20	4095147	4095227	81	Sat18CF	na	pericen
chr20	5686355	5686450	96	Sat62CF	na	na
chr20	5914346	5914401	56	Sat185CF	na	na
chr20	6265128	6265167	40	Sat5CF	stalk	na
chr20	6579584	6579623	40	Sat32CF	na	na
chr20	7165609	7165775	167	Carsat3	green	na
chr20	11632688	11633434	747	CarSat1	red	na
chr20	14061927	14061983	57	Sat128CF	na	na
chr20	16381027	16381079	53	Sat56CF	na	na
chr20	18869404	18869470	67	Sat19CF	na	na
chr20	23575206	23575265	60	Sat71CF	na	na
chr20	33078451	33078594	144	CarSat1	red	na
chr20	37394273	37394344	72	Sat20CF	na	na
chr20	42115202	42115267	66	Sat3CF	optpurple	na
chr20	43778598	43779165	568	Sat14CF	na	na
chr20	43779482	43779849	368	Sat14CF	na	na
chr20	43779556	43779938	383	Sat14CF	na	na
chr20	44902287	44902475	189	Sat30CF	na	na
chr20	44936978	44937226	249	Sat39CF	na	na

chr20	45112805	45112859	55	Sat66CF	na	na
chr20	45356700	45356795	96	Sat30CF	na	na
chr20	46520808	46520838	31	Sat27CF	na	na
chr20	46541975	46542781	807	Carsat3	green	na
chr20	46554641	46555833	1193	Carsat3	green	na
chr20	46848473	46849002	530	Sat30CF	na	na
chr20	46930476	46930639	164	Sat12CF	na	na
chr20	47754062	47754225	164	Sat70CF	na	na
chr20	47754084	47756019	1936	Sat70CF	na	na
chr20	47754316	47756253	1938	Sat70CF	na	na
chr20	47754550	47756443	1894	Sat70CF	na	na
chr20	47755018	47756645	1628	Sat70CF	na	na
chr20	47755876	47757567	1692	Sat70CF	na	na
chr20	47757250	47757631	382	Sat70CF	na	na
chr20	48241551	48241645	95	Sat130CF	na	na
chr20	48391461	48391863	403	SAT1_CF	blue	na
chr20	49634556	49634909	354	Sat41CF	na	na
chr20	49634890	49635105	216	Sat41CF	na	na
chr20	50985118	50985485	368	Carsat3	green	na
chr20	50985518	50986233	716	Carsat3	green	na
chr20	51308030	51308196	167	Sat163CF	na	na
chr20	51527824	51528010	187	Sat12CF	na	na
chr20	51864503	51864644	142	Sat79CF	na	na
chr20	52223534	52223721	188	Sat55CF	na	na
chr20	52223545	52223681	137	Sat39CF	na	na
chr20	52475654	52477312	1659	Carsat3	green	na
chr20	52539492	52541145	1654	Carsat3	green	na
chr20	52543171	52543334	164	Carsat3	green	na
chr20	52543506	52543766	261	Carsat3	green	na
chr20	52583054	52583144	91	Carsat3	green	na
chr20	52636132	52636270	139	Carsat3	green	na
chr20	52636337	52637139	803	Carsat3	green	na
chr20	52637237	52637877	641	Carsat3	green	na
chr20	52637984	52638119	136	Carsat3	green	na
chr20	52672674	52672798	125	Carsat3	green	na
chr20	52672859	52673093	235	Carsat3	green	na
chr20	52673190	52673438	249	Carsat3	green	na
chr20	52673619	52673769	151	Carsat3	green	na
chr20	52690978	52691505	528	Carsat3	green	na
chr20	52691679	52691742	64	Carsat3	green	na
chr20	52691846	52692128	283	Carsat3	green	na
chr20	52742215	52743713	1499	Carsat3	green	na

chr20	52980796	52980898	103	Sat12CF	na	na
chr20	52980798	52980912	115	Sat12CF	na	na
chr20	53089496	53089814	319	Sat12CF	na	na
chr20	53705086	53705175	90	Sat5CF	stalk	na
chr20	54209408	54210055	648	Carsat3	green	na
chr20	54210215	54210336	122	Carsat3	green	na
chr20	54214405	54214502	98	Carsat3	green	na
chr20	54242484	54243346	863	Carsat3	green	na
chr20	54244265	54244388	124	Carsat3	green	na
chr20	54298983	54299110	128	Carsat3	green	na
chr20	54299242	54300284	1043	Carsat3	green	na
chr20	54300320	54300425	106	Carsat3	green	na
chr20	54320168	54320966	799	Carsat3	green	na
chr20	54321115	54321215	101	Carsat3	green	na
chr20	54321245	54321383	139	Carsat3	green	na
chr20	54346220	54346421	202	Carsat3	green	na
chr20	54346652	54347104	453	Carsat3	green	na
chr20	54347277	54347330	54	Carsat3	green	na
chr20	54347773	54347900	128	Carsat3	green	na
chr20	54373700	54375164	1465	Carsat3	green	na
chr20	54511302	54512526	1225	Carsat3	green	na
chr20	54512592	54512687	96	Carsat3	green	na
chr20	54569587	54570138	552	Carsat3	green	na
chr20	54570172	54570729	558	Carsat3	green	na
chr20	54792673	54794336	1664	Sat32CF	na	na
chr20	54792852	54800504	7653	Sat32CF	na	na
chr20	54799019	54800737	1719	Sat32CF	na	na
chr20	54808022	54808089	68	Sat35CF	na	na
chr20	54915177	54915964	788	Carsat3	green	na
chr20	54916615	54916915	301	Carsat3	green	na
chr20	56470244	56470295	52	Sat8CF	dgreen	na
chr20	56652978	56653022	45	Sat13CF	na	na
chr20	56678415	56678515	101	Sat55CF	na	na
chr20	56748192	56748253	62	Sat27CF	na	na
chr20	56974345	56974469	125	Sat52CF	na	na
chr20	57003760	57004040	281	Carsat3	green	na
chr20	57004222	57004353	132	Carsat3	green	na
chr20	57534243	57534675	433	Sat98CF	na	na
chr20	57534296	57534722	427	Sat30CF	na	na
chr20	58142641	58143737	1097	Sat67CF	na	na
chr20	58142783	58146410	3628	Sat67CF	na	na
chr20	58145555	58146544	990	Sat67CF	na	na

chr20	58288272	58288442	171	Sat42CF	na	na
chr20	58311692	58311857	166	Sat12CF	na	na
chr20	58311739	58311904	166	Sat12CF	na	na
chr20	58312094	58312357	264	Sat12CF	na	na
chr20	58895361	58895584	224	Sat58CF	na	na
chr20	58895384	58896603	1220	Sat58CF	na	na
chr20	58896800	58897902	1103	Sat58CF	na	na
chr20	58897904	58898494	591	Sat58CF	na	na
chr20	59369082	59369282	201	Carsat3	green	na
chr20	59369464	59370853	1390	Carsat3	green	na
chr20	59418692	59420211	1520	Carsat3	green	na
chr20	59424563	59424756	194	Carsat3	green	na
chr20	59424878	59425416	539	Carsat3	green	na
chr20	59425694	59425788	95	Carsat3	green	na
chr20	59460314	59460421	108	Carsat3	green	na
chr20	59460465	59461456	992	Carsat3	green	na
chr20	59477057	59478242	1186	Carsat3	green	na
chr20	59504628	59505770	1143	Carsat3	green	na
chr20	59505808	59506016	209	Carsat3	green	na
chr20	59524059	59524730	672	Carsat3	green	na
chr20	59924255	59924605	351	CarSat1	red	na
chr20	60721387	60721442	56	Sat93CF	na	na
chr20	60721405	60722557	1153	Sat93CF	na	na
chr20	60722517	60722576	60	Sat93CF	na	na
chr20	60941879	60941940	62	Sat50CF	na	na
chr21	3049332	3051759	2428	SAT1_CF	blue	pericen
chr21	3055992	3057446	1455	CarSat1	red	pericen
chr21	3057733	3057975	243	CarSat1	red	pericen
chr21	5636472	5636563	92	Carsat3	green	na
chr21	8738301	8738355	55	Sat59CF	na	na
chr21	12562967	12563160	194	CarSat1	red	na
chr21	16509402	16509445	44	Sat91CF	na	na
chr21	19212344	19212772	429	Carsat3	green	na
chr21	19366508	19366578	71	Sat30CF	na	na
chr21	22695438	22695510	73	Sat32CF	na	na
chr21	23192527	23192587	61	Sat49CF	na	na
chr21	23259386	23259552	167	SAT1_CF	blue	na
chr21	26235725	26235891	167	Sat12CF	na	na
chr21	26265564	26265902	339	Sat12CF	na	na
chr21	26271773	26272053	281	Carsat3	green	na
chr21	26345098	26345197	100	Sat12CF	na	na
chr21	26365567	26365648	82	HSATII	na	na

chr21	26365669	26365830	162	HSATII	na	na
chr21	27962391	27962476	86	Sat5CF	stalk	na
chr21	29935314	29935360	47	Sat45CF	na	na
chr21	30239614	30240225	612	Sat7CF	lime	na
chr21	32127159	32127214	56	CarSat1	red	na
chr21	32537465	32537794	330	Sat17CF	na	na
chr21	33868637	33869135	499	Carsat3	green	na
chr21	33881688	33882481	794	Carsat3	green	na
chr21	33882506	33882672	167	Carsat3	green	na
chr21	34127859	34128079	221	Sat17CF	na	na
chr21	34989141	34989238	98	Sat21CF	na	na
chr21	35117841	35117920	80	Sat19CF	na	na
chr21	38915257	38916138	882	Sat28CF	na	na
chr21	38915266	38915961	696	Sat41CF	na	na
chr21	39317524	39317586	63	Sat39CF	na	na
chr21	39318012	39318257	246	Sat39CF	na	na
chr21	39318085	39318244	160	Sat39CF	na	na
chr21	40006770	40006811	42	Sat12CF	na	na
chr21	41377160	41377232	73	Sat108CF	na	na
chr21	43121421	43121573	153	Carsat2	orange	na
chr21	43189990	43190140	151	Sat12CF	na	na
chr21	43190021	43190165	145	Sat12CF	na	na
chr21	44556987	44557039	53	Sat4CF	lred	na
chr21	46491993	46492051	59	Sat137CF	na	na
chr21	48338809	48339049	241	Carsat3	green	na
chr21	48339312	48339467	156	Carsat3	green	na
chr21	51206572	51206977	406	Sat38CF	na	na
chr21	52067252	52068004	753	Sat131CF	na	na
chr21	53446494	53446711	218	Sat4CF	lred	na
chr21	53446524	53446763	240	Sat4CF	lred	na
chr22	3008788	3009787	1000	Sat29CF	na	pericen
chr22	3008996	3009994	999	Sat29CF	na	pericen
chr22	3019626	3019954	329	Sat23CF	na	pericen
chr22	3243165	3243262	98	Sat21CF	na	pericen
chr22	3786654	3787154	501	Sat38CF	na	pericen
chr22	4046644	4046890	247	Sat49CF	na	pericen
chr22	4047635	4047805	171	Sat4CF	lred	pericen
chr22	4047657	4047805	149	Sat4CF	lred	pericen
chr22	4202328	4202943	616	Sat97CF	na	pericen
chr22	4239319	4239397	79	Sat72CF	na	pericen
chr22	5097819	5097979	161	Sat19CF	na	na
chr22	5793449	5793899	451	Sat131CF	na	na

chr22	7806239	7806339	101	Sat21CF	na	na
chr22	8675524	8676004	481	CarSat1	red	na
chr22	11244780	11244838	59	Sat122CF	na	na
chr22	11654256	11654319	64	Sat33CF	na	na
chr22	12334656	12334727	72	Sat14CF	na	na
chr22	23781389	23781580	192	Sat17CF	na	na
chr22	24312480	24312527	48	Sat108CF	na	na
chr22	25376898	25376931	34	Sat138CF	na	na
chr22	33267830	33268142	313	SAT1_CF	blue	na
chr22	33349103	33349160	58	Sat5CF	stalk	na
chr22	34021802	34021853	52	CarSat1	red	na
chr22	50692578	50692644	67	Sat91CF	na	na
chr22	54412042	54412274	233	Sat65CF	na	na
chr22	55724098	55724236	139	Sat17CF	na	na
chr22	57257904	57258090	187	Sat17CF	na	na
chr22	58822532	58822588	57	Sat26CF	na	na
chr22	60382390	60382452	63	Sat36CF	na	na
chr22	61152503	61152581	79	Sat46CF	na	na
chr22	61902136	61902307	172	Sat72CF	na	na
chr22	62471944	62472013	70	Sat73CF	na	na
chr22	62747230	62747676	447	Sat160CF	na	na
chr22	63014273	63014450	178	Sat5CF	stalk	na
chr22	63225304	63225363	60	Sat27CF	na	na
chr22	63354235	63354476	242	Sat19CF	na	na
chr22	63376455	63376747	293	Carsat2	orange	na
chr22	64330527	64330988	462	Sat100CF	na	na
chr22	64342451	64342798	348	CarSat1	red	na
chr22	64385073	64386026	954	CarSat1	red	na
chr22	64386049	64387433	1385	CarSat1	red	na
chr22	64387646	64387750	105	CarSat1	red	na
chr22	64387763	64388482	720	CarSat1	red	na
chr22	64388500	64389181	682	CarSat1	red	na
chr22	64399050	64400528	1479	CarSat1	red	na
chr22	64400555	64401118	564	CarSat1	red	na
chr23	3693863	3693920	58	Sat41CF	na	pericen
chr23	3693869	3693917	49	Sat41CF	na	pericen
chr23	4819686	4820809	1124	Carsat3	green	pericen
chr23	4868796	4869914	1119	Carsat3	green	pericen
chr23	4934643	4934984	342	Carsat3	green	pericen
chr23	4934997	4935434	438	Carsat3	green	pericen
chr23	4935513	4936028	516	Carsat3	green	pericen
chr23	4936140	4936317	178	Carsat3	green	pericen

chr23	5544653	5544803	151	Sat111CF	na	na
chr23	5544690	5545809	1120	Sat111CF	na	na
chr23	5544944	5545906	963	Sat111CF	na	na
chr23	5545628	5545944	317	Sat111CF	na	na
chr23	6830211	6830359	149	Sat131CF	na	na
chr23	6860654	6860734	81	Sat17CF	na	na
chr23	6860937	6861073	137	Sat17CF	na	na
chr23	8076143	8076187	45	Sat36CF	na	na
chr23	8076144	8076183	40	Sat36CF	na	na
chr23	8532755	8533001	247	Carsat3	green	na
chr23	10647440	10647483	44	Sat137CF	na	na
chr23	10810052	10810188	137	Sat91CF	na	na
chr23	11998607	11998661	55	Sat63CF	na	na
chr23	12940481	12941362	882	Carsat3	green	na
chr23	12967438	12968108	671	Carsat3	green	na
chr23	12988512	12989123	612	Carsat3	green	na
chr23	14636402	14637578	1177	Sat14CF	na	na
chr23	14637683	14639033	1351	Sat14CF	na	na
chr23	15027323	15028008	686	Carsat3	green	na
chr23	15041084	15041742	659	Carsat3	green	na
chr23	16664699	16664757	59	Sat12CF	na	na
chr23	30372557	30372633	77	Sat13CF	na	na
chr23	30557523	30557585	63	Sat33CF	na	na
chr23	33859783	33859847	65	Sat98CF	na	na
chr23	37620049	37620104	56	Sat41CF	na	na
chr23	38114802	38114874	73	Sat63CF	na	na
chr23	38535271	38535337	67	Sat5CF	stalk	na
chr23	39318710	39318779	70	Sat103CF	na	na
chr23	39820805	39820885	81	Sat2CF	purple	na
chr23	40778296	40778343	48	Sat77CF	na	na
chr23	44905298	44905362	65	Sat108CF	na	na
chr23	47957726	47957808	83	Sat17CF	na	na
chr23	47957852	47957971	120	Sat17CF	na	na
chr23	49298764	49299948	1185	Sat162CF	na	na
chr23	51509691	51510869	1179	Sat138CF	na	na
chr23	51510914	51510948	35	Sat138CF	na	na
chr23	52811095	52811142	48	Sat76CF	na	na
chr23	53796567	53796668	102	SAT1_CF	blue	na
chr23	53841309	53841372	64	Sat30CF	na	na
chr23	54171470	54171806	337	Sat21CF	na	na
chr24	3001207	3001480	274	Sat2CF	purple	pericen
chr24	3006114	3006905	792	SAT1_CF	blue	pericen

chr24	3006950	3008534	1585	SAT1_CF	blue	pericen
chr24	3008546	3008668	123	SAT1_CF	blue	pericen
chr24	3018416	3020235	1820	CarSat1	red	pericen
chr24	3020512	3021060	549	CarSat1	red	pericen
chr24	3021087	3022673	1587	CarSat1	red	pericen
chr24	3022930	3023059	130	CarSat1	red	pericen
chr24	3024831	3026202	1372	CarSat1	red	pericen
chr24	3026264	3031196	4933	CarSat1	red	pericen
chr24	3031516	3032063	548	CarSat1	red	pericen
chr24	3035464	3037110	1647	CarSat1	red	pericen
chr24	3037215	3039395	2181	CarSat1	red	pericen
chr24	3039441	3040029	589	Carsat2	orange	pericen
chr24	3040220	3040665	446	Carsat2	orange	pericen
chr24	3040250	3041119	870	CarSat1	red	pericen
chr24	3041121	3041557	437	Carsat2	orange	pericen
chr24	3041731	3042595	865	Carsat2	orange	pericen
chr24	3043059	3043092	34	Carsat2	orange	pericen
chr24	3055047	3055952	906	Carsat2	orange	pericen
chr24	3055971	3056571	601	Carsat2	orange	pericen
chr24	3056993	3057071	79	Carsat2	orange	pericen
chr24	3057488	3057936	449	Carsat2	orange	pericen
chr24	3263206	3263302	97	Carsat3	green	pericen
chr24	3297319	3297471	153	Sat125CF	na	pericen
chr24	3297336	3298628	1293	Sat125CF	na	pericen
chr24	3540877	3541695	819	Sat91CF	na	pericen
chr24	3541559	3541695	137	Sat91CF	na	pericen
chr24	5072376	5072439	64	Sat26CF	na	na
chr24	7153264	7153327	64	Sat91CF	na	na
chr24	7816856	7817527	672	Carsat3	green	na
chr24	7817615	7818091	477	Carsat3	green	na
chr24	8348732	8349528	797	Sat168CF	na	na
chr24	8348942	8349528	587	Sat168CF	na	na
chr24	9492869	9492947	79	Sat114CF	na	na
chr24	14885310	14885379	70	SUBTEL_sa	na	na
chr24	18491230	18491270	41	Sat76CF	na	na
chr24	21031166	21031247	82	Sat105CF	na	na
chr24	21666849	21667182	334	Carsat3	green	na
chr24	22119134	22119208	75	Sat27CF	na	na
chr24	22678351	22678404	54	Sat18CF	na	na
chr24	23181083	23181386	304	Sat14CF	na	na
chr24	25370919	25370984	66	Sat49CF	na	na
chr24	25974776	25974839	64	Sat9CF	na	na

chr24	27549839	27549980	142	Sat43CF	na	na
chr24	30553279	30553483	205	Sat30CF	na	na
chr24	30637844	30637912	69	Sat26CF	na	na
chr24	31445187	31445260	74	Sat55CF	na	na
chr24	31636296	31636404	109	Sat69CF	na	na
chr24	31636309	31636415	107	Sat69CF	na	na
chr24	32463804	32463875	72	Sat12CF	na	na
chr24	34163807	34163892	86	Sat43CF	na	na
chr24	34163901	34163935	35	Sat72CF	na	na
chr24	35252287	35252369	83	Carsat2	orange	na
chr24	35482404	35482472	69	Sat14CF	na	na
chr24	36308287	36308525	239	Sat68CF	na	na
chr24	36660466	36661858	1393	Carsat3	green	na
chr24	36661871	36662119	249	Carsat3	green	na
chr24	36709734	36709874	141	Carsat3	green	na
chr24	36710340	36710924	585	Carsat3	green	na
chr24	36728732	36728816	85	Carsat3	green	na
chr24	36728878	36729016	139	Carsat3	green	na
chr24	36729173	36729874	702	Carsat3	green	na
chr24	36739890	36740026	137	Carsat3	green	na
chr24	36740097	36740498	402	Carsat3	green	na
chr24	36740510	36740967	458	Carsat3	green	na
chr24	36748533	36748666	134	Carsat3	green	na
chr24	37943864	37944016	153	Sat51CF	na	na
chr24	38245056	38245207	152	Sat51CF	na	na
chr24	41298319	41298363	45	Sat7CF	lime	na
chr24	44176315	44176421	107	Sat95CF	na	na
chr24	44176352	44176444	93	Sat95CF	na	na
chr24	44877484	44877526	43	Sat98CF	na	na
chr24	45106690	45107920	1231	Sat52CF	na	na
chr24	45106759	45107796	1038	Sat160CF	na	na
chr24	45486216	45486639	424	Sat164CF	na	na
chr24	45486261	45487619	1359	Sat164CF	na	na
chr24	45487426	45487647	222	Sat164CF	na	na
chr24	46228360	46228608	249	Sat119CF	na	na
chr24	46602107	46602336	230	Sat79CF	na	na
chr24	47030819	47030892	74	Sat62CF	na	na
chr24	47851644	47851698	55	Sat52CF	na	na
chr24	47924281	47924359	79	Sat2CF	purple	na
chr24	48453748	48454103	356	Sat100CF	na	na
chr24	48579434	48579525	92	Sat37CF	na	na
chr24	48835792	48836041	250	Sat5CF	stalk	na

chr24	48993784	48993859	76	Sat34CF	na	na
chr24	49044940	49044984	45	Sat5CF	stalk	na
chr24	49781618	49781712	95	SUBTEL_sa	na	na
chr24	50595872	50596016	145	Sat13CF	na	na
chr24	50630501	50630722	222	Sat5CF	stalk	na
chr24	50760449	50761547	1099	SAT1_CF	blue	na
chr25	3065103	3065230	128	SAT1_CF	blue	pericen
chr25	3075783	3075888	106	SAT1_CF	blue	pericen
chr25	3075926	3077018	1093	SAT1_CF	blue	pericen
chr25	3077001	3077828	828	CarSat1	red	pericen
chr25	3077852	3078190	339	CarSat1	red	pericen
chr25	3482490	3482557	68	Sat34CF	na	pericen
chr25	3510067	3510200	134	Sat17CF	na	pericen
chr25	3510413	3510495	83	Sat17CF	na	pericen
chr25	6668155	6668250	96	Sat33CF	na	na
chr25	6668171	6668250	80	Sat33CF	na	na
chr25	7283926	7283996	71	Carsat3	green	na
chr25	11726642	11726712	71	Sat35CF	na	na
chr25	11764484	11764660	177	Sat15CF	na	na
chr25	12087976	12088044	69	Sat32CF	na	na
chr25	19387717	19387953	237	Carsat3	green	na
chr25	19388838	19388900	63	Carsat3	green	na
chr25	22035718	22035995	278	Carsat2	orange	na
chr25	22063675	22064650	976	Carsat2	orange	na
chr25	22496048	22497049	1002	Sat173CF	na	na
chr25	22497013	22497071	59	Sat173CF	na	na
chr25	22898842	22899506	665	Sat15CF	na	na
chr25	22920629	22920885	257	Sat15CF	na	na
chr25	24494843	24494983	141	Carsat3	green	na
chr25	26880974	26881036	63	Sat93CF	na	na
chr25	27996637	27996780	144	Carsat3	green	na
chr25	30423473	30423522	50	Sat118CF	na	na
chr25	30506941	30507277	337	Carsat2	orange	na
chr25	30515004	30515427	424	Carsat2	orange	na
chr25	30516657	30517538	882	Sat7CF	lime	na
chr25	30516769	30520015	3247	Sat7CF	lime	na
chr25	30517700	30520942	3243	Sat7CF	lime	na
chr25	30518645	30521873	3229	Sat7CF	lime	na
chr25	30521415	30521949	535	Sat7CF	lime	na
chr25	30552986	30553111	126	Carsat2	orange	na
chr25	31248890	31248955	66	Sat86CF	na	na
chr25	37157571	37157619	49	Sat103CF	na	na

chr25	37291341	37291580	240	Sat171CF	na	na
chr25	37291591	37291679	89	Sat171CF	na	na
chr25	38072481	38072582	102	Sat13CF	na	na
chr25	39460279	39460555	277	Carsat2	orange	na
chr25	39691322	39691379	58	Sat76CF	na	na
chr25	40179497	40179598	102	CarSat1	red	na
chr25	40180270	40180524	255	Carsat2	orange	na
chr25	40180689	40181090	402	Carsat2	orange	na
chr25	40181503	40181827	325	Carsat2	orange	na
chr25	40181859	40182277	419	Carsat2	orange	na
chr25	40182950	40183110	161	Carsat2	orange	na
chr25	40186192	40187177	986	Carsat2	orange	na
chr25	40199876	40200139	264	SAT1_CF	blue	na
chr25	40200216	40200368	153	SAT1_CF	blue	na
chr25	40200455	40200692	238	SAT1_CF	blue	na
chr25	40253080	40254081	1002	Carsat3	green	na
chr25	40284438	40285585	1148	Carsat3	green	na
chr25	41622570	41623674	1105	Sat102CF	na	na
chr25	41670706	41671864	1159	Sat128CF	na	na
chr25	42991775	42991812	38	Sat66CF	na	na
chr25	45885727	45885788	62	Sat136CF	na	na
chr25	48688161	48688364	204	Sat79CF	na	na
chr25	49966351	49967763	1413	Sat84CF	na	na
chr25	49967584	49967794	211	Sat84CF	na	na
chr25	51095479	51095742	264	HSATII	na	na
chr25	51179615	51180173	559	Sat153CF	na	na
chr25	51400731	51401319	589	Sat131CF	na	na
chr25	51653497	51653917	421	Sat81CF	na	na
chr25	51780765	51781032	268	Sat3CF	optpurple	na
chr25	51801522	51802419	898	Sat79CF	na	na
chr25	52243677	52243898	222	Sat74CF	na	na
chr25	52775966	52776172	207	Sat83CF	na	na
chr25	53335892	53336515	624	Sat19CF	na	na
chr25	53844415	53844969	555	Sat14CF	na	na
chr25	53844421	53844831	411	Sat14CF	na	na
chr25	53844468	53845105	638	Sat14CF	na	na
chr25	53844517	53845027	511	Sat14CF	na	na
chr25	54358967	54359991	1025	Sat51CF	na	na
chr26	3046079	3048228	2150	Carsat3	green	pericen
chr26	3091013	3092028	1016	Carsat3	green	pericen
chr26	3118876	3119628	753	Carsat3	green	pericen
chr26	3137254	3138196	943	Carsat3	green	pericen

chr26	3176516	3176666	151	Carsat3	green	pericen
chr26	3176970	3178710	1741	Carsat3	green	pericen
chr26	3213132	3214286	1155	Carsat3	green	pericen
chr26	3235739	3235839	101	Carsat3	green	pericen
chr26	3257726	3259296	1571	Carsat3	green	pericen
chr26	3259368	3259501	134	Carsat3	green	pericen
chr26	3275165	3275297	133	Carsat3	green	pericen
chr26	3275339	3276116	778	Carsat3	green	pericen
chr26	3535292	3535538	247	Sat69CF	na	pericen
chr26	3956757	3957057	301	Sat16CF	na	pericen
chr26	4551988	4552218	231	Sat84CF	na	pericen
chr26	4728525	4728585	61	Sat26CF	na	pericen
chr26	4756707	4757232	526	Sat98CF	na	pericen
chr26	4759039	4759789	751	Sat30CF	na	pericen
chr26	4760906	4761437	532	Sat98CF	na	pericen
chr26	4796553	4797079	527	Sat144CF	na	pericen
chr26	4797537	4797936	400	Sat144CF	na	pericen
chr26	5169593	5170721	1129	Sat91CF	na	na
chr26	5918497	5918631	135	Sat5CF	stalk	na
chr26	6683936	6684047	112	Sat31CF	na	na
chr26	8018521	8019644	1124	Sat17CF	na	na
chr26	8718892	8719634	743	Carsat3	green	na
chr26	9500269	9500326	58	Sat33CF	na	na
chr26	10032363	10032810	448	Carsat3	green	na
chr26	12717997	12718050	54	Sat91CF	na	na
chr26	13797178	13797301	124	Sat13CF	na	na
chr26	16264880	16265266	387	Sat28CF	na	na
chr26	16323123	16323184	62	Sat27CF	na	na
chr26	16368961	16369005	45	Sat10CF	na	na
chr26	20093985	20094238	254	Carsat3	green	na
chr26	20902939	20903002	64	Sat21CF	na	na
chr26	24483851	24483911	61	Sat12CF	na	na
chr26	25757433	25757474	42	SUBTEL_sa	na	na
chr26	25762684	25762870	187	Sat130CF	na	na
chr26	25762884	25763928	1045	Sat130CF	na	na
chr26	27410471	27410525	55	Sat181CF	na	na
chr26	28147812	28147854	43	CarSat1	red	na
chr26	28335474	28335516	43	CarSat1	red	na
chr26	28591893	28591935	43	CarSat1	red	na
chr26	29154715	29154757	43	CarSat1	red	na
chr26	29501067	29501535	469	Carsat2	orange	na
chr26	29501552	29501690	139	Carsat2	orange	na

chr26	29502044	29502121	78	Carsat2	orange	na
chr26	31822132	31823204	1073	Carsat3	green	na
chr26	31991160	31991213	54	Sat105CF	na	na
chr26	32673255	32673307	53	Sat4CF	lred	na
chr26	33258688	33258807	120	Carsat3	green	na
chr26	33258843	33259866	1024	Carsat3	green	na
chr26	33259888	33260022	135	Carsat3	green	na
chr26	33280137	33281057	921	Carsat3	green	na
chr26	36170267	36170414	148	CarSat1	red	na
chr26	36171925	36172017	93	Sat52CF	na	na
chr26	38446692	38446766	75	Sat130CF	na	na
chr26	38656299	38657297	999	Sat182CF	na	na
chr26	38776171	38776337	167	SUBTEL_sa	na	na
chr26	39054535	39054638	104	Carsat3	green	na
chr26	40476838	40476952	115	Sat49CF	na	na
chr26	41672055	41672602	548	Sat111CF	na	na
chr26	41830629	41831239	611	Sat50CF	na	na
chr26	41834634	41834705	72	Sat19CF	na	na
chr26	41871670	41872705	1036	Sat106CF	na	na
chr26	42026963	42027140	178	SAT1_CF	blue	na
chr26	42027183	42028033	851	SAT1_CF	blue	na
chr27	3215016	3215264	249	Carsat2	orange	pericen
chr27	3215442	3215601	160	Carsat2	orange	pericen
chr27	6187799	6187845	47	Sat63CF	na	na
chr27	7537239	7537286	48	Sat118CF	na	na
chr27	8293948	8294059	112	Sat65CF	na	na
chr27	10277856	10278075	220	Carsat3	green	na
chr27	11058308	11058716	409	CarSat1	red	na
chr27	12462120	12462148	29	Sat57CF	na	na
chr27	18588382	18588483	102	Sat26CF	na	na
chr27	18588420	18588483	64	Sat26CF	na	na
chr27	21899216	21899284	69	Sat137CF	na	na
chr27	31612542	31612846	305	Carsat3	green	na
chr27	31613265	31613803	539	Carsat3	green	na
chr27	38440353	38441071	719	Sat7CF	lime	na
chr27	42992616	42992670	55	Sat100CF	na	na
chr27	44612256	44612542	287	Sat153CF	na	na
chr27	45701433	45701482	50	Sat147CF	na	na
chr27	46600165	46600237	73	Sat79CF	na	na
chr27	48859528	48859884	357	CarSat1	red	na
chr27	48862716	48863263	548	CarSat1	red	na
chr27	48863271	48867067	3797	SAT1_CF	blue	na

chr27	48872167	48872786	620	Sat2CF	purple	na
chr27	48890876	48893547	2672	SAT1_CF	blue	na
chr27	48893581	48893756	176	SAT1_CF	blue	na
chr27	48893758	48894996	1239	CarSat1	red	na
chr27	48895017	48895624	608	CarSat1	red	na
chr27	48895644	48895710	67	CarSat1	red	na
chr27	48895731	48897266	1536	CarSat1	red	na
chr27	48897284	48897856	573	CarSat1	red	na
chr27	48898094	48898994	901	CarSat1	red	na
chr27	48899015	48899864	850	CarSat1	red	na
chr27	48899887	48901335	1449	CarSat1	red	na
chr27	48901496	48903179	1684	CarSat1	red	na
chr27	48903200	48904051	852	CarSat1	red	na
chr27	48904134	48905029	896	CarSat1	red	na
chr28	3003157	3004108	952	CarSat1	red	pericen
chr28	3004135	3004974	840	CarSat1	red	pericen
chr28	3005142	3005846	705	CarSat1	red	pericen
chr28	3005867	3006721	855	CarSat1	red	pericen
chr28	3006871	3008334	1464	CarSat1	red	pericen
chr28	3008355	3009947	1593	CarSat1	red	pericen
chr28	3010107	3010749	643	CarSat1	red	pericen
chr28	3010784	3011562	779	CarSat1	red	pericen
chr28	3011585	3013056	1472	CarSat1	red	pericen
chr28	3018978	3019904	927	CarSat1	red	pericen
chr28	3019925	3020967	1043	CarSat1	red	pericen
chr28	3020984	3022248	1265	CarSat1	red	pericen
chr28	3022269	3023312	1044	CarSat1	red	pericen
chr28	3048327	3048839	513	Sat8CF	dgreen	pericen
chr28	3050321	3050823	503	Sat8CF	dgreen	pericen
chr28	5442475	5442910	436	Carsat3	green	na
chr28	6486826	6487393	568	Carsat3	green	na
chr28	6499758	6500734	977	Carsat3	green	na
chr28	6538508	6538745	238	Carsat3	green	na
chr28	6538760	6539191	432	Carsat3	green	na
chr28	6594284	6595029	746	Carsat3	green	na
chr28	6595212	6595332	121	Carsat3	green	na
chr28	6595375	6595669	295	Carsat3	green	na
chr28	7182309	7182677	369	Carsat3	green	na
chr28	7260897	7260989	93	Carsat3	green	na
chr28	7261042	7262418	1377	Carsat3	green	na
chr28	9069451	9069494	44	Sat66CF	na	na
chr28	11080470	11081555	1086	Sat155CF	na	na

chr28	12413860	12413903	44	Sat104CF	na	na
chr28	13922763	13922817	55	Sat5CF	stalk	na
chr28	18826495	18826613	119	Sat12CF	na	na
chr28	30229964	30230009	46	Sat35CF	na	na
chr28	30428376	30428451	76	Sat65CF	na	na
chr28	30746132	30746178	47	Sat13CF	na	na
chr28	32522776	32522897	122	Sat5CF	stalk	na
chr28	34200964	34201034	71	Sat9CF	na	na
chr28	35163778	35163837	60	Sat92CF	na	na
chr28	35900715	35900890	176	Sat5CF	stalk	na
chr28	39533777	39534531	755	Sat45CF	na	na
chr28	39533919	39534221	303	Sat45CF	na	na
chr28	39534042	39534335	294	Sat45CF	na	na
chr28	39842362	39842588	227	Sat30CF	na	na
chr28	39913610	39914444	835	Sat105CF	na	na
chr28	39913774	39914810	1037	Sat105CF	na	na
chr28	39915082	39915826	745	Sat105CF	na	na
chr28	39915180	39916192	1013	Sat105CF	na	na
chr28	39915525	39916552	1028	Sat105CF	na	na
chr28	39916626	39917659	1034	Sat105CF	na	na
chr28	39916989	39918025	1037	Sat105CF	na	na
chr28	39917355	39918315	961	Sat105CF	na	na
chr28	40437465	40437881	417	Sat30CF	na	na
chr28	40439882	40440748	867	Sat44CF	na	na
chr28	40463621	40463697	77	Sat13CF	na	na
chr28	41348631	41348723	93	Sat63CF	na	na
chr28	42238407	42238478	72	Sat172CF	na	na
chr28	42263857	42264276	420	Sat47CF	na	na
chr28	42678638	42678710	73	Sat19CF	na	na
chr28	42853712	42854139	428	Sat21CF	na	na
chr28	42898675	42898746	72	Sat123CF	na	na
chr28	42903269	42903322	54	Sat151CF	na	na
chr28	43103032	43103429	398	Sat19CF	na	na
chr28	43293749	43293779	31	Sat5CF	stalk	na
chr28	43730895	43731175	281	Sat14CF	na	na
chr28	43730924	43731633	710	Sat14CF	na	na
chr28	43831744	43832239	496	Sat28CF	na	na
chr28	43840670	43840899	230	Sat43CF	na	na
chr28	44035424	44035598	175	CarSat1	red	na
chr28	44154254	44155251	998	Carsat3	green	na
chr28	44188133	44188960	828	SAT1_CF	blue	na
chr29	3009011	3012952	3942	CarSat1	red	pericen

chr29	3012968	3014260	1293	CarSat1	red	pericen
chr29	3014619	3017179	2561	CarSat1	red	pericen
chr29	3538260	3538342	83	Sat49CF	na	pericen
chr29	3720115	3720173	59	Sat60CF	na	pericen
chr29	4455905	4456011	107	Sat146CF	na	pericen
chr29	4455916	4457330	1415	Sat146CF	na	pericen
chr29	9208078	9208169	92	Carsat3	green	na
chr29	9490110	9490167	58	Sat25CF	na	na
chr29	15053696	15053847	152	Carsat3	green	na
chr29	24741699	24741891	193	Sat17CF	na	na
chr29	27321614	27321881	268	SAT1_CF	blue	na
chr29	30526123	30526182	60	SUBTEL_sa	na	na
chr29	30947436	30948362	927	Sat53CF	na	na
chr29	32846262	32846528	267	Carsat3	green	na
chr29	34679810	34680284	475	Sat38CF	na	na
chr29	34730015	34730167	153	Sat129CF	na	na
chr29	36766753	36766835	83	Carsat3	green	na
chr29	36767005	36767280	276	Carsat3	green	na
chr29	36767548	36767784	237	Carsat3	green	na
chr29	36767808	36767894	87	Carsat3	green	na
chr29	38463014	38463695	682	Sat55CF	na	na
chr29	38482078	38483418	1341	Sat102CF	na	na
chr29	38485564	38485663	100	Sat24CF	na	na
chr29	38485664	38486724	1061	Sat24CF	na	na
chr29	39107247	39107505	259	Sat14CF	na	na
chr29	39107392	39107533	142	Sat14CF	na	na
chr29	39495244	39495312	69	Sat33CF	na	na
chr29	41585207	41585349	143	SUBTEL_sa	na	na
chr29	41585529	41585671	143	SUBTEL_sa	na	na
chr29	41964980	41965176	197	Sat38CF	na	na
chr29	42679866	42680323	458	Sat132CF	na	na
chr29	42867525	42867715	191	Sat37CF	na	na
chr29	43110730	43112098	1369	Sat83CF	na	na
chr29	43170929	43171354	426	Sat83CF	na	na
chr29	43810781	43812299	1519	Sat127CF	na	na
chr29	43811023	43812709	1687	Sat127CF	na	na
chr29	43812374	43813154	781	Sat127CF	na	na
chr29	43813222	43813351	130	Sat5CF	stalk	na
chr29	43813251	43813415	165	Sat5CF	stalk	na
chr29	43846246	43846310	65	Sat63CF	na	na
chr29	43969567	43969634	68	Sat53CF	na	na
chr29	44135350	44135470	121	Sat29CF	na	na

chr29	44188767	44189536	770	Sat97CF	na	na
chr29	44268615	44268926	312	Sat104CF	na	na
chr29	44729110	44729462	353	Carsat2	orange	na
chr3	3018973	3020080	1108	Sat2CF	purple	pericen
chr3	3022549	3022684	136	CarSat1	red	pericen
chr3	3022711	3024249	1539	CarSat1	red	pericen
chr3	3033061	3033772	712	CarSat1	red	pericen
chr3	3033966	3034109	144	CarSat1	red	pericen
chr3	3034143	3035333	1191	CarSat1	red	pericen
chr3	3035350	3037502	2153	CarSat1	red	pericen
chr3	3042246	3042675	430	SAT1_CF	blue	pericen
chr3	3047248	3048561	1314	Sat2CF	purple	pericen
chr3	3047428	3048688	1261	Sat2CF	purple	pericen
chr3	3075766	3075833	68	Sat49CF	na	pericen
chr3	6205762	6206765	1004	Sat176CF	na	na
chr3	6473525	6473767	243	Sat18CF	na	na
chr3	6473554	6473766	213	Sat18CF	na	na
chr3	6999576	6999696	121	Sat129CF	na	na
chr3	12591556	12591654	99	Carsat3	green	na
chr3	14642111	14642165	55	Sat155CF	na	na
chr3	18601397	18601567	171	SAT1_CF	blue	na
chr3	26792742	26792822	81	Sat35CF	na	na
chr3	26926610	26926657	48	Sat76CF	na	na
chr3	30796815	30796912	98	Sat36CF	na	na
chr3	31719581	31719640	60	Sat3CF	optpurple	na
chr3	31719809	31719850	42	Sat3CF	optpurple	na
chr3	31720807	31720832	26	Sat3CF	optpurple	na
chr3	33636496	33636829	334	Sat125CF	na	na
chr3	34130791	34132204	1414	CarSat1	red	na
chr3	34132225	34132367	143	CarSat1	red	na
chr3	34132397	34132512	116	CarSat1	red	na
chr3	34132549	34132691	143	CarSat1	red	na
chr3	34132760	34133028	269	CarSat1	red	na
chr3	34133058	34133199	142	CarSat1	red	na
chr3	34133220	34133936	717	CarSat1	red	na
chr3	34146228	34146915	688	Sat2CF	purple	na
chr3	34146302	34146954	653	Sat2CF	purple	na
chr3	34846637	34846691	55	SUBTEL_sa	na	na
chr3	39228539	39228609	71	Sat180CF	na	na
chr3	39589764	39590085	322	Carsat3	green	na
chr3	40048173	40048885	713	Sat9CF	na	na
chr3	40048409	40048887	479	Sat9CF	na	na

chr3	40105859	40106218	360	Carsat3	green	na
chr3	40386857	40386931	75	Sat18CF	na	na
chr3	40386883	40386931	49	Sat18CF	na	na
chr3	40707722	40707814	93	Sat36CF	na	na
chr3	40707727	40707804	78	Sat36CF	na	na
chr3	47307319	47307380	62	Sat77CF	na	na
chr3	50857358	50857395	38	Sat113CF	na	na
chr3	51386211	51386260	50	Sat80CF	na	na
chr3	52934181	52935182	1002	Sat183CF	na	na
chr3	53243589	53243675	87	Sat14CF	na	na
chr3	54875732	54875774	43	Sat88CF	na	na
chr3	54875753	54876992	1240	Sat88CF	na	na
chr3	54875929	54877148	1220	Sat88CF	na	na
chr3	54877149	54877359	211	Sat88CF	na	na
chr3	54878004	54878085	82	Sat88CF	na	na
chr3	55494669	55494837	169	Sat19CF	na	na
chr3	56683258	56683506	249	Carsat3	green	na
chr3	56683681	56684169	489	Carsat3	green	na
chr3	56743645	56744857	1213	Carsat3	green	na
chr3	56763106	56763883	778	Carsat3	green	na
chr3	58693172	58694267	1096	Sat156CF	na	na
chr3	58693320	58694809	1490	Sat156CF	na	na
chr3	59442531	59442582	52	Sat118CF	na	na
chr3	59578252	59578292	41	Sat27CF	na	na
chr3	59753195	59753247	53	Sat145CF	na	na
chr3	59797365	59797428	64	Sat86CF	na	na
chr3	60418107	60418394	288	Sat28CF	na	na
chr3	60735019	60735066	48	Sat109CF	na	na
chr3	60839118	60839559	442	Sat60CF	na	na
chr3	61072980	61073115	136	Sat21CF	na	na
chr3	62838696	62838918	223	Sat84CF	na	na
chr3	62863471	62863666	196	Sat80CF	na	na
chr3	63012838	63012890	53	Sat22CF	na	na
chr3	63149974	63150124	151	Sat30CF	na	na
chr3	63217235	63217373	139	Sat16CF	na	na
chr3	63262324	63262730	407	Sat47CF	na	na
chr3	63262485	63262765	281	Sat114CF	na	na
chr3	63262964	63263480	517	Sat47CF	na	na
chr3	63335723	63335907	185	Sat2CF	purple	na
chr3	63836790	63837153	364	Sat21CF	na	na
chr3	64919132	64919424	293	Sat30CF	na	na
chr3	64919182	64919466	285	Sat30CF	na	na

chr3	65288685	65288787	103	Sat13CF	na	na
chr3	65331453	65331719	267	Sat131CF	na	na
chr3	65339612	65339785	174	Sat52CF	na	na
chr3	65344724	65345123	400	Sat5CF	stalk	na
chr3	66706286	66706342	57	Sat36CF	na	na
chr3	66744284	66744372	89	Carsat3	green	na
chr3	67207303	67207514	212	Sat95CF	na	na
chr3	69291912	69292395	484	Sat119CF	na	na
chr3	76309672	76309742	71	Sat165CF	na	na
chr3	86811307	86811515	209	Carsat3	green	na
chr3	86811633	86811798	166	Carsat3	green	na
chr3	87256508	87256657	150	Sat52CF	na	na
chr3	87256519	87256635	117	Sat52CF	na	na
chr3	90143425	90143465	41	Sat19CF	na	na
chr3	90147282	90147881	600	Sat41CF	na	na
chr3	90147283	90147999	717	Sat41CF	na	na
chr3	90771747	90772108	362	Sat42CF	na	na
chr3	91986714	91987147	434	Sat30CF	na	na
chr3	93232279	93232499	221	Carsat3	green	na
chr3	94184490	94184628	139	CarSat1	red	na
chr3	94406431	94406890	460	Sat110CF	na	na
chr3	94406492	94407617	1126	Sat110CF	na	na
chr3	94407375	94407624	250	Sat110CF	na	na
chr3	94692561	94693582	1022	SAT1_CF	blue	na
chr3	94711078	94712068	991	SAT1_CF	blue	na
chr30	5664451	5664522	72	Carsat3	green	na
chr30	9780483	9780541	59	Sat21CF	na	na
chr30	11871228	11871331	104	Sat89CF	na	na
chr30	13210246	13210848	603	Carsat3	green	na
chr30	17886056	17886114	59	Sat88CF	na	na
chr30	18098951	18098979	29	SUBTEL_sa	na	na
chr30	19930024	19930067	44	Sat41CF	na	na
chr30	20919047	20919107	61	Sat163CF	na	na
chr30	22128721	22128766	46	Sat15CF	na	na
chr30	24736567	24736630	64	Sat57CF	na	na
chr30	26050787	26050828	42	Sat88CF	na	na
chr30	32168956	32169000	45	Sat65CF	na	na
chr30	33467658	33468979	1322	Sat139CF	na	na
chr30	33468931	33468986	56	Sat139CF	na	na
chr30	34012867	34012899	33	Sat91CF	na	na
chr30	36088780	36089125	346	Sat93CF	na	na
chr30	36088818	36089155	338	Sat93CF	na	na

chr30	36952433	36952508	76	Sat125CF	na	na
chr30	40000527	40000812	286	Sat110CF	na	na
chr30	40875684	40876150	467	SAT1_CF	blue	na
chr30	41131431	41131502	72	Sat149CF	na	na
chr30	41131481	41131502	22	Sat149CF	na	na
chr30	41792308	41792475	168	SAT1_CF	blue	na
chr30	42302865	42303058	194	Sat65CF	na	na
chr30	43020271	43020415	145	Sat186CF	na	na
chr30	43020336	43020415	80	Sat186CF	na	na
chr30	43195880	43196104	225	Sat2CF	purple	na
chr30	43195920	43196110	191	Sat2CF	purple	na
chr31	3031011	3031999	989	Sat2CF	purple	pericen
chr31	3031074	3031940	867	Sat2CF	purple	pericen
chr31	3036499	3036891	393	SAT1_CF	blue	pericen
chr31	3039107	3039772	666	Sat2CF	purple	pericen
chr31	3039211	3039737	527	Sat2CF	purple	pericen
chr31	3042554	3043625	1072	SAT1_CF	blue	pericen
chr31	3043639	3046154	2516	SAT1_CF	blue	pericen
chr31	3046193	3047145	953	SAT1_CF	blue	pericen
chr31	3047168	3047597	430	SAT1_CF	blue	pericen
chr31	3047605	3048903	1299	CarSat1	red	pericen
chr31	3067208	3068036	829	Sat2CF	purple	pericen
chr31	3075279	3076590	1312	CarSat1	red	pericen
chr31	3076609	3077761	1153	CarSat1	red	pericen
chr31	3077774	3082574	4801	CarSat1	red	pericen
chr31	3082483	3084118	1636	Carsat2	orange	pericen
chr31	3092278	3093025	748	Carsat2	orange	pericen
chr31	3093273	3094320	1048	Carsat2	orange	pericen
chr31	3094662	3094797	136	Carsat2	orange	pericen
chr31	3110574	3110912	339	Carsat2	orange	pericen
chr31	22275135	22276223	1089	Carsat2	orange	na
chr31	24257623	24257845	223	Carsat3	green	na
chr31	28931677	28931747	71	Sat83CF	na	na
chr31	29625299	29625836	538	Sat87CF	na	na
chr31	31191105	31191326	222	Sat112CF	na	na
chr31	32176555	32177416	862	SAT1_CF	blue	na
chr31	33908992	33909067	76	Sat86CF	na	na
chr31	34909716	34909787	72	Sat63CF	na	na
chr31	35164442	35164502	61	Sat154CF	na	na
chr31	38362540	38363117	578	Sat84CF	na	na
chr31	38365978	38366551	574	Sat94CF	na	na
chr31	38386138	38386186	49	Sat18CF	na	na

chr31	38386151	38387776	1626	Sat18CF	na	na
chr31	38388158	38388214	57	Sat18CF	na	na
chr31	38734315	38734454	140	Sat72CF	na	na
chr31	38747367	38747448	82	Sat5CF	stalk	na
chr31	39250809	39251648	840	CarSat1	red	na
chr31	39336310	39336502	193	Sat5CF	stalk	na
chr31	39376753	39377129	377	Sat63CF	na	na
chr31	39376792	39380651	3860	Sat63CF	na	na
chr31	39380318	39380693	376	Sat63CF	na	na
chr31	39824538	39824779	242	Sat21CF	na	na
chr31	39867453	39867530	78	Sat21CF	na	na
chr31	40752159	40752467	309	Sat94CF	na	na
chr31	40962282	40962436	155	SUBTEL_sa	na	na
chr31	41017346	41017428	83	Sat21CF	na	na
chr31	41019377	41019472	96	Sat21CF	na	na
chr31	41063064	41063851	788	Sat37CF	na	na
chr31	41272059	41272167	109	Sat5CF	stalk	na
chr31	41272071	41272169	99	Sat108CF	na	na
chr31	42227161	42227398	238	Sat29CF	na	na
chr31	42227187	42228193	1007	Sat29CF	na	na
chr31	42253948	42254625	678	Sat2CF	purple	na
chr31	42258576	42260013	1438	SAT1_CF	blue	na
chr32	3003993	3004385	393	SAT1_CF	blue	pericen
chr32	3006408	3006950	543	Sat2CF	purple	pericen
chr32	3006413	3006896	484	Sat2CF	purple	pericen
chr32	3009074	3010469	1396	CarSat1	red	pericen
chr32	3011059	3011387	329	Carsat2	orange	pericen
chr32	3011582	3013863	2282	Carsat2	orange	pericen
chr32	3013975	3014504	530	Carsat2	orange	pericen
chr32	3015096	3015358	263	Carsat2	orange	pericen
chr32	4873967	4874021	55	Sat12CF	na	pericen
chr32	7163862	7163936	75	Sat38CF	na	na
chr32	14554477	14554685	209	Carsat3	green	na
chr32	14826091	14826157	67	Sat59CF	na	na
chr32	23961414	23961476	63	Sat165CF	na	na
chr32	27181009	27181076	68	SUBTEL_sa	na	na
chr32	38782843	38783041	199	Carsat3	green	na
chr32	40656826	40657029	204	Carsat3	green	na
chr32	41263194	41263239	46	Sat8CF	dgreen	na
chr32	41721849	41722552	704	CarSat1	red	na
chr32	41722573	41724164	1592	CarSat1	red	na
chr32	41724185	41725426	1242	CarSat1	red	na

chr32	41725428	41725899	472	SAT1_CF	blue	na
chr32	41725911	41728713	2803	SAT1_CF	blue	na
chr32	41728731	41729251	521	SAT1_CF	blue	na
chr32	41729264	41729879	616	SAT1_CF	blue	na
chr32	41729874	41730592	719	CarSat1	red	na
chr32	41730743	41731048	306	CarSat1	red	na
chr33	3000078	3000371	294	CarSat1	red	pericen
chr33	5459011	5459117	107	Sat21CF	na	na
chr33	7480512	7480548	37	Sat86CF	na	na
chr33	8446010	8446110	101	Sat31CF	na	na
chr33	11646014	11646079	66	Sat77CF	na	na
chr33	17766693	17766738	46	Sat151CF	na	na
chr33	17766702	17767729	1028	Sat151CF	na	na
chr33	17767510	17767754	245	Sat151CF	na	na
chr33	20260917	20260988	72	Sat62CF	na	na
chr33	21034803	21035216	414	Sat24CF	na	na
chr33	21243729	21243777	49	Sat7CF	lime	na
chr33	21504100	21504205	106	Carsat3	green	na
chr33	21504316	21504867	552	Carsat3	green	na
chr33	22337607	22337661	55	Sat15CF	na	na
chr33	23280009	23280062	54	Sat112CF	na	na
chr33	26161659	26162861	1203	Sat149CF	na	na
chr33	26161837	26163026	1190	Sat149CF	na	na
chr33	26353372	26353398	27	SUBTEL_sa	na	na
chr33	29216605	29216847	243	Sat123CF	na	na
chr33	29602474	29602566	93	Sat88CF	na	na
chr33	29786806	29786918	113	Sat74CF	na	na
chr33	29896211	29896370	160	CarSat1	red	na
chr33	30562372	30562433	62	Sat139CF	na	na
chr33	30597192	30597228	37	Sat12CF	na	na
chr33	31062583	31062639	57	Sat21CF	na	na
chr33	31235316	31236018	703	CarSat1	red	na
chr33	31776357	31777505	1149	Sat152CF	na	na
chr33	31777507	31777535	29	Sat152CF	na	na
chr33	32125108	32126327	1220	Sat35CF	na	na
chr33	32125243	32129984	4742	Sat35CF	na	na
chr33	32127997	32130254	2258	Sat35CF	na	na
chr33	32283073	32283249	177	Sat99CF	na	na
chr33	32283779	32284211	433	Sat83CF	na	na
chr33	32331206	32331263	58	Sat65CF	na	na
chr33	33325825	33326090	266	Sat14CF	na	na
chr33	33439576	33439625	50	Sat6CF	select	na

chr33	33617537	33617763	227	Sat39CF	na	na
chr33	33830688	33830723	36	Sat50CF	na	na
chr33	34413813	34415135	1323	Carsat2	orange	na
chr33	34415171	34416592	1422	Carsat2	orange	na
chr33	34423376	34424071	696	Carsat2	orange	na
chr34	3000218	3000523	306	CarSat1	red	pericen
chr34	3000678	3001400	723	CarSat1	red	pericen
chr34	3001411	3003737	2327	SAT1_CF	blue	pericen
chr34	3003783	3004956	1174	SAT1_CF	blue	pericen
chr34	3004978	3005601	624	SAT1_CF	blue	pericen
chr34	3005641	3006041	401	SAT1_CF	blue	pericen
chr34	3006088	3006636	549	SAT1_CF	blue	pericen
chr34	3006678	3007202	525	SAT1_CF	blue	pericen
chr34	3007213	3009024	1812	SAT1_CF	blue	pericen
chr34	3009029	3011739	2711	CarSat1	red	pericen
chr34	3011760	3012284	525	CarSat1	red	pericen
chr34	3012684	3013804	1121	Sat2CF	purple	pericen
chr34	3013675	3013852	178	Sat2CF	purple	pericen
chr34	3028935	3029793	859	Sat2CF	purple	pericen
chr34	3043659	3044730	1072	Sat2CF	purple	pericen
chr34	3089823	3090344	522	Sat2CF	purple	pericen
chr34	3089887	3090715	829	Sat2CF	purple	pericen
chr34	3097407	3097559	153	CarSat1	red	pericen
chr34	3123942	3125564	1623	Carsat2	orange	pericen
chr34	6684056	6684336	281	Sat60CF	na	na
chr34	8438836	8439075	240	Sat18CF	na	na
chr34	10884230	10884335	106	Sat63CF	na	na
chr34	11378702	11378895	194	Sat120CF	na	na
chr34	11378896	11380778	1883	Sat120CF	na	na
chr34	11380779	11380831	53	Sat120CF	na	na
chr34	11604501	11604828	328	Sat5CF	stalk	na
chr34	11604534	11604862	329	Sat5CF	stalk	na
chr34	11604575	11604798	224	Sat5CF	stalk	na
chr34	13449412	13449676	265	Sat57CF	na	na
chr34	13449442	13449677	236	Sat57CF	na	na
chr34	13449547	13449768	222	Sat57CF	na	na
chr34	13477358	13477707	350	Sat72CF	na	na
chr34	13541580	13541631	52	Sat88CF	na	na
chr34	13682240	13682320	81	Sat107CF	na	na
chr34	14102384	14102686	303	Sat93CF	na	na
chr34	14102413	14102624	212	Sat93CF	na	na
chr34	14224936	14224991	56	Sat72CF	na	na

chr34	14513501	14513939	439	Sat74CF	na	na
chr34	14513553	14513841	289	Sat74CF	na	na
chr34	14522859	14522940	82	Sat97CF	na	na
chr34	14818401	14822334	3934	Sat109CF	na	na
chr34	14822260	14822361	102	Sat109CF	na	na
chr34	14914593	14914690	98	Sat104CF	na	na
chr34	15090687	15090836	150	Sat2CF	purple	na
chr34	15090726	15090829	104	Sat2CF	purple	na
chr34	15106894	15106946	53	SAT1_CF	blue	na
chr34	15107253	15107489	237	SAT1_CF	blue	na
chr34	15107822	15108061	240	SAT1_CF	blue	na
chr34	15122604	15123184	581	SAT1_CF	blue	na
chr34	15123580	15123646	67	SAT1_CF	blue	na
chr34	15146423	15146589	167	Sat2CF	purple	na
chr34	15146423	15146589	167	Sat2CF	purple	na
chr34	15232619	15232824	206	Sat2CF	purple	na
chr34	15232657	15232842	186	Sat2CF	purple	na
chr34	15232669	15232878	210	Sat2CF	purple	na
chr34	15245285	15245536	252	SAT1_CF	blue	na
chr34	15245840	15245996	157	SAT1_CF	blue	na
chr34	15273636	15273703	68	SAT1_CF	blue	na
chr34	15295922	15296298	377	Sat2CF	purple	na
chr34	15295991	15296245	255	Sat2CF	purple	na
chr34	15300997	15301096	100	Carsat2	orange	na
chr34	15309060	15309276	217	Sat2CF	purple	na
chr34	15309170	15309231	62	Sat2CF	purple	na
chr34	15313603	15313690	88	SAT1_CF	blue	na
chr34	15343387	15343450	64	SAT1_CF	blue	na
chr34	15343749	15343999	251	SAT1_CF	blue	na
chr34	15365335	15365513	179	Sat2CF	purple	na
chr34	15377459	15377570	112	SAT1_CF	blue	na
chr34	15392881	15392987	107	SAT1_CF	blue	na
chr34	15415197	15415255	59	SAT1_CF	blue	na
chr34	15415707	15415794	88	SAT1_CF	blue	na
chr34	15442157	15442333	177	Sat2CF	purple	na
chr34	15442201	15442487	287	Sat2CF	purple	na
chr34	15442293	15442533	241	Sat2CF	purple	na
chr34	15451528	15451589	62	SAT1_CF	blue	na
chr34	15452141	15452249	109	SAT1_CF	blue	na
chr34	15478190	15478524	335	Sat2CF	purple	na
chr34	15478235	15478539	305	Sat2CF	purple	na
chr34	15478275	15478512	238	Sat2CF	purple	na

chr34	15478303	15478577	275	Sat2CF	purple	na
chr34	15483408	15483687	280	Carsat2	orange	na
chr34	15496979	15497068	90	SAT1_CF	blue	na
chr34	15508081	15508177	97	Carsat2	orange	na
chr34	19564603	19564667	65	SUBTEL_sa	na	na
chr34	23123954	23124061	108	Carsat3	green	na
chr34	27866434	27866517	84	Sat36CF	na	na
chr34	29324844	29324967	124	Sat105CF	na	na
chr34	29325187	29325303	117	Sat105CF	na	na
chr34	30400736	30400773	38	Sat179CF	na	na
chr34	30593635	30594304	670	Sat7CF	lime	na
chr34	30593730	30594289	560	Sat7CF	lime	na
chr34	31984478	31984592	115	Sat32CF	na	na
chr34	32856931	32857597	667	Sat7CF	lime	na
chr34	32856946	32857502	557	Sat7CF	lime	na
chr34	34522926	34522975	50	Sat36CF	na	na
chr34	37304447	37304624	178	CarSat1	red	na
chr34	37304617	37304680	64	SAT1_CF	blue	na
chr34	38659055	38659114	60	Sat2CF	purple	na
chr34	39880962	39881060	99	Sat52CF	na	na
chr34	40591852	40591879	28	Sat53CF	na	na
chr34	44069108	44069391	284	Sat44CF	na	na
chr34	44338564	44339691	1128	Sat184CF	na	na
chr34	44414733	44414832	100	Sat27CF	na	na
chr34	44559485	44559830	346	Sat16CF	na	na
chr34	44680131	44680441	311	Sat14CF	na	na
chr34	44813398	44813600	203	Sat43CF	na	na
chr34	44965507	44965545	39	Sat96CF	na	na
chr34	45119549	45119852	304	Sat29CF	na	na
chr35	3016318	3017041	724	Sat2CF	purple	pericen
chr35	3016898	3017060	163	Sat2CF	purple	pericen
chr35	3035569	3036031	463	Sat2CF	purple	pericen
chr35	3061799	3061919	121	Sat2CF	purple	pericen
chr35	3061814	3061923	110	Sat2CF	purple	pericen
chr35	3724864	3725318	455	Sat42CF	na	pericen
chr35	4106968	4107077	110	Sat62CF	na	pericen
chr35	5016191	5016425	235	Sat21CF	na	na
chr35	11338762	11339733	972	Sat39CF	na	na
chr35	19264291	19264464	174	SAT1_CF	blue	na
chr35	19383877	19383946	70	Sat31CF	na	na
chr35	21344610	21344660	51	SUBTEL_sa	na	na
chr35	23134541	23134572	32	Sat11CF	na	na

chr35	24844183	24844260	78	Sat42CF	na	na
chr35	27396573	27396669	97	Carsat3	green	na
chr35	27396952	27397618	667	Carsat3	green	na
chr35	27726186	27727591	1406	Carsat3	green	na
chr35	27740793	27741463	671	Carsat3	green	na
chr35	27741650	27741679	30	Carsat3	green	na
chr35	27741690	27741938	249	Carsat3	green	na
chr35	27761759	27762556	798	Carsat3	green	na
chr35	27765790	27766425	636	Sat103CF	na	na
chr35	27765857	27766889	1033	Sat103CF	na	na
chr35	27766968	27767314	347	Sat103CF	na	na
chr35	27767504	27768862	1359	Sat103CF	na	na
chr35	27768663	27768885	223	Sat103CF	na	na
chr35	27794682	27796278	1597	Carsat3	green	na
chr35	28271167	28271694	528	Carsat3	green	na
chr35	28277093	28277225	133	Carsat3	green	na
chr35	28277257	28277445	189	Carsat3	green	na
chr35	28287142	28287939	798	Carsat3	green	na
chr35	28305828	28306237	410	Carsat3	green	na
chr35	28335173	28335963	791	Carsat3	green	na
chr35	28343956	28344118	163	Carsat3	green	na
chr35	28344229	28344537	309	Carsat3	green	na
chr35	28361824	28362405	582	Carsat3	green	na
chr35	28362448	28362808	361	Carsat3	green	na
chr35	28401964	28402131	168	Carsat3	green	na
chr35	28402232	28402624	393	Carsat3	green	na
chr35	28433324	28434043	720	Carsat3	green	na
chr35	28474454	28475047	594	Carsat3	green	na
chr35	28514550	28514942	393	Carsat3	green	na
chr35	28515043	28515210	168	Carsat3	green	na
chr35	28537443	28537886	444	Carsat3	green	na
chr35	28546496	28547534	1039	Carsat3	green	na
chr35	28568056	28568462	407	Carsat3	green	na
chr35	28584239	28584882	644	Carsat3	green	na
chr35	28999009	28999223	215	Carsat3	green	na
chr35	29403467	29403707	241	Sat74CF	na	na
chr35	29473840	29474512	673	Carsat2	orange	na
chr35	29474691	29475541	851	Carsat2	orange	na
chr35	29475718	29477627	1910	Carsat2	orange	na
chr35	29478874	29479226	353	Carsat2	orange	na
chr35	29539909	29540432	524	Carsat2	orange	na
chr35	29540639	29542582	1944	Carsat2	orange	na

chr36	3491042	3491547	506	Sat13CF	na	pericen
chr36	9161650	9161704	55	Sat104CF	na	na
chr36	10699068	10699286	219	Sat17CF	na	na
chr36	11199656	11199737	82	Sat27CF	na	na
chr36	13184296	13185938	1643	Sat50CF	na	na
chr36	13640354	13640427	74	Sat26CF	na	na
chr36	15118521	15118552	32	Sat46CF	na	na
chr36	16047531	16047573	43	Sat137CF	na	na
chr36	23858453	23858645	193	CarSat1	red	na
chr36	23994835	23995349	515	Sat8CF	dgreen	na
chr36	23996465	23996967	503	Sat8CF	dgreen	na
chr36	23996630	23996968	339	Sat8CF	dgreen	na
chr36	25295446	25295546	101	Sat61CF	na	na
chr36	25296709	25296818	110	Sat61CF	na	na
chr36	25297007	25297123	117	Sat61CF	na	na
chr36	25297266	25297360	95	Sat61CF	na	na
chr36	25297614	25297736	123	Sat61CF	na	na
chr36	25298194	25298247	54	Sat61CF	na	na
chr36	25298625	25299602	978	Sat61CF	na	na
chr36	25299494	25299617	124	Sat61CF	na	na
chr36	25299693	25299803	111	Sat61CF	na	na
chr36	25299709	25301157	1449	Sat61CF	na	na
chr36	25300096	25301585	1490	Sat61CF	na	na
chr36	25300287	25301777	1491	Sat61CF	na	na
chr36	25301717	25301791	75	Sat61CF	na	na
chr36	25302006	25302329	324	Sat61CF	na	na
chr36	25302590	25302668	79	Sat61CF	na	na
chr36	25304313	25304922	610	Sat61CF	na	na
chr36	25304422	25304965	544	Sat61CF	na	na
chr36	25304449	25304922	474	Sat61CF	na	na
chr36	25306428	25306527	100	Sat61CF	na	na
chr36	25307069	25307185	117	Sat61CF	na	na
chr36	25307720	25307825	106	Sat61CF	na	na
chr36	25309405	25309429	25	Sat61CF	na	na
chr36	25310677	25310771	95	Sat61CF	na	na
chr36	25312040	25312136	97	Sat61CF	na	na
chr36	25312660	25312781	122	Sat61CF	na	na
chr36	25312710	25312793	84	Sat61CF	na	na
chr36	25314061	25314155	95	Sat61CF	na	na
chr36	25314073	25314155	83	Sat61CF	na	na
chr36	25314500	25314545	46	Sat130CF	na	na
chr36	25314520	25314545	26	Sat51CF	na	na

chr36	25315017	25315118	102	Sat61CF	na	na
chr36	25315287	25315403	117	Sat61CF	na	na
chr36	25315304	25315403	100	Sat61CF	na	na
chr36	25315641	25315755	115	Sat61CF	na	na
chr36	25316209	25316303	95	Sat61CF	na	na
chr36	25316472	25316608	137	Sat61CF	na	na
chr36	25317149	25317251	103	Sat61CF	na	na
chr36	25321866	25321968	103	Sat61CF	na	na
chr36	25326089	25326182	94	Sat61CF	na	na
chr36	25329694	25329807	114	Sat61CF	na	na
chr36	28066013	28066817	805	Sat150CF	na	na
chr36	28066040	28066912	873	Sat64CF	na	na
chr36	31936356	31937679	1324	Sat95CF	na	na
chr36	32075425	32075465	41	Sat185CF	na	na
chr36	33222037	33222392	356	Sat94CF	na	na
chr37	3002799	3003510	712	CarSat1	red	pericen
chr37	3005142	3005339	198	Sat2CF	purple	pericen
chr37	3005210	3006294	1085	Sat2CF	purple	pericen
chr37	3027394	3028501	1108	Sat2CF	purple	pericen
chr37	3051274	3051632	359	Sat2CF	purple	pericen
chr37	3051351	3051695	345	Sat2CF	purple	pericen
chr37	3064180	3065255	1076	Sat2CF	purple	pericen
chr37	3088743	3089922	1180	Sat2CF	purple	pericen
chr37	3113193	3113700	508	Sat2CF	purple	pericen
chr37	3113246	3114583	1338	Sat2CF	purple	pericen
chr37	3142173	3142732	560	SAT1_CF	blue	pericen
chr37	3732909	3733291	383	Sat4CF	lred	pericen
chr37	4503586	4503976	391	Sat14CF	na	pericen
chr37	6790761	6791050	290	Sat18CF	na	na
chr37	8759079	8759492	414	Sat82CF	na	na
chr37	8759122	8759994	873	Sat82CF	na	na
chr37	9258638	9258804	167	CarSat1	red	na
chr37	9287051	9287202	152	Sat17CF	na	na
chr37	9287385	9287470	86	Sat17CF	na	na
chr37	13849309	13849372	64	Sat165CF	na	na
chr37	15375205	15375675	471	Sat17CF	na	na
chr37	16905874	16905960	87	Sat62CF	na	na
chr37	25784412	25784456	45	Sat139CF	na	na
chr37	25850831	25850914	84	Sat77CF	na	na
chr37	27782787	27783062	276	Sat30CF	na	na
chr37	30562301	30562412	112	Sat61CF	na	na
chr37	31800834	31801149	316	Sat65CF	na	na

chr37	31800854	31801132	279	Sat65CF	na	na
chr37	32001043	32001250	208	Sat42CF	na	na
chr37	33871061	33871801	741	Sat4CF	lred	na
chr38	3428370	3428419	50	Sat109CF	na	pericen
chr38	3975198	3975256	59	Sat52CF	na	pericen
chr38	16375482	16376726	1245	Sat137CF	na	na
chr38	17900830	17901187	358	Sat38CF	na	na
chr38	17976848	17976890	43	Sat45CF	na	na
chr38	18020983	18021079	97	Sat107CF	na	na
chr38	19431261	19431489	229	Sat140CF	na	na
chr38	19431290	19431489	200	Sat140CF	na	na
chr38	19800080	19800181	102	Sat42CF	na	na
chr38	21381053	21381121	69	Sat63CF	na	na
chr38	24257956	24258038	83	Sat107CF	na	na
chr38	24641354	24641517	164	CarSat1	red	na
chr38	24987467	24987500	34	Sat11CF	na	na
chr38	25202886	25203049	164	Sat51CF	na	na
chr38	25231901	25232303	403	CarSat1	red	na
chr38	25320995	25322765	1771	Sat166CF	na	na
chr38	25322766	25322854	89	Sat166CF	na	na
chr38	25806326	25807025	700	Sat28CF	na	na
chr38	26526592	26528232	1641	Sat68CF	na	na
chr38	26528202	26528237	36	Sat68CF	na	na
chr38	26579624	26579888	265	Sat74CF	na	na
chr38	26587070	26588145	1076	Sat69CF	na	na
chr38	26893494	26894756	1263	SAT1_CF	blue	na
chr38	26897623	26897726	104	Sat2CF	purple	na
chr4	3153148	3154074	927	Carsat3	green	pericen
chr4	3154085	3154235	151	Carsat3	green	pericen
chr4	3263752	3264634	883	Carsat3	green	pericen
chr4	3264667	3264793	127	Carsat3	green	pericen
chr4	3333315	3335012	1698	Carsat3	green	pericen
chr4	3394186	3394311	126	Carsat3	green	pericen
chr4	3394355	3395876	1522	Carsat3	green	pericen
chr4	3434857	3436229	1373	Carsat3	green	pericen
chr4	3436427	3436500	74	Carsat3	green	pericen
chr4	5914923	5915060	138	Carsat3	green	na
chr4	5915165	5915395	231	Carsat3	green	na
chr4	8485816	8485893	78	Carsat2	orange	na
chr4	8643305	8643614	310	Sat51CF	na	na
chr4	8643337	8643602	266	Sat51CF	na	na
chr4	10002922	10002988	67	Sat58CF	na	na

chr4	10523727	10523986	260	Sat60CF	na	na
chr4	10523750	10524014	265	Sat97CF	na	na
chr4	12206107	12206158	52	Sat69CF	na	na
chr4	12505827	12505881	55	Sat37CF	na	na
chr4	13511576	13511962	387	Carsat2	orange	na
chr4	13512176	13512598	423	Carsat2	orange	na
chr4	13512609	13513744	1136	Carsat2	orange	na
chr4	13513911	13514745	835	Carsat2	orange	na
chr4	13532340	13532805	466	Carsat2	orange	na
chr4	14945683	14945973	291	Sat17CF	na	na
chr4	16916896	16917385	490	Sat17CF	na	na
chr4	17516166	17516219	54	Sat110CF	na	na
chr4	17784193	17784325	133	CarSat1	red	na
chr4	23762145	23762428	284	Sat28CF	na	na
chr4	26167268	26167501	234	Sat50CF	na	na
chr4	26167284	26167482	199	Sat50CF	na	na
chr4	28755779	28755980	202	Sat12CF	na	na
chr4	30469997	30470064	68	Sat36CF	na	na
chr4	30765053	30765682	630	CarSat1	red	na
chr4	31448019	31448169	151	Sat49CF	na	na
chr4	33694129	33694186	58	Sat63CF	na	na
chr4	36743575	36743945	371	Sat47CF	na	na
chr4	36743653	36743958	306	Sat18CF	na	na
chr4	36888128	36888586	459	Sat64CF	na	na
chr4	36888129	36888642	514	Sat64CF	na	na
chr4	36961643	36961795	153	Sat53CF	na	na
chr4	37728604	37728952	349	Sat47CF	na	na
chr4	37779962	37780029	68	Sat21CF	na	na
chr4	37991493	37992066	574	Sat19CF	na	na
chr4	38331642	38332305	664	Carsat2	orange	na
chr4	38358961	38359703	743	CarSat1	red	na
chr4	38365927	38368704	2778	CarSat1	red	na
chr4	38368717	38369141	425	CarSat1	red	na
chr4	38379095	38380224	1130	Carsat2	orange	na
chr4	38380276	38380684	409	Carsat2	orange	na
chr4	38380883	38381505	623	Carsat2	orange	na
chr4	38395185	38395400	216	Carsat2	orange	na
chr4	38395567	38395634	68	Carsat2	orange	na
chr4	38396076	38397235	1160	Carsat2	orange	na
chr4	38397424	38397438	15	Carsat2	orange	na
chr4	38397541	38397633	93	Carsat2	orange	na
chr4	38397882	38399357	1476	Carsat2	orange	na

chr4	38399350	38400754	1405	CarSat1	red	na
chr4	38400972	38401517	546	CarSat1	red	na
chr4	38404507	38407662	3156	CarSat1	red	na
chr4	38407468	38408267	800	Carsat2	orange	na
chr4	38407851	38408338	488	CarSat1	red	na
chr4	38413660	38414393	734	Carsat2	orange	na
chr4	38414509	38415051	543	Carsat2	orange	na
chr4	38415283	38415882	600	Carsat2	orange	na
chr4	38415911	38416113	203	Carsat2	orange	na
chr4	38416276	38417074	799	Carsat2	orange	na
chr4	38417206	38418154	949	Carsat2	orange	na
chr4	38418966	38420247	1282	Carsat2	orange	na
chr4	38422918	38424201	1284	Carsat2	orange	na
chr4	38424397	38425680	1284	Carsat2	orange	na
chr4	38425866	38425973	108	CarSat1	red	na
chr4	43917168	43917227	60	Sat107CF	na	na
chr4	45520721	45520795	75	Sat12CF	na	na
chr4	55171845	55171920	76	Sat109CF	na	na
chr4	59244519	59244578	60	Sat94CF	na	na
chr4	61353947	61354025	79	Sat150CF	na	na
chr4	61399648	61399939	292	Sat5CF	stalk	na
chr4	61439010	61439718	709	Carsat3	green	na
chr4	61479483	61480602	1120	Carsat3	green	na
chr4	61507760	61508934	1175	Carsat3	green	na
chr4	63150623	63150761	139	Sat5CF	stalk	na
chr4	66498818	66498890	73	CarSat1	red	na
chr4	70867682	70868256	575	Carsat3	green	na
chr4	73436287	73436537	251	Sat17CF	na	na
chr4	73436308	73436411	104	Sat17CF	na	na
chr4	73436410	73436497	88	Sat17CF	na	na
chr4	76870835	76871163	329	Sat7CF	lime	na
chr4	81421154	81421236	83	Sat77CF	na	na
chr4	83027623	83027659	37	Sat108CF	na	na
chr4	84810523	84811055	533	Sat137CF	na	na
chr4	87622024	87622193	170	Sat47CF	na	na
chr4	89049659	89049937	279	Sat74CF	na	na
chr4	90565466	90565592	127	Sat129CF	na	na
chr4	90565500	90567088	1589	Sat129CF	na	na
chr4	90566987	90567128	142	Sat129CF	na	na
chr4	90829823	90830558	736	Sat82CF	na	na
chr4	91483001	91483774	774	Carsat2	orange	na
chr5	3104337	3104874	538	SAT1_CF	blue	pericen

chr5	3287415	3287615	201	Sat13CF	na	pericen
chr5	3287743	3289114	1372	Sat13CF	na	pericen
chr5	3349950	3351755	1806	Sat49CF	na	pericen
chr5	3931265	3931409	145	Sat42CF	na	pericen
chr5	3931342	3931409	68	Sat42CF	na	pericen
chr5	4376400	4377844	1445	Sat113CF	na	pericen
chr5	7314136	7314168	33	Sat94CF	na	na
chr5	7513878	7514255	378	Sat38CF	na	na
chr5	7513929	7514269	341	Sat38CF	na	na
chr5	7839065	7839742	678	Carsat3	green	na
chr5	7839974	7840193	220	Carsat3	green	na
chr5	9682522	9682647	126	Sat27CF	na	na
chr5	10349000	10349050	51	Sat59CF	na	na
chr5	11848393	11848663	271	Sat108CF	na	na
chr5	11848407	11848629	223	Sat108CF	na	na
chr5	12200885	12200922	38	Sat103CF	na	na
chr5	13496797	13496829	33	Sat139CF	na	na
chr5	13725211	13725366	156	Carsat3	green	na
chr5	13725447	13725673	227	Carsat3	green	na
chr5	13725815	13725955	141	Carsat3	green	na
chr5	17355426	17355514	89	SAT1_CF	blue	na
chr5	17396775	17396848	74	Sat21CF	na	na
chr5	18672915	18672972	58	Sat39CF	na	na
chr5	18752121	18752249	129	CarSat1	red	na
chr5	19751170	19751244	75	SUBTEL_sa	na	na
chr5	21378362	21378479	118	Sat9CF	na	na
chr5	22099920	22100094	175	Carsat3	green	na
chr5	26712074	26712177	104	Sat17CF	na	na
chr5	26712378	26712520	143	Sat17CF	na	na
chr5	33026342	33027364	1023	Sat2CF	purple	na
chr5	33027220	33027382	163	Sat2CF	purple	na
chr5	33085406	33085767	362	Sat7CF	lime	na
chr5	33085552	33085797	246	Sat7CF	lime	na
chr5	33092007	33092088	82	Carsat2	orange	na
chr5	33092342	33092834	493	Carsat2	orange	na
chr5	33093033	33093079	47	CarSat1	red	na
chr5	33093104	33093586	483	Carsat2	orange	na
chr5	33100719	33100911	193	CarSat1	red	na
chr5	33102937	33103252	316	Carsat2	orange	na
chr5	33105323	33105932	610	Sat2CF	purple	na
chr5	33105392	33106737	1346	Sat2CF	purple	na
chr5	33105562	33106863	1302	Sat2CF	purple	na

chr5	33105914	33107174	1261	Sat2CF	purple	na
chr5	33107081	33108386	1306	Sat2CF	purple	na
chr5	33108345	33109611	1267	Sat2CF	purple	na
chr5	33109697	33110949	1253	Sat2CF	purple	na
chr5	33110960	33111082	123	Sat2CF	purple	na
chr5	33111083	33112327	1245	Sat2CF	purple	na
chr5	33112330	33112884	555	Sat2CF	purple	na
chr5	33554451	33554705	255	Sat18CF	na	na
chr5	34076634	34076734	101	Sat72CF	na	na
chr5	34578651	34578818	168	Carsat3	green	na
chr5	34578851	34579748	898	Carsat3	green	na
chr5	34597176	34597415	240	Carsat3	green	na
chr5	35730591	35730992	402	CarSat1	red	na
chr5	36496178	36496574	397	Sat53CF	na	na
chr5	36496184	36496465	282	Sat21CF	na	na
chr5	36557292	36557361	70	Sat89CF	na	na
chr5	36812855	36813004	150	Sat43CF	na	na
chr5	37132279	37132374	96	SAT1_CF	blue	na
chr5	38967394	38967561	168	Sat60CF	na	na
chr5	39020625	39020831	207	Sat161CF	na	na
chr5	39020648	39021671	1024	Sat161CF	na	na
chr5	39053782	39054168	387	Carsat3	green	na
chr5	39536976	39537023	48	Sat118CF	na	na
chr5	39943401	39943480	80	Sat10CF	na	na
chr5	40436888	40436937	50	Sat2CF	purple	na
chr5	41161391	41161557	167	Sat17CF	na	na
chr5	42191159	42191288	130	Carsat3	green	na
chr5	42191334	42191462	129	Carsat3	green	na
chr5	42202690	42204109	1420	Carsat3	green	na
chr5	42244122	42245066	945	Carsat3	green	na
chr5	42276441	42276901	461	Carsat3	green	na
chr5	42276912	42277845	934	Carsat3	green	na
chr5	42278033	42278210	178	Carsat3	green	na
chr5	42278262	42278448	187	Carsat3	green	na
chr5	42320913	42322706	1794	Carsat3	green	na
chr5	42398606	42399877	1272	Carsat3	green	na
chr5	42412540	42412649	110	Carsat3	green	na
chr5	42412831	42412877	47	Carsat3	green	na
chr5	42413090	42413210	121	Carsat3	green	na
chr5	42413579	42413847	269	Carsat3	green	na
chr5	42534165	42534848	684	Sat17CF	na	na
chr5	44519507	44519593	87	Sat69CF	na	na

chr5	44712034	44712093	60	SUBTEL_sa	na	na
chr5	45249743	45249790	48	Sat129CF	na	na
chr5	45888666	45888766	101	Carsat3	green	na
chr5	46244734	46244796	63	Sat32CF	na	na
chr5	50891269	50891496	228	Sat17CF	na	na
chr5	51387602	51388224	623	Sat44CF	na	na
chr5	53417388	53417443	56	Sat2CF	purple	na
chr5	57579268	57579367	100	Sat5CF	stalk	na
chr5	59092110	59092318	209	SAT1_CF	blue	na
chr5	59262975	59263088	114	Sat16CF	na	na
chr5	60351913	60352106	194	Sat104CF	na	na
chr5	60553878	60554083	206	Sat5CF	stalk	na
chr5	60690262	60690332	71	Sat22CF	na	na
chr5	60749173	60749277	105	Sat5CF	stalk	na
chr5	60892561	60892609	49	Sat16CF	na	na
chr5	60968938	60969307	370	Sat72CF	na	na
chr5	60988760	60988887	128	Carsat3	green	na
chr5	61442217	61442254	38	Sat150CF	na	na
chr5	62110604	62111326	723	Sat65CF	na	na
chr5	62110626	62111547	922	Sat24CF	na	na
chr5	62982291	62982514	224	Sat166CF	na	na
chr5	63940929	63941139	211	Sat43CF	na	na
chr5	63947944	63948069	126	Sat180CF	na	na
chr5	63947996	63949115	1120	Sat180CF	na	na
chr5	65273036	65273079	44	Sat63CF	na	na
chr5	66553210	66553474	265	Sat2CF	purple	na
chr5	66553230	66553502	273	Sat2CF	purple	na
chr5	66566318	66566775	458	Carsat3	green	na
chr5	67468584	67469117	534	Sat112CF	na	na
chr5	67468787	67470246	1460	Sat112CF	na	na
chr5	67470204	67470251	48	Sat112CF	na	na
chr5	68136475	68137195	721	Sat60CF	na	na
chr5	69827318	69827438	121	Sat18CF	na	na
chr5	70473125	70474363	1239	Sat41CF	na	na
chr5	70501516	70501592	77	Sat9CF	na	na
chr5	73420956	73421009	54	Sat25CF	na	na
chr5	76273343	76273424	82	Sat58CF	na	na
chr5	76985756	76985947	192	Sat12CF	na	na
chr5	78548654	78549361	708	Carsat3	green	na
chr5	80253727	80255246	1520	Carsat3	green	na
chr5	80278287	80279070	784	Carsat3	green	na
chr5	80298666	80298832	167	Carsat3	green	na

chr5	80307115	80307251	137	Carsat3	green	na
chr5	80307435	80307920	486	Carsat3	green	na
chr5	83997955	83998723	769	Carsat3	green	na
chr5	83998739	83999240	502	Carsat3	green	na
chr5	83999257	83999422	166	Carsat3	green	na
chr5	84615900	84615945	46	Sat52CF	na	na
chr5	84929457	84929539	83	Sat110CF	na	na
chr5	84981437	84981567	131	Sat5CF	stalk	na
chr5	86044221	86044296	76	CarSat1	red	na
chr5	86248335	86248559	225	Sat28CF	na	na
chr5	87660125	87660447	323	Sat119CF	na	na
chr5	87660160	87662369	2210	Sat119CF	na	na
chr5	90615022	90615934	913	Sat111CF	na	na
chr5	90992351	90992858	508	Sat85CF	na	na
chr5	91060107	91060203	97	Sat101CF	na	na
chr5	91060120	91061524	1405	Sat101CF	na	na
chr5	91061299	91061550	252	Sat101CF	na	na
chr5	91975121	91975868	748	Carsat2	orange	na
chr6	3000002	3001036	1035	CarSat1	red	pericen
chr6	3001078	3001532	455	CarSat1	red	pericen
chr6	3001548	3002511	964	CarSat1	red	pericen
chr6	3002538	3003248	711	CarSat1	red	pericen
chr6	3003275	3004723	1449	CarSat1	red	pericen
chr6	3004750	3010352	5603	CarSat1	red	pericen
chr6	3010377	3011834	1458	CarSat1	red	pericen
chr6	3011850	3013288	1439	CarSat1	red	pericen
chr6	3013321	3014026	706	CarSat1	red	pericen
chr6	3014059	3014934	876	CarSat1	red	pericen
chr6	3015130	3015843	714	CarSat1	red	pericen
chr6	3024488	3025079	592	CarSat1	red	pericen
chr6	3025275	3026375	1101	CarSat1	red	pericen
chr6	3026409	3026554	146	CarSat1	red	pericen
chr6	3026930	3027203	274	CarSat1	red	pericen
chr6	3033240	3033617	378	Sat2CF	purple	pericen
chr6	3033300	3034161	862	Sat2CF	purple	pericen
chr6	3033301	3034267	967	Sat2CF	purple	pericen
chr6	3034476	3035852	1377	SAT1_CF	blue	pericen
chr6	3035881	3036283	403	CarSat1	red	pericen
chr6	3036302	3036995	694	CarSat1	red	pericen
chr6	3037181	3038023	843	CarSat1	red	pericen
chr6	3038041	3038724	684	CarSat1	red	pericen
chr6	3055079	3055878	800	Sat2CF	purple	pericen

chr6	3068425	3069676	1252	Sat2CF	purple	pericen
chr6	3068555	3069640	1086	Sat2CF	purple	pericen
chr6	3072557	3073302	746	CarSat1	red	pericen
chr6	3073320	3074095	776	CarSat1	red	pericen
chr6	3074122	3074965	844	CarSat1	red	pericen
chr6	3075163	3075828	666	CarSat1	red	pericen
chr6	3085088	3085360	273	Carsat2	orange	pericen
chr6	3085116	3085771	656	CarSat1	red	pericen
chr6	3085789	3086631	843	CarSat1	red	pericen
chr6	3086818	3086960	143	CarSat1	red	pericen
chr6	3090843	3090989	147	Sat2CF	purple	pericen
chr6	3090865	3091941	1077	Sat2CF	purple	pericen
chr6	3173219	3173932	714	Sat2CF	purple	pericen
chr6	3173799	3173960	162	Sat2CF	purple	pericen
chr6	3273299	3274319	1021	SAT1_CF	blue	pericen
chr6	3274335	3277392	3058	SAT1_CF	blue	pericen
chr6	3277422	3277821	400	SAT1_CF	blue	pericen
chr6	3277819	3279888	2070	CarSat1	red	pericen
chr6	3279906	3281832	1927	CarSat1	red	pericen
chr6	3281852	3282468	617	CarSat1	red	pericen
chr6	3282878	3283006	129	CarSat1	red	pericen
chr6	3283192	3284211	1020	CarSat1	red	pericen
chr6	3284393	3285135	743	CarSat1	red	pericen
chr6	3285160	3285298	139	CarSat1	red	pericen
chr6	3360453	3360998	546	Carsat3	green	pericen
chr6	3361153	3361237	85	Carsat3	green	pericen
chr6	8202675	8202768	94	Sat104CF	na	na
chr6	8757646	8757891	246	Carsat2	orange	na
chr6	9313802	9313875	74	Sat96CF	na	na
chr6	9383155	9383364	210	Sat12CF	na	na
chr6	10824341	10824743	403	CarSat1	red	na
chr6	11611685	11611980	296	Carsat3	green	na
chr6	11628381	11628560	180	CarSat1	red	na
chr6	11812622	11814753	2132	Sat62CF	na	na
chr6	11814754	11818479	3726	Sat62CF	na	na
chr6	11818493	11818612	120	Sat62CF	na	na
chr6	11842185	11845260	3076	Sat115CF	na	na
chr6	11842747	11846220	3474	Sat115CF	na	na
chr6	11846280	11847122	843	Sat115CF	na	na
chr6	11847140	11847264	125	Sat115CF	na	na
chr6	12515725	12516259	535	Carsat3	green	na
chr6	12516276	12516413	138	Carsat3	green	na

chr6	12531703	12532549	847	Carsat3	green	na
chr6	12546759	12547352	594	Carsat3	green	na
chr6	12573296	12573437	142	Carsat3	green	na
chr6	12576471	12577050	580	Carsat3	green	na
chr6	12942585	12942963	379	Carsat3	green	na
chr6	12977838	12978083	246	Carsat3	green	na
chr6	12978098	12978883	786	Carsat3	green	na
chr6	12993121	12993976	856	Carsat3	green	na
chr6	12993987	12994479	493	Carsat3	green	na
chr6	12994584	12994674	91	Carsat3	green	na
chr6	13021909	13022018	110	Carsat3	green	na
chr6	13022278	13022656	379	Carsat3	green	na
chr6	13022705	13023175	471	Carsat3	green	na
chr6	13025736	13026088	353	Carsat3	green	na
chr6	13057399	13057974	576	Carsat3	green	na
chr6	13064633	13065366	734	Carsat3	green	na
chr6	13099627	13099769	143	Carsat2	orange	na
chr6	13102210	13103216	1007	Carsat3	green	na
chr6	13634604	13634719	116	Carsat3	green	na
chr6	13698673	13698729	57	Sat10CF	na	na
chr6	15033092	15033306	215	Carsat3	green	na
chr6	15033422	15035588	2167	Carsat3	green	na
chr6	15047604	15047830	227	Carsat3	green	na
chr6	15047844	15049216	1373	Carsat3	green	na
chr6	15102687	15103761	1075	Carsat3	green	na
chr6	15103773	15104332	560	Carsat3	green	na
chr6	15104385	15104541	157	Carsat3	green	na
chr6	15136636	15136998	363	Carsat3	green	na
chr6	16226943	16227183	241	Sat84CF	na	na
chr6	17622686	17622798	113	Sat5CF	stalk	na
chr6	17624130	17624624	495	Sat57CF	na	na
chr6	18552206	18552400	195	Sat70CF	na	na
chr6	19636141	19636874	734	Sat2CF	purple	na
chr6	19636718	19636894	177	Sat2CF	purple	na
chr6	19798345	19798459	115	Carsat3	green	na
chr6	20404065	20404555	491	Carsat3	green	na
chr6	20404977	20405196	220	Carsat3	green	na
chr6	20601270	20601390	121	Carsat3	green	na
chr6	20608446	20608851	406	Sat42CF	na	na
chr6	20609785	20609960	176	Carsat3	green	na
chr6	20647834	20648260	427	Carsat3	green	na
chr6	20648318	20648379	62	Carsat3	green	na

chr6	20716538	20716640	103	Carsat3	green	na
chr6	20716721	20716885	165	Carsat3	green	na
chr6	20944061	20944395	335	Sat19CF	na	na
chr6	20951784	20951857	74	CarSat1	red	na
chr6	22305765	22305889	125	Sat5CF	stalk	na
chr6	23837740	23837814	75	Sat179CF	na	na
chr6	23992055	23992355	301	Carsat3	green	na
chr6	25585395	25585716	322	Carsat2	orange	na
chr6	34590118	34590481	364	Sat28CF	na	na
chr6	34835010	34835264	255	Sat12CF	na	na
chr6	34835078	34835258	181	Sat12CF	na	na
chr6	35378354	35378413	60	Sat165CF	na	na
chr6	35516691	35516741	51	Sat45CF	na	na
chr6	35516699	35520853	4155	Sat45CF	na	na
chr6	35520912	35523575	2664	Sat45CF	na	na
chr6	36523217	36523462	246	Sat85CF	na	na
chr6	36523463	36525338	1876	Sat85CF	na	na
chr6	36525334	36525598	265	Sat85CF	na	na
chr6	36525635	36525793	159	Sat85CF	na	na
chr6	36525796	36527672	1877	Sat85CF	na	na
chr6	36527673	36527749	77	Sat85CF	na	na
chr6	36570023	36570097	75	Sat126CF	na	na
chr6	39703439	39703687	249	Carsat3	green	na
chr6	40839330	40839607	278	Carsat3	green	na
chr6	40839636	40839724	89	Carsat3	green	na
chr6	40839876	40840110	235	Carsat3	green	na
chr6	40863562	40863827	266	Carsat3	green	na
chr6	40880041	40880316	276	Carsat3	green	na
chr6	40963221	40963648	428	Carsat3	green	na
chr6	40984374	40985150	777	Carsat3	green	na
chr6	41042419	41042699	281	Carsat3	green	na
chr6	41127000	41127162	163	Carsat3	green	na
chr6	41127419	41127581	163	Carsat3	green	na
chr6	41783943	41784007	65	Sat89CF	na	na
chr6	42134939	42136364	1426	Sat117CF	na	na
chr6	42148750	42148823	74	Sat107CF	na	na
chr6	42212458	42213526	1069	Sat159CF	na	na
chr6	42566330	42566684	355	Sat5CF	stalk	na
chr6	42903984	42904206	223	SAT1_CF	blue	na
chr6	43314588	43314805	218	Sat30CF	na	na
chr6	43494570	43494616	47	Sat114CF	na	na
chr6	43504082	43504272	191	Sat2CF	purple	na

chr6	43531062	43532425	1364	SAT1_CF	blue	na
chr6	43537080	43537406	327	Sat2CF	purple	na
chr6	43537112	43537402	291	Sat2CF	purple	na
chr6	43639399	43640269	871	Sat2CF	purple	na
chr6	43639462	43640241	780	Sat2CF	purple	na
chr6	43643039	43644121	1083	SAT1_CF	blue	na
chr6	43644166	43646491	2326	SAT1_CF	blue	na
chr6	43646502	43647425	924	SAT1_CF	blue	na
chr6	43647442	43647698	257	SAT1_CF	blue	na
chr6	43647726	43649397	1672	SAT1_CF	blue	na
chr6	43649427	43650887	1461	CarSat1	red	na
chr6	43650903	43651042	140	CarSat1	red	na
chr6	43651063	43651155	93	CarSat1	red	na
chr6	43651221	43651362	142	CarSat1	red	na
chr6	43651389	43651518	130	CarSat1	red	na
chr6	43651536	43652480	945	CarSat1	red	na
chr6	43652589	43654525	1937	CarSat1	red	na
chr6	43655007	43655194	188	CarSat1	red	na
chr6	43657275	43660479	3205	CarSat1	red	na
chr6	44375564	44375625	62	Sat30CF	na	na
chr6	44549617	44550191	575	CarSat1	red	na
chr6	44576283	44576449	167	Sat50CF	na	na
chr6	44781782	44783644	1863	Sat51CF	na	na
chr6	44918820	44918959	140	Sat4CF	lred	na
chr6	45205112	45206270	1159	Sat104CF	na	na
chr6	47787190	47787356	167	Sat17CF	na	na
chr6	49188173	49188517	345	Sat17CF	na	na
chr6	51908598	51909097	500	Sat44CF	na	na
chr6	53084252	53084795	544	Sat37CF	na	na
chr6	53504119	53504214	96	Sat18CF	na	na
chr6	56003751	56003879	129	SUBTEL_sa	na	na
chr6	58506860	58507061	202	Sat51CF	na	na
chr6	58506918	58507070	153	Sat51CF	na	na
chr6	59538799	59538946	148	Carsat3	green	na
chr6	60638277	60638319	43	Sat89CF	na	na
chr6	61884793	61884843	51	Sat63CF	na	na
chr6	64169460	64169860	401	Sat141CF	na	na
chr6	64440492	64440821	330	Sat19CF	na	na
chr6	64531309	64531593	285	CarSat1	red	na
chr6	64579330	64579390	61	Sat32CF	na	na
chr6	66068736	66068817	82	Sat19CF	na	na
chr6	69024880	69024925	46	Sat7CF	lime	na

chr6	69337802	69337861	60	Sat126CF	na	na
chr6	69649512	69649558	47	Sat51CF	na	na
chr6	72368560	72369083	524	Sat125CF	na	na
chr6	72368561	72368961	401	Sat125CF	na	na
chr6	73019795	73020109	315	Sat18CF	na	na
chr6	75303509	75303554	46	Sat36CF	na	na
chr6	75616579	75616614	36	Sat41CF	na	na
chr6	79134813	79135439	627	Sat19CF	na	na
chr6	79967448	79968138	691	Sat100CF	na	na
chr6	79967549	79969458	1910	Sat100CF	na	na
chr6	79969397	79969466	70	Sat100CF	na	na
chr6	80639978	80641043	1066	SAT1_CF	blue	na
chr7	3516351	3516425	75	Sat128CF	na	pericen
chr7	3898522	3898991	470	Sat19CF	na	pericen
chr7	3946701	3947084	384	Sat114CF	na	pericen
chr7	4278502	4278589	88	SUBTEL_sa	na	pericen
chr7	5263763	5263880	118	Sat17CF	na	na
chr7	5525199	5525284	86	SAT1_CF	blue	na
chr7	5926427	5926539	113	Sat80CF	na	na
chr7	6071680	6072813	1134	Sat119CF	na	na
chr7	6071818	6072813	996	Sat79CF	na	na
chr7	7375720	7375821	102	Sat43CF	na	na
chr7	7497487	7497863	377	Sat24CF	na	na
chr7	7963635	7963992	358	CarSat1	red	na
chr7	8453308	8453411	104	Sat89CF	na	na
chr7	8822109	8822160	52	Sat55CF	na	na
chr7	9810516	9810606	91	Sat56CF	na	na
chr7	11222115	11222486	372	Sat12CF	na	na
chr7	11222328	11222487	160	Sat12CF	na	na
chr7	11868612	11868749	138	Sat12CF	na	na
chr7	14706802	14706895	94	Sat9CF	na	na
chr7	16365777	16365857	81	Sat150CF	na	na
chr7	18431083	18431183	101	Carsat3	green	na
chr7	18644166	18644217	52	Sat58CF	na	na
chr7	23943804	23944038	235	Carsat3	green	na
chr7	30594477	30594550	74	Sat165CF	na	na
chr7	36204606	36205533	928	Sat43CF	na	na
chr7	38412381	38412485	105	Carsat2	orange	na
chr7	38728498	38728811	314	Sat97CF	na	na
chr7	42281429	42281529	101	Sat60CF	na	na
chr7	44285623	44285672	50	Sat9CF	na	na
chr7	45313729	45314104	376	Sat134CF	na	na

chr7	45313772	45315071	1300	Sat134CF	na	na
chr7	45313952	45315210	1259	Sat134CF	na	na
chr7	45315131	45315232	102	Sat134CF	na	na
chr7	45949264	45949300	37	Sat36CF	na	na
chr7	46085938	46086166	229	Sat17CF	na	na
chr7	46511593	46511658	66	Sat12CF	na	na
chr7	46569291	46569637	347	Sat2CF	purple	na
chr7	46875475	46875525	51	Sat55CF	na	na
chr7	47844712	47844859	148	Sat172CF	na	na
chr7	51616045	51616232	188	Sat60CF	na	na
chr7	53875173	53875242	70	Sat67CF	na	na
chr7	57653025	57653314	290	Carsat3	green	na
chr7	57677955	57678334	380	Carsat3	green	na
chr7	57697044	57698868	1825	Carsat3	green	na
chr7	57726804	57727452	649	Carsat3	green	na
chr7	57733297	57734147	851	Carsat3	green	na
chr7	59480431	59480486	56	Sat92CF	na	na
chr7	62208443	62208506	64	Carsat3	green	na
chr7	65379680	65380004	325	CarSat1	red	na
chr7	67742511	67742566	56	Sat59CF	na	na
chr7	74724862	74725017	156	Carsat3	green	na
chr7	80568468	80568629	162	Sat19CF	na	na
chr7	81599056	81599285	230	Carsat2	orange	na
chr7	81607574	81608030	457	Carsat2	orange	na
chr7	81608331	81609817	1487	Carsat2	orange	na
chr7	81611639	81611803	165	Carsat2	orange	na
chr7	81612051	81612190	140	Carsat2	orange	na
chr7	81614757	81615126	370	Carsat2	orange	na
chr7	82835159	82835240	82	Sat63CF	na	na
chr7	83597104	83597649	546	Carsat2	orange	na
chr7	83917838	83921472	3635	Carsat2	orange	na
chr7	83921385	83921541	157	CarSat1	red	na
chr7	83921736	83922491	756	CarSat1	red	na
chr8	3015729	3015884	156	CarSat1	red	pericen
chr8	3198231	3198406	176	Sat18CF	na	pericen
chr8	3198248	3198433	186	Sat21CF	na	pericen
chr8	3198257	3198489	233	Sat21CF	na	pericen
chr8	3372569	3372900	332	Carsat2	orange	pericen
chr8	3751311	3752416	1106	Carsat2	orange	pericen
chr8	3759741	3760264	524	Carsat2	orange	pericen
chr8	3760279	3760683	405	Carsat2	orange	pericen
chr8	3760735	3763535	2801	Carsat2	orange	pericen

chr8	3763651	3764070	420	Carsat2	orange	pericen
chr8	3764200	3767497	3298	Carsat2	orange	pericen
chr8	3767535	3768094	560	Carsat2	orange	pericen
chr8	3768108	3769476	1369	Carsat2	orange	pericen
chr8	5157634	5158387	754	Carsat2	orange	na
chr8	6048853	6050262	1410	SAT1_CF	blue	na
chr8	7385210	7385484	275	Sat47CF	na	na
chr8	8270693	8270896	204	Sat17CF	na	na
chr8	14277193	14277421	229	Carsat3	green	na
chr8	18166358	18166392	35	Sat62CF	na	na
chr8	20608837	20608881	45	Sat76CF	na	na
chr8	28565308	28565385	78	Sat32CF	na	na
chr8	30738944	30739035	92	Sat36CF	na	na
chr8	33708674	33708712	39	Sat21CF	na	na
chr8	41445609	41445660	52	Sat32CF	na	na
chr8	43873579	43873718	140	Carsat3	green	na
chr8	45057128	45057463	336	Carsat3	green	na
chr8	45844753	45844828	76	Sat89CF	na	na
chr8	51127613	51127778	166	Sat13CF	na	na
chr8	56697132	56697184	53	Sat179CF	na	na
chr8	58670603	58670708	106	Sat30CF	na	na
chr8	58842480	58842779	300	Carsat3	green	na
chr8	64052703	64052868	166	Carsat3	green	na
chr8	64053060	64053172	113	Carsat3	green	na
chr8	64053212	64053414	203	Carsat3	green	na
chr8	64513666	64513722	57	Sat46CF	na	na
chr8	65418180	65419268	1089	Carsat2	orange	na
chr8	65488531	65488589	59	Sat22CF	na	na
chr8	65715941	65716054	114	Carsat3	green	na
chr8	65716076	65716359	284	Carsat3	green	na
chr8	66053147	66053748	602	Sat3CF	optpurple	na
chr8	66485324	66485556	233	Sat17CF	na	na
chr8	66814663	66814859	197	Sat72CF	na	na
chr8	69749419	69749568	150	Sat14CF	na	na
chr8	70660711	70661262	552	CarSat1	red	na
chr8	72130594	72131685	1092	Sat76CF	na	na
chr8	72130723	72131904	1182	Sat76CF	na	na
chr8	72131783	72131926	144	Sat76CF	na	na
chr8	74130113	74130165	53	Sat130CF	na	na
chr8	74848414	74849085	672	Sat42CF	na	na
chr8	74983803	74984238	436	Sat4CF	lred	na
chr8	75015775	75016067	293	CarSat1	red	na

chr8	75021816	75022226	411	Sat79CF	na	na
chr8	75189660	75190036	377	CarSat1	red	na
chr8	75321989	75322156	168	Sat5CF	stalk	na
chr8	75397369	75397592	224	Sat21CF	na	na
chr8	75468169	75470438	2270	Sat40CF	na	na
chr8	75469219	75471346	2128	Sat40CF	na	na
chr8	75529412	75529674	263	Sat74CF	na	na
chr8	75554476	75554563	88	Sat13CF	na	na
chr8	75742227	75742412	186	Sat38CF	na	na
chr8	75885469	75885568	100	Sat60CF	na	na
chr8	75887579	75887667	89	Sat5CF	stalk	na
chr8	75888170	75888192	23	Sat60CF	na	na
chr8	75888491	75888629	139	Sat60CF	na	na
chr8	75889125	75889162	38	Sat5CF	stalk	na
chr8	75903481	75904641	1161	Sat38CF	na	na
chr8	76335937	76336699	763	Carsat3	green	na
chr8	76652681	76652910	230	CarSat1	red	na
chr8	76907728	76907973	246	CarSat1	red	na
chr9	3008904	3009312	409	Sat28CF	na	pericen
chr9	3008942	3009340	399	Sat118CF	na	pericen
chr9	3009341	3010457	1117	Sat118CF	na	pericen
chr9	3010084	3010501	418	Sat21CF	na	pericen
chr9	3010429	3010520	92	Sat28CF	na	pericen
chr9	3010459	3010510	52	Sat118CF	na	pericen
chr9	3521978	3522166	189	CarSat1	red	pericen
chr9	4764509	4764661	153	Sat176CF	na	pericen
chr9	4858043	4858641	599	Sat93CF	na	pericen
chr9	4871292	4871508	217	Sat17CF	na	pericen
chr9	4964052	4964088	37	Sat102CF	na	pericen
chr9	5080890	5080911	22	Sat16CF	na	na
chr9	5081005	5081033	29	Sat16CF	na	na
chr9	5126305	5126456	152	Sat172CF	na	na
chr9	5126328	5127697	1370	Sat172CF	na	na
chr9	5337566	5337692	127	Sat87CF	na	na
chr9	6530281	6530364	84	Sat18CF	na	na
chr9	6570854	6571400	547	Sat43CF	na	na
chr9	6912175	6912275	101	Sat34CF	na	na
chr9	9174164	9174418	255	Sat13CF	na	na
chr9	9611589	9612121	533	SAT1_CF	blue	na
chr9	9713730	9713876	147	Sat182CF	na	na
chr9	9833068	9833343	276	Sat135CF	na	na
chr9	12771507	12771552	46	Sat155CF	na	na

chr9	13367589	13368052	464	Sat10CF	na	na
chr9	13367636	13368806	1171	Sat10CF	na	na
chr9	13367744	13368052	309	Sat10CF	na	na
chr9	13367805	13368855	1051	Sat10CF	na	na
chr9	13367589	13368052	464	Sat10CF	na	na
chr9	13368468	13369182	715	Sat10CF	na	na
chr9	13368468	13369182	715	Sat10CF	na	na
chr9	14343589	14343703	115	Carsat3	green	na
chr9	14343788	14344098	311	Carsat3	green	na
chr9	14344294	14344419	126	Carsat3	green	na
chr9	20064758	20064798	41	Sat56CF	na	na
chr9	21273313	21274329	1017	Sat10CF	na	na
chr9	21312852	21314096	1245	Sat10CF	na	na
chr9	21313614	21314560	947	Sat10CF	na	na
chr9	21313712	21314729	1018	Sat10CF	na	na
chr9	21314947	21315554	608	Sat10CF	na	na
chr9	21315223	21315567	345	Sat10CF	na	na
chr9	21315311	21315554	244	Sat10CF	na	na
chr9	21341782	21342140	359	Sat10CF	na	na
chr9	21341795	21342402	608	Sat10CF	na	na
chr9	21342789	21343839	1051	Sat10CF	na	na
chr9	21343452	21344166	715	Sat10CF	na	na
chr9	21849859	21849983	125	Sat12CF	na	na
chr9	22179293	22179354	62	Sat12CF	na	na
chr9	23697424	23697468	45	Sat8CF	dgreen	na
chr9	24478482	24478559	78	Sat12CF	na	na
chr9	24480183	24480424	242	Sat12CF	na	na
chr9	24480325	24480531	207	Sat12CF	na	na
chr9	24523162	24523297	136	Sat12CF	na	na
chr9	25480578	25480669	92	Sat137CF	na	na
chr9	25485115	25485408	294	Sat12CF	na	na
chr9	25698038	25698104	67	Sat118CF	na	na
chr9	26173742	26174160	419	CarSat1	red	na
chr9	27016125	27016512	388	Sat12CF	na	na
chr9	27043013	27043096	84	Sat12CF	na	na
chr9	27063853	27064062	210	Sat12CF	na	na
chr9	27063947	27064062	116	Sat12CF	na	na
chr9	28761674	28761792	119	Carsat3	green	na
chr9	28830110	28830213	104	Carsat3	green	na
chr9	28830268	28830426	159	Carsat3	green	na
chr9	28843504	28843571	68	Sat27CF	na	na
chr9	28955809	28955888	80	Sat12CF	na	na

chr9	28955819	28955888	70	Sat12CF	na	na
chr9	29272748	29272801	54	Sat133CF	na	na
chr9	29332453	29332543	91	Sat12CF	na	na
chr9	29455013	29455125	113	Sat12CF	na	na
chr9	29465725	29466115	391	Sat12CF	na	na
chr9	35284804	35284877	74	Sat113CF	na	na
chr9	35686121	35686191	71	Sat13CF	na	na
chr9	36291234	36291440	207	CarSat1	red	na
chr9	39394743	39394809	67	Sat118CF	na	na
chr9	39886024	39886307	284	Sat12CF	na	na
chr9	39886038	39886341	304	Sat12CF	na	na
chr9	39888521	39888588	68	Sat16CF	na	na
chr9	39939594	39939898	305	Sat12CF	na	na
chr9	39939646	39939878	233	Sat12CF	na	na
chr9	41917670	41917886	217	Sat12CF	na	na
chr9	41945443	41945630	188	Sat12CF	na	na
chr9	42032757	42032896	140	Sat12CF	na	na
chr9	42032789	42032897	109	Sat12CF	na	na
chr9	42070415	42070495	81	Sat165CF	na	na
chr9	45688271	45688334	64	Carsat3	green	na
chr9	46253001	46253200	200	Sat12CF	na	na
chr9	46253058	46253292	235	Sat12CF	na	na
chr9	46829223	46829278	56	Sat95CF	na	na
chr9	47959434	47959592	159	SUBTEL_sa	na	na
chr9	49684301	49684523	223	Sat17CF	na	na
chr9	50098702	50098773	72	Sat157CF	na	na
chr9	51032313	51032726	414	Sat14CF	na	na
chr9	51032314	51032658	345	Sat14CF	na	na
chr9	51107809	51108102	294	SAT1_CF	blue	na
chr9	51132191	51132618	428	Sat2CF	purple	na
chr9	51132515	51132644	130	Sat2CF	purple	na
chr9	51509989	51510050	62	Sat165CF	na	na
chr9	52594074	52594141	68	Sat16CF	na	na
chr9	52609228	52609299	72	Sat5CF	stalk	na
chr9	52760313	52761137	825	Sat50CF	na	na
chr9	52820147	52820335	189	Carsat3	green	na
chr9	52841012	52841375	364	Sat91CF	na	na
chr9	53259371	53259589	219	Sat50CF	na	na
chr9	53281941	53282521	581	Sat19CF	na	na
chr9	53527471	53527536	66	Sat28CF	na	na
chr9	54420524	54420549	26	Sat5CF	stalk	na
chr9	54826491	54826572	82	Sat38CF	na	na

chr9	54827129	54827193	65	Sat38CF	na	na
chr9	55367091	55367303	213	Sat41CF	na	na
chr9	55375039	55375277	239	Sat38CF	na	na
chr9	55474052	55474147	96	Sat100CF	na	na
chr9	56410503	56410564	62	Sat90CF	na	na
chr9	58052205	58052370	166	CarSat1	red	na
chr9	58232289	58232398	110	Sat12CF	na	na
chr9	59245317	59246246	930	Carsat3	green	na
chr9	59521529	59521579	51	Sat10CF	na	na
chr9	61438859	61438910	52	Sat32CF	na	na
chr9	62453401	62453441	41	Sat33CF	na	na
chr9	64389918	64390948	1031	Carsat2	orange	na
chr9	64390989	64392563	1575	Carsat2	orange	na
chr9	64395464	64396305	842	Carsat2	orange	na
chr9	64396344	64397007	664	Carsat2	orange	na
chrUn	43	756	714	CarSat1	red	na
chrUn	790	3716	2927	CarSat1	red	na
chrUn	3741	4693	953	CarSat1	red	na
chrUn	4720	5871	1152	CarSat1	red	na
chrUn	6066	6888	823	CarSat1	red	na
chrUn	6904	9098	2195	CarSat1	red	na
chrUn	9123	10075	953	CarSat1	red	na
chrUn	10102	12035	1934	CarSat1	red	na
chrUn	12060	12769	710	CarSat1	red	na
chrUn	12807	14252	1446	CarSat1	red	na
chrUn	14277	18442	4166	CarSat1	red	na
chrUn	18468	19540	1073	CarSat1	red	na
chrUn	19674	19790	117	CarSat1	red	na
chrUn	19999	21669	1671	CarSat1	red	na
chrUn	28871	29819	949	Sat2CF	purple	na
chrUn	30030	30171	142	Sat2CF	purple	na
chrUn	30031	30199	169	Sat2CF	purple	na
chrUn	44444	45613	1170	Sat2CF	purple	na
chrUn	45505	45682	178	Sat2CF	purple	na
chrUn	61085	62024	940	Sat2CF	purple	na
chrUn	61096	62145	1050	Sat2CF	purple	na
chrUn	96730	97875	1146	Sat2CF	purple	na
chrUn	127474	127582	109	Sat2CF	purple	na
chrUn	127485	128314	830	Sat2CF	purple	na
chrUn	127931	128376	446	Sat2CF	purple	na
chrUn	150992	152128	1137	Sat2CF	purple	na
chrUn	170510	171322	813	Sat2CF	purple	na

chrUn	170515	170677	163	Sat2CF	purple	na
chrUn	170584	171309	726	Sat2CF	purple	na
chrUn	194239	194993	755	Sat2CF	purple	na
chrUn	194329	194991	663	Sat2CF	purple	na
chrUn	210969	212134	1166	Sat2CF	purple	na
chrUn	211177	212155	979	Sat2CF	purple	na
chrUn	212026	212205	180	Sat2CF	purple	na
chrUn	212095	212161	67	Sat2CF	purple	na
chrUn	227132	228011	880	Sat2CF	purple	na
chrUn	227229	228299	1071	Sat2CF	purple	na
chrUn	258280	259446	1167	Sat2CF	purple	na
chrUn	259301	259483	183	Sat2CF	purple	na
chrUn	280959	282171	1213	Sat2CF	purple	na
chrUn	282042	282191	150	Sat2CF	purple	na
chrUn	296752	297969	1218	Sat2CF	purple	na
chrUn	311799	312595	797	Sat2CF	purple	na
chrUn	325477	326728	1252	Sat2CF	purple	na
chrUn	325607	326723	1117	Sat2CF	purple	na
chrUn	349643	350819	1177	Sat2CF	purple	na
chrUn	378305	378695	391	Sat2CF	purple	na
chrUn	378371	378721	351	Sat2CF	purple	na
chrUn	403864	404998	1135	Sat2CF	purple	na
chrUn	403998	405083	1086	Sat2CF	purple	na
chrUn	434790	434898	109	Sat2CF	purple	na
chrUn	434801	435662	862	Sat2CF	purple	na
chrUn	435535	435696	162	Sat2CF	purple	na
chrUn	464635	465855	1221	Sat2CF	purple	na
chrUn	464770	465850	1081	Sat2CF	purple	na
chrUn	465726	465875	150	Sat2CF	purple	na
chrUn	471285	472187	903	Sat2CF	purple	na
chrUn	471307	472066	760	Sat2CF	purple	na
chrUn	497848	498851	1004	Sat2CF	purple	na
chrUn	511484	511874	391	Sat2CF	purple	na
chrUn	511550	511900	351	Sat2CF	purple	na
chrUn	533272	533412	141	Sat2CF	purple	na
chrUn	533301	534099	799	Sat2CF	purple	na
chrUn	533991	534115	125	Sat2CF	purple	na
chrUn	587627	588491	865	Sat2CF	purple	na
chrUn	620432	620540	109	Sat2CF	purple	na
chrUn	620443	621304	862	Sat2CF	purple	na
chrUn	620703	621375	673	Sat2CF	purple	na
chrUn	642430	643551	1122	Sat2CF	purple	na

chrUn	643423	643571	149	Sat2CF	purple	na
chrUn	658107	659339	1233	Sat2CF	purple	na
chrUn	702822	704001	1180	Sat2CF	purple	na
chrUn	725144	725282	139	Sat2CF	purple	na
chrUn	725171	725966	796	Sat2CF	purple	na
chrUn	725858	725982	125	Sat2CF	purple	na
chrUn	748889	750054	1166	Sat2CF	purple	na
chrUn	806044	806942	899	Sat2CF	purple	na
chrUn	806107	806891	785	Sat2CF	purple	na
chrUn	824212	824532	321	Sat2CF	purple	na
chrUn	824269	824563	295	Sat2CF	purple	na
chrUn	840482	841434	953	Sat2CF	purple	na
chrUn	840617	841354	738	Sat2CF	purple	na
chrUn	860964	861701	738	Sat2CF	purple	na
chrUn	907009	907931	923	Sat2CF	purple	na
chrUn	907072	907880	809	Sat2CF	purple	na
chrUn	932148	933366	1219	Sat2CF	purple	na
chrUn	933237	933386	150	Sat2CF	purple	na
chrUn	972151	973284	1134	Sat2CF	purple	na
chrUn	972285	973400	1116	Sat2CF	purple	na
chrUn	1002817	1003923	1107	Sat2CF	purple	na
chrUn	1027944	1028806	863	Sat2CF	purple	na
chrUn	1028089	1028794	706	Sat2CF	purple	na
chrUn	1047252	1047806	555	Sat2CF	purple	na
chrUn	1070812	1071758	947	Sat2CF	purple	na
chrUn	1070947	1071685	739	Sat2CF	purple	na
chrUn	1091513	1092250	738	Sat2CF	purple	na
chrUn	1091587	1092248	662	Sat2CF	purple	na
chrUn	1130780	1131998	1219	Sat2CF	purple	na
chrUn	1130907	1131993	1087	Sat2CF	purple	na
chrUn	1161424	1161799	376	Sat2CF	purple	na
chrUn	1161465	1162304	840	Sat2CF	purple	na
chrUn	1161487	1162225	739	Sat2CF	purple	na
chrUn	1177293	1178437	1145	Sat2CF	purple	na
chrUn	1177770	1178516	747	Sat2CF	purple	na
chrUn	1193329	1194161	833	Sat2CF	purple	na
chrUn	1193413	1194139	727	Sat2CF	purple	na
chrUn	1204879	1206054	1176	Sat2CF	purple	na
chrUn	1244561	1245424	864	Sat2CF	purple	na
chrUn	1277620	1277957	338	Sat2CF	purple	na
chrUn	1277655	1278514	860	Sat2CF	purple	na
chrUn	1278116	1278593	478	Sat2CF	purple	na

chrUn	1300086	1301306	1221	Sat2CF	purple	na
chrUn	1301177	1301326	150	Sat2CF	purple	na
chrUn	1316609	1317752	1144	Sat2CF	purple	na
chrUn	1316613	1317880	1268	Sat2CF	purple	na
chrUn	1334110	1335278	1169	Sat2CF	purple	na
chrUn	1335100	1335300	201	Sat2CF	purple	na
chrUn	1335170	1335349	180	Sat2CF	purple	na
chrUn	1345563	1346393	831	Sat2CF	purple	na
chrUn	1345647	1346372	726	Sat2CF	purple	na
chrUn	1346263	1346430	168	Sat2CF	purple	na
chrUn	1357127	1358325	1199	Sat2CF	purple	na
chrUn	1368237	1369467	1231	Sat2CF	purple	na
chrUn	1385209	1386463	1255	Sat2CF	purple	na
chrUn	1385339	1386430	1092	Sat2CF	purple	na
chrUn	1414795	1415981	1187	Sat2CF	purple	na
chrUn	1445635	1446801	1167	Sat2CF	purple	na
chrUn	1468630	1469066	437	Sat2CF	purple	na
chrUn	1468682	1469765	1084	Sat2CF	purple	na
chrUn	1469641	1469790	150	Sat2CF	purple	na
chrUn	1483007	1484185	1179	Sat2CF	purple	na
chrUn	1506923	1507715	793	Sat2CF	purple	na
chrUn	1526381	1527503	1123	Sat2CF	purple	na
chrUn	1544006	1544957	952	Sat2CF	purple	na
chrUn	1544141	1544877	737	Sat2CF	purple	na
chrUn	1574944	1575808	865	Sat2CF	purple	na
chrUn	1593624	1594757	1134	Sat2CF	purple	na
chrUn	1638169	1639029	861	Sat2CF	purple	na
chrUn	1652717	1653937	1221	Sat2CF	purple	na
chrUn	1653808	1653957	150	Sat2CF	purple	na
chrUn	1692349	1693412	1064	Sat2CF	purple	na
chrUn	1707960	1709081	1122	Sat2CF	purple	na
chrUn	1708952	1709129	178	Sat2CF	purple	na
chrUn	1720944	1722187	1244	Sat2CF	purple	na
chrUn	1750362	1751438	1077	Sat2CF	purple	na
chrUn	1758268	1758618	351	Sat2CF	purple	na
chrUn	1779739	1780565	827	Sat2CF	purple	na
chrUn	1780457	1780581	125	Sat2CF	purple	na
chrUn	1821541	1822665	1125	Sat2CF	purple	na
chrUn	1867583	1868820	1238	Sat2CF	purple	na
chrUn	1882894	1883284	391	Sat2CF	purple	na
chrUn	1882960	1883317	358	Sat2CF	purple	na
chrUn	1904740	1904878	139	Sat2CF	purple	na

chrUn	1904767	1905563	797	Sat2CF	purple	na
chrUn	1904742	1904878	137	Sat2CF	purple	na
chrUn	1905455	1905579	125	Sat2CF	purple	na
chrUn	1925911	1927077	1167	Sat2CF	purple	na
chrUn	1926932	1927114	183	Sat2CF	purple	na
chrUn	1955213	1955473	261	Sat2CF	purple	na
chrUn	1955656	1956616	961	Sat2CF	purple	na
chrUn	1956492	1956641	150	Sat2CF	purple	na
chrUn	1974983	1976162	1180	Sat2CF	purple	na
chrUn	1999142	2000154	1013	Sat2CF	purple	na
chrUn	2013277	2013621	345	Sat2CF	purple	na
chrUn	2013334	2013616	283	Sat2CF	purple	na
chrUn	2013340	2013686	347	Sat2CF	purple	na
chrUn	2035091	2035229	139	Sat2CF	purple	na
chrUn	2035118	2035659	542	Sat2CF	purple	na
chrUn	2035531	2035675	145	Sat2CF	purple	na
chrUn	2064934	2066149	1216	Sat2CF	purple	na
chrUn	2066020	2066169	150	Sat2CF	purple	na
chrUn	2088082	2088427	346	Sat2CF	purple	na
chrUn	2088141	2088931	791	Sat2CF	purple	na
chrUn	2106142	2106535	394	Sat2CF	purple	na
chrUn	2106211	2107550	1340	Sat2CF	purple	na
chrUn	2106347	2107685	1339	Sat2CF	purple	na
chrUn	2106663	2107803	1141	Sat2CF	purple	na
chrUn	2107646	2107822	177	Sat2CF	purple	na
chrUn	2113197	2113252	56	Sat21CF	na	na
chrUn	2127512	2128465	954	Sat2CF	purple	na
chrUn	2127647	2128385	739	Sat2CF	purple	na
chrUn	2143436	2143584	149	Sat2CF	purple	na
chrUn	2143509	2144348	840	Sat2CF	purple	na
chrUn	2179945	2181161	1217	Sat2CF	purple	na
chrUn	2180080	2181190	1111	Sat2CF	purple	na
chrUn	2196892	2198036	1145	Sat2CF	purple	na
chrUn	2196896	2198164	1269	Sat2CF	purple	na
chrUn	2211682	2212864	1183	Sat2CF	purple	na
chrUn	2225934	2226729	796	Sat2CF	purple	na
chrUn	2225999	2226713	715	Sat2CF	purple	na
chrUn	2226603	2226770	168	Sat2CF	purple	na
chrUn	2238065	2239240	1176	Sat2CF	purple	na
chrUn	2291978	2293157	1180	Sat2CF	purple	na
chrUn	2315936	2316281	346	Sat2CF	purple	na
chrUn	2315990	2316785	796	Sat2CF	purple	na

chrUn	2334722	2335245	524	Sat2CF	purple	na
chrUn	2334783	2335703	921	Sat2CF	purple	na
chrUn	2341141	2341196	56	Sat21CF	na	na
chrUn	2355474	2356426	953	Sat2CF	purple	na
chrUn	2355609	2356346	738	Sat2CF	purple	na
chrUn	2379036	2379773	738	Sat2CF	purple	na
chrUn	2379110	2379772	663	Sat2CF	purple	na
chrUn	2419710	2420912	1203	Sat2CF	purple	na
chrUn	2419836	2420922	1087	Sat2CF	purple	na
chrUn	2420798	2420947	150	Sat2CF	purple	na
chrUn	2435688	2435796	109	Sat2CF	purple	na
chrUn	2435699	2436559	861	Sat2CF	purple	na
chrUn	2435959	2436640	682	Sat2CF	purple	na
chrUn	2465909	2467073	1165	Sat2CF	purple	na
chrUn	2466966	2467187	222	Sat2CF	purple	na
chrUn	2482014	2482337	324	Sat2CF	purple	na
chrUn	2482047	2482407	361	Sat2CF	purple	na
chrUn	2482086	2482896	811	Sat2CF	purple	na
chrUn	2482088	2482814	727	Sat2CF	purple	na
chrUn	2541039	2541837	799	Sat2CF	purple	na
chrUn	2541720	2541887	168	Sat2CF	purple	na
chrUn	2560474	2560606	133	Sat2CF	purple	na
chrUn	2560499	2561292	794	Sat2CF	purple	na
chrUn	2561184	2561308	125	Sat2CF	purple	na
chrUn	2585038	2586171	1134	Sat2CF	purple	na
chrUn	2622441	2623518	1078	Sat2CF	purple	na
chrUn	2622576	2623504	929	Sat2CF	purple	na
chrUn	2623389	2623590	202	Sat2CF	purple	na
chrUn	2625225	2625635	411	CarSat1	red	na
chrUn	2626606	2627461	856	CarSat1	red	na
chrUn	2627482	2628262	781	CarSat1	red	na
chrUn	2661902	2662656	755	SAT1_CF	blue	na
chrUn	2662699	2663658	960	SAT1_CF	blue	na
chrUn	2663776	2664724	949	SAT1_CF	blue	na
chrUn	2664737	2665238	502	SAT1_CF	blue	na
chrUn	2665250	2665924	675	SAT1_CF	blue	na
chrUn	2665963	2667776	1814	SAT1_CF	blue	na
chrUn	2667855	2668395	541	SAT1_CF	blue	na
chrUn	2668483	2668984	502	SAT1_CF	blue	na
chrUn	2668996	2670828	1833	SAT1_CF	blue	na
chrUn	2670907	2671417	511	SAT1_CF	blue	na
chrUn	2671445	2671602	158	SAT1_CF	blue	na

chrUn	2671647	2672147	501	SAT1_CF	blue	na
chrUn	2672169	2675382	3214	SAT1_CF	blue	na
chrUn	2675427	2676013	587	SAT1_CF	blue	na
chrUn	2676070	2676623	554	SAT1_CF	blue	na
chrUn	2676635	2677318	684	SAT1_CF	blue	na
chrUn	2677330	2677987	658	SAT1_CF	blue	na
chrUn	2678031	2679100	1070	SAT1_CF	blue	na
chrUn	2679123	2680376	1254	SAT1_CF	blue	na
chrUn	2680389	2681643	1255	SAT1_CF	blue	na
chrUn	2681655	2681763	109	SAT1_CF	blue	na
chrUn	2681784	2682330	547	SAT1_CF	blue	na
chrUn	2682373	2683372	1000	SAT1_CF	blue	na
chrUn	2683392	2684363	972	SAT1_CF	blue	na
chrUn	2684397	2685634	1238	SAT1_CF	blue	na
chrUn	2685667	2686171	505	SAT1_CF	blue	na
chrUn	2686203	2686907	705	SAT1_CF	blue	na
chrUn	2686925	2687028	104	SAT1_CF	blue	na
chrUn	2687051	2687722	672	SAT1_CF	blue	na
chrUn	2687745	2688291	547	SAT1_CF	blue	na
chrUn	2688304	2690091	1788	SAT1_CF	blue	na
chrUn	2690171	2690336	166	SAT1_CF	blue	na
chrUn	2690521	2690705	185	SAT1_CF	blue	na
chrUn	2690722	2691241	520	SAT1_CF	blue	na
chrUn	2691254	2693077	1824	SAT1_CF	blue	na
chrUn	2693090	2693195	106	SAT1_CF	blue	na
chrUn	2693218	2693771	554	SAT1_CF	blue	na
chrUn	2693785	2694642	858	SAT1_CF	blue	na
chrUn	2694684	2696136	1453	SAT1_CF	blue	na
chrUn	2696148	2697608	1461	SAT1_CF	blue	na
chrUn	2697639	2698145	507	SAT1_CF	blue	na
chrUn	2698178	2698335	158	SAT1_CF	blue	na
chrUn	2698380	2698881	502	SAT1_CF	blue	na
chrUn	2698893	2699570	678	SAT1_CF	blue	na
chrUn	2699613	2701240	1628	SAT1_CF	blue	na
chrUn	2701254	2702084	831	SAT1_CF	blue	na
chrUn	2702226	2702800	575	SAT1_CF	blue	na
chrUn	2702812	2704560	1749	SAT1_CF	blue	na
chrUn	2704598	2704999	402	SAT1_CF	blue	na
chrUn	2705039	2705541	503	SAT1_CF	blue	na
chrUn	2705559	2705661	103	SAT1_CF	blue	na
chrUn	2705682	2706434	753	SAT1_CF	blue	na
chrUn	2706456	2707000	545	SAT1_CF	blue	na

chrUn	2707045	2710244	3200	SAT1_CF	blue	na
chrUn	2710259	2710959	701	SAT1_CF	blue	na
chrUn	2710972	2712421	1450	SAT1_CF	blue	na
chrUn	2712482	2713578	1097	SAT1_CF	blue	na
chrUn	2713664	2714861	1198	SAT1_CF	blue	na
chrUn	2714873	2715708	836	SAT1_CF	blue	na
chrUn	2715889	2716070	182	SAT1_CF	blue	na
chrUn	2716110	2716612	503	SAT1_CF	blue	na
chrUn	2716634	2717308	675	SAT1_CF	blue	na
chrUn	2717320	2717997	678	SAT1_CF	blue	na
chrUn	2718040	2718582	543	SAT1_CF	blue	na
chrUn	2718625	2719173	549	SAT1_CF	blue	na
chrUn	2719185	2719855	671	SAT1_CF	blue	na
chrUn	2719867	2720728	862	SAT1_CF	blue	na
chrUn	2720741	2720846	106	SAT1_CF	blue	na
chrUn	2720869	2722819	1951	SAT1_CF	blue	na
chrUn	2722849	2723724	876	SAT1_CF	blue	na
chrUn	2723744	2724679	936	SAT1_CF	blue	na
chrUn	2724718	2725340	623	SAT1_CF	blue	na
chrUn	2725361	2726573	1213	SAT1_CF	blue	na
chrUn	2726617	2728610	1994	SAT1_CF	blue	na
chrUn	2728697	2729195	499	SAT1_CF	blue	na
chrUn	2729207	2731010	1804	SAT1_CF	blue	na
chrUn	2731090	2731255	166	SAT1_CF	blue	na
chrUn	2731436	2731620	185	SAT1_CF	blue	na
chrUn	2731656	2732155	500	SAT1_CF	blue	na
chrUn	2732169	2732275	107	SAT1_CF	blue	na
chrUn	2732298	2732817	520	SAT1_CF	blue	na
chrUn	2732829	2733687	859	SAT1_CF	blue	na
chrUn	2733740	2734511	772	SAT1_CF	blue	na
chrUn	2734577	2735089	513	SAT1_CF	blue	na
chrUn	2735109	2735179	71	SAT1_CF	blue	na
chrUn	2735236	2735790	555	SAT1_CF	blue	na
chrUn	2735802	2736479	678	SAT1_CF	blue	na
chrUn	2736522	2737153	632	SAT1_CF	blue	na
chrUn	2737180	2738479	1300	SAT1_CF	blue	na
chrUn	2738491	2739161	671	SAT1_CF	blue	na
chrUn	2739173	2739850	678	SAT1_CF	blue	na
chrUn	2739947	2740489	543	SAT1_CF	blue	na
chrUn	2740533	2742930	2398	SAT1_CF	blue	na
chrUn	2742964	2743315	352	SAT1_CF	blue	na
chrUn	2743404	2743897	494	SAT1_CF	blue	na

chrUn	2743926	2745088	1163	SAT1_CF	blue	na
chrUn	2745103	2745259	157	SAT1_CF	blue	na
chrUn	2745304	2745804	501	SAT1_CF	blue	na
chrUn	2745817	2747193	1377	SAT1_CF	blue	na
chrUn	2747207	2747660	454	SAT1_CF	blue	na
chrUn	2747735	2748279	545	SAT1_CF	blue	na
chrUn	2748291	2749610	1320	SAT1_CF	blue	na
chrUn	2749634	2749994	361	SAT1_CF	blue	na
chrUn	2750038	2750549	512	SAT1_CF	blue	na
chrUn	2750592	2750965	374	SAT1_CF	blue	na
chrUn	2751001	2751502	502	SAT1_CF	blue	na
chrUn	2751515	2752274	760	SAT1_CF	blue	na
chrUn	2752286	2753418	1133	SAT1_CF	blue	na
chrUn	2753430	2754107	678	SAT1_CF	blue	na
chrUn	2754150	2755150	1001	SAT1_CF	blue	na
chrUn	2755216	2755901	686	SAT1_CF	blue	na
chrUn	2755953	2756652	700	SAT1_CF	blue	na
chrUn	2756665	2756771	107	SAT1_CF	blue	na
chrUn	2756795	2757423	629	SAT1_CF	blue	na
chrUn	2757458	2758305	848	SAT1_CF	blue	na
chrUn	2758323	2759869	1547	SAT1_CF	blue	na
chrUn	2759953	2761723	1771	SAT1_CF	blue	na
chrUn	2761840	2762357	518	SAT1_CF	blue	na
chrUn	2762415	2762961	547	SAT1_CF	blue	na
chrUn	2763004	2763548	545	SAT1_CF	blue	na
chrUn	2763590	2763870	281	SAT1_CF	blue	na
chrUn	2763891	2764821	931	SAT1_CF	blue	na
chrUn	2764861	2765361	501	SAT1_CF	blue	na
chrUn	2765376	2765455	80	SAT1_CF	blue	na
chrUn	2765468	2765971	504	SAT1_CF	blue	na
chrUn	2765998	2766667	670	SAT1_CF	blue	na
chrUn	2766680	2767657	978	SAT1_CF	blue	na
chrUn	2767681	2768231	551	SAT1_CF	blue	na
chrUn	2768299	2768552	254	SAT1_CF	blue	na
chrUn	2768568	2770242	1675	SAT1_CF	blue	na
chrUn	2770287	2771274	988	SAT1_CF	blue	na
chrUn	2771331	2772288	958	SAT1_CF	blue	na
chrUn	2772349	2772828	480	SAT1_CF	blue	na
chrUn	2772855	2773603	749	SAT1_CF	blue	na
chrUn	2773615	2774475	861	SAT1_CF	blue	na
chrUn	2774552	2775388	837	SAT1_CF	blue	na
chrUn	2775415	2776886	1472	SAT1_CF	blue	na

chrUn	2776949	2777523	575	SAT1_CF	blue	na
chrUn	2777535	2778211	677	SAT1_CF	blue	na
chrUn	2778257	2779334	1078	SAT1_CF	blue	na
chrUn	2779576	2780500	925	SAT1_CF	blue	na
chrUn	2780513	2781213	701	SAT1_CF	blue	na
chrUn	2781225	2781902	678	SAT1_CF	blue	na
chrUn	2781948	2782488	541	SAT1_CF	blue	na
chrUn	2782530	2782812	283	SAT1_CF	blue	na
chrUn	2782833	2783350	518	SAT1_CF	blue	na
chrUn	2783361	2784228	868	SAT1_CF	blue	na
chrUn	2784274	2784783	510	SAT1_CF	blue	na
chrUn	2784811	2784947	137	SAT1_CF	blue	na
chrUn	2785013	2785515	503	SAT1_CF	blue	na
chrUn	2785528	2786203	676	SAT1_CF	blue	na
chrUn	2786215	2786387	173	SAT1_CF	blue	na
chrUn	2786427	2788068	1642	SAT1_CF	blue	na
chrUn	2788117	2789523	1407	SAT1_CF	blue	na
chrUn	2789570	2790277	708	SAT1_CF	blue	na
chrUn	2790295	2791052	758	SAT1_CF	blue	na
chrUn	2791064	2791892	829	SAT1_CF	blue	na
chrUn	2791908	2792376	469	SAT1_CF	blue	na
chrUn	2792420	2792966	547	SAT1_CF	blue	na
chrUn	2793009	2793519	511	SAT1_CF	blue	na
chrUn	2793546	2793679	134	SAT1_CF	blue	na
chrUn	2793737	2794238	502	SAT1_CF	blue	na
chrUn	2794251	2796276	2026	SAT1_CF	blue	na
chrUn	2796301	2796643	343	SAT1_CF	blue	na
chrUn	2796670	2797882	1213	SAT1_CF	blue	na
chrUn	2797895	2799264	1370	SAT1_CF	blue	na
chrUn	2799307	2799937	631	SAT1_CF	blue	na
chrUn	2800000	2800559	560	SAT1_CF	blue	na
chrUn	2800604	2801231	628	SAT1_CF	blue	na
chrUn	2801243	2801917	675	SAT1_CF	blue	na
chrUn	2801960	2803997	2038	SAT1_CF	blue	na
chrUn	2804037	2804531	495	SAT1_CF	blue	na
chrUn	2804551	2805233	683	SAT1_CF	blue	na
chrUn	2805256	2805350	95	SAT1_CF	blue	na
chrUn	2805373	2805919	547	SAT1_CF	blue	na
chrUn	2805962	2807700	1739	SAT1_CF	blue	na
chrUn	2807712	2809075	1364	SAT1_CF	blue	na
chrUn	2809088	2809668	581	SAT1_CF	blue	na
chrUn	2809696	2810331	636	SAT1_CF	blue	na

chrUn	2810353	2810898	546	SAT1_CF	blue	na
chrUn	2810941	2812013	1073	SAT1_CF	blue	na
chrUn	2812045	2812734	690	SAT1_CF	blue	na
chrUn	2812781	2813294	514	SAT1_CF	blue	na
chrUn	2813328	2814512	1185	SAT1_CF	blue	na
chrUn	2814552	2815740	1189	SAT1_CF	blue	na
chrUn	2815783	2817437	1655	SAT1_CF	blue	na
chrUn	2817448	2817767	320	SAT1_CF	blue	na
chrUn	2817820	2818439	620	SAT1_CF	blue	na
chrUn	2818463	2819016	554	SAT1_CF	blue	na
chrUn	2819030	2820289	1260	SAT1_CF	blue	na
chrUn	2820376	2821659	1284	SAT1_CF	blue	na
chrUn	2821678	2822223	546	SAT1_CF	blue	na
chrUn	2822266	2823378	1113	SAT1_CF	blue	na
chrUn	2823421	2824004	584	SAT1_CF	blue	na
chrUn	2824016	2825612	1597	SAT1_CF	blue	na
chrUn	2825624	2826958	1335	SAT1_CF	blue	na
chrUn	2827002	2828336	1335	SAT1_CF	blue	na
chrUn	2828404	2830705	2302	SAT1_CF	blue	na
chrUn	2830749	2831705	957	SAT1_CF	blue	na
chrUn	2831748	2832249	502	SAT1_CF	blue	na
chrUn	2832261	2832333	73	SAT1_CF	blue	na
chrUn	2832390	2832867	478	SAT1_CF	blue	na
chrUn	2832879	2832988	110	SAT1_CF	blue	na
chrUn	2833008	2834208	1201	SAT1_CF	blue	na
chrUn	2834252	2834686	435	SAT1_CF	blue	na
chrUn	2834707	2835641	935	SAT1_CF	blue	na
chrUn	2835700	2836180	481	SAT1_CF	blue	na
chrUn	2836193	2837327	1135	SAT1_CF	blue	na
chrUn	2837360	2838243	884	SAT1_CF	blue	na
chrUn	2838332	2838830	499	SAT1_CF	blue	na
chrUn	2838857	2839467	611	SAT1_CF	blue	na
chrUn	2839479	2840139	661	SAT1_CF	blue	na
chrUn	2840187	2840720	534	SAT1_CF	blue	na
chrUn	2840749	2841414	666	SAT1_CF	blue	na
chrUn	2841458	2842836	1379	SAT1_CF	blue	na
chrUn	2843025	2843526	502	SAT1_CF	blue	na
chrUn	2843544	2844300	757	SAT1_CF	blue	na
chrUn	2844312	2844987	676	SAT1_CF	blue	na
chrUn	2845030	2845838	809	SAT1_CF	blue	na
chrUn	2845861	2846882	1022	SAT1_CF	blue	na
chrUn	2846910	2847047	138	SAT1_CF	blue	na

chrUn	2847094	2847615	522	SAT1_CF	blue	na
chrUn	2847633	2847735	103	SAT1_CF	blue	na
chrUn	2847756	2848508	753	SAT1_CF	blue	na
chrUn	2848530	2849074	545	SAT1_CF	blue	na
chrUn	2849119	2851345	2227	SAT1_CF	blue	na
chrUn	2851833	2852332	500	SAT1_CF	blue	na
chrUn	2852347	2853041	695	SAT1_CF	blue	na
chrUn	2853060	2854164	1105	SAT1_CF	blue	na
chrUn	2979836	2980227	392	SAT1_CF	blue	na
chrUn	2980296	2980844	549	SAT1_CF	blue	na
chrUn	2980913	2981643	731	SAT1_CF	blue	na
chrUn	2981685	2982571	887	SAT1_CF	blue	na
chrUn	2982614	2983122	509	SAT1_CF	blue	na
chrUn	2983155	2983535	381	SAT1_CF	blue	na
chrUn	2983554	2984071	518	SAT1_CF	blue	na
chrUn	2984089	2984192	104	SAT1_CF	blue	na
chrUn	2984213	2984787	575	SAT1_CF	blue	na
chrUn	2984799	2986047	1249	SAT1_CF	blue	na
chrUn	2986089	2986348	260	SAT1_CF	blue	na
chrUn	2986375	2988541	2167	SAT1_CF	blue	na
chrUn	2988553	2989228	676	SAT1_CF	blue	na
chrUn	2989271	2990637	1367	SAT1_CF	blue	na
chrUn	2990727	2991519	793	SAT1_CF	blue	na
chrUn	2991532	2994394	2863	SAT1_CF	blue	na
chrUn	2994434	2994936	503	SAT1_CF	blue	na
chrUn	2994960	2996785	1826	SAT1_CF	blue	na
chrUn	2996817	2996952	136	SAT1_CF	blue	na
chrUn	2996999	2997520	522	SAT1_CF	blue	na
chrUn	2997547	2998102	556	SAT1_CF	blue	na
chrUn	2998121	2999766	1646	SAT1_CF	blue	na
chrUn	2999843	3001106	1264	SAT1_CF	blue	na
chrUn	3001186	3002368	1183	SAT1_CF	blue	na
chrUn	3002382	3002489	108	SAT1_CF	blue	na
chrUn	3002511	3003065	555	SAT1_CF	blue	na
chrUn	3003077	3003939	863	SAT1_CF	blue	na
chrUn	3003956	3005424	1469	SAT1_CF	blue	na
chrUn	3005504	3006084	581	SAT1_CF	blue	na
chrUn	3006097	3008816	2720	SAT1_CF	blue	na
chrUn	3008856	3009355	500	SAT1_CF	blue	na
chrUn	3009377	3010051	675	SAT1_CF	blue	na
chrUn	3010063	3010705	643	SAT1_CF	blue	na
chrUn	3010730	3012477	1748	SAT1_CF	blue	na

chrUn	3012489	3012807	319	SAT1_CF	blue	na
chrUn	3012860	3013362	503	SAT1_CF	blue	na
chrUn	3013374	3014176	803	SAT1_CF	blue	na
chrUn	3014198	3015402	1205	SAT1_CF	blue	na
chrUn	3015446	3016786	1341	SAT1_CF	blue	na
chrUn	3016809	3017327	519	SAT1_CF	blue	na
chrUn	3017355	3018024	670	SAT1_CF	blue	na
chrUn	3018036	3018718	683	SAT1_CF	blue	na
chrUn	3018729	3019221	493	SAT1_CF	blue	na
chrUn	3019249	3020436	1188	SAT1_CF	blue	na
chrUn	3020480	3021439	960	SAT1_CF	blue	na
chrUn	3021460	3021980	521	SAT1_CF	blue	na
chrUn	3021998	3022676	679	SAT1_CF	blue	na
chrUn	3022687	3023362	676	SAT1_CF	blue	na
chrUn	3023404	3024390	987	SAT1_CF	blue	na
chrUn	3024423	3025271	849	SAT1_CF	blue	na
chrUn	3025283	3026174	892	SAT1_CF	blue	na
chrUn	3026187	3026868	682	SAT1_CF	blue	na
chrUn	3026895	3027547	653	SAT1_CF	blue	na
chrUn	3027589	3028549	961	SAT1_CF	blue	na
chrUn	3028608	3029155	548	SAT1_CF	blue	na
chrUn	3029195	3029598	404	SAT1_CF	blue	na
chrUn	3029641	3030142	502	SAT1_CF	blue	na
chrUn	3030160	3030836	677	SAT1_CF	blue	na
chrUn	3030850	3031524	675	SAT1_CF	blue	na
chrUn	3031557	3032073	517	SAT1_CF	blue	na
chrUn	3032161	3032662	502	SAT1_CF	blue	na
chrUn	3032680	3033356	677	SAT1_CF	blue	na
chrUn	3033370	3034628	1259	SAT1_CF	blue	na
chrUn	3034715	3035997	1283	SAT1_CF	blue	na
chrUn	3036016	3036556	541	SAT1_CF	blue	na
chrUn	3036589	3036752	164	SAT1_CF	blue	na
chrUn	3036774	3039447	2674	SAT1_CF	blue	na
chrUn	3039628	3039809	182	SAT1_CF	blue	na
chrUn	3039869	3040349	481	SAT1_CF	blue	na
chrUn	3040362	3040467	106	SAT1_CF	blue	na
chrUn	3040490	3041711	1222	SAT1_CF	blue	na
chrUn	3041776	3041912	137	SAT1_CF	blue	na
chrUn	3041978	3042564	587	SAT1_CF	blue	na
chrUn	3042620	3043410	791	SAT1_CF	blue	na
chrUn	3043452	3044119	668	SAT1_CF	blue	na
chrUn	3044155	3044497	343	SAT1_CF	blue	na

chrUn	3044561	3046042	1482	SAT1_CF	blue	na
chrUn	3046082	3046284	203	SAT1_CF	blue	na
chrUn	3046302	3046824	523	SAT1_CF	blue	na
chrUn	3046835	3047235	401	SAT1_CF	blue	na
chrUn	3047295	3047775	481	SAT1_CF	blue	na
chrUn	3047788	3049774	1987	SAT1_CF	blue	na
chrUn	3049817	3050323	507	SAT1_CF	blue	na
chrUn	3050406	3050571	166	SAT1_CF	blue	na
chrUn	3050752	3050932	181	SAT1_CF	blue	na
chrUn	3050993	3051463	471	SAT1_CF	blue	na
chrUn	3051486	3053513	2028	SAT1_CF	blue	na
chrUn	3053525	3054469	945	SAT1_CF	blue	na
chrUn	3054503	3055024	522	SAT1_CF	blue	na
chrUn	3055036	3055142	107	SAT1_CF	blue	na
chrUn	3055166	3055791	626	SAT1_CF	blue	na
chrUn	3055826	3056674	849	SAT1_CF	blue	na
chrUn	3056692	3057376	685	SAT1_CF	blue	na
chrUn	3057388	3058061	674	SAT1_CF	blue	na
chrUn	3058079	3058238	160	SAT1_CF	blue	na
chrUn	3058251	3060143	1893	SAT1_CF	blue	na
chrUn	3060183	3060684	502	SAT1_CF	blue	na
chrUn	3060711	3062084	1374	SAT1_CF	blue	na
chrUn	3062096	3062955	860	SAT1_CF	blue	na
chrUn	3063032	3063807	776	SAT1_CF	blue	na
chrUn	3063832	3064373	542	SAT1_CF	blue	na
chrUn	3064397	3065065	669	SAT1_CF	blue	na
chrUn	3065077	3065751	675	SAT1_CF	blue	na
chrUn	3065831	3066374	544	SAT1_CF	blue	na
chrUn	3066419	3066724	306	SAT1_CF	blue	na
chrUn	3066804	3067715	912	SAT1_CF	blue	na
chrUn	3067802	3068304	503	SAT1_CF	blue	na
chrUn	3068317	3070157	1841	SAT1_CF	blue	na
chrUn	3070202	3070703	502	SAT1_CF	blue	na
chrUn	3070716	3071395	680	SAT1_CF	blue	na
chrUn	3071419	3072852	1434	SAT1_CF	blue	na
chrUn	3072917	3073478	562	SAT1_CF	blue	na
chrUn	3073520	3074071	552	SAT1_CF	blue	na
chrUn	3074084	3074759	676	SAT1_CF	blue	na
chrUn	3074802	3076625	1824	SAT1_CF	blue	na
chrUn	3076664	3077162	499	SAT1_CF	blue	na
chrUn	3077175	3077781	607	SAT1_CF	blue	na
chrUn	3077793	3078468	676	SAT1_CF	blue	na

chrUn	3078484	3079167	684	SAT1_CF	blue	na
chrUn	3079253	3079351	99	SAT1_CF	blue	na
chrUn	3079376	3079818	443	SAT1_CF	blue	na
chrUn	3079854	3080409	556	SAT1_CF	blue	na
chrUn	3080421	3081454	1034	SAT1_CF	blue	na
chrUn	3081492	3081990	499	SAT1_CF	blue	na
chrUn	3082004	3082687	684	SAT1_CF	blue	na
chrUn	3082699	3084077	1379	SAT1_CF	blue	na
chrUn	3084120	3084709	590	SAT1_CF	blue	na
chrUn	3084738	3085481	744	SAT1_CF	blue	na
chrUn	3085560	3086521	962	SAT1_CF	blue	na
chrUn	3086561	3087062	502	SAT1_CF	blue	na
chrUn	3087075	3087755	681	SAT1_CF	blue	na
chrUn	3087767	3088589	823	SAT1_CF	blue	na
chrUn	3088665	3090467	1803	SAT1_CF	blue	na
chrUn	3090522	3091024	503	SAT1_CF	blue	na
chrUn	3091037	3091141	105	SAT1_CF	blue	na
chrUn	3091165	3091966	802	SAT1_CF	blue	na
chrUn	3091988	3092083	96	SAT1_CF	blue	na
chrUn	3092107	3092909	803	SAT1_CF	blue	na
chrUn	3092922	3093037	116	SAT1_CF	blue	na
chrUn	3093050	3093853	804	SAT1_CF	blue	na
chrUn	3093866	3093974	109	SAT1_CF	blue	na
chrUn	3093995	3094744	750	SAT1_CF	blue	na
chrUn	3094766	3096757	1992	SAT1_CF	blue	na
chrUn	3096800	3097431	632	SAT1_CF	blue	na
chrUn	3097459	3098863	1405	SAT1_CF	blue	na
chrUn	3098897	3099382	486	SAT1_CF	blue	na
chrUn	3099409	3100747	1339	SAT1_CF	blue	na
chrUn	3100761	3100937	177	SAT1_CF	blue	na
chrUn	3100950	3101633	684	SAT1_CF	blue	na
chrUn	3101645	3102498	854	SAT1_CF	blue	na
chrUn	3102548	3103364	817	SAT1_CF	blue	na
chrUn	3103408	3103916	509	SAT1_CF	blue	na
chrUn	3103930	3104284	355	SAT1_CF	blue	na
chrUn	3104392	3104950	559	SAT1_CF	blue	na
chrUn	3104978	3106874	1897	SAT1_CF	blue	na
chrUn	3106918	3107480	563	SAT1_CF	blue	na
chrUn	3107523	3107717	195	SAT1_CF	blue	na
chrUn	3107745	3108241	497	SAT1_CF	blue	na
chrUn	3108911	3109485	575	SAT1_CF	blue	na
chrUn	3109528	3110645	1118	SAT1_CF	blue	na

chrUn	3110660	3111021	362	SAT1_CF	blue	na
chrUn	3111077	3111559	483	SAT1_CF	blue	na
chrUn	3111572	3112247	676	SAT1_CF	blue	na
chrUn	3112290	3113290	1001	SAT1_CF	blue	na
chrUn	3113305	3113925	621	SAT1_CF	blue	na
chrUn	3113937	3115396	1460	SAT1_CF	blue	na
chrUn	3115427	3115933	507	SAT1_CF	blue	na
chrUn	3115966	3116123	158	SAT1_CF	blue	na
chrUn	3116168	3116667	500	SAT1_CF	blue	na
chrUn	3116680	3116755	76	SAT1_CF	blue	na
chrUn	3116810	3118119	1310	SAT1_CF	blue	na
chrUn	3118162	3118794	633	SAT1_CF	blue	na
chrUn	3118822	3119436	615	SAT1_CF	blue	na
chrUn	3119465	3119989	525	SAT1_CF	blue	na
chrUn	3120000	3120160	161	SAT1_CF	blue	na
chrUn	3120205	3121401	1197	SAT1_CF	blue	na
chrUn	3121413	3122265	853	SAT1_CF	blue	na
chrUn	3122303	3122851	549	SAT1_CF	blue	na
chrUn	3122930	3123095	166	SAT1_CF	blue	na
chrUn	3123276	3123461	186	SAT1_CF	blue	na
chrUn	3123478	3123997	520	SAT1_CF	blue	na
chrUn	3124010	3124119	110	SAT1_CF	blue	na
chrUn	3124139	3124696	558	SAT1_CF	blue	na
chrUn	3124708	3127412	2705	SAT1_CF	blue	na
chrUn	3127499	3127997	499	SAT1_CF	blue	na
chrUn	3128010	3128115	106	SAT1_CF	blue	na
chrUn	3128138	3129313	1176	SAT1_CF	blue	na
chrUn	3129346	3129502	157	SAT1_CF	blue	na
chrUn	3129547	3130046	500	SAT1_CF	blue	na
chrUn	3130059	3130163	105	SAT1_CF	blue	na
chrUn	3130184	3130737	554	SAT1_CF	blue	na
chrUn	3130749	3131660	912	SAT1_CF	blue	na
chrUn	3131681	3133060	1380	SAT1_CF	blue	na
chrUn	3133105	3134307	1203	SAT1_CF	blue	na
chrUn	3134319	3135110	792	SAT1_CF	blue	na
chrUn	3135157	3135705	549	SAT1_CF	blue	na
chrUn	3135732	3136102	371	SAT1_CF	blue	na
chrUn	3136136	3136656	521	SAT1_CF	blue	na
chrUn	3136698	3138493	1796	SAT1_CF	blue	na
chrUn	3138520	3138890	371	SAT1_CF	blue	na
chrUn	3138924	3139444	521	SAT1_CF	blue	na
chrUn	3139486	3139798	313	SAT1_CF	blue	na

chrUn	3139840	3140485	646	SAT1_CF	blue	na
chrUn	3140534	3141279	746	SAT1_CF	blue	na
chrUn	3141293	3141681	389	SAT1_CF	blue	na
chrUn	3141715	3142236	522	SAT1_CF	blue	na
chrUn	3142258	3142892	635	SAT1_CF	blue	na
chrUn	3142905	3144008	1104	SAT1_CF	blue	na
chrUn	3144034	3144640	607	SAT1_CF	blue	na
chrUn	3144651	3145143	493	SAT1_CF	blue	na
chrUn	3145157	3146521	1365	SAT1_CF	blue	na
chrUn	3146586	3147148	563	SAT1_CF	blue	na
chrUn	3147191	3147743	553	SAT1_CF	blue	na
chrUn	3147754	3148365	612	SAT1_CF	blue	na
chrUn	3148388	3149054	667	SAT1_CF	blue	na
chrUn	3149097	3149726	630	SAT1_CF	blue	na
chrUn	3170038	3170669	632	SAT1_CF	blue	na
chrUn	3170712	3171675	964	SAT1_CF	blue	na
chrUn	3171711	3172752	1042	SAT1_CF	blue	na
chrUn	3172792	3173293	502	SAT1_CF	blue	na
chrUn	3173306	3174146	841	SAT1_CF	blue	na
chrUn	3174158	3175309	1152	SAT1_CF	blue	na
chrUn	3175444	3176979	1536	SAT1_CF	blue	na
chrUn	3176991	3178212	1222	SAT1_CF	blue	na
chrUn	3178282	3178990	709	SAT1_CF	blue	na
chrUn	3179002	3180092	1091	SAT1_CF	blue	na
chrUn	3180120	3180644	525	SAT1_CF	blue	na
chrUn	3180659	3180816	158	SAT1_CF	blue	na
chrUn	3180861	3181380	520	SAT1_CF	blue	na
chrUn	3181398	3182095	698	SAT1_CF	blue	na
chrUn	3182107	3183862	1756	SAT1_CF	blue	na
chrUn	3183895	3184238	344	SAT1_CF	blue	na
chrUn	3184255	3184775	521	SAT1_CF	blue	na
chrUn	3184788	3185556	769	SAT1_CF	blue	na
chrUn	3185612	3186322	711	SAT1_CF	blue	na
chrUn	3186334	3187974	1641	SAT1_CF	blue	na
chrUn	3188054	3188602	549	SAT1_CF	blue	na
chrUn	3188613	3189015	403	SAT1_CF	blue	na
chrUn	3189054	3189556	503	SAT1_CF	blue	na
chrUn	3189569	3189645	77	SAT1_CF	blue	na
chrUn	3189698	3190331	634	SAT1_CF	blue	na
chrUn	3190366	3190866	501	SAT1_CF	blue	na
chrUn	3190983	3192116	1134	SAT1_CF	blue	na
chrUn	3192128	3193312	1185	SAT1_CF	blue	na

chrUn	3193359	3193730	372	SAT1_CF	blue	na
chrUn	3193785	3194374	590	SAT1_CF	blue	na
chrUn	3194387	3196633	2247	SAT1_CF	blue	na
chrUn	3196712	3197250	539	SAT1_CF	blue	na
chrUn	3197268	3197622	355	SAT1_CF	blue	na
chrUn	3197728	3198294	567	SAT1_CF	blue	na
chrUn	3198316	3200213	1898	SAT1_CF	blue	na
chrUn	3200257	3202198	1942	SAT1_CF	blue	na
chrUn	3202234	3202634	401	SAT1_CF	blue	na
chrUn	3202655	3203174	520	SAT1_CF	blue	na
chrUn	3203187	3203295	109	SAT1_CF	blue	na
chrUn	3203316	3203861	546	SAT1_CF	blue	na
chrUn	3203904	3204868	965	SAT1_CF	blue	na
chrUn	3204947	3205495	549	SAT1_CF	blue	na
chrUn	3205539	3206229	691	SAT1_CF	blue	na
chrUn	3206241	3207543	1303	SAT1_CF	blue	na
chrUn	3207571	3208271	701	SAT1_CF	blue	na
chrUn	3208284	3208966	683	SAT1_CF	blue	na
chrUn	3208978	3209655	678	SAT1_CF	blue	na
chrUn	3209698	3210658	961	SAT1_CF	blue	na
chrUn	3210725	3211245	521	SAT1_CF	blue	na
chrUn	3211258	3211446	189	SAT1_CF	blue	na
chrUn	3211498	3212257	760	SAT1_CF	blue	na
chrUn	3212334	3212881	548	SAT1_CF	blue	na
chrUn	3212892	3213246	355	SAT1_CF	blue	na
chrUn	3213332	3213834	503	SAT1_CF	blue	na
chrUn	3213847	3214531	685	SAT1_CF	blue	na
chrUn	3214543	3215206	664	SAT1_CF	blue	na
chrUn	3215267	3216139	873	SAT1_CF	blue	na
chrUn	3216166	3216841	676	SAT1_CF	blue	na
chrUn	3216859	3218045	1187	SAT1_CF	blue	na
chrUn	3218073	3218231	159	SAT1_CF	blue	na
chrUn	3218257	3218775	519	SAT1_CF	blue	na
chrUn	3218801	3220159	1359	SAT1_CF	blue	na
chrUn	3220206	3220317	112	SAT1_CF	blue	na
chrUn	3220402	3221075	674	SAT1_CF	blue	na
chrUn	3221117	3222719	1603	SAT1_CF	blue	na
chrUn	3222753	3224183	1431	SAT1_CF	blue	na
chrUn	3224226	3224764	539	SAT1_CF	blue	na
chrUn	3224780	3225852	1073	SAT1_CF	blue	na
chrUn	3225882	3226261	380	SAT1_CF	blue	na
chrUn	3226283	3226803	521	SAT1_CF	blue	na

chrUn	3226815	3226924	110	SAT1_CF	blue	na
chrUn	3226945	3227577	633	SAT1_CF	blue	na
chrUn	3227589	3228256	668	SAT1_CF	blue	na
chrUn	3228313	3228858	546	SAT1_CF	blue	na
chrUn	3228901	3229442	542	SAT1_CF	blue	na
chrUn	3229464	3229560	97	SAT1_CF	blue	na
chrUn	3229581	3230135	555	SAT1_CF	blue	na
chrUn	3230147	3230821	675	SAT1_CF	blue	na
chrUn	3230871	3231832	962	SAT1_CF	blue	na
chrUn	3231901	3232418	518	SAT1_CF	blue	na
chrUn	3232483	3233042	560	SAT1_CF	blue	na
chrUn	3233054	3235731	2678	SAT1_CF	blue	na
chrUn	3235774	3236200	427	SAT1_CF	blue	na
chrUn	3236213	3236755	543	SAT1_CF	blue	na
chrUn	3236767	3237449	683	SAT1_CF	blue	na
chrUn	3237461	3238138	678	SAT1_CF	blue	na
chrUn	3238158	3238996	839	SAT1_CF	blue	na
chrUn	3239009	3240197	1189	SAT1_CF	blue	na
chrUn	3240209	3241368	1160	SAT1_CF	blue	na
chrUn	3241388	3241997	610	SAT1_CF	blue	na
chrUn	3242077	3242241	165	SAT1_CF	blue	na
chrUn	3242423	3242608	186	SAT1_CF	blue	na
chrUn	3242644	3243223	580	SAT1_CF	blue	na
chrUn	3243285	3244557	1273	SAT1_CF	blue	na
chrUn	3244572	3244714	143	SAT1_CF	blue	na
chrUn	3244774	3245350	577	SAT1_CF	blue	na
chrUn	3245372	3245470	99	SAT1_CF	blue	na
chrUn	3245491	3246046	556	SAT1_CF	blue	na
chrUn	3246058	3246920	863	SAT1_CF	blue	na
chrUn	3246962	3247593	632	SAT1_CF	blue	na
chrUn	3247621	3248625	1005	SAT1_CF	blue	na
chrUn	3248659	3249008	350	SAT1_CF	blue	na
chrUn	3249064	3250292	1229	SAT1_CF	blue	na
chrUn	3250304	3250987	684	SAT1_CF	blue	na
chrUn	3250998	3251490	493	SAT1_CF	blue	na
chrUn	3251504	3252219	716	SAT1_CF	blue	na
chrUn	3252262	3252799	538	SAT1_CF	blue	na
chrUn	3252867	3254202	1336	SAT1_CF	blue	na
chrUn	3254225	3254768	544	SAT1_CF	blue	na
chrUn	3254811	3256289	1479	SAT1_CF	blue	na
chrUn	3256335	3256876	542	SAT1_CF	blue	na
chrUn	3256889	3257391	503	SAT1_CF	blue	na

chrUn	3257404	3257479	76	SAT1_CF	blue	na
chrUn	3257498	3258052	555	SAT1_CF	blue	na
chrUn	3258065	3258707	643	SAT1_CF	blue	na
chrUn	3258750	3259291	542	SAT1_CF	blue	na
chrUn	3259309	3259426	118	SAT1_CF	blue	na
chrUn	3259492	3259994	503	SAT1_CF	blue	na
chrUn	3260006	3260111	106	SAT1_CF	blue	na
chrUn	3260135	3260937	803	SAT1_CF	blue	na
chrUn	3260950	3261058	109	SAT1_CF	blue	na
chrUn	3261079	3261828	750	SAT1_CF	blue	na
chrUn	3261850	3263055	1206	SAT1_CF	blue	na
chrUn	3263098	3264430	1333	SAT1_CF	blue	na
chrUn	3264547	3265088	542	SAT1_CF	blue	na
chrUn	3265100	3265213	114	SAT1_CF	blue	na
chrUn	3265229	3265776	548	SAT1_CF	blue	na
chrUn	3265819	3266353	535	SAT1_CF	blue	na
chrUn	3266367	3269163	2797	SAT1_CF	blue	na
chrUn	3270825	3271286	462	SAT1_CF	blue	na
chrUn	3271298	3273171	1874	SAT1_CF	blue	na
chrUn	3321828	3322133	306	SAT1_CF	blue	na
chrUn	3322203	3322721	519	SAT1_CF	blue	na
chrUn	3322786	3323344	559	SAT1_CF	blue	na
chrUn	3323356	3324938	1583	SAT1_CF	blue	na
chrUn	3324966	3325104	139	SAT1_CF	blue	na
chrUn	3325828	3327256	1429	SAT1_CF	blue	na
chrUn	3327269	3327376	108	SAT1_CF	blue	na
chrUn	3327398	3327773	376	SAT1_CF	blue	na
chrUn	3328063	3330479	2417	SAT1_CF	blue	na
chrUn	3331891	3332466	576	SAT1_CF	blue	na
chrUn	3332509	3333633	1125	SAT1_CF	blue	na
chrUn	3333648	3334047	400	SAT1_CF	blue	na
chrUn	3334066	3334581	516	SAT1_CF	blue	na
chrUn	3334594	3334699	106	SAT1_CF	blue	na
chrUn	3334722	3335274	553	SAT1_CF	blue	na
chrUn	3335355	3335879	525	SAT1_CF	blue	na
chrUn	3335894	3336029	136	SAT1_CF	blue	na
chrUn	3336076	3336595	520	SAT1_CF	blue	na
chrUn	3336608	3337280	673	SAT1_CF	blue	na
chrUn	3337323	3339313	1991	SAT1_CF	blue	na
chrUn	3339432	3339952	521	SAT1_CF	blue	na
chrUn	3340009	3340740	732	SAT1_CF	blue	na
chrUn	3340810	3342189	1380	SAT1_CF	blue	na

chrUn	3342215	3343437	1223	SAT1_CF	blue	na
chrUn	3343449	3344806	1358	SAT1_CF	blue	na
chrUn	3344819	3345171	353	SAT1_CF	blue	na
chrUn	3345287	3345806	520	SAT1_CF	blue	na
chrUn	3345863	3346412	550	SAT1_CF	blue	na
chrUn	3346454	3346998	545	SAT1_CF	blue	na
chrUn	3347040	3347322	283	SAT1_CF	blue	na
chrUn	3347353	3347859	507	SAT1_CF	blue	na
chrUn	3347873	3348275	403	SAT1_CF	blue	na
chrUn	3348315	3348816	502	SAT1_CF	blue	na
chrUn	3348843	3349587	745	SAT1_CF	blue	na
chrUn	3349601	3350213	613	SAT1_CF	blue	na
chrUn	3350225	3350910	686	SAT1_CF	blue	na
chrUn	3350922	3351593	672	SAT1_CF	blue	na
chrUn	3351642	3352142	501	SAT1_CF	blue	na
chrUn	3352153	3352246	94	SAT1_CF	blue	na
chrUn	3352276	3353527	1252	SAT1_CF	blue	na
chrUn	3353538	3353613	76	SAT1_CF	blue	na
chrUn	3353669	3354142	474	SAT1_CF	blue	na
chrUn	3354161	3354257	97	SAT1_CF	blue	na
chrUn	3354281	3354833	553	SAT1_CF	blue	na
chrUn	3354845	3355710	866	SAT1_CF	blue	na
chrUn	3355752	3357593	1842	SAT1_CF	blue	na
chrUn	3357604	3358128	525	SAT1_CF	blue	na
chrUn	3358172	3359283	1112	SAT1_CF	blue	na
chrUn	3359445	3360030	586	SAT1_CF	blue	na
chrUn	3360047	3361271	1225	SAT1_CF	blue	na
chrUn	3361283	3362742	1460	SAT1_CF	blue	na
chrUn	3362780	3363327	548	SAT1_CF	blue	na
chrUn	3363342	3363684	343	SAT1_CF	blue	na
chrUn	3363741	3364242	502	SAT1_CF	blue	na
chrUn	3364256	3364937	682	SAT1_CF	blue	na
chrUn	3364950	3366110	1161	SAT1_CF	blue	na
chrUn	3366130	3366740	611	SAT1_CF	blue	na
chrUn	3366819	3366984	166	SAT1_CF	blue	na
chrUn	3367165	3367350	186	SAT1_CF	blue	na
chrUn	3367367	3367887	521	SAT1_CF	blue	na
chrUn	3367900	3368006	107	SAT1_CF	blue	na
chrUn	3368028	3368585	558	SAT1_CF	blue	na
chrUn	3368597	3370280	1684	SAT1_CF	blue	na
chrUn	3370394	3371307	914	SAT1_CF	blue	na
chrUn	3371393	3371892	500	SAT1_CF	blue	na

chrUn	3371905	3372010	106	SAT1_CF	blue	na
chrUn	3372033	3373208	1176	SAT1_CF	blue	na
chrUn	3373241	3373397	157	SAT1_CF	blue	na
chrUn	3373442	3373942	501	SAT1_CF	blue	na
chrUn	3373955	3374753	799	SAT1_CF	blue	na
chrUn	3374779	3375324	546	SAT1_CF	blue	na
chrUn	3375404	3375928	525	SAT1_CF	blue	na
chrUn	3375943	3376762	820	SAT1_CF	blue	na
chrUn	3376787	3378801	2015	SAT1_CF	blue	na
chrUn	3378844	3379649	806	SAT1_CF	blue	na
chrUn	3379680	3380841	1162	SAT1_CF	blue	na
chrUn	3380884	3381382	499	SAT1_CF	blue	na
chrUn	3381395	3382075	681	SAT1_CF	blue	na
chrUn	3382089	3382847	759	SAT1_CF	blue	na
chrUn	3382861	3384193	1333	SAT1_CF	blue	na
chrUn	3384218	3384576	359	SAT1_CF	blue	na
chrUn	3384620	3385167	548	SAT1_CF	blue	na
chrUn	3385181	3385842	662	SAT1_CF	blue	na
chrUn	3385885	3386512	628	SAT1_CF	blue	na
chrUn	3386540	3387745	1206	SAT1_CF	blue	na
chrUn	3387809	3387915	107	SAT1_CF	blue	na
chrUn	3387942	3388460	519	SAT1_CF	blue	na
chrUn	3388473	3388580	108	SAT1_CF	blue	na
chrUn	3388602	3389156	555	SAT1_CF	blue	na
chrUn	3389168	3389849	682	SAT1_CF	blue	na
chrUn	3389863	3390723	861	SAT1_CF	blue	na
chrUn	3390765	3391309	545	SAT1_CF	blue	na
chrUn	3391347	3392622	1276	SAT1_CF	blue	na
chrUn	3392662	3393162	501	SAT1_CF	blue	na
chrUn	3393175	3393858	684	SAT1_CF	blue	na
chrUn	3393870	3394548	679	SAT1_CF	blue	na
chrUn	3394593	3395554	962	SAT1_CF	blue	na
chrUn	3395633	3396555	923	SAT1_CF	blue	na
chrUn	3396595	3397097	503	SAT1_CF	blue	na
chrUn	3397109	3398018	910	SAT1_CF	blue	na
chrUn	3398046	3398591	546	SAT1_CF	blue	na
chrUn	3398670	3399192	523	SAT1_CF	blue	na
chrUn	3399207	3399908	702	SAT1_CF	blue	na
chrUn	3399920	3400036	117	SAT1_CF	blue	na
chrUn	3400050	3400760	711	SAT1_CF	blue	na
chrUn	3400772	3401633	862	SAT1_CF	blue	na
chrUn	3401675	3402711	1037	SAT1_CF	blue	na

chrUn	3402793	3403301	509	SAT1_CF	blue	na
chrUn	3403329	3403570	242	SAT1_CF	blue	na
chrUn	3403604	3404969	1366	SAT1_CF	blue	na
chrUn	3404982	3405058	77	SAT1_CF	blue	na
chrUn	3405111	3405639	529	SAT1_CF	blue	na
chrUn	3405651	3406950	1300	SAT1_CF	blue	na
chrUn	3407039	3407772	734	SAT1_CF	blue	na
chrUn	3407788	3408190	403	SAT1_CF	blue	na
chrUn	3408230	3408731	502	SAT1_CF	blue	na
chrUn	3408744	3409967	1224	SAT1_CF	blue	na
chrUn	3410033	3410761	729	SAT1_CF	blue	na
chrUn	3410789	3410925	137	SAT1_CF	blue	na
chrUn	3410991	3411492	502	SAT1_CF	blue	na
chrUn	3411519	3412352	834	SAT1_CF	blue	na
chrUn	3412373	3413610	1238	SAT1_CF	blue	na
chrUn	3413653	3414947	1295	SAT1_CF	blue	na
chrUn	3414965	3415722	758	SAT1_CF	blue	na
chrUn	3415733	3416356	624	SAT1_CF	blue	na
chrUn	3416399	3417920	1522	SAT1_CF	blue	na
chrUn	3418102	3418327	226	SAT1_CF	blue	na
chrUn	3418349	3419015	667	SAT1_CF	blue	na
chrUn	3419058	3420059	1002	SAT1_CF	blue	na
chrUn	3420091	3420612	522	SAT1_CF	blue	na
chrUn	3420625	3421027	403	SAT1_CF	blue	na
chrUn	3421067	3421569	503	SAT1_CF	blue	na
chrUn	3421582	3422429	848	SAT1_CF	blue	na
chrUn	3422450	3422571	122	SAT1_CF	blue	na
chrUn	3422812	3424029	1218	SAT1_CF	blue	na
chrUn	3424045	3424443	399	SAT1_CF	blue	na
chrUn	3424464	3424984	521	SAT1_CF	blue	na
chrUn	3424996	3425672	677	SAT1_CF	blue	na
chrUn	3425715	3427308	1594	SAT1_CF	blue	na
chrUn	3427320	3428200	881	SAT1_CF	blue	na
chrUn	3429360	3429849	490	SAT1_CF	blue	na
chrUn	3429894	3430396	503	SAT1_CF	blue	na
chrUn	3430416	3430517	102	SAT1_CF	blue	na
chrUn	3430538	3431090	553	SAT1_CF	blue	na
chrUn	3431133	3432475	1343	SAT1_CF	blue	na
chrUn	3432492	3433137	646	SAT1_CF	blue	na
chrUn	3433318	3434494	1177	SAT1_CF	blue	na
chrUn	3434536	3435912	1377	SAT1_CF	blue	na
chrUn	3435955	3436452	498	SAT1_CF	blue	na

chrUn	3436470	3436572	103	SAT1_CF	blue	na
chrUn	3436593	3437148	556	SAT1_CF	blue	na
chrUn	3437161	3438434	1274	SAT1_CF	blue	na
chrUn	3438519	3439021	503	SAT1_CF	blue	na
chrUn	3439034	3439140	107	SAT1_CF	blue	na
chrUn	3439162	3439707	546	SAT1_CF	blue	na
chrUn	3439777	3440240	464	SAT1_CF	blue	na
chrUn	3440252	3441759	1508	SAT1_CF	blue	na
chrUn	3441796	3442948	1153	SAT1_CF	blue	na
chrUn	3443027	3443570	544	SAT1_CF	blue	na
chrUn	3443613	3443986	374	SAT1_CF	blue	na
chrUn	3444003	3444523	521	SAT1_CF	blue	na
chrUn	3444541	3447008	2468	SAT1_CF	blue	na
chrUn	3447046	3447545	500	SAT1_CF	blue	na
chrUn	3447558	3447665	108	SAT1_CF	blue	na
chrUn	3447686	3448241	556	SAT1_CF	blue	na
chrUn	3448253	3450395	2143	SAT1_CF	blue	na
chrUn	3450406	3451696	1291	SAT1_CF	blue	na
chrUn	3451739	3452465	727	SAT1_CF	blue	na
chrUn	3452510	3453045	536	SAT1_CF	blue	na
chrUn	3453072	3453584	513	SAT1_CF	blue	na
chrUn	3453596	3454909	1314	SAT1_CF	blue	na
chrUn	3454922	3455757	836	SAT1_CF	blue	na
chrUn	3455779	3456473	695	SAT1_CF	blue	na
chrUn	3456485	3458973	2489	SAT1_CF	blue	na
chrUn	3459061	3459562	502	SAT1_CF	blue	na
chrUn	3459575	3460257	683	SAT1_CF	blue	na
chrUn	3460270	3460341	72	SAT1_CF	blue	na
chrUn	3460364	3460913	550	SAT1_CF	blue	na
chrUn	3460931	3462151	1221	SAT1_CF	blue	na
chrUn	3462180	3462336	157	SAT1_CF	blue	na
chrUn	3462381	3462883	503	SAT1_CF	blue	na
chrUn	3462896	3464035	1140	SAT1_CF	blue	na
chrUn	3464077	3464858	782	SAT1_CF	blue	na
chrUn	3464902	3465821	920	SAT1_CF	blue	na
chrUn	3465880	3466360	481	SAT1_CF	blue	na
chrUn	3466373	3466481	109	SAT1_CF	blue	na
chrUn	3466502	3467056	555	SAT1_CF	blue	na
chrUn	3467068	3467745	678	SAT1_CF	blue	na
chrUn	3467842	3468384	543	SAT1_CF	blue	na
chrUn	3468409	3468753	345	SAT1_CF	blue	na
chrUn	3468848	3470175	1328	SAT1_CF	blue	na

chrUn	3470187	3470872	686	SAT1_CF	blue	na
chrUn	3470884	3472685	1802	SAT1_CF	blue	na
chrUn	3472706	3473592	887	SAT1_CF	blue	na
chrUn	3473679	3474171	493	SAT1_CF	blue	na
chrUn	3474191	3474298	108	SAT1_CF	blue	na
chrUn	3474319	3474874	556	SAT1_CF	blue	na
chrUn	3474886	3476370	1485	SAT1_CF	blue	na
chrUn	3476382	3477057	676	SAT1_CF	blue	na
chrUn	3477105	3477733	629	SAT1_CF	blue	na
chrUn	3477787	3478322	536	SAT1_CF	blue	na
chrUn	3478404	3478928	525	SAT1_CF	blue	na
chrUn	3478943	3479079	137	SAT1_CF	blue	na
chrUn	3479145	3480265	1121	SAT1_CF	blue	na
chrUn	3480288	3480954	667	SAT1_CF	blue	na
chrUn	3480998	3481523	526	SAT1_CF	blue	na
chrUn	3481544	3482411	868	SAT1_CF	blue	na
chrUn	3482423	3483076	654	SAT1_CF	blue	na
chrUn	3483156	3483377	222	SAT1_CF	blue	na
chrUn	3483391	3483934	544	SAT1_CF	blue	na
chrUn	3483945	3484263	319	SAT1_CF	blue	na
chrUn	3484316	3484818	503	SAT1_CF	blue	na
chrUn	3484831	3485512	682	SAT1_CF	blue	na
chrUn	3485526	3486200	675	SAT1_CF	blue	na
chrUn	3486280	3486787	508	SAT1_CF	blue	na
chrUn	3486868	3486976	109	SAT1_CF	blue	na
chrUn	3487021	3488240	1220	SAT1_CF	blue	na
chrUn	3488273	3488778	506	SAT1_CF	blue	na
chrUn	3488810	3489513	704	SAT1_CF	blue	na
chrUn	3489526	3491593	2068	SAT1_CF	blue	na
chrUn	3491606	3491714	109	SAT1_CF	blue	na
chrUn	3491735	3492289	555	SAT1_CF	blue	na
chrUn	3492301	3492978	678	SAT1_CF	blue	na
chrUn	3493075	3493616	542	SAT1_CF	blue	na
chrUn	3493641	3493966	326	SAT1_CF	blue	na
chrUn	3493992	3494518	527	SAT1_CF	blue	na
chrUn	3494566	3495369	804	SAT1_CF	blue	na
chrUn	3495401	3496457	1057	SAT1_CF	blue	na
chrUn	3496534	3497079	546	SAT1_CF	blue	na
chrUn	3497092	3497198	107	SAT1_CF	blue	na
chrUn	3497221	3497793	573	SAT1_CF	blue	na
chrUn	3497805	3498567	763	SAT1_CF	blue	na
chrUn	3498580	3499226	647	SAT1_CF	blue	na

chrUn	3499238	3500096	859	SAT1_CF	blue	na
chrUn	3500138	3500680	543	SAT1_CF	blue	na
chrUn	3500727	3501989	1263	SAT1_CF	blue	na
chrUn	3502052	3502532	481	SAT1_CF	blue	na
chrUn	3502580	3503730	1151	SAT1_CF	blue	na
chrUn	3503773	3504858	1086	SAT1_CF	blue	na
chrUn	3504871	3505272	402	SAT1_CF	blue	na
chrUn	3505310	3505812	503	SAT1_CF	blue	na
chrUn	3505825	3506506	682	SAT1_CF	blue	na
chrUn	3506518	3509213	2696	SAT1_CF	blue	na
chrUn	3509257	3509800	544	SAT1_CF	blue	na
chrUn	3509813	3510188	376	SAT1_CF	blue	na
chrUn	3510254	3510756	503	SAT1_CF	blue	na
chrUn	3510769	3511452	684	SAT1_CF	blue	na
chrUn	3511464	3512324	861	SAT1_CF	blue	na
chrUn	3512366	3514031	1666	SAT1_CF	blue	na
chrUn	3514059	3514194	136	SAT1_CF	blue	na
chrUn	3514260	3514762	503	SAT1_CF	blue	na
chrUn	3514774	3514880	107	SAT1_CF	blue	na
chrUn	3514904	3515532	629	SAT1_CF	blue	na
chrUn	3515556	3516414	859	SAT1_CF	blue	na
chrUn	3516428	3517118	691	SAT1_CF	blue	na
chrUn	3517130	3519153	2024	SAT1_CF	blue	na
chrUn	3519184	3519708	525	SAT1_CF	blue	na
chrUn	3519723	3519963	241	SAT1_CF	blue	na
chrUn	3519981	3520473	493	SAT1_CF	blue	na
chrUn	3520530	3521701	1172	SAT1_CF	blue	na
chrUn	3521818	3522325	508	SAT1_CF	blue	na
chrUn	3522338	3522839	502	SAT1_CF	blue	na
chrUn	3522852	3523532	681	SAT1_CF	blue	na
chrUn	3523544	3526238	2695	SAT1_CF	blue	na
chrUn	3526282	3526826	545	SAT1_CF	blue	na
chrUn	3526839	3527194	356	SAT1_CF	blue	na
chrUn	3527281	3527783	503	SAT1_CF	blue	na
chrUn	3527796	3528467	672	SAT1_CF	blue	na
chrUn	3528514	3529185	672	SAT1_CF	blue	na
chrUn	3529216	3530073	858	SAT1_CF	blue	na
chrUn	3530085	3530942	858	SAT1_CF	blue	na
chrUn	3530995	3531528	534	SAT1_CF	blue	na
chrUn	3531562	3533430	1869	SAT1_CF	blue	na
chrUn	3533487	3534034	548	SAT1_CF	blue	na
chrUn	3534076	3534620	545	SAT1_CF	blue	na

chrUn	3534662	3534943	282	SAT1_CF	blue	na
chrUn	3534960	3536435	1476	SAT1_CF	blue	na
chrUn	3536462	3538271	1810	SAT1_CF	blue	na
chrUn	3538284	3539044	761	SAT1_CF	blue	na
chrUn	3539068	3539739	672	SAT1_CF	blue	na
chrUn	3539751	3540678	928	SAT1_CF	blue	na
chrUn	3540691	3541851	1161	SAT1_CF	blue	na
chrUn	3541893	3543570	1678	SAT1_CF	blue	na
chrUn	3543585	3543742	158	SAT1_CF	blue	na
chrUn	3543787	3544869	1083	SAT1_CF	blue	na
chrUn	3544880	3546618	1739	SAT1_CF	blue	na
chrUn	3546662	3547187	526	SAT1_CF	blue	na
chrUn	3547231	3547774	544	SAT1_CF	blue	na
chrUn	3547801	3547936	136	SAT1_CF	blue	na
chrUn	3547964	3549171	1208	SAT1_CF	blue	na
chrUn	3549185	3549887	703	SAT1_CF	blue	na
chrUn	3549900	3550580	681	SAT1_CF	blue	na
chrUn	3550592	3551351	760	SAT1_CF	blue	na
chrUn	3551365	3552038	674	SAT1_CF	blue	na
chrUn	3552081	3553080	1000	SAT1_CF	blue	na
chrUn	3553124	3553750	627	SAT1_CF	blue	na
chrUn	3553812	3555181	1370	SAT1_CF	blue	na
chrUn	3555192	3556124	933	SAT1_CF	blue	na
chrUn	3556164	3556665	502	SAT1_CF	blue	na
chrUn	3556678	3557438	761	SAT1_CF	blue	na
chrUn	3557459	3557524	66	SAT1_CF	blue	na
chrUn	3557545	3558064	520	SAT1_CF	blue	na
chrUn	3558076	3558933	858	SAT1_CF	blue	na
chrUn	3558975	3559519	545	SAT1_CF	blue	na
chrUn	3559557	3560671	1115	SAT1_CF	blue	na
chrUn	3560853	3561034	182	SAT1_CF	blue	na
chrUn	3561055	3561573	519	SAT1_CF	blue	na
chrUn	3561586	3561691	106	SAT1_CF	blue	na
chrUn	3561714	3563415	1702	SAT1_CF	blue	na
chrUn	3563427	3563555	129	SAT1_CF	blue	na
chrUn	3563613	3564171	559	SAT1_CF	blue	na
chrUn	3564183	3565899	1717	SAT1_CF	blue	na
chrUn	3565925	3566561	637	SAT1_CF	blue	na
chrUn	3566574	3566929	356	SAT1_CF	blue	na
chrUn	3567046	3567564	519	SAT1_CF	blue	na
chrUn	3567595	3568668	1074	SAT1_CF	blue	na
chrUn	3568682	3569609	928	SAT1_CF	blue	na

chrUn	3569658	3570159	502	SAT1_CF	blue	na
chrUn	3570173	3570931	759	SAT1_CF	blue	na
chrUn	3570943	3571052	110	SAT1_CF	blue	na
chrUn	3571073	3571617	545	SAT1_CF	blue	na
chrUn	3571714	3572254	541	SAT1_CF	blue	na
chrUn	3572297	3573955	1659	SAT1_CF	blue	na
chrUn	3573988	3574144	157	SAT1_CF	blue	na
chrUn	3574189	3574689	501	SAT1_CF	blue	na
chrUn	3574702	3575561	860	SAT1_CF	blue	na
chrUn	3575638	3576182	545	SAT1_CF	blue	na
chrUn	3576224	3576505	282	SAT1_CF	blue	na
chrUn	3576523	3577411	889	SAT1_CF	blue	na
chrUn	3577528	3578066	539	SAT1_CF	blue	na
chrUn	3578086	3578193	108	SAT1_CF	blue	na
chrUn	3578214	3578769	556	SAT1_CF	blue	na
chrUn	3578781	3579644	864	SAT1_CF	blue	na
chrUn	3579721	3580266	546	SAT1_CF	blue	na
chrUn	3580388	3580882	495	SAT1_CF	blue	na
chrUn	3580939	3581485	547	SAT1_CF	blue	na
chrUn	3581527	3582624	1098	SAT1_CF	blue	na
chrUn	3582652	3582788	137	SAT1_CF	blue	na
chrUn	3582854	3583356	503	SAT1_CF	blue	na
chrUn	3583369	3584131	763	SAT1_CF	blue	na
chrUn	3584143	3584931	789	SAT1_CF	blue	na
chrUn	3584959	3585523	565	SAT1_CF	blue	na
chrUn	3585566	3586113	548	SAT1_CF	blue	na
chrUn	3586178	3586335	158	SAT1_CF	blue	na
chrUn	3586380	3589533	3154	SAT1_CF	blue	na
chrUn	3589615	3590139	525	SAT1_CF	blue	na
chrUn	3590154	3590290	137	SAT1_CF	blue	na
chrUn	3590356	3590857	502	SAT1_CF	blue	na
chrUn	3590870	3591543	674	SAT1_CF	blue	na
chrUn	3591586	3592217	632	SAT1_CF	blue	na
chrUn	3592245	3592804	560	SAT1_CF	blue	na
chrUn	3592888	3593435	548	SAT1_CF	blue	na
chrUn	3593463	3593599	137	SAT1_CF	blue	na
chrUn	3593665	3594861	1197	SAT1_CF	blue	na
chrUn	3594873	3596791	1919	SAT1_CF	blue	na
chrUn	3596848	3597480	633	SAT1_CF	blue	na
chrUn	3597492	3598095	604	SAT1_CF	blue	na
chrUn	3598108	3598213	106	SAT1_CF	blue	na
chrUn	3598234	3598786	553	SAT1_CF	blue	na

chrUn	3598798	3599663	866	SAT1_CF	blue	na
chrUn	3599705	3601863	2159	SAT1_CF	blue	na
chrUn	3601875	3602739	865	SAT1_CF	blue	na
chrUn	3602921	3603102	182	SAT1_CF	blue	na
chrUn	3603123	3603642	520	SAT1_CF	blue	na
chrUn	3603655	3603762	108	SAT1_CF	blue	na
chrUn	3603783	3605054	1272	SAT1_CF	blue	na
chrUn	3605070	3605206	137	SAT1_CF	blue	na
chrUn	3605272	3605775	504	SAT1_CF	blue	na
chrUn	3605788	3605894	107	SAT1_CF	blue	na
chrUn	3605916	3606395	480	SAT1_CF	blue	na
chrUn	3606407	3608748	2342	SAT1_CF	blue	na
chrUn	3608761	3609115	355	SAT1_CF	blue	na
chrUn	3609231	3609750	520	SAT1_CF	blue	na
chrUn	3609781	3610307	527	SAT1_CF	blue	na
chrUn	3610351	3611040	690	SAT1_CF	blue	na
chrUn	3611055	3611542	488	SAT1_CF	blue	na
chrUn	3611642	3612188	547	SAT1_CF	blue	na
chrUn	3612201	3612277	77	SAT1_CF	blue	na
chrUn	3612330	3614501	2172	SAT1_CF	blue	na
chrUn	3614532	3615039	508	SAT1_CF	blue	na
chrUn	3615052	3615406	355	SAT1_CF	blue	na
chrUn	3615493	3615994	502	SAT1_CF	blue	na
chrUn	3616007	3616768	762	SAT1_CF	blue	na
chrUn	3616781	3616852	72	SAT1_CF	blue	na
chrUn	3616875	3617425	551	SAT1_CF	blue	na
chrUn	3617443	3618111	669	SAT1_CF	blue	na
chrUn	3618154	3621108	2955	SAT1_CF	blue	na
chrUn	3621158	3623419	2262	SAT1_CF	blue	na
chrUn	3623466	3624487	1022	SAT1_CF	blue	na
chrUn	3624508	3625029	522	SAT1_CF	blue	na
chrUn	3625042	3625744	703	SAT1_CF	blue	na
chrUn	3625756	3626433	678	SAT1_CF	blue	na
chrUn	3626476	3627020	545	SAT1_CF	blue	na
chrUn	3627061	3628068	1008	SAT1_CF	blue	na
chrUn	3628113	3628615	503	SAT1_CF	blue	na
chrUn	3628628	3628733	106	SAT1_CF	blue	na
chrUn	3628755	3628900	146	SAT1_CF	blue	na
chrUn	3628996	3629561	566	SAT1_CF	blue	na
chrUn	3629573	3630977	1405	SAT1_CF	blue	na
chrUn	3630988	3631341	354	SAT1_CF	blue	na
chrUn	3631428	3631928	501	SAT1_CF	blue	na

chrUn	3631941	3633895	1955	SAT1_CF	blue	na
chrUn	3633939	3634731	793	SAT1_CF	blue	na
chrUn	3634912	3635092	181	SAT1_CF	blue	na
chrUn	3635153	3635623	471	SAT1_CF	blue	na
chrUn	3635646	3636970	1325	SAT1_CF	blue	na
chrUn	3636985	3637116	132	SAT1_CF	blue	na
chrUn	3637168	3639088	1921	SAT1_CF	blue	na
chrUn	3639116	3639680	565	SAT1_CF	blue	na
chrUn	3639723	3640272	550	SAT1_CF	blue	na
chrUn	3640315	3641093	779	SAT1_CF	blue	na
chrUn	3641171	3641679	509	SAT1_CF	blue	na
chrUn	3641693	3642343	651	SAT1_CF	blue	na
chrUn	3642365	3643116	752	SAT1_CF	blue	na
chrUn	3643129	3643745	617	SAT1_CF	blue	na
chrUn	3643958	3644113	156	SAT1_CF	blue	na
chrUn	3644178	3644776	599	SAT1_CF	blue	na
chrUn	3644799	3646000	1202	SAT1_CF	blue	na
chrUn	3646045	3647421	1377	SAT1_CF	blue	na
chrUn	3647461	3647955	495	SAT1_CF	blue	na
chrUn	3647989	3649362	1374	SAT1_CF	blue	na
chrUn	3649374	3650232	859	SAT1_CF	blue	na
chrUn	3650275	3651056	782	SAT1_CF	blue	na
chrUn	3651135	3652083	949	SAT1_CF	blue	na
chrUn	3652136	3652635	500	SAT1_CF	blue	na
chrUn	3652654	3652757	104	SAT1_CF	blue	na
chrUn	3652778	3653321	544	SAT1_CF	blue	na
chrUn	3653364	3653907	544	SAT1_CF	blue	na
chrUn	3653935	3654092	158	SAT1_CF	blue	na
chrUn	3654137	3654631	495	SAT1_CF	blue	na
chrUn	3654660	3655207	548	SAT1_CF	blue	na
chrUn	3655250	3655798	549	SAT1_CF	blue	na
chrUn	3655875	3657506	1632	SAT1_CF	blue	na
chrUn	3657534	3657670	137	SAT1_CF	blue	na
chrUn	3657736	3659732	1997	SAT1_CF	blue	na
chrUn	3659759	3660283	525	SAT1_CF	blue	na
chrUn	3660328	3661605	1278	SAT1_CF	blue	na
chrUn	3661686	3661911	226	SAT1_CF	blue	na
chrUn	3661922	3662700	779	SAT1_CF	blue	na
chrUn	3662731	3663259	529	SAT1_CF	blue	na
chrUn	3663272	3663378	107	SAT1_CF	blue	na
chrUn	3663401	3664053	653	SAT1_CF	blue	na
chrUn	3664066	3664141	76	SAT1_CF	blue	na

chrUn	3664160	3664712	553	SAT1_CF	blue	na
chrUn	3664724	3666054	1331	SAT1_CF	blue	na
chrUn	3666098	3667480	1383	SAT1_CF	blue	na
chrUn	3667520	3668020	501	SAT1_CF	blue	na
chrUn	3668163	3668717	555	SAT1_CF	blue	na
chrUn	3668729	3669403	675	SAT1_CF	blue	na
chrUn	3669450	3670537	1088	SAT1_CF	blue	na
chrUn	3670617	3672052	1436	SAT1_CF	blue	na
chrUn	3672094	3672259	166	SAT1_CF	blue	na
chrUn	3672442	3672627	186	SAT1_CF	blue	na
chrUn	3672644	3673157	514	SAT1_CF	blue	na
chrUn	3673177	3673285	109	SAT1_CF	blue	na
chrUn	3673305	3673864	560	SAT1_CF	blue	na
chrUn	3673876	3674551	676	SAT1_CF	blue	na
chrUn	3674594	3675744	1151	SAT1_CF	blue	na
chrUn	3675770	3676289	520	SAT1_CF	blue	na
chrUn	3676302	3676984	683	SAT1_CF	blue	na
chrUn	3676996	3677945	950	SAT1_CF	blue	na
chrUn	3677959	3680305	2347	SAT1_CF	blue	na
chrUn	3680386	3680551	166	SAT1_CF	blue	na
chrUn	3680732	3680917	186	SAT1_CF	blue	na
chrUn	3680953	3681453	501	SAT1_CF	blue	na
chrUn	3681471	3681580	110	SAT1_CF	blue	na
chrUn	3681593	3682208	616	SAT1_CF	blue	na
chrUn	3682225	3683088	864	SAT1_CF	blue	na
chrUn	3683122	3683641	520	SAT1_CF	blue	na
chrUn	3683654	3683761	108	SAT1_CF	blue	na
chrUn	3683783	3685467	1685	SAT1_CF	blue	na
chrUn	3685490	3685840	351	SAT1_CF	blue	na
chrUn	3686029	3686243	215	SAT1_CF	blue	na
chrUn	3686255	3687964	1710	SAT1_CF	blue	na
chrUn	3687992	3688149	158	SAT1_CF	blue	na
chrUn	3688194	3688696	503	SAT1_CF	blue	na
chrUn	3688709	3689389	681	SAT1_CF	blue	na
chrUn	3689401	3691010	1610	SAT1_CF	blue	na
chrUn	3691220	3691377	158	SAT1_CF	blue	na
chrUn	3691442	3692036	595	SAT1_CF	blue	na
chrUn	3692057	3693114	1058	SAT1_CF	blue	na
chrUn	3693127	3693627	501	SAT1_CF	blue	na
chrUn	3693645	3694585	941	SAT1_CF	blue	na
chrUn	3694606	3695125	520	SAT1_CF	blue	na
chrUn	3695136	3695538	403	SAT1_CF	blue	na

chrUn	3695598	3696667	1070	SAT1_CF	blue	na
chrUn	3696685	3699411	2727	SAT1_CF	blue	na
chrUn	3699453	3700547	1095	SAT1_CF	blue	na
chrUn	3700559	3700668	110	SAT1_CF	blue	na
chrUn	3700689	3701162	474	SAT1_CF	blue	na
chrUn	3701189	3701776	588	SAT1_CF	blue	na
chrUn	3701803	3702477	675	SAT1_CF	blue	na
chrUn	3702522	3703921	1400	SAT1_CF	blue	na
chrUn	3703974	3704664	691	SAT1_CF	blue	na
chrUn	3704679	3705249	571	SAT1_CF	blue	na
chrUn	3705279	3705871	593	SAT1_CF	blue	na
chrUn	3705899	3707504	1606	SAT1_CF	blue	na
chrUn	3707519	3707676	158	SAT1_CF	blue	na
chrUn	3707721	3708221	501	SAT1_CF	blue	na
chrUn	3708235	3708918	684	SAT1_CF	blue	na
chrUn	3708930	3709039	110	SAT1_CF	blue	na
chrUn	3709060	3710743	1684	SAT1_CF	blue	na
chrUn	3710813	3711314	502	SAT1_CF	blue	na
chrUn	3711327	3712006	680	SAT1_CF	blue	na
chrUn	3712030	3713462	1433	SAT1_CF	blue	na
chrUn	3713527	3714083	557	SAT1_CF	blue	na
chrUn	3714125	3714650	526	SAT1_CF	blue	na
chrUn	3714665	3714822	158	SAT1_CF	blue	na
chrUn	3714867	3715364	498	SAT1_CF	blue	na
chrUn	3715377	3716748	1372	SAT1_CF	blue	na
chrUn	3716791	3718482	1692	SAT1_CF	blue	na
chrUn	3718493	3719095	603	SAT1_CF	blue	na
chrUn	3719107	3720444	1338	SAT1_CF	blue	na
chrUn	3720466	3721148	683	SAT1_CF	blue	na
chrUn	3721160	3722010	851	SAT1_CF	blue	na
chrUn	3722023	3722948	926	SAT1_CF	blue	na
chrUn	3722980	3723504	525	SAT1_CF	blue	na
chrUn	3723547	3724504	958	SAT1_CF	blue	na
chrUn	3724561	3726017	1457	SAT1_CF	blue	na
chrUn	3726031	3726969	939	SAT1_CF	blue	na
chrUn	3727009	3727503	495	SAT1_CF	blue	na
chrUn	3727527	3728962	1436	SAT1_CF	blue	na
chrUn	3728974	3729147	174	SAT1_CF	blue	na
chrUn	3729190	3729972	783	SAT1_CF	blue	na
chrUn	3730052	3730599	548	SAT1_CF	blue	na
chrUn	3730610	3731008	399	SAT1_CF	blue	na
chrUn	3731048	3731549	502	SAT1_CF	blue	na

chrUn	3731567	3732235	669	SAT1_CF	blue	na
chrUn	3732278	3733241	964	SAT1_CF	blue	na
chrUn	3733285	3733832	548	SAT1_CF	blue	na
chrUn	3733851	3733942	92	SAT1_CF	blue	na
chrUn	3733985	3734346	362	SAT1_CF	blue	na
chrUn	3734392	3734891	500	SAT1_CF	blue	na
chrUn	3734905	3735588	684	SAT1_CF	blue	na
chrUn	3735600	3736452	853	SAT1_CF	blue	na
chrUn	3736490	3737037	548	SAT1_CF	blue	na
chrUn	3737052	3737411	360	SAT1_CF	blue	na
chrUn	3737471	3737952	482	SAT1_CF	blue	na
chrUn	3737965	3738649	685	SAT1_CF	blue	na
chrUn	3738662	3739841	1180	SAT1_CF	blue	na
chrUn	3739884	3741819	1936	SAT1_CF	blue	na
chrUn	3741831	3742591	761	SAT1_CF	blue	na
chrUn	3742603	3743462	860	SAT1_CF	blue	na
chrUn	3743504	3744047	544	SAT1_CF	blue	na
chrUn	3744089	3744291	203	SAT1_CF	blue	na
chrUn	3744472	3744657	186	SAT1_CF	blue	na
chrUn	3744713	3745193	481	SAT1_CF	blue	na
chrUn	3745206	3745311	106	SAT1_CF	blue	na
chrUn	3745334	3746554	1221	SAT1_CF	blue	na
chrUn	3746568	3746670	103	SAT1_CF	blue	na
chrUn	3746695	3747639	945	SAT1_CF	blue	na
chrUn	3747651	3748334	684	SAT1_CF	blue	na
chrUn	3748346	3749353	1008	SAT1_CF	blue	na
chrUn	3749381	3749933	553	SAT1_CF	blue	na
chrUn	3749945	3750760	816	SAT1_CF	blue	na
chrUn	3750790	3751316	527	SAT1_CF	blue	na
chrUn	3751379	3751486	108	SAT1_CF	blue	na
chrUn	3751531	3752032	502	SAT1_CF	blue	na
chrUn	3752045	3753377	1333	SAT1_CF	blue	na
chrUn	3753426	3753761	336	SAT1_CF	blue	na
chrUn	3753793	3754280	488	SAT1_CF	blue	na
chrUn	3754291	3757519	3229	SAT1_CF	blue	na
chrUn	3757547	3758585	1039	SAT1_CF	blue	na
chrUn	3758597	3758914	318	SAT1_CF	blue	na
chrUn	3758927	3759428	502	SAT1_CF	blue	na
chrUn	3759446	3760120	675	SAT1_CF	blue	na
chrUn	3760165	3760855	691	SAT1_CF	blue	na
chrUn	3760880	3761973	1094	SAT1_CF	blue	na
chrUn	3761985	3762874	890	SAT1_CF	blue	na

chrUn	3762888	3764256	1369	SAT1_CF	blue	na
chrUn	3764302	3764824	523	SAT1_CF	blue	na
chrUn	3764837	3766792	1956	SAT1_CF	blue	na
chrUn	3766836	3767383	548	SAT1_CF	blue	na
chrUn	3767426	3767628	203	SAT1_CF	blue	na
chrUn	3767809	3767989	181	SAT1_CF	blue	na
chrUn	3768050	3768520	471	SAT1_CF	blue	na
chrUn	3768543	3769867	1325	SAT1_CF	blue	na
chrUn	3769882	3770039	158	SAT1_CF	blue	na
chrUn	3770065	3771456	1392	SAT1_CF	blue	na
chrUn	3771534	3772530	997	SAT1_CF	blue	na
chrUn	3772542	3773215	674	SAT1_CF	blue	na
chrUn	3773426	3773583	158	SAT1_CF	blue	na
chrUn	3773648	3774121	474	SAT1_CF	blue	na
chrUn	3774134	3774242	109	SAT1_CF	blue	na
chrUn	3774263	3774895	633	SAT1_CF	blue	na
chrUn	3774933	3775247	315	SAT1_CF	blue	na
chrUn	3775334	3775835	502	SAT1_CF	blue	na
chrUn	3775853	3776793	941	SAT1_CF	blue	na
chrUn	3776814	3777332	519	SAT1_CF	blue	na
chrUn	3777343	3777698	356	SAT1_CF	blue	na
chrUn	3777785	3778873	1089	SAT1_CF	blue	na
chrUn	3778891	3780216	1326	SAT1_CF	blue	na
chrUn	3780259	3781185	927	SAT1_CF	blue	na
chrUn	3781247	3781488	242	SAT1_CF	blue	na
chrUn	3781519	3782067	549	SAT1_CF	blue	na
chrUn	3782088	3782656	569	SAT1_CF	blue	na
chrUn	3782721	3783291	571	SAT1_CF	blue	na
chrUn	3783310	3784798	1489	SAT1_CF	blue	na
chrUn	3784877	3785791	915	SAT1_CF	blue	na
chrUn	3785878	3786378	501	SAT1_CF	blue	na
chrUn	3786391	3788564	2174	SAT1_CF	blue	na
chrUn	3788612	3789118	507	SAT1_CF	blue	na
chrUn	3789151	3789920	770	SAT1_CF	blue	na
chrUn	3789932	3792687	2756	SAT1_CF	blue	na
chrUn	3792727	3793227	501	SAT1_CF	blue	na
chrUn	3793272	3793924	653	SAT1_CF	blue	na
chrUn	3793936	3794610	675	SAT1_CF	blue	na
chrUn	3794654	3795741	1088	SAT1_CF	blue	na
chrUn	3795825	3797261	1437	SAT1_CF	blue	na
chrUn	3797303	3797468	166	SAT1_CF	blue	na
chrUn	3797647	3797832	186	SAT1_CF	blue	na

chrUn	3797849	3798368	520	SAT1_CF	blue	na
chrUn	3798381	3798489	109	SAT1_CF	blue	na
chrUn	3798509	3799068	560	SAT1_CF	blue	na
chrUn	3799080	3800824	1745	SAT1_CF	blue	na
chrUn	3800872	3801384	513	SAT1_CF	blue	na
chrUn	3801462	3802007	546	SAT1_CF	blue	na
chrUn	3802020	3802127	108	SAT1_CF	blue	na
chrUn	3802150	3802722	573	SAT1_CF	blue	na
chrUn	3802734	3803410	677	SAT1_CF	blue	na
chrUn	3803423	3804920	1498	SAT1_CF	blue	na
chrUn	3804960	3805454	495	SAT1_CF	blue	na
chrUn	3805474	3806156	683	SAT1_CF	blue	na
chrUn	3806169	3807394	1226	SAT1_CF	blue	na
chrUn	3807408	3807480	73	SAT1_CF	blue	na
chrUn	3807537	3808286	750	SAT1_CF	blue	na
chrUn	3808315	3808861	547	SAT1_CF	blue	na
chrUn	3808873	3809547	675	SAT1_CF	blue	na
chrUn	3809596	3809712	117	SAT1_CF	blue	na
chrUn	3809754	3810297	544	SAT1_CF	blue	na
chrUn	3810343	3811197	855	SAT1_CF	blue	na
chrUn	3811280	3811905	626	SAT1_CF	blue	na
chrUn	3811959	3812493	535	SAT1_CF	blue	na
chrUn	3812575	3813085	511	SAT1_CF	blue	na
chrUn	3813113	3813249	137	SAT1_CF	blue	na
chrUn	3813315	3814503	1189	SAT1_CF	blue	na
chrUn	3814546	3815178	633	SAT1_CF	blue	na
chrUn	3815205	3815767	563	SAT1_CF	blue	na
chrUn	3815847	3816161	315	SAT1_CF	blue	na
chrUn	3816180	3816722	543	SAT1_CF	blue	na
chrUn	3816793	3817861	1069	SAT1_CF	blue	na
chrUn	3818042	3818215	174	SAT1_CF	blue	na
chrUn	3818283	3818843	561	SAT1_CF	blue	na
chrUn	3818905	3820601	1697	SAT1_CF	blue	na
chrUn	3820614	3821372	759	SAT1_CF	blue	na
chrUn	3821385	3822092	708	SAT1_CF	blue	na
chrUn	3822134	3823991	1858	SAT1_CF	blue	na
chrUn	3824031	3824525	495	SAT1_CF	blue	na
chrUn	3824545	3824653	109	SAT1_CF	blue	na
chrUn	3824674	3825228	555	SAT1_CF	blue	na
chrUn	3825240	3825915	676	SAT1_CF	blue	na
chrUn	3825958	3826913	956	SAT1_CF	blue	na
chrUn	3826991	3827538	548	SAT1_CF	blue	na

chrUn	3827576	3827903	328	SAT1_CF	blue	na
chrUn	3827990	3828491	502	SAT1_CF	blue	na
chrUn	3828505	3829265	761	SAT1_CF	blue	na
chrUn	3829277	3829995	719	SAT1_CF	blue	na
chrUn	3830023	3831085	1063	SAT1_CF	blue	na
chrUn	3831128	3831960	833	SAT1_CF	blue	na
chrUn	3832190	3832738	549	SAT1_CF	blue	na
chrUn	3832760	3832849	90	SAT1_CF	blue	na
chrUn	3832873	3833880	1008	SAT1_CF	blue	na
chrUn	3833893	3834653	761	SAT1_CF	blue	na
chrUn	3834671	3836322	1652	SAT1_CF	blue	na
chrUn	3836334	3837700	1367	SAT1_CF	blue	na
chrUn	3837765	3838327	563	SAT1_CF	blue	na
chrUn	3838370	3838921	552	SAT1_CF	blue	na
chrUn	3838932	3839543	612	SAT1_CF	blue	na
chrUn	3839566	3840232	667	SAT1_CF	blue	na
chrUn	3840275	3840801	527	SAT1_CF	blue	na
chrUn	3840823	3842315	1493	SAT1_CF	blue	na
chrUn	3842355	3842856	502	SAT1_CF	blue	na
chrUn	3842869	3843064	196	SAT1_CF	blue	na
chrUn	3843080	3843624	545	SAT1_CF	blue	na
chrUn	3843666	3844210	545	SAT1_CF	blue	na
chrUn	3844226	3845825	1600	SAT1_CF	blue	na
chrUn	3845837	3846490	654	SAT1_CF	blue	na
chrUn	3846570	3846796	227	SAT1_CF	blue	na
chrUn	3846807	3848198	1392	SAT1_CF	blue	na
chrUn	3848277	3848801	525	SAT1_CF	blue	na
chrUn	3848865	3849410	546	SAT1_CF	blue	na
chrUn	3849423	3849529	107	SAT1_CF	blue	na
chrUn	3849552	3850126	575	SAT1_CF	blue	na
chrUn	3850138	3850822	685	SAT1_CF	blue	na
chrUn	3850835	3851481	647	SAT1_CF	blue	na
chrUn	3851493	3851601	109	SAT1_CF	blue	na
chrUn	3851624	3852350	727	SAT1_CF	blue	na
chrUn	3852392	3852933	542	SAT1_CF	blue	na
chrUn	3852980	3854078	1099	SAT1_CF	blue	na
chrUn	3854266	3854463	198	SAT1_CF	blue	na
chrUn	3854523	3855003	481	SAT1_CF	blue	na
chrUn	3855017	3857517	2501	SAT1_CF	blue	na
chrUn	3857597	3859032	1436	SAT1_CF	blue	na
chrUn	3859046	3859405	360	SAT1_CF	blue	na
chrUn	3859426	3859946	521	SAT1_CF	blue	na

chrUn	3859960	3861358	1399	SAT1_CF	blue	na
chrUn	3861375	3861990	616	SAT1_CF	blue	na
chrUn	3862002	3862678	677	SAT1_CF	blue	na
chrUn	3862721	3863260	540	SAT1_CF	blue	na
chrUn	3863274	3864820	1547	SAT1_CF	blue	na
chrUn	3864886	3865406	521	SAT1_CF	blue	na
chrUn	3865419	3865821	403	SAT1_CF	blue	na
chrUn	3865842	3866362	521	SAT1_CF	blue	na
chrUn	3866375	3866978	604	SAT1_CF	blue	na
chrUn	3866990	3867843	854	SAT1_CF	blue	na
chrUn	3867856	3868366	511	SAT1_CF	blue	na
chrUn	3868528	3869803	1276	SAT1_CF	blue	na
chrUn	3869824	3870710	887	SAT1_CF	blue	na
chrUn	3870797	3871295	499	SAT1_CF	blue	na
chrUn	3871308	3871415	108	SAT1_CF	blue	na
chrUn	3871436	3872681	1246	SAT1_CF	blue	na
chrUn	3872694	3873369	676	SAT1_CF	blue	na
chrUn	3873412	3873952	541	SAT1_CF	blue	na
chrUn	3873965	3875109	1145	SAT1_CF	blue	na
chrUn	3875120	3875227	108	SAT1_CF	blue	na
chrUn	3875253	3875773	521	SAT1_CF	blue	na
chrUn	3875786	3876392	607	SAT1_CF	blue	na
chrUn	3876404	3877081	678	SAT1_CF	blue	na
chrUn	3877124	3877666	543	SAT1_CF	blue	na
chrUn	3877714	3877929	216	SAT1_CF	blue	na
chrUn	3877947	3878533	587	SAT1_CF	blue	na
chrUn	3879916	3880493	578	SAT1_CF	blue	na
chrUn	3880533	3881027	495	SAT1_CF	blue	na
chrUn	3881047	3881731	685	SAT1_CF	blue	na
chrUn	3881743	3882940	1198	SAT1_CF	blue	na
chrUn	3882982	3883524	543	SAT1_CF	blue	na
chrUn	3883624	3885358	1735	SAT1_CF	blue	na
chrUn	3885376	3887399	2024	SAT1_CF	blue	na
chrUn	3887479	3887965	487	SAT1_CF	blue	na
chrUn	3888015	3888171	157	SAT1_CF	blue	na
chrUn	3888197	3889402	1206	SAT1_CF	blue	na
chrUn	3889422	3890784	1363	SAT1_CF	blue	na
chrUn	3890827	3891455	629	SAT1_CF	blue	na
chrUn	3891483	3892689	1207	SAT1_CF	blue	na
chrUn	3892704	3893406	703	SAT1_CF	blue	na
chrUn	3893419	3895769	2351	SAT1_CF	blue	na
chrUn	3895810	3896764	955	SAT1_CF	blue	na

chrUn	3896821	3897368	548	SAT1_CF	blue	na
chrUn	3897381	3899181	1801	SAT1_CF	blue	na
chrUn	3899268	3899769	502	SAT1_CF	blue	na
chrUn	3899782	3900542	761	SAT1_CF	blue	na
chrUn	3900559	3902795	2237	SAT1_CF	blue	na
chrUn	3902808	3903159	352	SAT1_CF	blue	na
chrUn	3903212	3903712	501	SAT1_CF	blue	na
chrUn	3903730	3904795	1066	SAT1_CF	blue	na
chrUn	3904821	3905231	411	SAT1_CF	blue	na
chrUn	3905257	3905737	481	SAT1_CF	blue	na
chrUn	3905764	3906447	684	SAT1_CF	blue	na
chrUn	3906460	3907326	867	SAT1_CF	blue	na
chrUn	3907356	3907892	537	SAT1_CF	blue	na
chrUn	3907967	3908213	247	SAT1_CF	blue	na
chrUn	3908247	3909170	924	SAT1_CF	blue	na
chrUn	3909210	3909711	502	SAT1_CF	blue	na
chrUn	3909725	3910406	682	SAT1_CF	blue	na
chrUn	3910418	3911090	673	SAT1_CF	blue	na
chrUn	3911133	3911765	633	SAT1_CF	blue	na
chrUn	3911793	3912435	643	SAT1_CF	blue	na
chrUn	3912474	3912986	513	SAT1_CF	blue	na
chrUn	3913014	3913718	705	SAT1_CF	blue	na
chrUn	3913729	3915028	1300	SAT1_CF	blue	na
chrUn	3915071	3916064	994	SAT1_CF	blue	na
chrUn	3916108	3917065	958	SAT1_CF	blue	na
chrUn	3917105	3917606	502	SAT1_CF	blue	na
chrUn	3917619	3918380	762	SAT1_CF	blue	na
chrUn	3918392	3919655	1264	SAT1_CF	blue	na
chrUn	3919705	3920249	545	SAT1_CF	blue	na
chrUn	3920287	3920634	348	SAT1_CF	blue	na
chrUn	3920676	3921261	586	SAT1_CF	blue	na
chrUn	3921273	3922433	1161	SAT1_CF	blue	na
chrUn	3922446	3923122	677	SAT1_CF	blue	na
chrUn	3923135	3924739	1605	SAT1_CF	blue	na
chrUn	3924764	3925597	834	SAT1_CF	blue	na
chrUn	3925610	3925718	109	SAT1_CF	blue	na
chrUn	3925739	3926117	379	SAT1_CF	blue	na
chrUn	3926204	3926706	503	SAT1_CF	blue	na
chrUn	3926731	3927400	670	SAT1_CF	blue	na
chrUn	3927412	3928271	860	SAT1_CF	blue	na
chrUn	3928313	3929214	902	SAT1_CF	blue	na
chrUn	3929235	3929751	517	SAT1_CF	blue	na

chrUn	3929789	3930120	332	SAT1_CF	blue	na
chrUn	3930207	3930709	503	SAT1_CF	blue	na
chrUn	3930721	3930827	107	SAT1_CF	blue	na
chrUn	3930851	3931395	545	SAT1_CF	blue	na
chrUn	3931439	3931982	544	SAT1_CF	blue	na
chrUn	3932000	3932686	687	SAT1_CF	blue	na
chrUn	3932698	3933274	577	SAT1_CF	blue	na
chrUn	3933289	3933548	260	SAT1_CF	blue	na
chrUn	3933561	3933601	41	SAT1_CF	blue	na
chrUn	3933634	3934671	1038	SAT1_CF	blue	na
chrUn	3934751	3935276	526	SAT1_CF	blue	na
chrUn	3935291	3935427	137	SAT1_CF	blue	na
chrUn	3935522	3936041	520	SAT1_CF	blue	na
chrUn	3936098	3936645	548	SAT1_CF	blue	na
chrUn	3936687	3937230	544	SAT1_CF	blue	na
chrUn	3937272	3937554	283	SAT1_CF	blue	na
chrUn	3937572	3938460	889	SAT1_CF	blue	na
chrUn	3938547	3939046	500	SAT1_CF	blue	na
chrUn	3939073	3939904	832	SAT1_CF	blue	na
chrUn	3939927	3940445	519	SAT1_CF	blue	na
chrUn	3940457	3941312	856	SAT1_CF	blue	na
chrUn	3941355	3942164	810	SAT1_CF	blue	na
chrUn	3942189	3942818	630	SAT1_CF	blue	na
chrUn	3942846	3943410	565	SAT1_CF	blue	na
chrUn	3943490	3944014	525	SAT1_CF	blue	na
chrUn	3944029	3944186	158	SAT1_CF	blue	na
chrUn	3944230	3944732	503	SAT1_CF	blue	na
chrUn	3944754	3946108	1355	SAT1_CF	blue	na
chrUn	3946158	3946532	375	SAT1_CF	blue	na
chrUn	3946588	3947780	1193	SAT1_CF	blue	na
chrUn	3947823	3948149	327	SAT1_CF	blue	na
chrUn	3948193	3949729	1537	SAT1_CF	blue	na
chrUn	3949742	3949808	67	SAT1_CF	blue	na
chrUn	3949836	3950351	516	SAT1_CF	blue	na
chrUn	3950369	3951221	853	SAT1_CF	blue	na
chrUn	3951263	3952228	966	SAT1_CF	blue	na
chrUn	3952240	3953591	1352	SAT1_CF	blue	na
chrUn	3953619	3956250	2632	SAT1_CF	blue	na
chrUn	3956293	3957127	835	SAT1_CF	blue	na
chrUn	3957182	3957682	501	SAT1_CF	blue	na
chrUn	3957695	3957801	107	SAT1_CF	blue	na
chrUn	3957824	3959030	1207	SAT1_CF	blue	na

chrUn	3959055	3959380	326	SAT1_CF	blue	na
chrUn	3959460	3959968	509	SAT1_CF	blue	na
chrUn	3959981	3960383	403	SAT1_CF	blue	na
chrUn	3960423	3960923	501	SAT1_CF	blue	na
chrUn	3960935	3961786	852	SAT1_CF	blue	na
chrUn	3961799	3962415	617	SAT1_CF	blue	na
chrUn	3962494	3963018	525	SAT1_CF	blue	na
chrUn	3963033	3963168	136	SAT1_CF	blue	na
chrUn	3963235	3963735	501	SAT1_CF	blue	na
chrUn	3963748	3964357	610	SAT1_CF	blue	na
chrUn	3964369	3965043	675	SAT1_CF	blue	na
chrUn	3965056	3965226	171	SAT1_CF	blue	na
chrUn	3965268	3967078	1811	SAT1_CF	blue	na
chrUn	3967165	3967666	502	SAT1_CF	blue	na
chrUn	3967690	3967792	103	SAT1_CF	blue	na
chrUn	3967809	3968793	985	SAT1_CF	blue	na
chrUn	3968815	3970481	1667	SAT1_CF	blue	na
chrUn	3970524	3971069	546	SAT1_CF	blue	na
chrUn	3971111	3972040	930	SAT1_CF	blue	na
chrUn	3972082	3972749	668	SAT1_CF	blue	na
chrUn	3972815	3973355	541	SAT1_CF	blue	na
chrUn	3973372	3974040	669	SAT1_CF	blue	na
chrUn	3974118	3975077	960	SAT1_CF	blue	na
chrUn	3975144	3976921	1778	SAT1_CF	blue	na
chrUn	3977118	3977332	215	SAT1_CF	blue	na
chrUn	3977344	3978148	805	SAT1_CF	blue	na
chrUn	3978162	3978679	518	SAT1_CF	blue	na
chrUn	3978723	3979287	565	SAT1_CF	blue	na
chrUn	3979330	3980027	698	SAT1_CF	blue	na
chrUn	3980094	3980739	646	SAT1_CF	blue	na
chrUn	3982164	3982681	518	SAT1_CF	blue	na
chrUn	3982768	3983262	495	SAT1_CF	blue	na
chrUn	3983282	3983964	683	SAT1_CF	blue	na
chrUn	3983976	3984652	677	SAT1_CF	blue	na
chrUn	3984694	3985915	1222	SAT1_CF	blue	na
chrUn	3985995	3986519	525	SAT1_CF	blue	na
chrUn	3986534	3986690	157	SAT1_CF	blue	na
chrUn	3986716	3987237	522	SAT1_CF	blue	na
chrUn	3987250	3987325	76	SAT1_CF	blue	na
chrUn	3987344	3987897	554	SAT1_CF	blue	na
chrUn	3987940	3988903	964	SAT1_CF	blue	na
chrUn	3988930	3990463	1534	SAT1_CF	blue	na

chrUn	3990485	3990977	493	SAT1_CF	blue	na
chrUn	3991020	3991985	966	SAT1_CF	blue	na
chrUn	3992052	3992564	513	SAT1_CF	blue	na
chrUn	3992607	3992979	373	SAT1_CF	blue	na
chrUn	3993015	3993517	503	SAT1_CF	blue	na
chrUn	3993529	3993637	109	SAT1_CF	blue	na
chrUn	3993658	3994713	1056	SAT1_CF	blue	na
chrUn	3994726	3994797	72	SAT1_CF	blue	na
chrUn	3994820	3995373	554	SAT1_CF	blue	na
chrUn	3995384	3995985	602	SAT1_CF	blue	na
chrUn	3995997	3996672	676	SAT1_CF	blue	na
chrUn	3996715	3997344	630	SAT1_CF	blue	na
chrUn	3997672	3998207	536	SAT1_CF	blue	na
chrUn	3998233	3998746	514	SAT1_CF	blue	na
chrUn	3998758	4000075	1318	SAT1_CF	blue	na
chrUn	4000110	4000922	813	SAT1_CF	blue	na
chrUn	4000935	4001011	77	SAT1_CF	blue	na
chrUn	4001064	4004359	3296	SAT1_CF	blue	na
chrUn	4004446	4005541	1096	SAT1_CF	blue	na
chrUn	4005572	4006058	487	SAT1_CF	blue	na
chrUn	4006109	4006266	158	SAT1_CF	blue	na
chrUn	4006331	4008156	1826	SAT1_CF	blue	na
chrUn	4008181	4010343	2163	SAT1_CF	blue	na
chrUn	4010396	4010898	503	SAT1_CF	blue	na
chrUn	4010910	4011016	107	SAT1_CF	blue	na
chrUn	4011040	4011665	626	SAT1_CF	blue	na
chrUn	4011689	4012548	860	SAT1_CF	blue	na
chrUn	4012566	4013250	685	SAT1_CF	blue	na
chrUn	4013262	4014111	850	SAT1_CF	blue	na
chrUn	4014124	4016489	2366	SAT1_CF	blue	na
chrUn	4016501	4017361	861	SAT1_CF	blue	na
chrUn	4017404	4018194	791	SAT1_CF	blue	na
chrUn	4018238	4018780	543	SAT1_CF	blue	na
chrUn	4018804	4019476	673	SAT1_CF	blue	na
chrUn	4019488	4020162	675	SAT1_CF	blue	na
chrUn	4020177	4020821	645	SAT1_CF	blue	na
chrUn	4020866	4021171	306	SAT1_CF	blue	na
chrUn	4021251	4022163	913	SAT1_CF	blue	na
chrUn	4022250	4022751	502	SAT1_CF	blue	na
chrUn	4022764	4023447	684	SAT1_CF	blue	na
chrUn	4023459	4024367	909	SAT1_CF	blue	na
chrUn	4024397	4026609	2213	SAT1_CF	blue	na

chrUn	4026637	4026794	158	SAT1_CF	blue	na
chrUn	4026839	4027339	501	SAT1_CF	blue	na
chrUn	4027353	4028036	684	SAT1_CF	blue	na
chrUn	4028048	4028157	110	SAT1_CF	blue	na
chrUn	4028178	4029861	1684	SAT1_CF	blue	na
chrUn	4029931	4030432	502	SAT1_CF	blue	na
chrUn	4030445	4031124	680	SAT1_CF	blue	na
chrUn	4031148	4032580	1433	SAT1_CF	blue	na
chrUn	4032645	4033206	562	SAT1_CF	blue	na
chrUn	4033249	4033800	552	SAT1_CF	blue	na
chrUn	4033813	4034488	676	SAT1_CF	blue	na
chrUn	4034531	4035072	542	SAT1_CF	blue	na
chrUn	4035085	4036229	1145	SAT1_CF	blue	na
chrUn	4036240	4036348	109	SAT1_CF	blue	na
chrUn	4036394	4036894	501	SAT1_CF	blue	na
chrUn	4036907	4037513	607	SAT1_CF	blue	na
chrUn	4037525	4038207	683	SAT1_CF	blue	na
chrUn	4038219	4039863	1645	SAT1_CF	blue	na
chrUn	4039927	4040316	390	SAT1_CF	blue	na
chrUn	4040371	4040873	503	SAT1_CF	blue	na
chrUn	4040885	4040990	106	SAT1_CF	blue	na
chrUn	4041014	4041816	803	SAT1_CF	blue	na
chrUn	4041829	4041937	109	SAT1_CF	blue	na
chrUn	4041958	4042707	750	SAT1_CF	blue	na
chrUn	4042729	4045192	2464	SAT1_CF	blue	na
chrUn	4045373	4045557	185	SAT1_CF	blue	na
chrUn	4045573	4046091	519	SAT1_CF	blue	na
chrUn	4046105	4046639	535	SAT1_CF	blue	na
chrUn	4046651	4047504	854	SAT1_CF	blue	na
chrUn	4047554	4048922	1369	SAT1_CF	blue	na
chrUn	4048936	4049290	355	SAT1_CF	blue	na
chrUn	4049395	4049876	482	SAT1_CF	blue	na
chrUn	4049889	4049964	76	SAT1_CF	blue	na
chrUn	4049983	4051877	1895	SAT1_CF	blue	na
chrUn	4051921	4052484	564	SAT1_CF	blue	na
chrUn	4052527	4052721	195	SAT1_CF	blue	na
chrUn	4052749	4053300	552	SAT1_CF	blue	na
chrUn	4053312	4054682	1371	SAT1_CF	blue	na
chrUn	4054725	4055356	632	SAT1_CF	blue	na
chrUn	4055384	4055945	562	SAT1_CF	blue	na
chrUn	4056025	4056574	550	SAT1_CF	blue	na
chrUn	4056602	4056739	138	SAT1_CF	blue	na

chrUn	4056786	4057306	521	SAT1_CF	blue	na
chrUn	4057332	4058692	1361	SAT1_CF	blue	na
chrUn	4058739	4058858	120	SAT1_CF	blue	na
chrUn	4058900	4059682	783	SAT1_CF	blue	na
chrUn	4059725	4060270	546	SAT1_CF	blue	na
chrUn	4060335	4060893	559	SAT1_CF	blue	na
chrUn	4060905	4062445	1541	SAT1_CF	blue	na
chrUn	4062458	4062860	403	SAT1_CF	blue	na
chrUn	4062900	4063400	501	SAT1_CF	blue	na
chrUn	4063413	4063521	109	SAT1_CF	blue	na
chrUn	4063542	4064376	835	SAT1_CF	blue	na
chrUn	4064401	4065190	790	SAT1_CF	blue	na
chrUn	4065233	4066983	1751	SAT1_CF	blue	na
chrUn	4067008	4067333	326	SAT1_CF	blue	na
chrUn	4067412	4068322	911	SAT1_CF	blue	na
chrUn	4068411	4068911	501	SAT1_CF	blue	na
chrUn	4068924	4070296	1373	SAT1_CF	blue	na
chrUn	4070339	4071330	992	SAT1_CF	blue	na
chrUn	4071352	4071897	546	SAT1_CF	blue	na
chrUn	4071908	4072142	235	SAT1_CF	blue	na
chrUn	4072322	4072455	134	SAT1_CF	blue	na
chrUn	4072561	4073032	472	SAT1_CF	blue	na
chrUn	4073055	4075089	2035	SAT1_CF	blue	na
chrUn	4075176	4075677	502	SAT1_CF	blue	na
chrUn	4075690	4076450	761	SAT1_CF	blue	na
chrUn	4076463	4077828	1366	SAT1_CF	blue	na
chrUn	4077871	4079900	2030	SAT1_CF	blue	na
chrUn	4079913	4080414	502	SAT1_CF	blue	na
chrUn	4080427	4081773	1347	SAT1_CF	blue	na
chrUn	4081801	4082359	559	SAT1_CF	blue	na
chrUn	4082443	4082990	548	SAT1_CF	blue	na
chrUn	4083018	4083154	137	SAT1_CF	blue	na
chrUn	4083220	4083718	499	SAT1_CF	blue	na
chrUn	4083741	4084413	673	SAT1_CF	blue	na
chrUn	4084425	4085261	837	SAT1_CF	blue	na
chrUn	4085303	4086341	1039	SAT1_CF	blue	na
chrUn	4086398	4087030	633	SAT1_CF	blue	na
chrUn	4087042	4087645	604	SAT1_CF	blue	na
chrUn	4087667	4088336	670	SAT1_CF	blue	na
chrUn	4088348	4089212	865	SAT1_CF	blue	na
chrUn	4089254	4092289	3036	SAT1_CF	blue	na
chrUn	4092471	4092652	182	SAT1_CF	blue	na

chrUn	4092673	4093192	520	SAT1_CF	blue	na
chrUn	4093205	4093310	106	SAT1_CF	blue	na
chrUn	4093332	4095060	1729	SAT1_CF	blue	na
chrUn	4095083	4095571	489	SAT1_CF	blue	na
chrUn	4095666	4096186	521	SAT1_CF	blue	na
chrUn	4096208	4097038	831	SAT1_CF	blue	na
chrUn	4097065	4098202	1138	SAT1_CF	blue	na
chrUn	4098244	4098785	542	SAT1_CF	blue	na
chrUn	4098824	4100099	1276	SAT1_CF	blue	na
chrUn	4100139	4100641	503	SAT1_CF	blue	na
chrUn	4100654	4103215	2562	SAT1_CF	blue	na
chrUn	4103257	4104039	783	SAT1_CF	blue	na
chrUn	4104118	4104626	509	SAT1_CF	blue	na
chrUn	4104640	4104995	356	SAT1_CF	blue	na
chrUn	4105082	4105584	503	SAT1_CF	blue	na
chrUn	4105597	4106268	672	SAT1_CF	blue	na
chrUn	4106317	4107842	1526	SAT1_CF	blue	na
chrUn	4107948	4108312	365	SAT1_CF	blue	na
chrUn	4108367	4108869	503	SAT1_CF	blue	na
chrUn	4108882	4108988	107	SAT1_CF	blue	na
chrUn	4109010	4109568	559	SAT1_CF	blue	na
chrUn	4109580	4111956	2377	SAT1_CF	blue	na
chrUn	4111969	4112324	356	SAT1_CF	blue	na
chrUn	4112440	4112960	521	SAT1_CF	blue	na
chrUn	4112991	4114064	1074	SAT1_CF	blue	na
chrUn	4114078	4114599	522	SAT1_CF	blue	na
chrUn	4114633	4114965	333	SAT1_CF	blue	na
chrUn	4115052	4115553	502	SAT1_CF	blue	na
chrUn	4115566	4116248	683	SAT1_CF	blue	na
chrUn	4116260	4117712	1453	SAT1_CF	blue	na
chrUn	4117725	4118299	575	SAT1_CF	blue	na
chrUn	4118432	4119127	696	SAT1_CF	blue	na
chrUn	4119192	4119972	781	SAT1_CF	blue	na
chrUn	4120034	4121730	1697	SAT1_CF	blue	na
chrUn	4121743	4122502	760	SAT1_CF	blue	na
chrUn	4122514	4123222	709	SAT1_CF	blue	na
chrUn	4123264	4124705	1442	SAT1_CF	blue	na
chrUn	4124719	4125121	403	SAT1_CF	blue	na
chrUn	4125161	4125783	623	SAT1_CF	blue	na
chrUn	4125804	4126358	555	SAT1_CF	blue	na
chrUn	4126370	4126478	109	SAT1_CF	blue	na
chrUn	4126499	4127044	546	SAT1_CF	blue	na

chrUn	4127087	4127920	834	SAT1_CF	blue	na
chrUn	4128110	4129875	1766	SAT1_CF	blue	na
chrUn	4129888	4130648	761	SAT1_CF	blue	na
chrUn	4130666	4130768	103	SAT1_CF	blue	na
chrUn	4130789	4132317	1529	SAT1_CF	blue	na
chrUn	4132329	4133695	1367	SAT1_CF	blue	na
chrUn	4133759	4134321	563	SAT1_CF	blue	na
chrUn	4134364	4134916	553	SAT1_CF	blue	na
chrUn	4134928	4135539	612	SAT1_CF	blue	na
chrUn	4135562	4136227	666	SAT1_CF	blue	na
chrUn	4136270	4138311	2042	SAT1_CF	blue	na
chrUn	4138351	4138853	503	SAT1_CF	blue	na
chrUn	4138866	4138971	106	SAT1_CF	blue	na
chrUn	4140376	4140893	518	SAT1_CF	blue	na
chrUn	4141021	4142699	1679	SAT1_CF	blue	na
chrUn	4142711	4143364	654	SAT1_CF	blue	na
chrUn	4143444	4143670	227	SAT1_CF	blue	na
chrUn	4143681	4144217	537	SAT1_CF	blue	na
chrUn	4144235	4144553	319	SAT1_CF	blue	na
chrUn	4144606	4145108	503	SAT1_CF	blue	na
chrUn	4145121	4145960	840	SAT1_CF	blue	na
chrUn	4145972	4147233	1262	SAT1_CF	blue	na
chrUn	4147269	4148279	1011	SAT1_CF	blue	na
chrUn	4148345	4148930	586	SAT1_CF	blue	na
chrUn	4149012	4149554	543	SAT1_CF	blue	na
chrUn	4149567	4150069	503	SAT1_CF	blue	na
chrUn	4150081	4150762	682	SAT1_CF	blue	na
chrUn	4150776	4151450	675	SAT1_CF	blue	na
chrUn	4151530	4152041	512	SAT1_CF	blue	na
chrUn	4152069	4152820	752	SAT1_CF	blue	na
chrUn	4152851	4153377	527	SAT1_CF	blue	na
chrUn	4153421	4154872	1452	SAT1_CF	blue	na
chrUn	4154912	4155414	503	SAT1_CF	blue	na
chrUn	4155427	4156111	685	SAT1_CF	blue	na
chrUn	4156123	4157457	1335	SAT1_CF	blue	na
chrUn	4157501	4158833	1333	SAT1_CF	blue	na
chrUn	4158920	4159421	502	SAT1_CF	blue	na
chrUn	4159448	4160283	836	SAT1_CF	blue	na
chrUn	4160302	4160821	520	SAT1_CF	blue	na
chrUn	4160833	4161693	861	SAT1_CF	blue	na
chrUn	4161736	4162525	790	SAT1_CF	blue	na
chrUn	4162569	4163109	541	SAT1_CF	blue	na

chrUn	4163121	4163694	574	SAT1_CF	blue	na
chrUn	4163949	4164083	135	SAT1_CF	blue	na
chrUn	4164149	4164649	501	SAT1_CF	blue	na
chrUn	4164662	4164767	106	SAT1_CF	blue	na
chrUn	4164790	4166485	1696	SAT1_CF	blue	na
chrUn	4166498	4167258	761	SAT1_CF	blue	na
chrUn	4167270	4167953	684	SAT1_CF	blue	na
chrUn	4167965	4168750	786	SAT1_CF	blue	na
chrUn	4168795	4169297	503	SAT1_CF	blue	na
chrUn	4169310	4169991	682	SAT1_CF	blue	na
chrUn	4170003	4170679	677	SAT1_CF	blue	na
chrUn	4170722	4171270	549	SAT1_CF	blue	na
chrUn	4171313	4171876	564	SAT1_CF	blue	na
chrUn	4171918	4172699	782	SAT1_CF	blue	na
chrUn	4172742	4173285	544	SAT1_CF	blue	na
chrUn	4173298	4174133	836	SAT1_CF	blue	na
chrUn	4174146	4174848	703	SAT1_CF	blue	na
chrUn	4174870	4176340	1471	SAT1_CF	blue	na
chrUn	4176365	4176926	562	SAT1_CF	blue	na
chrUn	4177013	4177513	501	SAT1_CF	blue	na
chrUn	4177526	4178718	1193	SAT1_CF	blue	na
chrUn	4178729	4179575	847	SAT1_CF	blue	na
chrUn	4179619	4180167	549	SAT1_CF	blue	na
chrUn	4180180	4180285	106	SAT1_CF	blue	na
chrUn	4180308	4181319	1012	SAT1_CF	blue	na
chrUn	4181376	4181708	333	SAT1_CF	blue	na
chrUn	4181795	4182296	502	SAT1_CF	blue	na
chrUn	4182313	4183534	1222	SAT1_CF	blue	na
chrUn	4183583	4183675	93	SAT1_CF	blue	na
chrUn	4183732	4184281	550	SAT1_CF	blue	na
chrUn	4184295	4184908	614	SAT1_CF	blue	na
chrUn	4184920	4185770	851	SAT1_CF	blue	na
chrUn	4185821	4187010	1190	SAT1_CF	blue	na
chrUn	4187090	4187613	524	SAT1_CF	blue	na
chrUn	4187628	4187763	136	SAT1_CF	blue	na
chrUn	4187829	4188331	503	SAT1_CF	blue	na
chrUn	4188344	4189674	1331	SAT1_CF	blue	na
chrUn	4189757	4190384	628	SAT1_CF	blue	na
chrUn	4190401	4190949	549	SAT1_CF	blue	na
chrUn	4190977	4191680	704	SAT1_CF	blue	na
chrUn	4191703	4192374	672	SAT1_CF	blue	na
chrUn	4192386	4192459	74	SAT1_CF	blue	na

chrUn	4192515	4193060	546	SAT1_CF	blue	na
chrUn	4193156	4193697	542	SAT1_CF	blue	na
chrUn	4193741	4195130	1390	SAT1_CF	blue	na
chrUn	4195172	4195664	493	SAT1_CF	blue	na
chrUn	4195684	4196354	671	SAT1_CF	blue	na
chrUn	4196375	4197075	701	SAT1_CF	blue	na
chrUn	4197087	4197948	862	SAT1_CF	blue	na
chrUn	4197990	4198769	780	SAT1_CF	blue	na
chrUn	4198849	4199796	948	SAT1_CF	blue	na
chrUn	4199809	4202006	2198	SAT1_CF	blue	na
chrUn	4202027	4202547	521	SAT1_CF	blue	na
chrUn	4202560	4203263	704	SAT1_CF	blue	na
chrUn	4203275	4203952	678	SAT1_CF	blue	na
chrUn	4203995	4204539	545	SAT1_CF	blue	na
chrUn	4204672	4205368	697	SAT1_CF	blue	na
chrUn	4205433	4205590	158	SAT1_CF	blue	na
chrUn	4205635	4206222	588	SAT1_CF	blue	na
chrUn	4206277	4207521	1245	SAT1_CF	blue	na
chrUn	4207564	4208561	998	SAT1_CF	blue	na
chrUn	4208605	4209846	1242	SAT1_CF	blue	na
chrUn	4209890	4211095	1206	SAT1_CF	blue	na
chrUn	4211122	4211685	564	SAT1_CF	blue	na
chrUn	4211765	4212077	313	SAT1_CF	blue	na
chrUn	4212096	4212638	543	SAT1_CF	blue	na
chrUn	4212709	4213339	631	SAT1_CF	blue	na
chrUn	4213351	4213460	110	SAT1_CF	blue	na
chrUn	4213481	4214510	1030	SAT1_CF	blue	na
chrUn	4214530	4215139	610	SAT1_CF	blue	na
chrUn	4215182	4215384	203	SAT1_CF	blue	na
chrUn	4215565	4215750	186	SAT1_CF	blue	na
chrUn	4215767	4216286	520	SAT1_CF	blue	na
chrUn	4216299	4216980	682	SAT1_CF	blue	na
chrUn	4216992	4217675	684	SAT1_CF	blue	na
chrUn	4217687	4219017	1331	SAT1_CF	blue	na
chrUn	4219061	4219620	560	SAT1_CF	blue	na
chrUn	4219704	4220250	547	SAT1_CF	blue	na
chrUn	4220278	4220413	136	SAT1_CF	blue	na
chrUn	4220479	4220980	502	SAT1_CF	blue	na
chrUn	4221003	4222073	1071	SAT1_CF	blue	na
chrUn	4222086	4223846	1761	SAT1_CF	blue	na
chrUn	4223867	4224390	524	SAT1_CF	blue	na
chrUn	4224433	4224802	370	SAT1_CF	blue	na

chrUn	4224842	4225344	503	SAT1_CF	blue	na
chrUn	4225357	4226039	683	SAT1_CF	blue	na
chrUn	4226051	4226812	762	SAT1_CF	blue	na
chrUn	4226824	4228156	1333	SAT1_CF	blue	na
chrUn	4228199	4228571	373	SAT1_CF	blue	na
chrUn	4228607	4229109	503	SAT1_CF	blue	na
chrUn	4229121	4229230	110	SAT1_CF	blue	na
chrUn	4229251	4229798	548	SAT1_CF	blue	na
chrUn	4229841	4231357	1517	SAT1_CF	blue	na
chrUn	4231405	4232185	781	SAT1_CF	blue	na
chrUn	4232240	4233298	1059	SAT1_CF	blue	na
chrUn	4233375	4233917	543	SAT1_CF	blue	na
chrUn	4233930	4234036	107	SAT1_CF	blue	na
chrUn	4234059	4235033	975	SAT1_CF	blue	na
chrUn	4235090	4235698	609	SAT1_CF	blue	na
chrUn	4235709	4236109	401	SAT1_CF	blue	na
chrUn	4236148	4236650	503	SAT1_CF	blue	na
chrUn	4236663	4238217	1555	SAT1_CF	blue	na
chrUn	4238259	4238803	545	SAT1_CF	blue	na
chrUn	4238819	4240538	1720	SAT1_CF	blue	na
chrUn	4240561	4240996	436	SAT1_CF	blue	na
chrUn	4241010	4241117	108	SAT1_CF	blue	na
chrUn	4241139	4241419	281	SAT1_CF	blue	na
chrUn	4241602	4241881	280	SAT1_CF	blue	na
chrUn	4241894	4243740	1847	SAT1_CF	blue	na
chrUn	4243784	4244286	503	SAT1_CF	blue	na
chrUn	4244297	4244972	676	SAT1_CF	blue	na
chrUn	4245015	4245740	726	SAT1_CF	blue	na
chrUn	4245785	4246320	536	SAT1_CF	blue	na
chrUn	4246342	4246859	518	SAT1_CF	blue	na
chrUn	4246871	4248187	1317	SAT1_CF	blue	na
chrUn	4248200	4248602	403	SAT1_CF	blue	na
chrUn	4248642	4249144	503	SAT1_CF	blue	na
chrUn	4249157	4250527	1371	SAT1_CF	blue	na
chrUn	4250570	4251534	965	SAT1_CF	blue	na
chrUn	4251650	4252563	914	SAT1_CF	blue	na
chrUn	4252631	4253150	520	SAT1_CF	blue	na
chrUn	4253163	4253846	684	SAT1_CF	blue	na
chrUn	4253858	4254520	663	SAT1_CF	blue	na
chrUn	4254563	4255190	628	SAT1_CF	blue	na
chrUn	4255218	4256423	1206	SAT1_CF	blue	na
chrUn	4256438	4256574	137	SAT1_CF	blue	na

chrUn	4256621	4257137	517	SAT1_CF	blue	na
chrUn	4257150	4257833	684	SAT1_CF	blue	na
chrUn	4257845	4258526	682	SAT1_CF	blue	na
chrUn	4258540	4259216	677	SAT1_CF	blue	na
chrUn	4259260	4259834	575	SAT1_CF	blue	na
chrUn	4259923	4260420	498	SAT1_CF	blue	na
chrUn	4260433	4261184	752	SAT1_CF	blue	na
chrUn	4261207	4262419	1213	SAT1_CF	blue	na
chrUn	4262445	4263242	798	SAT1_CF	blue	na
chrUn	4263422	4263603	182	SAT1_CF	blue	na
chrUn	4263642	4264142	501	SAT1_CF	blue	na
chrUn	4264155	4265519	1365	SAT1_CF	blue	na
chrUn	4265562	4266527	966	SAT1_CF	blue	na
chrUn	4266574	4267720	1147	SAT1_CF	blue	na
chrUn	4267743	4268267	525	SAT1_CF	blue	na
chrUn	4268324	4270879	2556	SAT1_CF	blue	na
chrUn	4270988	4271487	500	SAT1_CF	blue	na
chrUn	4271505	4272174	670	SAT1_CF	blue	na
chrUn	4272244	4272707	464	SAT1_CF	blue	na
chrUn	4272719	4273536	818	SAT1_CF	blue	na
chrUn	4273554	4273657	104	SAT1_CF	blue	na
chrUn	4273678	4274233	556	SAT1_CF	blue	na
chrUn	4274245	4277093	2849	SAT1_CF	blue	na
chrUn	4277148	4277640	493	SAT1_CF	blue	na
chrUn	4277674	4278334	661	SAT1_CF	blue	na
chrUn	4278377	4280402	2026	SAT1_CF	blue	na
chrUn	4280419	4281720	1302	SAT1_CF	blue	na
chrUn	4281760	4282261	502	SAT1_CF	blue	na
chrUn	4282274	4282957	684	SAT1_CF	blue	na
chrUn	4282969	4283693	725	SAT1_CF	blue	na
chrUn	4283704	4284196	493	SAT1_CF	blue	na
chrUn	4284210	4286418	2209	SAT1_CF	blue	na
chrUn	4287833	4288405	573	SAT1_CF	blue	na
chrUn	4288445	4288938	494	SAT1_CF	blue	na
chrUn	4288958	4289640	683	SAT1_CF	blue	na
chrUn	4289652	4290329	678	SAT1_CF	blue	na
chrUn	4290371	4291336	966	SAT1_CF	blue	na
chrUn	4291415	4292328	914	SAT1_CF	blue	na
chrUn	4292415	4292917	503	SAT1_CF	blue	na
chrUn	4292944	4293034	91	SAT1_CF	blue	na
chrUn	4293058	4295764	2707	SAT1_CF	blue	na
chrUn	4295807	4296307	501	SAT1_CF	blue	na

chrUn	4296325	4297004	680	SAT1_CF	blue	na
chrUn	4297016	4297125	110	SAT1_CF	blue	na
chrUn	4297145	4297689	545	SAT1_CF	blue	na
chrUn	4297733	4298842	1110	SAT1_CF	blue	na
chrUn	4298887	4299768	882	SAT1_CF	blue	na
chrUn	4299808	4300926	1119	SAT1_CF	blue	na
chrUn	4300937	4301014	78	SAT1_CF	blue	na
chrUn	4301033	4301585	553	SAT1_CF	blue	na
chrUn	4301620	4302186	567	SAT1_CF	blue	na
chrUn	4302224	4302718	495	SAT1_CF	blue	na
chrUn	4302738	4303855	1118	SAT1_CF	blue	na
chrUn	4303869	4305237	1369	SAT1_CF	blue	na
chrUn	4305283	4306643	1361	SAT1_CF	blue	na
chrUn	4306672	4307193	522	SAT1_CF	blue	na
chrUn	4307206	4307909	704	SAT1_CF	blue	na
chrUn	4307921	4308597	677	SAT1_CF	blue	na
chrUn	4308645	4309602	958	SAT1_CF	blue	na
chrUn	4309681	4310628	948	SAT1_CF	blue	na
chrUn	4310681	4311543	863	SAT1_CF	blue	na
chrUn	4311569	4312050	482	SAT1_CF	blue	na
chrUn	4312063	4312735	673	SAT1_CF	blue	na
chrUn	4312747	4312914	168	SAT1_CF	blue	na
chrUn	4312927	4312967	41	SAT1_CF	blue	na
chrUn	4312999	4314078	1080	SAT1_CF	blue	na
chrUn	4314121	4314675	555	SAT1_CF	blue	na
chrUn	4314687	4315461	775	SAT1_CF	blue	na
chrUn	4315479	4315949	471	SAT1_CF	blue	na
chrUn	4315983	4316503	521	SAT1_CF	blue	na
chrUn	4316516	4318695	2180	SAT1_CF	blue	na
chrUn	4318738	4319244	507	SAT1_CF	blue	na
chrUn	4319277	4319413	137	SAT1_CF	blue	na
chrUn	4319460	4320669	1210	SAT1_CF	blue	na
chrUn	4320739	4321372	634	SAT1_CF	blue	na
chrUn	4321384	4322060	677	SAT1_CF	blue	na
chrUn	4322078	4322719	642	SAT1_CF	blue	na
chrUn	4322762	4323694	933	SAT1_CF	blue	na
chrUn	4323705	4324105	401	SAT1_CF	blue	na
chrUn	4324145	4324647	503	SAT1_CF	blue	na
chrUn	4324661	4325420	760	SAT1_CF	blue	na
chrUn	4325432	4326983	1552	SAT1_CF	blue	na
chrUn	4327041	4328365	1325	SAT1_CF	blue	na
chrUn	4328376	4328916	541	SAT1_CF	blue	na

chrUn	4328930	4329612	683	SAT1_CF	blue	na
chrUn	4329624	4330303	680	SAT1_CF	blue	na
chrUn	4330314	4330916	603	SAT1_CF	blue	na
chrUn	4330928	4332155	1228	SAT1_CF	blue	na
chrUn	4332173	4332859	687	SAT1_CF	blue	na
chrUn	4332871	4333719	849	SAT1_CF	blue	na
chrUn	4333732	4333774	43	SAT1_CF	blue	na
chrUn	4333804	4335577	1774	SAT1_CF	blue	na
chrUn	4335664	4336164	501	SAT1_CF	blue	na
chrUn	4336191	4336936	746	SAT1_CF	blue	na
chrUn	4336948	4338155	1208	SAT1_CF	blue	na
chrUn	4338626	4339236	611	SAT1_CF	blue	na
chrUn	4339247	4339647	401	SAT1_CF	blue	na
chrUn	4339687	4341530	1844	SAT1_CF	blue	na
chrUn	4341576	4342135	560	SAT1_CF	blue	na
chrUn	4342219	4342766	548	SAT1_CF	blue	na
chrUn	4342794	4342930	137	SAT1_CF	blue	na
chrUn	4342996	4343497	502	SAT1_CF	blue	na
chrUn	4343520	4344191	672	SAT1_CF	blue	na
chrUn	4344203	4344312	110	SAT1_CF	blue	na
chrUn	4344333	4344916	584	SAT1_CF	blue	na
chrUn	4344945	4345488	544	SAT1_CF	blue	na
chrUn	4345533	4346338	806	SAT1_CF	blue	na
chrUn	4346425	4347388	964	SAT1_CF	blue	na
chrUn	4347416	4347551	136	SAT1_CF	blue	na
chrUn	4347598	4348082	485	SAT1_CF	blue	na
chrUn	4348095	4348769	675	SAT1_CF	blue	na
chrUn	4348787	4348954	168	SAT1_CF	blue	na
chrUn	4348996	4349681	686	SAT1_CF	blue	na
chrUn	4349709	4350806	1098	SAT1_CF	blue	na
chrUn	4350913	4351392	480	SAT1_CF	blue	na
chrUn	4351410	4352595	1186	SAT1_CF	blue	na
chrUn	4352662	4353029	368	SAT1_CF	blue	na
chrUn	4353225	4353356	132	SAT1_CF	blue	na
chrUn	4353400	4353922	523	SAT1_CF	blue	na
chrUn	4353938	4356441	2504	SAT1_CF	blue	na
chrUn	4356453	4358007	1555	SAT1_CF	blue	na
chrUn	4358030	4360263	2234	SAT1_CF	blue	na
chrUn	4360276	4360883	608	SAT1_CF	blue	na
chrUn	4360929	4361107	179	SAT1_CF	blue	na
chrUn	4361155	4361677	523	SAT1_CF	blue	na
chrUn	4361759	4361937	179	SAT1_CF	blue	na

chrUn	4361998	4363200	1203	SAT1_CF	blue	na
chrUn	4363243	4363874	632	SAT1_CF	blue	na
chrUn	4363902	4364963	1062	SAT1_CF	blue	na
chrUn	4365050	4365551	502	SAT1_CF	blue	na
chrUn	4365564	4365670	107	SAT1_CF	blue	na
chrUn	4365693	4366916	1224	SAT1_CF	blue	na
chrUn	4366980	4367136	157	SAT1_CF	blue	na
chrUn	4367181	4367682	502	SAT1_CF	blue	na
chrUn	4367700	4367764	65	SAT1_CF	blue	na
chrUn	4367789	4368516	728	SAT1_CF	blue	na
chrUn	4368558	4370365	1808	SAT1_CF	blue	na
chrUn	4370454	4370954	501	SAT1_CF	blue	na
chrUn	4370967	4373950	2984	SAT1_CF	blue	na
chrUn	4373962	4376147	2186	SAT1_CF	blue	na
chrUn	4376160	4376839	680	SAT1_CF	blue	na
chrUn	4376850	4377520	671	SAT1_CF	blue	na
chrUn	4377563	4378194	632	SAT1_CF	blue	na
chrUn	4378222	4378786	565	SAT1_CF	blue	na
chrUn	4378828	4379144	317	SAT1_CF	blue	na
chrUn	4379172	4379656	485	SAT1_CF	blue	na
chrUn	4379917	4380946	1030	SAT1_CF	blue	na
chrUn	4380976	4381757	782	SAT1_CF	blue	na
chrUn	4381789	4382795	1007	SAT1_CF	blue	na
chrUn	4382833	4383334	502	SAT1_CF	blue	na
chrUn	4383356	4384107	752	SAT1_CF	blue	na
chrUn	4384120	4385241	1122	SAT1_CF	blue	na
chrUn	4385320	4387323	2004	SAT1_CF	blue	na
chrUn	4387334	4387983	650	SAT1_CF	blue	na
chrUn	4388026	4388993	968	SAT1_CF	blue	na
chrUn	4389026	4389402	377	SAT1_CF	blue	na
chrUn	4389445	4389947	503	SAT1_CF	blue	na
chrUn	4389960	4390951	992	SAT1_CF	blue	na
chrUn	4390963	4392101	1139	SAT1_CF	blue	na
chrUn	4392115	4392636	522	SAT1_CF	blue	na
chrUn	4392702	4392838	137	SAT1_CF	blue	na
chrUn	4392905	4393404	500	SAT1_CF	blue	na
chrUn	4393417	4394738	1322	SAT1_CF	blue	na
chrUn	4394753	4395454	702	SAT1_CF	blue	na
chrUn	4395466	4395573	108	SAT1_CF	blue	na
chrUn	4395594	4396148	555	SAT1_CF	blue	na
chrUn	4396161	4396827	667	SAT1_CF	blue	na
chrUn	4396870	4397850	981	SAT1_CF	blue	na

chrUn	4397866	4398051	186	SAT1_CF	blue	na
chrUn	4398065	4399055	991	SAT1_CF	blue	na
chrUn	4399083	4399240	158	SAT1_CF	blue	na
chrUn	4399285	4399787	503	SAT1_CF	blue	na
chrUn	4399800	4399876	77	SAT1_CF	blue	na
chrUn	4399929	4400967	1039	SAT1_CF	blue	na
chrUn	4400981	4401118	138	SAT1_CF	blue	na
chrUn	4401214	4402347	1134	SAT1_CF	blue	na
chrUn	4402359	4403544	1186	SAT1_CF	blue	na
chrUn	4403558	4403912	355	SAT1_CF	blue	na
chrUn	4404018	4404586	569	SAT1_CF	blue	na
chrUn	4404607	4405953	1347	SAT1_CF	blue	na
chrUn	4405995	4407970	1976	SAT1_CF	blue	na
chrUn	4408013	4408551	539	SAT1_CF	blue	na
chrUn	4408633	4409196	564	SAT1_CF	blue	na
chrUn	4409274	4409900	627	SAT1_CF	blue	na
chrUn	4409912	4410585	674	SAT1_CF	blue	na
chrUn	4410628	4412666	2039	SAT1_CF	blue	na
chrUn	4412706	4413201	496	SAT1_CF	blue	na
chrUn	4413220	4413902	683	SAT1_CF	blue	na
chrUn	4413914	4414583	670	SAT1_CF	blue	na
chrUn	4414626	4415582	957	SAT1_CF	blue	na
chrUn	4415649	4416682	1034	SAT1_CF	blue	na
chrUn	4416716	4417236	521	SAT1_CF	blue	na
chrUn	4417263	4417892	630	SAT1_CF	blue	na
chrUn	4417905	4418546	642	SAT1_CF	blue	na
chrUn	4418591	4419798	1208	SAT1_CF	blue	na
chrUn	4419809	4420506	698	SAT1_CF	blue	na
chrUn	4420518	4421628	1111	SAT1_CF	blue	na
chrUn	4421642	4422325	684	SAT1_CF	blue	na
chrUn	4422337	4423014	678	SAT1_CF	blue	na
chrUn	4423056	4424036	981	SAT1_CF	blue	na
chrUn	4424115	4425111	997	SAT1_CF	blue	na
chrUn	4425151	4426345	1195	SAT1_CF	blue	na
chrUn	4426356	4426848	493	SAT1_CF	blue	na
chrUn	4426862	4427352	491	SAT1_CF	blue	na
chrUn	4427366	4429992	2627	SAT1_CF	blue	na
chrUn	4431561	4432089	529	SAT1_CF	blue	na
chrUn	4432157	4432676	520	SAT1_CF	blue	na
chrUn	4432689	4432797	109	SAT1_CF	blue	na
chrUn	4432818	4433363	546	SAT1_CF	blue	na
chrUn	4433406	4434406	1001	SAT1_CF	blue	na

chrUn	4434450	4435949	1500	SAT1_CF	blue	na
chrUn	4435962	4437329	1368	SAT1_CF	blue	na
chrUn	4437410	4437774	365	SAT1_CF	blue	na
chrUn	4437816	4438316	501	SAT1_CF	blue	na
chrUn	4438328	4439010	683	SAT1_CF	blue	na
chrUn	4439029	4439934	906	SAT1_CF	blue	na
chrUn	4439977	4440566	590	SAT1_CF	blue	na
chrUn	4440585	4441128	544	SAT1_CF	blue	na
chrUn	4441142	4441787	646	SAT1_CF	blue	na
chrUn	4441831	4442409	579	SAT1_CF	blue	na
chrUn	4442488	4443404	917	SAT1_CF	blue	na
chrUn	4443416	4443518	103	SAT1_CF	blue	na
chrUn	4443547	4444091	545	SAT1_CF	blue	na
chrUn	4444136	4445102	967	SAT1_CF	blue	na
chrUn	4445157	4445828	672	SAT1_CF	blue	na
chrUn	4445839	4447112	1274	SAT1_CF	blue	na
chrUn	4447153	4448709	1557	SAT1_CF	blue	na
chrUn	4448722	4449392	671	SAT1_CF	blue	na
chrUn	4449436	4449996	561	SAT1_CF	blue	na
chrUn	4450014	4451145	1132	SAT1_CF	blue	na
chrUn	4451185	4451686	502	SAT1_CF	blue	na
chrUn	4451699	4452300	602	SAT1_CF	blue	na
chrUn	4452312	4452987	676	SAT1_CF	blue	na
chrUn	4453036	4453833	798	SAT1_CF	blue	na
chrUn	4453847	4454844	998	SAT1_CF	blue	na
chrUn	4454859	4454995	137	SAT1_CF	blue	na
chrUn	4455042	4455561	520	SAT1_CF	blue	na
chrUn	4455574	4455681	108	SAT1_CF	blue	na
chrUn	4455703	4456247	545	SAT1_CF	blue	na
chrUn	4456290	4457918	1629	SAT1_CF	blue	na
chrUn	4457929	4458117	189	SAT1_CF	blue	na
chrUn	4458169	4458652	484	SAT1_CF	blue	na
chrUn	4458664	4459336	673	SAT1_CF	blue	na
chrUn	4459454	4459959	506	SAT1_CF	blue	na
chrUn	4459992	4460692	701	SAT1_CF	blue	na
chrUn	4460705	4461387	683	SAT1_CF	blue	na
chrUn	4461399	4462039	641	SAT1_CF	blue	na
chrUn	4462051	4463312	1262	SAT1_CF	blue	na
chrUn	4463340	4463723	384	SAT1_CF	blue	na
chrUn	4463744	4464264	521	SAT1_CF	blue	na
chrUn	4464276	4464385	110	SAT1_CF	blue	na
chrUn	4464406	4465725	1320	SAT1_CF	blue	na

chrUn	4465768	4466311	544	SAT1_CF	blue	na
chrUn	4466333	4466429	97	SAT1_CF	blue	na
chrUn	4466450	4467004	555	SAT1_CF	blue	na
chrUn	4467016	4468352	1337	SAT1_CF	blue	na
chrUn	4468377	4468699	323	SAT1_CF	blue	na
chrUn	4468770	4469287	518	SAT1_CF	blue	na
chrUn	4469352	4469910	559	SAT1_CF	blue	na
chrUn	4469922	4471785	1864	SAT1_CF	blue	na
chrUn	4471816	4472341	526	SAT1_CF	blue	na
chrUn	4472356	4472490	135	SAT1_CF	blue	na
chrUn	4472558	4473059	502	SAT1_CF	blue	na
chrUn	4473073	4474291	1219	SAT1_CF	blue	na
chrUn	4474334	4475739	1406	SAT1_CF	blue	na
chrUn	4475782	4476762	981	SAT1_CF	blue	na
chrUn	4476774	4477459	686	SAT1_CF	blue	na
chrUn	4477471	4478155	685	SAT1_CF	blue	na
chrUn	4478168	4480209	2042	SAT1_CF	blue	na
chrUn	4480221	4480302	82	SAT1_CF	blue	na
chrUn	4480316	4480859	544	SAT1_CF	blue	na
chrUn	4481120	4481818	699	SAT1_CF	blue	na
chrUn	4481830	4482671	842	SAT1_CF	blue	na
chrUn	4482713	4484564	1852	SAT1_CF	blue	na
chrUn	4484629	4486012	1384	SAT1_CF	blue	na
chrUn	4486058	4486375	318	SAT1_CF	blue	na
chrUn	4486424	4486930	507	SAT1_CF	blue	na
chrUn	4486948	4487051	104	SAT1_CF	blue	na
chrUn	4487072	4487645	574	SAT1_CF	blue	na
chrUn	4487657	4489785	2129	SAT1_CF	blue	na
chrUn	4489866	4490030	165	SAT1_CF	blue	na
chrUn	4490210	4490393	184	SAT1_CF	blue	na
chrUn	4490430	4490929	500	SAT1_CF	blue	na
chrUn	4490942	4491334	393	SAT1_CF	blue	na
chrUn	4491419	4491963	545	SAT1_CF	blue	na
chrUn	4491975	4492831	857	SAT1_CF	blue	na
chrUn	4492873	4494726	1854	SAT1_CF	blue	na
chrUn	4494747	4495261	515	SAT1_CF	blue	na
chrUn	4495278	4496106	829	SAT1_CF	blue	na
chrUn	4496119	4496794	676	SAT1_CF	blue	na
chrUn	4496842	4498873	2032	SAT1_CF	blue	na
chrUn	4498932	4500186	1255	SAT1_CF	blue	na
chrUn	4500198	4500307	110	SAT1_CF	blue	na
chrUn	4500327	4500873	547	SAT1_CF	blue	na

chrUn	4500916	4502779	1864	SAT1_CF	blue	na
chrUn	4502794	4502950	157	SAT1_CF	blue	na
chrUn	4502976	4504923	1948	SAT1_CF	blue	na
chrUn	4504935	4506259	1325	SAT1_CF	blue	na
chrUn	4506304	4506789	486	SAT1_CF	blue	na
chrUn	4506843	4506998	156	SAT1_CF	blue	na
chrUn	4507024	4507544	521	SAT1_CF	blue	na
chrUn	4507556	4507664	109	SAT1_CF	blue	na
chrUn	4507687	4508342	656	SAT1_CF	blue	na
chrUn	4508391	4510079	1689	SAT1_CF	blue	na
chrUn	4510091	4510197	107	SAT1_CF	blue	na
chrUn	4510218	4510982	765	SAT1_CF	blue	na
chrUn	4510995	4512462	1468	SAT1_CF	blue	na
chrUn	4512490	4513008	519	SAT1_CF	blue	na
chrUn	4513022	4514337	1316	SAT1_CF	blue	na
chrUn	4514382	4515073	692	SAT1_CF	blue	na
chrUn	4515105	4515193	89	SAT1_CF	blue	na
chrUn	4515213	4516209	997	SAT1_CF	blue	na
chrUn	4516221	4516897	677	SAT1_CF	blue	na
chrUn	4516940	4518398	1459	SAT1_CF	blue	na
chrUn	4519812	4520919	1108	SAT1_CF	blue	na
chrUn	4520939	4522281	1343	SAT1_CF	blue	na
chrUn	4522323	4524115	1793	SAT1_CF	blue	na
chrUn	4524139	4525717	1579	SAT1_CF	blue	na
chrUn	4525730	4527285	1556	SAT1_CF	blue	na
chrUn	4527327	4528805	1479	SAT1_CF	blue	na
chrUn	4528816	4529750	935	SAT1_CF	blue	na
chrUn	4529784	4530451	668	SAT1_CF	blue	na
chrUn	4530494	4531756	1263	SAT1_CF	blue	na
chrUn	4531789	4532165	377	SAT1_CF	blue	na
chrUn	4532211	4532709	499	SAT1_CF	blue	na
chrUn	4532721	4534012	1292	SAT1_CF	blue	na
chrUn	4534054	4535421	1368	SAT1_CF	blue	na
chrUn	4535469	4535946	478	SAT1_CF	blue	na
chrUn	4535993	4536494	502	SAT1_CF	blue	na
chrUn	4536507	4537189	683	SAT1_CF	blue	na
chrUn	4537213	4538061	849	SAT1_CF	blue	na
chrUn	4538103	4538637	535	SAT1_CF	blue	na
chrUn	4538660	4539309	650	SAT1_CF	blue	na
chrUn	4539353	4539699	347	SAT1_CF	blue	na
chrUn	4539720	4540301	582	SAT1_CF	blue	na
chrUn	4540313	4540990	678	SAT1_CF	blue	na

chrUn	4541033	4542459	1427	SAT1_CF	blue	na
chrUn	4542475	4543566	1092	SAT1_CF	blue	na
chrUn	4544575	4544664	90	SAT1_CF	blue	na
chrUn	4551042	4551587	546	SAT1_CF	blue	na
chrUn	4551651	4552449	799	SAT1_CF	blue	na
chrUn	4552471	4553418	948	SAT1_CF	blue	na
chrUn	4553435	4554472	1038	SAT1_CF	blue	na
chrUn	4554485	4555494	1010	SAT1_CF	blue	na
chrUn	4555502	4555867	366	CarSat1	red	na
chrUn	4556017	4557047	1031	CarSat1	red	na
chrUn	4557086	4557851	766	CarSat1	red	na
chrUn	4557870	4558396	527	CarSat1	red	na
chrUn	4558786	4558904	119	Sat2CF	purple	na
chrUn	4558803	4559970	1168	Sat2CF	purple	na
chrUn	4579049	4579211	163	Sat2CF	purple	na
chrUn	4579077	4579434	358	Sat2CF	purple	na
chrUn	4579116	4579944	829	Sat2CF	purple	na
chrUn	4579049	4579211	163	Sat2CF	purple	na
chrUn	4594740	4595549	810	Sat2CF	purple	na
chrUn	4594804	4595527	724	Sat2CF	purple	na
chrUn	4595418	4595586	169	Sat2CF	purple	na
chrUn	4609013	4609344	332	Sat2CF	purple	na
chrUn	4609055	4609568	514	Sat2CF	purple	na
chrUn	4609440	4609616	177	Sat2CF	purple	na
chrUn	4621707	4622847	1141	Sat2CF	purple	na
chrUn	4621815	4622799	985	Sat2CF	purple	na
chrUn	4633043	4633604	562	Sat2CF	purple	na
chrUn	4648995	4649815	821	Sat2CF	purple	na
chrUn	4649069	4649793	725	Sat2CF	purple	na
chrUn	4649484	4649852	369	Sat2CF	purple	na
chrUn	4662607	4663858	1252	Sat2CF	purple	na
chrUn	4662737	4663822	1086	Sat2CF	purple	na
chrUn	4666448	4666588	141	CarSat1	red	na
chrUn	4666609	4668225	1617	CarSat1	red	na
chrUn	4674796	4676007	1212	SAT1_CF	blue	na
chrUn	4676029	4676582	554	SAT1_CF	blue	na
chrUn	4676632	4677810	1179	SAT1_CF	blue	na
chrUn	4677846	4678168	323	SAT1_CF	blue	na
chrUn	4678184	4680082	1899	SAT1_CF	blue	na
chrUn	4680124	4681458	1335	SAT1_CF	blue	na
chrUn	4681479	4681626	148	SAT1_CF	blue	na
chrUn	4681638	4682113	476	SAT1_CF	blue	na

chrUn	4682183	4682706	524	SAT1_CF	blue	na
chrUn	4682734	4683281	548	SAT1_CF	blue	na
chrUn	4683324	4683872	549	SAT1_CF	blue	na
chrUn	4683893	4684001	109	SAT1_CF	blue	na
chrUn	4684014	4684513	500	SAT1_CF	blue	na
chrUn	4684552	4685514	963	SAT1_CF	blue	na
chrUn	4685630	4686401	772	SAT1_CF	blue	na
chrUn	4686454	4688412	1959	SAT1_CF	blue	na
chrUn	4688425	4688923	499	SAT1_CF	blue	na
chrUn	4688977	4689959	983	SAT1_CF	blue	na
chrUn	4690038	4691002	965	SAT1_CF	blue	na
chrUn	4691045	4691720	676	SAT1_CF	blue	na
chrUn	4691732	4692765	1034	SAT1_CF	blue	na
chrUn	4692805	4693175	371	SAT1_CF	blue	na
chrUn	4693214	4693762	549	SAT1_CF	blue	na
chrUn	4693806	4694805	1000	SAT1_CF	blue	na
chrUn	4694848	4695624	777	SAT1_CF	blue	na
chrUn	4695673	4696350	678	SAT1_CF	blue	na
chrUn	4696362	4697041	680	SAT1_CF	blue	na
chrUn	4697054	4697541	488	SAT1_CF	blue	na
chrUn	4697647	4699099	1453	SAT1_CF	blue	na
chrUn	4699142	4699816	675	SAT1_CF	blue	na
chrUn	4699828	4700903	1076	SAT1_CF	blue	na
chrUn	4700952	4701499	548	SAT1_CF	blue	na
chrUn	4701556	4702579	1024	SAT1_CF	blue	na
chrUn	4702607	4703241	635	SAT1_CF	blue	na
chrUn	4703267	4703424	158	SAT1_CF	blue	na
chrUn	4703452	4703960	509	SAT1_CF	blue	na
chrUn	4704028	4704838	811	SAT1_CF	blue	na
chrUn	4704850	4705527	678	SAT1_CF	blue	na
chrUn	4705545	4706229	685	SAT1_CF	blue	na
chrUn	4706241	4707794	1554	SAT1_CF	blue	na
chrUn	4707820	4708870	1051	SAT1_CF	blue	na
chrUn	4708883	4710077	1195	SAT1_CF	blue	na
chrUn	4710144	4710261	118	SAT1_CF	blue	na
chrUn	4710313	4710997	685	SAT1_CF	blue	na
chrUn	4711046	4711593	548	SAT1_CF	blue	na
chrUn	4711617	4711723	107	SAT1_CF	blue	na
chrUn	4711735	4712403	669	SAT1_CF	blue	na
chrUn	4712430	4712911	482	SAT1_CF	blue	na
chrUn	4712966	4713250	285	SAT1_CF	blue	na
chrUn	4713272	4713953	682	SAT1_CF	blue	na

chrUn	4713966	4714652	687	SAT1_CF	blue	na
chrUn	4714664	4715288	625	SAT1_CF	blue	na
chrUn	4715315	4715837	523	SAT1_CF	blue	na
chrUn	4715855	4718373	2519	SAT1_CF	blue	na
chrUn	4718420	4719095	676	SAT1_CF	blue	na
chrUn	4719107	4719661	555	SAT1_CF	blue	na
chrUn	4719686	4719790	105	SAT1_CF	blue	na
chrUn	4719803	4720305	503	SAT1_CF	blue	na
chrUn	4720371	4720507	137	SAT1_CF	blue	na
chrUn	4720572	4721094	523	SAT1_CF	blue	na
chrUn	4721108	4722246	1139	SAT1_CF	blue	na
chrUn	4722258	4723251	994	SAT1_CF	blue	na
chrUn	4723264	4723763	500	SAT1_CF	blue	na
chrUn	4723816	4725844	2029	SAT1_CF	blue	na
chrUn	4725887	4726559	673	SAT1_CF	blue	na
chrUn	4726572	4727340	769	SAT1_CF	blue	na
chrUn	4727358	4727860	503	SAT1_CF	blue	na
chrUn	4727920	4728062	143	SAT1_CF	blue	na
chrUn	4728095	4728601	507	SAT1_CF	blue	na
chrUn	4728631	4729296	666	SAT1_CF	blue	na
chrUn	4729338	4730161	824	SAT1_CF	blue	na
chrUn	4730173	4730751	579	SAT1_CF	blue	na
chrUn	4730772	4730879	108	SAT1_CF	blue	na
chrUn	4730892	4731594	703	SAT1_CF	blue	na
chrUn	4731609	4732822	1214	SAT1_CF	blue	na
chrUn	4732835	4733335	501	SAT1_CF	blue	na
chrUn	4733375	4734272	898	SAT1_CF	blue	na
chrUn	4734304	4735117	814	SAT1_CF	blue	na
chrUn	4735159	4735843	685	SAT1_CF	blue	na
chrUn	4735856	4737077	1222	SAT1_CF	blue	na
chrUn	4737120	4739024	1905	SAT1_CF	blue	na
chrUn	4739037	4739519	483	SAT1_CF	blue	na
chrUn	4739582	4740536	955	SAT1_CF	blue	na
chrUn	4740608	4741143	536	SAT1_CF	blue	na
chrUn	4741187	4741774	588	SAT1_CF	blue	na
chrUn	4741820	4743551	1732	SAT1_CF	blue	na
chrUn	4743563	4744563	1001	SAT1_CF	blue	na
chrUn	4744629	4745024	396	SAT1_CF	blue	na
chrUn	4745037	4746517	1481	SAT1_CF	blue	na
chrUn	4746559	4746690	132	SAT1_CF	blue	na
chrUn	4746709	4747378	670	SAT1_CF	blue	na
chrUn	4747391	4748095	705	SAT1_CF	blue	na

chrUn	4748123	4749352	1230	SAT1_CF	blue	na
chrUn	4749380	4750011	632	SAT1_CF	blue	na
chrUn	4750054	4750727	674	SAT1_CF	blue	na
chrUn	4750739	4751421	683	SAT1_CF	blue	na
chrUn	4751434	4751926	493	SAT1_CF	blue	na
chrUn	4751971	4752339	369	SAT1_CF	blue	na
chrUn	4752380	4752887	508	SAT1_CF	blue	na
chrUn	4752966	4753435	470	SAT1_CF	blue	na
chrUn	4753450	4754158	709	SAT1_CF	blue	na
chrUn	4754202	4754728	527	SAT1_CF	blue	na
chrUn	4754759	4754940	182	SAT1_CF	blue	na
chrUn	4755119	4755964	846	SAT1_CF	blue	na
chrUn	4756006	4756179	174	SAT1_CF	blue	na
chrUn	4756191	4756866	676	SAT1_CF	blue	na
chrUn	4756878	4757553	676	SAT1_CF	blue	na
chrUn	4757575	4758129	555	SAT1_CF	blue	na
chrUn	4758151	4758258	108	SAT1_CF	blue	na
chrUn	4758271	4759162	892	SAT1_CF	blue	na
chrUn	4759174	4759842	669	SAT1_CF	blue	na
chrUn	4759869	4760369	501	SAT1_CF	blue	na
chrUn	4760415	4760810	396	SAT1_CF	blue	na
chrUn	4760823	4761369	547	SAT1_CF	blue	na
chrUn	4761393	4762288	896	SAT1_CF	blue	na
chrUn	4762300	4762874	575	SAT1_CF	blue	na
chrUn	4762897	4763003	107	SAT1_CF	blue	na
chrUn	4763016	4763946	931	SAT1_CF	blue	na
chrUn	4763958	4764519	562	SAT1_CF	blue	na
chrUn	4764546	4764646	101	SAT1_CF	blue	na
chrUn	4764663	4765289	627	SAT1_CF	blue	na
chrUn	4765302	4766062	761	SAT1_CF	blue	na
chrUn	4766075	4766668	594	SAT1_CF	blue	na
chrUn	4766691	4767211	521	SAT1_CF	blue	na
chrUn	4767300	4767762	463	SAT1_CF	blue	na
chrUn	4767779	4768787	1009	SAT1_CF	blue	na
chrUn	4768837	4769512	676	SAT1_CF	blue	na
chrUn	4769524	4770079	556	SAT1_CF	blue	na
chrUn	4770100	4770196	97	SAT1_CF	blue	na
chrUn	4770218	4771957	1740	SAT1_CF	blue	na
chrUn	4771969	4772727	759	SAT1_CF	blue	na
chrUn	4772747	4773258	512	SAT1_CF	blue	na
chrUn	4773279	4774237	959	SAT1_CF	blue	na
chrUn	4774281	4775097	817	SAT1_CF	blue	na

chrUn	4775139	4775852	714	SAT1_CF	blue	na
chrUn	4775922	4776596	675	SAT1_CF	blue	na
chrUn	4776607	4777756	1150	SAT1_CF	blue	na
chrUn	4777785	4778294	510	SAT1_CF	blue	na
chrUn	4778337	4778900	564	SAT1_CF	blue	na
chrUn	4778928	4781491	2564	SAT1_CF	blue	na
chrUn	4781512	4782516	1005	SAT1_CF	blue	na
chrUn	4782597	4783136	540	SAT1_CF	blue	na
chrUn	4783179	4783727	549	SAT1_CF	blue	na
chrUn	4783797	4784479	683	SAT1_CF	blue	na
chrUn	4784493	4784992	500	SAT1_CF	blue	na
chrUn	4785038	4786204	1167	SAT1_CF	blue	na
chrUn	4786247	4786370	124	SAT1_CF	blue	na
chrUn	4786383	4787209	827	SAT1_CF	blue	na
chrUn	4787234	4789055	1822	SAT1_CF	blue	na
chrUn	4789125	4789797	673	SAT1_CF	blue	na
chrUn	4789810	4790292	483	SAT1_CF	blue	na
chrUn	4790306	4790609	304	SAT1_CF	blue	na
chrUn	4790653	4791748	1096	SAT1_CF	blue	na
chrUn	4791792	4793103	1312	SAT1_CF	blue	na
chrUn	4793143	4795182	2040	SAT1_CF	blue	na
chrUn	4795225	4795900	676	SAT1_CF	blue	na
chrUn	4795913	4796395	483	SAT1_CF	blue	na
chrUn	4796421	4797438	1018	SAT1_CF	blue	na
chrUn	4797454	4798461	1008	SAT1_CF	blue	na
chrUn	4798503	4799178	676	SAT1_CF	blue	na
chrUn	4799192	4799658	467	SAT1_CF	blue	na
chrUn	4799679	4799788	110	SAT1_CF	blue	na
chrUn	4799800	4800483	684	SAT1_CF	blue	na
chrUn	4800495	4802500	2006	SAT1_CF	blue	na
chrUn	4802547	4803221	675	SAT1_CF	blue	na
chrUn	4803233	4803787	555	SAT1_CF	blue	na
chrUn	4803814	4803916	103	SAT1_CF	blue	na
chrUn	4803929	4804430	502	SAT1_CF	blue	na
chrUn	4804496	4804632	137	SAT1_CF	blue	na
chrUn	4804699	4805218	520	SAT1_CF	blue	na
chrUn	4805232	4806904	1673	SAT1_CF	blue	na
chrUn	4806929	4807550	622	SAT1_CF	blue	na
chrUn	4807561	4808059	499	SAT1_CF	blue	na
chrUn	4808112	4808277	166	SAT1_CF	blue	na
chrUn	4808451	4809243	793	SAT1_CF	blue	na
chrUn	4809287	4809777	491	SAT1_CF	blue	na

chrUn	4809823	4810359	537	SAT1_CF	blue	na
chrUn	4810402	4810949	548	SAT1_CF	blue	na
chrUn	4810970	4811568	599	SAT1_CF	blue	na
chrUn	4811591	4811699	109	SAT1_CF	blue	na
chrUn	4811710	4812212	503	SAT1_CF	blue	na
chrUn	4812248	4813275	1028	SAT1_CF	blue	na
chrUn	4813291	4813925	635	SAT1_CF	blue	na
chrUn	4813969	4814513	545	SAT1_CF	blue	na
chrUn	4814556	4815232	677	SAT1_CF	blue	na
chrUn	4815244	4815835	592	SAT1_CF	blue	na
chrUn	4815862	4816361	500	SAT1_CF	blue	na
chrUn	4816406	4816563	158	SAT1_CF	blue	na
chrUn	4816578	4818254	1677	SAT1_CF	blue	na
chrUn	4818296	4820299	2004	SAT1_CF	blue	na
chrUn	4820339	4820740	402	SAT1_CF	blue	na
chrUn	4820754	4822368	1615	SAT1_CF	blue	na
chrUn	4822410	4823330	921	SAT1_CF	blue	na
chrUn	4823357	4824038	682	SAT1_CF	blue	na
chrUn	4824050	4824601	552	SAT1_CF	blue	na
chrUn	4824645	4825188	544	SAT1_CF	blue	na
chrUn	4825231	4825780	550	SAT1_CF	blue	na
chrUn	4825858	4826627	770	SAT1_CF	blue	na
chrUn	4826679	4827540	862	SAT1_CF	blue	na
chrUn	4827552	4829480	1929	SAT1_CF	blue	na
chrUn	4829523	4830196	674	SAT1_CF	blue	na
chrUn	4830208	4830896	689	SAT1_CF	blue	na
chrUn	4830909	4831777	869	SAT1_CF	blue	na
chrUn	4831824	4832500	677	SAT1_CF	blue	na
chrUn	4832512	4833894	1383	SAT1_CF	blue	na
chrUn	4833906	4835612	1707	SAT1_CF	blue	na
chrUn	4835625	4836123	499	SAT1_CF	blue	na
chrUn	4836165	4837120	956	SAT1_CF	blue	na
chrUn	4837164	4837836	673	SAT1_CF	blue	na
chrUn	4837848	4838444	597	SAT1_CF	blue	na
chrUn	4838466	4838984	519	SAT1_CF	blue	na
chrUn	4839003	4839263	261	SAT1_CF	blue	na
chrUn	4839307	4839854	548	SAT1_CF	blue	na
chrUn	4839868	4840856	989	SAT1_CF	blue	na
chrUn	4840898	4842308	1411	SAT1_CF	blue	na
chrUn	4842329	4843148	820	SAT1_CF	blue	na
chrUn	4843163	4844377	1215	SAT1_CF	blue	na
chrUn	4844419	4844898	480	SAT1_CF	blue	na

chrUn	4844960	4845920	961	SAT1_CF	blue	na
chrUn	4845999	4846757	759	SAT1_CF	blue	na
chrUn	4846776	4847898	1123	SAT1_CF	blue	na
chrUn	4847957	4848635	679	SAT1_CF	blue	na
chrUn	4848656	4849408	753	SAT1_CF	blue	na
chrUn	4849452	4849991	540	SAT1_CF	blue	na
chrUn	4850034	4850558	525	SAT1_CF	blue	na
chrUn	4850576	4850859	284	SAT1_CF	blue	na
chrUn	4850901	4851445	545	SAT1_CF	blue	na
chrUn	4851473	4852375	903	SAT1_CF	blue	na
chrUn	4852393	4852894	502	SAT1_CF	blue	na
chrUn	4852934	4853335	402	SAT1_CF	blue	na
chrUn	4853347	4855610	2264	SAT1_CF	blue	na
chrUn	4855642	4855823	182	SAT1_CF	blue	na
chrUn	4856003	4856374	372	SAT1_CF	blue	na
chrUn	4856402	4857032	631	SAT1_CF	blue	na
chrUn	4857075	4857748	674	SAT1_CF	blue	na
chrUn	4857759	4858361	603	SAT1_CF	blue	na
chrUn	4858374	4858855	482	SAT1_CF	blue	na
chrUn	4858910	4859315	406	SAT1_CF	blue	na
chrUn	4859329	4859835	507	SAT1_CF	blue	na
chrUn	4859866	4860697	832	SAT1_CF	blue	na
chrUn	4860758	4861435	678	SAT1_CF	blue	na
chrUn	4861447	4862019	573	SAT1_CF	blue	na
chrUn	4862042	4862148	107	SAT1_CF	blue	na
chrUn	4862162	4863490	1329	SAT1_CF	blue	na
chrUn	4863555	4865302	1748	SAT1_CF	blue	na
chrUn	4865314	4866307	994	SAT1_CF	blue	na
chrUn	4866320	4866820	501	SAT1_CF	blue	na
chrUn	4866860	4868893	2034	SAT1_CF	blue	na
chrUn	4868936	4869613	678	SAT1_CF	blue	na
chrUn	4869625	4870098	474	SAT1_CF	blue	na
chrUn	4870168	4870840	673	SAT1_CF	blue	na
chrUn	4870853	4871335	483	SAT1_CF	blue	na
chrUn	4871394	4872292	899	SAT1_CF	blue	na
chrUn	4872359	4873011	653	SAT1_CF	blue	na
chrUn	4873033	4873577	545	SAT1_CF	blue	na
chrUn	4873620	4874714	1095	SAT1_CF	blue	na
chrUn	4874737	4874843	107	SAT1_CF	blue	na
chrUn	4874856	4875356	501	SAT1_CF	blue	na
chrUn	4875402	4876354	953	SAT1_CF	blue	na
chrUn	4876389	4876961	573	SAT1_CF	blue	na

chrUn	4877005	4877784	780	SAT1_CF	blue	na
chrUn	4877826	4878668	843	SAT1_CF	blue	na
chrUn	4878680	4879361	682	SAT1_CF	blue	na
chrUn	4879374	4880532	1159	SAT1_CF	blue	na
chrUn	4880602	4881146	545	SAT1_CF	blue	na
chrUn	4881204	4881961	758	SAT1_CF	blue	na
chrUn	4881982	4882304	323	SAT1_CF	blue	na
chrUn	4882379	4883038	660	SAT1_CF	blue	na
chrUn	4883065	4883567	503	SAT1_CF	blue	na
chrUn	4883653	4884847	1195	SAT1_CF	blue	na
chrUn	4884858	4885334	477	SAT1_CF	blue	na
chrUn	4885394	4885793	400	SAT1_CF	blue	na
chrUn	4885805	4887251	1447	SAT1_CF	blue	na
chrUn	4887293	4888154	862	SAT1_CF	blue	na
chrUn	4888167	4888927	761	SAT1_CF	blue	na
chrUn	4888940	4889442	503	SAT1_CF	blue	na
chrUn	4889482	4890571	1090	SAT1_CF	blue	na
chrUn	4890583	4891254	672	SAT1_CF	blue	na
chrUn	4891265	4891766	502	SAT1_CF	blue	na
chrUn	4891806	4893370	1565	SAT1_CF	blue	na
chrUn	4893395	4893479	85	SAT1_CF	blue	na
chrUn	4893523	4894066	544	SAT1_CF	blue	na
chrUn	4894109	4894786	678	SAT1_CF	blue	na
chrUn	4894798	4895389	592	SAT1_CF	blue	na
chrUn	4895416	4895916	501	SAT1_CF	blue	na
chrUn	4895929	4897422	1494	SAT1_CF	blue	na
chrUn	4897469	4897626	158	SAT1_CF	blue	na
chrUn	4897654	4898166	513	SAT1_CF	blue	na
chrUn	4898246	4899282	1037	SAT1_CF	blue	na
chrUn	4899325	4899915	591	SAT1_CF	blue	na
chrUn	4899927	4900604	678	SAT1_CF	blue	na
chrUn	4900616	4901170	555	SAT1_CF	blue	na
chrUn	4901195	4901299	105	SAT1_CF	blue	na
chrUn	4901312	4902526	1215	SAT1_CF	blue	na
chrUn	4902540	4903677	1138	SAT1_CF	blue	na
chrUn	4903689	4904680	992	SAT1_CF	blue	na
chrUn	4904693	4905194	502	SAT1_CF	blue	na
chrUn	4905234	4906255	1022	SAT1_CF	blue	na
chrUn	4906310	4907274	965	SAT1_CF	blue	na
chrUn	4907318	4908138	821	SAT1_CF	blue	na
chrUn	4908188	4909809	1622	SAT1_CF	blue	na
chrUn	4909871	4910455	585	SAT1_CF	blue	na

chrUn	4910490	4910671	182	SAT1_CF	blue	na
chrUn	4910855	4911090	236	SAT1_CF	blue	na
chrUn	4911102	4911649	548	SAT1_CF	blue	na
chrUn	4911728	4912849	1122	SAT1_CF	blue	na
chrUn	4912862	4913338	477	SAT1_CF	blue	na
chrUn	4913408	4914081	674	SAT1_CF	blue	na
chrUn	4914093	4915091	999	SAT1_CF	blue	na
chrUn	4915152	4915553	402	SAT1_CF	blue	na
chrUn	4915566	4917884	2319	SAT1_CF	blue	na
chrUn	4917897	4918399	503	SAT1_CF	blue	na
chrUn	4918487	4919434	948	SAT1_CF	blue	na
chrUn	4919512	4920475	964	SAT1_CF	blue	na
chrUn	4920518	4921194	677	SAT1_CF	blue	na
chrUn	4921206	4921889	684	SAT1_CF	blue	na
chrUn	4921902	4922404	503	SAT1_CF	blue	na
chrUn	4922444	4922846	403	SAT1_CF	blue	na
chrUn	4922859	4923403	545	SAT1_CF	blue	na
chrUn	4923431	4924060	630	SAT1_CF	blue	na
chrUn	4924105	4924778	674	SAT1_CF	blue	na
chrUn	4924792	4925289	498	SAT1_CF	blue	na
chrUn	4925329	4927183	1855	SAT1_CF	blue	na
chrUn	4927224	4928616	1393	SAT1_CF	blue	na
chrUn	4928668	4930273	1606	SAT1_CF	blue	na
chrUn	4930349	4933154	2806	SAT1_CF	blue	na
chrUn	4933167	4933671	505	SAT1_CF	blue	na
chrUn	4933719	4934037	319	SAT1_CF	blue	na
chrUn	4934049	4936860	2812	SAT1_CF	blue	na
chrUn	4936883	4936989	107	SAT1_CF	blue	na
chrUn	4937002	4937499	498	SAT1_CF	blue	na
chrUn	4937567	4937703	137	SAT1_CF	blue	na
chrUn	4937731	4938922	1192	SAT1_CF	blue	na
chrUn	4938960	4940913	1954	SAT1_CF	blue	na
chrUn	4940936	4941042	107	SAT1_CF	blue	na
chrUn	4941055	4941557	503	SAT1_CF	blue	na
chrUn	4941602	4941757	156	SAT1_CF	blue	na
chrUn	4941821	4943156	1336	SAT1_CF	blue	na
chrUn	4943186	4943687	502	SAT1_CF	blue	na
chrUn	4943732	4943889	158	SAT1_CF	blue	na
chrUn	4943917	4944489	573	SAT1_CF	blue	na
chrUn	4944533	4945120	588	SAT1_CF	blue	na
chrUn	4945166	4946021	856	SAT1_CF	blue	na
chrUn	4946034	4946536	503	SAT1_CF	blue	na

chrUn	4946550	4946866	317	SAT1_CF	blue	na
chrUn	4946878	4947958	1081	SAT1_CF	blue	na
chrUn	4947986	4948617	632	SAT1_CF	blue	na
chrUn	4948660	4949335	676	SAT1_CF	blue	na
chrUn	4949348	4949812	465	SAT1_CF	blue	na
chrUn	4949857	4950876	1020	SAT1_CF	blue	na
chrUn	4950892	4951894	1003	SAT1_CF	blue	na
chrUn	4951941	4952613	673	SAT1_CF	blue	na
chrUn	4952625	4953179	555	SAT1_CF	blue	na
chrUn	4953204	4953308	105	SAT1_CF	blue	na
chrUn	4953321	4953822	502	SAT1_CF	blue	na
chrUn	4953888	4954024	137	SAT1_CF	blue	na
chrUn	4954089	4954612	524	SAT1_CF	blue	na
chrUn	4954626	4956452	1827	SAT1_CF	blue	na
chrUn	4956479	4956978	500	SAT1_CF	blue	na
chrUn	4957023	4957180	158	SAT1_CF	blue	na
chrUn	4957213	4958503	1291	SAT1_CF	blue	na
chrUn	4958527	4959615	1089	SAT1_CF	blue	na
chrUn	4959627	4960101	475	SAT1_CF	blue	na
chrUn	4960123	4960230	108	SAT1_CF	blue	na
chrUn	4960243	4960744	502	SAT1_CF	blue	na
chrUn	4960784	4961227	444	SAT1_CF	blue	na
chrUn	4961270	4961819	550	SAT1_CF	blue	na
chrUn	4961896	4962743	848	SAT1_CF	blue	na
chrUn	4962767	4963446	680	SAT1_CF	blue	na
chrUn	4963460	4964359	900	SAT1_CF	blue	na
chrUn	4964386	4964925	540	SAT1_CF	blue	na
chrUn	4964992	4965482	491	SAT1_CF	blue	na
chrUn	4965493	4967025	1533	SAT1_CF	blue	na
chrUn	4967048	4967842	795	SAT1_CF	blue	na
chrUn	4967885	4968501	617	SAT1_CF	blue	na
chrUn	4968515	4969332	818	SAT1_CF	blue	na
chrUn	4969374	4970234	861	SAT1_CF	blue	na
chrUn	4970246	4970800	555	SAT1_CF	blue	na
chrUn	4970821	4970923	103	SAT1_CF	blue	na
chrUn	4970941	4971871	931	SAT1_CF	blue	na
chrUn	4971913	4972849	937	SAT1_CF	blue	na
chrUn	4972867	4974663	1797	SAT1_CF	blue	na
chrUn	4974681	4975276	596	SAT1_CF	blue	na
chrUn	4975298	4975819	522	SAT1_CF	blue	na
chrUn	4975840	4976242	403	SAT1_CF	blue	na
chrUn	4976255	4976775	521	SAT1_CF	blue	na

chrUn	4976793	4977220	428	SAT1_CF	blue	na
chrUn	4977264	4977909	646	SAT1_CF	blue	na
chrUn	4977921	4978595	675	SAT1_CF	blue	na
chrUn	4978609	4980200	1592	SAT1_CF	blue	na
chrUn	4980242	4980360	119	SAT1_CF	blue	na
chrUn	4980407	4982391	1985	SAT1_CF	blue	na
chrUn	4982434	4982977	544	SAT1_CF	blue	na
chrUn	4983020	4984462	1443	SAT1_CF	blue	na
chrUn	4984504	4985364	861	SAT1_CF	blue	na
chrUn	4985376	4985935	560	SAT1_CF	blue	na
chrUn	4985957	4986064	108	SAT1_CF	blue	na
chrUn	4986077	4986945	869	SAT1_CF	blue	na
chrUn	4986992	4987727	736	SAT1_CF	blue	na
chrUn	4987754	4988432	679	SAT1_CF	blue	na
chrUn	4988450	4989149	700	SAT1_CF	blue	na
chrUn	4989162	4989707	546	SAT1_CF	blue	na
chrUn	4989751	4990181	431	SAT1_CF	blue	na
chrUn	4990218	4991700	1483	SAT1_CF	blue	na
chrUn	4991718	4992220	503	SAT1_CF	blue	na
chrUn	4992256	4993098	843	SAT1_CF	blue	na
chrUn	4993143	4993299	157	SAT1_CF	blue	na
chrUn	4993364	4993928	565	SAT1_CF	blue	na
chrUn	4993974	4994608	635	SAT1_CF	blue	na
chrUn	4994620	4995256	637	SAT1_CF	blue	na
chrUn	4995268	4995846	579	SAT1_CF	blue	na
chrUn	4995867	4995974	108	SAT1_CF	blue	na
chrUn	4995987	4996506	520	SAT1_CF	blue	na
chrUn	4996545	4996875	331	SAT1_CF	blue	na
chrUn	4996889	4997968	1080	SAT1_CF	blue	na
chrUn	4998027	4998656	630	SAT1_CF	blue	na
chrUn	4998699	4999243	545	SAT1_CF	blue	na
chrUn	4999300	4999965	666	SAT1_CF	blue	na
chrUn	4999978	5000480	503	SAT1_CF	blue	na
chrUn	5000520	5000922	403	SAT1_CF	blue	na
chrUn	5000933	5001455	523	SAT1_CF	blue	na
chrUn	5001473	5001901	429	SAT1_CF	blue	na
chrUn	5001945	5003675	1731	SAT1_CF	blue	na
chrUn	5003745	5004427	683	SAT1_CF	blue	na
chrUn	5004440	5004939	500	SAT1_CF	blue	na
chrUn	5004985	5006544	1560	SAT1_CF	blue	na
chrUn	5006588	5007209	622	SAT1_CF	blue	na
chrUn	5007222	5008602	1381	SAT1_CF	blue	na

chrUn	5008645	5009098	454	SAT1_CF	blue	na
chrUn	5009168	5009712	545	SAT1_CF	blue	na
chrUn	5009769	5010421	653	SAT1_CF	blue	na
chrUn	5010448	5011149	702	SAT1_CF	blue	na
chrUn	5011177	5011688	512	SAT1_CF	blue	na
chrUn	5011731	5012954	1224	SAT1_CF	blue	na
chrUn	5012966	5013597	632	SAT1_CF	blue	na
chrUn	5013627	5013794	168	SAT1_CF	blue	na
chrUn	5014054	5015872	1819	SAT1_CF	blue	na
chrUn	5015884	5016568	685	SAT1_CF	blue	na
chrUn	5016581	5017709	1129	SAT1_CF	blue	na
chrUn	5017722	5018204	483	SAT1_CF	blue	na
chrUn	5018263	5019224	962	SAT1_CF	blue	na
chrUn	5019268	5020242	975	SAT1_CF	blue	na
chrUn	5020291	5020968	678	SAT1_CF	blue	na
chrUn	5020980	5021661	682	SAT1_CF	blue	na
chrUn	5021674	5022175	502	SAT1_CF	blue	na
chrUn	5022262	5022767	506	SAT1_CF	blue	na
chrUn	5022807	5023352	546	SAT1_CF	blue	na
chrUn	5023379	5024326	948	SAT1_CF	blue	na
chrUn	5024370	5024730	361	SAT1_CF	blue	na
chrUn	5024755	5026088	1334	SAT1_CF	blue	na
chrUn	5026100	5026782	683	SAT1_CF	blue	na
chrUn	5026795	5029012	2218	SAT1_CF	blue	na
chrUn	5029024	5029596	573	SAT1_CF	blue	na
chrUn	5029619	5029725	107	SAT1_CF	blue	na
chrUn	5029739	5030835	1097	SAT1_CF	blue	na
chrUn	5030908	5032945	2038	SAT1_CF	blue	na
chrUn	5032988	5033661	674	SAT1_CF	blue	na
chrUn	5033673	5034155	483	SAT1_CF	blue	na
chrUn	5034175	5034329	155	SAT1_CF	blue	na
chrUn	5034390	5035067	678	SAT1_CF	blue	na
chrUn	5035079	5035844	766	SAT1_CF	blue	na
chrUn	5035858	5036530	673	SAT1_CF	blue	na
chrUn	5036552	5037095	544	SAT1_CF	blue	na
chrUn	5037140	5037683	544	SAT1_CF	blue	na
chrUn	5037740	5038276	537	SAT1_CF	blue	na
chrUn	5038299	5038405	107	SAT1_CF	blue	na
chrUn	5038418	5038517	100	SAT1_CF	blue	na
chrUn	5038529	5039082	554	SAT1_CF	blue	na
chrUn	5039105	5039211	107	SAT1_CF	blue	na
chrUn	5039224	5039724	501	SAT1_CF	blue	na

chrUn	5039760	5039945	186	SAT1_CF	blue	na
chrUn	5040126	5040290	165	SAT1_CF	blue	na
chrUn	5040371	5040919	549	SAT1_CF	blue	na
chrUn	5041014	5042166	1153	SAT1_CF	blue	na
chrUn	5042208	5042787	580	SAT1_CF	blue	na
chrUn	5042808	5042907	100	SAT1_CF	blue	na
chrUn	5042929	5043472	544	SAT1_CF	blue	na
chrUn	5043515	5044700	1186	SAT1_CF	blue	na
chrUn	5044719	5045217	499	SAT1_CF	blue	na
chrUn	5045303	5047285	1983	SAT1_CF	blue	na
chrUn	5047328	5048004	677	SAT1_CF	blue	na
chrUn	5048016	5048697	682	SAT1_CF	blue	na
chrUn	5048710	5049210	501	SAT1_CF	blue	na
chrUn	5049246	5051785	2540	SAT1_CF	blue	na
chrUn	5051797	5052550	754	SAT1_CF	blue	na
chrUn	5052572	5053093	522	SAT1_CF	blue	na
chrUn	5053160	5054310	1151	SAT1_CF	blue	na
chrUn	5054323	5054843	521	SAT1_CF	blue	na
chrUn	5054869	5055025	157	SAT1_CF	blue	na
chrUn	5055087	5055634	548	SAT1_CF	blue	na
chrUn	5055676	5056428	753	SAT1_CF	blue	na
chrUn	5056450	5056971	522	SAT1_CF	blue	na
chrUn	5056992	5057394	403	SAT1_CF	blue	na
chrUn	5057431	5057693	263	SAT1_CF	blue	na
chrUn	5057736	5058299	564	SAT1_CF	blue	na
chrUn	5058343	5059676	1334	SAT1_CF	blue	na
chrUn	5059689	5060241	553	SAT1_CF	blue	na
chrUn	5060264	5060370	107	SAT1_CF	blue	na
chrUn	5060383	5061245	863	SAT1_CF	blue	na
chrUn	5061257	5061865	609	SAT1_CF	blue	na
chrUn	5061878	5062534	657	SAT1_CF	blue	na
chrUn	5062568	5063073	506	SAT1_CF	blue	na
chrUn	5063116	5064945	1830	SAT1_CF	blue	na
chrUn	5064957	5065431	475	SAT1_CF	blue	na
chrUn	5065453	5065560	108	SAT1_CF	blue	na
chrUn	5065573	5066074	502	SAT1_CF	blue	na
chrUn	5066114	5066557	444	SAT1_CF	blue	na
chrUn	5066600	5067274	675	SAT1_CF	blue	na
chrUn	5067292	5067845	554	SAT1_CF	blue	na
chrUn	5067866	5067975	110	SAT1_CF	blue	na
chrUn	5067987	5068488	502	SAT1_CF	blue	na
chrUn	5068533	5068688	156	SAT1_CF	blue	na

chrUn	5068704	5069903	1200	SAT1_CF	blue	na
chrUn	5069947	5071970	2024	SAT1_CF	blue	na
chrUn	5071984	5072504	521	SAT1_CF	blue	na
chrUn	5072530	5074099	1570	SAT1_CF	blue	na
chrUn	5074112	5074615	504	SAT1_CF	blue	na
chrUn	5074655	5075616	962	SAT1_CF	blue	na
chrUn	5075675	5076475	801	SAT1_CF	blue	na
chrUn	5076517	5076997	481	SAT1_CF	blue	na
chrUn	5077056	5077967	912	SAT1_CF	blue	na
chrUn	5077994	5078548	555	SAT1_CF	blue	na
chrUn	5078588	5079512	925	SAT1_CF	blue	na
chrUn	5079530	5081445	1916	SAT1_CF	blue	na
chrUn	5081516	5082150	635	SAT1_CF	blue	na
chrUn	5082163	5082879	717	SAT1_CF	blue	na
chrUn	5082894	5083695	802	SAT1_CF	blue	na
chrUn	5083738	5086087	2350	SAT1_CF	blue	na
chrUn	5086099	5086651	553	SAT1_CF	blue	na
chrUn	5086672	5086774	103	SAT1_CF	blue	na
chrUn	5086792	5087345	554	SAT1_CF	blue	na
chrUn	5087486	5089401	1916	SAT1_CF	blue	na
chrUn	5089419	5090322	904	SAT1_CF	blue	na
chrUn	5090364	5090536	173	SAT1_CF	blue	na
chrUn	5090548	5091222	675	SAT1_CF	blue	na
chrUn	5091236	5092718	1483	SAT1_CF	blue	na
chrUn	5092768	5093730	963	SAT1_CF	blue	na
chrUn	5093743	5094265	523	SAT1_CF	blue	na
chrUn	5094297	5094805	509	SAT1_CF	blue	na
chrUn	5094885	5095921	1037	SAT1_CF	blue	na
chrUn	5095963	5096594	632	SAT1_CF	blue	na
chrUn	5096617	5096723	107	SAT1_CF	blue	na
chrUn	5096736	5097937	1202	SAT1_CF	blue	na
chrUn	5097968	5098439	472	SAT1_CF	blue	na
chrUn	5098475	5098659	185	SAT1_CF	blue	na
chrUn	5098841	5099043	203	SAT1_CF	blue	na
chrUn	5099086	5099724	639	SAT1_CF	blue	na
chrUn	5099737	5100239	503	SAT1_CF	blue	na
chrUn	5100305	5100440	136	SAT1_CF	blue	na
chrUn	5100455	5102776	2322	SAT1_CF	blue	na
chrUn	5102794	5103296	503	SAT1_CF	blue	na
chrUn	5103336	5104265	930	SAT1_CF	blue	na
chrUn	5104299	5104817	519	SAT1_CF	blue	na
chrUn	5104830	5105367	538	SAT1_CF	blue	na

chrUn	5105410	5106086	677	SAT1_CF	blue	na
chrUn	5106098	5106652	555	SAT1_CF	blue	na
chrUn	5106674	5106779	106	SAT1_CF	blue	na
chrUn	5106792	5107291	500	SAT1_CF	blue	na
chrUn	5107327	5108340	1014	SAT1_CF	blue	na
chrUn	5108444	5109118	675	SAT1_CF	blue	na
chrUn	5109139	5109613	475	SAT1_CF	blue	na
chrUn	5109646	5109962	317	SAT1_CF	blue	na
chrUn	5109974	5110521	548	SAT1_CF	blue	na
chrUn	5110544	5110650	107	SAT1_CF	blue	na
chrUn	5110670	5111226	557	SAT1_CF	blue	na
chrUn	5112864	5113768	905	SAT1_CF	blue	na
chrUn	5113802	5114797	996	SAT1_CF	blue	na
chrUn	5114841	5116176	1336	SAT1_CF	blue	na
chrUn	5116188	5116708	521	SAT1_CF	blue	na
chrUn	5116753	5116888	136	SAT1_CF	blue	na
chrUn	5116903	5118173	1271	SAT1_CF	blue	na
chrUn	5118196	5118301	106	SAT1_CF	blue	na
chrUn	5118314	5118843	530	SAT1_CF	blue	na
chrUn	5118883	5119064	182	SAT1_CF	blue	na
chrUn	5119246	5120358	1113	SAT1_CF	blue	na
chrUn	5120396	5120940	545	SAT1_CF	blue	na
chrUn	5120981	5121841	861	SAT1_CF	blue	na
chrUn	5121855	5122862	1008	SAT1_CF	blue	na
chrUn	5122911	5123455	545	SAT1_CF	blue	na
chrUn	5123476	5123585	110	SAT1_CF	blue	na
chrUn	5123597	5124225	629	SAT1_CF	blue	na
chrUn	5124285	5124806	522	SAT1_CF	blue	na
chrUn	5124849	5125887	1039	SAT1_CF	blue	na
chrUn	5125929	5126720	792	SAT1_CF	blue	na
chrUn	5126743	5126849	107	SAT1_CF	blue	na
chrUn	5126862	5127382	521	SAT1_CF	blue	na
chrUn	5127408	5127547	140	SAT1_CF	blue	na
chrUn	5127593	5128101	509	SAT1_CF	blue	na
chrUn	5128119	5128778	660	SAT1_CF	blue	na
chrUn	5128822	5130152	1331	SAT1_CF	blue	na
chrUn	5130166	5130925	760	SAT1_CF	blue	na
chrUn	5130937	5131618	682	SAT1_CF	blue	na
chrUn	5131631	5132131	501	SAT1_CF	blue	na
chrUn	5132171	5133315	1145	SAT1_CF	blue	na
chrUn	5133329	5134029	701	SAT1_CF	blue	na
chrUn	5134071	5134930	860	SAT1_CF	blue	na

chrUn	5134942	5135609	668	SAT1_CF	blue	na
chrUn	5135636	5136138	503	SAT1_CF	blue	na
chrUn	5136191	5136510	320	SAT1_CF	blue	na
chrUn	5136593	5139156	2564	SAT1_CF	blue	na
chrUn	5139170	5139690	521	SAT1_CF	blue	na
chrUn	5139759	5140070	312	SAT1_CF	blue	na
chrUn	5140084	5140940	857	SAT1_CF	blue	na
chrUn	5140987	5141529	543	SAT1_CF	blue	na
chrUn	5141571	5142429	859	SAT1_CF	blue	na
chrUn	5142441	5143087	647	SAT1_CF	blue	na
chrUn	5143100	5143862	763	SAT1_CF	blue	na
chrUn	5143874	5144374	501	SAT1_CF	blue	na
chrUn	5144414	5145592	1179	SAT1_CF	blue	na
chrUn	5145658	5146325	668	SAT1_CF	blue	na
chrUn	5146367	5146714	348	SAT1_CF	blue	na
chrUn	5146752	5147296	545	SAT1_CF	blue	na
chrUn	5147346	5148692	1347	SAT1_CF	blue	na
chrUn	5148704	5149386	683	SAT1_CF	blue	na
chrUn	5149399	5149900	502	SAT1_CF	blue	na
chrUn	5149940	5150342	403	SAT1_CF	blue	na
chrUn	5150356	5150901	546	SAT1_CF	blue	na
chrUn	5150944	5151626	683	SAT1_CF	blue	na
chrUn	5151639	5152121	483	SAT1_CF	blue	na
chrUn	5152227	5152581	355	SAT1_CF	blue	na
chrUn	5152592	5154024	1433	SAT1_CF	blue	na
chrUn	5154077	5154804	728	SAT1_CF	blue	na
chrUn	5154821	5154934	114	SAT1_CF	blue	na
chrUn	5154946	5155792	847	SAT1_CF	blue	na
chrUn	5155823	5156372	550	SAT1_CF	blue	na
chrUn	5156451	5156776	326	SAT1_CF	blue	na
chrUn	5156801	5157447	647	SAT1_CF	blue	na
chrUn	5157459	5158134	676	SAT1_CF	blue	na
chrUn	5158146	5158627	482	SAT1_CF	blue	na
chrUn	5158687	5159085	399	SAT1_CF	blue	na
chrUn	5159097	5160285	1189	SAT1_CF	blue	na
chrUn	5160316	5160836	521	SAT1_CF	blue	na
chrUn	5160953	5161955	1003	SAT1_CF	blue	na
chrUn	5161981	5162917	937	SAT1_CF	blue	na
chrUn	5162966	5163639	674	SAT1_CF	blue	na
chrUn	5163652	5164154	503	SAT1_CF	blue	na
chrUn	5164242	5164595	354	SAT1_CF	blue	na
chrUn	5164608	5165117	510	SAT1_CF	blue	na

chrUn	5165196	5165948	753	SAT1_CF	blue	na
chrUn	5165969	5166670	702	SAT1_CF	blue	na
chrUn	5166697	5168451	1755	SAT1_CF	blue	na
chrUn	5168474	5168579	106	SAT1_CF	blue	na
chrUn	5168592	5169094	503	SAT1_CF	blue	na
chrUn	5169107	5169614	508	SAT1_CF	blue	na
chrUn	5169730	5170274	545	SAT1_CF	blue	na
chrUn	5170288	5171170	883	SAT1_CF	blue	na
chrUn	5171213	5171878	666	SAT1_CF	blue	na
chrUn	5171900	5172450	551	SAT1_CF	blue	na
chrUn	5172493	5173055	563	SAT1_CF	blue	na
chrUn	5173120	5174484	1365	SAT1_CF	blue	na
chrUn	5174498	5174990	493	SAT1_CF	blue	na
chrUn	5175001	5175551	551	SAT1_CF	blue	na
chrUn	5175611	5175788	178	SAT1_CF	blue	na
chrUn	5175830	5177343	1514	SAT1_CF	blue	na
chrUn	5177365	5177867	503	SAT1_CF	blue	na
chrUn	5177933	5178069	137	SAT1_CF	blue	na
chrUn	5178084	5179289	1206	SAT1_CF	blue	na
chrUn	5179317	5179946	630	SAT1_CF	blue	na
chrUn	5179989	5182056	2068	SAT1_CF	blue	na
chrUn	5182076	5182550	475	SAT1_CF	blue	na
chrUn	5182611	5183182	572	SAT1_CF	blue	na
chrUn	5184572	5185158	587	SAT1_CF	blue	na
chrUn	5185172	5186055	884	SAT1_CF	blue	na
chrUn	5186098	5186700	603	SAT1_CF	blue	na
chrUn	5186711	5187308	598	SAT1_CF	blue	na
chrUn	5187330	5187850	521	SAT1_CF	blue	na
chrUn	5187876	5187984	109	SAT1_CF	blue	na
chrUn	5187995	5189123	1129	SAT1_CF	blue	na
chrUn	5189151	5189690	540	SAT1_CF	blue	na
chrUn	5189733	5190409	677	SAT1_CF	blue	na
chrUn	5190421	5191104	684	SAT1_CF	blue	na
chrUn	5191117	5191619	503	SAT1_CF	blue	na
chrUn	5191657	5192223	567	SAT1_CF	blue	na
chrUn	5192258	5192810	553	SAT1_CF	blue	na
chrUn	5192829	5193860	1032	SAT1_CF	blue	na
chrUn	5193907	5194454	548	SAT1_CF	blue	na
chrUn	5194533	5195513	981	SAT1_CF	blue	na
chrUn	5195555	5196230	676	SAT1_CF	blue	na
chrUn	5196242	5197360	1119	SAT1_CF	blue	na
chrUn	5197400	5197973	574	SAT1_CF	blue	na

chrUn	5199358	5199723	366	SAT1_CF	blue	na
chrUn	5199766	5201104	1339	SAT1_CF	blue	na
chrUn	5201157	5204569	3413	SAT1_CF	blue	na
chrUn	5204582	5205097	516	SAT1_CF	blue	na
chrUn	5205144	5205859	716	SAT1_CF	blue	na
chrUn	5205938	5206712	775	SAT1_CF	blue	na
chrUn	5206755	5206890	136	SAT1_CF	blue	na
chrUn	5206903	5207565	663	SAT1_CF	blue	na
chrUn	5207592	5208294	703	SAT1_CF	blue	na
chrUn	5208309	5210197	1889	SAT1_CF	blue	na
chrUn	5210241	5211976	1736	SAT1_CF	blue	na
chrUn	5211992	5212534	543	SAT1_CF	blue	na
chrUn	5212576	5213475	900	SAT1_CF	blue	na
chrUn	5213487	5214040	554	SAT1_CF	blue	na
chrUn	5214064	5214154	91	SAT1_CF	blue	na
chrUn	5214181	5214702	522	SAT1_CF	blue	na
chrUn	5214721	5214980	260	SAT1_CF	blue	na
chrUn	5215024	5216432	1409	SAT1_CF	blue	na
chrUn	5216451	5217021	571	SAT1_CF	blue	na
chrUn	5217086	5217243	158	SAT1_CF	blue	na
chrUn	5217294	5218254	961	SAT1_CF	blue	na
chrUn	5218292	5219832	1541	SAT1_CF	blue	na
chrUn	5219856	5220538	683	SAT1_CF	blue	na
chrUn	5220551	5222798	2248	SAT1_CF	blue	na
chrUn	5222826	5223271	446	SAT1_CF	blue	na
chrUn	5223285	5224046	762	SAT1_CF	blue	na
chrUn	5224059	5224761	703	SAT1_CF	blue	na
chrUn	5224776	5225301	526	SAT1_CF	blue	na
chrUn	5225332	5225869	538	SAT1_CF	blue	na
chrUn	5225913	5226635	723	SAT1_CF	blue	na
chrUn	5226678	5227338	661	SAT1_CF	blue	na
chrUn	5227351	5227899	549	SAT1_CF	blue	na
chrUn	5227943	5228283	341	SAT1_CF	blue	na
chrUn	5228327	5230431	2105	SAT1_CF	blue	na
chrUn	5230444	5230963	520	SAT1_CF	blue	na
chrUn	5231031	5233052	2022	SAT1_CF	blue	na
chrUn	5233065	5233839	775	SAT1_CF	blue	na
chrUn	5233852	5234352	501	SAT1_CF	blue	na
chrUn	5234412	5234554	143	SAT1_CF	blue	na
chrUn	5234569	5235315	747	SAT1_CF	blue	na
chrUn	5235376	5236708	1333	SAT1_CF	blue	na
chrUn	5236720	5237238	519	SAT1_CF	blue	na

chrUn	5237270	5237552	283	SAT1_CF	blue	na
chrUn	5237594	5238137	544	SAT1_CF	blue	na
chrUn	5238179	5238726	548	SAT1_CF	blue	na
chrUn	5238783	5239302	520	SAT1_CF	blue	na
chrUn	5239397	5239534	138	SAT1_CF	blue	na
chrUn	5239560	5240617	1058	SAT1_CF	blue	na
chrUn	5240648	5241452	805	SAT1_CF	blue	na
chrUn	5241500	5242804	1305	SAT1_CF	blue	na
chrUn	5242826	5242980	155	SAT1_CF	blue	na
chrUn	5243041	5243718	678	SAT1_CF	blue	na
chrUn	5243730	5244413	684	SAT1_CF	blue	na
chrUn	5244425	5245335	911	SAT1_CF	blue	na
chrUn	5245378	5246752	1375	SAT1_CF	blue	na
chrUn	5246764	5247283	520	SAT1_CF	blue	na
chrUn	5247309	5247688	380	SAT1_CF	blue	na
chrUn	5247711	5247816	106	SAT1_CF	blue	na
chrUn	5247829	5248348	520	SAT1_CF	blue	na
chrUn	5248395	5248531	137	SAT1_CF	blue	na
chrUn	5248559	5249070	512	SAT1_CF	blue	na
chrUn	5249149	5251188	2040	SAT1_CF	blue	na
chrUn	5251218	5252277	1060	SAT1_CF	blue	na
chrUn	5252298	5252407	110	SAT1_CF	blue	na
chrUn	5252419	5252921	503	SAT1_CF	blue	na
chrUn	5252961	5254453	1493	SAT1_CF	blue	na
chrUn	5254475	5254999	525	SAT1_CF	blue	na
chrUn	5255042	5255709	668	SAT1_CF	blue	na
chrUn	5255731	5257194	1464	SAT1_CF	blue	na
chrUn	5257310	5258276	967	SAT1_CF	blue	na
chrUn	5258319	5258987	669	SAT1_CF	blue	na
chrUn	5259007	5259519	513	SAT1_CF	blue	na
chrUn	5259538	5260470	933	SAT1_CF	blue	na
chrUn	5260491	5261401	911	SAT1_CF	blue	na
chrUn	5261413	5262616	1204	SAT1_CF	blue	na
chrUn	5262661	5262818	158	SAT1_CF	blue	na
chrUn	5262864	5264004	1141	SAT1_CF	blue	na
chrUn	5264090	5264935	846	SAT1_CF	blue	na
chrUn	5264948	5265502	555	SAT1_CF	blue	na
chrUn	5265566	5265630	65	SAT1_CF	blue	na
chrUn	5265650	5266420	771	SAT1_CF	blue	na
chrUn	5266485	5267158	674	SAT1_CF	blue	na
chrUn	5267171	5267671	501	SAT1_CF	blue	na
chrUn	5267716	5267872	157	SAT1_CF	blue	na

chrUn	5267905	5269080	1176	SAT1_CF	blue	na
chrUn	5269103	5269208	106	SAT1_CF	blue	na
chrUn	5269221	5269738	518	SAT1_CF	blue	na
chrUn	5269757	5271797	2041	SAT1_CF	blue	na
chrUn	5271841	5272516	676	SAT1_CF	blue	na
chrUn	5272528	5273166	639	SAT1_CF	blue	na
chrUn	5279705	5279793	89	SAT1_CF	blue	na
chrUn	5279806	5280288	483	SAT1_CF	blue	na
chrUn	5280348	5280743	396	SAT1_CF	blue	na
chrUn	5280758	5281306	549	SAT1_CF	blue	na
chrUn	5281344	5282195	852	SAT1_CF	blue	na
chrUn	5282207	5282852	646	SAT1_CF	blue	na
chrUn	5282901	5283400	500	SAT1_CF	blue	na
chrUn	5283440	5284409	970	SAT1_CF	blue	na
chrUn	5284440	5285344	905	SAT1_CF	blue	na
chrUn	5285386	5286251	866	SAT1_CF	blue	na
chrUn	5286263	5286817	555	SAT1_CF	blue	na
chrUn	5286884	5287733	850	SAT1_CF	blue	na
chrUn	5287850	5289892	2043	SAT1_CF	blue	na
chrUn	5289935	5290497	563	SAT1_CF	blue	na
chrUn	5290562	5291104	543	SAT1_CF	blue	na
chrUn	5291146	5292537	1392	SAT1_CF	blue	na
chrUn	5292550	5293030	481	SAT1_CF	blue	na
chrUn	5293092	5293492	401	SAT1_CF	blue	na
chrUn	5293505	5295121	1617	SAT1_CF	blue	na
chrUn	5295169	5295845	677	SAT1_CF	blue	na
chrUn	5295857	5296459	603	SAT1_CF	blue	na
chrUn	5296470	5297149	680	SAT1_CF	blue	na
chrUn	5297161	5297808	648	SAT1_CF	blue	na
chrUn	5297863	5298376	514	SAT1_CF	blue	na
chrUn	5298397	5299043	647	SAT1_CF	blue	na
chrUn	5299064	5300406	1343	SAT1_CF	blue	na
chrUn	5300426	5301760	1335	SAT1_CF	blue	na
chrUn	5301773	5302790	1018	SAT1_CF	blue	na
chrUn	5302833	5303509	677	SAT1_CF	blue	na
chrUn	5303521	5304087	567	SAT1_CF	blue	na
chrUn	5304138	5305094	957	SAT1_CF	blue	na
chrUn	5305173	5305954	782	SAT1_CF	blue	na
chrUn	5305997	5306730	734	SAT1_CF	blue	na
chrUn	5306749	5307319	571	SAT1_CF	blue	na
chrUn	5307384	5307541	158	SAT1_CF	blue	na
chrUn	5307556	5308758	1203	SAT1_CF	blue	na

chrUn	5308802	5310004	1203	SAT1_CF	blue	na
chrUn	5310060	5310196	137	SAT1_CF	blue	na
chrUn	5310261	5310803	543	SAT1_CF	blue	na
chrUn	5310846	5311521	676	SAT1_CF	blue	na
chrUn	5311533	5312087	555	SAT1_CF	blue	na
chrUn	5312130	5313208	1079	SAT1_CF	blue	na
chrUn	5313240	5313280	41	SAT1_CF	blue	na
chrUn	5313293	5313460	168	SAT1_CF	blue	na
chrUn	5313472	5314144	673	SAT1_CF	blue	na
chrUn	5314157	5314638	482	SAT1_CF	blue	na
chrUn	5314664	5315507	844	SAT1_CF	blue	na
chrUn	5315567	5316539	973	SAT1_CF	blue	na
chrUn	5316571	5317646	1076	SAT1_CF	blue	na
chrUn	5317690	5318364	675	SAT1_CF	blue	na
chrUn	5318391	5318977	587	SAT1_CF	blue	na
chrUn	5319004	5319477	474	SAT1_CF	blue	na
chrUn	5319505	5320714	1210	SAT1_CF	blue	na
chrUn	5320756	5321430	675	SAT1_CF	blue	na
chrUn	5321442	5321939	498	SAT1_CF	blue	na
chrUn	5321984	5322139	156	SAT1_CF	blue	na
chrUn	5322352	5322875	524	SAT1_CF	blue	na
chrUn	5322889	5322969	81	SAT1_CF	blue	na
chrUn	5322982	5323733	752	SAT1_CF	blue	na
chrUn	5323755	5324405	651	SAT1_CF	blue	na
chrUn	5324419	5324926	508	SAT1_CF	blue	na
chrUn	5324969	5325310	342	SAT1_CF	blue	na
chrUn	5325353	5327748	2396	SAT1_CF	blue	na
chrUn	5327835	5328190	356	SAT1_CF	blue	na
chrUn	5328201	5328814	614	SAT1_CF	blue	na
chrUn	5328825	5329712	888	SAT1_CF	blue	na
chrUn	5329730	5330230	501	SAT1_CF	blue	na
chrUn	5330243	5331301	1059	SAT1_CF	blue	na
chrUn	5331322	5331430	109	SAT1_CF	blue	na
chrUn	5331443	5331916	474	SAT1_CF	blue	na
chrUn	5331972	5332157	186	SAT1_CF	blue	na
chrUn	5332339	5332504	166	SAT1_CF	blue	na
chrUn	5332516	5333553	1038	SAT1_CF	blue	na
chrUn	5333581	5335500	1920	SAT1_CF	blue	na
chrUn	5335526	5335683	158	SAT1_CF	blue	na
chrUn	5335698	5336329	632	SAT1_CF	blue	na
chrUn	5336340	5336894	555	SAT1_CF	blue	na
chrUn	5336953	5337023	71	SAT1_CF	blue	na

chrUn	5337036	5337235	200	SAT1_CF	blue	na
chrUn	5351503	5353865	2363	SAT1_CF	blue	na
chrUn	5353878	5354380	503	SAT1_CF	blue	na
chrUn	5354482	5355967	1486	SAT1_CF	blue	na
chrUn	5355979	5356624	646	SAT1_CF	blue	na
chrUn	5356637	5357564	928	SAT1_CF	blue	na
chrUn	5357576	5358259	684	SAT1_CF	blue	na
chrUn	5358271	5360563	2293	SAT1_CF	blue	na
chrUn	5360589	5361070	482	SAT1_CF	blue	na
chrUn	5361130	5361831	702	SAT1_CF	blue	na
chrUn	5361911	5362474	564	SAT1_CF	blue	na
chrUn	5362501	5363131	631	SAT1_CF	blue	na
chrUn	5363174	5363838	665	SAT1_CF	blue	na
chrUn	5363858	5366073	2216	SAT1_CF	blue	na
chrUn	5366085	5366639	555	SAT1_CF	blue	na
chrUn	5366664	5366772	109	SAT1_CF	blue	na
chrUn	5366786	5367286	501	SAT1_CF	blue	na
chrUn	5367352	5367488	137	SAT1_CF	blue	na
chrUn	5367503	5368773	1271	SAT1_CF	blue	na
chrUn	5368831	5368901	71	SAT1_CF	blue	na
chrUn	5368914	5369413	500	SAT1_CF	blue	na
chrUn	5369449	5369634	186	SAT1_CF	blue	na
chrUn	5369816	5369985	170	SAT1_CF	blue	na
chrUn	5370065	5372413	2349	SAT1_CF	blue	na
chrUn	5372425	5373799	1375	SAT1_CF	blue	na
chrUn	5373842	5374302	461	SAT1_CF	blue	na
chrUn	5374323	5374952	630	SAT1_CF	blue	na
chrUn	5374976	5375082	107	SAT1_CF	blue	na
chrUn	5375094	5375618	525	SAT1_CF	blue	na
chrUn	5375670	5376417	748	SAT1_CF	blue	na
chrUn	5376474	5378643	2170	SAT1_CF	blue	na
chrUn	5378679	5378864	186	SAT1_CF	blue	na
chrUn	5379044	5379759	716	SAT1_CF	blue	na
chrUn	5379770	5381892	2123	SAT1_CF	blue	na
chrUn	5381904	5382750	847	SAT1_CF	blue	na
chrUn	5382781	5384409	1629	SAT1_CF	blue	na
chrUn	5384452	5385126	675	SAT1_CF	blue	na
chrUn	5385138	5385768	631	SAT1_CF	blue	na
chrUn	5385790	5385896	107	SAT1_CF	blue	na
chrUn	5385909	5386410	502	SAT1_CF	blue	na
chrUn	5386450	5387557	1108	SAT1_CF	blue	na
chrUn	5387568	5388070	503	SAT1_CF	blue	na

chrUn	5388123	5389253	1131	SAT1_CF	blue	na
chrUn	5389268	5389965	698	SAT1_CF	blue	na
chrUn	5390007	5390124	118	SAT1_CF	blue	na
chrUn	5390171	5390831	661	SAT1_CF	blue	na
chrUn	5390858	5391360	503	SAT1_CF	blue	na
chrUn	5391474	5391802	329	SAT1_CF	blue	na
chrUn	5391815	5392323	509	SAT1_CF	blue	na
chrUn	5392402	5393787	1386	SAT1_CF	blue	na
chrUn	5393904	5395096	1193	SAT1_CF	blue	na
chrUn	5395108	5395663	556	SAT1_CF	blue	na
chrUn	5395684	5396303	620	SAT1_CF	blue	na
chrUn	5396390	5398406	2017	SAT1_CF	blue	na
chrUn	5398424	5399755	1332	SAT1_CF	blue	na
chrUn	5399768	5400814	1047	SAT1_CF	blue	na
chrUn	5402395	5403316	922	SAT1_CF	blue	na
chrUn	5403329	5403830	502	SAT1_CF	blue	na
chrUn	5403875	5404032	158	SAT1_CF	blue	na
chrUn	5404060	5406881	2822	SAT1_CF	blue	na
chrUn	5406951	5407625	675	SAT1_CF	blue	na
chrUn	5407637	5408164	528	SAT1_CF	blue	na
chrUn	5408217	5408293	77	SAT1_CF	blue	na
chrUn	5408306	5409670	1365	SAT1_CF	blue	na
chrUn	5409717	5410185	469	SAT1_CF	blue	na
chrUn	5410201	5411126	926	SAT1_CF	blue	na
chrUn	5411170	5412505	1336	SAT1_CF	blue	na
chrUn	5412517	5413227	711	SAT1_CF	blue	na
chrUn	5413241	5413357	117	SAT1_CF	blue	na
chrUn	5413369	5413889	521	SAT1_CF	blue	na
chrUn	5413957	5415766	1810	SAT1_CF	blue	na
chrUn	5415808	5416660	853	SAT1_CF	blue	na
chrUn	5416678	5417357	680	SAT1_CF	blue	na
chrUn	5417371	5417863	493	SAT1_CF	blue	na
chrUn	5417874	5420253	2380	SAT1_CF	blue	na
chrUn	5420275	5420796	522	SAT1_CF	blue	na
chrUn	5420830	5421219	390	SAT1_CF	blue	na
chrUn	5421232	5421839	608	SAT1_CF	blue	na
chrUn	5421894	5422859	966	SAT1_CF	blue	na
chrUn	5422902	5423566	665	SAT1_CF	blue	na
chrUn	5423589	5424271	683	SAT1_CF	blue	na
chrUn	5424291	5424785	495	SAT1_CF	blue	na
chrUn	5424825	5425398	574	SAT1_CF	blue	na
chrUn	5426795	5428085	1291	SAT1_CF	blue	na

chrUn	5428127	5428439	313	SAT1_CF	blue	na
chrUn	5428481	5429001	521	SAT1_CF	blue	na
chrUn	5429026	5429400	375	SAT1_CF	blue	na
chrUn	5429432	5431770	2339	SAT1_CF	blue	na
chrUn	5431783	5432898	1116	SAT1_CF	blue	na
chrUn	5432983	5433834	852	SAT1_CF	blue	na
chrUn	5433847	5435218	1372	SAT1_CF	blue	na
chrUn	5435256	5436216	961	SAT1_CF	blue	na
chrUn	5436295	5436620	326	SAT1_CF	blue	na
chrUn	5436645	5437979	1335	SAT1_CF	blue	na
chrUn	5437991	5438493	503	SAT1_CF	blue	na
chrUn	5438529	5438901	373	SAT1_CF	blue	na
chrUn	5438944	5441049	2106	SAT1_CF	blue	na
chrUn	5441061	5441615	555	SAT1_CF	blue	na
chrUn	5441637	5441743	107	SAT1_CF	blue	na
chrUn	5441756	5442257	502	SAT1_CF	blue	na
chrUn	5442305	5442412	108	SAT1_CF	blue	na
chrUn	5442476	5443001	526	SAT1_CF	blue	na
chrUn	5443042	5443502	461	SAT1_CF	blue	na
chrUn	5443577	5444122	546	SAT1_CF	blue	na
chrUn	5444152	5445793	1642	SAT1_CF	blue	na
chrUn	5445820	5446301	482	SAT1_CF	blue	na
chrUn	5446327	5447393	1067	SAT1_CF	blue	na
chrUn	5447411	5447891	481	SAT1_CF	blue	na
chrUn	5447957	5448359	403	SAT1_CF	blue	na
chrUn	5448372	5450739	2368	SAT1_CF	blue	na
chrUn	5450761	5451318	558	SAT1_CF	blue	na
chrUn	5451340	5451488	149	SAT1_CF	blue	na
chrUn	5451501	5452003	503	SAT1_CF	blue	na
chrUn	5452058	5452444	387	SAT1_CF	blue	na
chrUn	5452481	5453491	1011	SAT1_CF	blue	na
chrUn	5453511	5453618	108	SAT1_CF	blue	na
chrUn	5453631	5454150	520	SAT1_CF	blue	na
chrUn	5454171	5454352	182	SAT1_CF	blue	na
chrUn	5454533	5456871	2339	SAT1_CF	blue	na
chrUn	5456916	5457565	650	SAT1_CF	blue	na
chrUn	5457609	5458462	854	SAT1_CF	blue	na
chrUn	5458485	5459038	554	SAT1_CF	blue	na
chrUn	5459059	5459164	106	SAT1_CF	blue	na
chrUn	5459177	5459780	604	SAT1_CF	blue	na
chrUn	5459792	5461042	1251	SAT1_CF	blue	na
chrUn	5461086	5462004	919	SAT1_CF	blue	na

chrUn	5462063	5462715	653	SAT1_CF	blue	na
chrUn	5462738	5463239	502	SAT1_CF	blue	na
chrUn	5463277	5463685	409	SAT1_CF	blue	na
chrUn	5463727	5464800	1074	SAT1_CF	blue	na
chrUn	5464814	5465353	540	SAT1_CF	blue	na
chrUn	5465396	5466058	663	SAT1_CF	blue	na
chrUn	5466070	5466829	760	SAT1_CF	blue	na
chrUn	5466842	5467394	553	SAT1_CF	blue	na
chrUn	5467417	5467523	107	SAT1_CF	blue	na
chrUn	5467536	5468037	502	SAT1_CF	blue	na
chrUn	5468077	5469185	1109	SAT1_CF	blue	na
chrUn	5469196	5469678	483	SAT1_CF	blue	na
chrUn	5469764	5469900	137	SAT1_CF	blue	na
chrUn	5469915	5471153	1239	SAT1_CF	blue	na
chrUn	5471197	5471722	526	SAT1_CF	blue	na
chrUn	5471753	5472245	493	SAT1_CF	blue	na
chrUn	5472389	5474355	1967	SAT1_CF	blue	na
chrUn	5474404	5475076	673	SAT1_CF	blue	na
chrUn	5475089	5475591	503	SAT1_CF	blue	na
chrUn	5475636	5475792	157	SAT1_CF	blue	na
chrUn	5475821	5477530	1710	SAT1_CF	blue	na
chrUn	5477542	5478040	499	SAT1_CF	blue	na
chrUn	5478080	5479163	1084	SAT1_CF	blue	na
chrUn	5479186	5479641	456	SAT1_CF	blue	na
chrUn	5479702	5479882	181	SAT1_CF	blue	na
chrUn	5480062	5482820	2759	SAT1_CF	blue	na
chrUn	5482862	5483416	555	SAT1_CF	blue	na
chrUn	5483460	5484723	1264	SAT1_CF	blue	na
chrUn	5484744	5484852	109	SAT1_CF	blue	na
chrUn	5484865	5485366	502	SAT1_CF	blue	na
chrUn	5485406	5486412	1007	SAT1_CF	blue	na
chrUn	5486479	5487444	966	SAT1_CF	blue	na
chrUn	5487487	5488164	678	SAT1_CF	blue	na
chrUn	5488176	5488677	502	SAT1_CF	blue	na
chrUn	5488715	5488975	261	SAT1_CF	blue	na
chrUn	5489020	5490262	1243	SAT1_CF	blue	na
chrUn	5490274	5490920	647	SAT1_CF	blue	na
chrUn	5490933	5491307	375	SAT1_CF	blue	na
chrUn	5491679	5492219	541	SAT1_CF	blue	na
chrUn	5492231	5492914	684	SAT1_CF	blue	na
chrUn	5492926	5493556	631	SAT1_CF	blue	na
chrUn	5493579	5493684	106	SAT1_CF	blue	na

chrUn	5493697	5494216	520	SAT1_CF	blue	na
chrUn	5494233	5494418	186	SAT1_CF	blue	na
chrUn	5494601	5494766	166	SAT1_CF	blue	na
chrUn	5494846	5495455	610	SAT1_CF	blue	na
chrUn	5495475	5496634	1160	SAT1_CF	blue	na
chrUn	5496646	5497278	633	SAT1_CF	blue	na
chrUn	5497348	5498975	1628	SAT1_CF	blue	na
chrUn	5498998	5499103	106	SAT1_CF	blue	na
chrUn	5499116	5499635	520	SAT1_CF	blue	na
chrUn	5499656	5499837	182	SAT1_CF	blue	na
chrUn	5500019	5501679	1661	SAT1_CF	blue	na
chrUn	5501755	5502615	861	SAT1_CF	blue	na
chrUn	5502629	5503257	629	SAT1_CF	blue	na
chrUn	5503279	5503388	110	SAT1_CF	blue	na
chrUn	5503399	5504468	1070	SAT1_CF	blue	na
chrUn	5504525	5505004	480	SAT1_CF	blue	na
chrUn	5505018	5505707	690	SAT1_CF	blue	na
chrUn	5505768	5506312	545	SAT1_CF	blue	na
chrUn	5506333	5506442	110	SAT1_CF	blue	na
chrUn	5506454	5507726	1273	SAT1_CF	blue	na
chrUn	5507771	5507928	158	SAT1_CF	blue	na
chrUn	5507993	5509335	1343	SAT1_CF	blue	na
chrUn	5509366	5509884	519	SAT1_CF	blue	na
chrUn	5510001	5511993	1993	SAT1_CF	blue	na
chrUn	5512036	5512710	675	SAT1_CF	blue	na
chrUn	5512723	5513223	501	SAT1_CF	blue	na
chrUn	5513288	5513423	136	SAT1_CF	blue	na
chrUn	5513456	5514631	1176	SAT1_CF	blue	na
chrUn	5514659	5514759	101	SAT1_CF	blue	na
chrUn	5514772	5515250	479	SAT1_CF	blue	na
chrUn	5515308	5516333	1026	SAT1_CF	blue	na
chrUn	5516350	5517346	997	SAT1_CF	blue	na
chrUn	5517390	5518065	676	SAT1_CF	blue	na
chrUn	5518077	5518845	769	SAT1_CF	blue	na
chrUn	5518866	5519657	792	SAT1_CF	blue	na
chrUn	5519701	5520377	677	SAT1_CF	blue	na
chrUn	5520397	5520891	495	SAT1_CF	blue	na
chrUn	5520978	5521495	518	SAT1_CF	blue	na
chrUn	5522912	5523490	579	SAT1_CF	blue	na
chrUn	5523543	5524845	1303	SAT1_CF	blue	na
chrUn	5524889	5525563	675	SAT1_CF	blue	na
chrUn	5525576	5526183	608	SAT1_CF	blue	na

chrUn	5526195	5526896	702	SAT1_CF	blue	na
chrUn	5526911	5528103	1193	SAT1_CF	blue	na
chrUn	5528126	5528231	106	SAT1_CF	blue	na
chrUn	5528244	5528724	481	SAT1_CF	blue	na
chrUn	5528784	5528965	182	SAT1_CF	blue	na
chrUn	5529147	5531023	1877	SAT1_CF	blue	na
chrUn	5531067	5531743	677	SAT1_CF	blue	na
chrUn	5531757	5532309	553	SAT1_CF	blue	na
chrUn	5532331	5532440	110	SAT1_CF	blue	na
chrUn	5532451	5534056	1606	SAT1_CF	blue	na
chrUn	5534070	5534614	545	SAT1_CF	blue	na
chrUn	5534657	5535491	835	SAT1_CF	blue	na
chrUn	5535505	5536134	630	SAT1_CF	blue	na
chrUn	5536156	5536776	621	SAT1_CF	blue	na
chrUn	5536863	5537751	889	SAT1_CF	blue	na
chrUn	5537765	5538478	714	SAT1_CF	blue	na
chrUn	5538548	5539201	654	SAT1_CF	blue	na
chrUn	5539213	5540929	1717	SAT1_CF	blue	na
chrUn	5540941	5541499	559	SAT1_CF	blue	na
chrUn	5541521	5541650	130	SAT1_CF	blue	na
chrUn	5541696	5542920	1225	SAT1_CF	blue	na
chrUn	5542933	5543433	501	SAT1_CF	blue	na
chrUn	5543478	5544918	1441	SAT1_CF	blue	na
chrUn	5544944	5545049	106	SAT1_CF	blue	na
chrUn	5545062	5545761	700	SAT1_CF	blue	na
chrUn	5642061	5642508	448	SAT1_CF	blue	na
chrUn	5642522	5643023	502	SAT1_CF	blue	na
chrUn	5643059	5643244	186	SAT1_CF	blue	na
chrUn	5643425	5644261	837	SAT1_CF	blue	na
chrUn	5644273	5645033	761	SAT1_CF	blue	na
chrUn	5645047	5645548	502	SAT1_CF	blue	na
chrUn	5645588	5646931	1344	SAT1_CF	blue	na
chrUn	5646974	5648308	1335	SAT1_CF	blue	na
chrUn	5648320	5649081	762	SAT1_CF	blue	na
chrUn	5649101	5649697	597	SAT1_CF	blue	na
chrUn	5649709	5650210	502	SAT1_CF	blue	na
chrUn	5650250	5650724	475	SAT1_CF	blue	na
chrUn	5650737	5652289	1553	SAT1_CF	blue	na
chrUn	5652332	5654418	2087	SAT1_CF	blue	na
chrUn	5654436	5654957	522	SAT1_CF	blue	na
chrUn	5654976	5656498	1523	SAT1_CF	blue	na
chrUn	5656542	5657199	658	SAT1_CF	blue	na

chrUn	5657243	5657791	549	SAT1_CF	blue	na
chrUn	5657870	5658195	326	SAT1_CF	blue	na
chrUn	5658220	5659553	1334	SAT1_CF	blue	na
chrUn	5659565	5660085	521	SAT1_CF	blue	na
chrUn	5660106	5660504	399	SAT1_CF	blue	na
chrUn	5660520	5661486	967	SAT1_CF	blue	na
chrUn	5661530	5662071	542	SAT1_CF	blue	na
chrUn	5662114	5662664	551	SAT1_CF	blue	na
chrUn	5662687	5663532	846	SAT1_CF	blue	na
chrUn	5663545	5664046	502	SAT1_CF	blue	na
chrUn	5664086	5665178	1093	SAT1_CF	blue	na
chrUn	5665205	5665707	503	SAT1_CF	blue	na
chrUn	5665773	5665909	137	SAT1_CF	blue	na
chrUn	5665924	5666447	524	SAT1_CF	blue	na
chrUn	5666527	5667583	1057	SAT1_CF	blue	na
chrUn	5667700	5668024	325	SAT1_CF	blue	na
chrUn	5668037	5669289	1253	SAT1_CF	blue	na
chrUn	5669302	5669804	503	SAT1_CF	blue	na
chrUn	5669844	5670245	402	SAT1_CF	blue	na
chrUn	5670258	5670799	542	SAT1_CF	blue	na
chrUn	5670815	5671354	540	SAT1_CF	blue	na
chrUn	5671433	5672917	1485	SAT1_CF	blue	na
chrUn	5672967	5673470	504	SAT1_CF	blue	na
chrUn	5673549	5674330	782	SAT1_CF	blue	na
chrUn	5674372	5675102	731	SAT1_CF	blue	na
chrUn	5675130	5675927	798	SAT1_CF	blue	na
chrUn	5675939	5676492	554	SAT1_CF	blue	na
chrUn	5676514	5676621	108	SAT1_CF	blue	na
chrUn	5676634	5677152	519	SAT1_CF	blue	na
chrUn	5677171	5677575	405	SAT1_CF	blue	na
chrUn	5677589	5678095	507	SAT1_CF	blue	na
chrUn	5678126	5678383	258	SAT1_CF	blue	na
chrUn	5684137	5684941	805	SAT1_CF	blue	na
chrUn	5684984	5685644	661	SAT1_CF	blue	na
chrUn	5685657	5686205	549	SAT1_CF	blue	na
chrUn	5686249	5686798	550	SAT1_CF	blue	na
chrUn	5686898	5687084	187	SAT1_CF	blue	na
chrUn	5687095	5687733	639	SAT1_CF	blue	na
chrUn	5687747	5688506	760	SAT1_CF	blue	na
chrUn	5688519	5689082	564	SAT1_CF	blue	na
chrUn	5689121	5689839	719	SAT1_CF	blue	na
chrUn	5689882	5690554	673	SAT1_CF	blue	na

chrUn	5690572	5691111	540	SAT1_CF	blue	na
chrUn	5691159	5691835	677	SAT1_CF	blue	na
chrUn	5691847	5692445	599	SAT1_CF	blue	na
chrUn	5692462	5693493	1032	SAT1_CF	blue	na
chrUn	5693536	5694177	642	SAT1_CF	blue	na
chrUn	5694195	5694857	663	SAT1_CF	blue	na
chrUn	5694880	5695562	683	SAT1_CF	blue	na
chrUn	5695582	5696076	495	SAT1_CF	blue	na
chrUn	5696116	5696517	402	SAT1_CF	blue	na
chrUn	5696532	5696764	233	SAT1_CF	blue	na
chrUn	5696785	5697802	1018	SAT1_CF	blue	na
chrUn	5697846	5698520	675	SAT1_CF	blue	na
chrUn	5698532	5699291	760	SAT1_CF	blue	na
chrUn	5699304	5700064	761	SAT1_CF	blue	na
chrUn	5700084	5700578	495	SAT1_CF	blue	na
chrUn	5700626	5701147	522	SAT1_CF	blue	na
chrUn	5701207	5701874	668	SAT1_CF	blue	na
chrUn	5701896	5702567	672	SAT1_CF	blue	na
chrUn	5702593	5703074	482	SAT1_CF	blue	na
chrUn	5703160	5703297	138	SAT1_CF	blue	na
chrUn	5703325	5703874	550	SAT1_CF	blue	na
chrUn	5703954	5704515	562	SAT1_CF	blue	na
chrUn	5704543	5705174	632	SAT1_CF	blue	na
chrUn	5705217	5705753	537	SAT1_CF	blue	na
chrUn	5705774	5706447	674	SAT1_CF	blue	na
chrUn	5706469	5707035	567	SAT1_CF	blue	na
chrUn	5707141	5707495	355	SAT1_CF	blue	na
chrUn	5707509	5708017	509	SAT1_CF	blue	na
chrUn	5708096	5708877	782	SAT1_CF	blue	na
chrUn	5708927	5709780	854	SAT1_CF	blue	na
chrUn	5709792	5710475	684	SAT1_CF	blue	na
chrUn	5710488	5710664	177	SAT1_CF	blue	na
chrUn	5710678	5713972	3295	SAT1_CF	blue	na
chrUn	5714000	5714632	633	SAT1_CF	blue	na
chrUn	5714675	5715340	666	SAT1_CF	blue	na
chrUn	5715353	5715854	502	SAT1_CF	blue	na
chrUn	5715894	5716816	923	SAT1_CF	blue	na
chrUn	5716835	5717557	723	SAT1_CF	blue	na
chrUn	5717605	5718280	676	SAT1_CF	blue	na
chrUn	5718298	5718779	482	SAT1_CF	blue	na
chrUn	5718839	5719241	403	SAT1_CF	blue	na
chrUn	5719252	5720914	1663	SAT1_CF	blue	na

chrUn	5720957	5722340	1384	SAT1_CF	blue	na
chrUn	5722358	5723122	765	SAT1_CF	blue	na
chrUn	5723253	5723934	682	SAT1_CF	blue	na
chrUn	5723947	5724449	503	SAT1_CF	blue	na
chrUn	5724495	5726562	2068	SAT1_CF	blue	na
chrUn	5726605	5727281	677	SAT1_CF	blue	na
chrUn	5727293	5727894	602	SAT1_CF	blue	na
chrUn	5727907	5728426	520	SAT1_CF	blue	na
chrUn	5728494	5728848	355	SAT1_CF	blue	na
chrUn	5728859	5729406	548	SAT1_CF	blue	na
chrUn	5729485	5730800	1316	SAT1_CF	blue	na
chrUn	5730814	5731315	502	SAT1_CF	blue	na
chrUn	5731328	5731934	607	SAT1_CF	blue	na
chrUn	5733311	5735543	2233	SAT1_CF	blue	na
chrUn	5735557	5736047	491	SAT1_CF	blue	na
chrUn	5736061	5737239	1179	SAT1_CF	blue	na
chrUn	5737257	5737756	500	SAT1_CF	blue	na
chrUn	5737796	5739642	1847	SAT1_CF	blue	na
chrUn	5739684	5741477	1794	SAT1_CF	blue	na
chrUn	5741522	5743767	2246	SAT1_CF	blue	na
chrUn	5743855	5744381	527	SAT1_CF	blue	na
chrUn	5745795	5747630	1836	SAT1_CF	blue	na
chrUn	5747642	5748134	493	SAT1_CF	blue	na
chrUn	5748145	5748828	684	SAT1_CF	blue	na
chrUn	5748841	5749633	793	SAT1_CF	blue	na
chrUn	5749646	5750889	1244	SAT1_CF	blue	na
chrUn	5750918	5751590	673	SAT1_CF	blue	na
chrUn	5751657	5752616	960	SAT1_CF	blue	na
chrUn	5752659	5753340	682	SAT1_CF	blue	na
chrUn	5753351	5754109	759	SAT1_CF	blue	na
chrUn	5754121	5754615	495	SAT1_CF	blue	na
chrUn	5754668	5756653	1986	SAT1_CF	blue	na
chrUn	5756696	5758063	1368	SAT1_CF	blue	na
chrUn	5758076	5758619	544	SAT1_CF	blue	na
chrUn	5758636	5758846	211	SAT1_CF	blue	na
chrUn	5758865	5758940	76	SAT1_CF	blue	na
chrUn	5758953	5759455	503	SAT1_CF	blue	na
chrUn	5759495	5760456	962	SAT1_CF	blue	na
chrUn	5760535	5760842	308	SAT1_CF	blue	na
chrUn	5760880	5762077	1198	SAT1_CF	blue	na
chrUn	5762099	5762900	802	SAT1_CF	blue	na
chrUn	5762914	5763434	521	SAT1_CF	blue	na

chrUn	5763460	5763615	156	SAT1_CF	blue	na
chrUn	5763645	5764153	509	SAT1_CF	blue	na
chrUn	5764185	5765424	1240	SAT1_CF	blue	na
chrUn	5765450	5765606	157	SAT1_CF	blue	na
chrUn	5765621	5767484	1864	SAT1_CF	blue	na
chrUn	5767528	5768074	547	SAT1_CF	blue	na
chrUn	5768094	5768203	110	SAT1_CF	blue	na
chrUn	5768215	5769468	1254	SAT1_CF	blue	na
chrUn	5769527	5770459	933	SAT1_CF	blue	na
chrUn	5770470	5771568	1099	SAT1_CF	blue	na
chrUn	5771611	5772285	675	SAT1_CF	blue	na
chrUn	5772297	5772902	606	SAT1_CF	blue	na
chrUn	5772915	5773435	521	SAT1_CF	blue	na
chrUn	5773503	5773853	351	SAT1_CF	blue	na
chrUn	5773866	5774373	508	SAT1_CF	blue	na
chrUn	5774404	5775308	905	SAT1_CF	blue	na
chrUn	5775350	5775521	172	SAT1_CF	blue	na
chrUn	5775533	5776210	678	SAT1_CF	blue	na
chrUn	5776222	5776974	753	SAT1_CF	blue	na
chrUn	5776999	5777494	496	SAT1_CF	blue	na
chrUn	5777566	5777695	130	SAT1_CF	blue	na
chrUn	5777710	5778234	525	SAT1_CF	blue	na
chrUn	5778268	5779319	1052	SAT1_CF	blue	na
chrUn	5779331	5780047	717	SAT1_CF	blue	na
chrUn	5780058	5781336	1279	SAT1_CF	blue	na
chrUn	5781358	5781467	110	SAT1_CF	blue	na
chrUn	5781478	5781959	482	SAT1_CF	blue	na
chrUn	5782019	5784060	2042	SAT1_CF	blue	na
chrUn	5784103	5784777	675	SAT1_CF	blue	na
chrUn	5784789	5785550	762	SAT1_CF	blue	na
chrUn	5785568	5787130	1563	SAT1_CF	blue	na
chrUn	5787144	5787823	680	SAT1_CF	blue	na
chrUn	5787837	5788338	502	SAT1_CF	blue	na
chrUn	5788398	5788540	143	SAT1_CF	blue	na
chrUn	5788555	5789079	525	SAT1_CF	blue	na
chrUn	5789122	5789634	513	SAT1_CF	blue	na
chrUn	5789668	5790881	1214	SAT1_CF	blue	na
chrUn	5790903	5791477	575	SAT1_CF	blue	na
chrUn	5791498	5791601	104	SAT1_CF	blue	na
chrUn	5791619	5792125	507	SAT1_CF	blue	na
chrUn	5792167	5792492	326	SAT1_CF	blue	na
chrUn	5792538	5793732	1195	SAT1_CF	blue	na

chrUn	5793819	5796034	2216	SAT1_CF	blue	na
chrUn	5796077	5796743	667	SAT1_CF	blue	na
chrUn	5796756	5798237	1482	SAT1_CF	blue	na
chrUn	5798280	5798954	675	SAT1_CF	blue	na
chrUn	5798966	5799728	763	SAT1_CF	blue	na
chrUn	5799746	5801308	1563	SAT1_CF	blue	na
chrUn	5801322	5801990	669	SAT1_CF	blue	na
chrUn	5802023	5802560	538	SAT1_CF	blue	na
chrUn	5802603	5803147	545	SAT1_CF	blue	na
chrUn	5803204	5803868	665	SAT1_CF	blue	na
chrUn	5803881	5804383	503	SAT1_CF	blue	na
chrUn	5804423	5805353	931	SAT1_CF	blue	na
chrUn	5805373	5807746	2374	SAT1_CF	blue	na
chrUn	5807767	5807863	97	SAT1_CF	blue	na
chrUn	5807885	5808099	215	SAT1_CF	blue	na
chrUn	5812768	5813314	547	SAT1_CF	blue	na
chrUn	5813358	5814357	1000	SAT1_CF	blue	na
chrUn	5814418	5815094	677	SAT1_CF	blue	na
chrUn	5815106	5815790	685	SAT1_CF	blue	na
chrUn	5815803	5816502	700	SAT1_CF	blue	na
chrUn	5816530	5817040	511	SAT1_CF	blue	na
chrUn	5817109	5817832	724	SAT1_CF	blue	na
chrUn	5817844	5818534	691	SAT1_CF	blue	na
chrUn	5818578	5819099	522	SAT1_CF	blue	na
chrUn	5819133	5820206	1074	SAT1_CF	blue	na
chrUn	5820249	5820794	546	SAT1_CF	blue	na
chrUn	5820815	5820923	109	SAT1_CF	blue	na
chrUn	5820936	5821455	520	SAT1_CF	blue	na
chrUn	5821524	5821878	355	SAT1_CF	blue	na
chrUn	5821892	5822498	607	SAT1_CF	blue	na
chrUn	5822518	5824447	1930	SAT1_CF	blue	na
chrUn	5824460	5824941	482	SAT1_CF	blue	na
chrUn	5825018	5826735	1718	SAT1_CF	blue	na
chrUn	5826782	5827633	852	SAT1_CF	blue	na
chrUn	5827675	5828844	1170	SAT1_CF	blue	na
chrUn	5828865	5829432	568	SAT1_CF	blue	na
chrUn	5829517	5829654	138	SAT1_CF	blue	na
chrUn	5829686	5830371	686	SAT1_CF	blue	na
chrUn	5830382	5830926	545	SAT1_CF	blue	na
chrUn	5831022	5832175	1154	SAT1_CF	blue	na
chrUn	5832228	5832304	77	SAT1_CF	blue	na
chrUn	5832317	5832838	522	SAT1_CF	blue	na

chrUn	5832859	5833260	402	SAT1_CF	blue	na
chrUn	5833271	5834899	1629	SAT1_CF	blue	na
chrUn	5834942	5835616	675	SAT1_CF	blue	na
chrUn	5835629	5836113	485	SAT1_CF	blue	na
chrUn	5836181	5838123	1943	SAT1_CF	blue	na
chrUn	5838180	5838673	494	SAT1_CF	blue	na
chrUn	5838701	5839245	545	SAT1_CF	blue	na
chrUn	5839288	5839955	668	SAT1_CF	blue	na
chrUn	5839977	5841034	1058	SAT1_CF	blue	na
chrUn	5841077	5842340	1264	SAT1_CF	blue	na
chrUn	5842352	5843485	1134	SAT1_CF	blue	na
chrUn	5843581	5843717	137	SAT1_CF	blue	na
chrUn	5843732	5844771	1040	SAT1_CF	blue	na
chrUn	5844824	5844900	77	SAT1_CF	blue	na
chrUn	5844913	5845414	502	SAT1_CF	blue	na
chrUn	5845453	5845855	403	SAT1_CF	blue	na
chrUn	5845866	5848170	2305	SAT1_CF	blue	na
chrUn	5848182	5848814	633	SAT1_CF	blue	na
chrUn	5848870	5849638	769	SAT1_CF	blue	na
chrUn	5849651	5851029	1379	SAT1_CF	blue	na
chrUn	5851041	5851813	773	SAT1_CF	blue	na
chrUn	5851893	5852562	670	SAT1_CF	blue	na
chrUn	5852575	5853096	522	SAT1_CF	blue	na
chrUn	5853143	5853279	137	SAT1_CF	blue	na
chrUn	5853344	5853973	630	SAT1_CF	blue	na
chrUn	5854009	5854973	965	SAT1_CF	blue	na
chrUn	5855016	5855688	673	SAT1_CF	blue	na
chrUn	5855700	5856462	763	SAT1_CF	blue	na
chrUn	5856480	5858040	1561	SAT1_CF	blue	na
chrUn	5858054	5858725	672	SAT1_CF	blue	na
chrUn	5858747	5859291	545	SAT1_CF	blue	na
chrUn	5859334	5860524	1191	SAT1_CF	blue	na
chrUn	5860537	5861555	1019	SAT1_CF	blue	na
chrUn	5861637	5862185	549	SAT1_CF	blue	na
chrUn	5862199	5863080	882	SAT1_CF	blue	na
chrUn	5863123	5863669	547	SAT1_CF	blue	na
chrUn	5863730	5866460	2731	SAT1_CF	blue	na
chrUn	5866471	5866964	494	SAT1_CF	blue	na
chrUn	5867004	5867406	403	SAT1_CF	blue	na
chrUn	5867440	5869151	1712	SAT1_CF	blue	na
chrUn	5869186	5869665	480	SAT1_CF	blue	na
chrUn	5869691	5870757	1067	SAT1_CF	blue	na

chrUn	5870775	5871256	482	SAT1_CF	blue	na
chrUn	5871323	5871839	517	SAT1_CF	blue	na
chrUn	5871872	5873129	1258	SAT1_CF	blue	na
chrUn	5873171	5873792	622	SAT1_CF	blue	na
chrUn	5873836	5875462	1627	SAT1_CF	blue	na
chrUn	5875505	5876178	674	SAT1_CF	blue	na
chrUn	5876190	5876356	167	SAT1_CF	blue	na
chrUn	5876374	5876856	483	SAT1_CF	blue	na
chrUn	5876962	5877835	874	SAT1_CF	blue	na
chrUn	5877846	5878189	344	SAT1_CF	blue	na
chrUn	5878234	5879652	1419	SAT1_CF	blue	na
chrUn	5879664	5880446	783	SAT1_CF	blue	na
chrUn	5880459	5881866	1408	SAT1_CF	blue	na
chrUn	5881909	5882584	676	SAT1_CF	blue	na
chrUn	5882596	5883279	684	SAT1_CF	blue	na
chrUn	5883292	5884815	1524	SAT1_CF	blue	na
chrUn	5884871	5885455	585	SAT1_CF	blue	na
chrUn	5885495	5886429	935	SAT1_CF	blue	na
chrUn	5886461	5887532	1072	SAT1_CF	blue	na
chrUn	5887575	5888249	675	SAT1_CF	blue	na
chrUn	5888261	5889641	1381	SAT1_CF	blue	na
chrUn	5889659	5890181	523	SAT1_CF	blue	na
chrUn	5890247	5890357	111	SAT1_CF	blue	na
chrUn	5890381	5890901	521	SAT1_CF	blue	na
chrUn	5890969	5891882	914	SAT1_CF	blue	na
chrUn	5891998	5893646	1649	SAT1_CF	blue	na
chrUn	5893659	5894416	758	SAT1_CF	blue	na
chrUn	5894429	5896897	2469	SAT1_CF	blue	na
chrUn	5896940	5898441	1502	SAT1_CF	blue	na
chrUn	5898460	5901249	2790	SAT1_CF	blue	na
chrUn	5901261	5901863	603	SAT1_CF	blue	na
chrUn	5901876	5902395	520	SAT1_CF	blue	na
chrUn	5902419	5904477	2059	SAT1_CF	blue	na
chrUn	5904519	5905889	1371	SAT1_CF	blue	na
chrUn	5905902	5907876	1975	SAT1_CF	blue	na
chrUn	5907898	5908393	496	SAT1_CF	blue	na
chrUn	5908433	5909357	925	SAT1_CF	blue	na
chrUn	5909436	5910397	962	SAT1_CF	blue	na
chrUn	5910439	5911116	678	SAT1_CF	blue	na
chrUn	5911128	5911803	676	SAT1_CF	blue	na
chrUn	5911828	5912322	495	SAT1_CF	blue	na
chrUn	5912335	5912914	580	SAT1_CF	blue	na

chrUn	5912932	5913180	249	CarSat1	red	na
chrUn	5913225	5915065	1841	CarSat1	red	na
chrUn	5915095	5917275	2181	CarSat1	red	na
chrUn	5917305	5918013	709	CarSat1	red	na
chrUn	5918028	5918154	127	Carsat2	orange	na
chrUn	5918043	5919110	1068	CarSat1	red	na
chrUn	5920301	5921474	1174	CarSat1	red	na
chrUn	5921504	5921565	62	CarSat1	red	na
chrUn	5921776	5922440	665	CarSat1	red	na
chrUn	5922470	5924919	2450	CarSat1	red	na
chrUn	5925129	5925588	460	CarSat1	red	na
chrUn	5925603	5925729	127	Carsat2	orange	na
chrUn	5925618	5926324	707	CarSat1	red	na
chrUn	5926339	5926465	127	Carsat2	orange	na
chrUn	5926354	5927064	711	CarSat1	red	na
chrUn	5927094	5928542	1449	CarSat1	red	na
chrUn	5928572	5930340	1769	CarSat1	red	na
chrUn	5930913	5931481	569	CarSat1	red	na
chrUn	5931511	5932222	712	CarSat1	red	na
chrUn	5932237	5932363	127	Carsat2	orange	na
chrUn	5932252	5934435	2184	CarSat1	red	na
chrUn	5934465	5935174	710	CarSat1	red	na
chrUn	5935189	5935315	127	Carsat2	orange	na
chrUn	5935204	5937161	1958	CarSat1	red	na
chrUn	5937185	5938125	941	CarSat1	red	na
chrUn	5938155	5938866	712	CarSat1	red	na
chrUn	5938881	5939007	127	Carsat2	orange	na
chrUn	5938896	5939381	486	CarSat1	red	na
chrUn	5939405	5942561	3157	CarSat1	red	na
chrUn	5942591	5944770	2180	CarSat1	red	na
chrUn	5944800	5945511	712	CarSat1	red	na
chrUn	5945526	5945653	128	Carsat2	orange	na
chrUn	5945541	5946249	709	CarSat1	red	na
chrUn	5946279	5946989	711	CarSat1	red	na
chrUn	5947004	5947131	128	Carsat2	orange	na
chrUn	5947019	5947726	708	CarSat1	red	na
chrUn	5947756	5948461	706	CarSat1	red	na
chrUn	5948476	5948602	127	Carsat2	orange	na
chrUn	5948491	5949708	1218	CarSat1	red	na
chrUn	5949852	5950822	971	CarSat1	red	na
chrUn	5950837	5950963	127	Carsat2	orange	na
chrUn	5950852	5952296	1445	CarSat1	red	na

chrUn	5952326	5953767	1442	CarSat1	red	na
chrUn	5953782	5953907	126	Carsat2	orange	na
chrUn	5953797	5955243	1447	CarSat1	red	na
chrUn	5955273	5955980	708	CarSat1	red	na
chrUn	5955995	5956109	115	Carsat2	orange	na
chrUn	5956010	5956494	485	CarSat1	red	na
chrUn	5956527	5957462	936	CarSat1	red	na
chrUn	5957477	5957604	128	Carsat2	orange	na
chrUn	5957492	5958189	698	CarSat1	red	na
chrUn	5958213	5958339	127	Carsat2	orange	na
chrUn	5958228	5959768	1541	CarSat1	red	na
chrUn	5982639	5983451	813	Sat2CF	purple	na
chrUn	5982652	5983375	724	Sat2CF	purple	na
chrUn	5982808	5983373	566	Sat2CF	purple	na
chrUn	6000677	6001828	1152	Sat2CF	purple	na
chrUn	6019870	6020052	183	Sat2CF	purple	na
chrUn	6019926	6021079	1154	Sat2CF	purple	na
chrUn	6043403	6043527	125	Sat2CF	purple	na
chrUn	6043419	6044217	799	Sat2CF	purple	na
chrUn	6044106	6044244	139	Sat2CF	purple	na
chrUn	6067010	6068117	1108	Sat2CF	purple	na
chrUn	6098702	6098866	165	Sat2CF	purple	na
chrUn	6098721	6099887	1167	Sat2CF	purple	na
chrUn	6120043	6120167	125	Sat2CF	purple	na
chrUn	6120059	6120856	798	Sat2CF	purple	na
chrUn	6120745	6120883	139	Sat2CF	purple	na
chrUn	6120745	6120883	139	Sat2CF	purple	na
chrUn	6148530	6148879	350	Sat2CF	purple	na
chrUn	6148627	6148943	317	Sat2CF	purple	na
chrUn	6161810	6162813	1004	Sat2CF	purple	na
chrUn	6186449	6186637	189	Sat2CF	purple	na
chrUn	6186479	6187657	1179	Sat2CF	purple	na
chrUn	6216941	6217785	845	Sat2CF	purple	na
chrUn	6229841	6231061	1221	Sat2CF	purple	na
chrUn	6243870	6244038	169	Sat2CF	purple	na
chrUn	6243907	6244738	832	Sat2CF	purple	na
chrUn	6243928	6244654	727	Sat2CF	purple	na
chrUn	6259808	6259996	189	Sat2CF	purple	na
chrUn	6259867	6261087	1221	Sat2CF	purple	na
chrUn	6408822	6409023	202	Sat2CF	purple	na
chrUn	6408863	6410038	1176	Sat2CF	purple	na
chrUn	6432172	6432645	474	Sat2CF	purple	na

chrUn	6432223	6433385	1163	Sat2CF	purple	na
chrUn	6447428	6447621	194	Sat2CF	purple	na
chrUn	6447457	6447621	165	Sat2CF	purple	na
chrUn	6447474	6447616	143	Sat2CF	purple	na
chrUn	6470357	6471536	1180	Sat2CF	purple	na
chrUn	6497983	6499167	1185	Sat2CF	purple	na
chrUn	6509426	6509592	167	Sat2CF	purple	na
chrUn	6509484	6510653	1170	Sat2CF	purple	na
chrUn	6523294	6524132	839	Sat2CF	purple	na
chrUn	6523355	6524081	727	Sat2CF	purple	na
chrUn	6523297	6523667	371	Sat2CF	purple	na
chrUn	6523988	6524152	165	Sat2CF	purple	na
chrUn	6538948	6540188	1241	Sat2CF	purple	na
chrUn	6552315	6553466	1152	Sat2CF	purple	na
chrUn	6559789	6559966	178	Sat2CF	purple	na
chrUn	6559802	6559960	159	Sat2CF	purple	na
chrUn	6559857	6559979	123	Sat2CF	purple	na
chrUn	6573620	6574433	814	Sat2CF	purple	na
chrUn	6573705	6574972	1268	Sat2CF	purple	na
chrUn	6590242	6590391	150	Sat2CF	purple	na
chrUn	6590283	6591484	1202	Sat2CF	purple	na
chrUn	6612483	6613293	811	Sat2CF	purple	na
chrUn	6612570	6613437	868	Sat2CF	purple	na
chrUn	6661778	6662578	801	Sat2CF	purple	na
chrUn	6690424	6691095	672	Sat2CF	purple	na
chrUn	6690495	6691343	849	Sat2CF	purple	na
chrUn	6691207	6691369	163	Sat2CF	purple	na
chrUn	6708409	6709147	739	Sat2CF	purple	na
chrUn	6708411	6709073	663	Sat2CF	purple	na
chrUn	6729363	6730316	954	Sat2CF	purple	na
chrUn	6729443	6730181	739	Sat2CF	purple	na
chrUn	6747563	6748698	1136	Sat2CF	purple	na
chrUn	6766726	6767528	803	Sat2CF	purple	na
chrUn	6766747	6767452	706	Sat2CF	purple	na
chrUn	6790730	6790931	202	Sat2CF	purple	na
chrUn	6790771	6791945	1175	Sat2CF	purple	na
chrUn	6814640	6814789	150	Sat2CF	purple	na
chrUn	6814660	6815877	1218	Sat2CF	purple	na
chrUn	6814665	6815749	1085	Sat2CF	purple	na
chrUn	6814640	6814789	150	Sat2CF	purple	na
chrUn	6864498	6864621	124	Sat2CF	purple	na
chrUn	6864514	6865284	771	Sat2CF	purple	na

chrUn	6865085	6865311	227	Sat2CF	purple	na
chrUn	6886871	6887221	351	Sat2CF	purple	na
chrUn	6886897	6887287	391	Sat2CF	purple	na
chrUn	6900157	6900334	178	Sat2CF	purple	na
chrUn	6900210	6901284	1075	Sat2CF	purple	na
chrUn	6921299	6922473	1175	Sat2CF	purple	na
chrUn	6950656	6951907	1252	Sat2CF	purple	na
chrUn	6950689	6951778	1090	Sat2CF	purple	na
chrUn	6966473	6967703	1231	Sat2CF	purple	na
chrUn	6981799	6982913	1115	Sat2CF	purple	na
chrUn	6988820	6990024	1205	CarSat1	red	na
chrUn	6990193	6990347	155	CarSat1	red	na
chrUn	6990363	6990489	127	Carsat2	orange	na
chrUn	6990377	6991084	708	CarSat1	red	na
chrUn	6991114	6991674	561	CarSat1	red	na
chrUn	6991859	6992015	157	CarSat1	red	na
chrUn	6992045	6993342	1298	CarSat1	red	na
chrUn	6993525	6993679	155	CarSat1	red	na
chrUn	6993709	6994417	709	CarSat1	red	na
chrUn	6994447	6995009	563	CarSat1	red	na
chrUn	6995196	6995350	155	CarSat1	red	na
chrUn	6995380	6996087	708	CarSat1	red	na
chrUn	6996117	6996679	563	CarSat1	red	na
chrUn	6996866	6997020	155	CarSat1	red	na
chrUn	6997050	6997755	706	CarSat1	red	na
chrUn	6997785	6998347	563	CarSat1	red	na
chrUn	6998528	6998682	155	CarSat1	red	na
chrUn	6998712	6999411	700	CarSat1	red	na
chrUn	6999447	7000013	567	CarSat1	red	na
chrUn	7000219	7000438	220	CarSat1	red	na
chrUn	7000634	7000827	194	CarSat1	red	na
chrUn	7000844	7001295	452	CarSat1	red	na
chrUn	7001325	7001804	480	CarSat1	red	na
chrUn	7002797	7003359	563	CarSat1	red	na
chrUn	7003546	7003700	155	CarSat1	red	na
chrUn	7003730	7005026	1297	CarSat1	red	na
chrUn	7005208	7005458	251	CarSat1	red	na
chrUn	7005650	7006306	657	CarSat1	red	na
chrUn	7006336	7007043	708	CarSat1	red	na
chrUn	7007058	7007181	124	Carsat2	orange	na
chrUn	7007073	7007818	746	CarSat1	red	na
chrUn	7007832	7008869	1038	Sat2CF	purple	na

chrUn	7007952	7008806	855	Sat2CF	purple	na
chrUn	7027315	7027500	186	Sat2CF	purple	na
chrUn	7027346	7027983	638	Sat2CF	purple	na
chrUn	7027661	7028062	402	Sat2CF	purple	na
chrUn	7029236	7029989	754	SAT1_CF	blue	na
chrUn	7030009	7030503	495	SAT1_CF	blue	na
chrUn	7030543	7030940	398	SAT1_CF	blue	na
chrUn	7030982	7032041	1060	SAT1_CF	blue	na
chrUn	7032054	7032730	677	SAT1_CF	blue	na
chrUn	7032742	7033316	575	SAT1_CF	blue	na
chrUn	7033458	7034289	832	SAT1_CF	blue	na
chrUn	7034303	7034845	543	SAT1_CF	blue	na
chrUn	7034858	7036480	1623	SAT1_CF	blue	na
chrUn	7036523	7037200	678	SAT1_CF	blue	na
chrUn	7037212	7037713	502	SAT1_CF	blue	na
chrUn	7037758	7037915	158	SAT1_CF	blue	na
chrUn	7037948	7038454	507	SAT1_CF	blue	na
chrUn	7038485	7039945	1461	SAT1_CF	blue	na
chrUn	7039957	7041081	1125	SAT1_CF	blue	na
chrUn	7041109	7041740	632	SAT1_CF	blue	na
chrUn	7041783	7043216	1434	SAT1_CF	blue	na
chrUn	7043251	7043722	472	SAT1_CF	blue	na
chrUn	7043782	7045161	1380	SAT1_CF	blue	na
chrUn	7045205	7046535	1331	SAT1_CF	blue	na
chrUn	7046549	7047230	682	SAT1_CF	blue	na
chrUn	7047250	7047889	640	SAT1_CF	blue	na
chrUn	7047902	7048830	929	SAT1_CF	blue	na
chrUn	7048841	7049523	683	SAT1_CF	blue	na
chrUn	7049535	7050166	632	SAT1_CF	blue	na
chrUn	7050189	7050294	106	SAT1_CF	blue	na
chrUn	7050307	7052003	1697	SAT1_CF	blue	na
chrUn	7052026	7052131	106	SAT1_CF	blue	na
chrUn	7052144	7052663	520	SAT1_CF	blue	na
chrUn	7052680	7052865	186	SAT1_CF	blue	na
chrUn	7053048	7053250	203	SAT1_CF	blue	na
chrUn	7053293	7053902	610	SAT1_CF	blue	na
chrUn	7053922	7055081	1160	SAT1_CF	blue	na
chrUn	7055093	7055724	632	SAT1_CF	blue	na
chrUn	7055795	7056336	542	SAT1_CF	blue	na
chrUn	7056355	7056667	313	SAT1_CF	blue	na
chrUn	7056745	7057308	564	SAT1_CF	blue	na
chrUn	7057335	7058557	1223	SAT1_CF	blue	na

chrUn	7058601	7059842	1242	SAT1_CF	blue	na
chrUn	7059921	7060883	963	SAT1_CF	blue	na
chrUn	7060926	7061605	680	SAT1_CF	blue	na
chrUn	7061617	7062119	503	SAT1_CF	blue	na
chrUn	7062164	7062320	157	SAT1_CF	blue	na
chrUn	7062385	7063081	697	SAT1_CF	blue	na
chrUn	7063108	7063757	650	SAT1_CF	blue	na
chrUn	7063800	7064475	676	SAT1_CF	blue	na
chrUn	7064487	7065142	656	SAT1_CF	blue	na
chrUn	7065155	7065674	520	SAT1_CF	blue	na
chrUn	7065695	7067517	1823	SAT1_CF	blue	na
chrUn	7067559	7068105	547	SAT1_CF	blue	na
chrUn	7068162	7068710	549	SAT1_CF	blue	na
chrUn	7068755	7071089	2335	SAT1_CF	blue	na
chrUn	7071101	7071655	555	SAT1_CF	blue	na
chrUn	7071676	7071783	108	SAT1_CF	blue	na
chrUn	7071803	7072341	539	SAT1_CF	blue	na
chrUn	7072458	7073345	888	SAT1_CF	blue	na
chrUn	7073363	7073644	282	SAT1_CF	blue	na
chrUn	7073686	7074411	726	SAT1_CF	blue	na
chrUn	7074472	7075015	544	SAT1_CF	blue	na
chrUn	7075036	7075145	110	SAT1_CF	blue	na
chrUn	7075157	7076428	1272	SAT1_CF	blue	na
chrUn	7076494	7076630	137	SAT1_CF	blue	na
chrUn	7076658	7077848	1191	SAT1_CF	blue	na
chrUn	7077893	7079223	1331	SAT1_CF	blue	na
chrUn	7079235	7079787	553	SAT1_CF	blue	na
chrUn	7079830	7080392	563	SAT1_CF	blue	na
chrUn	7080457	7081822	1366	SAT1_CF	blue	na
chrUn	7081836	7082985	1150	SAT1_CF	blue	na
chrUn	7083005	7083423	419	SAT1_CF	blue	na
chrUn	7083443	7083936	494	SAT1_CF	blue	na
chrUn	7083976	7084484	509	SAT1_CF	blue	na
chrUn	7086429	7087995	1567	SAT1_CF	blue	na
chrUn	7088040	7089064	1025	SAT1_CF	blue	na
chrUn	7089109	7090805	1697	SAT1_CF	blue	na
chrUn	7090817	7091503	687	SAT1_CF	blue	na
chrUn	7091515	7092034	520	SAT1_CF	blue	na
chrUn	7092053	7092538	486	SAT1_CF	blue	na
chrUn	7092556	7093330	775	SAT1_CF	blue	na
chrUn	7093342	7093895	554	SAT1_CF	blue	na
chrUn	7093938	7095145	1208	SAT1_CF	blue	na

chrUn	7095157	7096088	932	SAT1_CF	blue	na
chrUn	7096148	7097107	960	SAT1_CF	blue	na
chrUn	7097151	7098152	1002	SAT1_CF	blue	na
chrUn	7098195	7098864	670	SAT1_CF	blue	na
chrUn	7098882	7099971	1090	SAT1_CF	blue	na
chrUn	7100011	7100413	403	SAT1_CF	blue	na
chrUn	7100431	7100943	513	SAT1_CF	blue	na
chrUn	7100964	7101904	941	SAT1_CF	blue	na
chrUn	7101922	7102465	544	SAT1_CF	blue	na
chrUn	7102547	7103088	542	SAT1_CF	blue	na
chrUn	7103124	7103527	404	SAT1_CF	blue	na
chrUn	7103538	7104220	683	SAT1_CF	blue	na
chrUn	7104234	7104712	479	SAT1_CF	blue	na
chrUn	7104771	7104953	183	SAT1_CF	blue	na
chrUn	7105133	7105900	768	SAT1_CF	blue	na
chrUn	7105932	7107887	1956	SAT1_CF	blue	na
chrUn	7107900	7108400	501	SAT1_CF	blue	na
chrUn	7108440	7108840	401	SAT1_CF	blue	na
chrUn	7108851	7109398	548	SAT1_CF	blue	na
chrUn	7109455	7110440	986	SAT1_CF	blue	na
chrUn	7110483	7111157	675	SAT1_CF	blue	na
chrUn	7111169	7111855	687	SAT1_CF	blue	na
chrUn	7111867	7113196	1330	SAT1_CF	blue	na
chrUn	7113275	7113635	361	SAT1_CF	blue	na
chrUn	7113659	7114975	1317	SAT1_CF	blue	na
chrUn	7114987	7115542	556	SAT1_CF	blue	na
chrUn	7115563	7115671	109	SAT1_CF	blue	na
chrUn	7115684	7116184	501	SAT1_CF	blue	na
chrUn	7116224	7116616	393	SAT1_CF	blue	na
chrUn	7116633	7117488	856	SAT1_CF	blue	na
chrUn	7117536	7118870	1335	SAT1_CF	blue	na
chrUn	7118883	7119385	503	SAT1_CF	blue	na
chrUn	7119471	7119645	175	SAT1_CF	blue	na
chrUn	7119726	7121535	1810	SAT1_CF	blue	na
chrUn	7121557	7121624	68	SAT1_CF	blue	na
chrUn	7121642	7121913	272	SAT1_CF	blue	na
chrUn	7121971	7122570	600	SAT1_CF	blue	na
chrUn	7122582	7123265	684	SAT1_CF	blue	na
chrUn	7123277	7124039	763	SAT1_CF	blue	na
chrUn	7124051	7124606	556	SAT1_CF	blue	na
chrUn	7124629	7124735	107	SAT1_CF	blue	na
chrUn	7124748	7126912	2165	SAT1_CF	blue	na

chrUn	7126924	7127438	515	SAT1_CF	blue	na
chrUn	7127464	7127999	536	SAT1_CF	blue	na
chrUn	7128115	7128765	651	SAT1_CF	blue	na
chrUn	7128808	7129484	677	SAT1_CF	blue	na
chrUn	7129496	7130098	603	SAT1_CF	blue	na
chrUn	7130109	7130789	681	SAT1_CF	blue	na
chrUn	7130801	7131490	690	SAT1_CF	blue	na
chrUn	7131504	7133345	1842	SAT1_CF	blue	na
chrUn	7133358	7133895	538	SAT1_CF	blue	na
chrUn	7133937	7135004	1068	SAT1_CF	blue	na
chrUn	7135027	7135098	72	SAT1_CF	blue	na
chrUn	7135111	7136166	1056	SAT1_CF	blue	na
chrUn	7136187	7136296	110	SAT1_CF	blue	na
chrUn	7136308	7136809	502	SAT1_CF	blue	na
chrUn	7136896	7137249	354	SAT1_CF	blue	na
chrUn	7137260	7137772	513	SAT1_CF	blue	na
chrUn	7137839	7138803	965	SAT1_CF	blue	na
chrUn	7138846	7139392	547	SAT1_CF	blue	na
chrUn	7139414	7140088	675	SAT1_CF	blue	na
chrUn	7140111	7140217	107	SAT1_CF	blue	na
chrUn	7140229	7140731	503	SAT1_CF	blue	na
chrUn	7140776	7140933	158	SAT1_CF	blue	na
chrUn	7140961	7141471	511	SAT1_CF	blue	na
chrUn	7141514	7142312	799	SAT1_CF	blue	na
chrUn	7142356	7143531	1176	SAT1_CF	blue	na
chrUn	7143552	7144513	962	SAT1_CF	blue	na
chrUn	7144592	7145553	962	SAT1_CF	blue	na
chrUn	7145598	7146263	666	SAT1_CF	blue	na
chrUn	7146285	7146969	685	SAT1_CF	blue	na
chrUn	7146981	7147463	483	SAT1_CF	blue	na
chrUn	7147526	7147922	397	SAT1_CF	blue	na
chrUn	7147934	7148455	522	SAT1_CF	blue	na
chrUn	7148468	7149986	1519	SAT1_CF	blue	na
chrUn	7150004	7150504	501	SAT1_CF	blue	na
chrUn	7150540	7150913	374	SAT1_CF	blue	na
chrUn	7150957	7151500	544	SAT1_CF	blue	na
chrUn	7151579	7152537	959	SAT1_CF	blue	na
chrUn	7152548	7153844	1297	SAT1_CF	blue	na
chrUn	7153914	7154459	546	SAT1_CF	blue	na
chrUn	7154481	7154587	107	SAT1_CF	blue	na
chrUn	7154600	7155101	502	SAT1_CF	blue	na
chrUn	7155186	7156327	1142	SAT1_CF	blue	na

chrUn	7156349	7156455	107	SAT1_CF	blue	na
chrUn	7156468	7157023	556	SAT1_CF	blue	na
chrUn	7157044	7157141	98	SAT1_CF	blue	na
chrUn	7157163	7157658	496	SAT1_CF	blue	na
chrUn	7157745	7160488	2744	SAT1_CF	blue	na
chrUn	7160500	7161058	559	SAT1_CF	blue	na
chrUn	7161080	7161188	109	SAT1_CF	blue	na
chrUn	7161201	7161702	502	SAT1_CF	blue	na
chrUn	7161768	7161904	137	SAT1_CF	blue	na
chrUn	7161919	7162546	628	SAT1_CF	blue	na
chrUn	7162557	7164014	1458	SAT1_CF	blue	na
chrUn	7164196	7165268	1073	SAT1_CF	blue	na
chrUn	7165280	7165798	519	SAT1_CF	blue	na
chrUn	7165817	7166652	836	SAT1_CF	blue	na
chrUn	7166679	7167180	502	SAT1_CF	blue	na
chrUn	7167267	7168453	1187	SAT1_CF	blue	na
chrUn	7168495	7169039	545	SAT1_CF	blue	na
chrUn	7169081	7169626	546	SAT1_CF	blue	na
chrUn	7169683	7170636	954	SAT1_CF	blue	na
chrUn	7170681	7171413	733	SAT1_CF	blue	na
chrUn	7171428	7172990	1563	SAT1_CF	blue	na
chrUn	7173017	7173519	503	SAT1_CF	blue	na
chrUn	7173606	7173960	355	SAT1_CF	blue	na
chrUn	7173973	7174482	510	SAT1_CF	blue	na
chrUn	7174561	7175339	779	SAT1_CF	blue	na
chrUn	7175381	7175943	563	SAT1_CF	blue	na
chrUn	7176060	7177814	1755	SAT1_CF	blue	na
chrUn	7177869	7178457	589	SAT1_CF	blue	na
chrUn	7178497	7178899	403	SAT1_CF	blue	na
chrUn	7178912	7180077	1166	SAT1_CF	blue	na
chrUn	7180102	7180721	620	SAT1_CF	blue	na
chrUn	7180741	7182209	1469	SAT1_CF	blue	na
chrUn	7182241	7183949	1709	SAT1_CF	blue	na
chrUn	7183971	7184195	225	SAT1_CF	blue	na
chrUn	7184370	7185892	1523	SAT1_CF	blue	na
chrUn	7185935	7186559	625	SAT1_CF	blue	na
chrUn	7186570	7187202	633	SAT1_CF	blue	na
chrUn	7187259	7187331	73	SAT1_CF	blue	na
chrUn	7187345	7187920	576	SAT1_CF	blue	na
chrUn	7187997	7188857	861	SAT1_CF	blue	na
chrUn	7188869	7189551	683	SAT1_CF	blue	na
chrUn	7189565	7190138	574	SAT1_CF	blue	na

chrUn	7190159	7190267	109	SAT1_CF	blue	na
chrUn	7190280	7190825	546	SAT1_CF	blue	na
chrUn	7190902	7191958	1057	SAT1_CF	blue	na
chrUn	7192016	7192826	811	SAT1_CF	blue	na
chrUn	7192841	7193517	677	SAT1_CF	blue	na
chrUn	7193529	7194212	684	SAT1_CF	blue	na
chrUn	7194225	7194706	482	SAT1_CF	blue	na
chrUn	7194727	7195125	399	SAT1_CF	blue	na
chrUn	7195139	7196292	1154	SAT1_CF	blue	na
chrUn	7196305	7197040	736	SAT1_CF	blue	na
chrUn	7197054	7197568	515	SAT1_CF	blue	na
chrUn	7197594	7197751	158	SAT1_CF	blue	na
chrUn	7197766	7198290	525	SAT1_CF	blue	na
chrUn	7198369	7199603	1235	SAT1_CF	blue	na
chrUn	7199615	7200116	502	SAT1_CF	blue	na
chrUn	7200156	7201337	1182	SAT1_CF	blue	na
chrUn	7201403	7203039	1637	SAT1_CF	blue	na
chrUn	7203081	7204220	1140	SAT1_CF	blue	na
chrUn	7204247	7205077	831	SAT1_CF	blue	na
chrUn	7205099	7206203	1105	SAT1_CF	blue	na
chrUn	7206226	7207361	1136	SAT1_CF	blue	na
chrUn	7207382	7208875	1494	SAT1_CF	blue	na
chrUn	7208897	7209422	526	SAT1_CF	blue	na
chrUn	7209465	7210011	547	SAT1_CF	blue	na
chrUn	7210068	7210592	525	SAT1_CF	blue	na
chrUn	7210610	7210722	113	SAT1_CF	blue	na
chrUn	7210735	7211235	501	SAT1_CF	blue	na
chrUn	7211275	7211534	260	SAT1_CF	blue	na
chrUn	7211578	7212125	548	SAT1_CF	blue	na
chrUn	7212168	7212861	694	SAT1_CF	blue	na
chrUn	7212873	7213556	684	SAT1_CF	blue	na
chrUn	7213569	7214268	700	SAT1_CF	blue	na
chrUn	7214296	7215932	1637	SAT1_CF	blue	na
chrUn	7216001	7216845	845	SAT1_CF	blue	na
chrUn	7216857	7217540	684	SAT1_CF	blue	na
chrUn	7217553	7218072	520	SAT1_CF	blue	na
chrUn	7218140	7218495	356	SAT1_CF	blue	na
chrUn	7218506	7219015	510	SAT1_CF	blue	na
chrUn	7219046	7220633	1588	SAT1_CF	blue	na
chrUn	7220645	7221174	530	SAT1_CF	blue	na
chrUn	7221227	7221303	77	SAT1_CF	blue	na
chrUn	7221316	7221859	544	SAT1_CF	blue	na

chrUn	7221923	7223125	1203	SAT1_CF	blue	na
chrUn	7223192	7223453	262	SAT1_CF	blue	na
chrUn	7223537	7224063	527	SAT1_CF	blue	na
chrUn	7224143	7225441	1299	SAT1_CF	blue	na
chrUn	7225454	7226136	683	SAT1_CF	blue	na
chrUn	7226156	7226649	494	SAT1_CF	blue	na
chrUn	7226689	7227262	574	SAT1_CF	blue	na
chrUn	7228660	7229418	759	SAT1_CF	blue	na
chrUn	7229461	7230138	678	SAT1_CF	blue	na
chrUn	7230158	7230653	496	SAT1_CF	blue	na
chrUn	7230719	7230855	137	SAT1_CF	blue	na
chrUn	7230870	7231394	525	SAT1_CF	blue	na
chrUn	7231424	7232631	1208	SAT1_CF	blue	na
chrUn	7232716	7233568	853	SAT1_CF	blue	na
chrUn	7233580	7234193	614	SAT1_CF	blue	na
chrUn	7234207	7234951	745	SAT1_CF	blue	na
chrUn	7234979	7235479	501	SAT1_CF	blue	na
chrUn	7235524	7235681	158	SAT1_CF	blue	na
chrUn	7235709	7236965	1257	SAT1_CF	blue	na
chrUn	7236978	7238698	1721	SAT1_CF	blue	na
chrUn	7238740	7239595	856	SAT1_CF	blue	na
chrUn	7239607	7240292	686	SAT1_CF	blue	na
chrUn	7240304	7241631	1328	SAT1_CF	blue	na
chrUn	7241710	7242070	361	SAT1_CF	blue	na
chrUn	7242095	7242637	543	SAT1_CF	blue	na
chrUn	7242734	7243400	667	SAT1_CF	blue	na
chrUn	7243412	7243967	556	SAT1_CF	blue	na
chrUn	7243990	7244096	107	SAT1_CF	blue	na
chrUn	7244109	7244610	502	SAT1_CF	blue	na
chrUn	7244663	7245546	884	SAT1_CF	blue	na
chrUn	7245578	7245914	337	SAT1_CF	blue	na
chrUn	7245962	7246608	647	SAT1_CF	blue	na
chrUn	7246620	7247295	676	SAT1_CF	blue	na
chrUn	7247308	7247809	502	SAT1_CF	blue	na
chrUn	7247895	7249876	1982	SAT1_CF	blue	na
chrUn	7249888	7250882	995	SAT1_CF	blue	na
chrUn	7250895	7251593	699	SAT1_CF	blue	na
chrUn	7251621	7252812	1192	SAT1_CF	blue	na
chrUn	7252839	7253470	632	SAT1_CF	blue	na
chrUn	7253513	7254843	1331	SAT1_CF	blue	na
chrUn	7254856	7255603	748	SAT1_CF	blue	na
chrUn	7255630	7256147	518	SAT1_CF	blue	na

chrUn	7256168	7256938	771	SAT1_CF	blue	na
chrUn	7256981	7257644	664	SAT1_CF	blue	na
chrUn	7257667	7258349	683	SAT1_CF	blue	na
chrUn	7258369	7258860	492	SAT1_CF	blue	na
chrUn	7258900	7259473	574	SAT1_CF	blue	na
chrUn	7260872	7261467	596	SAT1_CF	blue	na
chrUn	7261516	7262161	646	SAT1_CF	blue	na
chrUn	7262203	7262515	313	SAT1_CF	blue	na
chrUn	7262557	7263077	521	SAT1_CF	blue	na
chrUn	7263124	7263260	137	SAT1_CF	blue	na
chrUn	7263288	7265124	1837	SAT1_CF	blue	na
chrUn	7265167	7265833	667	SAT1_CF	blue	na
chrUn	7265845	7266319	475	SAT1_CF	blue	na
chrUn	7266389	7267062	674	SAT1_CF	blue	na
chrUn	7267074	7267616	543	SAT1_CF	blue	na
chrUn	7267659	7268809	1151	SAT1_CF	blue	na
chrUn	7268822	7269637	816	SAT1_CF	blue	na
chrUn	7269650	7271020	1371	SAT1_CF	blue	na
chrUn	7271058	7272020	963	SAT1_CF	blue	na
chrUn	7272064	7273051	988	SAT1_CF	blue	na
chrUn	7273099	7273773	675	SAT1_CF	blue	na
chrUn	7273785	7275145	1361	SAT1_CF	blue	na
chrUn	7275261	7276386	1126	SAT1_CF	blue	na
chrUn	7276399	7277083	685	SAT1_CF	blue	na
chrUn	7277096	7277598	503	SAT1_CF	blue	na
chrUn	7277655	7277996	342	SAT1_CF	blue	na
chrUn	7278011	7279447	1437	SAT1_CF	blue	na
chrUn	7279459	7280106	648	SAT1_CF	blue	na
chrUn	7280156	7280655	500	SAT1_CF	blue	na
chrUn	7280700	7280860	161	SAT1_CF	blue	na
chrUn	7280871	7281395	525	SAT1_CF	blue	na
chrUn	7281425	7282010	586	SAT1_CF	blue	na
chrUn	7282067	7282699	633	SAT1_CF	blue	na
chrUn	7282741	7283406	666	SAT1_CF	blue	na
chrUn	7283418	7283894	477	SAT1_CF	blue	na
chrUn	7283964	7284636	673	SAT1_CF	blue	na
chrUn	7284648	7285408	761	SAT1_CF	blue	na
chrUn	7285420	7286101	682	SAT1_CF	blue	na
chrUn	7286114	7286616	503	SAT1_CF	blue	na
chrUn	7286672	7287613	942	SAT1_CF	blue	na
chrUn	7287692	7288655	964	SAT1_CF	blue	na
chrUn	7288698	7289374	677	SAT1_CF	blue	na

chrUn	7289386	7289888	503	SAT1_CF	blue	na
chrUn	7289928	7291669	1742	SAT1_CF	blue	na
chrUn	7291681	7292443	763	SAT1_CF	blue	na
chrUn	7292455	7293009	555	SAT1_CF	blue	na
chrUn	7293031	7293649	619	SAT1_CF	blue	na
chrUn	7293689	7293948	260	SAT1_CF	blue	na
chrUn	7293992	7294538	547	SAT1_CF	blue	na
chrUn	7294581	7295272	692	SAT1_CF	blue	na
chrUn	7295284	7297407	2124	SAT1_CF	blue	na
chrUn	7297449	7298179	731	SAT1_CF	blue	na
chrUn	7298199	7298308	110	SAT1_CF	blue	na
chrUn	7298320	7298822	503	SAT1_CF	blue	na
chrUn	7298904	7299259	356	SAT1_CF	blue	na
chrUn	7299270	7300246	977	SAT1_CF	blue	na
chrUn	7300290	7300935	646	SAT1_CF	blue	na
chrUn	7300947	7301801	855	SAT1_CF	blue	na
chrUn	7301829	7302340	512	SAT1_CF	blue	na
chrUn	7302385	7302541	157	SAT1_CF	blue	na
chrUn	7302721	7302923	203	SAT1_CF	blue	na
chrUn	7302966	7303511	546	SAT1_CF	blue	na
chrUn	7303555	7304335	781	SAT1_CF	blue	na
chrUn	7304377	7304549	173	SAT1_CF	blue	na
chrUn	7304561	7305231	671	SAT1_CF	blue	na
chrUn	7305249	7305749	501	SAT1_CF	blue	na
chrUn	7305785	7308646	2862	SAT1_CF	blue	na
chrUn	7308661	7309183	523	SAT1_CF	blue	na
chrUn	7309226	7310249	1024	SAT1_CF	blue	na
chrUn	7310302	7311091	790	SAT1_CF	blue	na
chrUn	7311112	7311220	109	SAT1_CF	blue	na
chrUn	7311233	7311734	502	SAT1_CF	blue	na
chrUn	7311789	7313158	1370	SAT1_CF	blue	na
chrUn	7313198	7314531	1334	SAT1_CF	blue	na
chrUn	7314543	7315063	521	SAT1_CF	blue	na
chrUn	7315089	7315246	158	SAT1_CF	blue	na
chrUn	7315279	7315785	507	SAT1_CF	blue	na
chrUn	7315816	7317542	1727	SAT1_CF	blue	na
chrUn	7317582	7317973	392	SAT1_CF	blue	na
chrUn	7317988	7318125	138	SAT1_CF	blue	na
chrUn	7318139	7319507	1369	SAT1_CF	blue	na
chrUn	7319550	7320225	676	SAT1_CF	blue	na
chrUn	7320238	7320720	483	SAT1_CF	blue	na
chrUn	7320780	7322817	2038	SAT1_CF	blue	na

chrUn	7322860	7323535	676	SAT1_CF	blue	na
chrUn	7323547	7324162	616	SAT1_CF	blue	na
chrUn	7324176	7324921	746	SAT1_CF	blue	na
chrUn	7324948	7325449	502	SAT1_CF	blue	na
chrUn	7325489	7325891	403	SAT1_CF	blue	na
chrUn	7325902	7327862	1961	SAT1_CF	blue	na
chrUn	7327919	7328438	520	SAT1_CF	blue	na
chrUn	7328510	7328876	367	SAT1_CF	blue	na
chrUn	7328919	7330277	1359	SAT1_CF	blue	na
chrUn	7330289	7330902	614	SAT1_CF	blue	na
chrUn	7330915	7331898	984	SAT1_CF	blue	na
chrUn	7331919	7332028	110	SAT1_CF	blue	na
chrUn	7332040	7332541	502	SAT1_CF	blue	na
chrUn	7332628	7332983	356	SAT1_CF	blue	na
chrUn	7332999	7334071	1073	SAT1_CF	blue	na
chrUn	7334093	7334619	527	SAT1_CF	blue	na
chrUn	7334662	7335322	661	SAT1_CF	blue	na
chrUn	7335349	7336774	1426	SAT1_CF	blue	na
chrUn	7336805	7337745	941	SAT1_CF	blue	na
chrUn	7337825	7338791	967	SAT1_CF	blue	na
chrUn	7338834	7339501	668	SAT1_CF	blue	na
chrUn	7339521	7340014	494	SAT1_CF	blue	na
chrUn	7340059	7340216	158	SAT1_CF	blue	na
chrUn	7340249	7341424	1176	SAT1_CF	blue	na
chrUn	7341447	7341552	106	SAT1_CF	blue	na
chrUn	7341565	7342082	518	SAT1_CF	blue	na
chrUn	7342101	7344141	2041	SAT1_CF	blue	na
chrUn	7344185	7344859	675	SAT1_CF	blue	na
chrUn	7344871	7345638	768	SAT1_CF	blue	na
chrUn	7345651	7346133	483	SAT1_CF	blue	na
chrUn	7346193	7346552	360	SAT1_CF	blue	na
chrUn	7346567	7347115	549	SAT1_CF	blue	na
chrUn	7347153	7348996	1844	SAT1_CF	blue	na
chrUn	7349039	7349712	674	SAT1_CF	blue	na
chrUn	7349725	7350226	502	SAT1_CF	blue	na
chrUn	7350292	7350427	136	SAT1_CF	blue	na
chrUn	7350442	7350967	526	SAT1_CF	blue	na
chrUn	7351047	7351611	565	SAT1_CF	blue	na
chrUn	7351654	7353348	1695	SAT1_CF	blue	na
chrUn	7353376	7353887	512	SAT1_CF	blue	na
chrUn	7354186	7356107	1922	SAT1_CF	blue	na
chrUn	7356140	7356975	836	SAT1_CF	blue	na

chrUn	7356987	7358366	1380	SAT1_CF	blue	na
chrUn	7358378	7358931	554	SAT1_CF	blue	na
chrUn	7358953	7359058	106	SAT1_CF	blue	na
chrUn	7359072	7359557	486	SAT1_CF	blue	na
chrUn	7359629	7360175	547	SAT1_CF	blue	na
chrUn	7360218	7360753	536	SAT1_CF	blue	na
chrUn	7360797	7362043	1247	SAT1_CF	blue	na
chrUn	7362065	7362130	66	SAT1_CF	blue	na
chrUn	7362150	7362911	762	SAT1_CF	blue	na
chrUn	7362924	7364275	1352	SAT1_CF	blue	na
chrUn	7364306	7365059	754	SAT1_CF	blue	na
chrUn	7365073	7366345	1273	SAT1_CF	blue	na
chrUn	7366366	7366473	108	SAT1_CF	blue	na
chrUn	7366486	7367005	520	SAT1_CF	blue	na
chrUn	7367026	7367207	182	SAT1_CF	blue	na
chrUn	7367389	7368503	1115	SAT1_CF	blue	na
chrUn	7368548	7369085	538	SAT1_CF	blue	na
chrUn	7369127	7370534	1408	SAT1_CF	blue	na
chrUn	7370578	7371368	791	SAT1_CF	blue	na
chrUn	7371411	7372271	861	SAT1_CF	blue	na
chrUn	7372283	7372802	520	SAT1_CF	blue	na
chrUn	7372824	7372889	66	SAT1_CF	blue	na
chrUn	7372909	7373665	757	SAT1_CF	blue	na
chrUn	7373683	7374184	502	SAT1_CF	blue	na
chrUn	7374224	7375244	1021	SAT1_CF	blue	na
chrUn	7375299	7375917	619	SAT1_CF	blue	na
chrUn	7375932	7377173	1242	SAT1_CF	blue	na
chrUn	7377239	7377375	137	SAT1_CF	blue	na
chrUn	7377390	7377913	524	SAT1_CF	blue	na
chrUn	7377956	7379279	1324	SAT1_CF	blue	na
chrUn	7379299	7379847	549	SAT1_CF	blue	na
chrUn	7379860	7380751	892	SAT1_CF	blue	na
chrUn	7380763	7383085	2323	SAT1_CF	blue	na
chrUn	7383128	7383775	648	SAT1_CF	blue	na
chrUn	7383788	7384309	522	SAT1_CF	blue	na
chrUn	7384329	7384729	401	SAT1_CF	blue	na
chrUn	7384740	7385608	869	SAT1_CF	blue	na
chrUn	7385654	7386197	544	SAT1_CF	blue	na
chrUn	7386239	7386410	172	SAT1_CF	blue	na
chrUn	7386424	7387438	1015	SAT1_CF	blue	na
chrUn	7387512	7389619	2108	SAT1_CF	blue	na
chrUn	7389675	7390048	374	SAT1_CF	blue	na

chrUn	7390095	7390766	672	SAT1_CF	blue	na
chrUn	7390778	7391463	686	SAT1_CF	blue	na
chrUn	7391474	7391976	503	SAT1_CF	blue	na
chrUn	7392061	7392176	116	SAT1_CF	blue	na
chrUn	7392204	7392714	511	SAT1_CF	blue	na
chrUn	7392794	7394737	1944	SAT1_CF	blue	na
chrUn	7394749	7395942	1194	SAT1_CF	blue	na
chrUn	7395982	7396555	574	SAT1_CF	blue	na
chrUn	7397970	7398534	565	SAT1_CF	blue	na
chrUn	7398548	7400178	1631	SAT1_CF	blue	na
chrUn	7400192	7400684	493	SAT1_CF	blue	na
chrUn	7400695	7401418	724	SAT1_CF	blue	na
chrUn	7401430	7402627	1198	SAT1_CF	blue	na
chrUn	7402667	7403971	1305	SAT1_CF	blue	na
chrUn	7403989	7405276	1288	SAT1_CF	blue	na
chrUn	7405288	7405843	556	SAT1_CF	blue	na
chrUn	7405864	7405970	107	SAT1_CF	blue	na
chrUn	7405983	7406586	604	SAT1_CF	blue	na
chrUn	7406598	7407229	632	SAT1_CF	blue	na
chrUn	7407261	7408298	1038	SAT1_CF	blue	na
chrUn	7408311	7409121	811	SAT1_CF	blue	na
chrUn	7409133	7410099	967	SAT1_CF	blue	na
chrUn	7410188	7411528	1341	SAT1_CF	blue	na
chrUn	7411556	7412186	631	SAT1_CF	blue	na
chrUn	7412229	7412904	676	SAT1_CF	blue	na
chrUn	7412916	7413597	682	SAT1_CF	blue	na
chrUn	7413610	7414091	482	SAT1_CF	blue	na
chrUn	7414153	7415171	1019	SAT1_CF	blue	na
chrUn	7415191	7416199	1009	SAT1_CF	blue	na
chrUn	7416242	7417935	1694	SAT1_CF	blue	na
chrUn	7417966	7418458	493	SAT1_CF	blue	na
chrUn	7418602	7418957	356	SAT1_CF	blue	na
chrUn	7418970	7420632	1663	SAT1_CF	blue	na
chrUn	7420674	7421346	673	SAT1_CF	blue	na
chrUn	7421358	7421915	558	SAT1_CF	blue	na
chrUn	7421942	7422045	104	SAT1_CF	blue	na
chrUn	7422058	7422761	704	SAT1_CF	blue	na
chrUn	7422789	7423974	1186	SAT1_CF	blue	na
chrUn	7424016	7424560	545	SAT1_CF	blue	na
chrUn	7424603	7425280	678	SAT1_CF	blue	na
chrUn	7425292	7426055	764	SAT1_CF	blue	na
chrUn	7426067	7426621	555	SAT1_CF	blue	na

chrUn	7426646	7426745	100	SAT1_CF	blue	na
chrUn	7426764	7427266	503	SAT1_CF	blue	na
chrUn	7427312	7427419	108	SAT1_CF	blue	na
chrUn	7427483	7428689	1207	SAT1_CF	blue	na
chrUn	7428716	7429347	632	SAT1_CF	blue	na
chrUn	7429390	7430801	1412	SAT1_CF	blue	na
chrUn	7430828	7432482	1655	SAT1_CF	blue	na
chrUn	7432520	7432875	356	SAT1_CF	blue	na
chrUn	7432899	7433420	522	SAT1_CF	blue	na
chrUn	7433451	7433840	390	SAT1_CF	blue	na
chrUn	7433910	7434451	542	SAT1_CF	blue	na
chrUn	7434494	7435045	552	SAT1_CF	blue	na
chrUn	7435070	7435134	65	SAT1_CF	blue	na
chrUn	7435152	7435913	762	SAT1_CF	blue	na
chrUn	7435926	7436630	705	SAT1_CF	blue	na
chrUn	7436645	7438039	1395	SAT1_CF	blue	na
chrUn	7438056	7438557	502	SAT1_CF	blue	na
chrUn	7438597	7439998	1402	SAT1_CF	blue	na
chrUn	7440022	7441351	1330	SAT1_CF	blue	na
chrUn	7441363	7442051	689	SAT1_CF	blue	na
chrUn	7442064	7442918	855	SAT1_CF	blue	na
chrUn	7442930	7444417	1488	SAT1_CF	blue	na
chrUn	7444443	7444954	512	SAT1_CF	blue	na
chrUn	7445014	7445191	178	SAT1_CF	blue	na
chrUn	7445233	7446603	1371	SAT1_CF	blue	na
chrUn	7446621	7447525	905	SAT1_CF	blue	na
chrUn	7447568	7448418	851	SAT1_CF	blue	na
chrUn	7448441	7449122	682	SAT1_CF	blue	na
chrUn	7449142	7449636	495	SAT1_CF	blue	na
chrUn	7449723	7450192	470	SAT1_CF	blue	na
chrUn	7451605	7451959	355	SAT1_CF	blue	na
chrUn	7452008	7452652	645	SAT1_CF	blue	na
chrUn	7452701	7453006	306	SAT1_CF	blue	na
chrUn	7453048	7453548	501	SAT1_CF	blue	na
chrUn	7453591	7453987	397	SAT1_CF	blue	na
chrUn	7453998	7455622	1625	SAT1_CF	blue	na
chrUn	7455665	7456337	673	SAT1_CF	blue	na
chrUn	7456351	7456852	502	SAT1_CF	blue	na
chrUn	7456939	7457293	355	SAT1_CF	blue	na
chrUn	7457440	7457987	548	SAT1_CF	blue	na
chrUn	7458001	7458873	873	SAT1_CF	blue	na
chrUn	7458921	7460894	1974	SAT1_CF	blue	na

chrUn	7460963	7461746	784	SAT1_CF	blue	na
chrUn	7461773	7464906	3134	SAT1_CF	blue	na
chrUn	7464926	7465400	475	SAT1_CF	blue	na
chrUn	7465459	7467739	2281	SAT1_CF	blue	na
chrUn	7467752	7468326	575	SAT1_CF	blue	na
chrUn	7468355	7468455	101	SAT1_CF	blue	na
chrUn	7468468	7469013	546	SAT1_CF	blue	na
chrUn	7469090	7470147	1058	SAT1_CF	blue	na
chrUn	7470201	7470360	160	SAT1_CF	blue	na
chrUn	7470372	7471017	646	SAT1_CF	blue	na
chrUn	7471030	7471907	878	SAT1_CF	blue	na
chrUn	7471932	7472474	543	SAT1_CF	blue	na
chrUn	7472571	7473248	678	SAT1_CF	blue	na
chrUn	7473260	7473814	555	SAT1_CF	blue	na
chrUn	7473879	7474855	977	SAT1_CF	blue	na
chrUn	7474869	7476021	1153	SAT1_CF	blue	na
chrUn	7476034	7478501	2468	SAT1_CF	blue	na
chrUn	7478566	7479234	669	SAT1_CF	blue	na
chrUn	7479276	7479507	232	SAT1_CF	blue	na
chrUn	7479806	7480649	844	SAT1_CF	blue	na
chrUn	7480660	7481341	682	SAT1_CF	blue	na
chrUn	7481353	7482113	761	SAT1_CF	blue	na
chrUn	7482126	7482286	161	SAT1_CF	blue	na
chrUn	7482799	7483175	377	SAT1_CF	blue	na
chrUn	7483209	7483527	319	SAT1_CF	blue	na
chrUn	7483538	7484359	822	SAT1_CF	blue	na
chrUn	7484398	7485744	1347	SAT1_CF	blue	na
chrUn	7485757	7486611	855	SAT1_CF	blue	na
chrUn	7486623	7488109	1487	SAT1_CF	blue	na
chrUn	7488135	7489117	983	SAT1_CF	blue	na
chrUn	7489160	7489834	675	SAT1_CF	blue	na
chrUn	7489846	7491052	1207	SAT1_CF	blue	na
chrUn	7491068	7492432	1365	SAT1_CF	blue	na
chrUn	7492446	7492938	493	SAT1_CF	blue	na
chrUn	7492949	7495271	2323	SAT1_CF	blue	na
chrUn	7495325	7496290	966	SAT1_CF	blue	na
chrUn	7496333	7496999	667	SAT1_CF	blue	na
chrUn	7497022	7497704	683	SAT1_CF	blue	na
chrUn	7497724	7498217	494	SAT1_CF	blue	na
chrUn	7498304	7498774	471	SAT1_CF	blue	na
chrUn	7500168	7501810	1643	SAT1_CF	blue	na
chrUn	7501852	7502351	500	SAT1_CF	blue	na

chrUn	7502394	7502789	396	SAT1_CF	blue	na
chrUn	7502800	7503410	611	SAT1_CF	blue	na
chrUn	7503430	7504245	816	SAT1_CF	blue	na
chrUn	7504286	7505713	1428	SAT1_CF	blue	na
chrUn	7505777	7506373	597	SAT1_CF	blue	na
chrUn	7506390	7506760	371	SAT1_CF	blue	na
chrUn	7506805	7509224	2420	SAT1_CF	blue	na
chrUn	7509251	7509841	591	SAT1_CF	blue	na
chrUn	7509868	7511002	1135	SAT1_CF	blue	na
chrUn	7511014	7511547	534	SAT1_CF	blue	na
chrUn	7511575	7512207	633	SAT1_CF	blue	na
chrUn	7512251	7512925	675	SAT1_CF	blue	na
chrUn	7512937	7513438	502	SAT1_CF	blue	na
chrUn	7513478	7514435	958	SAT1_CF	blue	na
chrUn	7514460	7514638	179	SAT1_CF	blue	na
chrUn	7514717	7515260	544	SAT1_CF	blue	na
chrUn	7515302	7516032	731	SAT1_CF	blue	na
chrUn	7516055	7516692	638	SAT1_CF	blue	na
chrUn	7516718	7516873	156	SAT1_CF	blue	na
chrUn	7517102	7517696	595	SAT1_CF	blue	na
chrUn	7517714	7518475	762	SAT1_CF	blue	na
chrUn	7518488	7520626	2139	SAT1_CF	blue	na
chrUn	7520644	7521145	502	SAT1_CF	blue	na
chrUn	7521158	7522215	1058	SAT1_CF	blue	na
chrUn	7522236	7522344	109	SAT1_CF	blue	na
chrUn	7522357	7522830	474	SAT1_CF	blue	na
chrUn	7522917	7523052	136	SAT1_CF	blue	na
chrUn	7523280	7525598	2319	SAT1_CF	blue	na
chrUn	7525611	7526358	748	SAT1_CF	blue	na
chrUn	7526385	7526905	521	SAT1_CF	blue	na
chrUn	7526926	7529677	2752	SAT1_CF	blue	na
chrUn	7529699	7531062	1364	SAT1_CF	blue	na
chrUn	7531085	7531190	106	SAT1_CF	blue	na
chrUn	7531203	7531703	501	SAT1_CF	blue	na
chrUn	7531743	7533378	1636	SAT1_CF	blue	na
chrUn	7533420	7534616	1197	SAT1_CF	blue	na
chrUn	7534629	7535129	501	SAT1_CF	blue	na
chrUn	7535167	7535570	404	SAT1_CF	blue	na
chrUn	7535586	7536128	543	SAT1_CF	blue	na
chrUn	7536206	7536988	783	SAT1_CF	blue	na
chrUn	7537030	7537761	732	SAT1_CF	blue	na
chrUn	7537782	7537891	110	SAT1_CF	blue	na

chrUn	7537905	7538586	682	SAT1_CF	blue	na
chrUn	7538599	7539119	521	SAT1_CF	blue	na
chrUn	7539161	7540004	844	SAT1_CF	blue	na
chrUn	7540048	7541306	1259	SAT1_CF	blue	na
chrUn	7541337	7542830	1494	SAT1_CF	blue	na
chrUn	7542842	7543775	934	SAT1_CF	blue	na
chrUn	7543845	7544518	674	SAT1_CF	blue	na
chrUn	7544530	7545764	1235	SAT1_CF	blue	na
chrUn	7545792	7546423	632	SAT1_CF	blue	na
chrUn	7546466	7547141	676	SAT1_CF	blue	na
chrUn	7547153	7548271	1119	SAT1_CF	blue	na
chrUn	7548305	7548489	185	SAT1_CF	blue	na
chrUn	7548670	7549506	837	SAT1_CF	blue	na
chrUn	7549518	7550278	761	SAT1_CF	blue	na
chrUn	7550292	7550793	502	SAT1_CF	blue	na
chrUn	7550846	7551235	390	SAT1_CF	blue	na
chrUn	7551269	7551535	267	SAT1_CF	blue	na
chrUn	7551578	7552140	563	SAT1_CF	blue	na
chrUn	7552167	7552799	633	SAT1_CF	blue	na
chrUn	7552842	7553518	677	SAT1_CF	blue	na
chrUn	7553530	7554291	762	SAT1_CF	blue	na
chrUn	7554311	7554779	469	SAT1_CF	blue	na
chrUn	7554800	7554897	98	SAT1_CF	blue	na
chrUn	7554920	7556935	2016	SAT1_CF	blue	na
chrUn	7556949	7557488	540	SAT1_CF	blue	na
chrUn	7557531	7558674	1144	SAT1_CF	blue	na
chrUn	7558688	7559369	682	SAT1_CF	blue	na
chrUn	7559381	7559935	555	SAT1_CF	blue	na
chrUn	7559957	7560064	108	SAT1_CF	blue	na
chrUn	7560077	7560594	518	SAT1_CF	blue	na
chrUn	7560611	7560984	374	SAT1_CF	blue	na
chrUn	7561027	7563146	2120	SAT1_CF	blue	na
chrUn	7563159	7563668	510	SAT1_CF	blue	na
chrUn	7563682	7564558	877	SAT1_CF	blue	na
chrUn	7564606	7565287	682	SAT1_CF	blue	na
chrUn	7565298	7565791	494	SAT1_CF	blue	na
chrUn	7565836	7565991	156	SAT1_CF	blue	na
chrUn	7566204	7568105	1902	SAT1_CF	blue	na
chrUn	7568148	7569357	1210	SAT1_CF	blue	na
chrUn	7569383	7569540	158	SAT1_CF	blue	na
chrUn	7569555	7570183	629	SAT1_CF	blue	na
chrUn	7570194	7570878	685	SAT1_CF	blue	na

chrUn	7570891	7571391	501	SAT1_CF	blue	na
chrUn	7571431	7572381	951	SAT1_CF	blue	na
chrUn	7572438	7574060	1623	SAT1_CF	blue	na
chrUn	7574145	7574276	132	SAT1_CF	blue	na
chrUn	7574291	7574814	524	SAT1_CF	blue	na
chrUn	7574894	7575419	526	SAT1_CF	blue	na
chrUn	7575463	7576004	542	SAT1_CF	blue	na
chrUn	7576057	7576163	107	SAT1_CF	blue	na
chrUn	7576176	7576728	553	SAT1_CF	blue	na
chrUn	7576749	7576857	109	SAT1_CF	blue	na
chrUn	7576870	7577390	521	SAT1_CF	blue	na
chrUn	7577416	7577573	158	SAT1_CF	blue	na
chrUn	7577601	7578701	1101	SAT1_CF	blue	na
chrUn	7578724	7578829	106	SAT1_CF	blue	na
chrUn	7578842	7579342	501	SAT1_CF	blue	na
chrUn	7579380	7580317	938	SAT1_CF	blue	na
chrUn	7580335	7580532	198	SAT1_CF	blue	na
chrUn	7580656	7581198	543	SAT1_CF	blue	na
chrUn	7581226	7581270	45	SAT1_CF	blue	na
chrUn	7581283	7582133	851	SAT1_CF	blue	na
chrUn	7582145	7582705	561	SAT1_CF	blue	na
chrUn	7582726	7582834	109	SAT1_CF	blue	na
chrUn	7582847	7583640	794	SAT1_CF	blue	na
chrUn	7583653	7583776	124	SAT1_CF	blue	na
chrUn	7583799	7584991	1193	SAT1_CF	blue	na
chrUn	7585013	7585085	73	SAT1_CF	blue	na
chrUn	7585098	7586021	924	SAT1_CF	blue	na
chrUn	7586033	7586412	380	SAT1_CF	blue	na
chrUn	7586430	7588213	1784	SAT1_CF	blue	na
chrUn	7588240	7588342	103	SAT1_CF	blue	na
chrUn	7588355	7588835	481	SAT1_CF	blue	na
chrUn	7588895	7589596	702	SAT1_CF	blue	na
chrUn	7589676	7590239	564	SAT1_CF	blue	na
chrUn	7590266	7590897	632	SAT1_CF	blue	na
chrUn	7590940	7591604	665	SAT1_CF	blue	na
chrUn	7591624	7592165	542	SAT1_CF	blue	na
chrUn	7592209	7593208	1000	SAT1_CF	blue	na
chrUn	7593251	7593923	673	SAT1_CF	blue	na
chrUn	7593937	7594696	760	SAT1_CF	blue	na
chrUn	7594708	7595391	684	SAT1_CF	blue	na
chrUn	7595404	7595906	503	SAT1_CF	blue	na
chrUn	7595993	7596953	961	SAT1_CF	blue	na

chrUn	7597020	7597984	965	SAT1_CF	blue	na
chrUn	7598027	7598697	671	SAT1_CF	blue	na
chrUn	7598715	7599267	553	SAT1_CF	blue	na
chrUn	7599289	7599361	73	SAT1_CF	blue	na
chrUn	7599374	7600171	798	SAT1_CF	blue	na
chrUn	7600192	7600301	110	SAT1_CF	blue	na
chrUn	7600314	7600996	683	SAT1_CF	blue	na
chrUn	7601008	7602547	1540	SAT1_CF	blue	na
chrUn	7602558	7603317	760	SAT1_CF	blue	na
chrUn	7603330	7604030	701	SAT1_CF	blue	na
chrUn	7604242	7605446	1205	SAT1_CF	blue	na
chrUn	7605489	7607492	2004	SAT1_CF	blue	na
chrUn	7607549	7608041	493	SAT1_CF	blue	na
chrUn	7608061	7608299	239	SAT1_CF	blue	na
chrUn	7608332	7608838	507	SAT1_CF	blue	na
chrUn	7608919	7610029	1111	SAT1_CF	blue	na
chrUn	7610042	7610762	721	SAT1_CF	blue	na
chrUn	7610782	7610891	110	SAT1_CF	blue	na
chrUn	7610903	7611588	686	SAT1_CF	blue	na
chrUn	7611606	7612437	832	SAT1_CF	blue	na
chrUn	7612489	7613117	629	SAT1_CF	blue	na
chrUn	7613141	7613247	107	SAT1_CF	blue	na
chrUn	7613260	7613761	502	SAT1_CF	blue	na
chrUn	7613827	7613962	136	SAT1_CF	blue	na
chrUn	7613990	7615179	1190	SAT1_CF	blue	na
chrUn	7615223	7615763	541	SAT1_CF	blue	na
chrUn	7615860	7616537	678	SAT1_CF	blue	na
chrUn	7616549	7617102	554	SAT1_CF	blue	na
chrUn	7617123	7617226	104	SAT1_CF	blue	na
chrUn	7617244	7617742	499	SAT1_CF	blue	na
chrUn	7617808	7617944	137	SAT1_CF	blue	na
chrUn	7617976	7618480	505	SAT1_CF	blue	na
chrUn	7618514	7619203	690	SAT1_CF	blue	na
chrUn	7619226	7619297	72	SAT1_CF	blue	na
chrUn	7619311	7620056	746	SAT1_CF	blue	na
chrUn	7620082	7620563	482	SAT1_CF	blue	na
chrUn	7620670	7621025	356	SAT1_CF	blue	na
chrUn	7621059	7621545	487	SAT1_CF	blue	na
chrUn	7621579	7621858	280	SAT1_CF	blue	na
chrUn	7621900	7622457	558	SAT1_CF	blue	na
chrUn	7622499	7623044	546	SAT1_CF	blue	na
chrUn	7623101	7624058	958	SAT1_CF	blue	na

chrUn	7624101	7625989	1889	SAT1_CF	blue	na
chrUn	7626015	7626394	380	SAT1_CF	blue	na
chrUn	7626415	7626522	108	SAT1_CF	blue	na
chrUn	7626535	7627034	500	SAT1_CF	blue	na
chrUn	7627074	7627645	572	SAT1_CF	blue	na
chrUn	7627669	7628216	548	SAT1_CF	blue	na
chrUn	7628230	7628930	701	SAT1_CF	blue	na
chrUn	7628972	7629834	863	SAT1_CF	blue	na
chrUn	7629846	7630516	671	SAT1_CF	blue	na
chrUn	7630543	7631932	1390	SAT1_CF	blue	na
chrUn	7631963	7632361	399	SAT1_CF	blue	na
chrUn	7632381	7632905	525	SAT1_CF	blue	na
chrUn	7633021	7633327	307	SAT1_CF	blue	na
chrUn	7633371	7635286	1916	SAT1_CF	blue	na
chrUn	7635298	7635986	689	SAT1_CF	blue	na
chrUn	7635999	7636500	502	SAT1_CF	blue	na
chrUn	7636545	7636942	398	SAT1_CF	blue	na
chrUn	7636955	7637498	544	SAT1_CF	blue	na
chrUn	7637577	7638540	964	SAT1_CF	blue	na
chrUn	7638583	7639259	677	SAT1_CF	blue	na
chrUn	7639271	7640082	812	SAT1_CF	blue	na
chrUn	7640189	7641349	1161	SAT1_CF	blue	na
chrUn	7641362	7642337	976	SAT1_CF	blue	na
chrUn	7642352	7643069	718	SAT1_CF	blue	na
chrUn	7643080	7643777	698	SAT1_CF	blue	na
chrUn	7643825	7644365	541	SAT1_CF	blue	na
chrUn	7644407	7644931	525	SAT1_CF	blue	na
chrUn	7644949	7645852	904	SAT1_CF	blue	na
chrUn	7645894	7646060	167	SAT1_CF	blue	na
chrUn	7646078	7646754	677	SAT1_CF	blue	na
chrUn	7646766	7647397	632	SAT1_CF	blue	na
chrUn	7647418	7647527	110	SAT1_CF	blue	na
chrUn	7647539	7648088	550	SAT1_CF	blue	na
chrUn	7648099	7649260	1162	SAT1_CF	blue	na
chrUn	7649276	7649354	79	SAT1_CF	blue	na
chrUn	7649367	7650055	689	SAT1_CF	blue	na
chrUn	7650068	7650641	574	SAT1_CF	blue	na
chrUn	7650662	7650765	104	SAT1_CF	blue	na
chrUn	7650783	7651617	835	SAT1_CF	blue	na
chrUn	7651687	7652213	527	SAT1_CF	blue	na
chrUn	7652292	7652598	307	SAT1_CF	blue	na
chrUn	7652642	7653980	1339	SAT1_CF	blue	na

chrUn	7653992	7654546	555	SAT1_CF	blue	na
chrUn	7654567	7654657	91	SAT1_CF	blue	na
chrUn	7654686	7655409	724	SAT1_CF	blue	na
chrUn	7655470	7656147	678	SAT1_CF	blue	na
chrUn	7656159	7656714	556	SAT1_CF	blue	na
chrUn	7656779	7657353	575	SAT1_CF	blue	na
chrUn	7657419	7657555	137	SAT1_CF	blue	na
chrUn	7657583	7658131	549	SAT1_CF	blue	na
chrUn	7658174	7658738	565	SAT1_CF	blue	na
chrUn	7658766	7660991	2226	SAT1_CF	blue	na
chrUn	7661018	7661559	542	SAT1_CF	blue	na
chrUn	7661601	7662796	1196	SAT1_CF	blue	na
chrUn	7662808	7663492	685	SAT1_CF	blue	na
chrUn	7663512	7664006	495	SAT1_CF	blue	na
chrUn	7664044	7664619	576	SAT1_CF	blue	na
chrUn	7666017	7666558	542	SAT1_CF	blue	na
chrUn	7666608	7667279	672	SAT1_CF	blue	na
chrUn	7667357	7668212	856	SAT1_CF	blue	na
chrUn	7668224	7668906	683	SAT1_CF	blue	na
chrUn	7668919	7669601	683	SAT1_CF	blue	na
chrUn	7669614	7670114	501	SAT1_CF	blue	na
chrUn	7670156	7670558	403	SAT1_CF	blue	na
chrUn	7670569	7672901	2333	SAT1_CF	blue	na
chrUn	7672913	7673594	682	SAT1_CF	blue	na
chrUn	7673607	7674856	1250	SAT1_CF	blue	na
chrUn	7674900	7675532	633	SAT1_CF	blue	na
chrUn	7675553	7677031	1479	SAT1_CF	blue	na
chrUn	7677051	7677158	108	SAT1_CF	blue	na
chrUn	7677178	7677716	539	SAT1_CF	blue	na
chrUn	7677784	7679825	2042	SAT1_CF	blue	na
chrUn	7679868	7680536	669	SAT1_CF	blue	na
chrUn	7680549	7681249	701	SAT1_CF	blue	na
chrUn	7681264	7682457	1194	SAT1_CF	blue	na
chrUn	7682483	7682585	103	SAT1_CF	blue	na
chrUn	7682598	7683076	479	SAT1_CF	blue	na
chrUn	7683183	7684095	913	SAT1_CF	blue	na
chrUn	7684210	7685174	965	SAT1_CF	blue	na
chrUn	7685217	7685891	675	SAT1_CF	blue	na
chrUn	7685903	7686546	644	SAT1_CF	blue	na
chrUn	7688199	7688824	626	SAT1_CF	blue	na
chrUn	7688855	7690158	1304	SAT1_CF	blue	na
chrUn	7690202	7690850	649	SAT1_CF	blue	na

chrUn	7690863	7691548	686	SAT1_CF	blue	na
chrUn	7691560	7692081	522	SAT1_CF	blue	na
chrUn	7692115	7692432	318	SAT1_CF	blue	na
chrUn	7692443	7694100	1658	SAT1_CF	blue	na
chrUn	7694143	7694807	665	SAT1_CF	blue	na
chrUn	7694827	7695381	555	SAT1_CF	blue	na
chrUn	7695402	7695504	103	SAT1_CF	blue	na
chrUn	7695522	7698507	2986	SAT1_CF	blue	na
chrUn	7698520	7699040	521	SAT1_CF	blue	na
chrUn	7699087	7699223	137	SAT1_CF	blue	na
chrUn	7699275	7699761	487	SAT1_CF	blue	na
chrUn	7699805	7700355	551	SAT1_CF	blue	na
chrUn	7700394	7700556	163	SAT1_CF	blue	na
chrUn	7700583	7701098	516	SAT1_CF	blue	na
chrUn	7701168	7702028	861	SAT1_CF	blue	na
chrUn	7702040	7702558	519	SAT1_CF	blue	na
chrUn	7702584	7702740	157	SAT1_CF	blue	na
chrUn	7702771	7703276	506	SAT1_CF	blue	na
chrUn	7703306	7703919	614	SAT1_CF	blue	na
chrUn	7703947	7704579	633	SAT1_CF	blue	na
chrUn	7704622	7705287	666	SAT1_CF	blue	na
chrUn	7705299	7705773	475	SAT1_CF	blue	na
chrUn	7705843	7706515	673	SAT1_CF	blue	na
chrUn	7706527	7707286	760	SAT1_CF	blue	na
chrUn	7707298	7707979	682	SAT1_CF	blue	na
chrUn	7707993	7708494	502	SAT1_CF	blue	na
chrUn	7708540	7709492	953	SAT1_CF	blue	na
chrUn	7709536	7710532	997	SAT1_CF	blue	na
chrUn	7710575	7711122	548	SAT1_CF	blue	na
chrUn	7711179	7712165	987	SAT1_CF	blue	na
chrUn	7712207	7712883	677	SAT1_CF	blue	na
chrUn	7712895	7713578	684	SAT1_CF	blue	na
chrUn	7713598	7714091	494	SAT1_CF	blue	na
chrUn	7714131	7714702	572	SAT1_CF	blue	na
chrUn	7716101	7717576	1476	SAT1_CF	blue	na
chrUn	7717619	7718987	1369	SAT1_CF	blue	na
chrUn	7719007	7719499	493	SAT1_CF	blue	na
chrUn	7719544	7719701	158	SAT1_CF	blue	na
chrUn	7719729	7720238	510	SAT1_CF	blue	na
chrUn	7720285	7720922	638	SAT1_CF	blue	na
chrUn	7720966	7722300	1335	SAT1_CF	blue	na
chrUn	7722312	7723065	754	SAT1_CF	blue	na

chrUn	7723083	7723584	502	SAT1_CF	blue	na
chrUn	7723650	7723784	135	SAT1_CF	blue	na
chrUn	7723800	7726892	3093	SAT1_CF	blue	na
chrUn	7726936	7727727	792	SAT1_CF	blue	na
chrUn	7727769	7728628	860	SAT1_CF	blue	na
chrUn	7728640	7729159	520	SAT1_CF	blue	na
chrUn	7729181	7729246	66	SAT1_CF	blue	na
chrUn	7729266	7730012	747	SAT1_CF	blue	na
chrUn	7730039	7730540	502	SAT1_CF	blue	na
chrUn	7730580	7730959	380	SAT1_CF	blue	na
chrUn	7730992	7731724	733	SAT1_CF	blue	na
chrUn	7731756	7731850	95	SAT1_CF	blue	na
chrUn	7732090	7732224	135	SAT1_CF	blue	na
chrUn	7732241	7733284	1044	SAT1_CF	blue	na
chrUn	7733295	7733849	555	SAT1_CF	blue	na
chrUn	7733869	7733978	110	SAT1_CF	blue	na
chrUn	7733990	7734539	550	SAT1_CF	blue	na
chrUn	7734550	7735178	629	SAT1_CF	blue	na
chrUn	7735190	7735836	647	SAT1_CF	blue	na
chrUn	7735849	7736611	763	SAT1_CF	blue	na
chrUn	7736623	7737237	615	SAT1_CF	blue	na
chrUn	7737275	7738422	1148	SAT1_CF	blue	na
chrUn	7738444	7739126	683	SAT1_CF	blue	na
chrUn	7739139	7739718	580	SAT1_CF	blue	na
chrUn	7739797	7740335	539	SAT1_CF	blue	na
chrUn	7740425	7741278	854	SAT1_CF	blue	na
chrUn	7741301	7741856	556	SAT1_CF	blue	na
chrUn	7741882	7741985	104	SAT1_CF	blue	na
chrUn	7741998	7742500	503	SAT1_CF	blue	na
chrUn	7742553	7742871	319	SAT1_CF	blue	na
chrUn	7742882	7743661	780	SAT1_CF	blue	na
chrUn	7743742	7744395	654	SAT1_CF	blue	na
chrUn	7744407	7745090	684	SAT1_CF	blue	na
chrUn	7745103	7745994	892	SAT1_CF	blue	na
chrUn	7746006	7747762	1757	SAT1_CF	blue	na
chrUn	7747778	7748431	654	SAT1_CF	blue	na
chrUn	7748474	7749120	647	SAT1_CF	blue	na
chrUn	7749133	7750201	1069	SAT1_CF	blue	na
chrUn	7750213	7751691	1479	SAT1_CF	blue	na
chrUn	7751705	7752196	492	SAT1_CF	blue	na
chrUn	7752207	7753838	1632	SAT1_CF	blue	na
chrUn	7753881	7754584	704	SAT1_CF	blue	na

chrUn	7754611	7755132	522	SAT1_CF	blue	na
chrUn	7755179	7755315	137	SAT1_CF	blue	na
chrUn	7755330	7757194	1865	SAT1_CF	blue	na
chrUn	7757237	7757900	664	SAT1_CF	blue	na
chrUn	7757923	7759117	1195	SAT1_CF	blue	na
chrUn	7759170	7759730	561	SAT1_CF	blue	na
chrUn	7761147	7763554	2408	SAT1_CF	blue	na
chrUn	7763566	7764240	675	SAT1_CF	blue	na
chrUn	7764269	7764762	494	SAT1_CF	blue	na
chrUn	7764805	7765196	392	SAT1_CF	blue	na
chrUn	7765211	7765733	523	SAT1_CF	blue	na
chrUn	7765751	7766034	284	SAT1_CF	blue	na
chrUn	7766111	7766653	543	SAT1_CF	blue	na
chrUn	7766695	7767884	1190	SAT1_CF	blue	na
chrUn	7767971	7768872	902	SAT1_CF	blue	na
chrUn	7768962	7769926	965	SAT1_CF	blue	na
chrUn	7769969	7770486	518	SAT1_CF	blue	na
chrUn	7770540	7771109	570	SAT1_CF	blue	na
chrUn	7771122	7773929	2808	SAT1_CF	blue	na
chrUn	7773953	7774506	554	SAT1_CF	blue	na
chrUn	7774532	7774635	104	SAT1_CF	blue	na
chrUn	7774648	7775168	521	SAT1_CF	blue	na
chrUn	7775189	7776148	960	SAT1_CF	blue	na
chrUn	7776192	7777266	1075	SAT1_CF	blue	na
chrUn	7777310	7777984	675	SAT1_CF	blue	na
chrUn	7778011	7778601	591	SAT1_CF	blue	na
chrUn	7778628	7779231	604	SAT1_CF	blue	na
chrUn	7779243	7779927	685	SAT1_CF	blue	na
chrUn	7779938	7780474	537	SAT1_CF	blue	na
chrUn	7780502	7781132	631	SAT1_CF	blue	na
chrUn	7781174	7781836	663	SAT1_CF	blue	na
chrUn	7781858	7782360	503	SAT1_CF	blue	na
chrUn	7782409	7783248	840	SAT1_CF	blue	na
chrUn	7783279	7785540	2262	SAT1_CF	blue	na
chrUn	7785627	7786251	625	SAT1_CF	blue	na
chrUn	7786294	7787154	861	SAT1_CF	blue	na
chrUn	7787166	7787685	520	SAT1_CF	blue	na
chrUn	7787707	7787772	66	SAT1_CF	blue	na
chrUn	7787792	7788538	747	SAT1_CF	blue	na
chrUn	7788565	7789066	502	SAT1_CF	blue	na
chrUn	7789153	7790924	1772	SAT1_CF	blue	na
chrUn	7790966	7791513	548	SAT1_CF	blue	na

chrUn	7791570	7792089	520	SAT1_CF	blue	na
chrUn	7792184	7792320	137	SAT1_CF	blue	na
chrUn	7792335	7792860	526	SAT1_CF	blue	na
chrUn	7792888	7793393	506	SAT1_CF	blue	na
chrUn	7793435	7794045	611	SAT1_CF	blue	na
chrUn	7794063	7794913	851	SAT1_CF	blue	na
chrUn	7794925	7795611	687	SAT1_CF	blue	na
chrUn	7795629	7796173	545	SAT1_CF	blue	na
chrUn	7796216	7796763	548	SAT1_CF	blue	na
chrUn	7796819	7797338	520	SAT1_CF	blue	na
chrUn	7797381	7798552	1172	SAT1_CF	blue	na
chrUn	7798596	7799324	729	SAT1_CF	blue	na
chrUn	7799702	7800745	1044	SAT1_CF	blue	na
chrUn	7800823	7801147	325	SAT1_CF	blue	na
chrUn	7801208	7801873	666	SAT1_CF	blue	na
chrUn	7801893	7802576	684	SAT1_CF	blue	na
chrUn	7802589	7803480	892	SAT1_CF	blue	na
chrUn	7803492	7804249	758	SAT1_CF	blue	na
chrUn	7804262	7804762	501	SAT1_CF	blue	na
chrUn	7804828	7804964	137	SAT1_CF	blue	na
chrUn	7805016	7805501	486	SAT1_CF	blue	na
chrUn	7805582	7806113	532	SAT1_CF	blue	na
chrUn	7806246	7806790	545	SAT1_CF	blue	na
chrUn	7806832	7807378	547	SAT1_CF	blue	na
chrUn	7807465	7808353	889	SAT1_CF	blue	na
chrUn	7808386	7809979	1594	SAT1_CF	blue	na
chrUn	7810002	7810073	72	SAT1_CF	blue	na
chrUn	7810086	7810717	632	SAT1_CF	blue	na
chrUn	7810743	7810846	104	SAT1_CF	blue	na
chrUn	7810859	7811358	500	SAT1_CF	blue	na
chrUn	7811398	7812492	1095	SAT1_CF	blue	na
chrUn	7812517	7813019	503	SAT1_CF	blue	na
chrUn	7813072	7814016	945	SAT1_CF	blue	na
chrUn	7814029	7815079	1051	SAT1_CF	blue	na
chrUn	7815110	7815603	494	SAT1_CF	blue	na
chrUn	7815747	7817839	2093	SAT1_CF	blue	na
chrUn	7818446	7818554	109	SAT1_CF	blue	na
chrUn	7818566	7819943	1378	SAT1_CF	blue	na
chrUn	7819963	7820456	494	SAT1_CF	blue	na
chrUn	7820498	7820900	403	SAT1_CF	blue	na
chrUn	7820911	7821455	545	SAT1_CF	blue	na
chrUn	7821499	7822527	1029	SAT1_CF	blue	na

chrUn	7822570	7823245	676	SAT1_CF	blue	na
chrUn	7823258	7823759	502	SAT1_CF	blue	na
chrUn	7823793	7824170	378	SAT1_CF	blue	na
chrUn	7824218	7824766	549	SAT1_CF	blue	na
chrUn	7824827	7824951	125	SAT1_CF	blue	na
chrUn	7824964	7825582	619	SAT1_CF	blue	na
chrUn	7825596	7826340	745	SAT1_CF	blue	na
chrUn	7826367	7826848	482	SAT1_CF	blue	na
chrUn	7826908	7827310	403	SAT1_CF	blue	na
chrUn	7827321	7827830	510	SAT1_CF	blue	na
chrUn	7827910	7829243	1334	SAT1_CF	blue	na
chrUn	7829300	7829848	549	SAT1_CF	blue	na
chrUn	7829935	7830284	350	SAT1_CF	blue	na
chrUn	7830297	7831654	1358	SAT1_CF	blue	na
chrUn	7831666	7832184	519	SAT1_CF	blue	na
chrUn	7832210	7832589	380	SAT1_CF	blue	na
chrUn	7832612	7832717	106	SAT1_CF	blue	na
chrUn	7832730	7833248	519	SAT1_CF	blue	na
chrUn	7833295	7833431	137	SAT1_CF	blue	na
chrUn	7833459	7833969	511	SAT1_CF	blue	na
chrUn	7834048	7836180	2133	SAT1_CF	blue	na
chrUn	7836193	7837175	983	SAT1_CF	blue	na
chrUn	7837196	7837305	110	SAT1_CF	blue	na
chrUn	7837317	7837819	503	SAT1_CF	blue	na
chrUn	7837859	7838878	1020	SAT1_CF	blue	na
chrUn	7838933	7839898	966	SAT1_CF	blue	na
chrUn	7839941	7840603	663	SAT1_CF	blue	na
chrUn	7840630	7841114	485	SAT1_CF	blue	na
chrUn	7841140	7842484	1345	SAT1_CF	blue	na
chrUn	7842504	7843026	523	SAT1_CF	blue	na
chrUn	7843142	7844108	967	SAT1_CF	blue	na
chrUn	7844151	7844819	669	SAT1_CF	blue	na
chrUn	7844839	7845333	495	SAT1_CF	blue	na
chrUn	7845371	7846874	1504	SAT1_CF	blue	na
chrUn	7846917	7847591	675	SAT1_CF	blue	na
chrUn	7847603	7848286	684	SAT1_CF	blue	na
chrUn	7848300	7848780	481	SAT1_CF	blue	na
chrUn	7848887	7849401	515	SAT1_CF	blue	na
chrUn	7849430	7850239	810	SAT1_CF	blue	na
chrUn	7850264	7851594	1331	SAT1_CF	blue	na
chrUn	7851606	7852252	647	SAT1_CF	blue	na
chrUn	7852265	7852811	547	SAT1_CF	blue	na

chrUn	7852841	7853543	703	SAT1_CF	blue	na
chrUn	7853597	7853724	128	SAT1_CF	blue	na
chrUn	7853768	7854309	542	SAT1_CF	blue	na
chrUn	7854406	7856272	1867	SAT1_CF	blue	na
chrUn	7856293	7856692	400	SAT1_CF	blue	na
chrUn	7856705	7858420	1716	SAT1_CF	blue	na
chrUn	7858465	7858622	158	SAT1_CF	blue	na
chrUn	7858637	7859161	525	SAT1_CF	blue	na
chrUn	7859241	7859916	676	SAT1_CF	blue	na
chrUn	7859930	7861126	1197	SAT1_CF	blue	na
chrUn	7861139	7862348	1210	SAT1_CF	blue	na
chrUn	7862414	7863081	668	SAT1_CF	blue	na
chrUn	7863123	7863470	348	SAT1_CF	blue	na
chrUn	7863508	7864052	545	SAT1_CF	blue	na
chrUn	7864102	7865365	1264	SAT1_CF	blue	na
chrUn	7865377	7866087	711	SAT1_CF	blue	na
chrUn	7866113	7866216	104	SAT1_CF	blue	na
chrUn	7866229	7866731	503	SAT1_CF	blue	na
chrUn	7866784	7867102	319	SAT1_CF	blue	na
chrUn	7867115	7867656	542	SAT1_CF	blue	na
chrUn	7867667	7867893	227	SAT1_CF	blue	na
chrUn	7867974	7869319	1346	SAT1_CF	blue	na
chrUn	7869332	7870187	856	SAT1_CF	blue	na
chrUn	7870199	7871685	1487	SAT1_CF	blue	na
chrUn	7871711	7872694	984	SAT1_CF	blue	na
chrUn	7872737	7873412	676	SAT1_CF	blue	na
chrUn	7873424	7874051	628	SAT1_CF	blue	na
chrUn	7874096	7874620	525	SAT1_CF	blue	na
chrUn	7874647	7875214	568	SAT1_CF	blue	na
chrUn	7875234	7881059	5826	CarSat1	red	na
chrUn	7881080	7881618	539	CarSat1	red	na
chrUn	7881802	7884883	3082	CarSat1	red	na
chrUn	7886045	7887170	1126	CarSat1	red	na
chrUn	7887197	7887907	711	CarSat1	red	na
chrUn	7887934	7888718	785	CarSat1	red	na
chrUn	7888973	7890119	1147	CarSat1	red	na
chrUn	7890146	7890859	714	CarSat1	red	na
chrUn	7890884	7892917	2034	CarSat1	red	na
chrUn	7892929	7895541	2613	CarSat1	red	na
chrUn	7897126	7897555	430	CarSat1	red	na
chrUn	7897573	7898291	719	CarSat1	red	na
chrUn	7898286	7898898	613	SAT1_CF	blue	na

chrUn	7898918	7899437	520	SAT1_CF	blue	na
chrUn	7899462	7900084	623	SAT1_CF	blue	na
chrUn	7900127	7902529	2403	SAT1_CF	blue	na
chrUn	7902531	7904476	1946	CarSat1	red	na
chrUn	7904502	7906091	1590	CarSat1	red	na
chrUn	7906192	7906816	625	CarSat1	red	na
chrUn	7921941	7922099	159	Sat2CF	purple	na
chrUn	7921962	7922655	694	Sat2CF	purple	na
chrUn	7922549	7922697	149	Sat2CF	purple	na
chrUn	7922549	7922697	149	Sat2CF	purple	na
chrUn	7953011	7954160	1150	Sat2CF	purple	na
chrUn	7953145	7954138	994	Sat2CF	purple	na
chrUn	7974630	7974792	163	Sat2CF	purple	na
chrUn	7974658	7975373	716	Sat2CF	purple	na
chrUn	7975247	7975395	149	Sat2CF	purple	na
chrUn	7975247	7975395	149	Sat2CF	purple	na
chrUn	7991856	7993024	1169	Sat2CF	purple	na
chrUn	7992846	7993055	210	Sat2CF	purple	na
chrUn	8007741	8008528	788	Sat2CF	purple	na
chrUn	8020422	8021598	1177	Sat2CF	purple	na
chrUn	8047292	8047537	246	Sat2CF	purple	na
chrUn	8048103	8048243	141	Sat2CF	purple	na
chrUn	8066287	8067422	1136	Sat2CF	purple	na
chrUn	8086534	8087452	919	Sat2CF	purple	na
chrUn	8086670	8087406	737	Sat2CF	purple	na
chrUn	8127051	8128268	1218	Sat2CF	purple	na
chrUn	8127179	8128263	1085	Sat2CF	purple	na
chrUn	8128139	8128288	150	Sat2CF	purple	na
chrUn	8149921	8150658	738	Sat2CF	purple	na
chrUn	8149995	8150656	662	Sat2CF	purple	na
chrUn	8206904	8207066	163	Sat2CF	purple	na
chrUn	8206930	8207778	849	Sat2CF	purple	na
chrUn	8207111	8207859	749	Sat2CF	purple	na
chrUn	8225662	8226839	1178	Sat2CF	purple	na
chrUn	8226682	8226830	149	Sat2CF	purple	na
chrUn	8266007	8267234	1228	Sat2CF	purple	na
chrUn	8266140	8267162	1023	Sat2CF	purple	na
chrUn	8266072	8266373	302	Sat2CF	purple	na
chrUn	8285554	8286362	809	Sat2CF	purple	na
chrUn	8286590	8286721	132	Sat2CF	purple	na
chrUn	8286592	8286752	161	Sat2CF	purple	na
chrUn	8286595	8286795	201	Sat2CF	purple	na

chrUn	8315790	8316967	1178	Sat2CF	purple	na
chrUn	8330589	8331662	1074	Sat2CF	purple	na
chrUn	8358161	8359314	1154	Sat2CF	purple	na
chrUn	8384166	8385383	1218	Sat2CF	purple	na
chrUn	8415201	8416115	915	Sat2CF	purple	na
chrUn	8415268	8416077	810	Sat2CF	purple	na
chrUn	8444758	8445969	1212	Sat2CF	purple	na
chrUn	8445811	8445990	180	Sat2CF	purple	na
chrUn	8461958	8462606	649	Sat2CF	purple	na
chrUn	8462032	8462695	664	Sat2CF	purple	na
chrUn	8480907	8481792	886	Sat2CF	purple	na
chrUn	8481042	8481776	735	Sat2CF	purple	na
chrUn	8497123	8497285	163	Sat2CF	purple	na
chrUn	8497151	8497933	783	Sat2CF	purple	na
chrUn	8506153	8507229	1077	Sat2CF	purple	na
chrUn	8507106	8507254	149	Sat2CF	purple	na
chrUn	8520227	8521457	1231	Sat2CF	purple	na
chrUn	8568093	8569272	1180	Sat2CF	purple	na
chrUn	8592442	8592786	345	Sat2CF	purple	na
chrUn	8592501	8593291	791	Sat2CF	purple	na
chrUn	8592965	8593328	364	Sat2CF	purple	na
chrUn	8612332	8613465	1134	Sat2CF	purple	na
chrUn	8651540	8652772	1233	Sat2CF	purple	na
chrUn	8651650	8652736	1087	Sat2CF	purple	na
chrUn	8700250	8701069	820	Sat2CF	purple	na
chrUn	8700328	8701048	721	Sat2CF	purple	na
chrUn	8700939	8701106	168	Sat2CF	purple	na
chrUn	8724849	8726081	1233	Sat2CF	purple	na
chrUn	8741103	8742236	1134	Sat2CF	purple	na
chrUn	8741235	8742324	1090	Sat2CF	purple	na
chrUn	8742197	8742343	147	Sat2CF	purple	na
chrUn	8769635	8770879	1245	SAT1_CF	blue	na
chrUn	8770915	8771433	519	SAT1_CF	blue	na
chrUn	8771446	8771949	504	SAT1_CF	blue	na
chrUn	8771967	8774007	2041	SAT1_CF	blue	na
chrUn	8850780	8850852	73	Carsat3	green	na
chrUn	9235036	9236134	1099	Sat7CF	lime	na
chrUn	9275813	9276769	957	Sat7CF	lime	na
chrUn	9602218	9603180	963	SAT1_CF	blue	na
chrUn	9603223	9603902	680	SAT1_CF	blue	na
chrUn	9603914	9606071	2158	SAT1_CF	blue	na
chrUn	9606098	9607570	1473	SAT1_CF	blue	na

chrUn	9607619	9607786	168	SAT1_CF	blue	na
chrUn	9607798	9608473	676	SAT1_CF	blue	na
chrUn	9608485	9608699	215	SAT1_CF	blue	na
chrUn	9608898	9611216	2319	SAT1_CF	blue	na
chrUn	9611228	9611786	559	SAT1_CF	blue	na
chrUn	9611806	9611914	109	SAT1_CF	blue	na
chrUn	9611927	9612447	521	SAT1_CF	blue	na
chrUn	9612464	9612649	186	SAT1_CF	blue	na
chrUn	9612831	9612996	166	SAT1_CF	blue	na
chrUn	9613038	9614474	1437	SAT1_CF	blue	na
chrUn	9614554	9615645	1092	SAT1_CF	blue	na
chrUn	9615689	9616363	675	SAT1_CF	blue	na
chrUn	9616375	9617058	684	SAT1_CF	blue	na
chrUn	9617072	9617561	490	SAT1_CF	blue	na
chrUn	9617601	9618983	1383	SAT1_CF	blue	na
chrUn	9619027	9619669	643	SAT1_CF	blue	na
chrUn	9619683	9620221	539	SAT1_CF	blue	na
chrUn	9620232	9620356	125	SAT1_CF	blue	na
chrUn	9620368	9621051	684	SAT1_CF	blue	na
chrUn	9621065	9621557	493	SAT1_CF	blue	na
chrUn	9621568	9623948	2381	SAT1_CF	blue	na
chrUn	9623970	9624491	522	SAT1_CF	blue	na
chrUn	9624538	9624674	137	SAT1_CF	blue	na
chrUn	9624702	9625214	513	SAT1_CF	blue	na
chrUn	9625232	9625895	664	SAT1_CF	blue	na
chrUn	9625923	9626552	630	SAT1_CF	blue	na
chrUn	9626595	9627261	667	SAT1_CF	blue	na
chrUn	9627284	9627966	683	SAT1_CF	blue	na
chrUn	9627986	9628479	494	SAT1_CF	blue	na
chrUn	9628532	9629092	561	SAT1_CF	blue	na
chrUn	9630492	9631086	595	SAT1_CF	blue	na
chrUn	9631135	9632154	1020	SAT1_CF	blue	na
chrUn	9632176	9632676	501	SAT1_CF	blue	na
chrUn	9632719	9633197	479	SAT1_CF	blue	na
chrUn	9633487	9634071	585	SAT1_CF	blue	na
chrUn	9634098	9635112	1015	SAT1_CF	blue	na
chrUn	9635125	9635607	483	SAT1_CF	blue	na
chrUn	9635667	9636963	1297	SAT1_CF	blue	na
chrUn	9636978	9637522	545	SAT1_CF	blue	na
chrUn	9637563	9639067	1505	SAT1_CF	blue	na
chrUn	9639088	9639196	109	SAT1_CF	blue	na
chrUn	9639210	9639689	480	SAT1_CF	blue	na

chrUn	9639745	9640115	371	SAT1_CF	blue	na
chrUn	9640160	9641055	896	SAT1_CF	blue	na
chrUn	9641068	9642376	1309	SAT1_CF	blue	na
chrUn	9642396	9642505	110	SAT1_CF	blue	na
chrUn	9642517	9643202	686	SAT1_CF	blue	na
chrUn	9643214	9643733	520	SAT1_CF	blue	na
chrUn	9643752	9644237	486	SAT1_CF	blue	na
chrUn	9644255	9644899	645	SAT1_CF	blue	na
chrUn	9644958	9646163	1206	SAT1_CF	blue	na
chrUn	9646207	9646751	545	SAT1_CF	blue	na
chrUn	9646800	9646967	168	SAT1_CF	blue	na
chrUn	9646979	9647650	672	SAT1_CF	blue	na
chrUn	9647663	9648144	482	SAT1_CF	blue	na
chrUn	9648170	9649033	864	SAT1_CF	blue	na
chrUn	9649073	9650032	960	SAT1_CF	blue	na
chrUn	9650111	9651150	1040	SAT1_CF	blue	na
chrUn	9651199	9651867	669	SAT1_CF	blue	na
chrUn	9651894	9652482	589	SAT1_CF	blue	na
chrUn	9652509	9653112	604	SAT1_CF	blue	na
chrUn	9653124	9654219	1096	SAT1_CF	blue	na
chrUn	9654261	9654935	675	SAT1_CF	blue	na
chrUn	9654947	9655467	521	SAT1_CF	blue	na
chrUn	9655488	9657270	1783	SAT1_CF	blue	na
chrUn	9657312	9658042	731	SAT1_CF	blue	na
chrUn	9658065	9658882	818	SAT1_CF	blue	na
chrUn	9659108	9659712	605	SAT1_CF	blue	na
chrUn	9659725	9660476	752	SAT1_CF	blue	na
chrUn	9660498	9661079	582	SAT1_CF	blue	na
chrUn	9661091	9661555	465	SAT1_CF	blue	na
chrUn	9661566	9662248	683	SAT1_CF	blue	na
chrUn	9662271	9662741	471	SAT1_CF	blue	na
chrUn	9662816	9662983	168	SAT1_CF	blue	na
chrUn	9663164	9663956	793	SAT1_CF	blue	na
chrUn	9664000	9664785	786	SAT1_CF	blue	na
chrUn	9664827	9665053	227	SAT1_CF	blue	na
chrUn	9665066	9665732	667	SAT1_CF	blue	na
chrUn	9665759	9666260	502	SAT1_CF	blue	na
chrUn	9666296	9669060	2765	SAT1_CF	blue	na
chrUn	9669072	9669574	503	SAT1_CF	blue	na
chrUn	9669619	9669776	158	SAT1_CF	blue	na
chrUn	9669827	9670313	487	SAT1_CF	blue	na
chrUn	9670344	9671803	1460	SAT1_CF	blue	na

chrUn	9671815	9672368	554	SAT1_CF	blue	na
chrUn	9672389	9672496	108	SAT1_CF	blue	na
chrUn	9672509	9673008	500	SAT1_CF	blue	na
chrUn	9673051	9675769	2719	SAT1_CF	blue	na
chrUn	9675781	9676336	556	SAT1_CF	blue	na
chrUn	9676359	9677176	818	SAT1_CF	blue	na
chrUn	9677402	9678006	605	SAT1_CF	blue	na
chrUn	9678019	9678762	744	SAT1_CF	blue	na
chrUn	9678792	9679373	582	SAT1_CF	blue	na
chrUn	9679385	9679849	465	SAT1_CF	blue	na
chrUn	9679860	9680542	683	SAT1_CF	blue	na
chrUn	9680565	9681035	471	SAT1_CF	blue	na
chrUn	9681096	9681277	182	SAT1_CF	blue	na
chrUn	9681458	9683042	1585	SAT1_CF	blue	na
chrUn	9683084	9683310	227	SAT1_CF	blue	na
chrUn	9683323	9683989	667	SAT1_CF	blue	na
chrUn	9684016	9684284	269	SAT1_CF	blue	na
chrUn	9684314	9684385	72	SAT1_CF	blue	na
chrUn	9684397	9685070	674	SAT1_CF	blue	na
chrUn	9685082	9685767	686	SAT1_CF	blue	na
chrUn	9685779	9686332	554	SAT1_CF	blue	na
chrUn	9686411	9686771	361	SAT1_CF	blue	na
chrUn	9686796	9687338	543	SAT1_CF	blue	na
chrUn	9687435	9687982	548	SAT1_CF	blue	na
chrUn	9688039	9688563	525	SAT1_CF	blue	na
chrUn	9688586	9689733	1148	SAT1_CF	blue	na
chrUn	9689776	9692156	2381	SAT1_CF	blue	na
chrUn	9692169	9692688	520	SAT1_CF	blue	na
chrUn	9692709	9692890	182	SAT1_CF	blue	na
chrUn	9693072	9693928	857	SAT1_CF	blue	na
chrUn	9693985	9695108	1124	SAT1_CF	blue	na
chrUn	9695121	9695874	754	SAT1_CF	blue	na
chrUn	9695896	9696396	501	SAT1_CF	blue	na
chrUn	9696443	9696600	158	SAT1_CF	blue	na
chrUn	9696622	9697809	1188	SAT1_CF	blue	na
chrUn	9697843	9698881	1039	SAT1_CF	blue	na
chrUn	9698903	9699006	104	SAT1_CF	blue	na
chrUn	9699019	9699719	701	SAT1_CF	blue	na
chrUn	9699734	9700259	526	SAT1_CF	blue	na
chrUn	9700302	9700828	527	SAT1_CF	blue	na
chrUn	9700872	9701413	542	SAT1_CF	blue	na
chrUn	9701455	9702305	851	SAT1_CF	blue	na

chrUn	9702317	9702793	477	SAT1_CF	blue	na
chrUn	9702863	9703536	674	SAT1_CF	blue	na
chrUn	9703548	9704983	1436	SAT1_CF	blue	na
chrUn	9704995	9705698	704	SAT1_CF	blue	na
chrUn	9705711	9706546	836	SAT1_CF	blue	na
chrUn	9706559	9707875	1317	SAT1_CF	blue	na
chrUn	9707887	9708401	515	SAT1_CF	blue	na
chrUn	9708427	9708962	536	SAT1_CF	blue	na
chrUn	9709007	9709733	727	SAT1_CF	blue	na
chrUn	9709776	9710452	677	SAT1_CF	blue	na
chrUn	9710464	9711065	602	SAT1_CF	blue	na
chrUn	9711079	9711757	679	SAT1_CF	blue	na
chrUn	9711769	9713198	1430	SAT1_CF	blue	na
chrUn	9713265	9714229	965	SAT1_CF	blue	na
chrUn	9714249	9714818	570	SAT1_CF	blue	na
chrUn	9714840	9715644	805	SAT1_CF	blue	na
chrUn	9715656	9716158	503	SAT1_CF	blue	na
chrUn	9716203	9716360	158	SAT1_CF	blue	na
chrUn	9716375	9716899	525	SAT1_CF	blue	na
chrUn	9716979	9717542	564	SAT1_CF	blue	na
chrUn	9717586	9718920	1335	SAT1_CF	blue	na
chrUn	9718932	9719485	554	SAT1_CF	blue	na
chrUn	9719506	9719614	109	SAT1_CF	blue	na
chrUn	9719634	9720128	495	SAT1_CF	blue	na
chrUn	9720185	9720745	561	SAT1_CF	blue	na
chrUn	9722156	9723215	1060	SAT1_CF	blue	na
chrUn	9723250	9723910	661	SAT1_CF	blue	na
chrUn	9723952	9724478	527	SAT1_CF	blue	na
chrUn	9724493	9726354	1862	SAT1_CF	blue	na
chrUn	9726398	9727075	678	SAT1_CF	blue	na
chrUn	9727087	9727589	503	SAT1_CF	blue	na
chrUn	9727634	9727791	158	SAT1_CF	blue	na
chrUn	9727842	9728328	487	SAT1_CF	blue	na
chrUn	9728359	9729818	1460	SAT1_CF	blue	na
chrUn	9729830	9730385	556	SAT1_CF	blue	na
chrUn	9730406	9730513	108	SAT1_CF	blue	na
chrUn	9730526	9731025	500	SAT1_CF	blue	na
chrUn	9731112	9733825	2714	SAT1_CF	blue	na
chrUn	9733837	9734444	608	SAT1_CF	blue	na
chrUn	9734456	9735296	841	SAT1_CF	blue	na
chrUn	9735309	9735809	501	SAT1_CF	blue	na
chrUn	9735847	9737014	1168	SAT1_CF	blue	na

chrUn	9737025	9737545	521	SAT1_CF	blue	na
chrUn	9737564	9737823	260	SAT1_CF	blue	na
chrUn	9737868	9738430	563	SAT1_CF	blue	na
chrUn	9738474	9739732	1259	SAT1_CF	blue	na
chrUn	9739811	9741219	1409	SAT1_CF	blue	na
chrUn	9741231	9741745	515	SAT1_CF	blue	na
chrUn	9741766	9741839	74	SAT1_CF	blue	na
chrUn	9741852	9742553	702	SAT1_CF	blue	na
chrUn	9742568	9743092	525	SAT1_CF	blue	na
chrUn	9743171	9745039	1869	SAT1_CF	blue	na
chrUn	9745060	9745167	108	SAT1_CF	blue	na
chrUn	9745180	9745679	500	SAT1_CF	blue	na
chrUn	9745766	9748442	2677	SAT1_CF	blue	na
chrUn	9748454	9749061	608	SAT1_CF	blue	na
chrUn	9749073	9750401	1329	SAT1_CF	blue	na
chrUn	9750480	9752200	1721	SAT1_CF	blue	na
chrUn	9752212	9752896	685	SAT1_CF	blue	na
chrUn	9752909	9753409	501	SAT1_CF	blue	na
chrUn	9753447	9754614	1168	SAT1_CF	blue	na
chrUn	9754625	9755145	521	SAT1_CF	blue	na
chrUn	9755164	9755423	260	SAT1_CF	blue	na
chrUn	9755468	9756152	685	SAT1_CF	blue	na
chrUn	9756163	9757613	1451	SAT1_CF	blue	na
chrUn	9757655	9758496	842	SAT1_CF	blue	na
chrUn	9758509	9759063	555	SAT1_CF	blue	na
chrUn	9759084	9759192	109	SAT1_CF	blue	na
chrUn	9759205	9759707	503	SAT1_CF	blue	na
chrUn	9759747	9760666	920	SAT1_CF	blue	na
chrUn	9760710	9762356	1647	SAT1_CF	blue	na
chrUn	9762369	9762998	630	SAT1_CF	blue	na
chrUn	9763021	9763127	107	SAT1_CF	blue	na
chrUn	9763140	9763976	837	SAT1_CF	blue	na
chrUn	9763988	9765392	1405	SAT1_CF	blue	na
chrUn	9765422	9766332	911	SAT1_CF	blue	na
chrUn	9766344	9766967	624	SAT1_CF	blue	na
chrUn	9767053	9767518	466	SAT1_CF	blue	na
chrUn	9767544	9768151	608	SAT1_CF	blue	na
chrUn	9768162	9768960	799	SAT1_CF	blue	na
chrUn	9768972	9770169	1198	SAT1_CF	blue	na
chrUn	9770209	9772280	2072	SAT1_CF	blue	na
chrUn	9772328	9772988	661	SAT1_CF	blue	na
chrUn	9773022	9773515	494	SAT1_CF	blue	na

chrUn	9773551	9773924	374	SAT1_CF	blue	na
chrUn	9773967	9775583	1617	SAT1_CF	blue	na
chrUn	9775595	9776417	823	SAT1_CF	blue	na
chrUn	9776429	9777114	686	SAT1_CF	blue	na
chrUn	9777126	9777589	464	SAT1_CF	blue	na
chrUn	9777659	9778328	670	SAT1_CF	blue	na
chrUn	9778346	9778845	500	SAT1_CF	blue	na
chrUn	9778932	9779818	887	SAT1_CF	blue	na
chrUn	9779839	9780264	426	SAT1_CF	blue	na
chrUn	9780306	9781637	1332	SAT1_CF	blue	na
chrUn	9781649	9782332	684	SAT1_CF	blue	na
chrUn	9782346	9783405	1060	SAT1_CF	blue	na
chrUn	9783434	9784200	767	SAT1_CF	blue	na
chrUn	9784304	9785603	1300	SAT1_CF	blue	na
chrUn	9785615	9786168	554	SAT1_CF	blue	na
chrUn	9786190	9786297	108	SAT1_CF	blue	na
chrUn	9786310	9787015	706	SAT1_CF	blue	na
chrUn	9787043	9790365	3323	SAT1_CF	blue	na
chrUn	9790385	9791515	1131	SAT1_CF	blue	na
chrUn	9791536	9792467	932	SAT1_CF	blue	na
chrUn	9792489	9793391	903	SAT1_CF	blue	na
chrUn	9793433	9795048	1616	SAT1_CF	blue	na
chrUn	9795104	9795703	600	SAT1_CF	blue	na
chrUn	9795721	9796223	503	SAT1_CF	blue	na
chrUn	9796268	9798119	1852	SAT1_CF	blue	na
chrUn	9798161	9799322	1162	SAT1_CF	blue	na
chrUn	9799335	9800395	1061	SAT1_CF	blue	na
chrUn	9800407	9800708	302	SAT1_CF	blue	na
chrUn	9800754	9801300	547	SAT1_CF	blue	na
chrUn	9801463	9801980	518	SAT1_CF	blue	na
chrUn	9801994	9802792	799	SAT1_CF	blue	na
chrUn	9802812	9803571	760	SAT1_CF	blue	na
chrUn	9803585	9804264	680	SAT1_CF	blue	na
chrUn	9804277	9804797	521	SAT1_CF	blue	na
chrUn	9804865	9805257	393	SAT1_CF	blue	na
chrUn	9805373	9806144	772	SAT1_CF	blue	na
chrUn	9806223	9806919	697	SAT1_CF	blue	na
chrUn	9807016	9808235	1220	SAT1_CF	blue	na
chrUn	9808249	9810065	1817	SAT1_CF	blue	na
chrUn	9810079	9810969	891	SAT1_CF	blue	na
chrUn	9811012	9812446	1435	SAT1_CF	blue	na
chrUn	9812474	9813016	543	SAT1_CF	blue	na

chrUn	9813059	9813735	677	SAT1_CF	blue	na
chrUn	9813747	9814570	824	SAT1_CF	blue	na
chrUn	9814590	9815085	496	SAT1_CF	blue	na
chrUn	9815171	9816670	1500	SAT1_CF	blue	na
chrUn	9816682	9817328	647	SAT1_CF	blue	na
chrUn	9817341	9818269	929	SAT1_CF	blue	na
chrUn	9818281	9818961	681	SAT1_CF	blue	na
chrUn	9818973	9819604	632	SAT1_CF	blue	na
chrUn	9819627	9819732	106	SAT1_CF	blue	na
chrUn	9819745	9820323	579	SAT1_CF	blue	na
chrUn	9820403	9821962	1560	SAT1_CF	blue	na
chrUn	9822013	9822864	852	SAT1_CF	blue	na
chrUn	9822876	9823489	614	SAT1_CF	blue	na
chrUn	9823503	9824247	745	SAT1_CF	blue	na
chrUn	9824274	9824775	502	SAT1_CF	blue	na
chrUn	9824862	9825217	356	SAT1_CF	blue	na
chrUn	9825228	9826636	1409	SAT1_CF	blue	na
chrUn	9826678	9827225	548	SAT1_CF	blue	na
chrUn	9827282	9827799	518	SAT1_CF	blue	na
chrUn	9827811	9828237	427	SAT1_CF	blue	na
chrUn	9828280	9828828	549	SAT1_CF	blue	na
chrUn	9828850	9831076	2227	SAT1_CF	blue	na
chrUn	9831107	9831505	399	SAT1_CF	blue	na
chrUn	9831525	9832049	525	SAT1_CF	blue	na
chrUn	9832165	9833131	967	SAT1_CF	blue	na
chrUn	9833174	9833839	666	SAT1_CF	blue	na
chrUn	9833859	9834352	494	SAT1_CF	blue	na
chrUn	9834392	9836250	1859	SAT1_CF	blue	na
chrUn	9836270	9836763	494	SAT1_CF	blue	na
chrUn	9836803	9837726	924	SAT1_CF	blue	na
chrUn	9837754	9838661	908	SAT1_CF	blue	na
chrUn	9838681	9839174	494	SAT1_CF	blue	na
chrUn	9839237	9839582	346	SAT1_CF	blue	na
chrUn	9839626	9840137	512	SAT1_CF	blue	na
chrUn	9840165	9841075	911	SAT1_CF	blue	na
chrUn	9841087	9842289	1203	SAT1_CF	blue	na
chrUn	9842334	9842846	513	SAT1_CF	blue	na
chrUn	9842927	9843742	816	SAT1_CF	blue	na
chrUn	9843784	9844642	859	SAT1_CF	blue	na
chrUn	9844654	9845336	683	SAT1_CF	blue	na
chrUn	9845349	9845847	499	SAT1_CF	blue	na
chrUn	9845895	9846291	397	SAT1_CF	blue	na

chrUn	9846304	9846847	544	SAT1_CF	blue	na
chrUn	9846926	9847890	965	SAT1_CF	blue	na
chrUn	9847933	9848608	676	SAT1_CF	blue	na
chrUn	9848620	9849946	1327	SAT1_CF	blue	na
chrUn	9850005	9850791	787	SAT1_CF	blue	na
chrUn	9850819	9852669	1851	SAT1_CF	blue	na
chrUn	9852712	9853255	544	SAT1_CF	blue	na
chrUn	9853281	9854078	798	SAT1_CF	blue	na
chrUn	9854091	9854591	501	SAT1_CF	blue	na
chrUn	9854631	9854814	184	SAT1_CF	blue	na
chrUn	9854994	9855896	903	SAT1_CF	blue	na
chrUn	9855923	9856017	95	SAT1_CF	blue	na
chrUn	9856029	9856706	678	SAT1_CF	blue	na
chrUn	9856720	9857896	1177	SAT1_CF	blue	na
chrUn	9857980	9858116	137	SAT1_CF	blue	na
chrUn	9858131	9859456	1326	SAT1_CF	blue	na
chrUn	9859469	9859969	501	SAT1_CF	blue	na
chrUn	9860056	9860584	529	SAT1_CF	blue	na
chrUn	9860604	9861880	1277	SAT1_CF	blue	na
chrUn	9861922	9862653	732	SAT1_CF	blue	na
chrUn	9862673	9862780	108	SAT1_CF	blue	na
chrUn	9862792	9863478	687	SAT1_CF	blue	na
chrUn	9863490	9864009	520	SAT1_CF	blue	na
chrUn	9864028	9864513	486	SAT1_CF	blue	na
chrUn	9864531	9865305	775	SAT1_CF	blue	na
chrUn	9865317	9865871	555	SAT1_CF	blue	na
chrUn	9865915	9866441	527	SAT1_CF	blue	na
chrUn	9866485	9867029	545	SAT1_CF	blue	na
chrUn	9867079	9867246	168	SAT1_CF	blue	na
chrUn	9867258	9867930	673	SAT1_CF	blue	na
chrUn	9867943	9869313	1371	SAT1_CF	blue	na
chrUn	9869353	9869754	402	SAT1_CF	blue	na
chrUn	9869765	9870302	538	SAT1_CF	blue	na
chrUn	9870392	9871431	1040	SAT1_CF	blue	na
chrUn	9871475	9872148	674	SAT1_CF	blue	na
chrUn	9872175	9872763	589	SAT1_CF	blue	na
chrUn	9872790	9873393	604	SAT1_CF	blue	na
chrUn	9873405	9874500	1096	SAT1_CF	blue	na
chrUn	9874542	9875217	676	SAT1_CF	blue	na
chrUn	9875229	9875735	507	SAT1_CF	blue	na
chrUn	9875770	9876171	402	SAT1_CF	blue	na
chrUn	9876185	9876692	508	SAT1_CF	blue	na

chrUn	9876770	9878719	1950	SAT1_CF	blue	na
chrUn	9878732	9879232	501	SAT1_CF	blue	na
chrUn	9879277	9881907	2631	SAT1_CF	blue	na
chrUn	9881971	9882366	396	SAT1_CF	blue	na
chrUn	9882381	9882888	508	SAT1_CF	blue	na
chrUn	9882915	9883695	781	SAT1_CF	blue	na
chrUn	9883722	9883997	276	SAT1_CF	blue	na
chrUn	9884010	9884562	553	SAT1_CF	blue	na
chrUn	9884618	9884690	73	SAT1_CF	blue	na
chrUn	9884703	9885204	502	SAT1_CF	blue	na
chrUn	9885259	9886625	1367	SAT1_CF	blue	na
chrUn	9886668	9887874	1207	SAT1_CF	blue	na
chrUn	9887895	9888004	110	SAT1_CF	blue	na
chrUn	9888016	9888518	503	SAT1_CF	blue	na
chrUn	9888563	9888664	102	SAT1_CF	blue	na
chrUn	9888771	9889257	487	SAT1_CF	blue	na
chrUn	9889288	9890747	1460	SAT1_CF	blue	na
chrUn	9890759	9891312	554	SAT1_CF	blue	na
chrUn	9891333	9891440	108	SAT1_CF	blue	na
chrUn	9891453	9891952	500	SAT1_CF	blue	na
chrUn	9892018	9892154	137	SAT1_CF	blue	na
chrUn	9892182	9893387	1206	SAT1_CF	blue	na
chrUn	9893440	9893516	77	SAT1_CF	blue	na
chrUn	9893529	9894341	813	SAT1_CF	blue	na
chrUn	9894377	9895693	1317	SAT1_CF	blue	na
chrUn	9895705	9896219	515	SAT1_CF	blue	na
chrUn	9896245	9896780	536	SAT1_CF	blue	na
chrUn	9896824	9897675	852	SAT1_CF	blue	na
chrUn	9898627	9899216	590	SAT1_CF	blue	na
chrUn	9899238	9899840	603	SAT1_CF	blue	na
chrUn	9899852	9900541	690	SAT1_CF	blue	na
chrUn	9900555	9901214	660	SAT1_CF	blue	na
chrUn	9901229	9902423	1195	SAT1_CF	blue	na
chrUn	9902446	9902551	106	SAT1_CF	blue	na
chrUn	9902564	9903063	500	SAT1_CF	blue	na
chrUn	9903108	9904062	955	SAT1_CF	blue	na
chrUn	9904141	9904923	783	SAT1_CF	blue	na
chrUn	9904965	9905968	1004	SAT1_CF	blue	na
chrUn	9905991	9906097	107	SAT1_CF	blue	na
chrUn	9906109	9906611	503	SAT1_CF	blue	na
chrUn	9906656	9906813	158	SAT1_CF	blue	na
chrUn	9906841	9907351	511	SAT1_CF	blue	na

chrUn	9907394	9908190	797	SAT1_CF	blue	na
chrUn	9908235	9908861	627	SAT1_CF	blue	na
chrUn	9908888	9909390	503	SAT1_CF	blue	na
chrUn	9909430	9911435	2006	SAT1_CF	blue	na
chrUn	9911477	9912152	676	SAT1_CF	blue	na
chrUn	9912172	9912647	476	SAT1_CF	blue	na
chrUn	9912753	9914817	2065	SAT1_CF	blue	na
chrUn	9914838	9914910	73	SAT1_CF	blue	na
chrUn	9914923	9915849	927	SAT1_CF	blue	na
chrUn	9915861	9916336	476	SAT1_CF	blue	na
chrUn	9916399	9917180	782	SAT1_CF	blue	na
chrUn	9917208	9917719	512	SAT1_CF	blue	na
chrUn	9917799	9918362	564	SAT1_CF	blue	na
chrUn	9918389	9919021	633	SAT1_CF	blue	na
chrUn	9919064	9919728	665	SAT1_CF	blue	na
chrUn	9919748	9920289	542	SAT1_CF	blue	na
chrUn	9920333	9921331	999	SAT1_CF	blue	na
chrUn	9921375	9922047	673	SAT1_CF	blue	na
chrUn	9922061	9922690	630	SAT1_CF	blue	na
chrUn	9922712	9923091	380	SAT1_CF	blue	na
chrUn	9923103	9923864	762	SAT1_CF	blue	na
chrUn	9923884	9924376	493	SAT1_CF	blue	na
chrUn	9924416	9924818	403	SAT1_CF	blue	na
chrUn	9924832	9925376	545	SAT1_CF	blue	na
chrUn	9925419	9926642	1224	SAT1_CF	blue	na
chrUn	9926662	9927416	755	SAT1_CF	blue	na
chrUn	9927429	9927930	502	SAT1_CF	blue	na
chrUn	9928017	9929079	1063	SAT1_CF	blue	na
chrUn	9929090	9929592	503	SAT1_CF	blue	na
chrUn	9929645	9931431	1787	SAT1_CF	blue	na
chrUn	9931459	9931652	194	SAT1_CF	blue	na
chrUn	9931683	9932203	521	SAT1_CF	blue	na
chrUn	9932320	9933549	1230	SAT1_CF	blue	na
chrUn	9933587	9934129	543	SAT1_CF	blue	na
chrUn	9934171	9934288	118	SAT1_CF	blue	na
chrUn	9934335	9934995	661	SAT1_CF	blue	na
chrUn	9935022	9935523	502	SAT1_CF	blue	na
chrUn	9935610	9935965	356	SAT1_CF	blue	na
chrUn	9935978	9937163	1186	SAT1_CF	blue	na
chrUn	9937190	9937372	183	SAT1_CF	blue	na
chrUn	9937388	9937951	564	SAT1_CF	blue	na
chrUn	9938068	9939258	1191	SAT1_CF	blue	na

chrUn	9939270	9939950	681	SAT1_CF	blue	na
chrUn	9939963	9940465	503	SAT1_CF	blue	na
chrUn	9940503	9940907	405	SAT1_CF	blue	na
chrUn	9940920	9942699	1780	SAT1_CF	blue	na
chrUn	9942750	9943224	475	SAT1_CF	blue	na
chrUn	9943287	9944218	932	SAT1_CF	blue	na
chrUn	9944285	9944585	301	SAT1_CF	blue	na
chrUn	9944635	9945152	518	SAT1_CF	blue	na
chrUn	9945166	9945969	804	SAT1_CF	blue	na
chrUn	9945982	9946539	558	SAT1_CF	blue	na
chrUn	9946559	9946667	109	SAT1_CF	blue	na
chrUn	9946680	9947200	521	SAT1_CF	blue	na
chrUn	9947217	9947402	186	SAT1_CF	blue	na
chrUn	9947582	9947747	166	SAT1_CF	blue	na
chrUn	9947789	9951075	3287	SAT1_CF	blue	na
chrUn	9951087	9951738	652	SAT1_CF	blue	na
chrUn	9951789	9952283	495	SAT1_CF	blue	na
chrUn	9952323	9953705	1383	SAT1_CF	blue	na
chrUn	9953749	9954942	1194	SAT1_CF	blue	na
chrUn	9954953	9955071	119	SAT1_CF	blue	na
chrUn	9955089	9955737	649	SAT1_CF	blue	na
chrUn	9955748	9956322	575	SAT1_CF	blue	na
chrUn	9956345	9956451	107	SAT1_CF	blue	na
chrUn	9956464	9957009	546	SAT1_CF	blue	na
chrUn	9957087	9958143	1057	SAT1_CF	blue	na
chrUn	9958198	9958978	781	SAT1_CF	blue	na
chrUn	9959026	9959702	677	SAT1_CF	blue	na
chrUn	9959714	9960269	556	SAT1_CF	blue	na
chrUn	9960334	9961166	833	SAT1_CF	blue	na
chrUn	9961180	9962333	1154	SAT1_CF	blue	na
chrUn	9962346	9963610	1265	SAT1_CF	blue	na
chrUn	9963636	9963793	158	SAT1_CF	blue	na
chrUn	9963808	9964331	524	SAT1_CF	blue	na
chrUn	9964410	9965085	676	SAT1_CF	blue	na
chrUn	9965099	9965781	683	SAT1_CF	blue	na
chrUn	9965793	9966372	580	SAT1_CF	blue	na
chrUn	9966417	9967641	1225	SAT1_CF	blue	na
chrUn	9967659	9968245	587	SAT1_CF	blue	na
chrUn	9968287	9968634	348	SAT1_CF	blue	na
chrUn	9968672	9969216	545	SAT1_CF	blue	na
chrUn	9969258	9970397	1140	SAT1_CF	blue	na
chrUn	9970423	9972022	1600	SAT1_CF	blue	na

chrUn	9972049	9973817	1769	SAT1_CF	blue	na
chrUn	9973845	9975122	1278	SAT1_CF	blue	na
chrUn	9975133	9975658	526	SAT1_CF	blue	na
chrUn	9975701	9976247	547	SAT1_CF	blue	na
chrUn	9976304	9976828	525	SAT1_CF	blue	na
chrUn	9976851	9977999	1149	SAT1_CF	blue	na
chrUn	9978042	9979013	972	SAT1_CF	blue	na
chrUn	9979057	9980420	1364	SAT1_CF	blue	na
chrUn	9980433	9980952	520	SAT1_CF	blue	na
chrUn	9980969	9981154	186	SAT1_CF	blue	na
chrUn	9981336	9981542	207	SAT1_CF	blue	na
chrUn	9981585	9982193	609	SAT1_CF	blue	na
chrUn	9982250	9983374	1125	SAT1_CF	blue	na
chrUn	9983388	9984068	681	SAT1_CF	blue	na
chrUn	9984080	9984634	555	SAT1_CF	blue	na
chrUn	9984679	9984761	83	SAT1_CF	blue	na
chrUn	9984774	9985274	501	SAT1_CF	blue	na
chrUn	9985310	9985495	186	SAT1_CF	blue	na
chrUn	9985676	9986513	838	SAT1_CF	blue	na
chrUn	9986525	9987284	760	SAT1_CF	blue	na
chrUn	9987305	9987799	495	SAT1_CF	blue	na
chrUn	9987839	9988796	958	SAT1_CF	blue	na
chrUn	9988840	9989802	963	SAT1_CF	blue	na
chrUn	9989845	9991223	1379	SAT1_CF	blue	na
chrUn	9991235	9991788	554	SAT1_CF	blue	na
chrUn	9991831	9992393	563	SAT1_CF	blue	na
chrUn	9992458	9993823	1366	SAT1_CF	blue	na
chrUn	9993837	9994329	493	SAT1_CF	blue	na
chrUn	9994340	9996720	2381	SAT1_CF	blue	na
chrUn	9996742	9997244	503	SAT1_CF	blue	na
chrUn	9997297	9997686	390	SAT1_CF	blue	na
chrUn	9997699	9997863	165	SAT1_CF	blue	na
chrUn	9997882	9999123	1242	SAT1_CF	blue	na
chrUn	9999191	9999570	380	SAT1_CF	blue	na
chrUn	9999603	10000126	524	SAT1_CF	blue	na
chrUn	10000148	10001046	899	SAT1_CF	blue	na
chrUn	10001088	10001946	859	SAT1_CF	blue	na
chrUn	10001959	10002591	633	SAT1_CF	blue	na
chrUn	10002608	10002721	114	SAT1_CF	blue	na
chrUn	10002733	10003033	301	SAT1_CF	blue	na
chrUn	10003046	10003545	500	SAT1_CF	blue	na
chrUn	10003611	10003747	137	SAT1_CF	blue	na

chrUn	10003775	10004284	510	SAT1_CF	blue	na
chrUn	10004364	10005439	1076	SAT1_CF	blue	na
chrUn	10005481	10006021	541	SAT1_CF	blue	na
chrUn	10006064	10007432	1369	SAT1_CF	blue	na
chrUn	10007445	10007945	501	SAT1_CF	blue	na
chrUn	10007985	10008386	402	SAT1_CF	blue	na
chrUn	10008399	10008919	521	SAT1_CF	blue	na
chrUn	10008950	10009329	380	SAT1_CF	blue	na
chrUn	10009410	10010703	1294	SAT1_CF	blue	na
chrUn	10010716	10011400	685	SAT1_CF	blue	na
chrUn	10011427	10011908	482	SAT1_CF	blue	na
chrUn	10011934	10013000	1067	SAT1_CF	blue	na
chrUn	10013018	10013518	501	SAT1_CF	blue	na
chrUn	10013571	10013922	352	SAT1_CF	blue	na
chrUn	10013935	10014456	522	SAT1_CF	blue	na
chrUn	10014597	10015031	435	SAT1_CF	blue	na
chrUn	10015718	10016184	467	SAT1_CF	blue	na
chrUn	10016201	10016866	666	SAT1_CF	blue	na
chrUn	10017031	10017143	113	SAT1_CF	blue	na
chrUn	10017156	10017657	502	SAT1_CF	blue	na
chrUn	10017697	10018076	380	SAT1_CF	blue	na
chrUn	10018110	10018655	546	SAT1_CF	blue	na
chrUn	10018988	10019761	774	SAT1_CF	blue	na
chrUn	10019792	10020285	494	SAT1_CF	blue	na
chrUn	10020429	10022420	1992	SAT1_CF	blue	na
chrUn	10022463	10023706	1244	SAT1_CF	blue	na
chrUn	10023751	10024088	338	SAT1_CF	blue	na
chrUn	10024132	10025333	1202	SAT1_CF	blue	na
chrUn	10025354	10025463	110	SAT1_CF	blue	na
chrUn	10025477	10026106	630	SAT1_CF	blue	na
chrUn	10026127	10026236	110	SAT1_CF	blue	na
chrUn	10026248	10026853	606	SAT1_CF	blue	na
chrUn	10026866	10027386	521	SAT1_CF	blue	na
chrUn	10027454	10028330	877	SAT1_CF	blue	na
chrUn	10028446	10028934	489	SAT1_CF	blue	na
chrUn	10028945	10030028	1084	SAT1_CF	blue	na
chrUn	10030048	10030548	501	SAT1_CF	blue	na
chrUn	10030629	10030803	175	SAT1_CF	blue	na
chrUn	10030884	10032128	1245	SAT1_CF	blue	na
chrUn	10032140	10032786	647	SAT1_CF	blue	na
chrUn	10032799	10033727	929	SAT1_CF	blue	na
chrUn	10033739	10034422	684	SAT1_CF	blue	na

chrUn	10034434	10035065	632	SAT1_CF	blue	na
chrUn	10035088	10035193	106	SAT1_CF	blue	na
chrUn	10035206	10036902	1697	SAT1_CF	blue	na
chrUn	10036925	10037030	106	SAT1_CF	blue	na
chrUn	10037043	10037562	520	SAT1_CF	blue	na
chrUn	10037579	10037764	186	SAT1_CF	blue	na
chrUn	10037945	10038147	203	SAT1_CF	blue	na
chrUn	10038190	10038799	610	SAT1_CF	blue	na
chrUn	10038819	10039977	1159	SAT1_CF	blue	na
chrUn	10039989	10041563	1575	SAT1_CF	blue	na
chrUn	10041759	10042869	1111	SAT1_CF	blue	na
chrUn	10042912	10043629	718	SAT1_CF	blue	na
chrUn	10043684	10044746	1063	SAT1_CF	blue	na
chrUn	10044825	10045787	963	SAT1_CF	blue	na
chrUn	10045830	10046509	680	SAT1_CF	blue	na
chrUn	10046521	10047194	674	SAT1_CF	blue	na
chrUn	10047215	10047716	502	SAT1_CF	blue	na
chrUn	10047769	10048158	390	SAT1_CF	blue	na
chrUn	10048171	10050013	1843	SAT1_CF	blue	na
chrUn	10050039	10051345	1307	SAT1_CF	blue	na
chrUn	10051388	10052064	677	SAT1_CF	blue	na
chrUn	10052076	10052678	603	SAT1_CF	blue	na
chrUn	10052689	10053368	680	SAT1_CF	blue	na
chrUn	10053380	10054068	689	SAT1_CF	blue	na
chrUn	10054082	10054741	660	SAT1_CF	blue	na
chrUn	10054756	10055952	1197	SAT1_CF	blue	na
chrUn	10055975	10056080	106	SAT1_CF	blue	na
chrUn	10056093	10056594	502	SAT1_CF	blue	na
chrUn	10056630	10056815	186	SAT1_CF	blue	na
chrUn	10056995	10057829	835	SAT1_CF	blue	na
chrUn	10057841	10058602	762	SAT1_CF	blue	na
chrUn	10058616	10059117	502	SAT1_CF	blue	na
chrUn	10059153	10061047	1895	SAT1_CF	blue	na
chrUn	10061060	10061906	847	SAT1_CF	blue	na
chrUn	10061918	10062600	683	SAT1_CF	blue	na
chrUn	10062613	10063315	703	SAT1_CF	blue	na
chrUn	10063330	10063855	526	SAT1_CF	blue	na
chrUn	10063898	10064450	553	SAT1_CF	blue	na
chrUn	10064494	10065036	543	SAT1_CF	blue	na
chrUn	10065133	10066582	1450	SAT1_CF	blue	na
chrUn	10066602	10067714	1113	SAT1_CF	blue	na
chrUn	10067752	10070227	2476	SAT1_CF	blue	na

chrUn	10070240	10071000	761	SAT1_CF	blue	na
chrUn	10071013	10071514	502	SAT1_CF	blue	na
chrUn	10071601	10072515	915	SAT1_CF	blue	na
chrUn	10072594	10074177	1584	SAT1_CF	blue	na
chrUn	10074190	10074711	522	SAT1_CF	blue	na
chrUn	10074732	10075261	530	SAT1_CF	blue	na
chrUn	10075290	10076602	1313	SAT1_CF	blue	na
chrUn	10076644	10077492	849	SAT1_CF	blue	na
chrUn	10077516	10078068	553	SAT1_CF	blue	na
chrUn	10078089	10078197	109	SAT1_CF	blue	na
chrUn	10078210	10078730	521	SAT1_CF	blue	na
chrUn	10078749	10079862	1114	SAT1_CF	blue	na
chrUn	10079910	10080586	677	SAT1_CF	blue	na
chrUn	10080598	10081529	932	SAT1_CF	blue	na
chrUn	10081589	10082549	961	SAT1_CF	blue	na
chrUn	10082593	10083557	965	SAT1_CF	blue	na
chrUn	10083580	10084217	638	SAT1_CF	blue	na
chrUn	10084243	10084398	156	SAT1_CF	blue	na
chrUn	10084611	10085225	615	SAT1_CF	blue	na
chrUn	10085238	10085989	752	SAT1_CF	blue	na
chrUn	10086011	10086592	582	SAT1_CF	blue	na
chrUn	10086675	10087183	509	SAT1_CF	blue	na
chrUn	10087242	10088098	857	SAT1_CF	blue	na
chrUn	10088116	10090326	2211	SAT1_CF	blue	na
chrUn	10090344	10090825	482	SAT1_CF	blue	na
chrUn	10090932	10091916	985	SAT1_CF	blue	na
chrUn	10091937	10092045	109	SAT1_CF	blue	na
chrUn	10092058	10092531	474	SAT1_CF	blue	na
chrUn	10092591	10092771	181	SAT1_CF	blue	na
chrUn	10092952	10094169	1218	SAT1_CF	blue	na
chrUn	10094197	10096116	1920	SAT1_CF	blue	na
chrUn	10096168	10096299	132	SAT1_CF	blue	na
chrUn	10096314	10097638	1325	SAT1_CF	blue	na
chrUn	10097661	10098131	471	SAT1_CF	blue	na
chrUn	10098192	10098373	182	SAT1_CF	blue	na
chrUn	10098554	10098756	203	SAT1_CF	blue	na
chrUn	10098799	10099346	548	SAT1_CF	blue	na
chrUn	10099390	10101345	1956	SAT1_CF	blue	na
chrUn	10101358	10101858	501	SAT1_CF	blue	na
chrUn	10101898	10102298	401	SAT1_CF	blue	na
chrUn	10102310	10102856	547	SAT1_CF	blue	na
chrUn	10102913	10103260	348	SAT1_CF	blue	na

chrUn	10103285	10104573	1289	SAT1_CF	blue	na
chrUn	10104594	10104972	379	SAT1_CF	blue	na
chrUn	10105009	10105516	508	SAT1_CF	blue	na
chrUn	10105559	10106376	818	SAT1_CF	blue	na
chrUn	10106418	10107208	791	SAT1_CF	blue	na
chrUn	10107264	10107336	73	SAT1_CF	blue	na
chrUn	10107349	10107851	503	SAT1_CF	blue	na
chrUn	10107896	10108053	158	SAT1_CF	blue	na
chrUn	10108104	10108590	487	SAT1_CF	blue	na
chrUn	10108621	10110284	1664	SAT1_CF	blue	na
chrUn	10110305	10110412	108	SAT1_CF	blue	na
chrUn	10110425	10110924	500	SAT1_CF	blue	na
chrUn	10111011	10113681	2671	SAT1_CF	blue	na
chrUn	10113693	10114302	610	SAT1_CF	blue	na
chrUn	10114315	10114817	503	SAT1_CF	blue	na
chrUn	10114903	10115077	175	SAT1_CF	blue	na
chrUn	10115158	10116403	1246	SAT1_CF	blue	na
chrUn	10116415	10116966	552	SAT1_CF	blue	na
chrUn	10116988	10117060	73	SAT1_CF	blue	na
chrUn	10117073	10118000	928	SAT1_CF	blue	na
chrUn	10118012	10118695	684	SAT1_CF	blue	na
chrUn	10118707	10119391	685	SAT1_CF	blue	na
chrUn	10119403	10120087	685	SAT1_CF	blue	na
chrUn	10120100	10120935	836	SAT1_CF	blue	na
chrUn	10120948	10122264	1317	SAT1_CF	blue	na
chrUn	10122276	10122790	515	SAT1_CF	blue	na
chrUn	10122805	10123349	545	SAT1_CF	blue	na
chrUn	10123394	10124120	727	SAT1_CF	blue	na
chrUn	10124163	10124839	677	SAT1_CF	blue	na
chrUn	10124851	10126144	1294	SAT1_CF	blue	na
chrUn	10126156	10126844	689	SAT1_CF	blue	na
chrUn	10126858	10128728	1871	SAT1_CF	blue	na
chrUn	10128751	10128856	106	SAT1_CF	blue	na
chrUn	10128869	10129369	501	SAT1_CF	blue	na
chrUn	10129414	10129571	158	SAT1_CF	blue	na
chrUn	10129599	10130757	1159	SAT1_CF	blue	na
chrUn	10130769	10132729	1961	SAT1_CF	blue	na
chrUn	10132771	10134139	1369	SAT1_CF	blue	na
chrUn	10134151	10134614	464	SAT1_CF	blue	na
chrUn	10134684	10135229	546	SAT1_CF	blue	na
chrUn	10135251	10135357	107	SAT1_CF	blue	na
chrUn	10135370	10135872	503	SAT1_CF	blue	na

chrUn	10135957	10137229	1273	SAT1_CF	blue	na
chrUn	10137242	10137797	556	SAT1_CF	blue	na
chrUn	10137818	10137920	103	SAT1_CF	blue	na
chrUn	10137938	10138435	498	SAT1_CF	blue	na
chrUn	10138544	10138796	253	SAT1_CF	blue	na
chrUn	10138815	10139741	927	SAT1_CF	blue	na
chrUn	10139756	10141226	1471	SAT1_CF	blue	na
chrUn	10141238	10141923	686	SAT1_CF	blue	na
chrUn	10141934	10142488	555	SAT1_CF	blue	na
chrUn	10142602	10143575	974	SAT1_CF	blue	na
chrUn	10143618	10145309	1692	SAT1_CF	blue	na
chrUn	10145340	10145859	520	SAT1_CF	blue	na
chrUn	10145976	10146331	356	SAT1_CF	blue	na
chrUn	10146343	10147312	970	SAT1_CF	blue	na
chrUn	10147356	10148771	1416	SAT1_CF	blue	na
chrUn	10148798	10149299	502	SAT1_CF	blue	na
chrUn	10149344	10150686	1343	SAT1_CF	blue	na
chrUn	10150942	10153566	2625	SAT1_CF	blue	na
chrUn	10153619	10154398	780	SAT1_CF	blue	na
chrUn	10154412	10155107	696	SAT1_CF	blue	na
chrUn	10155151	10155612	462	SAT1_CF	blue	na
chrUn	10155630	10156141	512	SAT1_CF	blue	na
chrUn	10156222	10156784	563	SAT1_CF	blue	na
chrUn	10156828	10158150	1323	SAT1_CF	blue	na
chrUn	10158162	10158682	521	SAT1_CF	blue	na
chrUn	10158749	10159660	912	SAT1_CF	blue	na
chrUn	10159739	10160704	966	SAT1_CF	blue	na
chrUn	10160747	10161421	675	SAT1_CF	blue	na
chrUn	10161433	10162114	682	SAT1_CF	blue	na
chrUn	10162127	10162627	501	SAT1_CF	blue	na
chrUn	10162693	10162829	137	SAT1_CF	blue	na
chrUn	10162844	10163367	524	SAT1_CF	blue	na
chrUn	10163398	10164485	1088	SAT1_CF	blue	na
chrUn	10164564	10165036	473	SAT1_CF	blue	na
chrUn	10165153	10166135	983	SAT1_CF	blue	na
chrUn	10166153	10167698	1546	SAT1_CF	blue	na
chrUn	10167711	10168213	503	SAT1_CF	blue	na
chrUn	10168253	10168655	403	SAT1_CF	blue	na
chrUn	10168668	10170444	1777	SAT1_CF	blue	na
chrUn	10170497	10170971	475	SAT1_CF	blue	na
chrUn	10171034	10171965	932	SAT1_CF	blue	na
chrUn	10171997	10173717	1721	SAT1_CF	blue	na

chrUn	10173729	10173943	215	SAT1_CF	blue	na
chrUn	10174138	10176324	2187	SAT1_CF	blue	na
chrUn	10176335	10177091	757	SAT1_CF	blue	na
chrUn	10177109	10178220	1112	SAT1_CF	blue	na
chrUn	10178262	10179128	867	SAT1_CF	blue	na
chrUn	10179140	10179695	556	SAT1_CF	blue	na
chrUn	10179750	10180381	632	SAT1_CF	blue	na
chrUn	10180424	10182272	1849	SAT1_CF	blue	na
chrUn	10182349	10183209	861	SAT1_CF	blue	na
chrUn	10183222	10183779	558	SAT1_CF	blue	na
chrUn	10183799	10183907	109	SAT1_CF	blue	na
chrUn	10183920	10184394	475	SAT1_CF	blue	na
chrUn	10184447	10184626	180	SAT1_CF	blue	na
chrUn	10184643	10186041	1399	SAT1_CF	blue	na
chrUn	10186062	10186411	350	SAT1_CF	blue	na
chrUn	10186491	10188242	1752	SAT1_CF	blue	na
chrUn	10188254	10188936	683	SAT1_CF	blue	na
chrUn	10188950	10189450	501	SAT1_CF	blue	na
chrUn	10189537	10190424	888	SAT1_CF	blue	na
chrUn	10190456	10190974	519	SAT1_CF	blue	na
chrUn	10190988	10191527	540	SAT1_CF	blue	na
chrUn	10191570	10192246	677	SAT1_CF	blue	na
chrUn	10192258	10192873	616	SAT1_CF	blue	na
chrUn	10192890	10194287	1398	SAT1_CF	blue	na
chrUn	10194301	10194820	520	SAT1_CF	blue	na
chrUn	10194841	10195200	360	SAT1_CF	blue	na
chrUn	10195214	10197783	2570	SAT1_CF	blue	na
chrUn	10197826	10198500	675	SAT1_CF	blue	na
chrUn	10198512	10199195	684	SAT1_CF	blue	na
chrUn	10199209	10199688	480	SAT1_CF	blue	na
chrUn	10199795	10200308	514	SAT1_CF	blue	na
chrUn	10200338	10202428	2091	SAT1_CF	blue	na
chrUn	10202440	10203086	647	SAT1_CF	blue	na
chrUn	10203099	10203860	762	SAT1_CF	blue	na
chrUn	10203872	10204445	574	SAT1_CF	blue	na
chrUn	10204468	10204574	107	SAT1_CF	blue	na
chrUn	10204587	10205132	546	SAT1_CF	blue	na
chrUn	10205209	10207246	2038	SAT1_CF	blue	na
chrUn	10207269	10207824	556	SAT1_CF	blue	na
chrUn	10207849	10207953	105	SAT1_CF	blue	na
chrUn	10207966	10208844	879	SAT1_CF	blue	na
chrUn	10208857	10210010	1154	SAT1_CF	blue	na

chrUn	10210023	10210524	502	SAT1_CF	blue	na
chrUn	10210590	10210726	137	SAT1_CF	blue	na
chrUn	10210758	10211263	506	SAT1_CF	blue	na
chrUn	10211295	10212000	706	SAT1_CF	blue	na
chrUn	10212020	10212534	515	SAT1_CF	blue	na
chrUn	10212560	10212717	158	SAT1_CF	blue	na
chrUn	10212732	10213256	525	SAT1_CF	blue	na
chrUn	10213336	10214010	675	SAT1_CF	blue	na
chrUn	10214024	10214706	683	SAT1_CF	blue	na
chrUn	10214718	10215216	499	SAT1_CF	blue	na
chrUn	10215233	10216442	1210	SAT1_CF	blue	na
chrUn	10216508	10217175	668	SAT1_CF	blue	na
chrUn	10217217	10217564	348	SAT1_CF	blue	na
chrUn	10217602	10218146	545	SAT1_CF	blue	na
chrUn	10218196	10218734	539	SAT1_CF	blue	na
chrUn	10218777	10219457	681	SAT1_CF	blue	na
chrUn	10219469	10220308	840	SAT1_CF	blue	na
chrUn	10220321	10220823	503	SAT1_CF	blue	na
chrUn	10220876	10221194	319	SAT1_CF	blue	na
chrUn	10221205	10221748	544	SAT1_CF	blue	na
chrUn	10221759	10221985	227	SAT1_CF	blue	na
chrUn	10222065	10222718	654	SAT1_CF	blue	na
chrUn	10222730	10224324	1595	SAT1_CF	blue	na
chrUn	10224424	10225786	1363	SAT1_CF	blue	na
chrUn	10225799	10227449	1651	SAT1_CF	blue	na
chrUn	10227467	10228226	760	SAT1_CF	blue	na
chrUn	10228239	10229486	1248	SAT1_CF	blue	na
chrUn	10229529	10230073	545	SAT1_CF	blue	na
chrUn	10230116	10230662	547	SAT1_CF	blue	na
chrUn	10230683	10230792	110	SAT1_CF	blue	na
chrUn	10230804	10231177	374	SAT1_CF	blue	na
chrUn	10231206	10231923	718	SAT1_CF	blue	na
chrUn	10231935	10232695	761	SAT1_CF	blue	na
chrUn	10232709	10233190	482	SAT1_CF	blue	na
chrUn	10233297	10233624	328	SAT1_CF	blue	na
chrUn	10233662	10235107	1446	SAT1_CF	blue	na
chrUn	10235149	10235778	630	SAT1_CF	blue	na
chrUn	10235791	10236370	580	SAT1_CF	blue	na
chrUn	10236449	10238238	1790	SAT1_CF	blue	na
chrUn	10238250	10238881	632	SAT1_CF	blue	na
chrUn	10238939	10239494	556	SAT1_CF	blue	na
chrUn	10239537	10240470	934	SAT1_CF	blue	na

chrUn	10240497	10241129	633	SAT1_CF	blue	na
chrUn	10241172	10241718	547	SAT1_CF	blue	na
chrUn	10241738	10241847	110	SAT1_CF	blue	na
chrUn	10241859	10242622	764	SAT1_CF	blue	na
chrUn	10242634	10243316	683	SAT1_CF	blue	na
chrUn	10243329	10243830	502	SAT1_CF	blue	na
chrUn	10243917	10244827	911	SAT1_CF	blue	na
chrUn	10244843	10245734	892	SAT1_CF	blue	na
chrUn	10245783	10246454	672	SAT1_CF	blue	na
chrUn	10246467	10246968	502	SAT1_CF	blue	na
chrUn	10247055	10247410	356	SAT1_CF	blue	na
chrUn	10247423	10247932	510	SAT1_CF	blue	na
chrUn	10248011	10248793	783	SAT1_CF	blue	na
chrUn	10248835	10249398	564	SAT1_CF	blue	na
chrUn	10249515	10251397	1883	SAT1_CF	blue	na
chrUn	10251410	10251912	503	SAT1_CF	blue	na
chrUn	10251952	10252352	401	SAT1_CF	blue	na
chrUn	10252367	10255050	2684	SAT1_CF	blue	na
chrUn	10255093	10255769	677	SAT1_CF	blue	na
chrUn	10255781	10256383	603	SAT1_CF	blue	na
chrUn	10256394	10257074	681	SAT1_CF	blue	na
chrUn	10257086	10257768	683	SAT1_CF	blue	na
chrUn	10257782	10258321	540	SAT1_CF	blue	na
chrUn	10258332	10258839	508	SAT1_CF	blue	na
chrUn	10258868	10259655	788	SAT1_CF	blue	na
chrUn	10259678	10259783	106	SAT1_CF	blue	na
chrUn	10259796	10260297	502	SAT1_CF	blue	na
chrUn	10260363	10260499	137	SAT1_CF	blue	na
chrUn	10260527	10262191	1665	SAT1_CF	blue	na
chrUn	10262233	10263393	1161	SAT1_CF	blue	na
chrUn	10263406	10264467	1062	SAT1_CF	blue	na
chrUn	10264488	10264597	110	SAT1_CF	blue	na
chrUn	10264609	10265109	501	SAT1_CF	blue	na
chrUn	10265196	10266529	1334	SAT1_CF	blue	na
chrUn	10266573	10267901	1329	SAT1_CF	blue	na
chrUn	10267919	10268473	555	SAT1_CF	blue	na
chrUn	10268495	10268602	108	SAT1_CF	blue	na
chrUn	10268615	10269134	520	SAT1_CF	blue	na
chrUn	10269151	10270031	881	SAT1_CF	blue	na
chrUn	10270044	10270664	621	SAT1_CF	blue	na
chrUn	10270687	10270792	106	SAT1_CF	blue	na
chrUn	10270805	10271306	502	SAT1_CF	blue	na

chrUn	10271342	10271527	186	SAT1_CF	blue	na
chrUn	10271710	10271875	166	SAT1_CF	blue	na
chrUn	10271956	10273584	1629	SAT1_CF	blue	na
chrUn	10273627	10274302	676	SAT1_CF	blue	na
chrUn	10274316	10275076	761	SAT1_CF	blue	na
chrUn	10275088	10275770	683	SAT1_CF	blue	na
chrUn	10275783	10277210	1428	SAT1_CF	blue	na
chrUn	10277233	10278563	1331	SAT1_CF	blue	na
chrUn	10278575	10279260	686	SAT1_CF	blue	na
chrUn	10279271	10279826	556	SAT1_CF	blue	na
chrUn	10279940	10280913	974	SAT1_CF	blue	na
chrUn	10280956	10282648	1693	SAT1_CF	blue	na
chrUn	10282679	10283197	519	SAT1_CF	blue	na
chrUn	10283314	10283669	356	SAT1_CF	blue	na
chrUn	10283682	10285309	1628	SAT1_CF	blue	na
chrUn	10285389	10286060	672	SAT1_CF	blue	na
chrUn	10286071	10286611	541	SAT1_CF	blue	na
chrUn	10286655	10286970	316	SAT1_CF	blue	na
chrUn	10287014	10288219	1206	SAT1_CF	blue	na
chrUn	10288276	10288795	520	SAT1_CF	blue	na
chrUn	10288890	10289026	137	SAT1_CF	blue	na
chrUn	10289054	10289565	512	SAT1_CF	blue	na
chrUn	10289593	10290098	506	SAT1_CF	blue	na
chrUn	10290140	10290756	617	SAT1_CF	blue	na
chrUn	10290769	10292345	1577	SAT1_CF	blue	na
chrUn	10292369	10292475	107	SAT1_CF	blue	na
chrUn	10292487	10293012	526	SAT1_CF	blue	na
chrUn	10293064	10293749	686	SAT1_CF	blue	na
chrUn	10293782	10294827	1046	SAT1_CF	blue	na
chrUn	10294839	10296035	1197	SAT1_CF	blue	na
chrUn	10296048	10296256	209	SAT1_CF	blue	na
chrUn	10296439	10297275	837	SAT1_CF	blue	na
chrUn	10297287	10298048	762	SAT1_CF	blue	na
chrUn	10298062	10298563	502	SAT1_CF	blue	na
chrUn	10298616	10299943	1328	SAT1_CF	blue	na
chrUn	10299986	10302093	2108	SAT1_CF	blue	na
chrUn	10302113	10302704	592	SAT1_CF	blue	na
chrUn	10302722	10305193	2472	SAT1_CF	blue	na
chrUn	10305236	10307762	2527	SAT1_CF	blue	na
chrUn	10307780	10308282	503	SAT1_CF	blue	na
chrUn	10308322	10309463	1142	SAT1_CF	blue	na
chrUn	10309560	10310433	874	SAT1_CF	blue	na

chrUn	10310477	10311002	526	SAT1_CF	blue	na
chrUn	10311033	10311805	773	SAT1_CF	blue	na
chrUn	10311817	10312490	674	SAT1_CF	blue	na
chrUn	10312502	10313185	684	SAT1_CF	blue	na
chrUn	10313198	10313698	501	SAT1_CF	blue	na
chrUn	10313740	10314660	921	SAT1_CF	blue	na
chrUn	10314721	10314980	260	SAT1_CF	blue	na
chrUn	10315093	10315295	203	SAT1_CF	blue	na
chrUn	10315336	10317737	2402	SAT1_CF	blue	na
chrUn	10317814	10319329	1516	SAT1_CF	blue	na
chrUn	10319342	10319842	501	SAT1_CF	blue	na
chrUn	10319908	10320044	137	SAT1_CF	blue	na
chrUn	10320059	10320806	748	SAT1_CF	blue	na
chrUn	10320867	10321414	548	SAT1_CF	blue	na
chrUn	10321435	10322091	657	SAT1_CF	blue	na
chrUn	10322104	10322605	502	SAT1_CF	blue	na
chrUn	10322645	10323047	403	SAT1_CF	blue	na
chrUn	10323058	10323566	509	SAT1_CF	blue	na
chrUn	10323597	10324557	961	SAT1_CF	blue	na
chrUn	10324569	10325279	711	SAT1_CF	blue	na
chrUn	10325293	10325409	117	SAT1_CF	blue	na
chrUn	10325421	10325941	521	SAT1_CF	blue	na
chrUn	10326009	10327818	1810	SAT1_CF	blue	na
chrUn	10327860	10328718	859	SAT1_CF	blue	na
chrUn	10328730	10329339	610	SAT1_CF	blue	na
chrUn	10329352	10330055	704	SAT1_CF	blue	na
chrUn	10330070	10330594	525	SAT1_CF	blue	na
chrUn	10330673	10332155	1483	SAT1_CF	blue	na
chrUn	10332167	10332669	503	SAT1_CF	blue	na
chrUn	10332687	10333410	724	SAT1_CF	blue	na
chrUn	10333453	10334014	562	SAT1_CF	blue	na
chrUn	10334042	10334670	629	SAT1_CF	blue	na
chrUn	10334715	10335393	679	SAT1_CF	blue	na
chrUn	10335405	10335958	554	SAT1_CF	blue	na
chrUn	10335980	10336087	108	SAT1_CF	blue	na
chrUn	10336100	10336599	500	SAT1_CF	blue	na
chrUn	10336635	10337647	1013	SAT1_CF	blue	na
chrUn	10337714	10339414	1701	SAT1_CF	blue	na
chrUn	10339436	10339957	522	SAT1_CF	blue	na
chrUn	10339983	10342017	2035	SAT1_CF	blue	na
chrUn	10342060	10342724	665	SAT1_CF	blue	na
chrUn	10342747	10343429	683	SAT1_CF	blue	na

chrUn	10343449	10343943	495	SAT1_CF	blue	na
chrUn	10343988	10344129	142	SAT1_CF	blue	na
chrUn	10345130	10345616	487	SAT1_CF	blue	na
chrUn	10345628	10348808	3181	SAT1_CF	blue	na
chrUn	10348850	10350192	1343	SAT1_CF	blue	na
chrUn	10350212	10350706	495	SAT1_CF	blue	na
chrUn	10350746	10351319	574	SAT1_CF	blue	na
chrUn	10352732	10354124	1393	SAT1_CF	blue	na
chrUn	10354136	10355132	997	SAT1_CF	blue	na
chrUn	10355152	10355240	89	SAT1_CF	blue	na
chrUn	10355272	10359102	3831	SAT1_CF	blue	na
chrUn	10359114	10359797	684	SAT1_CF	blue	na
chrUn	10359810	10360295	486	SAT1_CF	blue	na
chrUn	10360363	10361470	1108	SAT1_CF	blue	na
chrUn	10361564	10363079	1516	SAT1_CF	blue	na
chrUn	10363093	10363561	469	SAT1_CF	blue	na
chrUn	10363582	10363691	110	SAT1_CF	blue	na
chrUn	10363703	10364386	684	SAT1_CF	blue	na
chrUn	10364398	10365525	1128	SAT1_CF	blue	na
chrUn	10365604	10366326	723	SAT1_CF	blue	na
chrUn	10366373	10367561	1189	SAT1_CF	blue	na
chrUn	10367627	10367763	137	SAT1_CF	blue	na
chrUn	10367829	10368350	522	SAT1_CF	blue	na
chrUn	10368364	10370505	2142	SAT1_CF	blue	na
chrUn	10370518	10371020	503	SAT1_CF	blue	na
chrUn	10371063	10373098	2036	SAT1_CF	blue	na
chrUn	10373142	10374554	1413	SAT1_CF	blue	na
chrUn	10374597	10375146	550	SAT1_CF	blue	na
chrUn	10375225	10376346	1122	SAT1_CF	blue	na
chrUn	10376359	10377110	752	SAT1_CF	blue	na
chrUn	10377132	10377633	502	SAT1_CF	blue	na
chrUn	10377673	10380550	2878	SAT1_CF	blue	na
chrUn	10380809	10382140	1332	SAT1_CF	blue	na
chrUn	10382173	10382838	666	SAT1_CF	blue	na
chrUn	10382851	10383350	500	SAT1_CF	blue	na
chrUn	10383365	10383906	542	SAT1_CF	blue	na
chrUn	10384043	10384348	306	SAT1_CF	blue	na
chrUn	10384391	10387162	2772	SAT1_CF	blue	na
chrUn	10387175	10387676	502	SAT1_CF	blue	na
chrUn	10387742	10387878	137	SAT1_CF	blue	na
chrUn	10387906	10388453	548	SAT1_CF	blue	na
chrUn	10388537	10389096	560	SAT1_CF	blue	na

chrUn	10389121	10389754	634	SAT1_CF	blue	na
chrUn	10389797	10390984	1188	SAT1_CF	blue	na
chrUn	10391024	10391725	702	SAT1_CF	blue	na
chrUn	10391768	10392844	1077	SAT1_CF	blue	na
chrUn	10392887	10393747	861	SAT1_CF	blue	na
chrUn	10393759	10394504	746	SAT1_CF	blue	na
chrUn	10394531	10395033	503	SAT1_CF	blue	na
chrUn	10395073	10396893	1821	SAT1_CF	blue	na
chrUn	10396923	10396965	43	SAT1_CF	blue	na
chrUn	10396978	10397827	850	SAT1_CF	blue	na
chrUn	10397839	10398501	663	SAT1_CF	blue	na
chrUn	10398543	10399769	1227	SAT1_CF	blue	na
chrUn	10399781	10400383	603	SAT1_CF	blue	na
chrUn	10400394	10401073	680	SAT1_CF	blue	na
chrUn	10401085	10401767	683	SAT1_CF	blue	na
chrUn	10401781	10402321	541	SAT1_CF	blue	na
chrUn	10402332	10402840	509	SAT1_CF	blue	na
chrUn	10402871	10405062	2192	SAT1_CF	blue	na
chrUn	10405074	10405721	648	SAT1_CF	blue	na
chrUn	10405776	10406269	494	SAT1_CF	blue	na
chrUn	10406309	10406957	649	SAT1_CF	blue	na
chrUn	10406978	10409529	2552	SAT1_CF	blue	na
chrUn	10409548	10409862	315	SAT1_CF	blue	na
chrUn	10409942	10410506	565	SAT1_CF	blue	na
chrUn	10410549	10411191	643	SAT1_CF	blue	na
chrUn	10411209	10411885	677	SAT1_CF	blue	na
chrUn	10411897	10412659	763	SAT1_CF	blue	na
chrUn	10412679	10413809	1131	SAT1_CF	blue	na
chrUn	10413856	10413992	137	SAT1_CF	blue	na
chrUn	10414020	10414531	512	SAT1_CF	blue	na
chrUn	10414574	10416675	2102	SAT1_CF	blue	na
chrUn	10416688	10417189	502	SAT1_CF	blue	na
chrUn	10417238	10419193	1956	SAT1_CF	blue	na
chrUn	10419236	10419791	556	SAT1_CF	blue	na
chrUn	10419808	10420378	571	SAT1_CF	blue	na
chrUn	10420443	10420600	158	SAT1_CF	blue	na
chrUn	10420615	10421138	524	SAT1_CF	blue	na
chrUn	10421168	10421433	266	SAT1_CF	blue	na
chrUn	10421513	10423199	1687	SAT1_CF	blue	na
chrUn	10423223	10423905	683	SAT1_CF	blue	na
chrUn	10423918	10424438	521	SAT1_CF	blue	na
chrUn	10424457	10424920	464	SAT1_CF	blue	na

chrUn	10424964	10426167	1204	SAT1_CF	blue	na
chrUn	10426187	10426296	110	SAT1_CF	blue	na
chrUn	10426308	10426992	685	SAT1_CF	blue	na
chrUn	10427006	10427525	520	SAT1_CF	blue	na
chrUn	10427544	10428029	486	SAT1_CF	blue	na
chrUn	10428044	10428821	778	SAT1_CF	blue	na
chrUn	10428833	10430509	1677	SAT1_CF	blue	na
chrUn	10430541	10430581	41	SAT1_CF	blue	na
chrUn	10430594	10430761	168	SAT1_CF	blue	na
chrUn	10430773	10431445	673	SAT1_CF	blue	na
chrUn	10431458	10431939	482	SAT1_CF	blue	na
chrUn	10431965	10432827	863	SAT1_CF	blue	na
chrUn	10432867	10433801	935	SAT1_CF	blue	na
chrUn	10433868	10434825	958	SAT1_CF	blue	na
chrUn	10434874	10435548	675	SAT1_CF	blue	na
chrUn	10435560	10436490	931	SAT1_CF	blue	na
chrUn	10436550	10437509	960	SAT1_CF	blue	na
chrUn	10437553	10438628	1076	SAT1_CF	blue	na
chrUn	10438672	10439346	675	SAT1_CF	blue	na
chrUn	10439373	10439961	589	SAT1_CF	blue	na
chrUn	10439988	10440588	601	SAT1_CF	blue	na
chrUn	10440600	10441695	1096	SAT1_CF	blue	na
chrUn	10441737	10442412	676	SAT1_CF	blue	na
chrUn	10442424	10442925	502	SAT1_CF	blue	na
chrUn	10442937	10443146	210	SAT1_CF	blue	na
chrUn	10443328	10443648	321	SAT1_CF	blue	na
chrUn	10443919	10444757	839	SAT1_CF	blue	na
chrUn	10444779	10445429	651	SAT1_CF	blue	na
chrUn	10445443	10445951	509	SAT1_CF	blue	na
chrUn	10446029	10446335	307	SAT1_CF	blue	na
chrUn	10446378	10447709	1332	SAT1_CF	blue	na
chrUn	10447721	10448202	482	SAT1_CF	blue	na
chrUn	10448226	10448332	107	SAT1_CF	blue	na
chrUn	10448345	10449968	1624	SAT1_CF	blue	na
chrUn	10449980	10450741	762	SAT1_CF	blue	na
chrUn	10450753	10451307	555	SAT1_CF	blue	na
chrUn	10451328	10451433	106	SAT1_CF	blue	na
chrUn	10451447	10452433	987	SAT1_CF	blue	na
chrUn	10452517	10453077	561	SAT1_CF	blue	na
chrUn	10453142	10453772	631	SAT1_CF	blue	na
chrUn	10453815	10454489	675	SAT1_CF	blue	na
chrUn	10454509	10455004	496	SAT1_CF	blue	na

chrUn	10455070	10455206	137	SAT1_CF	blue	na
chrUn	10455221	10455744	524	SAT1_CF	blue	na
chrUn	10455774	10457013	1240	SAT1_CF	blue	na
chrUn	10457064	10457782	719	SAT1_CF	blue	na
chrUn	10463913	10464879	967	SAT1_CF	blue	na
chrUn	10464924	10465081	158	SAT1_CF	blue	na
chrUn	10465109	10465656	548	SAT1_CF	blue	na
chrUn	10465752	10466405	654	SAT1_CF	blue	na
chrUn	10466417	10468095	1679	SAT1_CF	blue	na
chrUn	10468156	10469208	1053	SAT1_CF	blue	na
chrUn	10469231	10470113	883	SAT1_CF	blue	na
chrUn	10470125	10470807	683	SAT1_CF	blue	na
chrUn	10470820	10471320	501	SAT1_CF	blue	na
chrUn	10471360	10471739	380	SAT1_CF	blue	na
chrUn	10471773	10472294	522	SAT1_CF	blue	na
chrUn	10472308	10473379	1072	SAT1_CF	blue	na
chrUn	10473410	10473930	521	SAT1_CF	blue	na
chrUn	10474046	10474401	356	SAT1_CF	blue	na
chrUn	10474414	10476039	1626	SAT1_CF	blue	na
chrUn	10476124	10476791	668	SAT1_CF	blue	na
chrUn	10476803	10477361	559	SAT1_CF	blue	na
chrUn	10477383	10477489	107	SAT1_CF	blue	na
chrUn	10477502	10478004	503	SAT1_CF	blue	na
chrUn	10478070	10478206	137	SAT1_CF	blue	na
chrUn	10478221	10479493	1273	SAT1_CF	blue	na
chrUn	10479516	10479621	106	SAT1_CF	blue	na
chrUn	10479634	10479947	314	SAT1_CF	blue	na
chrUn	10604574	10605193	620	SAT1_CF	blue	na
chrUn	10605207	10605600	394	SAT1_CF	blue	na
chrUn	10605644	10607185	1542	SAT1_CF	blue	na
chrUn	10607197	10607755	559	SAT1_CF	blue	na
chrUn	10607820	10608366	547	SAT1_CF	blue	na
chrUn	10608388	10609374	987	SAT1_CF	blue	na
chrUn	10609417	10610079	663	SAT1_CF	blue	na
chrUn	10610092	10610773	682	SAT1_CF	blue	na
chrUn	10610786	10611306	521	SAT1_CF	blue	na
chrUn	10611327	10613588	2262	SAT1_CF	blue	na
chrUn	10613631	10614178	548	SAT1_CF	blue	na
chrUn	10614193	10614306	114	SAT1_CF	blue	na
chrUn	10614318	10615297	980	SAT1_CF	blue	na
chrUn	10615340	10617682	2343	SAT1_CF	blue	na
chrUn	10617694	10618228	535	SAT1_CF	blue	na

chrUn	10618242	10618759	518	SAT1_CF	blue	na
chrUn	10618776	10618960	185	SAT1_CF	blue	na
chrUn	10619137	10621014	1878	SAT1_CF	blue	na
chrUn	10621058	10621604	547	SAT1_CF	blue	na
chrUn	10621626	10622375	750	SAT1_CF	blue	na
chrUn	10622396	10622504	109	SAT1_CF	blue	na
chrUn	10622517	10622999	483	SAT1_CF	blue	na
chrUn	10623106	10624078	973	SAT1_CF	blue	na
chrUn	10624133	10625096	964	SAT1_CF	blue	na
chrUn	10625139	10625787	649	SAT1_CF	blue	na
chrUn	10625798	10626493	696	SAT1_CF	blue	na
chrUn	10626572	10627248	677	SAT1_CF	blue	na
chrUn	10627262	10627944	683	SAT1_CF	blue	na
chrUn	10627956	10628866	911	SAT1_CF	blue	na
chrUn	10628909	10629679	771	SAT1_CF	blue	na
chrUn	10629748	10629816	69	SAT1_CF	blue	na
chrUn	10629827	10630412	586	SAT1_CF	blue	na
chrUn	10630454	10631565	1112	SAT1_CF	blue	na
chrUn	10631610	10632287	678	SAT1_CF	blue	na
chrUn	10632299	10632982	684	SAT1_CF	blue	na
chrUn	10632995	10633495	501	SAT1_CF	blue	na
chrUn	10633533	10633936	404	SAT1_CF	blue	na
chrUn	10633947	10635355	1409	SAT1_CF	blue	na
chrUn	10635397	10636181	785	SAT1_CF	blue	na
chrUn	10636212	10637670	1459	SAT1_CF	blue	na
chrUn	10637682	10638366	685	SAT1_CF	blue	na
chrUn	10638378	10639061	684	SAT1_CF	blue	na
chrUn	10639074	10639574	501	SAT1_CF	blue	na
chrUn	10639629	10640464	836	SAT1_CF	blue	na
chrUn	10640507	10642007	1501	SAT1_CF	blue	na
chrUn	10642057	10642554	498	SAT1_CF	blue	na
chrUn	10642595	10644464	1870	SAT1_CF	blue	na
chrUn	10644476	10645114	639	SAT1_CF	blue	na
chrUn	10645143	10645243	101	SAT1_CF	blue	na
chrUn	10645256	10645758	503	SAT1_CF	blue	na
chrUn	10645798	10646757	960	SAT1_CF	blue	na
chrUn	10646801	10647797	997	SAT1_CF	blue	na
chrUn	10647840	10648512	673	SAT1_CF	blue	na
chrUn	10648525	10649088	564	SAT1_CF	blue	na
chrUn	10649127	10649845	719	SAT1_CF	blue	na
chrUn	10649888	10650554	667	SAT1_CF	blue	na
chrUn	10650566	10651041	476	SAT1_CF	blue	na

chrUn	10651111	10651772	662	SAT1_CF	blue	na
chrUn	10651795	10652338	544	SAT1_CF	blue	na
chrUn	10652366	10653494	1129	SAT1_CF	blue	na
chrUn	10653544	10654394	851	SAT1_CF	blue	na
chrUn	10654407	10655778	1372	SAT1_CF	blue	na
chrUn	10655865	10656778	914	SAT1_CF	blue	na
chrUn	10656857	10657814	958	SAT1_CF	blue	na
chrUn	10657862	10658537	676	SAT1_CF	blue	na
chrUn	10658549	10659479	931	SAT1_CF	blue	na
chrUn	10659544	10659701	158	SAT1_CF	blue	na
chrUn	10659729	10660240	512	SAT1_CF	blue	na
chrUn	10660320	10661618	1299	SAT1_CF	blue	na
chrUn	10661662	10662349	688	SAT1_CF	blue	na
chrUn	10662363	10662953	591	SAT1_CF	blue	na
chrUn	10662980	10663454	475	SAT1_CF	blue	na
chrUn	10663511	10666301	2791	SAT1_CF	blue	na
chrUn	10666356	10668035	1680	SAT1_CF	blue	na
chrUn	10668047	10668597	551	SAT1_CF	blue	na
chrUn	10668609	10668724	116	SAT1_CF	blue	na
chrUn	10668737	10669258	522	SAT1_CF	blue	na
chrUn	10669292	10669679	388	SAT1_CF	blue	na
chrUn	10669694	10670200	507	SAT1_CF	blue	na
chrUn	10670234	10671107	874	SAT1_CF	blue	na
chrUn	10671130	10672770	1641	SAT1_CF	blue	na
chrUn	10672797	10673277	481	SAT1_CF	blue	na
chrUn	10673303	10674368	1066	SAT1_CF	blue	na
chrUn	10674386	10675217	832	SAT1_CF	blue	na
chrUn	10675299	10677453	2155	SAT1_CF	blue	na
chrUn	10677470	10678348	879	SAT1_CF	blue	na
chrUn	10678408	10679081	674	SAT1_CF	blue	na
chrUn	10679093	10679763	671	SAT1_CF	blue	na
chrUn	10679789	10680290	502	SAT1_CF	blue	na
chrUn	10680376	10680893	518	SAT1_CF	blue	na
chrUn	10682298	10682892	595	SAT1_CF	blue	na
chrUn	10682941	10683960	1020	SAT1_CF	blue	na
chrUn	10683982	10684482	501	SAT1_CF	blue	na
chrUn	10684525	10684921	397	SAT1_CF	blue	na
chrUn	10684932	10686559	1628	SAT1_CF	blue	na
chrUn	10686602	10687274	673	SAT1_CF	blue	na
chrUn	10687288	10687789	502	SAT1_CF	blue	na
chrUn	10687876	10688812	937	SAT1_CF	blue	na
chrUn	10688891	10689642	752	SAT1_CF	blue	na

chrUn	10689677	10690349	673	SAT1_CF	blue	na
chrUn	10690361	10691292	932	SAT1_CF	blue	na
chrUn	10691357	10691514	158	SAT1_CF	blue	na
chrUn	10691534	10691613	80	SAT1_CF	blue	na
chrUn	10692215	10692501	287	CarSat1	red	na
chrUn	10692653	10693372	720	CarSat1	red	na
chrUn	10693367	10694496	1130	SAT1_CF	blue	na
chrUn	10694536	10697262	2727	SAT1_CF	blue	na
chrUn	10697273	10697665	393	SAT1_CF	blue	na
chrUn	10697667	10698107	441	CarSat1	red	na
chrUn	10698125	10699643	1519	CarSat1	red	na
chrUn	10699660	10700172	513	CarSat1	red	na
chrUn	10700353	10701447	1095	CarSat1	red	na
chrUn	10701469	10702172	704	CarSat1	red	na
chrUn	10728870	10730051	1182	Sat2CF	purple	na
chrUn	10754742	10755640	899	Sat2CF	purple	na
chrUn	10754805	10755595	791	Sat2CF	purple	na
chrUn	10778284	10779418	1135	Sat2CF	purple	na
chrUn	10797367	10798251	885	Sat2CF	purple	na
chrUn	10797513	10798251	739	Sat2CF	purple	na
chrUn	10820911	10821648	738	Sat2CF	purple	na
chrUn	10820985	10821646	662	Sat2CF	purple	na
chrUn	10838032	10839244	1213	Sat2CF	purple	na
chrUn	10854746	10855890	1145	Sat2CF	purple	na
chrUn	10854750	10856020	1271	Sat2CF	purple	na
chrUn	10872840	10873670	831	Sat2CF	purple	na
chrUn	10872924	10873649	726	Sat2CF	purple	na
chrUn	10883645	10884488	844	Sat2CF	purple	na
chrUn	10935585	10936315	731	Sat2CF	purple	na
chrUn	10935660	10936487	828	Sat2CF	purple	na
chrUn	10935732	10936469	738	Sat2CF	purple	na
chrUn	10960220	10961331	1112	Sat2CF	purple	na
chrUn	10961176	10961377	202	Sat2CF	purple	na
chrUn	10984139	10985136	998	Sat2CF	purple	na
chrUn	10985020	10985189	170	Sat2CF	purple	na
chrUn	10997594	10997984	391	Sat2CF	purple	na
chrUn	10997657	10998015	359	Sat2CF	purple	na
chrUn	11023379	11024588	1210	Sat2CF	purple	na
chrUn	11050878	11052094	1217	Sat2CF	purple	na
chrUn	11051006	11052089	1084	Sat2CF	purple	na
chrUn	11051965	11052134	170	Sat2CF	purple	na
chrUn	11074360	11075462	1103	Sat2CF	purple	na

chrUn	11075317	11075518	202	Sat2CF	purple	na
chrUn	11077156	11077562	407	CarSat1	red	na
chrUn	11078532	11079385	854	CarSat1	red	na
chrUn	11079406	11080851	1446	CarSat1	red	na
chrUn	11081010	11082463	1454	CarSat1	red	na
chrUn	11082613	11083462	850	CarSat1	red	na
chrUn	11083490	11085061	1572	CarSat1	red	na
chrUn	11085168	11086002	835	CarSat1	red	na
chrUn	11091697	11092551	855	CarSat1	red	na
chrUn	11092701	11094154	1454	CarSat1	red	na
chrUn	11094175	11095029	855	CarSat1	red	na
chrUn	11095179	11096632	1454	CarSat1	red	na
chrUn	11096794	11097508	715	CarSat1	red	na
chrUn	11097658	11099123	1466	CarSat1	red	na
chrUn	11099154	11100735	1582	CarSat1	red	na
chrUn	11100766	11101801	1036	CarSat1	red	na
chrUn	11101831	11103275	1445	CarSat1	red	na
chrUn	11103305	11104283	979	CarSat1	red	na
chrUn	11108663	11109230	568	CarSat1	red	na
chrUn	11109419	11109540	122	CarSat1	red	na
chrUn	11109558	11110353	796	CarSat1	red	na
chrUn	11110380	11110822	443	CarSat1	red	na
chrUn	11111011	11112169	1159	CarSat1	red	na
chrUn	11114802	11116052	1251	Sat2CF	purple	na
chrUn	11132843	11133698	856	Sat2CF	purple	na
chrUn	11148631	11149085	455	Sat2CF	purple	na
chrUn	11165438	11166205	768	Sat2CF	purple	na
chrUn	11165454	11166144	691	Sat2CF	purple	na
chrUn	11187349	11188052	704	CarSat1	red	na
chrUn	11188082	11189664	1583	CarSat1	red	na
chrUn	11189814	11190541	728	CarSat1	red	na
chrUn	11190700	11191416	717	CarSat1	red	na
chrUn	11191566	11192941	1376	CarSat1	red	na
chrUn	11193190	11193816	627	CarSat1	red	na
chrUn	11194045	11195495	1451	CarSat1	red	na
chrUn	11195662	11196367	706	CarSat1	red	na
chrUn	11196517	11197164	648	CarSat1	red	na
chrUn	11197182	11197968	787	CarSat1	red	na
chrUn	11198138	11198755	618	CarSat1	red	na
chrUn	11198779	11199576	798	CarSat1	red	na
chrUn	11199597	11200450	854	CarSat1	red	na
chrUn	11201420	11201826	407	CarSat1	red	na

chrUn	11203463	11203664	202	Sat2CF	purple	na
chrUn	11203535	11204621	1087	Sat2CF	purple	na
chrUn	11203549	11204487	939	Sat2CF	purple	na
chrUn	11219157	11220409	1253	Sat2CF	purple	na
chrUn	11219193	11220278	1086	Sat2CF	purple	na
chrUn	11246719	11247233	515	Sat2CF	purple	na
chrUn	11258949	11259073	125	Sat2CF	purple	na
chrUn	11258962	11259751	790	Sat2CF	purple	na
chrUn	11280298	11281293	996	Sat2CF	purple	na
chrUn	11304489	11304690	202	Sat2CF	purple	na
chrUn	11304519	11305582	1064	Sat2CF	purple	na
chrUn	11304651	11305574	924	Sat2CF	purple	na
chrUn	11332565	11333817	1253	Sat2CF	purple	na
chrUn	11332598	11333687	1090	Sat2CF	purple	na
chrUn	11369812	11370515	704	Sat2CF	purple	na
chrUn	11369898	11370798	901	Sat2CF	purple	na
chrUn	11369916	11370652	737	Sat2CF	purple	na
chrUn	11383737	11384808	1072	Sat2CF	purple	na
chrUn	11413042	11414218	1177	Sat2CF	purple	na
chrUn	11413071	11414085	1015	Sat2CF	purple	na
chrUn	11424378	11424546	169	Sat2CF	purple	na
chrUn	11424415	11425246	832	Sat2CF	purple	na
chrUn	11424436	11425162	727	Sat2CF	purple	na
chrUn	11438618	11438797	180	Sat2CF	purple	na
chrUn	11438639	11438937	299	Sat2CF	purple	na
chrUn	11438689	11439857	1169	Sat2CF	purple	na
chrUn	11454923	11455072	150	Sat2CF	purple	na
chrUn	11454943	11456091	1149	Sat2CF	purple	na
chrUn	11483054	11483726	673	Sat2CF	purple	na
chrUn	11483125	11484008	884	Sat2CF	purple	na
chrUn	11508980	11509130	151	Sat2CF	purple	na
chrUn	11509005	11510083	1079	Sat2CF	purple	na
chrUn	11509013	11509953	941	Sat2CF	purple	na
chrUn	11552033	11552173	141	Sat2CF	purple	na
chrUn	11552056	11552794	739	Sat2CF	purple	na
chrUn	11552058	11552720	663	Sat2CF	purple	na
chrUn	11573956	11574849	894	Sat2CF	purple	na
chrUn	11574036	11574773	738	Sat2CF	purple	na
chrUn	11574528	11574904	377	Sat2CF	purple	na
chrUn	11599230	11600313	1084	Sat2CF	purple	na
chrUn	11599999	11600392	394	Sat2CF	purple	na
chrUn	11618475	11619728	1254	Sat2CF	purple	na

chrUn	11619594	11619756	163	Sat2CF	purple	na
chrUn	11657648	11657828	181	Sat2CF	purple	na
chrUn	11657704	11658580	877	Sat2CF	purple	na
chrUn	11674170	11675288	1119	Sat2CF	purple	na
chrUn	11685155	11685859	705	CarSat1	red	na
chrUn	11686009	11687456	1448	CarSat1	red	na
chrUn	11687477	11690193	2717	CarSat1	red	na
chrUn	11690198	11690588	391	SAT1_CF	blue	na
chrUn	11690600	11691463	864	SAT1_CF	blue	na
chrUn	11691505	11692753	1249	SAT1_CF	blue	na
chrUn	11692748	11693470	723	CarSat1	red	na
chrUn	11693532	11693928	397	CarSat1	red	na
chrUn	12100324	12100616	293	CarSat1	red	na
chrUn	12216399	12216424	26	Sat19CF	na	na
chrUn	12269855	12271021	1167	SAT1_CF	blue	na
chrUn	12271037	12271730	694	SAT1_CF	blue	na
chrUn	12271765	12272730	966	SAT1_CF	blue	na
chrUn	12272742	12273416	675	SAT1_CF	blue	na
chrUn	12273429	12273563	135	SAT1_CF	blue	na
chrUn	12273605	12274787	1183	SAT1_CF	blue	na
chrUn	12274833	12275376	544	SAT1_CF	blue	na
chrUn	12275444	12275964	521	SAT1_CF	blue	na
chrUn	12275976	12277307	1332	SAT1_CF	blue	na
chrUn	12277351	12277775	425	SAT1_CF	blue	na
chrUn	12277795	12278306	512	SAT1_CF	blue	na
chrUn	12278386	12278967	582	SAT1_CF	blue	na
chrUn	12278979	12280162	1184	SAT1_CF	blue	na
chrUn	12280193	12281708	1516	SAT1_CF	blue	na
chrUn	12281764	12282265	502	SAT1_CF	blue	na
chrUn	12282278	12283647	1370	SAT1_CF	blue	na
chrUn	12283689	12286108	2420	SAT1_CF	blue	na
chrUn	12286120	12287486	1367	SAT1_CF	blue	na
chrUn	12287506	12287989	484	SAT1_CF	blue	na
chrUn	12288001	12288677	677	SAT1_CF	blue	na
chrUn	12288720	12289831	1112	SAT1_CF	blue	na
chrUn	12289874	12290458	585	SAT1_CF	blue	na
chrUn	12290470	12292067	1598	SAT1_CF	blue	na
chrUn	12292095	12292938	844	SAT1_CF	blue	na
chrUn	12292980	12294826	1847	SAT1_CF	blue	na
chrUn	12294864	12295364	501	SAT1_CF	blue	na
chrUn	12295386	12296060	675	SAT1_CF	blue	na
chrUn	12296072	12296714	643	SAT1_CF	blue	na

chrUn	12296739	12297462	724	SAT1_CF	blue	na
chrUn	12297490	12298052	563	SAT1_CF	blue	na
chrUn	12298132	12298859	728	SAT1_CF	blue	na
chrUn	12298872	12299374	503	SAT1_CF	blue	na
chrUn	12299386	12300833	1448	SAT1_CF	blue	na
chrUn	12300878	12304077	3200	SAT1_CF	blue	na
chrUn	12304202	12304255	54	SAT1_CF	blue	na
chrUn	12304293	12304792	500	SAT1_CF	blue	na
chrUn	12304805	12305565	761	SAT1_CF	blue	na
chrUn	12305577	12306254	678	SAT1_CF	blue	na
chrUn	12306315	12307511	1197	SAT1_CF	blue	na
chrUn	12307542	12308053	512	SAT1_CF	blue	na
chrUn	12308081	12309230	1150	SAT1_CF	blue	na
chrUn	12309243	12309919	677	SAT1_CF	blue	na
chrUn	12309962	12310502	541	SAT1_CF	blue	na
chrUn	12310517	12311954	1438	SAT1_CF	blue	na
chrUn	12312060	12312541	482	SAT1_CF	blue	na
chrUn	12312554	12312627	74	SAT1_CF	blue	na
chrUn	12312683	12313236	554	SAT1_CF	blue	na
chrUn	12313248	12314421	1174	SAT1_CF	blue	na
chrUn	12314502	12315050	549	SAT1_CF	blue	na
chrUn	12315093	12315295	203	SAT1_CF	blue	na
chrUn	12315475	12315661	187	SAT1_CF	blue	na
chrUn	12315719	12316199	481	SAT1_CF	blue	na
chrUn	12316212	12317052	841	SAT1_CF	blue	na
chrUn	12317063	12317569	507	SAT1_CF	blue	na
chrUn	12317581	12320885	3305	SAT1_CF	blue	na
chrUn	12320925	12321426	502	SAT1_CF	blue	na
chrUn	12321438	12321547	110	SAT1_CF	blue	na
chrUn	12321568	12322198	631	SAT1_CF	blue	na
chrUn	12322311	12322817	507	SAT1_CF	blue	na
chrUn	12322831	12323235	405	SAT1_CF	blue	na
chrUn	12323273	12323996	724	SAT1_CF	blue	na
chrUn	12324037	12325898	1862	SAT1_CF	blue	na
chrUn	12325913	12326692	780	SAT1_CF	blue	na
chrUn	12326704	12327379	676	SAT1_CF	blue	na
chrUn	12327422	12328895	1474	SAT1_CF	blue	na
chrUn	12328966	12329485	520	SAT1_CF	blue	na
chrUn	12329499	12330182	684	SAT1_CF	blue	na
chrUn	12330194	12330868	675	SAT1_CF	blue	na
chrUn	12330890	12331660	771	SAT1_CF	blue	na
chrUn	12331702	12332237	536	SAT1_CF	blue	na

chrUn	12332250	12332586	337	SAT1_CF	blue	na
chrUn	12332603	12333730	1128	SAT1_CF	blue	na
chrUn	12333750	12334512	763	SAT1_CF	blue	na
chrUn	12334524	12335194	671	SAT1_CF	blue	na
chrUn	12335238	12336858	1621	SAT1_CF	blue	na
chrUn	12336891	12337311	421	SAT1_CF	blue	na
chrUn	12337351	12337850	500	SAT1_CF	blue	na
chrUn	12337863	12338566	704	SAT1_CF	blue	na
chrUn	12338578	12339826	1249	SAT1_CF	blue	na
chrUn	12339848	12340514	667	SAT1_CF	blue	na
chrUn	12340558	12341217	660	SAT1_CF	blue	na
chrUn	12341235	12341747	513	SAT1_CF	blue	na
chrUn	12341775	12341910	136	SAT1_CF	blue	na
chrUn	12341976	12342477	502	SAT1_CF	blue	na
chrUn	12342504	12343252	749	SAT1_CF	blue	na
chrUn	12343264	12344542	1279	SAT1_CF	blue	na
chrUn	12344592	12345390	799	SAT1_CF	blue	na
chrUn	12345418	12346119	702	SAT1_CF	blue	na
chrUn	12346132	12346237	106	SAT1_CF	blue	na
chrUn	12346260	12347954	1695	SAT1_CF	blue	na
chrUn	12347997	12350023	2027	SAT1_CF	blue	na
chrUn	12350078	12351052	975	SAT1_CF	blue	na
chrUn	12351158	12351599	442	SAT1_CF	blue	na
chrUn	12351611	12352286	676	SAT1_CF	blue	na
chrUn	12352333	12354312	1980	SAT1_CF	blue	na
chrUn	12354401	12354894	494	SAT1_CF	blue	na
chrUn	12354909	12354987	79	SAT1_CF	blue	na
chrUn	12355043	12356084	1042	SAT1_CF	blue	na
chrUn	12356099	12356255	157	SAT1_CF	blue	na
chrUn	12356300	12356799	500	SAT1_CF	blue	na
chrUn	12356812	12358188	1377	SAT1_CF	blue	na
chrUn	12358202	12358590	389	SAT1_CF	blue	na
chrUn	12358694	12359236	543	SAT1_CF	blue	na
chrUn	12359248	12360568	1321	SAT1_CF	blue	na
chrUn	12360592	12360952	361	SAT1_CF	blue	na
chrUn	12360996	12361504	509	SAT1_CF	blue	na
chrUn	12361540	12361917	378	SAT1_CF	blue	na
chrUn	12361957	12362458	502	SAT1_CF	blue	na
chrUn	12362471	12363231	761	SAT1_CF	blue	na
chrUn	12363243	12364375	1133	SAT1_CF	blue	na
chrUn	12364387	12366512	2126	SAT1_CF	blue	na
chrUn	12366592	12367130	539	SAT1_CF	blue	na

chrUn	12367176	12367722	547	SAT1_CF	blue	na
chrUn	12367734	12368131	398	SAT1_CF	blue	na
chrUn	12368174	12368676	503	SAT1_CF	blue	na
chrUn	12368689	12368796	108	SAT1_CF	blue	na
chrUn	12368818	12370601	1784	SAT1_CF	blue	na
chrUn	12370644	12371526	883	SAT1_CF	blue	na
chrUn	12371557	12372102	546	SAT1_CF	blue	na
chrUn	12372146	12373963	1818	SAT1_CF	blue	na
chrUn	12374023	12374504	482	SAT1_CF	blue	na
chrUn	12374517	12374625	109	SAT1_CF	blue	na
chrUn	12374644	12375316	673	SAT1_CF	blue	na
chrUn	12375339	12375884	546	SAT1_CF	blue	na
chrUn	12375926	12376910	985	SAT1_CF	blue	na
chrUn	12376936	12377792	857	SAT1_CF	blue	na
chrUn	12377804	12378843	1040	SAT1_CF	blue	na
chrUn	12378869	12381611	2743	SAT1_CF	blue	na
chrUn	12381624	12382305	682	SAT1_CF	blue	na
chrUn	12382320	12383176	857	SAT1_CF	blue	na
chrUn	12383216	12383755	540	SAT1_CF	blue	na
chrUn	12383793	12385035	1243	SAT1_CF	blue	na
chrUn	12385071	12385571	501	SAT1_CF	blue	na
chrUn	12385583	12386257	675	SAT1_CF	blue	na
chrUn	12386296	12388109	1814	SAT1_CF	blue	na
chrUn	12388141	12388598	458	SAT1_CF	blue	na
chrUn	12388613	12388748	136	SAT1_CF	blue	na
chrUn	12388814	12389315	502	SAT1_CF	blue	na
chrUn	12389327	12390693	1367	SAT1_CF	blue	na
chrUn	12390706	12391596	891	SAT1_CF	blue	na
chrUn	12391607	12391926	320	SAT1_CF	blue	na
chrUn	12391999	12393176	1178	SAT1_CF	blue	na
chrUn	12393190	12394306	1117	SAT1_CF	blue	na
chrUn	12394333	12395467	1135	SAT1_CF	blue	na
chrUn	12395479	12396812	1334	SAT1_CF	blue	na
chrUn	12396837	12397041	205	SAT1_CF	blue	na
chrUn	12397092	12397654	563	SAT1_CF	blue	na
chrUn	12399069	12399641	573	SAT1_CF	blue	na
chrUn	12399681	12400186	506	SAT1_CF	blue	na
chrUn	12400199	12400305	107	SAT1_CF	blue	na
chrUn	12400326	12400871	546	SAT1_CF	blue	na
chrUn	12400913	12402947	2035	SAT1_CF	blue	na
chrUn	12402993	12403486	494	SAT1_CF	blue	na
chrUn	12403516	12404164	649	SAT1_CF	blue	na

chrUn	12404196	12404700	505	SAT1_CF	blue	na
chrUn	12404732	12405436	705	SAT1_CF	blue	na
chrUn	12405449	12405555	107	SAT1_CF	blue	na
chrUn	12405580	12407170	1591	SAT1_CF	blue	na
chrUn	12407184	12407732	549	SAT1_CF	blue	na
chrUn	12407745	12408287	543	SAT1_CF	blue	na
chrUn	12408300	12408883	584	SAT1_CF	blue	na
chrUn	12408914	12410516	1603	SAT1_CF	blue	na
chrUn	12410583	12411105	523	SAT1_CF	blue	na
chrUn	12411148	12411350	203	SAT1_CF	blue	na
chrUn	12411530	12411715	186	SAT1_CF	blue	na
chrUn	12411751	12412251	501	SAT1_CF	blue	na
chrUn	12412264	12412877	614	SAT1_CF	blue	na
chrUn	12412895	12413563	669	SAT1_CF	blue	na
chrUn	12413607	12414259	653	SAT1_CF	blue	na
chrUn	12414291	12415233	943	SAT1_CF	blue	na
chrUn	12415276	12416956	1681	SAT1_CF	blue	na
chrUn	12416968	12417689	722	SAT1_CF	blue	na
chrUn	12417712	12418443	732	SAT1_CF	blue	na
chrUn	12418456	12419038	583	SAT1_CF	blue	na
chrUn	12419070	12420067	998	SAT1_CF	blue	na
chrUn	12420087	12420194	108	SAT1_CF	blue	na
chrUn	12420215	12421405	1191	SAT1_CF	blue	na
chrUn	12421474	12421845	372	SAT1_CF	blue	na
chrUn	12421932	12422511	580	SAT1_CF	blue	na
chrUn	12422574	12424795	2222	SAT1_CF	blue	na
chrUn	12424809	12426173	1365	SAT1_CF	blue	na
chrUn	12426215	12426354	140	SAT1_CF	blue	na
chrUn	12426377	12426992	616	SAT1_CF	blue	na
chrUn	12427004	12427680	677	SAT1_CF	blue	na
chrUn	12427729	12428704	976	SAT1_CF	blue	na
chrUn	12428746	12429253	508	SAT1_CF	blue	na
chrUn	12429267	12429669	403	SAT1_CF	blue	na
chrUn	12429709	12430211	503	SAT1_CF	blue	na
chrUn	12430224	12430331	108	SAT1_CF	blue	na
chrUn	12430353	12430906	554	SAT1_CF	blue	na
chrUn	12430919	12431564	646	SAT1_CF	blue	na
chrUn	12431576	12432910	1335	SAT1_CF	blue	na
chrUn	12432925	12433789	865	SAT1_CF	blue	na
chrUn	12433968	12434152	185	SAT1_CF	blue	na
chrUn	12434173	12434690	518	SAT1_CF	blue	na
chrUn	12434717	12435594	878	SAT1_CF	blue	na

chrUn	12435636	12436178	543	SAT1_CF	blue	na
chrUn	12436189	12436888	700	SAT1_CF	blue	na
chrUn	12436935	12437784	850	SAT1_CF	blue	na
chrUn	12437797	12438060	264	SAT1_CF	blue	na
chrUn	12438149	12438669	521	SAT1_CF	blue	na
chrUn	12438683	12441248	2566	SAT1_CF	blue	na
chrUn	12441261	12441665	405	SAT1_CF	blue	na
chrUn	12441703	12442205	503	SAT1_CF	blue	na
chrUn	12442218	12442323	106	SAT1_CF	blue	na
chrUn	12442346	12442898	553	SAT1_CF	blue	na
chrUn	12442910	12443593	684	SAT1_CF	blue	na
chrUn	12443605	12444364	760	SAT1_CF	blue	na
chrUn	12444378	12445051	674	SAT1_CF	blue	na
chrUn	12445094	12446092	999	SAT1_CF	blue	na
chrUn	12446136	12447310	1175	SAT1_CF	blue	na
chrUn	12447338	12447495	158	SAT1_CF	blue	na
chrUn	12447540	12448041	502	SAT1_CF	blue	na
chrUn	12448054	12448160	107	SAT1_CF	blue	na
chrUn	12448183	12448974	792	SAT1_CF	blue	na
chrUn	12449016	12450621	1606	SAT1_CF	blue	na
chrUn	12450635	12451337	703	SAT1_CF	blue	na
chrUn	12451419	12453178	1760	SAT1_CF	blue	na
chrUn	12453189	12454223	1035	SAT1_CF	blue	na
chrUn	12454279	12455361	1083	SAT1_CF	blue	na
chrUn	12455410	12455502	93	SAT1_CF	blue	na
chrUn	12455559	12456116	558	SAT1_CF	blue	na
chrUn	12456128	12457843	1716	SAT1_CF	blue	na
chrUn	12457869	12458505	637	SAT1_CF	blue	na
chrUn	12458518	12458871	354	SAT1_CF	blue	na
chrUn	12458988	12459507	520	SAT1_CF	blue	na
chrUn	12459538	12460611	1074	SAT1_CF	blue	na
chrUn	12460625	12461514	890	SAT1_CF	blue	na
chrUn	12461601	12462101	501	SAT1_CF	blue	na
chrUn	12462115	12462872	758	SAT1_CF	blue	na
chrUn	12462884	12462993	110	SAT1_CF	blue	na
chrUn	12463014	12463558	545	SAT1_CF	blue	na
chrUn	12463607	12464614	1008	SAT1_CF	blue	na
chrUn	12464628	12465488	861	SAT1_CF	blue	na
chrUn	12465529	12467224	1696	SAT1_CF	blue	na
chrUn	12467405	12467588	184	SAT1_CF	blue	na
chrUn	12467607	12468127	521	SAT1_CF	blue	na
chrUn	12468140	12468245	106	SAT1_CF	blue	na

chrUn	12468268	12469538	1271	SAT1_CF	blue	na
chrUn	12469553	12469688	136	SAT1_CF	blue	na
chrUn	12469733	12470254	522	SAT1_CF	blue	na
chrUn	12470266	12471601	1336	SAT1_CF	blue	na
chrUn	12471645	12473681	2037	SAT1_CF	blue	na
chrUn	12473739	12474217	479	SAT1_CF	blue	na
chrUn	12474235	12474345	111	SAT1_CF	blue	na
chrUn	12474358	12475533	1176	SAT1_CF	blue	na
chrUn	12475566	12475722	157	SAT1_CF	blue	na
chrUn	12475747	12476267	521	SAT1_CF	blue	na
chrUn	12476280	12476954	675	SAT1_CF	blue	na
chrUn	12476997	12479036	2040	SAT1_CF	blue	na
chrUn	12479104	12481207	2104	SAT1_CF	blue	na
chrUn	12481258	12482048	791	SAT1_CF	blue	na
chrUn	12482092	12482633	542	SAT1_CF	blue	na
chrUn	12482653	12482816	164	SAT1_CF	blue	na
chrUn	12482829	12483231	403	SAT1_CF	blue	na
chrUn	12483291	12483766	476	SAT1_CF	blue	na
chrUn	12483800	12484941	1142	SAT1_CF	blue	na
chrUn	12485262	12485568	307	SAT1_CF	blue	na
chrUn	12485684	12486560	877	SAT1_CF	blue	na
chrUn	12486647	12487148	502	SAT1_CF	blue	na
chrUn	12487161	12489080	1920	SAT1_CF	blue	na
chrUn	12489092	12489201	110	SAT1_CF	blue	na
chrUn	12489222	12489849	628	SAT1_CF	blue	na
chrUn	12489863	12490537	675	SAT1_CF	blue	na
chrUn	12490656	12491196	541	SAT1_CF	blue	na
chrUn	12491240	12492207	968	SAT1_CF	blue	na
chrUn	12492252	12492622	371	SAT1_CF	blue	na
chrUn	12492678	12493157	480	SAT1_CF	blue	na
chrUn	12493184	12494615	1432	SAT1_CF	blue	na
chrUn	12494658	12495286	629	SAT1_CF	blue	na
chrUn	12495316	12497019	1704	SAT1_CF	blue	na
chrUn	12497052	12497188	137	SAT1_CF	blue	na
chrUn	12497254	12497755	502	SAT1_CF	blue	na
chrUn	12497767	12498444	678	SAT1_CF	blue	na
chrUn	12498487	12500111	1625	SAT1_CF	blue	na
chrUn	12500124	12500491	368	SAT1_CF	blue	na
chrUn	12500529	12501920	1392	SAT1_CF	blue	na
chrUn	12501938	12501994	57	SAT1_CF	blue	na
chrUn	12502014	12502686	673	SAT1_CF	blue	na
chrUn	12502700	12503248	549	SAT1_CF	blue	na

chrUn	12503291	12505684	2394	SAT1_CF	blue	na
chrUn	12505696	12506010	315	SAT1_CF	blue	na
chrUn	12506027	12506527	501	SAT1_CF	blue	na
chrUn	12506540	12507866	1327	SAT1_CF	blue	na
chrUn	12507911	12509123	1213	SAT1_CF	blue	na
chrUn	12509145	12509688	544	SAT1_CF	blue	na
chrUn	12509701	12509936	236	SAT1_CF	blue	na
chrUn	12510116	12510283	168	SAT1_CF	blue	na
chrUn	12510357	12510828	472	SAT1_CF	blue	na
chrUn	12510851	12512037	1187	SAT1_CF	blue	na
chrUn	12513497	12513879	383	SAT1_CF	blue	na
chrUn	12513957	12515403	1447	SAT1_CF	blue	na
chrUn	12515414	12515601	188	SAT1_CF	blue	na
chrUn	12515874	12516573	700	SAT1_CF	blue	na
chrUn	12516586	12517348	763	SAT1_CF	blue	na
chrUn	12517360	12518034	675	SAT1_CF	blue	na
chrUn	12518077	12519701	1625	SAT1_CF	blue	na
chrUn	12519717	12520102	386	SAT1_CF	blue	na
chrUn	12520136	12520656	521	SAT1_CF	blue	na
chrUn	12520668	12522719	2052	SAT1_CF	blue	na
chrUn	12522762	12523021	260	SAT1_CF	blue	na
chrUn	12523040	12523559	520	SAT1_CF	blue	na
chrUn	12523572	12525546	1975	SAT1_CF	blue	na
chrUn	12525560	12525697	138	SAT1_CF	blue	na
chrUn	12525712	12526105	394	SAT1_CF	blue	na
chrUn	12631070	12631396	327	SAT1_CF	blue	na
chrUn	12631469	12632563	1095	SAT1_CF	blue	na
chrUn	12632612	12633132	521	SAT1_CF	blue	na
chrUn	12633146	12633385	240	SAT1_CF	blue	na
chrUn	12633564	12633748	185	SAT1_CF	blue	na
chrUn	12633769	12634285	517	SAT1_CF	blue	na
chrUn	12634303	12634968	666	SAT1_CF	blue	na
chrUn	12635011	12636674	1664	SAT1_CF	blue	na
chrUn	12636717	12637864	1148	SAT1_CF	blue	na
chrUn	12637887	12638411	525	SAT1_CF	blue	na
chrUn	12638468	12639198	731	SAT1_CF	blue	na
chrUn	12639211	12640062	852	SAT1_CF	blue	na
chrUn	12640080	12640692	613	SAT1_CF	blue	na
chrUn	12640734	12641800	1067	SAT1_CF	blue	na
chrUn	12641981	12642162	182	SAT1_CF	blue	na
chrUn	12642222	12642781	560	SAT1_CF	blue	na
chrUn	12642843	12643472	630	SAT1_CF	blue	na

chrUn	12643486	12644160	675	SAT1_CF	blue	na
chrUn	12644203	12645858	1656	SAT1_CF	blue	na
chrUn	12645870	12646211	342	SAT1_CF	blue	na
chrUn	12646295	12646777	483	SAT1_CF	blue	na
chrUn	12646790	12648361	1572	SAT1_CF	blue	na
chrUn	12648428	12649277	850	SAT1_CF	blue	na
chrUn	12649364	12651172	1809	SAT1_CF	blue	na
chrUn	12651221	12651923	703	SAT1_CF	blue	na
chrUn	12651969	12652495	527	SAT1_CF	blue	na
chrUn	12652552	12653026	475	SAT1_CF	blue	na
chrUn	12653053	12653643	591	SAT1_CF	blue	na
chrUn	12653657	12654344	688	SAT1_CF	blue	na
chrUn	12654388	12655464	1077	SAT1_CF	blue	na
chrUn	12655508	12656468	961	SAT1_CF	blue	na
chrUn	12656528	12657458	931	SAT1_CF	blue	na
chrUn	12657470	12658801	1332	SAT1_CF	blue	na
chrUn	12658825	12659150	326	SAT1_CF	blue	na
chrUn	12659229	12660191	963	SAT1_CF	blue	na
chrUn	12660229	12661600	1372	SAT1_CF	blue	na
chrUn	12661613	12662463	851	SAT1_CF	blue	na
chrUn	12662481	12663625	1145	SAT1_CF	blue	na
chrUn	12663668	12664211	544	SAT1_CF	blue	na
chrUn	12664223	12664896	674	SAT1_CF	blue	na
chrUn	12664966	12665441	476	SAT1_CF	blue	na
chrUn	12665453	12666119	667	SAT1_CF	blue	na
chrUn	12666167	12667786	1620	SAT1_CF	blue	na
chrUn	12667818	12668183	366	SAT1_CF	blue	na
chrUn	12668217	12668738	522	SAT1_CF	blue	na
chrUn	12668780	12669092	313	SAT1_CF	blue	na
chrUn	12669134	12669779	646	SAT1_CF	blue	na
chrUn	12669833	12670422	590	SAT1_CF	blue	na
chrUn	12671829	12672346	518	SAT1_CF	blue	na
chrUn	12672433	12672927	495	SAT1_CF	blue	na
chrUn	12672947	12673629	683	SAT1_CF	blue	na
chrUn	12673652	12674315	664	SAT1_CF	blue	na
chrUn	12674358	12674989	632	SAT1_CF	blue	na
chrUn	12675017	12676219	1203	SAT1_CF	blue	na
chrUn	12676238	12676374	137	SAT1_CF	blue	na
chrUn	12676421	12676942	522	SAT1_CF	blue	na
chrUn	12676964	12677598	635	SAT1_CF	blue	na
chrUn	12677611	12678937	1327	SAT1_CF	blue	na
chrUn	12679018	12679737	720	SAT1_CF	blue	na

chrUn	12679751	12680188	438	SAT1_CF	blue	na
chrUn	12680211	12680892	682	SAT1_CF	blue	na
chrUn	12680904	12681754	851	SAT1_CF	blue	na
chrUn	12681772	12682383	612	SAT1_CF	blue	na
chrUn	12682425	12683248	824	SAT1_CF	blue	na
chrUn	12683291	12683657	367	SAT1_CF	blue	na
chrUn	12683729	12684248	520	SAT1_CF	blue	na
chrUn	12684305	12684851	547	SAT1_CF	blue	na
chrUn	12684893	12685619	727	SAT1_CF	blue	na
chrUn	12685664	12686199	536	SAT1_CF	blue	na
chrUn	12686221	12686739	519	SAT1_CF	blue	na
chrUn	12686751	12688068	1318	SAT1_CF	blue	na
chrUn	12688081	12688916	836	SAT1_CF	blue	na
chrUn	12688929	12689632	704	SAT1_CF	blue	na
chrUn	12689644	12692131	2488	SAT1_CF	blue	na
chrUn	12692199	12692717	519	SAT1_CF	blue	na
chrUn	12692729	12693488	760	SAT1_CF	blue	na
chrUn	12693502	12694834	1333	SAT1_CF	blue	na
chrUn	12694878	12695808	931	SAT1_CF	blue	na
chrUn	12695821	12696141	321	SAT1_CF	blue	na
chrUn	12699170	12700783	1614	SAT1_CF	blue	na
chrUn	12700825	12700989	165	SAT1_CF	blue	na
chrUn	12701171	12701356	186	SAT1_CF	blue	na
chrUn	12701373	12701891	519	SAT1_CF	blue	na
chrUn	12701905	12703710	1806	SAT1_CF	blue	na
chrUn	12703754	12704714	961	SAT1_CF	blue	na
chrUn	12704774	12705644	871	SAT1_CF	blue	na
chrUn	12706530	12706663	134	SAT1_CF	blue	na
chrUn	12706721	12707221	501	SAT1_CF	blue	na
chrUn	12707235	12707918	684	SAT1_CF	blue	na
chrUn	12707930	12708615	686	SAT1_CF	blue	na
chrUn	12708627	12708735	109	SAT1_CF	blue	na
chrUn	12708755	12709705	951	SAT1_CF	blue	na
chrUn	12709739	12710258	520	SAT1_CF	blue	na
chrUn	12710270	12710956	687	SAT1_CF	blue	na
chrUn	12710980	12712666	1687	SAT1_CF	blue	na
chrUn	12712746	12713292	547	SAT1_CF	blue	na
chrUn	12713340	12713740	401	SAT1_CF	blue	na
chrUn	12713800	12714370	571	SAT1_CF	blue	na
chrUn	12714389	12715878	1490	SAT1_CF	blue	na
chrUn	12715957	12716912	956	SAT1_CF	blue	na
chrUn	12716939	12718497	1559	SAT1_CF	blue	na

chrUn	12718546	12719139	594	SAT1_CF	blue	na
chrUn	12720536	12721109	574	SAT1_CF	blue	na
chrUn	12721149	12721643	495	SAT1_CF	blue	na
chrUn	12721663	12722345	683	SAT1_CF	blue	na
chrUn	12722368	12723034	667	SAT1_CF	blue	na
chrUn	12723077	12723706	630	SAT1_CF	blue	na
chrUn	12723734	12724926	1193	SAT1_CF	blue	na
chrUn	12724954	12725090	137	SAT1_CF	blue	na
chrUn	12725136	12725658	523	SAT1_CF	blue	na
chrUn	12725680	12728060	2381	SAT1_CF	blue	na
chrUn	12728071	12728563	493	SAT1_CF	blue	na
chrUn	12728577	12729942	1366	SAT1_CF	blue	na
chrUn	12730007	12730569	563	SAT1_CF	blue	na
chrUn	12730612	12731165	554	SAT1_CF	blue	na
chrUn	12731187	12731852	666	SAT1_CF	blue	na
chrUn	12731895	12732784	890	SAT1_CF	blue	na
chrUn	12732798	12733683	886	SAT1_CF	blue	na
chrUn	12733694	12734387	694	SAT1_CF	blue	na
chrUn	12734399	12735079	681	SAT1_CF	blue	na
chrUn	12735121	12735381	261	SAT1_CF	blue	na
chrUn	12735405	12736065	661	SAT1_CF	blue	na
chrUn	12736083	12736584	502	SAT1_CF	blue	na
chrUn	12736617	12738132	1516	SAT1_CF	blue	na
chrUn	12738158	12738700	543	SAT1_CF	blue	na
chrUn	12738786	12739109	324	SAT1_CF	blue	na
chrUn	12739185	12739815	631	SAT1_CF	blue	na
chrUn	12739871	12741108	1238	SAT1_CF	blue	na
chrUn	12741120	12742756	1637	SAT1_CF	blue	na
chrUn	12742768	12743428	661	SAT1_CF	blue	na
chrUn	12743480	12744287	808	SAT1_CF	blue	na
chrUn	12744319	12744824	506	SAT1_CF	blue	na
chrUn	12744836	12745513	678	SAT1_CF	blue	na
chrUn	12745582	12747791	2210	SAT1_CF	blue	na
chrUn	12747824	12748184	361	SAT1_CF	blue	na
chrUn	12748203	12748720	518	SAT1_CF	blue	na
chrUn	12748738	12748841	104	SAT1_CF	blue	na
chrUn	12748862	12749436	575	SAT1_CF	blue	na
chrUn	12749448	12750107	660	SAT1_CF	blue	na
chrUn	12750155	12750695	541	SAT1_CF	blue	na
chrUn	12750717	12751383	667	SAT1_CF	blue	na
chrUn	12751427	12751915	489	SAT1_CF	blue	na
chrUn	12751956	12752624	669	SAT1_CF	blue	na

chrUn	12752644	12752779	136	SAT1_CF	blue	na
chrUn	12752845	12753346	502	SAT1_CF	blue	na
chrUn	12753373	12754121	749	SAT1_CF	blue	na
chrUn	12754133	12754808	676	SAT1_CF	blue	na
chrUn	12754851	12755392	542	SAT1_CF	blue	na
chrUn	12755405	12755905	501	SAT1_CF	blue	na
chrUn	12755918	12756023	106	SAT1_CF	blue	na
chrUn	12756046	12756615	570	SAT1_CF	blue	na
chrUn	12756649	12757985	1337	SAT1_CF	blue	na
chrUn	12758013	12759902	1890	SAT1_CF	blue	na
chrUn	12759935	12760956	1022	SAT1_CF	blue	na
chrUn	12760997	12761498	502	SAT1_CF	blue	na
chrUn	12761511	12761575	65	SAT1_CF	blue	na
chrUn	12761639	12762851	1213	SAT1_CF	blue	na
chrUn	12762895	12764916	2022	SAT1_CF	blue	na
chrUn	12764972	12765446	475	SAT1_CF	blue	na
chrUn	12765478	12765982	505	SAT1_CF	blue	na
chrUn	12766014	12766150	137	SAT1_CF	blue	na
chrUn	12766216	12766717	502	SAT1_CF	blue	na
chrUn	12766730	12767332	603	SAT1_CF	blue	na
chrUn	12767344	12768019	676	SAT1_CF	blue	na
chrUn	12768062	12769581	1520	SAT1_CF	blue	na
chrUn	12771165	12771914	750	SAT1_CF	blue	na
chrUn	12771926	12773731	1806	SAT1_CF	blue	na
chrUn	12773749	12774681	933	SAT1_CF	blue	na
chrUn	12774724	12775226	503	SAT1_CF	blue	na
chrUn	12775238	12775923	686	SAT1_CF	blue	na
chrUn	12775966	12776514	549	SAT1_CF	blue	na
chrUn	12776556	12777337	782	SAT1_CF	blue	na
chrUn	12777416	12778375	960	SAT1_CF	blue	na
chrUn	12778415	12778916	502	SAT1_CF	blue	na
chrUn	12778929	12779609	681	SAT1_CF	blue	na
chrUn	12779627	12780215	589	SAT1_CF	blue	na
chrUn	12780227	12780310	84	SAT1_CF	blue	na
chrUn	12780323	12781275	953	SAT1_CF	blue	na
chrUn	12781323	12781814	492	SAT1_CF	blue	na
chrUn	12781840	12782443	604	SAT1_CF	blue	na
chrUn	12782455	12786029	3575	SAT1_CF	blue	na
chrUn	12786065	12786566	502	SAT1_CF	blue	na
chrUn	12786578	12786694	117	SAT1_CF	blue	na
chrUn	12786708	12787233	526	SAT1_CF	blue	na
chrUn	12787289	12787835	547	SAT1_CF	blue	na

chrUn	12787878	12788411	534	SAT1_CF	blue	na
chrUn	12788425	12789507	1083	SAT1_CF	blue	na
chrUn	12789518	12789674	157	SAT1_CF	blue	na
chrUn	12789721	12790242	522	SAT1_CF	blue	na
chrUn	12790255	12790861	607	SAT1_CF	blue	na
chrUn	12790873	12791549	677	SAT1_CF	blue	na
chrUn	12791592	12792592	1001	SAT1_CF	blue	na
chrUn	12792624	12793626	1003	SAT1_CF	blue	na
chrUn	12793655	12794173	519	SAT1_CF	blue	na
chrUn	12794185	12795045	861	SAT1_CF	blue	na
chrUn	12795087	12797191	2105	SAT1_CF	blue	na
chrUn	12797383	12798168	786	SAT1_CF	blue	na
chrUn	12798210	12799133	924	SAT1_CF	blue	na
chrUn	12799178	12800134	957	SAT1_CF	blue	na
chrUn	12800174	12800676	503	SAT1_CF	blue	na
chrUn	12800700	12802472	1773	SAT1_CF	blue	na
chrUn	12802539	12803037	499	SAT1_CF	blue	na
chrUn	12803056	12804422	1367	SAT1_CF	blue	na
chrUn	12804465	12805570	1106	SAT1_CF	blue	na
chrUn	12805604	12806556	953	SAT1_CF	blue	na
chrUn	12806578	12807253	676	SAT1_CF	blue	na
chrUn	12807296	12807844	549	SAT1_CF	blue	na
chrUn	12807886	12808427	542	SAT1_CF	blue	na
chrUn	12808470	12808979	510	SAT1_CF	blue	na
chrUn	12809006	12809140	135	SAT1_CF	blue	na
chrUn	12809218	12809700	483	SAT1_CF	blue	na
chrUn	12809712	12811076	1365	SAT1_CF	blue	na
chrUn	12811125	12812419	1295	SAT1_CF	blue	na
chrUn	12812484	12813692	1209	SAT1_CF	blue	na
chrUn	12813757	12814455	699	SAT1_CF	blue	na
chrUn	12814468	12815267	800	SAT1_CF	blue	na
chrUn	12815290	12816136	847	SAT1_CF	blue	na
chrUn	12816172	12816785	614	SAT1_CF	blue	na
chrUn	12816799	12817310	512	SAT1_CF	blue	na
chrUn	12817330	12817850	521	SAT1_CF	blue	na
chrUn	12817863	12818545	683	SAT1_CF	blue	na
chrUn	12818560	12819233	674	SAT1_CF	blue	na
chrUn	12819276	12820899	1624	SAT1_CF	blue	na
chrUn	12820978	12821518	541	SAT1_CF	blue	na
chrUn	12821606	12822107	502	SAT1_CF	blue	na
chrUn	12822119	12824730	2612	SAT1_CF	blue	na
chrUn	12824770	12825270	501	SAT1_CF	blue	na

chrUn	12825292	12827465	2174	SAT1_CF	blue	na
chrUn	12827486	12828093	608	SAT1_CF	blue	na
chrUn	12828106	12828263	158	SAT1_CF	blue	na
chrUn	12828310	12828830	521	SAT1_CF	blue	na
chrUn	12828843	12829449	607	SAT1_CF	blue	na
chrUn	12829461	12830369	909	SAT1_CF	blue	na
chrUn	12830397	12830942	546	SAT1_CF	blue	na
chrUn	12831033	12831770	738	SAT1_CF	blue	na
chrUn	12831803	12832152	350	SAT1_CF	blue	na
chrUn	12832208	12832708	501	SAT1_CF	blue	na
chrUn	12832722	12833404	683	SAT1_CF	blue	na
chrUn	12833416	12834099	684	SAT1_CF	blue	na
chrUn	12834110	12834602	493	SAT1_CF	blue	na
chrUn	12834616	12835274	659	SAT1_CF	blue	na
chrUn	12835295	12835997	703	SAT1_CF	blue	na
chrUn	12836062	12836622	561	SAT1_CF	blue	na
chrUn	12836636	12837234	599	SAT1_CF	blue	na
chrUn	12837246	12837853	608	SAT1_CF	blue	na
chrUn	12837866	12838370	505	SAT1_CF	blue	na
chrUn	12838392	12839067	676	SAT1_CF	blue	na
chrUn	12839085	12839753	669	SAT1_CF	blue	na
chrUn	12839801	12840751	951	SAT1_CF	blue	na
chrUn	12840830	12841378	549	SAT1_CF	blue	na
chrUn	12841410	12841786	377	SAT1_CF	blue	na
chrUn	12841829	12842331	503	SAT1_CF	blue	na
chrUn	12842343	12843120	778	SAT1_CF	blue	na
chrUn	12844533	12845133	601	SAT1_CF	blue	na
chrUn	12845146	12845648	503	SAT1_CF	blue	na
chrUn	12845661	12846263	603	SAT1_CF	blue	na
chrUn	12846275	12846949	675	SAT1_CF	blue	na
chrUn	12846992	12848085	1094	SAT1_CF	blue	na
chrUn	12848096	12848622	527	SAT1_CF	blue	na
chrUn	12848654	12849030	377	SAT1_CF	blue	na
chrUn	12849073	12849574	502	SAT1_CF	blue	na
chrUn	12849592	12850340	749	SAT1_CF	blue	na
chrUn	12850362	12851035	674	SAT1_CF	blue	na
chrUn	12851077	12851928	852	SAT1_CF	blue	na
chrUn	12851998	12852543	546	SAT1_CF	blue	na
chrUn	12852586	12853217	632	SAT1_CF	blue	na
chrUn	12853245	12853806	562	SAT1_CF	blue	na
chrUn	12853849	12854370	522	SAT1_CF	blue	na
chrUn	12854385	12855082	698	SAT1_CF	blue	na

chrUn	12855102	12857346	2245	SAT1_CF	blue	na
chrUn	12857389	12858410	1022	SAT1_CF	blue	na
chrUn	12858497	12858999	503	SAT1_CF	blue	na
chrUn	12859011	12859117	107	SAT1_CF	blue	na
chrUn	12859141	12859769	629	SAT1_CF	blue	na
chrUn	12859793	12860653	861	SAT1_CF	blue	na
chrUn	12860671	12861357	687	SAT1_CF	blue	na
chrUn	12861369	12862848	1480	SAT1_CF	blue	na
chrUn	12862890	12863068	179	SAT1_CF	blue	na
chrUn	12863093	12863636	544	SAT1_CF	blue	na
chrUn	12863650	12864052	403	SAT1_CF	blue	na
chrUn	12864111	12864591	481	SAT1_CF	blue	na
chrUn	12864604	12865736	1133	SAT1_CF	blue	na
chrUn	12865769	12866291	523	SAT1_CF	blue	na
chrUn	12866348	12866850	503	SAT1_CF	blue	na
chrUn	12866863	12867624	762	SAT1_CF	blue	na
chrUn	12867648	12868426	779	SAT1_CF	blue	na
chrUn	12868454	12869014	561	SAT1_CF	blue	na
chrUn	12869057	12869218	162	SAT1_CF	blue	na
chrUn	12869304	12869785	482	SAT1_CF	blue	na
chrUn	12869798	12870481	684	SAT1_CF	blue	na
chrUn	12870493	12871827	1335	SAT1_CF	blue	na
chrUn	12871871	12872221	351	SAT1_CF	blue	na
chrUn	12872265	12873225	961	SAT1_CF	blue	na
chrUn	12873265	12873886	622	SAT1_CF	blue	na
chrUn	12873910	12874417	508	SAT1_CF	blue	na
chrUn	12874432	12875245	814	SAT1_CF	blue	na
chrUn	12875297	12876111	815	SAT1_CF	blue	na
chrUn	12876140	12876768	629	SAT1_CF	blue	na
chrUn	12876803	12877652	850	SAT1_CF	blue	na
chrUn	12877670	12878356	687	SAT1_CF	blue	na
chrUn	12878368	12879014	647	SAT1_CF	blue	na
chrUn	12879047	12879569	523	SAT1_CF	blue	na
chrUn	12879584	12879720	137	SAT1_CF	blue	na
chrUn	12879786	12880280	495	SAT1_CF	blue	na
chrUn	12880300	12880982	683	SAT1_CF	blue	na
chrUn	12880995	12881667	673	SAT1_CF	blue	na
chrUn	12881764	12882306	543	SAT1_CF	blue	na
chrUn	12882350	12883599	1250	SAT1_CF	blue	na
chrUn	12883611	12883686	76	SAT1_CF	blue	na
chrUn	12883742	12884214	473	SAT1_CF	blue	na
chrUn	12884227	12884332	106	SAT1_CF	blue	na

chrUn	12884353	12884904	552	SAT1_CF	blue	na
chrUn	12884916	12887764	2849	SAT1_CF	blue	na
chrUn	12887819	12888311	493	SAT1_CF	blue	na
chrUn	12888345	12889006	662	SAT1_CF	blue	na
chrUn	12889054	12889932	879	SAT1_CF	blue	na
chrUn	12889946	12890454	509	SAT1_CF	blue	na
chrUn	12890467	12890568	102	SAT1_CF	blue	na
chrUn	12890596	12891673	1078	SAT1_CF	blue	na
chrUn	12891690	12891938	249	SAT1_CF	blue	na
chrUn	12892128	12892522	395	SAT1_CF	blue	na
chrUn	12892550	12892705	156	SAT1_CF	blue	na
chrUn	12892732	12893341	610	SAT1_CF	blue	na
chrUn	12893360	12894884	1525	SAT1_CF	blue	na
chrUn	12894928	12895842	915	SAT1_CF	blue	na
chrUn	12895910	12896519	610	SAT1_CF	blue	na
chrUn	12896538	12897090	553	SAT1_CF	blue	na
chrUn	12897133	12898096	964	SAT1_CF	blue	na
chrUn	12898123	12899536	1414	SAT1_CF	blue	na
chrUn	12899558	12900436	879	SAT1_CF	blue	na
chrUn	12900449	12901115	667	SAT1_CF	blue	na
chrUn	12901136	12902192	1057	SAT1_CF	blue	na
chrUn	12902219	12903517	1299	SAT1_CF	blue	na
chrUn	12903576	12904695	1120	SAT1_CF	blue	na
chrUn	12904718	12905382	665	SAT1_CF	blue	na
chrUn	12905424	12906383	960	SAT1_CF	blue	na
chrUn	12906450	12906963	514	SAT1_CF	blue	na
chrUn	12906974	12907807	834	SAT1_CF	blue	na
chrUn	12907820	12907896	77	SAT1_CF	blue	na
chrUn	12907949	12909833	1885	SAT1_CF	blue	na
chrUn	12909929	12910123	195	SAT1_CF	blue	na
chrUn	12910151	12910657	507	SAT1_CF	blue	na
chrUn	12910671	12911026	356	SAT1_CF	blue	na
chrUn	12911113	12911614	502	SAT1_CF	blue	na
chrUn	12911627	12912310	684	SAT1_CF	blue	na
chrUn	12912323	12912399	77	SAT1_CF	blue	na
chrUn	12912417	12912967	551	SAT1_CF	blue	na
chrUn	12912985	12914311	1327	SAT1_CF	blue	na
chrUn	12914336	12914579	244	SAT1_CF	blue	na
chrUn	12914630	12915175	546	SAT1_CF	blue	na
chrUn	12915192	12915287	96	SAT1_CF	blue	na
chrUn	12915298	12915652	355	SAT1_CF	blue	na
chrUn	12915720	12916239	520	SAT1_CF	blue	na

chrUn	12916252	12917777	1526	SAT1_CF	blue	na
chrUn	12917791	12918964	1174	SAT1_CF	blue	na
chrUn	12918977	12920099	1123	SAT1_CF	blue	na
chrUn	12920123	12921122	1000	SAT1_CF	blue	na
chrUn	12921209	12921707	499	SAT1_CF	blue	na
chrUn	12921720	12921827	108	SAT1_CF	blue	na
chrUn	12921848	12922402	555	SAT1_CF	blue	na
chrUn	12922414	12923097	684	SAT1_CF	blue	na
chrUn	12923109	12923785	677	SAT1_CF	blue	na
chrUn	12923828	12924902	1075	SAT1_CF	blue	na
chrUn	12924948	12925784	837	SAT1_CF	blue	na
chrUn	12925871	12926366	496	SAT1_CF	blue	na
chrUn	12926395	12927069	675	SAT1_CF	blue	na
chrUn	12927081	12927989	909	SAT1_CF	blue	na
chrUn	12928019	12929750	1732	SAT1_CF	blue	na
chrUn	12929766	12930444	679	SAT1_CF	blue	na
chrUn	12930457	12930957	501	SAT1_CF	blue	na
chrUn	12930969	12931044	76	SAT1_CF	blue	na
chrUn	12931065	12931735	671	SAT1_CF	blue	na
chrUn	12931749	12932266	518	SAT1_CF	blue	na
chrUn	12932310	12933278	969	SAT1_CF	blue	na
chrUn	12933359	12933525	167	SAT1_CF	blue	na
chrUn	12933706	12933891	186	SAT1_CF	blue	na
chrUn	12933927	12934427	501	SAT1_CF	blue	na
chrUn	12934440	12935188	749	SAT1_CF	blue	na
chrUn	12935201	12935998	798	SAT1_CF	blue	na
chrUn	12939934	12941974	2041	SAT1_CF	blue	na
chrUn	12941992	12942915	924	SAT1_CF	blue	na
chrUn	12942948	12944309	1362	SAT1_CF	blue	na
chrUn	12959085	12959234	150	Sat2CF	purple	na
chrUn	12959105	12960318	1214	Sat2CF	purple	na
chrUn	13014633	13014762	130	Sat2CF	purple	na
chrUn	13014655	13015468	814	Sat2CF	purple	na
chrUn	13015266	13015495	230	Sat2CF	purple	na
chrUn	13029543	13029826	284	Sat2CF	purple	na
chrUn	13029555	13029889	335	Sat2CF	purple	na
chrUn	13042305	13042490	186	Sat2CF	purple	na
chrUn	13042361	13043364	1004	Sat2CF	purple	na
chrUn	13066267	13067444	1178	Sat2CF	purple	na
chrUn	13066298	13067316	1019	Sat2CF	purple	na
chrUn	13095986	13097232	1247	Sat2CF	purple	na
chrUn	13112364	13113593	1230	Sat2CF	purple	na

chrUn	13127660	13128766	1107	Sat2CF	purple	na
chrUn	13140268	13140637	370	Sat2CF	purple	na
chrUn	13140318	13141116	799	Sat2CF	purple	na
chrUn	13181819	13182993	1175	Sat2CF	purple	na
chrUn	13213567	13214528	962	CarSat1	red	na
chrUn	13214533	13214964	432	SAT1_CF	blue	na
chrUn	13214987	13216841	1855	SAT1_CF	blue	na
chrUn	13216883	13218242	1360	SAT1_CF	blue	na
chrUn	13218237	13219118	882	CarSat1	red	na
chrUn	13219150	13220387	1238	CarSat1	red	na
chrUn	13220408	13221258	851	CarSat1	red	na
chrUn	13221275	13221417	143	CarSat1	red	na
chrUn	13222223	13222636	414	CarSat1	red	na
chrUn	13224346	13225283	938	Sat2CF	purple	na
chrUn	13224487	13225429	943	Sat2CF	purple	na
chrUn	13240936	13241085	150	Sat2CF	purple	na
chrUn	13240956	13242149	1194	Sat2CF	purple	na
chrUn	13242015	13242177	163	Sat2CF	purple	na
chrUn	13285951	13286152	202	Sat2CF	purple	na
chrUn	13286044	13286840	797	Sat2CF	purple	na
chrUn	13286729	13286866	138	Sat2CF	purple	na
chrUn	13317618	13317766	149	Sat2CF	purple	na
chrUn	13317643	13318716	1074	Sat2CF	purple	na
chrUn	13318070	13318803	734	Sat2CF	purple	na
chrUn	13328910	13329813	904	Sat2CF	purple	na
chrUn	13328931	13329733	803	Sat2CF	purple	na
chrUn	13344778	13344957	180	Sat2CF	purple	na
chrUn	13344828	13346009	1182	Sat2CF	purple	na
chrUn	13361985	13362738	754	Sat2CF	purple	na
chrUn	13362008	13362676	669	Sat2CF	purple	na
chrUn	13385146	13386099	954	Sat2CF	purple	na
chrUn	13385226	13385964	739	Sat2CF	purple	na
chrUn	13385226	13385964	739	Sat2CF	purple	na
chrUn	13385146	13386099	954	Sat2CF	purple	na
chrUn	13404109	13405244	1136	Sat2CF	purple	na
chrUn	13425234	13426028	795	Sat2CF	purple	na
chrUn	13453171	13454181	1011	Sat2CF	purple	na
chrUn	13453282	13454321	1040	Sat2CF	purple	na
chrUn	13478597	13479304	708	CarSat1	red	na
chrUn	13479453	13480905	1453	CarSat1	red	na
chrUn	13480926	13481703	778	CarSat1	red	na
chrUn	13481726	13482442	717	CarSat1	red	na

chrUn	13482460	13483994	1535	CarSat1	red	na
chrUn	13484015	13485254	1240	CarSat1	red	na
chrUn	13485257	13487053	1797	SAT1_CF	blue	na
chrUn	13490731	13491690	960	Sat2CF	purple	na
chrUn	13490794	13491647	854	Sat2CF	purple	na
chrUn	13491882	13492757	876	SAT1_CF	blue	na
chrUn	13492750	13493472	723	CarSat1	red	na
chrUn	13493506	13493636	131	CarSat1	red	na
chrUn	13495006	13495645	640	SAT1_CF	blue	na
chrUn	13495661	13496223	563	SAT1_CF	blue	na
chrUn	13496266	13497524	1259	SAT1_CF	blue	na
chrUn	13497603	13498127	525	SAT1_CF	blue	na
chrUn	13498142	13498771	630	SAT1_CF	blue	na
chrUn	13498783	13499456	674	SAT1_CF	blue	na
chrUn	13499499	13503584	4086	SAT1_CF	blue	na
chrUn	13503627	13505252	1626	SAT1_CF	blue	na
chrUn	13505295	13505666	372	SAT1_CF	blue	na
chrUn	13505724	13506202	479	SAT1_CF	blue	na
chrUn	13506214	13506890	677	SAT1_CF	blue	na
chrUn	13506933	13507496	564	SAT1_CF	blue	na
chrUn	13507507	13508187	681	SAT1_CF	blue	na
chrUn	13508199	13508876	678	SAT1_CF	blue	na
chrUn	13508923	13509041	119	SAT1_CF	blue	na
chrUn	13509083	13510902	1820	SAT1_CF	blue	na
chrUn	13510961	13511443	483	SAT1_CF	blue	na
chrUn	13511470	13512134	665	SAT1_CF	blue	na
chrUn	13512146	13512829	684	SAT1_CF	blue	na
chrUn	13512841	13513725	885	SAT1_CF	blue	na
chrUn	13513747	13514596	850	SAT1_CF	blue	na
chrUn	13514609	13515293	685	SAT1_CF	blue	na
chrUn	13515321	13515979	659	SAT1_CF	blue	na
chrUn	13516022	13516561	540	SAT1_CF	blue	na
chrUn	13516593	13517649	1057	SAT1_CF	blue	na
chrUn	13517662	13518495	834	SAT1_CF	blue	na
chrUn	13518521	13519263	743	SAT1_CF	blue	na
chrUn	13519333	13520779	1447	SAT1_CF	blue	na
chrUn	13520798	13521940	1143	SAT1_CF	blue	na
chrUn	13521961	13522479	519	SAT1_CF	blue	na
chrUn	13522501	13523097	597	SAT1_CF	blue	na
chrUn	13523119	13523792	674	SAT1_CF	blue	na
chrUn	13523810	13524479	670	SAT1_CF	blue	na
chrUn	13524522	13526553	2032	SAT1_CF	blue	na

chrUn	13526612	13527094	483	SAT1_CF	blue	na
chrUn	13527121	13527791	671	SAT1_CF	blue	na
chrUn	13527803	13528657	855	SAT1_CF	blue	na
chrUn	13528707	13529480	774	SAT1_CF	blue	na
chrUn	13529524	13530071	548	SAT1_CF	blue	na
chrUn	13530136	13530272	137	SAT1_CF	blue	na
chrUn	13530319	13530839	521	SAT1_CF	blue	na
chrUn	13530852	13530960	109	SAT1_CF	blue	na
chrUn	13530981	13531526	546	SAT1_CF	blue	na
chrUn	13531569	13533158	1590	SAT1_CF	blue	na
chrUn	13533169	13533540	372	SAT1_CF	blue	na
chrUn	13533597	13534096	500	SAT1_CF	blue	na
chrUn	13534123	13534793	671	SAT1_CF	blue	na
chrUn	13534805	13535480	676	SAT1_CF	blue	na
chrUn	13535522	13536563	1042	SAT1_CF	blue	na
chrUn	13536606	13536979	374	SAT1_CF	blue	na
chrUn	13537034	13537651	618	SAT1_CF	blue	na
chrUn	13537977	13541334	3358	SAT1_CF	blue	na
chrUn	13541347	13541453	107	SAT1_CF	blue	na
chrUn	13541476	13542099	624	SAT1_CF	blue	na
chrUn	13542112	13544414	2303	SAT1_CF	blue	na
chrUn	13544428	13545263	836	SAT1_CF	blue	na
chrUn	13545276	13545382	107	SAT1_CF	blue	na
chrUn	13545405	13546029	625	SAT1_CF	blue	na
chrUn	13546042	13548719	2678	SAT1_CF	blue	na
chrUn	13548806	13549307	502	SAT1_CF	blue	na
chrUn	13549320	13549996	677	SAT1_CF	blue	na
chrUn	13550010	13550870	861	SAT1_CF	blue	na
chrUn	13550912	13551460	549	SAT1_CF	blue	na
chrUn	13551473	13552427	955	SAT1_CF	blue	na
chrUn	13552506	13553054	549	SAT1_CF	blue	na
chrUn	13553071	13553464	394	SAT1_CF	blue	na
chrUn	13553504	13554006	503	SAT1_CF	blue	na
chrUn	13554028	13554708	681	SAT1_CF	blue	na
chrUn	13554751	13556375	1625	SAT1_CF	blue	na
chrUn	13556418	13556791	374	SAT1_CF	blue	na
chrUn	13556827	13557329	503	SAT1_CF	blue	na
chrUn	13557341	13557450	110	SAT1_CF	blue	na
chrUn	13557471	13558584	1114	SAT1_CF	blue	na
chrUn	13558631	13559177	547	SAT1_CF	blue	na
chrUn	13559190	13559557	368	SAT1_CF	blue	na
chrUn	13559576	13560097	522	SAT1_CF	blue	na

chrUn	13560115	13560224	110	SAT1_CF	blue	na
chrUn	13560238	13560756	519	SAT1_CF	blue	na
chrUn	13560776	13562329	1554	SAT1_CF	blue	na
chrUn	13562365	13562867	503	SAT1_CF	blue	na
chrUn	13562880	13562987	108	SAT1_CF	blue	na
chrUn	13563009	13563559	551	SAT1_CF	blue	na
chrUn	13563638	13564163	526	SAT1_CF	blue	na
chrUn	13564178	13564873	696	SAT1_CF	blue	na
chrUn	13564907	13565652	746	SAT1_CF	blue	na
chrUn	13565664	13566519	856	SAT1_CF	blue	na
chrUn	13566532	13568424	1893	SAT1_CF	blue	na
chrUn	13568464	13568965	502	SAT1_CF	blue	na
chrUn	13568978	13570198	1221	SAT1_CF	blue	na
chrUn	13570241	13571629	1389	SAT1_CF	blue	na
chrUn	13571672	13572098	427	SAT1_CF	blue	na
chrUn	13572111	13572653	543	SAT1_CF	blue	na
chrUn	13572665	13573350	686	SAT1_CF	blue	na
chrUn	13573362	13574046	685	SAT1_CF	blue	na
chrUn	13574059	13574898	840	SAT1_CF	blue	na
chrUn	13574911	13576100	1190	SAT1_CF	blue	na
chrUn	13576143	13576633	491	SAT1_CF	blue	na
chrUn	13576677	13577468	792	SAT1_CF	blue	na
chrUn	13577648	13577829	182	SAT1_CF	blue	na
chrUn	13577888	13578363	476	SAT1_CF	blue	na
chrUn	13578396	13580398	2003	SAT1_CF	blue	na
chrUn	13580478	13581001	524	SAT1_CF	blue	na
chrUn	13581016	13581145	130	SAT1_CF	blue	na
chrUn	13581217	13581719	503	SAT1_CF	blue	na
chrUn	13581732	13582494	763	SAT1_CF	blue	na
chrUn	13582507	13583173	667	SAT1_CF	blue	na
chrUn	13583271	13583536	266	SAT1_CF	blue	na
chrUn	13583563	13585312	1750	SAT1_CF	blue	na
chrUn	13585324	13586806	1483	SAT1_CF	blue	na
chrUn	13586831	13587753	923	SAT1_CF	blue	na
chrUn	13587774	13588294	521	SAT1_CF	blue	na
chrUn	13588307	13588415	109	SAT1_CF	blue	na
chrUn	13588436	13588981	546	SAT1_CF	blue	na
chrUn	13589024	13589985	962	SAT1_CF	blue	na
chrUn	13590064	13591022	959	SAT1_CF	blue	na
chrUn	13591062	13591564	503	SAT1_CF	blue	na
chrUn	13591577	13592244	668	SAT1_CF	blue	na
chrUn	13592297	13592840	544	SAT1_CF	blue	na

chrUn	13592910	13594067	1158	SAT1_CF	blue	na
chrUn	13594085	13594195	111	SAT1_CF	blue	na
chrUn	13594208	13594759	552	SAT1_CF	blue	na
chrUn	13594773	13597682	2910	SAT1_CF	blue	na
chrUn	13597694	13597866	173	SAT1_CF	blue	na
chrUn	13597908	13599342	1435	SAT1_CF	blue	na
chrUn	13599359	13599920	562	SAT1_CF	blue	na
chrUn	13599947	13600650	704	SAT1_CF	blue	na
chrUn	13600662	13601996	1335	SAT1_CF	blue	na
chrUn	13602021	13602346	326	SAT1_CF	blue	na
chrUn	13602424	13602973	550	SAT1_CF	blue	na
chrUn	13603004	13603850	847	SAT1_CF	blue	na
chrUn	13603862	13603974	113	SAT1_CF	blue	na
chrUn	13603991	13604718	728	SAT1_CF	blue	na
chrUn	13604771	13606205	1435	SAT1_CF	blue	na
chrUn	13606249	13606962	714	SAT1_CF	blue	na
chrUn	13606978	13608769	1792	SAT1_CF	blue	na
chrUn	13608788	13610261	1474	SAT1_CF	blue	na
chrUn	13610304	13611376	1073	SAT1_CF	blue	na
chrUn	13611412	13612206	795	SAT1_CF	blue	na
chrUn	13613056	13613458	403	SAT1_CF	blue	na
chrUn	13613478	13614850	1373	SAT1_CF	blue	na
chrUn	13614911	13617035	2125	SAT1_CF	blue	na
chrUn	13617113	13617657	545	SAT1_CF	blue	na
chrUn	13617670	13617777	108	SAT1_CF	blue	na
chrUn	13617799	13618422	624	SAT1_CF	blue	na
chrUn	13618451	13619143	693	SAT1_CF	blue	na
chrUn	13619154	13619694	541	SAT1_CF	blue	na
chrUn	13619709	13620390	682	SAT1_CF	blue	na
chrUn	13620402	13621082	681	SAT1_CF	blue	na
chrUn	13621093	13621695	603	SAT1_CF	blue	na
chrUn	13621707	13623636	1930	SAT1_CF	blue	na
chrUn	13623648	13624497	850	SAT1_CF	blue	na
chrUn	13624510	13626402	1893	SAT1_CF	blue	na
chrUn	13626442	13626943	502	SAT1_CF	blue	na
chrUn	13626970	13627716	747	SAT1_CF	blue	na
chrUn	13627733	13628347	615	SAT1_CF	blue	na
chrUn	13628359	13629035	677	SAT1_CF	blue	na
chrUn	13629078	13630515	1438	SAT1_CF	blue	na
chrUn	13630581	13631437	857	SAT1_CF	blue	na
chrUn	13631636	13632396	761	SAT1_CF	blue	na
chrUn	13632436	13632937	502	SAT1_CF	blue	na

chrUn	13632950	13633553	604	SAT1_CF	blue	na
chrUn	13633565	13634419	855	SAT1_CF	blue	na
chrUn	13634432	13634941	510	SAT1_CF	blue	na
chrUn	13635103	13636260	1158	SAT1_CF	blue	na
chrUn	13636304	13636847	544	SAT1_CF	blue	na
chrUn	13636861	13637215	355	SAT1_CF	blue	na
chrUn	13637302	13637803	502	SAT1_CF	blue	na
chrUn	13637824	13640921	3098	SAT1_CF	blue	na
chrUn	13640965	13641514	550	SAT1_CF	blue	na
chrUn	13641557	13642187	631	SAT1_CF	blue	na
chrUn	13642214	13642777	564	SAT1_CF	blue	na
chrUn	13642857	13644431	1575	SAT1_CF	blue	na
chrUn	13644443	13646231	1789	SAT1_CF	blue	na
chrUn	13646311	13646476	166	SAT1_CF	blue	na
chrUn	13646657	13646842	186	SAT1_CF	blue	na
chrUn	13646859	13647378	520	SAT1_CF	blue	na
chrUn	13647391	13647496	106	SAT1_CF	blue	na
chrUn	13647519	13649214	1696	SAT1_CF	blue	na
chrUn	13649227	13649332	106	SAT1_CF	blue	na
chrUn	13649355	13649986	632	SAT1_CF	blue	na
chrUn	13649998	13650681	684	SAT1_CF	blue	na
chrUn	13650693	13650765	73	SAT1_CF	blue	na
chrUn	13650791	13651584	794	SAT1_CF	blue	na
chrUn	13651597	13652243	647	SAT1_CF	blue	na
chrUn	13652255	13653752	1498	SAT1_CF	blue	na
chrUn	13653838	13654333	496	SAT1_CF	blue	na
chrUn	13654353	13655034	682	SAT1_CF	blue	na
chrUn	13655048	13655157	110	SAT1_CF	blue	na
chrUn	13655178	13655905	728	SAT1_CF	blue	na
chrUn	13655947	13656491	545	SAT1_CF	blue	na
chrUn	13656506	13657392	887	SAT1_CF	blue	na
chrUn	13657435	13657802	368	SAT1_CF	blue	na
chrUn	13657862	13658344	483	SAT1_CF	blue	na
chrUn	13658357	13659032	676	SAT1_CF	blue	na
chrUn	13659077	13660149	1073	SAT1_CF	blue	na
chrUn	13660181	13660700	520	SAT1_CF	blue	na
chrUn	13660713	13661115	403	SAT1_CF	blue	na
chrUn	13661155	13661656	502	SAT1_CF	blue	na
chrUn	13661669	13661777	109	SAT1_CF	blue	na
chrUn	13661798	13662587	790	SAT1_CF	blue	na
chrUn	13662657	13663410	754	SAT1_CF	blue	na
chrUn	13663488	13663967	480	SAT1_CF	blue	na

chrUn	13663998	13665613	1616	SAT1_CF	blue	na
chrUn	13665670	13666626	957	SAT1_CF	blue	na
chrUn	13666668	13667167	500	SAT1_CF	blue	na
chrUn	13667180	13668552	1373	SAT1_CF	blue	na
chrUn	13668595	13669585	991	SAT1_CF	blue	na
chrUn	13669607	13670151	545	SAT1_CF	blue	na
chrUn	13670162	13670396	235	SAT1_CF	blue	na
chrUn	13670576	13670743	168	SAT1_CF	blue	na
chrUn	13670817	13671288	472	SAT1_CF	blue	na
chrUn	13671311	13672634	1324	SAT1_CF	blue	na
chrUn	13672649	13672806	158	SAT1_CF	blue	na
chrUn	13672832	13674226	1395	SAT1_CF	blue	na
chrUn	13674304	13675749	1446	SAT1_CF	blue	na
chrUn	13675830	13675996	167	SAT1_CF	blue	na
chrUn	13676177	13676361	185	SAT1_CF	blue	na
chrUn	13676397	13676894	498	SAT1_CF	blue	na
chrUn	13676907	13677008	102	SAT1_CF	blue	na
chrUn	13677036	13678068	1033	SAT1_CF	blue	na
chrUn	13678108	13678606	499	SAT1_CF	blue	na
chrUn	13678618	13679952	1335	SAT1_CF	blue	na
chrUn	13679996	13680137	142	SAT1_CF	blue	na
chrUn	13680381	13681329	949	SAT1_CF	blue	na
chrUn	13681342	13682464	1123	SAT1_CF	blue	na
chrUn	13682488	13683493	1006	SAT1_CF	blue	na
chrUn	13683579	13684081	503	SAT1_CF	blue	na
chrUn	13684094	13685428	1335	SAT1_CF	blue	na
chrUn	13685474	13685810	337	SAT1_CF	blue	na
chrUn	13685854	13686738	885	SAT1_CF	blue	na
chrUn	13686759	13687280	522	SAT1_CF	blue	na
chrUn	13687293	13687401	109	SAT1_CF	blue	na
chrUn	13687422	13687977	556	SAT1_CF	blue	na
chrUn	13687989	13688664	676	SAT1_CF	blue	na
chrUn	13688761	13689303	543	SAT1_CF	blue	na
chrUn	13689347	13690320	974	SAT1_CF	blue	na
chrUn	13690331	13692045	1715	SAT1_CF	blue	na
chrUn	13692089	13692962	874	SAT1_CF	blue	na
chrUn	13694370	13694946	577	SAT1_CF	blue	na
chrUn	13695006	13695480	475	SAT1_CF	blue	na
chrUn	13695500	13696395	896	SAT1_CF	blue	na
chrUn	13696412	13697870	1459	SAT1_CF	blue	na
chrUn	13697883	13699656	1774	SAT1_CF	blue	na
chrUn	13699743	13700245	503	SAT1_CF	blue	na

chrUn	13700272	13700362	91	SAT1_CF	blue	na
chrUn	13700386	13701639	1254	SAT1_CF	blue	na
chrUn	13701663	13702258	596	SAT1_CF	blue	na
chrUn	13702270	13703178	909	SAT1_CF	blue	na
chrUn	13703208	13704831	1624	SAT1_CF	blue	na
chrUn	13704910	13705458	549	SAT1_CF	blue	na
chrUn	13705470	13705579	110	SAT1_CF	blue	na
chrUn	13705600	13707308	1709	SAT1_CF	blue	na
chrUn	13707353	13707854	502	SAT1_CF	blue	na
chrUn	13707881	13708546	666	SAT1_CF	blue	na
chrUn	13708570	13709418	849	SAT1_CF	blue	na
chrUn	13709460	13710002	543	SAT1_CF	blue	na
chrUn	13710067	13711910	1844	SAT1_CF	blue	na
chrUn	13711958	13712916	959	SAT1_CF	blue	na
chrUn	13712995	13713908	914	SAT1_CF	blue	na
chrUn	13713995	13714496	502	SAT1_CF	blue	na
chrUn	13714515	13714618	104	SAT1_CF	blue	na
chrUn	13714639	13715182	544	SAT1_CF	blue	na
chrUn	13715225	13716852	1628	SAT1_CF	blue	na
chrUn	13716863	13717249	387	SAT1_CF	blue	na
chrUn	13717302	13717802	501	SAT1_CF	blue	na
chrUn	13717844	13718157	314	SAT1_CF	blue	na
chrUn	13718199	13718844	646	SAT1_CF	blue	na
chrUn	13718893	13719487	595	SAT1_CF	blue	na
chrUn	13720884	13721457	574	SAT1_CF	blue	na
chrUn	13721497	13721991	495	SAT1_CF	blue	na
chrUn	13722011	13722693	683	SAT1_CF	blue	na
chrUn	13722716	13723382	667	SAT1_CF	blue	na
chrUn	13723425	13724054	630	SAT1_CF	blue	na
chrUn	13724082	13725461	1380	SAT1_CF	blue	na
chrUn	13725505	13726007	503	SAT1_CF	blue	na
chrUn	13726029	13727311	1283	SAT1_CF	blue	na
chrUn	13727325	13729038	1714	SAT1_CF	blue	na
chrUn	13729080	13730115	1036	SAT1_CF	blue	na
chrUn	13730162	13730837	676	SAT1_CF	blue	na
chrUn	13730933	13731451	519	SAT1_CF	blue	na
chrUn	13731483	13732009	527	SAT1_CF	blue	na
chrUn	13732053	13733461	1409	SAT1_CF	blue	na
chrUn	13733486	13735352	1867	SAT1_CF	blue	na
chrUn	13735396	13736330	935	SAT1_CF	blue	na
chrUn	13736371	13737029	659	SAT1_CF	blue	na
chrUn	13737073	13737850	778	SAT1_CF	blue	na

chrUn	13737967	13738891	925	SAT1_CF	blue	na
chrUn	13738929	13739431	503	SAT1_CF	blue	na
chrUn	13739449	13740048	600	SAT1_CF	blue	na
chrUn	13740087	13741536	1450	SAT1_CF	blue	na
chrUn	13741579	13741918	340	SAT1_CF	blue	na
chrUn	13741944	13743281	1338	SAT1_CF	blue	na
chrUn	13743322	13743691	370	SAT1_CF	blue	na
chrUn	13743731	13744232	502	SAT1_CF	blue	na
chrUn	13744249	13746351	2103	SAT1_CF	blue	na
chrUn	13746432	13746995	564	SAT1_CF	blue	na
chrUn	13747045	13747562	518	SAT1_CF	blue	na
chrUn	13747577	13747713	137	SAT1_CF	blue	na
chrUn	13747779	13749623	1845	SAT1_CF	blue	na
chrUn	13749669	13750229	561	SAT1_CF	blue	na
chrUn	13750313	13750860	548	SAT1_CF	blue	na
chrUn	13750888	13751024	137	SAT1_CF	blue	na
chrUn	13751090	13751591	502	SAT1_CF	blue	na
chrUn	13751617	13752378	762	SAT1_CF	blue	na
chrUn	13752420	13754663	2244	SAT1_CF	blue	na
chrUn	13754694	13755202	509	SAT1_CF	blue	na
chrUn	13755215	13755570	356	SAT1_CF	blue	na
chrUn	13755686	13756206	521	SAT1_CF	blue	na
chrUn	13756237	13756763	527	SAT1_CF	blue	na
chrUn	13756807	13758699	1893	SAT1_CF	blue	na
chrUn	13758739	13759240	502	SAT1_CF	blue	na
chrUn	13759267	13760236	970	SAT1_CF	blue	na
chrUn	13760250	13760774	525	SAT1_CF	blue	na
chrUn	13760816	13761892	1077	SAT1_CF	blue	na
chrUn	13761935	13762482	548	SAT1_CF	blue	na
chrUn	13762510	13762646	137	SAT1_CF	blue	na
chrUn	13762711	13763212	502	SAT1_CF	blue	na
chrUn	13763238	13763908	671	SAT1_CF	blue	na
chrUn	13763920	13764593	674	SAT1_CF	blue	na
chrUn	13764653	13765531	879	SAT1_CF	blue	na
chrUn	13765548	13767749	2202	SAT1_CF	blue	na
chrUn	13767762	13768113	352	SAT1_CF	blue	na
chrUn	13768166	13768665	500	SAT1_CF	blue	na
chrUn	13768683	13769748	1066	SAT1_CF	blue	na
chrUn	13769776	13770254	479	SAT1_CF	blue	na
chrUn	13770281	13772817	2537	SAT1_CF	blue	na
chrUn	13772851	13773774	924	SAT1_CF	blue	na
chrUn	13773814	13774315	502	SAT1_CF	blue	na

chrUn	13774329	13775010	682	SAT1_CF	blue	na
chrUn	13775022	13775694	673	SAT1_CF	blue	na
chrUn	13775737	13777731	1995	SAT1_CF	blue	na
chrUn	13777799	13778320	522	SAT1_CF	blue	na
chrUn	13778333	13779014	682	SAT1_CF	blue	na
chrUn	13779026	13783550	4525	SAT1_CF	blue	na
chrUn	13783607	13784081	475	SAT1_CF	blue	na
chrUn	13784108	13784698	591	SAT1_CF	blue	na
chrUn	13784712	13786058	1347	SAT1_CF	blue	na
chrUn	13786178	13787331	1154	SAT1_CF	blue	na
chrUn	13787342	13787410	69	SAT1_CF	blue	na
chrUn	13787479	13789175	1697	SAT1_CF	blue	na
chrUn	13789189	13789864	676	SAT1_CF	blue	na
chrUn	13789943	13790450	508	SAT1_CF	blue	na
chrUn	13790482	13790639	158	SAT1_CF	blue	na
chrUn	13790684	13792005	1322	SAT1_CF	blue	na
chrUn	13792070	13792625	556	SAT1_CF	blue	na
chrUn	13792637	13793314	678	SAT1_CF	blue	na
chrUn	13793327	13793972	646	SAT1_CF	blue	na
chrUn	13794016	13795827	1812	SAT1_CF	blue	na
chrUn	13795840	13795946	107	SAT1_CF	blue	na
chrUn	13795969	13796543	575	SAT1_CF	blue	na
chrUn	13796554	13797202	649	SAT1_CF	blue	na
chrUn	13797214	13797336	123	SAT1_CF	blue	na
chrUn	13797347	13797884	538	SAT1_CF	blue	na
chrUn	13797926	13799148	1223	SAT1_CF	blue	na
chrUn	13799227	13799738	512	SAT1_CF	blue	na
chrUn	13799766	13799922	157	SAT1_CF	blue	na
chrUn	13799967	13800557	591	SAT1_CF	blue	na
chrUn	13800576	13801129	554	SAT1_CF	blue	na
chrUn	13801172	13802133	962	SAT1_CF	blue	na
chrUn	13802160	13803572	1413	SAT1_CF	blue	na
chrUn	13803596	13804914	1319	SAT1_CF	blue	na
chrUn	13805009	13805260	252	SAT1_CF	blue	na
chrUn	13805339	13806297	959	SAT1_CF	blue	na
chrUn	13806357	13806839	483	SAT1_CF	blue	na
chrUn	13806851	13807535	685	SAT1_CF	blue	na
chrUn	13807557	13808399	843	SAT1_CF	blue	na
chrUn	13808449	13808993	545	SAT1_CF	blue	na
chrUn	13809037	13809561	525	SAT1_CF	blue	na
chrUn	13809604	13810157	554	SAT1_CF	blue	na
chrUn	13810169	13810842	674	SAT1_CF	blue	na

chrUn	13810912	13811510	599	SAT1_CF	blue	na
chrUn	13811533	13812045	513	SAT1_CF	blue	na
chrUn	13812132	13812633	502	SAT1_CF	blue	na
chrUn	13812651	13814034	1384	SAT1_CF	blue	na
chrUn	13814046	13814906	861	SAT1_CF	blue	na
chrUn	13814949	13815739	791	SAT1_CF	blue	na
chrUn	13815783	13816324	542	SAT1_CF	blue	na
chrUn	13816349	13817021	673	SAT1_CF	blue	na
chrUn	13817033	13817706	674	SAT1_CF	blue	na
chrUn	13817721	13818365	645	SAT1_CF	blue	na
chrUn	13818409	13818714	306	SAT1_CF	blue	na
chrUn	13818728	13818822	95	SAT1_CF	blue	na
chrUn	13818855	13819529	675	SAT1_CF	blue	na
chrUn	13819555	13820075	521	SAT1_CF	blue	na
chrUn	13820088	13820767	680	SAT1_CF	blue	na
chrUn	13820791	13822217	1427	SAT1_CF	blue	na
chrUn	13822289	13822849	561	SAT1_CF	blue	na
chrUn	13822891	13823443	553	SAT1_CF	blue	na
chrUn	13823456	13824130	675	SAT1_CF	blue	na
chrUn	13824173	13825996	1824	SAT1_CF	blue	na
chrUn	13826035	13826534	500	SAT1_CF	blue	na
chrUn	13826547	13827152	606	SAT1_CF	blue	na
chrUn	13827164	13827843	680	SAT1_CF	blue	na
chrUn	13827855	13829078	1224	SAT1_CF	blue	na
chrUn	13829128	13829447	320	SAT1_CF	blue	na
chrUn	13829458	13830106	649	SAT1_CF	blue	na
chrUn	13830149	13831112	964	SAT1_CF	blue	na
chrUn	13831167	13832139	973	SAT1_CF	blue	na
chrUn	13832246	13832728	483	SAT1_CF	blue	na
chrUn	13832741	13832849	109	SAT1_CF	blue	na
chrUn	13832870	13833619	750	SAT1_CF	blue	na
chrUn	13833641	13834187	547	SAT1_CF	blue	na
chrUn	13834231	13836108	1878	SAT1_CF	blue	na
chrUn	13836284	13836468	185	SAT1_CF	blue	na
chrUn	13836485	13837002	518	SAT1_CF	blue	na
chrUn	13837016	13837551	536	SAT1_CF	blue	na
chrUn	13837563	13838892	1330	SAT1_CF	blue	na
chrUn	13838935	13839903	969	SAT1_CF	blue	na
chrUn	13839946	13840925	980	SAT1_CF	blue	na
chrUn	13840937	13841050	114	SAT1_CF	blue	na
chrUn	13841065	13841612	548	SAT1_CF	blue	na
chrUn	13841655	13843694	2040	SAT1_CF	blue	na

chrUn	13843715	13844235	521	SAT1_CF	blue	na
chrUn	13844248	13844929	682	SAT1_CF	blue	na
chrUn	13844942	13845604	663	SAT1_CF	blue	na
chrUn	13845647	13846278	632	SAT1_CF	blue	na
chrUn	13846306	13846867	562	SAT1_CF	blue	na
chrUn	13846947	13848015	1069	SAT1_CF	blue	na
chrUn	13848027	13849569	1543	SAT1_CF	blue	na
chrUn	13849613	13850006	394	SAT1_CF	blue	na
chrUn	13850020	13850518	499	SAT1_CF	blue	na
chrUn	13850530	13850639	110	SAT1_CF	blue	na
chrUn	13850660	13851367	708	SAT1_CF	blue	na
chrUn	13851379	13852056	678	SAT1_CF	blue	na
chrUn	13852099	13853063	965	SAT1_CF	blue	na
chrUn	13853140	13853685	546	SAT1_CF	blue	na
chrUn	13853698	13854528	831	SAT1_CF	blue	na
chrUn	13854613	13855156	544	SAT1_CF	blue	na
chrUn	13855248	13856393	1146	SAT1_CF	blue	na
chrUn	13856467	13857562	1096	SAT1_CF	blue	na
chrUn	13857576	13858198	623	SAT1_CF	blue	na
chrUn	13858210	13858885	676	SAT1_CF	blue	na
chrUn	13858946	13860317	1372	SAT1_CF	blue	na
chrUn	13860330	13860682	353	SAT1_CF	blue	na
chrUn	13860800	13862392	1593	SAT1_CF	blue	na
chrUn	13862403	13863031	629	SAT1_CF	blue	na
chrUn	13863046	13863151	106	SAT1_CF	blue	na
chrUn	13863230	13863712	483	SAT1_CF	blue	na
chrUn	13863738	13864410	673	SAT1_CF	blue	na
chrUn	13864471	13865336	866	SAT1_CF	blue	na
chrUn	13865356	13865460	105	SAT1_CF	blue	na
chrUn	13865485	13866036	552	SAT1_CF	blue	na
chrUn	13866048	13866725	678	SAT1_CF	blue	na
chrUn	13866772	13868143	1372	SAT1_CF	blue	na
chrUn	13868211	13868733	523	SAT1_CF	blue	na
chrUn	13868776	13870194	1419	SAT1_CF	blue	na
chrUn	13870207	13870314	108	SAT1_CF	blue	na
chrUn	13870336	13870966	631	SAT1_CF	blue	na
chrUn	13870980	13872524	1545	SAT1_CF	blue	na
chrUn	13872709	13873785	1077	SAT1_CF	blue	na
chrUn	13873967	13874148	182	SAT1_CF	blue	na
chrUn	13874188	13874688	501	SAT1_CF	blue	na
chrUn	13874701	13874807	107	SAT1_CF	blue	na
chrUn	13874830	13875841	1012	SAT1_CF	blue	na

chrUn	13875875	13877076	1202	SAT1_CF	blue	na
chrUn	13877124	13877331	208	SAT1_CF	blue	na
chrUn	13877357	13877902	546	SAT1_CF	blue	na
chrUn	13877927	13877978	52	SAT1_CF	blue	na
chrUn	13877995	13878387	393	SAT1_CF	blue	na
chrUn	13878408	13878927	520	SAT1_CF	blue	na
chrUn	13878940	13879041	102	SAT1_CF	blue	na
chrUn	13879069	13879624	556	SAT1_CF	blue	na
chrUn	13879636	13880313	678	SAT1_CF	blue	na
chrUn	13880410	13880952	543	SAT1_CF	blue	na
chrUn	13880977	13881337	361	SAT1_CF	blue	na
chrUn	13881415	13881970	556	SAT1_CF	blue	na
chrUn	13881982	13882667	686	SAT1_CF	blue	na
chrUn	13882680	13884010	1331	SAT1_CF	blue	na
chrUn	13884054	13885261	1208	SAT1_CF	blue	na
chrUn	13885473	13885630	158	SAT1_CF	blue	na
chrUn	13885675	13886174	500	SAT1_CF	blue	na
chrUn	13886187	13886295	109	SAT1_CF	blue	na
chrUn	13886316	13887348	1033	SAT1_CF	blue	na
chrUn	13887388	13888571	1184	SAT1_CF	blue	na
chrUn	13888594	13889921	1328	SAT1_CF	blue	na
chrUn	13889934	13890606	673	SAT1_CF	blue	na
chrUn	13890651	13891282	632	SAT1_CF	blue	na
chrUn	13891310	13894098	2789	SAT1_CF	blue	na
chrUn	13894127	13894802	676	SAT1_CF	blue	na
chrUn	13894870	13895370	501	SAT1_CF	blue	na
chrUn	13895383	13897535	2153	SAT1_CF	blue	na
chrUn	13897566	13898820	1255	SAT1_CF	blue	na
chrUn	13898845	13899170	326	SAT1_CF	blue	na
chrUn	13899250	13900173	924	SAT1_CF	blue	na
chrUn	13900211	13900712	502	SAT1_CF	blue	na
chrUn	13900725	13901586	862	SAT1_CF	blue	na
chrUn	13901663	13902207	545	SAT1_CF	blue	na
chrUn	13902286	13902810	525	SAT1_CF	blue	na
chrUn	13902825	13903525	701	SAT1_CF	blue	na
chrUn	13903538	13904147	610	SAT1_CF	blue	na
chrUn	13904159	13905017	859	SAT1_CF	blue	na
chrUn	13905059	13905757	699	SAT1_CF	blue	na
chrUn	13905771	13906318	548	SAT1_CF	blue	na
chrUn	13906400	13906876	477	SAT1_CF	blue	na
chrUn	13906954	13907455	502	SAT1_CF	blue	na
chrUn	13907468	13907581	114	SAT1_CF	blue	na

chrUn	13907598	13908997	1400	SAT1_CF	blue	na
chrUn	13909040	13910004	965	SAT1_CF	blue	na
chrUn	13910048	13911005	958	SAT1_CF	blue	na
chrUn	13911045	13911539	495	SAT1_CF	blue	na
chrUn	13911553	13911667	115	SAT1_CF	blue	na
chrUn	13911692	13912319	628	SAT1_CF	blue	na
chrUn	13912331	13913168	838	SAT1_CF	blue	na
chrUn	13913349	13913534	186	SAT1_CF	blue	na
chrUn	13913570	13914069	500	SAT1_CF	blue	na
chrUn	13914082	13914765	684	SAT1_CF	blue	na
chrUn	13914777	13915456	680	SAT1_CF	blue	na
chrUn	13915470	13917060	1591	SAT1_CF	blue	na
chrUn	13917077	13917144	68	SAT1_CF	blue	na
chrUn	13917167	13917691	525	SAT1_CF	blue	na
chrUn	13917748	13918294	547	SAT1_CF	blue	na
chrUn	13918337	13918862	526	SAT1_CF	blue	na
chrUn	13918873	13919928	1056	SAT1_CF	blue	na
chrUn	13919971	13920513	543	SAT1_CF	blue	na
chrUn	13920536	13922488	1953	SAT1_CF	blue	na
chrUn	13922515	13924125	1611	SAT1_CF	blue	na
chrUn	13924169	13925139	971	SAT1_CF	blue	na
chrUn	13925182	13925553	372	SAT1_CF	blue	na
chrUn	13925589	13926088	500	SAT1_CF	blue	na
chrUn	13926133	13926785	653	SAT1_CF	blue	na
chrUn	13926797	13928607	1811	SAT1_CF	blue	na
chrUn	13928646	13928811	166	SAT1_CF	blue	na
chrUn	13928995	13929180	186	SAT1_CF	blue	na
chrUn	13929217	13929711	495	SAT1_CF	blue	na
chrUn	13929731	13929839	109	SAT1_CF	blue	na
chrUn	13929859	13930417	559	SAT1_CF	blue	na
chrUn	13930430	13931282	853	SAT1_CF	blue	na
chrUn	13931367	13931911	545	SAT1_CF	blue	na
chrUn	13931953	13932131	179	SAT1_CF	blue	na
chrUn	13932191	13933105	915	SAT1_CF	blue	na
chrUn	13933192	13933693	502	SAT1_CF	blue	na
chrUn	13933706	13934382	677	SAT1_CF	blue	na
chrUn	13934407	13934596	190	SAT1_CF	blue	na
chrUn	13934645	13935239	595	SAT1_CF	blue	na
chrUn	13936641	13937158	518	SAT1_CF	blue	na
chrUn	13937245	13937739	495	SAT1_CF	blue	na
chrUn	13937759	13938441	683	SAT1_CF	blue	na
chrUn	13938464	13939129	666	SAT1_CF	blue	na

chrUn	13939143	13939785	643	SAT1_CF	blue	na
chrUn	13939829	13941034	1206	SAT1_CF	blue	na
chrUn	13941049	13941185	137	SAT1_CF	blue	na
chrUn	13941232	13941751	520	SAT1_CF	blue	na
chrUn	13941765	13942447	683	SAT1_CF	blue	na
chrUn	13942459	13945784	3326	SAT1_CF	blue	na
chrUn	13945826	13945990	165	SAT1_CF	blue	na
chrUn	13946172	13946357	186	SAT1_CF	blue	na
chrUn	13946374	13946894	521	SAT1_CF	blue	na
chrUn	13946907	13947015	109	SAT1_CF	blue	na
chrUn	13947035	13947594	560	SAT1_CF	blue	na
chrUn	13947606	13948273	668	SAT1_CF	blue	na
chrUn	13948317	13950759	2443	SAT1_CF	blue	na
chrUn	13950774	13951439	666	SAT1_CF	blue	na
chrUn	13951453	13952134	682	SAT1_CF	blue	na
chrUn	13952146	13952268	123	SAT1_CF	blue	na
chrUn	13952279	13952827	549	SAT1_CF	blue	na
chrUn	13952838	13953439	602	SAT1_CF	blue	na
chrUn	13953451	13954127	677	SAT1_CF	blue	na
chrUn	13954170	13956855	2686	SAT1_CF	blue	na
chrUn	13956868	13957270	403	SAT1_CF	blue	na
chrUn	13957310	13957812	503	SAT1_CF	blue	na
chrUn	13957825	13959579	1755	SAT1_CF	blue	na
chrUn	13959607	13960131	525	SAT1_CF	blue	na
chrUn	13960144	13960389	246	SAT1_CF	blue	na
chrUn	13960431	13961213	783	SAT1_CF	blue	na
chrUn	13961292	13961801	510	SAT1_CF	blue	na
chrUn	13961814	13962169	356	SAT1_CF	blue	na
chrUn	13962256	13962758	503	SAT1_CF	blue	na
chrUn	13962771	13965457	2687	SAT1_CF	blue	na
chrUn	13965574	13966094	521	SAT1_CF	blue	na
chrUn	13966125	13967735	1611	SAT1_CF	blue	na
chrUn	13967766	13968612	847	SAT1_CF	blue	na
chrUn	13968624	13968733	110	SAT1_CF	blue	na
chrUn	13968754	13969481	728	SAT1_CF	blue	na
chrUn	13969534	13970968	1435	SAT1_CF	blue	na
chrUn	13971012	13971669	658	SAT1_CF	blue	na
chrUn	13971713	13972253	541	SAT1_CF	blue	na
chrUn	13972265	13973529	1265	SAT1_CF	blue	na
chrUn	13973567	13974679	1113	SAT1_CF	blue	na
chrUn	13974699	13975460	762	SAT1_CF	blue	na
chrUn	13975472	13976147	676	SAT1_CF	blue	na

chrUn	13976208	13977401	1194	SAT1_CF	blue	na
chrUn	13977432	13977944	513	SAT1_CF	blue	na
chrUn	13977972	13978108	137	SAT1_CF	blue	na
chrUn	13978193	13978675	483	SAT1_CF	blue	na
chrUn	13978688	13979368	681	SAT1_CF	blue	na
chrUn	13979380	13980237	858	SAT1_CF	blue	na
chrUn	13980279	13980821	543	SAT1_CF	blue	na
chrUn	13980839	13981464	626	SAT1_CF	blue	na
chrUn	13981507	13982089	583	SAT1_CF	blue	na
chrUn	13982157	13982678	522	SAT1_CF	blue	na
chrUn	13982692	13982798	107	SAT1_CF	blue	na
chrUn	13982819	13984244	1426	SAT1_CF	blue	na
chrUn	13984286	13985102	817	SAT1_CF	blue	na
chrUn	13985183	13985731	549	SAT1_CF	blue	na
chrUn	13985745	13986576	832	SAT1_CF	blue	na
chrUn	13986589	13986696	108	SAT1_CF	blue	na
chrUn	13986718	13987292	575	SAT1_CF	blue	na
chrUn	13987304	13987980	677	SAT1_CF	blue	na
chrUn	13988023	13989582	1560	SAT1_CF	blue	na
chrUn	13989596	13990140	545	SAT1_CF	blue	na
chrUn	13990152	13990271	120	SAT1_CF	blue	na
chrUn	13990282	13990826	545	SAT1_CF	blue	na
chrUn	13990875	13990991	117	SAT1_CF	blue	na
chrUn	13991033	13992070	1038	SAT1_CF	blue	na
chrUn	13992127	13992759	633	SAT1_CF	blue	na
chrUn	13992771	13993374	604	SAT1_CF	blue	na
chrUn	13993387	13993493	107	SAT1_CF	blue	na
chrUn	13993514	13994067	554	SAT1_CF	blue	na
chrUn	13994079	13994944	866	SAT1_CF	blue	na
chrUn	13994986	13998022	3037	SAT1_CF	blue	na
chrUn	13998204	13998385	182	SAT1_CF	blue	na
chrUn	13998406	13998925	520	SAT1_CF	blue	na
chrUn	13998938	13999043	106	SAT1_CF	blue	na
chrUn	13999066	14000338	1273	SAT1_CF	blue	na
chrUn	14000353	14000489	137	SAT1_CF	blue	na
chrUn	14000555	14001057	503	SAT1_CF	blue	na
chrUn	14001070	14001176	107	SAT1_CF	blue	na
chrUn	14001198	14003053	1856	SAT1_CF	blue	na
chrUn	14003066	14003468	403	SAT1_CF	blue	na
chrUn	14003508	14004010	503	SAT1_CF	blue	na
chrUn	14004037	14004782	746	SAT1_CF	blue	na
chrUn	14004794	14005592	799	SAT1_CF	blue	na

chrUn	14007121	14007840	720	CarSat1	red	na
chrUn	14007835	14008450	616	SAT1_CF	blue	na
chrUn	14008464	14008967	504	SAT1_CF	blue	na
chrUn	14009007	14010503	1497	SAT1_CF	blue	na
chrUn	14010525	14011730	1206	SAT1_CF	blue	na
chrUn	14011742	14012184	443	SAT1_CF	blue	na
chrUn	14012186	14014156	1971	CarSat1	red	na
chrUn	14014177	14014477	301	CarSat1	red	na
chrUn	14014720	14016001	1282	CarSat1	red	na
chrUn	14016023	14016724	702	CarSat1	red	na
chrUn	14037327	14037489	163	Sat2CF	purple	na
chrUn	14037355	14038055	701	Sat2CF	purple	na
chrUn	14068419	14069656	1238	Sat2CF	purple	na
chrUn	14110066	14111251	1186	Sat2CF	purple	na
chrUn	14135971	14137190	1220	Sat2CF	purple	na
chrUn	14136105	14137185	1081	Sat2CF	purple	na
chrUn	14156850	14157166	317	Sat2CF	purple	na
chrUn	14156852	14157217	366	Sat2CF	purple	na
chrUn	14159680	14159821	142	CarSat1	red	na
chrUn	14159842	14160884	1043	CarSat1	red	na
chrUn	14160900	14161376	477	CarSat1	red	na
chrUn	14170293	14171026	734	CarSat1	red	na
chrUn	14171047	14172642	1596	CarSat1	red	na
chrUn	14172661	14174254	1594	CarSat1	red	na
chrUn	14174274	14175542	1269	CarSat1	red	na
chrUn	14182906	14183425	520	Sat2CF	purple	na
chrUn	14182908	14183531	624	Sat2CF	purple	na
chrUn	14182986	14183458	473	Sat2CF	purple	na
chrUn	14192471	14192802	332	Sat2CF	purple	na
chrUn	14192512	14193027	516	Sat2CF	purple	na
chrUn	14192898	14193047	150	Sat2CF	purple	na
chrUn	14207918	14209053	1136	Sat2CF	purple	na
chrUn	14208876	14209077	202	Sat2CF	purple	na
chrUn	14208946	14209122	177	Sat2CF	purple	na
chrUn	14237162	14237514	353	Sat2CF	purple	na
chrUn	14237221	14238011	791	Sat2CF	purple	na
chrUn	14252845	14253794	950	Sat2CF	purple	na
chrUn	14252908	14253714	807	Sat2CF	purple	na
chrUn	14276219	14276956	738	Sat2CF	purple	na
chrUn	14276293	14276954	662	Sat2CF	purple	na
chrUn	14307927	14308809	883	Sat2CF	purple	na
chrUn	14308016	14309098	1083	Sat2CF	purple	na

chrUn	14308974	14309123	150	Sat2CF	purple	na
chrUn	14324017	14324900	884	Sat2CF	purple	na
chrUn	14324163	14324973	811	Sat2CF	purple	na
chrUn	14353247	14354362	1116	Sat2CF	purple	na
chrUn	14399159	14400059	901	Sat2CF	purple	na
chrUn	14427733	14428840	1108	Sat2CF	purple	na
chrUn	14436844	14438055	1212	Sat2CF	purple	na
chrUn	14453321	14454570	1250	Sat2CF	purple	na
chrUn	14453451	14454537	1087	Sat2CF	purple	na
chrUn	14485290	14486505	1216	Sat2CF	purple	na
chrUn	14485418	14486500	1083	Sat2CF	purple	na
chrUn	14486376	14486525	150	Sat2CF	purple	na
chrUn	14505009	14505293	285	CarSat1	red	na
chrUn	14505521	14506163	643	CarSat1	red	na
chrUn	14506158	14506773	616	SAT1_CF	blue	na
chrUn	14506786	14507288	503	SAT1_CF	blue	na
chrUn	14507328	14510019	2692	SAT1_CF	blue	na
chrUn	14510031	14510502	472	SAT1_CF	blue	na
chrUn	14510504	14512479	1976	CarSat1	red	na
chrUn	14512500	14514088	1589	CarSat1	red	na
chrUn	14514189	14514811	623	CarSat1	red	na
chrUn	14527334	14528141	808	Sat2CF	purple	na
chrUn	14555522	14556678	1157	Sat2CF	purple	na
chrUn	14555659	14556656	998	Sat2CF	purple	na
chrUn	14577177	14577872	696	Sat2CF	purple	na
chrUn	14577765	14577914	150	Sat2CF	purple	na
chrUn	14597669	14598367	699	Sat2CF	purple	na
chrUn	14612862	14614030	1169	Sat2CF	purple	na
chrUn	14613577	14614082	506	Sat2CF	purple	na
chrUn	14628777	14629588	812	Sat2CF	purple	na
chrUn	14629457	14629625	169	Sat2CF	purple	na
chrUn	14641393	14642535	1143	Sat2CF	purple	na
chrUn	14642066	14642597	532	Sat2CF	purple	na
chrUn	14672770	14673946	1177	Sat2CF	purple	na
chrUn	14701778	14702677	900	Sat2CF	purple	na
chrUn	14701841	14702632	792	Sat2CF	purple	na
chrUn	14727495	14728663	1169	Sat2CF	purple	na
chrUn	14728555	14728734	180	Sat2CF	purple	na
chrUn	14743557	14744388	832	Sat2CF	purple	na
chrUn	14743641	14744367	727	Sat2CF	purple	na
chrUn	14744257	14744425	169	Sat2CF	purple	na
chrUn	14754593	14755769	1177	Sat2CF	purple	na

chrUn	14754735	14755740	1006	Sat2CF	purple	na
chrUn	14803091	14804318	1228	Sat2CF	purple	na
chrUn	14822579	14823713	1135	Sat2CF	purple	na
chrUn	14822706	14823644	939	Sat2CF	purple	na
chrUn	14823528	14823674	147	Sat2CF	purple	na
chrUn	14832571	14832908	338	Sat2CF	purple	na
chrUn	14832618	14833148	531	Sat2CF	purple	na
chrUn	14833004	14833180	177	Sat2CF	purple	na
chrUn	14848080	14848889	810	Sat2CF	purple	na
chrUn	14848761	14848928	168	Sat2CF	purple	na
chrUn	14861045	14862122	1078	Sat2CF	purple	na
chrUn	14861175	14862113	939	Sat2CF	purple	na
chrUn	14864781	14866368	1588	CarSat1	red	na
chrUn	14866395	14867879	1485	CarSat1	red	na
chrUn	14876403	14877115	713	CarSat1	red	na
chrUn	14877308	14877941	634	CarSat1	red	na
chrUn	14877957	14878929	973	CarSat1	red	na
chrUn	14878954	14879666	713	CarSat1	red	na
chrUn	14879682	14881140	1459	CarSat1	red	na
chrUn	14881156	14881625	470	CarSat1	red	na
chrUn	14881651	14884719	3069	CarSat1	red	na
chrUn	14884744	14886190	1447	CarSat1	red	na
chrUn	14886215	14886919	705	CarSat1	red	na
chrUn	14886944	14887375	432	CarSat1	red	na
chrUn	14887422	14888105	684	CarSat1	red	na
chrUn	14888188	14888831	644	CarSat1	red	na
chrUn	14888852	14890030	1179	CarSat1	red	na
chrUn	14890060	14890469	410	CarSat1	red	na
chrUn	14897096	14898263	1168	Sat2CF	purple	na
chrUn	14953591	14954533	943	Sat2CF	purple	na
chrUn	14953602	14954689	1088	Sat2CF	purple	na
chrUn	14954559	14954760	202	Sat2CF	purple	na
chrUn	14956442	14956854	413	CarSat1	red	na
chrUn	14957822	14959406	1585	CarSat1	red	na
chrUn	14959427	14960139	713	CarSat1	red	na
chrUn	14960299	14961537	1239	CarSat1	red	na
chrUn	14961575	14962457	883	CarSat1	red	na
chrUn	14962452	14963995	1544	SAT1_CF	blue	na
chrUn	14964037	14966170	2134	SAT1_CF	blue	na
chrUn	14966183	14966726	544	SAT1_CF	blue	na
chrUn	14966758	14967645	888	SAT1_CF	blue	na
chrUn	14973910	14978800	4891	SAT1_CF	blue	na

chrUn	14978799	14979978	1180	CarSat1	red	na
chrUn	14979997	14981511	1515	CarSat1	red	na
chrUn	14981533	14982524	992	CarSat1	red	na
chrUn	14998533	14998928	396	CarSat1	red	na
chrUn	14998990	14999714	725	CarSat1	red	na
chrUn	14999725	15001429	1705	SAT1_CF	blue	na
chrUn	15001472	15003149	1678	SAT1_CF	blue	na
chrUn	15003154	15004087	934	CarSat1	red	na
chrUn	15004100	15005322	1223	CarSat1	red	na
chrUn	15005346	15005876	531	CarSat1	red	na
chrUn	15005976	15007451	1476	CarSat1	red	na
chrUn	15007471	15009072	1602	CarSat1	red	na
chrUn	15009093	15009794	702	CarSat1	red	na
chrUn	15023323	15023485	163	Sat2CF	purple	na
chrUn	15023351	15024064	714	Sat2CF	purple	na
chrUn	15042904	15044080	1177	Sat2CF	purple	na
chrUn	15056093	15056444	352	Sat2CF	purple	na
chrUn	15056158	15056405	248	Sat2CF	purple	na
chrUn	15056162	15056438	277	Sat2CF	purple	na
chrUn	15071812	15073065	1254	Sat2CF	purple	na
chrUn	15071943	15073063	1121	Sat2CF	purple	na
chrUn	15091651	15092732	1082	Sat2CF	purple	na
chrUn	15092603	15092769	167	Sat2CF	purple	na
chrUn	15119915	15121093	1179	Sat2CF	purple	na
chrUn	15136374	15137407	1034	Sat2CF	purple	na
chrUn	15137262	15137442	181	Sat2CF	purple	na
chrUn	15162862	15163786	925	Sat2CF	purple	na
chrUn	15162873	15163670	798	Sat2CF	purple	na
chrUn	15174882	15175819	938	Sat2CF	purple	na
chrUn	15174893	15175940	1048	Sat2CF	purple	na
chrUn	15199286	15200469	1184	Sat2CF	purple	na
chrUn	15200303	15200501	199	Sat2CF	purple	na
chrUn	15230084	15231335	1252	Sat2CF	purple	na
chrUn	15234026	15234391	366	CarSat1	red	na
chrUn	15234440	15235456	1017	CarSat1	red	na
chrUn	15235472	15235945	474	CarSat1	red	na
chrUn	15241182	15242700	1519	CarSat1	red	na
chrUn	15242723	15243803	1081	CarSat1	red	na
chrUn	15247782	15248913	1132	Sat2CF	purple	na
chrUn	15248598	15248965	368	Sat2CF	purple	na
chrUn	15248806	15248985	180	Sat2CF	purple	na
chrUn	15276857	15277752	896	Sat2CF	purple	na

chrUn	15276920	15277707	788	Sat2CF	purple	na
chrUn	15415083	15415245	163	Sat2CF	purple	na
chrUn	15415111	15415449	339	Sat2CF	purple	na
chrUn	15415146	15416035	890	Sat2CF	purple	na
chrUn	15415222	15416001	780	Sat2CF	purple	na
chrUn	15444710	15445097	388	Sat2CF	purple	na
chrUn	15444784	15445538	755	Sat2CF	purple	na
chrUn	15445732	15445966	235	Sat2CF	purple	na
chrUn	15445734	15445933	200	Sat2CF	purple	na
chrUn	15480882	15482049	1168	Sat2CF	purple	na
chrUn	15481941	15482120	180	Sat2CF	purple	na
chrUn	15497138	15497964	827	Sat2CF	purple	na
chrUn	15497222	15497948	727	Sat2CF	purple	na
chrUn	15497838	15498006	169	Sat2CF	purple	na
chrUn	15508434	15509612	1179	Sat2CF	purple	na
chrUn	15549177	15549338	162	Sat2CF	purple	na
chrUn	15549205	15549561	357	Sat2CF	purple	na
chrUn	15549244	15550071	828	Sat2CF	purple	na
chrUn	15565450	15566247	798	Sat2CF	purple	na
chrUn	15566130	15566297	168	Sat2CF	purple	na
chrUn	15566130	15566297	168	Sat2CF	purple	na
chrUn	15576477	15576761	285	CarSat1	red	na
chrUn	15576914	15577633	720	CarSat1	red	na
chrUn	15577628	15578232	605	SAT1_CF	blue	na
chrUn	15578254	15578756	503	SAT1_CF	blue	na
chrUn	15578796	15579757	962	SAT1_CF	blue	na
chrUn	15579835	15580618	784	SAT1_CF	blue	na
chrUn	15580648	15581447	800	SAT1_CF	blue	na
chrUn	15581459	15581842	384	SAT1_CF	blue	na
chrUn	15581854	15583829	1976	CarSat1	red	na
chrUn	15583851	15584552	702	CarSat1	red	na
chrUn	15606737	15607503	767	Sat2CF	purple	na
chrUn	15639061	15640219	1159	Sat2CF	purple	na
chrUn	15639201	15640197	997	Sat2CF	purple	na
chrUn	15660514	15661279	766	Sat2CF	purple	na
chrUn	15680319	15681462	1144	Sat2CF	purple	na
chrUn	15681334	15681482	149	Sat2CF	purple	na
chrUn	15695980	15697149	1170	Sat2CF	purple	na
chrUn	15697041	15697218	178	Sat2CF	purple	na
chrUn	15712267	15713066	800	Sat2CF	purple	na
chrUn	15712948	15713116	169	Sat2CF	purple	na
chrUn	15725132	15726306	1175	Sat2CF	purple	na

chrUn	15800312	15801445	1134	Sat2CF	purple	na
chrUn	15800446	15801530	1085	Sat2CF	purple	na
chrUn	15830973	15831135	163	Sat2CF	purple	na
chrUn	15831001	15831339	339	Sat2CF	purple	na
chrUn	15831040	15831847	808	Sat2CF	purple	na
chrUn	15831248	15831918	671	Sat2CF	purple	na
chrUn	15860659	15861879	1221	Sat2CF	purple	na
chrUn	15861750	15861899	150	Sat2CF	purple	na
chrUn	15877425	15878694	1270	Sat2CF	purple	na
chrUn	15877428	15878562	1135	Sat2CF	purple	na
chrUn	15895606	15896786	1181	Sat2CF	purple	na
chrUn	15896680	15896858	179	Sat2CF	purple	na
chrUn	15911693	15912509	817	Sat2CF	purple	na
chrUn	15911767	15912488	722	Sat2CF	purple	na
chrUn	15912378	15912546	169	Sat2CF	purple	na
chrUn	15924565	15925466	902	Sat2CF	purple	na
chrUn	15968964	15969126	163	Sat2CF	purple	na
chrUn	15968992	15969349	358	Sat2CF	purple	na
chrUn	15969031	15969859	829	Sat2CF	purple	na
chrUn	16106711	16107878	1168	Sat2CF	purple	na
chrUn	16142104	16144373	2270	SAT1_CF	blue	na
chrUn	16144386	16144888	503	SAT1_CF	blue	na
chrUn	16144901	16145592	692	SAT1_CF	blue	na
chrUn	16145672	16146216	545	SAT1_CF	blue	na
chrUn	16146233	16146565	333	SAT1_CF	blue	na
chrUn	16146577	16148266	1690	SAT1_CF	blue	na
chrUn	16148309	16150470	2162	SAT1_CF	blue	na
chrUn	16150496	16150653	158	SAT1_CF	blue	na
chrUn	16150673	16151860	1188	SAT1_CF	blue	na
chrUn	16151922	16152501	580	SAT1_CF	blue	na
chrUn	16152541	16154701	2161	SAT1_CF	blue	na
chrUn	16154730	16155253	524	SAT1_CF	blue	na
chrUn	16155280	16155465	186	SAT1_CF	blue	na
chrUn	16155648	16155811	164	SAT1_CF	blue	na
chrUn	16155890	16157675	1786	SAT1_CF	blue	na
chrUn	16157688	16158520	833	SAT1_CF	blue	na
chrUn	16158537	16159099	563	SAT1_CF	blue	na
chrUn	16159127	16159757	631	SAT1_CF	blue	na
chrUn	16159800	16160346	547	SAT1_CF	blue	na
chrUn	16160366	16160475	110	SAT1_CF	blue	na
chrUn	16160487	16161249	763	SAT1_CF	blue	na
chrUn	16161263	16161813	551	SAT1_CF	blue	na

chrUn	16161835	16161942	108	SAT1_CF	blue	na
chrUn	16161955	16162456	502	SAT1_CF	blue	na
chrUn	16162522	16162658	137	SAT1_CF	blue	na
chrUn	16162673	16163197	525	SAT1_CF	blue	na
chrUn	16163277	16163801	525	SAT1_CF	blue	na
chrUn	16163819	16164435	617	SAT1_CF	blue	na
chrUn	16164484	16165164	681	SAT1_CF	blue	na
chrUn	16165176	16165731	556	SAT1_CF	blue	na
chrUn	16165752	16165859	108	SAT1_CF	blue	na
chrUn	16165872	16166373	502	SAT1_CF	blue	na
chrUn	16166439	16166574	136	SAT1_CF	blue	na
chrUn	16166602	16167165	564	SAT1_CF	blue	na
chrUn	16167209	16168542	1334	SAT1_CF	blue	na
chrUn	16168554	16169235	682	SAT1_CF	blue	na
chrUn	16169247	16169928	682	SAT1_CF	blue	na
chrUn	16169943	16170491	549	SAT1_CF	blue	na
chrUn	16170535	16170892	358	SAT1_CF	blue	na
chrUn	16170917	16172218	1302	SAT1_CF	blue	na
chrUn	16172231	16172752	522	SAT1_CF	blue	na
chrUn	16172786	16173105	320	SAT1_CF	blue	na
chrUn	16173148	16174828	1681	SAT1_CF	blue	na
chrUn	16174870	16175569	700	SAT1_CF	blue	na
chrUn	16175648	16176360	713	SAT1_CF	blue	na
chrUn	16177751	16178337	587	SAT1_CF	blue	na
chrUn	16178352	16180095	1744	SAT1_CF	blue	na
chrUn	16180695	16181377	683	SAT1_CF	blue	na
chrUn	16181389	16183916	2528	SAT1_CF	blue	na
chrUn	16183931	16184043	113	SAT1_CF	blue	na
chrUn	16184066	16184746	681	SAT1_CF	blue	na
chrUn	16184759	16185261	503	SAT1_CF	blue	na
chrUn	16185337	16186018	682	SAT1_CF	blue	na
chrUn	16186031	16186533	503	SAT1_CF	blue	na
chrUn	16186546	16188428	1883	SAT1_CF	blue	na
chrUn	16188470	16188642	173	SAT1_CF	blue	na
chrUn	16188654	16189330	677	SAT1_CF	blue	na
chrUn	16189342	16190019	678	SAT1_CF	blue	na
chrUn	16190044	16190538	495	SAT1_CF	blue	na
chrUn	16190625	16190965	341	SAT1_CF	blue	na
chrUn	16192380	16192567	188	SAT1_CF	blue	na
chrUn	16192579	16193802	1224	SAT1_CF	blue	na
chrUn	16193845	16194520	676	SAT1_CF	blue	na
chrUn	16194532	16195138	607	SAT1_CF	blue	na

chrUn	16195150	16196567	1418	SAT1_CF	blue	na
chrUn	16196594	16197213	620	SAT1_CF	blue	na
chrUn	16197260	16197934	675	SAT1_CF	blue	na
chrUn	16197946	16198620	675	SAT1_CF	blue	na
chrUn	16198642	16199160	519	SAT1_CF	blue	na
chrUn	16199228	16200140	913	SAT1_CF	blue	na
chrUn	16200184	16201000	817	SAT1_CF	blue	na
chrUn	16201042	16201902	861	SAT1_CF	blue	na
chrUn	16201914	16203382	1469	SAT1_CF	blue	na
chrUn	16203452	16204054	603	SAT1_CF	blue	na
chrUn	16204076	16204617	542	SAT1_CF	blue	na
chrUn	16204659	16205521	863	SAT1_CF	blue	na
chrUn	16205533	16206087	555	SAT1_CF	blue	na
chrUn	16206109	16206216	108	SAT1_CF	blue	na
chrUn	16206230	16207413	1184	SAT1_CF	blue	na
chrUn	16207493	16209529	2037	SAT1_CF	blue	na
chrUn	16209572	16210013	442	SAT1_CF	blue	na
chrUn	16210155	16210676	522	SAT1_CF	blue	na
chrUn	16210702	16210859	158	SAT1_CF	blue	na
chrUn	16210874	16211397	524	SAT1_CF	blue	na
chrUn	16211442	16211967	526	SAT1_CF	blue	na
chrUn	16211981	16212700	720	SAT1_CF	blue	na
chrUn	16212743	16213986	1244	SAT1_CF	blue	na
chrUn	16214042	16214117	76	SAT1_CF	blue	na
chrUn	16214129	16214629	501	SAT1_CF	blue	na
chrUn	16214684	16215623	940	SAT1_CF	blue	na
chrUn	16215668	16216027	360	SAT1_CF	blue	na
chrUn	16216050	16216589	540	SAT1_CF	blue	na
chrUn	16216631	16217965	1335	SAT1_CF	blue	na
chrUn	16217978	16218867	890	SAT1_CF	blue	na
chrUn	16218879	16219575	697	SAT1_CF	blue	na
chrUn	16219587	16221096	1510	SAT1_CF	blue	na
chrUn	16221138	16221687	550	SAT1_CF	blue	na
chrUn	16221813	16222380	568	SAT1_CF	blue	na
chrUn	16222392	16222910	519	SAT1_CF	blue	na
chrUn	16222939	16224975	2037	SAT1_CF	blue	na
chrUn	16225018	16225693	676	SAT1_CF	blue	na
chrUn	16225705	16226311	607	SAT1_CF	blue	na
chrUn	16226324	16226845	522	SAT1_CF	blue	na
chrUn	16226860	16228688	1829	SAT1_CF	blue	na
chrUn	16228731	16229277	547	SAT1_CF	blue	na
chrUn	16229306	16229408	103	SAT1_CF	blue	na

chrUn	16229420	16229896	477	SAT1_CF	blue	na
chrUn	16229910	16230026	117	SAT1_CF	blue	na
chrUn	16230038	16230539	502	SAT1_CF	blue	na
chrUn	16230579	16233218	2640	SAT1_CF	blue	na
chrUn	16233246	16235021	1776	SAT1_CF	blue	na
chrUn	16235048	16235149	102	SAT1_CF	blue	na
chrUn	16235163	16235653	491	SAT1_CF	blue	na
chrUn	16235664	16236345	682	SAT1_CF	blue	na
chrUn	16236358	16236860	503	SAT1_CF	blue	na
chrUn	16236947	16237488	542	SAT1_CF	blue	na
chrUn	16237534	16238208	675	SAT1_CF	blue	na
chrUn	16238220	16238902	683	SAT1_CF	blue	na
chrUn	16238915	16239434	520	SAT1_CF	blue	na
chrUn	16239502	16240015	514	SAT1_CF	blue	na
chrUn	16240034	16240556	523	SAT1_CF	blue	na
chrUn	16240640	16241820	1181	SAT1_CF	blue	na
chrUn	16241840	16241949	110	SAT1_CF	blue	na
chrUn	16241961	16242567	607	SAT1_CF	blue	na
chrUn	16242580	16243100	521	SAT1_CF	blue	na
chrUn	16243147	16244972	1826	SAT1_CF	blue	na
chrUn	16244985	16246355	1371	SAT1_CF	blue	na
chrUn	16246368	16247258	891	SAT1_CF	blue	na
chrUn	16247271	16247966	696	SAT1_CF	blue	na
chrUn	16247977	16248582	606	SAT1_CF	blue	na
chrUn	16248602	16249116	515	SAT1_CF	blue	na
chrUn	16249184	16249719	536	SAT1_CF	blue	na
chrUn	16251107	16251647	541	SAT1_CF	blue	na
chrUn	16251709	16252743	1035	SAT1_CF	blue	na
chrUn	16252755	16253453	699	SAT1_CF	blue	na
chrUn	16253504	16254045	542	SAT1_CF	blue	na
chrUn	16254124	16254360	237	SAT1_CF	blue	na
chrUn	16254382	16254921	540	SAT1_CF	blue	na
chrUn	16254963	16255824	862	SAT1_CF	blue	na
chrUn	16255836	16256390	555	SAT1_CF	blue	na
chrUn	16256412	16256518	107	SAT1_CF	blue	na
chrUn	16256531	16257030	500	SAT1_CF	blue	na
chrUn	16257087	16259095	2009	SAT1_CF	blue	na
chrUn	16259160	16260588	1429	SAT1_CF	blue	na
chrUn	16260612	16261804	1193	SAT1_CF	blue	na
chrUn	16261817	16263680	1864	SAT1_CF	blue	na
chrUn	16263692	16264347	656	SAT1_CF	blue	na
chrUn	16264390	16264907	518	SAT1_CF	blue	na

chrUn	16264936	16265789	854	SAT1_CF	blue	na
chrUn	16265802	16266324	523	SAT1_CF	blue	na
chrUn	16266371	16266528	158	SAT1_CF	blue	na
chrUn	16266541	16267061	521	SAT1_CF	blue	na
chrUn	16267079	16267982	904	SAT1_CF	blue	na
chrUn	16268031	16268884	854	SAT1_CF	blue	na
chrUn	16268896	16269477	582	SAT1_CF	blue	na
chrUn	16269557	16270423	867	SAT1_CF	blue	na
chrUn	16270458	16271000	543	SAT1_CF	blue	na
chrUn	16271042	16272732	1691	SAT1_CF	blue	na
chrUn	16272812	16273336	525	SAT1_CF	blue	na
chrUn	16273356	16274224	869	SAT1_CF	blue	na
chrUn	16274252	16274848	597	SAT1_CF	blue	na
chrUn	16274891	16275948	1058	SAT1_CF	blue	na
chrUn	16275967	16277052	1086	SAT1_CF	blue	na
chrUn	16277097	16277254	158	SAT1_CF	blue	na
chrUn	16277269	16277793	525	SAT1_CF	blue	na
chrUn	16277836	16278510	675	SAT1_CF	blue	na
chrUn	16278524	16279207	684	SAT1_CF	blue	na
chrUn	16279219	16279720	502	SAT1_CF	blue	na
chrUn	16279786	16279922	137	SAT1_CF	blue	na
chrUn	16279937	16280977	1041	SAT1_CF	blue	na
chrUn	16280991	16281685	695	SAT1_CF	blue	na
chrUn	16281727	16282983	1257	SAT1_CF	blue	na
chrUn	16282996	16283885	890	SAT1_CF	blue	na
chrUn	16283897	16286193	2297	SAT1_CF	blue	na
chrUn	16286235	16287094	860	SAT1_CF	blue	na
chrUn	16287109	16287624	516	SAT1_CF	blue	na
chrUn	16287653	16289689	2037	SAT1_CF	blue	na
chrUn	16289732	16290406	675	SAT1_CF	blue	na
chrUn	16290418	16291024	607	SAT1_CF	blue	na
chrUn	16291037	16291558	522	SAT1_CF	blue	na
chrUn	16291573	16293401	1829	SAT1_CF	blue	na
chrUn	16293444	16293990	547	SAT1_CF	blue	na
chrUn	16294019	16294121	103	SAT1_CF	blue	na
chrUn	16294133	16294663	531	SAT1_CF	blue	na
chrUn	16294675	16295177	503	SAT1_CF	blue	na
chrUn	16295264	16295805	542	SAT1_CF	blue	na
chrUn	16295851	16296519	669	SAT1_CF	blue	na
chrUn	16296537	16297141	605	SAT1_CF	blue	na
chrUn	16297154	16297674	521	SAT1_CF	blue	na
chrUn	16297721	16299545	1825	SAT1_CF	blue	na

chrUn	16299558	16300927	1370	SAT1_CF	blue	na
chrUn	16300940	16301830	891	SAT1_CF	blue	na
chrUn	16301843	16302538	696	SAT1_CF	blue	na
chrUn	16302549	16303154	606	SAT1_CF	blue	na
chrUn	16303174	16303685	512	SAT1_CF	blue	na
chrUn	16303712	16304641	930	SAT1_CF	blue	na
chrUn	16304654	16305743	1090	SAT1_CF	blue	na
chrUn	16305788	16306458	671	SAT1_CF	blue	na
chrUn	16306470	16308048	1579	SAT1_CF	blue	na
chrUn	16308060	16308718	659	SAT1_CF	blue	na
chrUn	16308798	16310007	1210	SAT1_CF	blue	na
chrUn	16310035	16310635	601	SAT1_CF	blue	na
chrUn	16310661	16311204	544	SAT1_CF	blue	na
chrUn	16311215	16311904	690	SAT1_CF	blue	na
chrUn	16311946	16312488	543	SAT1_CF	blue	na
chrUn	16312565	16312737	173	SAT1_CF	blue	na
chrUn	16312749	16313413	665	SAT1_CF	blue	na
chrUn	16313436	16315472	2037	SAT1_CF	blue	na
chrUn	16315486	16315904	419	SAT1_CF	blue	na
chrUn	16315915	16317345	1431	SAT1_CF	blue	na
chrUn	16317367	16318138	772	SAT1_CF	blue	na
chrUn	16318150	16318651	502	SAT1_CF	blue	na
chrUn	16318699	16319479	781	SAT1_CF	blue	na
chrUn	16319534	16320496	963	SAT1_CF	blue	na
chrUn	16320539	16321085	547	SAT1_CF	blue	na
chrUn	16321105	16321214	110	SAT1_CF	blue	na
chrUn	16321226	16322345	1120	SAT1_CF	blue	na
chrUn	16322395	16322481	87	SAT1_CF	blue	na
chrUn	16322548	16323058	511	SAT1_CF	blue	na
chrUn	16323137	16325564	2428	SAT1_CF	blue	na
chrUn	16325606	16326464	859	SAT1_CF	blue	na
chrUn	16326476	16326981	506	SAT1_CF	blue	na
chrUn	16327026	16327183	158	SAT1_CF	blue	na
chrUn	16327198	16328461	1264	SAT1_CF	blue	na
chrUn	16328531	16329124	594	SAT1_CF	blue	na
chrUn	16329171	16329307	137	SAT1_CF	blue	na
chrUn	16329335	16330518	1184	SAT1_CF	blue	na
chrUn	16330560	16331406	847	SAT1_CF	blue	na
chrUn	16331424	16332105	682	SAT1_CF	blue	na
chrUn	16332118	16332877	760	SAT1_CF	blue	na
chrUn	16332890	16333391	502	SAT1_CF	blue	na
chrUn	16333478	16333848	371	SAT1_CF	blue	na

chrUn	16333870	16334678	809	SAT1_CF	blue	na
chrUn	16334720	16334892	173	SAT1_CF	blue	na
chrUn	16334904	16336276	1373	SAT1_CF	blue	na
chrUn	16336289	16336766	478	SAT1_CF	blue	na
chrUn	16336824	16337009	186	SAT1_CF	blue	na
chrUn	16337190	16337392	203	SAT1_CF	blue	na
chrUn	16337435	16339223	1789	SAT1_CF	blue	na
chrUn	16339235	16339867	633	SAT1_CF	blue	na
chrUn	16339937	16340478	542	SAT1_CF	blue	na
chrUn	16340543	16340808	266	SAT1_CF	blue	na
chrUn	16340888	16341923	1036	SAT1_CF	blue	na
chrUn	16341965	16343022	1058	SAT1_CF	blue	na
chrUn	16343064	16344517	1454	SAT1_CF	blue	na
chrUn	16344543	16344700	158	SAT1_CF	blue	na
chrUn	16344728	16345986	1259	SAT1_CF	blue	na
chrUn	16345999	16346109	111	SAT1_CF	blue	na
chrUn	16346127	16347040	914	SAT1_CF	blue	na
chrUn	16347065	16348397	1333	SAT1_CF	blue	na
chrUn	16348411	16349170	760	SAT1_CF	blue	na
chrUn	16349182	16349863	682	SAT1_CF	blue	na
chrUn	16349876	16350396	521	SAT1_CF	blue	na
chrUn	16350422	16350578	157	SAT1_CF	blue	na
chrUn	16350606	16352947	2342	SAT1_CF	blue	na
chrUn	16352969	16353672	704	SAT1_CF	blue	na
chrUn	16353685	16355974	2290	SAT1_CF	blue	na
chrUn	16356016	16356881	866	SAT1_CF	blue	na
chrUn	16356893	16357560	668	SAT1_CF	blue	na
chrUn	16357588	16358060	473	SAT1_CF	blue	na
chrUn	16358116	16358191	76	SAT1_CF	blue	na
chrUn	16358202	16359460	1259	SAT1_CF	blue	na
chrUn	16359506	16360725	1220	SAT1_CF	blue	na
chrUn	16360737	16361281	545	SAT1_CF	blue	na
chrUn	16361398	16363242	1845	SAT1_CF	blue	na
chrUn	16363255	16364005	751	SAT1_CF	blue	na
chrUn	16364018	16364520	503	SAT1_CF	blue	na
chrUn	16364558	16364962	405	SAT1_CF	blue	na
chrUn	16364975	16366948	1974	SAT1_CF	blue	na
chrUn	16366991	16367537	547	SAT1_CF	blue	na
chrUn	16367580	16368254	675	SAT1_CF	blue	na
chrUn	16368266	16368948	683	SAT1_CF	blue	na
chrUn	16368968	16369481	514	SAT1_CF	blue	na
chrUn	16369502	16369892	391	SAT1_CF	blue	na

chrUn	16369909	16370419	511	SAT1_CF	blue	na
chrUn	16370463	16370765	303	SAT1_CF	blue	na
chrUn	16370813	16372146	1334	SAT1_CF	blue	na
chrUn	16372159	16372660	502	SAT1_CF	blue	na
chrUn	16372746	16372920	175	SAT1_CF	blue	na
chrUn	16373002	16374247	1246	SAT1_CF	blue	na
chrUn	16374259	16374904	646	SAT1_CF	blue	na
chrUn	16374917	16375238	322	SAT1_CF	blue	na
chrUn	16375249	16375844	596	SAT1_CF	blue	na
chrUn	16375856	16376539	684	SAT1_CF	blue	na
chrUn	16376551	16378006	1456	SAT1_CF	blue	na
chrUn	16378020	16379716	1697	SAT1_CF	blue	na
chrUn	16379778	16380335	558	SAT1_CF	blue	na
chrUn	16380395	16381095	701	SAT1_CF	blue	na
chrUn	16381175	16381738	564	SAT1_CF	blue	na
chrUn	16381781	16383264	1484	SAT1_CF	blue	na
chrUn	16383287	16383392	106	SAT1_CF	blue	na
chrUn	16383405	16383907	503	SAT1_CF	blue	na
chrUn	16383920	16384722	803	SAT1_CF	blue	na
chrUn	16384781	16384959	179	SAT1_CF	blue	na
chrUn	16385001	16385545	545	SAT1_CF	blue	na
chrUn	16385588	16386265	678	SAT1_CF	blue	na
chrUn	16386277	16386980	704	SAT1_CF	blue	na
chrUn	16386993	16387493	501	SAT1_CF	blue	na
chrUn	16387530	16387903	374	SAT1_CF	blue	na
chrUn	16387946	16389733	1788	SAT1_CF	blue	na
chrUn	16389746	16391224	1479	SAT1_CF	blue	na
chrUn	16391268	16391626	359	SAT1_CF	blue	na
chrUn	16391651	16392983	1333	SAT1_CF	blue	na
chrUn	16392997	16393626	630	SAT1_CF	blue	na
chrUn	16393647	16393756	110	SAT1_CF	blue	na
chrUn	16393768	16394451	684	SAT1_CF	blue	na
chrUn	16394464	16394983	520	SAT1_CF	blue	na
chrUn	16395051	16396190	1140	SAT1_CF	blue	na
chrUn	16396213	16396318	106	SAT1_CF	blue	na
chrUn	16396331	16396831	501	SAT1_CF	blue	na
chrUn	16396876	16397448	573	SAT1_CF	blue	na
chrUn	16397467	16398012	546	SAT1_CF	blue	na
chrUn	16398026	16398137	112	SAT1_CF	blue	na
chrUn	16398157	16398694	538	SAT1_CF	blue	na
chrUn	16398736	16399802	1067	SAT1_CF	blue	na
chrUn	16399825	16399896	72	SAT1_CF	blue	na

chrUn	16399909	16400964	1056	SAT1_CF	blue	na
chrUn	16400985	16401094	110	SAT1_CF	blue	na
chrUn	16401106	16402571	1466	SAT1_CF	blue	na
chrUn	16402638	16403602	965	SAT1_CF	blue	na
chrUn	16403645	16404191	547	SAT1_CF	blue	na
chrUn	16404213	16404887	675	SAT1_CF	blue	na
chrUn	16404910	16405016	107	SAT1_CF	blue	na
chrUn	16405028	16405311	284	SAT1_CF	blue	na
chrUn	16405324	16406497	1174	SAT1_CF	blue	na
chrUn	16406523	16407182	660	SAT1_CF	blue	na
chrUn	16407263	16408483	1221	SAT1_CF	blue	na
chrUn	16408527	16409899	1373	SAT1_CF	blue	na
chrUn	16409911	16410393	483	SAT1_CF	blue	na
chrUn	16410453	16411447	995	SAT1_CF	blue	na
chrUn	16411527	16412061	535	SAT1_CF	blue	na
chrUn	16412116	16412742	627	SAT1_CF	blue	na
chrUn	16412788	16414156	1369	SAT1_CF	blue	na
chrUn	16414169	16415060	892	SAT1_CF	blue	na
chrUn	16415080	16415574	495	SAT1_CF	blue	na
chrUn	16415612	16416178	567	SAT1_CF	blue	na
chrUn	16416213	16417354	1142	SAT1_CF	blue	na
chrUn	16417429	16417746	318	SAT1_CF	blue	na
chrUn	16417758	16418445	688	SAT1_CF	blue	na
chrUn	16418489	16419470	982	SAT1_CF	blue	na
chrUn	16419512	16420187	676	SAT1_CF	blue	na
chrUn	16420199	16421317	1119	SAT1_CF	blue	na
chrUn	16421357	16421930	574	SAT1_CF	blue	na
chrUn	16423319	16423684	366	SAT1_CF	blue	na
chrUn	16423727	16424884	1158	SAT1_CF	blue	na
chrUn	16424897	16425398	502	SAT1_CF	blue	na
chrUn	16425447	16426179	733	SAT1_CF	blue	na
chrUn	16426258	16427929	1672	SAT1_CF	blue	na
chrUn	16427953	16428043	91	SAT1_CF	blue	na
chrUn	16428070	16428572	503	SAT1_CF	blue	na
chrUn	16428617	16428774	158	SAT1_CF	blue	na
chrUn	16428802	16429961	1160	SAT1_CF	blue	na
chrUn	16429973	16432518	2546	SAT1_CF	blue	na
chrUn	16432545	16433045	501	SAT1_CF	blue	na
chrUn	16433081	16435766	2686	SAT1_CF	blue	na
chrUn	16435778	16436241	464	SAT1_CF	blue	na
chrUn	16436311	16436856	546	SAT1_CF	blue	na
chrUn	16436878	16436984	107	SAT1_CF	blue	na

chrUn	16436997	16438856	1860	SAT1_CF	blue	na
chrUn	16438869	16439422	554	SAT1_CF	blue	na
chrUn	16439443	16439545	103	SAT1_CF	blue	na
chrUn	16439563	16440060	498	SAT1_CF	blue	na
chrUn	16440147	16441479	1333	SAT1_CF	blue	na
chrUn	16441521	16442851	1331	SAT1_CF	blue	na
chrUn	16442863	16443548	686	SAT1_CF	blue	na
chrUn	16443559	16444113	555	SAT1_CF	blue	na
chrUn	16444192	16444552	361	SAT1_CF	blue	na
chrUn	16444577	16445119	543	SAT1_CF	blue	na
chrUn	16445216	16445893	678	SAT1_CF	blue	na
chrUn	16445905	16446460	556	SAT1_CF	blue	na
chrUn	16446481	16446581	101	SAT1_CF	blue	na
chrUn	16446602	16447104	503	SAT1_CF	blue	na
chrUn	16447144	16447536	393	SAT1_CF	blue	na
chrUn	16447553	16448521	969	SAT1_CF	blue	na
chrUn	16448565	16449896	1332	SAT1_CF	blue	na
chrUn	16449908	16450539	632	SAT1_CF	blue	na
chrUn	16450560	16450940	381	SAT1_CF	blue	na
chrUn	16450952	16451711	760	SAT1_CF	blue	na
chrUn	16451724	16452222	499	SAT1_CF	blue	na
chrUn	16452291	16452427	137	SAT1_CF	blue	na
chrUn	16452442	16452966	525	SAT1_CF	blue	na
chrUn	16452997	16453187	191	SAT1_CF	blue	na
chrUn	16453248	16454470	1223	SAT1_CF	blue	na
chrUn	16454482	16455024	543	SAT1_CF	blue	na
chrUn	16455049	16456764	1716	SAT1_CF	blue	na
chrUn	16456806	16457966	1161	SAT1_CF	blue	na
chrUn	16457979	16459181	1203	SAT1_CF	blue	na
chrUn	16459221	16459920	700	SAT1_CF	blue	na
chrUn	16459963	16460527	565	SAT1_CF	blue	na
chrUn	16460571	16461827	1257	SAT1_CF	blue	na
chrUn	16461839	16462391	553	SAT1_CF	blue	na
chrUn	16462413	16462485	73	SAT1_CF	blue	na
chrUn	16462498	16463424	927	SAT1_CF	blue	na
chrUn	16463436	16464117	682	SAT1_CF	blue	na
chrUn	16464129	16464812	684	SAT1_CF	blue	na
chrUn	16464824	16465584	761	SAT1_CF	blue	na
chrUn	16465597	16465772	176	SAT1_CF	blue	na
chrUn	16465987	16466526	540	SAT1_CF	blue	na
chrUn	16466538	16467221	684	SAT1_CF	blue	na
chrUn	16467233	16467993	761	SAT1_CF	blue	na

chrUn	16468006	16469700	1695	SAT1_CF	blue	na
chrUn	16469723	16469829	107	SAT1_CF	blue	na
chrUn	16469843	16470323	481	SAT1_CF	blue	na
chrUn	16470379	16470564	186	SAT1_CF	blue	na
chrUn	16470744	16470946	203	SAT1_CF	blue	na
chrUn	16470989	16471598	610	SAT1_CF	blue	na
chrUn	16471631	16472767	1137	SAT1_CF	blue	na
chrUn	16472789	16473421	633	SAT1_CF	blue	na
chrUn	16473492	16474035	544	SAT1_CF	blue	na
chrUn	16474054	16474367	314	SAT1_CF	blue	na
chrUn	16474447	16475011	565	SAT1_CF	blue	na
chrUn	16475054	16476261	1208	SAT1_CF	blue	na
chrUn	16476305	16476841	537	SAT1_CF	blue	na
chrUn	16476878	16478621	1744	SAT1_CF	blue	na
chrUn	16478664	16479341	678	SAT1_CF	blue	na
chrUn	16479353	16480056	704	SAT1_CF	blue	na
chrUn	16480069	16480590	522	SAT1_CF	blue	na
chrUn	16480658	16481013	356	SAT1_CF	blue	na
chrUn	16481026	16482809	1784	SAT1_CF	blue	na
chrUn	16482826	16484018	1193	SAT1_CF	blue	na
chrUn	16484071	16484604	534	SAT1_CF	blue	na
chrUn	16484618	16485169	552	SAT1_CF	blue	na
chrUn	16485212	16486894	1683	SAT1_CF	blue	na
chrUn	16486906	16488690	1785	SAT1_CF	blue	na
chrUn	16488703	16489205	503	SAT1_CF	blue	na
chrUn	16489245	16489647	403	SAT1_CF	blue	na
chrUn	16489660	16491469	1810	SAT1_CF	blue	na
chrUn	16491489	16492958	1470	SAT1_CF	blue	na
chrUn	16492990	16493991	1002	SAT1_CF	blue	na
chrUn	16494034	16494710	677	SAT1_CF	blue	na
chrUn	16494722	16494945	224	SAT1_CF	blue	na
chrUn	16495129	16496650	1522	SAT1_CF	blue	na
chrUn	16496693	16497372	680	SAT1_CF	blue	na
chrUn	16497384	16497938	555	SAT1_CF	blue	na
chrUn	16497993	16498624	632	SAT1_CF	blue	na
chrUn	16498692	16499654	963	SAT1_CF	blue	na
chrUn	16499768	16500548	781	SAT1_CF	blue	na
chrUn	16500590	16501450	861	SAT1_CF	blue	na
chrUn	16501463	16502020	558	SAT1_CF	blue	na
chrUn	16502040	16502148	109	SAT1_CF	blue	na
chrUn	16502161	16502681	521	SAT1_CF	blue	na
chrUn	16502698	16502883	186	SAT1_CF	blue	na

chrUn	16503065	16503230	166	SAT1_CF	blue	na
chrUn	16503272	16503819	548	SAT1_CF	blue	na
chrUn	16503899	16504671	773	SAT1_CF	blue	na
chrUn	16504751	16505275	525	SAT1_CF	blue	na
chrUn	16505322	16505789	468	SAT1_CF	blue	na
chrUn	16505805	16506344	540	SAT1_CF	blue	na
chrUn	16506424	16507389	966	SAT1_CF	blue	na
chrUn	16507432	16508094	663	SAT1_CF	blue	na
chrUn	16508107	16508300	194	SAT1_CF	blue	na
chrUn	16508331	16509003	673	SAT1_CF	blue	na
chrUn	16509015	16510590	1576	SAT1_CF	blue	na
chrUn	16510651	16511327	677	SAT1_CF	blue	na
chrUn	16511339	16512023	685	SAT1_CF	blue	na
chrUn	16512036	16512538	503	SAT1_CF	blue	na
chrUn	16512624	16512979	356	SAT1_CF	blue	na
chrUn	16512990	16513538	549	SAT1_CF	blue	na
chrUn	16513615	16514374	760	SAT1_CF	blue	na
chrUn	16514426	16514614	189	SAT1_CF	blue	na
chrUn	16514627	16515148	522	SAT1_CF	blue	na
chrUn	16515215	16516174	960	SAT1_CF	blue	na
chrUn	16516217	16516894	678	SAT1_CF	blue	na
chrUn	16516906	16517588	683	SAT1_CF	blue	na
chrUn	16517601	16518301	701	SAT1_CF	blue	na
chrUn	16518329	16518839	511	SAT1_CF	blue	na
chrUn	16518908	16519631	724	SAT1_CF	blue	na
chrUn	16519643	16520333	691	SAT1_CF	blue	na
chrUn	16520376	16520897	522	SAT1_CF	blue	na
chrUn	16520931	16522005	1075	SAT1_CF	blue	na
chrUn	16522048	16522592	545	SAT1_CF	blue	na
chrUn	16522613	16522721	109	SAT1_CF	blue	na
chrUn	16522734	16523253	520	SAT1_CF	blue	na
chrUn	16523291	16523653	363	SAT1_CF	blue	na
chrUn	16523690	16524294	605	SAT1_CF	blue	na
chrUn	16524316	16525994	1679	SAT1_CF	blue	na
chrUn	16526007	16526528	522	SAT1_CF	blue	na
chrUn	16526545	16527225	681	SAT1_CF	blue	na
chrUn	16527267	16528125	859	SAT1_CF	blue	na
chrUn	16528345	16528877	533	SAT1_CF	blue	na
chrUn	16528904	16530091	1188	SAT1_CF	blue	na
chrUn	16530103	16530657	555	SAT1_CF	blue	na
chrUn	16530682	16530786	105	SAT1_CF	blue	na
chrUn	16530799	16531672	874	SAT1_CF	blue	na

chrUn	16531733	16532409	677	SAT1_CF	blue	na
chrUn	16532421	16532994	574	SAT1_CF	blue	na
chrUn	16533019	16533339	321	SAT1_CF	blue	na
chrUn	16533383	16534094	712	SAT1_CF	blue	na
chrUn	16534105	16534598	494	SAT1_CF	blue	na
chrUn	16534611	16535871	1261	SAT1_CF	blue	na
chrUn	16535948	16536786	839	SAT1_CF	blue	na
chrUn	16536821	16537301	481	SAT1_CF	blue	na
chrUn	16537327	16537922	596	SAT1_CF	blue	na
chrUn	16538190	16539383	1194	SAT1_CF	blue	na
chrUn	16539395	16540310	916	SAT1_CF	blue	na
chrUn	16540323	16541004	682	SAT1_CF	blue	na
chrUn	16541024	16541537	514	SAT1_CF	blue	na
chrUn	16541554	16542451	898	SAT1_CF	blue	na
chrUn	16542472	16542935	464	SAT1_CF	blue	na
chrUn	16542978	16544194	1217	SAT1_CF	blue	na
chrUn	16544206	16544903	698	SAT1_CF	blue	na
chrUn	16544921	16545423	503	SAT1_CF	blue	na
chrUn	16545463	16545864	402	SAT1_CF	blue	na
chrUn	16546934	16548045	1112	SAT1_CF	blue	na
chrUn	16548059	16548947	889	SAT1_CF	blue	na
chrUn	16548959	16549636	678	SAT1_CF	blue	na
chrUn	16549661	16550155	495	SAT1_CF	blue	na
chrUn	16550193	16550759	567	SAT1_CF	blue	na
chrUn	16550829	16551346	518	SAT1_CF	blue	na
chrUn	16551365	16551935	571	SAT1_CF	blue	na
chrUn	16552010	16552327	318	SAT1_CF	blue	na
chrUn	16552407	16553024	618	SAT1_CF	blue	na
chrUn	16553035	16554015	981	SAT1_CF	blue	na
chrUn	16554057	16554732	676	SAT1_CF	blue	na
chrUn	16554744	16555863	1120	SAT1_CF	blue	na
chrUn	16555903	16556476	574	SAT1_CF	blue	na
chrUn	16557872	16559419	1548	SAT1_CF	blue	na
chrUn	16559432	16559953	522	SAT1_CF	blue	na
chrUn	16559979	16560087	109	SAT1_CF	blue	na
chrUn	16560098	16561752	1655	SAT1_CF	blue	na
chrUn	16561797	16561954	158	SAT1_CF	blue	na
chrUn	16561969	16562492	524	SAT1_CF	blue	na
chrUn	16562572	16564020	1449	SAT1_CF	blue	na
chrUn	16564040	16565169	1130	SAT1_CF	blue	na
chrUn	16565190	16566123	934	SAT1_CF	blue	na
chrUn	16566157	16566677	521	SAT1_CF	blue	na

chrUn	16566691	16567231	541	SAT1_CF	blue	na
chrUn	16567274	16567818	545	SAT1_CF	blue	na
chrUn	16567837	16567946	110	SAT1_CF	blue	na
chrUn	16567958	16568717	760	SAT1_CF	blue	na
chrUn	16568730	16569230	501	SAT1_CF	blue	na
chrUn	16569317	16569875	559	SAT1_CF	blue	na
chrUn	16569905	16570531	627	SAT1_CF	blue	na
chrUn	16570579	16571946	1368	SAT1_CF	blue	na
chrUn	16571959	16572460	502	SAT1_CF	blue	na
chrUn	16572498	16572902	405	SAT1_CF	blue	na
chrUn	16572915	16574693	1779	SAT1_CF	blue	na
chrUn	16574744	16575218	475	SAT1_CF	blue	na
chrUn	16575280	16575678	399	SAT1_CF	blue	na
chrUn	16575690	16576236	547	SAT1_CF	blue	na
chrUn	16576350	16577314	965	SAT1_CF	blue	na
chrUn	16577358	16578033	676	SAT1_CF	blue	na
chrUn	16578046	16578604	559	SAT1_CF	blue	na
chrUn	16578624	16578732	109	SAT1_CF	blue	na
chrUn	16578745	16579265	521	SAT1_CF	blue	na
chrUn	16579282	16579467	186	SAT1_CF	blue	na
chrUn	16579649	16579814	166	SAT1_CF	blue	na
chrUn	16579856	16581254	1399	SAT1_CF	blue	na
chrUn	16581334	16583620	2287	SAT1_CF	blue	na
chrUn	16583687	16584466	780	SAT1_CF	blue	na
chrUn	16584524	16586593	2070	SAT1_CF	blue	na
chrUn	16586658	16587631	974	SAT1_CF	blue	na
chrUn	16587648	16588799	1152	SAT1_CF	blue	na
chrUn	16588812	16590076	1265	SAT1_CF	blue	na
chrUn	16590102	16590259	158	SAT1_CF	blue	na
chrUn	16590274	16590798	525	SAT1_CF	blue	na
chrUn	16590877	16591692	816	SAT1_CF	blue	na
chrUn	16591723	16593181	1459	SAT1_CF	blue	na
chrUn	16593193	16593876	684	SAT1_CF	blue	na
chrUn	16593890	16594390	501	SAT1_CF	blue	na
chrUn	16594458	16594594	137	SAT1_CF	blue	na
chrUn	16594627	16595133	507	SAT1_CF	blue	na
chrUn	16595162	16599897	4736	SAT1_CF	blue	na
chrUn	16599923	16600080	158	SAT1_CF	blue	na
chrUn	16600095	16601420	1326	SAT1_CF	blue	na
chrUn	16601443	16601914	472	SAT1_CF	blue	na
chrUn	16601975	16602155	181	SAT1_CF	blue	na
chrUn	16602335	16602537	203	SAT1_CF	blue	na

chrUn	16602580	16603127	548	SAT1_CF	blue	na
chrUn	16603171	16604139	969	SAT1_CF	blue	na
chrUn	16604182	16604648	467	SAT1_CF	blue	na
chrUn	16604836	16605774	939	SAT1_CF	blue	na
chrUn	16605787	16606288	502	SAT1_CF	blue	na
chrUn	16606331	16607283	953	SAT1_CF	blue	na
chrUn	16607338	16608959	1622	SAT1_CF	blue	na
chrUn	16609065	16609418	354	SAT1_CF	blue	na
chrUn	16609433	16610397	965	SAT1_CF	blue	na
chrUn	16610440	16611764	1325	SAT1_CF	blue	na
chrUn	16611784	16612428	645	SAT1_CF	blue	na
chrUn	16612441	16613188	748	SAT1_CF	blue	na
chrUn	16613215	16613716	502	SAT1_CF	blue	na
chrUn	16613782	16613918	137	SAT1_CF	blue	na
chrUn	16613933	16614457	525	SAT1_CF	blue	na
chrUn	16614500	16615025	526	SAT1_CF	blue	na
chrUn	16615069	16615589	521	SAT1_CF	blue	na
chrUn	16615605	16615740	136	SAT1_CF	blue	na
chrUn	16615754	16616747	994	SAT1_CF	blue	na
chrUn	16616778	16617330	553	SAT1_CF	blue	na
chrUn	16617417	16617772	356	SAT1_CF	blue	na
chrUn	16617785	16618294	510	SAT1_CF	blue	na
chrUn	16618373	16619412	1040	SAT1_CF	blue	na
chrUn	16619456	16621758	2303	SAT1_CF	blue	na
chrUn	16621771	16622292	522	SAT1_CF	blue	na
chrUn	16622359	16622493	135	SAT1_CF	blue	na
chrUn	16622675	16622911	237	SAT1_CF	blue	na
chrUn	16622922	16625841	2920	SAT1_CF	blue	na
chrUn	16625862	16626820	959	SAT1_CF	blue	na
chrUn	16626864	16627193	330	SAT1_CF	blue	na
chrUn	16627217	16627862	646	SAT1_CF	blue	na
chrUn	16627874	16629243	1370	SAT1_CF	blue	na
chrUn	16629256	16629755	500	SAT1_CF	blue	na
chrUn	16629844	16630359	516	SAT1_CF	blue	na
chrUn	16630393	16631824	1432	SAT1_CF	blue	na
chrUn	16631851	16632331	481	SAT1_CF	blue	na
chrUn	16632387	16632757	371	SAT1_CF	blue	na
chrUn	16632802	16633772	971	SAT1_CF	blue	na
chrUn	16633816	16635014	1199	SAT1_CF	blue	na
chrUn	16635030	16635427	398	SAT1_CF	blue	na
chrUn	16635441	16636070	630	SAT1_CF	blue	na
chrUn	16636091	16636817	727	SAT1_CF	blue	na

chrUn	16636830	16637350	521	SAT1_CF	blue	na
chrUn	16637418	16639993	2576	SAT1_CF	blue	na
chrUn	16640013	16640508	496	SAT1_CF	blue	na
chrUn	16640594	16640768	175	SAT1_CF	blue	na
chrUn	16640849	16642094	1246	SAT1_CF	blue	na
chrUn	16642106	16642658	553	SAT1_CF	blue	na
chrUn	16642679	16643692	1014	SAT1_CF	blue	na
chrUn	16643704	16644375	672	SAT1_CF	blue	na
chrUn	16644398	16644899	502	SAT1_CF	blue	na
chrUn	16644965	16645101	137	SAT1_CF	blue	na
chrUn	16645129	16645677	549	SAT1_CF	blue	na
chrUn	16645761	16646830	1070	SAT1_CF	blue	na
chrUn	16646872	16647729	858	SAT1_CF	blue	na
chrUn	16647741	16648423	683	SAT1_CF	blue	na
chrUn	16648436	16648934	499	SAT1_CF	blue	na
chrUn	16648982	16649378	397	SAT1_CF	blue	na
chrUn	16649391	16649924	534	SAT1_CF	blue	na
chrUn	16650014	16650978	965	SAT1_CF	blue	na
chrUn	16651021	16651697	677	SAT1_CF	blue	na
chrUn	16651709	16652950	1242	SAT1_CF	blue	na
chrUn	16652982	16653600	619	SAT1_CF	blue	na
chrUn	16653687	16654040	354	SAT1_CF	blue	na
chrUn	16654055	16655382	1328	SAT1_CF	blue	na
chrUn	16655394	16656229	836	SAT1_CF	blue	na
chrUn	16656247	16656747	501	SAT1_CF	blue	na
chrUn	16656800	16657151	352	SAT1_CF	blue	na
chrUn	16657164	16657684	521	SAT1_CF	blue	na
chrUn	16657715	16658105	391	SAT1_CF	blue	na
chrUn	16658175	16659399	1225	SAT1_CF	blue	na
chrUn	16659416	16660002	587	SAT1_CF	blue	na
chrUn	16660167	16660279	113	SAT1_CF	blue	na
chrUn	16660292	16660793	502	SAT1_CF	blue	na
chrUn	16660833	16661202	370	SAT1_CF	blue	na
chrUn	16661245	16662895	1651	SAT1_CF	blue	na
chrUn	16662915	16664348	1434	SAT1_CF	blue	na
chrUn	16664375	16664877	503	SAT1_CF	blue	na
chrUn	16664963	16665850	888	SAT1_CF	blue	na
chrUn	16665870	16666937	1068	SAT1_CF	blue	na
chrUn	16666950	16667959	1010	SAT1_CF	blue	na
chrUn	16667971	16668602	632	SAT1_CF	blue	na
chrUn	16668625	16668730	106	SAT1_CF	blue	na
chrUn	16668743	16670439	1697	SAT1_CF	blue	na

chrUn	16670462	16670567	106	SAT1_CF	blue	na
chrUn	16670580	16671100	521	SAT1_CF	blue	na
chrUn	16671117	16671302	186	SAT1_CF	blue	na
chrUn	16671483	16671685	203	SAT1_CF	blue	na
chrUn	16671728	16674351	2624	SAT1_CF	blue	na
chrUn	16674395	16675636	1242	SAT1_CF	blue	na
chrUn	16675715	16676677	963	SAT1_CF	blue	na
chrUn	16676720	16677399	680	SAT1_CF	blue	na
chrUn	16677411	16678084	674	SAT1_CF	blue	na
chrUn	16678105	16678606	502	SAT1_CF	blue	na
chrUn	16678651	16678808	158	SAT1_CF	blue	na
chrUn	16678873	16679422	550	SAT1_CF	blue	na
chrUn	16679482	16679660	179	SAT1_CF	blue	na
chrUn	16679702	16680246	545	SAT1_CF	blue	na
chrUn	16680289	16680965	677	SAT1_CF	blue	na
chrUn	16680977	16681680	704	SAT1_CF	blue	na
chrUn	16681693	16682192	500	SAT1_CF	blue	na
chrUn	16682234	16684431	2198	SAT1_CF	blue	na
chrUn	16684444	16685468	1025	SAT1_CF	blue	na
chrUn	16685513	16686327	815	SAT1_CF	blue	na
chrUn	16686369	16687102	734	SAT1_CF	blue	na
chrUn	16687123	16687230	108	SAT1_CF	blue	na
chrUn	16687242	16687924	683	SAT1_CF	blue	na
chrUn	16687937	16688619	683	SAT1_CF	blue	na
chrUn	16688639	16689132	494	SAT1_CF	blue	na
chrUn	16689174	16689576	403	SAT1_CF	blue	na
chrUn	16689587	16690197	611	SAT1_CF	blue	na
chrUn	16690217	16691221	1005	SAT1_CF	blue	na
chrUn	16691264	16691939	676	SAT1_CF	blue	na
chrUn	16691951	16692554	604	SAT1_CF	blue	na
chrUn	16692567	16693068	502	SAT1_CF	blue	na
chrUn	16693108	16693510	403	SAT1_CF	blue	na
chrUn	16693523	16694706	1184	SAT1_CF	blue	na
chrUn	16694772	16696209	1438	SAT1_CF	blue	na
chrUn	16696253	16696800	548	SAT1_CF	blue	na
chrUn	16696821	16696930	110	SAT1_CF	blue	na
chrUn	16696942	16697982	1041	SAT1_CF	blue	na
chrUn	16697994	16698754	761	SAT1_CF	blue	na
chrUn	16698768	16699269	502	SAT1_CF	blue	na
chrUn	16699335	16699471	137	SAT1_CF	blue	na
chrUn	16699486	16700009	524	SAT1_CF	blue	na
chrUn	16700051	16701640	1590	SAT1_CF	blue	na

chrUn	16701683	16702870	1188	SAT1_CF	blue	na
chrUn	16702910	16704767	1858	SAT1_CF	blue	na
chrUn	16704809	16705516	708	SAT1_CF	blue	na
chrUn	16705529	16706283	755	SAT1_CF	blue	na
chrUn	16706296	16707993	1698	SAT1_CF	blue	na
chrUn	16708055	16708615	561	SAT1_CF	blue	na
chrUn	16708648	16708858	211	SAT1_CF	blue	na
chrUn	16709037	16709239	203	SAT1_CF	blue	na
chrUn	16709282	16710940	1659	SAT1_CF	blue	na
chrUn	16710960	16711069	110	SAT1_CF	blue	na
chrUn	16711081	16711634	554	SAT1_CF	blue	na
chrUn	16711689	16712277	589	SAT1_CF	blue	na
chrUn	16712290	16713239	950	SAT1_CF	blue	na
chrUn	16713372	16713916	545	SAT1_CF	blue	na
chrUn	16713959	16715411	1453	SAT1_CF	blue	na
chrUn	16715422	16716104	683	SAT1_CF	blue	na
chrUn	16716117	16716618	502	SAT1_CF	blue	na
chrUn	16716663	16716820	158	SAT1_CF	blue	na
chrUn	16716848	16717360	513	SAT1_CF	blue	na
chrUn	16717391	16717911	521	SAT1_CF	blue	na
chrUn	16718027	16718382	356	SAT1_CF	blue	na
chrUn	16718396	16720744	2349	SAT1_CF	blue	na
chrUn	16720756	16721436	681	SAT1_CF	blue	na
chrUn	16721449	16722052	604	SAT1_CF	blue	na
chrUn	16722064	16722696	633	SAT1_CF	blue	na
chrUn	16722753	16723791	1039	SAT1_CF	blue	na
chrUn	16723833	16723949	117	SAT1_CF	blue	na
chrUn	16723998	16724672	675	SAT1_CF	blue	na
chrUn	16724684	16725230	547	SAT1_CF	blue	na
chrUn	16725259	16725499	241	SAT1_CF	blue	na
chrUn	16725527	16726039	513	SAT1_CF	blue	na
chrUn	16726082	16727158	1077	SAT1_CF	blue	na
chrUn	16727201	16728061	861	SAT1_CF	blue	na
chrUn	16728073	16728632	560	SAT1_CF	blue	na
chrUn	16728652	16728760	109	SAT1_CF	blue	na
chrUn	16728773	16729293	521	SAT1_CF	blue	na
chrUn	16729310	16729495	186	SAT1_CF	blue	na
chrUn	16729677	16729842	166	SAT1_CF	blue	na
chrUn	16729884	16732454	2571	SAT1_CF	blue	na
chrUn	16732498	16733172	675	SAT1_CF	blue	na
chrUn	16733184	16734710	1527	SAT1_CF	blue	na
chrUn	16734753	16736125	1373	SAT1_CF	blue	na

chrUn	16736138	16736639	502	SAT1_CF	blue	na
chrUn	16736682	16737679	998	SAT1_CF	blue	na
chrUn	16737711	16739310	1600	SAT1_CF	blue	na
chrUn	16739374	16739769	396	SAT1_CF	blue	na
chrUn	16739784	16740302	519	SAT1_CF	blue	na
chrUn	16740315	16741406	1092	SAT1_CF	blue	na
chrUn	16741449	16742122	674	SAT1_CF	blue	na
chrUn	16742134	16742818	685	SAT1_CF	blue	na
chrUn	16742830	16744157	1328	SAT1_CF	blue	na
chrUn	16744236	16744596	361	SAT1_CF	blue	na
chrUn	16744621	16745163	543	SAT1_CF	blue	na
chrUn	16745260	16745936	677	SAT1_CF	blue	na
chrUn	16745948	16746503	556	SAT1_CF	blue	na
chrUn	16746526	16746632	107	SAT1_CF	blue	na
chrUn	16746645	16747146	502	SAT1_CF	blue	na
chrUn	16747186	16748082	897	SAT1_CF	blue	na
chrUn	16748114	16748449	336	SAT1_CF	blue	na
chrUn	16748497	16749142	646	SAT1_CF	blue	na
chrUn	16749154	16749829	676	SAT1_CF	blue	na
chrUn	16749842	16750362	521	SAT1_CF	blue	na
chrUn	16750388	16750495	108	SAT1_CF	blue	na
chrUn	16750558	16751084	527	SAT1_CF	blue	na
chrUn	16751114	16751930	817	SAT1_CF	blue	na
chrUn	16751942	16752494	553	SAT1_CF	blue	na
chrUn	16752516	16752588	73	SAT1_CF	blue	na
chrUn	16752601	16753529	929	SAT1_CF	blue	na
chrUn	16753541	16754222	682	SAT1_CF	blue	na
chrUn	16754234	16754802	569	SAT1_CF	blue	na
chrUn	16754845	16756540	1696	SAT1_CF	blue	na
chrUn	16756563	16756668	106	SAT1_CF	blue	na
chrUn	16756681	16757161	481	SAT1_CF	blue	na
chrUn	16757194	16757402	209	SAT1_CF	blue	na
chrUn	16757583	16757747	165	SAT1_CF	blue	na
chrUn	16757826	16758371	546	SAT1_CF	blue	na
chrUn	16758413	16759273	861	SAT1_CF	blue	na
chrUn	16759287	16759968	682	SAT1_CF	blue	na
chrUn	16759980	16761192	1213	SAT1_CF	blue	na
chrUn	16761239	16761375	137	SAT1_CF	blue	na
chrUn	16761403	16762595	1193	SAT1_CF	blue	na
chrUn	16762622	16763253	632	SAT1_CF	blue	na
chrUn	16763296	16763960	665	SAT1_CF	blue	na
chrUn	16763983	16764665	683	SAT1_CF	blue	na

chrUn	16764685	16765158	474	SAT1_CF	blue	na
chrUn	16765218	16765791	574	SAT1_CF	blue	na
chrUn	16767190	16768479	1290	SAT1_CF	blue	na
chrUn	16768521	16768832	312	SAT1_CF	blue	na
chrUn	16768874	16769394	521	SAT1_CF	blue	na
chrUn	16769428	16769793	366	SAT1_CF	blue	na
chrUn	16769825	16771449	1625	SAT1_CF	blue	na
chrUn	16771492	16772164	673	SAT1_CF	blue	na
chrUn	16772177	16772740	564	SAT1_CF	blue	na
chrUn	16772779	16773497	719	SAT1_CF	blue	na
chrUn	16773540	16774206	667	SAT1_CF	blue	na
chrUn	16774218	16774692	475	SAT1_CF	blue	na
chrUn	16774762	16775435	674	SAT1_CF	blue	na
chrUn	16775447	16778046	2600	SAT1_CF	blue	na
chrUn	16778059	16779430	1372	SAT1_CF	blue	na
chrUn	16779468	16779870	403	SAT1_CF	blue	na
chrUn	16779881	16780430	550	SAT1_CF	blue	na
chrUn	16780474	16782279	1806	SAT1_CF	blue	na
chrUn	16782293	16782883	591	SAT1_CF	blue	na
chrUn	16782910	16783969	1060	SAT1_CF	blue	na
chrUn	16784013	16784557	545	SAT1_CF	blue	na
chrUn	16784599	16785985	1387	SAT1_CF	blue	na
chrUn	16785998	16786496	499	SAT1_CF	blue	na
chrUn	16786544	16786940	397	SAT1_CF	blue	na
chrUn	16786972	16787388	417	SAT1_CF	blue	na
chrUn	16787430	16788466	1037	SAT1_CF	blue	na
chrUn	16788523	16789155	633	SAT1_CF	blue	na
chrUn	16789167	16789770	604	SAT1_CF	blue	na
chrUn	16789783	16789888	106	SAT1_CF	blue	na
chrUn	16789909	16790462	554	SAT1_CF	blue	na
chrUn	16790474	16791337	864	SAT1_CF	blue	na
chrUn	16791379	16793085	1707	SAT1_CF	blue	na
chrUn	16793120	16793289	170	SAT1_CF	blue	na
chrUn	16793302	16793803	502	SAT1_CF	blue	na
chrUn	16793843	16794243	401	SAT1_CF	blue	na
chrUn	16794254	16794801	548	SAT1_CF	blue	na
chrUn	16794881	16795185	305	SAT1_CF	blue	na
chrUn	16795230	16796563	1334	SAT1_CF	blue	na
chrUn	16796575	16797334	760	SAT1_CF	blue	na
chrUn	16797348	16798575	1228	SAT1_CF	blue	na
chrUn	16798587	16799059	473	SAT1_CF	blue	na
chrUn	16799080	16799189	110	SAT1_CF	blue	na

chrUn	16799200	16800319	1120	SAT1_CF	blue	na
chrUn	16800362	16801038	677	SAT1_CF	blue	na
chrUn	16801050	16801624	575	SAT1_CF	blue	na
chrUn	16801645	16801753	109	SAT1_CF	blue	na
chrUn	16801766	16802595	830	SAT1_CF	blue	na
chrUn	16802609	16803579	971	SAT1_CF	blue	na
chrUn	16803623	16804825	1203	SAT1_CF	blue	na
chrUn	16804847	16805521	675	SAT1_CF	blue	na
chrUn	16805543	16805650	108	SAT1_CF	blue	na
chrUn	16805663	16806163	501	SAT1_CF	blue	na
chrUn	16806203	16806608	406	SAT1_CF	blue	na
chrUn	16806622	16807692	1071	SAT1_CF	blue	na
chrUn	16807706	16808244	539	SAT1_CF	blue	na
chrUn	16808287	16808961	675	SAT1_CF	blue	na
chrUn	16808973	16809732	760	SAT1_CF	blue	na
chrUn	16809745	16810376	632	SAT1_CF	blue	na
chrUn	16810393	16810505	113	SAT1_CF	blue	na
chrUn	16810516	16811018	503	SAT1_CF	blue	na
chrUn	16811071	16813079	2009	SAT1_CF	blue	na
chrUn	16813110	16813603	494	SAT1_CF	blue	na
chrUn	16813705	16814634	930	SAT1_CF	blue	na
chrUn	16814703	16815674	972	SAT1_CF	blue	na
chrUn	16815723	16816380	658	SAT1_CF	blue	na
chrUn	16816407	16816909	503	SAT1_CF	blue	na
chrUn	16816996	16817351	356	SAT1_CF	blue	na
chrUn	16817364	16817873	510	SAT1_CF	blue	na
chrUn	16817952	16818991	1040	SAT1_CF	blue	na
chrUn	16819035	16819201	167	SAT1_CF	blue	na
chrUn	16819217	16819781	565	SAT1_CF	blue	na
chrUn	16819793	16820646	854	SAT1_CF	blue	na
chrUn	16820658	16821260	603	SAT1_CF	blue	na
chrUn	16821273	16821775	503	SAT1_CF	blue	na
chrUn	16821820	16821977	158	SAT1_CF	blue	na
chrUn	16822005	16823715	1711	SAT1_CF	blue	na
chrUn	16823727	16824225	499	SAT1_CF	blue	na
chrUn	16824265	16825297	1033	SAT1_CF	blue	na
chrUn	16825320	16825426	107	SAT1_CF	blue	na
chrUn	16825439	16825937	499	SAT1_CF	blue	na
chrUn	16825973	16826157	185	SAT1_CF	blue	na
chrUn	16826341	16826507	167	SAT1_CF	blue	na
chrUn	16826588	16828252	1665	SAT1_CF	blue	na
chrUn	16828295	16829505	1211	SAT1_CF	blue	na

chrUn	16829531	16829688	158	SAT1_CF	blue	na
chrUn	16829703	16831026	1324	SAT1_CF	blue	na
chrUn	16831049	16831520	472	SAT1_CF	blue	na
chrUn	16831594	16831761	168	SAT1_CF	blue	na
chrUn	16831941	16832175	235	SAT1_CF	blue	na
chrUn	16832186	16832731	546	SAT1_CF	blue	na
chrUn	16832753	16833744	992	SAT1_CF	blue	na
chrUn	16833788	16834975	1188	SAT1_CF	blue	na
chrUn	16835030	16835903	874	SAT1_CF	blue	na
chrUn	16835946	16836734	789	SAT1_CF	blue	na
chrUn	16836759	16837593	835	SAT1_CF	blue	na
chrUn	16837614	16837722	109	SAT1_CF	blue	na
chrUn	16837735	16838236	502	SAT1_CF	blue	na
chrUn	16838274	16838678	405	SAT1_CF	blue	na
chrUn	16838691	16839295	605	SAT1_CF	blue	na
chrUn	16839350	16840314	965	SAT1_CF	blue	na
chrUn	16840357	16840865	509	SAT1_CF	blue	na
chrUn	16840886	16840961	76	SAT1_CF	blue	na
chrUn	16840972	16841473	502	SAT1_CF	blue	na
chrUn	16841518	16841675	158	SAT1_CF	blue	na
chrUn	16841707	16842213	507	SAT1_CF	blue	na
chrUn	16842244	16843704	1461	SAT1_CF	blue	na
chrUn	16843716	16845353	1638	SAT1_CF	blue	na
chrUn	16845396	16846071	676	SAT1_CF	blue	na
chrUn	16846084	16846567	484	SAT1_CF	blue	na
chrUn	16846671	16847218	548	SAT1_CF	blue	na
chrUn	16847232	16847932	701	SAT1_CF	blue	na
chrUn	16847973	16849656	1684	SAT1_CF	blue	na
chrUn	16849683	16850163	481	SAT1_CF	blue	na
chrUn	16850219	16850589	371	SAT1_CF	blue	na
chrUn	16850634	16851249	616	SAT1_CF	blue	na
chrUn	16851265	16852259	995	SAT1_CF	blue	na
chrUn	16852302	16852978	677	SAT1_CF	blue	na
chrUn	16852992	16853750	759	SAT1_CF	blue	na
chrUn	16853762	16854900	1139	SAT1_CF	blue	na
chrUn	16854968	16855844	877	SAT1_CF	blue	na
chrUn	16855960	16857543	1584	SAT1_CF	blue	na
chrUn	16857563	16858058	496	SAT1_CF	blue	na
chrUn	16858098	16858280	183	SAT1_CF	blue	na
chrUn	16858651	16860339	1689	SAT1_CF	blue	na
chrUn	16860381	16861243	863	SAT1_CF	blue	na
chrUn	16861255	16862632	1378	SAT1_CF	blue	na

chrUn	16862652	16863145	494	SAT1_CF	blue	na
chrUn	16863187	16863589	403	SAT1_CF	blue	na
chrUn	16863600	16864210	611	SAT1_CF	blue	na
chrUn	16864230	16864832	603	SAT1_CF	blue	na
chrUn	16864912	16865458	547	SAT1_CF	blue	na
chrUn	16865471	16865587	117	SAT1_CF	blue	na
chrUn	16865599	16866270	672	SAT1_CF	blue	na
chrUn	16866293	16866794	502	SAT1_CF	blue	na
chrUn	16866860	16866996	137	SAT1_CF	blue	na
chrUn	16867024	16867572	549	SAT1_CF	blue	na
chrUn	16867602	16867868	267	SAT1_CF	blue	na
chrUn	16867947	16870121	2175	SAT1_CF	blue	na
chrUn	16870136	16870675	540	SAT1_CF	blue	na
chrUn	16870718	16871394	677	SAT1_CF	blue	na
chrUn	16871406	16872733	1328	SAT1_CF	blue	na
chrUn	16872758	16873376	619	SAT1_CF	blue	na
chrUn	16873422	16873816	395	SAT1_CF	blue	na
chrUn	16873831	16874351	521	SAT1_CF	blue	na
chrUn	16874382	16874921	540	SAT1_CF	blue	na
chrUn	16874932	16875457	526	SAT1_CF	blue	na
chrUn	16875500	16876173	674	SAT1_CF	blue	na
chrUn	16876185	16876865	681	SAT1_CF	blue	na
chrUn	16876877	16877723	847	SAT1_CF	blue	na
chrUn	16877754	16878622	869	SAT1_CF	blue	na
chrUn	16878663	16879189	527	SAT1_CF	blue	na
chrUn	16879241	16879359	119	SAT1_CF	blue	na
chrUn	16879406	16880079	674	SAT1_CF	blue	na
chrUn	16880091	16880257	167	SAT1_CF	blue	na
chrUn	16880275	16880757	483	SAT1_CF	blue	na
chrUn	16880863	16881752	890	SAT1_CF	blue	na
chrUn	16881784	16882637	854	SAT1_CF	blue	na
chrUn	16882669	16883556	888	SAT1_CF	blue	na
chrUn	16883568	16884327	760	SAT1_CF	blue	na
chrUn	16884340	16884839	500	SAT1_CF	blue	na
chrUn	16884884	16885041	158	SAT1_CF	blue	na
chrUn	16885080	16887378	2299	SAT1_CF	blue	na
chrUn	16887390	16887951	562	SAT1_CF	blue	na
chrUn	16887972	16888080	109	SAT1_CF	blue	na
chrUn	16888093	16888593	501	SAT1_CF	blue	na
chrUn	16888638	16889035	398	SAT1_CF	blue	na
chrUn	16889047	16890450	1404	SAT1_CF	blue	na
chrUn	16890481	16890522	42	SAT1_CF	blue	na

chrUn	16890535	16891258	724	SAT1_CF	blue	na
chrUn	16891279	16893791	2513	SAT1_CF	blue	na
chrUn	16893834	16894508	675	SAT1_CF	blue	na
chrUn	16894520	16894996	477	SAT1_CF	blue	na
chrUn	16895066	16895739	674	SAT1_CF	blue	na
chrUn	16895751	16896915	1165	SAT1_CF	blue	na
chrUn	16896933	16898617	1685	SAT1_CF	blue	na
chrUn	16898629	16899299	671	SAT1_CF	blue	na
chrUn	16899320	16899883	564	SAT1_CF	blue	na
chrUn	16899926	16900602	677	SAT1_CF	blue	na
chrUn	16900614	16901095	482	SAT1_CF	blue	na
chrUn	16901201	16901713	513	SAT1_CF	blue	na
chrUn	16901750	16902294	545	SAT1_CF	blue	na
chrUn	16902308	16903191	884	SAT1_CF	blue	na
chrUn	16903234	16903899	666	SAT1_CF	blue	na
chrUn	16903912	16904594	683	SAT1_CF	blue	na
chrUn	16904607	16905108	502	SAT1_CF	blue	na
chrUn	16905141	16906200	1060	SAT1_CF	blue	na
chrUn	16906216	16907176	961	SAT1_CF	blue	na
chrUn	16907224	16907889	666	SAT1_CF	blue	na
chrUn	16907911	16908385	475	SAT1_CF	blue	na
chrUn	16908456	16908999	544	SAT1_CF	blue	na
chrUn	16909021	16909129	109	SAT1_CF	blue	na
chrUn	16909143	16909829	687	SAT1_CF	blue	na
chrUn	16909841	16910415	575	SAT1_CF	blue	na
chrUn	16910438	16910544	107	SAT1_CF	blue	na
chrUn	16910558	16911608	1051	SAT1_CF	blue	na
chrUn	16911647	16913685	2039	SAT1_CF	blue	na
chrUn	16913728	16914402	675	SAT1_CF	blue	na
chrUn	16914414	16914970	557	SAT1_CF	blue	na
chrUn	16915009	16915739	731	SAT1_CF	blue	na
chrUn	16915918	16915994	77	SAT1_CF	blue	na
chrUn	16916010	16917876	1867	SAT1_CF	blue	na
chrUn	16917920	16918701	782	SAT1_CF	blue	na
chrUn	16918743	16920016	1274	SAT1_CF	blue	na
chrUn	16920027	16920709	683	SAT1_CF	blue	na
chrUn	16920722	16921241	520	SAT1_CF	blue	na
chrUn	16921265	16923323	2059	SAT1_CF	blue	na
chrUn	16923365	16924736	1372	SAT1_CF	blue	na
chrUn	16924756	16926725	1970	SAT1_CF	blue	na
chrUn	16926747	16927242	496	SAT1_CF	blue	na
chrUn	16927286	16927444	159	SAT1_CF	blue	na

chrUn	16927472	16929166	1695	SAT1_CF	blue	na
chrUn	16929208	16930069	862	SAT1_CF	blue	na
chrUn	16930083	16931298	1216	SAT1_CF	blue	na
chrUn	16931319	16933283	1965	SAT1_CF	blue	na
chrUn	16933325	16934694	1370	SAT1_CF	blue	na
chrUn	16934707	16935207	501	SAT1_CF	blue	na
chrUn	16935220	16935799	580	SAT1_CF	blue	na
chrUn	16935817	16937595	1779	CarSat1	red	na
chrUn	16937809	16939612	1804	CarSat1	red	na
chrUn	16939627	16939750	124	Carsat2	orange	na
chrUn	16939642	16941823	2182	CarSat1	red	na
chrUn	16941853	16943296	1444	CarSat1	red	na
chrUn	16943311	16943437	127	Carsat2	orange	na
chrUn	16943326	16944032	707	CarSat1	red	na
chrUn	16944061	16944772	712	CarSat1	red	na
chrUn	16944802	16945512	711	CarSat1	red	na
chrUn	16945527	16945654	128	Carsat2	orange	na
chrUn	16945542	16949718	4177	CarSat1	red	na
chrUn	16949734	16952139	2406	CarSat1	red	na
chrUn	16953735	16954164	430	CarSat1	red	na
chrUn	16954182	16954901	720	CarSat1	red	na
chrUn	16954896	16955511	616	SAT1_CF	blue	na
chrUn	16955524	16956032	509	SAT1_CF	blue	na
chrUn	16956073	16957347	1275	SAT1_CF	blue	na
chrUn	16957385	16958866	1482	SAT1_CF	blue	na
chrUn	16958878	16959349	472	SAT1_CF	blue	na
chrUn	16959351	16961314	1964	CarSat1	red	na
chrUn	16961340	16962929	1590	CarSat1	red	na
chrUn	16962951	16963653	703	CarSat1	red	na
chrUn	16978261	16978419	159	Sat2CF	purple	na
chrUn	16978284	16978994	711	Sat2CF	purple	na
chrUn	17009574	17010724	1151	Sat2CF	purple	na
chrUn	17030942	17031104	163	Sat2CF	purple	na
chrUn	17030970	17031685	716	Sat2CF	purple	na
chrUn	17055949	17057103	1155	Sat2CF	purple	na
chrUn	17056975	17057123	149	Sat2CF	purple	na
chrUn	17068005	17069173	1169	Sat2CF	purple	na
chrUn	17068645	17069240	596	Sat2CF	purple	na
chrUn	17083933	17084731	799	Sat2CF	purple	na
chrUn	17084613	17084781	169	Sat2CF	purple	na
chrUn	17096585	17097759	1175	Sat2CF	purple	na
chrUn	17136144	17136956	813	Sat2CF	purple	na

chrUn	17136218	17136943	726	Sat2CF	purple	na
chrUn	17160043	17160780	738	Sat2CF	purple	na
chrUn	17160117	17160778	662	Sat2CF	purple	na
chrUn	17192561	17193813	1253	Sat2CF	purple	na
chrUn	17192691	17193777	1087	Sat2CF	purple	na
chrUn	17217381	17217543	163	Sat2CF	purple	na
chrUn	17217407	17218255	849	Sat2CF	purple	na
chrUn	17217588	17218336	749	Sat2CF	purple	na
chrUn	17246651	17247274	624	Sat2CF	purple	na
chrUn	17246720	17247285	566	Sat2CF	purple	na
chrUn	17262398	17263541	1144	Sat2CF	purple	na
chrUn	17262402	17263670	1269	Sat2CF	purple	na
chrUn	17280938	17282106	1169	Sat2CF	purple	na
chrUn	17397352	17398603	1252	Sat2CF	purple	na
chrUn	17397482	17398567	1086	Sat2CF	purple	na
chrUn	17401274	17401413	140	CarSat1	red	na
chrUn	17401434	17403026	1593	CarSat1	red	na
chrUn	17403047	17404531	1485	CarSat1	red	na
chrUn	17414182	17414295	114	Carsat2	orange	na
chrUn	17414209	17415604	1396	CarSat1	red	na
chrUn	17415619	17416404	786	CarSat1	red	na
chrUn	17416423	17416825	403	CarSat1	red	na
chrUn	17416854	17418230	1377	SAT1_CF	blue	na
chrUn	17418425	17418587	163	Sat2CF	purple	na
chrUn	17418458	17419471	1014	Sat2CF	purple	na
chrUn	17418546	17419408	863	Sat2CF	purple	na
chrUn	17425256	17425668	413	CarSat1	red	na
chrUn	17425702	17426197	496	CarSat1	red	na
chrUn	17426213	17426801	589	CarSat1	red	na
chrUn	17427006	17427596	591	CarSat1	red	na
chrUn	17429264	17430787	1524	CarSat1	red	na
chrUn	17430808	17431522	715	CarSat1	red	na
chrUn	17431543	17432992	1450	CarSat1	red	na
chrUn	17433013	17433727	715	CarSat1	red	na
chrUn	17433748	17434462	715	CarSat1	red	na
chrUn	17434483	17435197	715	CarSat1	red	na
chrUn	17435218	17435932	715	CarSat1	red	na
chrUn	17435953	17436667	715	CarSat1	red	na
chrUn	17436688	17438389	1702	CarSat1	red	na
chrUn	17438405	17439126	722	CarSat1	red	na
chrUn	17439142	17439864	723	CarSat1	red	na
chrUn	17439880	17441828	1949	CarSat1	red	na

chrUn	17441855	17443553	1699	CarSat1	red	na
chrUn	17443569	17444291	723	CarSat1	red	na
chrUn	17444307	17445028	722	CarSat1	red	na
chrUn	17445044	17446500	1457	CarSat1	red	na
chrUn	17446516	17447721	1206	CarSat1	red	na
chrUn	17447748	17448709	962	CarSat1	red	na
chrUn	17448725	17452388	3664	CarSat1	red	na
chrUn	17452413	17453589	1177	CarSat1	red	na
chrUn	17454113	17454597	485	CarSat1	red	na
chrUn	17454622	17455087	466	CarSat1	red	na
chrUn	17455114	17460491	5378	CarSat1	red	na
chrUn	17460516	17461779	1264	CarSat1	red	na
chrUn	17473105	17473998	894	CarSat1	red	na
chrUn	17474221	17475666	1446	CarSat1	red	na
chrUn	17475697	17477285	1589	CarSat1	red	na
chrUn	17477316	17479477	2162	CarSat1	red	na
chrUn	17479682	17480395	714	CarSat1	red	na
chrUn	17640435	17640603	169	Sat2CF	purple	na
chrUn	17640485	17641279	795	Sat2CF	purple	na
chrUn	17656147	17656324	178	Sat2CF	purple	na
chrUn	17656195	17656710	516	Sat2CF	purple	na
chrUn	17656147	17656324	178	Sat2CF	purple	na
chrUn	17656420	17656751	332	Sat2CF	purple	na
chrUn	17665805	17666925	1121	Sat2CF	purple	na
chrUn	17681761	17682558	798	Sat2CF	purple	na
chrUn	17760057	17760794	738	Sat2CF	purple	na
chrUn	17760059	17760720	662	Sat2CF	purple	na
chrUn	17784094	17784906	813	Sat2CF	purple	na
chrUn	17784107	17784832	726	Sat2CF	purple	na
chrUn	17802841	17803957	1117	Sat2CF	purple	na
chrUn	17822481	17822994	514	Sat2CF	purple	na
chrUn	17822543	17823390	848	Sat2CF	purple	na
chrUn	17822767	17823465	699	Sat2CF	purple	na
chrUn	17850155	17851338	1184	Sat2CF	purple	na
chrUn	17879852	17881029	1178	Sat2CF	purple	na
chrUn	17892902	17893700	799	Sat2CF	purple	na
chrUn	17920440	17920588	149	Sat2CF	purple	na
chrUn	17920460	17921542	1083	Sat2CF	purple	na
chrUn	17920440	17920588	149	Sat2CF	purple	na
chrUn	17940626	17941342	717	Sat2CF	purple	na
chrUn	17941208	17941370	163	Sat2CF	purple	na
chrUn	17959866	17961001	1136	Sat2CF	purple	na

chrUn	17982809	17983523	715	Sat2CF	purple	na
chrUn	17983201	17983562	362	Sat2CF	purple	na
chrUn	17994262	17994965	704	CarSat1	red	na
chrUn	17994987	17996960	1974	CarSat1	red	na
chrUn	17996962	17997432	471	SAT1_CF	blue	na
chrUn	17997444	18000243	2800	SAT1_CF	blue	na
chrUn	18000283	18000785	503	SAT1_CF	blue	na
chrUn	18000803	18001413	611	SAT1_CF	blue	na
chrUn	18001408	18002121	714	CarSat1	red	na
chrUn	18002273	18002566	294	CarSat1	red	na
chrUn	18011686	18013172	1487	CarSat1	red	na
chrUn	18013451	18013663	213	CarSat1	red	na
chrUn	18454111	18455289	1179	Sat2CF	purple	na
chrUn	18478340	18479415	1076	Sat2CF	purple	na
chrUn	18492278	18492668	391	Sat2CF	purple	na
chrUn	18492344	18492694	351	Sat2CF	purple	na
chrUn	18552236	18553411	1176	Sat2CF	purple	na
chrUn	18570556	18571453	898	Sat2CF	purple	na
chrUn	18570619	18571408	790	Sat2CF	purple	na
chrUn	18588788	18589919	1132	Sat2CF	purple	na
chrUn	18608346	18609299	954	Sat2CF	purple	na
chrUn	18608409	18609219	811	Sat2CF	purple	na
chrUn	18627505	18628756	1252	Sat2CF	purple	na
chrUn	18627635	18628720	1086	Sat2CF	purple	na
chrUn	18664702	18665587	886	Sat2CF	purple	na
chrUn	18683451	18684238	788	Sat2CF	purple	na
chrUn	18699742	18700941	1200	Sat2CF	purple	na
chrUn	18699871	18700957	1087	Sat2CF	purple	na
chrUn	18734431	18734595	165	Sat2CF	purple	na
chrUn	18734459	18735310	852	Sat2CF	purple	na
chrUn	18734708	18735381	674	Sat2CF	purple	na
chrUn	18763691	18764911	1221	Sat2CF	purple	na
chrUn	18764782	18764931	150	Sat2CF	purple	na
chrUn	18780028	18780406	379	Sat2CF	purple	na
chrUn	18780464	18780815	352	Sat2CF	purple	na
chrUn	18780500	18781846	1347	Sat2CF	purple	na
chrUn	18780708	18782051	1344	Sat2CF	purple	na
chrUn	18781324	18782136	813	Sat2CF	purple	na
chrUn	18799713	18800881	1169	Sat2CF	purple	na
chrUn	18800703	18800901	199	Sat2CF	purple	na
chrUn	18800773	18800920	148	Sat2CF	purple	na
chrUn	18812984	18813795	812	Sat2CF	purple	na

chrUn	18813049	18813774	726	Sat2CF	purple	na
chrUn	18813664	18813832	169	Sat2CF	purple	na
chrUn	18826819	18827483	665	Sat2CF	purple	na
chrUn	18835423	18835523	101	SAT1_CF	blue	na
chrUn	18835542	18835585	44	SAT1_CF	blue	na
chrUn	18835616	18836160	545	SAT1_CF	blue	na
chrUn	18836203	18836750	548	SAT1_CF	blue	na
chrUn	18836761	18837215	455	SAT1_CF	blue	na
chrUn	18837280	18838386	1107	CarSat1	red	na
chrUn	18838540	18840131	1592	CarSat1	red	na
chrUn	18840152	18841601	1450	CarSat1	red	na
chrUn	18841760	18842465	706	CarSat1	red	na
chrUn	18867015	18868025	1011	Sat2CF	purple	na
chrUn	18867897	18868045	149	Sat2CF	purple	na
chrUn	18895974	18896908	935	Sat2CF	purple	na
chrUn	18896037	18896827	791	Sat2CF	purple	na
chrUn	18903044	18903389	346	Sat2CF	purple	na
chrUn	18903103	18903893	791	Sat2CF	purple	na
chrUn	18922278	18923394	1117	Sat2CF	purple	na
chrUn	18941587	18942528	942	Sat2CF	purple	na
chrUn	18963816	18964553	738	Sat2CF	purple	na
chrUn	18963890	18964551	662	Sat2CF	purple	na
chrUn	19004558	19005792	1235	Sat2CF	purple	na
chrUn	19004685	19005805	1121	Sat2CF	purple	na
chrUn	19029789	19030712	924	Sat2CF	purple	na
chrUn	19029852	19030661	810	Sat2CF	purple	na
chrUn	19062347	19062868	522	Sat2CF	purple	na
chrUn	19062411	19063238	828	Sat2CF	purple	na
chrUn	19079777	19080902	1126	Sat2CF	purple	na
chrUn	19079904	19080941	1038	Sat2CF	purple	na
chrUn	19112155	19113322	1168	Sat2CF	purple	na
chrUn	19142090	19143269	1180	Sat2CF	purple	na
chrUn	19143109	19143308	200	Sat2CF	purple	na
chrUn	19170997	19171934	938	Sat2CF	purple	na
chrUn	19173653	19174066	414	CarSat1	red	na
chrUn	19175034	19176270	1237	CarSat1	red	na
chrUn	19176308	19177179	872	CarSat1	red	na
chrUn	19177174	19180942	3769	SAT1_CF	blue	na
chrUn	19180954	19181119	166	SAT1_CF	blue	na
chrUn	19181131	19181737	607	SAT1_CF	blue	na
chrUn	19181748	19182292	545	SAT1_CF	blue	na
chrUn	19182305	19182935	631	SAT1_CF	blue	na

chrUn	19189247	19190947	1701	SAT1_CF	blue	na
chrUn	19190952	19191434	483	CarSat1	red	na
chrUn	19191463	19192841	1379	CarSat1	red	na
chrUn	19192871	19193575	705	CarSat1	red	na
chrUn	19214489	19215265	777	Sat2CF	purple	na
chrUn	19214554	19215245	692	Sat2CF	purple	na
chrUn	19217954	19218530	577	SAT1_CF	blue	na
chrUn	19218543	19219042	500	SAT1_CF	blue	na
chrUn	19219082	19220112	1031	SAT1_CF	blue	na
chrUn	19220139	19220332	194	SAT1_CF	blue	na
chrUn	19220412	19220974	563	SAT1_CF	blue	na
chrUn	19221018	19222223	1206	SAT1_CF	blue	na
chrUn	19222280	19222804	525	SAT1_CF	blue	na
chrUn	19222827	19223547	721	SAT1_CF	blue	na
chrUn	19223651	19224985	1335	SAT1_CF	blue	na
chrUn	19225029	19226393	1365	SAT1_CF	blue	na
chrUn	19226406	19226907	502	SAT1_CF	blue	na
chrUn	19226946	19227127	182	SAT1_CF	blue	na
chrUn	19227309	19229345	2037	SAT1_CF	blue	na
chrUn	19229359	19230120	762	SAT1_CF	blue	na
chrUn	19230133	19230632	500	SAT1_CF	blue	na
chrUn	19230672	19231702	1031	SAT1_CF	blue	na
chrUn	19231745	19232290	546	SAT1_CF	blue	na
chrUn	19232340	19233040	701	SAT1_CF	blue	na
chrUn	19233090	19233706	617	SAT1_CF	blue	na
chrUn	19233729	19233834	106	SAT1_CF	blue	na
chrUn	19233847	19234348	502	SAT1_CF	blue	na
chrUn	19234388	19234568	181	SAT1_CF	blue	na
chrUn	19234748	19236795	2048	SAT1_CF	blue	na
chrUn	19236809	19237490	682	SAT1_CF	blue	na
chrUn	19237503	19238203	701	SAT1_CF	blue	na
chrUn	19238254	19238742	489	SAT1_CF	blue	na
chrUn	19238773	19240076	1304	SAT1_CF	blue	na
chrUn	19240124	19241454	1331	SAT1_CF	blue	na
chrUn	19241467	19242227	761	SAT1_CF	blue	na
chrUn	19242240	19242740	501	SAT1_CF	blue	na
chrUn	19242800	19242942	143	SAT1_CF	blue	na
chrUn	19242957	19243702	746	SAT1_CF	blue	na
chrUn	19243763	19244986	1224	SAT1_CF	blue	na
chrUn	19244999	19245797	799	SAT1_CF	blue	na
chrUn	19245841	19246730	890	SAT1_CF	blue	na
chrUn	19246769	19248069	1301	SAT1_CF	blue	na

chrUn	19248081	19248779	699	SAT1_CF	blue	na
chrUn	19248797	19249387	591	SAT1_CF	blue	na
chrUn	19249406	19249515	110	SAT1_CF	blue	na
chrUn	19249527	19250205	679	SAT1_CF	blue	na
chrUn	19250218	19251162	945	SAT1_CF	blue	na
chrUn	19251175	19252984	1810	SAT1_CF	blue	na
chrUn	19253004	19253939	936	SAT1_CF	blue	na
chrUn	19253951	19254111	161	SAT1_CF	blue	na
chrUn	19254134	19254679	546	SAT1_CF	blue	na
chrUn	19254693	19255392	700	SAT1_CF	blue	na
chrUn	19255434	19256294	861	SAT1_CF	blue	na
chrUn	19256307	19256652	346	SAT1_CF	blue	na
chrUn	19263526	19264602	1077	SAT1_CF	blue	na
chrUn	19264628	19264784	157	SAT1_CF	blue	na
chrUn	19264799	19266124	1326	SAT1_CF	blue	na
chrUn	19266147	19266617	471	SAT1_CF	blue	na
chrUn	19266678	19266858	181	SAT1_CF	blue	na
chrUn	19267038	19267240	203	SAT1_CF	blue	na
chrUn	19267283	19269510	2228	SAT1_CF	blue	na
chrUn	19269535	19270037	503	SAT1_CF	blue	na
chrUn	19270153	19270364	212	SAT1_CF	blue	na
chrUn	19270385	19270488	104	SAT1_CF	blue	na
chrUn	19270506	19271050	545	SAT1_CF	blue	na
chrUn	19271127	19272185	1059	SAT1_CF	blue	na
chrUn	19272240	19273019	780	SAT1_CF	blue	na
chrUn	19273067	19274400	1334	SAT1_CF	blue	na
chrUn	19274442	19275693	1252	SAT1_CF	blue	na
chrUn	19275705	19276465	761	SAT1_CF	blue	na
chrUn	19276478	19276980	503	SAT1_CF	blue	na
chrUn	19277033	19277421	389	SAT1_CF	blue	na
chrUn	19277432	19278295	864	SAT1_CF	blue	na
chrUn	19278333	19279427	1095	SAT1_CF	blue	na
chrUn	19279484	19280031	548	SAT1_CF	blue	na
chrUn	19280074	19281174	1101	SAT1_CF	blue	na
chrUn	19281186	19281875	690	SAT1_CF	blue	na
chrUn	19281888	19282637	750	SAT1_CF	blue	na
chrUn	19282649	19283204	556	SAT1_CF	blue	na
chrUn	19283225	19283332	108	SAT1_CF	blue	na
chrUn	19283352	19283890	539	SAT1_CF	blue	na
chrUn	19284007	19284919	913	SAT1_CF	blue	na
chrUn	19284997	19285322	326	SAT1_CF	blue	na
chrUn	19285346	19286679	1334	SAT1_CF	blue	na

chrUn	19286692	19287192	501	SAT1_CF	blue	na
chrUn	19287237	19287393	157	SAT1_CF	blue	na
chrUn	19287426	19288600	1175	SAT1_CF	blue	na
chrUn	19288623	19288728	106	SAT1_CF	blue	na
chrUn	19288741	19288915	175	SAT1_CF	blue	na
chrUn	19288927	19289423	497	SAT1_CF	blue	na
chrUn	19289462	19289862	401	SAT1_CF	blue	na
chrUn	19289874	19291416	1543	SAT1_CF	blue	na
chrUn	19291428	19291986	559	SAT1_CF	blue	na
chrUn	19292051	19292597	547	SAT1_CF	blue	na
chrUn	19292640	19292965	326	SAT1_CF	blue	na
chrUn	19292990	19294323	1334	SAT1_CF	blue	na
chrUn	19294335	19295570	1236	SAT1_CF	blue	na
chrUn	19295613	19296251	639	SAT1_CF	blue	na
chrUn	19296264	19296940	677	SAT1_CF	blue	na
chrUn	19296974	19298467	1494	SAT1_CF	blue	na
chrUn	19298511	19298848	338	SAT1_CF	blue	na
chrUn	19298896	19301488	2593	SAT1_CF	blue	na
chrUn	19301516	19302316	801	SAT1_CF	blue	na
chrUn	19302360	19303010	651	SAT1_CF	blue	na
chrUn	19303024	19304365	1342	SAT1_CF	blue	na
chrUn	19304392	19304877	486	SAT1_CF	blue	na
chrUn	19304903	19306419	1517	SAT1_CF	blue	na
chrUn	19306434	19306975	542	SAT1_CF	blue	na
chrUn	19307018	19307683	666	SAT1_CF	blue	na
chrUn	19307696	19308197	502	SAT1_CF	blue	na
chrUn	19308237	19310269	2033	SAT1_CF	blue	na
chrUn	19310312	19310977	666	SAT1_CF	blue	na
chrUn	19310999	19312047	1049	SAT1_CF	blue	na
chrUn	19312152	19312975	824	SAT1_CF	blue	na
chrUn	19312988	19313929	942	SAT1_CF	blue	na
chrUn	19313981	19314169	189	SAT1_CF	blue	na
chrUn	19314182	19314703	522	SAT1_CF	blue	na
chrUn	19314770	19315547	778	SAT1_CF	blue	na
chrUn	19315589	19316318	730	SAT1_CF	blue	na
chrUn	19316423	19317107	685	SAT1_CF	blue	na
chrUn	19317120	19317971	852	SAT1_CF	blue	na
chrUn	19317993	19318878	886	SAT1_CF	blue	na
chrUn	19318890	19319567	678	SAT1_CF	blue	na
chrUn	19319585	19320249	665	SAT1_CF	blue	na
chrUn	19320276	19320758	483	SAT1_CF	blue	na
chrUn	19320864	19321424	561	SAT1_CF	blue	na

chrUn	19321489	19322120	632	SAT1_CF	blue	na
chrUn	19322163	19322828	666	SAT1_CF	blue	na
chrUn	19322841	19323522	682	SAT1_CF	blue	na
chrUn	19323535	19324335	801	SAT1_CF	blue	na
chrUn	19324351	19324464	114	SAT1_CF	blue	na
chrUn	19324476	19324991	516	SAT1_CF	blue	na
chrUn	19325135	19326046	912	SAT1_CF	blue	na
chrUn	19326106	19327803	1698	SAT1_CF	blue	na
chrUn	19327815	19329061	1247	SAT1_CF	blue	na
chrUn	19329205	19330116	912	SAT1_CF	blue	na
chrUn	19330195	19330684	490	SAT1_CF	blue	na
chrUn	19330695	19331872	1178	SAT1_CF	blue	na
chrUn	19331884	19333296	1413	SAT1_CF	blue	na
chrUn	19333319	19333790	472	SAT1_CF	blue	na
chrUn	19333850	19334030	181	SAT1_CF	blue	na
chrUn	19334210	19334412	203	SAT1_CF	blue	na
chrUn	19334455	19335830	1376	SAT1_CF	blue	na
chrUn	19335881	19336044	164	SAT1_CF	blue	na
chrUn	19336056	19337374	1319	SAT1_CF	blue	na
chrUn	19337397	19337502	106	SAT1_CF	blue	na
chrUn	19337515	19337995	481	SAT1_CF	blue	na
chrUn	19338051	19338236	186	SAT1_CF	blue	na
chrUn	19338417	19339209	793	SAT1_CF	blue	na
chrUn	19339289	19340460	1172	SAT1_CF	blue	na
chrUn	19340472	19341141	670	SAT1_CF	blue	na
chrUn	19341166	19341668	503	SAT1_CF	blue	na
chrUn	19341755	19342133	379	SAT1_CF	blue	na
chrUn	19342156	19342262	107	SAT1_CF	blue	na
chrUn	19342275	19342819	545	SAT1_CF	blue	na
chrUn	19342896	19344789	1894	SAT1_CF	blue	na
chrUn	19344837	19345714	878	SAT1_CF	blue	na
chrUn	19345739	19346279	541	SAT1_CF	blue	na
chrUn	19346376	19347053	678	SAT1_CF	blue	na
chrUn	19347065	19347748	684	SAT1_CF	blue	na
chrUn	19347761	19348262	502	SAT1_CF	blue	na
chrUn	19348302	19348671	370	SAT1_CF	blue	na
chrUn	19348714	19349237	524	SAT1_CF	blue	na
chrUn	19349269	19350929	1661	SAT1_CF	blue	na
chrUn	19350993	19351180	188	SAT1_CF	blue	na
chrUn	19351193	19351713	521	SAT1_CF	blue	na
chrUn	19351780	19352072	293	SAT1_CF	blue	na
chrUn	19352130	19352671	542	SAT1_CF	blue	na

chrUn	19352714	19353354	641	SAT1_CF	blue	na
chrUn	19353371	19354132	762	SAT1_CF	blue	na
chrUn	19354145	19354645	501	SAT1_CF	blue	na
chrUn	19354685	19355064	380	SAT1_CF	blue	na
chrUn	19355098	19355619	522	SAT1_CF	blue	na
chrUn	19355633	19356136	504	SAT1_CF	blue	na
chrUn	19356180	19356705	526	SAT1_CF	blue	na
chrUn	19356736	19357255	520	SAT1_CF	blue	na
chrUn	19357371	19357726	356	SAT1_CF	blue	na
chrUn	19357739	19359402	1664	SAT1_CF	blue	na
chrUn	19359445	19360117	673	SAT1_CF	blue	na
chrUn	19360129	19360686	558	SAT1_CF	blue	na
chrUn	19360708	19360814	107	SAT1_CF	blue	na
chrUn	19360827	19361329	503	SAT1_CF	blue	na
chrUn	19361395	19361531	137	SAT1_CF	blue	na
chrUn	19361546	19362818	1273	SAT1_CF	blue	na
chrUn	19362840	19365337	2498	SAT1_CF	blue	na
chrUn	19365379	19366244	866	SAT1_CF	blue	na
chrUn	19366256	19366709	454	SAT1_CF	blue	na
chrUn	19366722	19367223	502	SAT1_CF	blue	na
chrUn	19367263	19368184	922	SAT1_CF	blue	na
chrUn	19368215	19369109	895	SAT1_CF	blue	na
chrUn	19369122	19369669	548	SAT1_CF	blue	na
chrUn	19369726	19370683	958	SAT1_CF	blue	na
chrUn	19370726	19372912	2187	SAT1_CF	blue	na
chrUn	19372924	19373606	683	SAT1_CF	blue	na
chrUn	19373626	19374120	495	SAT1_CF	blue	na
chrUn	19374160	19374561	402	SAT1_CF	blue	na
chrUn	19374575	19374808	234	SAT1_CF	blue	na
chrUn	19374829	19375470	642	SAT1_CF	blue	na
chrUn	19375487	19375659	173	SAT1_CF	blue	na
chrUn	19375672	19376934	1263	SAT1_CF	blue	na
chrUn	19376956	19377063	108	SAT1_CF	blue	na
chrUn	19377076	19377905	830	SAT1_CF	blue	na
chrUn	19377920	19379257	1338	SAT1_CF	blue	na
chrUn	19379299	19379842	544	SAT1_CF	blue	na
chrUn	19379885	19380562	678	SAT1_CF	blue	na
chrUn	19380574	19381093	520	SAT1_CF	blue	na
chrUn	19381110	19381480	371	SAT1_CF	blue	na
chrUn	19381525	19382140	616	SAT1_CF	blue	na
chrUn	19382156	19382656	501	SAT1_CF	blue	na
chrUn	19382678	19384289	1612	SAT1_CF	blue	na

chrUn	19384346	19384864	519	SAT1_CF	blue	na
chrUn	19384936	19385302	367	SAT1_CF	blue	na
chrUn	19385345	19386703	1359	SAT1_CF	blue	na
chrUn	19386715	19388747	2033	SAT1_CF	blue	na
chrUn	19388789	19389328	540	SAT1_CF	blue	na
chrUn	19389372	19390052	681	SAT1_CF	blue	na
chrUn	19390064	19390619	556	SAT1_CF	blue	na
chrUn	19390640	19390747	108	SAT1_CF	blue	na
chrUn	19390767	19391305	539	SAT1_CF	blue	na
chrUn	19391388	19393413	2026	SAT1_CF	blue	na
chrUn	19393456	19394130	675	SAT1_CF	blue	na
chrUn	19394143	19394662	520	SAT1_CF	blue	na
chrUn	19394688	19394844	157	SAT1_CF	blue	na
chrUn	19394877	19396048	1172	SAT1_CF	blue	na
chrUn	19396071	19396176	106	SAT1_CF	blue	na
chrUn	19396189	19396667	479	SAT1_CF	blue	na
chrUn	19396725	19397106	382	SAT1_CF	blue	na
chrUn	19397139	19397687	549	SAT1_CF	blue	na
chrUn	19397802	19398762	961	SAT1_CF	blue	na
chrUn	19398806	19399481	676	SAT1_CF	blue	na
chrUn	19399493	19400261	769	SAT1_CF	blue	na
chrUn	19400282	19400756	475	SAT1_CF	blue	na
chrUn	19400821	19401479	659	SAT1_CF	blue	na
chrUn	19401527	19402021	495	SAT1_CF	blue	na
chrUn	19405621	19406271	651	SAT1_CF	blue	na
chrUn	19406315	19407521	1207	SAT1_CF	blue	na
chrUn	19407591	19407973	383	SAT1_CF	blue	na
chrUn	19409252	19409784	533	SAT1_CF	blue	na
chrUn	19409802	19410304	503	SAT1_CF	blue	na
chrUn	19410348	19410506	159	SAT1_CF	blue	na
chrUn	19410572	19411622	1051	SAT1_CF	blue	na
chrUn	19411683	19413420	1738	SAT1_CF	blue	na
chrUn	19413463	19413862	400	SAT1_CF	blue	na
chrUn	19413873	19414382	510	SAT1_CF	blue	na
chrUn	19414413	19416000	1588	SAT1_CF	blue	na
chrUn	19416012	19416575	564	SAT1_CF	blue	na
chrUn	19416593	19416669	77	SAT1_CF	blue	na
chrUn	19416682	19417519	838	SAT1_CF	blue	na
chrUn	19417562	19418086	525	SAT1_CF	blue	na
chrUn	19418120	19418361	242	SAT1_CF	blue	na
chrUn	19418389	19418898	510	SAT1_CF	blue	na
chrUn	19418980	19419542	563	SAT1_CF	blue	na

chrUn	19419570	19420202	633	SAT1_CF	blue	na
chrUn	19420245	19420788	544	SAT1_CF	blue	na
chrUn	19420822	19421769	948	SAT1_CF	blue	na
chrUn	19421797	19423716	1920	SAT1_CF	blue	na
chrUn	19423742	19423899	158	SAT1_CF	blue	na
chrUn	19423914	19425237	1324	SAT1_CF	blue	na
chrUn	19425260	19425731	472	SAT1_CF	blue	na
chrUn	19425805	19425972	168	SAT1_CF	blue	na
chrUn	19426152	19426386	235	SAT1_CF	blue	na
chrUn	19426400	19426942	543	SAT1_CF	blue	na
chrUn	19426964	19427771	808	SAT1_CF	blue	na
chrUn	19427813	19427985	173	SAT1_CF	blue	na
chrUn	19427997	19429367	1371	SAT1_CF	blue	na
chrUn	19429380	19429880	501	SAT1_CF	blue	na
chrUn	19429922	19430880	959	SAT1_CF	blue	na
chrUn	19430937	19433061	2125	SAT1_CF	blue	na
chrUn	19433104	19433921	818	SAT1_CF	blue	na
chrUn	19433963	19434752	790	SAT1_CF	blue	na
chrUn	19434773	19434881	109	SAT1_CF	blue	na
chrUn	19434894	19435597	704	SAT1_CF	blue	na
chrUn	19435649	19436241	593	SAT1_CF	blue	na
chrUn	19436252	19437887	1636	SAT1_CF	blue	na
chrUn	19437930	19438605	676	SAT1_CF	blue	na
chrUn	19438618	19439100	483	SAT1_CF	blue	na
chrUn	19439160	19439527	368	SAT1_CF	blue	na
chrUn	19439570	19440456	887	SAT1_CF	blue	na
chrUn	19440471	19441015	545	SAT1_CF	blue	na
chrUn	19441056	19442673	1618	SAT1_CF	blue	na
chrUn	19442707	19443180	474	SAT1_CF	blue	na
chrUn	19443240	19443641	402	SAT1_CF	blue	na
chrUn	19443652	19444262	611	SAT1_CF	blue	na
chrUn	19444278	19445261	984	SAT1_CF	blue	na
chrUn	19445310	19445855	546	SAT1_CF	blue	na
chrUn	19445868	19445984	117	SAT1_CF	blue	na
chrUn	19445996	19446667	672	SAT1_CF	blue	na
chrUn	19446690	19447191	502	SAT1_CF	blue	na
chrUn	19447257	19447393	137	SAT1_CF	blue	na
chrUn	19447421	19448650	1230	SAT1_CF	blue	na
chrUn	19448694	19450143	1450	SAT1_CF	blue	na
chrUn	19450882	19451153	272	SAT1_CF	blue	na
chrUn	19451180	19451682	503	SAT1_CF	blue	na
chrUn	19451748	19451884	137	SAT1_CF	blue	na

chrUn	19451899	19453580	1682	SAT1_CF	blue	na
chrUn	19453592	19454092	501	SAT1_CF	blue	na
chrUn	19454132	19454533	402	SAT1_CF	blue	na
chrUn	19454547	19455889	1343	SAT1_CF	blue	na
chrUn	19455901	19456590	690	SAT1_CF	blue	na
chrUn	19456603	19457105	503	SAT1_CF	blue	na
chrUn	19457118	19457799	682	SAT1_CF	blue	na
chrUn	19457813	19458375	563	SAT1_CF	blue	na
chrUn	19458402	19459461	1060	SAT1_CF	blue	na
chrUn	19459473	19460028	556	SAT1_CF	blue	na
chrUn	19460049	19460145	97	SAT1_CF	blue	na
chrUn	19460175	19460713	539	SAT1_CF	blue	na
chrUn	19460796	19462822	2027	SAT1_CF	blue	na
chrUn	19462865	19463540	676	SAT1_CF	blue	na
chrUn	19463553	19464053	501	SAT1_CF	blue	na
chrUn	19464098	19464254	157	SAT1_CF	blue	na
chrUn	19464287	19465460	1174	SAT1_CF	blue	na
chrUn	19465483	19465588	106	SAT1_CF	blue	na
chrUn	19465601	19466079	479	SAT1_CF	blue	na
chrUn	19466137	19468174	2038	SAT1_CF	blue	na
chrUn	19468218	19468763	546	SAT1_CF	blue	na
chrUn	19468776	19468892	117	SAT1_CF	blue	na
chrUn	19468904	19469593	690	SAT1_CF	blue	na
chrUn	19469614	19470088	475	SAT1_CF	blue	na
chrUn	19470148	19470635	488	SAT1_CF	blue	na
chrUn	19471256	19473188	1933	SAT1_CF	blue	na
chrUn	19473232	19473906	675	SAT1_CF	blue	na
chrUn	19473919	19474603	685	SAT1_CF	blue	na
chrUn	19474615	19475316	702	SAT1_CF	blue	na
chrUn	19475331	19476601	1271	SAT1_CF	blue	na
chrUn	19476658	19477146	489	SAT1_CF	blue	na
chrUn	19477159	19477696	538	SAT1_CF	blue	na
chrUn	19477738	19478438	701	SAT1_CF	blue	na
chrUn	19478488	19479245	758	SAT1_CF	blue	na
chrUn	19479259	19479779	521	SAT1_CF	blue	na
chrUn	19479847	19480736	890	SAT1_CF	blue	na
chrUn	19480750	19482353	1604	SAT1_CF	blue	na
chrUn	19482384	19482902	519	SAT1_CF	blue	na
chrUn	19483019	19483374	356	SAT1_CF	blue	na
chrUn	19483387	19484798	1412	SAT1_CF	blue	na
chrUn	19484810	19485329	520	SAT1_CF	blue	na
chrUn	19485350	19485415	66	SAT1_CF	blue	na

chrUn	19485435	19486192	758	SAT1_CF	blue	na
chrUn	19486209	19486710	502	SAT1_CF	blue	na
chrUn	19486750	19489159	2410	SAT1_CF	blue	na
chrUn	19489216	19489735	520	SAT1_CF	blue	na
chrUn	19489817	19490131	315	SAT1_CF	blue	na
chrUn	19490143	19490772	630	SAT1_CF	blue	na
chrUn	19490839	19492660	1822	SAT1_CF	blue	na
chrUn	19492675	19493199	525	SAT1_CF	blue	na
chrUn	19493242	19494280	1039	SAT1_CF	blue	na
chrUn	19494322	19495113	792	SAT1_CF	blue	na
chrUn	19495136	19495242	107	SAT1_CF	blue	na
chrUn	19495255	19495775	521	SAT1_CF	blue	na
chrUn	19495801	19495958	158	SAT1_CF	blue	na
chrUn	19495991	19497244	1254	SAT1_CF	blue	na
chrUn	19497308	19499530	2223	SAT1_CF	blue	na
chrUn	19499587	19500541	955	SAT1_CF	blue	na
chrUn	19500586	19501110	525	SAT1_CF	blue	na
chrUn	19501131	19502030	900	SAT1_CF	blue	na
chrUn	19502072	19502931	860	SAT1_CF	blue	na
chrUn	19502943	19503613	671	SAT1_CF	blue	na
chrUn	19503638	19504140	503	SAT1_CF	blue	na
chrUn	19504206	19504342	137	SAT1_CF	blue	na
chrUn	19504357	19504879	523	SAT1_CF	blue	na
chrUn	19504911	19505998	1088	SAT1_CF	blue	na
chrUn	19506075	19507924	1850	SAT1_CF	blue	na
chrUn	19507936	19508538	603	SAT1_CF	blue	na
chrUn	19508558	19509186	629	SAT1_CF	blue	na
chrUn	19509369	19509699	331	SAT1_CF	blue	na
chrUn	19509712	19510232	521	SAT1_CF	blue	na
chrUn	19510299	19511845	1547	SAT1_CF	blue	na
chrUn	19511859	19512398	540	SAT1_CF	blue	na
chrUn	19512441	19513117	677	SAT1_CF	blue	na
chrUn	19513129	19513744	616	SAT1_CF	blue	na
chrUn	19513761	19515158	1398	SAT1_CF	blue	na
chrUn	19515172	19515651	480	SAT1_CF	blue	na
chrUn	19515714	19517091	1378	SAT1_CF	blue	na
chrUn	19517135	19518465	1331	SAT1_CF	blue	na
chrUn	19518477	19519123	647	SAT1_CF	blue	na
chrUn	19519136	19519898	763	SAT1_CF	blue	na
chrUn	19519910	19520438	529	SAT1_CF	blue	na
chrUn	19520461	19520567	107	SAT1_CF	blue	na
chrUn	19520580	19521123	544	SAT1_CF	blue	na

chrUn	19521150	19521970	821	SAT1_CF	blue	na
chrUn	19521995	19522536	542	SAT1_CF	blue	na
chrUn	19522633	19523310	678	SAT1_CF	blue	na
chrUn	19523322	19523877	556	SAT1_CF	blue	na
chrUn	19523898	19524006	109	SAT1_CF	blue	na
chrUn	19524019	19524500	482	SAT1_CF	blue	na
chrUn	19524521	19524919	399	SAT1_CF	blue	na
chrUn	19524932	19526082	1151	SAT1_CF	blue	na
chrUn	19526095	19526576	482	SAT1_CF	blue	na
chrUn	19526597	19526996	400	SAT1_CF	blue	na
chrUn	19527009	19528162	1154	SAT1_CF	blue	na
chrUn	19528175	19528676	502	SAT1_CF	blue	na
chrUn	19528742	19528878	137	SAT1_CF	blue	na
chrUn	19528910	19529415	506	SAT1_CF	blue	na
chrUn	19529447	19530666	1220	SAT1_CF	blue	na
chrUn	19530711	19530868	158	SAT1_CF	blue	na
chrUn	19530883	19531407	525	SAT1_CF	blue	na
chrUn	19531487	19532161	675	SAT1_CF	blue	na
chrUn	19532175	19532728	554	SAT1_CF	blue	na
chrUn	19532751	19532856	106	SAT1_CF	blue	na
chrUn	19532868	19533370	503	SAT1_CF	blue	na
chrUn	19533383	19534591	1209	SAT1_CF	blue	na
chrUn	19534657	19536150	1494	SAT1_CF	blue	na
chrUn	19536163	19536665	503	SAT1_CF	blue	na
chrUn	19536701	19537589	889	SAT1_CF	blue	na
chrUn	19537600	19537826	227	SAT1_CF	blue	na
chrUn	19537906	19538559	654	SAT1_CF	blue	na
chrUn	19538571	19540249	1679	SAT1_CF	blue	na
chrUn	19540265	19541604	1340	SAT1_CF	blue	na
chrUn	19541617	19542119	503	SAT1_CF	blue	na
chrUn	19542165	19542491	327	SAT1_CF	blue	na
chrUn	19542571	19543652	1082	SAT1_CF	blue	na
chrUn	19543674	19544200	527	SAT1_CF	blue	na
chrUn	19544243	19544909	667	SAT1_CF	blue	na
chrUn	19544932	19545542	611	SAT1_CF	blue	na
chrUn	19545553	19546106	554	SAT1_CF	blue	na
chrUn	19546149	19546674	526	SAT1_CF	blue	na
chrUn	19546739	19548106	1368	SAT1_CF	blue	na
chrUn	19548118	19549622	1505	SAT1_CF	blue	na
chrUn	19549640	19550400	761	SAT1_CF	blue	na
chrUn	19550413	19552114	1702	SAT1_CF	blue	na
chrUn	19552375	19553206	832	SAT1_CF	blue	na

chrUn	19553249	19553796	548	SAT1_CF	blue	na
chrUn	19553817	19553926	110	SAT1_CF	blue	na
chrUn	19553938	19554260	323	SAT1_CF	blue	na
chrUn	19554339	19555057	719	SAT1_CF	blue	na
chrUn	19555069	19555751	683	SAT1_CF	blue	na
chrUn	19555765	19556446	682	SAT1_CF	blue	na
chrUn	19556460	19556961	502	SAT1_CF	blue	na
chrUn	19557048	19557375	328	SAT1_CF	blue	na
chrUn	19557413	19557958	546	SAT1_CF	blue	na
chrUn	19558036	19559002	967	SAT1_CF	blue	na
chrUn	19559045	19559719	675	SAT1_CF	blue	na
chrUn	19559731	19560285	555	SAT1_CF	blue	na
chrUn	19560306	19560928	623	SAT1_CF	blue	na
chrUn	19560968	19563536	2569	SAT1_CF	blue	na
chrUn	19563549	19564178	630	SAT1_CF	blue	na
chrUn	19564234	19565937	1704	SAT1_CF	blue	na
chrUn	19565992	19566580	589	SAT1_CF	blue	na
chrUn	19566593	19569509	2917	SAT1_CF	blue	na
chrUn	19569540	19571065	1526	SAT1_CF	blue	na
chrUn	19571114	19571788	675	SAT1_CF	blue	na
chrUn	19571800	19572346	547	SAT1_CF	blue	na
chrUn	19572375	19572616	242	SAT1_CF	blue	na
chrUn	19572644	19573191	548	SAT1_CF	blue	na
chrUn	19573234	19574309	1076	SAT1_CF	blue	na
chrUn	19574352	19575210	859	SAT1_CF	blue	na
chrUn	19575222	19575967	746	SAT1_CF	blue	na
chrUn	19575994	19576495	502	SAT1_CF	blue	na
chrUn	19576540	19576697	158	SAT1_CF	blue	na
chrUn	19576712	19577237	526	SAT1_CF	blue	na
chrUn	19577285	19577950	666	SAT1_CF	blue	na
chrUn	19577976	19578477	502	SAT1_CF	blue	na
chrUn	19578523	19579160	638	SAT1_CF	blue	na
chrUn	19579175	19581909	2735	SAT1_CF	blue	na
chrUn	19581921	19582480	560	SAT1_CF	blue	na
chrUn	19582499	19582607	109	SAT1_CF	blue	na
chrUn	19582620	19583140	521	SAT1_CF	blue	na
chrUn	19583157	19583342	186	SAT1_CF	blue	na
chrUn	19583524	19583689	166	SAT1_CF	blue	na
chrUn	19583731	19584701	971	SAT1_CF	blue	na
chrUn	19586084	19586686	603	CarSat1	red	na
chrUn	19586679	19587555	877	SAT1_CF	blue	na
chrUn	19587785	19588794	1010	Sat2CF	purple	na

chrUn	19592318	19592478	161	SAT1_CF	blue	na
chrUn	19592509	19593047	539	SAT1_CF	blue	na
chrUn	19593090	19594159	1070	SAT1_CF	blue	na
chrUn	19594162	19595402	1241	CarSat1	red	na
chrUn	19595423	19596219	797	CarSat1	red	na
chrUn	19596245	19596958	714	CarSat1	red	na
chrUn	19596976	19597689	714	CarSat1	red	na
chrUn	19597714	19598491	778	CarSat1	red	na
chrUn	19598512	19599959	1448	CarSat1	red	na
chrUn	19600118	19600823	706	CarSat1	red	na
chrUn	19622681	19623615	935	Sat2CF	purple	na
chrUn	19622744	19623534	791	Sat2CF	purple	na
chrUn	19641577	19642315	739	Sat2CF	purple	na
chrUn	19641654	19642659	1006	Sat2CF	purple	na
chrUn	19660637	19661454	818	Sat2CF	purple	na
chrUn	19660711	19661438	728	Sat2CF	purple	na
chrUn	19684299	19684529	231	Sat2CF	purple	na
chrUn	19684772	19684995	224	Sat2CF	purple	na
chrUn	19716811	19717945	1135	Sat2CF	purple	na
chrUn	19716945	19718030	1086	Sat2CF	purple	na
chrUn	19717906	19718055	150	Sat2CF	purple	na
chrUn	19771356	19772563	1208	Sat2CF	purple	na
chrUn	19772434	19772583	150	Sat2CF	purple	na
chrUn	19787224	19788368	1145	Sat2CF	purple	na
chrUn	19787228	19788497	1270	Sat2CF	purple	na
chrUn	19852681	19853932	1252	Sat2CF	purple	na
chrUn	19852811	19853896	1086	Sat2CF	purple	na
chrUn	19856432	19857779	1348	CarSat1	red	na
chrUn	19861657	19862346	690	CarSat1	red	na
chrUn	19862357	19863202	846	CarSat1	red	na
chrUn	19863405	19863995	591	CarSat1	red	na
chrUn	19872820	19873531	712	CarSat1	red	na
chrUn	19873726	19874824	1099	CarSat1	red	na
chrUn	19874853	19875343	491	CarSat1	red	na
chrUn	19875376	19876808	1433	CarSat1	red	na
chrUn	19876833	19878278	1446	CarSat1	red	na
chrUn	19878311	19878477	167	CarSat1	red	na
chrUn	19878671	19879960	1290	CarSat1	red	na
chrUn	19879995	19880585	591	CarSat1	red	na
chrUn	19880767	19881634	868	CarSat1	red	na
chrUn	19881667	19881845	179	CarSat1	red	na
chrUn	19882045	19883532	1488	CarSat1	red	na

chrUn	19883571	19885000	1430	CarSat1	red	na
chrUn	19885038	19886177	1140	CarSat1	red	na
chrUn	19892814	19894034	1221	Sat2CF	purple	na
chrUn	19893617	19893981	365	Sat2CF	purple	na
chrUn	19923513	19924729	1217	Sat2CF	purple	na
chrUn	19923641	19924724	1084	Sat2CF	purple	na
chrUn	19924600	19924749	150	Sat2CF	purple	na
chrUn	19948093	19949175	1083	Sat2CF	purple	na
chrUn	19948224	19949166	943	Sat2CF	purple	na
chrUn	19949051	19949228	178	Sat2CF	purple	na
chrUn	19951686	19951825	140	CarSat1	red	na
chrUn	19951847	19953227	1381	CarSat1	red	na
chrUn	19953224	19953275	52	SAT1_CF	blue	na
chrUn	19953306	19953434	129	SAT1_CF	blue	na
chrUn	19953452	19954314	863	SAT1_CF	blue	na
chrUn	19954509	19955621	1113	Sat2CF	purple	na
chrUn	19954561	19955556	996	Sat2CF	purple	na
chrUn	19961364	19962048	685	CarSat1	red	na
chrUn	19962066	19963578	1513	CarSat1	red	na
chrUn	19963605	19965088	1484	CarSat1	red	na
chrUn	19973653	19975978	2326	CarSat1	red	na
chrUn	19976003	19979409	3407	CarSat1	red	na
chrUn	19979430	19980142	713	CarSat1	red	na
chrUn	19980163	19981127	965	CarSat1	red	na
chrUn	19981153	19981214	62	CarSat1	red	na
chrUn	19981404	19982800	1397	CarSat1	red	na
chrUn	19982826	19984022	1197	CarSat1	red	na
chrUn	19984043	19984755	713	CarSat1	red	na
chrUn	19984780	19986046	1267	CarSat1	red	na
chrUn	19987071	19987871	801	SAT1_CF	blue	na
chrUn	19987883	19988733	851	SAT1_CF	blue	na
chrUn	19988746	19988805	60	SAT1_CF	blue	na
chrUn	19988818	19990638	1821	SAT1_CF	blue	na
chrUn	19990678	19991179	502	SAT1_CF	blue	na
chrUn	19991196	19991953	758	SAT1_CF	blue	na
chrUn	19991966	19992579	614	SAT1_CF	blue	na
chrUn	19992591	19993260	670	SAT1_CF	blue	na
chrUn	19993307	19993528	222	SAT1_CF	blue	na
chrUn	19994976	19996292	1317	SAT1_CF	blue	na
chrUn	19997778	19998308	531	SAT1_CF	blue	na
chrUn	19998376	19998897	522	SAT1_CF	blue	na
chrUn	19998924	19999013	90	SAT1_CF	blue	na

chrUn	19999037	19999640	604	SAT1_CF	blue	na
chrUn	19999662	20000446	785	SAT1_CF	blue	na
chrUn	20000490	20001401	912	SAT1_CF	blue	na
chrUn	20001469	20001988	520	SAT1_CF	blue	na
chrUn	20002001	20002979	979	SAT1_CF	blue	na
chrUn	20002991	20003630	640	SAT1_CF	blue	na
chrUn	20003673	20004198	526	SAT1_CF	blue	na
chrUn	20004213	20004349	137	SAT1_CF	blue	na
chrUn	20004396	20004913	518	SAT1_CF	blue	na
chrUn	20004925	20005596	672	SAT1_CF	blue	na
chrUn	20005639	20006712	1074	SAT1_CF	blue	na
chrUn	20006743	20007254	512	SAT1_CF	blue	na
chrUn	20007297	20007915	619	SAT1_CF	blue	na
chrUn	20007927	20008782	856	SAT1_CF	blue	na
chrUn	20008862	20010670	1809	SAT1_CF	blue	na
chrUn	20010723	20011224	502	SAT1_CF	blue	na
chrUn	20011237	20012752	1516	SAT1_CF	blue	na
chrUn	20012794	20013338	545	SAT1_CF	blue	na
chrUn	20013354	20015064	1711	SAT1_CF	blue	na
chrUn	20015086	20015577	492	SAT1_CF	blue	na
chrUn	20015590	20015753	164	SAT1_CF	blue	na
chrUn	20015792	20016052	261	SAT1_CF	blue	na
chrUn	20016072	20016555	484	SAT1_CF	blue	na
chrUn	20016567	20017252	686	SAT1_CF	blue	na
chrUn	20017270	20017940	671	SAT1_CF	blue	na
chrUn	20017983	20019966	1984	SAT1_CF	blue	na
chrUn	20020073	20020555	483	SAT1_CF	blue	na
chrUn	20020568	20020674	107	SAT1_CF	blue	na
chrUn	20020697	20022700	2004	SAT1_CF	blue	na
chrUn	20022712	20023392	681	SAT1_CF	blue	na
chrUn	20023435	20024806	1372	SAT1_CF	blue	na
chrUn	20024850	20025651	802	SAT1_CF	blue	na
chrUn	20025664	20026267	604	SAT1_CF	blue	na
chrUn	20026279	20027977	1699	SAT1_CF	blue	na
chrUn	20028056	20028707	652	SAT1_CF	blue	na
chrUn	20028719	20029038	320	SAT1_CF	blue	na
chrUn	20029072	20029579	508	SAT1_CF	blue	na
chrUn	20029599	20030243	645	SAT1_CF	blue	na
chrUn	20030288	20030918	631	SAT1_CF	blue	na
chrUn	20030946	20031507	562	SAT1_CF	blue	na
chrUn	20031587	20032095	509	SAT1_CF	blue	na
chrUn	20032123	20032256	134	SAT1_CF	blue	na

chrUn	20032314	20033511	1198	SAT1_CF	blue	na
chrUn	20033523	20034189	667	SAT1_CF	blue	na
chrUn	20034231	20035196	966	SAT1_CF	blue	na
chrUn	20035241	20036193	953	SAT1_CF	blue	na
chrUn	20036217	20036738	522	SAT1_CF	blue	na
chrUn	20036750	20036851	102	SAT1_CF	blue	na
chrUn	20036879	20037434	556	SAT1_CF	blue	na
chrUn	20037446	20037555	110	SAT1_CF	blue	na
chrUn	20037575	20041018	3444	SAT1_CF	blue	na
chrUn	20041061	20041560	500	SAT1_CF	blue	na
chrUn	20041572	20041682	111	SAT1_CF	blue	na
chrUn	20041702	20042227	526	SAT1_CF	blue	na
chrUn	20042284	20042830	547	SAT1_CF	blue	na
chrUn	20042843	20043013	171	SAT1_CF	blue	na
chrUn	20043054	20044900	1847	SAT1_CF	blue	na
chrUn	20044933	20045451	519	SAT1_CF	blue	na
chrUn	20045463	20046324	862	SAT1_CF	blue	na
chrUn	20046366	20047953	1588	SAT1_CF	blue	na
chrUn	20047965	20048793	829	SAT1_CF	blue	na
chrUn	20049037	20050434	1398	SAT1_CF	blue	na
chrUn	20050479	20051026	548	SAT1_CF	blue	na
chrUn	20051066	20051976	911	SAT1_CF	blue	na
chrUn	20052000	20053063	1064	SAT1_CF	blue	na
chrUn	20053094	20053419	326	SAT1_CF	blue	na
chrUn	20054471	20056227	1757	SAT1_CF	blue	na
chrUn	20056344	20056907	564	SAT1_CF	blue	na
chrUn	20056949	20057731	783	SAT1_CF	blue	na
chrUn	20057775	20058319	545	SAT1_CF	blue	na
chrUn	20058332	20058689	358	SAT1_CF	blue	na
chrUn	20058706	20059227	522	SAT1_CF	blue	na
chrUn	20059240	20059913	674	SAT1_CF	blue	na
chrUn	20059961	20061928	1968	SAT1_CF	blue	na
chrUn	20062045	20063873	1829	SAT1_CF	blue	na
chrUn	20063990	20064570	581	SAT1_CF	blue	na
chrUn	20064657	20065157	501	SAT1_CF	blue	na
chrUn	20065170	20065818	649	SAT1_CF	blue	na
chrUn	20065861	20066693	833	SAT1_CF	blue	na
chrUn	20066884	20067596	713	SAT1_CF	blue	na
chrUn	20067676	20068160	485	SAT1_CF	blue	na
chrUn	20068194	20068727	534	SAT1_CF	blue	na
chrUn	20068745	20070395	1651	SAT1_CF	blue	na
chrUn	20070407	20071773	1367	SAT1_CF	blue	na

chrUn	20071800	20073109	1310	SAT1_CF	blue	na
chrUn	20073149	20073651	503	SAT1_CF	blue	na
chrUn	20073678	20073859	182	SAT1_CF	blue	na
chrUn	20073875	20074420	546	SAT1_CF	blue	na
chrUn	20074462	20075006	545	SAT1_CF	blue	na
chrUn	20075022	20075170	149	SAT1_CF	blue	na
chrUn	20075195	20076620	1426	SAT1_CF	blue	na
chrUn	20076632	20077285	654	SAT1_CF	blue	na
chrUn	20077373	20077590	218	SAT1_CF	blue	na
chrUn	20077601	20078144	544	SAT1_CF	blue	na
chrUn	20078155	20078473	319	SAT1_CF	blue	na
chrUn	20078526	20079028	503	SAT1_CF	blue	na
chrUn	20079041	20079144	104	SAT1_CF	blue	na
chrUn	20079170	20079880	711	SAT1_CF	blue	na
chrUn	20079892	20081155	1264	SAT1_CF	blue	na
chrUn	20081205	20082133	929	SAT1_CF	blue	na
chrUn	20082175	20082842	668	SAT1_CF	blue	na
chrUn	20082908	20084092	1185	SAT1_CF	blue	na
chrUn	20084150	20084627	478	SAT1_CF	blue	na
chrUn	20084639	20084744	106	SAT1_CF	blue	na
chrUn	20084767	20085327	561	SAT1_CF	blue	na
chrUn	20085369	20086644	1276	SAT1_CF	blue	na
chrUn	20086682	20087544	863	SAT1_CF	blue	na
chrUn	20087555	20087910	356	SAT1_CF	blue	na
chrUn	20087997	20088498	502	SAT1_CF	blue	na
chrUn	20088525	20089268	744	SAT1_CF	blue	na
chrUn	20089282	20090767	1486	SAT1_CF	blue	na
chrUn	20090844	20092000	1157	SAT1_CF	blue	na
chrUn	20092080	20092604	525	SAT1_CF	blue	na
chrUn	20092619	20092776	158	SAT1_CF	blue	na
chrUn	20092821	20093323	503	SAT1_CF	blue	na
chrUn	20093336	20094665	1330	SAT1_CF	blue	na
chrUn	20094740	20095938	1199	SAT1_CF	blue	na
chrUn	20095966	20096102	137	SAT1_CF	blue	na
chrUn	20096168	20096669	502	SAT1_CF	blue	na
chrUn	20096692	20097363	672	SAT1_CF	blue	na
chrUn	20097375	20097490	116	SAT1_CF	blue	na
chrUn	20097503	20099705	2203	SAT1_CF	blue	na
chrUn	20099716	20100118	403	SAT1_CF	blue	na
chrUn	20100178	20100653	476	SAT1_CF	blue	na
chrUn	20100673	20101434	762	SAT1_CF	blue	na
chrUn	20101446	20103232	1787	SAT1_CF	blue	na

chrUn	20103275	20103638	364	SAT1_CF	blue	na
chrUn	20103665	20104185	521	SAT1_CF	blue	na
chrUn	20104198	20104299	102	SAT1_CF	blue	na
chrUn	20104327	20104901	575	SAT1_CF	blue	na
chrUn	20104913	20106213	1301	SAT1_CF	blue	na
chrUn	20106310	20107006	697	SAT1_CF	blue	na
chrUn	20107071	20107207	137	SAT1_CF	blue	na
chrUn	20107273	20107773	501	SAT1_CF	blue	na
chrUn	20107794	20108467	674	SAT1_CF	blue	na
chrUn	20108479	20109158	680	SAT1_CF	blue	na
chrUn	20109201	20110195	995	SAT1_CF	blue	na
chrUn	20110239	20111480	1242	SAT1_CF	blue	na
chrUn	20111524	20112073	550	SAT1_CF	blue	na
chrUn	20112116	20112746	631	SAT1_CF	blue	na
chrUn	20112773	20113335	563	SAT1_CF	blue	na
chrUn	20113415	20114991	1577	SAT1_CF	blue	na
chrUn	20115003	20116791	1789	SAT1_CF	blue	na
chrUn	20116834	20117036	203	SAT1_CF	blue	na
chrUn	20117217	20117398	182	SAT1_CF	blue	na
chrUn	20117419	20117937	519	SAT1_CF	blue	na
chrUn	20117950	20118055	106	SAT1_CF	blue	na
chrUn	20118078	20119774	1697	SAT1_CF	blue	na
chrUn	20119787	20119892	106	SAT1_CF	blue	na
chrUn	20119915	20120546	632	SAT1_CF	blue	na
chrUn	20120558	20121240	683	SAT1_CF	blue	na
chrUn	20121252	20121851	600	SAT1_CF	blue	na
chrUn	20121871	20122180	310	SAT1_CF	blue	na
chrUn	20122193	20122839	647	SAT1_CF	blue	na
chrUn	20122851	20123666	816	SAT1_CF	blue	na
chrUn	20123696	20124222	527	SAT1_CF	blue	na
chrUn	20124285	20124424	140	SAT1_CF	blue	na
chrUn	20124437	20124932	496	SAT1_CF	blue	na
chrUn	20124952	20126534	1583	SAT1_CF	blue	na
chrUn	20126650	20127250	601	SAT1_CF	blue	na
chrUn	20127394	20127700	307	SAT1_CF	blue	na
chrUn	20127816	20128692	877	SAT1_CF	blue	na
chrUn	20128779	20129280	502	SAT1_CF	blue	na
chrUn	20129293	20129897	605	SAT1_CF	blue	na
chrUn	20129909	20130018	110	SAT1_CF	blue	na
chrUn	20130039	20130670	632	SAT1_CF	blue	na
chrUn	20130683	20131288	606	SAT1_CF	blue	na
chrUn	20131300	20131409	110	SAT1_CF	blue	na

chrUn	20131430	20132059	630	SAT1_CF	blue	na
chrUn	20132073	20133405	1333	SAT1_CF	blue	na
chrUn	20133449	20134416	968	SAT1_CF	blue	na
chrUn	20134427	20134827	401	SAT1_CF	blue	na
chrUn	20134867	20135369	503	SAT1_CF	blue	na
chrUn	20135382	20136057	676	SAT1_CF	blue	na
chrUn	20136100	20137087	988	SAT1_CF	blue	na
chrUn	20137115	20138114	1000	SAT1_CF	blue	na
chrUn	20138149	20140756	2608	SAT1_CF	blue	na
chrUn	20140796	20141296	501	SAT1_CF	blue	na
chrUn	20141309	20142863	1555	SAT1_CF	blue	na
chrUn	20142906	20143529	624	SAT1_CF	blue	na
chrUn	20143616	20143714	99	SAT1_CF	blue	na
chrUn	20143762	20144278	517	SAT1_CF	blue	na
chrUn	20144301	20144967	667	SAT1_CF	blue	na
chrUn	20145009	20145640	632	SAT1_CF	blue	na
chrUn	20145668	20146232	565	SAT1_CF	blue	na
chrUn	20146312	20146837	526	SAT1_CF	blue	na
chrUn	20146852	20147661	810	SAT1_CF	blue	na
chrUn	20147691	20148319	629	SAT1_CF	blue	na
chrUn	20148343	20149204	862	SAT1_CF	blue	na
chrUn	20149222	20149907	686	SAT1_CF	blue	na
chrUn	20149919	20151963	2045	SAT1_CF	blue	na
chrUn	20152006	20152494	489	SAT1_CF	blue	na
chrUn	20152546	20152682	137	SAT1_CF	blue	na
chrUn	20152729	20153334	606	SAT1_CF	blue	na
chrUn	20153389	20153903	515	SAT1_CF	blue	na
chrUn	20153916	20154008	93	SAT1_CF	blue	na
chrUn	20154019	20156650	2632	SAT1_CF	blue	na
chrUn	20156663	20158682	2020	SAT1_CF	blue	na
chrUn	20158761	20159693	933	SAT1_CF	blue	na
chrUn	20159735	20160319	585	SAT1_CF	blue	na
chrUn	20160331	20161927	1597	SAT1_CF	blue	na
chrUn	20161950	20165513	3564	SAT1_CF	blue	na
chrUn	20165553	20166050	498	SAT1_CF	blue	na
chrUn	20166062	20166178	117	SAT1_CF	blue	na
chrUn	20166192	20166717	526	SAT1_CF	blue	na
chrUn	20166774	20167320	547	SAT1_CF	blue	na
chrUn	20167333	20167978	646	SAT1_CF	blue	na
chrUn	20168022	20169191	1170	SAT1_CF	blue	na
chrUn	20169206	20169727	522	SAT1_CF	blue	na
chrUn	20169754	20170346	593	SAT1_CF	blue	na

chrUn	20170358	20170467	110	SAT1_CF	blue	na
chrUn	20170487	20171032	546	SAT1_CF	blue	na
chrUn	20171075	20172451	1377	SAT1_CF	blue	na
chrUn	20172462	20173266	805	SAT1_CF	blue	na
chrUn	20173289	20173835	547	SAT1_CF	blue	na
chrUn	20173882	20175456	1575	SAT1_CF	blue	na
chrUn	20175474	20176145	672	SAT1_CF	blue	na
chrUn	20176188	20177928	1741	SAT1_CF	blue	na
chrUn	20177940	20179537	1598	SAT1_CF	blue	na
chrUn	20179549	20180227	679	SAT1_CF	blue	na
chrUn	20180270	20181225	956	SAT1_CF	blue	na
chrUn	20181341	20182301	961	SAT1_CF	blue	na
chrUn	20182322	20182843	522	SAT1_CF	blue	na
chrUn	20182856	20182962	107	SAT1_CF	blue	na
chrUn	20182987	20185061	2075	SAT1_CF	blue	na
chrUn	20185131	20186331	1201	SAT1_CF	blue	na
chrUn	20186375	20187576	1202	SAT1_CF	blue	na
chrUn	20187591	20187726	136	SAT1_CF	blue	na
chrUn	20187773	20188294	522	SAT1_CF	blue	na
chrUn	20188306	20188412	107	SAT1_CF	blue	na
chrUn	20188436	20189064	629	SAT1_CF	blue	na
chrUn	20189092	20189912	821	SAT1_CF	blue	na
chrUn	20189930	20190615	686	SAT1_CF	blue	na
chrUn	20190627	20191302	676	SAT1_CF	blue	na
chrUn	20191315	20192213	899	SAT1_CF	blue	na
chrUn	20192231	20193165	935	SAT1_CF	blue	na
chrUn	20193207	20193702	496	SAT1_CF	blue	na
chrUn	20193772	20194404	633	SAT1_CF	blue	na
chrUn	20194417	20195030	614	SAT1_CF	blue	na
chrUn	20195042	20196725	1684	SAT1_CF	blue	na
chrUn	20196804	20197517	714	SAT1_CF	blue	na
chrUn	20197564	20198083	520	SAT1_CF	blue	na
chrUn	20198095	20198772	678	SAT1_CF	blue	na
chrUn	20198815	20200471	1657	SAT1_CF	blue	na
chrUn	20200484	20200803	320	SAT1_CF	blue	na
chrUn	20200878	20201357	480	SAT1_CF	blue	na
chrUn	20201384	20202170	787	SAT1_CF	blue	na
chrUn	20202193	20202739	547	SAT1_CF	blue	na
chrUn	20202781	20203037	257	SAT1_CF	blue	na
chrUn	20203145	20203766	622	SAT1_CF	blue	na
chrUn	20203792	20205545	1754	SAT1_CF	blue	na
chrUn	20205558	20205664	107	SAT1_CF	blue	na

chrUn	20205689	20207944	2256	SAT1_CF	blue	na
chrUn	20208026	20208533	508	SAT1_CF	blue	na
chrUn	20208546	20209227	682	SAT1_CF	blue	na
chrUn	20209239	20209921	683	SAT1_CF	blue	na
chrUn	20209932	20210422	491	SAT1_CF	blue	na
chrUn	20210436	20211656	1221	SAT1_CF	blue	na
chrUn	20211683	20212068	386	SAT1_CF	blue	na
chrUn	20212083	20212681	599	SAT1_CF	blue	na
chrUn	20214093	20214698	606	SAT1_CF	blue	na
chrUn	20214890	20215214	325	SAT1_CF	blue	na
chrUn	20215227	20215828	602	SAT1_CF	blue	na
chrUn	20215859	20216147	289	SAT1_CF	blue	na
chrUn	20216167	20216276	110	SAT1_CF	blue	na
chrUn	20216288	20216896	609	SAT1_CF	blue	na
chrUn	20216908	20217409	502	SAT1_CF	blue	na
chrUn	20217454	20218281	828	SAT1_CF	blue	na
chrUn	20218307	20219271	965	SAT1_CF	blue	na
chrUn	20219314	20220553	1240	SAT1_CF	blue	na
chrUn	20220577	20220667	91	SAT1_CF	blue	na
chrUn	20220694	20221273	580	SAT1_CF	blue	na
chrUn	20221357	20222085	729	SAT1_CF	blue	na
chrUn	20222112	20222761	650	SAT1_CF	blue	na
chrUn	20222796	20223479	684	SAT1_CF	blue	na
chrUn	20223492	20223993	502	SAT1_CF	blue	na
chrUn	20224006	20224494	489	SAT1_CF	blue	na
chrUn	20224523	20225151	629	SAT1_CF	blue	na
chrUn	20225194	20225740	547	SAT1_CF	blue	na
chrUn	20225760	20225869	110	SAT1_CF	blue	na
chrUn	20225881	20226423	543	SAT1_CF	blue	na
chrUn	20226466	20227408	943	SAT1_CF	blue	na
chrUn	20227431	20228790	1360	SAT1_CF	blue	na
chrUn	20228803	20228974	172	SAT1_CF	blue	na
chrUn	20229016	20229798	783	SAT1_CF	blue	na
chrUn	20229876	20230865	990	SAT1_CF	blue	na
chrUn	20230913	20231410	498	SAT1_CF	blue	na
chrUn	20231428	20232093	666	SAT1_CF	blue	na
chrUn	20232119	20232579	461	SAT1_CF	blue	na
chrUn	20232656	20233161	506	SAT1_CF	blue	na
chrUn	20233182	20234381	1200	SAT1_CF	blue	na
chrUn	20234393	20235074	682	SAT1_CF	blue	na
chrUn	20235086	20235762	677	SAT1_CF	blue	na
chrUn	20235805	20236912	1108	SAT1_CF	blue	na

chrUn	20236955	20237531	577	SAT1_CF	blue	na
chrUn	20237543	20238696	1154	SAT1_CF	blue	na
chrUn	20238728	20238861	134	SAT1_CF	blue	na
chrUn	20238919	20239421	503	SAT1_CF	blue	na
chrUn	20239462	20240929	1468	SAT1_CF	blue	na
chrUn	20240972	20242081	1110	SAT1_CF	blue	na
chrUn	20242124	20243072	949	SAT1_CF	blue	na
chrUn	20243155	20243674	520	SAT1_CF	blue	na
chrUn	20243731	20244899	1169	SAT1_CF	blue	na
chrUn	20244996	20245726	731	SAT1_CF	blue	na
chrUn	20245806	20248051	2246	SAT1_CF	blue	na
chrUn	20248095	20248637	543	SAT1_CF	blue	na
chrUn	20248650	20249333	684	SAT1_CF	blue	na
chrUn	20249345	20250021	677	SAT1_CF	blue	na
chrUn	20250064	20251030	967	SAT1_CF	blue	na
chrUn	20251109	20251655	547	SAT1_CF	blue	na
chrUn	20251698	20252071	374	SAT1_CF	blue	na
chrUn	20252088	20253215	1128	SAT1_CF	blue	na
chrUn	20253242	20253998	757	SAT1_CF	blue	na
chrUn	20254010	20254119	110	SAT1_CF	blue	na
chrUn	20254140	20255877	1738	SAT1_CF	blue	na
chrUn	20255891	20256573	683	SAT1_CF	blue	na
chrUn	20256585	20257420	836	SAT1_CF	blue	na
chrUn	20257600	20257785	186	SAT1_CF	blue	na
chrUn	20257821	20258323	503	SAT1_CF	blue	na
chrUn	20258336	20259019	684	SAT1_CF	blue	na
chrUn	20259043	20260781	1739	SAT1_CF	blue	na
chrUn	20260860	20261024	165	SAT1_CF	blue	na
chrUn	20261205	20261390	186	SAT1_CF	blue	na
chrUn	20261426	20261925	500	SAT1_CF	blue	na
chrUn	20261938	20262551	614	SAT1_CF	blue	na
chrUn	20262563	20263237	675	SAT1_CF	blue	na
chrUn	20263280	20264392	1113	SAT1_CF	blue	na
chrUn	20264435	20265020	586	SAT1_CF	blue	na
chrUn	20265032	20265745	714	SAT1_CF	blue	na
chrUn	20265769	20265932	164	SAT1_CF	blue	na
chrUn	20265973	20266514	542	SAT1_CF	blue	na
chrUn	20266526	20266635	110	SAT1_CF	blue	na
chrUn	20266654	20267856	1203	SAT1_CF	blue	na
chrUn	20267900	20268181	282	SAT1_CF	blue	na
chrUn	20268250	20268795	546	SAT1_CF	blue	na
chrUn	20268807	20269260	454	SAT1_CF	blue	na

chrUn	20269336	20270386	1051	SAT1_CF	blue	na
chrUn	20270397	20270920	524	SAT1_CF	blue	na
chrUn	20270940	20271550	611	SAT1_CF	blue	na
chrUn	20271593	20271966	374	SAT1_CF	blue	na
chrUn	20271983	20272504	522	SAT1_CF	blue	na
chrUn	20272522	20272616	95	SAT1_CF	blue	na
chrUn	20272646	20273201	556	SAT1_CF	blue	na
chrUn	20273213	20273877	665	SAT1_CF	blue	na
chrUn	20273924	20274041	118	SAT1_CF	blue	na
chrUn	20274083	20275696	1614	SAT1_CF	blue	na
chrUn	20275762	20276263	502	SAT1_CF	blue	na
chrUn	20276275	20276383	109	SAT1_CF	blue	na
chrUn	20276406	20276713	308	SAT1_CF	blue	na
chrUn	20301126	20302110	985	SAT1_CF	blue	na
chrUn	20302122	20303530	1409	SAT1_CF	blue	na
chrUn	20303552	20304217	666	SAT1_CF	blue	na
chrUn	20304261	20305810	1550	SAT1_CF	blue	na
chrUn	20305824	20305931	108	SAT1_CF	blue	na
chrUn	20305953	20307257	1305	SAT1_CF	blue	na
chrUn	20307287	20308149	863	SAT1_CF	blue	na
chrUn	20308182	20309120	939	SAT1_CF	blue	na
chrUn	20309157	20309656	500	SAT1_CF	blue	na
chrUn	20309669	20311358	1690	SAT1_CF	blue	na
chrUn	20311370	20314913	3544	SAT1_CF	blue	na
chrUn	20314926	20316544	1619	SAT1_CF	blue	na
chrUn	20316586	20317367	782	SAT1_CF	blue	na
chrUn	20317399	20318395	997	SAT1_CF	blue	na
chrUn	20318484	20318984	501	SAT1_CF	blue	na
chrUn	20318996	20319599	604	SAT1_CF	blue	na
chrUn	20319611	20321068	1458	SAT1_CF	blue	na
chrUn	20321100	20322096	997	SAT1_CF	blue	na
chrUn	20322116	20322218	103	SAT1_CF	blue	na
chrUn	20322243	20323431	1189	SAT1_CF	blue	na
chrUn	20323502	20324063	562	SAT1_CF	blue	na
chrUn	20324075	20325554	1480	SAT1_CF	blue	na
chrUn	20325596	20326783	1188	SAT1_CF	blue	na
chrUn	20326901	20327419	519	SAT1_CF	blue	na
chrUn	20327476	20328022	547	SAT1_CF	blue	na
chrUn	20328065	20329885	1821	SAT1_CF	blue	na
chrUn	20329925	20330427	503	SAT1_CF	blue	na
chrUn	20330444	20331201	758	SAT1_CF	blue	na
chrUn	20331214	20331827	614	SAT1_CF	blue	na

chrUn	20331839	20332697	859	SAT1_CF	blue	na
chrUn	20332774	20333540	767	SAT1_CF	blue	na
chrUn	20333565	20334107	543	SAT1_CF	blue	na
chrUn	20334129	20334802	674	SAT1_CF	blue	na
chrUn	20334814	20335489	676	SAT1_CF	blue	na
chrUn	20335532	20336497	966	SAT1_CF	blue	na
chrUn	20336576	20337122	547	SAT1_CF	blue	na
chrUn	20337165	20337706	542	SAT1_CF	blue	na
chrUn	20338488	20339152	665	SAT1_CF	blue	na
chrUn	20339164	20339825	662	SAT1_CF	blue	na
chrUn	20339873	20340361	489	SAT1_CF	blue	na
chrUn	20340592	20341050	459	SAT1_CF	blue	na
chrUn	20341092	20341881	790	SAT1_CF	blue	na
chrUn	20341925	20342771	847	SAT1_CF	blue	na
chrUn	20342924	20343453	530	SAT1_CF	blue	na
chrUn	20343495	20344195	701	SAT1_CF	blue	na
chrUn	20344209	20345785	1577	SAT1_CF	blue	na
chrUn	20345798	20346900	1103	SAT1_CF	blue	na
chrUn	20346968	20347949	982	SAT1_CF	blue	na
chrUn	20347997	20349612	1616	SAT1_CF	blue	na
chrUn	20349642	20350149	508	SAT1_CF	blue	na
chrUn	20350164	20350977	814	SAT1_CF	blue	na
chrUn	20351029	20351730	702	SAT1_CF	blue	na
chrUn	20351742	20351848	107	SAT1_CF	blue	na
chrUn	20351871	20352497	627	SAT1_CF	blue	na
chrUn	20352533	20353379	847	SAT1_CF	blue	na
chrUn	20353397	20354083	687	SAT1_CF	blue	na
chrUn	20354095	20354204	110	SAT1_CF	blue	na
chrUn	20354225	20355162	938	SAT1_CF	blue	na
chrUn	20355305	20355773	469	SAT1_CF	blue	na
chrUn	20355817	20356510	694	SAT1_CF	blue	na
chrUn	20356553	20357146	594	SAT1_CF	blue	na
chrUn	20357355	20357687	333	SAT1_CF	blue	na
chrUn	20357700	20360369	2670	SAT1_CF	blue	na
chrUn	20360456	20361653	1198	SAT1_CF	blue	na
chrUn	20361665	20362552	888	SAT1_CF	blue	na
chrUn	20362565	20363247	683	SAT1_CF	blue	na
chrUn	20364278	20364538	261	SAT1_CF	blue	na
chrUn	20364557	20365081	525	SAT1_CF	blue	na
chrUn	20365138	20365684	547	SAT1_CF	blue	na
chrUn	20365727	20366256	530	SAT1_CF	blue	na
chrUn	20366274	20367766	1493	SAT1_CF	blue	na

chrUn	20367787	20368435	649	SAT1_CF	blue	na
chrUn	20368446	20368993	548	SAT1_CF	blue	na
chrUn	20369063	20371397	2335	SAT1_CF	blue	na
chrUn	20371477	20372001	525	SAT1_CF	blue	na
chrUn	20372016	20372172	157	SAT1_CF	blue	na
chrUn	20372198	20372807	610	SAT1_CF	blue	na
chrUn	20372826	20373379	554	SAT1_CF	blue	na
chrUn	20373422	20374385	964	SAT1_CF	blue	na
chrUn	20374412	20376726	2315	SAT1_CF	blue	na
chrUn	20376739	20377287	549	SAT1_CF	blue	na
chrUn	20377307	20378482	1176	SAT1_CF	blue	na
chrUn	20378509	20380080	1572	SAT1_CF	blue	na
chrUn	20380093	20380595	503	SAT1_CF	blue	na
chrUn	20380622	20380712	91	SAT1_CF	blue	na
chrUn	20380736	20382408	1673	SAT1_CF	blue	na
chrUn	20382487	20383024	538	SAT1_CF	blue	na
chrUn	20383041	20383439	399	SAT1_CF	blue	na
chrUn	20383482	20384433	952	SAT1_CF	blue	na
chrUn	20384456	20384554	99	SAT1_CF	blue	na
chrUn	20384574	20385118	545	SAT1_CF	blue	na
chrUn	20385162	20386271	1110	SAT1_CF	blue	na
chrUn	20386316	20387195	880	SAT1_CF	blue	na
chrUn	20387237	20387736	500	SAT1_CF	blue	na
chrUn	20387749	20388423	675	SAT1_CF	blue	na
chrUn	20388466	20389007	542	SAT1_CF	blue	na
chrUn	20389024	20390262	1239	SAT1_CF	blue	na
chrUn	20390309	20390830	522	SAT1_CF	blue	na
chrUn	20390843	20391999	1157	SAT1_CF	blue	na
chrUn	20392042	20392407	366	SAT1_CF	blue	na
chrUn	20393787	20394369	583	SAT1_CF	blue	na
chrUn	20394409	20395528	1120	SAT1_CF	blue	na
chrUn	20395540	20396214	675	SAT1_CF	blue	na
chrUn	20396256	20397236	981	SAT1_CF	blue	na
chrUn	20397280	20397415	136	SAT1_CF	blue	na
chrUn	20397427	20397967	541	SAT1_CF	blue	na
chrUn	20397979	20398296	318	SAT1_CF	blue	na
chrUn	20398371	20398937	567	SAT1_CF	blue	na
chrUn	20398956	20399473	518	SAT1_CF	blue	na
chrUn	20399543	20400060	518	SAT1_CF	blue	na
chrUn	20400128	20400641	514	SAT1_CF	blue	na
chrUn	20400661	20401778	1118	SAT1_CF	blue	na
chrUn	20401792	20403160	1369	SAT1_CF	blue	na

chrUn	20403206	20404163	958	SAT1_CF	blue	na
chrUn	20404242	20405201	960	SAT1_CF	blue	na
chrUn	20405222	20406732	1511	SAT1_CF	blue	na
chrUn	20406775	20407338	564	SAT1_CF	blue	na
chrUn	20407418	20407730	313	SAT1_CF	blue	na
chrUn	20407749	20408291	543	SAT1_CF	blue	na
chrUn	20408362	20408992	631	SAT1_CF	blue	na
chrUn	20409004	20410163	1160	SAT1_CF	blue	na
chrUn	20410183	20411444	1262	SAT1_CF	blue	na
chrUn	20411464	20413045	1582	SAT1_CF	blue	na
chrUn	20413100	20414037	938	SAT1_CF	blue	na
chrUn	20414105	20414624	520	SAT1_CF	blue	na
chrUn	20414637	20415242	606	SAT1_CF	blue	na
chrUn	20415254	20415358	105	SAT1_CF	blue	na
chrUn	20415384	20416013	630	SAT1_CF	blue	na
chrUn	20416027	20417358	1332	SAT1_CF	blue	na
chrUn	20417402	20418781	1380	SAT1_CF	blue	na
chrUn	20418841	20419321	481	SAT1_CF	blue	na
chrUn	20419334	20419442	109	SAT1_CF	blue	na
chrUn	20419463	20420782	1320	SAT1_CF	blue	na
chrUn	20420825	20422852	2028	SAT1_CF	blue	na
chrUn	20422902	20423402	501	SAT1_CF	blue	na
chrUn	20423415	20423523	109	SAT1_CF	blue	na
chrUn	20423543	20424381	839	SAT1_CF	blue	na
chrUn	20424401	20425190	790	SAT1_CF	blue	na
chrUn	20425233	20425954	722	SAT1_CF	blue	na
chrUn	20426136	20426319	184	SAT1_CF	blue	na
chrUn	20426355	20426854	500	SAT1_CF	blue	na
chrUn	20426867	20426973	107	SAT1_CF	blue	na
chrUn	20426996	20428028	1033	SAT1_CF	blue	na
chrUn	20428068	20428566	499	SAT1_CF	blue	na
chrUn	20428578	20430265	1688	SAT1_CF	blue	na
chrUn	20430317	20430474	158	SAT1_CF	blue	na
chrUn	20430519	20431105	587	SAT1_CF	blue	na
chrUn	20431160	20431712	553	SAT1_CF	blue	na
chrUn	20431724	20434418	2695	SAT1_CF	blue	na
chrUn	20434497	20435006	510	SAT1_CF	blue	na
chrUn	20435019	20435374	356	SAT1_CF	blue	na
chrUn	20435461	20435962	502	SAT1_CF	blue	na
chrUn	20435975	20436646	672	SAT1_CF	blue	na
chrUn	20436742	20437284	543	SAT1_CF	blue	na
chrUn	20437327	20438660	1334	SAT1_CF	blue	na

chrUn	20438804	20439297	494	SAT1_CF	blue	na
chrUn	20439328	20439521	194	SAT1_CF	blue	na
chrUn	20439549	20441335	1787	SAT1_CF	blue	na
chrUn	20441388	20441890	503	SAT1_CF	blue	na
chrUn	20441901	20442662	762	SAT1_CF	blue	na
chrUn	20442674	20443348	675	SAT1_CF	blue	na
chrUn	20443391	20443926	536	SAT1_CF	blue	na
chrUn	20443940	20445014	1075	SAT1_CF	blue	na
chrUn	20445055	20446010	956	SAT1_CF	blue	na
chrUn	20446067	20448040	1974	SAT1_CF	blue	na
chrUn	20448081	20448475	395	SAT1_CF	blue	na
chrUn	20448515	20449016	502	SAT1_CF	blue	na
chrUn	20449033	20450014	982	SAT1_CF	blue	na
chrUn	20450028	20450551	524	SAT1_CF	blue	na
chrUn	20450593	20451668	1076	SAT1_CF	blue	na
chrUn	20451711	20452258	548	SAT1_CF	blue	na
chrUn	20452286	20452422	137	SAT1_CF	blue	na
chrUn	20452487	20452988	502	SAT1_CF	blue	na
chrUn	20453011	20453605	595	SAT1_CF	blue	na
chrUn	20453617	20454290	674	SAT1_CF	blue	na
chrUn	20454338	20455224	887	SAT1_CF	blue	na
chrUn	20455258	20455637	380	SAT1_CF	blue	na
chrUn	20455677	20456178	502	SAT1_CF	blue	na
chrUn	20456196	20457696	1501	SAT1_CF	blue	na
chrUn	20457812	20458688	877	SAT1_CF	blue	na
chrUn	20458756	20459276	521	SAT1_CF	blue	na
chrUn	20459288	20459892	605	SAT1_CF	blue	na
chrUn	20459904	20460662	759	SAT1_CF	blue	na
chrUn	20460676	20461353	678	SAT1_CF	blue	na
chrUn	20461396	20462343	948	SAT1_CF	blue	na
chrUn	20462355	20463420	1066	SAT1_CF	blue	na
chrUn	20463441	20464081	641	SAT1_CF	blue	na
chrUn	20464103	20465421	1319	SAT1_CF	blue	na
chrUn	20465464	20466434	971	SAT1_CF	blue	na
chrUn	20466632	20467309	678	SAT1_CF	blue	na
chrUn	20467352	20467718	367	SAT1_CF	blue	na
chrUn	20467778	20468260	483	SAT1_CF	blue	na
chrUn	20468273	20469022	750	SAT1_CF	blue	na
chrUn	20469064	20469845	782	SAT1_CF	blue	na
chrUn	20469912	20470795	884	SAT1_CF	blue	na
chrUn	20470848	20472820	1973	SAT1_CF	blue	na
chrUn	20472862	20473406	545	SAT1_CF	blue	na

chrUn	20473450	20473978	529	SAT1_CF	blue	na
chrUn	20474035	20474509	475	SAT1_CF	blue	na
chrUn	20474536	20475679	1144	SAT1_CF	blue	na
chrUn	20475707	20475864	158	SAT1_CF	blue	na
chrUn	20475890	20476859	970	SAT1_CF	blue	na
chrUn	20476871	20477545	675	SAT1_CF	blue	na
chrUn	20477592	20478544	953	SAT1_CF	blue	na
chrUn	20478623	20479585	963	SAT1_CF	blue	na
chrUn	20479623	20480994	1372	SAT1_CF	blue	na
chrUn	20481007	20481856	850	SAT1_CF	blue	na
chrUn	20481906	20483568	1663	SAT1_CF	blue	na
chrUn	20483580	20484251	672	SAT1_CF	blue	na
chrUn	20484323	20484866	544	SAT1_CF	blue	na
chrUn	20484936	20485411	476	SAT1_CF	blue	na
chrUn	20485423	20486089	667	SAT1_CF	blue	na
chrUn	20486132	20486850	719	SAT1_CF	blue	na
chrUn	20486889	20487452	564	SAT1_CF	blue	na
chrUn	20487465	20488137	673	SAT1_CF	blue	na
chrUn	20488180	20489804	1625	SAT1_CF	blue	na
chrUn	20489836	20490211	376	SAT1_CF	blue	na
chrUn	20490236	20490756	521	SAT1_CF	blue	na
chrUn	20490798	20491110	313	SAT1_CF	blue	na
chrUn	20491152	20491797	646	SAT1_CF	blue	na
chrUn	20491851	20492441	591	SAT1_CF	blue	na
chrUn	20496332	20498301	1970	SAT1_CF	blue	na
chrUn	20498316	20498452	137	SAT1_CF	blue	na
chrUn	20498499	20499020	522	SAT1_CF	blue	na
chrUn	20499042	20501424	2383	SAT1_CF	blue	na
chrUn	20501435	20501927	493	SAT1_CF	blue	na
chrUn	20501941	20503310	1370	SAT1_CF	blue	na
chrUn	20503375	20503937	563	SAT1_CF	blue	na
chrUn	20503980	20504534	555	SAT1_CF	blue	na
chrUn	20504545	20505845	1301	SAT1_CF	blue	na
chrUn	20505888	20506421	534	SAT1_CF	blue	na
chrUn	20506435	20506850	416	SAT1_CF	blue	na
chrUn	20506929	20507888	960	SAT1_CF	blue	na
chrUn	20507928	20508430	503	SAT1_CF	blue	na
chrUn	20508443	20509900	1458	SAT1_CF	blue	na
chrUn	20509943	20510482	540	SAT1_CF	blue	na
chrUn	20510532	20511076	545	SAT1_CF	blue	na
chrUn	20511114	20511971	858	SAT1_CF	blue	na
chrUn	20512009	20512340	332	SAT1_CF	blue	na

chrUn	20512427	20513041	615	SAT1_CF	blue	na
chrUn	20513070	20513614	545	SAT1_CF	blue	na
chrUn	20513658	20514201	544	SAT1_CF	blue	na
chrUn	20514219	20514904	686	SAT1_CF	blue	na
chrUn	20514926	20515763	838	SAT1_CF	blue	na
chrUn	20515776	20516886	1111	SAT1_CF	blue	na
chrUn	20516967	20517491	525	SAT1_CF	blue	na
chrUn	20517506	20518257	752	SAT1_CF	blue	na
chrUn	20518313	20518860	548	SAT1_CF	blue	na
chrUn	20518902	20520676	1775	SAT1_CF	blue	na
chrUn	20520763	20521264	502	SAT1_CF	blue	na
chrUn	20521282	20522121	840	SAT1_CF	blue	na
chrUn	20522144	20522662	519	SAT1_CF	blue	na
chrUn	20522674	20523532	859	SAT1_CF	blue	na
chrUn	20523575	20524365	791	SAT1_CF	blue	na
chrUn	20524409	20524949	541	SAT1_CF	blue	na
chrUn	20524961	20525633	673	SAT1_CF	blue	na
chrUn	20525680	20526312	633	SAT1_CF	blue	na
chrUn	20526340	20526904	565	SAT1_CF	blue	na
chrUn	20526984	20527508	525	SAT1_CF	blue	na
chrUn	20527523	20527680	158	SAT1_CF	blue	na
chrUn	20527725	20528227	503	SAT1_CF	blue	na
chrUn	20528238	20528999	762	SAT1_CF	blue	na
chrUn	20529013	20529814	802	SAT1_CF	blue	na
chrUn	20529827	20530344	518	SAT1_CF	blue	na
chrUn	20530369	20530727	359	SAT1_CF	blue	na
chrUn	20530771	20531319	549	SAT1_CF	blue	na
chrUn	20531332	20531993	662	SAT1_CF	blue	na
chrUn	20532036	20533656	1621	SAT1_CF	blue	na
chrUn	20533672	20534070	399	SAT1_CF	blue	na
chrUn	20534091	20534611	521	SAT1_CF	blue	na
chrUn	20534623	20535953	1331	SAT1_CF	blue	na
chrUn	20535997	20537238	1242	SAT1_CF	blue	na
chrUn	20537949	20538769	821	SAT1_CF	blue	na
chrUn	20538792	20539037	246	SAT1_CF	blue	na
chrUn	20539056	20539577	522	SAT1_CF	blue	na
chrUn	20539595	20541686	2092	SAT1_CF	blue	na
chrUn	20541729	20542359	631	SAT1_CF	blue	na
chrUn	20542389	20542947	559	SAT1_CF	blue	na
chrUn	20543036	20543527	492	SAT1_CF	blue	na
chrUn	20543549	20544230	682	SAT1_CF	blue	na
chrUn	20544242	20546411	2170	SAT1_CF	blue	na

chrUn	20546451	20547648	1198	SAT1_CF	blue	na
chrUn	20547660	20548383	724	SAT1_CF	blue	na
chrUn	20548394	20548886	493	SAT1_CF	blue	na
chrUn	20548900	20550531	1632	SAT1_CF	blue	na
chrUn	20550545	20551109	565	SAT1_CF	blue	na
chrUn	20624420	20624562	143	SAT1_CF	blue	na
chrUn	20624622	20625122	501	SAT1_CF	blue	na
chrUn	20625140	20630036	4897	SAT1_CF	blue	na
chrUn	20630048	20630651	604	SAT1_CF	blue	na
chrUn	20630678	20631266	589	SAT1_CF	blue	na
chrUn	20631293	20631964	672	SAT1_CF	blue	na
chrUn	20632009	20633443	1435	SAT1_CF	blue	na
chrUn	20633496	20633997	502	SAT1_CF	blue	na
chrUn	20634009	20634122	114	SAT1_CF	blue	na
chrUn	20634138	20634684	547	SAT1_CF	blue	na
chrUn	20634727	20635261	535	SAT1_CF	blue	na
chrUn	20635281	20636765	1485	SAT1_CF	blue	na
chrUn	20636786	20637306	521	SAT1_CF	blue	na
chrUn	20637319	20638000	682	SAT1_CF	blue	na
chrUn	20638013	20638675	663	SAT1_CF	blue	na
chrUn	20638718	20639348	631	SAT1_CF	blue	na
chrUn	20639413	20639972	560	SAT1_CF	blue	na
chrUn	20640078	20640560	483	SAT1_CF	blue	na
chrUn	20640587	20641251	665	SAT1_CF	blue	na
chrUn	20641273	20641946	674	SAT1_CF	blue	na
chrUn	20641958	20642842	885	SAT1_CF	blue	na
chrUn	20642864	20643714	851	SAT1_CF	blue	na
chrUn	20643727	20644411	685	SAT1_CF	blue	na
chrUn	20644516	20645061	546	SAT1_CF	blue	na
chrUn	20645104	20646064	961	SAT1_CF	blue	na
chrUn	20646131	20646652	522	SAT1_CF	blue	na
chrUn	20646665	20647069	405	SAT1_CF	blue	na
chrUn	20647139	20648189	1051	SAT1_CF	blue	na
chrUn	20648211	20648310	100	SAT1_CF	blue	na
chrUn	20648331	20649061	731	SAT1_CF	blue	na
chrUn	20649113	20649638	526	SAT1_CF	blue	na
chrUn	20649676	20650948	1273	SAT1_CF	blue	na
chrUn	20650988	20652177	1190	SAT1_CF	blue	na
chrUn	20652220	20652852	633	SAT1_CF	blue	na
chrUn	20652880	20653439	560	SAT1_CF	blue	na
chrUn	20653519	20654068	550	SAT1_CF	blue	na
chrUn	20654096	20654255	160	SAT1_CF	blue	na

chrUn	20654280	20654800	521	SAT1_CF	blue	na
chrUn	20654826	20655488	663	SAT1_CF	blue	na
chrUn	20655501	20655629	129	SAT1_CF	blue	na
chrUn	20655642	20656258	617	SAT1_CF	blue	na
chrUn	20656344	20656679	336	SAT1_CF	blue	na
chrUn	20656760	20657284	525	SAT1_CF	blue	na
chrUn	20657299	20658627	1329	SAT1_CF	blue	na
chrUn	20658641	20659262	622	SAT1_CF	blue	na
chrUn	20659274	20659948	675	SAT1_CF	blue	na
chrUn	20659991	20660529	539	SAT1_CF	blue	na
chrUn	20660543	20661617	1075	SAT1_CF	blue	na
chrUn	20661648	20662494	847	SAT1_CF	blue	na
chrUn	20662506	20663179	674	SAT1_CF	blue	na
chrUn	20663223	20664848	1626	SAT1_CF	blue	na
chrUn	20664862	20665693	832	SAT1_CF	blue	na
chrUn	20665706	20665813	108	SAT1_CF	blue	na
chrUn	20665835	20666409	575	SAT1_CF	blue	na
chrUn	20666421	20667097	677	SAT1_CF	blue	na
chrUn	20667118	20668158	1041	SAT1_CF	blue	na
chrUn	20668176	20668861	686	SAT1_CF	blue	na
chrUn	20668873	20671578	2706	SAT1_CF	blue	na
chrUn	20671721	20672213	493	SAT1_CF	blue	na
chrUn	20672270	20674502	2233	SAT1_CF	blue	na
chrUn	20674517	20674674	158	SAT1_CF	blue	na
chrUn	20674719	20675211	493	SAT1_CF	blue	na
chrUn	20675235	20675991	757	SAT1_CF	blue	na
chrUn	20676003	20677337	1335	SAT1_CF	blue	na
chrUn	20677382	20677687	306	SAT1_CF	blue	na
chrUn	20677767	20678367	601	SAT1_CF	blue	na
chrUn	20678793	20678927	135	SAT1_CF	blue	na
chrUn	20678993	20680180	1188	SAT1_CF	blue	na
chrUn	20680223	20680854	632	SAT1_CF	blue	na
chrUn	20680919	20681477	559	SAT1_CF	blue	na
chrUn	20681525	20682069	545	SAT1_CF	blue	na
chrUn	20682097	20682233	137	SAT1_CF	blue	na
chrUn	20682299	20682797	499	SAT1_CF	blue	na
chrUn	20682820	20683493	674	SAT1_CF	blue	na
chrUn	20683505	20684265	761	SAT1_CF	blue	na
chrUn	20684277	20684949	673	SAT1_CF	blue	na
chrUn	20685019	20685495	477	SAT1_CF	blue	na
chrUn	20685507	20686172	666	SAT1_CF	blue	na
chrUn	20686215	20686742	528	SAT1_CF	blue	na

chrUn	20686763	20687845	1083	SAT1_CF	blue	na
chrUn	20687888	20688230	343	SAT1_CF	blue	na
chrUn	20688314	20688796	483	SAT1_CF	blue	na
chrUn	20688809	20689557	749	SAT1_CF	blue	na
chrUn	20689599	20690222	624	SAT1_CF	blue	na
chrUn	20690252	20690782	531	SAT1_CF	blue	na
chrUn	20690796	20691293	498	SAT1_CF	blue	na
chrUn	20691380	20693937	2558	SAT1_CF	blue	na
chrUn	20693981	20694509	529	SAT1_CF	blue	na
chrUn	20694566	20695656	1091	SAT1_CF	blue	na
chrUn	20695671	20696358	688	SAT1_CF	blue	na
chrUn	20696402	20696946	545	SAT1_CF	blue	na
chrUn	20696973	20697477	505	SAT1_CF	blue	na
chrUn	20697521	20698481	961	SAT1_CF	blue	na
chrUn	20698541	20699471	931	SAT1_CF	blue	na
chrUn	20699483	20700814	1332	SAT1_CF	blue	na
chrUn	20700970	20701505	536	SAT1_CF	blue	na
chrUn	20701527	20702034	508	SAT1_CF	blue	na
chrUn	20702046	20702833	788	SAT1_CF	blue	na
chrUn	20702856	20703363	508	SAT1_CF	blue	na
chrUn	20703376	20704211	836	SAT1_CF	blue	na
chrUn	20704224	20704927	704	SAT1_CF	blue	na
chrUn	20704939	20707067	2129	SAT1_CF	blue	na
chrUn	20707111	20707453	343	SAT1_CF	blue	na
chrUn	20707482	20708378	897	SAT1_CF	blue	na
chrUn	20708418	20708919	502	SAT1_CF	blue	na
chrUn	20708932	20709040	109	SAT1_CF	blue	na
chrUn	20709061	20709616	556	SAT1_CF	blue	na
chrUn	20709628	20710224	597	SAT1_CF	blue	na
chrUn	20710244	20712352	2109	SAT1_CF	blue	na
chrUn	20712395	20713738	1344	SAT1_CF	blue	na
chrUn	20713778	20714279	502	SAT1_CF	blue	na
chrUn	20714293	20714416	124	SAT1_CF	blue	na
chrUn	20714431	20715054	624	SAT1_CF	blue	na
chrUn	20715066	20715902	837	SAT1_CF	blue	na
chrUn	20716079	20716264	186	SAT1_CF	blue	na
chrUn	20716300	20717495	1196	SAT1_CF	blue	na
chrUn	20717513	20718445	933	SAT1_CF	blue	na
chrUn	20718524	20719269	746	SAT1_CF	blue	na
chrUn	20719321	20719959	639	SAT1_CF	blue	na
chrUn	20719988	20720614	627	SAT1_CF	blue	na
chrUn	20720649	20721497	849	SAT1_CF	blue	na

chrUn	20721510	20722193	684	SAT1_CF	blue	na
chrUn	20722205	20724710	2506	SAT1_CF	blue	na
chrUn	20724725	20724882	158	SAT1_CF	blue	na
chrUn	20724927	20725196	270	SAT1_CF	blue	na
chrUn	20725384	20725636	253	SAT1_CF	blue	na
chrUn	20725649	20727019	1371	SAT1_CF	blue	na
chrUn	20727062	20727402	341	SAT1_CF	blue	na
chrUn	20728824	20729341	518	SAT1_CF	blue	na
chrUn	20729428	20729922	495	SAT1_CF	blue	na
chrUn	20729942	20730547	606	SAT1_CF	blue	na
chrUn	20730559	20731234	676	SAT1_CF	blue	na
chrUn	20731276	20733331	2056	SAT1_CF	blue	na
chrUn	20733391	20733961	571	SAT1_CF	blue	na
chrUn	20733980	20734532	553	SAT1_CF	blue	na
chrUn	20734567	20735133	567	SAT1_CF	blue	na
chrUn	20735171	20735665	495	SAT1_CF	blue	na
chrUn	20735690	20736367	678	SAT1_CF	blue	na
chrUn	20736379	20737269	891	SAT1_CF	blue	na
chrUn	20737283	20738651	1369	SAT1_CF	blue	na
chrUn	20738697	20739654	958	SAT1_CF	blue	na
chrUn	20739733	20740281	549	SAT1_CF	blue	na
chrUn	20740294	20742249	1956	SAT1_CF	blue	na
chrUn	20742293	20742840	548	SAT1_CF	blue	na
chrUn	20742882	20743084	203	SAT1_CF	blue	na
chrUn	20743265	20743445	181	SAT1_CF	blue	na
chrUn	20743506	20743978	473	SAT1_CF	blue	na
chrUn	20743991	20744099	109	SAT1_CF	blue	na
chrUn	20744120	20745104	985	SAT1_CF	blue	na
chrUn	20745191	20745692	502	SAT1_CF	blue	na
chrUn	20745710	20746650	941	SAT1_CF	blue	na
chrUn	20746671	20747189	519	SAT1_CF	blue	na
chrUn	20747200	20747602	403	SAT1_CF	blue	na
chrUn	20747642	20748732	1091	SAT1_CF	blue	na
chrUn	20748750	20750074	1325	SAT1_CF	blue	na
chrUn	20750117	20750423	307	SAT1_CF	blue	na
chrUn	20750501	20751008	508	SAT1_CF	blue	na
chrUn	20751022	20751672	651	SAT1_CF	blue	na
chrUn	20751684	20752368	685	SAT1_CF	blue	na
chrUn	20752381	20752983	603	SAT1_CF	blue	na
chrUn	20753010	20753683	674	SAT1_CF	blue	na
chrUn	20753727	20754767	1041	SAT1_CF	blue	na
chrUn	20754846	20755760	915	SAT1_CF	blue	na

chrUn	20755847	20756348	502	SAT1_CF	blue	na
chrUn	20756361	20758461	2101	SAT1_CF	blue	na
chrUn	20758504	20759015	512	SAT1_CF	blue	na
chrUn	20759046	20759178	133	SAT1_CF	blue	na
chrUn	20759225	20760786	1562	SAT1_CF	blue	na
chrUn	20760835	20761429	595	SAT1_CF	blue	na
chrUn	20762825	20763398	574	SAT1_CF	blue	na
chrUn	20763438	20763932	495	SAT1_CF	blue	na
chrUn	20763952	20764631	680	SAT1_CF	blue	na
chrUn	20764654	20765320	667	SAT1_CF	blue	na
chrUn	20765363	20765900	538	SAT1_CF	blue	na
chrUn	20765911	20766987	1077	SAT1_CF	blue	na
chrUn	20767070	20768505	1436	SAT1_CF	blue	na
chrUn	20768547	20768712	166	SAT1_CF	blue	na
chrUn	20768894	20769079	186	SAT1_CF	blue	na
chrUn	20769096	20769614	519	SAT1_CF	blue	na
chrUn	20769627	20769733	107	SAT1_CF	blue	na
chrUn	20769755	20770314	560	SAT1_CF	blue	na
chrUn	20770326	20772073	1748	SAT1_CF	blue	na
chrUn	20772115	20773829	1715	SAT1_CF	blue	na
chrUn	20773906	20774522	617	SAT1_CF	blue	na
chrUn	20774536	20775218	683	SAT1_CF	blue	na
chrUn	20775230	20776562	1333	SAT1_CF	blue	na
chrUn	20776606	20777268	663	SAT1_CF	blue	na
chrUn	20777286	20777798	513	SAT1_CF	blue	na
chrUn	20777826	20778531	706	SAT1_CF	blue	na
chrUn	20778544	20778655	112	SAT1_CF	blue	na
chrUn	20778672	20779913	1242	SAT1_CF	blue	na
chrUn	20779956	20780437	482	SAT1_CF	blue	na
chrUn	20798064	20799229	1166	SAT1_CF	blue	na
chrUn	20799241	20799925	685	SAT1_CF	blue	na
chrUn	20799938	20800440	503	SAT1_CF	blue	na
chrUn	20800527	20802049	1523	SAT1_CF	blue	na
chrUn	20802063	20802607	545	SAT1_CF	blue	na
chrUn	20802651	20802696	46	SAT1_CF	blue	na
chrUn	20802696	20803943	1248	CarSat1	red	na
chrUn	20803964	20804436	473	CarSat1	red	na
chrUn	20804609	20804678	70	CarSat1	red	na
chrUn	20804695	20805003	309	CarSat1	red	na
chrUn	20805152	20805832	681	CarSat1	red	na
chrUn	20806848	20807258	411	CarSat1	red	na
chrUn	20808892	20809093	202	Sat2CF	purple	na

chrUn	20808969	20810050	1082	Sat2CF	purple	na
chrUn	20808978	20809903	926	Sat2CF	purple	na
chrUn	20834178	20834327	150	Sat2CF	purple	na
chrUn	20834198	20835410	1213	Sat2CF	purple	na
chrUn	20884555	20884679	125	Sat2CF	purple	na
chrUn	20884571	20885369	799	Sat2CF	purple	na
chrUn	20904666	20905738	1073	Sat2CF	purple	na
chrUn	20929010	20930184	1175	Sat2CF	purple	na
chrUn	20958025	20959063	1039	Sat2CF	purple	na
chrUn	20958132	20959265	1134	Sat2CF	purple	na
chrUn	20981022	20982230	1209	Sat2CF	purple	na
chrUn	20996320	20997404	1085	Sat2CF	purple	na
chrUn	21129703	21130407	705	CarSat1	red	na
chrUn	21130564	21132011	1448	CarSat1	red	na
chrUn	21132032	21133547	1516	CarSat1	red	na
chrUn	21133565	21135101	1537	CarSat1	red	na
chrUn	21135122	21136713	1592	CarSat1	red	na
chrUn	21136867	21137977	1111	CarSat1	red	na
chrUn	21137980	21139057	1078	SAT1_CF	blue	na
chrUn	21139100	21139645	546	SAT1_CF	blue	na
chrUn	21139676	21139719	44	SAT1_CF	blue	na
chrUn	21139739	21139839	101	SAT1_CF	blue	na
chrUn	21143394	21144401	1008	Sat2CF	purple	na
chrUn	21144629	21145233	605	SAT1_CF	blue	na
chrUn	21145244	21145505	262	SAT1_CF	blue	na
chrUn	21145498	21146230	733	CarSat1	red	na
chrUn	21147930	21148595	666	SAT1_CF	blue	na
chrUn	21148608	21148927	320	SAT1_CF	blue	na
chrUn	21148980	21149481	502	SAT1_CF	blue	na
chrUn	21149493	21150979	1487	SAT1_CF	blue	na
chrUn	21150990	21151392	403	SAT1_CF	blue	na
chrUn	21151432	21152544	1113	SAT1_CF	blue	na
chrUn	21152587	21153731	1145	SAT1_CF	blue	na
chrUn	21153743	21154418	676	SAT1_CF	blue	na
chrUn	21154460	21156168	1709	SAT1_CF	blue	na
chrUn	21156195	21156783	589	SAT1_CF	blue	na
chrUn	21156810	21157483	674	SAT1_CF	blue	na
chrUn	21157497	21158125	629	SAT1_CF	blue	na
chrUn	21158137	21159880	1744	SAT1_CF	blue	na
chrUn	21159920	21160422	503	SAT1_CF	blue	na
chrUn	21160434	21161111	678	SAT1_CF	blue	na
chrUn	21161154	21162153	1000	SAT1_CF	blue	na

chrUn	21162197	21163123	927	SAT1_CF	blue	na
chrUn	21163195	21163696	502	SAT1_CF	blue	na
chrUn	21163710	21164391	682	SAT1_CF	blue	na
chrUn	21164403	21165073	671	SAT1_CF	blue	na
chrUn	21165143	21165619	477	SAT1_CF	blue	na
chrUn	21165631	21165740	110	SAT1_CF	blue	na
chrUn	21165752	21166296	545	SAT1_CF	blue	na
chrUn	21166339	21166970	632	SAT1_CF	blue	na
chrUn	21166998	21167562	565	SAT1_CF	blue	na
chrUn	21167605	21168129	525	SAT1_CF	blue	na
chrUn	21168140	21168300	161	SAT1_CF	blue	na
chrUn	21168345	21168844	500	SAT1_CF	blue	na
chrUn	21168858	21169541	684	SAT1_CF	blue	na
chrUn	21169553	21170404	852	SAT1_CF	blue	na
chrUn	21170443	21171367	925	SAT1_CF	blue	na
chrUn	21171402	21171904	503	SAT1_CF	blue	na
chrUn	21171917	21172023	107	SAT1_CF	blue	na
chrUn	21172045	21172602	558	SAT1_CF	blue	na
chrUn	21172614	21173289	676	SAT1_CF	blue	na
chrUn	21173333	21173964	632	SAT1_CF	blue	na
chrUn	21173991	21175032	1042	SAT1_CF	blue	na
chrUn	21175045	21175843	799	SAT1_CF	blue	na
chrUn	21175869	21176412	544	SAT1_CF	blue	na
chrUn	21176455	21178084	1630	SAT1_CF	blue	na
chrUn	21178127	21178494	368	SAT1_CF	blue	na
chrUn	21178554	21179036	483	SAT1_CF	blue	na
chrUn	21179049	21179380	332	SAT1_CF	blue	na
chrUn	21179397	21179950	554	SAT1_CF	blue	na
chrUn	21179962	21181422	1461	SAT1_CF	blue	na
chrUn	21181453	21181957	505	SAT1_CF	blue	na
chrUn	21181990	21182147	158	SAT1_CF	blue	na
chrUn	21182192	21182693	502	SAT1_CF	blue	na
chrUn	21182705	21183382	678	SAT1_CF	blue	na
chrUn	21183425	21185275	1851	SAT1_CF	blue	na
chrUn	21185302	21185438	137	SAT1_CF	blue	na
chrUn	21185485	21186004	520	SAT1_CF	blue	na
chrUn	21186016	21187350	1335	SAT1_CF	blue	na
chrUn	21187375	21188328	954	SAT1_CF	blue	na
chrUn	21188359	21189205	847	SAT1_CF	blue	na
chrUn	21189217	21189891	675	SAT1_CF	blue	na
chrUn	21189934	21190485	552	SAT1_CF	blue	na
chrUn	21190566	21191772	1207	SAT1_CF	blue	na

chrUn	21191787	21192492	706	SAT1_CF	blue	na
chrUn	21192505	21192612	108	SAT1_CF	blue	na
chrUn	21192634	21193876	1243	SAT1_CF	blue	na
chrUn	21193920	21194548	629	SAT1_CF	blue	na
chrUn	21194576	21195138	563	SAT1_CF	blue	na
chrUn	21195181	21195703	523	SAT1_CF	blue	na
chrUn	21195718	21195875	158	SAT1_CF	blue	na
chrUn	21195920	21196188	269	SAT1_CF	blue	na
chrUn	21196376	21196627	252	SAT1_CF	blue	na
chrUn	21196640	21198009	1370	SAT1_CF	blue	na
chrUn	21198051	21198437	387	SAT1_CF	blue	na
chrUn	21198452	21200008	1557	SAT1_CF	blue	na
chrUn	21200020	21201396	1377	SAT1_CF	blue	na
chrUn	21201409	21202650	1242	SAT1_CF	blue	na
chrUn	21202665	21203284	620	SAT1_CF	blue	na
chrUn	21203308	21204359	1052	SAT1_CF	blue	na
chrUn	21204399	21204901	503	SAT1_CF	blue	na
chrUn	21204928	21205018	91	SAT1_CF	blue	na
chrUn	21205042	21205634	593	SAT1_CF	blue	na
chrUn	21205678	21206714	1037	SAT1_CF	blue	na
chrUn	21206793	21207748	956	SAT1_CF	blue	na
chrUn	21207772	21209445	1674	SAT1_CF	blue	na
chrUn	21209456	21209564	109	SAT1_CF	blue	na
chrUn	21209616	21210136	521	SAT1_CF	blue	na
chrUn	21210154	21211132	979	SAT1_CF	blue	na
chrUn	21211496	21211690	195	SAT1_CF	blue	na
chrUn	21213116	21213721	606	SAT1_CF	blue	na
chrUn	21213733	21214408	676	SAT1_CF	blue	na
chrUn	21214450	21215431	982	SAT1_CF	blue	na
chrUn	21215475	21216162	688	SAT1_CF	blue	na
chrUn	21216174	21216485	312	SAT1_CF	blue	na
chrUn	21216566	21217135	570	SAT1_CF	blue	na
chrUn	21217154	21218300	1147	SAT1_CF	blue	na
chrUn	21218365	21219506	1142	SAT1_CF	blue	na
chrUn	21219541	21220107	567	SAT1_CF	blue	na
chrUn	21220145	21220639	495	SAT1_CF	blue	na
chrUn	21220669	21221339	671	SAT1_CF	blue	na
chrUn	21221351	21222241	891	SAT1_CF	blue	na
chrUn	21222255	21223302	1048	SAT1_CF	blue	na
chrUn	21223857	21224039	183	SAT1_CF	blue	na
chrUn	21224066	21224623	558	SAT1_CF	blue	na
chrUn	21224739	21225287	549	SAT1_CF	blue	na

chrUn	21225300	21227048	1749	SAT1_CF	blue	na
chrUn	21227090	21227272	183	SAT1_CF	blue	na
chrUn	21227298	21228090	793	SAT1_CF	blue	na
chrUn	21228267	21228438	172	SAT1_CF	blue	na
chrUn	21228512	21228982	471	SAT1_CF	blue	na
chrUn	21229005	21229108	104	SAT1_CF	blue	na
chrUn	21229134	21230329	1196	SAT1_CF	blue	na
chrUn	21230344	21230501	158	SAT1_CF	blue	na
chrUn	21230527	21232445	1919	SAT1_CF	blue	na
chrUn	21232471	21233511	1041	SAT1_CF	blue	na
chrUn	21233523	21233688	166	SAT1_CF	blue	na
chrUn	21233871	21234055	185	SAT1_CF	blue	na
chrUn	21234111	21234591	481	SAT1_CF	blue	na
chrUn	21234618	21235363	746	SAT1_CF	blue	na
chrUn	21235375	21236235	861	SAT1_CF	blue	na
chrUn	21236278	21237353	1076	SAT1_CF	blue	na
chrUn	21237396	21237920	525	SAT1_CF	blue	na
chrUn	21237935	21238071	137	SAT1_CF	blue	na
chrUn	21238137	21239325	1189	SAT1_CF	blue	na
chrUn	21239394	21240048	655	SAT1_CF	blue	na
chrUn	21240093	21240593	501	SAT1_CF	blue	na
chrUn	21240620	21241287	668	SAT1_CF	blue	na
chrUn	21241299	21241408	110	SAT1_CF	blue	na
chrUn	21241428	21241973	546	SAT1_CF	blue	na
chrUn	21242016	21242648	633	SAT1_CF	blue	na
chrUn	21242675	21243238	564	SAT1_CF	blue	na
chrUn	21243318	21243632	315	SAT1_CF	blue	na
chrUn	21243651	21244193	543	SAT1_CF	blue	na
chrUn	21244264	21244896	633	SAT1_CF	blue	na
chrUn	21244908	21246067	1160	SAT1_CF	blue	na
chrUn	21246087	21246696	610	SAT1_CF	blue	na
chrUn	21246739	21246941	203	SAT1_CF	blue	na
chrUn	21247122	21247298	177	SAT1_CF	blue	na
chrUn	21247362	21249659	2298	SAT1_CF	blue	na
chrUn	21249671	21249985	315	SAT1_CF	blue	na
chrUn	21250067	21250586	520	SAT1_CF	blue	na
chrUn	21250643	21251190	548	SAT1_CF	blue	na
chrUn	21251232	21251776	545	SAT1_CF	blue	na
chrUn	21251818	21251996	179	SAT1_CF	blue	na
chrUn	21252056	21252604	549	SAT1_CF	blue	na
chrUn	21252618	21252970	353	SAT1_CF	blue	na
chrUn	21253057	21253558	502	SAT1_CF	blue	na

chrUn	21253572	21254329	758	SAT1_CF	blue	na
chrUn	21254341	21254450	110	SAT1_CF	blue	na
chrUn	21254471	21255015	545	SAT1_CF	blue	na
chrUn	21255076	21255766	691	SAT1_CF	blue	na
chrUn	21255780	21257385	1606	SAT1_CF	blue	na
chrUn	21257398	21257505	108	SAT1_CF	blue	na
chrUn	21257527	21258157	631	SAT1_CF	blue	na
chrUn	21258171	21259031	861	SAT1_CF	blue	na
chrUn	21259072	21259616	545	SAT1_CF	blue	na
chrUn	21259665	21259946	282	SAT1_CF	blue	na
chrUn	21259973	21260770	798	SAT1_CF	blue	na
chrUn	21260952	21261086	135	SAT1_CF	blue	na
chrUn	21261154	21261673	520	SAT1_CF	blue	na
chrUn	21261686	21261791	106	SAT1_CF	blue	na
chrUn	21261814	21262907	1094	SAT1_CF	blue	na
chrUn	21262929	21263798	870	SAT1_CF	blue	na
chrUn	21263810	21264495	686	SAT1_CF	blue	na
chrUn	21264508	21266220	1713	SAT1_CF	blue	na
chrUn	21266254	21267155	902	SAT1_CF	blue	na
chrUn	21268568	21269138	571	SAT1_CF	blue	na
chrUn	21269162	21269682	521	SAT1_CF	blue	na
chrUn	21269695	21270376	682	SAT1_CF	blue	na
chrUn	21270388	21271149	762	SAT1_CF	blue	na
chrUn	21271161	21272490	1330	SAT1_CF	blue	na
chrUn	21272503	21272904	402	SAT1_CF	blue	na
chrUn	21272944	21273446	503	SAT1_CF	blue	na
chrUn	21273458	21274427	970	SAT1_CF	blue	na
chrUn	21274442	21276000	1559	SAT1_CF	blue	na
chrUn	21276048	21276828	781	SAT1_CF	blue	na
chrUn	21276883	21278047	1165	SAT1_CF	blue	na
chrUn	21278094	21278596	503	SAT1_CF	blue	na
chrUn	21278609	21280162	1554	SAT1_CF	blue	na
chrUn	21280204	21280748	545	SAT1_CF	blue	na
chrUn	21280764	21282365	1602	SAT1_CF	blue	na
chrUn	21282383	21282490	108	SAT1_CF	blue	na
chrUn	21282507	21282941	435	SAT1_CF	blue	na
chrUn	21282955	21283062	108	SAT1_CF	blue	na
chrUn	21283084	21283364	281	SAT1_CF	blue	na
chrUn	21283547	21283826	280	SAT1_CF	blue	na
chrUn	21283839	21284954	1116	SAT1_CF	blue	na
chrUn	21284972	21285682	711	SAT1_CF	blue	na
chrUn	21285728	21286221	494	SAT1_CF	blue	na

chrUn	21286241	21286916	676	SAT1_CF	blue	na
chrUn	21286959	21288396	1438	SAT1_CF	blue	na
chrUn	21288462	21289644	1183	SAT1_CF	blue	na
chrUn	21289657	21290056	400	SAT1_CF	blue	na
chrUn	21290099	21290601	503	SAT1_CF	blue	na
chrUn	21290614	21292158	1545	SAT1_CF	blue	na
chrUn	21292171	21292680	510	SAT1_CF	blue	na
chrUn	21292693	21293048	356	SAT1_CF	blue	na
chrUn	21293135	21293637	503	SAT1_CF	blue	na
chrUn	21293650	21294322	673	SAT1_CF	blue	na
chrUn	21294369	21294486	118	SAT1_CF	blue	na
chrUn	21294528	21296337	1810	SAT1_CF	blue	na
chrUn	21296454	21296972	519	SAT1_CF	blue	na
chrUn	21297004	21297198	195	SAT1_CF	blue	na
chrUn	21297226	21298079	854	SAT1_CF	blue	na
chrUn	21298745	21298985	241	SAT1_CF	blue	na
chrUn	21299053	21299573	521	SAT1_CF	blue	na
chrUn	21299585	21300918	1334	SAT1_CF	blue	na
chrUn	21300962	21302078	1117	SAT1_CF	blue	na
chrUn	21302131	21303637	1507	SAT1_CF	blue	na
chrUn	21303660	21303905	246	SAT1_CF	blue	na
chrUn	21303924	21304445	522	SAT1_CF	blue	na
chrUn	21304463	21305062	600	SAT1_CF	blue	na
chrUn	21305101	21306553	1453	SAT1_CF	blue	na
chrUn	21306596	21307486	891	SAT1_CF	blue	na
chrUn	21307529	21308635	1107	SAT1_CF	blue	na
chrUn	21308656	21309786	1131	SAT1_CF	blue	na
chrUn	21309806	21311256	1451	SAT1_CF	blue	na
chrUn	21311353	21311895	543	SAT1_CF	blue	na
chrUn	21311939	21312499	561	SAT1_CF	blue	na
chrUn	21312542	21313066	525	SAT1_CF	blue	na
chrUn	21313081	21313217	137	SAT1_CF	blue	na
chrUn	21313302	21313782	481	SAT1_CF	blue	na
chrUn	21313795	21314476	682	SAT1_CF	blue	na
chrUn	21314500	21315165	666	SAT1_CF	blue	na
chrUn	21315209	21315840	632	SAT1_CF	blue	na
chrUn	21315852	21316570	719	SAT1_CF	blue	na
chrUn	21316598	21316971	374	SAT1_CF	blue	na
chrUn	21316983	21317092	110	SAT1_CF	blue	na
chrUn	21317113	21317659	547	SAT1_CF	blue	na
chrUn	21317702	21318533	832	SAT1_CF	blue	na
chrUn	21318723	21319730	1008	SAT1_CF	blue	na

chrUn	21319754	21320489	736	SAT1_CF	blue	na
chrUn	21320502	21321262	761	SAT1_CF	blue	na
chrUn	21321280	21322929	1650	SAT1_CF	blue	na
chrUn	21322941	21324307	1367	SAT1_CF	blue	na
chrUn	21324372	21326074	1703	SAT1_CF	blue	na
chrUn	21326097	21326763	667	SAT1_CF	blue	na
chrUn	21326806	21327332	527	SAT1_CF	blue	na
chrUn	21327354	21328436	1083	SAT1_CF	blue	na
chrUn	21328516	21328841	326	SAT1_CF	blue	na
chrUn	21328887	21329388	502	SAT1_CF	blue	na
chrUn	21329401	21329991	591	SAT1_CF	blue	na
chrUn	21330019	21330581	563	SAT1_CF	blue	na
chrUn	21330593	21331856	1264	SAT1_CF	blue	na
chrUn	21331892	21332724	833	SAT1_CF	blue	na
chrUn	21332736	21333585	850	SAT1_CF	blue	na
chrUn	21333674	21334860	1187	SAT1_CF	blue	na
chrUn	21335130	21335651	522	SAT1_CF	blue	na
chrUn	21335678	21335746	69	SAT1_CF	blue	na
chrUn	21335758	21336310	553	SAT1_CF	blue	na
chrUn	21336353	21336843	491	SAT1_CF	blue	na
chrUn	21336909	21337429	521	SAT1_CF	blue	na
chrUn	21337443	21338093	651	SAT1_CF	blue	na
chrUn	21338115	21338866	752	SAT1_CF	blue	na
chrUn	21338879	21339482	604	SAT1_CF	blue	na
chrUn	21339707	21339862	156	SAT1_CF	blue	na
chrUn	21339927	21340525	599	SAT1_CF	blue	na
chrUn	21340548	21341055	508	SAT1_CF	blue	na
chrUn	21341069	21341613	545	SAT1_CF	blue	na
chrUn	21341634	21344212	2579	SAT1_CF	blue	na
chrUn	21344256	21344706	451	SAT1_CF	blue	na
chrUn	21344763	21345237	475	SAT1_CF	blue	na
chrUn	21345264	21345854	591	SAT1_CF	blue	na
chrUn	21345868	21346021	154	SAT1_CF	blue	na
chrUn	21346082	21346607	526	SAT1_CF	blue	na
chrUn	21346622	21346779	158	SAT1_CF	blue	na
chrUn	21346844	21347774	931	SAT1_CF	blue	na
chrUn	21347792	21348461	670	SAT1_CF	blue	na
chrUn	21348509	21349466	958	SAT1_CF	blue	na
chrUn	21349545	21350507	963	SAT1_CF	blue	na
chrUn	21350545	21351914	1370	SAT1_CF	blue	na
chrUn	21351927	21355135	3209	SAT1_CF	blue	na
chrUn	21355205	21355678	474	SAT1_CF	blue	na

chrUn	21355690	21356356	667	SAT1_CF	blue	na
chrUn	21356399	21357116	718	SAT1_CF	blue	na
chrUn	21357155	21357718	564	SAT1_CF	blue	na
chrUn	21357731	21358403	673	SAT1_CF	blue	na
chrUn	21358446	21360070	1625	SAT1_CF	blue	na
chrUn	21360102	21360467	366	SAT1_CF	blue	na
chrUn	21360501	21361020	520	SAT1_CF	blue	na
chrUn	21361062	21361374	313	SAT1_CF	blue	na
chrUn	21361416	21362688	1273	SAT1_CF	blue	na
chrUn	21364107	21364667	561	SAT1_CF	blue	na
chrUn	21364720	21365214	495	SAT1_CF	blue	na
chrUn	21365243	21366601	1359	SAT1_CF	blue	na
chrUn	21366644	21367275	632	SAT1_CF	blue	na
chrUn	21367303	21367978	676	SAT1_CF	blue	na
chrUn	21368180	21368626	447	SAT1_CF	blue	na
chrUn	21368660	21369181	522	SAT1_CF	blue	na
chrUn	21369203	21370952	1750	SAT1_CF	blue	na
chrUn	21370978	21371584	607	SAT1_CF	blue	na
chrUn	21371595	21372087	493	SAT1_CF	blue	na
chrUn	21372101	21373466	1366	SAT1_CF	blue	na
chrUn	21373482	21373760	279	SAT1_CF	blue	na
chrUn	21373804	21374352	549	SAT1_CF	blue	na
chrUn	21374406	21375058	653	SAT1_CF	blue	na
chrUn	21375069	21375679	611	SAT1_CF	blue	na
chrUn	21375702	21376368	667	SAT1_CF	blue	na
chrUn	21376411	21377515	1105	SAT1_CF	blue	na
chrUn	21377526	21378069	544	SAT1_CF	blue	na
chrUn	21378080	21378398	319	SAT1_CF	blue	na
chrUn	21378451	21378953	503	SAT1_CF	blue	na
chrUn	21378966	21379727	762	SAT1_CF	blue	na
chrUn	21379739	21381009	1271	SAT1_CF	blue	na
chrUn	21381051	21381594	544	SAT1_CF	blue	na
chrUn	21381762	21382765	1004	SAT1_CF	blue	na
chrUn	21382842	21383386	545	SAT1_CF	blue	na
chrUn	21383399	21383505	107	SAT1_CF	blue	na
chrUn	21383528	21383906	379	SAT1_CF	blue	na
chrUn	21383993	21384495	503	SAT1_CF	blue	na
chrUn	21384520	21385189	670	SAT1_CF	blue	na
chrUn	21385201	21385875	675	SAT1_CF	blue	na
chrUn	21385893	21386059	167	SAT1_CF	blue	na
chrUn	21386101	21387905	1805	SAT1_CF	blue	na
chrUn	21387992	21388604	613	SAT1_CF	blue	na

chrUn	21388635	21389179	545	SAT1_CF	blue	na
chrUn	21389223	21389766	544	SAT1_CF	blue	na
chrUn	21389784	21390463	680	SAT1_CF	blue	na
chrUn	21390495	21390776	282	SAT1_CF	blue	na
chrUn	21390956	21391231	276	SAT1_CF	blue	na
chrUn	21391274	21392000	727	SAT1_CF	blue	na
chrUn	21392045	21392580	536	SAT1_CF	blue	na
chrUn	21392602	21393120	519	SAT1_CF	blue	na
chrUn	21393132	21394490	1359	SAT1_CF	blue	na
chrUn	21394502	21394805	304	SAT1_CF	blue	na
chrUn	21401141	21402524	1384	SAT1_CF	blue	na
chrUn	21402545	21403228	684	SAT1_CF	blue	na
chrUn	21403240	21404211	972	SAT1_CF	blue	na
chrUn	21404255	21404810	556	SAT1_CF	blue	na
chrUn	21404861	21405371	511	SAT1_CF	blue	na
chrUn	21405399	21405556	158	SAT1_CF	blue	na
chrUn	21405601	21406102	502	SAT1_CF	blue	na
chrUn	21406115	21406219	105	SAT1_CF	blue	na
chrUn	21406243	21407669	1427	SAT1_CF	blue	na
chrUn	21407746	21408253	508	SAT1_CF	blue	na
chrUn	21408288	21409153	866	SAT1_CF	blue	na
chrUn	21409166	21409555	390	SAT1_CF	blue	na
chrUn	21409608	21411665	2058	SAT1_CF	blue	na
chrUn	21411689	21412746	1058	SAT1_CF	blue	na
chrUn	21412760	21413972	1213	SAT1_CF	blue	na
chrUn	21413983	21414471	489	SAT1_CF	blue	na
chrUn	21414539	21414868	330	SAT1_CF	blue	na
chrUn	21415090	21415175	86	SAT1_CF	blue	na
chrUn	21416593	21417209	617	SAT1_CF	blue	na
chrUn	21417232	21417778	547	SAT1_CF	blue	na
chrUn	21417825	21418180	356	SAT1_CF	blue	na
chrUn	21418289	21418769	481	SAT1_CF	blue	na
chrUn	21418787	21419429	643	SAT1_CF	blue	na
chrUn	21419441	21420117	677	SAT1_CF	blue	na
chrUn	21420160	21421492	1333	SAT1_CF	blue	na
chrUn	21421532	21422519	988	SAT1_CF	blue	na
chrUn	21422563	21423483	921	SAT1_CF	blue	na
chrUn	21423523	21424060	538	SAT1_CF	blue	na
chrUn	21424087	21424679	593	SAT1_CF	blue	na
chrUn	21424691	21425366	676	SAT1_CF	blue	na
chrUn	21425409	21427442	2034	SAT1_CF	blue	na
chrUn	21427488	21428649	1162	SAT1_CF	blue	na

chrUn	21428691	21430293	1603	SAT1_CF	blue	na
chrUn	21430372	21431917	1546	SAT1_CF	blue	na
chrUn	21431931	21432614	684	SAT1_CF	blue	na
chrUn	21432626	21432735	110	SAT1_CF	blue	na
chrUn	21432755	21434352	1598	SAT1_CF	blue	na
chrUn	21434373	21435927	1555	SAT1_CF	blue	na
chrUn	21435969	21436667	699	SAT1_CF	blue	na
chrUn	21436681	21437243	563	SAT1_CF	blue	na
chrUn	21438665	21439225	561	SAT1_CF	blue	na
chrUn	21439278	21441162	1885	SAT1_CF	blue	na
chrUn	21441204	21442833	1630	SAT1_CF	blue	na
chrUn	21442876	21443398	523	SAT1_CF	blue	na
chrUn	21443413	21443549	137	SAT1_CF	blue	na
chrUn	21443616	21444117	502	SAT1_CF	blue	na
chrUn	21444129	21444813	685	SAT1_CF	blue	na
chrUn	21444825	21446157	1333	SAT1_CF	blue	na
chrUn	21446182	21446425	244	SAT1_CF	blue	na
chrUn	21446439	21446948	510	SAT1_CF	blue	na
chrUn	21446960	21447076	117	SAT1_CF	blue	na
chrUn	21447089	21447944	856	SAT1_CF	blue	na
chrUn	21447976	21448864	889	SAT1_CF	blue	na
chrUn	21448951	21449451	501	SAT1_CF	blue	na
chrUn	21449469	21450146	678	SAT1_CF	blue	na
chrUn	21450158	21450267	110	SAT1_CF	blue	na
chrUn	21450288	21450764	477	SAT1_CF	blue	na
chrUn	21450776	21450878	103	SAT1_CF	blue	na
chrUn	21450907	21451452	546	SAT1_CF	blue	na
chrUn	21451465	21453289	1825	SAT1_CF	blue	na
chrUn	21453356	21453857	502	SAT1_CF	blue	na
chrUn	21453870	21454476	607	SAT1_CF	blue	na
chrUn	21454488	21455165	678	SAT1_CF	blue	na
chrUn	21455177	21455350	174	SAT1_CF	blue	na
chrUn	21455427	21458226	2800	SAT1_CF	blue	na
chrUn	21458239	21458922	684	SAT1_CF	blue	na
chrUn	21458934	21460120	1187	SAT1_CF	blue	na
chrUn	21460134	21462748	2615	SAT1_CF	blue	na
chrUn	21462763	21462920	158	SAT1_CF	blue	na
chrUn	21462985	21464498	1514	SAT1_CF	blue	na
chrUn	21464542	21465269	728	SAT1_CF	blue	na
chrUn	21465312	21465982	671	SAT1_CF	blue	na
chrUn	21465995	21466955	961	SAT1_CF	blue	na
chrUn	21466998	21468423	1426	SAT1_CF	blue	na

chrUn	21468491	21468730	240	SAT1_CF	blue	na
chrUn	21468760	21469268	509	SAT1_CF	blue	na
chrUn	21469296	21470654	1359	SAT1_CF	blue	na
chrUn	21471658	21472080	423	SAT1_CF	blue	na
chrUn	21472147	21472208	62	SAT1_CF	blue	na
chrUn	21472221	21473156	936	SAT1_CF	blue	na
chrUn	21473196	21474416	1221	SAT1_CF	blue	na
chrUn	21474448	21474969	522	SAT1_CF	blue	na
chrUn	21474984	21475141	158	SAT1_CF	blue	na
chrUn	21475167	21475688	522	SAT1_CF	blue	na
chrUn	21475701	21475807	107	SAT1_CF	blue	na
chrUn	21475830	21477906	2077	SAT1_CF	blue	na
chrUn	21477976	21479223	1248	SAT1_CF	blue	na
chrUn	21479234	21479727	494	SAT1_CF	blue	na
chrUn	21479740	21480342	603	SAT1_CF	blue	na
chrUn	21480354	21481029	676	SAT1_CF	blue	na
chrUn	21481072	21482574	1503	SAT1_CF	blue	na
chrUn	21483974	21485024	1051	SAT1_CF	blue	na
chrUn	21485039	21485566	528	SAT1_CF	blue	na
chrUn	21485586	21486889	1304	SAT1_CF	blue	na
chrUn	21486922	21487271	350	SAT1_CF	blue	na
chrUn	21487327	21488523	1197	SAT1_CF	blue	na
chrUn	21488535	21489216	682	SAT1_CF	blue	na
chrUn	21489227	21489719	493	SAT1_CF	blue	na
chrUn	21489733	21491365	1633	SAT1_CF	blue	na
chrUn	21491380	21491941	562	SAT1_CF	blue	na
chrUn	21493358	21493958	601	SAT1_CF	blue	na
chrUn	21493971	21494734	764	SAT1_CF	blue	na
chrUn	21495185	21495372	188	SAT1_CF	blue	na
chrUn	21495392	21495501	110	SAT1_CF	blue	na
chrUn	21495513	21496121	609	SAT1_CF	blue	na
chrUn	21496133	21496633	501	SAT1_CF	blue	na
chrUn	21496678	21497504	827	SAT1_CF	blue	na
chrUn	21497530	21497946	417	SAT1_CF	blue	na
chrUn	21498269	21499689	1421	SAT1_CF	blue	na
chrUn	21499713	21499803	91	SAT1_CF	blue	na
chrUn	21499830	21500409	580	SAT1_CF	blue	na
chrUn	21500489	21501241	753	SAT1_CF	blue	na
chrUn	21502145	21502805	661	SAT1_CF	blue	na
chrUn	21502818	21503319	502	SAT1_CF	blue	na
chrUn	21503332	21503820	489	SAT1_CF	blue	na
chrUn	21503864	21505066	1203	SAT1_CF	blue	na

chrUn	21505086	21505156	71	SAT1_CF	blue	na
chrUn	21505683	21505926	244	SAT1_CF	blue	na
chrUn	21505959	21506735	777	SAT1_CF	blue	na
chrUn	21506748	21508119	1372	SAT1_CF	blue	na
chrUn	21508162	21509862	1701	SAT1_CF	blue	na
chrUn	21509874	21510549	676	SAT1_CF	blue	na
chrUn	21510565	21511360	796	SAT1_CF	blue	na
chrUn	21511372	21513134	1763	SAT1_CF	blue	na
chrUn	21513178	21513724	547	SAT1_CF	blue	na
chrUn	21513794	21515852	2059	SAT1_CF	blue	na
chrUn	21515863	21516255	393	SAT1_CF	blue	na
chrUn	21516301	21516793	493	SAT1_CF	blue	na
chrUn	21516813	21516919	107	SAT1_CF	blue	na
chrUn	21516941	21517582	642	SAT1_CF	blue	na
chrUn	21517601	21519265	1665	SAT1_CF	blue	na
chrUn	21519308	21519893	586	SAT1_CF	blue	na
chrUn	21519905	21521502	1598	SAT1_CF	blue	na
chrUn	21521514	21522930	1417	SAT1_CF	blue	na
chrUn	21522973	21523612	640	SAT1_CF	blue	na
chrUn	21523624	21524386	763	SAT1_CF	blue	na
chrUn	21524398	21526483	2086	SAT1_CF	blue	na
chrUn	21526545	21527086	542	SAT1_CF	blue	na
chrUn	21527173	21527675	503	SAT1_CF	blue	na
chrUn	21527687	21527793	107	SAT1_CF	blue	na
chrUn	21527817	21528445	629	SAT1_CF	blue	na
chrUn	21528469	21528916	448	SAT1_CF	blue	na
chrUn	21528933	21529088	156	SAT1_CF	blue	na
chrUn	21529118	21529643	526	SAT1_CF	blue	na
chrUn	21529658	21529794	137	SAT1_CF	blue	na
chrUn	21529890	21530409	520	SAT1_CF	blue	na
chrUn	21530466	21531634	1169	SAT1_CF	blue	na
chrUn	21531731	21532827	1097	SAT1_CF	blue	na
chrUn	21532916	21533414	499	SAT1_CF	blue	na
chrUn	21533431	21534110	680	SAT1_CF	blue	na
chrUn	21534123	21534736	614	SAT1_CF	blue	na
chrUn	21534748	21535607	860	SAT1_CF	blue	na
chrUn	21535650	21537939	2290	SAT1_CF	blue	na
chrUn	21537952	21538028	77	SAT1_CF	blue	na
chrUn	21538081	21538577	497	SAT1_CF	blue	na
chrUn	21538589	21539438	850	SAT1_CF	blue	na
chrUn	21539488	21540928	1441	SAT1_CF	blue	na
chrUn	21540986	21541617	632	SAT1_CF	blue	na

chrUn	21541629	21542002	374	SAT1_CF	blue	na
chrUn	21542187	21542872	686	SAT1_CF	blue	na
chrUn	21542886	21544642	1757	SAT1_CF	blue	na
chrUn	21544653	21545221	569	SAT1_CF	blue	na
chrUn	21545234	21545917	684	SAT1_CF	blue	na
chrUn	21545997	21547239	1243	SAT1_CF	blue	na
chrUn	21547257	21547934	678	SAT1_CF	blue	na
chrUn	21547946	21548624	679	SAT1_CF	blue	na
chrUn	21548666	21549517	852	SAT1_CF	blue	na
chrUn	21549587	21550132	546	SAT1_CF	blue	na
chrUn	21550175	21551057	883	SAT1_CF	blue	na
chrUn	21551071	21551616	546	SAT1_CF	blue	na
chrUn	21551658	21552212	555	SAT1_CF	blue	na
chrUn	21552250	21552750	501	SAT1_CF	blue	na
chrUn	21552763	21553446	684	SAT1_CF	blue	na
chrUn	21553458	21554442	985	SAT1_CF	blue	na
chrUn	21554469	21555479	1011	SAT1_CF	blue	na
chrUn	21555497	21556434	938	SAT1_CF	blue	na
chrUn	21556472	21556975	504	SAT1_CF	blue	na
chrUn	21556993	21557677	685	SAT1_CF	blue	na
chrUn	21557689	21559124	1436	SAT1_CF	blue	na
chrUn	21559136	21559854	719	SAT1_CF	blue	na
chrUn	21559875	21560763	889	SAT1_CF	blue	na
chrUn	21560850	21561967	1118	SAT1_CF	blue	na
chrUn	21561981	21563535	1555	SAT1_CF	blue	na
chrUn	21563556	21564917	1362	SAT1_CF	blue	na
chrUn	21564960	21565923	964	SAT1_CF	blue	na
chrUn	21566003	21566929	927	SAT1_CF	blue	na
chrUn	21566965	21567465	501	SAT1_CF	blue	na
chrUn	21567483	21568530	1048	SAT1_CF	blue	na
chrUn	21568551	21569093	543	SAT1_CF	blue	na
chrUn	21569163	21570364	1202	SAT1_CF	blue	na
chrUn	21570408	21570748	341	SAT1_CF	blue	na
chrUn	21570793	21571646	854	SAT1_CF	blue	na
chrUn	21571676	21572634	959	SAT1_CF	blue	na
chrUn	21572684	21574175	1492	SAT1_CF	blue	na
chrUn	21574204	21575372	1169	SAT1_CF	blue	na
chrUn	21575396	21575649	254	SAT1_CF	blue	na
chrUn	21575714	21576195	482	SAT1_CF	blue	na
chrUn	21576208	21577432	1225	SAT1_CF	blue	na
chrUn	21577444	21578087	644	SAT1_CF	blue	na
chrUn	21578131	21578950	820	SAT1_CF	blue	na

chrUn	21579015	21579464	450	SAT1_CF	blue	na
chrUn	21579551	21580051	501	SAT1_CF	blue	na
chrUn	21580079	21581315	1237	SAT1_CF	blue	na
chrUn	21581363	21581887	525	SAT1_CF	blue	na
chrUn	21581902	21582652	751	SAT1_CF	blue	na
chrUn	21582709	21583255	547	SAT1_CF	blue	na
chrUn	21583277	21583842	566	SAT1_CF	blue	na
chrUn	21583884	21585070	1187	SAT1_CF	blue	na
chrUn	21585138	21585658	521	SAT1_CF	blue	na
chrUn	21585685	21586431	747	SAT1_CF	blue	na
chrUn	21586443	21587301	859	SAT1_CF	blue	na
chrUn	21587372	21588787	1416	SAT1_CF	blue	na
chrUn	21588800	21589633	834	SAT1_CF	blue	na
chrUn	21589646	21590345	700	SAT1_CF	blue	na
chrUn	21590357	21591040	684	SAT1_CF	blue	na
chrUn	21591051	21591543	493	SAT1_CF	blue	na
chrUn	21591557	21592770	1214	SAT1_CF	blue	na
chrUn	21592781	21593231	451	SAT1_CF	blue	na
chrUn	21596644	21597462	819	SAT1_CF	blue	na
chrUn	21597474	21597640	167	SAT1_CF	blue	na
chrUn	21597822	21598028	207	SAT1_CF	blue	na
chrUn	21598062	21598538	477	SAT1_CF	blue	na
chrUn	21598551	21600218	1668	SAT1_CF	blue	na
chrUn	21600254	21601111	858	SAT1_CF	blue	na
chrUn	21601151	21601651	501	SAT1_CF	blue	na
chrUn	21601678	21602305	628	SAT1_CF	blue	na
chrUn	21602382	21602927	546	SAT1_CF	blue	na
chrUn	21602940	21603697	758	SAT1_CF	blue	na
chrUn	21603710	21603952	243	SAT1_CF	blue	na
chrUn	21604140	21605582	1443	SAT1_CF	blue	na
chrUn	21605649	21606660	1012	SAT1_CF	blue	na
chrUn	21606677	21607289	613	SAT1_CF	blue	na
chrUn	21607338	21607813	476	SAT1_CF	blue	na
chrUn	21607825	21607934	110	SAT1_CF	blue	na
chrUn	21607955	21608861	907	SAT1_CF	blue	na
chrUn	21608879	21609830	952	SAT1_CF	blue	na
chrUn	21609841	21611264	1424	SAT1_CF	blue	na
chrUn	21611289	21612157	869	SAT1_CF	blue	na
chrUn	21612202	21612570	369	SAT1_CF	blue	na
chrUn	21612631	21613174	544	SAT1_CF	blue	na
chrUn	21613217	21614288	1072	SAT1_CF	blue	na
chrUn	21614322	21615253	932	SAT1_CF	blue	na

chrUn	21615293	21615789	497	SAT1_CF	blue	na
chrUn	21615806	21616470	665	SAT1_CF	blue	na
chrUn	21616527	21617071	545	SAT1_CF	blue	na
chrUn	21617114	21617658	545	SAT1_CF	blue	na
chrUn	21617680	21618351	672	SAT1_CF	blue	na
chrUn	21618365	21619926	1562	SAT1_CF	blue	na
chrUn	21619944	21620706	763	SAT1_CF	blue	na
chrUn	21620718	21622740	2023	SAT1_CF	blue	na
chrUn	21622771	21623296	526	SAT1_CF	blue	na
chrUn	21623311	21623467	157	SAT1_CF	blue	na
chrUn	21623493	21624722	1230	SAT1_CF	blue	na
chrUn	21624754	21625237	484	SAT1_CF	blue	na
chrUn	21625291	21625446	156	SAT1_CF	blue	na
chrUn	21625472	21625992	521	SAT1_CF	blue	na
chrUn	21626004	21626087	84	SAT1_CF	blue	na
chrUn	21626135	21626688	554	SAT1_CF	blue	na
chrUn	21626700	21627556	857	SAT1_CF	blue	na
chrUn	21627598	21628141	544	SAT1_CF	blue	na
chrUn	21628157	21631005	2849	SAT1_CF	blue	na
chrUn	21631084	21631633	550	SAT1_CF	blue	na
chrUn	21631713	21632029	317	SAT1_CF	blue	na
chrUn	21632085	21634270	2186	SAT1_CF	blue	na
chrUn	21634338	21634966	629	SAT1_CF	blue	na
chrUn	21634977	21635296	320	SAT1_CF	blue	na
chrUn	21635349	21635851	503	SAT1_CF	blue	na
chrUn	21635869	21637234	1366	SAT1_CF	blue	na
chrUn	21637277	21638235	959	SAT1_CF	blue	na
chrUn	21638302	21639458	1157	SAT1_CF	blue	na
chrUn	21639470	21640150	681	SAT1_CF	blue	na
chrUn	21640161	21640653	493	SAT1_CF	blue	na
chrUn	21640665	21641992	1328	SAT1_CF	blue	na
chrUn	21642043	21643008	966	SAT1_CF	blue	na
chrUn	21643037	21643556	520	SAT1_CF	blue	na
chrUn	21643569	21644251	683	SAT1_CF	blue	na
chrUn	21644274	21645450	1177	SAT1_CF	blue	na
chrUn	21645463	21646147	685	SAT1_CF	blue	na
chrUn	21646159	21647584	1426	SAT1_CF	blue	na
chrUn	21647663	21648209	547	SAT1_CF	blue	na
chrUn	21648287	21648835	549	SAT1_CF	blue	na
chrUn	21648847	21648949	103	SAT1_CF	blue	na
chrUn	21648978	21649522	545	SAT1_CF	blue	na
chrUn	21649537	21649709	173	SAT1_CF	blue	na

chrUn	21649762	21651197	1436	SAT1_CF	blue	na
chrUn	21651208	21651606	399	SAT1_CF	blue	na
chrUn	21651649	21652349	701	SAT1_CF	blue	na
chrUn	21653758	21654319	562	SAT1_CF	blue	na
chrUn	21654376	21655572	1197	SAT1_CF	blue	na
chrUn	21655584	21656437	854	SAT1_CF	blue	na
chrUn	21656479	21657382	904	SAT1_CF	blue	na
chrUn	21657413	21659233	1821	SAT1_CF	blue	na
chrUn	21659305	21659818	514	SAT1_CF	blue	na
chrUn	21659972	21660203	232	SAT1_CF	blue	na
chrUn	21660246	21660830	585	SAT1_CF	blue	na
chrUn	21660842	21661555	714	SAT1_CF	blue	na
chrUn	21661637	21662177	541	SAT1_CF	blue	na
chrUn	21662190	21662691	502	SAT1_CF	blue	na
chrUn	21662704	21663378	675	SAT1_CF	blue	na
chrUn	21663421	21664386	966	SAT1_CF	blue	na
chrUn	21664465	21665008	544	SAT1_CF	blue	na
chrUn	21665021	21665178	158	SAT1_CF	blue	na
chrUn	21665225	21665745	521	SAT1_CF	blue	na
chrUn	21665758	21666364	607	SAT1_CF	blue	na
chrUn	21666376	21667049	674	SAT1_CF	blue	na
chrUn	21667092	21667724	633	SAT1_CF	blue	na
chrUn	21667751	21668315	565	SAT1_CF	blue	na
chrUn	21668396	21668883	488	SAT1_CF	blue	na
chrUn	21668935	21669092	158	SAT1_CF	blue	na
chrUn	21669137	21669634	498	SAT1_CF	blue	na
chrUn	21669647	21670320	674	SAT1_CF	blue	na
chrUn	21670364	21670874	511	SAT1_CF	blue	na
chrUn	21670944	21671499	556	SAT1_CF	blue	na
chrUn	21671510	21672817	1308	SAT1_CF	blue	na
chrUn	21672860	21673633	774	SAT1_CF	blue	na
chrUn	21673712	21674671	960	SAT1_CF	blue	na
chrUn	21674711	21675909	1199	SAT1_CF	blue	na
chrUn	21675921	21676598	678	SAT1_CF	blue	na
chrUn	21676641	21677602	962	SAT1_CF	blue	na
chrUn	21677669	21678189	521	SAT1_CF	blue	na
chrUn	21678202	21678390	189	SAT1_CF	blue	na
chrUn	21678442	21679200	759	SAT1_CF	blue	na
chrUn	21679278	21679825	548	SAT1_CF	blue	na
chrUn	21679836	21680191	356	SAT1_CF	blue	na
chrUn	21680277	21680779	503	SAT1_CF	blue	na
chrUn	21680792	21681546	755	SAT1_CF	blue	na

chrUn	21681616	21682162	547	SAT1_CF	blue	na
chrUn	21682229	21683028	800	SAT1_CF	blue	na
chrUn	21683052	21685250	2199	SAT1_CF	blue	na
chrUn	21685278	21685659	382	SAT1_CF	blue	na
chrUn	21685681	21686201	521	SAT1_CF	blue	na
chrUn	21686213	21686322	110	SAT1_CF	blue	na
chrUn	21686343	21686975	633	SAT1_CF	blue	na
chrUn	21686987	21687654	668	SAT1_CF	blue	na
chrUn	21687711	21688256	546	SAT1_CF	blue	na
chrUn	21688299	21688842	544	SAT1_CF	blue	na
chrUn	21688864	21688960	97	SAT1_CF	blue	na
chrUn	21688979	21689533	555	SAT1_CF	blue	na
chrUn	21689545	21690220	676	SAT1_CF	blue	na
chrUn	21690270	21691230	961	SAT1_CF	blue	na
chrUn	21691345	21691831	487	SAT1_CF	blue	na
chrUn	21691883	21692442	560	SAT1_CF	blue	na
chrUn	21692454	21693682	1229	SAT1_CF	blue	na
chrUn	21693725	21694266	542	SAT1_CF	blue	na
chrUn	21694308	21694590	283	SAT1_CF	blue	na
chrUn	21694608	21696154	1547	SAT1_CF	blue	na
chrUn	21696166	21696849	684	SAT1_CF	blue	na
chrUn	21696861	21697543	683	SAT1_CF	blue	na
chrUn	21697556	21698395	840	SAT1_CF	blue	na
chrUn	21698408	21699596	1189	SAT1_CF	blue	na
chrUn	21699608	21700767	1160	SAT1_CF	blue	na
chrUn	21700787	21701396	610	SAT1_CF	blue	na
chrUn	21701476	21701640	165	SAT1_CF	blue	na
chrUn	21701822	21701981	160	SAT1_CF	blue	na
chrUn	21702042	21702621	580	SAT1_CF	blue	na
chrUn	21702683	21703984	1302	SAT1_CF	blue	na
chrUn	21703996	21704858	863	SAT1_CF	blue	na
chrUn	21704900	21705531	632	SAT1_CF	blue	na
chrUn	21705559	21706562	1004	SAT1_CF	blue	na
chrUn	21706596	21706945	350	SAT1_CF	blue	na
chrUn	21707001	21707501	501	SAT1_CF	blue	na
chrUn	21707515	21708198	684	SAT1_CF	blue	na
chrUn	21708210	21708893	684	SAT1_CF	blue	na
chrUn	21708904	21709396	493	SAT1_CF	blue	na
chrUn	21709410	21711035	1626	SAT1_CF	blue	na
chrUn	21711049	21711590	542	SAT1_CF	blue	na
chrUn	21712988	21713561	574	SAT1_CF	blue	na
chrUn	21713625	21714218	594	SAT1_CF	blue	na

chrUn	21714243	21714788	546	SAT1_CF	blue	na
chrUn	21714831	21716694	1864	SAT1_CF	blue	na
chrUn	21716709	21717685	977	SAT1_CF	blue	na
chrUn	21717708	21719274	1567	SAT1_CF	blue	na
chrUn	21719295	21720511	1217	SAT1_CF	blue	na
chrUn	21720522	21720632	111	SAT1_CF	blue	na
chrUn	21720653	21721197	545	SAT1_CF	blue	na
chrUn	21721240	21723101	1862	SAT1_CF	blue	na
chrUn	21723116	21723252	137	SAT1_CF	blue	na
chrUn	21723318	21723820	503	SAT1_CF	blue	na
chrUn	21723833	21723908	76	SAT1_CF	blue	na
chrUn	21723927	21724481	555	SAT1_CF	blue	na
chrUn	21724494	21725136	643	SAT1_CF	blue	na
chrUn	21725179	21727012	1834	SAT1_CF	blue	na
chrUn	21727099	21727601	503	SAT1_CF	blue	na
chrUn	21727613	21727718	106	SAT1_CF	blue	na
chrUn	21727742	21728544	803	SAT1_CF	blue	na
chrUn	21728557	21728665	109	SAT1_CF	blue	na
chrUn	21728686	21729435	750	SAT1_CF	blue	na
chrUn	21729457	21730003	547	SAT1_CF	blue	na
chrUn	21730047	21731884	1838	SAT1_CF	blue	na
chrUn	21732065	21732240	176	SAT1_CF	blue	na
chrUn	21732265	21733257	993	SAT1_CF	blue	na
chrUn	21733269	21734598	1330	SAT1_CF	blue	na
chrUn	21734622	21735974	1353	SAT1_CF	blue	na
chrUn	21736091	21736633	543	SAT1_CF	blue	na
chrUn	21736645	21736758	114	SAT1_CF	blue	na
chrUn	21736774	21737321	548	SAT1_CF	blue	na
chrUn	21737364	21739404	2041	SAT1_CF	blue	na
chrUn	21739425	21739942	518	SAT1_CF	blue	na
chrUn	21739969	21740636	668	SAT1_CF	blue	na
chrUn	21740649	21740757	109	SAT1_CF	blue	na
chrUn	21740778	21741313	536	SAT1_CF	blue	na
chrUn	21741356	21742653	1298	SAT1_CF	blue	na
chrUn	21742718	21743200	483	SAT1_CF	blue	na
chrUn	21743227	21743891	665	SAT1_CF	blue	na
chrUn	21743903	21744586	684	SAT1_CF	blue	na
chrUn	21744598	21745483	886	SAT1_CF	blue	na
chrUn	21745505	21746356	852	SAT1_CF	blue	na
chrUn	21746369	21747703	1335	SAT1_CF	blue	na
chrUn	21747746	21748284	539	SAT1_CF	blue	na
chrUn	21748311	21748588	278	SAT1_CF	blue	na

chrUn	21748602	21749149	548	SAT1_CF	blue	na
chrUn	21749192	21749743	552	SAT1_CF	blue	na
chrUn	21749755	21751126	1372	SAT1_CF	blue	na
chrUn	21751169	21751801	633	SAT1_CF	blue	na
chrUn	21751829	21752388	560	SAT1_CF	blue	na
chrUn	21752468	21753017	550	SAT1_CF	blue	na
chrUn	21753045	21753182	138	SAT1_CF	blue	na
chrUn	21753229	21753749	521	SAT1_CF	blue	na
chrUn	21753778	21754614	837	SAT1_CF	blue	na
chrUn	21754699	21755242	544	SAT1_CF	blue	na
chrUn	21755334	21756442	1109	SAT1_CF	blue	na
chrUn	21756516	21757611	1096	SAT1_CF	blue	na
chrUn	21757625	21758248	624	SAT1_CF	blue	na
chrUn	21758260	21758935	676	SAT1_CF	blue	na
chrUn	21758996	21759538	543	SAT1_CF	blue	na
chrUn	21759635	21760367	733	SAT1_CF	blue	na
chrUn	21760380	21760732	353	SAT1_CF	blue	na
chrUn	21760849	21762078	1230	SAT1_CF	blue	na
chrUn	21762090	21762735	646	SAT1_CF	blue	na
chrUn	21762759	21764086	1328	SAT1_CF	blue	na
chrUn	21764115	21764494	380	SAT1_CF	blue	na
chrUn	21764537	21766201	1665	SAT1_CF	blue	na
chrUn	21766214	21766318	105	SAT1_CF	blue	na
chrUn	21766343	21766973	631	SAT1_CF	blue	na
chrUn	21766987	21767846	860	SAT1_CF	blue	na
chrUn	21767887	21768431	545	SAT1_CF	blue	na
chrUn	21768469	21769581	1113	SAT1_CF	blue	na
chrUn	21769763	21770484	722	SAT1_CF	blue	na
chrUn	21770497	21770604	108	SAT1_CF	blue	na
chrUn	21770625	21771968	1344	SAT1_CF	blue	na
chrUn	21772178	21772312	135	SAT1_CF	blue	na
chrUn	21772399	21772875	477	SAT1_CF	blue	na
chrUn	21772892	21774215	1324	SAT1_CF	blue	na
chrUn	21774230	21774387	158	SAT1_CF	blue	na
chrUn	21774413	21775357	945	SAT1_CF	blue	na
chrUn	21775397	21775898	502	SAT1_CF	blue	na
chrUn	21775911	21778156	2246	SAT1_CF	blue	na
chrUn	21778169	21778524	356	SAT1_CF	blue	na
chrUn	21778640	21779160	521	SAT1_CF	blue	na
chrUn	21779191	21779717	527	SAT1_CF	blue	na
chrUn	21779761	21780264	504	SAT1_CF	blue	na
chrUn	21780278	21780799	522	SAT1_CF	blue	na

chrUn	21780833	21781212	380	SAT1_CF	blue	na
chrUn	21781252	21781753	502	SAT1_CF	blue	na
chrUn	21781766	21781878	113	SAT1_CF	blue	na
chrUn	21782043	21782708	666	SAT1_CF	blue	na
chrUn	21782725	21783948	1224	SAT1_CF	blue	na
chrUn	21784018	21784408	391	SAT1_CF	blue	na
chrUn	21784439	21784960	522	SAT1_CF	blue	na
chrUn	21784973	21785324	352	SAT1_CF	blue	na
chrUn	21785377	21785877	501	SAT1_CF	blue	na
chrUn	21785895	21786961	1067	SAT1_CF	blue	na
chrUn	21786987	21787468	482	SAT1_CF	blue	na
chrUn	21787495	21788179	685	SAT1_CF	blue	na
chrUn	21788192	21788827	636	SAT1_CF	blue	na
chrUn	21788870	21790497	1628	SAT1_CF	blue	na
chrUn	21790540	21792245	1706	SAT1_CF	blue	na
chrUn	21795681	21797719	2039	SAT1_CF	blue	na
chrUn	21797737	21800052	2316	SAT1_CF	blue	na
chrUn	21820091	21821311	1221	Sat2CF	purple	na
chrUn	21844985	21846163	1179	Sat2CF	purple	na
chrUn	21845016	21846035	1020	Sat2CF	purple	na
chrUn	21846012	21846193	182	Sat2CF	purple	na
chrUn	21874795	21874941	147	Sat2CF	purple	na
chrUn	21874817	21875962	1146	Sat2CF	purple	na
chrUn	21891012	21892243	1232	Sat2CF	purple	na
chrUn	21891061	21892043	983	Sat2CF	purple	na
chrUn	21906693	21907768	1076	Sat2CF	purple	na
chrUn	21915472	21916320	849	Sat2CF	purple	na
chrUn	21930346	21931579	1234	Sat2CF	purple	na
chrUn	21931428	21931608	181	Sat2CF	purple	na
chrUn	21997000	21997167	168	Sat2CF	purple	na
chrUn	21997050	21997848	799	Sat2CF	purple	na
chrUn	22013107	22013991	885	Sat2CF	purple	na
chrUn	22013123	22013856	734	Sat2CF	purple	na
chrUn	22026834	22027579	746	Sat2CF	purple	na
chrUn	22049715	22050850	1136	Sat2CF	purple	na
chrUn	22075598	22076440	843	Sat2CF	purple	na
chrUn	22100237	22101415	1179	Sat2CF	purple	na
chrUn	22105239	22105668	430	CarSat1	red	na
chrUn	22105686	22106404	719	CarSat1	red	na
chrUn	22106399	22107015	617	SAT1_CF	blue	na
chrUn	22107028	22107536	509	SAT1_CF	blue	na
chrUn	22107577	22110685	3109	SAT1_CF	blue	na

chrUn	22110687	22112633	1947	CarSat1	red	na
chrUn	22112659	22114248	1590	CarSat1	red	na
chrUn	22114333	22114973	641	CarSat1	red	na
chrUn	22129938	22130096	159	Sat2CF	purple	na
chrUn	22129961	22130672	712	Sat2CF	purple	na
chrUn	22143450	22143608	159	Sat2CF	purple	na
chrUn	22143473	22144174	702	Sat2CF	purple	na
chrUn	22214630	22214792	163	Sat2CF	purple	na
chrUn	22214648	22215852	1205	Sat2CF	purple	na
chrUn	22215723	22215903	181	Sat2CF	purple	na
chrUn	22231629	22232848	1220	Sat2CF	purple	na
chrUn	22252723	22252881	159	Sat2CF	purple	na
chrUn	22252751	22253463	713	Sat2CF	purple	na
chrUn	22253336	22253485	150	Sat2CF	purple	na
chrUn	22253336	22253485	150	Sat2CF	purple	na
chrUn	22272527	22273611	1085	Sat2CF	purple	na
chrUn	22313835	22315086	1252	Sat2CF	purple	na
chrUn	22313965	22315050	1086	Sat2CF	purple	na
chrUn	22314926	22315144	219	Sat2CF	purple	na
chrUn	22317962	22318162	201	CarSat1	red	na
chrUn	22318345	22319701	1357	CarSat1	red	na
chrUn	22319728	22321212	1485	CarSat1	red	na
chrUn	22330321	22331003	683	CarSat1	red	na
chrUn	22331021	22332540	1520	CarSat1	red	na
chrUn	22332559	22332960	402	CarSat1	red	na
chrUn	22333013	22334365	1353	SAT1_CF	blue	na
chrUn	22334560	22334721	162	Sat2CF	purple	na
chrUn	22334593	22335676	1084	Sat2CF	purple	na
chrUn	22340864	22341577	714	CarSat1	red	na
chrUn	22341770	22343388	1619	CarSat1	red	na
chrUn	22343413	22344123	711	CarSat1	red	na
chrUn	22344149	22344859	711	CarSat1	red	na
chrUn	22344885	22346081	1197	CarSat1	red	na
chrUn	22346108	22348547	2440	CarSat1	red	na
chrUn	22348563	22349165	603	CarSat1	red	na
chrUn	22349433	22350508	1076	CarSat1	red	na
chrUn	22350535	22352716	2182	CarSat1	red	na
chrUn	22352743	22353505	763	CarSat1	red	na
chrUn	22467934	22469069	1136	Sat2CF	purple	na
chrUn	22468068	22469154	1087	Sat2CF	purple	na
chrUn	22469030	22469179	150	Sat2CF	purple	na
chrUn	22493440	22494304	865	Sat2CF	purple	na

chrUn	22493582	22494389	808	Sat2CF	purple	na
chrUn	22539151	22539819	669	SAT1_CF	blue	na
chrUn	22539832	22540535	704	SAT1_CF	blue	na
chrUn	22540557	22542912	2356	SAT1_CF	blue	na
chrUn	22542926	22543061	136	SAT1_CF	blue	na
chrUn	22543108	22543628	521	SAT1_CF	blue	na
chrUn	22543641	22544321	681	SAT1_CF	blue	na
chrUn	22544333	22545092	760	SAT1_CF	blue	na
chrUn	22545106	22545779	674	SAT1_CF	blue	na
chrUn	22545822	22546821	1000	SAT1_CF	blue	na
chrUn	22546865	22547492	628	SAT1_CF	blue	na
chrUn	22547554	22548306	753	SAT1_CF	blue	na
chrUn	22548321	22548923	603	SAT1_CF	blue	na
chrUn	22548934	22549861	928	SAT1_CF	blue	na
chrUn	22550186	22550595	410	SAT1_CF	blue	na
chrUn	22550616	22550681	66	SAT1_CF	blue	na
chrUn	22550702	22551332	631	SAT1_CF	blue	na
chrUn	22551353	22551418	66	SAT1_CF	blue	na
chrUn	22551439	22551958	520	SAT1_CF	blue	na
chrUn	22551970	22553304	1335	SAT1_CF	blue	na
chrUn	22553329	22553780	452	SAT1_CF	blue	na
chrUn	22553795	22554568	774	SAT1_CF	blue	na
chrUn	22554749	22554932	184	SAT1_CF	blue	na
chrUn	22554972	22555472	501	SAT1_CF	blue	na
chrUn	22555485	22555590	106	SAT1_CF	blue	na
chrUn	22555613	22557560	1948	SAT1_CF	blue	na
chrUn	22557572	22558244	673	SAT1_CF	blue	na
chrUn	22558287	22559288	1002	SAT1_CF	blue	na
chrUn	22559343	22559950	608	SAT1_CF	blue	na
chrUn	22559963	22560318	356	SAT1_CF	blue	na
chrUn	22560435	22560953	519	SAT1_CF	blue	na
chrUn	22560984	22562057	1074	SAT1_CF	blue	na
chrUn	22562071	22562997	927	SAT1_CF	blue	na
chrUn	22563046	22563547	502	SAT1_CF	blue	na
chrUn	22563561	22564319	759	SAT1_CF	blue	na
chrUn	22564331	22564440	110	SAT1_CF	blue	na
chrUn	22564461	22565005	545	SAT1_CF	blue	na
chrUn	22565066	22565614	549	SAT1_CF	blue	na
chrUn	22565656	22566199	544	SAT1_CF	blue	na
chrUn	22566237	22567191	955	SAT1_CF	blue	na
chrUn	22567218	22568399	1182	SAT1_CF	blue	na
chrUn	22568431	22568586	156	SAT1_CF	blue	na

chrUn	22568612	22569131	520	SAT1_CF	blue	na
chrUn	22569144	22569818	675	SAT1_CF	blue	na
chrUn	22569861	22570826	966	SAT1_CF	blue	na
chrUn	22570904	22571815	912	SAT1_CF	blue	na
chrUn	22571934	22572470	537	SAT1_CF	blue	na
chrUn	22572490	22572597	108	SAT1_CF	blue	na
chrUn	22572618	22573173	556	SAT1_CF	blue	na
chrUn	22573185	22574868	1684	SAT1_CF	blue	na
chrUn	22574883	22575019	137	SAT1_CF	blue	na
chrUn	22575085	22575586	502	SAT1_CF	blue	na
chrUn	22575599	22576929	1331	SAT1_CF	blue	na
chrUn	22576975	22578203	1229	SAT1_CF	blue	na
chrUn	22578231	22578367	137	SAT1_CF	blue	na
chrUn	22578433	22578934	502	SAT1_CF	blue	na
chrUn	22578957	22579627	671	SAT1_CF	blue	na
chrUn	22579639	22579755	117	SAT1_CF	blue	na
chrUn	22579768	22581971	2204	SAT1_CF	blue	na
chrUn	22581982	22582384	403	SAT1_CF	blue	na
chrUn	22582426	22582919	494	SAT1_CF	blue	na
chrUn	22582939	22583621	683	SAT1_CF	blue	na
chrUn	22583634	22584316	683	SAT1_CF	blue	na
chrUn	22584328	22585189	862	SAT1_CF	blue	na
chrUn	22585231	22586045	815	SAT1_CF	blue	na
chrUn	22586090	22587115	1026	SAT1_CF	blue	na
chrUn	22587128	22588913	1786	SAT1_CF	blue	na
chrUn	22588956	22589158	203	SAT1_CF	blue	na
chrUn	22589339	22589524	186	SAT1_CF	blue	na
chrUn	22589541	22590060	520	SAT1_CF	blue	na
chrUn	22590073	22590178	106	SAT1_CF	blue	na
chrUn	22590201	22590832	632	SAT1_CF	blue	na
chrUn	22590856	22591880	1025	SAT1_CF	blue	na
chrUn	22591905	22592915	1011	SAT1_CF	blue	na
chrUn	22592958	22593505	548	SAT1_CF	blue	na
chrUn	22593533	22593669	137	SAT1_CF	blue	na
chrUn	22593735	22594236	502	SAT1_CF	blue	na
chrUn	22594259	22594932	674	SAT1_CF	blue	na
chrUn	22594944	22595617	674	SAT1_CF	blue	na
chrUn	22595666	22596641	976	SAT1_CF	blue	na
chrUn	22596657	22597551	895	SAT1_CF	blue	na
chrUn	22597563	22598166	604	SAT1_CF	blue	na
chrUn	22598179	22598284	106	SAT1_CF	blue	na
chrUn	22598305	22598780	476	SAT1_CF	blue	na

chrUn	22598792	22599657	866	SAT1_CF	blue	na
chrUn	22599699	22601393	1695	SAT1_CF	blue	na
chrUn	22601573	22601756	184	SAT1_CF	blue	na
chrUn	22601777	22602296	520	SAT1_CF	blue	na
chrUn	22602309	22602414	106	SAT1_CF	blue	na
chrUn	22602437	22603707	1271	SAT1_CF	blue	na
chrUn	22603722	22603858	137	SAT1_CF	blue	na
chrUn	22603924	22604426	503	SAT1_CF	blue	na
chrUn	22604439	22604545	107	SAT1_CF	blue	na
chrUn	22604567	22605039	473	SAT1_CF	blue	na
chrUn	22605051	22605713	663	SAT1_CF	blue	na
chrUn	22605756	22607380	1625	SAT1_CF	blue	na
chrUn	22607412	22607777	366	SAT1_CF	blue	na
chrUn	22607811	22609267	1457	SAT1_CF	blue	na
chrUn	22609282	22609834	553	SAT1_CF	blue	na
chrUn	22609856	22610490	635	SAT1_CF	blue	na
chrUn	22610533	22610969	437	SAT1_CF	blue	na
chrUn	22610981	22612114	1134	SAT1_CF	blue	na
chrUn	22612129	22612287	159	SAT1_CF	blue	na
chrUn	22612331	22612826	496	SAT1_CF	blue	na
chrUn	22612845	22613528	684	SAT1_CF	blue	na
chrUn	22613540	22614217	678	SAT1_CF	blue	na
chrUn	22614314	22616413	2100	SAT1_CF	blue	na
chrUn	22616457	22617019	563	SAT1_CF	blue	na
chrUn	22617103	22618104	1002	SAT1_CF	blue	na
chrUn	22618147	22618696	550	SAT1_CF	blue	na
chrUn	22618773	22619317	545	SAT1_CF	blue	na
chrUn	22619330	22619406	77	SAT1_CF	blue	na
chrUn	22619424	22619987	564	SAT1_CF	blue	na
chrUn	22619999	22620750	752	SAT1_CF	blue	na
chrUn	22620761	22621592	832	SAT1_CF	blue	na
chrUn	22621623	22622132	510	SAT1_CF	blue	na
chrUn	22622143	22622545	403	SAT1_CF	blue	na
chrUn	22622585	22623086	502	SAT1_CF	blue	na
chrUn	22623099	22624556	1458	SAT1_CF	blue	na
chrUn	22624604	22625089	486	SAT1_CF	blue	na
chrUn	22625344	22625478	135	SAT1_CF	blue	na
chrUn	22625525	22626043	519	SAT1_CF	blue	na
chrUn	22626056	22627420	1365	SAT1_CF	blue	na
chrUn	22627464	22627893	430	SAT1_CF	blue	na
chrUn	22627910	22628798	889	SAT1_CF	blue	na
chrUn	22628885	22629623	739	SAT1_CF	blue	na

chrUn	22629646	22630170	525	SAT1_CF	blue	na
chrUn	22630192	22631371	1180	SAT1_CF	blue	na
chrUn	22631468	22632231	764	SAT1_CF	blue	na
chrUn	22632245	22633613	1369	SAT1_CF	blue	na
chrUn	22633659	22634579	921	SAT1_CF	blue	na
chrUn	22634695	22635244	550	SAT1_CF	blue	na
chrUn	22635256	22635653	398	SAT1_CF	blue	na
chrUn	22635713	22636195	483	SAT1_CF	blue	na
chrUn	22636207	22636892	686	SAT1_CF	blue	na
chrUn	22636914	22637579	666	SAT1_CF	blue	na
chrUn	22637624	22638843	1220	SAT1_CF	blue	na
chrUn	22638924	22639448	525	SAT1_CF	blue	na
chrUn	22639463	22639620	158	SAT1_CF	blue	na
chrUn	22639646	22640166	521	SAT1_CF	blue	na
chrUn	22640308	22640820	513	SAT1_CF	blue	na
chrUn	22640864	22641662	799	SAT1_CF	blue	na
chrUn	22641705	22642287	583	SAT1_CF	blue	na
chrUn	22642330	22642817	488	SAT1_CF	blue	na
chrUn	22642868	22643025	158	SAT1_CF	blue	na
chrUn	22643070	22643572	503	SAT1_CF	blue	na
chrUn	22643584	22643690	107	SAT1_CF	blue	na
chrUn	22643713	22644387	675	SAT1_CF	blue	na
chrUn	22644409	22645737	1329	SAT1_CF	blue	na
chrUn	22645768	22646254	487	SAT1_CF	blue	na
chrUn	22646305	22646462	158	SAT1_CF	blue	na
chrUn	22646507	22647008	502	SAT1_CF	blue	na
chrUn	22647020	22648827	1808	SAT1_CF	blue	na
chrUn	22648845	22649367	523	SAT1_CF	blue	na
chrUn	22649378	22649732	355	SAT1_CF	blue	na
chrUn	22649838	22652309	2472	SAT1_CF	blue	na
chrUn	22652491	22652676	186	SAT1_CF	blue	na
chrUn	22652732	22653205	474	SAT1_CF	blue	na
chrUn	22653218	22653326	109	SAT1_CF	blue	na
chrUn	22653347	22654331	985	SAT1_CF	blue	na
chrUn	22654418	22654919	502	SAT1_CF	blue	na
chrUn	22654937	22655606	670	SAT1_CF	blue	na
chrUn	22655640	22656415	776	SAT1_CF	blue	na
chrUn	22656426	22656823	398	SAT1_CF	blue	na
chrUn	22656868	22657957	1090	SAT1_CF	blue	na
chrUn	22657975	22659300	1326	SAT1_CF	blue	na
chrUn	22659343	22659649	307	SAT1_CF	blue	na
chrUn	22659727	22660235	509	SAT1_CF	blue	na

chrUn	22660249	22660899	651	SAT1_CF	blue	na
chrUn	22660921	22661672	752	SAT1_CF	blue	na
chrUn	22661685	22662301	617	SAT1_CF	blue	na
chrUn	22662515	22662669	155	SAT1_CF	blue	na
chrUn	22662714	22663332	619	SAT1_CF	blue	na
chrUn	22663355	22664815	1461	SAT1_CF	blue	na
chrUn	22664902	22665401	500	SAT1_CF	blue	na
chrUn	22665424	22666217	794	SAT1_CF	blue	na
chrUn	22666239	22666784	546	SAT1_CF	blue	na
chrUn	22666827	22667730	904	SAT1_CF	blue	na
chrUn	22667744	22668291	548	SAT1_CF	blue	na
chrUn	22669719	22670289	571	SAT1_CF	blue	na
chrUn	22670332	22670826	495	SAT1_CF	blue	na
chrUn	22670846	22671530	685	SAT1_CF	blue	na
chrUn	22671542	22672739	1198	SAT1_CF	blue	na
chrUn	22672781	22673323	543	SAT1_CF	blue	na
chrUn	22673423	22675158	1736	SAT1_CF	blue	na
chrUn	22675172	22675277	106	SAT1_CF	blue	na
chrUn	22675300	22675859	560	SAT1_CF	blue	na
chrUn	22675903	22677499	1597	SAT1_CF	blue	na
chrUn	22677511	22678187	677	SAT1_CF	blue	na
chrUn	22678235	22679192	958	SAT1_CF	blue	na
chrUn	22679271	22680231	961	SAT1_CF	blue	na
chrUn	22680271	22680771	501	SAT1_CF	blue	na
chrUn	22680790	22680893	104	SAT1_CF	blue	na
chrUn	22680914	22682113	1200	SAT1_CF	blue	na
chrUn	22682157	22683363	1207	SAT1_CF	blue	na
chrUn	22683378	22683514	137	SAT1_CF	blue	na
chrUn	22683561	22684080	520	SAT1_CF	blue	na
chrUn	22684102	22684808	707	SAT1_CF	blue	na
chrUn	22684851	22685578	728	SAT1_CF	blue	na
chrUn	22685605	22686129	525	SAT1_CF	blue	na
chrUn	22686174	22687466	1293	SAT1_CF	blue	na
chrUn	22687547	22687773	227	SAT1_CF	blue	na
chrUn	22687784	22688566	783	SAT1_CF	blue	na
chrUn	22688594	22689239	646	SAT1_CF	blue	na
chrUn	22689262	22689836	575	SAT1_CF	blue	na
chrUn	22689848	22690611	764	SAT1_CF	blue	na
chrUn	22690625	22691270	646	SAT1_CF	blue	na
chrUn	22691282	22694603	3322	SAT1_CF	blue	na
chrUn	22694645	22694810	166	SAT1_CF	blue	na
chrUn	22694991	22695176	186	SAT1_CF	blue	na

chrUn	22695193	22695706	514	SAT1_CF	blue	na
chrUn	22695726	22695834	109	SAT1_CF	blue	na
chrUn	22695854	22696413	560	SAT1_CF	blue	na
chrUn	22696425	22698946	2522	SAT1_CF	blue	na
chrUn	22698958	22699436	479	SAT1_CF	blue	na
chrUn	22699626	22701250	1625	SAT1_CF	blue	na
chrUn	22701366	22701762	397	SAT1_CF	blue	na
chrUn	22701830	22702349	520	SAT1_CF	blue	na
chrUn	22702362	22703041	680	SAT1_CF	blue	na
chrUn	22703053	22703813	761	SAT1_CF	blue	na
chrUn	22703827	22704502	676	SAT1_CF	blue	na
chrUn	22704545	22706171	1627	SAT1_CF	blue	na
chrUn	22706252	22706417	166	SAT1_CF	blue	na
chrUn	22706599	22706776	178	SAT1_CF	blue	na
chrUn	22706822	22707321	500	SAT1_CF	blue	na
chrUn	22707334	22708494	1161	SAT1_CF	blue	na
chrUn	22708536	22709080	545	SAT1_CF	blue	na
chrUn	22709092	22710411	1320	SAT1_CF	blue	na
chrUn	22710454	22710826	373	SAT1_CF	blue	na
chrUn	22710862	22711362	501	SAT1_CF	blue	na
chrUn	22711376	22712059	684	SAT1_CF	blue	na
chrUn	22712081	22713229	1149	SAT1_CF	blue	na
chrUn	22713267	22713881	615	SAT1_CF	blue	na
chrUn	22713893	22714655	763	SAT1_CF	blue	na
chrUn	22714668	22715236	569	SAT1_CF	blue	na
chrUn	22715248	22715877	630	SAT1_CF	blue	na
chrUn	22715888	22716437	550	SAT1_CF	blue	na
chrUn	22716449	22716558	110	SAT1_CF	blue	na
chrUn	22716578	22717132	555	SAT1_CF	blue	na
chrUn	22717144	22717814	671	SAT1_CF	blue	na
chrUn	22717901	22718400	500	SAT1_CF	blue	na
chrUn	22718449	22719738	1290	SAT1_CF	blue	na
chrUn	22719753	22719889	137	SAT1_CF	blue	na
chrUn	22719974	22720577	604	SAT1_CF	blue	na
chrUn	22720598	22721153	556	SAT1_CF	blue	na
chrUn	22721164	22721766	603	SAT1_CF	blue	na
chrUn	22721778	22722451	674	SAT1_CF	blue	na
chrUn	22722494	22724009	1516	SAT1_CF	blue	na
chrUn	22724125	22724645	521	SAT1_CF	blue	na
chrUn	22724676	22726519	1844	SAT1_CF	blue	na
chrUn	22726534	22727237	704	SAT1_CF	blue	na
chrUn	22727262	22728803	1542	SAT1_CF	blue	na

chrUn	22728845	22729626	782	SAT1_CF	blue	na
chrUn	22729704	22730210	507	SAT1_CF	blue	na
chrUn	22730224	22730579	356	SAT1_CF	blue	na
chrUn	22730685	22731166	482	SAT1_CF	blue	na
chrUn	22731179	22731861	683	SAT1_CF	blue	na
chrUn	22731873	22732732	860	SAT1_CF	blue	na
chrUn	22732774	22734433	1660	SAT1_CF	blue	na
chrUn	22734466	22735170	705	SAT1_CF	blue	na
chrUn	22735184	22735787	604	SAT1_CF	blue	na
chrUn	22735799	22736484	686	SAT1_CF	blue	na
chrUn	22736496	22737331	836	SAT1_CF	blue	na
chrUn	22737345	22738020	676	SAT1_CF	blue	na
chrUn	22738063	22738401	339	SAT1_CF	blue	na
chrUn	22738431	22738957	527	SAT1_CF	blue	na
chrUn	22738970	22739076	107	SAT1_CF	blue	na
chrUn	22739098	22740912	1815	SAT1_CF	blue	na
chrUn	22740927	22741062	136	SAT1_CF	blue	na
chrUn	22741128	22741630	503	SAT1_CF	blue	na
chrUn	22741642	22741748	107	SAT1_CF	blue	na
chrUn	22741772	22742399	628	SAT1_CF	blue	na
chrUn	22742423	22743281	859	SAT1_CF	blue	na
chrUn	22743308	22744300	993	SAT1_CF	blue	na
chrUn	22744349	22744850	502	SAT1_CF	blue	na
chrUn	22744863	22746087	1225	SAT1_CF	blue	na
chrUn	22746148	22746309	162	SAT1_CF	blue	na
chrUn	22746373	22746884	512	SAT1_CF	blue	na
chrUn	22746912	22747048	137	SAT1_CF	blue	na
chrUn	22747134	22747615	482	SAT1_CF	blue	na
chrUn	22747633	22748310	678	SAT1_CF	blue	na
chrUn	22748323	22749655	1333	SAT1_CF	blue	na
chrUn	22749698	22750329	632	SAT1_CF	blue	na
chrUn	22750431	22750958	528	SAT1_CF	blue	na
chrUn	22751000	22751526	527	SAT1_CF	blue	na
chrUn	22751541	22751697	157	SAT1_CF	blue	na
chrUn	22751723	22752243	521	SAT1_CF	blue	na
chrUn	22752256	22752940	685	SAT1_CF	blue	na
chrUn	22752952	22753710	759	SAT1_CF	blue	na
chrUn	22753724	22754395	672	SAT1_CF	blue	na
chrUn	22754438	22755430	993	SAT1_CF	blue	na
chrUn	22755474	22756022	549	SAT1_CF	blue	na
chrUn	22756035	22756699	665	SAT1_CF	blue	na
chrUn	22756742	22757373	632	SAT1_CF	blue	na

chrUn	22757400	22757963	564	SAT1_CF	blue	na
chrUn	22758043	22758309	267	SAT1_CF	blue	na
chrUn	22758375	22758916	542	SAT1_CF	blue	na
chrUn	22758987	22759619	633	SAT1_CF	blue	na
chrUn	22759631	22760672	1042	SAT1_CF	blue	na
chrUn	22760686	22761233	548	SAT1_CF	blue	na
chrUn	22761313	22761800	488	SAT1_CF	blue	na
chrUn	22761856	22762008	153	SAT1_CF	blue	na
chrUn	22762053	22762553	501	SAT1_CF	blue	na
chrUn	22762566	22762674	109	SAT1_CF	blue	na
chrUn	22762695	22763891	1197	SAT1_CF	blue	na
chrUn	22763912	22764711	800	SAT1_CF	blue	na
chrUn	22764766	22765207	442	SAT1_CF	blue	na
chrUn	22766621	22767194	574	SAT1_CF	blue	na
chrUn	22767234	22768430	1197	SAT1_CF	blue	na
chrUn	22768442	22769119	678	SAT1_CF	blue	na
chrUn	22769161	22769793	633	SAT1_CF	blue	na
chrUn	22769821	22770385	565	SAT1_CF	blue	na
chrUn	22770465	22770989	525	SAT1_CF	blue	na
chrUn	22771004	22771161	158	SAT1_CF	blue	na
chrUn	22771206	22771708	503	SAT1_CF	blue	na
chrUn	22771719	22772402	684	SAT1_CF	blue	na
chrUn	22772414	22773086	673	SAT1_CF	blue	na
chrUn	22773133	22773507	375	SAT1_CF	blue	na
chrUn	22773546	22774065	520	SAT1_CF	blue	na
chrUn	22774078	22775669	1592	SAT1_CF	blue	na
chrUn	22775735	22777086	1352	SAT1_CF	blue	na
chrUn	22777111	22777773	663	SAT1_CF	blue	na
chrUn	22777816	22778813	998	SAT1_CF	blue	na
chrUn	22778857	22779813	957	SAT1_CF	blue	na
chrUn	22779856	22780358	503	SAT1_CF	blue	na
chrUn	22780370	22780473	104	SAT1_CF	blue	na
chrUn	22780499	22781053	555	SAT1_CF	blue	na
chrUn	22781077	22781741	665	SAT1_CF	blue	na
chrUn	22781784	22782416	633	SAT1_CF	blue	na
chrUn	22782444	22783006	563	SAT1_CF	blue	na
chrUn	22783086	22783566	481	SAT1_CF	blue	na
chrUn	22783618	22783754	137	SAT1_CF	blue	na
chrUn	22783801	22784320	520	SAT1_CF	blue	na
chrUn	22784333	22784641	309	SAT1_CF	blue	na
chrUn	22784654	22784725	72	SAT1_CF	blue	na
chrUn	22784747	22785188	442	SAT1_CF	blue	na

chrUn	22785229	22785729	501	SAT1_CF	blue	na
chrUn	22785742	22786425	684	SAT1_CF	blue	na
chrUn	22786443	22786561	119	SAT1_CF	blue	na
chrUn	22786572	22788113	1542	SAT1_CF	blue	na
chrUn	22788125	22788801	677	SAT1_CF	blue	na
chrUn	22788814	22789798	985	SAT1_CF	blue	na
chrUn	22789825	22791780	1956	SAT1_CF	blue	na
chrUn	22791808	22791965	158	SAT1_CF	blue	na
chrUn	22791991	22793020	1030	SAT1_CF	blue	na
chrUn	22793032	22794526	1495	SAT1_CF	blue	na
chrUn	22794623	22795718	1096	SAT1_CF	blue	na
chrUn	22795805	22796307	503	SAT1_CF	blue	na
chrUn	22796320	22797004	685	SAT1_CF	blue	na
chrUn	22797016	22797321	306	SAT1_CF	blue	na
chrUn	22797320	22797552	233	CarSat1	red	na
chrUn	22797702	22798549	848	CarSat1	red	na
chrUn	22798579	22799532	954	CarSat1	red	na
chrUn	22824216	22825398	1183	Sat2CF	purple	na
chrUn	22828299	22829828	1530	CarSat1	red	na
chrUn	22829849	22831333	1485	CarSat1	red	na
chrUn	22840426	22840799	374	CarSat1	red	na
chrUn	22841331	22842236	906	CarSat1	red	na
chrUn	22842270	22844051	1782	CarSat1	red	na
chrUn	22844070	22844471	402	CarSat1	red	na
chrUn	22844487	22845649	1163	SAT1_CF	blue	na
chrUn	22852887	22854426	1540	CarSat1	red	na
chrUn	22854635	22855226	592	CarSat1	red	na
chrUn	22855874	22858279	2406	CarSat1	red	na
chrUn	22858295	22862966	4672	CarSat1	red	na
chrUn	22863208	22867585	4378	CarSat1	red	na
chrUn	22867612	22870039	2428	CarSat1	red	na
chrUn	22870063	22870774	712	CarSat1	red	na
chrUn	22870807	22872740	1934	CarSat1	red	na
chrUn	22872767	22873713	947	CarSat1	red	na
chrUn	22873754	22874460	707	CarSat1	red	na
chrUn	22874494	22875679	1186	CarSat1	red	na
chrUn	22875700	22876872	1173	CarSat1	red	na
chrUn	22876894	22877021	128	Carsat2	orange	na
chrUn	22876909	22878141	1233	CarSat1	red	na
chrUn	22878157	22879612	1456	CarSat1	red	na
chrUn	22879645	22880505	861	CarSat1	red	na
chrUn	22880734	22882758	2025	CarSat1	red	na

chrUn	22882774	22889099	6326	CarSat1	red	na
chrUn	22889133	22894521	5389	CarSat1	red	na
chrUn	22894537	22895255	719	CarSat1	red	na
chrUn	22895271	22897461	2191	CarSat1	red	na
chrUn	22897487	22899663	2177	CarSat1	red	na
chrUn	22899687	22900407	721	CarSat1	red	na
chrUn	22900433	22904572	4140	CarSat1	red	na
chrUn	22904599	22905033	435	CarSat1	red	na
chrUn	22905067	22905773	707	CarSat1	red	na
chrUn	22905804	22906290	487	CarSat1	red	na
chrUn	22906314	22908542	2229	CarSat1	red	na
chrUn	22908576	22912003	3428	CarSat1	red	na
chrUn	22912028	22912656	629	CarSat1	red	na
chrUn	22920332	22921104	773	Sat2CF	purple	na
chrUn	22949956	22951114	1159	Sat2CF	purple	na
chrUn	22968406	22969572	1167	Sat2CF	purple	na
chrUn	22978555	22979109	555	Sat2CF	purple	na
chrUn	22978980	22979157	178	Sat2CF	purple	na
chrUn	22994032	22994853	822	Sat2CF	purple	na
chrUn	22994106	22994831	726	Sat2CF	purple	na
chrUn	22994722	22994890	169	Sat2CF	purple	na
chrUn	23007580	23008832	1253	Sat2CF	purple	na
chrUn	23011500	23013040	1541	CarSat1	red	na
chrUn	23013067	23014551	1485	CarSat1	red	na
chrUn	23024047	23024475	429	CarSat1	red	na
chrUn	23024487	23026265	1779	CarSat1	red	na
chrUn	23026284	23026685	402	CarSat1	red	na
chrUn	23026701	23028090	1390	SAT1_CF	blue	na
chrUn	23028285	23028445	161	Sat2CF	purple	na
chrUn	23028296	23028475	180	Sat2CF	purple	na
chrUn	23028319	23028475	157	Sat2CF	purple	na
chrUn	23028681	23029482	802	Sat2CF	purple	na
chrUn	23029166	23029543	378	Sat2CF	purple	na
chrUn	23035569	23037108	1540	CarSat1	red	na
chrUn	23037321	23037913	593	CarSat1	red	na
chrUn	23046465	23047164	700	CarSat1	red	na
chrUn	23047362	23047487	126	CarSat1	red	na
chrUn	23047512	23047973	462	CarSat1	red	na
chrUn	23047990	23048704	715	CarSat1	red	na
chrUn	23048731	23049440	710	CarSat1	red	na
chrUn	23049466	23050176	711	CarSat1	red	na
chrUn	23050203	23050913	711	CarSat1	red	na

chrUn	23050940	23051650	711	CarSat1	red	na
chrUn	23051677	23052387	711	CarSat1	red	na
chrUn	23052414	23053123	710	CarSat1	red	na
chrUn	23053150	23054112	963	CarSat1	red	na
chrUn	23054137	23055335	1199	CarSat1	red	na
chrUn	23055362	23056071	710	CarSat1	red	na
chrUn	23056097	23057059	963	CarSat1	red	na
chrUn	23057075	23057544	470	CarSat1	red	na
chrUn	23057571	23058534	964	CarSat1	red	na
chrUn	23058550	23059270	721	CarSat1	red	na
chrUn	23059301	23060491	1191	CarSat1	red	na
chrUn	23060518	23063010	2493	CarSat1	red	na
chrUn	23063573	23064173	601	CarSat1	red	na
chrUn	23064200	23065843	1644	CarSat1	red	na
chrUn	23067472	23068101	630	CarSat1	red	na
chrUn	23068117	23069575	1459	CarSat1	red	na
chrUn	23069591	23070308	718	CarSat1	red	na
chrUn	23070324	23070793	470	CarSat1	red	na
chrUn	23070820	23071781	962	CarSat1	red	na
chrUn	23071797	23073740	1944	CarSat1	red	na
chrUn	23073767	23074730	964	CarSat1	red	na
chrUn	23074746	23075215	470	CarSat1	red	na
chrUn	23075242	23076204	963	CarSat1	red	na
chrUn	23076220	23078416	2197	CarSat1	red	na
chrUn	23078432	23079153	722	CarSat1	red	na
chrUn	23079169	23081841	2673	CarSat1	red	na
chrUn	23081868	23083041	1174	CarSat1	red	na
chrUn	23083249	23087601	4353	CarSat1	red	na
chrUn	23088029	23088948	920	CarSat1	red	na
chrUn	23088962	23089089	128	Carsat2	orange	na
chrUn	23088977	23089956	980	CarSat1	red	na
chrUn	23089983	23091683	1701	CarSat1	red	na
chrUn	23091699	23094993	3295	CarSat1	red	na
chrUn	23101505	23101821	317	Sat2CF	purple	na
chrUn	23102163	23102611	449	Sat2CF	purple	na
chrUn	23102283	23102664	382	Sat2CF	purple	na
chrUn	23115414	23115648	235	Sat2CF	purple	na
chrUn	23115482	23115830	349	Sat2CF	purple	na
chrUn	23142763	23143929	1167	Sat2CF	purple	na
chrUn	23180897	23182072	1176	Sat2CF	purple	na
chrUn	23223618	23223779	162	Sat2CF	purple	na
chrUn	23223644	23224753	1110	Sat2CF	purple	na

chrUn	23242944	23243896	953	Sat2CF	purple	na
chrUn	23266630	23267368	739	Sat2CF	purple	na
chrUn	23266704	23267366	663	Sat2CF	purple	na
chrUn	23301583	23302834	1252	Sat2CF	purple	na
chrUn	23301713	23302832	1120	Sat2CF	purple	na
chrUn	23357853	23359089	1237	Sat2CF	purple	na
chrUn	23372731	23373862	1132	Sat2CF	purple	na
chrUn	23372735	23374022	1288	Sat2CF	purple	na
chrUn	23389991	23391159	1169	Sat2CF	purple	na
chrUn	23391051	23391230	180	Sat2CF	purple	na
chrUn	23406450	23406830	381	Sat2CF	purple	na
chrUn	23406509	23407295	787	Sat2CF	purple	na
chrUn	23417450	23417809	360	SAT1_CF	blue	na
chrUn	23417852	23418354	503	SAT1_CF	blue	na
chrUn	23418367	23418969	603	SAT1_CF	blue	na
chrUn	23418981	23419656	676	SAT1_CF	blue	na
chrUn	23419699	23421549	1851	SAT1_CF	blue	na
chrUn	23421578	23421735	158	SAT1_CF	blue	na
chrUn	23421780	23422719	940	SAT1_CF	blue	na
chrUn	23422741	23423414	674	SAT1_CF	blue	na
chrUn	23423438	23424981	1544	SAT1_CF	blue	na
chrUn	23424993	23425676	684	SAT1_CF	blue	na
chrUn	23425703	23426409	707	SAT1_CF	blue	na
chrUn	23426432	23428788	2357	SAT1_CF	blue	na
chrUn	23428822	23429614	793	SAT1_CF	blue	na
chrUn	23429626	23431324	1699	SAT1_CF	blue	na
chrUn	23431366	23431556	191	SAT1_CF	blue	na
chrUn	23431587	23432112	526	SAT1_CF	blue	na
chrUn	23432127	23432263	137	SAT1_CF	blue	na
chrUn	23432329	23432831	503	SAT1_CF	blue	na
chrUn	23432844	23432951	108	SAT1_CF	blue	na
chrUn	23432973	23433526	554	SAT1_CF	blue	na
chrUn	23433538	23434300	763	SAT1_CF	blue	na
chrUn	23434312	23435169	858	SAT1_CF	blue	na
chrUn	23435211	23436124	914	SAT1_CF	blue	na
chrUn	23436441	23436942	502	SAT1_CF	blue	na
chrUn	23436955	23437638	684	SAT1_CF	blue	na
chrUn	23437650	23438638	989	SAT1_CF	blue	na
chrUn	23438654	23439177	524	SAT1_CF	blue	na
chrUn	23439220	23439422	203	SAT1_CF	blue	na
chrUn	23439601	23439789	189	SAT1_CF	blue	na
chrUn	23439845	23440404	560	SAT1_CF	blue	na

chrUn	23440466	23441275	810	SAT1_CF	blue	na
chrUn	23441293	23443587	2295	SAT1_CF	blue	na
chrUn	23443631	23444822	1192	SAT1_CF	blue	na
chrUn	23444850	23444986	137	SAT1_CF	blue	na
chrUn	23445052	23445553	502	SAT1_CF	blue	na
chrUn	23445565	23445674	110	SAT1_CF	blue	na
chrUn	23445695	23446325	631	SAT1_CF	blue	na
chrUn	23446343	23447004	662	SAT1_CF	blue	na
chrUn	23447060	23447605	546	SAT1_CF	blue	na
chrUn	23447648	23448192	545	SAT1_CF	blue	na
chrUn	23448214	23449393	1180	SAT1_CF	blue	na
chrUn	23449436	23449975	540	SAT1_CF	blue	na
chrUn	23449988	23451475	1488	SAT1_CF	blue	na
chrUn	23451496	23452623	1128	SAT1_CF	blue	na
chrUn	23452650	23453408	759	SAT1_CF	blue	na
chrUn	23453431	23454096	666	SAT1_CF	blue	na
chrUn	23454140	23456141	2002	SAT1_CF	blue	na
chrUn	23456182	23456683	502	SAT1_CF	blue	na
chrUn	23456701	23457378	678	SAT1_CF	blue	na
chrUn	23457396	23458224	829	SAT1_CF	blue	na
chrUn	23458405	23458580	176	SAT1_CF	blue	na
chrUn	23458626	23459128	503	SAT1_CF	blue	na
chrUn	23459141	23459902	762	SAT1_CF	blue	na
chrUn	23459926	23461663	1738	SAT1_CF	blue	na
chrUn	23461743	23461907	165	SAT1_CF	blue	na
chrUn	23462089	23462274	186	SAT1_CF	blue	na
chrUn	23462330	23462809	480	SAT1_CF	blue	na
chrUn	23462822	23462900	79	SAT1_CF	blue	na
chrUn	23462913	23463392	480	SAT1_CF	blue	na
chrUn	23463413	23463954	542	SAT1_CF	blue	na
chrUn	23464024	23465227	1204	SAT1_CF	blue	na
chrUn	23465271	23465577	307	SAT1_CF	blue	na
chrUn	23465657	23466162	506	SAT1_CF	blue	na
chrUn	23466177	23467498	1322	SAT1_CF	blue	na
chrUn	23467583	23469356	1774	SAT1_CF	blue	na
chrUn	23469426	23469592	167	SAT1_CF	blue	na
chrUn	23469636	23470178	543	SAT1_CF	blue	na
chrUn	23470205	23471741	1537	SAT1_CF	blue	na
chrUn	23471789	23472285	497	SAT1_CF	blue	na
chrUn	23472298	23473520	1223	SAT1_CF	blue	na
chrUn	23473532	23474177	646	SAT1_CF	blue	na
chrUn	23476354	23477476	1123	SAT1_CF	blue	na

chrUn	23477524	23478069	546	SAT1_CF	blue	na
chrUn	23478111	23478219	109	SAT1_CF	blue	na
chrUn	23478264	23479219	956	SAT1_CF	blue	na
chrUn	23479237	23479906	670	SAT1_CF	blue	na
chrUn	23479949	23481937	1989	SAT1_CF	blue	na
chrUn	23482007	23482523	517	SAT1_CF	blue	na
chrUn	23482537	23482644	108	SAT1_CF	blue	na
chrUn	23482666	23483220	555	SAT1_CF	blue	na
chrUn	23483232	23483341	110	SAT1_CF	blue	na
chrUn	23483360	23483912	553	SAT1_CF	blue	na
chrUn	23483923	23484415	493	SAT1_CF	blue	na
chrUn	23484429	23485153	725	SAT1_CF	blue	na
chrUn	23485164	23485407	244	SAT1_CF	blue	na
chrUn	23485434	23486954	1521	SAT1_CF	blue	na
chrUn	23486967	23489550	2584	SAT1_CF	blue	na
chrUn	23489593	23490133	541	SAT1_CF	blue	na
chrUn	23490148	23491245	1098	SAT1_CF	blue	na
chrUn	23491279	23493527	2249	SAT1_CF	blue	na
chrUn	23493563	23494056	494	SAT1_CF	blue	na
chrUn	23494069	23494871	803	SAT1_CF	blue	na
chrUn	23494894	23495440	547	SAT1_CF	blue	na
chrUn	23495487	23496359	873	SAT1_CF	blue	na
chrUn	23496379	23497055	677	SAT1_CF	blue	na
chrUn	23497098	23498837	1740	SAT1_CF	blue	na
chrUn	23498849	23499526	678	SAT1_CF	blue	na
chrUn	23499568	23500114	547	SAT1_CF	blue	na
chrUn	23500126	23501034	909	SAT1_CF	blue	na
chrUn	23501062	23501708	647	SAT1_CF	blue	na
chrUn	23501740	23502429	690	SAT1_CF	blue	na
chrUn	23502465	23502815	351	SAT1_CF	blue	na
chrUn	23502871	23503761	891	SAT1_CF	blue	na
chrUn	23503773	23503856	84	SAT1_CF	blue	na
chrUn	23503869	23504414	546	SAT1_CF	blue	na
chrUn	23504458	23505458	1001	SAT1_CF	blue	na
chrUn	23505490	23506485	996	SAT1_CF	blue	na
chrUn	23506533	23507913	1381	SAT1_CF	blue	na
chrUn	23507955	23508641	687	SAT1_CF	blue	na
chrUn	23508657	23510294	1638	SAT1_CF	blue	na
chrUn	23510316	23510862	547	SAT1_CF	blue	na
chrUn	23510905	23511536	632	SAT1_CF	blue	na
chrUn	23511564	23512206	643	SAT1_CF	blue	na
chrUn	23512271	23514853	2583	SAT1_CF	blue	na

chrUn	23514928	23515509	582	SAT1_CF	blue	na
chrUn	23515521	23516198	678	SAT1_CF	blue	na
chrUn	23516246	23516871	626	SAT1_CF	blue	na
chrUn	23516899	23518711	1813	SAT1_CF	blue	na
chrUn	23518724	23518800	77	SAT1_CF	blue	na
chrUn	23518853	23519424	572	SAT1_CF	blue	na
chrUn	23519436	23520113	678	SAT1_CF	blue	na
chrUn	23520156	23521928	1773	SAT1_CF	blue	na
chrUn	23522015	23522517	503	SAT1_CF	blue	na
chrUn	23522530	23523204	675	SAT1_CF	blue	na
chrUn	23523217	23523827	611	SAT1_CF	blue	na
chrUn	23523871	23524437	567	SAT1_CF	blue	na
chrUn	23524480	23524991	512	SAT1_CF	blue	na
chrUn	23525019	23525260	242	SAT1_CF	blue	na
chrUn	23525325	23525877	553	SAT1_CF	blue	na
chrUn	23525890	23527332	1443	SAT1_CF	blue	na
chrUn	23527360	23527516	157	SAT1_CF	blue	na
chrUn	23527561	23528148	588	SAT1_CF	blue	na
chrUn	23528204	23528965	762	SAT1_CF	blue	na
chrUn	23528978	23530359	1382	SAT1_CF	blue	na
chrUn	23530383	23530992	610	SAT1_CF	blue	na
chrUn	23531071	23532314	1244	SAT1_CF	blue	na
chrUn	23533728	23534300	573	SAT1_CF	blue	na
chrUn	23534323	23535559	1237	SAT1_CF	blue	na
chrUn	23535591	23536112	522	SAT1_CF	blue	na
chrUn	23536127	23536831	705	SAT1_CF	blue	na
chrUn	23536844	23536950	107	SAT1_CF	blue	na
chrUn	23536973	23538800	1828	SAT1_CF	blue	na
chrUn	23538868	23539386	519	SAT1_CF	blue	na
chrUn	23539399	23540002	604	SAT1_CF	blue	na
chrUn	23540014	23540689	676	SAT1_CF	blue	na
chrUn	23540732	23542243	1512	SAT1_CF	blue	na
chrUn	23543639	23544211	573	SAT1_CF	blue	na
chrUn	23544251	23544757	507	SAT1_CF	blue	na
chrUn	23544770	23544876	107	SAT1_CF	blue	na
chrUn	23544898	23545629	732	SAT1_CF	blue	na
chrUn	23545671	23547562	1892	SAT1_CF	blue	na
chrUn	23547583	23548101	519	SAT1_CF	blue	na
chrUn	23548120	23548214	95	SAT1_CF	blue	na
chrUn	23548244	23549872	1629	SAT1_CF	blue	na
chrUn	23549959	23550461	503	SAT1_CF	blue	na
chrUn	23550474	23551268	795	SAT1_CF	blue	na

chrUn	23551281	23551962	682	SAT1_CF	blue	na
chrUn	23551980	23553194	1215	SAT1_CF	blue	na
chrUn	23553299	23556268	2970	SAT1_CF	blue	na
chrUn	23556311	23556814	504	SAT1_CF	blue	na
chrUn	23556851	23556987	137	SAT1_CF	blue	na
chrUn	23557034	23557555	522	SAT1_CF	blue	na
chrUn	23557568	23558737	1170	SAT1_CF	blue	na
chrUn	23558818	23559366	549	SAT1_CF	blue	na
chrUn	23559409	23559611	203	SAT1_CF	blue	na
chrUn	23559791	23559976	186	SAT1_CF	blue	na
chrUn	23560012	23560512	501	SAT1_CF	blue	na
chrUn	23560525	23563293	2769	SAT1_CF	blue	na
chrUn	23563316	23565271	1956	SAT1_CF	blue	na
chrUn	23565338	23566463	1126	SAT1_CF	blue	na
chrUn	23566649	23566981	333	SAT1_CF	blue	na
chrUn	23567013	23567568	556	SAT1_CF	blue	na
chrUn	23567581	23568415	835	SAT1_CF	blue	na
chrUn	23568459	23569235	777	SAT1_CF	blue	na
chrUn	23569247	23569923	677	SAT1_CF	blue	na
chrUn	23570020	23570560	541	SAT1_CF	blue	na
chrUn	23570585	23570945	361	SAT1_CF	blue	na
chrUn	23570987	23571494	508	SAT1_CF	blue	na
chrUn	23571508	23571910	403	SAT1_CF	blue	na
chrUn	23571948	23572563	616	SAT1_CF	blue	na
chrUn	23572592	23573220	629	SAT1_CF	blue	na
chrUn	23573244	23574104	861	SAT1_CF	blue	na
chrUn	23574131	23574807	677	SAT1_CF	blue	na
chrUn	23574819	23575669	851	SAT1_CF	blue	na
chrUn	23575682	23576298	617	SAT1_CF	blue	na
chrUn	23576340	23577158	819	SAT1_CF	blue	na
chrUn	23577189	23577568	380	SAT1_CF	blue	na
chrUn	23577610	23578108	499	SAT1_CF	blue	na
chrUn	23578125	23578880	756	SAT1_CF	blue	na
chrUn	23578894	23579632	739	SAT1_CF	blue	na
chrUn	23579645	23580366	722	SAT1_CF	blue	na
chrUn	23580394	23580900	507	SAT1_CF	blue	na
chrUn	23580935	23581799	865	SAT1_CF	blue	na
chrUn	23581812	23582670	859	SAT1_CF	blue	na
chrUn	23582684	23583750	1067	SAT1_CF	blue	na
chrUn	23583768	23585256	1489	SAT1_CF	blue	na
chrUn	23585326	23585879	554	SAT1_CF	blue	na
chrUn	23585892	23586531	640	SAT1_CF	blue	na

chrUn	23586574	23588454	1881	SAT1_CF	blue	na
chrUn	23588468	23588544	77	SAT1_CF	blue	na
chrUn	23588595	23589137	543	SAT1_CF	blue	na
chrUn	23589149	23590175	1027	SAT1_CF	blue	na
chrUn	23590217	23591267	1051	SAT1_CF	blue	na
chrUn	23591298	23591810	513	SAT1_CF	blue	na
chrUn	23591838	23592795	958	SAT1_CF	blue	na
chrUn	23592808	23593482	675	SAT1_CF	blue	na
chrUn	23593525	23594073	549	SAT1_CF	blue	na
chrUn	23594106	23594623	518	SAT1_CF	blue	na
chrUn	23594709	23595210	502	SAT1_CF	blue	na
chrUn	23595223	23595906	684	SAT1_CF	blue	na
chrUn	23595918	23596677	760	SAT1_CF	blue	na
chrUn	23596691	23597647	957	SAT1_CF	blue	na
chrUn	23597689	23598191	503	SAT1_CF	blue	na
chrUn	23598204	23598309	106	SAT1_CF	blue	na
chrUn	23598332	23599480	1149	SAT1_CF	blue	na
chrUn	23599492	23602387	2896	SAT1_CF	blue	na
chrUn	23602430	23602803	374	SAT1_CF	blue	na
chrUn	23602820	23603330	511	SAT1_CF	blue	na
chrUn	23603357	23604113	757	SAT1_CF	blue	na
chrUn	23604126	23605841	1716	SAT1_CF	blue	na
chrUn	23605885	23607085	1201	SAT1_CF	blue	na
chrUn	23607165	23607329	165	SAT1_CF	blue	na
chrUn	23607510	23607695	186	SAT1_CF	blue	na
chrUn	23607731	23608233	503	SAT1_CF	blue	na
chrUn	23608246	23609007	762	SAT1_CF	blue	na
chrUn	23609031	23609688	658	SAT1_CF	blue	na
chrUn	23609715	23610945	1231	SAT1_CF	blue	na
chrUn	23610975	23613239	2265	SAT1_CF	blue	na
chrUn	23613269	23613651	383	SAT1_CF	blue	na
chrUn	23613689	23614186	498	SAT1_CF	blue	na
chrUn	23614204	23614307	104	SAT1_CF	blue	na
chrUn	23614328	23614902	575	SAT1_CF	blue	na
chrUn	23614914	23615574	661	SAT1_CF	blue	na
chrUn	23615622	23616155	534	SAT1_CF	blue	na
chrUn	23616184	23616849	666	SAT1_CF	blue	na
chrUn	23616893	23618269	1377	SAT1_CF	blue	na
chrUn	23618461	23619736	1276	SAT1_CF	blue	na
chrUn	23619760	23620907	1148	SAT1_CF	blue	na
chrUn	23620988	23621536	549	SAT1_CF	blue	na
chrUn	23621616	23621780	165	SAT1_CF	blue	na

chrUn	23621962	23622147	186	SAT1_CF	blue	na
chrUn	23622183	23622682	500	SAT1_CF	blue	na
chrUn	23622695	23623308	614	SAT1_CF	blue	na
chrUn	23623320	23624180	861	SAT1_CF	blue	na
chrUn	23624223	23625003	781	SAT1_CF	blue	na
chrUn	23625047	23625589	543	SAT1_CF	blue	na
chrUn	23625611	23626281	671	SAT1_CF	blue	na
chrUn	23626293	23627629	1337	SAT1_CF	blue	na
chrUn	23627654	23628379	726	SAT1_CF	blue	na
chrUn	23628422	23628970	549	SAT1_CF	blue	na
chrUn	23628982	23629651	670	SAT1_CF	blue	na
chrUn	23629663	23630526	864	SAT1_CF	blue	na
chrUn	23630539	23631903	1365	SAT1_CF	blue	na
chrUn	23631948	23632576	629	SAT1_CF	blue	na
chrUn	23632604	23633166	563	SAT1_CF	blue	na
chrUn	23633208	23633731	524	SAT1_CF	blue	na
chrUn	23633746	23633882	137	SAT1_CF	blue	na
chrUn	23633967	23634772	806	SAT1_CF	blue	na
chrUn	23634792	23635567	776	SAT1_CF	blue	na
chrUn	23635614	23636138	525	SAT1_CF	blue	na
chrUn	23636153	23636309	157	SAT1_CF	blue	na
chrUn	23636355	23636853	499	SAT1_CF	blue	na
chrUn	23636871	23636974	104	SAT1_CF	blue	na
chrUn	23636995	23637569	575	SAT1_CF	blue	na
chrUn	23637581	23638241	661	SAT1_CF	blue	na
chrUn	23638289	23638829	541	SAT1_CF	blue	na
chrUn	23638851	23639516	666	SAT1_CF	blue	na
chrUn	23639560	23640062	503	SAT1_CF	blue	na
chrUn	23640088	23640636	549	SAT1_CF	blue	na
chrUn	23640680	23642085	1406	SAT1_CF	blue	na
chrUn	23642165	23642689	525	SAT1_CF	blue	na
chrUn	23642704	23643405	702	SAT1_CF	blue	na
chrUn	23643418	23643523	106	SAT1_CF	blue	na
chrUn	23643546	23645335	1790	SAT1_CF	blue	na
chrUn	23645367	23645999	633	SAT1_CF	blue	na
chrUn	23646010	23646404	395	SAT1_CF	blue	na
chrUn	23646470	23646948	479	SAT1_CF	blue	na
chrUn	23646961	23647645	685	SAT1_CF	blue	na
chrUn	23647657	23648498	842	SAT1_CF	blue	na
chrUn	23648540	23649907	1368	SAT1_CF	blue	na
chrUn	23649921	23651323	1403	SAT1_CF	blue	na
chrUn	23651337	23651732	396	SAT1_CF	blue	na

chrUn	23651796	23653010	1215	SAT1_CF	blue	na
chrUn	23653022	23653130	109	SAT1_CF	blue	na
chrUn	23653151	23653702	552	SAT1_CF	blue	na
chrUn	23653751	23654266	516	SAT1_CF	blue	na
chrUn	23654281	23655107	827	SAT1_CF	blue	na
chrUn	23655150	23655772	623	SAT1_CF	blue	na
chrUn	23655802	23657117	1316	SAT1_CF	blue	na
chrUn	23657155	23657655	501	SAT1_CF	blue	na
chrUn	23657667	23658343	677	SAT1_CF	blue	na
chrUn	23658386	23659009	624	SAT1_CF	blue	na
chrUn	23659048	23659571	524	SAT1_CF	blue	na
chrUn	23659586	23659742	157	SAT1_CF	blue	na
chrUn	23659787	23660281	495	SAT1_CF	blue	na
chrUn	23660301	23660984	684	SAT1_CF	blue	na
chrUn	23660996	23661671	676	SAT1_CF	blue	na
chrUn	23661713	23662723	1011	SAT1_CF	blue	na
chrUn	23662755	23663763	1009	SAT1_CF	blue	na
chrUn	23663803	23664304	502	SAT1_CF	blue	na
chrUn	23664317	23664954	638	SAT1_CF	blue	na
chrUn	23664969	23665580	612	SAT1_CF	blue	na
chrUn	23665594	23665782	189	SAT1_CF	blue	na
chrUn	23665834	23666535	702	SAT1_CF	blue	na
chrUn	23666547	23666653	107	SAT1_CF	blue	na
chrUn	23666677	23667227	551	SAT1_CF	blue	na
chrUn	23667250	23668111	862	SAT1_CF	blue	na
chrUn	23668129	23668815	687	SAT1_CF	blue	na
chrUn	23668827	23669677	851	SAT1_CF	blue	na
chrUn	23669690	23670435	746	SAT1_CF	blue	na
chrUn	23670474	23671607	1134	SAT1_CF	blue	na
chrUn	23671724	23672242	519	SAT1_CF	blue	na
chrUn	23672299	23672845	547	SAT1_CF	blue	na
chrUn	23672888	23673430	543	SAT1_CF	blue	na
chrUn	23673472	23673752	281	SAT1_CF	blue	na
chrUn	23673783	23674704	922	SAT1_CF	blue	na
chrUn	23674744	23675245	502	SAT1_CF	blue	na
chrUn	23675272	23676019	748	SAT1_CF	blue	na
chrUn	23676032	23677583	1552	SAT1_CF	blue	na
chrUn	23677651	23677904	254	SAT1_CF	blue	na
chrUn	23677920	23679594	1675	SAT1_CF	blue	na
chrUn	23679639	23680627	989	SAT1_CF	blue	na
chrUn	23680684	23681594	911	SAT1_CF	blue	na
chrUn	23681681	23682181	501	SAT1_CF	blue	na

chrUn	23682208	23682983	776	SAT1_CF	blue	na
chrUn	23684089	23684335	247	SAT1_CF	blue	na
chrUn	23684354	23684878	525	SAT1_CF	blue	na
chrUn	23684935	23686041	1107	SAT1_CF	blue	na
chrUn	23686253	23686408	156	SAT1_CF	blue	na
chrUn	23686453	23686952	500	SAT1_CF	blue	na
chrUn	23686965	23687647	683	SAT1_CF	blue	na
chrUn	23687690	23688236	547	SAT1_CF	blue	na
chrUn	23688278	23688945	668	SAT1_CF	blue	na
chrUn	23688976	23690099	1124	SAT1_CF	blue	na
chrUn	23690120	23690640	521	SAT1_CF	blue	na
chrUn	23690652	23690902	251	SAT1_CF	blue	na
chrUn	23692395	23692820	426	SAT1_CF	blue	na
chrUn	23692899	23693423	525	SAT1_CF	blue	na
chrUn	23693438	23693595	158	SAT1_CF	blue	na
chrUn	23693640	23694135	496	SAT1_CF	blue	na
chrUn	23694155	23696047	1893	SAT1_CF	blue	na
chrUn	23696061	23697038	978	SAT1_CF	blue	na
chrUn	23697103	23697658	556	SAT1_CF	blue	na
chrUn	23697670	23698532	863	SAT1_CF	blue	na
chrUn	23698609	23699485	877	SAT1_CF	blue	na
chrUn	23699506	23700062	557	SAT1_CF	blue	na
chrUn	23700073	23700181	109	SAT1_CF	blue	na
chrUn	23700207	23700727	521	SAT1_CF	blue	na
chrUn	23700740	23701346	607	SAT1_CF	blue	na
chrUn	23701358	23702033	676	SAT1_CF	blue	na
chrUn	23702049	23702732	684	SAT1_CF	blue	na
chrUn	23702771	23702916	146	SAT1_CF	blue	na
chrUn	23702941	23703384	444	SAT1_CF	blue	na
chrUn	23703420	23703975	556	SAT1_CF	blue	na
chrUn	23703987	23704661	675	SAT1_CF	blue	na
chrUn	23704705	23705665	961	SAT1_CF	blue	na
chrUn	23705745	23706659	915	SAT1_CF	blue	na
chrUn	23706746	23707247	502	SAT1_CF	blue	na
chrUn	23707259	23707364	106	SAT1_CF	blue	na
chrUn	23707388	23708190	803	SAT1_CF	blue	na
chrUn	23708202	23708311	110	SAT1_CF	blue	na
chrUn	23708332	23709003	672	SAT1_CF	blue	na
chrUn	23709025	23709567	543	SAT1_CF	blue	na
chrUn	23709611	23710154	544	SAT1_CF	blue	na
chrUn	23710167	23710858	692	SAT1_CF	blue	na
chrUn	23710870	23711720	851	SAT1_CF	blue	na

chrUn	23711733	23712349	617	SAT1_CF	blue	na
chrUn	23712391	23713214	824	SAT1_CF	blue	na
chrUn	23713259	23713623	365	SAT1_CF	blue	na
chrUn	23713695	23714214	520	SAT1_CF	blue	na
chrUn	23714271	23714817	547	SAT1_CF	blue	na
chrUn	23714859	23716165	1307	SAT1_CF	blue	na
chrUn	23716187	23716705	519	SAT1_CF	blue	na
chrUn	23716717	23718034	1318	SAT1_CF	blue	na
chrUn	23718047	23718882	836	SAT1_CF	blue	na
chrUn	23718895	23719598	704	SAT1_CF	blue	na
chrUn	23719610	23721734	2125	SAT1_CF	blue	na
chrUn	23721767	23722146	380	SAT1_CF	blue	na
chrUn	23722186	23723924	1739	SAT1_CF	blue	na
chrUn	23723985	23724146	162	SAT1_CF	blue	na
chrUn	23724207	23724718	512	SAT1_CF	blue	na
chrUn	23724746	23724888	143	SAT1_CF	blue	na
chrUn	23724948	23725448	501	SAT1_CF	blue	na
chrUn	23725461	23726215	755	SAT1_CF	blue	na
chrUn	23726235	23728047	1813	SAT1_CF	blue	na
chrUn	23728060	23728462	403	SAT1_CF	blue	na
chrUn	23728502	23729004	503	SAT1_CF	blue	na
chrUn	23729017	23730895	1879	SAT1_CF	blue	na
chrUn	23731012	23731575	564	SAT1_CF	blue	na
chrUn	23731617	23732396	780	SAT1_CF	blue	na
chrUn	23732440	23732984	545	SAT1_CF	blue	na
chrUn	23732997	23733506	510	SAT1_CF	blue	na
chrUn	23733519	23733590	72	SAT1_CF	blue	na
chrUn	23733613	23734764	1152	SAT1_CF	blue	na
chrUn	23734794	23735353	560	SAT1_CF	blue	na
chrUn	23735440	23735940	501	SAT1_CF	blue	na
chrUn	23735958	23736712	755	SAT1_CF	blue	na
chrUn	23736724	23738058	1335	SAT1_CF	blue	na
chrUn	23738083	23738405	323	SAT1_CF	blue	na
chrUn	23738485	23739007	523	SAT1_CF	blue	na
chrUn	23739223	23739377	155	SAT1_CF	blue	na
chrUn	23739403	23740619	1217	SAT1_CF	blue	na
chrUn	23740631	23742395	1765	SAT1_CF	blue	na
chrUn	23742447	23743065	619	SAT1_CF	blue	na
chrUn	23743114	23743741	628	SAT1_CF	blue	na
chrUn	23743753	23744623	871	SAT1_CF	blue	na
chrUn	23744641	23745327	687	SAT1_CF	blue	na
chrUn	23745339	23746189	851	SAT1_CF	blue	na

chrUn	23746202	23747683	1482	SAT1_CF	blue	na
chrUn	23747728	23748152	425	SAT1_CF	blue	na
chrUn	23748164	23748683	520	SAT1_CF	blue	na
chrUn	23748740	23749286	547	SAT1_CF	blue	na
chrUn	23749328	23750174	847	SAT1_CF	blue	na
chrUn	23750207	23751094	888	SAT1_CF	blue	na
chrUn	23751181	23751675	495	SAT1_CF	blue	na
chrUn	23751700	23751807	108	SAT1_CF	blue	na
chrUn	23751824	23752540	717	SAT1_CF	blue	na
chrUn	23752563	23753080	518	SAT1_CF	blue	na
chrUn	23753113	23753880	768	SAT1_CF	blue	na
chrUn	23753957	23755727	1771	SAT1_CF	blue	na
chrUn	23755814	23756311	498	SAT1_CF	blue	na
chrUn	23756329	23756431	103	SAT1_CF	blue	na
chrUn	23756452	23757007	556	SAT1_CF	blue	na
chrUn	23757020	23758292	1273	SAT1_CF	blue	na
chrUn	23758377	23758879	503	SAT1_CF	blue	na
chrUn	23758892	23758998	107	SAT1_CF	blue	na
chrUn	23759020	23759565	546	SAT1_CF	blue	na
chrUn	23759635	23760098	464	SAT1_CF	blue	na
chrUn	23760110	23761625	1516	SAT1_CF	blue	na
chrUn	23761637	23762606	970	SAT1_CF	blue	na
chrUn	23762659	23764267	1609	SAT1_CF	blue	na
chrUn	23764300	23764458	159	SAT1_CF	blue	na
chrUn	23764483	23765003	521	SAT1_CF	blue	na
chrUn	23765021	23766413	1393	SAT1_CF	blue	na
chrUn	23766480	23767549	1070	SAT1_CF	blue	na
chrUn	23767561	23768450	890	SAT1_CF	blue	na
chrUn	23768464	23769832	1369	SAT1_CF	blue	na
chrUn	23769878	23770396	519	SAT1_CF	blue	na
chrUn	23770435	23770580	146	SAT1_CF	blue	na
chrUn	23770640	23771189	550	SAT1_CF	blue	na
chrUn	23771232	23771602	371	SAT1_CF	blue	na
chrUn	23771659	23772138	480	SAT1_CF	blue	na
chrUn	23772150	23772833	684	SAT1_CF	blue	na
chrUn	23772845	23774799	1955	SAT1_CF	blue	na
chrUn	23774843	23775635	793	SAT1_CF	blue	na
chrUn	23775816	23775983	168	SAT1_CF	blue	na
chrUn	23776057	23776527	471	SAT1_CF	blue	na
chrUn	23776550	23777874	1325	SAT1_CF	blue	na
chrUn	23777889	23778046	158	SAT1_CF	blue	na
chrUn	23778072	23779991	1920	SAT1_CF	blue	na

chrUn	23780019	23781236	1218	SAT1_CF	blue	na
chrUn	23781418	23781599	182	SAT1_CF	blue	na
chrUn	23781659	23782132	474	SAT1_CF	blue	na
chrUn	23782145	23782250	106	SAT1_CF	blue	na
chrUn	23782274	23783332	1059	SAT1_CF	blue	na
chrUn	23783345	23783846	502	SAT1_CF	blue	na
chrUn	23783864	23784804	941	SAT1_CF	blue	na
chrUn	23784825	23785344	520	SAT1_CF	blue	na
chrUn	23785355	23785710	356	SAT1_CF	blue	na
chrUn	23785797	23786885	1089	SAT1_CF	blue	na
chrUn	23786903	23787572	670	SAT1_CF	blue	na
chrUn	23787615	23788577	963	SAT1_CF	blue	na
chrUn	23788655	23789163	509	SAT1_CF	blue	na
chrUn	23789246	23789827	582	SAT1_CF	blue	na
chrUn	23789849	23790600	752	SAT1_CF	blue	na
chrUn	23790613	23791229	617	SAT1_CF	blue	na
chrUn	23791442	23791597	156	SAT1_CF	blue	na
chrUn	23791623	23792141	519	SAT1_CF	blue	na
chrUn	23792159	23792842	684	SAT1_CF	blue	na
chrUn	23792886	23794184	1299	SAT1_CF	blue	na
chrUn	23794264	23794788	525	SAT1_CF	blue	na
chrUn	23794803	23794960	158	SAT1_CF	blue	na
chrUn	23795025	23795956	932	SAT1_CF	blue	na
chrUn	23795969	23796641	673	SAT1_CF	blue	na
chrUn	23796653	23796820	168	SAT1_CF	blue	na
chrUn	23796833	23796873	41	SAT1_CF	blue	na
chrUn	23796905	23797984	1080	SAT1_CF	blue	na
chrUn	23798027	23798581	555	SAT1_CF	blue	na
chrUn	23798593	23799855	1263	SAT1_CF	blue	na
chrUn	23799889	23800408	520	SAT1_CF	blue	na
chrUn	23800420	23801106	687	SAT1_CF	blue	na
chrUn	23801118	23801226	109	SAT1_CF	blue	na
chrUn	23801246	23803494	2249	SAT1_CF	blue	na
chrUn	23803507	23804189	683	SAT1_CF	blue	na
chrUn	23804213	23805899	1687	SAT1_CF	blue	na
chrUn	23805944	23806973	1030	SAT1_CF	blue	na
chrUn	23807033	23807603	571	SAT1_CF	blue	na
chrUn	23807622	23809111	1490	SAT1_CF	blue	na
chrUn	23809190	23810432	1243	SAT1_CF	blue	na
chrUn	23810476	23811698	1223	SAT1_CF	blue	na
chrUn	23811725	23812288	564	SAT1_CF	blue	na
chrUn	23812368	23813955	1588	SAT1_CF	blue	na

chrUn	23813967	23815126	1160	SAT1_CF	blue	na
chrUn	23815207	23815755	549	SAT1_CF	blue	na
chrUn	23815835	23816000	166	SAT1_CF	blue	na
chrUn	23816181	23816366	186	SAT1_CF	blue	na
chrUn	23816383	23816902	520	SAT1_CF	blue	na
chrUn	23816915	23817020	106	SAT1_CF	blue	na
chrUn	23817043	23818191	1149	SAT1_CF	blue	na
chrUn	23818223	23818739	517	SAT1_CF	blue	na
chrUn	23818752	23820123	1372	SAT1_CF	blue	na
chrUn	23820135	23820307	173	SAT1_CF	blue	na
chrUn	23820349	23821156	808	SAT1_CF	blue	na
chrUn	23821200	23821721	522	SAT1_CF	blue	na
chrUn	23821754	23821966	213	SAT1_CF	blue	na
chrUn	23822145	23822312	168	SAT1_CF	blue	na
chrUn	23822386	23822857	472	SAT1_CF	blue	na
chrUn	23822880	23824203	1324	SAT1_CF	blue	na
chrUn	23824218	23824375	158	SAT1_CF	blue	na
chrUn	23824401	23825795	1395	SAT1_CF	blue	na
chrUn	23825873	23826654	782	SAT1_CF	blue	na
chrUn	23826709	23827276	568	SAT1_CF	blue	na
chrUn	23827292	23827677	386	SAT1_CF	blue	na
chrUn	23827711	23828230	520	SAT1_CF	blue	na
chrUn	23828242	23828918	677	SAT1_CF	blue	na
chrUn	23828962	23830184	1223	SAT1_CF	blue	na
chrUn	23830264	23830774	511	SAT1_CF	blue	na
chrUn	23830802	23831431	630	SAT1_CF	blue	na
chrUn	23831443	23832117	675	SAT1_CF	blue	na
chrUn	23832160	23834023	1864	SAT1_CF	blue	na
chrUn	23834038	23834743	706	SAT1_CF	blue	na
chrUn	23834779	23835433	655	SAT1_CF	blue	na
chrUn	23835453	23836824	1372	SAT1_CF	blue	na
chrUn	23836866	23837405	540	SAT1_CF	blue	na
chrUn	23837450	23839864	2415	SAT1_CF	blue	na
chrUn	23839878	23839985	108	SAT1_CF	blue	na
chrUn	23840007	23840287	281	SAT1_CF	blue	na
chrUn	23840471	23840753	283	SAT1_CF	blue	na
chrUn	23840796	23841701	906	SAT1_CF	blue	na
chrUn	23841754	23842256	503	SAT1_CF	blue	na
chrUn	23842280	23842942	663	SAT1_CF	blue	na
chrUn	23842985	23843711	727	SAT1_CF	blue	na
chrUn	23843756	23844291	536	SAT1_CF	blue	na
chrUn	23844313	23844830	518	SAT1_CF	blue	na

chrUn	23844842	23846159	1318	SAT1_CF	blue	na
chrUn	23846172	23846360	189	SAT1_CF	blue	na
chrUn	23846463	23847007	545	SAT1_CF	blue	na
chrUn	23847020	23847722	703	SAT1_CF	blue	na
chrUn	23847734	23849678	1945	SAT1_CF	blue	na
chrUn	23849709	23850226	518	SAT1_CF	blue	na
chrUn	23850313	23850814	502	SAT1_CF	blue	na
chrUn	23850827	23852050	1224	SAT1_CF	blue	na
chrUn	23852111	23852272	162	SAT1_CF	blue	na
chrUn	23852333	23852843	511	SAT1_CF	blue	na
chrUn	23852871	23853026	156	SAT1_CF	blue	na
chrUn	23853072	23853574	503	SAT1_CF	blue	na
chrUn	23853586	23855069	1484	SAT1_CF	blue	na
chrUn	23855148	23855672	525	SAT1_CF	blue	na
chrUn	23855687	23856390	704	SAT1_CF	blue	na
chrUn	23856403	23857012	610	SAT1_CF	blue	na
chrUn	23857030	23857882	853	SAT1_CF	blue	na
chrUn	23857924	23859733	1810	SAT1_CF	blue	na
chrUn	23859801	23860321	521	SAT1_CF	blue	na
chrUn	23860333	23860449	117	SAT1_CF	blue	na
chrUn	23860463	23861173	711	SAT1_CF	blue	na
chrUn	23861185	23862773	1589	SAT1_CF	blue	na
chrUn	23862804	23863313	510	SAT1_CF	blue	na
chrUn	23863324	23863723	400	SAT1_CF	blue	na
chrUn	23863766	23864266	501	SAT1_CF	blue	na
chrUn	23864279	23865720	1442	SAT1_CF	blue	na
chrUn	23865788	23866307	520	SAT1_CF	blue	na
chrUn	23866320	23867003	684	SAT1_CF	blue	na
chrUn	23867015	23867677	663	SAT1_CF	blue	na
chrUn	23867720	23868347	628	SAT1_CF	blue	na
chrUn	23868375	23869567	1193	SAT1_CF	blue	na
chrUn	23869595	23870294	700	SAT1_CF	blue	na
chrUn	23870307	23870989	683	SAT1_CF	blue	na
chrUn	23871000	23871685	686	SAT1_CF	blue	na
chrUn	23871697	23874379	2683	SAT1_CF	blue	na
chrUn	23874488	23874987	500	SAT1_CF	blue	na
chrUn	23875005	23875673	669	SAT1_CF	blue	na
chrUn	23875744	23876207	464	SAT1_CF	blue	na
chrUn	23876219	23877157	939	SAT1_CF	blue	na
chrUn	23877178	23877733	556	SAT1_CF	blue	na
chrUn	23877745	23878567	823	SAT1_CF	blue	na
chrUn	23878579	23879202	624	SAT1_CF	blue	na

chrUn	23879230	23880393	1164	SAT1_CF	blue	na
chrUn	23880445	23880581	137	SAT1_CF	blue	na
chrUn	23880647	23881140	494	SAT1_CF	blue	na
chrUn	23881174	23881800	627	SAT1_CF	blue	na
chrUn	23882061	23882760	700	SAT1_CF	blue	na
chrUn	23882774	23883283	510	SAT1_CF	blue	na
chrUn	23883296	23883397	102	SAT1_CF	blue	na
chrUn	23883425	23883980	556	SAT1_CF	blue	na
chrUn	23883991	23884483	493	SAT1_CF	blue	na
chrUn	23884497	23885878	1382	SAT1_CF	blue	na
chrUn	23885906	23886469	564	SAT1_CF	blue	na
chrUn	23886549	23887073	525	SAT1_CF	blue	na
chrUn	23887088	23887244	157	SAT1_CF	blue	na
chrUn	23887289	23887879	591	SAT1_CF	blue	na
chrUn	23887897	23888289	393	SAT1_CF	blue	na
chrUn	23889688	23890264	577	SAT1_CF	blue	na
chrUn	23890323	23890805	483	SAT1_CF	blue	na
chrUn	23890818	23892153	1336	SAT1_CF	blue	na
chrUn	23892197	23892621	425	SAT1_CF	blue	na
chrUn	23892639	23893525	887	SAT1_CF	blue	na
chrUn	23893595	23894113	519	SAT1_CF	blue	na
chrUn	23894141	23895503	1363	SAT1_CF	blue	na
chrUn	23895531	23896549	1019	SAT1_CF	blue	na
chrUn	23896614	23897175	562	SAT1_CF	blue	na
chrUn	23898587	23899264	678	SAT1_CF	blue	na
chrUn	23899308	23900446	1139	SAT1_CF	blue	na
chrUn	23900512	23901011	500	SAT1_CF	blue	na
chrUn	23901023	23901131	109	SAT1_CF	blue	na
chrUn	23901154	23901709	556	SAT1_CF	blue	na
chrUn	23901721	23901830	110	SAT1_CF	blue	na
chrUn	23901850	23902111	262	SAT1_CF	blue	na
chrUn	23902606	23903525	920	SAT1_CF	blue	na
chrUn	23903561	23904071	511	SAT1_CF	blue	na
chrUn	23904103	23904483	381	SAT1_CF	blue	na
chrUn	23904524	23905025	502	SAT1_CF	blue	na
chrUn	23905038	23906229	1192	SAT1_CF	blue	na
chrUn	23906241	23908273	2033	SAT1_CF	blue	na
chrUn	23908455	23908636	182	SAT1_CF	blue	na
chrUn	23908676	23909255	580	SAT1_CF	blue	na
chrUn	23909317	23910588	1272	SAT1_CF	blue	na
chrUn	23910603	23910745	143	SAT1_CF	blue	na
chrUn	23910805	23911382	578	SAT1_CF	blue	na

chrUn	23911404	23911502	99	SAT1_CF	blue	na
chrUn	23911523	23912076	554	SAT1_CF	blue	na
chrUn	23912088	23912949	862	SAT1_CF	blue	na
chrUn	23912991	23913621	631	SAT1_CF	blue	na
chrUn	23913649	23914907	1259	SAT1_CF	blue	na
chrUn	23914925	23915611	687	SAT1_CF	blue	na
chrUn	23915623	23916472	850	SAT1_CF	blue	na
chrUn	23916522	23918312	1791	SAT1_CF	blue	na
chrUn	23918348	23918848	501	SAT1_CF	blue	na
chrUn	23918861	23918968	108	SAT1_CF	blue	na
chrUn	23918990	23919544	555	SAT1_CF	blue	na
chrUn	23919556	23920859	1304	SAT1_CF	blue	na
chrUn	23920871	23921035	165	SAT1_CF	blue	na
chrUn	23921083	23921864	782	SAT1_CF	blue	na
chrUn	23921930	23922453	524	SAT1_CF	blue	na
chrUn	23922500	23922902	403	SAT1_CF	blue	na
chrUn	23922942	23923910	969	SAT1_CF	blue	na
chrUn	23923922	23924606	685	SAT1_CF	blue	na
chrUn	23924619	23925451	833	SAT1_CF	blue	na
chrUn	23926494	23927001	508	SAT1_CF	blue	na
chrUn	23927019	23927781	763	SAT1_CF	blue	na
chrUn	23927793	23928467	675	SAT1_CF	blue	na
chrUn	23928510	23930537	2028	SAT1_CF	blue	na
chrUn	23930590	23931092	503	SAT1_CF	blue	na
chrUn	23931109	23931187	79	SAT1_CF	blue	na
chrUn	23931234	23933134	1901	SAT1_CF	blue	na
chrUn	23933158	23933400	243	SAT1_CF	blue	na
chrUn	23933451	23934000	550	SAT1_CF	blue	na
chrUn	23934011	23934086	76	SAT1_CF	blue	na
chrUn	23934142	23934687	546	SAT1_CF	blue	na
chrUn	23934699	23935783	1085	SAT1_CF	blue	na
chrUn	23935838	23936340	503	SAT1_CF	blue	na
chrUn	23936353	23937233	881	SAT1_CF	blue	na
chrUn	23937414	23937621	208	SAT1_CF	blue	na
chrUn	23937634	23938134	501	SAT1_CF	blue	na
chrUn	23938147	23939288	1142	SAT1_CF	blue	na
chrUn	23939310	23941522	2213	SAT1_CF	blue	na
chrUn	23941535	23941932	398	SAT1_CF	blue	na
chrUn	23941953	23942474	522	SAT1_CF	blue	na
chrUn	23942487	23944834	2348	SAT1_CF	blue	na
chrUn	23944913	23945461	549	SAT1_CF	blue	na
chrUn	23945473	23945873	401	SAT1_CF	blue	na

chrUn	23945892	23946410	519	SAT1_CF	blue	na
chrUn	23946423	23947112	690	SAT1_CF	blue	na
chrUn	23947124	23947950	827	SAT1_CF	blue	na
chrUn	23947963	23948822	860	SAT1_CF	blue	na
chrUn	23948864	23949405	542	SAT1_CF	blue	na
chrUn	23949417	23950685	1269	SAT1_CF	blue	na
chrUn	23950696	23951051	356	SAT1_CF	blue	na
chrUn	23951168	23951710	543	SAT1_CF	blue	na
chrUn	23951722	23952406	685	SAT1_CF	blue	na
chrUn	23952418	23953102	685	SAT1_CF	blue	na
chrUn	23953115	23953954	840	SAT1_CF	blue	na
chrUn	23953967	23955154	1188	SAT1_CF	blue	na
chrUn	23955166	23956325	1160	SAT1_CF	blue	na
chrUn	23956345	23956952	608	SAT1_CF	blue	na
chrUn	23957032	23957196	165	SAT1_CF	blue	na
chrUn	23957378	23957562	185	SAT1_CF	blue	na
chrUn	23957598	23958165	568	SAT1_CF	blue	na
chrUn	23958227	23959529	1303	SAT1_CF	blue	na
chrUn	23959541	23960403	863	SAT1_CF	blue	na
chrUn	23960445	23961076	632	SAT1_CF	blue	na
chrUn	23961104	23961593	490	SAT1_CF	blue	na
chrUn	23961604	23962108	505	SAT1_CF	blue	na
chrUn	23962142	23962491	350	SAT1_CF	blue	na
chrUn	23962547	23963047	501	SAT1_CF	blue	na
chrUn	23963061	23964389	1329	SAT1_CF	blue	na
chrUn	23964549	23964689	141	SAT1_CF	blue	na
chrUn	23964710	23965244	535	SAT1_CF	blue	na
chrUn	23965255	23965743	489	SAT1_CF	blue	na
chrUn	23965811	23966438	628	SAT1_CF	blue	na
chrUn	23968110	23968312	203	SAT1_CF	blue	na
chrUn	23968525	23968682	158	SAT1_CF	blue	na
chrUn	23968727	23969343	617	SAT1_CF	blue	na
chrUn	23969368	23969914	547	SAT1_CF	blue	na
chrUn	23969957	23971820	1864	SAT1_CF	blue	na
chrUn	23971835	23972693	859	SAT1_CF	blue	na
chrUn	23972705	23972818	114	SAT1_CF	blue	na
chrUn	23972835	23974401	1567	SAT1_CF	blue	na
chrUn	23974422	23975679	1258	SAT1_CF	blue	na
chrUn	23975702	23976919	1218	SAT1_CF	blue	na
chrUn	23976947	23977511	565	SAT1_CF	blue	na
chrUn	23977559	23978061	503	SAT1_CF	blue	na
chrUn	23978074	23978149	76	SAT1_CF	blue	na

chrUn	23978168	23978722	555	SAT1_CF	blue	na
chrUn	23978735	23979377	643	SAT1_CF	blue	na
chrUn	23979420	23979958	539	SAT1_CF	blue	na
chrUn	23979971	23981251	1281	SAT1_CF	blue	na
chrUn	23981338	23981840	503	SAT1_CF	blue	na
chrUn	23981852	23981957	106	SAT1_CF	blue	na
chrUn	23981981	23982783	803	SAT1_CF	blue	na
chrUn	23982796	23982904	109	SAT1_CF	blue	na
chrUn	23982925	23984102	1178	SAT1_CF	blue	na
chrUn	23984114	23985443	1330	SAT1_CF	blue	na
chrUn	23985486	23986818	1333	SAT1_CF	blue	na
chrUn	23986935	23987477	543	SAT1_CF	blue	na
chrUn	23987489	23988350	862	SAT1_CF	blue	na
chrUn	23988363	23989697	1335	SAT1_CF	blue	na
chrUn	23989739	23990699	961	SAT1_CF	blue	na
chrUn	23990779	23991287	509	SAT1_CF	blue	na
chrUn	23991300	23991488	189	SAT1_CF	blue	na
chrUn	23991540	23992483	944	SAT1_CF	blue	na
chrUn	23992496	23993160	665	SAT1_CF	blue	na
chrUn	23993202	23994162	961	SAT1_CF	blue	na
chrUn	23994216	23995234	1019	SAT1_CF	blue	na
chrUn	23995274	23995775	502	SAT1_CF	blue	na
chrUn	23995788	23996453	666	SAT1_CF	blue	na
chrUn	23996496	23998047	1552	SAT1_CF	blue	na
chrUn	23998061	23998414	354	SAT1_CF	blue	na
chrUn	23998520	23999086	567	SAT1_CF	blue	na
chrUn	23999108	24001004	1897	SAT1_CF	blue	na
chrUn	24001048	24001611	564	SAT1_CF	blue	na
chrUn	24001654	24001848	195	SAT1_CF	blue	na
chrUn	24001876	24002427	552	SAT1_CF	blue	na
chrUn	24002439	24003241	803	SAT1_CF	blue	na
chrUn	24003262	24004466	1205	SAT1_CF	blue	na
chrUn	24004510	24005073	564	SAT1_CF	blue	na
chrUn	24005152	24005677	526	SAT1_CF	blue	na
chrUn	24005692	24005834	143	SAT1_CF	blue	na
chrUn	24005894	24006395	502	SAT1_CF	blue	na
chrUn	24006407	24006516	110	SAT1_CF	blue	na
chrUn	24006537	24007169	633	SAT1_CF	blue	na
chrUn	24007181	24007848	668	SAT1_CF	blue	na
chrUn	24007905	24008451	547	SAT1_CF	blue	na
chrUn	24008494	24009730	1237	SAT1_CF	blue	na
chrUn	24009742	24010602	861	SAT1_CF	blue	na

chrUn	24010644	24011424	781	SAT1_CF	blue	na
chrUn	24011494	24012012	519	SAT1_CF	blue	na
chrUn	24012077	24012635	559	SAT1_CF	blue	na
chrUn	24012647	24014189	1543	SAT1_CF	blue	na
chrUn	24014201	24014601	401	SAT1_CF	blue	na
chrUn	24014621	24015139	519	SAT1_CF	blue	na
chrUn	24015151	24015260	110	SAT1_CF	blue	na
chrUn	24015281	24016542	1262	SAT1_CF	blue	na
chrUn	24016556	24017179	624	SAT1_CF	blue	na
chrUn	24017191	24017299	109	SAT1_CF	blue	na
chrUn	24017320	24017867	548	SAT1_CF	blue	na
chrUn	24017916	24019298	1383	SAT1_CF	blue	na
chrUn	24019415	24020576	1162	SAT1_CF	blue	na
chrUn	24020588	24021272	685	SAT1_CF	blue	na
chrUn	24021286	24022118	833	SAT1_CF	blue	na
chrUn	24022135	24023327	1193	SAT1_CF	blue	na
chrUn	24023339	24024006	668	SAT1_CF	blue	na
chrUn	24024049	24026039	1991	SAT1_CF	blue	na
chrUn	24026125	24026618	494	SAT1_CF	blue	na
chrUn	24026629	24029593	2965	SAT1_CF	blue	na
chrUn	24029605	24030892	1288	SAT1_CF	blue	na
chrUn	24030919	24032110	1192	SAT1_CF	blue	na
chrUn	24032159	24032560	402	SAT1_CF	blue	na
chrUn	24032620	24033093	474	SAT1_CF	blue	na
chrUn	24033114	24033882	769	SAT1_CF	blue	na
chrUn	24033894	24034570	677	SAT1_CF	blue	na
chrUn	24034613	24035575	963	SAT1_CF	blue	na
chrUn	24035631	24036651	1021	SAT1_CF	blue	na
chrUn	24036710	24037188	479	SAT1_CF	blue	na
chrUn	24037201	24037306	106	SAT1_CF	blue	na
chrUn	24037329	24038503	1175	SAT1_CF	blue	na
chrUn	24038536	24038692	157	SAT1_CF	blue	na
chrUn	24038718	24039237	520	SAT1_CF	blue	na
chrUn	24039250	24039924	675	SAT1_CF	blue	na
chrUn	24039967	24041959	1993	SAT1_CF	blue	na
chrUn	24042076	24042613	538	SAT1_CF	blue	na
chrUn	24042633	24042740	108	SAT1_CF	blue	na
chrUn	24042761	24043331	571	SAT1_CF	blue	na
chrUn	24043343	24044413	1071	SAT1_CF	blue	na
chrUn	24044425	24045649	1225	SAT1_CF	blue	na
chrUn	24045694	24046195	502	SAT1_CF	blue	na
chrUn	24046208	24047431	1224	SAT1_CF	blue	na

chrUn	24047443	24048089	647	SAT1_CF	blue	na
chrUn	24048113	24048465	353	SAT1_CF	blue	na
chrUn	24048487	24049276	790	SAT1_CF	blue	na
chrUn	24049456	24050168	713	SAT1_CF	blue	na
chrUn	24050191	24050924	734	SAT1_CF	blue	na
chrUn	24050991	24051514	524	SAT1_CF	blue	na
chrUn	24051529	24051686	158	SAT1_CF	blue	na
chrUn	24051712	24053633	1922	SAT1_CF	blue	na
chrUn	24053661	24054630	970	SAT1_CF	blue	na
chrUn	24054641	24054877	237	SAT1_CF	blue	na
chrUn	24055058	24055238	181	SAT1_CF	blue	na
chrUn	24055278	24055777	500	SAT1_CF	blue	na
chrUn	24055790	24055896	107	SAT1_CF	blue	na
chrUn	24055919	24056938	1020	SAT1_CF	blue	na
chrUn	24057061	24057565	505	SAT1_CF	blue	na
chrUn	24057577	24059288	1712	SAT1_CF	blue	na
chrUn	24059316	24059473	158	SAT1_CF	blue	na
chrUn	24059518	24060020	503	SAT1_CF	blue	na
chrUn	24060033	24060706	674	SAT1_CF	blue	na
chrUn	24060718	24061394	677	SAT1_CF	blue	na
chrUn	24061437	24063426	1990	SAT1_CF	blue	na
chrUn	24063570	24064063	494	SAT1_CF	blue	na
chrUn	24064094	24066102	2009	SAT1_CF	blue	na
chrUn	24066155	24066656	502	SAT1_CF	blue	na
chrUn	24066668	24067776	1109	SAT1_CF	blue	na
chrUn	24067816	24068316	501	SAT1_CF	blue	na
chrUn	24068329	24069010	682	SAT1_CF	blue	na
chrUn	24069023	24069782	760	SAT1_CF	blue	na
chrUn	24069794	24070429	636	SAT1_CF	blue	na
chrUn	24070443	24071513	1071	SAT1_CF	blue	na
chrUn	24071529	24071914	386	SAT1_CF	blue	na
chrUn	24071948	24072468	521	SAT1_CF	blue	na
chrUn	24072480	24073814	1335	SAT1_CF	blue	na
chrUn	24073839	24074164	326	SAT1_CF	blue	na
chrUn	24074243	24074792	550	SAT1_CF	blue	na
chrUn	24074806	24075204	399	SAT1_CF	blue	na
chrUn	24075225	24075742	518	SAT1_CF	blue	na
chrUn	24075755	24075860	106	SAT1_CF	blue	na
chrUn	24075882	24077153	1272	SAT1_CF	blue	na
chrUn	24077168	24077310	143	SAT1_CF	blue	na
chrUn	24077370	24077872	503	SAT1_CF	blue	na
chrUn	24077885	24077991	107	SAT1_CF	blue	na

chrUn	24078013	24078570	558	SAT1_CF	blue	na
chrUn	24078582	24079949	1368	SAT1_CF	blue	na
chrUn	24079992	24080960	969	SAT1_CF	blue	na
chrUn	24080973	24081328	356	SAT1_CF	blue	na
chrUn	24081444	24081964	521	SAT1_CF	blue	na
chrUn	24081995	24082189	195	SAT1_CF	blue	na
chrUn	24082217	24083068	852	SAT1_CF	blue	na
chrUn	24083082	24083603	522	SAT1_CF	blue	na
chrUn	24083637	24083969	333	SAT1_CF	blue	na
chrUn	24084056	24084557	502	SAT1_CF	blue	na
chrUn	24084570	24084682	113	SAT1_CF	blue	na
chrUn	24084848	24085512	665	SAT1_CF	blue	na
chrUn	24085529	24087215	1687	SAT1_CF	blue	na
chrUn	24087244	24087765	522	SAT1_CF	blue	na
chrUn	24087778	24088129	352	SAT1_CF	blue	na
chrUn	24088182	24088682	501	SAT1_CF	blue	na
chrUn	24088700	24089766	1067	SAT1_CF	blue	na
chrUn	24089792	24090265	474	SAT1_CF	blue	na
chrUn	24090318	24090818	501	SAT1_CF	blue	na
chrUn	24090836	24091902	1067	SAT1_CF	blue	na
chrUn	24091928	24092409	482	SAT1_CF	blue	na
chrUn	24092436	24094077	1642	SAT1_CF	blue	na
chrUn	24094107	24094962	856	SAT1_CF	blue	na
chrUn	24094994	24095930	937	SAT1_CF	blue	na
chrUn	24095951	24096473	523	SAT1_CF	blue	na
chrUn	24096486	24097168	683	SAT1_CF	blue	na
chrUn	24097180	24098467	1288	SAT1_CF	blue	na
chrUn	24098509	24099411	903	SAT1_CF	blue	na
chrUn	24099429	24099952	524	SAT1_CF	blue	na
chrUn	24099985	24100364	380	SAT1_CF	blue	na
chrUn	24100432	24100968	537	SAT1_CF	blue	na
chrUn	24100988	24101095	108	SAT1_CF	blue	na
chrUn	24101116	24101670	555	SAT1_CF	blue	na
chrUn	24101682	24102593	912	SAT1_CF	blue	na
chrUn	24102614	24103470	857	SAT1_CF	blue	na
chrUn	24103481	24103993	513	SAT1_CF	blue	na
chrUn	24104038	24105241	1204	SAT1_CF	blue	na
chrUn	24105253	24106587	1335	SAT1_CF	blue	na
chrUn	24106661	24109525	2865	SAT1_CF	blue	na
chrUn	24109622	24110164	543	SAT1_CF	blue	na
chrUn	24110208	24110770	563	SAT1_CF	blue	na
chrUn	24110827	24111301	475	SAT1_CF	blue	na

chrUn	24111328	24111918	591	SAT1_CF	blue	na
chrUn	24111936	24114359	2424	SAT1_CF	blue	na
chrUn	24114397	24115243	847	SAT1_CF	blue	na
chrUn	24115255	24115367	113	SAT1_CF	blue	na
chrUn	24115383	24117641	2259	SAT1_CF	blue	na
chrUn	24117685	24118344	660	SAT1_CF	blue	na
chrUn	24118414	24119082	669	SAT1_CF	blue	na
chrUn	24119096	24119829	734	SAT1_CF	blue	na
chrUn	24119840	24120282	443	SAT1_CF	blue	na
chrUn	24120560	24122850	2291	SAT1_CF	blue	na
chrUn	24122886	24123817	932	SAT1_CF	blue	na
chrUn	24123838	24124477	640	SAT1_CF	blue	na
chrUn	24124500	24124968	469	SAT1_CF	blue	na
chrUn	24124988	24125962	975	SAT1_CF	blue	na
chrUn	24126017	24126519	503	SAT1_CF	blue	na
chrUn	24126531	24126637	107	SAT1_CF	blue	na
chrUn	24126661	24127289	629	SAT1_CF	blue	na
chrUn	24127340	24128170	831	SAT1_CF	blue	na
chrUn	24128183	24128873	691	SAT1_CF	blue	na
chrUn	24128885	24130215	1331	SAT1_CF	blue	na
chrUn	24130258	24130820	563	SAT1_CF	blue	na
chrUn	24130901	24131425	525	SAT1_CF	blue	na
chrUn	24131440	24131680	241	SAT1_CF	blue	na
chrUn	24131698	24132190	493	SAT1_CF	blue	na
chrUn	24132247	24132794	548	SAT1_CF	blue	na
chrUn	24132836	24134292	1457	SAT1_CF	blue	na
chrUn	24134548	24134680	133	SAT1_CF	blue	na
chrUn	24134768	24135247	480	SAT1_CF	blue	na
chrUn	24135260	24135365	106	SAT1_CF	blue	na
chrUn	24135388	24137082	1695	SAT1_CF	blue	na
chrUn	24137095	24137855	761	SAT1_CF	blue	na
chrUn	24137879	24138550	672	SAT1_CF	blue	na
chrUn	24138562	24139490	929	SAT1_CF	blue	na
chrUn	24139503	24140664	1162	SAT1_CF	blue	na
chrUn	24140706	24141330	625	SAT1_CF	blue	na
chrUn	24141343	24142382	1040	SAT1_CF	blue	na
chrUn	24142397	24142554	158	SAT1_CF	blue	na
chrUn	24142599	24143681	1083	SAT1_CF	blue	na
chrUn	24143692	24144294	603	SAT1_CF	blue	na
chrUn	24144306	24145092	787	SAT1_CF	blue	na
chrUn	24145137	24145639	503	SAT1_CF	blue	na
chrUn	24145652	24147021	1370	SAT1_CF	blue	na

chrUn	24147101	24148216	1116	SAT1_CF	blue	na
chrUn	24148258	24149039	782	SAT1_CF	blue	na
chrUn	24149082	24149626	545	SAT1_CF	blue	na
chrUn	24149639	24150474	836	SAT1_CF	blue	na
chrUn	24150487	24151189	703	SAT1_CF	blue	na
chrUn	24151211	24153567	2357	SAT1_CF	blue	na
chrUn	24153581	24153737	157	SAT1_CF	blue	na
chrUn	24153782	24154283	502	SAT1_CF	blue	na
chrUn	24154310	24155929	1620	SAT1_CF	blue	na
chrUn	24155971	24156515	545	SAT1_CF	blue	na
chrUn	24156553	24157667	1115	SAT1_CF	blue	na
chrUn	24157849	24158030	182	SAT1_CF	blue	na
chrUn	24158051	24158570	520	SAT1_CF	blue	na
chrUn	24158583	24158688	106	SAT1_CF	blue	na
chrUn	24158711	24159344	634	SAT1_CF	blue	na
chrUn	24159386	24160412	1027	SAT1_CF	blue	na
chrUn	24160461	24160553	93	SAT1_CF	blue	na
chrUn	24160610	24160983	374	SAT1_CF	blue	na
chrUn	24162728	24163154	427	CarSat1	red	na
chrUn	24163186	24163910	725	CarSat1	red	na
chrUn	24163921	24164914	994	SAT1_CF	blue	na
chrUn	24164921	24166016	1096	CarSat1	red	na
chrUn	24166175	24167030	856	CarSat1	red	na
chrUn	24167052	24167904	853	CarSat1	red	na
chrUn	24167925	24169505	1581	CarSat1	red	na
chrUn	24169634	24170239	606	CarSat1	red	na
chrUn	24187685	24187847	163	Sat2CF	purple	na
chrUn	24187713	24188406	694	Sat2CF	purple	na
chrUn	24188303	24188423	121	Sat2CF	purple	na
chrUn	24212904	24214062	1159	Sat2CF	purple	na
chrUn	24216543	24216677	135	CarSat1	red	na
chrUn	24216704	24218188	1485	CarSat1	red	na
chrUn	24227674	24228081	408	CarSat1	red	na
chrUn	24228115	24229554	1440	CarSat1	red	na
chrUn	24229739	24230127	389	CarSat1	red	na
chrUn	24230146	24230548	403	CarSat1	red	na
chrUn	24230584	24231953	1370	SAT1_CF	blue	na
chrUn	24232148	24232309	162	Sat2CF	purple	na
chrUn	24232181	24233045	865	Sat2CF	purple	na
chrUn	24232949	24233056	108	Sat2CF	purple	na
chrUn	24246454	24246769	316	SAT1_CF	blue	na
chrUn	24388120	24388696	577	CarSat1	red	na

chrUn	24388726	24389766	1041	CarSat1	red	na
chrUn	24389787	24390189	403	CarSat1	red	na
chrUn	24390203	24390260	58	CarSat1	red	na
chrUn	24396291	24396964	674	CarSat1	red	na
chrUn	24396984	24398484	1501	CarSat1	red	na
chrUn	24398505	24399362	858	CarSat1	red	na
chrUn	24399383	24401257	1875	CarSat1	red	na
chrUn	24401265	24402656	1392	SAT1_CF	blue	na
chrUn	24402851	24403013	163	Sat2CF	purple	na
chrUn	24402884	24403879	996	Sat2CF	purple	na
chrUn	24415682	24417747	2066	SAT1_CF	blue	na
chrUn	24417801	24418331	531	SAT1_CF	blue	na
chrUn	24418375	24419563	1189	SAT1_CF	blue	na
chrUn	24419575	24419874	300	SAT1_CF	blue	na
chrUn	24419879	24420817	939	CarSat1	red	na
chrUn	24420829	24422593	1765	CarSat1	red	na
chrUn	24422614	24424202	1589	CarSat1	red	na
chrUn	24424223	24424747	525	CarSat1	red	na
chrUn	24425123	24425320	198	Sat2CF	purple	na
chrUn	24425145	24426312	1168	Sat2CF	purple	na
chrUn	24426204	24426381	178	Sat2CF	purple	na
chrUn	24441454	24442260	807	Sat2CF	purple	na
chrUn	24441516	24442238	723	Sat2CF	purple	na
chrUn	24498436	24498598	163	Sat2CF	purple	na
chrUn	24498464	24498821	358	Sat2CF	purple	na
chrUn	24498503	24499331	829	Sat2CF	purple	na
chrUn	24521218	24521380	163	Sat2CF	purple	na
chrUn	24521246	24521605	360	Sat2CF	purple	na
chrUn	24521285	24522069	785	Sat2CF	purple	na
chrUn	24537525	24538659	1135	Sat2CF	purple	na
chrUn	24569851	24571018	1168	Sat2CF	purple	na
chrUn	24623998	24624518	521	Sat2CF	purple	na
chrUn	24624063	24624518	456	Sat2CF	purple	na
chrUn	24624709	24624846	138	Sat2CF	purple	na
chrUn	24624723	24625273	551	Sat2CF	purple	na
chrUn	24625149	24625350	202	Sat2CF	purple	na
chrUn	24626978	24627391	414	CarSat1	red	na
chrUn	24628195	24628337	143	CarSat1	red	na
chrUn	24628358	24629072	715	CarSat1	red	na
chrUn	24629231	24631264	2034	CarSat1	red	na
chrUn	24637770	24639346	1577	SAT1_CF	blue	na
chrUn	24639360	24641721	2362	SAT1_CF	blue	na

chrUn	24641733	24642340	608	SAT1_CF	blue	na
chrUn	24642351	24642896	546	SAT1_CF	blue	na
chrUn	24642909	24643875	967	SAT1_CF	blue	na
chrUn	24643919	24644435	517	SAT1_CF	blue	na
chrUn	24650896	24653680	2785	SAT1_CF	blue	na
chrUn	24653723	24654253	531	SAT1_CF	blue	na
chrUn	24654297	24655811	1515	SAT1_CF	blue	na
chrUn	24655816	24656753	938	CarSat1	red	na
chrUn	24656785	24658533	1749	CarSat1	red	na
chrUn	24658598	24659543	946	CarSat1	red	na
chrUn	24671662	24672191	530	SAT1_CF	blue	na
chrUn	24672277	24672413	137	SAT1_CF	blue	na
chrUn	24672433	24673340	908	SAT1_CF	blue	na
chrUn	24673410	24674470	1061	SAT1_CF	blue	na
chrUn	24674568	24675220	653	SAT1_CF	blue	na
chrUn	24675270	24676228	959	SAT1_CF	blue	na
chrUn	24676258	24678742	2485	SAT1_CF	blue	na
chrUn	24678812	24680427	1616	SAT1_CF	blue	na
chrUn	24680438	24680939	502	SAT1_CF	blue	na
chrUn	24680979	24681344	366	SAT1_CF	blue	na
chrUn	24681391	24681897	507	SAT1_CF	blue	na
chrUn	24681977	24682940	964	SAT1_CF	blue	na
chrUn	24682985	24684349	1365	SAT1_CF	blue	na
chrUn	24684362	24686760	2399	SAT1_CF	blue	na
chrUn	24686803	24687478	676	SAT1_CF	blue	na
chrUn	24687490	24688121	632	SAT1_CF	blue	na
chrUn	24688142	24688251	110	SAT1_CF	blue	na
chrUn	24688263	24688763	501	SAT1_CF	blue	na
chrUn	24688850	24689205	356	SAT1_CF	blue	na
chrUn	24689219	24689830	612	SAT1_CF	blue	na
chrUn	24689874	24690547	674	SAT1_CF	blue	na
chrUn	24690589	24691155	567	SAT1_CF	blue	na
chrUn	24691175	24691284	110	SAT1_CF	blue	na
chrUn	24691296	24691773	478	SAT1_CF	blue	na
chrUn	24691830	24691902	73	SAT1_CF	blue	na
chrUn	24691914	24692416	503	SAT1_CF	blue	na
chrUn	24692469	24693377	909	SAT1_CF	blue	na
chrUn	24693458	24695752	2295	SAT1_CF	blue	na
chrUn	24695820	24697153	1334	SAT1_CF	blue	na
chrUn	24697197	24698530	1334	SAT1_CF	blue	na
chrUn	24698542	24700207	1666	SAT1_CF	blue	na
chrUn	24700221	24700904	684	SAT1_CF	blue	na

chrUn	24700916	24701417	502	SAT1_CF	blue	na
chrUn	24701463	24702465	1003	SAT1_CF	blue	na
chrUn	24702497	24702994	498	SAT1_CF	blue	na
chrUn	24703382	24705080	1699	SAT1_CF	blue	na
chrUn	24705107	24705208	102	SAT1_CF	blue	na
chrUn	24705222	24705712	491	SAT1_CF	blue	na
chrUn	24705723	24706406	684	SAT1_CF	blue	na
chrUn	24706418	24707099	682	SAT1_CF	blue	na
chrUn	24707112	24707619	508	SAT1_CF	blue	na
chrUn	24707701	24709197	1497	SAT1_CF	blue	na
chrUn	24709224	24710444	1221	SAT1_CF	blue	na
chrUn	24710458	24710950	493	SAT1_CF	blue	na
chrUn	24710961	24711643	683	SAT1_CF	blue	na
chrUn	24711655	24712209	555	SAT1_CF	blue	na
chrUn	24712279	24714355	2077	SAT1_CF	blue	na
chrUn	24714380	24714486	107	SAT1_CF	blue	na
chrUn	24714499	24715203	705	SAT1_CF	blue	na
chrUn	24715235	24715739	505	SAT1_CF	blue	na
chrUn	24715772	24716989	1218	SAT1_CF	blue	na
chrUn	24717034	24717191	158	SAT1_CF	blue	na
chrUn	24717293	24717816	524	SAT1_CF	blue	na
chrUn	24717957	24719630	1674	SAT1_CF	blue	na
chrUn	24719642	24720222	581	SAT1_CF	blue	na
chrUn	24720243	24721917	1675	SAT1_CF	blue	na
chrUn	24721960	24722634	675	SAT1_CF	blue	na
chrUn	24722646	24723332	687	SAT1_CF	blue	na
chrUn	24723343	24723843	501	SAT1_CF	blue	na
chrUn	24723901	24723979	79	SAT1_CF	blue	na
chrUn	24724033	24724557	525	SAT1_CF	blue	na
chrUn	24724636	24725198	563	SAT1_CF	blue	na
chrUn	24725241	24725879	639	SAT1_CF	blue	na
chrUn	24725892	24726566	675	SAT1_CF	blue	na
chrUn	24726578	24728175	1598	SAT1_CF	blue	na
chrUn	24728187	24728741	555	SAT1_CF	blue	na
chrUn	24728763	24729519	757	SAT1_CF	blue	na
chrUn	24729531	24730212	682	SAT1_CF	blue	na
chrUn	24730225	24730726	502	SAT1_CF	blue	na
chrUn	24730769	24732081	1313	SAT1_CF	blue	na
chrUn	24732988	24733525	538	SAT1_CF	blue	na
chrUn	24733537	24735453	1917	SAT1_CF	blue	na
chrUn	24735498	24736037	540	SAT1_CF	blue	na
chrUn	24736048	24736140	93	SAT1_CF	blue	na

chrUn	24736153	24737310	1158	SAT1_CF	blue	na
chrUn	24737376	24737510	135	SAT1_CF	blue	na
chrUn	24737564	24738765	1202	SAT1_CF	blue	na
chrUn	24738792	24739033	242	SAT1_CF	blue	na
chrUn	24739047	24739538	492	SAT1_CF	blue	na
chrUn	24739549	24740232	684	SAT1_CF	blue	na
chrUn	24740244	24740922	679	SAT1_CF	blue	na
chrUn	24740941	24741459	519	SAT1_CF	blue	na
chrUn	24741529	24741987	459	SAT1_CF	blue	na
chrUn	24742042	24742719	678	SAT1_CF	blue	na
chrUn	24742731	24744470	1740	SAT1_CF	blue	na
chrUn	24744514	24745060	547	SAT1_CF	blue	na
chrUn	24745080	24745189	110	SAT1_CF	blue	na
chrUn	24745201	24745873	673	SAT1_CF	blue	na
chrUn	24745903	24746396	494	SAT1_CF	blue	na
chrUn	24746442	24747009	568	SAT1_CF	blue	na
chrUn	24748421	24748983	563	SAT1_CF	blue	na
chrUn	24749034	24750628	1595	SAT1_CF	blue	na
chrUn	24750642	24752709	2068	SAT1_CF	blue	na
chrUn	24752725	24754155	1431	SAT1_CF	blue	na
chrUn	24754179	24754844	666	SAT1_CF	blue	na
chrUn	24754871	24755372	502	SAT1_CF	blue	na
chrUn	24755415	24756335	921	SAT1_CF	blue	na
chrUn	24756397	24757415	1019	SAT1_CF	blue	na
chrUn	24757438	24758120	683	SAT1_CF	blue	na
chrUn	24758132	24758633	502	SAT1_CF	blue	na
chrUn	24758689	24759038	350	SAT1_CF	blue	na
chrUn	24759071	24760441	1371	SAT1_CF	blue	na
chrUn	24760518	24761378	861	SAT1_CF	blue	na
chrUn	24761390	24762244	855	SAT1_CF	blue	na
chrUn	24762262	24762866	605	SAT1_CF	blue	na
chrUn	24762886	24763379	494	SAT1_CF	blue	na
chrUn	24763422	24763820	399	SAT1_CF	blue	na
chrUn	24763831	24764354	524	SAT1_CF	blue	na
chrUn	24764386	24765241	856	SAT1_CF	blue	na
chrUn	24765318	24765858	541	SAT1_CF	blue	na
chrUn	24765908	24766593	686	SAT1_CF	blue	na
chrUn	24766605	24767210	606	SAT1_CF	blue	na
chrUn	24767223	24770032	2810	SAT1_CF	blue	na
chrUn	24770051	24770492	442	SAT1_CF	blue	na
chrUn	24770548	24771116	569	SAT1_CF	blue	na
chrUn	24771181	24771338	158	SAT1_CF	blue	na

chrUn	24771353	24771877	525	SAT1_CF	blue	na
chrUn	24771958	24772483	526	SAT1_CF	blue	na
chrUn	24772564	24773936	1373	SAT1_CF	blue	na
chrUn	24773949	24774449	501	SAT1_CF	blue	na
chrUn	24774507	24774640	134	SAT1_CF	blue	na
chrUn	24774667	24775177	511	SAT1_CF	blue	na
chrUn	24775257	24776479	1223	SAT1_CF	blue	na
chrUn	24776525	24777864	1340	SAT1_CF	blue	na
chrUn	24777881	24778764	884	SAT1_CF	blue	na
chrUn	24778776	24780360	1585	SAT1_CF	blue	na
chrUn	24780374	24780535	162	SAT1_CF	blue	na
chrUn	24780551	24781087	537	SAT1_CF	blue	na
chrUn	24781129	24781737	609	SAT1_CF	blue	na
chrUn	24786702	24787290	589	SAT1_CF	blue	na
chrUn	24787311	24787712	402	SAT1_CF	blue	na
chrUn	24787763	24788305	543	SAT1_CF	blue	na
chrUn	24788384	24789809	1426	SAT1_CF	blue	na
chrUn	24789822	24790343	522	SAT1_CF	blue	na
chrUn	24790377	24791104	728	SAT1_CF	blue	na
chrUn	24791185	24792149	965	SAT1_CF	blue	na
chrUn	24792192	24793548	1357	SAT1_CF	blue	na
chrUn	24793575	24794466	892	SAT1_CF	blue	na
chrUn	24794478	24795172	695	SAT1_CF	blue	na
chrUn	24795184	24795783	600	SAT1_CF	blue	na
chrUn	24795808	24796321	514	SAT1_CF	blue	na
chrUn	24796347	24797300	954	SAT1_CF	blue	na
chrUn	24797379	24798340	962	SAT1_CF	blue	na
chrUn	24798383	24800486	2104	SAT1_CF	blue	na
chrUn	24800529	24801212	684	SAT1_CF	blue	na
chrUn	24801224	24801726	503	SAT1_CF	blue	na
chrUn	24801792	24801949	158	SAT1_CF	blue	na
chrUn	24801983	24803588	1606	SAT1_CF	blue	na
chrUn	24803633	24804178	546	SAT1_CF	blue	na
chrUn	24804234	24804306	73	SAT1_CF	blue	na
chrUn	24804318	24804838	521	SAT1_CF	blue	na
chrUn	24804858	24806672	1815	SAT1_CF	blue	na
chrUn	24806698	24807909	1212	SAT1_CF	blue	na
chrUn	24807978	24808284	307	SAT1_CF	blue	na
chrUn	24808297	24808798	502	SAT1_CF	blue	na
chrUn	24808864	24809000	137	SAT1_CF	blue	na
chrUn	24809033	24809537	505	SAT1_CF	blue	na
chrUn	24809569	24810145	577	SAT1_CF	blue	na

chrUn	24810168	24810274	107	SAT1_CF	blue	na
chrUn	24810287	24810786	500	SAT1_CF	blue	na
chrUn	24810834	24812787	1954	SAT1_CF	blue	na
chrUn	24812830	24814190	1361	SAT1_CF	blue	na
chrUn	24814212	24815641	1430	SAT1_CF	blue	na
chrUn	24815694	24816060	367	SAT1_CF	blue	na
chrUn	24816093	24818430	2338	SAT1_CF	blue	na
chrUn	24818448	24819677	1230	SAT1_CF	blue	na
chrUn	24819691	24821205	1515	SAT1_CF	blue	na
chrUn	24821292	24821809	518	SAT1_CF	blue	na
chrUn	24823231	24823792	562	SAT1_CF	blue	na
chrUn	24823807	24825438	1632	SAT1_CF	blue	na
chrUn	24825452	24825943	492	SAT1_CF	blue	na
chrUn	24825954	24827329	1376	SAT1_CF	blue	na
chrUn	24827342	24827834	493	SAT1_CF	blue	na
chrUn	24827876	24830662	2787	SAT1_CF	blue	na
chrUn	24830676	24831698	1023	SAT1_CF	blue	na
chrUn	24831719	24832678	960	SAT1_CF	blue	na
chrUn	24832757	24833716	960	SAT1_CF	blue	na
chrUn	24833760	24835131	1372	SAT1_CF	blue	na
chrUn	24835144	24835643	500	SAT1_CF	blue	na
chrUn	24835683	24836644	962	SAT1_CF	blue	na
chrUn	24836723	24837890	1168	SAT1_CF	blue	na
chrUn	24837902	24839048	1147	SAT1_CF	blue	na
chrUn	24839083	24840048	966	SAT1_CF	blue	na
chrUn	24840091	24840761	671	SAT1_CF	blue	na
chrUn	24840786	24841280	495	SAT1_CF	blue	na
chrUn	24841367	24841883	517	SAT1_CF	blue	na
chrUn	24843307	24843830	524	SAT1_CF	blue	na
chrUn	24843881	24844725	845	SAT1_CF	blue	na
chrUn	24844768	24846416	1649	SAT1_CF	blue	na
chrUn	24846443	24846962	520	SAT1_CF	blue	na
chrUn	24846988	24847145	158	SAT1_CF	blue	na
chrUn	24847160	24848101	942	SAT1_CF	blue	na
chrUn	24848112	24849316	1205	SAT1_CF	blue	na
chrUn	24849336	24849445	110	SAT1_CF	blue	na
chrUn	24849457	24850655	1199	SAT1_CF	blue	na
chrUn	24850700	24851479	780	SAT1_CF	blue	na
chrUn	24851497	24852181	685	SAT1_CF	blue	na
chrUn	24852197	24852983	787	SAT1_CF	blue	na
chrUn	24853005	24853806	802	SAT1_CF	blue	na
chrUn	24853826	24854319	494	SAT1_CF	blue	na

chrUn	24854359	24856393	2035	SAT1_CF	blue	na
chrUn	24856435	24857099	665	SAT1_CF	blue	na
chrUn	24857122	24858317	1196	SAT1_CF	blue	na
chrUn	24858357	24858924	568	SAT1_CF	blue	na
chrUn	24858925	24860062	1138	CarSat1	red	na
chrUn	24860079	24862166	2088	CarSat1	red	na
chrUn	24862191	24862901	711	CarSat1	red	na
chrUn	24862926	24863634	709	CarSat1	red	na
chrUn	24863651	24867310	3660	CarSat1	red	na
chrUn	24867525	24872117	4593	CarSat1	red	na
chrUn	24872144	24876899	4756	CarSat1	red	na
chrUn	24877373	24879212	1840	CarSat1	red	na
chrUn	24879242	24880689	1448	CarSat1	red	na
chrUn	24880719	24882161	1443	CarSat1	red	na
chrUn	24882191	24883636	1446	CarSat1	red	na
chrUn	24883651	24883777	127	Carsat2	orange	na
chrUn	24883666	24884373	708	CarSat1	red	na
chrUn	24884388	24884515	128	Carsat2	orange	na
chrUn	24884403	24888063	3661	CarSat1	red	na
chrUn	24888093	24894691	6599	CarSat1	red	na
chrUn	24894721	24897710	2990	CarSat1	red	na
chrUn	24898728	24900359	1632	SAT1_CF	blue	na
chrUn	24900371	24901487	1117	SAT1_CF	blue	na
chrUn	24901515	24902146	632	SAT1_CF	blue	na
chrUn	24902189	24902859	671	SAT1_CF	blue	na
chrUn	24902877	24903562	686	SAT1_CF	blue	na
chrUn	24903574	24904056	483	SAT1_CF	blue	na
chrUn	24904076	24904336	261	SAT1_CF	blue	na
chrUn	24904375	24904537	163	SAT1_CF	blue	na
chrUn	24904550	24907736	3187	SAT1_CF	blue	na
chrUn	24907782	24908638	857	SAT1_CF	blue	na
chrUn	24908650	24909836	1187	SAT1_CF	blue	na
chrUn	24909857	24910282	426	SAT1_CF	blue	na
chrUn	24910326	24911528	1203	SAT1_CF	blue	na
chrUn	24911550	24912188	639	SAT1_CF	blue	na
chrUn	24912256	24912755	500	SAT1_CF	blue	na
chrUn	24912774	24914890	2117	SAT1_CF	blue	na
chrUn	24914902	24915866	965	SAT1_CF	blue	na
chrUn	24915893	24916412	520	SAT1_CF	blue	na
chrUn	24916440	24916834	395	SAT1_CF	blue	na
chrUn	24916867	24917331	465	SAT1_CF	blue	na
chrUn	24917362	24918221	860	SAT1_CF	blue	na

chrUn	24918243	24918846	604	SAT1_CF	blue	na
chrUn	24918870	24918959	90	SAT1_CF	blue	na
chrUn	24918986	24919466	481	SAT1_CF	blue	na
chrUn	24919575	24920101	527	SAT1_CF	blue	na
chrUn	24921515	24922758	1244	SAT1_CF	blue	na
chrUn	24922837	24923455	619	SAT1_CF	blue	na
chrUn	24923467	24924724	1258	SAT1_CF	blue	na
chrUn	24924789	24925515	727	SAT1_CF	blue	na
chrUn	24925526	24926145	620	SAT1_CF	blue	na
chrUn	24926217	24926806	590	SAT1_CF	blue	na
chrUn	24926823	24927196	374	SAT1_CF	blue	na
chrUn	24927242	24927868	627	SAT1_CF	blue	na
chrUn	24927933	24928412	480	SAT1_CF	blue	na
chrUn	24928519	24929243	725	SAT1_CF	blue	na
chrUn	24929257	24929749	493	SAT1_CF	blue	na
chrUn	24929760	24930442	683	SAT1_CF	blue	na
chrUn	24930454	24931137	684	SAT1_CF	blue	na
chrUn	24931150	24932425	1276	SAT1_CF	blue	na
chrUn	24932465	24933609	1145	SAT1_CF	blue	na
chrUn	24933703	24934316	614	SAT1_CF	blue	na
chrUn	24934328	24935011	684	SAT1_CF	blue	na
chrUn	24935024	24935525	502	SAT1_CF	blue	na
chrUn	24935591	24935728	138	SAT1_CF	blue	na
chrUn	24935755	24936877	1123	SAT1_CF	blue	na
chrUn	24936911	24937545	635	SAT1_CF	blue	na
chrUn	24937570	24938811	1242	SAT1_CF	blue	na
chrUn	24938824	24939325	502	SAT1_CF	blue	na
chrUn	24939412	24940744	1333	SAT1_CF	blue	na
chrUn	24940788	24941433	646	SAT1_CF	blue	na
chrUn	24941445	24943180	1736	SAT1_CF	blue	na
chrUn	24943218	24945076	1859	SAT1_CF	blue	na
chrUn	24945118	24945903	786	SAT1_CF	blue	na
chrUn	24945932	24946672	741	SAT1_CF	blue	na
chrUn	24946770	24947422	653	SAT1_CF	blue	na
chrUn	24947472	24948783	1312	SAT1_CF	blue	na
chrUn	24948804	24949310	507	SAT1_CF	blue	na
chrUn	24949390	24950353	964	SAT1_CF	blue	na
chrUn	24950398	24951764	1367	SAT1_CF	blue	na
chrUn	24951777	24954175	2399	SAT1_CF	blue	na
chrUn	24954218	24954763	546	SAT1_CF	blue	na
chrUn	24954785	24955536	752	SAT1_CF	blue	na
chrUn	24955557	24955666	110	SAT1_CF	blue	na

chrUn	24955678	24956178	501	SAT1_CF	blue	na
chrUn	24956265	24956620	356	SAT1_CF	blue	na
chrUn	24956634	24958249	1616	SAT1_CF	blue	na
chrUn	24958292	24959532	1241	SAT1_CF	blue	na
chrUn	24959554	24959661	108	SAT1_CF	blue	na
chrUn	24959674	24960174	501	SAT1_CF	blue	na
chrUn	24960214	24960615	402	SAT1_CF	blue	na
chrUn	24960626	24961248	623	SAT1_CF	blue	na
chrUn	24961290	24962252	963	SAT1_CF	blue	na
chrUn	24962295	24962840	546	SAT1_CF	blue	na
chrUn	24962860	24962969	110	SAT1_CF	blue	na
chrUn	24962981	24963458	478	SAT1_CF	blue	na
chrUn	24963515	24963587	73	SAT1_CF	blue	na
chrUn	24963599	24964101	503	SAT1_CF	blue	na
chrUn	24964154	24965063	910	SAT1_CF	blue	na
chrUn	24965093	24966897	1805	SAT1_CF	blue	na
chrUn	24966909	24967856	948	SAT1_CF	blue	na
chrUn	24967899	24968568	670	SAT1_CF	blue	na
chrUn	24968582	24969262	681	SAT1_CF	blue	na
chrUn	24969275	24970163	889	SAT1_CF	blue	na
chrUn	24970175	24971839	1665	SAT1_CF	blue	na
chrUn	24971885	24972745	861	SAT1_CF	blue	na
chrUn	24972757	24973258	502	SAT1_CF	blue	na
chrUn	24973304	24974679	1376	SAT1_CF	blue	na
chrUn	24974723	24976055	1333	SAT1_CF	blue	na
chrUn	24976067	24976673	607	SAT1_CF	blue	na
chrUn	24976686	24977205	520	SAT1_CF	blue	na
chrUn	24977239	24977376	138	SAT1_CF	blue	na
chrUn	24977417	24978983	1567	SAT1_CF	blue	na
chrUn	24979100	24980897	1798	SAT1_CF	blue	na
chrUn	24980910	24981409	500	SAT1_CF	blue	na
chrUn	24981436	24981537	102	SAT1_CF	blue	na
chrUn	24981551	24982041	491	SAT1_CF	blue	na
chrUn	24982052	24982735	684	SAT1_CF	blue	na
chrUn	24982747	24983428	682	SAT1_CF	blue	na
chrUn	24983441	24983948	508	SAT1_CF	blue	na
chrUn	24983979	24984990	1012	SAT1_CF	blue	na
chrUn	24985057	24985526	470	SAT1_CF	blue	na
chrUn	24985553	24986773	1221	SAT1_CF	blue	na
chrUn	24986787	24987279	493	SAT1_CF	blue	na
chrUn	24987290	24987972	683	SAT1_CF	blue	na
chrUn	24987984	24988537	554	SAT1_CF	blue	na

chrUn	24988607	24990683	2077	SAT1_CF	blue	na
chrUn	24990708	24990814	107	SAT1_CF	blue	na
chrUn	24990827	24991531	705	SAT1_CF	blue	na
chrUn	24991563	24992067	505	SAT1_CF	blue	na
chrUn	24992099	24993318	1220	SAT1_CF	blue	na
chrUn	24993384	24993520	137	SAT1_CF	blue	na
chrUn	24993553	24994057	505	SAT1_CF	blue	na
chrUn	24994089	24994663	575	SAT1_CF	blue	na
chrUn	24994686	24994792	107	SAT1_CF	blue	na
chrUn	24994805	24995287	483	SAT1_CF	blue	na
chrUn	24995352	24996266	915	SAT1_CF	blue	na
chrUn	24996345	24997306	962	SAT1_CF	blue	na
chrUn	24997349	24998717	1369	SAT1_CF	blue	na
chrUn	24998730	25000159	1430	SAT1_CF	blue	na
chrUn	25000205	25004890	4686	SAT1_CF	blue	na
chrUn	25004903	25005403	501	SAT1_CF	blue	na
chrUn	25005445	25008235	2791	SAT1_CF	blue	na
chrUn	25008249	25009253	1005	SAT1_CF	blue	na
chrUn	25009340	25011295	1956	SAT1_CF	blue	na
chrUn	25011338	25012997	1660	SAT1_CF	blue	na
chrUn	25013010	25013529	520	SAT1_CF	blue	na
chrUn	25013553	25013951	399	SAT1_CF	blue	na
chrUn	25013962	25014509	548	SAT1_CF	blue	na
chrUn	25014553	25015550	998	SAT1_CF	blue	na
chrUn	25015593	25016831	1239	SAT1_CF	blue	na
chrUn	25016860	25017718	859	SAT1_CF	blue	na
chrUn	25017771	25018090	320	SAT1_CF	blue	na
chrUn	25018102	25018681	580	SAT1_CF	blue	na
chrUn	25018760	25019726	967	SAT1_CF	blue	na
chrUn	25019769	25020440	672	SAT1_CF	blue	na
chrUn	25020452	25021461	1010	SAT1_CF	blue	na
chrUn	25021508	25021642	135	SAT1_CF	blue	na
chrUn	25021678	25022290	613	SAT1_CF	blue	na
chrUn	25022308	25023303	996	SAT1_CF	blue	na
chrUn	25023326	25023868	543	SAT1_CF	blue	na
chrUn	25023890	25023997	108	SAT1_CF	blue	na
chrUn	25024011	25024627	617	SAT1_CF	blue	na
chrUn	25024733	25025618	886	SAT1_CF	blue	na
chrUn	25025809	25026653	845	SAT1_CF	blue	na
chrUn	25026695	25027913	1219	SAT1_CF	blue	na
chrUn	25027957	25029286	1330	SAT1_CF	blue	na
chrUn	25029298	25029780	483	SAT1_CF	blue	na

chrUn	25029798	25030421	624	SAT1_CF	blue	na
chrUn	25030464	25031429	966	SAT1_CF	blue	na
chrUn	25031472	25032586	1115	SAT1_CF	blue	na
chrUn	25033991	25034515	525	SAT1_CF	blue	na
chrUn	25034529	25036298	1770	SAT1_CF	blue	na
chrUn	25036320	25036820	501	SAT1_CF	blue	na
chrUn	25036865	25037022	158	SAT1_CF	blue	na
chrUn	25037042	25037945	904	SAT1_CF	blue	na
chrUn	25037988	25039192	1205	SAT1_CF	blue	na
chrUn	25039212	25039321	110	SAT1_CF	blue	na
chrUn	25039333	25040531	1199	SAT1_CF	blue	na
chrUn	25040571	25041136	566	SAT1_CF	blue	na
chrUn	25041154	25041838	685	SAT1_CF	blue	na
chrUn	25041854	25043463	1610	SAT1_CF	blue	na
chrUn	25043476	25043996	521	SAT1_CF	blue	na
chrUn	25044017	25046053	2037	SAT1_CF	blue	na
chrUn	25046095	25046772	678	SAT1_CF	blue	na
chrUn	25046784	25048160	1377	SAT1_CF	blue	na
chrUn	25048180	25048674	495	SAT1_CF	blue	na
chrUn	25048714	25049283	570	SAT1_CF	blue	na
chrUn	25049327	25049892	566	CarSat1	red	na
chrUn	25050100	25050227	128	Carsat2	orange	na
chrUn	25050117	25050608	492	CarSat1	red	na
chrUn	25050633	25051372	740	CarSat1	red	na
chrUn	25051624	25052174	551	CarSat1	red	na
chrUn	25052217	25052713	497	CarSat1	red	na
chrUn	25052738	25053451	714	CarSat1	red	na
chrUn	25053476	25054819	1344	CarSat1	red	na
chrUn	25054844	25055556	713	CarSat1	red	na
chrUn	25055581	25057408	1828	CarSat1	red	na
chrUn	25057452	25058915	1464	CarSat1	red	na
chrUn	25058942	25060852	1911	CarSat1	red	na
chrUn	25060867	25060993	127	Carsat2	orange	na
chrUn	25060882	25066010	5129	CarSat1	red	na
chrUn	25066026	25066152	127	Carsat2	orange	na
chrUn	25066040	25066529	490	CarSat1	red	na
chrUn	25066546	25067266	721	CarSat1	red	na
chrUn	25067291	25068634	1344	CarSat1	red	na
chrUn	25068659	25070102	1444	CarSat1	red	na
chrUn	25070119	25070837	719	CarSat1	red	na
chrUn	25070862	25071572	711	CarSat1	red	na
chrUn	25071597	25072521	925	CarSat1	red	na

chrUn	25072536	25072663	128	Carsat2	orange	na
chrUn	25072551	25073040	490	CarSat1	red	na
chrUn	25073056	25073772	717	CarSat1	red	na
chrUn	25073789	25074506	718	CarSat1	red	na
chrUn	25074531	25075241	711	CarSat1	red	na
chrUn	25075266	25076305	1040	CarSat1	red	na
chrUn	25076653	25077452	800	CarSat1	red	na
chrUn	25077477	25078259	783	CarSat1	red	na
chrUn	25078560	25078744	185	Carsat2	orange	na
chrUn	25078596	25079305	710	CarSat1	red	na
chrUn	25079332	25081337	2006	CarSat1	red	na
chrUn	25082388	25084558	2171	CarSat1	red	na
chrUn	25084572	25091075	6504	CarSat1	red	na
chrUn	25091108	25091810	703	CarSat1	red	na
chrUn	25091834	25093775	1942	CarSat1	red	na
chrUn	25093797	25094757	961	CarSat1	red	na
chrUn	25094781	25096231	1451	CarSat1	red	na
chrUn	25096255	25099670	3416	CarSat1	red	na
chrUn	25099691	25100881	1191	CarSat1	red	na
chrUn	25100896	25101023	128	Carsat2	orange	na
chrUn	25100911	25103363	2453	CarSat1	red	na
chrUn	25103390	25104100	711	CarSat1	red	na
chrUn	25104127	25104839	713	CarSat1	red	na
chrUn	25104864	25108472	3609	CarSat1	red	na
chrUn	25108797	25109265	469	CarSat1	red	na
chrUn	25109292	25110003	712	CarSat1	red	na
chrUn	25110030	25110738	709	CarSat1	red	na
chrUn	25110765	25111727	963	CarSat1	red	na
chrUn	25111743	25112211	469	CarSat1	red	na
chrUn	25112238	25112948	711	CarSat1	red	na
chrUn	25112975	25113684	710	CarSat1	red	na
chrUn	25113711	25114417	707	CarSat1	red	na
chrUn	25114444	25115155	712	CarSat1	red	na
chrUn	25115182	25116143	962	CarSat1	red	na
chrUn	25116159	25116879	721	CarSat1	red	na
chrUn	25116895	25117830	936	CarSat1	red	na
chrUn	25117860	25118568	709	CarSat1	red	na
chrUn	25118583	25118710	128	Carsat2	orange	na
chrUn	25118599	25120315	1717	CarSat1	red	na
chrUn	25120342	25122445	2104	CarSat1	red	na
chrUn	25123477	25124036	560	SAT1_CF	blue	na
chrUn	25124048	25127123	3076	SAT1_CF	blue	na

chrUn	25127210	25127711	502	SAT1_CF	blue	na
chrUn	25127725	25129280	1556	SAT1_CF	blue	na
chrUn	25129298	25130656	1359	SAT1_CF	blue	na
chrUn	25130701	25131664	964	SAT1_CF	blue	na
chrUn	25131744	25132670	927	SAT1_CF	blue	na
chrUn	25132706	25133206	501	SAT1_CF	blue	na
chrUn	25133224	25134271	1048	SAT1_CF	blue	na
chrUn	25134292	25134834	543	SAT1_CF	blue	na
chrUn	25134904	25136296	1393	SAT1_CF	blue	na
chrUn	25136956	25137043	88	SAT1_CF	blue	na
chrUn	25137058	25138380	1323	SAT1_CF	blue	na
chrUn	25138430	25139129	700	SAT1_CF	blue	na
chrUn	25139180	25139921	742	SAT1_CF	blue	na
chrUn	25139950	25141207	1258	SAT1_CF	blue	na
chrUn	25141232	25142109	878	SAT1_CF	blue	na
chrUn	25142120	25142474	355	SAT1_CF	blue	na
chrUn	25142561	25143062	502	SAT1_CF	blue	na
chrUn	25143075	25144298	1224	SAT1_CF	blue	na
chrUn	25144310	25146039	1730	SAT1_CF	blue	na
chrUn	25146051	25146356	306	SAT1_CF	blue	na
chrUn	25146678	25146948	271	SAT1_CF	blue	na
chrUn	25146975	25147939	965	SAT1_CF	blue	na
chrUn	25147951	25148623	673	SAT1_CF	blue	na
chrUn	25148637	25148772	136	SAT1_CF	blue	na
chrUn	25148814	25149354	541	SAT1_CF	blue	na
chrUn	25149433	25149924	492	SAT1_CF	blue	na
chrUn	25152918	25153347	430	SAT1_CF	blue	na
chrUn	25153364	25153875	512	SAT1_CF	blue	na
chrUn	25153955	25154536	582	SAT1_CF	blue	na
chrUn	25154548	25155404	857	SAT1_CF	blue	na
chrUn	25155484	25156673	1190	SAT1_CF	blue	na
chrUn	25156716	25157216	501	SAT1_CF	blue	na
chrUn	25157229	25158067	839	SAT1_CF	blue	na
chrUn	25158079	25158750	672	SAT1_CF	blue	na
chrUn	25158796	25159445	650	SAT1_CF	blue	na
chrUn	25159465	25159948	484	SAT1_CF	blue	na
chrUn	25159960	25160644	685	SAT1_CF	blue	na
chrUn	25160662	25161332	671	SAT1_CF	blue	na
chrUn	25161375	25162487	1113	SAT1_CF	blue	na
chrUn	25162530	25163113	584	SAT1_CF	blue	na
chrUn	25163125	25164639	1515	SAT1_CF	blue	na
chrUn	25164681	25165452	772	SAT1_CF	blue	na

chrUn	25165593	25166530	938	SAT1_CF	blue	na
chrUn	25166568	25169334	2767	SAT1_CF	blue	na
chrUn	25169404	25170651	1248	SAT1_CF	blue	na
chrUn	25170662	25171153	492	SAT1_CF	blue	na
chrUn	25171167	25172380	1214	SAT1_CF	blue	na
chrUn	25172391	25172881	491	SAT1_CF	blue	na
chrUn	25172920	25173741	822	SAT1_CF	blue	na
chrUn	25173756	25174456	701	SAT1_CF	blue	na
chrUn	25174469	25175069	601	SAT1_CF	blue	na
chrUn	25175081	25175755	675	SAT1_CF	blue	na
chrUn	25175798	25177130	1333	SAT1_CF	blue	na
chrUn	25177147	25177317	171	SAT1_CF	blue	na
chrUn	25178700	25179231	532	SAT1_CF	blue	na
chrUn	25179299	25179820	522	SAT1_CF	blue	na
chrUn	25179833	25180811	979	SAT1_CF	blue	na
chrUn	25180823	25183498	2676	SAT1_CF	blue	na
chrUn	25183525	25184163	639	SAT1_CF	blue	na
chrUn	25184185	25184730	546	SAT1_CF	blue	na
chrUn	25184772	25185735	964	SAT1_CF	blue	na
chrUn	25185790	25187062	1273	SAT1_CF	blue	na
chrUn	25187074	25187930	857	SAT1_CF	blue	na
chrUn	25187975	25190336	2362	SAT1_CF	blue	na
chrUn	25190348	25191239	892	SAT1_CF	blue	na
chrUn	25191266	25191713	448	SAT1_CF	blue	na
chrUn	25191733	25192216	484	SAT1_CF	blue	na
chrUn	25192228	25192912	685	SAT1_CF	blue	na
chrUn	25192930	25193600	671	SAT1_CF	blue	na
chrUn	25193643	25194599	957	SAT1_CF	blue	na
chrUn	25194666	25194727	62	SAT1_CF	blue	na
chrUn	25194740	25195675	936	SAT1_CF	blue	na
chrUn	25195715	25196934	1220	SAT1_CF	blue	na
chrUn	25196966	25197487	522	SAT1_CF	blue	na
chrUn	25197502	25197659	158	SAT1_CF	blue	na
chrUn	25197685	25200424	2740	SAT1_CF	blue	na
chrUn	25200494	25200806	313	SAT1_CF	blue	na
chrUn	25205074	25205823	750	SAT1_CF	blue	na
chrUn	25205835	25207141	1307	SAT1_CF	blue	na
chrUn	25207172	25207501	330	SAT1_CF	blue	na
chrUn	25207577	25208774	1198	SAT1_CF	blue	na
chrUn	25208786	25209454	669	SAT1_CF	blue	na
chrUn	25209497	25211462	1966	SAT1_CF	blue	na
chrUn	25211522	25212003	482	SAT1_CF	blue	na

chrUn	25212015	25212670	656	SAT1_CF	blue	na
chrUn	25212727	25213273	547	SAT1_CF	blue	na
chrUn	25213286	25213931	646	SAT1_CF	blue	na
chrUn	25213975	25215113	1139	SAT1_CF	blue	na
chrUn	25215160	25215681	522	SAT1_CF	blue	na
chrUn	25215708	25216300	593	SAT1_CF	blue	na
chrUn	25216312	25218032	1721	SAT1_CF	blue	na
chrUn	25218064	25219066	1003	SAT1_CF	blue	na
chrUn	25219095	25219613	519	SAT1_CF	blue	na
chrUn	25219625	25220486	862	SAT1_CF	blue	na
chrUn	25220528	25224905	4378	SAT1_CF	blue	na
chrUn	25224945	25225447	503	SAT1_CF	blue	na
chrUn	25225471	25226429	959	SAT1_CF	blue	na
chrUn	25226474	25227503	1030	SAT1_CF	blue	na
chrUn	25227524	25228133	610	SAT1_CF	blue	na
chrUn	25228186	25228704	519	SAT1_CF	blue	na
chrUn	25228747	25229708	962	SAT1_CF	blue	na
chrUn	25229787	25230331	545	SAT1_CF	blue	na
chrUn	25230345	25230747	403	SAT1_CF	blue	na
chrUn	25230807	25231288	482	SAT1_CF	blue	na
chrUn	25231315	25232097	783	SAT1_CF	blue	na
chrUn	25232120	25232665	546	SAT1_CF	blue	na
chrUn	25232707	25233690	984	SAT1_CF	blue	na
chrUn	25233716	25234572	857	SAT1_CF	blue	na
chrUn	25234584	25235474	891	SAT1_CF	blue	na
chrUn	25235487	25236851	1365	SAT1_CF	blue	na
chrUn	25236898	25238523	1626	SAT1_CF	blue	na
chrUn	25238602	25239141	540	SAT1_CF	blue	na
chrUn	25239217	25239717	501	SAT1_CF	blue	na
chrUn	25239738	25240414	677	SAT1_CF	blue	na
chrUn	25240426	25241108	683	SAT1_CF	blue	na
chrUn	25241119	25241605	487	SAT1_CF	blue	na
chrUn	25241625	25241936	312	SAT1_CF	blue	na
chrUn	25241989	25242484	496	SAT1_CF	blue	na
chrUn	25242499	25242579	81	SAT1_CF	blue	na
chrUn	25242633	25243151	519	SAT1_CF	blue	na
chrUn	25243194	25245197	2004	SAT1_CF	blue	na
chrUn	25245237	25245739	503	SAT1_CF	blue	na
chrUn	25245752	25246546	795	SAT1_CF	blue	na
chrUn	25246558	25247239	682	SAT1_CF	blue	na
chrUn	25247255	25248471	1217	SAT1_CF	blue	na
chrUn	25248483	25248555	73	SAT1_CF	blue	na

chrUn	25248577	25249130	554	SAT1_CF	blue	na
chrUn	25249142	25250739	1598	SAT1_CF	blue	na
chrUn	25250751	25251527	777	SAT1_CF	blue	na
chrUn	25251548	25252484	937	SAT1_CF	blue	na
chrUn	25252499	25252656	158	SAT1_CF	blue	na
chrUn	25252700	25253204	505	SAT1_CF	blue	na
chrUn	25253226	25253901	676	SAT1_CF	blue	na
chrUn	25253919	25254587	669	SAT1_CF	blue	na
chrUn	25254636	25255238	603	SAT1_CF	blue	na
chrUn	25255281	25255843	563	SAT1_CF	blue	na
chrUn	25255922	25256446	525	SAT1_CF	blue	na
chrUn	25256500	25256578	79	SAT1_CF	blue	na
chrUn	25256636	25257649	1014	SAT1_CF	blue	na
chrUn	25257661	25259335	1675	SAT1_CF	blue	na
chrUn	25259476	25259999	524	SAT1_CF	blue	na
chrUn	25260101	25260258	158	SAT1_CF	blue	na
chrUn	25260286	25261520	1235	SAT1_CF	blue	na
chrUn	25261552	25264654	3103	SAT1_CF	blue	na
chrUn	25264724	25265749	1026	SAT1_CF	blue	na
chrUn	25265761	25267095	1335	SAT1_CF	blue	na
chrUn	25267139	25268516	1378	SAT1_CF	blue	na
chrUn	25268545	25269063	519	SAT1_CF	blue	na
chrUn	25269075	25269936	862	SAT1_CF	blue	na
chrUn	25269978	25272273	2296	SAT1_CF	blue	na
chrUn	25272285	25273174	890	SAT1_CF	blue	na
chrUn	25273187	25274521	1335	SAT1_CF	blue	na
chrUn	25274563	25275101	539	SAT1_CF	blue	na
chrUn	25275117	25276468	1352	SAT1_CF	blue	na
chrUn	25276523	25277023	501	SAT1_CF	blue	na
chrUn	25277035	25277110	76	SAT1_CF	blue	na
chrUn	25277166	25278584	1419	SAT1_CF	blue	na
chrUn	25278626	25279168	543	SAT1_CF	blue	na
chrUn	25279206	25280032	827	SAT1_CF	blue	na
chrUn	25280061	25280430	370	SAT1_CF	blue	na
chrUn	25280470	25280970	501	SAT1_CF	blue	na
chrUn	25280983	25281664	682	SAT1_CF	blue	na
chrUn	25281676	25281785	110	SAT1_CF	blue	na
chrUn	25281805	25282898	1094	SAT1_CF	blue	na
chrUn	25282966	25283888	923	SAT1_CF	blue	na
chrUn	25283922	25285634	1713	SAT1_CF	blue	na
chrUn	25285647	25286537	891	SAT1_CF	blue	na
chrUn	25286552	25288071	1520	SAT1_CF	blue	na

chrUn	25288099	25288608	510	SAT1_CF	blue	na
chrUn	25288645	25291805	3161	SAT1_CF	blue	na
chrUn	25291848	25292961	1114	SAT1_CF	blue	na
chrUn	25293004	25293588	585	SAT1_CF	blue	na
chrUn	25293600	25296058	2459	SAT1_CF	blue	na
chrUn	25296069	25297080	1012	SAT1_CF	blue	na
chrUn	25297131	25297268	138	SAT1_CF	blue	na
chrUn	25297315	25297906	592	SAT1_CF	blue	na
chrUn	25297976	25299044	1069	SAT1_CF	blue	na
chrUn	25299065	25299570	506	SAT1_CF	blue	na
chrUn	25299604	25300985	1382	SAT1_CF	blue	na
chrUn	25300997	25301888	892	SAT1_CF	blue	na
chrUn	25301911	25302582	672	SAT1_CF	blue	na
chrUn	25302594	25303451	858	SAT1_CF	blue	na
chrUn	25303528	25305349	1822	SAT1_CF	blue	na
chrUn	25305389	25305885	497	SAT1_CF	blue	na
chrUn	25305898	25306581	684	SAT1_CF	blue	na
chrUn	25306592	25308975	2384	SAT1_CF	blue	na
chrUn	25308991	25312077	3087	SAT1_CF	blue	na
chrUn	25312159	25312666	508	SAT1_CF	blue	na
chrUn	25312693	25313362	670	SAT1_CF	blue	na
chrUn	25313374	25314056	683	SAT1_CF	blue	na
chrUn	25314067	25314485	419	SAT1_CF	blue	na
chrUn	25314499	25316707	2209	SAT1_CF	blue	na
chrUn	25317799	25318430	632	SAT1_CF	blue	na
chrUn	25318442	25319117	676	SAT1_CF	blue	na
chrUn	25319160	25320380	1221	SAT1_CF	blue	na
chrUn	25321747	25321925	179	SAT1_CF	blue	na
chrUn	25321942	25322494	553	SAT1_CF	blue	na
chrUn	25322506	25324310	1805	SAT1_CF	blue	na
chrUn	25324328	25324852	525	SAT1_CF	blue	na
chrUn	25325014	25325346	333	SAT1_CF	blue	na
chrUn	25325423	25325964	542	SAT1_CF	blue	na
chrUn	25326054	25326724	671	SAT1_CF	blue	na
chrUn	25326842	25327398	557	SAT1_CF	blue	na
chrUn	25329210	25329679	470	SAT1_CF	blue	na
chrUn	25329692	25331530	1839	SAT1_CF	blue	na
chrUn	25331564	25332085	522	SAT1_CF	blue	na
chrUn	25332098	25332704	607	SAT1_CF	blue	na
chrUn	25332716	25332825	110	SAT1_CF	blue	na
chrUn	25332845	25333390	546	SAT1_CF	blue	na
chrUn	25333403	25335203	1801	SAT1_CF	blue	na

chrUn	25335237	25337193	1957	SAT1_CF	blue	na
chrUn	25337272	25337814	543	SAT1_CF	blue	na
chrUn	25337865	25338264	400	SAT1_CF	blue	na
chrUn	25338285	25338803	519	SAT1_CF	blue	na
chrUn	25338816	25339498	683	SAT1_CF	blue	na
chrUn	25339522	25340187	666	SAT1_CF	blue	na
chrUn	25340231	25341215	985	SAT1_CF	blue	na
chrUn	25341293	25341849	557	SAT1_CF	blue	na
chrUn	25341883	25342117	235	SAT1_CF	blue	na
chrUn	25342136	25342645	510	SAT1_CF	blue	na
chrUn	25342658	25343341	684	SAT1_CF	blue	na
chrUn	25343353	25344026	674	SAT1_CF	blue	na
chrUn	25344047	25344687	641	SAT1_CF	blue	na
chrUn	25344731	25345293	563	SAT1_CF	blue	na
chrUn	25345373	25345883	511	SAT1_CF	blue	na
chrUn	25345912	25346042	131	SAT1_CF	blue	na
chrUn	25346100	25346600	501	SAT1_CF	blue	na
chrUn	25346613	25347296	684	SAT1_CF	blue	na
chrUn	25347308	25347984	677	SAT1_CF	blue	na
chrUn	25348027	25348656	630	SAT1_CF	blue	na
chrUn	25348759	25349280	522	SAT1_CF	blue	na
chrUn	25349366	25349889	524	SAT1_CF	blue	na
chrUn	25349904	25350061	158	SAT1_CF	blue	na
chrUn	25350106	25350576	471	SAT1_CF	blue	na
chrUn	25351589	25351862	274	SAT1_CF	blue	na
chrUn	25351873	25353020	1148	SAT1_CF	blue	na
chrUn	25353031	25353836	806	SAT1_CF	blue	na
chrUn	25353859	25354404	546	SAT1_CF	blue	na
chrUn	25354484	25354846	363	SAT1_CF	blue	na
chrUn	25354915	25355393	479	SAT1_CF	blue	na
chrUn	25355405	25356089	685	SAT1_CF	blue	na
chrUn	25356140	25356671	532	SAT1_CF	blue	na
chrUn	25356721	25357386	666	SAT1_CF	blue	na
chrUn	25357467	25358009	543	SAT1_CF	blue	na
chrUn	25358022	25358887	866	SAT1_CF	blue	na
chrUn	25359809	25360852	1044	SAT1_CF	blue	na
chrUn	25360865	25361646	782	SAT1_CF	blue	na
chrUn	25361658	25362334	677	SAT1_CF	blue	na
chrUn	25362431	25363156	726	SAT1_CF	blue	na
chrUn	25363226	25364644	1419	SAT1_CF	blue	na
chrUn	25364688	25365929	1242	SAT1_CF	blue	na
chrUn	25365971	25367167	1197	SAT1_CF	blue	na

chrUn	25367224	25367705	482	SAT1_CF	blue	na
chrUn	25367723	25368789	1067	SAT1_CF	blue	na
chrUn	25368815	25369294	480	SAT1_CF	blue	na
chrUn	25369329	25371475	2147	SAT1_CF	blue	na
chrUn	25371515	25372008	494	SAT1_CF	blue	na
chrUn	25372019	25373442	1424	SAT1_CF	blue	na
chrUn	25373464	25375355	1892	SAT1_CF	blue	na
chrUn	25375398	25376028	631	SAT1_CF	blue	na
chrUn	25376056	25376717	662	SAT1_CF	blue	na
chrUn	25376738	25377282	545	SAT1_CF	blue	na
chrUn	25377312	25377468	157	SAT1_CF	blue	na
chrUn	25377494	25378014	521	SAT1_CF	blue	na
chrUn	25378027	25380395	2369	SAT1_CF	blue	na
chrUn	25380409	25380953	545	SAT1_CF	blue	na
chrUn	25381060	25381152	93	SAT1_CF	blue	na
chrUn	25381182	25381562	381	SAT1_CF	blue	na
chrUn	25381583	25382102	520	SAT1_CF	blue	na
chrUn	25382114	25382223	110	SAT1_CF	blue	na
chrUn	25382244	25382875	632	SAT1_CF	blue	na
chrUn	25382887	25383554	668	SAT1_CF	blue	na
chrUn	25383611	25384156	546	SAT1_CF	blue	na
chrUn	25384199	25384740	542	SAT1_CF	blue	na
chrUn	25384762	25384858	97	SAT1_CF	blue	na
chrUn	25384879	25385433	555	SAT1_CF	blue	na
chrUn	25385445	25386307	863	SAT1_CF	blue	na
chrUn	25386349	25387131	783	SAT1_CF	blue	na
chrUn	25387200	25387717	518	SAT1_CF	blue	na
chrUn	25387782	25388340	559	SAT1_CF	blue	na
chrUn	25388362	25389346	985	SAT1_CF	blue	na
chrUn	25389389	25390460	1072	SAT1_CF	blue	na
chrUn	25390494	25391424	931	SAT1_CF	blue	na
chrUn	25391464	25391966	503	SAT1_CF	blue	na
chrUn	25391992	25394236	2245	SAT1_CF	blue	na
chrUn	25394254	25395016	763	SAT1_CF	blue	na
chrUn	25395028	25395702	675	SAT1_CF	blue	na
chrUn	25395767	25396253	487	SAT1_CF	blue	na
chrUn	25396306	25396808	503	SAT1_CF	blue	na
chrUn	25396825	25396928	104	SAT1_CF	blue	na
chrUn	25396950	25398846	1897	SAT1_CF	blue	na
chrUn	25398870	25399719	850	SAT1_CF	blue	na
chrUn	25399730	25399805	76	SAT1_CF	blue	na
chrUn	25399862	25400789	928	SAT1_CF	blue	na

chrUn	25400817	25401341	525	SAT1_CF	blue	na
chrUn	25401356	25401485	130	SAT1_CF	blue	na
chrUn	25401557	25402059	503	SAT1_CF	blue	na
chrUn	25402204	25403586	1383	SAT1_CF	blue	na
chrUn	25403657	25404409	753	SAT1_CF	blue	na
chrUn	25404488	25405036	549	SAT1_CF	blue	na
chrUn	25405048	25405448	401	SAT1_CF	blue	na
chrUn	25405467	25405986	520	SAT1_CF	blue	na
chrUn	25405999	25406688	690	SAT1_CF	blue	na
chrUn	25406700	25407525	826	SAT1_CF	blue	na
chrUn	25407538	25408397	860	SAT1_CF	blue	na
chrUn	25408439	25409218	780	SAT1_CF	blue	na
chrUn	25409272	25410292	1021	SAT1_CF	blue	na
chrUn	25410351	25412023	1673	SAT1_CF	blue	na
chrUn	25412067	25412684	618	SAT1_CF	blue	na
chrUn	25412697	25412851	155	SAT1_CF	blue	na
chrUn	25412898	25415214	2317	SAT1_CF	blue	na
chrUn	25415227	25415729	503	SAT1_CF	blue	na
chrUn	25415756	25416423	668	SAT1_CF	blue	na
chrUn	25416447	25417296	850	SAT1_CF	blue	na
chrUn	25417367	25418227	861	SAT1_CF	blue	na
chrUn	25418261	25418782	522	SAT1_CF	blue	na
chrUn	25418793	25418980	188	SAT1_CF	blue	na
chrUn	25419032	25419515	484	SAT1_CF	blue	na
chrUn	25419527	25420828	1302	SAT1_CF	blue	na
chrUn	25420856	25421013	158	SAT1_CF	blue	na
chrUn	25421077	25421555	479	SAT1_CF	blue	na
chrUn	25421568	25422250	683	SAT1_CF	blue	na
chrUn	25422262	25422939	678	SAT1_CF	blue	na
chrUn	25422982	25423943	962	SAT1_CF	blue	na
chrUn	25424010	25425542	1533	SAT1_CF	blue	na
chrUn	25425620	25426168	549	SAT1_CF	blue	na
chrUn	25426179	25426580	402	SAT1_CF	blue	na
chrUn	25426602	25427241	640	SAT1_CF	blue	na
chrUn	25427264	25427896	633	SAT1_CF	blue	na
chrUn	25427908	25428575	668	SAT1_CF	blue	na
chrUn	25428632	25429177	546	SAT1_CF	blue	na
chrUn	25429220	25429763	544	SAT1_CF	blue	na
chrUn	25429776	25429928	153	SAT1_CF	blue	na
chrUn	25429954	25432427	2474	SAT1_CF	blue	na
chrUn	25432439	25433224	786	SAT1_CF	blue	na
chrUn	25433247	25433795	549	SAT1_CF	blue	na

chrUn	25433808	25434160	353	SAT1_CF	blue	na
chrUn	25434276	25434793	518	SAT1_CF	blue	na
chrUn	25434852	25435412	561	SAT1_CF	blue	na
chrUn	25435424	25436780	1357	SAT1_CF	blue	na
chrUn	25436793	25437145	353	SAT1_CF	blue	na
chrUn	25437232	25437780	549	SAT1_CF	blue	na
chrUn	25437837	25438561	725	SAT1_CF	blue	na
chrUn	25438646	25439420	775	SAT1_CF	blue	na
chrUn	25439445	25440440	996	SAT1_CF	blue	na
chrUn	25440488	25441446	959	SAT1_CF	blue	na
chrUn	25441486	25441987	502	SAT1_CF	blue	na
chrUn	25442000	25442673	674	SAT1_CF	blue	na
chrUn	25442716	25443249	534	SAT1_CF	blue	na
chrUn	25443263	25445685	2423	SAT1_CF	blue	na
chrUn	25445729	25445935	207	SAT1_CF	blue	na
chrUn	25446117	25446302	186	SAT1_CF	blue	na
chrUn	25446319	25446837	519	SAT1_CF	blue	na
chrUn	25446850	25446955	106	SAT1_CF	blue	na
chrUn	25446977	25448249	1273	SAT1_CF	blue	na
chrUn	25448264	25448968	705	SAT1_CF	blue	na
chrUn	25448981	25449087	107	SAT1_CF	blue	na
chrUn	25449109	25449666	558	SAT1_CF	blue	na
chrUn	25449678	25450349	672	SAT1_CF	blue	na
chrUn	25450392	25451060	669	SAT1_CF	blue	na
chrUn	25451087	25452455	1369	SAT1_CF	blue	na
chrUn	25452468	25452580	113	SAT1_CF	blue	na
chrUn	25452745	25453410	666	SAT1_CF	blue	na
chrUn	25453427	25454651	1225	SAT1_CF	blue	na
chrUn	25454709	25456122	1414	SAT1_CF	blue	na
chrUn	25456160	25457621	1462	SAT1_CF	blue	na
chrUn	25457634	25458318	685	SAT1_CF	blue	na
chrUn	25458331	25459491	1161	SAT1_CF	blue	na
chrUn	25459511	25460121	611	SAT1_CF	blue	na
chrUn	25460200	25460365	166	SAT1_CF	blue	na
chrUn	25460546	25460731	186	SAT1_CF	blue	na
chrUn	25460748	25461268	521	SAT1_CF	blue	na
chrUn	25461284	25461389	106	SAT1_CF	blue	na
chrUn	25461409	25461966	558	SAT1_CF	blue	na
chrUn	25461978	25463579	1602	SAT1_CF	blue	na
chrUn	25463593	25464688	1096	SAT1_CF	blue	na
chrUn	25464775	25465273	499	SAT1_CF	blue	na
chrUn	25465286	25465390	105	SAT1_CF	blue	na

chrUn	25465415	25467429	2015	SAT1_CF	blue	na
chrUn	25467472	25469056	1585	SAT1_CF	blue	na
chrUn	25469069	25469465	397	SAT1_CF	blue	na
chrUn	25469513	25470011	499	SAT1_CF	blue	na
chrUn	25470024	25470706	683	SAT1_CF	blue	na
chrUn	25470718	25471393	676	SAT1_CF	blue	na
chrUn	25471437	25472398	962	SAT1_CF	blue	na
chrUn	25472448	25472740	293	SAT1_CF	blue	na
chrUn	25472771	25473281	511	SAT1_CF	blue	na
chrUn	25473309	25473445	137	SAT1_CF	blue	na
chrUn	25473511	25474011	501	SAT1_CF	blue	na
chrUn	25474024	25474324	301	SAT1_CF	blue	na
chrUn	25474336	25475966	1631	SAT1_CF	blue	na
chrUn	25476010	25476912	903	SAT1_CF	blue	na
chrUn	25476930	25477453	524	SAT1_CF	blue	na
chrUn	25477486	25477865	380	SAT1_CF	blue	na
chrUn	25477933	25478469	537	SAT1_CF	blue	na
chrUn	25478489	25478592	104	SAT1_CF	blue	na
chrUn	25478616	25479171	556	SAT1_CF	blue	na
chrUn	25479183	25480094	912	SAT1_CF	blue	na
chrUn	25480115	25481494	1380	SAT1_CF	blue	na
chrUn	25481539	25483664	2126	SAT1_CF	blue	na
chrUn	25483692	25484617	926	SAT1_CF	blue	na
chrUn	25484655	25485478	824	SAT1_CF	blue	na
chrUn	25485489	25486014	526	SAT1_CF	blue	na
chrUn	25486095	25488759	2665	SAT1_CF	blue	na
chrUn	25491226	25491978	753	SAT1_CF	blue	na
chrUn	25492033	25492923	891	SAT1_CF	blue	na
chrUn	25492956	25493473	518	SAT1_CF	blue	na
chrUn	25493541	25494061	521	SAT1_CF	blue	na
chrUn	25494073	25494186	114	SAT1_CF	blue	na
chrUn	25494203	25494912	710	SAT1_CF	blue	na
chrUn	25494924	25495786	863	SAT1_CF	blue	na
chrUn	25495828	25497273	1446	SAT1_CF	blue	na
chrUn	25497284	25497686	403	SAT1_CF	blue	na
chrUn	25497726	25498227	502	SAT1_CF	blue	na
chrUn	25498240	25499464	1225	SAT1_CF	blue	na
chrUn	25499525	25499686	162	SAT1_CF	blue	na
chrUn	25499747	25500272	526	SAT1_CF	blue	na
chrUn	25500287	25500423	137	SAT1_CF	blue	na
chrUn	25500489	25500990	502	SAT1_CF	blue	na
chrUn	25501038	25501901	864	SAT1_CF	blue	na

chrUn	25501943	25503753	1811	SAT1_CF	blue	na
chrUn	25503821	25504341	521	SAT1_CF	blue	na
chrUn	25504353	25504466	114	SAT1_CF	blue	na
chrUn	25504483	25505192	710	SAT1_CF	blue	na
chrUn	25505204	25505881	678	SAT1_CF	blue	na
chrUn	25505924	25506465	542	SAT1_CF	blue	na
chrUn	25506479	25507553	1075	SAT1_CF	blue	na
chrUn	25507564	25507966	403	SAT1_CF	blue	na
chrUn	25508006	25508507	502	SAT1_CF	blue	na
chrUn	25508520	25509744	1225	SAT1_CF	blue	na
chrUn	25509805	25510552	748	SAT1_CF	blue	na
chrUn	25510567	25510703	137	SAT1_CF	blue	na
chrUn	25510789	25511270	482	SAT1_CF	blue	na
chrUn	25511297	25512044	748	SAT1_CF	blue	na
chrUn	25512057	25513388	1332	SAT1_CF	blue	na
chrUn	25513431	25515477	2047	SAT1_CF	blue	na
chrUn	25515489	25516008	520	SAT1_CF	blue	na
chrUn	25516021	25516702	682	SAT1_CF	blue	na
chrUn	25516713	25517336	624	SAT1_CF	blue	na
chrUn	25517379	25518900	1522	SAT1_CF	blue	na
chrUn	25519082	25519307	226	SAT1_CF	blue	na
chrUn	25519329	25519995	667	SAT1_CF	blue	na
chrUn	25520038	25521039	1002	SAT1_CF	blue	na
chrUn	25521071	25521592	522	SAT1_CF	blue	na
chrUn	25521605	25521743	139	SAT1_CF	blue	na
chrUn	25521917	25522033	117	SAT1_CF	blue	na
chrUn	25522078	25522580	503	SAT1_CF	blue	na
chrUn	25522607	25523267	661	SAT1_CF	blue	na
chrUn	25523310	25524898	1589	SAT1_CF	blue	na
chrUn	25524914	25525313	400	SAT1_CF	blue	na
chrUn	25525373	25525854	482	SAT1_CF	blue	na
chrUn	25525866	25527199	1334	SAT1_CF	blue	na
chrUn	25527224	25527548	325	SAT1_CF	blue	na
chrUn	25527627	25528174	548	SAT1_CF	blue	na
chrUn	25528218	25529496	1279	SAT1_CF	blue	na
chrUn	25529537	25530355	819	SAT1_CF	blue	na
chrUn	25530368	25531205	838	SAT1_CF	blue	na
chrUn	25531223	25532964	1742	SAT1_CF	blue	na
chrUn	25533007	25533380	374	SAT1_CF	blue	na
chrUn	25533397	25533917	521	SAT1_CF	blue	na
chrUn	25533930	25534633	704	SAT1_CF	blue	na
chrUn	25534645	25535322	678	SAT1_CF	blue	na

chrUn	25535365	25537105	1741	SAT1_CF	blue	na
chrUn	25537144	25538948	1805	SAT1_CF	blue	na
chrUn	25538975	25539538	564	SAT1_CF	blue	na
chrUn	25539618	25540143	526	SAT1_CF	blue	na
chrUn	25540355	25540510	156	SAT1_CF	blue	na
chrUn	25540555	25541055	501	SAT1_CF	blue	na
chrUn	25541068	25541173	106	SAT1_CF	blue	na
chrUn	25541196	25542891	1696	SAT1_CF	blue	na
chrUn	25542904	25543664	761	SAT1_CF	blue	na
chrUn	25543676	25544359	684	SAT1_CF	blue	na
chrUn	25544371	25544968	598	SAT1_CF	blue	na
chrUn	25544988	25545297	310	SAT1_CF	blue	na
chrUn	25545310	25545390	81	SAT1_CF	blue	na
chrUn	25545404	25545956	553	SAT1_CF	blue	na
chrUn	25545968	25546393	426	SAT1_CF	blue	na
chrUn	25546605	25547222	618	SAT1_CF	blue	na
chrUn	25547247	25547572	326	SAT1_CF	blue	na
chrUn	25547652	25548191	540	SAT1_CF	blue	na
chrUn	25548202	25548538	337	SAT1_CF	blue	na
chrUn	25548574	25549068	495	SAT1_CF	blue	na
chrUn	25549088	25549769	682	SAT1_CF	blue	na
chrUn	25549782	25549888	107	SAT1_CF	blue	na
chrUn	25549909	25550464	556	SAT1_CF	blue	na
chrUn	25550476	25551341	866	SAT1_CF	blue	na
chrUn	25551383	25553226	1844	SAT1_CF	blue	na
chrUn	25553279	25553780	502	SAT1_CF	blue	na
chrUn	25553793	25553899	107	SAT1_CF	blue	na
chrUn	25553921	25554466	546	SAT1_CF	blue	na
chrUn	25554536	25554999	464	SAT1_CF	blue	na
chrUn	25555011	25556521	1511	SAT1_CF	blue	na
chrUn	25556559	25557712	1154	SAT1_CF	blue	na
chrUn	25557791	25558331	541	SAT1_CF	blue	na
chrUn	25558377	25558750	374	SAT1_CF	blue	na
chrUn	25558767	25561708	2942	SAT1_CF	blue	na
chrUn	25561748	25562247	500	SAT1_CF	blue	na
chrUn	25562260	25563020	761	SAT1_CF	blue	na
chrUn	25563033	25563104	72	SAT1_CF	blue	na
chrUn	25563127	25563677	551	SAT1_CF	blue	na
chrUn	25563695	25564394	700	SAT1_CF	blue	na
chrUn	25564427	25564932	506	SAT1_CF	blue	na
chrUn	25564964	25565667	704	SAT1_CF	blue	na
chrUn	25565680	25566833	1154	SAT1_CF	blue	na

chrUn	25566846	25567245	400	SAT1_CF	blue	na
chrUn	25567266	25567747	482	SAT1_CF	blue	na
chrUn	25567760	25567868	109	SAT1_CF	blue	na
chrUn	25567889	25568417	529	SAT1_CF	blue	na
chrUn	25568474	25569184	711	SAT1_CF	blue	na
chrUn	25569226	25570559	1334	SAT1_CF	blue	na
chrUn	25570572	25571245	674	SAT1_CF	blue	na
chrUn	25572392	25572900	509	SAT1_CF	blue	na
chrUn	25572928	25573085	158	SAT1_CF	blue	na
chrUn	25573130	25573630	501	SAT1_CF	blue	na
chrUn	25573642	25573751	110	SAT1_CF	blue	na
chrUn	25573772	25574403	632	SAT1_CF	blue	na
chrUn	25574415	25575079	665	SAT1_CF	blue	na
chrUn	25575136	25577246	2111	SAT1_CF	blue	na
chrUn	25577289	25578345	1057	SAT1_CF	blue	na
chrUn	25578367	25579034	668	SAT1_CF	blue	na
chrUn	25579077	25579621	545	SAT1_CF	blue	na
chrUn	25579649	25580142	494	SAT1_CF	blue	na
chrUn	25580199	25581998	1800	SAT1_CF	blue	na
chrUn	25582026	25582693	668	SAT1_CF	blue	na
chrUn	25582706	25583380	675	SAT1_CF	blue	na
chrUn	25583423	25583967	545	SAT1_CF	blue	na
chrUn	25583995	25584488	494	SAT1_CF	blue	na
chrUn	25584545	25585111	567	SAT1_CF	blue	na
chrUn	25585191	25585716	526	SAT1_CF	blue	na
chrUn	25585731	25585867	137	SAT1_CF	blue	na
chrUn	25585963	25587520	1558	SAT1_CF	blue	na
chrUn	25587565	25588071	507	SAT1_CF	blue	na
chrUn	25588118	25588490	373	SAT1_CF	blue	na
chrUn	25588545	25589134	590	SAT1_CF	blue	na
chrUn	25589147	25590382	1236	SAT1_CF	blue	na
chrUn	25590395	25591041	647	SAT1_CF	blue	na
chrUn	25591085	25592193	1109	SAT1_CF	blue	na
chrUn	25592235	25593086	852	SAT1_CF	blue	na
chrUn	25593113	25593369	257	SAT1_CF	blue	na
chrUn	25593382	25595532	2151	SAT1_CF	blue	na
chrUn	25595545	25596208	664	SAT1_CF	blue	na
chrUn	25596265	25596817	553	SAT1_CF	blue	na
chrUn	25596834	25598859	2026	SAT1_CF	blue	na
chrUn	25598883	25599732	850	SAT1_CF	blue	na
chrUn	25599743	25599818	76	SAT1_CF	blue	na
chrUn	25599874	25600418	545	SAT1_CF	blue	na

chrUn	25600430	25601528	1099	SAT1_CF	blue	na
chrUn	25601549	25602068	520	SAT1_CF	blue	na
chrUn	25602081	25602770	690	SAT1_CF	blue	na
chrUn	25602782	25603608	827	SAT1_CF	blue	na
chrUn	25603621	25604476	856	SAT1_CF	blue	na
chrUn	25604518	25605297	780	SAT1_CF	blue	na
chrUn	25605412	25605958	547	SAT1_CF	blue	na
chrUn	25605971	25607696	1726	SAT1_CF	blue	na
chrUn	25607756	25608304	549	SAT1_CF	blue	na
chrUn	25608351	25608753	403	SAT1_CF	blue	na
chrUn	25608774	25610013	1240	SAT1_CF	blue	na
chrUn	25610045	25610552	508	SAT1_CF	blue	na
chrUn	25610582	25611283	702	SAT1_CF	blue	na
chrUn	25611325	25612864	1540	SAT1_CF	blue	na
chrUn	25612943	25613855	913	SAT1_CF	blue	na
chrUn	25613942	25614489	548	SAT1_CF	blue	na
chrUn	25614501	25615175	675	SAT1_CF	blue	na
chrUn	25615217	25616197	981	SAT1_CF	blue	na
chrUn	25616276	25616823	548	SAT1_CF	blue	na
chrUn	25616940	25617263	324	SAT1_CF	blue	na
chrUn	25617293	25617814	522	SAT1_CF	blue	na
chrUn	25617827	25618833	1007	SAT1_CF	blue	na
chrUn	25618845	25619663	819	SAT1_CF	blue	na
chrUn	25619707	25620661	955	SAT1_CF	blue	na
chrUn	25620704	25621205	502	SAT1_CF	blue	na
chrUn	25621217	25621323	107	SAT1_CF	blue	na
chrUn	25621346	25622019	674	SAT1_CF	blue	na
chrUn	25622041	25622586	546	SAT1_CF	blue	na
chrUn	25622629	25623628	1000	SAT1_CF	blue	na
chrUn	25623643	25624623	981	SAT1_CF	blue	na
chrUn	25624691	25625211	521	SAT1_CF	blue	na
chrUn	25625233	25625864	632	SAT1_CF	blue	na
chrUn	25625909	25626997	1089	SAT1_CF	blue	na
chrUn	25627046	25627522	477	SAT1_CF	blue	na
chrUn	25627569	25628068	500	SAT1_CF	blue	na
chrUn	25628081	25628763	683	SAT1_CF	blue	na
chrUn	25628787	25629636	850	SAT1_CF	blue	na
chrUn	25629678	25630213	536	SAT1_CF	blue	na
chrUn	25630279	25630623	345	SAT1_CF	blue	na
chrUn	25630668	25631875	1208	SAT1_CF	blue	na
chrUn	25631887	25632566	680	SAT1_CF	blue	na
chrUn	25632609	25634036	1428	SAT1_CF	blue	na

chrUn	25634048	25635473	1426	SAT1_CF	blue	na
chrUn	25635552	25636098	547	SAT1_CF	blue	na
chrUn	25636176	25636724	549	SAT1_CF	blue	na
chrUn	25636736	25636838	103	SAT1_CF	blue	na
chrUn	25636867	25637412	546	SAT1_CF	blue	na
chrUn	25637432	25637599	168	SAT1_CF	blue	na
chrUn	25637641	25638423	783	SAT1_CF	blue	na
chrUn	25638478	25639086	609	SAT1_CF	blue	na
chrUn	25639097	25639451	355	SAT1_CF	blue	na
chrUn	25639538	25640238	701	SAT1_CF	blue	na
chrUn	25641664	25642183	520	SAT1_CF	blue	na
chrUn	25642268	25642762	495	SAT1_CF	blue	na
chrUn	25642796	25643465	670	SAT1_CF	blue	na
chrUn	25643477	25645841	2365	SAT1_CF	blue	na
chrUn	25645854	25647127	1274	SAT1_CF	blue	na
chrUn	25647168	25647821	654	SAT1_CF	blue	na
chrUn	25647866	25648097	232	SAT1_CF	blue	na
chrUn	25648140	25648724	585	SAT1_CF	blue	na
chrUn	25648736	25649449	714	SAT1_CF	blue	na
chrUn	25649531	25650071	541	SAT1_CF	blue	na
chrUn	25650084	25650585	502	SAT1_CF	blue	na
chrUn	25650598	25651835	1238	SAT1_CF	blue	na
chrUn	25651874	25652682	809	SAT1_CF	blue	na
chrUn	25652710	25652867	158	SAT1_CF	blue	na
chrUn	25652893	25653862	970	SAT1_CF	blue	na
chrUn	25653874	25654550	677	SAT1_CF	blue	na
chrUn	25654598	25655555	958	SAT1_CF	blue	na
chrUn	25655634	25656594	961	SAT1_CF	blue	na
chrUn	25656634	25658004	1371	SAT1_CF	blue	na
chrUn	25658017	25658689	673	SAT1_CF	blue	na
chrUn	25658703	25658868	166	SAT1_CF	blue	na
chrUn	25658918	25659461	544	SAT1_CF	blue	na
chrUn	25659505	25660627	1123	SAT1_CF	blue	na
chrUn	25660639	25661909	1271	SAT1_CF	blue	na
chrUn	25661935	25662453	519	SAT1_CF	blue	na
chrUn	25662465	25662570	106	SAT1_CF	blue	na
chrUn	25662596	25663150	555	SAT1_CF	blue	na
chrUn	25663162	25663270	109	SAT1_CF	blue	na
chrUn	25663290	25663835	546	SAT1_CF	blue	na
chrUn	25663878	25664999	1122	SAT1_CF	blue	na
chrUn	25665018	25665538	521	SAT1_CF	blue	na
chrUn	25665551	25666233	683	SAT1_CF	blue	na

chrUn	25666257	25667943	1687	SAT1_CF	blue	na
chrUn	25667988	25668674	687	SAT1_CF	blue	na
chrUn	25668686	25669003	318	SAT1_CF	blue	na
chrUn	25669076	25669646	571	SAT1_CF	blue	na
chrUn	25669665	25671155	1491	SAT1_CF	blue	na
chrUn	25671234	25672147	914	SAT1_CF	blue	na
chrUn	25672234	25673849	1616	SAT1_CF	blue	na
chrUn	25673898	25674492	595	SAT1_CF	blue	na
chrUn	25675899	25676416	518	SAT1_CF	blue	na
chrUn	25676503	25676997	495	SAT1_CF	blue	na
chrUn	25677017	25677123	107	SAT1_CF	blue	na
chrUn	25677146	25677700	555	SAT1_CF	blue	na
chrUn	25677723	25678386	664	SAT1_CF	blue	na
chrUn	25678431	25679060	630	SAT1_CF	blue	na
chrUn	25679088	25680280	1193	SAT1_CF	blue	na
chrUn	25680308	25680444	137	SAT1_CF	blue	na
chrUn	25680491	25681532	1042	SAT1_CF	blue	na
chrUn	25681543	25682035	493	SAT1_CF	blue	na
chrUn	25682049	25683414	1366	SAT1_CF	blue	na
chrUn	25683441	25683962	522	SAT1_CF	blue	na
chrUn	25684010	25684520	511	SAT1_CF	blue	na
chrUn	25684538	25684999	462	SAT1_CF	blue	na
chrUn	25685043	25686862	1820	SAT1_CF	blue	na
chrUn	25686900	25688011	1112	SAT1_CF	blue	na
chrUn	25688031	25689480	1450	SAT1_CF	blue	na
chrUn	25689577	25690119	543	SAT1_CF	blue	na
chrUn	25690163	25690723	561	SAT1_CF	blue	na
chrUn	25690766	25691291	526	SAT1_CF	blue	na
chrUn	25691306	25691442	137	SAT1_CF	blue	na
chrUn	25691527	25692009	483	SAT1_CF	blue	na
chrUn	25692022	25692704	683	SAT1_CF	blue	na
chrUn	25692716	25693574	859	SAT1_CF	blue	na
chrUn	25693616	25695062	1447	SAT1_CF	blue	na
chrUn	25695076	25695907	832	SAT1_CF	blue	na
chrUn	25695920	25696027	108	SAT1_CF	blue	na
chrUn	25696049	25696623	575	SAT1_CF	blue	na
chrUn	25696635	25697311	677	SAT1_CF	blue	na
chrUn	25697324	25698153	830	SAT1_CF	blue	na
chrUn	25698183	25698822	640	SAT1_CF	blue	na
chrUn	25698843	25699356	514	SAT1_CF	blue	na
chrUn	25699376	25700058	683	SAT1_CF	blue	na
chrUn	25700070	25701369	1300	SAT1_CF	blue	na

chrUn	25701473	25702570	1098	SAT1_CF	blue	na
chrUn	25702584	25702689	106	SAT1_CF	blue	na
chrUn	25702712	25703273	562	SAT1_CF	blue	na
chrUn	25703285	25704191	907	SAT1_CF	blue	na
chrUn	25704220	25706039	1820	SAT1_CF	blue	na
chrUn	25706079	25706580	502	SAT1_CF	blue	na
chrUn	25706606	25707349	744	SAT1_CF	blue	na
chrUn	25707361	25708221	861	SAT1_CF	blue	na
chrUn	25708264	25709893	1630	SAT1_CF	blue	na
chrUn	25709921	25710056	136	SAT1_CF	blue	na
chrUn	25710122	25711309	1188	SAT1_CF	blue	na
chrUn	25711352	25712449	1098	SAT1_CF	blue	na
chrUn	25712461	25712562	102	SAT1_CF	blue	na
chrUn	25712591	25713138	548	SAT1_CF	blue	na
chrUn	25713180	25714281	1102	SAT1_CF	blue	na
chrUn	25714299	25714822	524	SAT1_CF	blue	na
chrUn	25714837	25714993	157	SAT1_CF	blue	na
chrUn	25715039	25715540	502	SAT1_CF	blue	na
chrUn	25715567	25716402	836	SAT1_CF	blue	na
chrUn	25716421	25716940	520	SAT1_CF	blue	na
chrUn	25716952	25718644	1693	SAT1_CF	blue	na
chrUn	25718688	25719228	541	SAT1_CF	blue	na
chrUn	25719240	25720098	859	SAT1_CF	blue	na
chrUn	25720140	25721010	871	SAT1_CF	blue	na
chrUn	25721037	25721586	550	SAT1_CF	blue	na
chrUn	25721668	25721835	168	SAT1_CF	blue	na
chrUn	25722018	25722203	186	SAT1_CF	blue	na
chrUn	25722239	25722739	501	SAT1_CF	blue	na
chrUn	25722752	25722857	106	SAT1_CF	blue	na
chrUn	25722880	25724573	1694	SAT1_CF	blue	na
chrUn	25724586	25725345	760	SAT1_CF	blue	na
chrUn	25725357	25726040	684	SAT1_CF	blue	na
chrUn	25726052	25726980	929	SAT1_CF	blue	na
chrUn	25726993	25728075	1083	SAT1_CF	blue	na
chrUn	25728117	25731079	2963	SAT1_CF	blue	na
chrUn	25731090	25731692	603	SAT1_CF	blue	na
chrUn	25731704	25732490	787	SAT1_CF	blue	na
chrUn	25732535	25733037	503	SAT1_CF	blue	na
chrUn	25733050	25733156	107	SAT1_CF	blue	na
chrUn	25733178	25733731	554	SAT1_CF	blue	na
chrUn	25733743	25734749	1007	SAT1_CF	blue	na
chrUn	25734831	25735357	527	SAT1_CF	blue	na

chrUn	25735401	25736440	1040	SAT1_CF	blue	na
chrUn	25736483	25737027	545	SAT1_CF	blue	na
chrUn	25737040	25737876	837	SAT1_CF	blue	na
chrUn	25737890	25738591	702	SAT1_CF	blue	na
chrUn	25738613	25740964	2352	SAT1_CF	blue	na
chrUn	25740979	25741136	158	SAT1_CF	blue	na
chrUn	25741181	25741681	501	SAT1_CF	blue	na
chrUn	25741698	25742453	756	SAT1_CF	blue	na
chrUn	25742474	25742539	66	SAT1_CF	blue	na
chrUn	25742560	25743077	518	SAT1_CF	blue	na
chrUn	25743089	25743949	861	SAT1_CF	blue	na
chrUn	25743991	25745687	1697	SAT1_CF	blue	na
chrUn	25745869	25746590	722	SAT1_CF	blue	na
chrUn	25746603	25748431	1829	SAT1_CF	blue	na
chrUn	25748477	25748607	131	SAT1_CF	blue	na
chrUn	25748629	25749187	559	SAT1_CF	blue	na
chrUn	25749199	25750911	1713	SAT1_CF	blue	na
chrUn	25750966	25751573	608	SAT1_CF	blue	na
chrUn	25751586	25751941	356	SAT1_CF	blue	na
chrUn	25752058	25752576	519	SAT1_CF	blue	na
chrUn	25752607	25753133	527	SAT1_CF	blue	na
chrUn	25753177	25754416	1240	SAT1_CF	blue	na
chrUn	25754431	25754588	158	SAT1_CF	blue	na
chrUn	25754633	25755906	1274	SAT1_CF	blue	na
chrUn	25755918	25756027	110	SAT1_CF	blue	na
chrUn	25756048	25756592	545	SAT1_CF	blue	na
chrUn	25756653	25757378	726	SAT1_CF	blue	na
chrUn	25757511	25758655	1145	SAT1_CF	blue	na
chrUn	25758713	25759191	479	SAT1_CF	blue	na
chrUn	25759204	25759309	106	SAT1_CF	blue	na
chrUn	25759332	25760506	1175	SAT1_CF	blue	na
chrUn	25760538	25760672	135	SAT1_CF	blue	na
chrUn	25760738	25761237	500	SAT1_CF	blue	na
chrUn	25761250	25761924	675	SAT1_CF	blue	na
chrUn	25761967	25763993	2027	SAT1_CF	blue	na
chrUn	25764076	25764614	539	SAT1_CF	blue	na
chrUn	25764634	25764741	108	SAT1_CF	blue	na
chrUn	25764762	25765317	556	SAT1_CF	blue	na
chrUn	25765329	25766536	1208	SAT1_CF	blue	na
chrUn	25766551	25766687	137	SAT1_CF	blue	na
chrUn	25766782	25767301	520	SAT1_CF	blue	na
chrUn	25767358	25767904	547	SAT1_CF	blue	na

chrUn	25767946	25768800	855	SAT1_CF	blue	na
chrUn	25768845	25769353	509	SAT1_CF	blue	na
chrUn	25769364	25769766	403	SAT1_CF	blue	na
chrUn	25769806	25770307	502	SAT1_CF	blue	na
chrUn	25770334	25771005	672	SAT1_CF	blue	na
chrUn	25771019	25771096	78	SAT1_CF	blue	na
chrUn	25771113	25771632	520	SAT1_CF	blue	na
chrUn	25771644	25773291	1648	SAT1_CF	blue	na
chrUn	25773370	25773918	549	SAT1_CF	blue	na
chrUn	25773961	25774361	401	SAT1_CF	blue	na
chrUn	25774407	25774908	502	SAT1_CF	blue	na
chrUn	25774931	25775602	672	SAT1_CF	blue	na
chrUn	25775614	25775730	117	SAT1_CF	blue	na
chrUn	25775743	25777391	1649	SAT1_CF	blue	na
chrUn	25777439	25778359	921	SAT1_CF	blue	na
chrUn	25778401	25778893	493	SAT1_CF	blue	na
chrUn	25778913	25780289	1377	SAT1_CF	blue	na
chrUn	25780301	25781054	754	SAT1_CF	blue	na
chrUn	25781074	25783373	2300	SAT1_CF	blue	na
chrUn	25783386	25784580	1195	SAT1_CF	blue	na
chrUn	25784594	25785937	1344	SAT1_CF	blue	na
chrUn	25785981	25786838	858	SAT1_CF	blue	na
chrUn	26178913	26179715	803	SAT1_CF	blue	na
chrUn	26179728	26180243	516	SAT1_CF	blue	na
chrUn	26180279	26180652	374	SAT1_CF	blue	na
chrUn	26180695	26181244	550	SAT1_CF	blue	na
chrUn	26181323	26183614	2292	SAT1_CF	blue	na
chrUn	26183635	26184202	568	SAT1_CF	blue	na
chrUn	26184260	26186118	1859	SAT1_CF	blue	na
chrUn	26186188	26186860	673	SAT1_CF	blue	na
chrUn	26186872	26188813	1942	SAT1_CF	blue	na
chrUn	26188835	26190034	1200	SAT1_CF	blue	na
chrUn	26190074	26190475	402	SAT1_CF	blue	na
chrUn	26190486	26191008	523	SAT1_CF	blue	na
chrUn	26191026	26191745	720	SAT1_CF	blue	na
chrUn	26191772	26192370	599	SAT1_CF	blue	na
chrUn	26192383	26193374	992	SAT1_CF	blue	na
chrUn	26193392	26195196	1805	SAT1_CF	blue	na
chrUn	26195208	26195969	762	SAT1_CF	blue	na
chrUn	26195982	26196483	502	SAT1_CF	blue	na
chrUn	26196540	26196922	383	SAT1_CF	blue	na
chrUn	26196934	26198720	1787	SAT1_CF	blue	na

chrUn	26198732	26199923	1192	SAT1_CF	blue	na
chrUn	26199935	26200496	562	SAT1_CF	blue	na
chrUn	26200516	26200624	109	SAT1_CF	blue	na
chrUn	26200637	26201339	703	SAT1_CF	blue	na
chrUn	26201354	26202626	1273	SAT1_CF	blue	na
chrUn	26202654	26202754	101	SAT1_CF	blue	na
chrUn	26202767	26203268	502	SAT1_CF	blue	na
chrUn	26203355	26203897	543	SAT1_CF	blue	na
chrUn	26203948	26204831	884	SAT1_CF	blue	na
chrUn	26204874	26206715	1842	SAT1_CF	blue	na
chrUn	26206787	26207322	536	SAT1_CF	blue	na
chrUn	26207401	26208065	665	SAT1_CF	blue	na
chrUn	26208079	26208570	492	SAT1_CF	blue	na
chrUn	26208581	26210961	2381	SAT1_CF	blue	na
chrUn	26210983	26211504	522	SAT1_CF	blue	na
chrUn	26211551	26211687	137	SAT1_CF	blue	na
chrUn	26211702	26213567	1866	SAT1_CF	blue	na
chrUn	26213610	26214286	677	SAT1_CF	blue	na
chrUn	26214299	26214980	682	SAT1_CF	blue	na
chrUn	26215000	26215494	495	SAT1_CF	blue	na
chrUn	26215534	26216106	573	SAT1_CF	blue	na
chrUn	26217523	26219181	1659	SAT1_CF	blue	na
chrUn	26219192	26219875	684	SAT1_CF	blue	na
chrUn	26219887	26220570	684	SAT1_CF	blue	na
chrUn	26220583	26221084	502	SAT1_CF	blue	na
chrUn	26221124	26222082	959	SAT1_CF	blue	na
chrUn	26222113	26222832	720	SAT1_CF	blue	na
chrUn	26222876	26223418	543	SAT1_CF	blue	na
chrUn	26223444	26224242	799	SAT1_CF	blue	na
chrUn	26224255	26225146	892	SAT1_CF	blue	na
chrUn	26225158	26225915	758	SAT1_CF	blue	na
chrUn	26225928	26226429	502	SAT1_CF	blue	na
chrUn	26226469	26227980	1512	SAT1_CF	blue	na
chrUn	26228008	26228640	633	SAT1_CF	blue	na
chrUn	26228683	26229229	547	SAT1_CF	blue	na
chrUn	26229287	26229958	672	SAT1_CF	blue	na
chrUn	26229981	26231479	1499	SAT1_CF	blue	na
chrUn	26231560	26232524	965	SAT1_CF	blue	na
chrUn	26232566	26233110	545	SAT1_CF	blue	na
chrUn	26233122	26233231	110	SAT1_CF	blue	na
chrUn	26233243	26233717	475	SAT1_CF	blue	na
chrUn	26233787	26234459	673	SAT1_CF	blue	na

chrUn	26234471	26235231	761	SAT1_CF	blue	na
chrUn	26235243	26235918	676	SAT1_CF	blue	na
chrUn	26235937	26236436	500	SAT1_CF	blue	na
chrUn	26236468	26236844	377	SAT1_CF	blue	na
chrUn	26236887	26238219	1333	SAT1_CF	blue	na
chrUn	26238231	26238726	496	SAT1_CF	blue	na
chrUn	26238801	26239346	546	SAT1_CF	blue	na
chrUn	26239376	26241018	1643	SAT1_CF	blue	na
chrUn	26241045	26241526	482	SAT1_CF	blue	na
chrUn	26241552	26242618	1067	SAT1_CF	blue	na
chrUn	26242636	26243135	500	SAT1_CF	blue	na
chrUn	26243188	26245175	1988	SAT1_CF	blue	na
chrUn	26245218	26245897	680	SAT1_CF	blue	na
chrUn	26245909	26247137	1229	SAT1_CF	blue	na
chrUn	26247218	26247740	523	SAT1_CF	blue	na
chrUn	26247762	26248040	279	SAT1_CF	blue	na
chrUn	26248082	26248626	545	SAT1_CF	blue	na
chrUn	26248668	26249215	548	SAT1_CF	blue	na
chrUn	26249272	26249790	519	SAT1_CF	blue	na
chrUn	26249905	26250257	353	SAT1_CF	blue	na
chrUn	26250270	26251627	1358	SAT1_CF	blue	na
chrUn	26251639	26252158	520	SAT1_CF	blue	na
chrUn	26252184	26252691	508	SAT1_CF	blue	na
chrUn	26252704	26253222	519	SAT1_CF	blue	na
chrUn	26253241	26254166	926	SAT1_CF	blue	na
chrUn	26254194	26255104	911	SAT1_CF	blue	na
chrUn	26255116	26256299	1184	SAT1_CF	blue	na
chrUn	26256359	26256762	404	SAT1_CF	blue	na
chrUn	26256773	26257295	523	SAT1_CF	blue	na
chrUn	26257327	26258376	1050	SAT1_CF	blue	na
chrUn	26258419	26259093	675	SAT1_CF	blue	na
chrUn	26259106	26259606	501	SAT1_CF	blue	na
chrUn	26259672	26259807	136	SAT1_CF	blue	na
chrUn	26259840	26261107	1268	SAT1_CF	blue	na
chrUn	26261120	26261803	684	SAT1_CF	blue	na
chrUn	26261816	26262333	518	SAT1_CF	blue	na
chrUn	26262360	26263620	1261	SAT1_CF	blue	na
chrUn	26263632	26264314	683	SAT1_CF	blue	na
chrUn	26264334	26264847	514	SAT1_CF	blue	na
chrUn	26264873	26265030	158	SAT1_CF	blue	na
chrUn	26265058	26265566	509	SAT1_CF	blue	na
chrUn	26265609	26266173	565	SAT1_CF	blue	na

chrUn	26266201	26266828	628	SAT1_CF	blue	na
chrUn	26266873	26267545	673	SAT1_CF	blue	na
chrUn	26267557	26268111	555	SAT1_CF	blue	na
chrUn	26268173	26268729	557	SAT1_CF	blue	na
chrUn	26268785	26268970	186	SAT1_CF	blue	na
chrUn	26269150	26269352	203	SAT1_CF	blue	na
chrUn	26269395	26269943	549	SAT1_CF	blue	na
chrUn	26270022	26271648	1627	SAT1_CF	blue	na
chrUn	26271671	26272494	824	SAT1_CF	blue	na
chrUn	26273017	26273241	225	SAT1_CF	blue	na
chrUn	26273252	26274031	780	SAT1_CF	blue	na
chrUn	26274112	26274764	653	SAT1_CF	blue	na
chrUn	26274776	26275459	684	SAT1_CF	blue	na
chrUn	26275472	26278089	2618	SAT1_CF	blue	na
chrUn	26278105	26278531	427	SAT1_CF	blue	na
chrUn	26278545	26279182	638	SAT1_CF	blue	na
chrUn	26279200	26280023	824	SAT1_CF	blue	na
chrUn	26280036	26280555	520	SAT1_CF	blue	na
chrUn	26280589	26281278	690	SAT1_CF	blue	na
chrUn	26281308	26282929	1622	SAT1_CF	blue	na
chrUn	26282943	26283624	682	SAT1_CF	blue	na
chrUn	26283636	26284190	555	SAT1_CF	blue	na
chrUn	26284253	26284849	597	SAT1_CF	blue	na
chrUn	26284875	26285030	156	SAT1_CF	blue	na
chrUn	26285045	26285571	527	SAT1_CF	blue	na
chrUn	26285602	26287551	1950	SAT1_CF	blue	na
chrUn	26287564	26288065	502	SAT1_CF	blue	na
chrUn	26288118	26288507	390	SAT1_CF	blue	na
chrUn	26288520	26289041	522	SAT1_CF	blue	na
chrUn	26289108	26290066	959	SAT1_CF	blue	na
chrUn	26290114	26290791	678	SAT1_CF	blue	na
chrUn	26290803	26291487	685	SAT1_CF	blue	na
chrUn	26291500	26292000	501	SAT1_CF	blue	na
chrUn	26292087	26292441	355	SAT1_CF	blue	na
chrUn	26292452	26292503	52	SAT1_CF	blue	na
chrUn	26292526	26293206	681	SAT1_CF	blue	na
chrUn	26293219	26293739	521	SAT1_CF	blue	na
chrUn	26293809	26293980	172	SAT1_CF	blue	na
chrUn	26294062	26294624	563	SAT1_CF	blue	na
chrUn	26294667	26295925	1259	SAT1_CF	blue	na
chrUn	26296004	26297414	1411	SAT1_CF	blue	na
chrUn	26297427	26298110	684	SAT1_CF	blue	na

chrUn	26298122	26298805	684	SAT1_CF	blue	na
chrUn	26298818	26299317	500	SAT1_CF	blue	na
chrUn	26299357	26299616	260	SAT1_CF	blue	na
chrUn	26299660	26300207	548	SAT1_CF	blue	na
chrUn	26300250	26300941	692	SAT1_CF	blue	na
chrUn	26300953	26303076	2124	SAT1_CF	blue	na
chrUn	26303118	26303848	731	SAT1_CF	blue	na
chrUn	26303868	26303977	110	SAT1_CF	blue	na
chrUn	26303989	26305455	1467	SAT1_CF	blue	na
chrUn	26305522	26307114	1593	SAT1_CF	blue	na
chrUn	26307256	26307775	520	SAT1_CF	blue	na
chrUn	26307801	26307958	158	SAT1_CF	blue	na
chrUn	26307973	26308497	525	SAT1_CF	blue	na
chrUn	26308578	26309142	565	SAT1_CF	blue	na
chrUn	26309169	26309797	629	SAT1_CF	blue	na
chrUn	26309842	26310507	666	SAT1_CF	blue	na
chrUn	26310529	26311214	686	SAT1_CF	blue	na
chrUn	26311226	26311708	483	SAT1_CF	blue	na
chrUn	26311768	26312165	398	SAT1_CF	blue	na
chrUn	26312177	26312726	550	SAT1_CF	blue	na
chrUn	26312805	26313766	962	SAT1_CF	blue	na
chrUn	26313809	26316080	2272	SAT1_CF	blue	na
chrUn	26316092	26316775	684	SAT1_CF	blue	na
chrUn	26316795	26317289	495	SAT1_CF	blue	na
chrUn	26317329	26317907	579	SAT1_CF	blue	na
chrUn	26317928	26318480	553	SAT1_CF	blue	na
chrUn	26318499	26319069	571	SAT1_CF	blue	na
chrUn	26319102	26319642	541	SAT1_CF	blue	na
chrUn	26319691	26321001	1311	SAT1_CF	blue	na
chrUn	26321043	26321773	731	SAT1_CF	blue	na
chrUn	26321794	26321902	109	SAT1_CF	blue	na
chrUn	26321915	26322417	503	SAT1_CF	blue	na
chrUn	26322457	26323376	920	SAT1_CF	blue	na
chrUn	26323420	26323721	302	SAT1_CF	blue	na
chrUn	26323769	26325102	1334	SAT1_CF	blue	na
chrUn	26325115	26325617	503	SAT1_CF	blue	na
chrUn	26325703	26327202	1500	SAT1_CF	blue	na
chrUn	26327214	26327765	552	SAT1_CF	blue	na
chrUn	26327787	26327859	73	SAT1_CF	blue	na
chrUn	26327872	26328799	928	SAT1_CF	blue	na
chrUn	26328811	26329494	684	SAT1_CF	blue	na
chrUn	26329506	26330527	1022	SAT1_CF	blue	na

chrUn	26330571	26331353	783	SAT1_CF	blue	na
chrUn	26331395	26332260	866	SAT1_CF	blue	na
chrUn	26332272	26332826	555	SAT1_CF	blue	na
chrUn	26332848	26332953	106	SAT1_CF	blue	na
chrUn	26332966	26333439	474	SAT1_CF	blue	na
chrUn	26333495	26333570	76	SAT1_CF	blue	na
chrUn	26333581	26335234	1654	SAT1_CF	blue	na
chrUn	26335246	26336020	775	SAT1_CF	blue	na
chrUn	26336035	26338203	2169	SAT1_CF	blue	na
chrUn	26338295	26338834	540	SAT1_CF	blue	na
chrUn	26338878	26339554	677	SAT1_CF	blue	na
chrUn	26339566	26340326	761	SAT1_CF	blue	na
chrUn	26340338	26341019	682	SAT1_CF	blue	na
chrUn	26341033	26342560	1528	SAT1_CF	blue	na
chrUn	26342604	26343601	998	SAT1_CF	blue	na
chrUn	26343644	26344190	547	SAT1_CF	blue	na
chrUn	26344211	26344320	110	SAT1_CF	blue	na
chrUn	26344332	26344853	522	SAT1_CF	blue	na
chrUn	26344870	26345242	373	SAT1_CF	blue	na
chrUn	26345285	26345895	611	SAT1_CF	blue	na
chrUn	26345950	26346617	668	SAT1_CF	blue	na
chrUn	26346629	26347391	763	SAT1_CF	blue	na
chrUn	26347403	26348085	683	SAT1_CF	blue	na
chrUn	26348098	26348600	503	SAT1_CF	blue	na
chrUn	26348646	26348753	108	SAT1_CF	blue	na
chrUn	26348817	26349341	525	SAT1_CF	blue	na
chrUn	26349384	26349784	401	SAT1_CF	blue	na
chrUn	26349864	26350446	583	SAT1_CF	blue	na
chrUn	26350473	26351103	631	SAT1_CF	blue	na
chrUn	26351146	26352555	1410	SAT1_CF	blue	na
chrUn	26352582	26353063	482	SAT1_CF	blue	na
chrUn	26353089	26353517	429	SAT1_CF	blue	na
chrUn	26353556	26354097	542	SAT1_CF	blue	na
chrUn	26354145	26354397	253	SAT1_CF	blue	na
chrUn	26354431	26354925	495	SAT1_CF	blue	na
chrUn	26355012	26355547	536	SAT1_CF	blue	na
chrUn	26356948	26357548	601	SAT1_CF	blue	na
chrUn	26357579	26358042	464	SAT1_CF	blue	na
chrUn	26358054	26358874	821	SAT1_CF	blue	na
chrUn	26358920	26360254	1335	SAT1_CF	blue	na
chrUn	26360266	26360768	503	SAT1_CF	blue	na
chrUn	26360832	26360967	136	SAT1_CF	blue	na

chrUn	26360982	26362251	1270	SAT1_CF	blue	na
chrUn	26362274	26362379	106	SAT1_CF	blue	na
chrUn	26362392	26362910	519	SAT1_CF	blue	na
chrUn	26362931	26363112	182	SAT1_CF	blue	na
chrUn	26363294	26364505	1212	SAT1_CF	blue	na
chrUn	26364516	26365060	545	SAT1_CF	blue	na
chrUn	26365101	26366402	1302	SAT1_CF	blue	na
chrUn	26366416	26366967	552	SAT1_CF	blue	na
chrUn	26366989	26367096	108	SAT1_CF	blue	na
chrUn	26367109	26368178	1070	SAT1_CF	blue	na
chrUn	26368235	26368714	480	SAT1_CF	blue	na
chrUn	26368728	26369272	545	SAT1_CF	blue	na
chrUn	26369314	26370023	710	SAT1_CF	blue	na
chrUn	26370044	26370153	110	SAT1_CF	blue	na
chrUn	26370165	26371552	1388	SAT1_CF	blue	na
chrUn	26371575	26371680	106	SAT1_CF	blue	na
chrUn	26371693	26372241	549	SAT1_CF	blue	na
chrUn	26372285	26373283	999	SAT1_CF	blue	na
chrUn	26373326	26373999	674	SAT1_CF	blue	na
chrUn	26374013	26374642	630	SAT1_CF	blue	na
chrUn	26374663	26374772	110	SAT1_CF	blue	na
chrUn	26374784	26375387	604	SAT1_CF	blue	na
chrUn	26375400	26375920	521	SAT1_CF	blue	na
chrUn	26375946	26376102	157	SAT1_CF	blue	na
chrUn	26376117	26378472	2356	SAT1_CF	blue	na
chrUn	26378494	26379196	703	SAT1_CF	blue	na
chrUn	26379209	26379474	266	SAT1_CF	blue	na
chrUn	26380474	26380923	450	SAT1_CF	blue	na
chrUn	26380973	26381673	701	SAT1_CF	blue	na
chrUn	26381723	26383044	1322	SAT1_CF	blue	na
chrUn	26383059	26383564	506	SAT1_CF	blue	na
chrUn	26383644	26383950	307	SAT1_CF	blue	na
chrUn	26383994	26385195	1202	SAT1_CF	blue	na
chrUn	26385265	26385807	543	SAT1_CF	blue	na
chrUn	26385828	26386116	289	SAT1_CF	blue	na
chrUn	26386158	26386874	717	SAT1_CF	blue	na
chrUn	26386892	26387393	502	SAT1_CF	blue	na
chrUn	26387433	26387828	396	SAT1_CF	blue	na
chrUn	26387849	26388355	507	SAT1_CF	blue	na
chrUn	26388435	26389398	964	SAT1_CF	blue	na
chrUn	26389443	26390808	1366	SAT1_CF	blue	na
chrUn	26390821	26391300	480	SAT1_CF	blue	na

chrUn	26391370	26391937	568	SAT1_CF	blue	na
chrUn	26393351	26393914	564	SAT1_CF	blue	na
chrUn	26393928	26394334	407	SAT1_CF	blue	na
chrUn	26394345	26395553	1209	SAT1_CF	blue	na
chrUn	26395571	26396061	491	SAT1_CF	blue	na
chrUn	26396075	26396756	682	SAT1_CF	blue	na
chrUn	26396768	26397451	684	SAT1_CF	blue	na
chrUn	26397465	26397964	500	SAT1_CF	blue	na
chrUn	26398020	26398369	350	SAT1_CF	blue	na
chrUn	26398403	26398912	510	SAT1_CF	blue	na
chrUn	26399028	26399990	963	SAT1_CF	blue	na
chrUn	26400033	26400706	674	SAT1_CF	blue	na
chrUn	26400718	26401958	1241	SAT1_CF	blue	na
chrUn	26401971	26402470	500	SAT1_CF	blue	na
chrUn	26402515	26402672	158	SAT1_CF	blue	na
chrUn	26402705	26404007	1303	SAT1_CF	blue	na
chrUn	26404019	26404502	484	SAT1_CF	blue	na
chrUn	26404554	26404742	189	SAT1_CF	blue	na
chrUn	26404753	26405273	521	SAT1_CF	blue	na
chrUn	26405294	26407628	2335	SAT1_CF	blue	na
chrUn	26407696	26408051	356	SAT1_CF	blue	na
chrUn	26408065	26409802	1738	SAT1_CF	blue	na
chrUn	26409815	26411650	1836	SAT1_CF	blue	na
chrUn	26411663	26411978	316	SAT1_CF	blue	na
chrUn	26412031	26412882	852	SAT1_CF	blue	na
chrUn	26412924	26414033	1110	SAT1_CF	blue	na
chrUn	26414077	26415274	1198	SAT1_CF	blue	na
chrUn	26415355	26415917	563	SAT1_CF	blue	na
chrUn	26415929	26417059	1131	SAT1_CF	blue	na
chrUn	26417176	26417684	509	SAT1_CF	blue	na
chrUn	26417712	26419600	1889	SAT1_CF	blue	na
chrUn	26419622	26420166	545	SAT1_CF	blue	na
chrUn	26420209	26420743	535	SAT1_CF	blue	na
chrUn	26420800	26421336	537	SAT1_CF	blue	na
chrUn	26421350	26421460	111	SAT1_CF	blue	na
chrUn	26421478	26421980	503	SAT1_CF	blue	na
chrUn	26421993	26422533	541	SAT1_CF	blue	na
chrUn	26422615	26423162	548	SAT1_CF	blue	na
chrUn	26423176	26424054	879	SAT1_CF	blue	na
chrUn	26424097	26424643	547	SAT1_CF	blue	na
chrUn	26424664	26424772	109	SAT1_CF	blue	na
chrUn	26424785	26425304	520	SAT1_CF	blue	na

chrUn	26425325	26426247	923	SAT1_CF	blue	na
chrUn	26426291	26427252	962	SAT1_CF	blue	na
chrUn	26427297	26427962	666	SAT1_CF	blue	na
chrUn	26427984	26429254	1271	SAT1_CF	blue	na
chrUn	26429297	26429963	667	SAT1_CF	blue	na
chrUn	26429976	26431158	1183	SAT1_CF	blue	na
chrUn	26431179	26431930	752	SAT1_CF	blue	na
chrUn	26431951	26432059	109	SAT1_CF	blue	na
chrUn	26432072	26432547	476	SAT1_CF	blue	na
chrUn	26432618	26433290	673	SAT1_CF	blue	na
chrUn	26433302	26433989	688	SAT1_CF	blue	na
chrUn	26434001	26434684	684	SAT1_CF	blue	na
chrUn	26434698	26435196	499	SAT1_CF	blue	na
chrUn	26435234	26436270	1037	SAT1_CF	blue	na
chrUn	26436282	26436833	552	SAT1_CF	blue	na
chrUn	26436861	26437055	195	SAT1_CF	blue	na
chrUn	26437098	26437662	565	SAT1_CF	blue	na
chrUn	26437706	26439021	1316	SAT1_CF	blue	na
chrUn	26439036	26439712	677	SAT1_CF	blue	na
chrUn	26439725	26440226	502	SAT1_CF	blue	na
chrUn	26443778	26444737	960	SAT1_CF	blue	na
chrUn	26444816	26445780	965	SAT1_CF	blue	na
chrUn	26445823	26447047	1225	SAT1_CF	blue	na
chrUn	26447082	26448321	1240	SAT1_CF	blue	na
chrUn	26448437	26449217	781	SAT1_CF	blue	na
chrUn	26449278	26449940	663	SAT1_CF	blue	na
chrUn	26449952	26450583	632	SAT1_CF	blue	na
chrUn	26450605	26450713	109	SAT1_CF	blue	na
chrUn	26450725	26451226	502	SAT1_CF	blue	na
chrUn	26451271	26451427	157	SAT1_CF	blue	na
chrUn	26451455	26453112	1658	SAT1_CF	blue	na
chrUn	26453154	26454711	1558	SAT1_CF	blue	na
chrUn	26454724	26455225	502	SAT1_CF	blue	na
chrUn	26455264	26455904	641	SAT1_CF	blue	na
chrUn	26455931	26457820	1890	SAT1_CF	blue	na
chrUn	26457841	26458480	640	SAT1_CF	blue	na
chrUn	26458501	26460538	2038	SAT1_CF	blue	na
chrUn	26460580	26461945	1366	SAT1_CF	blue	na
chrUn	26461958	26462458	501	SAT1_CF	blue	na
chrUn	26462496	26463264	769	SAT1_CF	blue	na
chrUn	26463285	26463363	79	SAT1_CF	blue	na
chrUn	26463378	26463887	510	SAT1_CF	blue	na

chrUn	26463913	26464069	157	SAT1_CF	blue	na
chrUn	26464134	26465451	1318	SAT1_CF	blue	na
chrUn	26465497	26466999	1503	SAT1_CF	blue	na
chrUn	26467011	26468570	1560	SAT1_CF	blue	na
chrUn	26468712	26469258	547	SAT1_CF	blue	na
chrUn	26469301	26470082	782	SAT1_CF	blue	na
chrUn	26470124	26470986	863	SAT1_CF	blue	na
chrUn	26470998	26472233	1236	SAT1_CF	blue	na
chrUn	26472276	26472822	547	SAT1_CF	blue	na
chrUn	26472879	26473546	668	SAT1_CF	blue	na
chrUn	26473558	26474320	763	SAT1_CF	blue	na
chrUn	26474332	26474833	502	SAT1_CF	blue	na
chrUn	26474873	26475255	383	SAT1_CF	blue	na
chrUn	26475283	26476354	1072	SAT1_CF	blue	na
chrUn	26476370	26476910	541	SAT1_CF	blue	na
chrUn	26476953	26477626	674	SAT1_CF	blue	na
chrUn	26477638	26478397	760	SAT1_CF	blue	na
chrUn	26478417	26478911	495	SAT1_CF	blue	na
chrUn	26478951	26479908	958	SAT1_CF	blue	na
chrUn	26479952	26480952	1001	SAT1_CF	blue	na
chrUn	26480995	26481659	665	SAT1_CF	blue	na
chrUn	26481672	26482172	501	SAT1_CF	blue	na
chrUn	26482212	26483134	923	SAT1_CF	blue	na
chrUn	26483163	26483363	201	SAT1_CF	blue	na
chrUn	26483622	26484281	660	SAT1_CF	blue	na
chrUn	26484331	26485184	854	SAT1_CF	blue	na
chrUn	26485196	26486068	873	SAT1_CF	blue	na
chrUn	26486082	26487345	1264	SAT1_CF	blue	na
chrUn	26487372	26488589	1218	SAT1_CF	blue	na
chrUn	26488632	26489297	666	SAT1_CF	blue	na
chrUn	26489310	26489811	502	SAT1_CF	blue	na
chrUn	26489851	26491123	1273	SAT1_CF	blue	na
chrUn	26491161	26491692	532	SAT1_CF	blue	na
chrUn	26491744	26492593	850	SAT1_CF	blue	na
chrUn	26492615	26493655	1041	SAT1_CF	blue	na
chrUn	26493697	26495777	2081	SAT1_CF	blue	na
chrUn	26495835	26495968	134	SAT1_CF	blue	na
chrUn	26495996	26497036	1041	SAT1_CF	blue	na
chrUn	26497064	26497694	631	SAT1_CF	blue	na
chrUn	26497737	26498289	553	SAT1_CF	blue	na
chrUn	26498310	26498629	320	SAT1_CF	blue	na
chrUn	26498641	26499327	687	SAT1_CF	blue	na

chrUn	26499338	26499840	503	SAT1_CF	blue	na
chrUn	26499876	26500249	374	SAT1_CF	blue	na
chrUn	26500292	26500838	547	SAT1_CF	blue	na
chrUn	26500866	26501039	174	SAT1_CF	blue	na
chrUn	26501117	26502287	1171	SAT1_CF	blue	na
chrUn	26502368	26504807	2440	SAT1_CF	blue	na
chrUn	26504821	26505312	492	SAT1_CF	blue	na
chrUn	26505323	26506005	683	SAT1_CF	blue	na
chrUn	26506017	26507699	1683	SAT1_CF	blue	na
chrUn	26507945	26508972	1028	SAT1_CF	blue	na
chrUn	26508997	26509100	104	SAT1_CF	blue	na
chrUn	26509113	26509634	522	SAT1_CF	blue	na
chrUn	26509655	26510055	401	SAT1_CF	blue	na
chrUn	26510066	26511760	1695	SAT1_CF	blue	na
chrUn	26511774	26513581	1808	SAT1_CF	blue	na
chrUn	26513623	26514956	1334	SAT1_CF	blue	na
chrUn	26514969	26515650	682	SAT1_CF	blue	na
chrUn	26515663	26516547	885	SAT1_CF	blue	na
chrUn	26516569	26517252	684	SAT1_CF	blue	na
chrUn	26517264	26517818	555	SAT1_CF	blue	na
chrUn	26517873	26517947	75	SAT1_CF	blue	na
chrUn	26517966	26518459	494	SAT1_CF	blue	na
chrUn	26518546	26519458	913	SAT1_CF	blue	na
chrUn	26519537	26520500	964	SAT1_CF	blue	na
chrUn	26520543	26521912	1370	SAT1_CF	blue	na
chrUn	26521924	26522607	684	SAT1_CF	blue	na
chrUn	26522619	26523170	552	SAT1_CF	blue	na
chrUn	26523198	26523392	195	SAT1_CF	blue	na
chrUn	26523435	26523997	563	SAT1_CF	blue	na
chrUn	26524041	26525889	1849	SAT1_CF	blue	na
chrUn	26526141	26526277	137	SAT1_CF	blue	na
chrUn	26526290	26526790	501	SAT1_CF	blue	na
chrUn	26526843	26527718	876	SAT1_CF	blue	na
chrUn	26527745	26528524	780	SAT1_CF	blue	na
chrUn	26528546	26532207	3662	SAT1_CF	blue	na
chrUn	26532240	26534304	2065	SAT1_CF	blue	na
chrUn	26534316	26534999	684	SAT1_CF	blue	na
chrUn	26535012	26535513	502	SAT1_CF	blue	na
chrUn	26535571	26535703	133	SAT1_CF	blue	na
chrUn	26535731	26536240	510	SAT1_CF	blue	na
chrUn	26536283	26536844	562	SAT1_CF	blue	na
chrUn	26536872	26537502	631	SAT1_CF	blue	na

chrUn	26537545	26538098	554	SAT1_CF	blue	na
chrUn	26538119	26538438	320	SAT1_CF	blue	na
chrUn	26538450	26539136	687	SAT1_CF	blue	na
chrUn	26539147	26539649	503	SAT1_CF	blue	na
chrUn	26539685	26540058	374	SAT1_CF	blue	na
chrUn	26540101	26541118	1018	SAT1_CF	blue	na
chrUn	26541146	26542770	1625	SAT1_CF	blue	na
chrUn	26542813	26543490	678	SAT1_CF	blue	na
chrUn	26543502	26544056	555	SAT1_CF	blue	na
chrUn	26544113	26544185	73	SAT1_CF	blue	na
chrUn	26544198	26544898	701	SAT1_CF	blue	na
chrUn	26544926	26545436	511	SAT1_CF	blue	na
chrUn	26545504	26546749	1246	SAT1_CF	blue	na
chrUn	26546760	26547308	549	SAT1_CF	blue	na
chrUn	26547425	26549064	1640	SAT1_CF	blue	na
chrUn	26549076	26549992	917	SAT1_CF	blue	na
chrUn	26550005	26550687	683	SAT1_CF	blue	na
chrUn	26550700	26551219	520	SAT1_CF	blue	na
chrUn	26551236	26552134	899	SAT1_CF	blue	na
chrUn	26552166	26553020	855	SAT1_CF	blue	na
chrUn	26553062	26553792	731	SAT1_CF	blue	na
chrUn	26553808	26553921	114	SAT1_CF	blue	na
chrUn	26553933	26554475	543	SAT1_CF	blue	na
chrUn	26554592	26556260	1669	SAT1_CF	blue	na
chrUn	26556272	26556969	698	SAT1_CF	blue	na
chrUn	26556987	26557489	503	SAT1_CF	blue	na
chrUn	26557529	26558486	958	SAT1_CF	blue	na
chrUn	26558527	26558699	173	SAT1_CF	blue	na
chrUn	26558711	26559349	639	SAT1_CF	blue	na
chrUn	26559361	26560042	682	SAT1_CF	blue	na
chrUn	26560055	26560538	484	SAT1_CF	blue	na
chrUn	26560621	26560756	136	SAT1_CF	blue	na
chrUn	26560784	26561295	512	SAT1_CF	blue	na
chrUn	26561339	26561903	565	SAT1_CF	blue	na
chrUn	26561930	26562560	631	SAT1_CF	blue	na
chrUn	26562603	26563149	547	SAT1_CF	blue	na
chrUn	26563170	26563278	109	SAT1_CF	blue	na
chrUn	26563290	26564068	779	SAT1_CF	blue	na
chrUn	26564133	26564729	597	SAT1_CF	blue	na
chrUn	26564750	26566234	1485	SAT1_CF	blue	na
chrUn	26566263	26566787	525	SAT1_CF	blue	na
chrUn	26566830	26567507	678	SAT1_CF	blue	na

chrUn	26567519	26568228	710	SAT1_CF	blue	na
chrUn	26568285	26568357	73	SAT1_CF	blue	na
chrUn	26568369	26568871	503	SAT1_CF	blue	na
chrUn	26568924	26569832	909	SAT1_CF	blue	na
chrUn	26569913	26572207	2295	SAT1_CF	blue	na
chrUn	26572275	26573608	1334	SAT1_CF	blue	na
chrUn	26573652	26574985	1334	SAT1_CF	blue	na
chrUn	26574997	26576662	1666	SAT1_CF	blue	na
chrUn	26576676	26577359	684	SAT1_CF	blue	na
chrUn	26577372	26577872	501	SAT1_CF	blue	na
chrUn	26577918	26579454	1537	SAT1_CF	blue	na
chrUn	26579481	26580701	1221	SAT1_CF	blue	na
chrUn	26580715	26581967	1253	SAT1_CF	blue	na
chrUn	26581988	26582197	210	SAT1_CF	blue	na
chrUn	26583541	26583946	406	CarSat1	red	na
chrUn	26583978	26584702	725	CarSat1	red	na
chrUn	26584697	26585303	607	SAT1_CF	blue	na
chrUn	26585325	26585711	387	SAT1_CF	blue	na
chrUn	26585714	26586153	440	CarSat1	red	na
chrUn	26586171	26586811	641	CarSat1	red	na
chrUn	26586970	26587826	857	CarSat1	red	na
chrUn	26587847	26589419	1573	CarSat1	red	na
chrUn	26589449	26590153	705	CarSat1	red	na
chrUn	26611434	26612203	770	Sat2CF	purple	na
chrUn	26611499	26612188	690	Sat2CF	purple	na
chrUn	26624950	26626080	1131	Sat2CF	purple	na
chrUn	26635210	26635541	332	Sat2CF	purple	na
chrUn	26635251	26635766	516	Sat2CF	purple	na
chrUn	26635637	26635814	178	Sat2CF	purple	na
chrUn	26635637	26635814	178	Sat2CF	purple	na
chrUn	26650682	26651481	800	Sat2CF	purple	na
chrUn	26651362	26651531	170	Sat2CF	purple	na
chrUn	26664172	26665423	1252	Sat2CF	purple	na
chrUn	26665263	26665451	189	Sat2CF	purple	na
chrUn	26668051	26669638	1588	CarSat1	red	na
chrUn	26669665	26670073	409	CarSat1	red	na
chrUn	26679630	26680313	684	CarSat1	red	na
chrUn	26680331	26681849	1519	CarSat1	red	na
chrUn	26681868	26682270	403	CarSat1	red	na
chrUn	26682288	26682439	152	SAT1_CF	blue	na
chrUn	26682480	26683640	1161	SAT1_CF	blue	na
chrUn	26683835	26683995	161	Sat2CF	purple	na

chrUn	26683887	26684950	1064	Sat2CF	purple	na
chrUn	26690907	26691591	685	CarSat1	red	na
chrUn	26691609	26692446	838	CarSat1	red	na
chrUn	26692636	26693227	592	CarSat1	red	na
chrUn	26703303	26704016	714	CarSat1	red	na
chrUn	26704211	26704357	147	CarSat1	red	na
chrUn	26704382	26707518	3137	CarSat1	red	na
chrUn	26707548	26709727	2180	CarSat1	red	na
chrUn	26709757	26710979	1223	CarSat1	red	na
chrUn	26711005	26712634	1630	CarSat1	red	na
chrUn	26712825	26714551	1727	CarSat1	red	na
chrUn	26714584	26715047	464	CarSat1	red	na
chrUn	26715238	26718197	2960	CarSat1	red	na
chrUn	26718388	26724745	6358	CarSat1	red	na
chrUn	26724775	26725477	703	CarSat1	red	na
chrUn	26725507	26726000	494	CarSat1	red	na
chrUn	26726025	26726949	925	CarSat1	red	na
chrUn	26726979	26727469	491	CarSat1	red	na
chrUn	26727502	26727669	168	CarSat1	red	na
chrUn	26727862	26728626	765	CarSat1	red	na
chrUn	26728656	26729353	698	CarSat1	red	na
chrUn	26729391	26730093	703	CarSat1	red	na
chrUn	26730123	26730617	495	CarSat1	red	na
chrUn	26730650	26731269	620	CarSat1	red	na
chrUn	26738113	26739333	1221	Sat2CF	purple	na
chrUn	26784099	26785210	1112	Sat2CF	purple	na
chrUn	26785052	26785253	202	Sat2CF	purple	na
chrUn	26786882	26787293	412	CarSat1	red	na
chrUn	26788261	26789845	1585	CarSat1	red	na
chrUn	26789866	26790717	852	CarSat1	red	na
chrUn	26790738	26792896	2159	CarSat1	red	na
chrUn	26792891	26794434	1544	SAT1_CF	blue	na
chrUn	26794476	26796610	2135	SAT1_CF	blue	na
chrUn	26796623	26797590	968	SAT1_CF	blue	na
chrUn	26797636	26798152	517	SAT1_CF	blue	na
chrUn	26801502	26802037	536	SAT1_CF	blue	na
chrUn	26802124	26802618	495	SAT1_CF	blue	na
chrUn	26802647	26803316	670	SAT1_CF	blue	na
chrUn	26803340	26805408	2069	SAT1_CF	blue	na
chrUn	26805450	26807078	1629	SAT1_CF	blue	na
chrUn	26807117	26807648	532	SAT1_CF	blue	na
chrUn	26807660	26807766	107	SAT1_CF	blue	na

chrUn	26807790	26809250	1461	SAT1_CF	blue	na
chrUn	26809266	26809532	267	SAT1_CF	blue	na
chrUn	26809611	26810158	548	SAT1_CF	blue	na
chrUn	26810171	26810566	396	SAT1_CF	blue	na
chrUn	26810630	26811110	481	SAT1_CF	blue	na
chrUn	26811122	26812665	1544	SAT1_CF	blue	na
chrUn	26812707	26813251	545	SAT1_CF	blue	na
chrUn	26813289	26814515	1227	SAT1_CF	blue	na
chrUn	26814597	26815102	506	SAT1_CF	blue	na
chrUn	26815115	26816208	1094	SAT1_CF	blue	na
chrUn	26816219	26818296	2078	SAT1_CF	blue	na
chrUn	26818319	26820821	2503	SAT1_CF	blue	na
chrUn	26820868	26821382	515	SAT1_CF	blue	na
chrUn	26821402	26822079	678	SAT1_CF	blue	na
chrUn	26822122	26824069	1948	SAT1_CF	blue	na
chrUn	26824081	26824756	676	SAT1_CF	blue	na
chrUn	26824799	26826391	1593	SAT1_CF	blue	na
chrUn	26826439	26826838	400	SAT1_CF	blue	na
chrUn	26826878	26827377	500	SAT1_CF	blue	na
chrUn	26827391	26828665	1275	SAT1_CF	blue	na
chrUn	26828686	26830737	2052	SAT1_CF	blue	na
chrUn	26830781	26831301	521	SAT1_CF	blue	na
chrUn	26831313	26832910	1598	SAT1_CF	blue	na
chrUn	26832933	26833784	852	SAT1_CF	blue	na
chrUn	26833826	26835675	1850	SAT1_CF	blue	na
chrUn	26835696	26838182	2487	SAT1_CF	blue	na
chrUn	26838260	26838769	510	SAT1_CF	blue	na
chrUn	26838836	26838922	87	SAT1_CF	blue	na
chrUn	26838972	26840092	1121	SAT1_CF	blue	na
chrUn	26840104	26840213	110	SAT1_CF	blue	na
chrUn	26840233	26840779	547	SAT1_CF	blue	na
chrUn	26840822	26842632	1811	SAT1_CF	blue	na
chrUn	26842680	26843181	502	SAT1_CF	blue	na
chrUn	26843193	26843964	772	SAT1_CF	blue	na
chrUn	26843986	26844537	552	SAT1_CF	blue	na
chrUn	26844580	26845933	1354	SAT1_CF	blue	na
chrUn	26845945	26846050	106	SAT1_CF	blue	na
chrUn	26846074	26846565	492	SAT1_CF	blue	na
chrUn	26846632	26847087	456	SAT1_CF	blue	na
chrUn	26847100	26847717	618	SAT1_CF	blue	na
chrUn	26847742	26848296	555	SAT1_CF	blue	na
chrUn	26848308	26849634	1327	SAT1_CF	blue	na

chrUn	26849678	26850102	425	SAT1_CF	blue	na
chrUn	26850123	26850631	509	SAT1_CF	blue	na
chrUn	26850644	26851542	899	SAT1_CF	blue	na
chrUn	26851571	26852089	519	SAT1_CF	blue	na
chrUn	26852106	26855028	2923	SAT1_CF	blue	na
chrUn	26855057	26855576	520	SAT1_CF	blue	na
chrUn	26855594	26856265	672	SAT1_CF	blue	na
chrUn	26856308	26857419	1112	SAT1_CF	blue	na
chrUn	26857462	26858045	584	SAT1_CF	blue	na
chrUn	26858057	26859655	1599	SAT1_CF	blue	na
chrUn	26859678	26860527	850	SAT1_CF	blue	na
chrUn	26860569	26862001	1433	SAT1_CF	blue	na
chrUn	26862014	26862418	405	SAT1_CF	blue	na
chrUn	26862456	26863674	1219	SAT1_CF	blue	na
chrUn	26863706	26864209	504	SAT1_CF	blue	na
chrUn	26864242	26864946	705	SAT1_CF	blue	na
chrUn	26864959	26865060	102	SAT1_CF	blue	na
chrUn	26865088	26867162	2075	SAT1_CF	blue	na
chrUn	26867232	26867785	554	SAT1_CF	blue	na
chrUn	26867797	26868479	683	SAT1_CF	blue	na
chrUn	26868490	26868975	486	SAT1_CF	blue	na
chrUn	26868989	26871441	2453	SAT1_CF	blue	na
chrUn	26871455	26871772	318	SAT1_CF	blue	na
chrUn	26871825	26873016	1192	SAT1_CF	blue	na
chrUn	26873033	26873716	684	SAT1_CF	blue	na
chrUn	26873727	26875448	1722	SAT1_CF	blue	na
chrUn	26875459	26877019	1561	SAT1_CF	blue	na
chrUn	26877064	26877563	500	SAT1_CF	blue	na
chrUn	26877577	26878258	682	SAT1_CF	blue	na
chrUn	26878270	26878877	608	SAT1_CF	blue	na
chrUn	26878889	26879564	676	SAT1_CF	blue	na
chrUn	26879607	26881643	2037	SAT1_CF	blue	na
chrUn	26881690	26882191	502	SAT1_CF	blue	na
chrUn	26882202	26883286	1085	SAT1_CF	blue	na
chrUn	26883312	26884687	1376	SAT1_CF	blue	na
chrUn	26884731	26885273	543	SAT1_CF	blue	na
chrUn	26885300	26886619	1320	SAT1_CF	blue	na
chrUn	26886687	26887167	481	SAT1_CF	blue	na
chrUn	26887180	26887786	607	SAT1_CF	blue	na
chrUn	26887798	26888471	674	SAT1_CF	blue	na
chrUn	26888514	26889053	540	SAT1_CF	blue	na
chrUn	26889071	26890581	1511	SAT1_CF	blue	na

chrUn	26890594	26891095	502	SAT1_CF	blue	na
chrUn	26891143	26891811	669	SAT1_CF	blue	na
chrUn	26891890	26892845	956	SAT1_CF	blue	na
chrUn	26892872	26893383	512	SAT1_CF	blue	na
chrUn	26893403	26894008	606	SAT1_CF	blue	na
chrUn	26894019	26894713	695	SAT1_CF	blue	na
chrUn	26894726	26895615	890	SAT1_CF	blue	na
chrUn	26895628	26896988	1361	SAT1_CF	blue	na
chrUn	26897001	26898836	1836	SAT1_CF	blue	na
chrUn	26898870	26899391	522	SAT1_CF	blue	na
chrUn	26899404	26900010	607	SAT1_CF	blue	na
chrUn	26900022	26900131	110	SAT1_CF	blue	na
chrUn	26900151	26902521	2371	SAT1_CF	blue	na
chrUn	26902538	26903057	520	SAT1_CF	blue	na
chrUn	26903070	26903752	683	SAT1_CF	blue	na
chrUn	26903764	26907048	3285	SAT1_CF	blue	na
chrUn	26907088	26907587	500	SAT1_CF	blue	na
chrUn	26907600	26907716	117	SAT1_CF	blue	na
chrUn	26907730	26908206	477	SAT1_CF	blue	na
chrUn	26908218	26908320	103	SAT1_CF	blue	na
chrUn	26908349	26908895	547	SAT1_CF	blue	na
chrUn	26908938	26910085	1148	SAT1_CF	blue	na
chrUn	26910098	26910817	720	SAT1_CF	blue	na
chrUn	26910871	26912806	1936	SAT1_CF	blue	na
chrUn	26912885	26913370	486	SAT1_CF	blue	na
chrUn	26913404	26913804	401	SAT1_CF	blue	na
chrUn	26913825	26914344	520	SAT1_CF	blue	na
chrUn	26914357	26915039	683	SAT1_CF	blue	na
chrUn	26915063	26915713	651	SAT1_CF	blue	na
chrUn	26915731	26915772	42	SAT1_CF	blue	na
chrUn	26915796	26916308	513	SAT1_CF	blue	na
chrUn	26916325	26916568	244	SAT1_CF	blue	na
chrUn	26916646	26917201	556	SAT1_CF	blue	na
chrUn	26917235	26917998	764	SAT1_CF	blue	na
chrUn	26918042	26918828	787	SAT1_CF	blue	na
chrUn	26918872	26919333	462	SAT1_CF	blue	na
chrUn	26919373	26919873	501	SAT1_CF	blue	na
chrUn	26919886	26920569	684	SAT1_CF	blue	na
chrUn	26920581	26921004	424	SAT1_CF	blue	na
chrUn	26921027	26921548	522	SAT1_CF	blue	na
chrUn	26921561	26922166	606	SAT1_CF	blue	na
chrUn	26922178	26922287	110	SAT1_CF	blue	na

chrUn	26922307	26924906	2600	SAT1_CF	blue	na
chrUn	26924984	26925496	513	SAT1_CF	blue	na
chrUn	26925524	26925660	137	SAT1_CF	blue	na
chrUn	26925746	26926227	482	SAT1_CF	blue	na
chrUn	26926240	26926923	684	SAT1_CF	blue	na
chrUn	26926935	26927602	668	SAT1_CF	blue	na
chrUn	26927645	26928364	720	SAT1_CF	blue	na
chrUn	26928443	26929006	564	SAT1_CF	blue	na
chrUn	26929034	26929170	137	SAT1_CF	blue	na
chrUn	26929236	26929738	503	SAT1_CF	blue	na
chrUn	26929880	26930393	514	SAT1_CF	blue	na
chrUn	26930437	26932249	1813	SAT1_CF	blue	na
chrUn	26932261	26933582	1322	SAT1_CF	blue	na
chrUn	26933661	26935849	2189	SAT1_CF	blue	na
chrUn	26935897	26937306	1410	SAT1_CF	blue	na
chrUn	26937330	26938538	1209	SAT1_CF	blue	na
chrUn	26938618	26939127	510	SAT1_CF	blue	na
chrUn	26939194	26939280	87	SAT1_CF	blue	na
chrUn	26939330	26941163	1834	SAT1_CF	blue	na
chrUn	26941242	26941805	564	SAT1_CF	blue	na
chrUn	26941833	26941969	137	SAT1_CF	blue	na
chrUn	26942035	26942577	543	SAT1_CF	blue	na
chrUn	26942620	26943583	964	SAT1_CF	blue	na
chrUn	26943627	26943711	85	SAT1_CF	blue	na
chrUn	26943724	26944428	705	SAT1_CF	blue	na
chrUn	26944462	26944958	497	SAT1_CF	blue	na
chrUn	26945014	26946254	1241	SAT1_CF	blue	na
chrUn	26946302	26947262	961	SAT1_CF	blue	na
chrUn	26947317	26948833	1517	SAT1_CF	blue	na
chrUn	26948850	26949984	1135	SAT1_CF	blue	na
chrUn	26949996	26952308	2313	SAT1_CF	blue	na
chrUn	26952337	26952856	520	SAT1_CF	blue	na
chrUn	26952870	26953554	685	SAT1_CF	blue	na
chrUn	26953566	26953675	110	SAT1_CF	blue	na
chrUn	26953695	26954898	1204	SAT1_CF	blue	na
chrUn	26954942	26955943	1002	SAT1_CF	blue	na
chrUn	26955956	26956634	679	SAT1_CF	blue	na
chrUn	26956658	26958086	1429	SAT1_CF	blue	na
chrUn	26958102	26958345	244	SAT1_CF	blue	na
chrUn	26958389	26959621	1233	SAT1_CF	blue	na
chrUn	26959664	26960628	965	SAT1_CF	blue	na
chrUn	26960654	26961460	807	SAT1_CF	blue	na

chrUn	26961528	26962028	501	SAT1_CF	blue	na
chrUn	26962041	26962716	676	SAT1_CF	blue	na
chrUn	26962746	26963408	663	SAT1_CF	blue	na
chrUn	26963447	26964249	803	SAT1_CF	blue	na
chrUn	26964260	26965260	1001	SAT1_CF	blue	na
chrUn	26965297	26965671	375	SAT1_CF	blue	na
chrUn	26965711	26966205	495	SAT1_CF	blue	na
chrUn	26966230	26966906	677	SAT1_CF	blue	na
chrUn	26966918	26967594	677	SAT1_CF	blue	na
chrUn	26967606	26968710	1105	SAT1_CF	blue	na
chrUn	26968742	26969627	886	SAT1_CF	blue	na
chrUn	26969714	26970212	499	SAT1_CF	blue	na
chrUn	26970225	26971163	939	SAT1_CF	blue	na
chrUn	26971234	26971747	514	SAT1_CF	blue	na
chrUn	26971761	26973122	1362	SAT1_CF	blue	na
chrUn	26973165	26974120	956	SAT1_CF	blue	na
chrUn	26974236	26975298	1063	SAT1_CF	blue	na
chrUn	26975328	26975618	291	SAT1_CF	blue	na
chrUn	26975639	26976159	521	SAT1_CF	blue	na
chrUn	26976173	26976280	108	SAT1_CF	blue	na
chrUn	26976302	26976855	554	SAT1_CF	blue	na
chrUn	26976867	26976976	110	SAT1_CF	blue	na
chrUn	26976997	26977540	544	SAT1_CF	blue	na
chrUn	26977553	26978272	720	SAT1_CF	blue	na
chrUn	26978294	26980414	2121	SAT1_CF	blue	na
chrUn	26980457	26980969	513	SAT1_CF	blue	na
chrUn	26980997	26981132	136	SAT1_CF	blue	na
chrUn	26981198	26983470	2273	SAT1_CF	blue	na
chrUn	26983514	26984061	548	SAT1_CF	blue	na
chrUn	26984104	26984246	143	SAT1_CF	blue	na
chrUn	26984314	26984795	482	SAT1_CF	blue	na
chrUn	26984814	26984914	101	SAT1_CF	blue	na
chrUn	26984938	26985493	556	SAT1_CF	blue	na
chrUn	26985505	26986838	1334	SAT1_CF	blue	na
chrUn	26986908	26988026	1119	SAT1_CF	blue	na
chrUn	26988066	26988586	521	SAT1_CF	blue	na
chrUn	26988598	26989295	698	SAT1_CF	blue	na
chrUn	26989307	26990431	1125	SAT1_CF	blue	na
chrUn	26990456	26990780	325	SAT1_CF	blue	na
chrUn	26990859	26991400	542	SAT1_CF	blue	na
chrUn	26991451	26992150	700	SAT1_CF	blue	na
chrUn	26992162	26993196	1035	SAT1_CF	blue	na

chrUn	26993258	26993800	543	SAT1_CF	blue	na
chrUn	26995205	26995722	518	SAT1_CF	blue	na
chrUn	26995790	26996310	521	SAT1_CF	blue	na
chrUn	26996322	26997009	688	SAT1_CF	blue	na
chrUn	26997021	26998145	1125	SAT1_CF	blue	na
chrUn	26998170	26998494	325	SAT1_CF	blue	na
chrUn	26998573	26999114	542	SAT1_CF	blue	na
chrUn	26999165	26999864	700	SAT1_CF	blue	na
chrUn	26999876	27000708	833	SAT1_CF	blue	na
chrUn	27000736	27001298	563	SAT1_CF	blue	na
chrUn	27002391	27002694	304	SAT1_CF	blue	na
chrUn	27002964	27004737	1774	SAT1_CF	blue	na
chrUn	27004779	27005325	547	SAT1_CF	blue	na
chrUn	27005382	27005901	520	SAT1_CF	blue	na
chrUn	27005996	27006132	137	SAT1_CF	blue	na
chrUn	27006147	27006669	523	SAT1_CF	blue	na
chrUn	27006697	27007202	506	SAT1_CF	blue	na
chrUn	27007244	27007860	617	SAT1_CF	blue	na
chrUn	27007873	27008723	851	SAT1_CF	blue	na
chrUn	27008735	27009421	687	SAT1_CF	blue	na
chrUn	27009439	27009982	544	SAT1_CF	blue	na
chrUn	27010026	27010294	269	SAT1_CF	blue	na
chrUn	27010322	27010950	629	SAT1_CF	blue	na
chrUn	27010979	27011593	615	SAT1_CF	blue	na
chrUn	27011680	27012030	351	SAT1_CF	blue	na
chrUn	27012049	27013009	961	SAT1_CF	blue	na
chrUn	27013054	27014386	1333	SAT1_CF	blue	na
chrUn	27014398	27015044	647	SAT1_CF	blue	na
chrUn	27015057	27015819	763	SAT1_CF	blue	na
chrUn	27015831	27016327	497	SAT1_CF	blue	na
chrUn	27016350	27016456	107	SAT1_CF	blue	na
chrUn	27016469	27017013	545	SAT1_CF	blue	na
chrUn	27017090	27018148	1059	SAT1_CF	blue	na
chrUn	27018203	27018348	146	SAT1_CF	blue	na
chrUn	27018373	27019018	646	SAT1_CF	blue	na
chrUn	27019031	27021248	2218	SAT1_CF	blue	na
chrUn	27021260	27021815	556	SAT1_CF	blue	na
chrUn	27021836	27021935	100	SAT1_CF	blue	na
chrUn	27021957	27022534	578	SAT1_CF	blue	na
chrUn	27022554	27022856	303	SAT1_CF	blue	na
chrUn	27022869	27024022	1154	SAT1_CF	blue	na
chrUn	27024035	27024737	703	SAT1_CF	blue	na

chrUn	27024769	27025273	505	SAT1_CF	blue	na
chrUn	27025305	27025964	660	SAT1_CF	blue	na
chrUn	27025976	27026478	503	SAT1_CF	blue	na
chrUn	27026491	27027819	1329	SAT1_CF	blue	na
chrUn	27027841	27028491	651	SAT1_CF	blue	na
chrUn	27028505	27029025	521	SAT1_CF	blue	na
chrUn	27029091	27029397	307	SAT1_CF	blue	na
chrUn	27029440	27030765	1326	SAT1_CF	blue	na
chrUn	27030783	27031872	1090	SAT1_CF	blue	na
chrUn	27031912	27032314	403	SAT1_CF	blue	na
chrUn	27032326	27032939	614	SAT1_CF	blue	na
chrUn	27032950	27033568	619	SAT1_CF	blue	na
chrUn	27033589	27033692	104	SAT1_CF	blue	na
chrUn	27033710	27034210	501	SAT1_CF	blue	na
chrUn	27034297	27035245	949	SAT1_CF	blue	na
chrUn	27035266	27035374	109	SAT1_CF	blue	na
chrUn	27035387	27035860	474	SAT1_CF	blue	na
chrUn	27035920	27036101	182	SAT1_CF	blue	na
chrUn	27036283	27037500	1218	SAT1_CF	blue	na
chrUn	27037528	27038785	1258	SAT1_CF	blue	na
chrUn	27038806	27039447	642	SAT1_CF	blue	na
chrUn	27039473	27039629	157	SAT1_CF	blue	na
chrUn	27039644	27040275	632	SAT1_CF	blue	na
chrUn	27040286	27040968	683	SAT1_CF	blue	na
chrUn	27040991	27041682	692	SAT1_CF	blue	na
chrUn	27041906	27042418	513	SAT1_CF	blue	na
chrUn	27042437	27043219	783	SAT1_CF	blue	na
chrUn	27043232	27043733	502	SAT1_CF	blue	na
chrUn	27043786	27044174	389	SAT1_CF	blue	na
chrUn	27044211	27044972	762	SAT1_CF	blue	na
chrUn	27044999	27045152	154	SAT1_CF	blue	na
chrUn	27045196	27046530	1335	SAT1_CF	blue	na
chrUn	27046543	27047045	503	SAT1_CF	blue	na
chrUn	27047131	27049194	2064	SAT1_CF	blue	na
chrUn	27049219	27049283	65	SAT1_CF	blue	na
chrUn	27049301	27050228	928	SAT1_CF	blue	na
chrUn	27050240	27050923	684	SAT1_CF	blue	na
chrUn	27050935	27051565	631	SAT1_CF	blue	na
chrUn	27051634	27052191	558	SAT1_CF	blue	na
chrUn	27052253	27052812	560	SAT1_CF	blue	na
chrUn	27052872	27053053	182	SAT1_CF	blue	na
chrUn	27053234	27053436	203	SAT1_CF	blue	na

chrUn	27053479	27055266	1788	SAT1_CF	blue	na
chrUn	27055278	27056855	1578	SAT1_CF	blue	na
chrUn	27056935	27057972	1038	SAT1_CF	blue	na
chrUn	27058014	27059153	1140	SAT1_CF	blue	na
chrUn	27059166	27060285	1120	SAT1_CF	blue	na
chrUn	27060297	27061000	704	SAT1_CF	blue	na
chrUn	27061013	27061534	522	SAT1_CF	blue	na
chrUn	27061551	27061924	374	SAT1_CF	blue	na
chrUn	27061967	27063121	1155	SAT1_CF	blue	na
chrUn	27063164	27063716	553	SAT1_CF	blue	na
chrUn	27063728	27063796	69	SAT1_CF	blue	na
chrUn	27063823	27064344	522	SAT1_CF	blue	na
chrUn	27064614	27066738	2125	SAT1_CF	blue	na
chrUn	27066750	27067432	683	SAT1_CF	blue	na
chrUn	27067445	27069197	1753	SAT1_CF	blue	na
chrUn	27069215	27069737	523	SAT1_CF	blue	na
chrUn	27069789	27070568	780	SAT1_CF	blue	na
chrUn	27070582	27071278	697	SAT1_CF	blue	na
chrUn	27071322	27072312	991	SAT1_CF	blue	na
chrUn	27072393	27072955	563	SAT1_CF	blue	na
chrUn	27072999	27074332	1334	SAT1_CF	blue	na
chrUn	27074344	27074864	521	SAT1_CF	blue	na
chrUn	27074889	27075047	159	SAT1_CF	blue	na
chrUn	27075074	27077225	2152	SAT1_CF	blue	na
chrUn	27077252	27077772	521	SAT1_CF	blue	na
chrUn	27077797	27077953	157	SAT1_CF	blue	na
chrUn	27077968	27078489	522	SAT1_CF	blue	na
chrUn	27078522	27079476	955	SAT1_CF	blue	na
chrUn	27079496	27079991	496	SAT1_CF	blue	na
chrUn	27080077	27080251	175	SAT1_CF	blue	na
chrUn	27080332	27081577	1246	SAT1_CF	blue	na
chrUn	27081589	27082141	553	SAT1_CF	blue	na
chrUn	27082162	27083178	1017	SAT1_CF	blue	na
chrUn	27083190	27083821	632	SAT1_CF	blue	na
chrUn	27083844	27083949	106	SAT1_CF	blue	na
chrUn	27083962	27085581	1620	SAT1_CF	blue	na
chrUn	27085606	27086410	805	SAT1_CF	blue	na
chrUn	27086437	27087067	631	SAT1_CF	blue	na
chrUn	27087110	27087786	677	SAT1_CF	blue	na
chrUn	27087800	27089252	1453	SAT1_CF	blue	na
chrUn	27089265	27089785	521	SAT1_CF	blue	na
chrUn	27089853	27090206	354	SAT1_CF	blue	na

chrUn	27090218	27090765	548	SAT1_CF	blue	na
chrUn	27090844	27091625	782	SAT1_CF	blue	na
chrUn	27091667	27092528	862	SAT1_CF	blue	na
chrUn	27092540	27093058	519	SAT1_CF	blue	na
chrUn	27093077	27093834	758	SAT1_CF	blue	na
chrUn	27093861	27094361	501	SAT1_CF	blue	na
chrUn	27094407	27094563	157	SAT1_CF	blue	na
chrUn	27094591	27095092	502	SAT1_CF	blue	na
chrUn	27095137	27095226	90	SAT1_CF	blue	na
chrUn	27101237	27101719	483	SAT1_CF	blue	na
chrUn	27101763	27102031	269	SAT1_CF	blue	na
chrUn	27102059	27102409	351	SAT1_CF	blue	na
chrUn	27205678	27206202	525	SAT1_CF	blue	na
chrUn	27206252	27206750	499	SAT1_CF	blue	na
chrUn	27206763	27207159	397	SAT1_CF	blue	na
chrUn	27207205	27207726	522	SAT1_CF	blue	na
chrUn	27207793	27208752	960	SAT1_CF	blue	na
chrUn	27209053	27209364	312	SAT1_CF	blue	na
chrUn	27209386	27209456	71	SAT1_CF	blue	na
chrUn	27209469	27210153	685	SAT1_CF	blue	na
chrUn	27210166	27211017	852	SAT1_CF	blue	na
chrUn	27211039	27211924	886	SAT1_CF	blue	na
chrUn	27211936	27212619	684	SAT1_CF	blue	na
chrUn	27212630	27213294	665	SAT1_CF	blue	na
chrUn	27213321	27215166	1846	SAT1_CF	blue	na
chrUn	27215209	27215754	546	SAT1_CF	blue	na
chrUn	27215770	27215883	114	SAT1_CF	blue	na
chrUn	27215895	27216415	521	SAT1_CF	blue	na
chrUn	27216449	27217885	1437	SAT1_CF	blue	na
chrUn	27217930	27218603	674	SAT1_CF	blue	na
chrUn	27218630	27219218	589	SAT1_CF	blue	na
chrUn	27219245	27219848	604	SAT1_CF	blue	na
chrUn	27219860	27220953	1094	SAT1_CF	blue	na
chrUn	27220995	27221670	676	SAT1_CF	blue	na
chrUn	27221682	27222829	1148	SAT1_CF	blue	na
chrUn	27222907	27224300	1394	SAT1_CF	blue	na
chrUn	27224321	27225244	924	SAT1_CF	blue	na
chrUn	27225287	27226104	818	SAT1_CF	blue	na
chrUn	27226146	27227065	920	SAT1_CF	blue	na
chrUn	27227078	27227577	500	SAT1_CF	blue	na
chrUn	27227666	27228626	961	SAT1_CF	blue	na
chrUn	27228693	27229658	966	SAT1_CF	blue	na

chrUn	27229701	27230378	678	SAT1_CF	blue	na
chrUn	27230390	27230891	502	SAT1_CF	blue	na
chrUn	27230936	27231093	158	SAT1_CF	blue	na
chrUn	27231144	27231630	487	SAT1_CF	blue	na
chrUn	27231661	27232191	531	SAT1_CF	blue	na
chrUn	27232212	27232319	108	SAT1_CF	blue	na
chrUn	27232332	27232831	500	SAT1_CF	blue	na
chrUn	27232918	27235626	2709	SAT1_CF	blue	na
chrUn	27235638	27236322	685	SAT1_CF	blue	na
chrUn	27236334	27237609	1276	SAT1_CF	blue	na
chrUn	27237653	27237809	157	SAT1_CF	blue	na
chrUn	27237837	27239062	1226	SAT1_CF	blue	na
chrUn	27239086	27240398	1313	SAT1_CF	blue	na
chrUn	27240410	27240885	476	SAT1_CF	blue	na
chrUn	27240955	27241628	674	SAT1_CF	blue	na
chrUn	27241640	27242193	554	SAT1_CF	blue	na
chrUn	27242236	27243386	1151	SAT1_CF	blue	na
chrUn	27243399	27244236	838	SAT1_CF	blue	na
chrUn	27244256	27244796	541	SAT1_CF	blue	na
chrUn	27244840	27245179	340	SAT1_CF	blue	na
chrUn	27245223	27246555	1333	SAT1_CF	blue	na
chrUn	27246569	27247198	630	SAT1_CF	blue	na
chrUn	27247219	27247327	109	SAT1_CF	blue	na
chrUn	27247339	27248022	684	SAT1_CF	blue	na
chrUn	27248035	27248554	520	SAT1_CF	blue	na
chrUn	27248622	27249523	902	SAT1_CF	blue	na
chrUn	27249613	27250576	964	SAT1_CF	blue	na
chrUn	27250619	27251287	669	SAT1_CF	blue	na
chrUn	27251305	27251855	551	SAT1_CF	blue	na
chrUn	27251878	27251949	72	SAT1_CF	blue	na
chrUn	27251962	27252723	762	SAT1_CF	blue	na
chrUn	27252736	27253237	502	SAT1_CF	blue	na
chrUn	27253290	27254437	1148	SAT1_CF	blue	na
chrUn	27254518	27255816	1299	SAT1_CF	blue	na
chrUn	27255828	27256402	575	SAT1_CF	blue	na
chrUn	27256455	27256531	77	SAT1_CF	blue	na
chrUn	27256544	27257379	836	SAT1_CF	blue	na
chrUn	27257392	27258709	1318	SAT1_CF	blue	na
chrUn	27258721	27259239	519	SAT1_CF	blue	na
chrUn	27259264	27259708	445	SAT1_CF	blue	na
chrUn	27259774	27260441	668	SAT1_CF	blue	na
chrUn	27260483	27260830	348	SAT1_CF	blue	na

chrUn	27260868	27261412	545	SAT1_CF	blue	na
chrUn	27261462	27262001	540	SAT1_CF	blue	na
chrUn	27262044	27263501	1458	SAT1_CF	blue	na
chrUn	27263514	27264016	503	SAT1_CF	blue	na
chrUn	27264056	27265015	960	SAT1_CF	blue	na
chrUn	27265094	27266056	963	SAT1_CF	blue	na
chrUn	27266099	27267326	1228	SAT1_CF	blue	na
chrUn	27267340	27267832	493	SAT1_CF	blue	na
chrUn	27267843	27269579	1737	SAT1_CF	blue	na
chrUn	27269592	27270226	635	SAT1_CF	blue	na
chrUn	27270248	27270750	503	SAT1_CF	blue	na
chrUn	27270803	27271812	1010	SAT1_CF	blue	na
chrUn	27271867	27272831	965	SAT1_CF	blue	na
chrUn	27272874	27273538	665	SAT1_CF	blue	na
chrUn	27273561	27274244	684	SAT1_CF	blue	na
chrUn	27274264	27274758	495	SAT1_CF	blue	na
chrUn	27274798	27275371	574	SAT1_CF	blue	na
chrUn	27276770	27277365	596	SAT1_CF	blue	na
chrUn	27277414	27278059	646	SAT1_CF	blue	na
chrUn	27278101	27278412	312	SAT1_CF	blue	na
chrUn	27278454	27278974	521	SAT1_CF	blue	na
chrUn	27279008	27279373	366	SAT1_CF	blue	na
chrUn	27279405	27281489	2085	SAT1_CF	blue	na
chrUn	27281567	27282958	1392	SAT1_CF	blue	na
chrUn	27282987	27286080	3094	SAT1_CF	blue	na
chrUn	27286127	27286912	786	SAT1_CF	blue	na
chrUn	27286937	27287770	834	SAT1_CF	blue	na
chrUn	27287791	27287899	109	SAT1_CF	blue	na
chrUn	27287912	27288413	502	SAT1_CF	blue	na
chrUn	27288453	27288854	402	SAT1_CF	blue	na
chrUn	27288868	27289386	519	SAT1_CF	blue	na
chrUn	27289407	27289595	189	SAT1_CF	blue	na
chrUn	27289729	27291043	1315	SAT1_CF	blue	na
chrUn	27291064	27291172	109	SAT1_CF	blue	na
chrUn	27291185	27291665	481	SAT1_CF	blue	na
chrUn	27291721	27292093	373	SAT1_CF	blue	na
chrUn	27292136	27292684	549	SAT1_CF	blue	na
chrUn	27292765	27293924	1160	SAT1_CF	blue	na
chrUn	27293936	27294568	633	SAT1_CF	blue	na
chrUn	27294639	27295179	541	SAT1_CF	blue	na
chrUn	27295198	27295510	313	SAT1_CF	blue	na
chrUn	27295590	27296154	565	SAT1_CF	blue	na

chrUn	27296181	27296809	629	SAT1_CF	blue	na
chrUn	27296852	27297401	550	SAT1_CF	blue	na
chrUn	27297445	27298686	1242	SAT1_CF	blue	na
chrUn	27298765	27300622	1858	SAT1_CF	blue	na
chrUn	27300643	27301603	961	SAT1_CF	blue	na
chrUn	27301682	27303539	1858	SAT1_CF	blue	na
chrUn	27303560	27304678	1119	SAT1_CF	blue	na
chrUn	27306023	27306216	194	SAT1_CF	blue	na
chrUn	27306243	27306855	613	SAT1_CF	blue	na
chrUn	27306869	27307610	742	SAT1_CF	blue	na
chrUn	27307638	27308138	501	SAT1_CF	blue	na
chrUn	27310002	27311077	1076	SAT1_CF	blue	na
chrUn	27311104	27312337	1234	SAT1_CF	blue	na
chrUn	27312363	27313037	675	SAT1_CF	blue	na
chrUn	27314624	27315347	724	CarSat1	red	na
chrUn	27315340	27316609	1270	SAT1_CF	blue	na
chrUn	27316674	27317914	1241	CarSat1	red	na
chrUn	27317935	27318725	791	CarSat1	red	na
chrUn	27318749	27319531	783	CarSat1	red	na
chrUn	27319552	27321087	1536	CarSat1	red	na
chrUn	27321106	27321821	716	CarSat1	red	na
chrUn	27321844	27322620	777	CarSat1	red	na
chrUn	27322641	27323970	1330	CarSat1	red	na
chrUn	27323995	27324172	178	CarSat1	red	na
chrUn	27324331	27325037	707	CarSat1	red	na
chrUn	27352146	27353396	1251	Sat2CF	purple	na
chrUn	27352277	27353394	1118	Sat2CF	purple	na
chrUn	27370605	27371730	1126	Sat2CF	purple	na
chrUn	27386521	27386898	378	Sat2CF	purple	na
chrUn	27386576	27387470	895	Sat2CF	purple	na
chrUn	27386652	27387390	739	Sat2CF	purple	na
chrUn	27401285	27402027	743	Sat2CF	purple	na
chrUn	27401361	27402027	667	Sat2CF	purple	na
chrUn	27441624	27442843	1220	Sat2CF	purple	na
chrUn	27441752	27442838	1087	Sat2CF	purple	na
chrUn	27442716	27442863	148	Sat2CF	purple	na
chrUn	27469486	27470368	883	Sat2CF	purple	na
chrUn	27469701	27470450	750	Sat2CF	purple	na
chrUn	27498520	27498912	393	Sat2CF	purple	na
chrUn	27498594	27499304	711	Sat2CF	purple	na
chrUn	27515977	27516736	760	SAT1_CF	blue	na
chrUn	27516749	27517354	606	SAT1_CF	blue	na

chrUn	27517366	27520758	3393	SAT1_CF	blue	na
chrUn	27520770	27521453	684	SAT1_CF	blue	na
chrUn	27521476	27522030	555	SAT1_CF	blue	na
chrUn	27522042	27522950	909	SAT1_CF	blue	na
chrUn	27522971	27524539	1569	SAT1_CF	blue	na
chrUn	27524584	27525084	501	SAT1_CF	blue	na
chrUn	27525097	27525702	606	SAT1_CF	blue	na
chrUn	27525714	27526388	675	SAT1_CF	blue	na
chrUn	27526431	27528085	1655	SAT1_CF	blue	na
chrUn	27528099	27528501	403	SAT1_CF	blue	na
chrUn	27528541	27529041	501	SAT1_CF	blue	na
chrUn	27529054	27531115	2062	SAT1_CF	blue	na
chrUn	27531127	27531236	110	SAT1_CF	blue	na
chrUn	27531256	27531801	546	SAT1_CF	blue	na
chrUn	27531813	27532458	646	SAT1_CF	blue	na
chrUn	27532502	27532990	489	SAT1_CF	blue	na
chrUn	27533003	27534200	1198	SAT1_CF	blue	na
chrUn	27534212	27534884	673	SAT1_CF	blue	na
chrUn	27534911	27535630	720	SAT1_CF	blue	na
chrUn	27535642	27536188	547	SAT1_CF	blue	na
chrUn	27536258	27537198	941	SAT1_CF	blue	na
chrUn	27537210	27538138	929	SAT1_CF	blue	na
chrUn	27538181	27538870	690	SAT1_CF	blue	na
chrUn	27538904	27540122	1219	SAT1_CF	blue	na
chrUn	27540134	27540809	676	SAT1_CF	blue	na
chrUn	27540852	27543214	2363	SAT1_CF	blue	na
chrUn	27543226	27543334	109	SAT1_CF	blue	na
chrUn	27543357	27543912	556	SAT1_CF	blue	na
chrUn	27543924	27544033	110	SAT1_CF	blue	na
chrUn	27544053	27544598	546	SAT1_CF	blue	na
chrUn	27544641	27545272	632	SAT1_CF	blue	na
chrUn	27545320	27546566	1247	SAT1_CF	blue	na
chrUn	27546606	27547401	796	SAT1_CF	blue	na
chrUn	27547413	27548271	859	SAT1_CF	blue	na
chrUn	27548313	27549343	1031	SAT1_CF	blue	na
chrUn	27549424	27549947	524	SAT1_CF	blue	na
chrUn	27549978	27550157	180	SAT1_CF	blue	na
chrUn	27550201	27550695	495	SAT1_CF	blue	na
chrUn	27550713	27551381	669	SAT1_CF	blue	na
chrUn	27551402	27551862	461	SAT1_CF	blue	na
chrUn	27551904	27552444	541	SAT1_CF	blue	na
chrUn	27552465	27553476	1012	SAT1_CF	blue	na

chrUn	27553488	27554164	677	SAT1_CF	blue	na
chrUn	27554180	27554975	796	SAT1_CF	blue	na
chrUn	27554987	27555845	859	SAT1_CF	blue	na
chrUn	27555915	27557559	1645	SAT1_CF	blue	na
chrUn	27557574	27557769	196	SAT1_CF	blue	na
chrUn	27557813	27558307	495	SAT1_CF	blue	na
chrUn	27558325	27558994	670	SAT1_CF	blue	na
chrUn	27559015	27559475	461	SAT1_CF	blue	na
chrUn	27559517	27560057	541	SAT1_CF	blue	na
chrUn	27560078	27561089	1012	SAT1_CF	blue	na
chrUn	27561101	27561777	677	SAT1_CF	blue	na
chrUn	27561793	27562588	796	SAT1_CF	blue	na
chrUn	27562601	27563214	614	SAT1_CF	blue	na
chrUn	27563226	27564084	859	SAT1_CF	blue	na
chrUn	27564161	27564927	767	SAT1_CF	blue	na
chrUn	27564952	27565494	543	SAT1_CF	blue	na
chrUn	27565516	27566188	673	SAT1_CF	blue	na
chrUn	27566200	27566875	676	SAT1_CF	blue	na
chrUn	27566918	27567883	966	SAT1_CF	blue	na
chrUn	27567962	27568915	954	SAT1_CF	blue	na
chrUn	27568955	27569456	502	SAT1_CF	blue	na
chrUn	27569483	27570231	749	SAT1_CF	blue	na
chrUn	27570243	27571447	1205	SAT1_CF	blue	na
chrUn	27571489	27572277	789	SAT1_CF	blue	na
chrUn	27572321	27573030	710	SAT1_CF	blue	na
chrUn	27573060	27573843	784	SAT1_CF	blue	na
chrUn	27573885	27574585	701	SAT1_CF	blue	na
chrUn	27574599	27576175	1577	SAT1_CF	blue	na
chrUn	27576188	27576264	77	SAT1_CF	blue	na
chrUn	27576317	27576812	496	SAT1_CF	blue	na
chrUn	27576824	27577673	850	SAT1_CF	blue	na
chrUn	27577686	27577728	43	SAT1_CF	blue	na
chrUn	27577758	27578302	545	SAT1_CF	blue	na
chrUn	27578383	27578692	310	SAT1_CF	blue	na
chrUn	27578725	27581040	2316	SAT1_CF	blue	na
chrUn	27581053	27581812	760	SAT1_CF	blue	na
chrUn	27581824	27582505	682	SAT1_CF	blue	na
chrUn	27582517	27583191	675	SAT1_CF	blue	na
chrUn	27583238	27584193	956	SAT1_CF	blue	na
chrUn	27584237	27584780	544	SAT1_CF	blue	na
chrUn	27584827	27584936	110	SAT1_CF	blue	na
chrUn	27585125	27585406	282	SAT1_CF	blue	na

chrUn	27585465	27585945	481	SAT1_CF	blue	na
chrUn	27585958	27587092	1135	SAT1_CF	blue	na
chrUn	27587125	27588008	884	SAT1_CF	blue	na
chrUn	27588097	27588590	494	SAT1_CF	blue	na
chrUn	27588610	27589293	684	SAT1_CF	blue	na
chrUn	27589305	27589982	678	SAT1_CF	blue	na
chrUn	27590051	27590596	546	SAT1_CF	blue	na
chrUn	27590639	27591179	541	SAT1_CF	blue	na
chrUn	27591194	27592266	1073	SAT1_CF	blue	na
chrUn	27592308	27592510	203	SAT1_CF	blue	na
chrUn	27592690	27592878	189	SAT1_CF	blue	na
chrUn	27592934	27593414	481	SAT1_CF	blue	na
chrUn	27593427	27593532	106	SAT1_CF	blue	na
chrUn	27593555	27594907	1353	SAT1_CF	blue	na
chrUn	27594922	27595079	158	SAT1_CF	blue	na
chrUn	27595105	27596316	1212	SAT1_CF	blue	na
chrUn	27596340	27596913	574	SAT1_CF	blue	na
chrUn	27596954	27597455	502	SAT1_CF	blue	na
chrUn	27597469	27598152	684	SAT1_CF	blue	na
chrUn	27598164	27598999	836	SAT1_CF	blue	na
chrUn	27599180	27599365	186	SAT1_CF	blue	na
chrUn	27599401	27599903	503	SAT1_CF	blue	na
chrUn	27599925	27600599	675	SAT1_CF	blue	na
chrUn	27600611	27601288	678	SAT1_CF	blue	na
chrUn	27601331	27601874	544	SAT1_CF	blue	na
chrUn	27601917	27602465	549	SAT1_CF	blue	na
chrUn	27602477	27605397	2921	SAT1_CF	blue	na
chrUn	27605442	27606439	998	SAT1_CF	blue	na
chrUn	27606484	27607392	909	SAT1_CF	blue	na
chrUn	27607426	27607946	521	SAT1_CF	blue	na
chrUn	27608028	27608351	324	SAT1_CF	blue	na
chrUn	27608393	27609683	1291	SAT1_CF	blue	na
chrUn	27609773	27610272	500	SAT1_CF	blue	na
chrUn	27610286	27611010	725	SAT1_CF	blue	na
chrUn	27611022	27611702	681	SAT1_CF	blue	na
chrUn	27611714	27613009	1296	SAT1_CF	blue	na
chrUn	27613021	27614245	1225	SAT1_CF	blue	na
chrUn	27614258	27614886	629	SAT1_CF	blue	na
chrUn	27614897	27615365	469	SAT1_CF	blue	na
chrUn	27615385	27616836	1452	SAT1_CF	blue	na
chrUn	27616905	27617635	731	SAT1_CF	blue	na
chrUn	27617677	27618563	887	SAT1_CF	blue	na

chrUn	27618606	27619114	509	SAT1_CF	blue	na
chrUn	27619147	27619527	381	SAT1_CF	blue	na
chrUn	27619565	27620064	500	SAT1_CF	blue	na
chrUn	27620082	27620185	104	SAT1_CF	blue	na
chrUn	27620206	27620780	575	SAT1_CF	blue	na
chrUn	27620792	27622040	1249	SAT1_CF	blue	na
chrUn	27622062	27622728	667	SAT1_CF	blue	na
chrUn	27622772	27623333	562	SAT1_CF	blue	na
chrUn	27623376	27623900	525	SAT1_CF	blue	na
chrUn	27623915	27624051	137	SAT1_CF	blue	na
chrUn	27624117	27624619	503	SAT1_CF	blue	na
chrUn	27624632	27624739	108	SAT1_CF	blue	na
chrUn	27624761	27625314	554	SAT1_CF	blue	na
chrUn	27625326	27626088	763	SAT1_CF	blue	na
chrUn	27626100	27627432	1333	SAT1_CF	blue	na
chrUn	27627476	27628807	1332	SAT1_CF	blue	na
chrUn	27628894	27629396	503	SAT1_CF	blue	na
chrUn	27629408	27629514	107	SAT1_CF	blue	na
chrUn	27629538	27630166	629	SAT1_CF	blue	na
chrUn	27630190	27630637	448	SAT1_CF	blue	na
chrUn	27630654	27631363	710	SAT1_CF	blue	na
chrUn	27631378	27631514	137	SAT1_CF	blue	na
chrUn	27631610	27632129	520	SAT1_CF	blue	na
chrUn	27632186	27632731	546	SAT1_CF	blue	na
chrUn	27632774	27634594	1821	SAT1_CF	blue	na
chrUn	27634636	27635134	499	SAT1_CF	blue	na
chrUn	27635151	27635830	680	SAT1_CF	blue	na
chrUn	27635843	27635923	81	SAT1_CF	blue	na
chrUn	27635938	27636457	520	SAT1_CF	blue	na
chrUn	27636469	27638152	1684	SAT1_CF	blue	na
chrUn	27638196	27638738	543	SAT1_CF	blue	na
chrUn	27638760	27639434	675	SAT1_CF	blue	na
chrUn	27639446	27640352	907	SAT1_CF	blue	na
chrUn	27640383	27641289	907	SAT1_CF	blue	na
chrUn	27641307	27641829	523	SAT1_CF	blue	na
chrUn	27641857	27642238	382	SAT1_CF	blue	na
chrUn	27642259	27642749	491	SAT1_CF	blue	na
chrUn	27642761	27642870	110	SAT1_CF	blue	na
chrUn	27642891	27643428	538	SAT1_CF	blue	na
chrUn	27643485	27644028	544	SAT1_CF	blue	na
chrUn	27644071	27645141	1071	SAT1_CF	blue	na
chrUn	27645175	27646109	935	SAT1_CF	blue	na

chrUn	27646147	27646649	503	SAT1_CF	blue	na
chrUn	27646662	27647326	665	SAT1_CF	blue	na
chrUn	27647383	27647926	544	SAT1_CF	blue	na
chrUn	27647969	27648506	538	SAT1_CF	blue	na
chrUn	27648539	27649207	669	SAT1_CF	blue	na
chrUn	27649220	27650782	1563	SAT1_CF	blue	na
chrUn	27650800	27651562	763	SAT1_CF	blue	na
chrUn	27651574	27652248	675	SAT1_CF	blue	na
chrUn	27652291	27654486	2196	SAT1_CF	blue	na
chrUn	27654498	27655324	827	SAT1_CF	blue	na
chrUn	27655337	27656012	676	SAT1_CF	blue	na
chrUn	27656056	27656566	511	SAT1_CF	blue	na
chrUn	27656636	27657191	556	SAT1_CF	blue	na
chrUn	27657203	27658508	1306	SAT1_CF	blue	na
chrUn	27658551	27659508	958	SAT1_CF	blue	na
chrUn	27659587	27660547	961	SAT1_CF	blue	na
chrUn	27660587	27661088	502	SAT1_CF	blue	na
chrUn	27661100	27661784	685	SAT1_CF	blue	na
chrUn	27661796	27662472	677	SAT1_CF	blue	na
chrUn	27662515	27663883	1369	SAT1_CF	blue	na
chrUn	27663895	27664801	907	SAT1_CF	blue	na
chrUn	27664826	27665339	514	SAT1_CF	blue	na
chrUn	27665472	27666664	1193	SAT1_CF	blue	na
chrUn	27666676	27667254	579	SAT1_CF	blue	na
chrUn	27667292	27668155	864	SAT1_CF	blue	na
chrUn	27668198	27668571	374	SAT1_CF	blue	na
chrUn	27668607	27669107	501	SAT1_CF	blue	na
chrUn	27669121	27669803	683	SAT1_CF	blue	na
chrUn	27669817	27670495	679	SAT1_CF	blue	na
chrUn	27670575	27671203	629	SAT1_CF	blue	na
chrUn	27671215	27671892	678	SAT1_CF	blue	na
chrUn	27671937	27672459	523	SAT1_CF	blue	na
chrUn	27672498	27672643	146	SAT1_CF	blue	na
chrUn	27672703	27673664	962	SAT1_CF	blue	na
chrUn	27673685	27674330	646	SAT1_CF	blue	na
chrUn	27694255	27695167	913	SAT1_CF	blue	na
chrUn	27695233	27695714	482	SAT1_CF	blue	na
chrUn	27695726	27696586	861	SAT1_CF	blue	na
chrUn	27696628	27698427	1800	SAT1_CF	blue	na
chrUn	27698503	27699680	1178	SAT1_CF	blue	na
chrUn	27699724	27700266	543	SAT1_CF	blue	na
chrUn	27700293	27700678	386	SAT1_CF	blue	na

chrUn	27700692	27701252	561	SAT1_CF	blue	na
chrUn	27702659	27703225	567	SAT1_CF	blue	na
chrUn	27703254	27704299	1046	SAT1_CF	blue	na
chrUn	27704351	27704508	158	SAT1_CF	blue	na
chrUn	27704553	27705055	503	SAT1_CF	blue	na
chrUn	27705068	27706483	1416	SAT1_CF	blue	na
chrUn	27706499	27708257	1759	SAT1_CF	blue	na
chrUn	27708269	27709122	854	SAT1_CF	blue	na
chrUn	27709135	27711578	2444	SAT1_CF	blue	na
chrUn	27711589	27711912	324	SAT1_CF	blue	na
chrUn	27711961	27712463	503	SAT1_CF	blue	na
chrUn	27712475	27713109	635	SAT1_CF	blue	na
chrUn	27713135	27714101	967	SAT1_CF	blue	na
chrUn	27714114	27714271	158	SAT1_CF	blue	na
chrUn	27714318	27714839	522	SAT1_CF	blue	na
chrUn	27714852	27715457	606	SAT1_CF	blue	na
chrUn	27715469	27716641	1173	SAT1_CF	blue	na
chrUn	27716693	27717385	693	SAT1_CF	blue	na
chrUn	27731554	27732382	829	Sat2CF	purple	na
chrUn	27732064	27732421	358	Sat2CF	purple	na
chrUn	27776167	27776974	808	Sat2CF	purple	na
chrUn	27776252	27777116	865	Sat2CF	purple	na
chrUn	27801898	27802077	180	Sat2CF	purple	na
chrUn	27801917	27803169	1253	Sat2CF	purple	na
chrUn	27801953	27803039	1087	Sat2CF	purple	na
chrUn	27880596	27881503	908	CarSat1	red	na
chrUn	27881534	27882783	1250	CarSat1	red	na
chrUn	27914453	27915409	957	CarSat1	red	na
chrUn	27915569	27917019	1451	CarSat1	red	na
chrUn	27917040	27918636	1597	CarSat1	red	na
chrUn	27918657	27920254	1598	CarSat1	red	na
chrUn	27920285	27921864	1580	CarSat1	red	na
chrUn	27923192	27924507	1316	CarSat1	red	na
chrUn	27924538	27926126	1589	CarSat1	red	na
chrUn	27926157	27927745	1589	CarSat1	red	na
chrUn	27927776	27929364	1589	CarSat1	red	na
chrUn	27929395	27931394	2000	CarSat1	red	na
chrUn	27931410	27932291	882	CarSat1	red	na
chrUn	27932488	27933201	714	CarSat1	red	na
chrUn	27941781	27942368	588	CarSat1	red	na
chrUn	27942567	27943665	1099	CarSat1	red	na
chrUn	27943699	27944106	408	CarSat1	red	na

chrUn	27949893	27950055	163	Sat2CF	purple	na
chrUn	27949921	27950974	1054	Sat2CF	purple	na
chrUn	27950851	27950995	145	Sat2CF	purple	na
chrUn	27951204	27952133	930	SAT1_CF	blue	na
chrUn	27952163	27952565	403	SAT1_CF	blue	na
chrUn	27952597	27952999	403	CarSat1	red	na
chrUn	27953018	27954797	1780	CarSat1	red	na
chrUn	27954831	27955238	408	CarSat1	red	na
chrUn	27964304	27964871	568	CarSat1	red	na
chrUn	27965060	27965181	122	CarSat1	red	na
chrUn	27965199	27965994	796	CarSat1	red	na
chrUn	27966021	27966463	443	CarSat1	red	na
chrUn	27966636	27966745	110	CarSat1	red	na
chrUn	27966757	27967747	991	CarSat1	red	na
chrUn	27987937	27988105	169	Sat2CF	purple	na
chrUn	27987987	27988781	795	Sat2CF	purple	na
chrUn	28011929	28012397	469	CarSat1	red	na
chrUn	28012413	28013870	1458	CarSat1	red	na
chrUn	28013886	28014029	144	CarSat1	red	na
chrUn	28014229	28014942	714	CarSat1	red	na
chrUn	28023535	28024174	640	CarSat1	red	na
chrUn	28029979	28030356	378	Sat2CF	purple	na
chrUn	28030040	28031039	1000	Sat2CF	purple	na
chrUn	28030931	28031091	161	Sat2CF	purple	na
chrUn	28031285	28032679	1395	SAT1_CF	blue	na
chrUn	28032690	28033092	403	CarSat1	red	na
chrUn	28033111	28035369	2259	CarSat1	red	na
chrUn	28035390	28036107	718	CarSat1	red	na
chrUn	28036133	28036843	711	CarSat1	red	na
chrUn	28036864	28037838	975	CarSat1	red	na
chrUn	28037868	28038963	1096	CarSat1	red	na
chrUn	28039158	28039257	100	CarSat1	red	na
chrUn	28039275	28039870	596	CarSat1	red	na
chrUn	28048273	28049753	1481	CarSat1	red	na
chrUn	28049786	28051316	1531	CarSat1	red	na
chrUn	28053983	28055234	1252	Sat2CF	purple	na
chrUn	28054019	28055104	1086	Sat2CF	purple	na
chrUn	28067516	28068302	787	Sat2CF	purple	na
chrUn	28067981	28068361	381	Sat2CF	purple	na
chrUn	28083258	28083435	178	Sat2CF	purple	na
chrUn	28083277	28083866	590	Sat2CF	purple	na
chrUn	28083306	28083821	516	Sat2CF	purple	na

chrUn	28092702	28092888	187	Sat2CF	purple	na
chrUn	28092764	28093847	1084	Sat2CF	purple	na
chrUn	28092849	28093981	1133	Sat2CF	purple	na
chrUn	28112070	28112753	684	Sat2CF	purple	na
chrUn	28112142	28112829	688	Sat2CF	purple	na
chrUn	28156332	28157438	1107	Sat2CF	purple	na
chrUn	28169429	28170300	872	Sat2CF	purple	na
chrUn	28169504	28170224	721	Sat2CF	purple	na
chrUn	28184977	28185154	178	Sat2CF	purple	na
chrUn	28185046	28186215	1170	Sat2CF	purple	na
chrUn	28186614	28187059	446	CarSat1	red	na
chrUn	28187152	28188743	1592	CarSat1	red	na
chrUn	28188764	28190277	1514	CarSat1	red	na
chrUn	28190293	28191478	1186	CarSat1	red	na
chrUn	28191483	28193567	2085	SAT1_CF	blue	na
chrUn	28193621	28194154	534	SAT1_CF	blue	na
chrUn	28194171	28195519	1349	SAT1_CF	blue	na
chrUn	28195541	28196321	781	SAT1_CF	blue	na
chrUn	28196351	28196897	547	SAT1_CF	blue	na
chrUn	28196935	28198181	1247	SAT1_CF	blue	na
chrUn	28198216	28198717	502	SAT1_CF	blue	na
chrUn	28198731	28199931	1201	SAT1_CF	blue	na
chrUn	28199973	28201744	1772	SAT1_CF	blue	na
chrUn	28201757	28202884	1128	SAT1_CF	blue	na
chrUn	28202880	28203604	725	CarSat1	red	na
chrUn	28203755	28204053	299	CarSat1	red	na
chrUn	28205483	28206690	1208	CarSat1	red	na
chrUn	28206717	28207422	706	CarSat1	red	na
chrUn	28207449	28209383	1935	CarSat1	red	na
chrUn	28209408	28211219	1812	CarSat1	red	na
chrUn	28211235	28212423	1189	CarSat1	red	na
chrUn	28212453	28213466	1014	CarSat1	red	na
chrUn	28213491	28214213	723	CarSat1	red	na
chrUn	28214230	28215850	1621	CarSat1	red	na
chrUn	28216049	28216762	714	CarSat1	red	na
chrUn	28221951	28222328	378	Sat2CF	purple	na
chrUn	28222012	28222813	802	Sat2CF	purple	na
chrUn	28223018	28223174	157	Sat2CF	purple	na
chrUn	28223048	28223208	161	Sat2CF	purple	na
chrUn	28223402	28224789	1388	SAT1_CF	blue	na
chrUn	28224807	28225208	402	CarSat1	red	na
chrUn	28225227	28227098	1872	CarSat1	red	na

chrUn	28255598	28255765	168	Sat2CF	purple	na
chrUn	28255619	28256451	833	Sat2CF	purple	na
chrUn	28255657	28256377	721	Sat2CF	purple	na
chrUn	28271381	28271931	551	Sat2CF	purple	na
chrUn	28280752	28280953	202	Sat2CF	purple	na
chrUn	28280793	28281969	1177	Sat2CF	purple	na
chrUn	28280824	28281842	1019	Sat2CF	purple	na
chrUn	28306003	28307252	1250	Sat2CF	purple	na
chrUn	28339320	28340057	738	Sat2CF	purple	na
chrUn	28339322	28339983	662	Sat2CF	purple	na
chrUn	28362536	28363348	813	Sat2CF	purple	na
chrUn	28362549	28363274	726	Sat2CF	purple	na
chrUn	28381548	28382664	1117	Sat2CF	purple	na
chrUn	28401148	28402046	899	Sat2CF	purple	na
chrUn	28401193	28401983	791	Sat2CF	purple	na
chrUn	28433530	28434427	898	Sat2CF	purple	na
chrUn	28433575	28434364	790	Sat2CF	purple	na
chrUn	28461373	28462568	1196	Sat2CF	purple	na
chrUn	28461404	28462422	1019	Sat2CF	purple	na
chrUn	28490946	28492049	1104	Sat2CF	purple	na
chrUn	28504434	28505291	858	Sat2CF	purple	na
chrUn	28519995	28520134	140	Sat2CF	purple	na
chrUn	28520026	28521216	1191	Sat2CF	purple	na
chrUn	28591653	28592364	712	Sat2CF	purple	na
chrUn	28592229	28592387	159	Sat2CF	purple	na
chrUn	28606947	28608531	1585	CarSat1	red	na
chrUn	28608557	28610521	1965	CarSat1	red	na
chrUn	28610523	28613730	3208	SAT1_CF	blue	na
chrUn	28613770	28614415	646	SAT1_CF	blue	na
chrUn	28622653	28623444	792	Sat2CF	purple	na
chrUn	28623194	28623507	314	Sat2CF	purple	na
chrUn	28641419	28642535	1117	Sat2CF	purple	na
chrUn	28654762	28655877	1116	Sat2CF	purple	na
chrUn	28659694	28662369	2676	CarSat1	red	na
chrUn	28662391	28665584	3194	CarSat1	red	na
chrUn	28665604	28667197	1594	CarSat1	red	na
chrUn	28667216	28668217	1002	CarSat1	red	na
chrUn	28668274	28668883	610	CarSat1	red	na
chrUn	28668904	28669637	734	CarSat1	red	na
chrUn	28678616	28679092	477	CarSat1	red	na
chrUn	28679108	28679639	532	CarSat1	red	na
chrUn	28700829	28702007	1179	Sat2CF	purple	na

chrUn	28723069	28724178	1110	Sat2CF	purple	na
chrUn	28742281	28742575	295	Sat2CF	purple	na
chrUn	28742327	28743026	700	Sat2CF	purple	na
chrUn	28742892	28743054	163	Sat2CF	purple	na
chrUn	28763069	28764225	1157	Sat2CF	purple	na
chrUn	28763091	28764088	998	Sat2CF	purple	na
chrUn	28786175	28786940	766	Sat2CF	purple	na
chrUn	28801843	28802424	582	CarSat1	red	na
chrUn	28802446	28804030	1585	CarSat1	red	na
chrUn	28804051	28806025	1975	CarSat1	red	na
chrUn	28806027	28806496	470	SAT1_CF	blue	na
chrUn	28806511	28809308	2798	SAT1_CF	blue	na
chrUn	28809348	28810403	1056	SAT1_CF	blue	na
chrUn	28810398	28811117	720	CarSat1	red	na
chrUn	28811269	28811564	296	CarSat1	red	na
chrUn	28813245	28814460	1216	SAT1_CF	blue	na
chrUn	28814641	28814826	186	SAT1_CF	blue	na
chrUn	28814843	28816047	1205	SAT1_CF	blue	na
chrUn	28816072	28816406	335	SAT1_CF	blue	na
chrUn	28816485	28817232	748	SAT1_CF	blue	na
chrUn	28817284	28817809	526	SAT1_CF	blue	na
chrUn	28817821	28817927	107	SAT1_CF	blue	na
chrUn	28817951	28818578	628	SAT1_CF	blue	na
chrUn	28818613	28819461	849	SAT1_CF	blue	na
chrUn	28819479	28820165	687	SAT1_CF	blue	na
chrUn	28820177	28820850	674	SAT1_CF	blue	na
chrUn	28820894	28821437	544	SAT1_CF	blue	na
chrUn	28821455	28822141	687	SAT1_CF	blue	na
chrUn	28822153	28823003	851	SAT1_CF	blue	na
chrUn	28823016	28823631	616	SAT1_CF	blue	na
chrUn	28823673	28824857	1185	SAT1_CF	blue	na
chrUn	28824944	28825441	498	SAT1_CF	blue	na
chrUn	28825459	28825561	103	SAT1_CF	blue	na
chrUn	28825582	28826137	556	SAT1_CF	blue	na
chrUn	28826150	28827388	1239	SAT1_CF	blue	na
chrUn	28827431	28828394	964	SAT1_CF	blue	na
chrUn	28828461	28828973	513	SAT1_CF	blue	na
chrUn	28829016	28829386	371	SAT1_CF	blue	na
chrUn	28829441	28829923	483	SAT1_CF	blue	na
chrUn	28829935	28830620	686	SAT1_CF	blue	na
chrUn	28830642	28831307	666	SAT1_CF	blue	na
chrUn	28831352	28832680	1329	SAT1_CF	blue	na

chrUn	28832692	28833553	862	SAT1_CF	blue	na
chrUn	28833566	28833674	109	SAT1_CF	blue	na
chrUn	28833695	28835552	1858	SAT1_CF	blue	na
chrUn	28835565	28838252	2688	SAT1_CF	blue	na
chrUn	28838294	28839203	910	SAT1_CF	blue	na
chrUn	28839276	28839757	482	SAT1_CF	blue	na
chrUn	28839770	28840453	684	SAT1_CF	blue	na
chrUn	28840465	28841140	676	SAT1_CF	blue	na
chrUn	28841183	28842193	1011	SAT1_CF	blue	na
chrUn	28842274	28843231	958	SAT1_CF	blue	na
chrUn	28843274	28843776	503	SAT1_CF	blue	na
chrUn	28843789	28844426	638	SAT1_CF	blue	na
chrUn	28844441	28845254	814	SAT1_CF	blue	na
chrUn	28845306	28846007	702	SAT1_CF	blue	na
chrUn	28846019	28846125	107	SAT1_CF	blue	na
chrUn	28846149	28846777	629	SAT1_CF	blue	na
chrUn	28846812	28847661	850	SAT1_CF	blue	na
chrUn	28847679	28848365	687	SAT1_CF	blue	na
chrUn	28848377	28849227	851	SAT1_CF	blue	na
chrUn	28849277	28849976	700	SAT1_CF	blue	na
chrUn	28849990	28851528	1539	SAT1_CF	blue	na
chrUn	28851541	28851648	108	SAT1_CF	blue	na
chrUn	28851670	28852224	555	SAT1_CF	blue	na
chrUn	28852236	28853723	1488	SAT1_CF	blue	na
chrUn	28853763	28855880	2118	SAT1_CF	blue	na
chrUn	28855908	28856042	135	SAT1_CF	blue	na
chrUn	28856110	28857078	969	SAT1_CF	blue	na
chrUn	28857090	28857774	685	SAT1_CF	blue	na
chrUn	28857787	28858626	840	SAT1_CF	blue	na
chrUn	28858639	28859831	1193	SAT1_CF	blue	na
chrUn	28859843	28861876	2034	SAT1_CF	blue	na
chrUn	28862058	28862239	182	SAT1_CF	blue	na
chrUn	28862279	28862858	580	SAT1_CF	blue	na
chrUn	28862920	28863720	801	SAT1_CF	blue	na
chrUn	28863737	28863889	153	SAT1_CF	blue	na
chrUn	28863911	28864417	507	SAT1_CF	blue	na
chrUn	28864437	28865042	606	SAT1_CF	blue	na
chrUn	28865057	28865459	403	SAT1_CF	blue	na
chrUn	28865499	28866074	576	SAT1_CF	blue	na
chrUn	28866096	28866770	675	SAT1_CF	blue	na
chrUn	28866782	28867644	863	SAT1_CF	blue	na
chrUn	28867686	28868317	632	SAT1_CF	blue	na

chrUn	28868345	28868908	564	SAT1_CF	blue	na
chrUn	28868988	28869464	477	SAT1_CF	blue	na
chrUn	28869527	28869663	137	SAT1_CF	blue	na
chrUn	28869710	28870230	521	SAT1_CF	blue	na
chrUn	28870242	28870916	675	SAT1_CF	blue	na
chrUn	28870959	28872051	1093	SAT1_CF	blue	na
chrUn	28872063	28872574	512	SAT1_CF	blue	na
chrUn	28872654	28873235	582	SAT1_CF	blue	na
chrUn	28873259	28873923	665	SAT1_CF	blue	na
chrUn	28873966	28875484	1519	SAT1_CF	blue	na
chrUn	28877068	28877817	750	SAT1_CF	blue	na
chrUn	28877829	28879634	1806	SAT1_CF	blue	na
chrUn	28879652	28880584	933	SAT1_CF	blue	na
chrUn	28880626	28881128	503	SAT1_CF	blue	na
chrUn	28881140	28881824	685	SAT1_CF	blue	na
chrUn	28881867	28883238	1372	SAT1_CF	blue	na
chrUn	28883317	28883866	550	SAT1_CF	blue	na
chrUn	28883902	28884276	375	SAT1_CF	blue	na
chrUn	28884316	28884904	589	SAT1_CF	blue	na
chrUn	28884960	28885512	553	SAT1_CF	blue	na
chrUn	28885530	28886119	590	SAT1_CF	blue	na
chrUn	28886131	28886214	84	SAT1_CF	blue	na
chrUn	28886227	28887174	948	SAT1_CF	blue	na
chrUn	28887227	28887729	503	SAT1_CF	blue	na
chrUn	28887742	28888227	486	SAT1_CF	blue	na
chrUn	28888328	28889024	697	SAT1_CF	blue	na
chrUn	28889067	28890049	983	SAT1_CF	blue	na
chrUn	28890104	28890707	604	SAT1_CF	blue	na
chrUn	28890790	28891109	320	SAT1_CF	blue	na
chrUn	28891143	28891657	515	SAT1_CF	blue	na
chrUn	28891691	28892317	627	SAT1_CF	blue	na
chrUn	28892362	28892992	631	SAT1_CF	blue	na
chrUn	28893020	28893582	563	SAT1_CF	blue	na
chrUn	28893662	28894171	510	SAT1_CF	blue	na
chrUn	28894199	28894332	134	SAT1_CF	blue	na
chrUn	28894390	28895587	1198	SAT1_CF	blue	na
chrUn	28895599	28896268	670	SAT1_CF	blue	na
chrUn	28896310	28897275	966	SAT1_CF	blue	na
chrUn	28897320	28898271	952	SAT1_CF	blue	na
chrUn	28898314	28898815	502	SAT1_CF	blue	na
chrUn	28898827	28898933	107	SAT1_CF	blue	na
chrUn	28898956	28899511	556	SAT1_CF	blue	na

chrUn	28899523	28899632	110	SAT1_CF	blue	na
chrUn	28899652	28901914	2263	SAT1_CF	blue	na
chrUn	28901925	28902922	998	SAT1_CF	blue	na
chrUn	28902937	28903093	157	SAT1_CF	blue	na
chrUn	28903138	28903639	502	SAT1_CF	blue	na
chrUn	28903651	28903767	117	SAT1_CF	blue	na
chrUn	28903781	28904306	526	SAT1_CF	blue	na
chrUn	28904362	28904908	547	SAT1_CF	blue	na
chrUn	28904951	28905582	632	SAT1_CF	blue	na
chrUn	28905613	28906252	640	SAT1_CF	blue	na
chrUn	28906317	28908283	1967	SAT1_CF	blue	na
chrUn	28908296	28909519	1224	SAT1_CF	blue	na
chrUn	28909562	28910542	981	SAT1_CF	blue	na
chrUn	28910587	28911614	1028	SAT1_CF	blue	na
chrUn	28911638	28912247	610	SAT1_CF	blue	na
chrUn	28912300	28912817	518	SAT1_CF	blue	na
chrUn	28912860	28915413	2554	SAT1_CF	blue	na
chrUn	28916200	28917236	1037	SAT1_CF	blue	na
chrUn	28917247	28917566	320	SAT1_CF	blue	na
chrUn	28917619	28918120	502	SAT1_CF	blue	na
chrUn	28918132	28918805	674	SAT1_CF	blue	na
chrUn	28918844	28920012	1169	SAT1_CF	blue	na
chrUn	28920109	28920657	549	SAT1_CF	blue	na
chrUn	28920689	28921065	377	SAT1_CF	blue	na
chrUn	28921108	28921609	502	SAT1_CF	blue	na
chrUn	28921621	28923453	1833	SAT1_CF	blue	na
chrUn	28923532	28924042	511	SAT1_CF	blue	na
chrUn	28924070	28924227	158	SAT1_CF	blue	na
chrUn	28924272	28924772	501	SAT1_CF	blue	na
chrUn	28924794	28926857	2064	SAT1_CF	blue	na
chrUn	28926877	28927077	201	SAT1_CF	blue	na
chrUn	28927090	28928447	1358	SAT1_CF	blue	na
chrUn	28928490	28930778	2289	SAT1_CF	blue	na
chrUn	28930821	28931322	502	SAT1_CF	blue	na
chrUn	28931340	28932016	677	SAT1_CF	blue	na
chrUn	28932030	28933288	1259	SAT1_CF	blue	na
chrUn	28933375	28933867	493	SAT1_CF	blue	na
chrUn	28933887	28934657	771	SAT1_CF	blue	na
chrUn	28934676	28937085	2410	SAT1_CF	blue	na
chrUn	28937110	28938575	1466	SAT1_CF	blue	na
chrUn	28938587	28940974	2388	SAT1_CF	blue	na
chrUn	28941162	28941505	344	SAT1_CF	blue	na

chrUn	28941573	28943497	1925	SAT1_CF	blue	na
chrUn	28943554	28944089	536	SAT1_CF	blue	na
chrUn	28944169	28944678	510	SAT1_CF	blue	na
chrUn	28944706	28944863	158	SAT1_CF	blue	na
chrUn	28944889	28945410	522	SAT1_CF	blue	na
chrUn	28945422	28945494	73	SAT1_CF	blue	na
chrUn	28945551	28946303	753	SAT1_CF	blue	na
chrUn	28946325	28946869	545	SAT1_CF	blue	na
chrUn	28946912	28948283	1372	SAT1_CF	blue	na
chrUn	28948305	28948979	675	SAT1_CF	blue	na
chrUn	28948991	28951243	2253	SAT1_CF	blue	na
chrUn	28951261	28952240	980	SAT1_CF	blue	na
chrUn	28952264	28952668	405	SAT1_CF	blue	na
chrUn	28952706	28953210	505	SAT1_CF	blue	na
chrUn	28953228	28953912	685	SAT1_CF	blue	na
chrUn	28953924	28956997	3074	SAT1_CF	blue	na
chrUn	28957065	28958201	1137	SAT1_CF	blue	na
chrUn	28958215	28959585	1371	SAT1_CF	blue	na
chrUn	28959633	28961397	1765	SAT1_CF	blue	na
chrUn	28961408	28962014	607	SAT1_CF	blue	na
chrUn	28962084	28962638	555	SAT1_CF	blue	na
chrUn	28962651	28962722	72	SAT1_CF	blue	na
chrUn	28962742	28963937	1196	SAT1_CF	blue	na
chrUn	28963981	28964952	972	SAT1_CF	blue	na
chrUn	28964963	28965611	649	SAT1_CF	blue	na
chrUn	28965690	28966321	632	SAT1_CF	blue	na
chrUn	28966333	28967013	681	SAT1_CF	blue	na
chrUn	28967025	28967701	677	SAT1_CF	blue	na
chrUn	28967748	28968703	956	SAT1_CF	blue	na
chrUn	28968782	28969289	508	SAT1_CF	blue	na
chrUn	28969303	28969705	403	SAT1_CF	blue	na
chrUn	28969764	28970330	567	SAT1_CF	blue	na
chrUn	28970351	28971390	1040	SAT1_CF	blue	na
chrUn	28971423	28972306	884	SAT1_CF	blue	na
chrUn	28972395	28973591	1197	SAT1_CF	blue	na
chrUn	28973603	28974268	666	SAT1_CF	blue	na
chrUn	28974337	28974882	546	SAT1_CF	blue	na
chrUn	28974923	28975468	546	SAT1_CF	blue	na
chrUn	28975510	28975789	280	SAT1_CF	blue	na
chrUn	28975805	28976327	523	SAT1_CF	blue	na
chrUn	28976357	28976740	384	SAT1_CF	blue	na
chrUn	28976778	28977276	499	SAT1_CF	blue	na

chrUn	28977294	28977397	104	SAT1_CF	blue	na
chrUn	28977418	28979333	1916	SAT1_CF	blue	na
chrUn	28979348	28979484	137	SAT1_CF	blue	na
chrUn	28979580	28980099	520	SAT1_CF	blue	na
chrUn	28980156	28980701	546	SAT1_CF	blue	na
chrUn	28980781	28981324	544	SAT1_CF	blue	na
chrUn	28981421	28982563	1143	SAT1_CF	blue	na
chrUn	28982605	28983103	499	SAT1_CF	blue	na
chrUn	28983120	28983799	680	SAT1_CF	blue	na
chrUn	28983812	28983887	76	SAT1_CF	blue	na
chrUn	28983905	28984413	509	SAT1_CF	blue	na
chrUn	28984425	28986106	1682	SAT1_CF	blue	na
chrUn	28986185	28986731	547	SAT1_CF	blue	na
chrUn	28986774	28987312	539	SAT1_CF	blue	na
chrUn	28987393	28987930	538	SAT1_CF	blue	na
chrUn	28987947	28988607	661	SAT1_CF	blue	na
chrUn	28988655	28989188	534	SAT1_CF	blue	na
chrUn	28989222	28991301	2080	SAT1_CF	blue	na
chrUn	28991497	28992772	1276	SAT1_CF	blue	na
chrUn	28992796	28993587	792	SAT1_CF	blue	na
chrUn	29236836	29237509	674	Sat7CF	lime	na
chrUn	29239817	29239868	52	Sat109CF	na	na
chrUn	29239817	29239868	52	Sat109CF	na	na
chrUn	29344043	29344711	669	Sat7CF	lime	na
chrUn	29344121	29344704	584	Sat7CF	lime	na
chrUn	29393654	29394902	1249	Sat2CF	purple	na
chrUn	29408189	29409357	1169	Sat2CF	purple	na
chrUn	29409249	29409373	125	Sat2CF	purple	na
chrUn	29427150	29428370	1221	Sat2CF	purple	na
chrUn	29428241	29428390	150	Sat2CF	purple	na
chrUn	29439751	29441003	1253	Sat2CF	purple	na
chrUn	29457866	29459034	1169	Sat2CF	purple	na
chrUn	29458926	29459050	125	Sat2CF	purple	na
chrUn	29474138	29474969	832	Sat2CF	purple	na
chrUn	29474222	29474948	727	Sat2CF	purple	na
chrUn	29474838	29475006	169	Sat2CF	purple	na
chrUn	29487804	29489025	1222	Sat2CF	purple	na
chrUn	29504252	29505327	1076	Sat2CF	purple	na
chrUn	29534904	29536058	1155	Sat2CF	purple	na
chrUn	29562049	29562926	878	CarSat1	red	na
chrUn	29563165	29564609	1445	CarSat1	red	na
chrUn	29564640	29566228	1589	CarSat1	red	na

chrUn	29566259	29567848	1590	CarSat1	red	na
chrUn	29567879	29569467	1589	CarSat1	red	na
chrUn	29569498	29571086	1589	CarSat1	red	na
chrUn	29571117	29572705	1589	CarSat1	red	na
chrUn	29572736	29574324	1589	CarSat1	red	na
chrUn	29574355	29575943	1589	CarSat1	red	na
chrUn	29575974	29577562	1589	CarSat1	red	na
chrUn	29577593	29579181	1589	CarSat1	red	na
chrUn	29579212	29580801	1590	CarSat1	red	na
chrUn	29580832	29582829	1998	CarSat1	red	na
chrUn	29582845	29583730	886	CarSat1	red	na
chrUn	29583930	29584029	100	CarSat1	red	na
chrUn	29584047	29584639	593	CarSat1	red	na
chrUn	29589111	29589698	588	CarSat1	red	na
chrUn	29589899	29589998	100	CarSat1	red	na
chrUn	29590016	29590997	982	CarSat1	red	na
chrUn	29591031	29591438	408	CarSat1	red	na
chrUn	29597225	29598237	1013	Sat2CF	purple	na
chrUn	29597288	29598151	864	Sat2CF	purple	na
chrUn	29598112	29598272	161	Sat2CF	purple	na
chrUn	29598466	29599046	581	SAT1_CF	blue	na
chrUn	29599103	29599842	740	SAT1_CF	blue	na
chrUn	29599860	29600262	403	CarSat1	red	na
chrUn	29600281	29602060	1780	CarSat1	red	na
chrUn	29602094	29602495	402	CarSat1	red	na
chrUn	29611525	29612092	568	CarSat1	red	na
chrUn	29612281	29612402	122	CarSat1	red	na
chrUn	29612420	29613215	796	CarSat1	red	na
chrUn	29613236	29613686	451	CarSat1	red	na
chrUn	29613859	29614937	1079	CarSat1	red	na
chrUn	29617590	29617808	219	Sat2CF	purple	na
chrUn	29617648	29618897	1250	Sat2CF	purple	na
chrUn	29617684	29618767	1084	Sat2CF	purple	na
chrUn	29635263	29635431	169	Sat2CF	purple	na
chrUn	29635305	29636103	799	Sat2CF	purple	na
chrUn	29651023	29651200	178	Sat2CF	purple	na
chrUn	29651071	29651625	555	Sat2CF	purple	na
chrUn	29660515	29661692	1178	Sat2CF	purple	na
chrUn	29660562	29661565	1004	Sat2CF	purple	na
chrUn	29679969	29681121	1153	Sat2CF	purple	na
chrUn	29694288	29694466	179	Sat2CF	purple	na
chrUn	29694344	29695394	1051	Sat2CF	purple	na

chrUn	29716502	29717205	704	CarSat1	red	na
chrUn	29717226	29718817	1592	CarSat1	red	na
chrUn	29718838	29719691	854	CarSat1	red	na
chrUn	29719713	29720568	856	CarSat1	red	na
chrUn	29720589	29721367	779	CarSat1	red	na
chrUn	29721381	29721824	444	CarSat1	red	na
chrUn	29721827	29722212	386	SAT1_CF	blue	na
chrUn	29722224	29722840	617	SAT1_CF	blue	na
chrUn	29722835	29723559	725	CarSat1	red	na
chrUn	29723718	29724017	300	CarSat1	red	na
chrUn	29725420	29726081	662	SAT1_CF	blue	na
chrUn	29726093	29727279	1187	SAT1_CF	blue	na
chrUn	29727292	29727895	604	SAT1_CF	blue	na
chrUn	29727907	29728582	676	SAT1_CF	blue	na
chrUn	29728625	29729620	996	SAT1_CF	blue	na
chrUn	29729664	29730623	960	SAT1_CF	blue	na
chrUn	29730663	29731164	502	SAT1_CF	blue	na
chrUn	29731177	29731859	683	SAT1_CF	blue	na
chrUn	29731871	29732544	674	SAT1_CF	blue	na
chrUn	29732560	29732844	285	SAT1_CF	blue	na
chrUn	29732864	29733408	545	SAT1_CF	blue	na
chrUn	29733454	29734147	694	SAT1_CF	blue	na
chrUn	29734159	29734811	653	SAT1_CF	blue	na
chrUn	29734846	29735645	800	SAT1_CF	blue	na
chrUn	29735657	29736515	859	SAT1_CF	blue	na
chrUn	29736557	29737453	897	SAT1_CF	blue	na
chrUn	29737471	29738435	965	SAT1_CF	blue	na
chrUn	29738483	29738980	498	SAT1_CF	blue	na
chrUn	29738998	29739663	666	SAT1_CF	blue	na
chrUn	29739689	29740149	461	SAT1_CF	blue	na
chrUn	29740191	29740730	540	SAT1_CF	blue	na
chrUn	29740749	29742087	1339	SAT1_CF	blue	na
chrUn	29742205	29742724	520	SAT1_CF	blue	na
chrUn	29742781	29743950	1170	SAT1_CF	blue	na
chrUn	29744031	29746751	2721	SAT1_CF	blue	na
chrUn	29746776	29747687	912	SAT1_CF	blue	na
chrUn	29747709	29748383	675	SAT1_CF	blue	na
chrUn	29748395	29749071	677	SAT1_CF	blue	na
chrUn	29749114	29750079	966	SAT1_CF	blue	na
chrUn	29750158	29751112	955	SAT1_CF	blue	na
chrUn	29751152	29751653	502	SAT1_CF	blue	na
chrUn	29751680	29752350	671	SAT1_CF	blue	na

chrUn	29752362	29753402	1041	SAT1_CF	blue	na
chrUn	29753416	29754053	638	SAT1_CF	blue	na
chrUn	29754066	29755105	1040	SAT1_CF	blue	na
chrUn	29755184	29755752	569	SAT1_CF	blue	na
chrUn	29755765	29756448	684	SAT1_CF	blue	na
chrUn	29756528	29757016	489	SAT1_CF	blue	na
chrUn	29757067	29757224	158	SAT1_CF	blue	na
chrUn	29757269	29757770	502	SAT1_CF	blue	na
chrUn	29757781	29759157	1377	SAT1_CF	blue	na
chrUn	29759199	29760049	851	SAT1_CF	blue	na
chrUn	29760119	29760664	546	SAT1_CF	blue	na
chrUn	29760711	29761667	957	SAT1_CF	blue	na
chrUn	29761711	29762253	543	SAT1_CF	blue	na
chrUn	29762267	29762666	400	SAT1_CF	blue	na
chrUn	29762728	29763208	481	SAT1_CF	blue	na
chrUn	29763221	29764354	1134	SAT1_CF	blue	na
chrUn	29764387	29765269	883	SAT1_CF	blue	na
chrUn	29765358	29765851	494	SAT1_CF	blue	na
chrUn	29765870	29766553	684	SAT1_CF	blue	na
chrUn	29766565	29767241	677	SAT1_CF	blue	na
chrUn	29767310	29768623	1314	SAT1_CF	blue	na
chrUn	29768661	29769883	1223	SAT1_CF	blue	na
chrUn	29769970	29770468	499	SAT1_CF	blue	na
chrUn	29770481	29771183	703	SAT1_CF	blue	na
chrUn	29771195	29771855	661	SAT1_CF	blue	na
chrUn	29771903	29772436	534	SAT1_CF	blue	na
chrUn	29772465	29773130	666	SAT1_CF	blue	na
chrUn	29773174	29774551	1378	SAT1_CF	blue	na
chrUn	29774746	29775246	501	SAT1_CF	blue	na
chrUn	29775264	29776684	1421	SAT1_CF	blue	na
chrUn	29776726	29777272	547	SAT1_CF	blue	na
chrUn	29777285	29777687	403	SAT1_CF	blue	na
chrUn	29777727	29778228	502	SAT1_CF	blue	na
chrUn	29778241	29779578	1338	SAT1_CF	blue	na
chrUn	29779657	29780181	525	SAT1_CF	blue	na
chrUn	29780196	29780984	789	SAT1_CF	blue	na
chrUn	29781040	29781594	555	SAT1_CF	blue	na
chrUn	29781606	29782778	1173	SAT1_CF	blue	na
chrUn	29782859	29783650	792	SAT1_CF	blue	na
chrUn	29783830	29784014	185	SAT1_CF	blue	na
chrUn	29784074	29784547	474	SAT1_CF	blue	na
chrUn	29784567	29784672	106	SAT1_CF	blue	na

chrUn	29784695	29785955	1261	SAT1_CF	blue	na
chrUn	29785983	29786140	158	SAT1_CF	blue	na
chrUn	29786166	29787292	1127	SAT1_CF	blue	na
chrUn	29787319	29789421	2103	SAT1_CF	blue	na
chrUn	29789464	29790807	1344	SAT1_CF	blue	na
chrUn	29790851	29791352	502	SAT1_CF	blue	na
chrUn	29791366	29791974	609	SAT1_CF	blue	na
chrUn	29791986	29792821	836	SAT1_CF	blue	na
chrUn	29793002	29793187	186	SAT1_CF	blue	na
chrUn	29793223	29793725	503	SAT1_CF	blue	na
chrUn	29793743	29794499	757	SAT1_CF	blue	na
chrUn	29794523	29796503	1981	SAT1_CF	blue	na
chrUn	29796685	29797405	721	SAT1_CF	blue	na
chrUn	29797418	29798030	613	SAT1_CF	blue	na
chrUn	29798042	29798712	671	SAT1_CF	blue	na
chrUn	29798755	29800123	1369	SAT1_CF	blue	na
chrUn	29800166	29800714	549	SAT1_CF	blue	na
chrUn	29800737	29803644	2908	SAT1_CF	blue	na
chrUn	29803689	29804687	999	SAT1_CF	blue	na
chrUn	29804732	29805584	853	SAT1_CF	blue	na
chrUn	29805674	29806193	520	SAT1_CF	blue	na
chrUn	29806205	29806342	138	SAT1_CF	blue	na
chrUn	29806385	29808320	1936	SAT1_CF	blue	na
chrUn	29808335	29809114	780	SAT1_CF	blue	na
chrUn	29809126	29809802	677	SAT1_CF	blue	na
chrUn	29809845	29811281	1437	SAT1_CF	blue	na
chrUn	29811370	29811869	500	SAT1_CF	blue	na
chrUn	29811883	29812556	674	SAT1_CF	blue	na
chrUn	29812575	29813152	578	SAT1_CF	blue	na
chrUn	29813192	29813691	500	SAT1_CF	blue	na
chrUn	29813718	29814466	749	SAT1_CF	blue	na
chrUn	29814478	29815144	667	SAT1_CF	blue	na
chrUn	29815192	29816280	1089	SAT1_CF	blue	na
chrUn	29816291	29816812	522	SAT1_CF	blue	na
chrUn	29816856	29817250	395	SAT1_CF	blue	na
chrUn	29817263	29817848	586	SAT1_CF	blue	na
chrUn	29817904	29818478	575	SAT1_CF	blue	na
chrUn	29818490	29819150	661	SAT1_CF	blue	na
chrUn	29819198	29819737	540	SAT1_CF	blue	na
chrUn	29819759	29820424	666	SAT1_CF	blue	na
chrUn	29820468	29821025	558	SAT1_CF	blue	na
chrUn	29821105	29821629	525	SAT1_CF	blue	na

chrUn	29821644	29822345	702	SAT1_CF	blue	na
chrUn	29822358	29822589	232	SAT1_CF	blue	na
chrUn	29822486	29825477	2992	SAT1_CF	blue	na
chrUn	29825658	29825843	186	SAT1_CF	blue	na
chrUn	29825879	29826378	500	SAT1_CF	blue	na
chrUn	29826392	29827005	614	SAT1_CF	blue	na
chrUn	29827017	29827868	852	SAT1_CF	blue	na
chrUn	29827919	29828699	781	SAT1_CF	blue	na
chrUn	29828765	29829277	513	SAT1_CF	blue	na
chrUn	29829302	29829403	102	SAT1_CF	blue	na
chrUn	29829424	29829978	555	SAT1_CF	blue	na
chrUn	29829990	29830664	675	SAT1_CF	blue	na
chrUn	29830679	29830849	171	SAT1_CF	blue	na
chrUn	29830862	29832228	1367	SAT1_CF	blue	na
chrUn	29832241	29832886	646	SAT1_CF	blue	na
chrUn	29832911	29833236	326	SAT1_CF	blue	na
chrUn	29833316	29833822	507	SAT1_CF	blue	na
chrUn	29833843	29834238	396	SAT1_CF	blue	na
chrUn	29834278	29834778	501	SAT1_CF	blue	na
chrUn	29834796	29835843	1048	SAT1_CF	blue	na
chrUn	29835864	29836406	543	SAT1_CF	blue	na
chrUn	29836476	29837677	1202	SAT1_CF	blue	na
chrUn	29837721	29838027	307	SAT1_CF	blue	na
chrUn	29838107	29838612	506	SAT1_CF	blue	na
chrUn	29838627	29840265	1639	SAT1_CF	blue	na
chrUn	29840307	29841365	1059	SAT1_CF	blue	na
chrUn	29841435	29841860	426	SAT1_CF	blue	na
chrUn	29841902	29843749	1848	SAT1_CF	blue	na
chrUn	29843797	29844297	501	SAT1_CF	blue	na
chrUn	29844324	29845533	1210	SAT1_CF	blue	na
chrUn	29845545	29846189	645	SAT1_CF	blue	na
chrUn	29846214	29847046	833	SAT1_CF	blue	na
chrUn	29847111	29847560	450	SAT1_CF	blue	na
chrUn	29847647	29848148	502	SAT1_CF	blue	na
chrUn	29848161	29849741	1581	SAT1_CF	blue	na
chrUn	29849759	29850516	758	SAT1_CF	blue	na
chrUn	29850528	29851358	831	SAT1_CF	blue	na
chrUn	29851374	29852404	1031	SAT1_CF	blue	na
chrUn	29852425	29853056	632	SAT1_CF	blue	na
chrUn	29853069	29853753	685	SAT1_CF	blue	na
chrUn	29853766	29855938	2173	SAT1_CF	blue	na
chrUn	29857353	29858154	802	SAT1_CF	blue	na

chrUn	29858693	29859152	460	SAT1_CF	blue	na
chrUn	29859194	29860815	1622	SAT1_CF	blue	na
chrUn	29860849	29861368	520	SAT1_CF	blue	na
chrUn	29861395	29862055	661	SAT1_CF	blue	na
chrUn	29862068	29862203	136	SAT1_CF	blue	na
chrUn	29862245	29863023	779	SAT1_CF	blue	na
chrUn	29863101	29863647	547	SAT1_CF	blue	na
chrUn	29863730	29864309	580	SAT1_CF	blue	na
chrUn	29864322	29865875	1554	SAT1_CF	blue	na
chrUn	29865917	29866460	544	SAT1_CF	blue	na
chrUn	29866476	29867272	797	SAT1_CF	blue	na
chrUn	29867352	29867862	511	SAT1_CF	blue	na
chrUn	29867889	29868022	134	SAT1_CF	blue	na
chrUn	29868102	29868581	480	SAT1_CF	blue	na
chrUn	29868594	29869962	1369	SAT1_CF	blue	na
chrUn	29870004	29872492	2489	SAT1_CF	blue	na
chrUn	29872504	29873395	892	SAT1_CF	blue	na
chrUn	29873408	29873570	163	SAT1_CF	blue	na
chrUn	29873609	29873811	203	SAT1_CF	blue	na
chrUn	29873828	29874372	545	SAT1_CF	blue	na
chrUn	29874384	29875069	686	SAT1_CF	blue	na
chrUn	29875087	29875757	671	SAT1_CF	blue	na
chrUn	29875800	29876431	632	SAT1_CF	blue	na
chrUn	29876458	29877573	1116	SAT1_CF	blue	na
chrUn	29877585	29879182	1598	SAT1_CF	blue	na
chrUn	29879205	29880055	851	SAT1_CF	blue	na
chrUn	29880132	29880984	853	SAT1_CF	blue	na
chrUn	29881006	29881941	936	SAT1_CF	blue	na
chrUn	29881981	29883201	1221	SAT1_CF	blue	na
chrUn	29883233	29883754	522	SAT1_CF	blue	na
chrUn	29883769	29883926	158	SAT1_CF	blue	na
chrUn	29883952	29885301	1350	SAT1_CF	blue	na
chrUn	29885371	29885925	555	SAT1_CF	blue	na
chrUn	29885937	29886615	679	SAT1_CF	blue	na
chrUn	29886626	29887115	490	SAT1_CF	blue	na
chrUn	29887129	29888759	1631	SAT1_CF	blue	na
chrUn	29888774	29889335	562	SAT1_CF	blue	na
chrUn	29895540	29896377	838	Sat2CF	purple	na
chrUn	29908242	29909346	1105	Sat2CF	purple	na
chrUn	29958740	29959109	370	Sat2CF	purple	na
chrUn	29958792	29959618	827	Sat2CF	purple	na
chrUn	29975156	29976388	1233	Sat2CF	purple	na

chrUn	29975284	29976368	1085	Sat2CF	purple	na
chrUn	29999716	30000236	521	Sat2CF	purple	na
chrUn	30000429	30000904	476	Sat2CF	purple	na
chrUn	30000441	30000957	517	Sat2CF	purple	na
chrUn	30000802	30000998	197	Sat2CF	purple	na
chrUn	30002630	30003043	414	CarSat1	red	na
chrUn	30003848	30003988	141	CarSat1	red	na
chrUn	30004010	30005593	1584	CarSat1	red	na
chrUn	30005614	30007201	1588	CarSat1	red	na
chrUn	30007223	30007934	712	CarSat1	red	na
chrUn	30008092	30009331	1240	CarSat1	red	na
chrUn	30009369	30010250	882	CarSat1	red	na
chrUn	30010245	30011791	1547	SAT1_CF	blue	na
chrUn	30011833	30013933	2101	SAT1_CF	blue	na
chrUn	30013968	30014190	223	SAT1_CF	blue	na
chrUn	30014202	30014808	607	SAT1_CF	blue	na
chrUn	30014819	30015364	546	SAT1_CF	blue	na
chrUn	30015377	30016345	969	SAT1_CF	blue	na
chrUn	30016389	30016911	523	SAT1_CF	blue	na
chrUn	30023208	30026906	3699	SAT1_CF	blue	na
chrUn	30026950	30028151	1202	SAT1_CF	blue	na
chrUn	30028191	30028889	699	SAT1_CF	blue	na
chrUn	30028931	30031236	2306	SAT1_CF	blue	na
chrUn	30031241	30032651	1411	CarSat1	red	na
chrUn	30297091	30297984	894	Sat21CF	na	na
chrUn	30297150	30297941	792	Sat18CF	na	na
chrUn	30336964	30337317	354	SAT1_CF	blue	na
chrUn	30337359	30337991	633	SAT1_CF	blue	na
chrUn	30338011	30338114	104	SAT1_CF	blue	na
chrUn	30338132	30338682	551	SAT1_CF	blue	na
chrUn	30338693	30339322	630	SAT1_CF	blue	na
chrUn	30339334	30339902	569	SAT1_CF	blue	na
chrUn	30339915	30340677	763	SAT1_CF	blue	na
chrUn	30340689	30341303	615	SAT1_CF	blue	na
chrUn	30341353	30342488	1136	SAT1_CF	blue	na
chrUn	30342510	30343141	632	SAT1_CF	blue	na
chrUn	30343167	30343270	104	SAT1_CF	blue	na
chrUn	30343283	30345043	1761	SAT1_CF	blue	na
chrUn	30345114	30345657	544	SAT1_CF	blue	na
chrUn	30345703	30346249	547	SAT1_CF	blue	na
chrUn	30346328	30347953	1626	SAT1_CF	blue	na
chrUn	30347976	30348651	676	SAT1_CF	blue	na

chrUn	30348673	30349175	503	SAT1_CF	blue	na
chrUn	30349214	30349614	401	SAT1_CF	blue	na
chrUn	30349627	30350173	547	SAT1_CF	blue	na
chrUn	30350233	30350378	146	SAT1_CF	blue	na
chrUn	30350417	30351069	653	SAT1_CF	blue	na
chrUn	30351081	30351763	683	SAT1_CF	blue	na
chrUn	30351776	30352667	892	SAT1_CF	blue	na
chrUn	30352679	30353358	680	SAT1_CF	blue	na
chrUn	30353370	30354528	1159	SAT1_CF	blue	na
chrUn	30354600	30355956	1357	SAT1_CF	blue	na
chrUn	30355970	30356445	476	SAT1_CF	blue	na
chrUn	30356456	30357105	650	SAT1_CF	blue	na
chrUn	30357160	30358145	986	SAT1_CF	blue	na
chrUn	30358156	30358776	621	SAT1_CF	blue	na
chrUn	30358806	30358900	95	SAT1_CF	blue	na
chrUn	30358918	30359439	522	SAT1_CF	blue	na
chrUn	30359507	30360842	1336	SAT1_CF	blue	na
chrUn	30360886	30362216	1331	SAT1_CF	blue	na
chrUn	30362228	30362781	554	SAT1_CF	blue	na
chrUn	30362805	30362895	91	SAT1_CF	blue	na
chrUn	30362922	30363443	522	SAT1_CF	blue	na
chrUn	30363464	30363839	376	SAT1_CF	blue	na
chrUn	30363872	30364381	510	SAT1_CF	blue	na
chrUn	30364497	30365113	617	SAT1_CF	blue	na
chrUn	30365131	30365684	554	SAT1_CF	blue	na
chrUn	30365705	30365804	100	SAT1_CF	blue	na
chrUn	30365833	30366326	494	SAT1_CF	blue	na
chrUn	30366361	30366528	168	SAT1_CF	blue	na
chrUn	30366580	30367067	488	SAT1_CF	blue	na
chrUn	30367147	30367820	674	SAT1_CF	blue	na
chrUn	30367833	30368362	530	SAT1_CF	blue	na
chrUn	30368407	30369464	1058	SAT1_CF	blue	na
chrUn	30369487	30369556	70	SAT1_CF	blue	na
chrUn	30369571	30370123	553	SAT1_CF	blue	na
chrUn	30370188	30370427	240	SAT1_CF	blue	na
chrUn	30370442	30371647	1206	SAT1_CF	blue	na
chrUn	30371691	30372598	908	SAT1_CF	blue	na
chrUn	30372641	30373318	678	SAT1_CF	blue	na
chrUn	30373330	30373962	633	SAT1_CF	blue	na
chrUn	30373976	30374092	117	SAT1_CF	blue	na
chrUn	30374104	30374635	532	SAT1_CF	blue	na
chrUn	30374703	30375057	355	SAT1_CF	blue	na

chrUn	30375103	30375651	549	SAT1_CF	blue	na
chrUn	30375729	30376510	782	SAT1_CF	blue	na
chrUn	30376560	30377179	620	SAT1_CF	blue	na
chrUn	30377202	30377307	106	SAT1_CF	blue	na
chrUn	30377320	30377821	502	SAT1_CF	blue	na
chrUn	30377861	30378042	182	SAT1_CF	blue	na
chrUn	30378222	30379085	864	SAT1_CF	blue	na
chrUn	30379100	30380258	1159	SAT1_CF	blue	na
chrUn	30380271	30381033	763	SAT1_CF	blue	na
chrUn	30381046	30381528	483	SAT1_CF	blue	na
chrUn	30381588	30382486	899	SAT1_CF	blue	na
chrUn	30382553	30383518	966	SAT1_CF	blue	na
chrUn	30383561	30384994	1434	SAT1_CF	blue	na
chrUn	30385021	30385546	526	SAT1_CF	blue	na
chrUn	30385577	30386097	521	SAT1_CF	blue	na
chrUn	30386172	30386568	397	SAT1_CF	blue	na
chrUn	30386581	30388958	2378	SAT1_CF	blue	na
chrUn	30388970	30389449	480	SAT1_CF	blue	na
chrUn	30389471	30389577	107	SAT1_CF	blue	na
chrUn	30389590	30390294	705	SAT1_CF	blue	na
chrUn	30390309	30391502	1194	SAT1_CF	blue	na
chrUn	30391524	30391629	106	SAT1_CF	blue	na
chrUn	30391642	30392142	501	SAT1_CF	blue	na
chrUn	30392182	30392363	182	SAT1_CF	blue	na
chrUn	30392545	30393414	870	SAT1_CF	blue	na
chrUn	30393458	30395578	2121	SAT1_CF	blue	na
chrUn	30395620	30396484	865	SAT1_CF	blue	na
chrUn	30396496	30397126	631	SAT1_CF	blue	na
chrUn	30397183	30399101	1919	SAT1_CF	blue	na
chrUn	30399113	30399786	674	SAT1_CF	blue	na
chrUn	30399809	30400310	502	SAT1_CF	blue	na
chrUn	30400376	30400512	137	SAT1_CF	blue	na
chrUn	30400540	30401087	548	SAT1_CF	blue	na
chrUn	30401171	30401731	561	SAT1_CF	blue	na
chrUn	30401759	30402390	632	SAT1_CF	blue	na
chrUn	30402433	30403106	674	SAT1_CF	blue	na
chrUn	30403119	30403620	502	SAT1_CF	blue	na
chrUn	30403660	30404060	401	SAT1_CF	blue	na
chrUn	30404071	30404618	548	SAT1_CF	blue	na
chrUn	30404698	30405469	772	SAT1_CF	blue	na
chrUn	30405522	30406382	861	SAT1_CF	blue	na
chrUn	30406394	30407153	760	SAT1_CF	blue	na

chrUn	30407166	30407666	501	SAT1_CF	blue	na
chrUn	30407706	30408814	1109	SAT1_CF	blue	na
chrUn	30408825	30409327	503	SAT1_CF	blue	na
chrUn	30409380	30410818	1439	SAT1_CF	blue	na
chrUn	30410862	30411388	527	SAT1_CF	blue	na
chrUn	30411419	30412041	623	SAT1_CF	blue	na
chrUn	30412054	30412982	929	SAT1_CF	blue	na
chrUn	30412994	30413677	684	SAT1_CF	blue	na
chrUn	30413689	30414318	630	SAT1_CF	blue	na
chrUn	30414341	30414446	106	SAT1_CF	blue	na
chrUn	30414459	30416156	1698	SAT1_CF	blue	na
chrUn	30416169	30416279	111	SAT1_CF	blue	na
chrUn	30416297	30416814	518	SAT1_CF	blue	na
chrUn	30416831	30417015	185	SAT1_CF	blue	na
chrUn	30417196	30417398	203	SAT1_CF	blue	na
chrUn	30417441	30418050	610	SAT1_CF	blue	na
chrUn	30418070	30419228	1159	SAT1_CF	blue	na
chrUn	30419240	30419872	633	SAT1_CF	blue	na
chrUn	30419943	30420485	543	SAT1_CF	blue	na
chrUn	30420503	30420816	314	SAT1_CF	blue	na
chrUn	30420896	30421459	564	SAT1_CF	blue	na
chrUn	30421486	30422866	1381	SAT1_CF	blue	na
chrUn	30422878	30423436	559	SAT1_CF	blue	na
chrUn	30423458	30423585	128	SAT1_CF	blue	na
chrUn	30423634	30424309	676	SAT1_CF	blue	na
chrUn	30424321	30424861	541	SAT1_CF	blue	na
chrUn	30424886	30424984	99	SAT1_CF	blue	na
chrUn	30425114	30425660	547	SAT1_CF	blue	na
chrUn	30425737	30426597	861	SAT1_CF	blue	na
chrUn	30426609	30427128	520	SAT1_CF	blue	na
chrUn	30427145	30427191	47	SAT1_CF	blue	na
chrUn	30427235	30427981	747	SAT1_CF	blue	na
chrUn	30428008	30428509	502	SAT1_CF	blue	na
chrUn	30428596	30429484	889	SAT1_CF	blue	na
chrUn	30429502	30429784	283	SAT1_CF	blue	na
chrUn	30429827	30430370	544	SAT1_CF	blue	na
chrUn	30430412	30430959	548	SAT1_CF	blue	na
chrUn	30431016	30431535	520	SAT1_CF	blue	na
chrUn	30431607	30431971	365	SAT1_CF	blue	na
chrUn	30432020	30433098	1079	SAT1_CF	blue	na
chrUn	30433120	30433646	527	SAT1_CF	blue	na
chrUn	30433689	30434355	667	SAT1_CF	blue	na

chrUn	30434378	30434910	533	SAT1_CF	blue	na
chrUn	30434921	30435633	713	SAT1_CF	blue	na
chrUn	30435645	30436687	1043	SAT1_CF	blue	na
chrUn	30436876	30437708	833	SAT1_CF	blue	na
chrUn	30437751	30438297	547	SAT1_CF	blue	na
chrUn	30438318	30438427	110	SAT1_CF	blue	na
chrUn	30438439	30438761	323	SAT1_CF	blue	na
chrUn	30438840	30439558	719	SAT1_CF	blue	na
chrUn	30439570	30440330	761	SAT1_CF	blue	na
chrUn	30440344	30440844	501	SAT1_CF	blue	na
chrUn	30440884	30441584	701	SAT1_CF	blue	na
chrUn	30441664	30442227	564	SAT1_CF	blue	na
chrUn	30442254	30442886	633	SAT1_CF	blue	na
chrUn	30442929	30443605	677	SAT1_CF	blue	na
chrUn	30443617	30444284	668	SAT1_CF	blue	na
chrUn	30444311	30445013	703	SAT1_CF	blue	na
chrUn	30445078	30445627	550	SAT1_CF	blue	na
chrUn	30445706	30446282	577	SAT1_CF	blue	na
chrUn	30446305	30447187	883	SAT1_CF	blue	na
chrUn	30447199	30447880	682	SAT1_CF	blue	na
chrUn	30447893	30448394	502	SAT1_CF	blue	na
chrUn	30448434	30448835	402	SAT1_CF	blue	na
chrUn	30448846	30449390	545	SAT1_CF	blue	na
chrUn	30449415	30449592	178	SAT1_CF	blue	na
chrUn	30449619	30450453	835	SAT1_CF	blue	na
chrUn	30450484	30451004	521	SAT1_CF	blue	na
chrUn	30451120	30451475	356	SAT1_CF	blue	na
chrUn	30451488	30452456	969	SAT1_CF	blue	na
chrUn	30452499	30453865	1367	SAT1_CF	blue	na
chrUn	30453877	30454346	470	SAT1_CF	blue	na
chrUn	30454368	30454474	107	SAT1_CF	blue	na
chrUn	30454487	30454987	501	SAT1_CF	blue	na
chrUn	30455032	30455189	158	SAT1_CF	blue	na
chrUn	30455222	30455729	508	SAT1_CF	blue	na
chrUn	30455777	30456445	669	SAT1_CF	blue	na
chrUn	30456468	30457171	704	SAT1_CF	blue	na
chrUn	30457199	30457745	547	SAT1_CF	blue	na
chrUn	30457829	30458389	561	SAT1_CF	blue	na
chrUn	30458435	30460244	1810	SAT1_CF	blue	na
chrUn	30460255	30460935	681	SAT1_CF	blue	na
chrUn	30460947	30461629	683	SAT1_CF	blue	na
chrUn	30461642	30462496	855	SAT1_CF	blue	na

chrUn	30462508	30463203	696	SAT1_CF	blue	na
chrUn	30463214	30463928	715	SAT1_CF	blue	na
chrUn	30463947	30464930	984	SAT1_CF	blue	na
chrUn	30464973	30465638	666	SAT1_CF	blue	na
chrUn	30465660	30466867	1208	SAT1_CF	blue	na
chrUn	30466883	30468754	1872	SAT1_CF	blue	na
chrUn	30468765	30469381	617	SAT1_CF	blue	na
chrUn	30469396	30471144	1749	SAT1_CF	blue	na
chrUn	30471166	30471668	503	SAT1_CF	blue	na
chrUn	30471734	30471870	137	SAT1_CF	blue	na
chrUn	30471898	30473089	1192	SAT1_CF	blue	na
chrUn	30473114	30473746	633	SAT1_CF	blue	na
chrUn	30473789	30474454	666	SAT1_CF	blue	na
chrUn	30474477	30475159	683	SAT1_CF	blue	na
chrUn	30475179	30475673	495	SAT1_CF	blue	na
chrUn	30475760	30476277	518	SAT1_CF	blue	na
chrUn	30620736	30621276	541	SAT1_CF	blue	na
chrUn	30621289	30621790	502	SAT1_CF	blue	na
chrUn	30621806	30622564	759	SAT1_CF	blue	na
chrUn	30622576	30623412	837	SAT1_CF	blue	na
chrUn	30623593	30623801	209	SAT1_CF	blue	na
chrUn	30623814	30625183	1370	SAT1_CF	blue	na
chrUn	30625196	30625867	672	SAT1_CF	blue	na
chrUn	30625912	30626544	633	SAT1_CF	blue	na
chrUn	30626572	30627805	1234	SAT1_CF	blue	na
chrUn	30627817	30629083	1267	SAT1_CF	blue	na
chrUn	30629113	30629632	520	SAT1_CF	blue	na
chrUn	30629646	30630336	691	SAT1_CF	blue	na
chrUn	30630348	30630456	109	SAT1_CF	blue	na
chrUn	30630476	30632774	2299	SAT1_CF	blue	na
chrUn	30632786	30633103	318	SAT1_CF	blue	na
chrUn	30633176	30633746	571	SAT1_CF	blue	na
chrUn	30633765	30635054	1290	SAT1_CF	blue	na
chrUn	30635097	30635726	630	SAT1_CF	blue	na
chrUn	30635754	30636947	1194	SAT1_CF	blue	na
chrUn	30636975	30637132	158	SAT1_CF	blue	na
chrUn	30637177	30637679	503	SAT1_CF	blue	na
chrUn	30637701	30638333	633	SAT1_CF	blue	na
chrUn	30638346	30638848	503	SAT1_CF	blue	na
chrUn	30638890	30640338	1449	SAT1_CF	blue	na
chrUn	30640349	30642219	1871	SAT1_CF	blue	na
chrUn	30642235	30643441	1207	SAT1_CF	blue	na

chrUn	30643463	30644128	666	SAT1_CF	blue	na
chrUn	30644171	30646667	2497	SAT1_CF	blue	na
chrUn	30646679	30647533	855	SAT1_CF	blue	na
chrUn	30647546	30648892	1347	SAT1_CF	blue	na
chrUn	30648989	30649971	983	SAT1_CF	blue	na
chrUn	30650018	30650546	529	SAT1_CF	blue	na
chrUn	30650559	30650665	107	SAT1_CF	blue	na
chrUn	30650688	30651262	575	SAT1_CF	blue	na
chrUn	30651274	30652036	763	SAT1_CF	blue	na
chrUn	30652050	30652699	650	SAT1_CF	blue	na
chrUn	30652710	30652785	76	SAT1_CF	blue	na
chrUn	30652806	30653358	553	SAT1_CF	blue	na
chrUn	30653370	30656126	2757	SAT1_CF	blue	na
chrUn	30656166	30656665	500	SAT1_CF	blue	na
chrUn	30656679	30657362	684	SAT1_CF	blue	na
chrUn	30657374	30658048	675	SAT1_CF	blue	na
chrUn	30658092	30660697	2606	SAT1_CF	blue	na
chrUn	30660740	30660905	166	SAT1_CF	blue	na
chrUn	30661088	30661273	186	SAT1_CF	blue	na
chrUn	30661290	30661810	521	SAT1_CF	blue	na
chrUn	30661823	30661931	109	SAT1_CF	blue	na
chrUn	30661951	30662510	560	SAT1_CF	blue	na
chrUn	30662522	30663197	676	SAT1_CF	blue	na
chrUn	30663241	30664205	965	SAT1_CF	blue	na
chrUn	30664321	30664717	397	SAT1_CF	blue	na
chrUn	30664785	30665305	521	SAT1_CF	blue	na
chrUn	30665318	30665389	72	SAT1_CF	blue	na
chrUn	30665446	30666000	555	SAT1_CF	blue	na
chrUn	30666012	30666772	761	SAT1_CF	blue	na
chrUn	30666786	30667459	674	SAT1_CF	blue	na
chrUn	30667502	30669130	1629	SAT1_CF	blue	na
chrUn	30669211	30669376	166	SAT1_CF	blue	na
chrUn	30669558	30669990	433	SAT1_CF	blue	na
chrUn	30670032	30670849	818	SAT1_CF	blue	na
chrUn	30670930	30671479	550	SAT1_CF	blue	na
chrUn	30671493	30672324	832	SAT1_CF	blue	na
chrUn	30672337	30672444	108	SAT1_CF	blue	na
chrUn	30672466	30673040	575	SAT1_CF	blue	na
chrUn	30673052	30675238	2187	SAT1_CF	blue	na
chrUn	30675259	30675772	514	SAT1_CF	blue	na
chrUn	30675792	30676474	683	SAT1_CF	blue	na
chrUn	30676486	30678491	2006	SAT1_CF	blue	na

chrUn	30678556	30679211	656	SAT1_CF	blue	na
chrUn	30679222	30679762	541	SAT1_CF	blue	na
chrUn	30679776	30680458	683	SAT1_CF	blue	na
chrUn	30680470	30681150	681	SAT1_CF	blue	na
chrUn	30681167	30681762	596	SAT1_CF	blue	na
chrUn	30681774	30683380	1607	SAT1_CF	blue	na
chrUn	30683406	30684152	747	SAT1_CF	blue	na
chrUn	30684164	30685498	1335	SAT1_CF	blue	na
chrUn	30685568	30686103	536	SAT1_CF	blue	na
chrUn	30686185	30686708	524	SAT1_CF	blue	na
chrUn	30686723	30686859	137	SAT1_CF	blue	na
chrUn	30686925	30688768	1844	SAT1_CF	blue	na
chrUn	30688814	30689120	307	SAT1_CF	blue	na
chrUn	30689199	30690299	1101	SAT1_CF	blue	na
chrUn	30690360	30691544	1185	SAT1_CF	blue	na
chrUn	30691601	30692233	633	SAT1_CF	blue	na
chrUn	30692245	30692848	604	SAT1_CF	blue	na
chrUn	30692861	30692967	107	SAT1_CF	blue	na
chrUn	30692988	30693540	553	SAT1_CF	blue	na
chrUn	30693552	30694417	866	SAT1_CF	blue	na
chrUn	30694459	30695979	1521	SAT1_CF	blue	na
chrUn	30695996	30696603	608	SAT1_CF	blue	na
chrUn	30696625	30697319	695	SAT1_CF	blue	na
chrUn	30697360	30699157	1798	SAT1_CF	blue	na
chrUn	30699170	30699930	761	SAT1_CF	blue	na
chrUn	30699948	30701599	1652	SAT1_CF	blue	na
chrUn	30701611	30702977	1367	SAT1_CF	blue	na
chrUn	30703042	30703604	563	SAT1_CF	blue	na
chrUn	30703647	30704200	554	SAT1_CF	blue	na
chrUn	30704211	30704821	611	SAT1_CF	blue	na
chrUn	30704844	30705510	667	SAT1_CF	blue	na
chrUn	30705553	30706079	527	SAT1_CF	blue	na
chrUn	30706100	30706185	86	SAT1_CF	blue	na
chrUn	30706212	30706776	565	SAT1_CF	blue	na
chrUn	30706834	30707355	522	SAT1_CF	blue	na
chrUn	30707366	30707909	544	SAT1_CF	blue	na
chrUn	30707920	30708238	319	SAT1_CF	blue	na
chrUn	30708291	30708792	502	SAT1_CF	blue	na
chrUn	30708805	30708906	102	SAT1_CF	blue	na
chrUn	30708934	30709644	711	SAT1_CF	blue	na
chrUn	30709656	30710337	682	SAT1_CF	blue	na
chrUn	30710380	30710919	540	SAT1_CF	blue	na

chrUn	30710969	30711513	545	SAT1_CF	blue	na
chrUn	30711551	30711898	348	SAT1_CF	blue	na
chrUn	30711940	30712607	668	SAT1_CF	blue	na
chrUn	30712673	30713751	1079	SAT1_CF	blue	na
chrUn	30713766	30713923	158	SAT1_CF	blue	na
chrUn	30713968	30715188	1221	SAT1_CF	blue	na
chrUn	30715220	30715723	504	SAT1_CF	blue	na
chrUn	30715755	30715891	137	SAT1_CF	blue	na
chrUn	30715957	30716455	499	SAT1_CF	blue	na
chrUn	30716473	30716584	112	SAT1_CF	blue	na
chrUn	30716597	30717151	555	SAT1_CF	blue	na
chrUn	30717163	30717840	678	SAT1_CF	blue	na
chrUn	30717937	30718478	542	SAT1_CF	blue	na
chrUn	30718503	30719380	878	SAT1_CF	blue	na
chrUn	30719393	30720506	1114	SAT1_CF	blue	na
chrUn	30720527	30721051	525	SAT1_CF	blue	na
chrUn	30721096	30721459	364	SAT1_CF	blue	na
chrUn	30721531	30722050	520	SAT1_CF	blue	na
chrUn	30722107	30722654	548	SAT1_CF	blue	na
chrUn	30722696	30724354	1659	SAT1_CF	blue	na
chrUn	30724535	30725373	839	SAT1_CF	blue	na
chrUn	30725399	30726687	1289	SAT1_CF	blue	na
chrUn	30726699	30726838	140	SAT1_CF	blue	na
chrUn	30726883	30727382	500	SAT1_CF	blue	na
chrUn	30727395	30728619	1225	SAT1_CF	blue	na
chrUn	30728665	30728794	130	SAT1_CF	blue	na
chrUn	30728816	30729374	559	SAT1_CF	blue	na
chrUn	30729386	30730864	1479	SAT1_CF	blue	na
chrUn	30730881	30731434	554	SAT1_CF	blue	na
chrUn	30731446	30732374	929	SAT1_CF	blue	na
chrUn	30732387	30733033	647	SAT1_CF	blue	na
chrUn	30733045	30734545	1501	SAT1_CF	blue	na
chrUn	30734631	30735126	496	SAT1_CF	blue	na
chrUn	30735146	30736761	1616	SAT1_CF	blue	na
chrUn	30736777	30736855	79	SAT1_CF	blue	na
chrUn	30736868	30737803	936	SAT1_CF	blue	na
chrUn	30737863	30738343	481	SAT1_CF	blue	na
chrUn	30738370	30739803	1434	SAT1_CF	blue	na
chrUn	30739846	30741882	2037	SAT1_CF	blue	na
chrUn	30741942	30742424	483	SAT1_CF	blue	na
chrUn	30742437	30743112	676	SAT1_CF	blue	na
chrUn	30743127	30744790	1664	SAT1_CF	blue	na

chrUn	30744802	30746261	1460	SAT1_CF	blue	na
chrUn	30746292	30746798	507	SAT1_CF	blue	na
chrUn	30746887	30749012	2126	SAT1_CF	blue	na
chrUn	30749030	30749835	806	SAT1_CF	blue	na
chrUn	30749865	30751985	2121	SAT1_CF	blue	na
chrUn	30752165	30752389	225	SAT1_CF	blue	na
chrUn	30752401	30753077	677	SAT1_CF	blue	na
chrUn	30753120	30754121	1002	SAT1_CF	blue	na
chrUn	30754153	30755083	931	SAT1_CF	blue	na
chrUn	30755146	30755619	474	SAT1_CF	blue	na
chrUn	30755639	30756049	411	SAT1_CF	blue	na
chrUn	30756241	30757321	1081	SAT1_CF	blue	na
chrUn	30757373	30757893	521	SAT1_CF	blue	na
chrUn	30757924	30758117	194	SAT1_CF	blue	na
chrUn	30758145	30758884	740	SAT1_CF	blue	na
chrUn	30790907	30792110	1204	Sat2CF	purple	na
chrUn	30791384	30792195	812	Sat2CF	purple	na
chrUn	30809147	30810396	1250	Sat2CF	purple	na
chrUn	30856924	30858082	1159	Sat2CF	purple	na
chrUn	30876352	30877530	1179	Sat2CF	purple	na
chrUn	30876482	30877501	1020	Sat2CF	purple	na
chrUn	30877372	30877573	202	Sat2CF	purple	na
chrUn	30886439	30886770	332	Sat2CF	purple	na
chrUn	30886480	30886995	516	Sat2CF	purple	na
chrUn	30886866	30887043	178	Sat2CF	purple	na
chrUn	30923008	30923676	669	Sat2CF	purple	na
chrUn	30923079	30923935	857	Sat2CF	purple	na
chrUn	30923839	30923946	108	Sat2CF	purple	na
chrUn	30941822	30942558	737	Sat2CF	purple	na
chrUn	30941824	30942484	661	Sat2CF	purple	na
chrUn	30964966	30965918	953	Sat2CF	purple	na
chrUn	30965046	30965783	738	Sat2CF	purple	na
chrUn	30984111	30984288	178	Sat2CF	purple	na
chrUn	30984159	30985448	1290	Sat2CF	purple	na
chrUn	31041162	31041331	170	SAT1_CF	blue	na
chrUn	31058639	31060120	1482	CarSat1	red	na
chrUn	31060150	31060997	848	CarSat1	red	na
chrUn	31061018	31062610	1593	CarSat1	red	na
chrUn	31065218	31065395	178	Sat2CF	purple	na
chrUn	31065237	31066415	1179	Sat2CF	purple	na
chrUn	31065266	31066285	1020	Sat2CF	purple	na
chrUn	31078533	31078883	351	Sat2CF	purple	na

chrUn	31078570	31079419	850	Sat2CF	purple	na
chrUn	31094301	31094478	178	Sat2CF	purple	na
chrUn	31094349	31094888	540	Sat2CF	purple	na
chrUn	31094784	31094900	117	Sat2CF	purple	na
chrUn	31104092	31104828	737	Sat2CF	purple	na
chrUn	31104167	31105298	1132	Sat2CF	purple	na
chrUn	31123443	31124558	1116	Sat2CF	purple	na
chrUn	31137521	31138676	1156	Sat2CF	purple	na
chrUn	31161756	31162458	703	CarSat1	red	na
chrUn	31162480	31163336	857	CarSat1	red	na
chrUn	31163357	31164135	779	CarSat1	red	na
chrUn	31164153	31164592	440	CarSat1	red	na
chrUn	31164599	31165607	1009	SAT1_CF	blue	na
chrUn	31165602	31166326	725	CarSat1	red	na
chrUn	31166358	31166784	427	CarSat1	red	na
chrUn	31168134	31168628	495	SAT1_CF	blue	na
chrUn	31168648	31170012	1365	SAT1_CF	blue	na
chrUn	31170058	31170576	519	SAT1_CF	blue	na
chrUn	31170615	31170760	146	SAT1_CF	blue	na
chrUn	31170820	31171369	550	SAT1_CF	blue	na
chrUn	31171412	31171782	371	SAT1_CF	blue	na
chrUn	31171838	31173705	1868	SAT1_CF	blue	na
chrUn	31173750	31174970	1221	SAT1_CF	blue	na
chrUn	31175051	31175575	525	SAT1_CF	blue	na
chrUn	31175590	31175747	158	SAT1_CF	blue	na
chrUn	31175773	31176948	1176	SAT1_CF	blue	na
chrUn	31176961	31177792	832	SAT1_CF	blue	na
chrUn	31177835	31178345	511	SAT1_CF	blue	na
chrUn	31178373	31178509	137	SAT1_CF	blue	na
chrUn	31178575	31179076	502	SAT1_CF	blue	na
chrUn	31179088	31179763	676	SAT1_CF	blue	na
chrUn	31179805	31180972	1168	SAT1_CF	blue	na
chrUn	31181042	31181515	474	SAT1_CF	blue	na
chrUn	31181528	31182123	596	SAT1_CF	blue	na
chrUn	31182157	31183950	1794	SAT1_CF	blue	na
chrUn	31183994	31184954	961	SAT1_CF	blue	na
chrUn	31185014	31185943	930	SAT1_CF	blue	na
chrUn	31185956	31186628	673	SAT1_CF	blue	na
chrUn	31186640	31186807	168	SAT1_CF	blue	na
chrUn	31186820	31187401	582	SAT1_CF	blue	na
chrUn	31187445	31187971	527	SAT1_CF	blue	na
chrUn	31188014	31188568	555	SAT1_CF	blue	na

chrUn	31188580	31189350	771	SAT1_CF	blue	na
chrUn	31189370	31189855	486	SAT1_CF	blue	na
chrUn	31189893	31190393	501	SAT1_CF	blue	na
chrUn	31190405	31191090	686	SAT1_CF	blue	na
chrUn	31191102	31191209	108	SAT1_CF	blue	na
chrUn	31191229	31192738	1510	SAT1_CF	blue	na
chrUn	31192798	31193280	483	SAT1_CF	blue	na
chrUn	31193307	31194876	1570	SAT1_CF	blue	na
chrUn	31194955	31195869	915	SAT1_CF	blue	na
chrUn	31195956	31196456	501	SAT1_CF	blue	na
chrUn	31196469	31198648	2180	SAT1_CF	blue	na
chrUn	31198691	31199197	507	SAT1_CF	blue	na
chrUn	31199230	31199366	137	SAT1_CF	blue	na
chrUn	31199413	31200972	1560	SAT1_CF	blue	na
chrUn	31201021	31201615	595	SAT1_CF	blue	na
chrUn	31203200	31203773	574	SAT1_CF	blue	na
chrUn	31203813	31204307	495	SAT1_CF	blue	na
chrUn	31204327	31204433	107	SAT1_CF	blue	na
chrUn	31204456	31206276	1821	SAT1_CF	blue	na
chrUn	31206320	31207512	1193	SAT1_CF	blue	na
chrUn	31207540	31207676	137	SAT1_CF	blue	na
chrUn	31207723	31208244	522	SAT1_CF	blue	na
chrUn	31208266	31209156	891	SAT1_CF	blue	na
chrUn	31209233	31209777	545	SAT1_CF	blue	na
chrUn	31209819	31210644	826	SAT1_CF	blue	na
chrUn	31210655	31211147	493	SAT1_CF	blue	na
chrUn	31211161	31213515	2355	SAT1_CF	blue	na
chrUn	31213643	31213791	149	SAT1_CF	blue	na
chrUn	31213805	31215397	1593	SAT1_CF	blue	na
chrUn	31215409	31216263	855	SAT1_CF	blue	na
chrUn	31216295	31216817	523	SAT1_CF	blue	na
chrUn	31216907	31217124	218	SAT1_CF	blue	na
chrUn	31217135	31217913	779	SAT1_CF	blue	na
chrUn	31217944	31218472	529	SAT1_CF	blue	na
chrUn	31218485	31218591	107	SAT1_CF	blue	na
chrUn	31218614	31219188	575	SAT1_CF	blue	na
chrUn	31219200	31219962	763	SAT1_CF	blue	na
chrUn	31219976	31220621	646	SAT1_CF	blue	na
chrUn	31220633	31221964	1332	SAT1_CF	blue	na
chrUn	31222008	31222978	971	SAT1_CF	blue	na
chrUn	31223020	31223185	166	SAT1_CF	blue	na
chrUn	31223367	31223552	186	SAT1_CF	blue	na

chrUn	31223569	31224089	521	SAT1_CF	blue	na
chrUn	31224102	31224210	109	SAT1_CF	blue	na
chrUn	31224230	31225661	1432	SAT1_CF	blue	na
chrUn	31225703	31226246	544	SAT1_CF	blue	na
chrUn	31226283	31227544	1262	SAT1_CF	blue	na
chrUn	31227597	31228098	502	SAT1_CF	blue	na
chrUn	31228125	31228869	745	SAT1_CF	blue	na
chrUn	31228881	31229555	675	SAT1_CF	blue	na
chrUn	31229598	31230089	492	SAT1_CF	blue	na
chrUn	31230169	31231126	958	SAT1_CF	blue	na
chrUn	31231166	31232348	1183	SAT1_CF	blue	na
chrUn	31232362	31233007	646	SAT1_CF	blue	na
chrUn	31233021	31233652	632	SAT1_CF	blue	na
chrUn	31233695	31234240	546	SAT1_CF	blue	na
chrUn	31234268	31234404	137	SAT1_CF	blue	na
chrUn	31234470	31234971	502	SAT1_CF	blue	na
chrUn	31234997	31235661	665	SAT1_CF	blue	na
chrUn	31235709	31236234	526	SAT1_CF	blue	na
chrUn	31236298	31236844	547	SAT1_CF	blue	na
chrUn	31236857	31236963	107	SAT1_CF	blue	na
chrUn	31236986	31237560	575	SAT1_CF	blue	na
chrUn	31237572	31238256	685	SAT1_CF	blue	na
chrUn	31238269	31238914	646	SAT1_CF	blue	na
chrUn	31238926	31240256	1331	SAT1_CF	blue	na
chrUn	31240300	31241681	1382	SAT1_CF	blue	na
chrUn	31241721	31242222	502	SAT1_CF	blue	na
chrUn	31242236	31244265	2030	SAT1_CF	blue	na
chrUn	31244284	31244954	671	SAT1_CF	blue	na
chrUn	31244997	31247094	2098	SAT1_CF	blue	na
chrUn	31247174	31247680	507	SAT1_CF	blue	na
chrUn	31247693	31248097	405	SAT1_CF	blue	na
chrUn	31248135	31248637	503	SAT1_CF	blue	na
chrUn	31248650	31248756	107	SAT1_CF	blue	na
chrUn	31248779	31249378	600	SAT1_CF	blue	na
chrUn	31249421	31249627	207	SAT1_CF	blue	na
chrUn	31249642	31250166	525	SAT1_CF	blue	na
chrUn	31250181	31250317	137	SAT1_CF	blue	na
chrUn	31250402	31252697	2296	SAT1_CF	blue	na
chrUn	31252709	31253023	315	SAT1_CF	blue	na
chrUn	31253105	31253624	520	SAT1_CF	blue	na
chrUn	31253680	31254227	548	SAT1_CF	blue	na
chrUn	31254269	31254807	539	SAT1_CF	blue	na

chrUn	31254819	31255246	428	SAT1_CF	blue	na
chrUn	31255333	31255833	501	SAT1_CF	blue	na
chrUn	31255850	31256437	588	SAT1_CF	blue	na
chrUn	31256449	31257091	643	SAT1_CF	blue	na
chrUn	31257288	31257533	246	SAT1_CF	blue	na
chrUn	31257575	31259271	1697	SAT1_CF	blue	na
chrUn	31259453	31259636	184	SAT1_CF	blue	na
chrUn	31259657	31261342	1686	SAT1_CF	blue	na
chrUn	31261357	31262059	703	SAT1_CF	blue	na
chrUn	31262072	31263297	1226	SAT1_CF	blue	na
chrUn	31263346	31263438	93	SAT1_CF	blue	na
chrUn	31263495	31264053	559	SAT1_CF	blue	na
chrUn	31264065	31265781	1717	SAT1_CF	blue	na
chrUn	31265807	31266441	635	SAT1_CF	blue	na
chrUn	31266454	31266809	356	SAT1_CF	blue	na
chrUn	31266926	31267396	471	SAT1_CF	blue	na
chrUn	31267475	31268547	1073	SAT1_CF	blue	na
chrUn	31268561	31269487	927	SAT1_CF	blue	na
chrUn	31269536	31270158	623	SAT1_CF	blue	na
chrUn	31270180	31270808	629	SAT1_CF	blue	na
chrUn	31270858	31271394	537	SAT1_CF	blue	na
chrUn	31271443	31272047	605	SAT1_CF	blue	na
chrUn	31272077	31272590	514	SAT1_CF	blue	na
chrUn	31272603	31273963	1361	SAT1_CF	blue	na
chrUn	31273978	31274679	702	SAT1_CF	blue	na
chrUn	31274691	31275376	686	SAT1_CF	blue	na
chrUn	31275389	31276063	675	SAT1_CF	blue	na
chrUn	31276107	31278040	1934	SAT1_CF	blue	na
chrUn	31278661	31279148	488	SAT1_CF	blue	na
chrUn	31279208	31279682	475	SAT1_CF	blue	na
chrUn	31279703	31280393	691	SAT1_CF	blue	na
chrUn	31280405	31280521	117	SAT1_CF	blue	na
chrUn	31280534	31281079	546	SAT1_CF	blue	na
chrUn	31281123	31283165	2043	SAT1_CF	blue	na
chrUn	31283220	31283698	479	SAT1_CF	blue	na
chrUn	31283711	31283816	106	SAT1_CF	blue	na
chrUn	31283839	31285014	1176	SAT1_CF	blue	na
chrUn	31285047	31285748	702	SAT1_CF	blue	na
chrUn	31285761	31286830	1070	SAT1_CF	blue	na
chrUn	31286842	31287403	562	SAT1_CF	blue	na
chrUn	31287417	31288098	682	SAT1_CF	blue	na
chrUn	31288111	31289381	1271	SAT1_CF	blue	na

chrUn	31289395	31290701	1307	SAT1_CF	blue	na
chrUn	31290713	31291515	803	SAT1_CF	blue	na
chrUn	31291545	31292714	1170	SAT1_CF	blue	na
chrUn	31292725	31293975	1251	SAT1_CF	blue	na
chrUn	31409066	31409998	933	Carsat2	orange	na
chrUn	31410084	31411191	1108	Carsat2	orange	na
chrUn	31423423	31423714	292	SAT1_CF	blue	na
chrUn	31423774	31424451	678	SAT1_CF	blue	na
chrUn	31424463	31425017	555	SAT1_CF	blue	na
chrUn	31425040	31425146	107	SAT1_CF	blue	na
chrUn	31425159	31425680	522	SAT1_CF	blue	na
chrUn	31425747	31426658	912	SAT1_CF	blue	na
chrUn	31426773	31427735	963	SAT1_CF	blue	na
chrUn	31427779	31429226	1448	SAT1_CF	blue	na
chrUn	31429249	31429279	31	SAT1_CF	blue	na
chrUn	31433887	31435232	1346	SAT1_CF	blue	na
chrUn	31435287	31435961	675	SAT1_CF	blue	na
chrUn	31435974	31437299	1326	SAT1_CF	blue	na
chrUn	31437322	31437427	106	SAT1_CF	blue	na
chrUn	31437440	31438121	682	SAT1_CF	blue	na
chrUn	31438134	31438654	521	SAT1_CF	blue	na
chrUn	31438722	31441216	2495	SAT1_CF	blue	na
chrUn	31441229	31441749	521	SAT1_CF	blue	na
chrUn	31441775	31441931	157	SAT1_CF	blue	na
chrUn	31441983	31444301	2319	SAT1_CF	blue	na
chrUn	31444323	31444897	575	SAT1_CF	blue	na
chrUn	31444960	31445026	67	SAT1_CF	blue	na
chrUn	31445039	31445583	545	SAT1_CF	blue	na
chrUn	31445667	31446174	508	SAT1_CF	blue	na
chrUn	31446217	31446875	659	SAT1_CF	blue	na
chrUn	31446887	31447427	541	SAT1_CF	blue	na
chrUn	31447471	31448261	791	SAT1_CF	blue	na
chrUn	31448304	31449164	861	SAT1_CF	blue	na
chrUn	31449176	31449690	515	SAT1_CF	blue	na
chrUn	31449713	31450548	836	SAT1_CF	blue	na
chrUn	31450575	31451076	502	SAT1_CF	blue	na
chrUn	31451163	31452898	1736	SAT1_CF	blue	na
chrUn	31452983	31453499	517	SAT1_CF	blue	na
chrUn	31453514	31453706	193	SAT1_CF	blue	na
chrUn	31453720	31455285	1566	SAT1_CF	blue	na
chrUn	31455297	31455966	670	SAT1_CF	blue	na
chrUn	31455991	31456493	503	SAT1_CF	blue	na

chrUn	31456546	31457081	536	SAT1_CF	blue	na
chrUn	31457096	31457763	668	SAT1_CF	blue	na
chrUn	31457775	31458390	616	SAT1_CF	blue	na
chrUn	31458407	31459805	1399	SAT1_CF	blue	na
chrUn	31459819	31460320	502	SAT1_CF	blue	na
chrUn	31460365	31461023	659	SAT1_CF	blue	na
chrUn	31461055	31462294	1240	SAT1_CF	blue	na
chrUn	31462320	31462807	488	SAT1_CF	blue	na
chrUn	31462839	31463496	658	SAT1_CF	blue	na
chrUn	31463562	31464229	668	SAT1_CF	blue	na
chrUn	31464271	31465199	929	SAT1_CF	blue	na
chrUn	31465249	31466512	1264	SAT1_CF	blue	na
chrUn	31466524	31467285	762	SAT1_CF	blue	na
chrUn	31467298	31467799	502	SAT1_CF	blue	na
chrUn	31467839	31468238	400	SAT1_CF	blue	na
chrUn	31468249	31468955	707	SAT1_CF	blue	na
chrUn	31469040	31469693	654	SAT1_CF	blue	na
chrUn	31469705	31470255	551	SAT1_CF	blue	na
chrUn	31470269	31470401	133	SAT1_CF	blue	na
chrUn	31470414	31471299	886	SAT1_CF	blue	na
chrUn	31471313	31471461	149	SAT1_CF	blue	na
chrUn	31471589	31472130	542	SAT1_CF	blue	na
chrUn	31472173	31472819	647	SAT1_CF	blue	na
chrUn	31472832	31473333	502	SAT1_CF	blue	na
chrUn	31473374	31474868	1495	SAT1_CF	blue	na
chrUn	31474890	31475416	527	SAT1_CF	blue	na
chrUn	31475459	31476125	667	SAT1_CF	blue	na
chrUn	31476148	31477272	1125	SAT1_CF	blue	na
chrUn	31477359	31477713	355	SAT1_CF	blue	na
chrUn	31477724	31478269	546	SAT1_CF	blue	na
chrUn	31478347	31478672	326	SAT1_CF	blue	na
chrUn	31478697	31480021	1325	SAT1_CF	blue	na
chrUn	31480044	31481168	1125	SAT1_CF	blue	na
chrUn	31481255	31481582	328	SAT1_CF	blue	na
chrUn	31481620	31482165	546	SAT1_CF	blue	na
chrUn	31482243	31482568	326	SAT1_CF	blue	na
chrUn	31482593	31483920	1328	SAT1_CF	blue	na
chrUn	31483932	31484614	683	SAT1_CF	blue	na
chrUn	31484627	31485109	483	SAT1_CF	blue	na
chrUn	31485194	31485330	137	SAT1_CF	blue	na
chrUn	31485345	31485870	526	SAT1_CF	blue	na
chrUn	31485913	31486473	561	SAT1_CF	blue	na

chrUn	31486517	31487059	543	SAT1_CF	blue	na
chrUn	31487156	31488850	1695	SAT1_CF	blue	na
chrUn	31488892	31490169	1278	SAT1_CF	blue	na
chrUn	31490213	31490723	511	SAT1_CF	blue	na
chrUn	31490764	31492360	1597	SAT1_CF	blue	na
chrUn	31492374	31493176	803	SAT1_CF	blue	na
chrUn	31493188	31493746	559	SAT1_CF	blue	na
chrUn	31493766	31493874	109	SAT1_CF	blue	na
chrUn	31493887	31494407	521	SAT1_CF	blue	na
chrUn	31494424	31494607	184	SAT1_CF	blue	na
chrUn	31494790	31494955	166	SAT1_CF	blue	na
chrUn	31494997	31496441	1445	SAT1_CF	blue	na
chrUn	31496521	31497612	1092	SAT1_CF	blue	na
chrUn	31497656	31498330	675	SAT1_CF	blue	na
chrUn	31498342	31499025	684	SAT1_CF	blue	na
chrUn	31499039	31499539	501	SAT1_CF	blue	na
chrUn	31499579	31500539	961	SAT1_CF	blue	na
chrUn	31500583	31500861	279	SAT1_CF	blue	na
chrUn	31500878	31502242	1365	SAT1_CF	blue	na
chrUn	31502256	31502748	493	SAT1_CF	blue	na
chrUn	31502759	31505138	2380	SAT1_CF	blue	na
chrUn	31505160	31505681	522	SAT1_CF	blue	na
chrUn	31505728	31505864	137	SAT1_CF	blue	na
chrUn	31505879	31507084	1206	SAT1_CF	blue	na
chrUn	31507112	31507741	630	SAT1_CF	blue	na
chrUn	31507784	31508450	667	SAT1_CF	blue	na
chrUn	31508473	31509155	683	SAT1_CF	blue	na
chrUn	31509175	31509334	160	SAT1_CF	blue	na
chrUn	31509558	31510269	712	SAT1_CF	blue	na
chrUn	31511667	31514025	2359	SAT1_CF	blue	na
chrUn	31514038	31514539	502	SAT1_CF	blue	na
chrUn	31514626	31515540	915	SAT1_CF	blue	na
chrUn	31515619	31517108	1490	SAT1_CF	blue	na
chrUn	31517127	31517697	571	SAT1_CF	blue	na
chrUn	31517757	31518157	401	SAT1_CF	blue	na
chrUn	31518205	31518751	547	SAT1_CF	blue	na
chrUn	31518831	31520517	1687	SAT1_CF	blue	na
chrUn	31520541	31521093	553	SAT1_CF	blue	na
chrUn	31521114	31521222	109	SAT1_CF	blue	na
chrUn	31521235	31521755	521	SAT1_CF	blue	na
chrUn	31521774	31522888	1115	SAT1_CF	blue	na
chrUn	31522936	31523610	675	SAT1_CF	blue	na

chrUn	31523623	31524374	752	SAT1_CF	blue	na
chrUn	31524396	31524976	581	SAT1_CF	blue	na
chrUn	31525059	31525567	509	SAT1_CF	blue	na
chrUn	31525645	31525951	307	SAT1_CF	blue	na
chrUn	31525994	31527319	1326	SAT1_CF	blue	na
chrUn	31527337	31529544	2208	SAT1_CF	blue	na
chrUn	31529562	31530063	502	SAT1_CF	blue	na
chrUn	31530123	31530800	678	SAT1_CF	blue	na
chrUn	31530821	31530929	109	SAT1_CF	blue	na
chrUn	31530942	31531415	474	SAT1_CF	blue	na
chrUn	31531475	31531655	181	SAT1_CF	blue	na
chrUn	31531836	31533527	1692	SAT1_CF	blue	na
chrUn	31533581	31534488	908	SAT1_CF	blue	na
chrUn	31534501	31535002	502	SAT1_CF	blue	na
chrUn	31535048	31536531	1484	SAT1_CF	blue	na
chrUn	31536542	31537082	541	SAT1_CF	blue	na
chrUn	31537125	31537801	677	SAT1_CF	blue	na
chrUn	31537813	31538315	503	SAT1_CF	blue	na
chrUn	31538360	31538517	158	SAT1_CF	blue	na
chrUn	31538568	31539054	487	SAT1_CF	blue	na
chrUn	31539085	31539652	568	SAT1_CF	blue	na
chrUn	31539676	31540539	864	SAT1_CF	blue	na
chrUn	31540551	31541106	556	SAT1_CF	blue	na
chrUn	31541127	31541234	108	SAT1_CF	blue	na
chrUn	31541247	31541746	500	SAT1_CF	blue	na
chrUn	31541833	31544539	2707	SAT1_CF	blue	na
chrUn	31544551	31545236	686	SAT1_CF	blue	na
chrUn	31545248	31546010	763	SAT1_CF	blue	na
chrUn	31546022	31547583	1562	SAT1_CF	blue	na
chrUn	31547680	31548357	678	SAT1_CF	blue	na
chrUn	31548369	31549038	670	SAT1_CF	blue	na
chrUn	31549065	31549565	501	SAT1_CF	blue	na
chrUn	31549605	31550868	1264	SAT1_CF	blue	na
chrUn	31550916	31551657	742	SAT1_CF	blue	na
chrUn	31686210	31686647	438	CarSat1	red	na
chrUn	31686666	31687384	719	CarSat1	red	na
chrUn	31687379	31688507	1129	SAT1_CF	blue	na
chrUn	31688551	31690760	2210	SAT1_CF	blue	na
chrUn	31690765	31690976	212	CarSat1	red	na
chrUn	31691168	31692160	993	CarSat1	red	na
chrUn	31692180	31692900	721	CarSat1	red	na
chrUn	31692918	31694428	1511	CarSat1	red	na

chrUn	31694512	31695304	793	CarSat1	red	na
chrUn	31695325	31695849	525	CarSat1	red	na
chrUn	31696249	31697431	1183	Sat2CF	purple	na
chrUn	31697323	31697502	180	Sat2CF	purple	na
chrUn	31712002	31712876	875	Sat2CF	purple	na
chrUn	31712078	31712801	724	Sat2CF	purple	na
chrUn	31724923	31726173	1251	Sat2CF	purple	na
chrUn	31725053	31726137	1085	Sat2CF	purple	na
chrUn	31729000	31730592	1593	CarSat1	red	na
chrUn	31730613	31731452	840	CarSat1	red	na
chrUn	31731480	31733654	2175	CarSat1	red	na
chrUn	31819961	31820327	367	CarSat1	red	na
chrUn	31820322	31821340	1019	SAT1_CF	blue	na
chrUn	31821368	31821994	627	SAT1_CF	blue	na
chrUn	31822042	31822717	676	SAT1_CF	blue	na
chrUn	31822729	31823333	605	SAT1_CF	blue	na
chrUn	31823346	31823848	503	SAT1_CF	blue	na
chrUn	31823884	31824069	186	SAT1_CF	blue	na
chrUn	31824250	31825085	836	SAT1_CF	blue	na
chrUn	31825097	31826313	1217	SAT1_CF	blue	na
chrUn	31826339	31826496	158	SAT1_CF	blue	na
chrUn	31826511	31827863	1353	SAT1_CF	blue	na
chrUn	31827886	31827991	106	SAT1_CF	blue	na
chrUn	31828004	31828484	481	SAT1_CF	blue	na
chrUn	31828540	31828728	189	SAT1_CF	blue	na
chrUn	31828908	31829110	203	SAT1_CF	blue	na
chrUn	31829152	31831367	2216	SAT1_CF	blue	na
chrUn	31831436	31832112	677	SAT1_CF	blue	na
chrUn	31832124	31833320	1197	SAT1_CF	blue	na
chrUn	31833408	31834291	884	SAT1_CF	blue	na
chrUn	31834324	31835458	1135	SAT1_CF	blue	na
chrUn	31835471	31835951	481	SAT1_CF	blue	na
chrUn	31836010	31836412	403	SAT1_CF	blue	na
chrUn	31836426	31836968	543	SAT1_CF	blue	na
chrUn	31837012	31837967	956	SAT1_CF	blue	na
chrUn	31838014	31838688	675	SAT1_CF	blue	na
chrUn	31838700	31839380	681	SAT1_CF	blue	na
chrUn	31839393	31840152	760	SAT1_CF	blue	na
chrUn	31840165	31841795	1631	SAT1_CF	blue	na
chrUn	31841839	31843130	1292	SAT1_CF	blue	na
chrUn	31843143	31843520	378	SAT1_CF	blue	na
chrUn	31843709	31844534	826	SAT1_CF	blue	na

chrUn	31844571	31845509	939	SAT1_CF	blue	na
chrUn	31845529	31846404	876	SAT1_CF	blue	na
chrUn	31846480	31847854	1375	SAT1_CF	blue	na
chrUn	31847881	31849084	1204	SAT1_CF	blue	na
chrUn	31849128	31849433	306	SAT1_CF	blue	na
chrUn	31849477	31850023	547	SAT1_CF	blue	na
chrUn	31850046	31850137	92	SAT1_CF	blue	na
chrUn	31850164	31850704	541	SAT1_CF	blue	na
chrUn	31850752	31852656	1905	SAT1_CF	blue	na
chrUn	31852669	31853066	398	SAT1_CF	blue	na
chrUn	31853108	31854176	1069	SAT1_CF	blue	na
chrUn	31854202	31854734	533	SAT1_CF	blue	na
chrUn	31854777	31855451	675	SAT1_CF	blue	na
chrUn	31855463	31857096	1634	SAT1_CF	blue	na
chrUn	31857109	31857225	117	SAT1_CF	blue	na
chrUn	31857237	31858032	796	SAT1_CF	blue	na
chrUn	31858048	31860018	1971	SAT1_CF	blue	na
chrUn	31860040	31860564	525	SAT1_CF	blue	na
chrUn	31860608	31861427	820	SAT1_CF	blue	na
chrUn	31861445	31861938	494	SAT1_CF	blue	na
chrUn	31861982	31862177	196	SAT1_CF	blue	na
chrUn	31862192	31863836	1645	SAT1_CF	blue	na
chrUn	31863906	31864764	859	SAT1_CF	blue	na
chrUn	31864776	31865357	582	SAT1_CF	blue	na
chrUn	31865437	31866906	1470	SAT1_CF	blue	na
chrUn	31866918	31867535	618	SAT1_CF	blue	na
chrUn	31867605	31868198	594	SAT1_CF	blue	na
chrUn	31868217	31868808	592	SAT1_CF	blue	na
chrUn	31868858	31869549	692	SAT1_CF	blue	na
chrUn	31869591	31870444	854	SAT1_CF	blue	na
chrUn	31870456	31871626	1171	SAT1_CF	blue	na
chrUn	31871700	31872321	622	SAT1_CF	blue	na
chrUn	31872344	31874631	2288	SAT1_CF	blue	na
chrUn	31874665	31876298	1634	SAT1_CF	blue	na
chrUn	31876321	31877554	1234	SAT1_CF	blue	na
chrUn	31877582	31878208	627	SAT1_CF	blue	na
chrUn	31878256	31878800	545	SAT1_CF	blue	na
chrUn	31878820	31878928	109	SAT1_CF	blue	na
chrUn	31878940	31879495	556	SAT1_CF	blue	na
chrUn	31879518	31879626	109	SAT1_CF	blue	na
chrUn	31879638	31880158	521	SAT1_CF	blue	na
chrUn	31880205	31881010	806	SAT1_CF	blue	na

chrUn	31881036	31882000	965	SAT1_CF	blue	na
chrUn	31882043	31882718	676	SAT1_CF	blue	na
chrUn	31882730	31883285	556	SAT1_CF	blue	na
chrUn	31883355	31883948	594	SAT1_CF	blue	na
chrUn	31883995	31884132	138	SAT1_CF	blue	na
chrUn	31884159	31886595	2437	SAT1_CF	blue	na
chrUn	31886665	31887211	547	SAT1_CF	blue	na
chrUn	31887223	31887847	625	SAT1_CF	blue	na
chrUn	31887873	31889508	1636	SAT1_CF	blue	na
chrUn	31889552	31890754	1203	SAT1_CF	blue	na
chrUn	31890774	31890883	110	SAT1_CF	blue	na
chrUn	31890895	31891436	542	SAT1_CF	blue	na
chrUn	31891479	31891645	167	SAT1_CF	blue	na
chrUn	31891664	31892377	714	SAT1_CF	blue	na
chrUn	31892389	31892974	586	SAT1_CF	blue	na
chrUn	31893017	31893364	348	SAT1_CF	blue	na
chrUn	31893402	31893946	545	SAT1_CF	blue	na
chrUn	31893988	31895537	1550	SAT1_CF	blue	na
chrUn	31895580	31896254	675	SAT1_CF	blue	na
chrUn	31896266	31896871	606	SAT1_CF	blue	na
chrUn	31896884	31898997	2114	SAT1_CF	blue	na
chrUn	31899021	31899926	906	SAT1_CF	blue	na
chrUn	31899938	31901134	1197	SAT1_CF	blue	na
chrUn	31901179	31902747	1569	SAT1_CF	blue	na
chrUn	31902771	31903676	906	SAT1_CF	blue	na
chrUn	31903688	31904904	1217	SAT1_CF	blue	na
chrUn	31904920	31905957	1038	SAT1_CF	blue	na
chrUn	31906006	31910552	4547	SAT1_CF	blue	na
chrUn	31910564	31911169	606	SAT1_CF	blue	na
chrUn	31911182	31911877	696	SAT1_CF	blue	na
chrUn	31911895	31912578	684	SAT1_CF	blue	na
chrUn	31912592	31913092	501	SAT1_CF	blue	na
chrUn	31913148	31913497	350	SAT1_CF	blue	na
chrUn	31913530	31914040	511	SAT1_CF	blue	na
chrUn	31914156	31914938	783	SAT1_CF	blue	na
chrUn	31914980	31915725	746	SAT1_CF	blue	na
chrUn	31915737	31917145	1409	SAT1_CF	blue	na
chrUn	31917158	31918049	892	SAT1_CF	blue	na
chrUn	31918061	31919344	1284	SAT1_CF	blue	na
chrUn	31919389	31919928	540	SAT1_CF	blue	na
chrUn	31919970	31920644	675	SAT1_CF	blue	na
chrUn	31920655	31921339	685	SAT1_CF	blue	na

chrUn	31921352	31921872	521	SAT1_CF	blue	na
chrUn	31921893	31923884	1992	SAT1_CF	blue	na
chrUn	31923927	31924602	676	SAT1_CF	blue	na
chrUn	31924614	31925453	840	SAT1_CF	blue	na
chrUn	31925466	31925968	503	SAT1_CF	blue	na
chrUn	31926004	31926191	188	SAT1_CF	blue	na
chrUn	31926369	31927204	836	SAT1_CF	blue	na
chrUn	31927216	31927899	684	SAT1_CF	blue	na
chrUn	31927913	31928415	503	SAT1_CF	blue	na
chrUn	31928455	31929936	1482	SAT1_CF	blue	na
chrUn	31929953	31930489	537	SAT1_CF	blue	na
chrUn	31930539	31930785	247	SAT1_CF	blue	na
chrUn	31930830	31931403	574	SAT1_CF	blue	na
chrUn	31931465	31932760	1296	SAT1_CF	blue	na
chrUn	31932802	31934178	1377	SAT1_CF	blue	na
chrUn	31934189	31934690	502	SAT1_CF	blue	na
chrUn	31934735	31934892	158	SAT1_CF	blue	na
chrUn	31934943	31935431	489	SAT1_CF	blue	na
chrUn	31935511	31936194	684	SAT1_CF	blue	na
chrUn	31936207	31938019	1813	SAT1_CF	blue	na
chrUn	31938032	31938542	511	SAT1_CF	blue	na
chrUn	31938556	31939592	1037	SAT1_CF	blue	na
chrUn	31992823	31993060	238	Sat7CF	lime	na
chrUn	31992854	31993158	305	Sat7CF	lime	na
chrUn	32056502	32057171	670	Sat7CF	lime	na
chrUn	32096534	32097710	1177	Sat2CF	purple	na
chrUn	32119131	32119330	200	Sat2CF	purple	na
chrUn	32119170	32120347	1178	Sat2CF	purple	na
chrUn	32154239	32154975	737	Sat2CF	purple	na
chrUn	32154241	32154901	661	Sat2CF	purple	na
chrUn	32175666	32176619	954	Sat2CF	purple	na
chrUn	32175746	32176484	739	Sat2CF	purple	na
chrUn	32194626	32195803	1178	Sat2CF	purple	na
chrUn	32292423	32292822	400	Sat7CF	lime	na
chrUn	32444289	32444918	630	SAT1_CF	blue	na
chrUn	32445128	32445283	156	SAT1_CF	blue	na
chrUn	32445309	32445821	513	SAT1_CF	blue	na
chrUn	32445850	32446510	661	SAT1_CF	blue	na
chrUn	32446556	32447934	1379	SAT1_CF	blue	na
chrUn	32448123	32448624	502	SAT1_CF	blue	na
chrUn	32448642	32449398	757	SAT1_CF	blue	na
chrUn	32449410	32450084	675	SAT1_CF	blue	na

chrUn	32450128	32452672	2545	SAT1_CF	blue	na
chrUn	32453128	32453733	606	SAT1_CF	blue	na
chrUn	32453933	32455273	1341	SAT1_CF	blue	na
chrUn	32455286	32456024	739	SAT1_CF	blue	na
chrUn	32456049	32457311	1263	SAT1_CF	blue	na
chrUn	32457324	32458007	684	SAT1_CF	blue	na
chrUn	32458019	32458778	760	SAT1_CF	blue	na
chrUn	32458792	32460235	1444	SAT1_CF	blue	na
chrUn	32460263	32460826	564	SAT1_CF	blue	na
chrUn	32460869	32461357	489	SAT1_CF	blue	na
chrUn	32461409	32461545	137	SAT1_CF	blue	na
chrUn	32461611	32462160	550	SAT1_CF	blue	na
chrUn	32462173	32462849	677	SAT1_CF	blue	na
chrUn	32462892	32463432	541	SAT1_CF	blue	na
chrUn	32463447	32464512	1066	SAT1_CF	blue	na
chrUn	32464528	32464930	403	SAT1_CF	blue	na
chrUn	32464970	32465472	503	SAT1_CF	blue	na
chrUn	32465484	32465593	110	SAT1_CF	blue	na
chrUn	32465614	32466244	631	SAT1_CF	blue	na
chrUn	32466357	32466863	507	SAT1_CF	blue	na
chrUn	32466877	32467281	405	SAT1_CF	blue	na
chrUn	32467319	32467821	503	SAT1_CF	blue	na
chrUn	32467833	32467970	138	SAT1_CF	blue	na
chrUn	32468013	32468715	703	SAT1_CF	blue	na
chrUn	32468745	32469946	1202	SAT1_CF	blue	na
chrUn	32469961	32471426	1466	SAT1_CF	blue	na
chrUn	32471469	32472943	1475	SAT1_CF	blue	na
chrUn	32473033	32473533	501	SAT1_CF	blue	na
chrUn	32473547	32474230	684	SAT1_CF	blue	na
chrUn	32474242	32477230	2989	SAT1_CF	blue	na
chrUn	32477310	32477966	657	SAT1_CF	blue	na
chrUn	32478028	32478658	631	SAT1_CF	blue	na
chrUn	32478670	32479352	683	SAT1_CF	blue	na
chrUn	32479364	32479964	601	SAT1_CF	blue	na
chrUn	32479976	32482399	2424	SAT1_CF	blue	na
chrUn	32482444	32483431	988	SAT1_CF	blue	na
chrUn	32483488	32484443	956	SAT1_CF	blue	na
chrUn	32484483	32484983	501	SAT1_CF	blue	na
chrUn	32485010	32485758	749	SAT1_CF	blue	na
chrUn	32485776	32486443	668	SAT1_CF	blue	na
chrUn	32486455	32487102	648	SAT1_CF	blue	na
chrUn	32487126	32487450	325	SAT1_CF	blue	na

chrUn	32487551	32488071	521	SAT1_CF	blue	na
chrUn	32488115	32488486	372	SAT1_CF	blue	na
chrUn	32488522	32489023	502	SAT1_CF	blue	na
chrUn	32489041	32489144	104	SAT1_CF	blue	na
chrUn	32489165	32489739	575	SAT1_CF	blue	na
chrUn	32489751	32490411	661	SAT1_CF	blue	na
chrUn	32490459	32491439	981	SAT1_CF	blue	na
chrUn	32491460	32492494	1035	SAT1_CF	blue	na
chrUn	32492514	32493034	521	SAT1_CF	blue	na
chrUn	32493047	32493719	673	SAT1_CF	blue	na
chrUn	32493768	32494269	502	SAT1_CF	blue	na
chrUn	32494282	32494966	685	SAT1_CF	blue	na
chrUn	32494978	32495831	854	SAT1_CF	blue	na
chrUn	32495873	32497601	1729	SAT1_CF	blue	na
chrUn	32497614	32498259	646	SAT1_CF	blue	na
chrUn	32498303	32499440	1138	SAT1_CF	blue	na
chrUn	32499506	32500007	502	SAT1_CF	blue	na
chrUn	32500019	32500127	109	SAT1_CF	blue	na
chrUn	32500150	32500705	556	SAT1_CF	blue	na
chrUn	32500717	32501395	679	SAT1_CF	blue	na
chrUn	32501440	32503069	1630	SAT1_CF	blue	na
chrUn	32503100	32503481	382	SAT1_CF	blue	na
chrUn	32503522	32504023	502	SAT1_CF	blue	na
chrUn	32504036	32504710	675	SAT1_CF	blue	na
chrUn	32504753	32505383	631	SAT1_CF	blue	na
chrUn	32505411	32506610	1200	SAT1_CF	blue	na
chrUn	32506667	32507727	1061	SAT1_CF	blue	na
chrUn	32507795	32508299	505	SAT1_CF	blue	na
chrUn	32508333	32511045	2713	SAT1_CF	blue	na
chrUn	32511126	32511685	560	SAT1_CF	blue	na
chrUn	32511696	32512310	615	SAT1_CF	blue	na
chrUn	32512322	32512997	676	SAT1_CF	blue	na
chrUn	32513040	32515077	2038	SAT1_CF	blue	na
chrUn	32515117	32515696	580	SAT1_CF	blue	na
chrUn	32515759	32516433	675	SAT1_CF	blue	na
chrUn	32516456	32517000	545	SAT1_CF	blue	na
chrUn	32517043	32518866	1824	SAT1_CF	blue	na
chrUn	32518885	32519398	514	SAT1_CF	blue	na
chrUn	32519420	32520100	681	SAT1_CF	blue	na
chrUn	32520112	32520221	110	SAT1_CF	blue	na
chrUn	32520241	32521041	801	SAT1_CF	blue	na
chrUn	32521053	32522384	1332	SAT1_CF	blue	na

chrUn	32522427	32523100	674	SAT1_CF	blue	na
chrUn	32523128	32525234	2107	SAT1_CF	blue	na
chrUn	32525291	32525826	536	SAT1_CF	blue	na
chrUn	32525906	32526415	510	SAT1_CF	blue	na
chrUn	32526443	32526600	158	SAT1_CF	blue	na
chrUn	32526645	32527147	503	SAT1_CF	blue	na
chrUn	32527159	32527231	73	SAT1_CF	blue	na
chrUn	32527288	32528575	1288	SAT1_CF	blue	na
chrUn	32528586	32528940	355	SAT1_CF	blue	na
chrUn	32529027	32529527	501	SAT1_CF	blue	na
chrUn	32529540	32529644	105	SAT1_CF	blue	na
chrUn	32529669	32531565	1897	SAT1_CF	blue	na
chrUn	32531645	32532938	1294	SAT1_CF	blue	na
chrUn	32533025	32533527	503	SAT1_CF	blue	na
chrUn	32533539	32533648	110	SAT1_CF	blue	na
chrUn	32533669	32534420	752	SAT1_CF	blue	na
chrUn	32534442	32534986	545	SAT1_CF	blue	na
chrUn	32535029	32537095	2067	SAT1_CF	blue	na
chrUn	32537107	32537783	677	SAT1_CF	blue	na
chrUn	32537795	32539866	2072	SAT1_CF	blue	na
chrUn	32539906	32540481	576	SAT1_CF	blue	na
chrUn	32540494	32540601	108	SAT1_CF	blue	na
chrUn	32540622	32541177	556	SAT1_CF	blue	na
chrUn	32541189	32541872	684	SAT1_CF	blue	na
chrUn	32541914	32542764	851	SAT1_CF	blue	na
chrUn	32542834	32544037	1204	SAT1_CF	blue	na
chrUn	32544081	32544739	659	SAT1_CF	blue	na
chrUn	32544757	32545259	503	SAT1_CF	blue	na
chrUn	32545292	32546693	1402	SAT1_CF	blue	na
chrUn	32546711	32547385	675	SAT1_CF	blue	na
chrUn	32547434	32549328	1895	SAT1_CF	blue	na
chrUn	32549381	32549882	502	SAT1_CF	blue	na
chrUn	32549909	32550693	785	SAT1_CF	blue	na
chrUn	32550719	32551266	548	SAT1_CF	blue	na
chrUn	32551309	32552306	998	SAT1_CF	blue	na
chrUn	32552348	32553303	956	SAT1_CF	blue	na
chrUn	32553346	32554498	1153	SAT1_CF	blue	na
chrUn	32554513	32555326	814	SAT1_CF	blue	na
chrUn	32555378	32556192	815	SAT1_CF	blue	na
chrUn	32557198	32558238	1041	CarSat1	red	na
chrUn	32558272	32558980	709	CarSat1	red	na
chrUn	32559004	32559940	937	CarSat1	red	na

chrUn	32559955	32560082	128	Carsat2	orange	na
chrUn	32559970	32560678	709	CarSat1	red	na
chrUn	32560693	32560820	128	Carsat2	orange	na
chrUn	32560708	32562151	1444	CarSat1	red	na
chrUn	32562166	32562292	127	Carsat2	orange	na
chrUn	32562181	32564366	2186	CarSat1	red	na
chrUn	32564377	32564507	131	Carsat2	orange	na
chrUn	32564396	32565823	1428	CarSat1	red	na
chrUn	32566965	32570321	3357	CarSat1	red	na
chrUn	32570345	32571285	941	CarSat1	red	na
chrUn	32571300	32571426	127	Carsat2	orange	na
chrUn	32571315	32572021	707	CarSat1	red	na
chrUn	32572051	32572759	709	CarSat1	red	na
chrUn	32572774	32572900	127	Carsat2	orange	na
chrUn	32572789	32574971	2183	CarSat1	red	na
chrUn	32574986	32575113	128	Carsat2	orange	na
chrUn	32575001	32575709	709	CarSat1	red	na
chrUn	32575739	32577187	1449	CarSat1	red	na
chrUn	32577217	32577924	708	CarSat1	red	na
chrUn	32577953	32579365	1413	CarSat1	red	na
chrUn	32579798	32581615	1818	CarSat1	red	na
chrUn	32581630	32581756	127	Carsat2	orange	na
chrUn	32581645	32583355	1711	CarSat1	red	na
chrUn	32583382	32584558	1177	CarSat1	red	na
chrUn	32584573	32584699	127	Carsat2	orange	na
chrUn	32584588	32585806	1219	CarSat1	red	na
chrUn	32585830	32587511	1682	CarSat1	red	na
chrUn	32587526	32587653	128	Carsat2	orange	na
chrUn	32587541	32588247	707	CarSat1	red	na
chrUn	32588277	32589498	1222	CarSat1	red	na
chrUn	32589522	32591712	2191	CarSat1	red	na
chrUn	32591736	32592453	718	CarSat1	red	na
chrUn	32592477	32593332	856	CarSat1	red	na
chrUn	32593780	32594892	1113	CarSat1	red	na
chrUn	32594922	32596368	1447	CarSat1	red	na
chrUn	32596398	32597105	708	CarSat1	red	na
chrUn	32597120	32597246	127	Carsat2	orange	na
chrUn	32597135	32597840	706	CarSat1	red	na
chrUn	32597855	32597980	126	Carsat2	orange	na
chrUn	32597870	32600048	2179	CarSat1	red	na
chrUn	32600078	32600784	707	CarSat1	red	na
chrUn	32600799	32600925	127	Carsat2	orange	na

chrUn	32600814	32602999	2186	CarSat1	red	na
chrUn	32603014	32603141	128	Carsat2	orange	na
chrUn	32603029	32604477	1449	CarSat1	red	na
chrUn	32604492	32604619	128	Carsat2	orange	na
chrUn	32604507	32605728	1222	CarSat1	red	na
chrUn	32605752	32606949	1198	CarSat1	red	na
chrUn	32606961	32607936	976	CarSat1	red	na
chrUn	32607960	32608857	898	CarSat1	red	na
chrUn	32610507	32613289	2783	CarSat1	red	na
chrUn	32613304	32613430	127	Carsat2	orange	na
chrUn	32613319	32615496	2178	CarSat1	red	na
chrUn	32615511	32615637	127	Carsat2	orange	na
chrUn	32615526	32617239	1714	CarSat1	red	na
chrUn	32617260	32618442	1183	CarSat1	red	na
chrUn	32618457	32618583	127	Carsat2	orange	na
chrUn	32618472	32619179	708	CarSat1	red	na
chrUn	32619209	32620182	974	CarSat1	red	na
chrUn	32620193	32621390	1198	CarSat1	red	na
chrUn	32621420	32622864	1445	CarSat1	red	na
chrUn	32622879	32623006	128	Carsat2	orange	na
chrUn	32622894	32623380	487	CarSat1	red	na
chrUn	32623404	32627797	4394	CarSat1	red	na
chrUn	32627809	32629484	1676	CarSat1	red	na
chrUn	32629500	32629627	128	Carsat2	orange	na
chrUn	32629515	32631220	1706	CarSat1	red	na
chrUn	32631232	32632428	1197	CarSat1	red	na
chrUn	32632443	32632569	127	Carsat2	orange	na
chrUn	32632458	32633017	560	CarSat1	red	na
chrUn	32635173	32636613	1441	CarSat1	red	na
chrUn	32636637	32638572	1936	CarSat1	red	na
chrUn	32638583	32639780	1198	CarSat1	red	na
chrUn	32639795	32639921	127	Carsat2	orange	na
chrUn	32639810	32640517	708	CarSat1	red	na
chrUn	32640532	32640659	128	Carsat2	orange	na
chrUn	32640547	32645451	4905	CarSat1	red	na
chrUn	32645475	32647415	1941	CarSat1	red	na
chrUn	32647442	32648396	955	CarSat1	red	na
chrUn	32648420	32649354	935	CarSat1	red	na
chrUn	32649384	32650092	709	CarSat1	red	na
chrUn	32650107	32650234	128	Carsat2	orange	na
chrUn	32650122	32652086	1965	CarSat1	red	na
chrUn	32652102	32652816	715	CarSat1	red	na

chrUn	32652840	32655035	2196	CarSat1	red	na
chrUn	32655067	32656503	1437	CarSat1	red	na
chrUn	32656519	32660649	4131	CarSat1	red	na
chrUn	32660670	32661381	712	CarSat1	red	na
chrUn	32661402	32662834	1433	CarSat1	red	na
chrUn	32663015	32664020	1006	CarSat1	red	na
chrUn	32664047	32665225	1179	CarSat1	red	na
chrUn	32665407	32668221	2815	CarSat1	red	na
chrUn	32668411	32669041	631	CarSat1	red	na
chrUn	32669074	32669769	696	CarSat1	red	na
chrUn	32669794	32671724	1931	CarSat1	red	na
chrUn	32671751	32673364	1614	CarSat1	red	na
chrUn	32788268	32788542	275	SAT1_CF	blue	na
chrUn	32788563	32789105	543	SAT1_CF	blue	na
chrUn	32789175	32790376	1202	SAT1_CF	blue	na
chrUn	32790420	32790726	307	SAT1_CF	blue	na
chrUn	32790806	32791659	854	SAT1_CF	blue	na
chrUn	32791689	32792647	959	SAT1_CF	blue	na
chrUn	32792697	32793397	701	SAT1_CF	blue	na
chrUn	32793447	32794416	970	SAT1_CF	blue	na
chrUn	32794439	32795000	562	SAT1_CF	blue	na
chrUn	32795018	32796065	1048	SAT1_CF	blue	na
chrUn	32796086	32796628	543	SAT1_CF	blue	na
chrUn	32796698	32797899	1202	SAT1_CF	blue	na
chrUn	32797943	32798249	307	SAT1_CF	blue	na
chrUn	32798329	32799182	854	SAT1_CF	blue	na
chrUn	32799212	32799741	530	SAT1_CF	blue	na
chrUn	32800663	32801025	363	SAT1_CF	blue	na
chrUn	32801075	32802680	1606	SAT1_CF	blue	na
chrUn	32802706	32804528	1823	SAT1_CF	blue	na
chrUn	32804566	32805066	501	SAT1_CF	blue	na
chrUn	32805079	32806301	1223	SAT1_CF	blue	na
chrUn	32806343	32808070	1728	SAT1_CF	blue	na
chrUn	32808142	32808641	500	SAT1_CF	blue	na
chrUn	32808654	32809337	684	SAT1_CF	blue	na
chrUn	32809349	32809969	621	SAT1_CF	blue	na
chrUn	32810056	32811200	1145	SAT1_CF	blue	na
chrUn	32811240	32812437	1198	SAT1_CF	blue	na
chrUn	32812449	32813131	683	SAT1_CF	blue	na
chrUn	32813142	32813634	493	SAT1_CF	blue	na
chrUn	32813648	32814680	1033	SAT1_CF	blue	na
chrUn	32814745	32815292	548	SAT1_CF	blue	na

chrUn	32816721	32817294	574	SAT1_CF	blue	na
chrUn	32817329	32817836	508	SAT1_CF	blue	na
chrUn	32817849	32819563	1715	SAT1_CF	blue	na
chrUn	32819647	32820192	546	SAT1_CF	blue	na
chrUn	32820205	32820362	158	SAT1_CF	blue	na
chrUn	32820409	32820930	522	SAT1_CF	blue	na
chrUn	32820943	32822233	1291	SAT1_CF	blue	na
chrUn	32822303	32822805	503	SAT1_CF	blue	na
chrUn	32822819	32823365	547	SAT1_CF	blue	na
chrUn	32823396	32824579	1184	SAT1_CF	blue	na
chrUn	32824591	32826352	1762	SAT1_CF	blue	na
chrUn	32826364	32827039	676	SAT1_CF	blue	na
chrUn	32827082	32828046	965	SAT1_CF	blue	na
chrUn	32828088	32829106	1019	SAT1_CF	blue	na
chrUn	32829159	32829653	495	SAT1_CF	blue	na
chrUn	32829673	32830355	683	SAT1_CF	blue	na
chrUn	32830367	32831041	675	SAT1_CF	blue	na
chrUn	32831084	32831609	526	SAT1_CF	blue	na
chrUn	32831690	32832273	584	SAT1_CF	blue	na
chrUn	32832285	32832601	317	SAT1_CF	blue	na
chrUn	32832614	32833116	503	SAT1_CF	blue	na
chrUn	32833133	32833235	103	SAT1_CF	blue	na
chrUn	32833257	32834494	1238	SAT1_CF	blue	na
chrUn	32834514	32835273	760	SAT1_CF	blue	na
chrUn	32835285	32836047	763	SAT1_CF	blue	na
chrUn	32836059	32837390	1332	SAT1_CF	blue	na
chrUn	32837434	32838633	1200	SAT1_CF	blue	na
chrUn	32838648	32838783	136	SAT1_CF	blue	na
chrUn	32838851	32839351	501	SAT1_CF	blue	na
chrUn	32839364	32840665	1302	SAT1_CF	blue	na
chrUn	32840677	32841901	1225	SAT1_CF	blue	na
chrUn	32841943	32843797	1855	SAT1_CF	blue	na
chrUn	32843818	32845658	1841	SAT1_CF	blue	na
chrUn	32845699	32846849	1151	SAT1_CF	blue	na
chrUn	32846860	32847467	608	SAT1_CF	blue	na
chrUn	32847537	32848091	555	SAT1_CF	blue	na
chrUn	32848104	32849522	1419	SAT1_CF	blue	na
chrUn	32849553	32850080	528	SAT1_CF	blue	na
chrUn	32850092	32851278	1187	SAT1_CF	blue	na
chrUn	32851365	32851857	493	SAT1_CF	blue	na
chrUn	32851877	32851982	106	SAT1_CF	blue	na
chrUn	32852005	32856133	4129	SAT1_CF	blue	na

chrUn	32856145	32857947	1803	SAT1_CF	blue	na
chrUn	32858027	32858192	166	SAT1_CF	blue	na
chrUn	32858372	32858556	185	SAT1_CF	blue	na
chrUn	32858592	32859091	500	SAT1_CF	blue	na
chrUn	32859105	32859211	107	SAT1_CF	blue	na
chrUn	32859234	32859753	520	SAT1_CF	blue	na
chrUn	32859765	32860616	852	SAT1_CF	blue	na
chrUn	32860701	32861448	748	SAT1_CF	blue	na
chrUn	32861514	32862026	513	SAT1_CF	blue	na
chrUn	32862051	32862152	102	SAT1_CF	blue	na
chrUn	32862173	32862727	555	SAT1_CF	blue	na
chrUn	32862739	32863647	909	SAT1_CF	blue	na
chrUn	32863677	32864224	548	SAT1_CF	blue	na
chrUn	32864236	32865570	1335	SAT1_CF	blue	na
chrUn	32865583	32865689	107	SAT1_CF	blue	na
chrUn	32865712	32866263	552	SAT1_CF	blue	na
chrUn	32866275	32867570	1296	SAT1_CF	blue	na
chrUn	32867583	32868085	503	SAT1_CF	blue	na
chrUn	32868098	32868860	763	SAT1_CF	blue	na
chrUn	32868872	32869549	678	SAT1_CF	blue	na
chrUn	32869618	32871200	1583	SAT1_CF	blue	na
chrUn	32871213	32871401	189	SAT1_CF	blue	na
chrUn	32871453	32872153	701	SAT1_CF	blue	na
chrUn	32872165	32872271	107	SAT1_CF	blue	na
chrUn	32872294	32872922	629	SAT1_CF	blue	na
chrUn	32872958	32873804	847	SAT1_CF	blue	na
chrUn	32873822	32874508	687	SAT1_CF	blue	na
chrUn	32874520	32875369	850	SAT1_CF	blue	na
chrUn	32875453	32875997	545	SAT1_CF	blue	na
chrUn	32876040	32876396	357	SAT1_CF	blue	na
chrUn	32876412	32877300	889	SAT1_CF	blue	na
chrUn	32877417	32877933	517	SAT1_CF	blue	na
chrUn	32877991	32878537	547	SAT1_CF	blue	na
chrUn	32878580	32879236	657	SAT1_CF	blue	na
chrUn	32879255	32879810	556	SAT1_CF	blue	na
chrUn	32879839	32881513	1675	SAT1_CF	blue	na
chrUn	32881558	32882632	1075	SAT1_CF	blue	na
chrUn	32882677	32883185	509	SAT1_CF	blue	na
chrUn	32883198	32883386	189	SAT1_CF	blue	na
chrUn	32883438	32884138	701	SAT1_CF	blue	na
chrUn	32884150	32884256	107	SAT1_CF	blue	na
chrUn	32884279	32884907	629	SAT1_CF	blue	na

chrUn	32884943	32885789	847	SAT1_CF	blue	na
chrUn	32885807	32886493	687	SAT1_CF	blue	na
chrUn	32886505	32887354	850	SAT1_CF	blue	na
chrUn	32887438	32887982	545	SAT1_CF	blue	na
chrUn	32888025	32888381	357	SAT1_CF	blue	na
chrUn	32888397	32889285	889	SAT1_CF	blue	na
chrUn	32889402	32889918	517	SAT1_CF	blue	na
chrUn	32889976	32890522	547	SAT1_CF	blue	na
chrUn	32890565	32891221	657	SAT1_CF	blue	na
chrUn	32891240	32891795	556	SAT1_CF	blue	na
chrUn	32891824	32894508	2685	SAT1_CF	blue	na
chrUn	32894575	32895170	596	SAT1_CF	blue	na
chrUn	32895183	32895371	189	SAT1_CF	blue	na
chrUn	32895423	32896123	701	SAT1_CF	blue	na
chrUn	32896135	32896241	107	SAT1_CF	blue	na
chrUn	32896264	32896890	627	SAT1_CF	blue	na
chrUn	32896926	32897043	118	SAT1_CF	blue	na
chrUn	32898743	32899181	439	CarSat1	red	na
chrUn	32899202	32900056	855	CarSat1	red	na
chrUn	32900077	32900872	796	CarSat1	red	na
chrUn	32900890	32901657	768	CarSat1	red	na
chrUn	32901687	32902391	705	CarSat1	red	na
chrUn	32923104	32923874	771	Sat2CF	purple	na
chrUn	32937010	32938238	1229	Sat2CF	purple	na
chrUn	32957724	32958587	864	Sat2CF	purple	na
chrUn	32957800	32958525	726	Sat2CF	purple	na
chrUn	32971546	32972797	1252	Sat2CF	purple	na
chrUn	32973116	32974386	1271	CarSat1	red	na
chrUn	32974407	32975892	1486	CarSat1	red	na
chrUn	32984444	32985044	601	CarSat1	red	na
chrUn	32985061	32985158	98	CarSat1	red	na
chrUn	32985352	32988426	3075	CarSat1	red	na
chrUn	32988450	32991134	2685	CarSat1	red	na
chrUn	32991327	32993047	1721	CarSat1	red	na
chrUn	32995150	32996200	1051	CarSat1	red	na
chrUn	32996230	32999141	2912	CarSat1	red	na
chrUn	32999158	32999285	128	Carsat2	orange	na
chrUn	32999173	33001761	2589	CarSat1	red	na
chrUn	33001947	33002077	131	CarSat1	red	na
chrUn	33002102	33004931	2830	CarSat1	red	na
chrUn	33012326	33013538	1213	Sat2CF	purple	na
chrUn	33034878	33036009	1132	Sat2CF	purple	na

chrUn	33035561	33036059	499	Sat2CF	purple	na
chrUn	33055634	33056589	956	Sat2CF	purple	na
chrUn	33055709	33056568	860	Sat2CF	purple	na
chrUn	33069260	33069650	391	Sat2CF	purple	na
chrUn	33069326	33069677	352	Sat2CF	purple	na
chrUn	33087705	33087841	137	Sat2CF	purple	na
chrUn	33087730	33088528	799	Sat2CF	purple	na
chrUn	33113171	33114325	1155	Sat2CF	purple	na
chrUn	33118525	33119213	689	SAT1_CF	blue	na
chrUn	33119226	33120076	851	SAT1_CF	blue	na
chrUn	33120088	33120765	678	SAT1_CF	blue	na
chrUn	33120792	33121335	544	SAT1_CF	blue	na
chrUn	33121379	33121923	545	SAT1_CF	blue	na
chrUn	33121947	33122548	602	SAT1_CF	blue	na
chrUn	33122618	33122749	132	SAT1_CF	blue	na
chrUn	33122777	33124623	1847	SAT1_CF	blue	na
chrUn	33124666	33125333	668	SAT1_CF	blue	na
chrUn	33125356	33125967	612	SAT1_CF	blue	na
chrUn	33125978	33126531	554	SAT1_CF	blue	na
chrUn	33126574	33127136	563	SAT1_CF	blue	na
chrUn	33127201	33128566	1366	SAT1_CF	blue	na
chrUn	33128580	33129072	493	SAT1_CF	blue	na
chrUn	33129087	33129687	601	SAT1_CF	blue	na
chrUn	33129713	33130802	1090	SAT1_CF	blue	na
chrUn	33130829	33131463	635	SAT1_CF	blue	na
chrUn	33131485	33132006	522	SAT1_CF	blue	na
chrUn	33132053	33132189	137	SAT1_CF	blue	na
chrUn	33132204	33133410	1207	SAT1_CF	blue	na
chrUn	33133438	33134069	632	SAT1_CF	blue	na
chrUn	33134112	33134776	665	SAT1_CF	blue	na
chrUn	33134799	33135482	684	SAT1_CF	blue	na
chrUn	33135494	33135969	476	SAT1_CF	blue	na
chrUn	33136039	33136712	674	SAT1_CF	blue	na
chrUn	33136724	33137266	543	SAT1_CF	blue	na
chrUn	33137309	33138458	1150	SAT1_CF	blue	na
chrUn	33138471	33139321	851	SAT1_CF	blue	na
chrUn	33139334	33139856	523	SAT1_CF	blue	na
chrUn	33139883	33140147	265	SAT1_CF	blue	na
chrUn	33140174	33140676	503	SAT1_CF	blue	na
chrUn	33140714	33141676	963	SAT1_CF	blue	na
chrUn	33141755	33142080	326	SAT1_CF	blue	na
chrUn	33142104	33143434	1331	SAT1_CF	blue	na

chrUn	33143446	33143997	552	SAT1_CF	blue	na
chrUn	33144016	33144430	415	SAT1_CF	blue	na
chrUn	33144448	33145038	591	SAT1_CF	blue	na
chrUn	33145065	33145567	503	SAT1_CF	blue	na
chrUn	33145620	33146503	884	SAT1_CF	blue	na
chrUn	33146535	33147349	815	SAT1_CF	blue	na
chrUn	33147391	33148139	749	SAT1_CF	blue	na
chrUn	33148152	33148634	483	SAT1_CF	blue	na
chrUn	33148718	33149060	343	SAT1_CF	blue	na
chrUn	33149103	33150073	971	SAT1_CF	blue	na
chrUn	33150117	33151435	1319	SAT1_CF	blue	na
chrUn	33151447	33152136	690	SAT1_CF	blue	na
chrUn	33152149	33152650	502	SAT1_CF	blue	na
chrUn	33152695	33154074	1380	SAT1_CF	blue	na
chrUn	33154144	33155006	863	SAT1_CF	blue	na
chrUn	33155018	33155650	633	SAT1_CF	blue	na
chrUn	33155667	33155780	114	SAT1_CF	blue	na
chrUn	33155792	33156092	301	SAT1_CF	blue	na
chrUn	33156105	33157663	1559	SAT1_CF	blue	na
chrUn	33157718	33158679	962	SAT1_CF	blue	na
chrUn	33158723	33159399	677	SAT1_CF	blue	na
chrUn	33159411	33160093	683	SAT1_CF	blue	na
chrUn	33160106	33160604	499	SAT1_CF	blue	na
chrUn	33160660	33161529	870	SAT1_CF	blue	na
chrUn	33161558	33161762	205	SAT1_CF	blue	na
chrUn	33161776	33162425	650	SAT1_CF	blue	na
chrUn	33162502	33163364	863	SAT1_CF	blue	na
chrUn	33163376	33163931	556	SAT1_CF	blue	na
chrUn	33163952	33164059	108	SAT1_CF	blue	na
chrUn	33164079	33164573	495	SAT1_CF	blue	na
chrUn	33164619	33164982	364	SAT1_CF	blue	na
chrUn	33165028	33165921	894	SAT1_CF	blue	na
chrUn	33165934	33167239	1306	SAT1_CF	blue	na
chrUn	33167265	33168063	799	SAT1_CF	blue	na
chrUn	33168076	33168764	689	SAT1_CF	blue	na
chrUn	33168841	33169380	540	SAT1_CF	blue	na
chrUn	33169402	33169550	149	SAT1_CF	blue	na
chrUn	33169594	33170925	1332	SAT1_CF	blue	na
chrUn	33170939	33171560	622	SAT1_CF	blue	na
chrUn	33171585	33171689	105	SAT1_CF	blue	na
chrUn	33171701	33172306	606	SAT1_CF	blue	na
chrUn	33172319	33172839	521	SAT1_CF	blue	na

chrUn	33172907	33175483	2577	SAT1_CF	blue	na
chrUn	33175503	33175998	496	SAT1_CF	blue	na
chrUn	33176084	33176960	877	SAT1_CF	blue	na
chrUn	33177066	33177605	540	SAT1_CF	blue	na
chrUn	33177617	33178170	554	SAT1_CF	blue	na
chrUn	33178193	33178298	106	SAT1_CF	blue	na
chrUn	33178311	33179517	1207	SAT1_CF	blue	na
chrUn	33179632	33180248	617	SAT1_CF	blue	na
chrUn	33180260	33181024	765	SAT1_CF	blue	na
chrUn	33181047	33181838	792	SAT1_CF	blue	na
chrUn	33181882	33182558	677	SAT1_CF	blue	na
chrUn	33182578	33183071	494	SAT1_CF	blue	na
chrUn	33183119	33183470	352	SAT1_CF	blue	na
chrUn	33184878	33186811	1934	SAT1_CF	blue	na
chrUn	33186855	33187530	676	SAT1_CF	blue	na
chrUn	33187544	33188302	759	SAT1_CF	blue	na
chrUn	33188315	33189384	1070	SAT1_CF	blue	na
chrUn	33189405	33189514	110	SAT1_CF	blue	na
chrUn	33189526	33190024	499	SAT1_CF	blue	na
chrUn	33190063	33190463	401	SAT1_CF	blue	na
chrUn	33190475	33192016	1542	SAT1_CF	blue	na
chrUn	33192028	33192586	559	SAT1_CF	blue	na
chrUn	33192638	33192802	165	SAT1_CF	blue	na
chrUn	33192836	33194774	1939	SAT1_CF	blue	na
chrUn	33194800	33195320	521	SAT1_CF	blue	na
chrUn	33195346	33195504	159	SAT1_CF	blue	na
chrUn	33195532	33196081	550	SAT1_CF	blue	na
chrUn	33196161	33196721	561	SAT1_CF	blue	na
chrUn	33196749	33197380	632	SAT1_CF	blue	na
chrUn	33197423	33198791	1369	SAT1_CF	blue	na
chrUn	33198803	33199354	552	SAT1_CF	blue	na
chrUn	33199382	33199576	195	SAT1_CF	blue	na
chrUn	33199619	33200181	563	SAT1_CF	blue	na
chrUn	33200225	33202251	2027	SAT1_CF	blue	na
chrUn	33202264	33202440	177	SAT1_CF	blue	na
chrUn	33202454	33203005	552	SAT1_CF	blue	na
chrUn	33203048	33203792	745	SAT1_CF	blue	na
chrUn	33203819	33204304	486	SAT1_CF	blue	na
chrUn	33204330	33205742	1413	SAT1_CF	blue	na
chrUn	33205770	33206402	633	SAT1_CF	blue	na
chrUn	33206445	33208439	1995	SAT1_CF	blue	na
chrUn	33208461	33209184	724	SAT1_CF	blue	na

chrUn	33209197	33209717	521	SAT1_CF	blue	na
chrUn	33209743	33209851	109	SAT1_CF	blue	na
chrUn	33209862	33210418	557	SAT1_CF	blue	na
chrUn	33210451	33210785	335	SAT1_CF	blue	na
chrUn	33210830	33211474	645	SAT1_CF	blue	na
chrUn	33211489	33212161	673	SAT1_CF	blue	na
chrUn	33212173	33212844	672	SAT1_CF	blue	na
chrUn	33212868	33213404	537	SAT1_CF	blue	na
chrUn	33213435	33214210	776	SAT1_CF	blue	na
chrUn	33214287	33215149	863	SAT1_CF	blue	na
chrUn	33215161	33215716	556	SAT1_CF	blue	na
chrUn	33215781	33216339	559	SAT1_CF	blue	na
chrUn	33216360	33216758	399	SAT1_CF	blue	na
chrUn	33216772	33218665	1894	SAT1_CF	blue	na
chrUn	33218685	33219180	496	SAT1_CF	blue	na
chrUn	33219225	33219382	158	SAT1_CF	blue	na
chrUn	33219397	33219921	525	SAT1_CF	blue	na
chrUn	33220000	33221124	1125	SAT1_CF	blue	na
chrUn	33221136	33221656	521	SAT1_CF	blue	na
chrUn	33221677	33222800	1124	SAT1_CF	blue	na
chrUn	33222831	33223401	571	SAT1_CF	blue	na
chrUn	33224422	33225426	1005	SAT1_CF	blue	na
chrUn	33225438	33226295	858	SAT1_CF	blue	na
chrUn	33226348	33227782	1435	SAT1_CF	blue	na
chrUn	33227826	33228483	658	SAT1_CF	blue	na
chrUn	33228527	33229066	540	SAT1_CF	blue	na
chrUn	33229078	33230343	1266	SAT1_CF	blue	na
chrUn	33230381	33230883	503	SAT1_CF	blue	na
chrUn	33230901	33232274	1374	SAT1_CF	blue	na
chrUn	33232286	33232961	676	SAT1_CF	blue	na
chrUn	33233058	33233600	543	SAT1_CF	blue	na
chrUn	33233644	33234203	560	SAT1_CF	blue	na
chrUn	33234246	33234758	513	SAT1_CF	blue	na
chrUn	33234786	33234922	137	SAT1_CF	blue	na
chrUn	33235007	33235489	483	SAT1_CF	blue	na
chrUn	33235502	33236952	1451	SAT1_CF	blue	na
chrUn	33236995	33237539	545	SAT1_CF	blue	na
chrUn	33237581	33237758	178	SAT1_CF	blue	na
chrUn	33237818	33238367	550	SAT1_CF	blue	na
chrUn	33238432	33239214	783	SAT1_CF	blue	na
chrUn	33239276	33241051	1776	SAT1_CF	blue	na
chrUn	33241114	33241737	624	SAT1_CF	blue	na

chrUn	33241757	33242464	708	SAT1_CF	blue	na
chrUn	33242506	33244363	1858	SAT1_CF	blue	na
chrUn	33244403	33245025	623	SAT1_CF	blue	na
chrUn	33245046	33245600	555	SAT1_CF	blue	na
chrUn	33245612	33246287	676	SAT1_CF	blue	na
chrUn	33246330	33247296	967	SAT1_CF	blue	na
chrUn	33247374	33247918	545	SAT1_CF	blue	na
chrUn	33247956	33248283	328	SAT1_CF	blue	na
chrUn	33248370	33249645	1276	SAT1_CF	blue	na
chrUn	33249657	33250375	719	SAT1_CF	blue	na
chrUn	33250454	33250776	323	SAT1_CF	blue	na
chrUn	33250788	33250897	110	SAT1_CF	blue	na
chrUn	33250918	33251465	548	SAT1_CF	blue	na
chrUn	33251508	33252235	728	SAT1_CF	blue	na
chrUn	33252300	33254079	1780	SAT1_CF	blue	na
chrUn	33254102	33254768	667	SAT1_CF	blue	na
chrUn	33254811	33255337	527	SAT1_CF	blue	na
chrUn	33255358	33256440	1083	SAT1_CF	blue	na
chrUn	33256451	33256852	402	SAT1_CF	blue	na
chrUn	33256892	33257394	503	SAT1_CF	blue	na
chrUn	33257407	33257601	195	SAT1_CF	blue	na
chrUn	33257617	33258162	546	SAT1_CF	blue	na
chrUn	33258204	33258748	545	SAT1_CF	blue	na
chrUn	33258764	33260442	1679	SAT1_CF	blue	na
chrUn	33260454	33261107	654	SAT1_CF	blue	na
chrUn	33261203	33261750	548	SAT1_CF	blue	na
chrUn	33261778	33261934	157	SAT1_CF	blue	na
chrUn	33261979	33263182	1204	SAT1_CF	blue	na
chrUn	33263194	33264550	1357	SAT1_CF	blue	na
chrUn	33264593	33264958	366	SAT1_CF	blue	na
chrUn	33265030	33265549	520	SAT1_CF	blue	na
chrUn	33265606	33266151	546	SAT1_CF	blue	na
chrUn	33266193	33267601	1409	SAT1_CF	blue	na
chrUn	33267645	33268013	369	SAT1_CF	blue	na
chrUn	33268053	33268554	502	SAT1_CF	blue	na
chrUn	33268576	33268679	104	SAT1_CF	blue	na
chrUn	33268696	33269325	630	SAT1_CF	blue	na
chrUn	33269339	33269951	613	SAT1_CF	blue	na
chrUn	33269963	33270821	859	SAT1_CF	blue	na
chrUn	33270898	33271444	547	SAT1_CF	blue	na
chrUn	33271575	33272054	480	SAT1_CF	blue	na
chrUn	33272134	33272657	524	SAT1_CF	blue	na

chrUn	33272672	33273369	698	SAT1_CF	blue	na
chrUn	33273389	33274719	1331	SAT1_CF	blue	na
chrUn	33274802	33275362	561	SAT1_CF	blue	na
chrUn	33275446	33275994	549	SAT1_CF	blue	na
chrUn	33276022	33276725	704	SAT1_CF	blue	na
chrUn	33276746	33277419	674	SAT1_CF	blue	na
chrUn	33277431	33278292	862	SAT1_CF	blue	na
chrUn	33278334	33279113	780	SAT1_CF	blue	na
chrUn	33279193	33280138	946	SAT1_CF	blue	na
chrUn	33280208	33282276	2069	SAT1_CF	blue	na
chrUn	33282297	33282817	521	SAT1_CF	blue	na
chrUn	33282830	33282936	107	SAT1_CF	blue	na
chrUn	33282959	33283533	575	SAT1_CF	blue	na
chrUn	33283545	33285673	2129	SAT1_CF	blue	na
chrUn	33285701	33285858	158	SAT1_CF	blue	na
chrUn	33285903	33286403	501	SAT1_CF	blue	na
chrUn	33286430	33287097	668	SAT1_CF	blue	na
chrUn	33287109	33287788	680	SAT1_CF	blue	na
chrUn	33287831	33288793	963	SAT1_CF	blue	na
chrUn	33288872	33289830	959	SAT1_CF	blue	na
chrUn	33289851	33290629	779	SAT1_CF	blue	na
chrUn	33290673	33291220	548	SAT1_CF	blue	na
chrUn	33291263	33291893	631	SAT1_CF	blue	na
chrUn	33291920	33292482	563	SAT1_CF	blue	na
chrUn	33292562	33292874	313	SAT1_CF	blue	na
chrUn	33292893	33293435	543	SAT1_CF	blue	na
chrUn	33293506	33294141	636	SAT1_CF	blue	na
chrUn	33294153	33295312	1160	SAT1_CF	blue	na
chrUn	33295332	33295941	610	SAT1_CF	blue	na
chrUn	33296021	33296186	166	SAT1_CF	blue	na
chrUn	33296367	33296552	186	SAT1_CF	blue	na
chrUn	33296569	33297088	520	SAT1_CF	blue	na
chrUn	33297101	33297206	106	SAT1_CF	blue	na
chrUn	33297229	33297860	632	SAT1_CF	blue	na
chrUn	33297872	33298555	684	SAT1_CF	blue	na
chrUn	33298567	33299166	600	SAT1_CF	blue	na
chrUn	33299224	33299581	358	SAT1_CF	blue	na
chrUn	33299602	33300154	553	SAT1_CF	blue	na
chrUn	33300166	33301046	881	SAT1_CF	blue	na
chrUn	33301085	33301610	526	SAT1_CF	blue	na
chrUn	33301625	33302330	706	SAT1_CF	blue	na
chrUn	33302357	33303101	745	SAT1_CF	blue	na

chrUn	33303113	33303936	824	SAT1_CF	blue	na
chrUn	33303979	33304761	783	SAT1_CF	blue	na
chrUn	33304841	33305388	548	SAT1_CF	blue	na
chrUn	33305399	33305799	401	SAT1_CF	blue	na
chrUn	33305839	33307211	1373	SAT1_CF	blue	na
chrUn	33307253	33308329	1077	SAT1_CF	blue	na
chrUn	33308372	33308919	548	SAT1_CF	blue	na
chrUn	33308947	33309083	137	SAT1_CF	blue	na
chrUn	33309149	33309650	502	SAT1_CF	blue	na
chrUn	33309673	33310346	674	SAT1_CF	blue	na
chrUn	33310358	33311196	839	SAT1_CF	blue	na
chrUn	33311238	33312276	1039	SAT1_CF	blue	na
chrUn	33312333	33313009	677	SAT1_CF	blue	na
chrUn	33313037	33314422	1386	SAT1_CF	blue	na
chrUn	33314456	33314835	380	SAT1_CF	blue	na
chrUn	33314875	33315376	502	SAT1_CF	blue	na
chrUn	33315389	33316150	762	SAT1_CF	blue	na
chrUn	33316167	33317391	1225	SAT1_CF	blue	na
chrUn	33317416	33317561	146	SAT1_CF	blue	na
chrUn	33317616	33318674	1059	SAT1_CF	blue	na
chrUn	33318751	33319293	543	SAT1_CF	blue	na
chrUn	33319306	33319412	107	SAT1_CF	blue	na
chrUn	33319435	33320829	1395	SAT1_CF	blue	na
chrUn	33320842	33320943	102	SAT1_CF	blue	na
chrUn	33320971	33322127	1157	SAT1_CF	blue	na
chrUn	33322144	33323448	1305	SAT1_CF	blue	na
chrUn	33323488	33324779	1292	SAT1_CF	blue	na
chrUn	33324830	33325365	536	SAT1_CF	blue	na
chrUn	33326773	33327484	712	SAT1_CF	blue	na
chrUn	33327503	33328036	534	SAT1_CF	blue	na
chrUn	33328078	33329066	989	SAT1_CF	blue	na
chrUn	33330111	33331711	1601	SAT1_CF	blue	na
chrUn	33331737	33332282	546	SAT1_CF	blue	na
chrUn	33332327	33332958	632	SAT1_CF	blue	na
chrUn	33332986	33333548	563	SAT1_CF	blue	na
chrUn	33333628	33334137	510	SAT1_CF	blue	na
chrUn	33334165	33334298	134	SAT1_CF	blue	na
chrUn	33334356	33336240	1885	SAT1_CF	blue	na
chrUn	33336282	33336826	545	SAT1_CF	blue	na
chrUn	33336862	33337723	862	SAT1_CF	blue	na
chrUn	33337737	33338139	403	SAT1_CF	blue	na
chrUn	33338179	33338678	500	SAT1_CF	blue	na

chrUn	33338705	33340715	2011	SAT1_CF	blue	na
chrUn	33340759	33341247	489	SAT1_CF	blue	na
chrUn	33341260	33341760	501	SAT1_CF	blue	na
chrUn	33341773	33342456	684	SAT1_CF	blue	na
chrUn	33342468	33343899	1432	SAT1_CF	blue	na
chrUn	33343942	33344558	617	SAT1_CF	blue	na
chrUn	33344585	33344675	91	SAT1_CF	blue	na
chrUn	33344699	33345251	553	SAT1_CF	blue	na
chrUn	33345263	33345938	676	SAT1_CF	blue	na
chrUn	33345981	33348019	2039	SAT1_CF	blue	na
chrUn	33348081	33348561	481	SAT1_CF	blue	na
chrUn	33348574	33349247	674	SAT1_CF	blue	na
chrUn	33349290	33349921	632	SAT1_CF	blue	na
chrUn	33349949	33351182	1234	SAT1_CF	blue	na
chrUn	33351205	33351730	526	SAT1_CF	blue	na
chrUn	33351748	33352263	516	SAT1_CF	blue	na
chrUn	33352350	33352849	500	SAT1_CF	blue	na
chrUn	33352872	33354230	1359	SAT1_CF	blue	na
chrUn	33354243	33354414	172	SAT1_CF	blue	na
chrUn	33354456	33355000	545	SAT1_CF	blue	na
chrUn	33355038	33356069	1032	SAT1_CF	blue	na
chrUn	33356136	33356637	502	SAT1_CF	blue	na
chrUn	33356664	33358692	2029	SAT1_CF	blue	na
chrUn	33358771	33359331	561	SAT1_CF	blue	na
chrUn	33359472	33360438	967	SAT1_CF	blue	na
chrUn	33360484	33360981	498	SAT1_CF	blue	na
chrUn	33360999	33361669	671	SAT1_CF	blue	na
chrUn	33361690	33362150	461	SAT1_CF	blue	na
chrUn	33362192	33362726	535	SAT1_CF	blue	na
chrUn	33362770	33363738	969	SAT1_CF	blue	na
chrUn	33363750	33364427	678	SAT1_CF	blue	na
chrUn	33364446	33365151	706	SAT1_CF	blue	na
chrUn	33365229	33365941	713	SAT1_CF	blue	na
chrUn	33365988	33366501	514	SAT1_CF	blue	na
chrUn	33366526	33367204	679	SAT1_CF	blue	na
chrUn	33367216	33367325	110	SAT1_CF	blue	na
chrUn	33367345	33368588	1244	SAT1_CF	blue	na
chrUn	33368631	33370285	1655	SAT1_CF	blue	na
chrUn	33370296	33370615	320	SAT1_CF	blue	na
chrUn	33370668	33371168	501	SAT1_CF	blue	na
chrUn	33371180	33371863	684	SAT1_CF	blue	na
chrUn	33371877	33373658	1782	SAT1_CF	blue	na

chrUn	33373671	33374025	355	SAT1_CF	blue	na
chrUn	33374112	33374613	502	SAT1_CF	blue	na
chrUn	33374626	33374734	109	SAT1_CF	blue	na
chrUn	33374755	33375316	562	SAT1_CF	blue	na
chrUn	33375329	33376175	847	SAT1_CF	blue	na
chrUn	33376217	33376760	544	SAT1_CF	blue	na
chrUn	33376798	33377104	307	SAT1_CF	blue	na
chrUn	33377136	33377659	524	SAT1_CF	blue	na
chrUn	33377692	33378071	380	SAT1_CF	blue	na
chrUn	33378141	33378686	546	SAT1_CF	blue	na
chrUn	33378699	33379499	801	SAT1_CF	blue	na
chrUn	33379525	33381245	1721	SAT1_CF	blue	na
chrUn	33381257	33381330	74	SAT1_CF	blue	na
chrUn	33381386	33381940	555	SAT1_CF	blue	na
chrUn	33381952	33384438	2487	SAT1_CF	blue	na
chrUn	33384458	33384565	108	SAT1_CF	blue	na
chrUn	33384586	33385741	1156	SAT1_CF	blue	na
chrUn	33385796	33386405	610	SAT1_CF	blue	na
chrUn	33386447	33386802	356	SAT1_CF	blue	na
chrUn	33386889	33387389	501	SAT1_CF	blue	na
chrUn	33387416	33388771	1356	SAT1_CF	blue	na
chrUn	33388814	33389778	965	SAT1_CF	blue	na
chrUn	33389845	33391210	1366	SAT1_CF	blue	na
chrUn	33391252	33392030	779	SAT1_CF	blue	na
chrUn	33392042	33393205	1164	SAT1_CF	blue	na
chrUn	33393216	33393742	527	SAT1_CF	blue	na
chrUn	33393784	33394295	512	SAT1_CF	blue	na
chrUn	33394328	33394659	332	SAT1_CF	blue	na
chrUn	33394748	33395248	501	SAT1_CF	blue	na
chrUn	33395260	33396018	759	SAT1_CF	blue	na
chrUn	33396042	33396901	860	SAT1_CF	blue	na
chrUn	33396919	33397604	686	SAT1_CF	blue	na
chrUn	33397616	33398466	851	SAT1_CF	blue	na
chrUn	33398516	33400398	1883	SAT1_CF	blue	na
chrUn	33400515	33401034	520	SAT1_CF	blue	na
chrUn	33401091	33401637	547	SAT1_CF	blue	na
chrUn	33401717	33402260	544	SAT1_CF	blue	na
chrUn	33402357	33403499	1143	SAT1_CF	blue	na
chrUn	33403541	33404039	499	SAT1_CF	blue	na
chrUn	33404057	33404739	683	SAT1_CF	blue	na
chrUn	33404752	33404823	72	SAT1_CF	blue	na
chrUn	33404846	33406301	1456	SAT1_CF	blue	na

chrUn	33406369	33408312	1944	SAT1_CF	blue	na
chrUn	33408356	33408781	426	SAT1_CF	blue	na
chrUn	33408803	33409732	930	SAT1_CF	blue	na
chrUn	33409770	33410268	499	SAT1_CF	blue	na
chrUn	33410281	33411040	760	SAT1_CF	blue	na
chrUn	33411052	33411974	923	SAT1_CF	blue	na
chrUn	33411992	33412510	519	SAT1_CF	blue	na
chrUn	33412541	33413065	525	SAT1_CF	blue	na
chrUn	33413109	33413576	468	SAT1_CF	blue	na
chrUn	33413737	33413823	87	SAT1_CF	blue	na
chrUn	33413851	33414411	561	SAT1_CF	blue	na
chrUn	33414492	33414666	175	SAT1_CF	blue	na
chrUn	33414734	33415254	521	SAT1_CF	blue	na
chrUn	33415267	33415915	649	SAT1_CF	blue	na
chrUn	33416046	33416837	792	SAT1_CF	blue	na
chrUn	33416881	33417141	261	SAT1_CF	blue	na
chrUn	33417179	33418366	1188	SAT1_CF	blue	na
chrUn	33418409	33418921	513	SAT1_CF	blue	na
chrUn	33418999	33419543	545	SAT1_CF	blue	na
chrUn	33419585	33423323	3739	SAT1_CF	blue	na
chrUn	33423351	33423982	632	SAT1_CF	blue	na
chrUn	33423994	33424532	539	SAT1_CF	blue	na
chrUn	33428884	33428968	85	SAT1_CF	blue	na
chrUn	33429012	33430696	1685	SAT1_CF	blue	na
chrUn	33453079	33453358	280	Sat2CF	purple	na
chrUn	33453143	33453353	211	Sat2CF	purple	na
chrUn	33453579	33453867	289	Sat2CF	purple	na
chrUn	33475414	33475615	202	Sat2CF	purple	na
chrUn	33475455	33476631	1177	Sat2CF	purple	na
chrUn	33500583	33500732	150	Sat2CF	purple	na
chrUn	33500603	33501820	1218	Sat2CF	purple	na
chrUn	33532787	33533941	1155	Sat2CF	purple	na
chrUn	33684209	33684719	511	SAT1_CF	blue	na
chrUn	33684734	33684891	158	SAT1_CF	blue	na
chrUn	33684938	33685438	501	SAT1_CF	blue	na
chrUn	33685450	33685556	107	SAT1_CF	blue	na
chrUn	33685580	33686134	555	SAT1_CF	blue	na
chrUn	33686156	33687684	1529	SAT1_CF	blue	na
chrUn	33687727	33689647	1921	SAT1_CF	blue	na
chrUn	33689700	33690202	503	SAT1_CF	blue	na
chrUn	33690215	33690897	683	SAT1_CF	blue	na
chrUn	33690921	33691630	710	SAT1_CF	blue	na

chrUn	33691654	33692168	515	SAT1_CF	blue	na
chrUn	33692184	33692427	244	SAT1_CF	blue	na
chrUn	33692505	33694564	2060	SAT1_CF	blue	na
chrUn	33694576	33695436	861	SAT1_CF	blue	na
chrUn	33695478	33696019	542	SAT1_CF	blue	na
chrUn	33696065	33696882	818	SAT1_CF	blue	na
chrUn	33696915	33697566	652	SAT1_CF	blue	na
chrUn	33697585	33697748	164	SAT1_CF	blue	na
chrUn	33697761	33699279	1519	SAT1_CF	blue	na
chrUn	33699307	33700532	1226	SAT1_CF	blue	na
chrUn	33700612	33701133	522	SAT1_CF	blue	na
chrUn	33701165	33701259	95	SAT1_CF	blue	na
chrUn	33701274	33701794	521	SAT1_CF	blue	na
chrUn	33701822	33701913	92	SAT1_CF	blue	na
chrUn	33701937	33702491	555	SAT1_CF	blue	na
chrUn	33702503	33703365	863	SAT1_CF	blue	na
chrUn	33703407	33703948	542	SAT1_CF	blue	na
chrUn	33703983	33704849	867	SAT1_CF	blue	na
chrUn	33704929	33705510	582	SAT1_CF	blue	na
chrUn	33705522	33706198	677	SAT1_CF	blue	na
chrUn	33706241	33706870	630	SAT1_CF	blue	na
chrUn	33706898	33707462	565	SAT1_CF	blue	na
chrUn	33707541	33708064	524	SAT1_CF	blue	na
chrUn	33708079	33708237	159	SAT1_CF	blue	na
chrUn	33708281	33708781	501	SAT1_CF	blue	na
chrUn	33708803	33709477	675	SAT1_CF	blue	na
chrUn	33709489	33710360	872	SAT1_CF	blue	na
chrUn	33710383	33711404	1022	SAT1_CF	blue	na
chrUn	33711503	33712325	823	SAT1_CF	blue	na
chrUn	33712358	33712732	375	SAT1_CF	blue	na
chrUn	33712757	33713278	522	SAT1_CF	blue	na
chrUn	33713291	33714625	1335	SAT1_CF	blue	na
chrUn	33714669	33716048	1380	SAT1_CF	blue	na
chrUn	33716068	33716588	521	SAT1_CF	blue	na
chrUn	33716601	33717284	684	SAT1_CF	blue	na
chrUn	33717296	33720267	2972	SAT1_CF	blue	na
chrUn	33720368	33720816	449	SAT1_CF	blue	na
chrUn	33720859	33721823	965	SAT1_CF	blue	na
chrUn	33721849	33722654	806	SAT1_CF	blue	na
chrUn	33722720	33723221	502	SAT1_CF	blue	na
chrUn	33723233	33723341	109	SAT1_CF	blue	na
chrUn	33723364	33723919	556	SAT1_CF	blue	na

chrUn	33723931	33724040	110	SAT1_CF	blue	na
chrUn	33724060	33724604	545	SAT1_CF	blue	na
chrUn	33724647	33726008	1362	SAT1_CF	blue	na
chrUn	33726036	33726172	137	SAT1_CF	blue	na
chrUn	33726219	33726785	567	SAT1_CF	blue	na
chrUn	33726855	33727472	618	SAT1_CF	blue	na
chrUn	33727484	33729221	1738	SAT1_CF	blue	na
chrUn	33729233	33729909	677	SAT1_CF	blue	na
chrUn	33729925	33730720	796	SAT1_CF	blue	na
chrUn	33730732	33732492	1761	SAT1_CF	blue	na
chrUn	33732536	33733082	547	SAT1_CF	blue	na
chrUn	33733152	33733668	517	SAT1_CF	blue	na
chrUn	33733706	33734977	1272	SAT1_CF	blue	na
chrUn	33735017	33735511	495	SAT1_CF	blue	na
chrUn	33735545	33736213	669	SAT1_CF	blue	na
chrUn	33736225	33736900	676	SAT1_CF	blue	na
chrUn	33736943	33738680	1738	SAT1_CF	blue	na
chrUn	33738692	33740053	1362	SAT1_CF	blue	na
chrUn	33740066	33740645	580	SAT1_CF	blue	na
chrUn	33740708	33741945	1238	SAT1_CF	blue	na
chrUn	33741988	33743643	1656	SAT1_CF	blue	na
chrUn	33743654	33743973	320	SAT1_CF	blue	na
chrUn	33744026	33744527	502	SAT1_CF	blue	na
chrUn	33744539	33745222	684	SAT1_CF	blue	na
chrUn	33745236	33746494	1259	SAT1_CF	blue	na
chrUn	33746581	33747073	493	SAT1_CF	blue	na
chrUn	33747093	33747208	116	SAT1_CF	blue	na
chrUn	33747221	33747862	642	SAT1_CF	blue	na
chrUn	33747881	33749545	1665	SAT1_CF	blue	na
chrUn	33749588	33750173	586	SAT1_CF	blue	na
chrUn	33750185	33751782	1598	SAT1_CF	blue	na
chrUn	33751794	33753207	1414	SAT1_CF	blue	na
chrUn	33753253	33753626	374	SAT1_CF	blue	na
chrUn	33753662	33754162	501	SAT1_CF	blue	na
chrUn	33754175	33754282	108	SAT1_CF	blue	na
chrUn	33754304	33754858	555	SAT1_CF	blue	na
chrUn	33754876	33755550	675	SAT1_CF	blue	na
chrUn	33755611	33756155	545	SAT1_CF	blue	na
chrUn	33756213	33756714	502	SAT1_CF	blue	na
chrUn	33756751	33756908	158	SAT1_CF	blue	na
chrUn	33756934	33757455	522	SAT1_CF	blue	na
chrUn	33757468	33758602	1135	SAT1_CF	blue	na

chrUn	33758614	33759266	653	SAT1_CF	blue	na
chrUn	33759309	33759511	203	SAT1_CF	blue	na
chrUn	33759691	33759876	186	SAT1_CF	blue	na
chrUn	33759912	33760410	499	SAT1_CF	blue	na
chrUn	33760437	33761143	707	SAT1_CF	blue	na
chrUn	33761166	33761898	733	SAT1_CF	blue	na
chrUn	33761911	33763524	1614	SAT1_CF	blue	na
chrUn	33763544	33764350	807	SAT1_CF	blue	na
chrUn	33764362	33765038	677	SAT1_CF	blue	na
chrUn	33765087	33766062	976	SAT1_CF	blue	na
chrUn	33766104	33766611	508	SAT1_CF	blue	na
chrUn	33766625	33767026	402	SAT1_CF	blue	na
chrUn	33767066	33767568	503	SAT1_CF	blue	na
chrUn	33767581	33767688	108	SAT1_CF	blue	na
chrUn	33767710	33768263	554	SAT1_CF	blue	na
chrUn	33768275	33769729	1455	SAT1_CF	blue	na
chrUn	33769741	33769847	107	SAT1_CF	blue	na
chrUn	33769871	33770421	551	SAT1_CF	blue	na
chrUn	33770456	33771619	1164	SAT1_CF	blue	na
chrUn	33771634	33771770	137	SAT1_CF	blue	na
chrUn	33771866	33772385	520	SAT1_CF	blue	na
chrUn	33772442	33772988	547	SAT1_CF	blue	na
chrUn	33773007	33773584	578	SAT1_CF	blue	na
chrUn	33773619	33774820	1202	SAT1_CF	blue	na
chrUn	33774833	33775446	614	SAT1_CF	blue	na
chrUn	33775458	33776317	860	SAT1_CF	blue	na
chrUn	33776360	33777135	776	SAT1_CF	blue	na
chrUn	33777179	33777721	543	SAT1_CF	blue	na
chrUn	33777734	33779630	1897	SAT1_CF	blue	na
chrUn	33779685	33780290	606	SAT1_CF	blue	na
chrUn	33780306	33780706	401	SAT1_CF	blue	na
chrUn	33780727	33781245	519	SAT1_CF	blue	na
chrUn	33781258	33781366	109	SAT1_CF	blue	na
chrUn	33781387	33781932	546	SAT1_CF	blue	na
chrUn	33781975	33782939	965	SAT1_CF	blue	na
chrUn	33783018	33783565	548	SAT1_CF	blue	na
chrUn	33783576	33784181	606	SAT1_CF	blue	na
chrUn	33790906	33791105	200	Sat2CF	purple	na
chrUn	33790947	33792052	1106	Sat2CF	purple	na
chrUn	33851664	33852770	1107	Sat2CF	purple	na
chrUn	33864645	33865482	838	Sat2CF	purple	na
chrUn	33864695	33865417	723	Sat2CF	purple	na

chrUn	33877603	33877780	178	Sat2CF	purple	na
chrUn	33877672	33878841	1170	Sat2CF	purple	na
chrUn	33879240	33879674	435	CarSat1	red	na
chrUn	33879858	33879980	123	CarSat1	red	na
chrUn	33880001	33880851	851	CarSat1	red	na
chrUn	33880872	33882416	1545	CarSat1	red	na
chrUn	33882483	33883013	531	CarSat1	red	na
chrUn	33883030	33885222	2193	CarSat1	red	na
chrUn	33885227	33886736	1510	SAT1_CF	blue	na
chrUn	33886780	33887310	531	SAT1_CF	blue	na
chrUn	33887355	33888429	1075	SAT1_CF	blue	na
chrUn	33888444	33889269	826	SAT1_CF	blue	na
chrUn	33889289	33891003	1715	SAT1_CF	blue	na
chrUn	33891024	33891896	873	SAT1_CF	blue	na
chrUn	33891909	33893043	1135	SAT1_CF	blue	na
chrUn	33893029	33893749	721	CarSat1	red	na
chrUn	33893809	33894205	397	CarSat1	red	na
chrUn	33968916	33969877	962	Sat7CF	lime	na
chrUn	33993562	33994351	790	Sat7CF	lime	na
chrUn	34020824	34020961	138	SAT1_CF	blue	na
chrUn	34021027	34022456	1430	SAT1_CF	blue	na
chrUn	34022469	34023838	1370	SAT1_CF	blue	na
chrUn	34023881	34025835	1955	SAT1_CF	blue	na
chrUn	34025900	34026382	483	SAT1_CF	blue	na
chrUn	34026395	34026501	107	SAT1_CF	blue	na
chrUn	34026524	34027764	1241	SAT1_CF	blue	na
chrUn	34027807	34028914	1108	SAT1_CF	blue	na
chrUn	34028959	34029514	556	SAT1_CF	blue	na
chrUn	34029548	34029786	239	SAT1_CF	blue	na
chrUn	34029802	34030370	569	SAT1_CF	blue	na
chrUn	34036646	34037287	642	SAT1_CF	blue	na
chrUn	34037299	34038152	854	SAT1_CF	blue	na
chrUn	34038194	34038730	537	SAT1_CF	blue	na
chrUn	34038758	34039650	893	SAT1_CF	blue	na
chrUn	34039696	34040678	983	SAT1_CF	blue	na
chrUn	34040690	34041573	884	SAT1_CF	blue	na
chrUn	34041593	34042929	1337	SAT1_CF	blue	na
chrUn	34042975	34044197	1223	SAT1_CF	blue	na
chrUn	34044277	34044787	511	SAT1_CF	blue	na
chrUn	34044814	34044947	134	SAT1_CF	blue	na
chrUn	34045005	34045505	501	SAT1_CF	blue	na
chrUn	34045518	34047334	1817	SAT1_CF	blue	na

chrUn	34047451	34047878	428	SAT1_CF	blue	na
chrUn	34047891	34048496	606	SAT1_CF	blue	na
chrUn	34048539	34049087	549	SAT1_CF	blue	na
chrUn	34049164	34049710	547	SAT1_CF	blue	na
chrUn	34049834	34050985	1152	SAT1_CF	blue	na
chrUn	34051025	34051525	501	SAT1_CF	blue	na
chrUn	34051538	34051639	102	SAT1_CF	blue	na
chrUn	34051667	34052340	674	SAT1_CF	blue	na
chrUn	34052363	34052906	544	SAT1_CF	blue	na
chrUn	34052989	34053362	374	SAT1_CF	blue	na
chrUn	34053400	34053900	501	SAT1_CF	blue	na
chrUn	34053912	34054597	686	SAT1_CF	blue	na
chrUn	34054608	34055187	580	SAT1_CF	blue	na
chrUn	34055229	34057084	1856	SAT1_CF	blue	na
chrUn	34057124	34057624	501	SAT1_CF	blue	na
chrUn	34057637	34058320	684	SAT1_CF	blue	na
chrUn	34058338	34059015	678	SAT1_CF	blue	na
chrUn	34059026	34061088	2063	SAT1_CF	blue	na
chrUn	34061130	34061674	545	SAT1_CF	blue	na
chrUn	34061690	34062301	612	SAT1_CF	blue	na
chrUn	34062344	34062897	554	SAT1_CF	blue	na
chrUn	34062915	34063584	670	SAT1_CF	blue	na
chrUn	34063627	34064592	966	SAT1_CF	blue	na
chrUn	34064637	34065664	1028	SAT1_CF	blue	na
chrUn	34065707	34066200	494	SAT1_CF	blue	na
chrUn	34066220	34066871	652	SAT1_CF	blue	na
chrUn	34066889	34067744	856	SAT1_CF	blue	na
chrUn	34067756	34069352	1597	SAT1_CF	blue	na
chrUn	34069364	34071633	2270	SAT1_CF	blue	na
chrUn	34071666	34072014	349	SAT1_CF	blue	na
chrUn	34072070	34072571	502	SAT1_CF	blue	na
chrUn	34072583	34073265	683	SAT1_CF	blue	na
chrUn	34073288	34075336	2049	SAT1_CF	blue	na
chrUn	34075379	34075880	502	SAT1_CF	blue	na
chrUn	34075893	34076572	680	SAT1_CF	blue	na
chrUn	34076596	34077256	661	SAT1_CF	blue	na
chrUn	34077304	34080110	2807	SAT1_CF	blue	na
chrUn	34080124	34081718	1595	SAT1_CF	blue	na
chrUn	34081769	34082331	563	SAT1_CF	blue	na
chrUn	34083744	34084311	568	SAT1_CF	blue	na
chrUn	34084357	34084850	494	SAT1_CF	blue	na
chrUn	34084880	34085552	673	SAT1_CF	blue	na

chrUn	34085564	34085673	110	SAT1_CF	blue	na
chrUn	34085693	34086239	547	SAT1_CF	blue	na
chrUn	34086283	34087394	1112	SAT1_CF	blue	na
chrUn	34087437	34088022	586	SAT1_CF	blue	na
chrUn	34088034	34089261	1228	SAT1_CF	blue	na
chrUn	34089348	34089849	502	SAT1_CF	blue	na
chrUn	34089863	34090546	684	SAT1_CF	blue	na
chrUn	34090558	34091241	684	SAT1_CF	blue	na
chrUn	34091252	34091744	493	SAT1_CF	blue	na
chrUn	34091758	34092482	725	SAT1_CF	blue	na
chrUn	34092494	34092564	71	SAT1_CF	blue	na
chrUn	34092589	34092736	148	SAT1_CF	blue	na
chrUn	34092763	34093734	972	SAT1_CF	blue	na
chrUn	34093808	34094182	375	SAT1_CF	blue	na
chrUn	34094218	34094805	588	SAT1_CF	blue	na
chrUn	34094861	34095375	515	SAT1_CF	blue	na
chrUn	34095388	34095480	93	SAT1_CF	blue	na
chrUn	34095491	34096477	987	SAT1_CF	blue	na
chrUn	34096503	34098007	1505	SAT1_CF	blue	na
chrUn	34098052	34099215	1164	SAT1_CF	blue	na
chrUn	34099227	34099737	511	SAT1_CF	blue	na
chrUn	34099817	34101802	1986	SAT1_CF	blue	na
chrUn	34101848	34102349	502	SAT1_CF	blue	na
chrUn	34102362	34103732	1371	SAT1_CF	blue	na
chrUn	34103774	34104677	904	SAT1_CF	blue	na
chrUn	34104728	34105275	548	SAT1_CF	blue	na
chrUn	34105392	34105902	511	SAT1_CF	blue	na
chrUn	34105929	34106062	134	SAT1_CF	blue	na
chrUn	34106120	34106620	501	SAT1_CF	blue	na
chrUn	34106633	34107317	685	SAT1_CF	blue	na
chrUn	34107329	34107438	110	SAT1_CF	blue	na
chrUn	34107458	34108004	547	SAT1_CF	blue	na
chrUn	34108017	34108237	221	SAT1_CF	blue	na
chrUn	34108265	34109326	1062	SAT1_CF	blue	na
chrUn	34109353	34109487	135	SAT1_CF	blue	na
chrUn	34109545	34110046	502	SAT1_CF	blue	na
chrUn	34110058	34110741	684	SAT1_CF	blue	na
chrUn	34110753	34111249	497	SAT1_CF	blue	na
chrUn	34111289	34111837	549	SAT1_CF	blue	na
chrUn	34112281	34114139	1859	SAT1_CF	blue	na
chrUn	34114204	34116024	1821	SAT1_CF	blue	na
chrUn	34116067	34116607	541	SAT1_CF	blue	na

chrUn	34116627	34117341	715	SAT1_CF	blue	na
chrUn	34117420	34117944	525	SAT1_CF	blue	na
chrUn	34117998	34118076	79	SAT1_CF	blue	na
chrUn	34118134	34118634	501	SAT1_CF	blue	na
chrUn	34118645	34119331	687	SAT1_CF	blue	na
chrUn	34119343	34120017	675	SAT1_CF	blue	na
chrUn	34120060	34120448	389	SAT1_CF	blue	na
chrUn	34224205	34225834	1630	CarSat1	red	na
chrUn	34225864	34231989	6126	CarSat1	red	na
chrUn	34232000	34232975	976	CarSat1	red	na
chrUn	34232999	34233711	713	CarSat1	red	na
chrUn	34233744	34234449	706	CarSat1	red	na
chrUn	34234473	34236141	1669	CarSat1	red	na
chrUn	34236171	34236657	487	CarSat1	red	na
chrUn	34236691	34237395	705	CarSat1	red	na
chrUn	34237419	34238353	935	CarSat1	red	na
chrUn	34238383	34238869	487	CarSat1	red	na
chrUn	34238903	34239607	705	CarSat1	red	na
chrUn	34239631	34242034	2404	CarSat1	red	na
chrUn	34242064	34246966	4903	CarSat1	red	na
chrUn	34246990	34250649	3660	CarSat1	red	na
chrUn	34250683	34255288	4606	CarSat1	red	na
chrUn	34255318	34256539	1222	CarSat1	red	na
chrUn	34256564	34262420	5857	CarSat1	red	na
chrUn	34262444	34262911	468	CarSat1	red	na
chrUn	34262937	34264850	1914	CarSat1	red	na
chrUn	34264880	34266102	1223	CarSat1	red	na
chrUn	34266124	34270006	3883	CarSat1	red	na
chrUn	34270036	34270744	709	CarSat1	red	na
chrUn	34270759	34270885	127	Carsat2	orange	na
chrUn	34270774	34276919	6146	CarSat1	red	na
chrUn	34276948	34277647	700	CarSat1	red	na
chrUn	34277684	34278661	978	CarSat1	red	na
chrUn	34278698	34279126	429	CarSat1	red	na
chrUn	34279141	34279249	109	Carsat2	orange	na
chrUn	34279156	34286444	7289	CarSat1	red	na
chrUn	34287205	34289437	2233	CarSat1	red	na
chrUn	34289467	34290170	704	CarSat1	red	na
chrUn	34290201	34290903	703	CarSat1	red	na
chrUn	34290933	34291760	828	CarSat1	red	na
chrUn	34291945	34292579	635	CarSat1	red	na
chrUn	34292612	34293307	696	CarSat1	red	na

chrUn	34293323	34294044	722	CarSat1	red	na
chrUn	34294073	34294990	918	CarSat1	red	na
chrUn	34295020	34296163	1144	CarSat1	red	na
chrUn	34303887	34305042	1156	Sat2CF	purple	na
chrUn	34319830	34321046	1217	Sat2CF	purple	na
chrUn	34319958	34321041	1084	Sat2CF	purple	na
chrUn	34323237	34323663	427	CarSat1	red	na
chrUn	34323697	34324418	722	CarSat1	red	na
chrUn	34324404	34325562	1159	SAT1_CF	blue	na
chrUn	34325584	34325985	402	SAT1_CF	blue	na
chrUn	34325999	34328044	2046	SAT1_CF	blue	na
chrUn	34328087	34329731	1645	SAT1_CF	blue	na
chrUn	34329774	34330501	728	SAT1_CF	blue	na
chrUn	34330545	34332057	1513	SAT1_CF	blue	na
chrUn	34332122	34332279	158	SAT1_CF	blue	na
chrUn	34332294	34333329	1036	SAT1_CF	blue	na
chrUn	34333372	34334047	676	SAT1_CF	blue	na
chrUn	34334059	34334535	477	SAT1_CF	blue	na
chrUn	34334600	34335216	617	SAT1_CF	blue	na
chrUn	34335231	34336340	1110	SAT1_CF	blue	na
chrUn	34336352	34337471	1120	SAT1_CF	blue	na
chrUn	34337511	34338083	573	SAT1_CF	blue	na
chrUn	34339499	34340061	563	SAT1_CF	blue	na
chrUn	34340075	34340481	407	SAT1_CF	blue	na
chrUn	34340492	34341705	1214	SAT1_CF	blue	na
chrUn	34341719	34342211	493	SAT1_CF	blue	na
chrUn	34342222	34342905	684	SAT1_CF	blue	na
chrUn	34342917	34343600	684	SAT1_CF	blue	na
chrUn	34343613	34346422	2810	SAT1_CF	blue	na
chrUn	34346499	34347361	863	SAT1_CF	blue	na
chrUn	34347373	34347925	553	SAT1_CF	blue	na
chrUn	34347954	34348834	881	SAT1_CF	blue	na
chrUn	34348868	34350243	1376	SAT1_CF	blue	na
chrUn	34350287	34351619	1333	SAT1_CF	blue	na
chrUn	34351631	34352236	606	SAT1_CF	blue	na
chrUn	34352249	34352750	502	SAT1_CF	blue	na
chrUn	34352817	34353954	1138	SAT1_CF	blue	na
chrUn	34353998	34354643	646	SAT1_CF	blue	na
chrUn	34354656	34355176	521	SAT1_CF	blue	na
chrUn	34355231	34355333	103	SAT1_CF	blue	na
chrUn	34355345	34355821	477	SAT1_CF	blue	na
chrUn	34355842	34355951	110	SAT1_CF	blue	na

chrUn	34355963	34357157	1195	SAT1_CF	blue	na
chrUn	34357246	34358134	889	SAT1_CF	blue	na
chrUn	34358157	34358354	198	SAT1_CF	blue	na
chrUn	34358476	34359021	546	SAT1_CF	blue	na
chrUn	34359034	34359150	117	SAT1_CF	blue	na
chrUn	34359162	34361285	2124	SAT1_CF	blue	na
chrUn	34361297	34361981	685	SAT1_CF	blue	na
chrUn	34361993	34362494	502	SAT1_CF	blue	na
chrUn	34362538	34363493	956	SAT1_CF	blue	na
chrUn	34363538	34364537	1000	SAT1_CF	blue	na
chrUn	34364579	34365786	1208	SAT1_CF	blue	na
chrUn	34365809	34365917	109	SAT1_CF	blue	na
chrUn	34365929	34366446	518	SAT1_CF	blue	na
chrUn	34366480	34366960	481	SAT1_CF	blue	na
chrUn	34367027	34367982	956	SAT1_CF	blue	na
chrUn	34368025	34368691	667	SAT1_CF	blue	na
chrUn	34368714	34369544	831	SAT1_CF	blue	na
chrUn	34369558	34370077	520	SAT1_CF	blue	na
chrUn	34370103	34373200	3098	SAT1_CF	blue	na
chrUn	34373221	34373434	214	SAT1_CF	blue	na
chrUn	34374094	34374482	389	SAT1_CF	blue	na
chrUn	34375899	34376461	563	SAT1_CF	blue	na
chrUn	34376475	34377430	956	SAT1_CF	blue	na
chrUn	34377509	34378464	956	SAT1_CF	blue	na
chrUn	34378507	34379048	542	SAT1_CF	blue	na
chrUn	34379270	34380100	831	SAT1_CF	blue	na
chrUn	34380114	34381787	1674	SAT1_CF	blue	na
chrUn	34381807	34381916	110	SAT1_CF	blue	na
chrUn	34381928	34382612	685	SAT1_CF	blue	na
chrUn	34382626	34383120	495	SAT1_CF	blue	na
chrUn	34383146	34384171	1026	SAT1_CF	blue	na
chrUn	34384250	34385212	963	SAT1_CF	blue	na
chrUn	34385255	34385929	675	SAT1_CF	blue	na
chrUn	34385941	34386547	607	SAT1_CF	blue	na
chrUn	34386560	34387060	501	SAT1_CF	blue	na
chrUn	34387100	34388060	961	SAT1_CF	blue	na
chrUn	34388104	34389381	1278	SAT1_CF	blue	na
chrUn	34389394	34390247	854	SAT1_CF	blue	na
chrUn	34390259	34391054	796	SAT1_CF	blue	na
chrUn	34391068	34391228	161	SAT1_CF	blue	na
chrUn	34391244	34392687	1444	SAT1_CF	blue	na
chrUn	34392698	34393359	662	SAT1_CF	blue	na

chrUn	34393399	34393892	494	SAT1_CF	blue	na
chrUn	34393932	34394888	957	SAT1_CF	blue	na
chrUn	34394971	34395725	755	SAT1_CF	blue	na
chrUn	34395795	34397153	1359	SAT1_CF	blue	na
chrUn	34397186	34398932	1747	SAT1_CF	blue	na
chrUn	34398946	34399447	502	SAT1_CF	blue	na
chrUn	34399523	34401033	1511	SAT1_CF	blue	na
chrUn	34401045	34402803	1759	SAT1_CF	blue	na
chrUn	34402819	34404068	1250	SAT1_CF	blue	na
chrUn	34404087	34404696	610	SAT1_CF	blue	na
chrUn	34404717	34405118	402	SAT1_CF	blue	na
chrUn	34405129	34406238	1110	SAT1_CF	blue	na
chrUn	34406254	34406794	541	SAT1_CF	blue	na
chrUn	34406836	34407511	676	SAT1_CF	blue	na
chrUn	34407523	34408205	683	SAT1_CF	blue	na
chrUn	34408218	34409641	1424	SAT1_CF	blue	na
chrUn	34409654	34410723	1070	SAT1_CF	blue	na
chrUn	34410765	34411926	1162	SAT1_CF	blue	na
chrUn	34411972	34412976	1005	SAT1_CF	blue	na
chrUn	34413042	34414006	965	SAT1_CF	blue	na
chrUn	34414049	34414724	676	SAT1_CF	blue	na
chrUn	34414736	34415892	1157	SAT1_CF	blue	na
chrUn	34415905	34416916	1012	SAT1_CF	blue	na
chrUn	34521941	34523272	1332	SAT1_CF	blue	na
chrUn	34523310	34523860	551	SAT1_CF	blue	na
chrUn	34523901	34524645	745	SAT1_CF	blue	na
chrUn	34524667	34525290	624	SAT1_CF	blue	na
chrUn	34525330	34526275	946	SAT1_CF	blue	na
chrUn	34526315	34526525	211	SAT1_CF	blue	na
chrUn	34526567	34528412	1846	SAT1_CF	blue	na
chrUn	34528417	34529586	1170	CarSat1	red	na
chrUn	34529775	34530540	766	CarSat1	red	na
chrUn	34530558	34531332	775	CarSat1	red	na
chrUn	34531353	34532943	1591	CarSat1	red	na
chrUn	34533043	34533819	777	CarSat1	red	na
chrUn	34533840	34534364	525	CarSat1	red	na
chrUn	34534764	34535932	1169	Sat2CF	purple	na
chrUn	34535824	34536001	178	Sat2CF	purple	na
chrUn	34546445	34546811	367	Sat2CF	purple	na
chrUn	34546451	34546923	473	Sat2CF	purple	na
chrUn	34546526	34546870	345	Sat2CF	purple	na
chrUn	34558697	34559639	943	Sat2CF	purple	na

chrUn	34558843	34559780	938	Sat2CF	purple	na
chrUn	34567855	34569157	1303	CarSat1	red	na
chrUn	34569178	34570029	852	CarSat1	red	na
chrUn	34570050	34571290	1241	CarSat1	red	na
chrUn	34571328	34572209	882	CarSat1	red	na
chrUn	34572204	34573412	1209	SAT1_CF	blue	na
chrUn	34573442	34575025	1584	SAT1_CF	blue	na
chrUn	34575041	34575260	220	SAT1_CF	blue	na
chrUn	34575275	34576506	1232	SAT1_CF	blue	na
chrUn	34576519	34577061	543	SAT1_CF	blue	na
chrUn	34577386	34577541	156	SAT1_CF	blue	na
chrUn	34577585	34578103	519	SAT1_CF	blue	na
chrUn	34584459	34586402	1944	SAT1_CF	blue	na
chrUn	34586430	34586942	513	SAT1_CF	blue	na
chrUn	34587020	34587569	550	SAT1_CF	blue	na
chrUn	34587612	34588654	1043	SAT1_CF	blue	na
chrUn	34588698	34590087	1390	SAT1_CF	blue	na
chrUn	34590141	34590629	489	SAT1_CF	blue	na
chrUn	34590658	34590876	219	SAT1_CF	blue	na
chrUn	34590893	34592867	1975	SAT1_CF	blue	na
chrUn	34592872	34595588	2717	CarSat1	red	na
chrUn	34595607	34596564	958	CarSat1	red	na
chrUn	34618453	34619528	1076	SAT1_CF	blue	na
chrUn	34619572	34620759	1188	SAT1_CF	blue	na
chrUn	34620802	34621528	727	SAT1_CF	blue	na
chrUn	34621573	34622107	535	SAT1_CF	blue	na
chrUn	34622129	34622646	518	SAT1_CF	blue	na
chrUn	34622658	34623975	1318	SAT1_CF	blue	na
chrUn	34623988	34624823	836	SAT1_CF	blue	na
chrUn	34624836	34625539	704	SAT1_CF	blue	na
chrUn	34625551	34626228	678	SAT1_CF	blue	na
chrUn	34626271	34628092	1822	SAT1_CF	blue	na
chrUn	34628132	34628634	503	SAT1_CF	blue	na
chrUn	34628646	34629835	1190	SAT1_CF	blue	na
chrUn	34629848	34630456	609	SAT1_CF	blue	na
chrUn	34630504	34631051	548	SAT1_CF	blue	na
chrUn	34633900	34634610	711	SAT1_CF	blue	na
chrUn	34634622	34635299	678	SAT1_CF	blue	na
chrUn	34635342	34635883	542	SAT1_CF	blue	na
chrUn	34635897	34637194	1298	SAT1_CF	blue	na
chrUn	34637222	34637924	703	SAT1_CF	blue	na
chrUn	34637937	34639161	1225	SAT1_CF	blue	na

chrUn	34639222	34639969	748	SAT1_CF	blue	na
chrUn	34639984	34640120	137	SAT1_CF	blue	na
chrUn	34640186	34640736	551	SAT1_CF	blue	na
chrUn	34640763	34641306	544	SAT1_CF	blue	na
chrUn	34641348	34641526	179	SAT1_CF	blue	na
chrUn	34641574	34642535	962	SAT1_CF	blue	na
chrUn	34642603	34643121	519	SAT1_CF	blue	na
chrUn	34643134	34643817	684	SAT1_CF	blue	na
chrUn	34643829	34644674	846	SAT1_CF	blue	na
chrUn	34644715	34645826	1112	SAT1_CF	blue	na
chrUn	34645867	34646379	513	SAT1_CF	blue	na
chrUn	34646407	34647106	700	SAT1_CF	blue	na
chrUn	34647119	34647800	682	SAT1_CF	blue	na
chrUn	34647814	34648674	861	SAT1_CF	blue	na
chrUn	34648716	34649261	546	SAT1_CF	blue	na
chrUn	34649303	34649504	202	SAT1_CF	blue	na
chrUn	34649685	34649870	186	SAT1_CF	blue	na
chrUn	34649906	34650405	500	SAT1_CF	blue	na
chrUn	34650418	34650525	108	SAT1_CF	blue	na
chrUn	34650546	34651528	983	SAT1_CF	blue	na
chrUn	34651615	34652797	1183	SAT1_CF	blue	na
chrUn	34652820	34653770	951	SAT1_CF	blue	na
chrUn	34653798	34656428	2631	SAT1_CF	blue	na
chrUn	34656471	34657319	849	SAT1_CF	blue	na
chrUn	34657339	34657861	523	SAT1_CF	blue	na
chrUn	34657874	34658557	684	SAT1_CF	blue	na
chrUn	34658569	34659371	803	SAT1_CF	blue	na
chrUn	34659394	34660608	1215	SAT1_CF	blue	na
chrUn	34660684	34661225	542	SAT1_CF	blue	na
chrUn	34661236	34661348	113	SAT1_CF	blue	na
chrUn	34661362	34662055	694	SAT1_CF	blue	na
chrUn	34662066	34662469	404	SAT1_CF	blue	na
chrUn	34662488	34663006	519	SAT1_CF	blue	na
chrUn	34663019	34663703	685	SAT1_CF	blue	na
chrUn	34663715	34665055	1341	SAT1_CF	blue	na
chrUn	34665073	34665750	678	SAT1_CF	blue	na
chrUn	34665762	34665871	110	SAT1_CF	blue	na
chrUn	34665892	34666365	474	SAT1_CF	blue	na
chrUn	34666392	34666980	589	SAT1_CF	blue	na
chrUn	34667007	34667681	675	SAT1_CF	blue	na
chrUn	34667725	34668800	1076	SAT1_CF	blue	na
chrUn	34668880	34669803	924	SAT1_CF	blue	na

chrUn	34669863	34670794	932	SAT1_CF	blue	na
chrUn	34670816	34671992	1177	SAT1_CF	blue	na
chrUn	34672073	34672304	232	SAT1_CF	blue	na
chrUn	34672352	34672845	494	SAT1_CF	blue	na
chrUn	34672865	34673547	683	SAT1_CF	blue	na
chrUn	34673571	34675085	1515	SAT1_CF	blue	na
chrUn	34675149	34675379	231	SAT1_CF	blue	na
chrUn	34675412	34676961	1550	SAT1_CF	blue	na
chrUn	34676980	34678470	1491	SAT1_CF	blue	na
chrUn	34678549	34679463	915	SAT1_CF	blue	na
chrUn	34679550	34680050	501	SAT1_CF	blue	na
chrUn	34680063	34680744	682	SAT1_CF	blue	na
chrUn	34680783	34682242	1460	SAT1_CF	blue	na
chrUn	34682285	34682809	525	SAT1_CF	blue	na
chrUn	34682824	34682966	143	SAT1_CF	blue	na
chrUn	34683026	34683520	495	SAT1_CF	blue	na
chrUn	34683540	34683646	107	SAT1_CF	blue	na
chrUn	34683669	34684219	551	SAT1_CF	blue	na
chrUn	34684242	34684908	667	SAT1_CF	blue	na
chrUn	34684951	34685580	630	SAT1_CF	blue	na
chrUn	34685608	34686800	1193	SAT1_CF	blue	na
chrUn	34686828	34686966	139	SAT1_CF	blue	na
chrUn	34687011	34687532	522	SAT1_CF	blue	na
chrUn	34687554	34689267	1714	SAT1_CF	blue	na
chrUn	34689382	34689931	550	SAT1_CF	blue	na
chrUn	34689945	34691310	1366	SAT1_CF	blue	na
chrUn	34691410	34692533	1124	SAT1_CF	blue	na
chrUn	34692555	34693217	663	SAT1_CF	blue	na
chrUn	34693260	34694149	890	SAT1_CF	blue	na
chrUn	34694163	34695755	1593	SAT1_CF	blue	na
chrUn	34695767	34696621	855	SAT1_CF	blue	na
chrUn	34696634	34697979	1346	SAT1_CF	blue	na
chrUn	34698076	34698623	548	SAT1_CF	blue	na
chrUn	34698669	34699633	965	SAT1_CF	blue	na
chrUn	34699646	34699752	107	SAT1_CF	blue	na
chrUn	34699775	34700348	574	SAT1_CF	blue	na
chrUn	34700360	34701122	763	SAT1_CF	blue	na
chrUn	34701136	34701206	71	SAT1_CF	blue	na
chrUn	34701229	34701781	553	SAT1_CF	blue	na
chrUn	34701793	34703122	1330	SAT1_CF	blue	na
chrUn	34703166	34704552	1387	SAT1_CF	blue	na
chrUn	34704588	34705088	501	SAT1_CF	blue	na

chrUn	34705133	34705785	653	SAT1_CF	blue	na
chrUn	34705797	34709119	3323	SAT1_CF	blue	na
chrUn	34709161	34709326	166	SAT1_CF	blue	na
chrUn	34709508	34709693	186	SAT1_CF	blue	na
chrUn	34709710	34710230	521	SAT1_CF	blue	na
chrUn	34710257	34710495	239	SAT1_CF	blue	na
chrUn	34711496	34712382	887	SAT1_CF	blue	na
chrUn	34712394	34712933	540	SAT1_CF	blue	na
chrUn	34712977	34713634	658	SAT1_CF	blue	na
chrUn	34713678	34715284	1607	SAT1_CF	blue	na
chrUn	34715302	34715415	114	SAT1_CF	blue	na
chrUn	34715427	34716272	846	SAT1_CF	blue	na
chrUn	34716303	34717895	1593	SAT1_CF	blue	na
chrUn	34717939	34718615	677	SAT1_CF	blue	na
chrUn	34718627	34719147	521	SAT1_CF	blue	na
chrUn	34719168	34721188	2021	SAT1_CF	blue	na
chrUn	34721219	34721739	521	SAT1_CF	blue	na
chrUn	34721856	34723664	1809	SAT1_CF	blue	na
chrUn	34723706	34725158	1453	SAT1_CF	blue	na
chrUn	34725169	34725854	686	SAT1_CF	blue	na
chrUn	34725866	34726547	682	SAT1_CF	blue	na
chrUn	34726561	34727226	666	SAT1_CF	blue	na
chrUn	34727241	34728642	1402	SAT1_CF	blue	na
chrUn	34728654	34730401	1748	SAT1_CF	blue	na
chrUn	34730413	34730972	560	SAT1_CF	blue	na
chrUn	34730992	34731100	109	SAT1_CF	blue	na
chrUn	34731113	34731633	521	SAT1_CF	blue	na
chrUn	34731650	34731835	186	SAT1_CF	blue	na
chrUn	34732016	34732180	165	SAT1_CF	blue	na
chrUn	34732222	34733658	1437	SAT1_CF	blue	na
chrUn	34733682	34735547	1866	SAT1_CF	blue	na
chrUn	34735559	34736061	503	SAT1_CF	blue	na
chrUn	34736147	34736321	175	SAT1_CF	blue	na
chrUn	34736433	34737679	1247	SAT1_CF	blue	na
chrUn	34737691	34738242	552	SAT1_CF	blue	na
chrUn	34738264	34738336	73	SAT1_CF	blue	na
chrUn	34738349	34739277	929	SAT1_CF	blue	na
chrUn	34739289	34739972	684	SAT1_CF	blue	na
chrUn	34739984	34741142	1159	SAT1_CF	blue	na
chrUn	34741208	34741344	137	SAT1_CF	blue	na
chrUn	34741372	34742564	1193	SAT1_CF	blue	na
chrUn	34742592	34743221	630	SAT1_CF	blue	na

chrUn	34743264	34743930	667	SAT1_CF	blue	na
chrUn	34743953	34744635	683	SAT1_CF	blue	na
chrUn	34744655	34745149	495	SAT1_CF	blue	na
chrUn	34745189	34745762	574	SAT1_CF	blue	na
chrUn	34747160	34747754	595	SAT1_CF	blue	na
chrUn	34747803	34748448	646	SAT1_CF	blue	na
chrUn	34748490	34748802	313	SAT1_CF	blue	na
chrUn	34748844	34749344	501	SAT1_CF	blue	na
chrUn	34749384	34749783	400	SAT1_CF	blue	na
chrUn	34749794	34751421	1628	SAT1_CF	blue	na
chrUn	34751464	34752136	673	SAT1_CF	blue	na
chrUn	34752150	34753651	1502	SAT1_CF	blue	na
chrUn	34753730	34754687	958	SAT1_CF	blue	na
chrUn	34754735	34755410	676	SAT1_CF	blue	na
chrUn	34755422	34757364	1943	SAT1_CF	blue	na
chrUn	34757408	34758536	1129	SAT1_CF	blue	na
chrUn	34758548	34759221	674	SAT1_CF	blue	na
chrUn	34759242	34759743	502	SAT1_CF	blue	na
chrUn	34759788	34759945	158	SAT1_CF	blue	na
chrUn	34760010	34760558	549	SAT1_CF	blue	na
chrUn	34760618	34760796	179	SAT1_CF	blue	na
chrUn	34760838	34761382	545	SAT1_CF	blue	na
chrUn	34761425	34763089	1665	SAT1_CF	blue	na
chrUn	34763131	34763678	548	SAT1_CF	blue	na
chrUn	34763735	34764689	955	SAT1_CF	blue	na
chrUn	34764734	34766181	1448	SAT1_CF	blue	na
chrUn	34766223	34767083	861	SAT1_CF	blue	na
chrUn	34767095	34767629	535	SAT1_CF	blue	na
chrUn	34767679	34768469	791	SAT1_CF	blue	na
chrUn	34768512	34769372	861	SAT1_CF	blue	na
chrUn	34769384	34770757	1374	SAT1_CF	blue	na
chrUn	34770784	34771285	502	SAT1_CF	blue	na
chrUn	34771331	34772022	692	SAT1_CF	blue	na
chrUn	34772040	34772700	661	SAT1_CF	blue	na
chrUn	34772728	34773353	626	SAT1_CF	blue	na
chrUn	34773401	34774077	677	SAT1_CF	blue	na
chrUn	34774089	34776154	2066	SAT1_CF	blue	na
chrUn	34776197	34776741	545	SAT1_CF	blue	na
chrUn	34776763	34777514	752	SAT1_CF	blue	na
chrUn	34777535	34777644	110	SAT1_CF	blue	na
chrUn	34777656	34778158	503	SAT1_CF	blue	na
chrUn	34778244	34779574	1331	SAT1_CF	blue	na

chrUn	34779618	34780949	1332	SAT1_CF	blue	na
chrUn	34780961	34781514	554	SAT1_CF	blue	na
chrUn	34781539	34781643	105	SAT1_CF	blue	na
chrUn	34781656	34782156	501	SAT1_CF	blue	na
chrUn	34782196	34782597	402	SAT1_CF	blue	na
chrUn	34782608	34783893	1286	SAT1_CF	blue	na
chrUn	34783950	34784022	73	SAT1_CF	blue	na
chrUn	34784034	34784536	503	SAT1_CF	blue	na
chrUn	34784549	34785496	948	SAT1_CF	blue	na
chrUn	34785577	34786574	998	SAT1_CF	blue	na
chrUn	34786619	34787164	546	SAT1_CF	blue	na
chrUn	34787184	34787292	109	SAT1_CF	blue	na
chrUn	34787304	34787910	607	SAT1_CF	blue	na
chrUn	34787923	34789628	1706	SAT1_CF	blue	na
chrUn	34789672	34790315	644	SAT1_CF	blue	na
chrUn	34790328	34791570	1243	SAT1_CF	blue	na
chrUn	34791026	34793996	2971	SAT1_CF	blue	na
chrUn	34794030	34795663	1634	SAT1_CF	blue	na
chrUn	34795723	34797578	1856	SAT1_CF	blue	na
chrUn	34797621	34798295	675	SAT1_CF	blue	na
chrUn	34798308	34798809	502	SAT1_CF	blue	na
chrUn	34798845	34799244	400	SAT1_CF	blue	na
chrUn	34897708	34898288	581	SAT1_CF	blue	na
chrUn	34898301	34899892	1592	SAT1_CF	blue	na
chrUn	34899905	34900425	521	SAT1_CF	blue	na
chrUn	34900446	34901418	973	SAT1_CF	blue	na
chrUn	34901441	34902435	995	SAT1_CF	blue	na
chrUn	34902478	34903153	676	SAT1_CF	blue	na
chrUn	34903165	34903778	614	SAT1_CF	blue	na
chrUn	34903791	34904290	500	SAT1_CF	blue	na
chrUn	34904330	34904511	182	SAT1_CF	blue	na
chrUn	34904692	34905483	792	SAT1_CF	blue	na
chrUn	34905527	34906673	1147	SAT1_CF	blue	na
chrUn	34906697	34907458	762	SAT1_CF	blue	na
chrUn	34907471	34907973	503	SAT1_CF	blue	na
chrUn	34908009	34908194	186	SAT1_CF	blue	na
chrUn	34908374	34909208	835	SAT1_CF	blue	na
chrUn	34909220	34909903	684	SAT1_CF	blue	na
chrUn	34909917	34910416	500	SAT1_CF	blue	na
chrUn	34910453	34910824	372	SAT1_CF	blue	na
chrUn	34910871	34911414	544	SAT1_CF	blue	na
chrUn	34911455	34911799	345	SAT1_CF	blue	na

chrUn	34911842	34913176	1335	SAT1_CF	blue	na
chrUn	34913188	34913944	757	SAT1_CF	blue	na
chrUn	34913971	34915098	1128	SAT1_CF	blue	na
chrUn	34915115	34915488	374	SAT1_CF	blue	na
chrUn	34915531	34916053	523	SAT1_CF	blue	na
chrUn	34916085	34917157	1073	SAT1_CF	blue	na
chrUn	34917200	34917744	545	SAT1_CF	blue	na
chrUn	34917800	34918467	668	SAT1_CF	blue	na
chrUn	34918479	34919109	631	SAT1_CF	blue	na
chrUn	34919130	34919239	110	SAT1_CF	blue	na
chrUn	34919251	34919753	503	SAT1_CF	blue	na
chrUn	34919793	34921275	1483	SAT1_CF	blue	na
chrUn	34921292	34922645	1354	SAT1_CF	blue	na
chrUn	34922659	34923419	761	SAT1_CF	blue	na
chrUn	34923431	34924112	682	SAT1_CF	blue	na
chrUn	34924125	34924626	502	SAT1_CF	blue	na
chrUn	34924670	34924827	158	SAT1_CF	blue	na
chrUn	34924855	34925367	513	SAT1_CF	blue	na
chrUn	34925410	34926745	1336	SAT1_CF	blue	na
chrUn	34926792	34926929	138	SAT1_CF	blue	na
chrUn	34926956	34927467	512	SAT1_CF	blue	na
chrUn	34927485	34928806	1322	SAT1_CF	blue	na
chrUn	34928849	34929524	676	SAT1_CF	blue	na
chrUn	34929536	34930168	633	SAT1_CF	blue	na
chrUn	34930190	34930293	104	SAT1_CF	blue	na
chrUn	34930311	34930812	502	SAT1_CF	blue	na
chrUn	34931007	34931408	402	SAT1_CF	blue	na
chrUn	34931419	34933703	2285	SAT1_CF	blue	na
chrUn	34933773	34934625	853	SAT1_CF	blue	na
chrUn	34934666	34935168	503	SAT1_CF	blue	na
chrUn	34935224	34935599	376	SAT1_CF	blue	na
chrUn	34935635	34936544	910	SAT1_CF	blue	na
chrUn	34936556	34937589	1034	SAT1_CF	blue	na
chrUn	34937615	34938119	505	SAT1_CF	blue	na
chrUn	34938196	34939346	1151	SAT1_CF	blue	na
chrUn	34939364	34939861	498	SAT1_CF	blue	na
chrUn	34939909	34940860	952	SAT1_CF	blue	na
chrUn	34940891	34941787	897	SAT1_CF	blue	na
chrUn	34941829	34942685	857	SAT1_CF	blue	na
chrUn	34942697	34943239	543	SAT1_CF	blue	na
chrUn	34943276	34943493	218	SAT1_CF	blue	na
chrUn	34943544	34944182	639	SAT1_CF	blue	na

chrUn	34944194	34944901	708	SAT1_CF	blue	na
chrUn	34944970	34946550	1581	SAT1_CF	blue	na
chrUn	34946593	34947135	543	SAT1_CF	blue	na
chrUn	34947147	34947256	110	SAT1_CF	blue	na
chrUn	34947276	34948479	1204	SAT1_CF	blue	na
chrUn	34948523	34949010	488	SAT1_CF	blue	na
chrUn	34949023	34949524	502	SAT1_CF	blue	na
chrUn	34949537	34950220	684	SAT1_CF	blue	na
chrUn	34950232	34950903	672	SAT1_CF	blue	na
chrUn	34950930	34951662	733	SAT1_CF	blue	na
chrUn	34951742	34952321	580	SAT1_CF	blue	na
chrUn	34952348	34952438	91	SAT1_CF	blue	na
chrUn	34952462	34953701	1240	SAT1_CF	blue	na
chrUn	34953744	34954706	963	SAT1_CF	blue	na
chrUn	34954732	34955559	828	SAT1_CF	blue	na
chrUn	34955604	34956105	502	SAT1_CF	blue	na
chrUn	34956117	34956225	109	SAT1_CF	blue	na
chrUn	34956248	34956836	589	SAT1_CF	blue	na
chrUn	34957420	34957860	441	SAT1_CF	blue	na
chrUn	34957873	34958374	502	SAT1_CF	blue	na
chrUn	34958387	34958992	606	SAT1_CF	blue	na
chrUn	34960387	34960949	563	SAT1_CF	blue	na
chrUn	34960963	34962594	1632	SAT1_CF	blue	na
chrUn	34962608	34962875	268	SAT1_CF	blue	na
chrUn	34962902	34963481	580	SAT1_CF	blue	na
chrUn	34963551	34964040	490	SAT1_CF	blue	na
chrUn	34964120	34965017	898	SAT1_CF	blue	na
chrUn	34965075	34965153	79	SAT1_CF	blue	na
chrUn	34965207	34965730	524	SAT1_CF	blue	na
chrUn	34965809	34966345	537	SAT1_CF	blue	na
chrUn	34966414	34967053	640	SAT1_CF	blue	na
chrUn	34967066	34967734	669	SAT1_CF	blue	na
chrUn	34967752	34968307	556	SAT1_CF	blue	na
chrUn	34968329	34968435	107	SAT1_CF	blue	na
chrUn	34968448	34968953	506	SAT1_CF	blue	na
chrUn	34969013	34969155	143	SAT1_CF	blue	na
chrUn	34969170	34970781	1612	SAT1_CF	blue	na
chrUn	34970802	34971535	734	SAT1_CF	blue	na
chrUn	34971644	34973132	1489	SAT1_CF	blue	na
chrUn	34973158	34973660	503	SAT1_CF	blue	na
chrUn	34973685	34973755	71	SAT1_CF	blue	na
chrUn	34973767	34974487	721	SAT1_CF	blue	na

chrUn	34974501	34975392	892	SAT1_CF	blue	na
chrUn	34975404	34976431	1028	SAT1_CF	blue	na
chrUn	34976451	34976559	109	SAT1_CF	blue	na
chrUn	34976571	34977172	602	SAT1_CF	blue	na
chrUn	34977189	34977903	715	SAT1_CF	blue	na
chrUn	34977915	34979606	1692	SAT1_CF	blue	na
chrUn	34979649	34980319	671	SAT1_CF	blue	na
chrUn	34980337	34980890	554	SAT1_CF	blue	na
chrUn	34980915	34981019	105	SAT1_CF	blue	na
chrUn	34981039	34981549	511	SAT1_CF	blue	na
chrUn	34981577	34981734	158	SAT1_CF	blue	na
chrUn	34981762	34982658	897	SAT1_CF	blue	na
chrUn	34982696	34984238	1543	SAT1_CF	blue	na
chrUn	35183213	35184505	1293	SAT1_CF	blue	na
chrUn	35184518	35185036	519	SAT1_CF	blue	na
chrUn	35185065	35186160	1096	SAT1_CF	blue	na
chrUn	35186353	35187156	804	SAT1_CF	blue	na
chrUn	35187200	35188527	1328	SAT1_CF	blue	na
chrUn	35188539	35189209	671	SAT1_CF	blue	na
chrUn	35189232	35190123	892	SAT1_CF	blue	na
chrUn	35190135	35191519	1385	SAT1_CF	blue	na
chrUn	35191553	35192039	487	SAT1_CF	blue	na
chrUn	35192052	35192558	507	SAT1_CF	blue	na
chrUn	35192638	35193313	676	SAT1_CF	blue	na
chrUn	35193326	35193845	520	SAT1_CF	blue	na
chrUn	35193892	35194027	136	SAT1_CF	blue	na
chrUn	35194055	35195495	1441	SAT1_CF	blue	na
chrUn	35195510	35197273	1764	SAT1_CF	blue	na
chrUn	35197286	35198677	1392	SAT1_CF	blue	na
chrUn	35198704	35199376	673	SAT1_CF	blue	na
chrUn	35199388	35200071	684	SAT1_CF	blue	na
chrUn	35200084	35200584	501	SAT1_CF	blue	na
chrUn	35200597	35202331	1735	SAT1_CF	blue	na
chrUn	35202351	35202460	110	SAT1_CF	blue	na
chrUn	35202472	35203014	543	SAT1_CF	blue	na
chrUn	35203057	35203955	899	SAT1_CF	blue	na
chrUn	35203967	35205706	1740	SAT1_CF	blue	na
chrUn	35205749	35207118	1370	SAT1_CF	blue	na
chrUn	35207131	35209664	2534	SAT1_CF	blue	na
chrUn	35209707	35210381	675	SAT1_CF	blue	na
chrUn	35210393	35210997	605	SAT1_CF	blue	na
chrUn	35211010	35211510	501	SAT1_CF	blue	na

chrUn	35211557	35215872	4316	SAT1_CF	blue	na
chrUn	35215917	35216456	540	SAT1_CF	blue	na
chrUn	35216467	35217160	694	SAT1_CF	blue	na
chrUn	35217241	35217787	547	SAT1_CF	blue	na
chrUn	35217810	35218987	1178	SAT1_CF	blue	na
chrUn	35219007	35219550	544	SAT1_CF	blue	na
chrUn	35219561	35220734	1174	SAT1_CF	blue	na
chrUn	35220757	35221438	682	SAT1_CF	blue	na
chrUn	35221451	35221953	503	SAT1_CF	blue	na
chrUn	35222009	35222901	893	SAT1_CF	blue	na
chrUn	35223017	35223970	954	SAT1_CF	blue	na
chrUn	35224013	35224690	678	SAT1_CF	blue	na
chrUn	35224702	35225200	499	SAT1_CF	blue	na
chrUn	35225245	35225423	179	SAT1_CF	blue	na
chrUn	35225454	35227125	1672	SAT1_CF	blue	na
chrUn	35227167	35228026	860	SAT1_CF	blue	na
chrUn	35228038	35228711	674	SAT1_CF	blue	na
chrUn	35228733	35229458	726	SAT1_CF	blue	na
chrUn	35229470	35229990	521	SAT1_CF	blue	na
chrUn	35230017	35232490	2474	SAT1_CF	blue	na
chrUn	35232535	35232692	158	SAT1_CF	blue	na
chrUn	35232724	35233229	506	SAT1_CF	blue	na
chrUn	35233261	35234302	1042	SAT1_CF	blue	na
chrUn	35234345	35234956	612	SAT1_CF	blue	na
chrUn	35234971	35235518	548	SAT1_CF	blue	na
chrUn	35235569	35237246	1678	SAT1_CF	blue	na
chrUn	35237259	35237759	501	SAT1_CF	blue	na
chrUn	35237815	35239829	2015	SAT1_CF	blue	na
chrUn	35239876	35240553	678	SAT1_CF	blue	na
chrUn	35240565	35241242	678	SAT1_CF	blue	na
chrUn	35241261	35241762	502	SAT1_CF	blue	na
chrUn	35241849	35242188	340	SAT1_CF	blue	na
chrUn	35243604	35245047	1444	SAT1_CF	blue	na
chrUn	35245090	35245763	674	SAT1_CF	blue	na
chrUn	35245775	35246380	606	SAT1_CF	blue	na
chrUn	35246392	35247810	1419	SAT1_CF	blue	na
chrUn	35247837	35248456	620	SAT1_CF	blue	na
chrUn	35248504	35249745	1242	SAT1_CF	blue	na
chrUn	35249765	35249864	100	SAT1_CF	blue	na
chrUn	35249886	35250404	519	SAT1_CF	blue	na
chrUn	35250425	35252495	2071	SAT1_CF	blue	na
chrUn	35252538	35253213	676	SAT1_CF	blue	na

chrUn	35253225	35253828	604	SAT1_CF	blue	na
chrUn	35253840	35254393	554	SAT1_CF	blue	na
chrUn	35254421	35254522	102	SAT1_CF	blue	na
chrUn	35254535	35255034	500	SAT1_CF	blue	na
chrUn	35255079	35255863	785	SAT1_CF	blue	na
chrUn	35255876	35256376	501	SAT1_CF	blue	na
chrUn	35256429	35258454	2026	SAT1_CF	blue	na
chrUn	35258497	35259868	1372	SAT1_CF	blue	na
chrUn	35259882	35260401	520	SAT1_CF	blue	na
chrUn	35260445	35261578	1134	SAT1_CF	blue	na
chrUn	35261589	35262903	1315	SAT1_CF	blue	na
chrUn	35262925	35263031	107	SAT1_CF	blue	na
chrUn	35263044	35263570	527	SAT1_CF	blue	na
chrUn	35263627	35264927	1301	SAT1_CF	blue	na
chrUn	35264939	35265147	209	SAT1_CF	blue	na
chrUn	35265160	35265762	603	SAT1_CF	blue	na
chrUn	35265775	35266458	684	SAT1_CF	blue	na
chrUn	35351001	35351212	212	SAT1_CF	blue	na
chrUn	35351226	35352385	1160	SAT1_CF	blue	na
chrUn	35352455	35354529	2075	SAT1_CF	blue	na
chrUn	35354552	35355154	603	SAT1_CF	blue	na
chrUn	35355199	35355356	158	SAT1_CF	blue	na
chrUn	35355388	35355892	505	SAT1_CF	blue	na
chrUn	35355924	35356420	497	SAT1_CF	blue	na
chrUn	35356487	35357258	772	SAT1_CF	blue	na
chrUn	35357300	35358149	850	SAT1_CF	blue	na
chrUn	35358172	35359769	1598	SAT1_CF	blue	na
chrUn	35359781	35360872	1092	SAT1_CF	blue	na
chrUn	35360926	35361556	631	SAT1_CF	blue	na
chrUn	35361599	35362264	666	SAT1_CF	blue	na
chrUn	35362286	35362893	608	SAT1_CF	blue	na
chrUn	35362905	35363388	484	SAT1_CF	blue	na
chrUn	35363408	35363668	261	SAT1_CF	blue	na
chrUn	35363707	35363869	163	SAT1_CF	blue	na
chrUn	35363882	35364773	892	SAT1_CF	blue	na
chrUn	35364785	35366125	1341	SAT1_CF	blue	na
chrUn	35366146	35368185	2040	SAT1_CF	blue	na
chrUn	35368238	35369159	922	SAT1_CF	blue	na
chrUn	35369190	35370079	890	SAT1_CF	blue	na
chrUn	35370124	35370980	857	SAT1_CF	blue	na
chrUn	35370992	35371572	581	SAT1_CF	blue	na
chrUn	35371668	35371834	167	SAT1_CF	blue	na

chrUn	35371853	35372387	535	SAT1_CF	blue	na
chrUn	35372434	35372625	192	SAT1_CF	blue	na
chrUn	35372670	35373871	1202	SAT1_CF	blue	na
chrUn	35373893	35374531	639	SAT1_CF	blue	na
chrUn	35374558	35377231	2674	SAT1_CF	blue	na
chrUn	35377243	35378222	980	SAT1_CF	blue	na
chrUn	35378235	35378756	522	SAT1_CF	blue	na
chrUn	35378824	35379354	531	SAT1_CF	blue	na
chrUn	35380763	35382006	1244	SAT1_CF	blue	na
chrUn	35382085	35384098	2014	SAT1_CF	blue	na
chrUn	35384111	35384999	889	SAT1_CF	blue	na
chrUn	35385011	35387612	2602	SAT1_CF	blue	na
chrUn	35387625	35388139	515	SAT1_CF	blue	na
chrUn	35388195	35388801	607	SAT1_CF	blue	na
chrUn	35388818	35389192	375	SAT1_CF	blue	na
chrUn	35389203	35389865	663	SAT1_CF	blue	na
chrUn	35389930	35390409	480	SAT1_CF	blue	na
chrUn	35390434	35390504	71	SAT1_CF	blue	na
chrUn	35390516	35391240	725	SAT1_CF	blue	na
chrUn	35391254	35391746	493	SAT1_CF	blue	na
chrUn	35391757	35392429	673	SAT1_CF	blue	na
chrUn	35392451	35393822	1372	SAT1_CF	blue	na
chrUn	35393865	35394410	546	SAT1_CF	blue	na
chrUn	35394432	35395183	752	SAT1_CF	blue	na
chrUn	35395204	35395313	110	SAT1_CF	blue	na
chrUn	35395327	35395825	499	SAT1_CF	blue	na
chrUn	35395912	35396267	356	SAT1_CF	blue	na
chrUn	35396281	35396892	612	SAT1_CF	blue	na
chrUn	35396936	35397685	750	SAT1_CF	blue	na
chrUn	35397711	35398617	907	SAT1_CF	blue	na
chrUn	35398629	35399182	554	SAT1_CF	blue	na
chrUn	35399204	35399311	108	SAT1_CF	blue	na
chrUn	35399324	35399824	501	SAT1_CF	blue	na
chrUn	35399864	35400264	401	SAT1_CF	blue	na
chrUn	35400275	35401688	1414	SAT1_CF	blue	na
chrUn	35401700	35402383	684	SAT1_CF	blue	na
chrUn	35402403	35403364	962	SAT1_CF	blue	na
chrUn	35403408	35404609	1202	SAT1_CF	blue	na
chrUn	35404629	35405227	599	SAT1_CF	blue	na
chrUn	35405284	35405356	73	SAT1_CF	blue	na
chrUn	35405368	35405889	522	SAT1_CF	blue	na
chrUn	35405915	35406072	158	SAT1_CF	blue	na

chrUn	35406100	35406610	511	SAT1_CF	blue	na
chrUn	35406690	35407254	565	SAT1_CF	blue	na
chrUn	35407282	35409206	1925	SAT1_CF	blue	na
chrUn	35409274	35410321	1048	SAT1_CF	blue	na
chrUn	35410339	35411265	927	SAT1_CF	blue	na
chrUn	35411307	35411977	671	SAT1_CF	blue	na
chrUn	35411995	35412676	682	SAT1_CF	blue	na
chrUn	35412689	35413580	892	SAT1_CF	blue	na
chrUn	35413592	35414595	1004	SAT1_CF	blue	na
chrUn	35414608	35415129	522	SAT1_CF	blue	na
chrUn	35415150	35415552	403	SAT1_CF	blue	na
chrUn	35415602	35416144	543	SAT1_CF	blue	na
chrUn	35416224	35417206	983	SAT1_CF	blue	na
chrUn	35417249	35417795	547	SAT1_CF	blue	na
chrUn	35417824	35417926	103	SAT1_CF	blue	na
chrUn	35417938	35419556	1619	SAT1_CF	blue	na
chrUn	35419598	35420451	854	SAT1_CF	blue	na
chrUn	35420463	35421147	685	SAT1_CF	blue	na
chrUn	35421160	35421681	522	SAT1_CF	blue	na
chrUn	35421710	35422382	673	SAT1_CF	blue	na
chrUn	35422395	35423896	1502	SAT1_CF	blue	na
chrUn	35423995	35424991	997	SAT1_CF	blue	na
chrUn	35425034	35425709	676	SAT1_CF	blue	na
chrUn	35425722	35426224	503	SAT1_CF	blue	na
chrUn	35426237	35426837	601	SAT1_CF	blue	na
chrUn	35428253	35429577	1325	SAT1_CF	blue	na
chrUn	35429591	35430083	493	SAT1_CF	blue	na
chrUn	35430094	35432559	2466	SAT1_CF	blue	na
chrUn	35433899	35434321	423	CarSat1	red	na
chrUn	35434353	35435072	720	CarSat1	red	na
chrUn	35435067	35436198	1132	SAT1_CF	blue	na
chrUn	35436211	35437181	971	SAT1_CF	blue	na
chrUn	35437200	35439638	2439	SAT1_CF	blue	na
chrUn	35439716	35440301	586	SAT1_CF	blue	na
chrUn	35440381	35441422	1042	SAT1_CF	blue	na
chrUn	35441465	35441973	509	SAT1_CF	blue	na
chrUn	35441987	35442379	393	SAT1_CF	blue	na
chrUn	35442384	35445098	2715	CarSat1	red	na
chrUn	35445119	35445911	793	CarSat1	red	na
chrUn	35445929	35446704	776	CarSat1	red	na
chrUn	35446725	35447249	525	CarSat1	red	na
chrUn	35447649	35448816	1168	Sat2CF	purple	na

chrUn	35448708	35448885	178	Sat2CF	purple	na
chrUn	35463785	35464568	784	Sat2CF	purple	na
chrUn	35464273	35464618	346	Sat2CF	purple	na
chrUn	35477946	35478825	880	Sat2CF	purple	na
chrUn	35481844	35481978	135	CarSat1	red	na
chrUn	35482005	35483352	1348	CarSat1	red	na
chrUn	35483373	35483804	432	CarSat1	red	na
chrUn	35494295	35494612	318	CarSat1	red	na
chrUn	35494811	35495695	885	CarSat1	red	na
chrUn	35495727	35497414	1688	CarSat1	red	na
chrUn	35497662	35498392	731	CarSat1	red	na
chrUn	35498414	35499129	716	CarSat1	red	na
chrUn	35499151	35500839	1689	CarSat1	red	na
chrUn	35500879	35501573	695	CarSat1	red	na
chrUn	35501607	35504519	2913	CarSat1	red	na
chrUn	35504543	35505257	715	CarSat1	red	na
chrUn	35505281	35509900	4620	CarSat1	red	na
chrUn	35509915	35510041	127	Carsat2	orange	na
chrUn	35509930	35510643	714	CarSat1	red	na
chrUn	35510673	35512117	1445	CarSat1	red	na
chrUn	35512132	35512258	127	Carsat2	orange	na
chrUn	35512147	35512852	706	CarSat1	red	na
chrUn	35512867	35512994	128	Carsat2	orange	na
chrUn	35512882	35515330	2449	CarSat1	red	na
chrUn	35515351	35515943	593	CarSat1	red	na
chrUn	35629794	35630928	1135	Sat2CF	purple	na
chrUn	35629927	35631013	1087	Sat2CF	purple	na
chrUn	35630889	35631038	150	Sat2CF	purple	na
chrUn	35644751	35645630	880	Sat2CF	purple	na
chrUn	35644903	35645715	813	Sat2CF	purple	na
chrUn	35671309	35672430	1122	Sat2CF	purple	na
chrUn	35687246	35688398	1153	Sat2CF	purple	na
chrUn	35710378	35710555	178	Sat2CF	purple	na
chrUn	35710426	35710941	516	Sat2CF	purple	na
chrUn	35710651	35710982	332	Sat2CF	purple	na
chrUn	35719859	35721025	1167	Sat2CF	purple	na
chrUn	35739073	35740231	1159	Sat2CF	purple	na
chrUn	35782290	35782994	705	CarSat1	red	na
chrUn	35783024	35784606	1583	CarSat1	red	na
chrUn	35784627	35785477	851	CarSat1	red	na
chrUn	35785500	35786356	857	CarSat1	red	na
chrUn	35786377	35787155	779	CarSat1	red	na

chrUn	35787169	35787612	444	CarSat1	red	na
chrUn	35787615	35788629	1015	SAT1_CF	blue	na
chrUn	35788624	35789348	725	CarSat1	red	na
chrUn	35789380	35789806	427	CarSat1	red	na
chrUn	35791246	35792027	782	SAT1_CF	blue	na
chrUn	35792107	35792631	525	SAT1_CF	blue	na
chrUn	35792646	35792782	137	SAT1_CF	blue	na
chrUn	35792829	35793349	521	SAT1_CF	blue	na
chrUn	35793361	35794035	675	SAT1_CF	blue	na
chrUn	35794078	35795224	1147	SAT1_CF	blue	na
chrUn	35795294	35797855	2562	SAT1_CF	blue	na
chrUn	35797875	35798358	484	SAT1_CF	blue	na
chrUn	35798370	35799055	686	SAT1_CF	blue	na
chrUn	35799073	35799743	671	SAT1_CF	blue	na
chrUn	35799786	35801408	1623	SAT1_CF	blue	na
chrUn	35801419	35802981	1563	SAT1_CF	blue	na
chrUn	35803016	35804614	1599	SAT1_CF	blue	na
chrUn	35804633	35805504	872	SAT1_CF	blue	na
chrUn	35805517	35806119	603	SAT1_CF	blue	na
chrUn	35806131	35806805	675	SAT1_CF	blue	na
chrUn	35806848	35807812	965	SAT1_CF	blue	na
chrUn	35807856	35808921	1066	SAT1_CF	blue	na
chrUn	35808984	35809466	483	SAT1_CF	blue	na
chrUn	35809478	35810162	685	SAT1_CF	blue	na
chrUn	35810205	35810801	597	SAT1_CF	blue	na
chrUn	35810829	35811575	747	SAT1_CF	blue	na
chrUn	35811654	35812613	960	SAT1_CF	blue	na
chrUn	35812653	35813155	503	SAT1_CF	blue	na
chrUn	35813182	35813849	668	SAT1_CF	blue	na
chrUn	35813867	35814456	590	SAT1_CF	blue	na
chrUn	35814468	35814551	84	SAT1_CF	blue	na
chrUn	35814564	35815511	948	SAT1_CF	blue	na
chrUn	35815564	35818109	2546	SAT1_CF	blue	na
chrUn	35818122	35818804	683	SAT1_CF	blue	na
chrUn	35818819	35819492	674	SAT1_CF	blue	na
chrUn	35819535	35821190	1656	SAT1_CF	blue	na
chrUn	35821201	35821520	320	SAT1_CF	blue	na
chrUn	35821573	35822073	501	SAT1_CF	blue	na
chrUn	35822085	35822744	660	SAT1_CF	blue	na
chrUn	35822798	35823947	1150	SAT1_CF	blue	na
chrUn	35824026	35824574	549	SAT1_CF	blue	na
chrUn	35824690	35825230	541	SAT1_CF	blue	na

chrUn	35825318	35825819	502	SAT1_CF	blue	na
chrUn	35825831	35827106	1276	SAT1_CF	blue	na
chrUn	35827134	35827697	564	SAT1_CF	blue	na
chrUn	35827740	35828262	523	SAT1_CF	blue	na
chrUn	35828277	35828413	137	SAT1_CF	blue	na
chrUn	35828479	35828977	499	SAT1_CF	blue	na
chrUn	35828999	35831171	2173	SAT1_CF	blue	na
chrUn	35831192	35831798	607	SAT1_CF	blue	na
chrUn	35831812	35831987	176	SAT1_CF	blue	na
chrUn	35832016	35832537	522	SAT1_CF	blue	na
chrUn	35832550	35833155	606	SAT1_CF	blue	na
chrUn	35833173	35834077	905	SAT1_CF	blue	na
chrUn	35834105	35834650	546	SAT1_CF	blue	na
chrUn	35834729	35836293	1565	SAT1_CF	blue	na
chrUn	35836307	35836988	682	SAT1_CF	blue	na
chrUn	35837000	35837683	684	SAT1_CF	blue	na
chrUn	35837694	35838186	493	SAT1_CF	blue	na
chrUn	35838200	35838907	708	SAT1_CF	blue	na
chrUn	35838950	35839828	879	SAT1_CF	blue	na
chrUn	35839842	35840404	563	SAT1_CF	blue	na
chrUn	35841817	35842390	574	SAT1_CF	blue	na
chrUn	35842430	35842922	493	SAT1_CF	blue	na
chrUn	35842939	35843621	683	SAT1_CF	blue	na
chrUn	35843632	35844236	605	SAT1_CF	blue	na
chrUn	35844279	35844779	501	SAT1_CF	blue	na
chrUn	35844792	35845595	804	SAT1_CF	blue	na
chrUn	35845618	35846163	546	SAT1_CF	blue	na
chrUn	35846243	35846605	363	SAT1_CF	blue	na
chrUn	35846654	35847153	500	SAT1_CF	blue	na
chrUn	35847165	35847848	684	SAT1_CF	blue	na
chrUn	35847900	35848440	541	SAT1_CF	blue	na
chrUn	35848511	35850338	1828	SAT1_CF	blue	na
chrUn	35850378	35850879	502	SAT1_CF	blue	na
chrUn	35850892	35851650	759	SAT1_CF	blue	na
chrUn	35851663	35852338	676	SAT1_CF	blue	na
chrUn	35852381	35853311	931	SAT1_CF	blue	na
chrUn	35853427	35854340	914	SAT1_CF	blue	na
chrUn	35854427	35854928	502	SAT1_CF	blue	na
chrUn	35854952	35855622	671	SAT1_CF	blue	na
chrUn	35855640	35856301	662	SAT1_CF	blue	na
chrUn	35856313	35856466	154	SAT1_CF	blue	na
chrUn	35856508	35857020	513	SAT1_CF	blue	na

chrUn	35857032	35857706	675	SAT1_CF	blue	na
chrUn	35857749	35858291	543	SAT1_CF	blue	na
chrUn	35858341	35858661	321	SAT1_CF	blue	na
chrUn	35858726	35859282	557	SAT1_CF	blue	na
chrUn	35859295	35859997	703	SAT1_CF	blue	na
chrUn	35860009	35860678	670	SAT1_CF	blue	na
chrUn	35860733	35861235	503	SAT1_CF	blue	na
chrUn	35861247	35861356	110	SAT1_CF	blue	na
chrUn	35861377	35861913	537	SAT1_CF	blue	na
chrUn	35861951	35862264	314	SAT1_CF	blue	na
chrUn	35862305	35863985	1681	SAT1_CF	blue	na
chrUn	35864000	35864703	704	SAT1_CF	blue	na
chrUn	35864716	35865399	684	SAT1_CF	blue	na
chrUn	35865411	35866087	677	SAT1_CF	blue	na
chrUn	35866184	35867392	1209	SAT1_CF	blue	na
chrUn	35867420	35867555	136	SAT1_CF	blue	na
chrUn	35867638	35868121	484	SAT1_CF	blue	na
chrUn	35868134	35869087	954	SAT1_CF	blue	na
chrUn	35869109	35869617	509	SAT1_CF	blue	na
chrUn	35869660	35870386	727	SAT1_CF	blue	na
chrUn	35870429	35870946	518	SAT1_CF	blue	na
chrUn	36103357	36104082	726	SAT1_CF	blue	na
chrUn	36104125	36104635	511	SAT1_CF	blue	na
chrUn	36104664	36104821	158	SAT1_CF	blue	na
chrUn	36104866	36106071	1206	SAT1_CF	blue	na
chrUn	36106083	36106755	673	SAT1_CF	blue	na
chrUn	36106798	36108793	1996	SAT1_CF	blue	na
chrUn	36108879	36110696	1818	SAT1_CF	blue	na
chrUn	36110721	36111779	1059	SAT1_CF	blue	na
chrUn	36111791	36112109	319	SAT1_CF	blue	na
chrUn	36112157	36112663	507	SAT1_CF	blue	na
chrUn	36112676	36113443	768	SAT1_CF	blue	na
chrUn	36113456	36114784	1329	SAT1_CF	blue	na
chrUn	36114830	36116241	1412	SAT1_CF	blue	na
chrUn	36116281	36116783	503	SAT1_CF	blue	na
chrUn	36116796	36117480	685	SAT1_CF	blue	na
chrUn	36117492	36118174	683	SAT1_CF	blue	na
chrUn	36118186	36118860	675	SAT1_CF	blue	na
chrUn	36118905	36119535	631	SAT1_CF	blue	na
chrUn	36119563	36119934	372	SAT1_CF	blue	na
chrUn	36120115	36120298	184	SAT1_CF	blue	na
chrUn	36120328	36122599	2272	SAT1_CF	blue	na

chrUn	36122610	36123010	401	SAT1_CF	blue	na
chrUn	36123050	36123551	502	SAT1_CF	blue	na
chrUn	36123562	36124168	607	SAT1_CF	blue	na
chrUn	36124179	36124799	621	SAT1_CF	blue	na
chrUn	36124823	36124959	137	SAT1_CF	blue	na
chrUn	36125043	36125525	483	SAT1_CF	blue	na
chrUn	36125538	36126297	760	SAT1_CF	blue	na
chrUn	36126310	36127151	842	SAT1_CF	blue	na
chrUn	36127193	36129086	1894	SAT1_CF	blue	na
chrUn	36129126	36129628	503	SAT1_CF	blue	na
chrUn	36129641	36130316	676	SAT1_CF	blue	na
chrUn	36130359	36131731	1373	SAT1_CF	blue	na
chrUn	36131774	36132336	563	SAT1_CF	blue	na
chrUn	36132348	36133029	682	SAT1_CF	blue	na
chrUn	36133041	36133150	110	SAT1_CF	blue	na
chrUn	36133171	36133639	469	SAT1_CF	blue	na
chrUn	36133653	36133762	110	SAT1_CF	blue	na
chrUn	36133783	36134328	546	SAT1_CF	blue	na
chrUn	36134371	36135175	805	SAT1_CF	blue	na
chrUn	36135262	36135746	485	SAT1_CF	blue	na
chrUn	36135762	36136906	1145	SAT1_CF	blue	na
chrUn	36136919	36137593	675	SAT1_CF	blue	na
chrUn	36137636	36138266	631	SAT1_CF	blue	na
chrUn	36138294	36138858	565	SAT1_CF	blue	na
chrUn	36138871	36138994	124	SAT1_CF	blue	na
chrUn	36139005	36139408	404	SAT1_CF	blue	na
chrUn	36139446	36139948	503	SAT1_CF	blue	na
chrUn	36139974	36142121	2148	SAT1_CF	blue	na
chrUn	36142163	36142341	179	SAT1_CF	blue	na
chrUn	36142401	36143361	961	SAT1_CF	blue	na
chrUn	36143382	36143901	520	SAT1_CF	blue	na
chrUn	36143928	36144673	746	SAT1_CF	blue	na
chrUn	36144685	36145544	860	SAT1_CF	blue	na
chrUn	36145585	36147029	1445	SAT1_CF	blue	na
chrUn	36147057	36148090	1034	SAT1_CF	blue	na
chrUn	36148102	36148211	110	SAT1_CF	blue	na
chrUn	36148232	36148862	631	SAT1_CF	blue	na
chrUn	36148874	36150069	1196	SAT1_CF	blue	na
chrUn	36150116	36151076	961	SAT1_CF	blue	na
chrUn	36151143	36151664	522	SAT1_CF	blue	na
chrUn	36151677	36152079	403	SAT1_CF	blue	na
chrUn	36152119	36152620	502	SAT1_CF	blue	na

chrUn	36152633	36153316	684	SAT1_CF	blue	na
chrUn	36153328	36154004	677	SAT1_CF	blue	na
chrUn	36154047	36154678	632	SAT1_CF	blue	na
chrUn	36154706	36155935	1230	SAT1_CF	blue	na
chrUn	36155963	36156099	137	SAT1_CF	blue	na
chrUn	36156146	36156667	522	SAT1_CF	blue	na
chrUn	36156680	36157364	685	SAT1_CF	blue	na
chrUn	36157376	36158059	684	SAT1_CF	blue	na
chrUn	36158071	36159842	1772	SAT1_CF	blue	na
chrUn	36159867	36160828	962	SAT1_CF	blue	na
chrUn	36160868	36161443	576	SAT1_CF	blue	na
chrUn	36161456	36161563	108	SAT1_CF	blue	na
chrUn	36161584	36162123	540	SAT1_CF	blue	na
chrUn	36162136	36162896	761	SAT1_CF	blue	na
chrUn	36162908	36163570	663	SAT1_CF	blue	na
chrUn	36163627	36165842	2216	SAT1_CF	blue	na
chrUn	36165855	36166849	995	SAT1_CF	blue	na
chrUn	36166861	36167471	611	SAT1_CF	blue	na
chrUn	36167490	36167655	166	SAT1_CF	blue	na
chrUn	36167668	36168517	850	SAT1_CF	blue	na
chrUn	36168594	36169608	1015	SAT1_CF	blue	na
chrUn	36169630	36170295	666	SAT1_CF	blue	na
chrUn	36170338	36171411	1074	SAT1_CF	blue	na
chrUn	36439828	36440032	205	SAT1_CF	blue	na
chrUn	36440077	36440576	500	SAT1_CF	blue	na
chrUn	36440589	36441189	601	SAT1_CF	blue	na
chrUn	36441201	36441876	676	SAT1_CF	blue	na
chrUn	36441919	36443439	1521	SAT1_CF	blue	na
chrUn	36444823	36445572	750	SAT1_CF	blue	na
chrUn	36445584	36447389	1806	SAT1_CF	blue	na
chrUn	36447407	36448329	923	SAT1_CF	blue	na
chrUn	36448362	36448883	522	SAT1_CF	blue	na
chrUn	36448895	36449580	686	SAT1_CF	blue	na
chrUn	36449623	36450220	598	SAT1_CF	blue	na
chrUn	36450248	36450793	546	SAT1_CF	blue	na
chrUn	36450835	36451013	179	SAT1_CF	blue	na
chrUn	36451073	36452574	1502	SAT1_CF	blue	na
chrUn	36452587	36453875	1289	SAT1_CF	blue	na
chrUn	36453887	36453970	84	SAT1_CF	blue	na
chrUn	36453983	36454526	544	SAT1_CF	blue	na
chrUn	36454611	36454929	319	SAT1_CF	blue	na
chrUn	36454982	36455473	492	SAT1_CF	blue	na

chrUn	36455499	36456102	604	SAT1_CF	blue	na
chrUn	36456114	36456784	671	SAT1_CF	blue	na
chrUn	36456832	36457789	958	SAT1_CF	blue	na
chrUn	36457844	36458430	587	SAT1_CF	blue	na
chrUn	36458533	36459114	582	SAT1_CF	blue	na
chrUn	36459126	36459802	677	SAT1_CF	blue	na
chrUn	36459845	36460466	622	SAT1_CF	blue	na
chrUn	36460502	36461066	565	SAT1_CF	blue	na
chrUn	36461145	36461668	524	SAT1_CF	blue	na
chrUn	36461683	36461841	159	SAT1_CF	blue	na
chrUn	36461885	36462385	501	SAT1_CF	blue	na
chrUn	36462407	36463081	675	SAT1_CF	blue	na
chrUn	36463093	36463735	643	SAT1_CF	blue	na
chrUn	36463760	36465509	1750	SAT1_CF	blue	na
chrUn	36465521	36465839	319	SAT1_CF	blue	na
chrUn	36465892	36466394	503	SAT1_CF	blue	na
chrUn	36466420	36467209	790	SAT1_CF	blue	na
chrUn	36467231	36468252	1022	SAT1_CF	blue	na
chrUn	36468351	36469174	824	SAT1_CF	blue	na
chrUn	36469217	36469581	365	SAT1_CF	blue	na
chrUn	36469606	36470126	521	SAT1_CF	blue	na
chrUn	36470139	36470814	676	SAT1_CF	blue	na
chrUn	36470857	36472527	1671	SAT1_CF	blue	na
chrUn	36472539	36473438	900	SAT1_CF	blue	na
chrUn	36473481	36474023	543	SAT1_CF	blue	na
chrUn	36474035	36474144	110	SAT1_CF	blue	na
chrUn	36474164	36476490	2327	SAT1_CF	blue	na
chrUn	36476502	36477865	1364	SAT1_CF	blue	na
chrUn	36477878	36478457	580	SAT1_CF	blue	na
chrUn	36478520	36479074	555	SAT1_CF	blue	na
chrUn	36479086	36479760	675	SAT1_CF	blue	na
chrUn	36479803	36480876	1074	SAT1_CF	blue	na
chrUn	36480908	36481598	691	SAT1_CF	blue	na
chrUn	36481645	36482158	514	SAT1_CF	blue	na
chrUn	36482180	36482861	682	SAT1_CF	blue	na
chrUn	36482873	36482981	109	SAT1_CF	blue	na
chrUn	36483002	36483541	540	SAT1_CF	blue	na
chrUn	36483569	36483803	235	SAT1_CF	blue	na
chrUn	36483815	36484492	678	SAT1_CF	blue	na
chrUn	36484535	36486191	1657	SAT1_CF	blue	na
chrUn	36486202	36486521	320	SAT1_CF	blue	na
chrUn	36486595	36487074	480	SAT1_CF	blue	na

chrUn	36487086	36487769	684	SAT1_CF	blue	na
chrUn	36487781	36487851	71	SAT1_CF	blue	na
chrUn	36487876	36490168	2293	SAT1_CF	blue	na
chrUn	36490180	36491962	1783	SAT1_CF	blue	na
chrUn	36491974	36492080	107	SAT1_CF	blue	na
chrUn	36492104	36492732	629	SAT1_CF	blue	na
chrUn	36492756	36493203	448	SAT1_CF	blue	na
chrUn	36493220	36493375	156	SAT1_CF	blue	na
chrUn	36493405	36493930	526	SAT1_CF	blue	na
chrUn	36493945	36494081	137	SAT1_CF	blue	na
chrUn	36494177	36494696	520	SAT1_CF	blue	na
chrUn	36494753	36495298	546	SAT1_CF	blue	na
chrUn	36495340	36495884	545	SAT1_CF	blue	na
chrUn	36495926	36496946	1021	SAT1_CF	blue	na
chrUn	36496998	36497155	158	SAT1_CF	blue	na
chrUn	36497202	36497700	499	SAT1_CF	blue	na
chrUn	36497717	36498396	680	SAT1_CF	blue	na
chrUn	36498409	36499021	613	SAT1_CF	blue	na
chrUn	36499033	36499892	860	SAT1_CF	blue	na
chrUn	36499969	36501677	1709	SAT1_CF	blue	na
chrUn	36501691	36502201	511	SAT1_CF	blue	na
chrUn	36502214	36503861	1648	SAT1_CF	blue	na
chrUn	36503889	36504046	158	SAT1_CF	blue	na
chrUn	36504091	36504592	502	SAT1_CF	blue	na
chrUn	36504605	36505288	684	SAT1_CF	blue	na
chrUn	36505300	36506930	1631	SAT1_CF	blue	na
chrUn	36507005	36507248	244	SAT1_CF	blue	na
chrUn	36507266	36508194	929	SAT1_CF	blue	na
chrUn	36508232	36508736	505	SAT1_CF	blue	na
chrUn	36508754	36509438	685	SAT1_CF	blue	na
chrUn	36509450	36510885	1436	SAT1_CF	blue	na
chrUn	36510897	36511859	963	SAT1_CF	blue	na
chrUn	36511906	36512447	542	SAT1_CF	blue	na
chrUn	36512492	36513455	964	SAT1_CF	blue	na
chrUn	36513535	36514444	910	SAT1_CF	blue	na
chrUn	36514497	36514997	501	SAT1_CF	blue	na
chrUn	36515015	36516625	1611	SAT1_CF	blue	na
chrUn	36516695	36517241	547	SAT1_CF	blue	na
chrUn	36517289	36517875	587	SAT1_CF	blue	na
chrUn	36518877	36519121	245	SAT1_CF	blue	na
chrUn	36519133	36520500	1368	SAT1_CF	blue	na
chrUn	36520543	36521537	995	SAT1_CF	blue	na

chrUn	36521550	36522070	521	SAT1_CF	blue	na
chrUn	36522101	36522295	195	SAT1_CF	blue	na
chrUn	36522323	36523050	728	SAT1_CF	blue	na
chrUn	36523089	36523633	545	SAT1_CF	blue	na
chrUn	36523646	36523946	301	SAT1_CF	blue	na
chrUn	36523958	36524071	114	SAT1_CF	blue	na
chrUn	36524088	36524720	633	SAT1_CF	blue	na
chrUn	36524733	36525407	675	SAT1_CF	blue	na
chrUn	36525450	36526923	1474	SAT1_CF	blue	na
chrUn	36531035	36531266	232	SAT1_CF	blue	na
chrUn	36531308	36532504	1197	SAT1_CF	blue	na
chrUn	36532532	36532690	159	SAT1_CF	blue	na
chrUn	36532734	36533236	503	SAT1_CF	blue	na
chrUn	36533248	36533930	683	SAT1_CF	blue	na
chrUn	36533942	36534619	678	SAT1_CF	blue	na
chrUn	36534716	36535257	542	SAT1_CF	blue	na
chrUn	36535282	36536159	878	SAT1_CF	blue	na
chrUn	36536207	36537176	970	SAT1_CF	blue	na
chrUn	36537255	36537888	634	SAT1_CF	blue	na
chrUn	36537941	36538319	379	SAT1_CF	blue	na
chrUn	36538406	36538908	503	SAT1_CF	blue	na
chrUn	36538933	36539602	670	SAT1_CF	blue	na
chrUn	36539614	36540290	677	SAT1_CF	blue	na
chrUn	36540334	36540673	340	SAT1_CF	blue	na
chrUn	36540697	36541196	500	SAT1_CF	blue	na
chrUn	36541216	36541898	683	SAT1_CF	blue	na
chrUn	36541921	36542585	665	SAT1_CF	blue	na
chrUn	36542628	36543259	632	SAT1_CF	blue	na
chrUn	36543287	36544480	1194	SAT1_CF	blue	na
chrUn	36544508	36544644	137	SAT1_CF	blue	na
chrUn	36544691	36545212	522	SAT1_CF	blue	na
chrUn	36545234	36546176	943	SAT1_CF	blue	na
chrUn	36546206	36546747	542	SAT1_CF	blue	na
chrUn	36546758	36547614	857	SAT1_CF	blue	na
chrUn	36547625	36548117	493	SAT1_CF	blue	na
chrUn	36548140	36549496	1357	SAT1_CF	blue	na
chrUn	36549560	36550123	564	SAT1_CF	blue	na
chrUn	36550166	36550719	554	SAT1_CF	blue	na
chrUn	36550730	36551341	612	SAT1_CF	blue	na
chrUn	36551364	36552030	667	SAT1_CF	blue	na
chrUn	36552073	36553809	1737	SAT1_CF	blue	na
chrUn	36554116	36555075	960	SAT1_CF	blue	na

chrUn	36555115	36555617	503	SAT1_CF	blue	na
chrUn	36555629	36556312	684	SAT1_CF	blue	na
chrUn	36556324	36557297	974	SAT1_CF	blue	na
chrUn	36557320	36557392	73	SAT1_CF	blue	na
chrUn	36557448	36559168	1721	SAT1_CF	blue	na
chrUn	36559231	36559609	379	SAT1_CF	blue	na
chrUn	36559696	36560198	503	SAT1_CF	blue	na
chrUn	36560223	36560892	670	SAT1_CF	blue	na
chrUn	36560904	36562381	1478	SAT1_CF	blue	na
chrUn	36562423	36562927	505	SAT1_CF	blue	na
chrUn	36562955	36563480	526	SAT1_CF	blue	na
chrUn	36563495	36563729	235	SAT1_CF	blue	na
chrUn	36563753	36564245	493	SAT1_CF	blue	na
chrUn	36564302	36564849	548	SAT1_CF	blue	na
chrUn	36564891	36565617	727	SAT1_CF	blue	na
chrUn	36565662	36566197	536	SAT1_CF	blue	na
chrUn	36566219	36566825	607	SAT1_CF	blue	na
chrUn	36567344	36567510	167	SAT1_CF	blue	na
chrUn	36567582	36568089	508	SAT1_CF	blue	na
chrUn	36568102	36568290	189	SAT1_CF	blue	na
chrUn	36568392	36568936	545	SAT1_CF	blue	na
chrUn	36568949	36569652	704	SAT1_CF	blue	na
chrUn	36569664	36570885	1222	SAT1_CF	blue	na
chrUn	36570953	36571472	520	SAT1_CF	blue	na
chrUn	36571485	36572245	761	SAT1_CF	blue	na
chrUn	36572258	36573588	1331	SAT1_CF	blue	na
chrUn	36573631	36575477	1847	SAT1_CF	blue	na
chrUn	36575510	36575666	157	SAT1_CF	blue	na
chrUn	36575711	36576212	502	SAT1_CF	blue	na
chrUn	36576225	36577669	1445	SAT1_CF	blue	na
chrUn	36577695	36578322	628	SAT1_CF	blue	na
chrUn	36578366	36579297	932	SAT1_CF	blue	na
chrUn	36579310	36579970	661	SAT1_CF	blue	na
chrUn	36580013	36580549	537	SAT1_CF	blue	na
chrUn	36580562	36582002	1441	SAT1_CF	blue	na
chrUn	36582109	36582589	481	SAT1_CF	blue	na
chrUn	36582602	36583284	683	SAT1_CF	blue	na
chrUn	36583296	36583973	678	SAT1_CF	blue	na
chrUn	36584019	36584979	961	SAT1_CF	blue	na
chrUn	36585045	36585566	522	SAT1_CF	blue	na
chrUn	36585579	36585934	356	SAT1_CF	blue	na
chrUn	36586021	36586527	507	SAT1_CF	blue	na

chrUn	36586545	36586936	392	SAT1_CF	blue	na
chrUn	36586976	36587478	503	SAT1_CF	blue	na
chrUn	36587519	36588371	853	SAT1_CF	blue	na
chrUn	36588441	36590114	1674	SAT1_CF	blue	na
chrUn	36590132	36590656	525	SAT1_CF	blue	na
chrUn	36590669	36591019	351	SAT1_CF	blue	na
chrUn	36591276	36591634	359	SAT1_CF	blue	na
chrUn	36591646	36592946	1301	SAT1_CF	blue	na
chrUn	36593032	36593551	520	SAT1_CF	blue	na
chrUn	36593644	36594101	458	SAT1_CF	blue	na
chrUn	36594188	36594689	502	SAT1_CF	blue	na
chrUn	36594716	36595057	342	SAT1_CF	blue	na
chrUn	36595080	36595597	518	SAT1_CF	blue	na
chrUn	36595609	36596465	857	SAT1_CF	blue	na
chrUn	36596508	36597298	791	SAT1_CF	blue	na
chrUn	36597342	36598700	1359	SAT1_CF	blue	na
chrUn	36598780	36599304	525	SAT1_CF	blue	na
chrUn	36599319	36599476	158	SAT1_CF	blue	na
chrUn	36599520	36600022	503	SAT1_CF	blue	na
chrUn	36600044	36600794	751	SAT1_CF	blue	na
chrUn	36605210	36605541	332	Sat2CF	purple	na
chrUn	36605251	36605766	516	Sat2CF	purple	na
chrUn	36605637	36605814	178	Sat2CF	purple	na
chrUn	36620694	36621534	841	Sat2CF	purple	na
chrUn	36620759	36621484	726	Sat2CF	purple	na
chrUn	36634103	36635355	1253	Sat2CF	purple	na
chrUn	36638031	36639571	1541	CarSat1	red	na
chrUn	36639598	36641082	1485	CarSat1	red	na
chrUn	36649773	36650489	717	CarSat1	red	na
chrUn	36650687	36651779	1093	CarSat1	red	na
chrUn	36651809	36657437	5629	CarSat1	red	na
chrUn	36657453	36658383	931	CarSat1	red	na
chrUn	36658413	36659393	981	CarSat1	red	na
chrUn	36659414	36661850	2437	CarSat1	red	na
chrUn	36661866	36663055	1190	CarSat1	red	na
chrUn	36663604	36665348	1745	CarSat1	red	na
chrUn	36665381	36666077	697	CarSat1	red	na
chrUn	36666093	36667543	1451	CarSat1	red	na
chrUn	36667559	36668273	715	CarSat1	red	na
chrUn	36668297	36669502	1206	CarSat1	red	na
chrUn	36669529	36671438	1910	CarSat1	red	na
chrUn	36671468	36673906	2439	CarSat1	red	na

chrUn	36674087	36674610	524	CarSat1	red	na
chrUn	36674874	36675502	629	CarSat1	red	na
chrUn	36675518	36676239	722	CarSat1	red	na
chrUn	36676268	36678359	2092	CarSat1	red	na
chrUn	36679898	36680148	251	CarSat1	red	na
chrUn	36680180	36680897	718	CarSat1	red	na
chrUn	36680892	36682044	1153	SAT1_CF	blue	na
chrUn	36682064	36684136	2073	SAT1_CF	blue	na
chrUn	36684217	36685996	1780	SAT1_CF	blue	na
chrUn	36685999	36686928	930	CarSat1	red	na
chrUn	36686941	36687977	1037	CarSat1	red	na
chrUn	36687995	36688723	729	CarSat1	red	na
chrUn	36688716	36691994	3279	SAT1_CF	blue	na
chrUn	36692006	36695544	3539	SAT1_CF	blue	na
chrUn	36695549	36695760	212	CarSat1	red	na
chrUn	36695951	36696944	994	CarSat1	red	na
chrUn	36696976	36698473	1498	CarSat1	red	na
chrUn	36698494	36699018	525	CarSat1	red	na
chrUn	36699418	36700587	1170	Sat2CF	purple	na
chrUn	36700479	36700656	178	Sat2CF	purple	na
chrUn	36715575	36716435	861	Sat2CF	purple	na
chrUn	36728721	36729972	1252	Sat2CF	purple	na
chrUn	36732648	36734085	1438	CarSat1	red	na
chrUn	36751051	36751365	315	SAT1_CF	blue	na
chrUn	36756028	36756721	694	Sat2CF	purple	na
chrUn	36756405	36756807	403	Sat2CF	purple	na
chrUn	36774401	36775486	1086	Sat2CF	purple	na
chrUn	36775357	36775558	202	Sat2CF	purple	na
chrUn	36777230	36777604	375	CarSat1	red	na
chrUn	36778572	36779428	857	CarSat1	red	na
chrUn	36779449	36780350	902	CarSat1	red	na
chrUn	36805987	36806710	724	SAT1_CF	blue	na
chrUn	36806753	36808270	1518	SAT1_CF	blue	na
chrUn	36808313	36810423	2111	SAT1_CF	blue	na
chrUn	36810467	36811264	798	SAT1_CF	blue	na
chrUn	36937289	36938212	924	Sat2CF	purple	na
chrUn	36937340	36938149	810	Sat2CF	purple	na
chrUn	36964968	36966220	1253	Sat2CF	purple	na
chrUn	36965004	36966090	1087	Sat2CF	purple	na
chrUn	37007278	37008016	739	Sat2CF	purple	na
chrUn	37007280	37007942	663	Sat2CF	purple	na
chrUn	37091387	37092152	766	CarSat1	red	na

chrUn	37210519	37211157	639	Sat49CF	na	na
chrUn	37264333	37264696	364	Sat28CF	na	na
chrUn	37282857	37288508	5652	CarSat1	red	na
chrUn	37288538	37289944	1407	CarSat1	red	na
chrUn	37293643	37294403	761	CarSat1	red	na
chrUn	37294433	37295433	1001	CarSat1	red	na
chrUn	37295992	37296354	363	CarSat1	red	na
chrUn	37296381	37298827	2447	CarSat1	red	na
chrUn	37300219	37302728	2510	CarSat1	red	na
chrUn	37302761	37303734	974	CarSat1	red	na
chrUn	37304094	37304719	626	CarSat1	red	na
chrUn	37304749	37306206	1458	CarSat1	red	na
chrUn	37306221	37306943	723	CarSat1	red	na
chrUn	37306958	37309266	2309	CarSat1	red	na
chrUn	37309464	37313424	3961	CarSat1	red	na
chrUn	37313488	37314013	526	SAT1_CF	blue	na
chrUn	37314066	37314560	495	SAT1_CF	blue	na
chrUn	37314580	37315261	682	SAT1_CF	blue	na
chrUn	37315274	37315951	678	SAT1_CF	blue	na
chrUn	37315993	37317486	1494	SAT1_CF	blue	na
chrUn	37317532	37318074	543	SAT1_CF	blue	na
chrUn	37318087	37318586	500	SAT1_CF	blue	na
chrUn	37318599	37320565	1967	SAT1_CF	blue	na
chrUn	37320607	37322529	1923	SAT1_CF	blue	na
chrUn	37322572	37323389	818	SAT1_CF	blue	na
chrUn	37323565	37323738	174	SAT1_CF	blue	na
chrUn	37323817	37324779	963	SAT1_CF	blue	na
chrUn	37324837	37325319	483	SAT1_CF	blue	na
chrUn	37325337	37325503	167	SAT1_CF	blue	na
chrUn	37325515	37325624	110	SAT1_CF	blue	na
chrUn	37325645	37326188	544	SAT1_CF	blue	na
chrUn	37326231	37327858	1628	SAT1_CF	blue	na
chrUn	37327902	37330380	2479	SAT1_CF	blue	na
chrUn	37330437	37330918	482	SAT1_CF	blue	na
chrUn	37330936	37332005	1070	SAT1_CF	blue	na
chrUn	37332028	37332508	481	SAT1_CF	blue	na
chrUn	37332543	37333381	839	SAT1_CF	blue	na
chrUn	37333398	37334092	695	SAT1_CF	blue	na
chrUn	37334116	37334688	573	SAT1_CF	blue	na
chrUn	37334709	37335221	513	SAT1_CF	blue	na
chrUn	37335232	37335942	711	SAT1_CF	blue	na
chrUn	37335955	37337040	1086	SAT1_CF	blue	na

chrUn	37337060	37338079	1020	SAT1_CF	blue	na
chrUn	37338119	37339394	1276	SAT1_CF	blue	na
chrUn	37339406	37340079	674	SAT1_CF	blue	na
chrUn	37340122	37341087	966	SAT1_CF	blue	na
chrUn	37341131	37342167	1037	SAT1_CF	blue	na
chrUn	37342203	37342698	496	SAT1_CF	blue	na
chrUn	37342718	37343927	1210	SAT1_CF	blue	na
chrUn	37343950	37344076	127	SAT1_CF	blue	na
chrUn	37344089	37344693	605	SAT1_CF	blue	na
chrUn	37344705	37345745	1041	SAT1_CF	blue	na
chrUn	37345758	37346441	684	SAT1_CF	blue	na
chrUn	37346453	37347360	908	SAT1_CF	blue	na
chrUn	37347383	37348132	750	SAT1_CF	blue	na
chrUn	37348211	37349172	962	SAT1_CF	blue	na
chrUn	37349229	37349712	484	SAT1_CF	blue	na
chrUn	37349725	37350483	759	SAT1_CF	blue	na
chrUn	37350495	37351587	1093	SAT1_CF	blue	na
chrUn	37351615	37352138	524	SAT1_CF	blue	na
chrUn	37352153	37352856	704	SAT1_CF	blue	na
chrUn	37352882	37353553	672	SAT1_CF	blue	na
chrUn	37353565	37354240	676	SAT1_CF	blue	na
chrUn	37354301	37355165	865	SAT1_CF	blue	na
chrUn	37355185	37355289	105	SAT1_CF	blue	na
chrUn	37355314	37357066	1753	SAT1_CF	blue	na
chrUn	37357093	37357781	689	SAT1_CF	blue	na
chrUn	37357795	37358782	988	SAT1_CF	blue	na
chrUn	37358810	37358967	158	SAT1_CF	blue	na
chrUn	37359012	37359513	502	SAT1_CF	blue	na
chrUn	37359525	37359634	110	SAT1_CF	blue	na
chrUn	37359655	37360286	632	SAT1_CF	blue	na
chrUn	37360298	37360962	665	SAT1_CF	blue	na
chrUn	37361019	37361730	712	SAT1_CF	blue	na
chrUn	37361772	37362424	653	SAT1_CF	blue	na
chrUn	37373545	37374099	555	Sat2CF	purple	na
chrUn	37382958	37383691	734	Sat2CF	purple	na
chrUn	37383033	37384161	1129	Sat2CF	purple	na
chrUn	37402509	37403738	1230	Sat2CF	purple	na
chrUn	37416535	37417302	768	Sat2CF	purple	na
chrUn	37416550	37417237	688	Sat2CF	purple	na
chrUn	37431497	37432201	705	CarSat1	red	na
chrUn	37432228	37433801	1574	CarSat1	red	na
chrUn	37433822	37434676	855	CarSat1	red	na

chrUn	37434698	37435554	857	CarSat1	red	na
chrUn	37435713	37436353	641	CarSat1	red	na
chrUn	37436371	37436810	440	CarSat1	red	na
chrUn	37436813	37437151	339	SAT1_CF	blue	na
chrUn	37447361	37448126	766	Sat2CF	purple	na
chrUn	37472217	37473373	1157	Sat2CF	purple	na
chrUn	37473399	37474293	895	Sat2CF	purple	na
chrUn	37505059	37506276	1218	Sat2CF	purple	na
chrUn	37536987	37537366	380	Sat2CF	purple	na
chrUn	37537049	37537763	715	Sat2CF	purple	na
chrUn	37542341	37542770	430	SAT1_CF	blue	na
chrUn	37559805	37561286	1482	CarSat1	red	na
chrUn	37561314	37562164	851	CarSat1	red	na
chrUn	37562185	37562419	235	CarSat1	red	na
chrUn	37562472	37563769	1298	CarSat1	red	na
chrUn	37566453	37567703	1251	Sat2CF	purple	na
chrUn	37580032	37580828	797	Sat2CF	purple	na
chrUn	37595738	37595915	178	Sat2CF	purple	na
chrUn	37595786	37596906	1121	Sat2CF	purple	na
chrUn	37597305	37597829	525	CarSat1	red	na
chrUn	37597850	37599627	1778	CarSat1	red	na
chrUn	37599640	37600373	734	CarSat1	red	na
chrUn	37600559	37600770	212	CarSat1	red	na
chrUn	37600775	37604084	3310	SAT1_CF	blue	na
chrUn	37604105	37605940	1836	SAT1_CF	blue	na
chrUn	37605986	37607265	1280	SAT1_CF	blue	na
chrUn	37607286	37608432	1147	SAT1_CF	blue	na
chrUn	37608432	37609146	715	CarSat1	red	na
chrUn	37609165	37609603	439	CarSat1	red	na
chrUn	37614711	37615137	427	CarSat1	red	na
chrUn	37615245	37615893	649	CarSat1	red	na
chrUn	37615888	37616901	1014	SAT1_CF	blue	na
chrUn	37616904	37617343	440	CarSat1	red	na
chrUn	37617361	37618138	778	CarSat1	red	na
chrUn	37618159	37619017	859	CarSat1	red	na
chrUn	37619039	37619892	854	CarSat1	red	na
chrUn	37619913	37621493	1581	CarSat1	red	na
chrUn	37621523	37622227	705	CarSat1	red	na
chrUn	37643176	37643946	771	Sat2CF	purple	na
chrUn	37679347	37679435	89	Sat2CF	purple	na
chrUn	37688340	37688894	555	Sat2CF	purple	na
chrUn	37691259	37693441	2183	CarSat1	red	na

chrUn	37693456	37693581	126	Carsat2	orange	na
chrUn	37693471	37695426	1956	CarSat1	red	na
chrUn	37695460	37696163	704	CarSat1	red	na
chrUn	37696187	37697291	1105	CarSat1	red	na
chrUn	37697476	37698078	603	CarSat1	red	na
chrUn	37698108	37698345	238	CarSat1	red	na
chrUn	37698528	37702660	4133	CarSat1	red	na
chrUn	37703419	37704917	1499	CarSat1	red	na
chrUn	37704947	37706383	1437	CarSat1	red	na
chrUn	37706413	37708658	2246	CarSat1	red	na
chrUn	37708839	37709296	458	CarSat1	red	na
chrUn	37709377	37711673	2297	CarSat1	red	na
chrUn	37711714	37712446	733	CarSat1	red	na
chrUn	37712730	37713363	634	CarSat1	red	na
chrUn	37713396	37714091	696	CarSat1	red	na
chrUn	37714116	37715033	918	CarSat1	red	na
chrUn	37715071	37715818	748	CarSat1	red	na
chrUn	37796756	37797146	391	SAT1_CF	blue	na
chrUn	37797216	37798422	1207	SAT1_CF	blue	na
chrUn	37798465	37798770	306	SAT1_CF	blue	na
chrUn	37798907	37799448	542	SAT1_CF	blue	na
chrUn	37799463	37799962	500	SAT1_CF	blue	na
chrUn	37799975	37800639	665	SAT1_CF	blue	na
chrUn	37800672	37801683	1012	SAT1_CF	blue	na
chrUn	37801718	37802787	1070	SAT1_CF	blue	na
chrUn	37802808	37803937	1130	SAT1_CF	blue	na
chrUn	37804024	37804652	629	SAT1_CF	blue	na
chrUn	37804679	37804937	259	SAT1_CF	blue	na
chrUn	37804971	37805534	564	SAT1_CF	blue	na
chrUn	37805577	37806101	525	SAT1_CF	blue	na
chrUn	37806116	37806252	137	SAT1_CF	blue	na
chrUn	37806299	37807258	960	SAT1_CF	blue	na
chrUn	37807276	37807953	678	SAT1_CF	blue	na
chrUn	37807972	37808074	103	SAT1_CF	blue	na
chrUn	37808095	37808648	554	SAT1_CF	blue	na
chrUn	37808660	37809519	860	SAT1_CF	blue	na
chrUn	37809561	37811007	1447	SAT1_CF	blue	na
chrUn	37811050	37811853	804	SAT1_CF	blue	na
chrUn	37811867	37812584	718	SAT1_CF	blue	na
chrUn	37812597	37813217	621	SAT1_CF	blue	na
chrUn	37813288	37815287	2000	SAT1_CF	blue	na
chrUn	37815329	37816219	891	SAT1_CF	blue	na

chrUn	37816263	37816522	260	SAT1_CF	blue	na
chrUn	37816560	37817062	503	SAT1_CF	blue	na
chrUn	37817073	37818832	1760	SAT1_CF	blue	na
chrUn	37818844	37819243	400	SAT1_CF	blue	na
chrUn	37819303	37821206	1904	SAT1_CF	blue	na
chrUn	37821250	37821848	599	SAT1_CF	blue	na
chrUn	37821874	37822925	1052	SAT1_CF	blue	na
chrUn	37822965	37825623	2659	SAT1_CF	blue	na
chrUn	37825637	37826156	520	SAT1_CF	blue	na
chrUn	37826223	37826359	137	SAT1_CF	blue	na
chrUn	37826425	37827620	1196	SAT1_CF	blue	na
chrUn	37827632	37827705	74	SAT1_CF	blue	na
chrUn	37827762	37828230	469	SAT1_CF	blue	na
chrUn	37828244	37828918	675	SAT1_CF	blue	na
chrUn	37828961	37829767	807	SAT1_CF	blue	na
chrUn	37829854	37830338	485	SAT1_CF	blue	na
chrUn	37830381	37831003	623	SAT1_CF	blue	na
chrUn	37831029	37831512	484	SAT1_CF	blue	na
chrUn	37831525	37832200	676	SAT1_CF	blue	na
chrUn	37832243	37834263	2021	SAT1_CF	blue	na
chrUn	37834318	37834820	503	SAT1_CF	blue	na
chrUn	37834833	37835689	857	SAT1_CF	blue	na
chrUn	37835702	37836902	1201	SAT1_CF	blue	na
chrUn	37836967	37837124	158	SAT1_CF	blue	na
chrUn	37837169	37837670	502	SAT1_CF	blue	na
chrUn	37837683	37837789	107	SAT1_CF	blue	na
chrUn	37837812	37838404	593	SAT1_CF	blue	na
chrUn	37839752	37839948	197	SAT1_CF	blue	na
chrUn	37839967	37840694	728	SAT1_CF	blue	na
chrUn	37840736	37842576	1841	SAT1_CF	blue	na
chrUn	37842631	37843130	500	SAT1_CF	blue	na
chrUn	37843143	37844645	1503	SAT1_CF	blue	na
chrUn	37844665	37844801	137	SAT1_CF	blue	na
chrUn	37844897	37845441	545	SAT1_CF	blue	na
chrUn	37845453	37845525	73	SAT1_CF	blue	na
chrUn	37845583	37846214	632	SAT1_CF	blue	na
chrUn	37846236	37848979	2744	SAT1_CF	blue	na
chrUn	37849022	37849524	503	SAT1_CF	blue	na
chrUn	37849537	37851678	2142	SAT1_CF	blue	na
chrUn	37851692	37852213	522	SAT1_CF	blue	na
chrUn	37852279	37852415	137	SAT1_CF	blue	na
chrUn	37852481	37853670	1190	SAT1_CF	blue	na

chrUn	37853717	37854439	723	SAT1_CF	blue	na
chrUn	37854864	37855912	1049	SAT1_CF	blue	na
chrUn	37855924	37856607	684	SAT1_CF	blue	na
chrUn	37856619	37856728	110	SAT1_CF	blue	na
chrUn	37856749	37857217	469	SAT1_CF	blue	na
chrUn	37857231	37858746	1516	SAT1_CF	blue	na
chrUn	37858840	37859324	485	SAT1_CF	blue	na
chrUn	37859340	37859995	656	SAT1_CF	blue	na
chrUn	37860014	37860498	485	SAT1_CF	blue	na
chrUn	37860525	37862353	1829	SAT1_CF	blue	na
chrUn	37862366	37864672	2307	SAT1_CF	blue	na
chrUn	37864684	37865914	1231	SAT1_CF	blue	na
chrUn	37865957	37866468	512	SAT1_CF	blue	na
chrUn	37866496	37866652	157	SAT1_CF	blue	na
chrUn	37866678	37867319	642	SAT1_CF	blue	na
chrUn	37878518	37879156	639	SAT1_CF	blue	na
chrUn	37879177	37880111	935	SAT1_CF	blue	na
chrUn	37880143	37880662	520	SAT1_CF	blue	na
chrUn	37880676	37881215	540	SAT1_CF	blue	na
chrUn	37881258	37881935	678	SAT1_CF	blue	na
chrUn	37881947	37882575	629	SAT1_CF	blue	na
chrUn	37882655	37883325	671	SAT1_CF	blue	na
chrUn	37883345	37884542	1198	SAT1_CF	blue	na
chrUn	37884587	37884744	158	SAT1_CF	blue	na
chrUn	37884772	37885930	1159	SAT1_CF	blue	na
chrUn	37885942	37886764	823	SAT1_CF	blue	na
chrUn	37886776	37888022	1247	SAT1_CF	blue	na
chrUn	37888065	37888321	257	SAT1_CF	blue	na
chrUn	37888355	37888869	515	SAT1_CF	blue	na
chrUn	37888916	37889052	137	SAT1_CF	blue	na
chrUn	37889080	37890944	1865	SAT1_CF	blue	na
chrUn	37890987	37891663	677	SAT1_CF	blue	na
chrUn	37891675	37892175	501	SAT1_CF	blue	na
chrUn	37892241	37892378	138	SAT1_CF	blue	na
chrUn	37892405	37894097	1693	SAT1_CF	blue	na
chrUn	37894154	37894649	496	SAT1_CF	blue	na
chrUn	37894675	37895723	1049	SAT1_CF	blue	na
chrUn	37895761	37896302	542	SAT1_CF	blue	na
chrUn	37896344	37897204	861	SAT1_CF	blue	na
chrUn	37897216	37897769	554	SAT1_CF	blue	na
chrUn	37897812	37898374	563	SAT1_CF	blue	na
chrUn	37898439	37898981	543	SAT1_CF	blue	na

chrUn	37899023	37899883	861	SAT1_CF	blue	na
chrUn	37899895	37900578	684	SAT1_CF	blue	na
chrUn	37900598	37901092	495	SAT1_CF	blue	na
chrUn	37901177	37901696	520	SAT1_CF	blue	na
chrUn	37903118	37903818	701	SAT1_CF	blue	na
chrUn	37903861	37904259	399	SAT1_CF	blue	na
chrUn	37904270	37904878	609	SAT1_CF	blue	na
chrUn	37904933	37906613	1681	SAT1_CF	blue	na
chrUn	37906626	37908416	1791	SAT1_CF	blue	na
chrUn	37908458	37909107	650	SAT1_CF	blue	na
chrUn	37909119	37909670	552	SAT1_CF	blue	na
chrUn	37909692	37909800	109	SAT1_CF	blue	na
chrUn	37909820	37910314	495	SAT1_CF	blue	na
chrUn	37910401	37910918	518	SAT1_CF	blue	na
chrUn	37912340	37912913	574	SAT1_CF	blue	na
chrUn	37912953	37913157	205	SAT1_CF	blue	na
chrUn	37913182	37914386	1205	SAT1_CF	blue	na
chrUn	37914409	37914858	450	SAT1_CF	blue	na
chrUn	37914946	37915233	288	SAT1_CF	blue	na
chrUn	37915246	37915748	503	SAT1_CF	blue	na
chrUn	37915794	37916120	327	SAT1_CF	blue	na
chrUn	37916132	37917756	1625	SAT1_CF	blue	na
chrUn	37917799	37918344	546	SAT1_CF	blue	na
chrUn	37918364	37918473	110	SAT1_CF	blue	na
chrUn	37918485	37919088	604	SAT1_CF	blue	na
chrUn	37919101	37919583	483	SAT1_CF	blue	na
chrUn	37919656	37922213	2558	SAT1_CF	blue	na
chrUn	37922227	37923009	783	SAT1_CF	blue	na
chrUn	37923021	37923696	676	SAT1_CF	blue	na
chrUn	37923718	37924220	503	SAT1_CF	blue	na
chrUn	37924286	37924422	137	SAT1_CF	blue	na
chrUn	37924437	37927457	3021	SAT1_CF	blue	na
chrUn	37927479	37927586	108	SAT1_CF	blue	na
chrUn	37927599	37929839	2241	SAT1_CF	blue	na
chrUn	37929861	37931019	1159	SAT1_CF	blue	na
chrUn	37931088	37931630	543	SAT1_CF	blue	na
chrUn	37931651	37931990	340	SAT1_CF	blue	na
chrUn	37932033	37932578	546	SAT1_CF	blue	na
chrUn	37932599	37932707	109	SAT1_CF	blue	na
chrUn	37932720	37933240	521	SAT1_CF	blue	na
chrUn	37933287	37933423	137	SAT1_CF	blue	na
chrUn	37933475	37934478	1004	SAT1_CF	blue	na

chrUn	37934522	37935484	963	SAT1_CF	blue	na
chrUn	37935527	37936193	667	SAT1_CF	blue	na
chrUn	37936206	37936907	702	SAT1_CF	blue	na
chrUn	37936972	37938373	1402	SAT1_CF	blue	na
chrUn	37938386	37939468	1083	SAT1_CF	blue	na
chrUn	37940469	37941319	851	CarSat1	red	na
chrUn	37941335	37942055	721	CarSat1	red	na
chrUn	37942071	37943030	960	CarSat1	red	na
chrUn	37943057	37944264	1208	CarSat1	red	na
chrUn	37944288	37944774	487	CarSat1	red	na
chrUn	37944804	37946983	2180	CarSat1	red	na
chrUn	37947013	37950154	3142	CarSat1	red	na
chrUn	37950178	37952359	2182	CarSat1	red	na
chrUn	37952383	37953093	711	CarSat1	red	na
chrUn	37953117	37953830	714	CarSat1	red	na
chrUn	37953854	37954814	961	CarSat1	red	na
chrUn	37954835	37957685	2851	CarSat1	red	na
chrUn	37958615	37964700	6086	CarSat1	red	na
chrUn	37964724	37967651	2928	CarSat1	red	na
chrUn	37967675	37969121	1447	CarSat1	red	na
chrUn	37969137	37973577	4441	CarSat1	red	na
chrUn	37973648	37978838	5191	CarSat1	red	na
chrUn	37978868	37981700	2833	CarSat1	red	na
chrUn	37984096	37986229	2134	CarSat1	red	na
chrUn	37986263	37987703	1441	CarSat1	red	na
chrUn	37987737	37989187	1451	CarSat1	red	na
chrUn	37989211	37991905	2695	CarSat1	red	na
chrUn	37991936	37993379	1444	CarSat1	red	na
chrUn	37993409	37996804	3396	CarSat1	red	na
chrUn	37996825	37997736	912	CarSat1	red	na
chrUn	37998571	38000215	1645	CarSat1	red	na
chrUn	38000230	38004341	4112	CarSat1	red	na
chrUn	38005150	38008176	3027	CarSat1	red	na
chrUn	38008253	38008979	727	CarSat1	red	na
chrUn	38008994	38010171	1178	CarSat1	red	na
chrUn	38010198	38010667	470	CarSat1	red	na
chrUn	38010683	38011404	722	CarSat1	red	na
chrUn	38011420	38011916	497	CarSat1	red	na
chrUn	38011946	38012889	944	CarSat1	red	na
chrUn	38012914	38014068	1155	CarSat1	red	na
chrUn	38084503	38085930	1428	CarSat1	red	na
chrUn	38087849	38087973	125	CarSat1	red	na

chrUn	38123303	38124391	1089	Sat2CF	purple	na
chrUn	38123319	38124520	1202	Sat2CF	purple	na
chrUn	38140084	38140252	169	Sat2CF	purple	na
chrUn	38140121	38140951	831	Sat2CF	purple	na
chrUn	38140142	38140867	726	Sat2CF	purple	na
chrUn	38155853	38156696	844	Sat2CF	purple	na
chrUn	38156716	38157223	508	Sat2CF	purple	na
chrUn	38164629	38166540	1912	CarSat1	red	na
chrUn	38166561	38167276	716	CarSat1	red	na
chrUn	38167303	38168264	962	CarSat1	red	na
chrUn	38168280	38169216	937	CarSat1	red	na
chrUn	38169246	38169732	487	CarSat1	red	na
chrUn	38169756	38171424	1669	CarSat1	red	na
chrUn	38171454	38173631	2178	CarSat1	red	na
chrUn	38173648	38173771	124	Carsat2	orange	na
chrUn	38173661	38177951	4291	CarSat1	red	na
chrUn	38178342	38179236	895	CarSat1	red	na
chrUn	38179260	38180710	1451	CarSat1	red	na
chrUn	38180734	38183875	3142	CarSat1	red	na
chrUn	38183905	38184613	709	CarSat1	red	na
chrUn	38184643	38185129	487	CarSat1	red	na
chrUn	38185153	38186817	1665	CarSat1	red	na
chrUn	38186847	38189542	2696	CarSat1	red	na
chrUn	38189566	38191012	1447	CarSat1	red	na
chrUn	38191264	38192008	745	CarSat1	red	na
chrUn	38193172	38194132	961	CarSat1	red	na
chrUn	38194156	38194869	714	CarSat1	red	na
chrUn	38194902	38195607	706	CarSat1	red	na
chrUn	38195631	38196342	712	CarSat1	red	na
chrUn	38196366	38197394	1029	CarSat1	red	na
chrUn	38197583	38198182	600	CarSat1	red	na
chrUn	38198371	38199774	1404	CarSat1	red	na
chrUn	38199804	38199865	62	CarSat1	red	na
chrUn	38200054	38205157	5104	CarSat1	red	na
chrUn	38205168	38206390	1223	CarSat1	red	na
chrUn	38206414	38207139	726	CarSat1	red	na
chrUn	38207155	38207623	469	CarSat1	red	na
chrUn	38207650	38209096	1447	CarSat1	red	na
chrUn	38209123	38209538	416	CarSat1	red	na
chrUn	38223950	38227299	3350	CarSat1	red	na
chrUn	38227332	38228037	706	CarSat1	red	na
chrUn	38228070	38229510	1441	CarSat1	red	na

chrUn	38229534	38230246	713	CarSat1	red	na
chrUn	38230270	38230344	75	CarSat1	red	na
chrUn	38230539	38231506	968	CarSat1	red	na
chrUn	38231743	38232717	975	CarSat1	red	na
chrUn	38232741	38234927	2187	CarSat1	red	na
chrUn	38234960	38235886	927	CarSat1	red	na
chrUn	38235901	38236027	127	Carsat2	orange	na
chrUn	38235916	38237362	1447	CarSat1	red	na
chrUn	38237392	38238840	1449	CarSat1	red	na
chrUn	38238870	38240318	1449	CarSat1	red	na
chrUn	38240348	38242534	2187	CarSat1	red	na
chrUn	38242564	38243271	708	CarSat1	red	na
chrUn	38243286	38243412	127	Carsat2	orange	na
chrUn	38243301	38243785	485	CarSat1	red	na
chrUn	38243822	38244744	923	CarSat1	red	na
chrUn	38244759	38244885	127	Carsat2	orange	na
chrUn	38244774	38245258	485	CarSat1	red	na
chrUn	38245295	38249068	3774	CarSat1	red	na
chrUn	38331713	38332256	544	SAT1_CF	blue	na
chrUn	38332298	38333779	1482	SAT1_CF	blue	na
chrUn	38333791	38334290	500	SAT1_CF	blue	na
chrUn	38334301	38334699	399	SAT1_CF	blue	na
chrUn	38334742	38335710	969	SAT1_CF	blue	na
chrUn	38335753	38336717	965	SAT1_CF	blue	na
chrUn	38336908	38337286	379	SAT1_CF	blue	na
chrUn	38337304	38338393	1090	SAT1_CF	blue	na
chrUn	38338433	38338835	403	SAT1_CF	blue	na
chrUn	38338846	38339365	520	SAT1_CF	blue	na
chrUn	38339398	38340806	1409	SAT1_CF	blue	na
chrUn	38340876	38341549	674	SAT1_CF	blue	na
chrUn	38341561	38342114	554	SAT1_CF	blue	na
chrUn	38342157	38342682	526	SAT1_CF	blue	na
chrUn	38342726	38344163	1438	SAT1_CF	blue	na
chrUn	38344181	38344824	644	SAT1_CF	blue	na
chrUn	38344837	38345598	762	SAT1_CF	blue	na
chrUn	38345611	38346112	502	SAT1_CF	blue	na
chrUn	38346199	38347386	1188	SAT1_CF	blue	na
chrUn	38347428	38347972	545	SAT1_CF	blue	na
chrUn	38348015	38348691	677	SAT1_CF	blue	na
chrUn	38348704	38349277	574	SAT1_CF	blue	na
chrUn	38349309	38349406	98	SAT1_CF	blue	na
chrUn	38349419	38351300	1882	SAT1_CF	blue	na

chrUn	38351312	38352001	690	SAT1_CF	blue	na
chrUn	38352015	38352674	660	SAT1_CF	blue	na
chrUn	38352689	38353883	1195	SAT1_CF	blue	na
chrUn	38353906	38354011	106	SAT1_CF	blue	na
chrUn	38354024	38354524	501	SAT1_CF	blue	na
chrUn	38354569	38356419	1851	SAT1_CF	blue	na
chrUn	38356461	38357528	1068	SAT1_CF	blue	na
chrUn	38357551	38357622	72	SAT1_CF	blue	na
chrUn	38357635	38358690	1056	SAT1_CF	blue	na
chrUn	38358711	38358820	110	SAT1_CF	blue	na
chrUn	38358832	38359334	503	SAT1_CF	blue	na
chrUn	38359421	38360299	879	SAT1_CF	blue	na
chrUn	38360366	38361328	963	SAT1_CF	blue	na
chrUn	38361371	38361869	499	SAT1_CF	blue	na
chrUn	38361891	38362565	675	SAT1_CF	blue	na
chrUn	38362588	38362694	107	SAT1_CF	blue	na
chrUn	38362706	38363208	503	SAT1_CF	blue	na
chrUn	38363274	38363410	137	SAT1_CF	blue	na
chrUn	38363438	38364084	647	SAT1_CF	blue	na
chrUn	38365337	38366033	697	SAT1_CF	blue	na
chrUn	38366053	38366601	549	SAT1_CF	blue	na
chrUn	38366614	38367505	892	SAT1_CF	blue	na
chrUn	38367517	38368035	519	SAT1_CF	blue	na
chrUn	38368107	38369738	1632	SAT1_CF	blue	na
chrUn	38369780	38370516	737	SAT1_CF	blue	na
chrUn	38370535	38371125	591	SAT1_CF	blue	na
chrUn	38371212	38371543	332	SAT1_CF	blue	na
chrUn	38371576	38373167	1592	SAT1_CF	blue	na
chrUn	38373182	38373341	160	SAT1_CF	blue	na
chrUn	38373650	38373888	239	SAT1_CF	blue	na
chrUn	38373902	38374231	330	SAT1_CF	blue	na
chrUn	38374271	38374940	670	SAT1_CF	blue	na
chrUn	38374954	38375444	491	SAT1_CF	blue	na
chrUn	38375457	38375966	510	SAT1_CF	blue	na
chrUn	38375980	38376858	879	SAT1_CF	blue	na
chrUn	38376906	38377568	663	SAT1_CF	blue	na
chrUn	38377602	38378095	494	SAT1_CF	blue	na
chrUn	38378182	38378697	516	SAT1_CF	blue	na
chrUn	38378769	38379299	531	SAT1_CF	blue	na
chrUn	38379461	38380429	969	SAT1_CF	blue	na
chrUn	38380478	38381722	1245	SAT1_CF	blue	na
chrUn	38381743	38381851	109	SAT1_CF	blue	na

chrUn	38381864	38382366	503	SAT1_CF	blue	na
chrUn	38382411	38383324	914	SAT1_CF	blue	na
chrUn	38383368	38383669	302	SAT1_CF	blue	na
chrUn	38383717	38385051	1335	SAT1_CF	blue	na
chrUn	38385064	38385585	522	SAT1_CF	blue	na
chrUn	38385604	38385871	268	SAT1_CF	blue	na
chrUn	38385916	38387162	1247	SAT1_CF	blue	na
chrUn	38387174	38387724	551	SAT1_CF	blue	na
chrUn	38387829	38388756	928	SAT1_CF	blue	na
chrUn	38388768	38389451	684	SAT1_CF	blue	na
chrUn	38389463	38391643	2181	SAT1_CF	blue	na
chrUn	38391706	38392264	559	SAT1_CF	blue	na
chrUn	38392324	38393668	1345	SAT1_CF	blue	na
chrUn	38393695	38394326	632	SAT1_CF	blue	na
chrUn	38394369	38395033	665	SAT1_CF	blue	na
chrUn	38395053	38395548	496	SAT1_CF	blue	na
chrUn	38395601	38396545	945	SAT1_CF	blue	na
chrUn	38396570	38397387	818	SAT1_CF	blue	na
chrUn	38397415	38397608	194	SAT1_CF	blue	na
chrUn	38397639	38398159	521	SAT1_CF	blue	na
chrUn	38398215	38398373	159	SAT1_CF	blue	na
chrUn	38398525	38399291	767	SAT1_CF	blue	na
chrUn	38399483	38399893	411	SAT1_CF	blue	na
chrUn	38399913	38400386	474	SAT1_CF	blue	na
chrUn	38400451	38400545	95	SAT1_CF	blue	na
chrUn	38470017	38470210	194	SAT1_CF	blue	na
chrUn	38470435	38471166	732	SAT1_CF	blue	na
chrUn	38471184	38473256	2073	SAT1_CF	blue	na
chrUn	38473438	38473619	182	SAT1_CF	blue	na
chrUn	38473659	38474159	501	SAT1_CF	blue	na
chrUn	38474172	38474277	106	SAT1_CF	blue	na
chrUn	38474300	38475108	809	SAT1_CF	blue	na
chrUn	38475125	38475648	524	SAT1_CF	blue	na
chrUn	38475663	38475799	137	SAT1_CF	blue	na
chrUn	38475865	38476977	1113	SAT1_CF	blue	na
chrUn	38477058	38477581	524	SAT1_CF	blue	na
chrUn	38477596	38477752	157	SAT1_CF	blue	na
chrUn	38477797	38478297	501	SAT1_CF	blue	na
chrUn	38478310	38478984	675	SAT1_CF	blue	na
chrUn	38479027	38481018	1992	SAT1_CF	blue	na
chrUn	38481135	38481669	535	SAT1_CF	blue	na
chrUn	38481689	38481796	108	SAT1_CF	blue	na

chrUn	38481817	38482387	571	SAT1_CF	blue	na
chrUn	38482399	38483471	1073	SAT1_CF	blue	na
chrUn	38483483	38485883	2401	SAT1_CF	blue	na
chrUn	38485895	38487252	1358	SAT1_CF	blue	na
chrUn	38487265	38487617	353	SAT1_CF	blue	na
chrUn	38487733	38488252	520	SAT1_CF	blue	na
chrUn	38488309	38489475	1167	SAT1_CF	blue	na
chrUn	38489564	38490064	501	SAT1_CF	blue	na
chrUn	38490077	38490780	704	SAT1_CF	blue	na
chrUn	38490791	38491395	605	SAT1_CF	blue	na
chrUn	38491438	38491982	545	SAT1_CF	blue	na
chrUn	38492115	38492811	697	SAT1_CF	blue	na
chrUn	38492876	38493033	158	SAT1_CF	blue	na
chrUn	38493078	38493578	501	SAT1_CF	blue	na
chrUn	38493605	38494272	668	SAT1_CF	blue	na
chrUn	38494284	38494961	678	SAT1_CF	blue	na
chrUn	38495004	38497284	2281	SAT1_CF	blue	na
chrUn	38497328	38498988	1661	SAT1_CF	blue	na
chrUn	38499015	38499578	564	SAT1_CF	blue	na
chrUn	38499658	38502106	2449	SAT1_CF	blue	na
chrUn	38502148	38502968	821	SAT1_CF	blue	na
chrUn	38503000	38503522	523	SAT1_CF	blue	na
chrUn	38503533	38503767	235	SAT1_CF	blue	na
chrUn	38503947	38504114	168	SAT1_CF	blue	na
chrUn	38504188	38504659	472	SAT1_CF	blue	na
chrUn	38504682	38506004	1323	SAT1_CF	blue	na
chrUn	38506019	38506176	158	SAT1_CF	blue	na
chrUn	38506202	38509119	2918	SAT1_CF	blue	na
chrUn	38509200	38509366	167	SAT1_CF	blue	na
chrUn	38509548	38509732	185	SAT1_CF	blue	na
chrUn	38509768	38510266	499	SAT1_CF	blue	na
chrUn	38510279	38510385	107	SAT1_CF	blue	na
chrUn	38510408	38511361	954	SAT1_CF	blue	na
chrUn	38511401	38511899	499	SAT1_CF	blue	na
chrUn	38511911	38513621	1711	SAT1_CF	blue	na
chrUn	38513649	38513805	157	SAT1_CF	blue	na
chrUn	38513850	38514350	501	SAT1_CF	blue	na
chrUn	38514363	38515122	760	SAT1_CF	blue	na
chrUn	38515147	38515992	846	SAT1_CF	blue	na
chrUn	38516035	38516817	783	SAT1_CF	blue	na
chrUn	38516897	38517442	546	SAT1_CF	blue	na
chrUn	38517453	38517806	354	SAT1_CF	blue	na

chrUn	38517922	38518442	521	SAT1_CF	blue	na
chrUn	38518473	38518634	162	SAT1_CF	blue	na
chrUn	38518695	38520081	1387	SAT1_CF	blue	na
chrUn	38520115	38520494	380	SAT1_CF	blue	na
chrUn	38520534	38521035	502	SAT1_CF	blue	na
chrUn	38521057	38521160	104	SAT1_CF	blue	na
chrUn	38521325	38521988	664	SAT1_CF	blue	na
chrUn	38522005	38523193	1189	SAT1_CF	blue	na
chrUn	38523281	38523478	198	SAT1_CF	blue	na
chrUn	38523499	38524020	522	SAT1_CF	blue	na
chrUn	38524033	38524384	352	SAT1_CF	blue	na
chrUn	38524437	38524937	501	SAT1_CF	blue	na
chrUn	38524959	38526020	1062	SAT1_CF	blue	na
chrUn	38526046	38526527	482	SAT1_CF	blue	na
chrUn	38526554	38529090	2537	SAT1_CF	blue	na
chrUn	38529111	38530046	936	SAT1_CF	blue	na
chrUn	38530067	38530589	523	SAT1_CF	blue	na
chrUn	38530602	38531283	682	SAT1_CF	blue	na
chrUn	38531295	38532057	763	SAT1_CF	blue	na
chrUn	38532069	38532770	702	SAT1_CF	blue	na
chrUn	38532850	38533809	960	SAT1_CF	blue	na
chrUn	38533869	38534351	483	SAT1_CF	blue	na
chrUn	38534363	38535040	678	SAT1_CF	blue	na
chrUn	38535083	38535711	629	SAT1_CF	blue	na
chrUn	38535739	38536537	799	SAT1_CF	blue	na
chrUn	38541124	38542021	898	Sat2CF	purple	na
chrUn	38541169	38541958	790	Sat2CF	purple	na
chrUn	38568308	38569559	1252	Sat2CF	purple	na
chrUn	38593350	38593844	495	CarSat1	red	na
chrUn	38594029	38594253	225	CarSat1	red	na
chrUn	38594401	38595860	1460	CarSat1	red	na
chrUn	38595881	38598942	3062	CarSat1	red	na
chrUn	38598963	38600202	1240	CarSat1	red	na
chrUn	38600205	38602075	1871	SAT1_CF	blue	na
chrUn	38605596	38606844	1249	SAT1_CF	blue	na
chrUn	38606873	38606971	99	SAT1_CF	blue	na
chrUn	38606993	38607539	547	SAT1_CF	blue	na
chrUn	38607552	38608197	646	SAT1_CF	blue	na
chrUn	38608241	38609399	1159	SAT1_CF	blue	na
chrUn	38609444	38609945	502	SAT1_CF	blue	na
chrUn	38609957	38610065	109	SAT1_CF	blue	na
chrUn	38610088	38610643	556	SAT1_CF	blue	na

chrUn	38610655	38611333	679	SAT1_CF	blue	na
chrUn	38611378	38613419	2042	SAT1_CF	blue	na
chrUn	38613460	38613961	502	SAT1_CF	blue	na
chrUn	38613974	38614648	675	SAT1_CF	blue	na
chrUn	38614691	38615322	632	SAT1_CF	blue	na
chrUn	38615350	38616546	1197	SAT1_CF	blue	na
chrUn	38616606	38620559	3954	SAT1_CF	blue	na
chrUn	38620582	38621940	1359	SAT1_CF	blue	na
chrUn	38621953	38622124	172	SAT1_CF	blue	na
chrUn	38622166	38623779	1614	SAT1_CF	blue	na
chrUn	38623826	38624347	522	SAT1_CF	blue	na
chrUn	38624360	38624966	607	SAT1_CF	blue	na
chrUn	38624978	38625086	109	SAT1_CF	blue	na
chrUn	38625106	38625885	780	SAT1_CF	blue	na
chrUn	38625913	38627682	1770	SAT1_CF	blue	na
chrUn	38627735	38628237	503	SAT1_CF	blue	na
chrUn	38628249	38628321	73	SAT1_CF	blue	na
chrUn	38628378	38629663	1286	SAT1_CF	blue	na
chrUn	38629674	38631385	1712	SAT1_CF	blue	na
chrUn	38631397	38632073	677	SAT1_CF	blue	na
chrUn	38632085	38632258	174	SAT1_CF	blue	na
chrUn	38632300	38634156	1857	SAT1_CF	blue	na
chrUn	38634196	38634771	576	SAT1_CF	blue	na
chrUn	38634793	38635467	675	SAT1_CF	blue	na
chrUn	38635479	38636162	684	SAT1_CF	blue	na
chrUn	38636204	38637054	851	SAT1_CF	blue	na
chrUn	38637124	38637669	546	SAT1_CF	blue	na
chrUn	38637712	38638343	632	SAT1_CF	blue	na
chrUn	38638371	38639030	660	SAT1_CF	blue	na
chrUn	38639048	38639550	503	SAT1_CF	blue	na
chrUn	38639583	38640368	786	SAT1_CF	blue	na
chrUn	38640429	38640983	555	SAT1_CF	blue	na
chrUn	38641001	38641675	675	SAT1_CF	blue	na
chrUn	38641772	38642314	543	SAT1_CF	blue	na
chrUn	38642358	38642897	540	SAT1_CF	blue	na
chrUn	38642928	38643453	526	SAT1_CF	blue	na
chrUn	38643468	38643603	136	SAT1_CF	blue	na
chrUn	38643688	38644170	483	SAT1_CF	blue	na
chrUn	38644183	38644288	106	SAT1_CF	blue	na
chrUn	38644311	38644865	555	SAT1_CF	blue	na
chrUn	38644878	38645553	676	SAT1_CF	blue	na
chrUn	38645596	38646606	1011	SAT1_CF	blue	na

chrUn	38646687	38647647	961	SAT1_CF	blue	na
chrUn	38647687	38648308	622	SAT1_CF	blue	na
chrUn	38648332	38648838	507	SAT1_CF	blue	na
chrUn	38648858	38649465	608	SAT1_CF	blue	na
chrUn	38649478	38649880	403	SAT1_CF	blue	na
chrUn	38649920	38650495	576	SAT1_CF	blue	na
chrUn	38650517	38650615	99	SAT1_CF	blue	na
chrUn	38650636	38651191	556	SAT1_CF	blue	na
chrUn	38651203	38652065	863	SAT1_CF	blue	na
chrUn	38652107	38652738	632	SAT1_CF	blue	na
chrUn	38652766	38653329	564	SAT1_CF	blue	na
chrUn	38653506	38654208	703	SAT1_CF	blue	na
chrUn	38654227	38654747	521	SAT1_CF	blue	na
chrUn	38654760	38656313	1554	SAT1_CF	blue	na
chrUn	38656355	38657857	1503	SAT1_CF	blue	na
chrUn	38657879	38658748	870	SAT1_CF	blue	na
chrUn	38658761	38658867	107	SAT1_CF	blue	na
chrUn	38658890	38659464	575	SAT1_CF	blue	na
chrUn	38659477	38661000	1524	SAT1_CF	blue	na
chrUn	38661042	38661586	545	SAT1_CF	blue	na
chrUn	38661602	38663393	1792	SAT1_CF	blue	na
chrUn	38663416	38663851	436	SAT1_CF	blue	na
chrUn	38663865	38663972	108	SAT1_CF	blue	na
chrUn	38663994	38664274	281	SAT1_CF	blue	na
chrUn	38664457	38664737	281	SAT1_CF	blue	na
chrUn	38664750	38666370	1621	SAT1_CF	blue	na
chrUn	38666403	38666782	380	SAT1_CF	blue	na
chrUn	38666822	38667324	503	SAT1_CF	blue	na
chrUn	38667336	38667989	654	SAT1_CF	blue	na
chrUn	38668046	38668774	729	SAT1_CF	blue	na
chrUn	38668816	38670626	1811	SAT1_CF	blue	na
chrUn	38670694	38671214	521	SAT1_CF	blue	na
chrUn	38671226	38671339	114	SAT1_CF	blue	na
chrUn	38671356	38671782	427	SAT1_CF	blue	na
chrUn	38672783	38673598	816	CarSat1	red	na
chrUn	38673614	38674844	1231	CarSat1	red	na
chrUn	38674874	38676055	1182	CarSat1	red	na
chrUn	38676077	38676789	713	CarSat1	red	na
chrUn	38676816	38677524	709	CarSat1	red	na
chrUn	38677551	38678999	1449	CarSat1	red	na
chrUn	38679026	38679486	461	CarSat1	red	na
chrUn	38679518	38680468	951	CarSat1	red	na

chrUn	38680495	38681198	704	CarSat1	red	na
chrUn	38681225	38682671	1447	CarSat1	red	na
chrUn	38682698	38683905	1208	CarSat1	red	na
chrUn	38683921	38684633	713	CarSat1	red	na
chrUn	38684658	38685620	963	CarSat1	red	na
chrUn	38685647	38687091	1445	CarSat1	red	na
chrUn	38687119	38687579	461	CarSat1	red	na
chrUn	38687604	38688566	963	CarSat1	red	na
chrUn	38688593	38689570	978	CarSat1	red	na
chrUn	38691184	38692255	1072	CarSat1	red	na
chrUn	38692282	38693488	1207	CarSat1	red	na
chrUn	38693504	38695208	1705	CarSat1	red	na
chrUn	38695230	38696944	1715	CarSat1	red	na
chrUn	38696969	38697601	633	CarSat1	red	na
chrUn	38697628	38698097	470	CarSat1	red	na
chrUn	38698113	38699074	962	CarSat1	red	na
chrUn	38699101	38703256	4156	CarSat1	red	na
chrUn	38703272	38704503	1232	CarSat1	red	na
chrUn	38705125	38706944	1820	CarSat1	red	na
chrUn	38706960	38708418	1459	CarSat1	red	na
chrUn	38708445	38709106	662	CarSat1	red	na
chrUn	38709607	38712102	2496	CarSat1	red	na
chrUn	38712118	38713578	1461	CarSat1	red	na
chrUn	38713594	38714555	962	CarSat1	red	na
chrUn	38714582	38716030	1449	CarSat1	red	na
chrUn	38716071	38716767	697	CarSat1	red	na
chrUn	38716794	38717504	711	CarSat1	red	na
chrUn	38717531	38718241	711	CarSat1	red	na
chrUn	38718268	38718977	710	CarSat1	red	na
chrUn	38719004	38719711	708	CarSat1	red	na
chrUn	38719738	38721181	1444	CarSat1	red	na
chrUn	38721208	38722656	1449	CarSat1	red	na
chrUn	38722683	38723151	469	CarSat1	red	na
chrUn	38723167	38724130	964	CarSat1	red	na
chrUn	38724157	38724866	710	CarSat1	red	na
chrUn	38724908	38725602	695	CarSat1	red	na
chrUn	38725629	38726337	709	CarSat1	red	na
chrUn	38726379	38729554	3176	CarSat1	red	na
chrUn	38729567	38730128	562	CarSat1	red	na
chrUn	38732024	38732942	919	CarSat1	red	na
chrUn	38749814	38750397	584	CarSat1	red	na
chrUn	38750424	38751135	712	CarSat1	red	na

chrUn	38751162	38752878	1717	CarSat1	red	na
chrUn	38752908	38754582	1675	CarSat1	red	na
chrUn	38754598	38755306	709	CarSat1	red	na
chrUn	38755331	38756291	961	CarSat1	red	na
chrUn	38756318	38757029	712	CarSat1	red	na
chrUn	38757056	38757768	713	CarSat1	red	na
chrUn	38757795	38758505	711	CarSat1	red	na
chrUn	38758532	38759000	469	CarSat1	red	na
chrUn	38759016	38760351	1336	CarSat1	red	na
chrUn	38847035	38848021	987	SAT1_CF	blue	na
chrUn	38848033	38848889	857	SAT1_CF	blue	na
chrUn	38848915	38849898	984	SAT1_CF	blue	na
chrUn	38849940	38850485	546	SAT1_CF	blue	na
chrUn	38850508	38851307	800	SAT1_CF	blue	na
chrUn	38851320	38851799	480	SAT1_CF	blue	na
chrUn	38851861	38852794	934	SAT1_CF	blue	na
chrUn	38852825	38853679	855	SAT1_CF	blue	na
chrUn	38853759	38854615	857	SAT1_CF	blue	na
chrUn	38854627	38855207	581	SAT1_CF	blue	na
chrUn	38855303	38855469	167	SAT1_CF	blue	na
chrUn	38855488	38856021	534	SAT1_CF	blue	na
chrUn	38856068	38856917	850	SAT1_CF	blue	na
chrUn	38856959	38857504	546	SAT1_CF	blue	na
chrUn	38857526	38858164	639	SAT1_CF	blue	na
chrUn	38858232	38858731	500	SAT1_CF	blue	na
chrUn	38858821	38860848	2028	SAT1_CF	blue	na
chrUn	38860860	38861828	969	SAT1_CF	blue	na
chrUn	38861841	38862362	522	SAT1_CF	blue	na
chrUn	38862377	38862955	579	SAT1_CF	blue	na
chrUn	38864369	38864795	427	SAT1_CF	blue	na
chrUn	38864862	38865403	542	SAT1_CF	blue	na
chrUn	38865473	38866149	677	SAT1_CF	blue	na
chrUn	38866161	38866666	506	SAT1_CF	blue	na
chrUn	38866698	38867506	809	SAT1_CF	blue	na
chrUn	38867551	38868219	669	SAT1_CF	blue	na
chrUn	38868237	38868999	763	SAT1_CF	blue	na
chrUn	38869019	38870148	1130	SAT1_CF	blue	na
chrUn	38870167	38871362	1196	SAT1_CF	blue	na
chrUn	38871376	38871876	501	SAT1_CF	blue	na
chrUn	38871966	38873438	1473	SAT1_CF	blue	na
chrUn	38873483	38874158	676	SAT1_CF	blue	na
chrUn	38874170	38874856	687	SAT1_CF	blue	na

chrUn	38874874	38875734	861	SAT1_CF	blue	na
chrUn	38875758	38876386	629	SAT1_CF	blue	na
chrUn	38876410	38876516	107	SAT1_CF	blue	na
chrUn	38876528	38877030	503	SAT1_CF	blue	na
chrUn	38877117	38878447	1331	SAT1_CF	blue	na
chrUn	38878493	38879825	1333	SAT1_CF	blue	na
chrUn	38879837	38880597	761	SAT1_CF	blue	na
chrUn	38880609	38881162	554	SAT1_CF	blue	na
chrUn	38881184	38881291	108	SAT1_CF	blue	na
chrUn	38881304	38881806	503	SAT1_CF	blue	na
chrUn	38881846	38882248	403	SAT1_CF	blue	na
chrUn	38882262	38882769	508	SAT1_CF	blue	na
chrUn	38882811	38883628	818	SAT1_CF	blue	na
chrUn	38883679	38884531	853	SAT1_CF	blue	na
chrUn	38884543	38885155	613	SAT1_CF	blue	na
chrUn	38885168	38885649	482	SAT1_CF	blue	na
chrUn	38885705	38885890	186	SAT1_CF	blue	na
chrUn	38886070	38886235	166	SAT1_CF	blue	na
chrUn	38886315	38886965	651	SAT1_CF	blue	na
chrUn	38886977	38888110	1134	SAT1_CF	blue	na
chrUn	38888123	38888644	522	SAT1_CF	blue	na
chrUn	38888663	38889577	915	SAT1_CF	blue	na
chrUn	38889644	38891249	1606	SAT1_CF	blue	na
chrUn	38891280	38891864	585	SAT1_CF	blue	na
chrUn	38891877	38892739	863	SAT1_CF	blue	na
chrUn	38892751	38893921	1171	SAT1_CF	blue	na
chrUn	38893977	38894563	587	SAT1_CF	blue	na
chrUn	38894605	38895006	402	SAT1_CF	blue	na
chrUn	38895017	38895104	88	SAT1_CF	blue	na
chrUn	38895202	38895876	675	SAT1_CF	blue	na
chrUn	38895888	38896569	682	SAT1_CF	blue	na
chrUn	38896582	38897064	483	SAT1_CF	blue	na
chrUn	38897128	38897525	398	SAT1_CF	blue	na
chrUn	38897539	38898045	507	SAT1_CF	blue	na
chrUn	38898089	38899030	942	SAT1_CF	blue	na
chrUn	38899079	38899752	674	SAT1_CF	blue	na
chrUn	38899770	38901837	2068	SAT1_CF	blue	na
chrUn	38901880	38902555	676	SAT1_CF	blue	na
chrUn	38902567	38903198	632	SAT1_CF	blue	na
chrUn	38903219	38903328	110	SAT1_CF	blue	na
chrUn	38903340	38903842	503	SAT1_CF	blue	na
chrUn	38903929	38904601	673	SAT1_CF	blue	na

chrUn	38904613	38905196	584	SAT1_CF	blue	na
chrUn	38905239	38906351	1113	SAT1_CF	blue	na
chrUn	38906394	38906939	546	SAT1_CF	blue	na
chrUn	38906958	38907728	771	SAT1_CF	blue	na
chrUn	38907748	38908240	493	SAT1_CF	blue	na
chrUn	38908327	38909586	1260	SAT1_CF	blue	na
chrUn	38909600	38910282	683	SAT1_CF	blue	na
chrUn	38910294	38910795	502	SAT1_CF	blue	na
chrUn	38910848	38911167	320	SAT1_CF	blue	na
chrUn	38911178	38912866	1689	SAT1_CF	blue	na
chrUn	38961295	38963335	2041	CarSat1	red	na
chrUn	38963326	38964372	1047	SAT1_CF	blue	na
chrUn	38964415	38964831	417	SAT1_CF	blue	na
chrUn	38967505	38967733	229	SAT1_CF	blue	na
chrUn	38967804	38970680	2877	SAT1_CF	blue	na
chrUn	38970704	38972097	1394	SAT1_CF	blue	na
chrUn	38972141	38972618	478	SAT1_CF	blue	na
chrUn	38972652	38974417	1766	SAT1_CF	blue	na
chrUn	38974429	38975107	679	SAT1_CF	blue	na
chrUn	38975150	38977772	2623	SAT1_CF	blue	na
chrUn	38977767	38978478	712	CarSat1	red	na
chrUn	38978509	38978932	424	CarSat1	red	na
chrUn	38985624	38986387	764	SAT1_CF	blue	na
chrUn	38986409	38987308	900	SAT1_CF	blue	na
chrUn	38987336	38989786	2451	SAT1_CF	blue	na
chrUn	38989803	38990318	516	SAT1_CF	blue	na
chrUn	38990997	38991637	641	SAT1_CF	blue	na
chrUn	38991677	38992177	501	SAT1_CF	blue	na
chrUn	38992190	38994215	2026	SAT1_CF	blue	na
chrUn	38994278	38994749	472	SAT1_CF	blue	na
chrUn	38994769	38995452	684	SAT1_CF	blue	na
chrUn	38995464	38996632	1169	SAT1_CF	blue	na
chrUn	38996647	38996804	158	SAT1_CF	blue	na
chrUn	38996849	38997351	503	SAT1_CF	blue	na
chrUn	38997364	38997533	170	SAT1_CF	blue	na
chrUn	38997560	38998746	1187	SAT1_CF	blue	na
chrUn	38998826	38999035	210	SAT1_CF	blue	na
chrUn	38999049	39001316	2268	SAT1_CF	blue	na
chrUn	39001359	39001883	525	SAT1_CF	blue	na
chrUn	39001962	39002921	960	SAT1_CF	blue	na
chrUn	39002961	39003455	495	SAT1_CF	blue	na
chrUn	39003475	39004156	682	SAT1_CF	blue	na

chrUn	39004168	39005029	862	SAT1_CF	blue	na
chrUn	39005071	39005852	782	SAT1_CF	blue	na
chrUn	39005931	39006479	549	SAT1_CF	blue	na
chrUn	39006512	39006891	380	SAT1_CF	blue	na
chrUn	39006931	39008126	1196	SAT1_CF	blue	na
chrUn	39008138	39008537	400	SAT1_CF	blue	na
chrUn	39008564	39009137	574	SAT1_CF	blue	na
chrUn	39009169	39009690	522	SAT1_CF	blue	na
chrUn	39009705	39010408	704	SAT1_CF	blue	na
chrUn	39010421	39010527	107	SAT1_CF	blue	na
chrUn	39010550	39012432	1883	SAT1_CF	blue	na
chrUn	39012476	39012740	265	SAT1_CF	blue	na
chrUn	39012806	39013331	526	SAT1_CF	blue	na
chrUn	39013346	39013503	158	SAT1_CF	blue	na
chrUn	39013548	39014049	502	SAT1_CF	blue	na
chrUn	39014077	39014744	668	SAT1_CF	blue	na
chrUn	39014756	39015615	860	SAT1_CF	blue	na
chrUn	39015692	39016349	658	SAT1_CF	blue	na
chrUn	39016363	39016908	546	SAT1_CF	blue	na
chrUn	39016951	39017579	629	SAT1_CF	blue	na
chrUn	39017591	39018921	1331	SAT1_CF	blue	na
chrUn	39019002	39019565	564	SAT1_CF	blue	na
chrUn	39019645	39020333	689	SAT1_CF	blue	na
chrUn	39024307	39025070	764	SAT1_CF	blue	na
chrUn	39025228	39026579	1352	SAT1_CF	blue	na
chrUn	39026596	39027116	521	SAT1_CF	blue	na
chrUn	39027129	39027810	682	SAT1_CF	blue	na
chrUn	39027834	39027931	98	SAT1_CF	blue	na
chrUn	39027952	39028683	732	SAT1_CF	blue	na
chrUn	39028725	39030528	1804	SAT1_CF	blue	na
chrUn	39030619	39031115	497	SAT1_CF	blue	na
chrUn	39031128	39032078	951	SAT1_CF	blue	na
chrUn	39032145	39032707	563	SAT1_CF	blue	na
chrUn	39032787	39033263	477	SAT1_CF	blue	na
chrUn	39033275	39034654	1380	SAT1_CF	blue	na
chrUn	39034666	39035287	622	SAT1_CF	blue	na
chrUn	39035366	39036868	1503	SAT1_CF	blue	na
chrUn	39036880	39038256	1377	SAT1_CF	blue	na
chrUn	39038268	39038889	622	SAT1_CF	blue	na
chrUn	39038968	39040470	1503	SAT1_CF	blue	na
chrUn	39040482	39041166	685	SAT1_CF	blue	na
chrUn	39041178	39041852	675	SAT1_CF	blue	na

chrUn	39041895	39043478	1584	SAT1_CF	blue	na
chrUn	39043492	39043893	402	SAT1_CF	blue	na
chrUn	39043915	39045072	1158	SAT1_CF	blue	na
chrUn	39045058	39045782	725	CarSat1	red	na
chrUn	39045814	39046240	427	CarSat1	red	na
chrUn	39047578	39049270	1693	SAT1_CF	blue	na
chrUn	39049282	39050519	1238	SAT1_CF	blue	na
chrUn	39050562	39051994	1433	SAT1_CF	blue	na
chrUn	39052028	39052502	475	SAT1_CF	blue	na
chrUn	39052562	39053520	959	SAT1_CF	blue	na
chrUn	39053564	39053901	338	SAT1_CF	blue	na
chrUn	39053949	39055967	2019	SAT1_CF	blue	na
chrUn	39055993	39056513	521	SAT1_CF	blue	na
chrUn	39056560	39056697	138	SAT1_CF	blue	na
chrUn	39056725	39057274	550	SAT1_CF	blue	na
chrUn	39057354	39057915	562	SAT1_CF	blue	na
chrUn	39057959	39058426	468	SAT1_CF	blue	na
chrUn	39058439	39058982	544	SAT1_CF	blue	na
chrUn	39059009	39059203	195	SAT1_CF	blue	na
chrUn	39059246	39059809	564	SAT1_CF	blue	na
chrUn	39059853	39061750	1898	SAT1_CF	blue	na
chrUn	39061772	39062338	567	SAT1_CF	blue	na
chrUn	39062370	39062946	577	SAT1_CF	blue	na
chrUn	39062957	39063621	665	SAT1_CF	blue	na
chrUn	39063643	39064145	503	SAT1_CF	blue	na
chrUn	39064211	39064347	137	SAT1_CF	blue	na
chrUn	39064375	39064886	512	SAT1_CF	blue	na
chrUn	39064929	39066004	1076	SAT1_CF	blue	na
chrUn	39066047	39067664	1618	SAT1_CF	blue	na
chrUn	39067691	39068192	502	SAT1_CF	blue	na
chrUn	39068232	39070087	1856	SAT1_CF	blue	na
chrUn	39070137	39070985	849	SAT1_CF	blue	na
chrUn	39070997	39072928	1932	SAT1_CF	blue	na
chrUn	39072940	39073542	603	SAT1_CF	blue	na
chrUn	39073553	39074233	681	SAT1_CF	blue	na
chrUn	39074245	39074927	683	SAT1_CF	blue	na
chrUn	39074947	39075481	535	SAT1_CF	blue	na
chrUn	39075492	39076253	762	SAT1_CF	blue	na
chrUn	39076264	39076817	554	SAT1_CF	blue	na
chrUn	39076843	39076946	104	SAT1_CF	blue	na
chrUn	39076969	39077439	471	SAT1_CF	blue	na
chrUn	39077513	39077684	172	SAT1_CF	blue	na

chrUn	39077861	39078653	793	SAT1_CF	blue	na
chrUn	39078697	39079022	326	SAT1_CF	blue	na
chrUn	39079046	39080645	1600	SAT1_CF	blue	na
chrUn	39080658	39081205	548	SAT1_CF	blue	na
chrUn	39081284	39082241	958	SAT1_CF	blue	na
chrUn	39082287	39083377	1091	SAT1_CF	blue	na
chrUn	39084306	39084624	319	SAT1_CF	blue	na
chrUn	39084636	39085313	678	SAT1_CF	blue	na
chrUn	39085338	39085832	495	SAT1_CF	blue	na
chrUn	39085870	39086436	567	SAT1_CF	blue	na
chrUn	39087727	39088280	554	SAT1_CF	blue	na
chrUn	39088292	39089344	1053	SAT1_CF	blue	na
chrUn	39089388	39090717	1330	SAT1_CF	blue	na
chrUn	39090729	39091334	606	SAT1_CF	blue	na
chrUn	39091354	39091848	495	SAT1_CF	blue	na
chrUn	39091888	39092461	574	SAT1_CF	blue	na
chrUn	39093888	39094253	366	SAT1_CF	blue	na
chrUn	39094296	39095411	1116	SAT1_CF	blue	na
chrUn	39095429	39095949	521	SAT1_CF	blue	na
chrUn	39095975	39096083	109	SAT1_CF	blue	na
chrUn	39096094	39097767	1674	SAT1_CF	blue	na
chrUn	39097791	39100498	2708	SAT1_CF	blue	na
chrUn	39100521	39101140	620	SAT1_CF	blue	na
chrUn	39101180	39102210	1031	SAT1_CF	blue	na
chrUn	39102256	39102875	620	SAT1_CF	blue	na
chrUn	39102890	39104131	1242	SAT1_CF	blue	na
chrUn	39104144	39105520	1377	SAT1_CF	blue	na
chrUn	39105532	39106611	1080	SAT1_CF	blue	na
chrUn	39106655	39107199	545	SAT1_CF	blue	na
chrUn	39107212	39108903	1692	SAT1_CF	blue	na
chrUn	39108916	39109416	501	SAT1_CF	blue	na
chrUn	39109452	39112142	2691	SAT1_CF	blue	na
chrUn	39112154	39112708	555	SAT1_CF	blue	na
chrUn	39112763	39113394	632	SAT1_CF	blue	na
chrUn	39113407	39114875	1469	SAT1_CF	blue	na
chrUn	39205013	39206583	1571	CarSat1	red	na
chrUn	39206598	39207303	706	CarSat1	red	na
chrUn	39207333	39211218	3886	CarSat1	red	na
chrUn	39211243	39213019	1777	CarSat1	red	na
chrUn	39216038	39217615	1578	CarSat1	red	na
chrUn	39217630	39218577	948	CarSat1	red	na
chrUn	39218601	39223258	4658	CarSat1	red	na

chrUn	39223269	39224475	1207	CarSat1	red	na
chrUn	39224499	39225463	965	CarSat1	red	na
chrUn	39225502	39226467	966	CarSat1	red	na
chrUn	39226482	39227388	907	CarSat1	red	na
chrUn	39227442	39229965	2524	CarSat1	red	na
chrUn	39229976	39231184	1209	CarSat1	red	na
chrUn	39231208	39232658	1451	CarSat1	red	na
chrUn	39232682	39233851	1170	CarSat1	red	na
chrUn	39257385	39257982	598	CarSat1	red	na
chrUn	39258006	39259606	1601	CarSat1	red	na
chrUn	39259914	39260676	763	CarSat1	red	na
chrUn	39261329	39261915	587	CarSat1	red	na
chrUn	39261942	39263395	1454	CarSat1	red	na
chrUn	39263422	39264635	1214	CarSat1	red	na
chrUn	39264659	39265626	968	CarSat1	red	na
chrUn	39265647	39267372	1726	CarSat1	red	na
chrUn	39267387	39269872	2486	CarSat1	red	na
chrUn	39270803	39271792	990	CarSat1	red	na
chrUn	39271807	39274944	3138	CarSat1	red	na
chrUn	39275602	39276193	592	CarSat1	red	na
chrUn	39276223	39276929	707	CarSat1	red	na
chrUn	39276959	39278402	1444	CarSat1	red	na
chrUn	39278432	39280110	1679	CarSat1	red	na
chrUn	39280134	39280839	706	CarSat1	red	na
chrUn	39280872	39288957	8086	CarSat1	red	na
chrUn	39288981	39291917	2937	CarSat1	red	na
chrUn	39291941	39292895	955	CarSat1	red	na
chrUn	39292922	39293632	711	CarSat1	red	na
chrUn	39293659	39294368	710	CarSat1	red	na
chrUn	39294395	39294864	470	CarSat1	red	na
chrUn	39294880	39295842	963	CarSat1	red	na
chrUn	39295869	39296579	711	CarSat1	red	na
chrUn	39296606	39297446	841	CarSat1	red	na
chrUn	39369772	39373236	3465	CarSat1	red	na
chrUn	39373269	39373770	502	CarSat1	red	na
chrUn	39373785	39374507	723	CarSat1	red	na
chrUn	39374522	39376721	2200	CarSat1	red	na
chrUn	39376736	39377680	945	CarSat1	red	na
chrUn	39378838	39380655	1818	CarSat1	red	na
chrUn	39380667	39381891	1225	CarSat1	red	na
chrUn	39381922	39383364	1443	CarSat1	red	na
chrUn	39383394	39385575	2182	CarSat1	red	na

chrUn	39385612	39388543	2932	CarSat1	red	na
chrUn	39388557	39389284	728	CarSat1	red	na
chrUn	39389299	39390482	1184	CarSat1	red	na
chrUn	39390503	39391497	995	CarSat1	red	na
chrUn	39391512	39391956	445	CarSat1	red	na
chrUn	39391977	39398015	6039	CarSat1	red	na
chrUn	39398035	39398332	298	SAT1_CF	blue	na
chrUn	39399010	39399939	930	SAT1_CF	blue	na
chrUn	39399951	39400617	667	SAT1_CF	blue	na
chrUn	39400659	39402622	1964	SAT1_CF	blue	na
chrUn	39402643	39403163	521	SAT1_CF	blue	na
chrUn	39403181	39403860	680	SAT1_CF	blue	na
chrUn	39403872	39404550	679	SAT1_CF	blue	na
chrUn	39404592	39406469	1878	SAT1_CF	blue	na
chrUn	39406497	39406655	159	SAT1_CF	blue	na
chrUn	39406699	39407193	495	SAT1_CF	blue	na
chrUn	39407215	39409191	1977	SAT1_CF	blue	na
chrUn	39409204	39410575	1372	SAT1_CF	blue	na
chrUn	39410617	39412675	2059	SAT1_CF	blue	na
chrUn	39412699	39413218	520	SAT1_CF	blue	na
chrUn	39413231	39413833	603	SAT1_CF	blue	na
chrUn	39413845	39416632	2788	SAT1_CF	blue	na
chrUn	39416651	39418151	1501	SAT1_CF	blue	na
chrUn	39418194	39418911	718	SAT1_CF	blue	na
chrUn	39418955	39419506	552	SAT1_CF	blue	na
chrUn	39419519	39420196	678	SAT1_CF	blue	na
chrUn	39420238	39421055	818	SAT1_CF	blue	na
chrUn	39421237	39421328	92	SAT1_CF	blue	na
chrUn	39421342	39423377	2036	SAT1_CF	blue	na
chrUn	39423389	39424061	673	SAT1_CF	blue	na
chrUn	39424131	39424606	476	SAT1_CF	blue	na
chrUn	39424618	39425295	678	SAT1_CF	blue	na
chrUn	39425338	39425881	544	SAT1_CF	blue	na
chrUn	39425925	39426387	463	SAT1_CF	blue	na
chrUn	39426879	39427633	755	SAT1_CF	blue	na
chrUn	39427823	39428203	381	SAT1_CF	blue	na
chrUn	39428219	39429000	782	SAT1_CF	blue	na
chrUn	39429068	39429549	482	SAT1_CF	blue	na
chrUn	39429561	39429674	114	SAT1_CF	blue	na
chrUn	39429691	39430401	711	SAT1_CF	blue	na
chrUn	39430413	39431089	677	SAT1_CF	blue	na
chrUn	39431133	39432096	964	SAT1_CF	blue	na

chrUn	39432175	39432721	547	SAT1_CF	blue	na
chrUn	39432734	39433134	401	SAT1_CF	blue	na
chrUn	39433174	39433308	135	SAT1_CF	blue	na
chrUn	39433493	39433892	400	SAT1_CF	blue	na
chrUn	39433910	39434408	499	SAT1_CF	blue	na
chrUn	39438056	39438483	428	SAT1_CF	blue	na
chrUn	39438530	39439245	716	SAT1_CF	blue	na
chrUn	39439324	39440106	783	SAT1_CF	blue	na
chrUn	39440148	39440319	172	SAT1_CF	blue	na
chrUn	39440332	39440879	548	SAT1_CF	blue	na
chrUn	39440908	39441008	101	SAT1_CF	blue	na
chrUn	39441022	39441628	607	SAT1_CF	blue	na
chrUn	39441640	39442326	687	SAT1_CF	blue	na
chrUn	39442337	39442838	502	SAT1_CF	blue	na
chrUn	39442881	39445454	2574	SAT1_CF	blue	na
chrUn	39445467	39445988	522	SAT1_CF	blue	na
chrUn	39446035	39446171	137	SAT1_CF	blue	na
chrUn	39446199	39448439	2241	SAT1_CF	blue	na
chrUn	39448451	39449004	554	SAT1_CF	blue	na
chrUn	39449069	39449138	70	SAT1_CF	blue	na
chrUn	39449153	39449667	515	SAT1_CF	blue	na
chrUn	39449693	39450708	1016	SAT1_CF	blue	na
chrUn	39450763	39451382	620	SAT1_CF	blue	na
chrUn	39451394	39452078	685	SAT1_CF	blue	na
chrUn	39452091	39452573	483	SAT1_CF	blue	na
chrUn	39452649	39453149	501	SAT1_CF	blue	na
chrUn	39453166	39454079	914	SAT1_CF	blue	na
chrUn	39496181	39496901	721	SAT1_CF	blue	na
chrUn	39496944	39500700	3757	SAT1_CF	blue	na
chrUn	39500744	39501274	531	SAT1_CF	blue	na
chrUn	39501316	39502274	959	SAT1_CF	blue	na
chrUn	39502296	39503453	1158	SAT1_CF	blue	na
chrUn	39503439	39504157	719	CarSat1	red	na
chrUn	39504315	39504543	229	CarSat1	red	na
chrUn	39505548	39506369	822	SAT1_CF	blue	na
chrUn	39506412	39508475	2064	SAT1_CF	blue	na
chrUn	39508487	39509850	1364	SAT1_CF	blue	na
chrUn	39509894	39510450	557	SAT1_CF	blue	na
chrUn	39510530	39511114	585	SAT1_CF	blue	na
chrUn	39511126	39512139	1014	SAT1_CF	blue	na
chrUn	39512404	39513054	651	SAT1_CF	blue	na
chrUn	39513079	39513402	324	SAT1_CF	blue	na

chrUn	39513512	39514054	543	SAT1_CF	blue	na
chrUn	39514075	39514470	396	SAT1_CF	blue	na
chrUn	39514510	39515997	1488	SAT1_CF	blue	na
chrUn	39516018	39516560	543	SAT1_CF	blue	na
chrUn	39516630	39517832	1203	SAT1_CF	blue	na
chrUn	39517876	39518182	307	SAT1_CF	blue	na
chrUn	39518263	39519115	853	SAT1_CF	blue	na
chrUn	39519145	39520103	959	SAT1_CF	blue	na
chrUn	39520153	39520852	700	SAT1_CF	blue	na
chrUn	39520903	39521963	1061	SAT1_CF	blue	na
chrUn	39522033	39522458	426	SAT1_CF	blue	na
chrUn	39522500	39524357	1858	SAT1_CF	blue	na
chrUn	39524395	39524895	501	SAT1_CF	blue	na
chrUn	39524922	39526131	1210	SAT1_CF	blue	na
chrUn	39526143	39526787	645	SAT1_CF	blue	na
chrUn	39526812	39527644	833	SAT1_CF	blue	na
chrUn	39527709	39528158	450	SAT1_CF	blue	na
chrUn	39528245	39528746	502	SAT1_CF	blue	na
chrUn	39528774	39530908	2135	SAT1_CF	blue	na
chrUn	39530966	39531598	633	SAT1_CF	blue	na
chrUn	39531610	39532506	897	SAT1_CF	blue	na
chrUn	39532566	39533493	928	SAT1_CF	blue	na
chrUn	39533510	39534496	987	SAT1_CF	blue	na
chrUn	39534508	39535185	678	SAT1_CF	blue	na
chrUn	39535228	39536190	963	SAT1_CF	blue	na
chrUn	39536257	39536843	587	SAT1_CF	blue	na
chrUn	39536857	39537401	545	SAT1_CF	blue	na
chrUn	39537413	39538059	647	SAT1_CF	blue	na
chrUn	39538139	39539437	1299	SAT1_CF	blue	na
chrUn	39539524	39540025	502	SAT1_CF	blue	na
chrUn	39540037	39540146	110	SAT1_CF	blue	na
chrUn	39540167	39540799	633	SAT1_CF	blue	na
chrUn	39540812	39541495	684	SAT1_CF	blue	na
chrUn	39541507	39542189	683	SAT1_CF	blue	na
chrUn	39542200	39542692	493	SAT1_CF	blue	na
chrUn	39542706	39543430	725	SAT1_CF	blue	na
chrUn	39543442	39543512	71	SAT1_CF	blue	na
chrUn	39543537	39543684	148	SAT1_CF	blue	na
chrUn	39543711	39544911	1201	SAT1_CF	blue	na
chrUn	39544963	39545099	137	SAT1_CF	blue	na
chrUn	39545165	39545752	588	SAT1_CF	blue	na
chrUn	39545808	39546322	515	SAT1_CF	blue	na

chrUn	39546335	39547424	1090	SAT1_CF	blue	na
chrUn	39547450	39548937	1488	SAT1_CF	blue	na
chrUn	39548949	39549567	619	SAT1_CF	blue	na
chrUn	39549646	39550889	1244	SAT1_CF	blue	na
chrUn	39717304	39718733	1430	SAT1_CF	blue	na
chrUn	39720066	39720639	574	SAT1_CF	blue	na
chrUn	39720674	39721181	508	SAT1_CF	blue	na
chrUn	39721194	39721869	676	SAT1_CF	blue	na
chrUn	39721912	39722877	966	SAT1_CF	blue	na
chrUn	39722920	39723954	1035	SAT1_CF	blue	na
chrUn	39723974	39725179	1206	SAT1_CF	blue	na
chrUn	39725208	39725729	522	SAT1_CF	blue	na
chrUn	39725742	39726612	871	SAT1_CF	blue	na
chrUn	39726634	39727178	545	SAT1_CF	blue	na
chrUn	39727220	39727759	540	SAT1_CF	blue	na
chrUn	39727793	39729075	1283	SAT1_CF	blue	na
chrUn	39729087	39729978	892	SAT1_CF	blue	na
chrUn	39729991	39731282	1292	SAT1_CF	blue	na
chrUn	39731324	39732284	961	SAT1_CF	blue	na
chrUn	39732325	39733159	835	SAT1_CF	blue	na
chrUn	39823387	39823968	582	Sat7CF	lime	na
chrUn	39862266	39862741	476	SAT1_CF	blue	na
chrUn	39862807	39862943	137	SAT1_CF	blue	na
chrUn	39862971	39863531	561	SAT1_CF	blue	na
chrUn	39863562	39864111	550	SAT1_CF	blue	na
chrUn	39864198	39864550	353	SAT1_CF	blue	na
chrUn	39864563	39865920	1358	SAT1_CF	blue	na
chrUn	39865932	39866621	690	SAT1_CF	blue	na
chrUn	39866633	39868010	1378	SAT1_CF	blue	na
chrUn	39868022	39869091	1070	SAT1_CF	blue	na
chrUn	39869103	39869946	844	SAT1_CF	blue	na
chrUn	39870831	39871821	991	SAT1_CF	blue	na
chrUn	39871848	39872480	633	SAT1_CF	blue	na
chrUn	39872523	39873197	675	SAT1_CF	blue	na
chrUn	39873210	39873765	556	SAT1_CF	blue	na
chrUn	39873788	39873893	106	SAT1_CF	blue	na
chrUn	39873906	39874423	518	SAT1_CF	blue	na
chrUn	39874442	39876479	2038	SAT1_CF	blue	na
chrUn	39876522	39877198	677	SAT1_CF	blue	na
chrUn	39877210	39877978	769	SAT1_CF	blue	na
chrUn	39877999	39878473	475	SAT1_CF	blue	na
chrUn	39878533	39878934	402	SAT1_CF	blue	na

chrUn	39878983	39880175	1193	SAT1_CF	blue	na
chrUn	39880202	39881358	1157	SAT1_CF	blue	na
chrUn	39881378	39881487	110	SAT1_CF	blue	na
chrUn	39881499	39884463	2965	SAT1_CF	blue	na
chrUn	39884474	39884986	513	SAT1_CF	blue	na
chrUn	39885007	39885580	574	SAT1_CF	blue	na
chrUn	39885604	39886299	696	SAT1_CF	blue	na
chrUn	39886316	39887154	839	SAT1_CF	blue	na
chrUn	39887189	39887669	481	SAT1_CF	blue	na
chrUn	39887695	39888145	451	SAT1_CF	blue	na
chrUn	39888162	39888761	600	SAT1_CF	blue	na
chrUn	39888779	39889260	482	SAT1_CF	blue	na
chrUn	39889332	39889843	512	SAT1_CF	blue	na
chrUn	39889876	39890422	547	SAT1_CF	blue	na
chrUn	39890483	39890580	98	SAT1_CF	blue	na
chrUn	39890592	39891134	543	SAT1_CF	blue	na
chrUn	39891176	39891886	711	SAT1_CF	blue	na
chrUn	39891916	39892262	347	SAT1_CF	blue	na
chrUn	39892277	39892819	543	SAT1_CF	blue	na
chrUn	39892860	39893719	860	SAT1_CF	blue	na
chrUn	39893734	39894442	709	SAT1_CF	blue	na
chrUn	39894463	39894572	110	SAT1_CF	blue	na
chrUn	39894584	39895105	522	SAT1_CF	blue	na
chrUn	39895129	39895286	158	SAT1_CF	blue	na
chrUn	39895314	39896318	1005	SAT1_CF	blue	na
chrUn	39896342	39896892	551	SAT1_CF	blue	na
chrUn	39896904	39897450	547	SAT1_CF	blue	na
chrUn	39897477	39898002	526	SAT1_CF	blue	na
chrUn	39898082	39899122	1041	SAT1_CF	blue	na
chrUn	39899136	39899543	408	SAT1_CF	blue	na
chrUn	39899584	39900444	861	SAT1_CF	blue	na
chrUn	39900458	39901139	682	SAT1_CF	blue	na
chrUn	39901151	39901834	684	SAT1_CF	blue	na
chrUn	39901847	39902550	704	SAT1_CF	blue	na
chrUn	39902565	39903543	979	SAT1_CF	blue	na
chrUn	39903579	39904428	850	SAT1_CF	blue	na
chrUn	39904471	39905708	1238	SAT1_CF	blue	na
chrUn	39905755	39907342	1588	SAT1_CF	blue	na
chrUn	39907385	39908059	675	SAT1_CF	blue	na
chrUn	39908071	39908830	760	SAT1_CF	blue	na
chrUn	39908843	39909396	554	SAT1_CF	blue	na
chrUn	39909419	39909525	107	SAT1_CF	blue	na

chrUn	39909538	39910038	501	SAT1_CF	blue	na
chrUn	39910125	39911180	1056	SAT1_CF	blue	na
chrUn	39911191	39911693	503	SAT1_CF	blue	na
chrUn	39911759	39911895	137	SAT1_CF	blue	na
chrUn	39911910	39912433	524	SAT1_CF	blue	na
chrUn	39912513	39914958	2446	SAT1_CF	blue	na
chrUn	39915001	39915550	550	SAT1_CF	blue	na
chrUn	39915564	39917095	1532	SAT1_CF	blue	na
chrUn	39917116	39917224	109	SAT1_CF	blue	na
chrUn	39917237	39917719	483	SAT1_CF	blue	na
chrUn	39917778	39918785	1008	SAT1_CF	blue	na
chrUn	39918817	39919817	1001	SAT1_CF	blue	na
chrUn	39919860	39920537	678	SAT1_CF	blue	na
chrUn	39920549	39921050	502	SAT1_CF	blue	na
chrUn	39921095	39921252	158	SAT1_CF	blue	na
chrUn	39921285	39921790	506	SAT1_CF	blue	na
chrUn	39921821	39923689	1869	SAT1_CF	blue	na
chrUn	39923732	39924406	675	SAT1_CF	blue	na
chrUn	39924419	39924680	262	SAT1_CF	blue	na
chrUn	39939138	39940148	1011	Sat2CF	purple	na
chrUn	39940027	39940208	182	Sat2CF	purple	na
chrUn	40144493	40144602	110	Sat16CF	na	na
chrUn	40155724	40156773	1050	Sat9CF	na	na
chrUn	40206710	40206976	267	SAT1_CF	blue	na
chrUn	40207018	40208874	1857	SAT1_CF	blue	na
chrUn	40208914	40209417	504	SAT1_CF	blue	na
chrUn	40209530	40211872	2343	SAT1_CF	blue	na
chrUn	40211992	40212518	527	SAT1_CF	blue	na
chrUn	40212597	40213119	523	SAT1_CF	blue	na
chrUn	40213134	40214599	1466	SAT1_CF	blue	na
chrUn	40214642	40215274	633	SAT1_CF	blue	na
chrUn	40215302	40215826	525	SAT1_CF	blue	na
chrUn	40216070	40216118	49	SAT1_CF	blue	na
chrUn	40216137	40216656	520	SAT1_CF	blue	na
chrUn	40216670	40217352	683	SAT1_CF	blue	na
chrUn	40217364	40219892	2529	SAT1_CF	blue	na
chrUn	40219964	40220456	493	SAT1_CF	blue	na
chrUn	40220652	40221599	948	SAT1_CF	blue	na
chrUn	40221611	40222286	676	SAT1_CF	blue	na
chrUn	40222329	40223316	988	SAT1_CF	blue	na
chrUn	40223373	40224328	956	SAT1_CF	blue	na
chrUn	40224368	40224868	501	SAT1_CF	blue	na

chrUn	40224895	40225641	747	SAT1_CF	blue	na
chrUn	40225653	40226323	671	SAT1_CF	blue	na
chrUn	40226335	40226508	174	SAT1_CF	blue	na
chrUn	40226550	40228839	2290	SAT1_CF	blue	na
chrUn	40228852	40228958	107	SAT1_CF	blue	na
chrUn	40228981	40230357	1377	SAT1_CF	blue	na
chrUn	40230397	40232037	1641	SAT1_CF	blue	na
chrUn	40232086	40232215	130	SAT1_CF	blue	na
chrUn	40232259	40232803	545	SAT1_CF	blue	na
chrUn	40232852	40232987	136	SAT1_CF	blue	na
chrUn	40233048	40233573	526	SAT1_CF	blue	na
chrUn	40233588	40233724	137	SAT1_CF	blue	na
chrUn	40233788	40236323	2536	SAT1_CF	blue	na
chrUn	40236370	40237077	708	SAT1_CF	blue	na
chrUn	40237095	40237852	758	SAT1_CF	blue	na
chrUn	40237864	40238694	831	SAT1_CF	blue	na
chrUn	40238710	40239767	1058	SAT1_CF	blue	na
chrUn	40239847	40240842	996	SAT1_CF	blue	na
chrUn	40240854	40241527	674	SAT1_CF	blue	na
chrUn	40241570	40242479	910	SAT1_CF	blue	na
chrUn	40242536	40243090	555	SAT1_CF	blue	na
chrUn	40243102	40243990	889	SAT1_CF	blue	na
chrUn	40244003	40246016	2014	SAT1_CF	blue	na
chrUn	40246095	40246631	537	SAT1_CF	blue	na
chrUn	40246685	40247355	671	SAT1_CF	blue	na
chrUn	40248750	40249499	750	SAT1_CF	blue	na
chrUn	40249522	40250815	1294	SAT1_CF	blue	na
chrUn	40250848	40251197	350	SAT1_CF	blue	na
chrUn	40251253	40252450	1198	SAT1_CF	blue	na
chrUn	40252462	40253130	669	SAT1_CF	blue	na
chrUn	40253173	40255133	1961	SAT1_CF	blue	na
chrUn	40255178	40255679	502	SAT1_CF	blue	na
chrUn	40255691	40256346	656	SAT1_CF	blue	na
chrUn	40256403	40256949	547	SAT1_CF	blue	na
chrUn	40256992	40258361	1370	SAT1_CF	blue	na
chrUn	40258373	40258481	109	SAT1_CF	blue	na
chrUn	40258504	40259059	556	SAT1_CF	blue	na
chrUn	40259071	40259180	110	SAT1_CF	blue	na
chrUn	40259200	40259747	548	SAT1_CF	blue	na
chrUn	40259790	40261230	1441	SAT1_CF	blue	na
chrUn	40261286	40261787	502	SAT1_CF	blue	na
chrUn	40261799	40262481	683	SAT1_CF	blue	na

chrUn	40262493	40263160	668	SAT1_CF	blue	na
chrUn	40263209	40264513	1305	SAT1_CF	blue	na
chrUn	40264569	40265778	1210	SAT1_CF	blue	na
chrUn	40265843	40266542	700	SAT1_CF	blue	na
chrUn	40266555	40267668	1114	SAT1_CF	blue	na
chrUn	40342487	40343155	669	SAT1_CF	blue	na
chrUn	40343183	40343749	567	SAT1_CF	blue	na
chrUn	40346418	40346648	231	SAT1_CF	blue	na
chrUn	40346717	40349525	2809	SAT1_CF	blue	na
chrUn	40349553	40352700	3148	SAT1_CF	blue	na
chrUn	40352729	40355897	3169	SAT1_CF	blue	na
chrUn	40355937	40356997	1061	SAT1_CF	blue	na
chrUn	40421904	40422606	703	SAT1_CF	blue	na
chrUn	40422648	40424308	1661	SAT1_CF	blue	na
chrUn	40424320	40426673	2354	SAT1_CF	blue	na
chrUn	40426701	40427265	565	SAT1_CF	blue	na
chrUn	40427345	40427855	511	SAT1_CF	blue	na
chrUn	40427883	40428040	158	SAT1_CF	blue	na
chrUn	40428085	40428587	503	SAT1_CF	blue	na
chrUn	40428603	40429360	758	SAT1_CF	blue	na
chrUn	40429372	40430628	1257	SAT1_CF	blue	na
chrUn	40430645	40431602	958	SAT1_CF	blue	na
chrUn	40431622	40432488	867	SAT1_CF	blue	na
chrUn	40432508	40433178	671	SAT1_CF	blue	na
chrUn	40433189	40434754	1566	SAT1_CF	blue	na
chrUn	40434794	40435295	502	SAT1_CF	blue	na
chrUn	40435322	40436754	1433	SAT1_CF	blue	na
chrUn	40436802	40438066	1265	SAT1_CF	blue	na
chrUn	40438109	40438653	545	SAT1_CF	blue	na
chrUn	40438737	40439283	547	SAT1_CF	blue	na
chrUn	40439311	40440014	704	SAT1_CF	blue	na
chrUn	40440040	40440710	671	SAT1_CF	blue	na
chrUn	40440722	40441396	675	SAT1_CF	blue	na
chrUn	40441445	40441561	117	SAT1_CF	blue	na
chrUn	40441603	40442641	1039	SAT1_CF	blue	na
chrUn	40442698	40443330	633	SAT1_CF	blue	na
chrUn	40443342	40443945	604	SAT1_CF	blue	na
chrUn	40443958	40444064	107	SAT1_CF	blue	na
chrUn	40444085	40444832	748	SAT1_CF	blue	na
chrUn	40444847	40445314	468	SAT1_CF	blue	na
chrUn	40445392	40445937	546	SAT1_CF	blue	na
chrUn	40445975	40446317	343	SAT1_CF	blue	na

chrUn	40446389	40446889	501	SAT1_CF	blue	na
chrUn	40446903	40447663	761	SAT1_CF	blue	na
chrUn	40447675	40448393	719	SAT1_CF	blue	na
chrUn	40448421	40448794	374	SAT1_CF	blue	na
chrUn	40448806	40448915	110	SAT1_CF	blue	na
chrUn	40448936	40449483	548	SAT1_CF	blue	na
chrUn	40449526	40450358	833	SAT1_CF	blue	na
chrUn	40450549	40451556	1008	SAT1_CF	blue	na
chrUn	40451580	40452316	737	SAT1_CF	blue	na
chrUn	40452329	40453168	840	SAT1_CF	blue	na
chrUn	40453180	40454451	1272	SAT1_CF	blue	na
chrUn	40454493	40456158	1666	SAT1_CF	blue	na
chrUn	40456186	40456321	136	SAT1_CF	blue	na
chrUn	40456387	40456888	502	SAT1_CF	blue	na
chrUn	40456900	40457006	107	SAT1_CF	blue	na
chrUn	40457030	40457658	629	SAT1_CF	blue	na
chrUn	40457710	40458535	826	SAT1_CF	blue	na
chrUn	40458553	40459232	680	SAT1_CF	blue	na
chrUn	40459244	40460572	1329	SAT1_CF	blue	na
chrUn	40460597	40460922	326	SAT1_CF	blue	na
chrUn	40460989	40461511	523	SAT1_CF	blue	na
chrUn	40461524	40461886	363	SAT1_CF	blue	na
chrUn	40461946	40462420	475	SAT1_CF	blue	na
chrUn	40462440	40463123	684	SAT1_CF	blue	na
chrUn	40463135	40463809	675	SAT1_CF	blue	na
chrUn	40463857	40464355	499	SAT1_CF	blue	na
chrUn	40464444	40464944	501	SAT1_CF	blue	na
chrUn	40464957	40465064	108	SAT1_CF	blue	na
chrUn	40465086	40465639	554	SAT1_CF	blue	na
chrUn	40465652	40468533	2882	SAT1_CF	blue	na
chrUn	40468548	40468705	158	SAT1_CF	blue	na
chrUn	40468750	40469832	1083	SAT1_CF	blue	na
chrUn	40469843	40470445	603	SAT1_CF	blue	na
chrUn	40470457	40471243	787	SAT1_CF	blue	na
chrUn	40471288	40471789	502	SAT1_CF	blue	na
chrUn	40471803	40472557	755	SAT1_CF	blue	na
chrUn	40472569	40472678	110	SAT1_CF	blue	na
chrUn	40472699	40473243	545	SAT1_CF	blue	na
chrUn	40473292	40473895	604	SAT1_CF	blue	na
chrUn	40473925	40475612	1688	SAT1_CF	blue	na
chrUn	40475625	40475732	108	SAT1_CF	blue	na
chrUn	40475754	40476384	631	SAT1_CF	blue	na

chrUn	40476398	40478993	2596	SAT1_CF	blue	na
chrUn	40479175	40479356	182	SAT1_CF	blue	na
chrUn	40479396	40479925	530	SAT1_CF	blue	na
chrUn	40479938	40480043	106	SAT1_CF	blue	na
chrUn	40480066	40481335	1270	SAT1_CF	blue	na
chrUn	40481350	40481485	136	SAT1_CF	blue	na
chrUn	40481530	40482237	708	SAT1_CF	blue	na
chrUn	40626906	40627605	700	SAT1_CF	blue	na
chrUn	40627616	40628429	814	SAT1_CF	blue	na
chrUn	40628509	40629731	1223	SAT1_CF	blue	na
chrUn	40629751	40630354	604	SAT1_CF	blue	na
chrUn	40630366	40632671	2306	SAT1_CF	blue	na
chrUn	40632684	40632841	158	SAT1_CF	blue	na
chrUn	40632908	40633409	502	SAT1_CF	blue	na
chrUn	40633422	40634027	606	SAT1_CF	blue	na
chrUn	40634040	40634898	859	SAT1_CF	blue	na
chrUn	40634911	40636280	1370	SAT1_CF	blue	na
chrUn	40636323	40637275	953	SAT1_CF	blue	na
chrUn	40637354	40638314	961	SAT1_CF	blue	na
chrUn	40638354	40638856	503	SAT1_CF	blue	na
chrUn	40638868	40639553	686	SAT1_CF	blue	na
chrUn	40639565	40642006	2442	SAT1_CF	blue	na
chrUn	40642018	40643460	1443	SAT1_CF	blue	na
chrUn	40643476	40645117	1642	SAT1_CF	blue	na
chrUn	40645129	40646540	1412	SAT1_CF	blue	na
chrUn	40646553	40647055	503	SAT1_CF	blue	na
chrUn	40647068	40647751	684	SAT1_CF	blue	na
chrUn	40647763	40648440	678	SAT1_CF	blue	na
chrUn	40648483	40649444	962	SAT1_CF	blue	na
chrUn	40649511	40650031	521	SAT1_CF	blue	na
chrUn	40650044	40650231	188	SAT1_CF	blue	na
chrUn	40650283	40651042	760	SAT1_CF	blue	na
chrUn	40651120	40652066	947	SAT1_CF	blue	na
chrUn	40652119	40652695	577	SAT1_CF	blue	na
chrUn	40652717	40652815	99	SAT1_CF	blue	na
chrUn	40652836	40653391	556	SAT1_CF	blue	na
chrUn	40653403	40654265	863	SAT1_CF	blue	na
chrUn	40654306	40654937	632	SAT1_CF	blue	na
chrUn	40654965	40655930	966	SAT1_CF	blue	na
chrUn	40655944	40656344	401	SAT1_CF	blue	na
chrUn	40656384	40656882	499	SAT1_CF	blue	na
chrUn	40656895	40657573	679	SAT1_CF	blue	na

chrUn	40657619	40657944	326	SAT1_CF	blue	na
chrUn	40657986	40658492	507	SAT1_CF	blue	na
chrUn	40658510	40658613	104	SAT1_CF	blue	na
chrUn	40658634	40659208	575	SAT1_CF	blue	na
chrUn	40659220	40661347	2128	SAT1_CF	blue	na
chrUn	40661428	40661591	164	SAT1_CF	blue	na
chrUn	40661772	40661957	186	SAT1_CF	blue	na
chrUn	40661994	40662493	500	SAT1_CF	blue	na
chrUn	40662506	40662898	393	SAT1_CF	blue	na
chrUn	40662925	40663527	603	SAT1_CF	blue	na
chrUn	40663539	40664212	674	SAT1_CF	blue	na
chrUn	40664224	40664395	172	SAT1_CF	blue	na
chrUn	40664437	40664980	544	SAT1_CF	blue	na
chrUn	40664996	40666120	1125	SAT1_CF	blue	na
chrUn	40666132	40667023	892	SAT1_CF	blue	na
chrUn	40667036	40668405	1370	SAT1_CF	blue	na
chrUn	40668446	40670476	2031	SAT1_CF	blue	na
chrUn	40670519	40671018	500	SAT1_CF	blue	na
chrUn	40671031	40673527	2497	SAT1_CF	blue	na
chrUn	40673540	40674161	622	SAT1_CF	blue	na
chrUn	40674182	40674856	675	SAT1_CF	blue	na
chrUn	40674879	40675422	544	SAT1_CF	blue	na
chrUn	40675465	40676313	849	SAT1_CF	blue	na
chrUn	40676350	40676841	492	SAT1_CF	blue	na
chrUn	40676852	40677657	806	SAT1_CF	blue	na
chrUn	40677680	40679449	1770	SAT1_CF	blue	na
chrUn	40679489	40679981	493	SAT1_CF	blue	na
chrUn	40680001	40682438	2438	SAT1_CF	blue	na
chrUn	40682476	40683495	1020	SAT1_CF	blue	na
chrUn	40683506	40683998	493	SAT1_CF	blue	na
chrUn	40684012	40685643	1632	SAT1_CF	blue	na
chrUn	40685657	40686205	549	SAT1_CF	blue	na
chrUn	40688925	40690177	1253	Sat2CF	purple	na
chrUn	40690017	40690205	189	Sat2CF	purple	na
chrUn	40692807	40694399	1593	CarSat1	red	na
chrUn	40694420	40695905	1486	CarSat1	red	na
chrUn	40705418	40705824	407	CarSat1	red	na
chrUn	40705858	40707639	1782	CarSat1	red	na
chrUn	40707658	40708059	402	CarSat1	red	na
chrUn	40708075	40709462	1388	SAT1_CF	blue	na
chrUn	40709657	40709817	161	Sat2CF	purple	na
chrUn	40709690	40710638	949	Sat2CF	purple	na

chrUn	40716470	40718009	1540	CarSat1	red	na
chrUn	40718217	40718808	592	CarSat1	red	na
chrUn	40727530	40728247	718	CarSat1	red	na
chrUn	40728439	40728779	341	CarSat1	red	na
chrUn	40728791	40728920	130	Carsat2	orange	na
chrUn	40728809	40730250	1442	CarSat1	red	na
chrUn	40730280	40731591	1312	CarSat1	red	na
chrUn	40738131	40739010	880	CarSat1	red	na
chrUn	40739040	40740754	1715	CarSat1	red	na
chrUn	40740775	40742227	1453	CarSat1	red	na
chrUn	40742248	40742963	716	CarSat1	red	na
chrUn	40742984	40743943	960	CarSat1	red	na
chrUn	40743968	40744437	470	CarSat1	red	na
chrUn	40744464	40746649	2186	CarSat1	red	na
chrUn	40746676	40747555	880	CarSat1	red	na
chrUn	40838845	40839949	1105	Sat2CF	purple	na
chrUn	40849275	40850122	848	Sat2CF	purple	na
chrUn	40864613	40864988	376	CarSat1	red	na
chrUn	40865009	40865855	847	CarSat1	red	na
chrUn	40865872	40867466	1595	CarSat1	red	na
chrUn	40867487	40870201	2715	CarSat1	red	na
chrUn	40870213	40870481	269	SAT1_CF	blue	na
chrUn	40870501	40872458	1958	SAT1_CF	blue	na
chrUn	40872500	40873048	549	SAT1_CF	blue	na
chrUn	40873095	40873495	401	SAT1_CF	blue	na
chrUn	40873516	40874728	1213	SAT1_CF	blue	na
chrUn	40874772	40876805	2034	SAT1_CF	blue	na
chrUn	40876838	40877874	1037	SAT1_CF	blue	na
chrUn	40877869	40878587	719	CarSat1	red	na
chrUn	40878619	40879044	426	CarSat1	red	na
chrUn	40950173	40951513	1341	SAT1_CF	blue	na
chrUn	40951556	40952750	1195	SAT1_CF	blue	na
chrUn	40952762	40953582	821	SAT1_CF	blue	na
chrUn	40953624	40954164	541	SAT1_CF	blue	na
chrUn	40954181	40954712	532	SAT1_CF	blue	na
chrUn	40954724	40955398	675	SAT1_CF	blue	na
chrUn	40955441	40957055	1615	SAT1_CF	blue	na
chrUn	40957135	40957716	582	SAT1_CF	blue	na
chrUn	40957728	40958404	677	SAT1_CF	blue	na
chrUn	40958417	40958584	168	SAT1_CF	blue	na
chrUn	40958629	40959173	545	SAT1_CF	blue	na
chrUn	40959189	40959350	162	SAT1_CF	blue	na

chrUn	40959401	40959948	548	SAT1_CF	blue	na
chrUn	40960065	40960575	511	SAT1_CF	blue	na
chrUn	40960651	40961222	572	SAT1_CF	blue	na
chrUn	40961235	40963260	2026	SAT1_CF	blue	na
chrUn	40963340	40965146	1807	SAT1_CF	blue	na
chrUn	40965173	40965829	657	SAT1_CF	blue	na
chrUn	40965875	40966524	650	SAT1_CF	blue	na
chrUn	40966544	40967027	484	SAT1_CF	blue	na
chrUn	40967039	40967724	686	SAT1_CF	blue	na
chrUn	40967742	40968412	671	SAT1_CF	blue	na
chrUn	40968455	40969567	1113	SAT1_CF	blue	na
chrUn	40969610	40970194	585	SAT1_CF	blue	na
chrUn	40970206	40971803	1598	SAT1_CF	blue	na
chrUn	40971826	40972676	851	SAT1_CF	blue	na
chrUn	40972753	40973489	737	SAT1_CF	blue	na
chrUn	40973630	40974567	938	SAT1_CF	blue	na
chrUn	40974605	40977292	2688	SAT1_CF	blue	na
chrUn	40977362	40977914	553	SAT1_CF	blue	na
chrUn	40977926	40978639	714	SAT1_CF	blue	na
chrUn	40978671	40979583	913	SAT1_CF	blue	na
chrUn	40979601	40980533	933	SAT1_CF	blue	na
chrUn	40980576	40981078	503	SAT1_CF	blue	na
chrUn	40981090	40981199	110	SAT1_CF	blue	na
chrUn	40981220	40981775	556	SAT1_CF	blue	na
chrUn	40981818	40983189	1372	SAT1_CF	blue	na
chrUn	40983268	40984226	959	SAT1_CF	blue	na
chrUn	40984266	40984768	503	SAT1_CF	blue	na
chrUn	40984781	40985462	682	SAT1_CF	blue	na
chrUn	40985480	40986069	590	SAT1_CF	blue	na
chrUn	40986081	40986164	84	SAT1_CF	blue	na
chrUn	40986177	40987894	1718	SAT1_CF	blue	na
chrUn	40987906	40988015	110	SAT1_CF	blue	na
chrUn	40988035	40991071	3037	SAT1_CF	blue	na
chrUn	40991112	40991484	373	SAT1_CF	blue	na
chrUn	40991501	40992021	521	SAT1_CF	blue	na
chrUn	40992033	40992149	117	SAT1_CF	blue	na
chrUn	40992163	40992688	526	SAT1_CF	blue	na
chrUn	40992745	40993291	547	SAT1_CF	blue	na
chrUn	40993304	40993474	171	SAT1_CF	blue	na
chrUn	40993515	40995128	1614	SAT1_CF	blue	na
chrUn	40995214	40995696	483	SAT1_CF	blue	na
chrUn	40995709	40996315	607	SAT1_CF	blue	na

chrUn	40996327	40997002	676	SAT1_CF	blue	na
chrUn	40997045	40997676	632	SAT1_CF	blue	na
chrUn	40997704	40998791	1088	SAT1_CF	blue	na
chrUn	40998835	40999383	549	SAT1_CF	blue	na
chrUn	40999425	40999968	544	SAT1_CF	blue	na
chrUn	41000011	41000520	510	SAT1_CF	blue	na
chrUn	41000547	41000681	135	SAT1_CF	blue	na
chrUn	41000739	41001241	503	SAT1_CF	blue	na
chrUn	41001253	41002504	1252	SAT1_CF	blue	na
chrUn	41002594	41005714	3121	SAT1_CF	blue	na
chrUn	41005727	41006949	1223	SAT1_CF	blue	na
chrUn	41006962	41008291	1330	SAT1_CF	blue	na
chrUn	41037643	41038023	381	Sat7CF	lime	na
chrUn	41094311	41096260	1950	CarSat1	red	na
chrUn	41096275	41096986	712	CarSat1	red	na
chrUn	41097016	41099931	2916	CarSat1	red	na
chrUn	41099961	41100669	709	CarSat1	red	na
chrUn	41100699	41101421	723	CarSat1	red	na
chrUn	41101436	41102157	722	CarSat1	red	na
chrUn	41102172	41103846	1675	CarSat1	red	na
chrUn	41103870	41105092	1223	CarSat1	red	na
chrUn	41105122	41106797	1676	CarSat1	red	na
chrUn	41106821	41109519	2699	CarSat1	red	na
chrUn	41109549	41110993	1445	CarSat1	red	na
chrUn	41111023	41111735	713	CarSat1	red	na
chrUn	41111765	41112432	668	CarSat1	red	na
chrUn	41112621	41112682	62	CarSat1	red	na
chrUn	41112712	41114949	2238	CarSat1	red	na
chrUn	41114964	41116143	1180	CarSat1	red	na
chrUn	41116170	41116881	712	CarSat1	red	na
chrUn	41116908	41118625	1718	CarSat1	red	na
chrUn	41118655	41119363	709	CarSat1	red	na
chrUn	41119393	41120105	713	CarSat1	red	na
chrUn	41120135	41123047	2913	CarSat1	red	na
chrUn	41123086	41125256	2171	CarSat1	red	na
chrUn	41125286	41127228	1943	CarSat1	red	na
chrUn	41127454	41130883	3430	CarSat1	red	na
chrUn	41130907	41132132	1226	CarSat1	red	na
chrUn	41132162	41134352	2191	CarSat1	red	na
chrUn	41134381	41135837	1457	CarSat1	red	na
chrUn	41135852	41138045	2194	CarSat1	red	na
chrUn	41138060	41138772	713	CarSat1	red	na

chrUn	41138802	41139468	667	CarSat1	red	na
chrUn	41139657	41139718	62	CarSat1	red	na
chrUn	41139748	41141272	1525	CarSat1	red	na
chrUn	41141961	41143957	1997	CarSat1	red	na
chrUn	41144311	41145614	1304	CarSat1	red	na
chrUn	41145644	41147324	1681	CarSat1	red	na
chrUn	41147348	41150267	2920	CarSat1	red	na
chrUn	41150291	41152249	1959	CarSat1	red	na
chrUn	41152279	41152960	682	CarSat1	red	na
chrUn	41153996	41155078	1083	CarSat1	red	na
chrUn	41155102	41158663	3562	CarSat1	red	na
chrUn	41159594	41162122	2529	CarSat1	red	na
chrUn	41162159	41163378	1220	CarSat1	red	na
chrUn	41163402	41164114	713	CarSat1	red	na
chrUn	41164138	41164850	713	CarSat1	red	na
chrUn	41164874	41166078	1205	CarSat1	red	na
chrUn	41166105	41167794	1690	CarSat1	red	na
chrUn	41167818	41171015	3198	CarSat1	red	na
chrUn	41179679	41181412	1734	CarSat1	red	na
chrUn	41181441	41186350	4910	CarSat1	red	na
chrUn	41186374	41188043	1670	CarSat1	red	na
chrUn	41188073	41189523	1451	CarSat1	red	na
chrUn	41189553	41192249	2697	CarSat1	red	na
chrUn	41192273	41193207	935	CarSat1	red	na
chrUn	41193237	41195196	1960	CarSat1	red	na
chrUn	41195220	41196894	1675	CarSat1	red	na
chrUn	41196924	41197410	487	CarSat1	red	na
chrUn	41197434	41198146	713	CarSat1	red	na
chrUn	41198170	41198884	715	CarSat1	red	na
chrUn	41198908	41199620	713	CarSat1	red	na
chrUn	41199644	41200584	941	CarSat1	red	na
chrUn	41200614	41204180	3567	CarSat1	red	na
chrUn	41204495	41207450	2956	CarSat1	red	na
chrUn	41207474	41208402	929	CarSat1	red	na
chrUn	41208439	41210406	1968	CarSat1	red	na
chrUn	41210430	41211146	717	CarSat1	red	na
chrUn	41211170	41212621	1452	CarSat1	red	na
chrUn	41212643	41213357	715	CarSat1	red	na
chrUn	41213381	41215053	1673	CarSat1	red	na
chrUn	41215068	41215195	128	Carsat2	orange	na
chrUn	41215083	41215791	709	CarSat1	red	na
chrUn	41215806	41215932	127	Carsat2	orange	na

chrUn	41215821	41217043	1223	CarSat1	red	na
chrUn	41217067	41217784	718	CarSat1	red	na
chrUn	41217800	41218733	934	CarSat1	red	na
chrUn	41218764	41219356	593	CarSat1	red	na
chrUn	41277888	41279285	1398	CarSat1	red	na
chrUn	41279316	41280903	1588	CarSat1	red	na
chrUn	41280934	41282522	1589	CarSat1	red	na
chrUn	41282553	41284143	1591	CarSat1	red	na
chrUn	41284174	41285764	1591	CarSat1	red	na
chrUn	41285795	41287380	1586	CarSat1	red	na
chrUn	41287672	41289023	1352	CarSat1	red	na
chrUn	41289054	41290646	1593	CarSat1	red	na
chrUn	41290677	41292265	1589	CarSat1	red	na
chrUn	41292296	41293882	1587	CarSat1	red	na
chrUn	41293913	41295501	1589	CarSat1	red	na
chrUn	41295532	41297117	1586	CarSat1	red	na
chrUn	41297148	41298733	1586	CarSat1	red	na
chrUn	41298764	41300351	1588	CarSat1	red	na
chrUn	41300382	41301966	1585	CarSat1	red	na
chrUn	41317369	41318418	1050	CarSat1	red	na
chrUn	41318439	41320041	1603	CarSat1	red	na
chrUn	41320072	41321664	1593	CarSat1	red	na
chrUn	41321685	41323287	1603	CarSat1	red	na
chrUn	41323318	41324570	1253	CarSat1	red	na
chrUn	41324602	41327265	2664	CarSat1	red	na
chrUn	41327286	41327857	572	CarSat1	red	na
chrUn	41328058	41328811	754	CarSat1	red	na
chrUn	41328842	41330433	1592	CarSat1	red	na
chrUn	41330464	41332052	1589	CarSat1	red	na
chrUn	41332083	41333673	1591	CarSat1	red	na
chrUn	41333704	41335292	1589	CarSat1	red	na
chrUn	41335323	41336849	1527	CarSat1	red	na
chrUn	41430934	41432473	1540	CarSat1	red	na
chrUn	41432686	41433278	593	CarSat1	red	na
chrUn	41441795	41442494	700	CarSat1	red	na
chrUn	41442688	41443299	612	CarSat1	red	na
chrUn	41443316	41447225	3910	CarSat1	red	na
chrUn	41447251	41448700	1450	CarSat1	red	na
chrUn	41448716	41449922	1207	CarSat1	red	na
chrUn	41449949	41452871	2923	CarSat1	red	na
chrUn	41452898	41453608	711	CarSat1	red	na
chrUn	41453635	41454598	964	CarSat1	red	na

chrUn	41454614	41455083	470	CarSat1	red	na
chrUn	41455110	41455820	711	CarSat1	red	na
chrUn	41455847	41456558	712	CarSat1	red	na
chrUn	41456585	41458771	2187	CarSat1	red	na
chrUn	41458798	41459776	979	CarSat1	red	na
chrUn	41459801	41461245	1445	CarSat1	red	na
chrUn	41461265	41461717	453	CarSat1	red	na
chrUn	41461750	41461947	198	Carsat2	orange	na
chrUn	41461779	41462753	975	CarSat1	red	na
chrUn	41462777	41463489	713	CarSat1	red	na
chrUn	41463513	41464233	721	CarSat1	red	na
chrUn	41464259	41466434	2176	CarSat1	red	na
chrUn	41466458	41467178	721	CarSat1	red	na
chrUn	41467204	41467907	704	CarSat1	red	na
chrUn	41467931	41469602	1672	CarSat1	red	na
chrUn	41469632	41470339	708	CarSat1	red	na
chrUn	41470369	41471588	1220	CarSat1	red	na
chrUn	41471621	41472324	704	CarSat1	red	na
chrUn	41472348	41473068	721	CarSat1	red	na
chrUn	41473094	41473797	704	CarSat1	red	na
chrUn	41473821	41475492	1672	CarSat1	red	na
chrUn	41475522	41476229	708	CarSat1	red	na
chrUn	41476259	41479171	2913	CarSat1	red	na
chrUn	41479201	41479905	705	CarSat1	red	na
chrUn	41479920	41480046	127	Carsat2	orange	na
chrUn	41479935	41481160	1226	CarSat1	red	na
chrUn	41481192	41482102	911	CarSat1	red	na
chrUn	41482132	41483247	1116	CarSat1	red	na
chrUn	41539618	41541233	1616	CarSat1	red	na
chrUn	41542460	41543076	617	CarSat1	red	na
chrUn	41543123	41543806	684	CarSat1	red	na
chrUn	41543889	41544403	515	CarSat1	red	na
chrUn	41545546	41546843	1298	CarSat1	red	na
chrUn	41546875	41547777	903	CarSat1	red	na
chrUn	41549202	41551610	2409	CarSat1	red	na
chrUn	41553298	41553730	433	CarSat1	red	na
chrUn	41553764	41555932	2169	CarSat1	red	na
chrUn	41556544	41557394	851	CarSat1	red	na
chrUn	41557424	41558914	1491	CarSat1	red	na
chrUn	41558925	41565836	6912	CarSat1	red	na
chrUn	41568221	41571696	3476	CarSat1	red	na
chrUn	41571760	41572719	960	CarSat1	red	na

chrUn	41572735	41573619	885	CarSat1	red	na
chrUn	41575178	41576127	950	CarSat1	red	na
chrUn	41576154	41576708	555	CarSat1	red	na
chrUn	41577599	41579833	2235	CarSat1	red	na
chrUn	41580586	41581913	1328	CarSat1	red	na
chrUn	41581929	41582650	722	CarSat1	red	na
chrUn	41582675	41583134	460	CarSat1	red	na
chrUn	41583161	41585088	1928	CarSat1	red	na
chrUn	41585114	41585175	62	CarSat1	red	na
chrUn	41585364	41586760	1397	CarSat1	red	na
chrUn	41586786	41587495	710	CarSat1	red	na
chrUn	41587521	41587582	62	CarSat1	red	na
chrUn	41587771	41589052	1282	CarSat1	red	na
chrUn	41589650	41590496	847	CarSat1	red	na
chrUn	41590521	41590980	460	CarSat1	red	na
chrUn	41591007	41592898	1892	CarSat1	red	na
chrUn	41593515	41594686	1172	CarSat1	red	na
chrUn	41594713	41595420	708	CarSat1	red	na
chrUn	41595445	41596408	964	CarSat1	red	na
chrUn	41597022	41597585	564	CarSat1	red	na
chrUn	41597622	41598766	1145	CarSat1	red	na
chrUn	41600087	41600998	912	CarSat1	red	na
chrUn	41601025	41601731	707	CarSat1	red	na
chrUn	41601758	41602712	955	CarSat1	red	na
chrUn	41602737	41603617	881	CarSat1	red	na
chrUn	41604297	41605301	1005	CarSat1	red	na
chrUn	41605494	41608289	2796	CarSat1	red	na
chrUn	41608318	41608749	432	CarSat1	red	na
chrUn	41609837	41610372	536	Sat2CF	purple	na
chrUn	41623276	41624496	1221	Sat2CF	purple	na
chrUn	41639622	41640808	1187	Sat2CF	purple	na
chrUn	41726929	41728046	1118	SAT1_CF	blue	na
chrUn	41728126	41729352	1227	SAT1_CF	blue	na
chrUn	41729429	41730900	1472	SAT1_CF	blue	na
chrUn	41730913	41731076	164	SAT1_CF	blue	na
chrUn	41731095	41731536	442	SAT1_CF	blue	na
chrUn	41731678	41732199	522	SAT1_CF	blue	na
chrUn	41732246	41732381	136	SAT1_CF	blue	na
chrUn	41732413	41732920	508	SAT1_CF	blue	na
chrUn	41732963	41733526	564	SAT1_CF	blue	na
chrUn	41733607	41734258	652	SAT1_CF	blue	na
chrUn	41734301	41734967	667	SAT1_CF	blue	na

chrUn	41734989	41735671	683	SAT1_CF	blue	na
chrUn	41735684	41736202	519	SAT1_CF	blue	na
chrUn	41736239	41736587	349	SAT1_CF	blue	na
chrUn	41736620	41737656	1037	SAT1_CF	blue	na
chrUn	41737684	41738209	526	SAT1_CF	blue	na
chrUn	41738254	41738926	673	SAT1_CF	blue	na
chrUn	41738938	41741343	2406	SAT1_CF	blue	na
chrUn	41741385	41742054	670	SAT1_CF	blue	na
chrUn	41742066	41743260	1195	SAT1_CF	blue	na
chrUn	41743316	41743665	350	SAT1_CF	blue	na
chrUn	41743698	41744247	550	SAT1_CF	blue	na
chrUn	41744324	41745287	964	SAT1_CF	blue	na
chrUn	41745332	41745958	627	SAT1_CF	blue	na
chrUn	41745992	41747447	1456	SAT1_CF	blue	na
chrUn	41747477	41747905	429	SAT1_CF	blue	na
chrUn	41747949	41749280	1332	SAT1_CF	blue	na
chrUn	41749292	41749895	604	SAT1_CF	blue	na
chrUn	41749908	41750429	522	SAT1_CF	blue	na
chrUn	41750463	41751410	948	SAT1_CF	blue	na
chrUn	41751434	41751506	73	SAT1_CF	blue	na
chrUn	41751518	41752106	589	SAT1_CF	blue	na
chrUn	41752124	41752804	681	SAT1_CF	blue	na
chrUn	41752817	41753319	503	SAT1_CF	blue	na
chrUn	41753372	41753691	320	SAT1_CF	blue	na
chrUn	41753703	41754305	603	SAT1_CF	blue	na
chrUn	41754355	41755726	1372	SAT1_CF	blue	na
chrUn	41755769	41756531	763	SAT1_CF	blue	na
chrUn	41756543	41757045	503	SAT1_CF	blue	na
chrUn	41757087	41758019	933	SAT1_CF	blue	na
chrUn	41758037	41758214	178	SAT1_CF	blue	na
chrUn	41758692	41759784	1093	SAT1_CF	blue	na
chrUn	41759797	41760350	554	SAT1_CF	blue	na
chrUn	41760404	41760545	142	SAT1_CF	blue	na
chrUn	41761928	41763442	1515	SAT1_CF	blue	na
chrUn	41763500	41764175	676	SAT1_CF	blue	na
chrUn	41764187	41764789	603	SAT1_CF	blue	na
chrUn	41764802	41765323	522	SAT1_CF	blue	na
chrUn	41765349	41765506	158	SAT1_CF	blue	na
chrUn	41765538	41766041	504	SAT1_CF	blue	na
chrUn	41766073	41767310	1238	SAT1_CF	blue	na
chrUn	41767333	41768267	935	SAT1_CF	blue	na
chrUn	41768299	41769543	1245	SAT1_CF	blue	na

chrUn	41769571	41771435	1865	SAT1_CF	blue	na
chrUn	41771478	41772148	671	SAT1_CF	blue	na
chrUn	41772166	41772850	685	SAT1_CF	blue	na
chrUn	41772863	41773753	891	SAT1_CF	blue	na
chrUn	41773765	41775364	1600	SAT1_CF	blue	na
chrUn	41775445	41775994	550	SAT1_CF	blue	na
chrUn	41776037	41777100	1064	SAT1_CF	blue	na
chrUn	41777151	41777312	162	SAT1_CF	blue	na
chrUn	41777328	41777871	544	SAT1_CF	blue	na
chrUn	41777913	41779467	1555	SAT1_CF	blue	na
chrUn	41779480	41779998	519	SAT1_CF	blue	na
chrUn	41904988	41905720	733	SAT1_CF	blue	na
chrUn	41905762	41907551	1790	SAT1_CF	blue	na
chrUn	41907563	41908454	892	SAT1_CF	blue	na
chrUn	41908467	41909835	1369	SAT1_CF	blue	na
chrUn	41909880	41911654	1775	SAT1_CF	blue	na
chrUn	41911694	41914236	2543	SAT1_CF	blue	na
chrUn	41914249	41915616	1368	SAT1_CF	blue	na
chrUn	41915659	41917686	2028	SAT1_CF	blue	na
chrUn	41917699	41918201	503	SAT1_CF	blue	na
chrUn	41918223	41920287	2065	SAT1_CF	blue	na
chrUn	41920307	41920507	201	SAT1_CF	blue	na
chrUn	41920520	41921877	1358	SAT1_CF	blue	na
chrUn	41921920	41924035	2116	SAT1_CF	blue	na
chrUn	41924050	41924746	697	SAT1_CF	blue	na
chrUn	41924766	41925449	684	SAT1_CF	blue	na
chrUn	41925467	41926141	675	SAT1_CF	blue	na
chrUn	41926190	41927794	1605	SAT1_CF	blue	na
chrUn	41927838	41928636	799	SAT1_CF	blue	na
chrUn	41928649	41930017	1369	SAT1_CF	blue	na
chrUn	41930050	41930614	565	SAT1_CF	blue	na
chrUn	41930654	41931241	588	SAT1_CF	blue	na
chrUn	41931297	41932338	1042	SAT1_CF	blue	na
chrUn	41932350	41933212	863	SAT1_CF	blue	na
chrUn	41933392	41934114	723	SAT1_CF	blue	na
chrUn	41934127	41934739	613	SAT1_CF	blue	na
chrUn	41934751	41935599	849	SAT1_CF	blue	na
chrUn	41935684	41936229	546	SAT1_CF	blue	na
chrUn	41936255	41937089	835	SAT1_CF	blue	na
chrUn	41937109	41937456	348	SAT1_CF	blue	na
chrUn	41937574	41938812	1239	SAT1_CF	blue	na
chrUn	41938824	41939545	722	SAT1_CF	blue	na

chrUn	41939568	41940896	1329	SAT1_CF	blue	na
chrUn	41940928	41941925	998	SAT1_CF	blue	na
chrUn	41941959	41942751	793	SAT1_CF	blue	na
chrUn	41942763	41943439	677	SAT1_CF	blue	na
chrUn	41943488	41944682	1195	SAT1_CF	blue	na
chrUn	41944725	41945250	526	SAT1_CF	blue	na
chrUn	41945265	41945401	137	SAT1_CF	blue	na
chrUn	41945469	41945969	501	SAT1_CF	blue	na
chrUn	41945980	41946089	110	SAT1_CF	blue	na
chrUn	41946111	41946664	554	SAT1_CF	blue	na
chrUn	41946676	41947438	763	SAT1_CF	blue	na
chrUn	41947450	41948782	1333	SAT1_CF	blue	na
chrUn	41948826	41950152	1327	SAT1_CF	blue	na
chrUn	41950239	41950740	502	SAT1_CF	blue	na
chrUn	41950752	41950858	107	SAT1_CF	blue	na
chrUn	41950882	41951508	627	SAT1_CF	blue	na
chrUn	41951532	41951983	452	SAT1_CF	blue	na
chrUn	41951996	41952868	873	SAT1_CF	blue	na
chrUn	41952953	41953472	520	SAT1_CF	blue	na
chrUn	41953529	41954697	1169	SAT1_CF	blue	na
chrUn	41954794	41955936	1143	SAT1_CF	blue	na
chrUn	41955978	41956476	499	SAT1_CF	blue	na
chrUn	41956493	41957172	680	SAT1_CF	blue	na
chrUn	41957185	41957260	76	SAT1_CF	blue	na
chrUn	41957278	41957515	238	SAT1_CF	blue	na
chrUn	41958537	41959195	659	SAT1_CF	blue	na
chrUn	41959207	41960098	892	SAT1_CF	blue	na
chrUn	41960111	41960274	164	SAT1_CF	blue	na
chrUn	41960313	41960515	203	SAT1_CF	blue	na
chrUn	41960532	41961076	545	SAT1_CF	blue	na
chrUn	41961088	41961773	686	SAT1_CF	blue	na
chrUn	41961791	41962461	671	SAT1_CF	blue	na
chrUn	41962504	41964123	1620	SAT1_CF	blue	na
chrUn	41964134	41964614	481	SAT1_CF	blue	na
chrUn	41964753	41965689	937	SAT1_CF	blue	na
chrUn	41965727	41966227	501	SAT1_CF	blue	na
chrUn	41966249	41967872	1624	SAT1_CF	blue	na
chrUn	41967886	41968448	563	SAT1_CF	blue	na
chrUn	41969856	41970434	579	SAT1_CF	blue	na
chrUn	41970494	41971706	1213	SAT1_CF	blue	na
chrUn	41971982	41973380	1399	SAT1_CF	blue	na
chrUn	41973424	41974352	929	SAT1_CF	blue	na

chrUn	41974365	41975047	683	SAT1_CF	blue	na
chrUn	41975058	41975687	630	SAT1_CF	blue	na
chrUn	41975702	41976404	703	SAT1_CF	blue	na
chrUn	41976430	41977101	672	SAT1_CF	blue	na
chrUn	41977113	41977789	677	SAT1_CF	blue	na
chrUn	41977850	41978722	873	SAT1_CF	blue	na
chrUn	41978735	41978839	105	SAT1_CF	blue	na
chrUn	41978864	41980104	1241	SAT1_CF	blue	na
chrUn	41980151	41981330	1180	SAT1_CF	blue	na
chrUn	41981344	41982331	988	SAT1_CF	blue	na
chrUn	41982358	41982515	158	SAT1_CF	blue	na
chrUn	41982560	41983058	499	SAT1_CF	blue	na
chrUn	41983073	41983182	110	SAT1_CF	blue	na
chrUn	41983203	41983834	632	SAT1_CF	blue	na
chrUn	41983846	41984245	400	SAT1_CF	blue	na
chrUn	41984257	41984776	520	SAT1_CF	blue	na
chrUn	41984822	41985732	911	SAT1_CF	blue	na
chrUn	41985777	41989003	3227	SAT1_CF	blue	na
chrUn	41989016	41989191	176	SAT1_CF	blue	na
chrUn	41989220	41989741	522	SAT1_CF	blue	na
chrUn	41989754	41991767	2014	SAT1_CF	blue	na
chrUn	41991786	41992339	554	SAT1_CF	blue	na
chrUn	41992381	41993010	630	SAT1_CF	blue	na
chrUn	41993038	41993548	511	SAT1_CF	blue	na
chrUn	41993618	41994951	1334	SAT1_CF	blue	na
chrUn	41994963	41995823	861	SAT1_CF	blue	na
chrUn	41995865	41996406	542	SAT1_CF	blue	na
chrUn	41996444	41997492	1049	SAT1_CF	blue	na
chrUn	41997518	41998039	522	SAT1_CF	blue	na
chrUn	41998052	41998734	683	SAT1_CF	blue	na
chrUn	41998746	41999610	865	SAT1_CF	blue	na
chrUn	41999652	42001503	1852	SAT1_CF	blue	na
chrUn	42001524	42002167	644	SAT1_CF	blue	na
chrUn	42117810	42119148	1339	SAT1_CF	blue	na
chrUn	42119165	42120428	1264	SAT1_CF	blue	na
chrUn	42120440	42121201	762	SAT1_CF	blue	na
chrUn	42121214	42121716	503	SAT1_CF	blue	na
chrUn	42121756	42122714	959	SAT1_CF	blue	na
chrUn	42122793	42123755	963	SAT1_CF	blue	na
chrUn	42123798	42124463	666	SAT1_CF	blue	na
chrUn	42124486	42125097	612	SAT1_CF	blue	na
chrUn	42125108	42125661	554	SAT1_CF	blue	na

chrUn	42125704	42126266	563	SAT1_CF	blue	na
chrUn	42126331	42127020	690	SAT1_CF	blue	na
chrUn	42127040	42127695	656	SAT1_CF	blue	na
chrUn	42127709	42128201	493	SAT1_CF	blue	na
chrUn	42128212	42129292	1081	SAT1_CF	blue	na
chrUn	42129303	42129946	644	SAT1_CF	blue	na
chrUn	42129959	42130593	635	SAT1_CF	blue	na
chrUn	42130615	42131136	522	SAT1_CF	blue	na
chrUn	42131183	42131319	137	SAT1_CF	blue	na
chrUn	42131347	42133199	1853	SAT1_CF	blue	na
chrUn	42133242	42133906	665	SAT1_CF	blue	na
chrUn	42133929	42134936	1008	SAT1_CF	blue	na
chrUn	42136338	42136933	596	SAT1_CF	blue	na
chrUn	42136982	42137627	646	SAT1_CF	blue	na
chrUn	42137669	42137981	313	SAT1_CF	blue	na
chrUn	42138023	42138543	521	SAT1_CF	blue	na
chrUn	42138577	42138941	365	SAT1_CF	blue	na
chrUn	42138973	42141759	2787	SAT1_CF	blue	na
chrUn	42141801	42142666	866	SAT1_CF	blue	na
chrUn	42142678	42143231	554	SAT1_CF	blue	na
chrUn	42143252	42143357	106	SAT1_CF	blue	na
chrUn	42143370	42143972	603	SAT1_CF	blue	na
chrUn	42143984	42144616	633	SAT1_CF	blue	na
chrUn	42144673	42146591	1919	SAT1_CF	blue	na
chrUn	42146603	42147158	556	SAT1_CF	blue	na
chrUn	42147179	42147276	98	SAT1_CF	blue	na
chrUn	42147299	42149389	2091	SAT1_CF	blue	na
chrUn	42149416	42149896	481	SAT1_CF	blue	na
chrUn	42149956	42151335	1380	SAT1_CF	blue	na
chrUn	42151378	42153367	1990	SAT1_CF	blue	na
chrUn	42153387	42153882	496	SAT1_CF	blue	na
chrUn	42153927	42154034	108	SAT1_CF	blue	na
chrUn	42154097	42154619	523	SAT1_CF	blue	na
chrUn	42154649	42155463	815	SAT1_CF	blue	na
chrUn	42155476	42157063	1588	SAT1_CF	blue	na
chrUn	42157075	42157758	684	SAT1_CF	blue	na
chrUn	42157770	42158529	760	SAT1_CF	blue	na
chrUn	42158542	42159041	500	SAT1_CF	blue	na
chrUn	42159086	42159241	156	SAT1_CF	blue	na
chrUn	42159453	42160559	1107	SAT1_CF	blue	na
chrUn	42160616	42161140	525	SAT1_CF	blue	na
chrUn	42161158	42162310	1153	SAT1_CF	blue	na

chrUn	42162353	42163250	898	SAT1_CF	blue	na
chrUn	42163272	42163563	292	SAT1_CF	blue	na
chrUn	42163607	42164241	635	SAT1_CF	blue	na
chrUn	42164263	42164784	522	SAT1_CF	blue	na
chrUn	42164831	42164967	137	SAT1_CF	blue	na
chrUn	42164995	42166848	1854	SAT1_CF	blue	na
chrUn	42166891	42169358	2468	SAT1_CF	blue	na
chrUn	42170887	42171474	588	CarSat1	red	na
chrUn	42171675	42172589	915	CarSat1	red	na
chrUn	42172620	42174040	1421	CarSat1	red	na
chrUn	42174245	42174958	714	CarSat1	red	na
chrUn	42183532	42184119	588	CarSat1	red	na
chrUn	42184320	42185418	1099	CarSat1	red	na
chrUn	42185452	42185859	408	CarSat1	red	na
chrUn	42191646	42192655	1010	Sat2CF	purple	na
chrUn	42191709	42192569	861	Sat2CF	purple	na
chrUn	42192884	42193813	930	SAT1_CF	blue	na
chrUn	42193843	42194256	414	SAT1_CF	blue	na
chrUn	42194278	42194679	402	CarSat1	red	na
chrUn	42194698	42196477	1780	CarSat1	red	na
chrUn	42196511	42196916	406	CarSat1	red	na
chrUn	42205957	42206523	567	CarSat1	red	na
chrUn	42206713	42206834	122	CarSat1	red	na
chrUn	42206852	42207647	796	CarSat1	red	na
chrUn	42207674	42208116	443	CarSat1	red	na
chrUn	42208293	42209387	1095	CarSat1	red	na
chrUn	42212041	42212242	202	Sat2CF	purple	na
chrUn	42212082	42213332	1251	Sat2CF	purple	na
chrUn	42212118	42213202	1085	Sat2CF	purple	na
chrUn	42239571	42240212	642	SAT1_CF	blue	na
chrUn	42240233	42241217	985	SAT1_CF	blue	na
chrUn	42241233	42242305	1073	SAT1_CF	blue	na
chrUn	42242349	42242991	643	SAT1_CF	blue	na
chrUn	42243003	42243686	684	SAT1_CF	blue	na
chrUn	42243699	42244219	521	SAT1_CF	blue	na
chrUn	42244266	42244423	158	SAT1_CF	blue	na
chrUn	42244438	42245879	1442	SAT1_CF	blue	na
chrUn	42245921	42246780	860	SAT1_CF	blue	na
chrUn	42246792	42247305	514	SAT1_CF	blue	na
chrUn	42247327	42247399	73	SAT1_CF	blue	na
chrUn	42247412	42247912	501	SAT1_CF	blue	na
chrUn	42247999	42249220	1222	SAT1_CF	blue	na

chrUn	42249232	42249752	521	SAT1_CF	blue	na
chrUn	42249768	42250256	489	SAT1_CF	blue	na
chrUn	42250299	42250799	501	SAT1_CF	blue	na
chrUn	42250839	42251982	1144	SAT1_CF	blue	na
chrUn	42251996	42252391	396	SAT1_CF	blue	na
chrUn	42252412	42252534	123	SAT1_CF	blue	na
chrUn	42252546	42253796	1251	SAT1_CF	blue	na
chrUn	42253819	42253925	107	SAT1_CF	blue	na
chrUn	42253938	42254642	705	SAT1_CF	blue	na
chrUn	42254657	42255179	523	SAT1_CF	blue	na
chrUn	42255211	42255784	574	SAT1_CF	blue	na
chrUn	42255811	42256210	400	SAT1_CF	blue	na
chrUn	42256222	42257396	1175	SAT1_CF	blue	na
chrUn	42257459	42259558	2100	SAT1_CF	blue	na
chrUn	42259571	42260273	703	SAT1_CF	blue	na
chrUn	42260288	42260754	467	SAT1_CF	blue	na
chrUn	42260940	42261648	709	SAT1_CF	blue	na
chrUn	42261664	42262207	544	SAT1_CF	blue	na
chrUn	42262249	42263532	1284	SAT1_CF	blue	na
chrUn	42263576	42266035	2460	SAT1_CF	blue	na
chrUn	42381729	42381876	148	SAT1_CF	blue	na
chrUn	42381931	42382433	503	SAT1_CF	blue	na
chrUn	42382450	42383328	879	SAT1_CF	blue	na
chrUn	42384961	42385534	574	SAT1_CF	blue	na
chrUn	42385574	42386080	507	SAT1_CF	blue	na
chrUn	42386093	42386199	107	SAT1_CF	blue	na
chrUn	42386221	42386939	719	SAT1_CF	blue	na
chrUn	42386994	42388781	1788	SAT1_CF	blue	na
chrUn	42388853	42389353	501	SAT1_CF	blue	na
chrUn	42389367	42390038	672	SAT1_CF	blue	na
chrUn	42390050	42390159	110	SAT1_CF	blue	na
chrUn	42390179	42390724	546	SAT1_CF	blue	na
chrUn	42390756	42391344	589	SAT1_CF	blue	na
chrUn	42391357	42391859	503	SAT1_CF	blue	na
chrUn	42391872	42392666	795	SAT1_CF	blue	na
chrUn	42392678	42393360	683	SAT1_CF	blue	na
chrUn	42393373	42394054	682	SAT1_CF	blue	na
chrUn	42394066	42395658	1593	SAT1_CF	blue	na
chrUn	42395670	42396344	675	SAT1_CF	blue	na
chrUn	42396364	42397100	737	SAT1_CF	blue	na
chrUn	42397118	42397786	669	SAT1_CF	blue	na
chrUn	42397799	42397916	118	SAT1_CF	blue	na

chrUn	42397969	42398469	501	SAT1_CF	blue	na
chrUn	42398494	42399166	673	SAT1_CF	blue	na
chrUn	42399184	42401132	1949	SAT1_CF	blue	na
chrUn	42401219	42402501	1283	SAT1_CF	blue	na
chrUn	42402520	42403065	546	SAT1_CF	blue	na
chrUn	42403108	42403648	541	SAT1_CF	blue	na
chrUn	42403666	42404219	554	SAT1_CF	blue	na
chrUn	42404262	42404847	586	SAT1_CF	blue	na
chrUn	42404859	42406456	1598	SAT1_CF	blue	na
chrUn	42406468	42407802	1335	SAT1_CF	blue	na
chrUn	42407846	42409182	1337	SAT1_CF	blue	na
chrUn	42409250	42411175	1926	SAT1_CF	blue	na
chrUn	42411232	42411817	586	SAT1_CF	blue	na
chrUn	42411847	42412357	511	SAT1_CF	blue	na
chrUn	42412385	42412541	157	SAT1_CF	blue	na
chrUn	42412586	42413087	502	SAT1_CF	blue	na
chrUn	42413099	42413826	728	SAT1_CF	blue	na
chrUn	42413846	42415048	1203	SAT1_CF	blue	na
chrUn	42415092	42416053	962	SAT1_CF	blue	na
chrUn	42416073	42418143	2071	SAT1_CF	blue	na
chrUn	42418154	42418508	355	SAT1_CF	blue	na
chrUn	42418595	42419095	501	SAT1_CF	blue	na
chrUn	42419108	42419215	108	SAT1_CF	blue	na
chrUn	42419237	42420333	1097	SAT1_CF	blue	na
chrUn	42420376	42421338	963	SAT1_CF	blue	na
chrUn	42421417	42422377	961	SAT1_CF	blue	na
chrUn	42422436	42422917	482	SAT1_CF	blue	na
chrUn	42422944	42423729	786	SAT1_CF	blue	na
chrUn	42423752	42424297	546	SAT1_CF	blue	na
chrUn	42424364	42425128	765	SAT1_CF	blue	na
chrUn	42425141	42427078	1938	SAT1_CF	blue	na
chrUn	42427112	42427632	521	SAT1_CF	blue	na
chrUn	42427644	42428317	674	SAT1_CF	blue	na
chrUn	42428356	42430786	2431	SAT1_CF	blue	na
chrUn	42430874	42431458	585	SAT1_CF	blue	na
chrUn	42723233	42726253	3021	Sat7CF	lime	na
chrUn	42723629	42726873	3245	Sat7CF	lime	na
chrUn	42724245	42727460	3216	Sat7CF	lime	na
chrUn	42727628	42730851	3224	Sat7CF	lime	na
chrUn	42728237	42731472	3236	Sat7CF	lime	na
chrUn	42729162	42732540	3379	Sat7CF	lime	na
chrUn	42729779	42733151	3373	Sat7CF	lime	na

chrUn	42732086	42735434	3349	Sat7CF	lime	na
chrUn	42733758	42736977	3220	Sat7CF	lime	na
chrUn	42734373	42737595	3223	Sat7CF	lime	na
chrUn	42737446	42737628	183	Sat7CF	lime	na
chrUn	42762883	42763492	610	SAT1_CF	blue	na
chrUn	42763504	42764059	556	SAT1_CF	blue	na
chrUn	42764080	42764186	107	SAT1_CF	blue	na
chrUn	42764199	42764671	473	SAT1_CF	blue	na
chrUn	42764727	42764802	76	SAT1_CF	blue	na
chrUn	42764813	42765445	633	SAT1_CF	blue	na
chrUn	42765502	42765998	497	SAT1_CF	blue	na
chrUn	42766011	42767336	1326	SAT1_CF	blue	na
chrUn	42767348	42768331	984	SAT1_CF	blue	na
chrUn	42768374	42768898	525	SAT1_CF	blue	na
chrUn	42769010	42769789	780	SAT1_CF	blue	na
chrUn	42769802	42770652	851	SAT1_CF	blue	na
chrUn	42770664	42771341	678	SAT1_CF	blue	na
chrUn	42771368	42772216	849	SAT1_CF	blue	na
chrUn	42772251	42772873	623	SAT1_CF	blue	na
chrUn	42772906	42773520	615	SAT1_CF	blue	na
chrUn	42773565	42774351	787	SAT1_CF	blue	na
chrUn	42774363	42774965	603	SAT1_CF	blue	na
chrUn	42774977	42775656	680	SAT1_CF	blue	na
chrUn	42775668	42777018	1351	SAT1_CF	blue	na
chrUn	42777098	42777423	326	SAT1_CF	blue	na
chrUn	42777448	42778704	1257	SAT1_CF	blue	na
chrUn	42778716	42779268	553	SAT1_CF	blue	na
chrUn	42779282	42779362	81	SAT1_CF	blue	na
chrUn	42779375	42780302	928	SAT1_CF	blue	na
chrUn	42780314	42780996	683	SAT1_CF	blue	na
chrUn	42781008	42781768	761	SAT1_CF	blue	na
chrUn	42781781	42783476	1696	SAT1_CF	blue	na
chrUn	42783499	42783604	106	SAT1_CF	blue	na
chrUn	42783617	42784117	501	SAT1_CF	blue	na
chrUn	42784153	42784338	186	SAT1_CF	blue	na
chrUn	42784518	42787269	2752	SAT1_CF	blue	na
chrUn	42787297	42787398	102	SAT1_CF	blue	na
chrUn	42787411	42787931	521	SAT1_CF	blue	na
chrUn	42787948	42788321	374	SAT1_CF	blue	na
chrUn	42788364	42788913	550	SAT1_CF	blue	na
chrUn	42788991	42790886	1896	SAT1_CF	blue	na
chrUn	42790898	42792273	1376	SAT1_CF	blue	na

chrUn	42792286	42792805	520	SAT1_CF	blue	na
chrUn	42792824	42793084	261	SAT1_CF	blue	na
chrUn	42793128	42793674	547	SAT1_CF	blue	na
chrUn	42793806	42795116	1311	SAT1_CF	blue	na
chrUn	42795128	42795676	549	SAT1_CF	blue	na
chrUn	42795755	42796080	326	SAT1_CF	blue	na
chrUn	42796105	42797438	1334	SAT1_CF	blue	na
chrUn	42797450	42797931	482	SAT1_CF	blue	na
chrUn	42797991	42799476	1486	SAT1_CF	blue	na
chrUn	42799490	42800030	541	SAT1_CF	blue	na
chrUn	42800073	42800733	661	SAT1_CF	blue	na
chrUn	42800760	42801274	515	SAT1_CF	blue	na
chrUn	42801314	42801716	403	SAT1_CF	blue	na
chrUn	42801729	42802250	522	SAT1_CF	blue	na
chrUn	42802282	42803992	1711	SAT1_CF	blue	na
chrUn	42804014	42804166	153	SAT1_CF	blue	na
chrUn	42804398	42805919	1522	SAT1_CF	blue	na
chrUn	42805962	42806585	624	SAT1_CF	blue	na
chrUn	42806596	42807264	669	SAT1_CF	blue	na
chrUn	42807291	42807792	502	SAT1_CF	blue	na
chrUn	42807858	42808756	899	SAT1_CF	blue	na
chrUn	42808817	42810041	1225	SAT1_CF	blue	na
chrUn	42810054	42810555	502	SAT1_CF	blue	na
chrUn	42810595	42810964	370	SAT1_CF	blue	na
chrUn	42811081	42811604	524	SAT1_CF	blue	na
chrUn	42811635	42811913	279	SAT1_CF	blue	na
chrUn	42812937	42814136	1200	CarSat1	red	na
chrUn	42814166	42814748	583	CarSat1	red	na
chrUn	42817134	42818742	1609	CarSat1	red	na
chrUn	42819627	42823623	3997	CarSat1	red	na
chrUn	42823653	42826361	2709	CarSat1	red	na
chrUn	42826394	42829801	3408	CarSat1	red	na
chrUn	42829904	42834105	4202	CarSat1	red	na
chrUn	42835650	42838589	2940	CarSat1	red	na
chrUn	42838661	42839105	445	CarSat1	red	na
chrUn	42839135	42839196	62	CarSat1	red	na
chrUn	42839385	42840788	1404	CarSat1	red	na
chrUn	42840818	42841923	1106	CarSat1	red	na
chrUn	42842952	42844045	1094	CarSat1	red	na
chrUn	42844776	42845225	450	CarSat1	red	na
chrUn	42845240	42845367	128	Carsat2	orange	na
chrUn	42845255	42847946	2692	CarSat1	red	na

chrUn	42847972	42848987	1016	CarSat1	red	na
chrUn	42849731	42851220	1490	CarSat1	red	na
chrUn	42851254	42851441	188	Carsat2	orange	na
chrUn	42851273	42852983	1711	CarSat1	red	na
chrUn	42853007	42854676	1670	CarSat1	red	na
chrUn	42854691	42854817	127	Carsat2	orange	na
chrUn	42854706	42855412	707	CarSat1	red	na
chrUn	42855442	42856415	974	CarSat1	red	na
chrUn	42856426	42857799	1374	CarSat1	red	na
chrUn	42857829	42859273	1445	CarSat1	red	na
chrUn	42859288	42859415	128	Carsat2	orange	na
chrUn	42859303	42860011	709	CarSat1	red	na
chrUn	42860041	42860527	487	CarSat1	red	na
chrUn	42860551	42861124	574	CarSat1	red	na
chrUn	42861183	42862892	1710	CarSat1	red	na
chrUn	42862904	42863850	947	CarSat1	red	na
chrUn	42863880	42864441	562	CarSat1	red	na
chrUn	42864626	42865170	545	CarSat1	red	na
chrUn	42865419	42865545	127	Carsat2	orange	na
chrUn	42865434	42866405	972	CarSat1	red	na
chrUn	42866417	42866845	429	CarSat1	red	na
chrUn	42869079	42870662	1584	CarSat1	red	na
chrUn	42891186	42891555	370	Sat15CF	na	na
chrUn	42891186	42891555	370	Sat15CF	na	na
chrUn	42891613	42891869	257	Sat15CF	na	na
chrUn	42891613	42891869	257	Sat15CF	na	na
chrUn	42907948	42908402	455	CarSat1	red	na
chrUn	42947299	42947582	284	SAT1_CF	blue	na
chrUn	42948992	42949562	571	SAT1_CF	blue	na
chrUn	42949605	42950099	495	SAT1_CF	blue	na
chrUn	42950119	42950795	677	SAT1_CF	blue	na
chrUn	42950839	42951630	792	SAT1_CF	blue	na
chrUn	42951654	42952419	766	SAT1_CF	blue	na
chrUn	42952431	42954014	1584	SAT1_CF	blue	na
chrUn	42954032	42954919	888	SAT1_CF	blue	na
chrUn	42955036	42955574	539	SAT1_CF	blue	na
chrUn	42955594	42955701	108	SAT1_CF	blue	na
chrUn	42955722	42956295	574	SAT1_CF	blue	na
chrUn	42956307	42957534	1228	SAT1_CF	blue	na
chrUn	42957547	42957653	107	SAT1_CF	blue	na
chrUn	42957676	42958630	955	SAT1_CF	blue	na
chrUn	42958670	42959168	499	SAT1_CF	blue	na

chrUn	42959180	42960891	1712	SAT1_CF	blue	na
chrUn	42960919	42961075	157	SAT1_CF	blue	na
chrUn	42961120	42961619	500	SAT1_CF	blue	na
chrUn	42961633	42962321	689	SAT1_CF	blue	na
chrUn	42962333	42963243	911	SAT1_CF	blue	na
chrUn	42963271	42964194	924	SAT1_CF	blue	na
chrUn	42964234	42965057	824	SAT1_CF	blue	na
chrUn	42965068	42965558	491	SAT1_CF	blue	na
chrUn	42965674	42966550	877	SAT1_CF	blue	na
chrUn	42966636	42967136	501	SAT1_CF	blue	na
chrUn	42967149	42968468	1320	SAT1_CF	blue	na
chrUn	42968512	42969480	969	SAT1_CF	blue	na
chrUn	42969525	42969894	370	SAT1_CF	blue	na
chrUn	42969911	42970430	520	SAT1_CF	blue	na
chrUn	42970457	42972076	1620	SAT1_CF	blue	na
chrUn	42972117	42972661	545	SAT1_CF	blue	na
chrUn	42972676	42973562	887	SAT1_CF	blue	na
chrUn	42973605	42973970	366	SAT1_CF	blue	na
chrUn	42974030	42974512	483	SAT1_CF	blue	na
chrUn	42974525	42975128	604	SAT1_CF	blue	na
chrUn	42975146	42975828	683	SAT1_CF	blue	na
chrUn	42975842	42976908	1067	SAT1_CF	blue	na
chrUn	42976987	42977949	963	SAT1_CF	blue	na
chrUn	42977987	42979358	1372	SAT1_CF	blue	na
chrUn	42979376	42980045	670	SAT1_CF	blue	na
chrUn	42980057	42980222	166	SAT1_CF	blue	na
chrUn	42980272	42980815	544	SAT1_CF	blue	na
chrUn	42980858	42981384	527	SAT1_CF	blue	na
chrUn	42981427	42981970	544	SAT1_CF	blue	na
chrUn	42981982	42982655	674	SAT1_CF	blue	na
chrUn	42982725	42983198	474	SAT1_CF	blue	na
chrUn	42983210	42983876	667	SAT1_CF	blue	na
chrUn	42983919	42984637	719	SAT1_CF	blue	na
chrUn	42984676	42985239	564	SAT1_CF	blue	na
chrUn	42985252	42987587	2336	SAT1_CF	blue	na
chrUn	42987600	42987998	399	SAT1_CF	blue	na
chrUn	42988023	42988537	515	SAT1_CF	blue	na
chrUn	42988566	42991310	2745	SAT1_CF	blue	na
chrUn	42991321	42991806	486	SAT1_CF	blue	na
chrUn	42991826	42993191	1366	SAT1_CF	blue	na
chrUn	42993291	42994413	1123	SAT1_CF	blue	na
chrUn	42994424	42995710	1287	SAT1_CF	blue	na

chrUn	43124416	43125300	885	SAT1_CF	blue	na
chrUn	43159559	43160232	674	Sat29CF	na	na
chrUn	43160020	43160258	239	Sat29CF	na	na
chrUn	43228275	43229433	1159	Sat2CF	purple	na
chrUn	43247482	43248648	1167	Sat2CF	purple	na
chrUn	43274152	43274706	555	SAT1_CF	blue	na
chrUn	43274793	43275148	356	SAT1_CF	blue	na
chrUn	43275161	43275705	545	SAT1_CF	blue	na
chrUn	43275749	43276592	844	SAT1_CF	blue	na
chrUn	43276612	43278155	1544	SAT1_CF	blue	na
chrUn	43278168	43278670	503	SAT1_CF	blue	na
chrUn	43278710	43279112	403	SAT1_CF	blue	na
chrUn	43279125	43279632	508	SAT1_CF	blue	na
chrUn	43279717	43280489	773	SAT1_CF	blue	na
chrUn	43280531	43281391	861	SAT1_CF	blue	na
chrUn	43281404	43281962	559	SAT1_CF	blue	na
chrUn	43281982	43282090	109	SAT1_CF	blue	na
chrUn	43282103	43282623	521	SAT1_CF	blue	na
chrUn	43282648	43282804	157	SAT1_CF	blue	na
chrUn	43283017	43284170	1154	SAT1_CF	blue	na
chrUn	43284183	43285221	1039	SAT1_CF	blue	na
chrUn	43285264	43285938	675	SAT1_CF	blue	na
chrUn	43285950	43286708	759	SAT1_CF	blue	na
chrUn	43286721	43287223	503	SAT1_CF	blue	na
chrUn	43287266	43287447	182	SAT1_CF	blue	na
chrUn	43287494	43288039	546	SAT1_CF	blue	na
chrUn	43288118	43288621	504	SAT1_CF	blue	na
chrUn	43289331	43290195	865	SAT1_CF	blue	na
chrUn	43290251	43290624	374	SAT1_CF	blue	na
chrUn	43290669	43291341	673	SAT1_CF	blue	na
chrUn	43291353	43292038	686	SAT1_CF	blue	na
chrUn	43292049	43292551	503	SAT1_CF	blue	na
chrUn	43292597	43294004	1408	SAT1_CF	blue	na
chrUn	43294046	43294902	857	SAT1_CF	blue	na
chrUn	43294914	43295929	1016	SAT1_CF	blue	na
chrUn	43295952	43296025	74	SAT1_CF	blue	na
chrUn	43296036	43296538	503	SAT1_CF	blue	na
chrUn	43296584	43296758	175	SAT1_CF	blue	na
chrUn	43296940	43297176	237	SAT1_CF	blue	na
chrUn	43298971	43300277	1307	SAT1_CF	blue	na
chrUn	43300306	43301373	1068	SAT1_CF	blue	na
chrUn	43301495	43302863	1369	SAT1_CF	blue	na

chrUn	43302885	43304854	1970	SAT1_CF	blue	na
chrUn	43304877	43304982	106	SAT1_CF	blue	na
chrUn	43304995	43305573	579	SAT1_CF	blue	na
chrUn	43305653	43306262	610	SAT1_CF	blue	na
chrUn	43306282	43307440	1159	SAT1_CF	blue	na
chrUn	43307452	43308083	632	SAT1_CF	blue	na
chrUn	43308106	43308309	204	SAT1_CF	blue	na
chrUn	43310985	43311716	732	SAT1_CF	blue	na
chrUn	43311739	43311844	106	SAT1_CF	blue	na
chrUn	43311857	43312337	481	SAT1_CF	blue	na
chrUn	43312370	43312876	507	SAT1_CF	blue	na
chrUn	43314049	43315386	1338	SAT1_CF	blue	na
chrUn	43315399	43316103	705	SAT1_CF	blue	na
chrUn	43316168	43316715	548	SAT1_CF	blue	na
chrUn	43316775	43316953	179	SAT1_CF	blue	na
chrUn	43316995	43317539	545	SAT1_CF	blue	na
chrUn	43317582	43318259	678	SAT1_CF	blue	na
chrUn	43318271	43318896	626	SAT1_CF	blue	na
chrUn	43318909	43319430	522	SAT1_CF	blue	na
chrUn	43319447	43319820	374	SAT1_CF	blue	na
chrUn	43319863	43320387	525	SAT1_CF	blue	na
chrUn	43320400	43320484	85	SAT1_CF	blue	na
chrUn	43320528	43321650	1123	SAT1_CF	blue	na
chrUn	43321663	43322687	1025	SAT1_CF	blue	na
chrUn	43322757	43324365	1609	SAT1_CF	blue	na
chrUn	43324377	43324821	445	SAT1_CF	blue	na
chrUn	43324844	43324915	72	SAT1_CF	blue	na
chrUn	43324928	43325689	762	SAT1_CF	blue	na
chrUn	43325709	43326501	793	SAT1_CF	blue	na
chrUn	43327530	43328138	609	SAT1_CF	blue	na
chrUn	43328159	43328704	546	SAT1_CF	blue	na
chrUn	43328722	43330215	1494	SAT1_CF	blue	na
chrUn	43330258	43330933	676	SAT1_CF	blue	na
chrUn	43330945	43331639	695	SAT1_CF	blue	na
chrUn	43331666	43332176	511	SAT1_CF	blue	na
chrUn	43332219	43332763	545	SAT1_CF	blue	na
chrUn	43332805	43332977	173	SAT1_CF	blue	na
chrUn	43332990	43333536	547	SAT1_CF	blue	na
chrUn	43333556	43333665	110	SAT1_CF	blue	na
chrUn	43333677	43334232	556	SAT1_CF	blue	na
chrUn	43334255	43334363	109	SAT1_CF	blue	na
chrUn	43334375	43334893	519	SAT1_CF	blue	na

chrUn	43334939	43337204	2266	SAT1_CF	blue	na
chrUn	43337244	43338611	1368	SAT1_CF	blue	na
chrUn	43338626	43339166	541	SAT1_CF	blue	na
chrUn	43339208	43341603	2396	SAT1_CF	blue	na
chrUn	43341631	43342262	632	SAT1_CF	blue	na
chrUn	43342305	43342850	546	SAT1_CF	blue	na
chrUn	43342870	43342979	110	SAT1_CF	blue	na
chrUn	43342991	43344589	1599	SAT1_CF	blue	na
chrUn	43344601	43345568	968	SAT1_CF	blue	na
chrUn	43345612	43346155	544	SAT1_CF	blue	na
chrUn	43346197	43348193	1997	SAT1_CF	blue	na
chrUn	43348206	43348707	502	SAT1_CF	blue	na
chrUn	43348760	43349682	923	SAT1_CF	blue	na
chrUn	43349699	43350741	1043	SAT1_CF	blue	na
chrUn	43350762	43352507	1746	SAT1_CF	blue	na
chrUn	43352553	43353188	636	SAT1_CF	blue	na
chrUn	43353200	43353753	554	SAT1_CF	blue	na
chrUn	43353796	43354451	656	SAT1_CF	blue	na
chrUn	43354475	43355155	681	SAT1_CF	blue	na
chrUn	43355168	43355670	503	SAT1_CF	blue	na
chrUn	43355710	43356667	958	SAT1_CF	blue	na
chrUn	43356746	43358116	1371	SAT1_CF	blue	na
chrUn	43358127	43359602	1476	SAT1_CF	blue	na
chrUn	43359643	43360356	714	SAT1_CF	blue	na
chrUn	43360368	43360869	502	SAT1_CF	blue	na
chrUn	43360922	43361867	946	SAT1_CF	blue	na
chrUn	43361946	43362906	961	SAT1_CF	blue	na
chrUn	43362949	43363752	804	SAT1_CF	blue	na
chrUn	43363766	43364463	698	SAT1_CF	blue	na
chrUn	43364505	43365050	546	SAT1_CF	blue	na
chrUn	43365093	43365776	684	SAT1_CF	blue	na
chrUn	43365788	43366998	1211	SAT1_CF	blue	na
chrUn	43367024	43367543	520	SAT1_CF	blue	na
chrUn	43367590	43367726	137	SAT1_CF	blue	na
chrUn	43367741	43368262	522	SAT1_CF	blue	na
chrUn	43368331	43369063	733	SAT1_CF	blue	na
chrUn	43369076	43371379	2304	SAT1_CF	blue	na
chrUn	43371397	43371878	482	SAT1_CF	blue	na
chrUn	43371945	43372464	520	SAT1_CF	blue	na
chrUn	43372497	43373043	547	SAT1_CF	blue	na
chrUn	43373057	43374198	1142	SAT1_CF	blue	na
chrUn	43504477	43504879	403	Sat2CF	purple	na

chrUn	43504557	43505122	566	Sat2CF	purple	na
chrUn	43504972	43505140	169	Sat2CF	purple	na
chrUn	43509804	43510119	316	SAT1_CF	blue	na
chrUn	43526198	43527681	1484	CarSat1	red	na
chrUn	43527709	43528476	768	CarSat1	red	na
chrUn	43546005	43546850	846	Sat2CF	purple	na
chrUn	43546113	43546836	724	Sat2CF	purple	na
chrUn	43561688	43561934	247	Sat2CF	purple	na
chrUn	43561724	43561940	217	Sat2CF	purple	na
chrUn	43597756	43598136	381	CarSat1	red	na
chrUn	43599104	43599957	854	CarSat1	red	na
chrUn	43599978	43600869	892	CarSat1	red	na
chrUn	43726018	43726262	245	CarSat1	red	na
chrUn	43726291	43726598	308	CarSat1	red	na
chrUn	43726619	43728208	1590	CarSat1	red	na
chrUn	43728229	43730569	2341	CarSat1	red	na
chrUn	43730590	43732566	1977	CarSat1	red	na
chrUn	43732569	43734045	1477	SAT1_CF	blue	na
chrUn	43736724	43736954	231	SAT1_CF	blue	na
chrUn	43737023	43740598	3576	SAT1_CF	blue	na
chrUn	43740616	43741287	672	SAT1_CF	blue	na
chrUn	43741330	43741809	480	SAT1_CF	blue	na
chrUn	43741843	43742371	529	SAT1_CF	blue	na
chrUn	43742391	43743038	648	SAT1_CF	blue	na
chrUn	43743080	43744065	986	SAT1_CF	blue	na
chrUn	43744077	43744742	666	SAT1_CF	blue	na
chrUn	43744753	43746238	1486	SAT1_CF	blue	na
chrUn	43746279	43747282	1004	SAT1_CF	blue	na
chrUn	43747309	43747815	507	SAT1_CF	blue	na
chrUn	43747887	43749025	1139	SAT1_CF	blue	na
chrUn	43749011	43749724	714	CarSat1	red	na
chrUn	43749743	43750180	438	CarSat1	red	na
chrUn	43770166	43771393	1228	Sat2CF	purple	na
chrUn	43810807	43811628	822	Sat2CF	purple	na
chrUn	43810881	43811606	726	Sat2CF	purple	na
chrUn	43811498	43811665	168	Sat2CF	purple	na
chrUn	43833894	43834245	352	Sat2CF	purple	na
chrUn	43833921	43834311	391	Sat2CF	purple	na
chrUn	43846306	43846483	178	Sat2CF	purple	na
chrUn	43846325	43847432	1108	Sat2CF	purple	na
chrUn	43884378	43884875	498	Sat11CF	na	na
chrUn	43884516	43884875	360	Sat11CF	na	na

chrUn	43885110	43885280	171	Sat11CF	na	na
chrUn	43943777	43944244	468	Sat11CF	na	na
chrUn	43943914	43944244	331	Sat11CF	na	na
chrUn	43994465	43995418	954	Sat7CF	lime	na
chrUn	43994465	43995418	954	Sat7CF	lime	na
chrUn	44202465	44202588	124	Sat2CF	purple	na
chrUn	44202496	44203157	662	Sat2CF	purple	na
chrUn	44214480	44214950	471	Sat2CF	purple	na
chrUn	44214532	44215016	485	Sat2CF	purple	na
chrUn	44236514	44237435	922	Sat2CF	purple	na
chrUn	44236577	44237389	813	Sat2CF	purple	na
chrUn	44263390	44264639	1250	Sat2CF	purple	na
chrUn	44266312	44266725	414	CarSat1	red	na
chrUn	44267533	44267675	143	CarSat1	red	na
chrUn	44267722	44268547	826	CarSat1	red	na
chrUn	44268568	44270162	1595	CarSat1	red	na
chrUn	44270183	44271036	854	CarSat1	red	na
chrUn	44271057	44272047	991	CarSat1	red	na
chrUn	44307102	44307381	280	Sat15CF	na	na
chrUn	44307491	44307819	329	Sat15CF	na	na
chrUn	44318219	44318475	257	Sat15CF	na	na
chrUn	44318219	44318475	257	Sat15CF	na	na
chrUn	44318537	44318906	370	Sat15CF	na	na
chrUn	44462357	44463244	888	CarSat1	red	na
chrUn	44463259	44463385	127	Carsat2	orange	na
chrUn	44463274	44463759	486	CarSat1	red	na
chrUn	44463792	44465786	1995	CarSat1	red	na
chrUn	44465808	44466511	704	CarSat1	red	na
chrUn	44474528	44474955	428	CarSat1	red	na
chrUn	44475154	44475288	135	CarSat1	red	na
chrUn	44475326	44476812	1487	CarSat1	red	na
chrUn	44506873	44506977	105	SAT1_CF	blue	na
chrUn	44511657	44512334	678	Sat2CF	purple	na
chrUn	44512255	44512344	90	Sat2CF	purple	na
chrUn	44681461	44681536	76	Sat15CF	na	na
chrUn	44796650	44797726	1077	Sat2CF	purple	na
chrUn	44816193	44816419	227	Sat2CF	purple	na
chrUn	44816222	44816419	198	Sat2CF	purple	na
chrUn	44831620	44832323	704	CarSat1	red	na
chrUn	44832345	44834314	1970	CarSat1	red	na
chrUn	44834316	44834751	436	SAT1_CF	blue	na
chrUn	44835751	44836362	612	SAT1_CF	blue	na

chrUn	44836404	44837775	1372	SAT1_CF	blue	na
chrUn	44837788	44838670	883	SAT1_CF	blue	na
chrUn	44838693	44839373	681	SAT1_CF	blue	na
chrUn	44839393	44839887	495	SAT1_CF	blue	na
chrUn	44839974	44840456	483	SAT1_CF	blue	na
chrUn	44840488	44841103	616	CarSat1	red	na
chrUn	44841118	44841244	127	Carsat2	orange	na
chrUn	44841133	44844561	3429	CarSat1	red	na
chrUn	44844594	44846257	1664	CarSat1	red	na
chrUn	44846287	44848252	1966	CarSat1	red	na
chrUn	44848277	44849204	928	CarSat1	red	na
chrUn	44849234	44849945	712	CarSat1	red	na
chrUn	44849975	44850801	827	CarSat1	red	na
chrUn	44851975	44852553	579	CarSat1	red	na
chrUn	44852583	44853295	713	CarSat1	red	na
chrUn	44853325	44854773	1449	CarSat1	red	na
chrUn	44854803	44856769	1967	CarSat1	red	na
chrUn	44856793	44857733	941	CarSat1	red	na
chrUn	44857748	44857874	127	Carsat2	orange	na
chrUn	44857763	44858470	708	CarSat1	red	na
chrUn	44858485	44858611	127	Carsat2	orange	na
chrUn	44858500	44859207	708	CarSat1	red	na
chrUn	44859237	44859944	708	CarSat1	red	na
chrUn	44859959	44860085	127	Carsat2	orange	na
chrUn	44859974	44862153	2180	CarSat1	red	na
chrUn	44862183	44862668	486	CarSat1	red	na
chrUn	44862692	44863634	943	CarSat1	red	na
chrUn	44863664	44865625	1962	CarSat1	red	na
chrUn	44865649	44867107	1459	CarSat1	red	na
chrUn	44867129	44868584	1456	CarSat1	red	na
chrUn	44868608	44870286	1679	CarSat1	red	na
chrUn	44870316	44872500	2185	CarSat1	red	na
chrUn	44872515	44872642	128	Carsat2	orange	na
chrUn	44872530	44873016	487	CarSat1	red	na
chrUn	44873040	44873976	937	CarSat1	red	na
chrUn	44874006	44876195	2190	CarSat1	red	na
chrUn	44876210	44876337	128	Carsat2	orange	na
chrUn	44876225	44876710	486	CarSat1	red	na
chrUn	44876734	44878197	1464	CarSat1	red	na
chrUn	44878213	44879146	934	CarSat1	red	na
chrUn	44879161	44879287	127	Carsat2	orange	na
chrUn	44879176	44880030	855	CarSat1	red	na

chrUn	44942469	44943466	998	SAT1_CF	blue	na
chrUn	44943479	44943979	501	SAT1_CF	blue	na
chrUn	44944016	44944278	263	SAT1_CF	blue	na
chrUn	44944322	44944869	548	SAT1_CF	blue	na
chrUn	44944912	44945474	563	SAT1_CF	blue	na
chrUn	44945497	44945603	107	SAT1_CF	blue	na
chrUn	44945616	44946140	525	SAT1_CF	blue	na
chrUn	44946166	44946323	158	SAT1_CF	blue	na
chrUn	44946535	44947742	1208	SAT1_CF	blue	na
chrUn	44947770	44949685	1916	SAT1_CF	blue	na
chrUn	44949699	44949868	170	SAT1_CF	blue	na
chrUn	44949883	44951208	1326	SAT1_CF	blue	na
chrUn	44951231	44951723	493	SAT1_CF	blue	na
chrUn	44951768	44951923	156	SAT1_CF	blue	na
chrUn	44952151	44952656	506	SAT1_CF	blue	na
chrUn	44952699	44953229	531	SAT1_CF	blue	na
chrUn	44953256	44953889	634	SAT1_CF	blue	na
chrUn	44953932	44955304	1373	SAT1_CF	blue	na
chrUn	44955317	44955818	502	SAT1_CF	blue	na
chrUn	44955861	44958481	2621	SAT1_CF	blue	na
chrUn	44958587	44958941	355	SAT1_CF	blue	na
chrUn	44958956	44961295	2340	SAT1_CF	blue	na
chrUn	44961307	44963330	2024	SAT1_CF	blue	na
chrUn	44963409	44963769	361	SAT1_CF	blue	na
chrUn	44963794	44964336	543	SAT1_CF	blue	na
chrUn	44964433	44965110	678	SAT1_CF	blue	na
chrUn	44965122	44965806	685	SAT1_CF	blue	na
chrUn	44965819	44966320	502	SAT1_CF	blue	na
chrUn	44966373	44967256	884	SAT1_CF	blue	na
chrUn	44967288	44967624	337	SAT1_CF	blue	na
chrUn	44967672	44969005	1334	SAT1_CF	blue	na
chrUn	44969018	44969520	503	SAT1_CF	blue	na
chrUn	44969565	44969672	108	SAT1_CF	blue	na
chrUn	44969735	44970261	527	SAT1_CF	blue	na
chrUn	44970279	44971070	792	SAT1_CF	blue	na
chrUn	44971082	44971633	552	SAT1_CF	blue	na
chrUn	44971651	44971727	77	SAT1_CF	blue	na
chrUn	44971740	44972011	272	SAT1_CF	blue	na
chrUn	44972069	44973153	1085	SAT1_CF	blue	na
chrUn	44973165	44974236	1072	SAT1_CF	blue	na
chrUn	44974250	44975942	1693	SAT1_CF	blue	na
chrUn	44975965	44976070	106	SAT1_CF	blue	na

chrUn	44976083	44976783	701	SAT1_CF	blue	na
chrUn	44977045	44977367	323	SAT1_CF	blue	na
chrUn	44977414	44977956	543	SAT1_CF	blue	na
chrUn	44978000	44978676	677	SAT1_CF	blue	na
chrUn	44978690	44979367	678	SAT1_CF	blue	na
chrUn	44979379	44980061	683	SAT1_CF	blue	na
chrUn	44980074	44980773	700	SAT1_CF	blue	na
chrUn	44980801	44982437	1637	SAT1_CF	blue	na
chrUn	44982506	44983350	845	SAT1_CF	blue	na
chrUn	44983363	44984642	1280	SAT1_CF	blue	na
chrUn	44984667	44985269	603	SAT1_CF	blue	na
chrUn	44986274	44986832	559	CarSat1	red	na
chrUn	44986857	44987493	637	CarSat1	red	na
chrUn	45020444	45022212	1769	CarSat1	red	na
chrUn	45022228	45023191	964	CarSat1	red	na
chrUn	45023218	45023930	713	CarSat1	red	na
chrUn	45023957	45025281	1325	CarSat1	red	na
chrUn	45025308	45026155	848	CarSat1	red	na
chrUn	45026194	45027157	964	CarSat1	red	na
chrUn	45027173	45028135	963	CarSat1	red	na
chrUn	45028162	45028871	710	CarSat1	red	na
chrUn	45028898	45029810	913	CarSat1	red	na
chrUn	45031610	45032796	1187	CarSat1	red	na
chrUn	45043854	45044570	717	Sat2CF	purple	na
chrUn	45043931	45044638	708	Sat2CF	purple	na
chrUn	45091704	45092653	950	CarSat1	red	na
chrUn	45092802	45093523	722	CarSat1	red	na
chrUn	45093550	45094019	470	CarSat1	red	na
chrUn	45094204	45094552	349	CarSat1	red	na
chrUn	45094765	45095414	650	CarSat1	red	na
chrUn	45095518	45096366	849	CarSat1	red	na
chrUn	45096387	45097172	786	CarSat1	red	na
chrUn	45097384	45098032	649	CarSat1	red	na
chrUn	45098050	45098523	474	CarSat1	red	na
chrUn	45098540	45099980	1441	CarSat1	red	na
chrUn	45099983	45100369	387	SAT1_CF	blue	na
chrUn	45100380	45105339	4960	SAT1_CF	blue	na
chrUn	45105358	45105881	524	SAT1_CF	blue	na
chrUn	45105908	45106644	737	SAT1_CF	blue	na
chrUn	45106661	45108201	1541	SAT1_CF	blue	na
chrUn	45108254	45108364	111	SAT1_CF	blue	na
chrUn	45108393	45109132	740	SAT1_CF	blue	na

chrUn	45109127	45109851	725	CarSat1	red	na
chrUn	45110000	45111461	1462	CarSat1	red	na
chrUn	45111479	45113451	1973	CarSat1	red	na
chrUn	45113442	45114538	1097	SAT1_CF	blue	na
chrUn	45114550	45115954	1405	SAT1_CF	blue	na
chrUn	45115997	45117389	1393	SAT1_CF	blue	na
chrUn	45117409	45118561	1153	SAT1_CF	blue	na
chrUn	45118556	45119274	719	CarSat1	red	na
chrUn	45119432	45119715	284	CarSat1	red	na
chrUn	45121459	45122023	565	SAT1_CF	blue	na
chrUn	45122095	45122767	673	SAT1_CF	blue	na
chrUn	45122779	45123461	683	SAT1_CF	blue	na
chrUn	45123474	45123956	483	SAT1_CF	blue	na
chrUn	45124032	45124407	376	SAT1_CF	blue	na
chrUn	45124418	45126040	1623	SAT1_CF	blue	na
chrUn	45126083	45126913	831	SAT1_CF	blue	na
chrUn	45127254	45128936	1683	SAT1_CF	blue	na
chrUn	45128956	45129094	139	SAT1_CF	blue	na
chrUn	45131061	45131831	771	SAT1_CF	blue	na
chrUn	45131845	45132346	502	SAT1_CF	blue	na
chrUn	45133346	45134023	678	SAT1_CF	blue	na
chrUn	45134035	45134709	675	SAT1_CF	blue	na
chrUn	45134731	45135208	478	SAT1_CF	blue	na
chrUn	45135315	45135830	516	SAT1_CF	blue	na
chrUn	45136533	45137117	585	SAT1_CF	blue	na
chrUn	45137159	45137888	730	SAT1_CF	blue	na
chrUn	45137958	45139326	1369	SAT1_CF	blue	na
chrUn	45139369	45141026	1658	SAT1_CF	blue	na
chrUn	45141039	45143898	2860	SAT1_CF	blue	na
chrUn	45143910	45144578	669	SAT1_CF	blue	na
chrUn	45144590	45146551	1962	SAT1_CF	blue	na
chrUn	45146595	45147807	1213	SAT1_CF	blue	na
chrUn	45147828	45148450	623	SAT1_CF	blue	na
chrUn	45148536	45149037	502	SAT1_CF	blue	na
chrUn	45149086	45149625	540	SAT1_CF	blue	na
chrUn	45149674	45150345	672	SAT1_CF	blue	na
chrUn	45150357	45151038	682	SAT1_CF	blue	na
chrUn	45151050	45151681	632	SAT1_CF	blue	na
chrUn	45151736	45154787	3052	SAT1_CF	blue	na
chrUn	45154800	45155354	555	SAT1_CF	blue	na
chrUn	45155424	45156030	607	SAT1_CF	blue	na
chrUn	45156041	45156929	889	SAT1_CF	blue	na

chrUn	45156944	45157135	192	SAT1_CF	blue	na
chrUn	45157268	45157806	539	SAT1_CF	blue	na
chrUn	45157854	45159072	1219	SAT1_CF	blue	na
chrUn	45159093	45160948	1856	SAT1_CF	blue	na
chrUn	45160990	45162214	1225	SAT1_CF	blue	na
chrUn	45162226	45162834	609	SAT1_CF	blue	na
chrUn	45162846	45163401	556	SAT1_CF	blue	na
chrUn	45163424	45163532	109	SAT1_CF	blue	na
chrUn	45163544	45164064	521	SAT1_CF	blue	na
chrUn	45164090	45165245	1156	SAT1_CF	blue	na
chrUn	45165273	45165903	631	SAT1_CF	blue	na
chrUn	45165946	45166491	546	SAT1_CF	blue	na
chrUn	45166513	45167399	887	SAT1_CF	blue	na
chrUn	45224796	45225006	211	Sat2CF	purple	na
chrUn	45224799	45224926	128	Sat2CF	purple	na
chrUn	45302020	45302783	764	SAT1_CF	blue	na
chrUn	45302805	45303704	900	SAT1_CF	blue	na
chrUn	45303732	45305690	1959	SAT1_CF	blue	na
chrUn	45305775	45306277	503	SAT1_CF	blue	na
chrUn	45306290	45307510	1221	SAT1_CF	blue	na
chrUn	45307576	45307733	158	SAT1_CF	blue	na
chrUn	45307778	45308272	495	SAT1_CF	blue	na
chrUn	45308292	45308973	682	SAT1_CF	blue	na
chrUn	45308985	45309662	678	SAT1_CF	blue	na
chrUn	45309705	45310539	835	SAT1_CF	blue	na
chrUn	45310589	45311150	562	SAT1_CF	blue	na
chrUn	45312531	45313057	527	SAT1_CF	blue	na
chrUn	45313126	45314158	1033	SAT1_CF	blue	na
chrUn	45314223	45314380	158	SAT1_CF	blue	na
chrUn	45314425	45315015	591	SAT1_CF	blue	na
chrUn	45315034	45316355	1322	SAT1_CF	blue	na
chrUn	45316371	45318130	1760	SAT1_CF	blue	na
chrUn	45318142	45319033	892	SAT1_CF	blue	na
chrUn	45319046	45321195	2150	SAT1_CF	blue	na
chrUn	45321206	45321529	324	SAT1_CF	blue	na
chrUn	45321578	45322080	503	SAT1_CF	blue	na
chrUn	45322092	45322724	633	SAT1_CF	blue	na
chrUn	45322750	45323916	1167	SAT1_CF	blue	na
chrUn	45323931	45324451	521	SAT1_CF	blue	na
chrUn	45324464	45325070	607	SAT1_CF	blue	na
chrUn	45325082	45326291	1210	SAT1_CF	blue	na
chrUn	45326306	45327010	705	SAT1_CF	blue	na

chrUn	45327023	45327704	682	SAT1_CF	blue	na
chrUn	45327716	45328392	677	SAT1_CF	blue	na
chrUn	45328435	45330492	2058	SAT1_CF	blue	na
chrUn	45330534	45332425	1892	SAT1_CF	blue	na
chrUn	45332446	45333091	646	SAT1_CF	blue	na
chrUn	45341114	45341785	672	CarSat1	red	na
chrUn	45341806	45346680	4875	CarSat1	red	na
chrUn	45346712	45347195	484	CarSat1	red	na
chrUn	45347228	45347931	704	CarSat1	red	na
chrUn	45347961	45348888	928	CarSat1	red	na
chrUn	45348913	45350361	1449	CarSat1	red	na
chrUn	45350386	45354049	3664	CarSat1	red	na
chrUn	45354065	45354559	495	CarSat1	red	na
chrUn	45354593	45355515	923	CarSat1	red	na
chrUn	45355540	45356254	715	CarSat1	red	na
chrUn	45356279	45359203	2925	CarSat1	red	na
chrUn	45359228	45359946	719	CarSat1	red	na
chrUn	45359962	45362156	2195	CarSat1	red	na
chrUn	45362172	45362884	713	CarSat1	red	na
chrUn	45362909	45365829	2921	CarSat1	red	na
chrUn	45365854	45366764	911	CarSat1	red	na
chrUn	45368150	45369294	1145	CarSat1	red	na
chrUn	45369324	45370248	925	CarSat1	red	na
chrUn	45370277	45370993	717	CarSat1	red	na
chrUn	45371009	45371721	713	CarSat1	red	na
chrUn	45371746	45372456	711	CarSat1	red	na
chrUn	45372481	45373199	719	CarSat1	red	na
chrUn	45373215	45375783	2569	CarSat1	red	na
chrUn	45375831	45376534	704	CarSat1	red	na
chrUn	45376550	45377517	968	CarSat1	red	na
chrUn	45377538	45378006	469	CarSat1	red	na
chrUn	45378022	45378735	714	CarSat1	red	na
chrUn	45378760	45379988	1229	CarSat1	red	na
chrUn	45380019	45380468	450	CarSat1	red	na
chrUn	45380485	45381853	1369	CarSat1	red	na
chrUn	45382052	45382151	100	CarSat1	red	na
chrUn	45382169	45382765	597	CarSat1	red	na
chrUn	45385293	45385952	660	SAT1_CF	blue	na
chrUn	45385990	45386951	962	SAT1_CF	blue	na
chrUn	45386995	45388669	1675	SAT1_CF	blue	na
chrUn	45388681	45389157	477	SAT1_CF	blue	na
chrUn	45389227	45389899	673	SAT1_CF	blue	na

chrUn	45389911	45390668	758	SAT1_CF	blue	na
chrUn	45390680	45391356	677	SAT1_CF	blue	na
chrUn	45391375	45391874	500	SAT1_CF	blue	na
chrUn	45391914	45392872	959	SAT1_CF	blue	na
chrUn	45392916	45393915	1000	SAT1_CF	blue	na
chrUn	45393958	45394634	677	SAT1_CF	blue	na
chrUn	45394646	45395148	503	SAT1_CF	blue	na
chrUn	45395188	45396931	1744	SAT1_CF	blue	na
chrUn	45396943	45398246	1304	SAT1_CF	blue	na
chrUn	45398261	45399531	1271	SAT1_CF	blue	na
chrUn	45399554	45399659	106	SAT1_CF	blue	na
chrUn	45399672	45400192	521	SAT1_CF	blue	na
chrUn	45400213	45400393	181	SAT1_CF	blue	na
chrUn	45400575	45401346	772	SAT1_CF	blue	na
chrUn	45401359	45402076	718	SAT1_CF	blue	na
chrUn	45402101	45402745	645	SAT1_CF	blue	na
chrUn	45402757	45403434	678	SAT1_CF	blue	na
chrUn	45403446	45404973	1528	SAT1_CF	blue	na
chrUn	45404988	45406061	1074	SAT1_CF	blue	na
chrUn	45406086	45406795	710	SAT1_CF	blue	na
chrUn	45406806	45407933	1128	SAT1_CF	blue	na
chrUn	45407951	45408432	482	SAT1_CF	blue	na
chrUn	45408502	45409013	512	SAT1_CF	blue	na
chrUn	45409233	45409803	571	SAT1_CF	blue	na
chrUn	45409839	45411357	1519	SAT1_CF	blue	na
chrUn	45411369	45412606	1238	SAT1_CF	blue	na
chrUn	45412723	45413075	353	SAT1_CF	blue	na
chrUn	45413088	45413597	510	SAT1_CF	blue	na
chrUn	45413628	45414458	831	SAT1_CF	blue	na
chrUn	45414519	45415195	677	SAT1_CF	blue	na
chrUn	45415207	45415830	624	SAT1_CF	blue	na
chrUn	45415844	45416938	1095	SAT1_CF	blue	na
chrUn	45417035	45417171	137	SAT1_CF	blue	na
chrUn	45417186	45417710	525	SAT1_CF	blue	na
chrUn	45417761	45418168	408	SAT1_CF	blue	na
chrUn	45418252	45418796	545	SAT1_CF	blue	na
chrUn	45418881	45420010	1130	SAT1_CF	blue	na
chrUn	45420668	45421298	631	SAT1_CF	blue	na
chrUn	45421341	45422018	678	SAT1_CF	blue	na
chrUn	45422030	45422737	708	SAT1_CF	blue	na
chrUn	45422758	45422867	110	SAT1_CF	blue	na
chrUn	45422879	45423397	519	SAT1_CF	blue	na

chrUn	45423440	45423785	346	SAT1_CF	blue	na
chrUn	45423829	45425372	1544	SAT1_CF	blue	na
chrUn	45425384	45425942	559	SAT1_CF	blue	na
chrUn	45426007	45426553	547	SAT1_CF	blue	na
chrUn	45426596	45427401	806	SAT1_CF	blue	na
chrUn	45428485	45428885	401	SAT1_CF	blue	na
chrUn	45428897	45430093	1197	SAT1_CF	blue	na
chrUn	45430129	45430314	186	SAT1_CF	blue	na
chrUn	45430495	45432213	1719	SAT1_CF	blue	na
chrUn	45432226	45432727	502	SAT1_CF	blue	na
chrUn	45432767	45433747	981	SAT1_CF	blue	na
chrUn	45433808	45434385	578	SAT1_CF	blue	na
chrUn	45434438	45436444	2007	SAT1_CF	blue	na
chrUn	45436523	45436994	472	SAT1_CF	blue	na
chrUn	45437067	45441521	4455	SAT1_CF	blue	na
chrUn	45441534	45442016	483	SAT1_CF	blue	na
chrUn	45442076	45442478	403	SAT1_CF	blue	na
chrUn	45442491	45444265	1775	SAT1_CF	blue	na
chrUn	45444285	45444759	475	SAT1_CF	blue	na
chrUn	45444822	45445392	571	SAT1_CF	blue	na
chrUn	45445415	45445960	546	SAT1_CF	blue	na
chrUn	45445974	45446856	883	SAT1_CF	blue	na
chrUn	45446900	45448144	1245	SAT1_CF	blue	na
chrUn	45448164	45448272	109	SAT1_CF	blue	na
chrUn	45448285	45448805	521	SAT1_CF	blue	na
chrUn	45448822	45449007	186	SAT1_CF	blue	na
chrUn	45449188	45449353	166	SAT1_CF	blue	na
chrUn	45449395	45450940	1546	SAT1_CF	blue	na
chrUn	45450951	45451963	1013	SAT1_CF	blue	na
chrUn	45452007	45452681	675	SAT1_CF	blue	na
chrUn	45452693	45453376	684	SAT1_CF	blue	na
chrUn	45453390	45453890	501	SAT1_CF	blue	na
chrUn	45453930	45454865	936	SAT1_CF	blue	na
chrUn	45454883	45455233	351	SAT1_CF	blue	na
chrUn	45455245	45455788	544	SAT1_CF	blue	na
chrUn	45455830	45456690	861	SAT1_CF	blue	na
chrUn	45456704	45457385	682	SAT1_CF	blue	na
chrUn	45457397	45457951	555	SAT1_CF	blue	na
chrUn	45457973	45458080	108	SAT1_CF	blue	na
chrUn	45458100	45458611	512	SAT1_CF	blue	na
chrUn	45458630	45458757	128	SAT1_CF	blue	na
chrUn	45458809	45461372	2564	SAT1_CF	blue	na

chrUn	45461385	45462975	1591	SAT1_CF	blue	na
chrUn	45463018	45463691	674	SAT1_CF	blue	na
chrUn	45463705	45464462	758	SAT1_CF	blue	na
chrUn	45464474	45464993	520	SAT1_CF	blue	na
chrUn	45465019	45465175	157	SAT1_CF	blue	na
chrUn	45465226	45467053	1828	SAT1_CF	blue	na
chrUn	45467096	45467764	669	SAT1_CF	blue	na
chrUn	45467782	45468426	645	SAT1_CF	blue	na
chrUn	45468439	45469713	1275	SAT1_CF	blue	na
chrUn	45469758	45469897	140	SAT1_CF	blue	na
chrUn	45657314	45658013	700	Sat2CF	purple	na
chrUn	45657389	45658237	849	Sat2CF	purple	na
chrUn	45657785	45658299	515	Sat2CF	purple	na
chrUn	45745249	45746550	1302	SAT1_CF	blue	na
chrUn	45746576	45746733	158	SAT1_CF	blue	na
chrUn	45746748	45748100	1353	SAT1_CF	blue	na
chrUn	45748162	45748721	560	SAT1_CF	blue	na
chrUn	45748786	45748941	156	SAT1_CF	blue	na
chrUn	45749193	45749679	487	SAT1_CF	blue	na
chrUn	45749730	45750230	501	SAT1_CF	blue	na
chrUn	45750242	45751185	944	SAT1_CF	blue	na
chrUn	45751254	45751931	678	SAT1_CF	blue	na
chrUn	45751943	45752626	684	SAT1_CF	blue	na
chrUn	45752646	45753139	494	SAT1_CF	blue	na
chrUn	45753228	45754197	970	SAT1_CF	blue	na
chrUn	45756455	45757201	747	SAT1_CF	blue	na
chrUn	45757229	45757850	622	SAT1_CF	blue	na
chrUn	45757897	45758571	675	SAT1_CF	blue	na
chrUn	45758583	45759134	552	SAT1_CF	blue	na
chrUn	45759177	45760620	1444	SAT1_CF	blue	na
chrUn	45760674	45761445	772	SAT1_CF	blue	na
chrUn	45761459	45762184	726	SAT1_CF	blue	na
chrUn	45762228	45763017	790	SAT1_CF	blue	na
chrUn	45763059	45764133	1075	SAT1_CF	blue	na
chrUn	45763527	45764776	1250	SAT1_CF	blue	na
chrUn	45764816	45765391	576	SAT1_CF	blue	na
chrUn	45765410	45766814	1405	SAT1_CF	blue	na
chrUn	45766857	45767532	676	SAT1_CF	blue	na
chrUn	45767544	45768225	682	SAT1_CF	blue	na
chrUn	45768238	45768780	543	SAT1_CF	blue	na
chrUn	45768824	45769524	701	SAT1_CF	blue	na
chrUn	45769713	45770713	1001	SAT1_CF	blue	na

chrUn	45770725	45771244	520	SAT1_CF	blue	na
chrUn	45771259	45771339	81	SAT1_CF	blue	na
chrUn	45771352	45772147	796	SAT1_CF	blue	na
chrUn	45772163	45772839	677	SAT1_CF	blue	na
chrUn	45772851	45774680	1830	SAT1_CF	blue	na
chrUn	45774724	45774925	202	SAT1_CF	blue	na
chrUn	45774946	45775615	670	SAT1_CF	blue	na
chrUn	45775633	45776127	495	SAT1_CF	blue	na
chrUn	45776171	45776350	180	SAT1_CF	blue	na
chrUn	45776394	45776904	511	SAT1_CF	blue	na
chrUn	45777022	45778053	1032	SAT1_CF	blue	na
chrUn	45778095	45778953	859	SAT1_CF	blue	na
chrUn	45778965	45779760	796	SAT1_CF	blue	na
chrUn	45779835	45782198	2364	SAT1_CF	blue	na
chrUn	45782210	45783490	1281	SAT1_CF	blue	na
chrUn	45783558	45784841	1284	SAT1_CF	blue	na
chrUn	45784883	45785054	172	SAT1_CF	blue	na
chrUn	45785068	45785610	543	SAT1_CF	blue	na
chrUn	45785630	45785739	110	SAT1_CF	blue	na
chrUn	45785751	45786306	556	SAT1_CF	blue	na
chrUn	45786329	45786437	109	SAT1_CF	blue	na
chrUn	45786449	45786969	521	SAT1_CF	blue	na
chrUn	45786998	45788659	1662	SAT1_CF	blue	na
chrUn	45831635	45832229	595	SAT1_CF	blue	na
chrUn	45832309	45832818	510	SAT1_CF	blue	na
chrUn	45832831	45833186	356	SAT1_CF	blue	na
chrUn	45833273	45833816	544	SAT1_CF	blue	na
chrUn	45833828	45835328	1501	SAT1_CF	blue	na
chrUn	45835372	45835921	550	SAT1_CF	blue	na
chrUn	45835986	45836143	158	SAT1_CF	blue	na
chrUn	45836188	45836766	579	SAT1_CF	blue	na
chrUn	45836829	45837384	556	SAT1_CF	blue	na
chrUn	45837396	45838074	679	SAT1_CF	blue	na
chrUn	45838117	45840404	2288	SAT1_CF	blue	na
chrUn	45840448	45840997	550	SAT1_CF	blue	na
chrUn	45841040	45841668	629	SAT1_CF	blue	na
chrUn	45841695	45842625	931	SAT1_CF	blue	na
chrUn	45842668	45843209	542	SAT1_CF	blue	na
chrUn	45843280	45843831	552	SAT1_CF	blue	na
chrUn	45843876	45847952	4077	SAT1_CF	blue	na
chrUn	45847970	45848823	854	SAT1_CF	blue	na
chrUn	45849141	45849801	661	SAT1_CF	blue	na

chrUn	45849880	45850794	915	SAT1_CF	blue	na
chrUn	45850881	45851382	502	SAT1_CF	blue	na
chrUn	45851401	45851504	104	SAT1_CF	blue	na
chrUn	45851525	45852068	544	SAT1_CF	blue	na
chrUn	45852113	45852933	821	SAT1_CF	blue	na
chrUn	45852964	45853510	547	SAT1_CF	blue	na
chrUn	45853530	45854212	683	SAT1_CF	blue	na
chrUn	45854235	45854901	667	SAT1_CF	blue	na
chrUn	45854944	45855573	630	SAT1_CF	blue	na
chrUn	45855601	45856807	1207	SAT1_CF	blue	na
chrUn	45856822	45856958	137	SAT1_CF	blue	na
chrUn	45857024	45857524	501	SAT1_CF	blue	na
chrUn	45857546	45858178	633	SAT1_CF	blue	na
chrUn	45858191	45859022	832	SAT1_CF	blue	na
chrUn	45859049	45859573	525	SAT1_CF	blue	na
chrUn	45859618	45860910	1293	SAT1_CF	blue	na
chrUn	45860991	45861217	227	SAT1_CF	blue	na
chrUn	45861228	45862006	779	SAT1_CF	blue	na
chrUn	45862037	45862565	529	SAT1_CF	blue	na
chrUn	45862583	45862686	104	SAT1_CF	blue	na
chrUn	45862707	45863280	574	SAT1_CF	blue	na
chrUn	45863292	45863975	684	SAT1_CF	blue	na
chrUn	45863987	45864747	761	SAT1_CF	blue	na
chrUn	45864761	45865465	705	SAT1_CF	blue	na
chrUn	45865508	45866157	650	SAT1_CF	blue	na
chrUn	45866184	45866880	697	SAT1_CF	blue	na
chrUn	45866945	45867102	158	SAT1_CF	blue	na
chrUn	45867147	45867648	502	SAT1_CF	blue	na
chrUn	45867669	45868264	596	SAT1_CF	blue	na
chrUn	45868276	45868955	680	SAT1_CF	blue	na
chrUn	45868998	45869960	963	SAT1_CF	blue	na
chrUn	45870039	45871871	1833	SAT1_CF	blue	na
chrUn	45871883	45872823	941	SAT1_CF	blue	na
chrUn	45915840	45917183	1344	SAT1_CF	blue	na
chrUn	45917375	45918408	1034	SAT1_CF	blue	na
chrUn	45918589	45918723	135	SAT1_CF	blue	na
chrUn	45918810	45919309	500	SAT1_CF	blue	na
chrUn	45919322	45919935	614	SAT1_CF	blue	na
chrUn	45919947	45920657	711	SAT1_CF	blue	na
chrUn	45920679	45920777	99	SAT1_CF	blue	na
chrUn	45920798	45921353	556	SAT1_CF	blue	na
chrUn	45921365	45922227	863	SAT1_CF	blue	na

chrUn	45922280	45923049	770	SAT1_CF	blue	na
chrUn	45923159	45923698	540	SAT1_CF	blue	na
chrUn	45923721	45924116	396	SAT1_CF	blue	na
chrUn	45924156	45924656	501	SAT1_CF	blue	na
chrUn	45924674	45925721	1048	SAT1_CF	blue	na
chrUn	45925742	45926284	543	SAT1_CF	blue	na
chrUn	45926354	45927556	1203	SAT1_CF	blue	na
chrUn	45927600	45927906	307	SAT1_CF	blue	na
chrUn	45927987	45928839	853	SAT1_CF	blue	na
chrUn	45928869	45929827	959	SAT1_CF	blue	na
chrUn	45929877	45930576	700	SAT1_CF	blue	na
chrUn	45930627	45931686	1060	SAT1_CF	blue	na
chrUn	45931756	45932655	900	SAT1_CF	blue	na
chrUn	45932680	45933499	820	SAT1_CF	blue	na
chrUn	45934914	45935487	574	SAT1_CF	blue	na
chrUn	45935508	45936029	522	SAT1_CF	blue	na
chrUn	45936056	45936145	90	SAT1_CF	blue	na
chrUn	45936169	45937388	1220	SAT1_CF	blue	na
chrUn	45937408	45937956	549	SAT1_CF	blue	na
chrUn	45937989	45938387	399	SAT1_CF	blue	na
chrUn	45938411	45938930	520	SAT1_CF	blue	na
chrUn	45938956	45939920	965	SAT1_CF	blue	na
chrUn	45939932	45941143	1212	SAT1_CF	blue	na
chrUn	45941155	45942011	857	SAT1_CF	blue	na
chrUn	45942091	45943685	1595	SAT1_CF	blue	na
chrUn	45943713	45943849	137	SAT1_CF	blue	na
chrUn	45943915	45944415	501	SAT1_CF	blue	na
chrUn	45944437	45945798	1362	SAT1_CF	blue	na
chrUn	45945840	45947377	1538	SAT1_CF	blue	na
chrUn	45947457	45947966	510	SAT1_CF	blue	na
chrUn	45948042	45948613	572	SAT1_CF	blue	na
chrUn	45948626	45949994	1369	SAT1_CF	blue	na
chrUn	45950036	45951021	986	SAT1_CF	blue	na
chrUn	45951047	45952535	1489	SAT1_CF	blue	na
chrUn	45952547	45953232	686	SAT1_CF	blue	na
chrUn	45953250	45953920	671	SAT1_CF	blue	na
chrUn	45953963	45955075	1113	SAT1_CF	blue	na
chrUn	45955118	45955702	585	SAT1_CF	blue	na
chrUn	45955714	45956591	878	SAT1_CF	blue	na
chrUn	46005130	46005834	705	CarSat1	red	na
chrUn	46005864	46007530	1667	CarSat1	red	na
chrUn	46007555	46008781	1227	CarSat1	red	na

chrUn	46008811	46009532	722	CarSat1	red	na
chrUn	46009547	46010253	707	CarSat1	red	na
chrUn	46010846	46012682	1837	CarSat1	red	na
chrUn	46012715	46015643	2929	CarSat1	red	na
chrUn	46015676	46016915	1240	CarSat1	red	na
chrUn	46016930	46017657	728	CarSat1	red	na
chrUn	46017672	46018397	726	CarSat1	red	na
chrUn	46018412	46019137	726	CarSat1	red	na
chrUn	46019152	46019879	728	CarSat1	red	na
chrUn	46019894	46020614	721	CarSat1	red	na
chrUn	46020629	46021339	711	CarSat1	red	na
chrUn	46021369	46023029	1661	CarSat1	red	na
chrUn	46023062	46023564	503	CarSat1	red	na
chrUn	46023579	46024286	708	CarSat1	red	na
chrUn	46024316	46026726	2411	CarSat1	red	na
chrUn	46026759	46027462	704	CarSat1	red	na
chrUn	46028859	46029654	796	CarSat1	red	na
chrUn	46029669	46030396	728	CarSat1	red	na
chrUn	46030411	46031338	928	CarSat1	red	na
chrUn	46031371	46031872	502	CarSat1	red	na
chrUn	46031887	46032610	724	CarSat1	red	na
chrUn	46032625	46033336	712	CarSat1	red	na
chrUn	46033366	46035771	2406	CarSat1	red	na
chrUn	46035804	46036512	709	CarSat1	red	na
chrUn	46036545	46037769	1225	CarSat1	red	na
chrUn	46037799	46038520	722	CarSat1	red	na
chrUn	46038535	46039254	720	CarSat1	red	na
chrUn	46039271	46039993	723	CarSat1	red	na
chrUn	46040008	46042408	2401	CarSat1	red	na
chrUn	46042441	46042947	507	CarSat1	red	na
chrUn	46042962	46044149	1188	CarSat1	red	na
chrUn	46044170	46045367	1198	CarSat1	red	na
chrUn	46045400	46045900	501	CarSat1	red	na
chrUn	46045915	46046636	722	CarSat1	red	na
chrUn	46046651	46047372	722	CarSat1	red	na
chrUn	46047387	46047831	445	CarSat1	red	na
chrUn	46061195	46061836	642	SAT1_CF	blue	na
chrUn	46061857	46062236	380	SAT1_CF	blue	na
chrUn	46062269	46064615	2347	SAT1_CF	blue	na
chrUn	46064627	46065309	683	SAT1_CF	blue	na
chrUn	46065322	46065843	522	SAT1_CF	blue	na
chrUn	46065872	46066046	175	SAT1_CF	blue	na

chrUn	46066059	46067122	1064	SAT1_CF	blue	na
chrUn	46067139	46068398	1260	SAT1_CF	blue	na
chrUn	46068410	46069519	1110	SAT1_CF	blue	na
chrUn	46069620	46070132	513	SAT1_CF	blue	na
chrUn	46070157	46071056	900	SAT1_CF	blue	na
chrUn	46071076	46071757	682	SAT1_CF	blue	na
chrUn	46071770	46072272	503	SAT1_CF	blue	na
chrUn	46072317	46072473	157	SAT1_CF	blue	na
chrUn	46072525	46073697	1173	SAT1_CF	blue	na
chrUn	46073709	46074314	606	SAT1_CF	blue	na
chrUn	46074327	46074848	522	SAT1_CF	blue	na
chrUn	46074877	46075415	539	SAT1_CF	blue	na
chrUn	46075441	46075684	244	SAT1_CF	blue	na
chrUn	46075700	46076244	545	SAT1_CF	blue	na
chrUn	46076286	46077116	831	SAT1_CF	blue	na
chrUn	46077129	46077631	503	SAT1_CF	blue	na
chrUn	46077676	46077833	158	SAT1_CF	blue	na
chrUn	46077898	46079058	1161	SAT1_CF	blue	na
chrUn	46079071	46079521	451	SAT1_CF	blue	na
chrUn	46080932	46083098	2167	SAT1_CF	blue	na
chrUn	46083112	46083605	494	SAT1_CF	blue	na
chrUn	46083616	46084299	684	SAT1_CF	blue	na
chrUn	46084311	46084992	682	SAT1_CF	blue	na
chrUn	46085006	46085507	502	SAT1_CF	blue	na
chrUn	46085604	46086109	506	SAT1_CF	blue	na
chrUn	46086216	46087400	1185	SAT1_CF	blue	na
chrUn	46087454	46088302	849	SAT1_CF	blue	na
chrUn	46088314	46088795	482	SAT1_CF	blue	na
chrUn	46088868	46089355	488	SAT1_CF	blue	na
chrUn	46112471	46113044	574	CarSat1	red	na
chrUn	46113227	46113301	75	CarSat1	red	na
chrUn	46113318	46114041	724	CarSat1	red	na
chrUn	46114073	46114797	725	CarSat1	red	na
chrUn	46114818	46116409	1592	CarSat1	red	na
chrUn	46116430	46117219	790	CarSat1	red	na
chrUn	46117309	46119280	1972	CarSat1	red	na
chrUn	46119282	46119994	713	SAT1_CF	blue	na
chrUn	46120039	46120795	757	SAT1_CF	blue	na
chrUn	46123480	46123710	231	SAT1_CF	blue	na
chrUn	46123779	46125144	1366	SAT1_CF	blue	na
chrUn	46125173	46126402	1230	SAT1_CF	blue	na
chrUn	46126413	46126812	400	SAT1_CF	blue	na

chrUn	46126834	46127356	523	SAT1_CF	blue	na
chrUn	46127368	46128553	1186	SAT1_CF	blue	na
chrUn	46128597	46129126	530	SAT1_CF	blue	na
chrUn	46129146	46129756	611	SAT1_CF	blue	na
chrUn	46129835	46130808	974	SAT1_CF	blue	na
chrUn	46130821	46131162	342	SAT1_CF	blue	na
chrUn	46132180	46132575	396	Sat2CF	purple	na
chrUn	46135041	46135175	135	CarSat1	red	na
chrUn	46135265	46136789	1525	CarSat1	red	na
chrUn	46136816	46137224	409	CarSat1	red	na
chrUn	46145600	46146299	700	CarSat1	red	na
chrUn	46146507	46146632	126	CarSat1	red	na
chrUn	46146658	46147368	711	CarSat1	red	na
chrUn	46147394	46148095	702	CarSat1	red	na
chrUn	46148130	46148839	710	CarSat1	red	na
chrUn	46148865	46151991	3127	CarSat1	red	na
chrUn	46152021	46152514	494	CarSat1	red	na
chrUn	46152539	46153464	926	CarSat1	red	na
chrUn	46153494	46154715	1222	CarSat1	red	na
chrUn	46154740	46155209	470	CarSat1	red	na
chrUn	46155236	46155946	711	CarSat1	red	na
chrUn	46155973	46159631	3659	CarSat1	red	na
chrUn	46159658	46160367	710	CarSat1	red	na
chrUn	46160394	46161105	712	CarSat1	red	na
chrUn	46161132	46162548	1417	CarSat1	red	na
chrUn	46166649	46169835	3187	CarSat1	red	na
chrUn	46169862	46172158	2297	CarSat1	red	na
chrUn	46173127	46173672	546	CarSat1	red	na
chrUn	46173699	46175389	1691	CarSat1	red	na
chrUn	46175414	46177356	1943	CarSat1	red	na
chrUn	46177383	46177851	469	CarSat1	red	na
chrUn	46226857	46228216	1360	CarSat1	red	na
chrUn	46228246	46228953	708	CarSat1	red	na
chrUn	46228983	46229691	709	CarSat1	red	na
chrUn	46229706	46229832	127	Carsat2	orange	na
chrUn	46229721	46230426	706	CarSat1	red	na
chrUn	46230456	46231164	709	CarSat1	red	na
chrUn	46231179	46231305	127	Carsat2	orange	na
chrUn	46231194	46231905	712	CarSat1	red	na
chrUn	46231935	46232643	709	CarSat1	red	na
chrUn	46232658	46232784	127	Carsat2	orange	na
chrUn	46232673	46233897	1225	CarSat1	red	na

chrUn	46233921	46235870	1950	CarSat1	red	na
chrUn	46235891	46237071	1181	CarSat1	red	na
chrUn	46237101	46238329	1229	CarSat1	red	na
chrUn	46238345	46239790	1446	CarSat1	red	na
chrUn	46239814	46240282	469	CarSat1	red	na
chrUn	46240303	46244700	4398	CarSat1	red	na
chrUn	46244727	46245904	1178	CarSat1	red	na
chrUn	46245934	46249183	3250	CarSat1	red	na
chrUn	46251166	46251832	667	CarSat1	red	na
chrUn	46251847	46251970	124	Carsat2	orange	na
chrUn	46251862	46252573	712	CarSat1	red	na
chrUn	46252603	46253307	705	CarSat1	red	na
chrUn	46253322	46253448	127	Carsat2	orange	na
chrUn	46253337	46254045	709	CarSat1	red	na
chrUn	46254075	46255052	978	CarSat1	red	na
chrUn	46255073	46255518	446	CarSat1	red	na
chrUn	46255548	46256256	709	CarSat1	red	na
chrUn	46256271	46256394	124	Carsat2	orange	na
chrUn	46256286	46257999	1714	CarSat1	red	na
chrUn	46258029	46258740	712	CarSat1	red	na
chrUn	46259824	46261370	1547	CarSat1	red	na
chrUn	46261385	46261499	115	Carsat2	orange	na
chrUn	46261400	46261885	486	CarSat1	red	na
chrUn	46261918	46262843	926	CarSat1	red	na
chrUn	46262873	46264314	1442	CarSat1	red	na
chrUn	46264344	46264829	486	CarSat1	red	na
chrUn	46264862	46267999	3138	CarSat1	red	na
chrUn	46268014	46268141	128	Carsat2	orange	na
chrUn	46268029	46268739	711	CarSat1	red	na
chrUn	46268754	46268881	128	Carsat2	orange	na
chrUn	46268769	46269661	893	CarSat1	red	na
chrUn	46318767	46319893	1127	Sat2CF	purple	na
chrUn	46319577	46319946	370	Sat2CF	purple	na
chrUn	46329511	46329673	163	Sat2CF	purple	na
chrUn	46329560	46330543	984	Sat2CF	purple	na
chrUn	46397269	46397680	412	SAT1_CF	blue	na
chrUn	46397693	46398375	683	SAT1_CF	blue	na
chrUn	46398399	46399248	850	SAT1_CF	blue	na
chrUn	46399290	46401531	2242	SAT1_CF	blue	na
chrUn	46401551	46402409	859	SAT1_CF	blue	na
chrUn	46402451	46404295	1845	SAT1_CF	blue	na
chrUn	46404335	46405528	1194	SAT1_CF	blue	na

chrUn	46405539	46406031	493	SAT1_CF	blue	na
chrUn	46406045	46407543	1499	SAT1_CF	blue	na
chrUn	46407554	46408223	670	SAT1_CF	blue	na
chrUn	46408235	46408918	684	SAT1_CF	blue	na
chrUn	46408930	46409567	638	SAT1_CF	blue	na
chrUn	46409610	46410153	544	SAT1_CF	blue	na
chrUn	46410172	46410338	167	SAT1_CF	blue	na
chrUn	46410370	46411183	814	SAT1_CF	blue	na
chrUn	46411226	46413216	1991	SAT1_CF	blue	na
chrUn	46413303	46413926	624	SAT1_CF	blue	na
chrUn	46413947	46414501	555	SAT1_CF	blue	na
chrUn	46414513	46415195	683	SAT1_CF	blue	na
chrUn	46415207	46416092	886	SAT1_CF	blue	na
chrUn	46416114	46416965	852	SAT1_CF	blue	na
chrUn	46416978	46418334	1357	SAT1_CF	blue	na
chrUn	46418348	46418993	646	SAT1_CF	blue	na
chrUn	46419004	46419305	302	SAT1_CF	blue	na
chrUn	46419385	46420120	736	SAT1_CF	blue	na
chrUn	46420146	46421089	944	SAT1_CF	blue	na
chrUn	46421102	46421950	849	SAT1_CF	blue	na
chrUn	46422027	46423075	1049	SAT1_CF	blue	na
chrUn	46423097	46423761	665	SAT1_CF	blue	na
chrUn	46423804	46424895	1092	SAT1_CF	blue	na
chrUn	46424914	46425803	890	SAT1_CF	blue	na
chrUn	46425839	46426340	502	SAT1_CF	blue	na
chrUn	46426353	46427025	673	SAT1_CF	blue	na
chrUn	46427068	46427700	633	SAT1_CF	blue	na
chrUn	46427728	46428290	563	SAT1_CF	blue	na
chrUn	46428333	46428853	521	SAT1_CF	blue	na
chrUn	46428875	46429008	134	SAT1_CF	blue	na
chrUn	46429093	46429710	618	SAT1_CF	blue	na
chrUn	46436519	46437256	738	SAT1_CF	blue	na
chrUn	46437268	46439296	2029	SAT1_CF	blue	na
chrUn	46439340	46439903	564	SAT1_CF	blue	na
chrUn	46439982	46440507	526	SAT1_CF	blue	na
chrUn	46440522	46440679	158	SAT1_CF	blue	na
chrUn	46440724	46441225	502	SAT1_CF	blue	na
chrUn	46441238	46441902	665	SAT1_CF	blue	na
chrUn	46441945	46442577	633	SAT1_CF	blue	na
chrUn	46442605	46443167	563	SAT1_CF	blue	na
chrUn	46443210	46443403	194	SAT1_CF	blue	na
chrUn	46494014	46494426	413	CarSat1	red	na

chrUn	46495394	46496645	1252	CarSat1	red	na
chrUn	46496683	46497565	883	CarSat1	red	na
chrUn	46497560	46499103	1544	SAT1_CF	blue	na
chrUn	46499145	46501285	2141	SAT1_CF	blue	na
chrUn	46501298	46502266	969	SAT1_CF	blue	na
chrUn	46502312	46502834	523	SAT1_CF	blue	na
chrUn	46509086	46511073	1988	SAT1_CF	blue	na
chrUn	46511101	46511613	513	SAT1_CF	blue	na
chrUn	46511642	46513936	2295	SAT1_CF	blue	na
chrUn	46513941	46515121	1181	CarSat1	red	na
chrUn	46515140	46516514	1375	CarSat1	red	na
chrUn	46516673	46517664	992	CarSat1	red	na
chrUn	46532993	46534210	1218	SAT1_CF	blue	na
chrUn	46534225	46534382	158	SAT1_CF	blue	na
chrUn	46534408	46536327	1920	SAT1_CF	blue	na
chrUn	46536355	46537548	1194	SAT1_CF	blue	na
chrUn	46537773	46537930	158	SAT1_CF	blue	na
chrUn	46537995	46538474	480	SAT1_CF	blue	na
chrUn	46538487	46538595	109	SAT1_CF	blue	na
chrUn	46538616	46539247	632	SAT1_CF	blue	na
chrUn	46539259	46539876	618	SAT1_CF	blue	na
chrUn	46541198	46541751	554	SAT1_CF	blue	na
chrUn	46541762	46542164	403	SAT1_CF	blue	na
chrUn	46542204	46543293	1090	SAT1_CF	blue	na
chrUn	46543311	46544636	1326	SAT1_CF	blue	na
chrUn	46544679	46544985	307	SAT1_CF	blue	na
chrUn	46545063	46545571	509	SAT1_CF	blue	na
chrUn	46545654	46546235	582	SAT1_CF	blue	na
chrUn	46546257	46547008	752	SAT1_CF	blue	na
chrUn	46547021	46547636	616	SAT1_CF	blue	na
chrUn	46548034	46548852	819	SAT1_CF	blue	na
chrUn	46548875	46550463	1589	SAT1_CF	blue	na
chrUn	46550507	46552448	1942	SAT1_CF	blue	na
chrUn	46552460	46553220	761	SAT1_CF	blue	na
chrUn	46553234	46553879	646	SAT1_CF	blue	na
chrUn	46553891	46554747	857	SAT1_CF	blue	na
chrUn	46554789	46556270	1482	SAT1_CF	blue	na
chrUn	46556281	46556840	560	SAT1_CF	blue	na
chrUn	46556854	46557978	1125	SAT1_CF	blue	na
chrUn	46558021	46559316	1296	SAT1_CF	blue	na
chrUn	46559359	46559871	513	SAT1_CF	blue	na
chrUn	46559899	46560837	939	SAT1_CF	blue	na

chrUn	46560881	46562507	1627	SAT1_CF	blue	na
chrUn	46562550	46563352	803	SAT1_CF	blue	na
chrUn	46563374	46563472	99	SAT1_CF	blue	na
chrUn	46563494	46565262	1769	SAT1_CF	blue	na
chrUn	46565902	46566525	624	SAT1_CF	blue	na
chrUn	46566537	46568484	1948	SAT1_CF	blue	na
chrUn	46568519	46569042	524	SAT1_CF	blue	na
chrUn	46569057	46569214	158	SAT1_CF	blue	na
chrUn	46569259	46569761	503	SAT1_CF	blue	na
chrUn	46569773	46569882	110	SAT1_CF	blue	na
chrUn	46569903	46571840	1938	SAT1_CF	blue	na
chrUn	46571853	46573536	1684	SAT1_CF	blue	na
chrUn	46640595	46640932	338	SAT1_CF	blue	na
chrUn	46640954	46641757	804	SAT1_CF	blue	na
chrUn	46641769	46642142	374	SAT1_CF	blue	na
chrUn	46642325	46643811	1487	SAT1_CF	blue	na
chrUn	46643848	46644526	679	SAT1_CF	blue	na
chrUn	46644551	46645193	643	SAT1_CF	blue	na
chrUn	46645205	46645879	675	SAT1_CF	blue	na
chrUn	46645901	46646401	501	SAT1_CF	blue	na
chrUn	46646441	46647400	960	SAT1_CF	blue	na
chrUn	46647479	46649160	1682	SAT1_CF	blue	na
chrUn	46649172	46649753	582	SAT1_CF	blue	na
chrUn	46649833	46650443	611	SAT1_CF	blue	na
chrUn	46650498	46651454	957	SAT1_CF	blue	na
chrUn	46651502	46652179	678	SAT1_CF	blue	na
chrUn	46652191	46652745	555	SAT1_CF	blue	na
chrUn	46652767	46652874	108	SAT1_CF	blue	na
chrUn	46652888	46653482	595	SAT1_CF	blue	na
chrUn	46653504	46653611	108	SAT1_CF	blue	na
chrUn	46653625	46654145	521	SAT1_CF	blue	na
chrUn	46654170	46655022	853	SAT1_CF	blue	na
chrUn	46655036	46655809	774	SAT1_CF	blue	na
chrUn	46656398	46656861	464	SAT1_CF	blue	na
chrUn	46656872	46658124	1253	SAT1_CF	blue	na
chrUn	46658143	46658583	441	SAT1_CF	blue	na
chrUn	46658639	46659227	589	SAT1_CF	blue	na
chrUn	46659293	46659429	137	SAT1_CF	blue	na
chrUn	46659449	46659951	503	SAT1_CF	blue	na
chrUn	46660017	46660153	137	SAT1_CF	blue	na
chrUn	46660185	46660916	732	SAT1_CF	blue	na
chrUn	46661157	46661790	634	SAT1_CF	blue	na

chrUn	46661844	46662380	537	SAT1_CF	blue	na
chrUn	46662459	46664370	1912	SAT1_CF	blue	na
chrUn	46666832	46667060	229	SAT1_CF	blue	na
chrUn	46667075	46667600	526	SAT1_CF	blue	na
chrUn	46667643	46668210	568	SAT1_CF	blue	na
chrUn	46668240	46668582	343	SAT1_CF	blue	na
chrUn	46669786	46670245	460	SAT1_CF	blue	na
chrUn	46670263	46671112	850	SAT1_CF	blue	na
chrUn	46671147	46671774	628	SAT1_CF	blue	na
chrUn	46671798	46671904	107	SAT1_CF	blue	na
chrUn	46671916	46672518	603	SAT1_CF	blue	na
chrUn	46673232	46673461	230	SAT1_CF	blue	na
chrUn	46673488	46673968	481	SAT1_CF	blue	na
chrUn	46678245	46679021	777	SAT1_CF	blue	na
chrUn	46679068	46679574	507	SAT1_CF	blue	na
chrUn	46679599	46680654	1056	SAT1_CF	blue	na
chrUn	46680868	46681408	541	SAT1_CF	blue	na
chrUn	46681451	46681989	539	SAT1_CF	blue	na
chrUn	46682002	46682893	892	SAT1_CF	blue	na
chrUn	46682905	46684587	1683	SAT1_CF	blue	na
chrUn	46684603	46685254	652	SAT1_CF	blue	na
chrUn	46686237	46686702	466	SAT1_CF	blue	na
chrUn	46686726	46687290	565	SAT1_CF	blue	na
chrUn	46690188	46691141	954	SAT1_CF	blue	na
chrUn	46691166	46691272	107	SAT1_CF	blue	na
chrUn	46691285	46691648	364	SAT1_CF	blue	na
chrUn	46732284	46733262	979	SAT1_CF	blue	na
chrUn	46733309	46733985	677	SAT1_CF	blue	na
chrUn	46733997	46735396	1400	SAT1_CF	blue	na
chrUn	46735421	46735525	105	SAT1_CF	blue	na
chrUn	46735545	46736020	476	SAT1_CF	blue	na
chrUn	46736099	46736204	106	SAT1_CF	blue	na
chrUn	46736219	46736846	628	SAT1_CF	blue	na
chrUn	46736857	46737488	632	SAT1_CF	blue	na
chrUn	46737510	46737617	108	SAT1_CF	blue	na
chrUn	46737630	46738132	503	SAT1_CF	blue	na
chrUn	46738170	46738574	405	SAT1_CF	blue	na
chrUn	46738624	46739296	673	SAT1_CF	blue	na
chrUn	46739308	46740835	1528	SAT1_CF	blue	na
chrUn	46740850	46741904	1055	SAT1_CF	blue	na
chrUn	46741948	46742646	699	SAT1_CF	blue	na
chrUn	46742663	46743162	500	SAT1_CF	blue	na

chrUn	46743202	46743604	403	SAT1_CF	blue	na
chrUn	46743710	46744487	778	SAT1_CF	blue	na
chrUn	46744509	46745347	839	SAT1_CF	blue	na
chrUn	46745382	46745862	481	SAT1_CF	blue	na
chrUn	46745888	46746949	1062	SAT1_CF	blue	na
chrUn	46746971	46747452	482	SAT1_CF	blue	na
chrUn	46747524	46748430	907	SAT1_CF	blue	na
chrUn	46748546	46749466	921	SAT1_CF	blue	na
chrUn	46749479	46749815	337	SAT1_CF	blue	na
chrUn	46751566	46751668	103	SAT1_CF	blue	na
chrUn	46751717	46752394	678	SAT1_CF	blue	na
chrUn	46752406	46753089	684	SAT1_CF	blue	na
chrUn	46753102	46753604	503	SAT1_CF	blue	na
chrUn	46753691	46754575	885	SAT1_CF	blue	na
chrUn	46754594	46755023	430	SAT1_CF	blue	na
chrUn	46755066	46756399	1334	SAT1_CF	blue	na
chrUn	46756414	46757122	709	SAT1_CF	blue	na
chrUn	46757143	46757252	110	SAT1_CF	blue	na
chrUn	46757264	46757785	522	SAT1_CF	blue	na
chrUn	46757804	46758172	369	SAT1_CF	blue	na
chrUn	46758216	46759756	1541	SAT1_CF	blue	na
chrUn	46759768	46763549	3782	SAT1_CF	blue	na
chrUn	46763575	46764057	483	SAT1_CF	blue	na
chrUn	46764116	46764297	182	SAT1_CF	blue	na
chrUn	46764477	46765270	794	SAT1_CF	blue	na
chrUn	46765314	46766438	1125	SAT1_CF	blue	na
chrUn	46766451	46767291	841	SAT1_CF	blue	na
chrUn	46767304	46767988	685	SAT1_CF	blue	na
chrUn	46768000	46769599	1600	SAT1_CF	blue	na
chrUn	46769642	46770913	1272	SAT1_CF	blue	na
chrUn	46770974	46771650	677	SAT1_CF	blue	na
chrUn	46771662	46772281	620	SAT1_CF	blue	na
chrUn	46817769	46818414	646	SAT1_CF	blue	na
chrUn	46818435	46819763	1329	SAT1_CF	blue	na
chrUn	46819808	46821252	1445	SAT1_CF	blue	na
chrUn	46821264	46822631	1368	SAT1_CF	blue	na
chrUn	46822674	46823350	677	SAT1_CF	blue	na
chrUn	46823362	46824047	686	SAT1_CF	blue	na
chrUn	46824059	46824561	503	SAT1_CF	blue	na
chrUn	46824610	46824933	324	SAT1_CF	blue	na
chrUn	46824944	46826599	1656	SAT1_CF	blue	na
chrUn	46826642	46828013	1372	SAT1_CF	blue	na

chrUn	46828026	46828917	892	SAT1_CF	blue	na
chrUn	46828929	46829528	600	SAT1_CF	blue	na
chrUn	46829548	46831428	1881	SAT1_CF	blue	na
chrUn	46831470	46832118	649	SAT1_CF	blue	na
chrUn	46832131	46832633	503	SAT1_CF	blue	na
chrUn	46832678	46832835	158	SAT1_CF	blue	na
chrUn	46832850	46834523	1674	SAT1_CF	blue	na
chrUn	46835933	46837352	1420	SAT1_CF	blue	na
chrUn	46837395	46838061	667	SAT1_CF	blue	na
chrUn	46838084	46838766	683	SAT1_CF	blue	na
chrUn	46838786	46839260	475	SAT1_CF	blue	na
chrUn	46839320	46839895	576	SAT1_CF	blue	na
chrUn	46841306	46841830	525	SAT1_CF	blue	na
chrUn	46841881	46843493	1613	SAT1_CF	blue	na
chrUn	46843516	46843622	107	SAT1_CF	blue	na
chrUn	46843635	46844135	501	SAT1_CF	blue	na
chrUn	46844189	46844355	167	SAT1_CF	blue	na
chrUn	46844374	46845815	1442	SAT1_CF	blue	na
chrUn	46845857	46846023	167	SAT1_CF	blue	na
chrUn	46846041	46846716	676	SAT1_CF	blue	na
chrUn	46846735	46847417	683	SAT1_CF	blue	na
chrUn	46847428	46848113	686	SAT1_CF	blue	na
chrUn	46848130	46848621	492	SAT1_CF	blue	na
chrUn	46848666	46848823	158	SAT1_CF	blue	na
chrUn	46848875	46849363	489	SAT1_CF	blue	na
chrUn	46849442	46851559	2118	SAT1_CF	blue	na
chrUn	46859077	46859664	588	SAT1_CF	blue	na
chrUn	46859679	46860602	924	SAT1_CF	blue	na
chrUn	46860645	46861365	721	SAT1_CF	blue	na
chrUn	46941041	46941843	803	SAT1_CF	blue	na
chrUn	46941885	46942425	541	SAT1_CF	blue	na
chrUn	46942494	46942942	449	SAT1_CF	blue	na
chrUn	46943029	46943529	501	SAT1_CF	blue	na
chrUn	46943557	46944251	695	SAT1_CF	blue	na
chrUn	46944330	46947167	2838	SAT1_CF	blue	na
chrUn	46947205	46947706	502	SAT1_CF	blue	na
chrUn	46947719	46948321	603	SAT1_CF	blue	na
chrUn	46948347	46948986	640	SAT1_CF	blue	na
chrUn	46949029	46949937	909	SAT1_CF	blue	na
chrUn	46949949	46950055	107	SAT1_CF	blue	na
chrUn	46950079	46950706	628	SAT1_CF	blue	na
chrUn	46950730	46951591	862	SAT1_CF	blue	na

chrUn	46951609	46952295	687	SAT1_CF	blue	na
chrUn	46952307	46954184	1878	SAT1_CF	blue	na
chrUn	46954200	46955088	889	SAT1_CF	blue	na
chrUn	46955205	46955723	519	SAT1_CF	blue	na
chrUn	46955780	46956876	1097	SAT1_CF	blue	na
chrUn	46956894	46957587	694	SAT1_CF	blue	na
chrUn	46957674	46958175	502	SAT1_CF	blue	na
chrUn	46958202	46958948	747	SAT1_CF	blue	na
chrUn	46958961	46959031	71	SAT1_CF	blue	na
chrUn	46959055	46960512	1458	SAT1_CF	blue	na
chrUn	46960580	46960833	254	SAT1_CF	blue	na
chrUn	46960849	46962522	1674	SAT1_CF	blue	na
chrUn	46962567	46963555	989	SAT1_CF	blue	na
chrUn	46963612	46964157	546	SAT1_CF	blue	na
chrUn	46964200	46964568	369	SAT1_CF	blue	na
chrUn	46964608	46965108	501	SAT1_CF	blue	na
chrUn	46965135	46966363	1229	SAT1_CF	blue	na
chrUn	46966375	46967058	684	SAT1_CF	blue	na
chrUn	46967069	46967561	493	SAT1_CF	blue	na
chrUn	46967575	46968788	1214	SAT1_CF	blue	na
chrUn	46968799	46969288	490	SAT1_CF	blue	na
chrUn	46969327	46970149	823	SAT1_CF	blue	na
chrUn	46970164	46970321	158	SAT1_CF	blue	na
chrUn	46970347	46970864	518	SAT1_CF	blue	na
chrUn	46970877	46971576	700	SAT1_CF	blue	na
chrUn	46971588	46972271	684	SAT1_CF	blue	na
chrUn	46972282	46972774	493	SAT1_CF	blue	na
chrUn	46972788	46974001	1214	SAT1_CF	blue	na
chrUn	46974012	46974502	491	SAT1_CF	blue	na
chrUn	46974541	46975363	823	SAT1_CF	blue	na
chrUn	46975378	46975535	158	SAT1_CF	blue	na
chrUn	46975561	46976079	519	SAT1_CF	blue	na
chrUn	46976097	46976693	597	SAT1_CF	blue	na
chrUn	46976705	46978720	2016	SAT1_CF	blue	na
chrUn	46979825	46981323	1499	CarSat1	red	na
chrUn	46981338	46984208	2871	CarSat1	red	na
chrUn	46984359	46986401	2043	CarSat1	red	na
chrUn	46986434	46987634	1201	CarSat1	red	na
chrUn	46987658	46988372	715	CarSat1	red	na
chrUn	46988396	46989435	1040	CarSat1	red	na
chrUn	46989456	46992927	3472	CarSat1	red	na
chrUn	47015411	47017899	2489	CarSat1	red	na

chrUn	47017932	47020116	2185	CarSat1	red	na
chrUn	47020140	47022327	2188	CarSat1	red	na
chrUn	47022351	47023801	1451	CarSat1	red	na
chrUn	47023825	47025048	1224	CarSat1	red	na
chrUn	47025078	47026012	935	CarSat1	red	na
chrUn	47026036	47026521	486	CarSat1	red	na
chrUn	47026551	47027273	723	CarSat1	red	na
chrUn	47027288	47028960	1673	CarSat1	red	na
chrUn	47028984	47029695	712	CarSat1	red	na
chrUn	47029719	47032642	2924	CarSat1	red	na
chrUn	47032666	47033622	957	CarSat1	red	na
chrUn	47033649	47034856	1208	CarSat1	red	na
chrUn	47034872	47035832	961	CarSat1	red	na
chrUn	47035859	47036570	712	CarSat1	red	na
chrUn	47036597	47037576	980	CarSat1	red	na
chrUn	47037606	47038540	935	CarSat1	red	na
chrUn	47038556	47039266	711	CarSat1	red	na
chrUn	47040279	47041618	1340	CarSat1	red	na
chrUn	47046622	47047183	562	CarSat1	red	na
chrUn	47047208	47048206	999	CarSat1	red	na
chrUn	47048605	47049045	441	CarSat1	red	na
chrUn	47049072	47049541	470	CarSat1	red	na
chrUn	47049557	47050283	727	CarSat1	red	na
chrUn	47050299	47051001	703	CarSat1	red	na
chrUn	47051376	47052085	710	CarSat1	red	na
chrUn	47052112	47053296	1185	CarSat1	red	na
chrUn	47054304	47054794	491	CarSat1	red	na
chrUn	47054821	47057569	2749	CarSat1	red	na
chrUn	47057599	47058523	925	CarSat1	red	na
chrUn	47058549	47059043	495	CarSat1	red	na
chrUn	47059073	47060421	1349	CarSat1	red	na
chrUn	47060791	47062236	1446	CarSat1	red	na
chrUn	47062248	47062362	115	CarSat1	red	na
chrUn	47062544	47064412	1869	CarSat1	red	na
chrUn	47064439	47065150	712	CarSat1	red	na
chrUn	47065177	47065646	470	CarSat1	red	na
chrUn	47065662	47066998	1337	CarSat1	red	na
chrUn	47067312	47068129	818	CarSat1	red	na
chrUn	47068156	47070549	2394	CarSat1	red	na
chrUn	47071077	47072341	1265	CarSat1	red	na
chrUn	47072755	47073915	1161	CarSat1	red	na
chrUn	47078718	47080661	1944	CarSat1	red	na

chrUn	47080677	47082506	1830	CarSat1	red	na
chrUn	47083684	47085138	1455	CarSat1	red	na
chrUn	47085737	47087122	1386	CarSat1	red	na
chrUn	47087152	47088256	1105	CarSat1	red	na
chrUn	47089335	47090732	1398	CarSat1	red	na
chrUn	47094294	47095397	1104	CarSat1	red	na
chrUn	47096870	47097383	514	CarSat1	red	na
chrUn	47097410	47097878	469	CarSat1	red	na
chrUn	47097894	47098662	769	CarSat1	red	na
chrUn	47099667	47100726	1060	SAT1_CF	blue	na
chrUn	47100805	47106592	5788	SAT1_CF	blue	na
chrUn	47106634	47106799	166	SAT1_CF	blue	na
chrUn	47106981	47107165	185	SAT1_CF	blue	na
chrUn	47107202	47107702	501	SAT1_CF	blue	na
chrUn	47107720	47107823	104	SAT1_CF	blue	na
chrUn	47107843	47108389	547	SAT1_CF	blue	na
chrUn	47108409	47110217	1809	SAT1_CF	blue	na
chrUn	47110230	47110632	403	SAT1_CF	blue	na
chrUn	47110672	47111174	503	SAT1_CF	blue	na
chrUn	47111187	47114063	2877	SAT1_CF	blue	na
chrUn	47114087	47114177	91	SAT1_CF	blue	na
chrUn	47114204	47114725	522	SAT1_CF	blue	na
chrUn	47114793	47115987	1195	SAT1_CF	blue	na
chrUn	47115999	47116681	683	SAT1_CF	blue	na
chrUn	47116694	47117239	546	SAT1_CF	blue	na
chrUn	47117252	47118265	1014	SAT1_CF	blue	na
chrUn	47118344	47119735	1392	SAT1_CF	blue	na
chrUn	47119753	47120256	504	SAT1_CF	blue	na
chrUn	47120308	47122236	1929	SAT1_CF	blue	na
chrUn	47122248	47123444	1197	SAT1_CF	blue	na
chrUn	47123480	47123667	188	SAT1_CF	blue	na
chrUn	47123849	47124683	835	SAT1_CF	blue	na
chrUn	47124695	47125327	633	SAT1_CF	blue	na
chrUn	47125349	47125456	108	SAT1_CF	blue	na
chrUn	47125470	47125971	502	SAT1_CF	blue	na
chrUn	47126011	47126411	401	SAT1_CF	blue	na
chrUn	47126445	47126711	267	SAT1_CF	blue	na
chrUn	47126791	47127354	564	SAT1_CF	blue	na
chrUn	47127381	47128013	633	SAT1_CF	blue	na
chrUn	47128056	47129505	1450	SAT1_CF	blue	na
chrUn	47129525	47130654	1130	SAT1_CF	blue	na
chrUn	47130675	47132162	1488	SAT1_CF	blue	na

chrUn	47132177	47134202	2026	SAT1_CF	blue	na
chrUn	47134215	47134715	501	SAT1_CF	blue	na
chrUn	47134802	47135361	560	SAT1_CF	blue	na
chrUn	47135389	47137045	1657	SAT1_CF	blue	na
chrUn	47163597	47164384	788	Sat7CF	lime	na
chrUn	47191346	47191761	416	SAT1_CF	blue	na
chrUn	47191773	47192824	1052	SAT1_CF	blue	na
chrUn	47195517	47196245	729	SAT1_CF	blue	na
chrUn	47196312	47196576	265	SAT1_CF	blue	na
chrUn	47196619	47197201	583	SAT1_CF	blue	na
chrUn	47200396	47201449	1054	SAT1_CF	blue	na
chrUn	47203255	47203572	318	SAT1_CF	blue	na
chrUn	47203619	47204163	545	SAT1_CF	blue	na
chrUn	47204177	47204990	814	SAT1_CF	blue	na
chrUn	47205002	47205987	986	SAT1_CF	blue	na
chrUn	47206008	47206966	959	SAT1_CF	blue	na
chrUn	47206991	47207243	253	SAT1_CF	blue	na
chrUn	47208488	47210224	1737	SAT1_CF	blue	na
chrUn	47211822	47212420	599	SAT1_CF	blue	na
chrUn	47212485	47213779	1295	SAT1_CF	blue	na
chrUn	47213821	47214682	862	SAT1_CF	blue	na
chrUn	47216270	47218021	1752	SAT1_CF	blue	na
chrUn	47371174	47371928	755	SAT1_CF	blue	na
chrUn	47372016	47372517	502	SAT1_CF	blue	na
chrUn	47372535	47373085	551	SAT1_CF	blue	na
chrUn	47374865	47375172	308	SAT1_CF	blue	na
chrUn	47375225	47375725	501	SAT1_CF	blue	na
chrUn	47375747	47376102	356	SAT1_CF	blue	na
chrUn	47377642	47378041	400	SAT1_CF	blue	na
chrUn	47378053	47379224	1172	SAT1_CF	blue	na
chrUn	47379238	47380453	1216	SAT1_CF	blue	na
chrUn	47380466	47381034	569	SAT1_CF	blue	na
chrUn	47383180	47383714	535	SAT1_CF	blue	na
chrUn	47383726	47384338	613	SAT1_CF	blue	na
chrUn	47384468	47384980	513	SAT1_CF	blue	na
chrUn	47385008	47385247	240	SAT1_CF	blue	na
chrUn	47385374	47385863	490	SAT1_CF	blue	na
chrUn	47385942	47386450	509	SAT1_CF	blue	na
chrUn	47386464	47386861	398	SAT1_CF	blue	na
chrUn	47386925	47387406	482	SAT1_CF	blue	na
chrUn	47387419	47388100	682	SAT1_CF	blue	na
chrUn	47388112	47388962	851	SAT1_CF	blue	na

chrUn	47389178	47389722	545	SAT1_CF	blue	na
chrUn	47389773	47390175	403	SAT1_CF	blue	na
chrUn	47390214	47391126	913	SAT1_CF	blue	na
chrUn	47391139	47391822	684	SAT1_CF	blue	na
chrUn	47391834	47392201	368	SAT1_CF	blue	na
chrUn	47392513	47392778	266	SAT1_CF	blue	na
chrUn	47392856	47394499	1644	SAT1_CF	blue	na
chrUn	47400496	47401891	1396	SAT1_CF	blue	na
chrUn	47401933	47402458	526	SAT1_CF	blue	na
chrUn	47404370	47404671	302	SAT1_CF	blue	na
chrUn	47404793	47405138	346	SAT1_CF	blue	na
chrUn	47405168	47405722	555	SAT1_CF	blue	na
chrUn	47405733	47407033	1301	SAT1_CF	blue	na
chrUn	47407076	47408038	963	SAT1_CF	blue	na
chrUn	47408117	47408666	550	SAT1_CF	blue	na
chrUn	47408745	47409282	538	SAT1_CF	blue	na
chrUn	47409295	47409797	503	SAT1_CF	blue	na
chrUn	47409810	47410571	762	SAT1_CF	blue	na
chrUn	47410583	47411847	1265	SAT1_CF	blue	na
chrUn	47411897	47412441	545	SAT1_CF	blue	na
chrUn	47412485	47414037	1553	SAT1_CF	blue	na
chrUn	47415133	47416838	1706	SAT1_CF	blue	na
chrUn	47416893	47417842	950	SAT1_CF	blue	na
chrUn	47417861	47418380	520	SAT1_CF	blue	na
chrUn	47418393	47419076	684	SAT1_CF	blue	na
chrUn	47419088	47419197	110	SAT1_CF	blue	na
chrUn	47419218	47419847	630	SAT1_CF	blue	na
chrUn	47419861	47420534	674	SAT1_CF	blue	na
chrUn	47420577	47421118	542	SAT1_CF	blue	na
chrUn	47459974	47460689	716	CarSat1	red	na
chrUn	47460710	47462405	1696	CarSat1	red	na
chrUn	47462430	47462898	469	CarSat1	red	na
chrUn	47462919	47464615	1697	CarSat1	red	na
chrUn	47464649	47465846	1198	CarSat1	red	na
chrUn	47465867	47466583	717	CarSat1	red	na
chrUn	47466604	47468056	1453	CarSat1	red	na
chrUn	47468077	47469032	956	CarSat1	red	na
chrUn	47469057	47470722	1666	CarSat1	red	na
chrUn	47470759	47471244	486	CarSat1	red	na
chrUn	47471278	47471980	703	CarSat1	red	na
chrUn	47472005	47472715	711	CarSat1	red	na
chrUn	47472740	47473461	722	CarSat1	red	na

chrUn	47473486	47474060	575	CarSat1	red	na
chrUn	47475775	47476403	629	CarSat1	red	na
chrUn	47476428	47477512	1085	CarSat1	red	na
chrUn	47502932	47504984	2053	CarSat1	red	na
chrUn	47505011	47508430	3420	CarSat1	red	na
chrUn	47508457	47509418	962	CarSat1	red	na
chrUn	47509434	47511847	2414	CarSat1	red	na
chrUn	47511877	47513321	1445	CarSat1	red	na
chrUn	47513351	47515805	2455	CarSat1	red	na
chrUn	47515827	47517010	1184	CarSat1	red	na
chrUn	47517025	47517152	128	Carsat2	orange	na
chrUn	47517040	47519490	2451	CarSat1	red	na
chrUn	47519511	47522904	3394	CarSat1	red	na
chrUn	47522920	47523046	127	Carsat2	orange	na
chrUn	47522934	47523711	778	CarSat1	red	na
chrUn	47524711	47525197	487	SAT1_CF	blue	na
chrUn	47525209	47526051	843	SAT1_CF	blue	na
chrUn	47528049	47528547	499	SAT1_CF	blue	na
chrUn	47528566	47529118	553	SAT1_CF	blue	na
chrUn	47529161	47529744	584	SAT1_CF	blue	na
chrUn	47531163	47531600	438	SAT1_CF	blue	na
chrUn	47531625	47532502	878	SAT1_CF	blue	na
chrUn	47532515	47533063	549	SAT1_CF	blue	na
chrUn	47533083	47533748	666	SAT1_CF	blue	na
chrUn	47533791	47535413	1623	SAT1_CF	blue	na
chrUn	47535427	47535893	467	SAT1_CF	blue	na
chrUn	47536656	47537210	555	SAT1_CF	blue	na
chrUn	47537223	47537294	72	SAT1_CF	blue	na
chrUn	47537317	47537867	551	SAT1_CF	blue	na
chrUn	47537885	47538424	540	SAT1_CF	blue	na
chrUn	47539925	47540698	774	SAT1_CF	blue	na
chrUn	47540715	47541355	641	SAT1_CF	blue	na
chrUn	47543340	47543960	621	SAT1_CF	blue	na
chrUn	47544002	47544982	981	SAT1_CF	blue	na
chrUn	47545026	47545160	135	SAT1_CF	blue	na
chrUn	47545172	47545712	541	SAT1_CF	blue	na
chrUn	47545724	47546041	318	SAT1_CF	blue	na
chrUn	47546116	47546686	571	SAT1_CF	blue	na
chrUn	47546705	47547257	553	SAT1_CF	blue	na
chrUn	47547306	47547629	324	SAT1_CF	blue	na
chrUn	47547877	47548188	312	SAT1_CF	blue	na
chrUn	47548215	47548962	748	SAT1_CF	blue	na

chrUn	47548982	47549047	66	SAT1_CF	blue	na
chrUn	47549069	47549593	525	SAT1_CF	blue	na
chrUn	47549650	47550762	1113	SAT1_CF	blue	na
chrUn	47553707	47554954	1248	SAT1_CF	blue	na
chrUn	47555721	47555797	77	SAT1_CF	blue	na
chrUn	47555843	47556344	502	SAT1_CF	blue	na
chrUn	47556357	47557935	1579	SAT1_CF	blue	na
chrUn	47558159	47558297	139	SAT1_CF	blue	na
chrUn	47558316	47558821	506	SAT1_CF	blue	na
chrUn	47558864	47559270	407	SAT1_CF	blue	na
chrUn	47561059	47562660	1602	SAT1_CF	blue	na
chrUn	47564360	47564858	499	SAT1_CF	blue	na
chrUn	47564871	47565632	762	SAT1_CF	blue	na
chrUn	47565652	47565717	66	SAT1_CF	blue	na
chrUn	47565739	47566529	791	SAT1_CF	blue	na
chrUn	47568890	47569675	786	SAT1_CF	blue	na
chrUn	47569720	47570884	1165	SAT1_CF	blue	na
chrUn	47571002	47571609	608	SAT1_CF	blue	na
chrUn	47571620	47572097	478	SAT1_CF	blue	na
chrUn	47574369	47574935	567	SAT1_CF	blue	na
chrUn	47574979	47575990	1012	SAT1_CF	blue	na
chrUn	47576149	47576224	76	SAT1_CF	blue	na
chrUn	47576263	47576764	502	SAT1_CF	blue	na
chrUn	47576776	47576885	110	SAT1_CF	blue	na
chrUn	47576906	47578013	1108	SAT1_CF	blue	na
chrUn	47578035	47578403	369	SAT1_CF	blue	na
chrUn	47578429	47580345	1917	SAT1_CF	blue	na
chrUn	47580372	47581163	792	SAT1_CF	blue	na
chrUn	47591134	47591897	764	SAT1_CF	blue	na
chrUn	47591919	47592817	899	SAT1_CF	blue	na
chrUn	47592845	47593352	508	SAT1_CF	blue	na
chrUn	47593396	47593957	562	SAT1_CF	blue	na
chrUn	47594000	47594805	806	SAT1_CF	blue	na
chrUn	47594848	47596299	1452	SAT1_CF	blue	na
chrUn	47596484	47596950	467	SAT1_CF	blue	na
chrUn	47596965	47597122	158	SAT1_CF	blue	na
chrUn	47597167	47597667	501	SAT1_CF	blue	na
chrUn	47597680	47599779	2100	SAT1_CF	blue	na
chrUn	47599842	47600313	472	SAT1_CF	blue	na
chrUn	47600333	47601016	684	SAT1_CF	blue	na
chrUn	47601028	47601704	677	SAT1_CF	blue	na
chrUn	47601747	47602579	833	SAT1_CF	blue	na

chrUn	47602629	47603190	562	SAT1_CF	blue	na
chrUn	47604571	47605097	527	SAT1_CF	blue	na
chrUn	47605166	47606248	1083	SAT1_CF	blue	na
chrUn	47606263	47606420	158	SAT1_CF	blue	na
chrUn	47606446	47607055	610	SAT1_CF	blue	na
chrUn	47607074	47608395	1322	SAT1_CF	blue	na
chrUn	47608422	47610170	1749	SAT1_CF	blue	na
chrUn	47610190	47610863	674	SAT1_CF	blue	na
chrUn	47610943	47611571	629	SAT1_CF	blue	na
chrUn	47611583	47614481	2899	SAT1_CF	blue	na
chrUn	47619979	47620640	662	SAT1_CF	blue	na
chrUn	47620658	47621160	503	SAT1_CF	blue	na
chrUn	47621226	47621361	136	SAT1_CF	blue	na
chrUn	47621389	47622575	1187	SAT1_CF	blue	na
chrUn	47622620	47623952	1333	SAT1_CF	blue	na
chrUn	47623964	47624516	553	SAT1_CF	blue	na
chrUn	47624623	47625385	763	SAT1_CF	blue	na
chrUn	47625397	47625971	575	SAT1_CF	blue	na
chrUn	47625994	47626100	107	SAT1_CF	blue	na
chrUn	47626113	47626657	545	SAT1_CF	blue	na
chrUn	47626734	47628447	1714	SAT1_CF	blue	na
chrUn	47628489	47628663	175	SAT1_CF	blue	na
chrUn	47628675	47629549	875	SAT1_CF	blue	na
chrUn	47629574	47630115	542	SAT1_CF	blue	na
chrUn	47630212	47630889	678	SAT1_CF	blue	na
chrUn	47630901	47631456	556	SAT1_CF	blue	na
chrUn	47631477	47631576	100	SAT1_CF	blue	na
chrUn	47631598	47632173	576	SAT1_CF	blue	na
chrUn	47632501	47633651	1151	SAT1_CF	blue	na
chrUn	47633664	47634144	481	SAT1_CF	blue	na
chrUn	47634217	47635134	918	SAT1_CF	blue	na
chrUn	47635183	47635730	548	SAT1_CF	blue	na
chrUn	47635751	47635860	110	SAT1_CF	blue	na
chrUn	47635872	47636245	374	SAT1_CF	blue	na
chrUn	47636273	47636989	717	SAT1_CF	blue	na
chrUn	47637001	47638651	1651	SAT1_CF	blue	na
chrUn	47638669	47639429	761	SAT1_CF	blue	na
chrUn	47639442	47640449	1008	SAT1_CF	blue	na
chrUn	47640473	47641019	547	SAT1_CF	blue	na
chrUn	47641063	47641212	150	SAT1_CF	blue	na
chrUn	47641397	47642229	833	SAT1_CF	blue	na
chrUn	47642272	47642819	548	SAT1_CF	blue	na

chrUn	47642840	47642949	110	SAT1_CF	blue	na
chrUn	47642961	47643334	374	SAT1_CF	blue	na
chrUn	47643362	47644080	719	SAT1_CF	blue	na
chrUn	47644092	47644852	761	SAT1_CF	blue	na
chrUn	47644866	47645365	500	SAT1_CF	blue	na
chrUn	47645452	47645950	499	SAT1_CF	blue	na
chrUn	47645968	47646526	559	SAT1_CF	blue	na
chrUn	47646548	47646655	108	SAT1_CF	blue	na
chrUn	47646668	47647213	546	SAT1_CF	blue	na
chrUn	47647315	47647803	489	SAT1_CF	blue	na
chrUn	47648022	47648206	185	SAT1_CF	blue	na
chrUn	47648220	47649665	1446	SAT1_CF	blue	na
chrUn	47649707	47649877	171	SAT1_CF	blue	na
chrUn	47649890	47651131	1242	SAT1_CF	blue	na
chrUn	47651153	47651260	108	SAT1_CF	blue	na
chrUn	47651273	47651976	704	SAT1_CF	blue	na
chrUn	47651991	47652515	525	SAT1_CF	blue	na
chrUn	47652558	47653830	1273	SAT1_CF	blue	na
chrUn	47653879	47654424	546	SAT1_CF	blue	na
chrUn	47654437	47654553	117	SAT1_CF	blue	na
chrUn	47654565	47655118	554	SAT1_CF	blue	na
chrUn	47655139	47655236	98	SAT1_CF	blue	na
chrUn	47655259	47655760	502	SAT1_CF	blue	na
chrUn	47655826	47655962	137	SAT1_CF	blue	na
chrUn	47655990	47656538	549	SAT1_CF	blue	na
chrUn	47656568	47656847	280	SAT1_CF	blue	na
chrUn	47657893	47659945	2053	SAT1_CF	blue	na
chrUn	47659958	47660574	617	SAT1_CF	blue	na
chrUn	47660616	47661285	670	SAT1_CF	blue	na
chrUn	47661466	47661862	397	SAT1_CF	blue	na
chrUn	47661895	47662031	137	SAT1_CF	blue	na
chrUn	47662126	47662645	520	SAT1_CF	blue	na
chrUn	47662702	47663249	548	SAT1_CF	blue	na
chrUn	47663291	47664916	1626	SAT1_CF	blue	na
chrUn	47664928	47666169	1242	SAT1_CF	blue	na
chrUn	47666250	47666424	175	SAT1_CF	blue	na
chrUn	47666510	47667005	496	SAT1_CF	blue	na
chrUn	47667025	47667901	877	SAT1_CF	blue	na
chrUn	47667934	47668455	522	SAT1_CF	blue	na
chrUn	47668470	47668626	157	SAT1_CF	blue	na
chrUn	47668652	47669172	521	SAT1_CF	blue	na
chrUn	47669198	47671351	2154	SAT1_CF	blue	na

chrUn	47671375	47671511	137	SAT1_CF	blue	na
chrUn	47671579	47672079	501	SAT1_CF	blue	na
chrUn	47672092	47673463	1372	SAT1_CF	blue	na
chrUn	47673506	47674819	1314	SAT1_CF	blue	na
chrUn	47674868	47676493	1626	SAT1_CF	blue	na
chrUn	47676574	47677683	1110	SAT1_CF	blue	na
chrUn	47677736	47678236	501	SAT1_CF	blue	na
chrUn	47678249	47678932	684	SAT1_CF	blue	na
chrUn	47678944	47679739	796	SAT1_CF	blue	na
chrUn	47679750	47680283	534	SAT1_CF	blue	na
chrUn	47680296	47681781	1486	SAT1_CF	blue	na
chrUn	47681793	47682608	816	SAT1_CF	blue	na
chrUn	47683554	47684049	496	SAT1_CF	blue	na
chrUn	47684070	47684589	520	SAT1_CF	blue	na
chrUn	47684602	47684684	83	SAT1_CF	blue	na
chrUn	47684734	47686018	1285	SAT1_CF	blue	na
chrUn	47686070	47686611	542	SAT1_CF	blue	na
chrUn	47686655	47686961	307	SAT1_CF	blue	na
chrUn	47687041	47687547	507	SAT1_CF	blue	na
chrUn	47687560	47690140	2581	SAT1_CF	blue	na
chrUn	47690182	47690963	782	SAT1_CF	blue	na
chrUn	47691042	47691551	510	SAT1_CF	blue	na
chrUn	47691564	47691919	356	SAT1_CF	blue	na
chrUn	47692006	47692507	502	SAT1_CF	blue	na
chrUn	47692521	47693194	674	SAT1_CF	blue	na
chrUn	47693242	47695181	1940	SAT1_CF	blue	na
chrUn	47696182	47697686	1505	SAT1_CF	blue	na
chrUn	47697714	47697870	157	SAT1_CF	blue	na
chrUn	47697915	47698515	601	SAT1_CF	blue	na
chrUn	47698539	47699083	545	SAT1_CF	blue	na
chrUn	47699127	47699670	544	SAT1_CF	blue	na
chrUn	47699697	47700374	678	SAT1_CF	blue	na
chrUn	47700386	47701236	851	SAT1_CF	blue	na
chrUn	47701249	47701946	698	SAT1_CF	blue	na
chrUn	47701987	47702730	744	SAT1_CF	blue	na
chrUn	47702747	47703095	349	SAT1_CF	blue	na
chrUn	47703211	47703728	518	SAT1_CF	blue	na
chrUn	47703787	47704332	546	SAT1_CF	blue	na
chrUn	47704374	47705100	727	SAT1_CF	blue	na
chrUn	47705145	47705680	536	SAT1_CF	blue	na
chrUn	47705702	47706220	519	SAT1_CF	blue	na
chrUn	47706232	47707549	1318	SAT1_CF	blue	na

chrUn	47707562	47708397	836	SAT1_CF	blue	na
chrUn	47708410	47709170	761	SAT1_CF	blue	na
chrUn	47709183	47710512	1330	SAT1_CF	blue	na
chrUn	47710555	47712181	1627	SAT1_CF	blue	na
chrUn	47712194	47712549	356	SAT1_CF	blue	na
chrUn	47712617	47713135	519	SAT1_CF	blue	na
chrUn	47713153	47713831	679	SAT1_CF	blue	na
chrUn	47713843	47714688	846	SAT1_CF	blue	na
chrUn	47714757	47715270	514	SAT1_CF	blue	na
chrUn	47715308	47716167	860	SAT1_CF	blue	na
chrUn	47716214	47716587	374	SAT1_CF	blue	na
chrUn	47716604	47717120	517	SAT1_CF	blue	na
chrUn	47717133	47717816	684	SAT1_CF	blue	na
chrUn	47717828	47718686	859	SAT1_CF	blue	na
chrUn	47718728	47719516	789	SAT1_CF	blue	na
chrUn	47719696	47719864	169	SAT1_CF	blue	na
chrUn	47719937	47720417	481	SAT1_CF	blue	na
chrUn	47720430	47720535	106	SAT1_CF	blue	na
chrUn	47720558	47722253	1696	SAT1_CF	blue	na
chrUn	47722267	47723338	1072	SAT1_CF	blue	na
chrUn	47723350	47724033	684	SAT1_CF	blue	na
chrUn	47724045	47724973	929	SAT1_CF	blue	na
chrUn	47724986	47725058	73	SAT1_CF	blue	na
chrUn	47725080	47725632	553	SAT1_CF	blue	na
chrUn	47725644	47726890	1247	SAT1_CF	blue	na
chrUn	47726971	47727174	204	SAT1_CF	blue	na
chrUn	47727231	47727732	502	SAT1_CF	blue	na
chrUn	47727745	47728420	676	SAT1_CF	blue	na
chrUn	47728517	47729059	543	SAT1_CF	blue	na
chrUn	47729103	47730068	966	SAT1_CF	blue	na
chrUn	47730083	47730436	354	SAT1_CF	blue	na
chrUn	47730542	47732015	1474	SAT1_CF	blue	na
chrUn	47732062	47732583	522	SAT1_CF	blue	na
chrUn	47733610	47735479	1870	SAT1_CF	blue	na
chrUn	47735529	47736379	851	SAT1_CF	blue	na
chrUn	47736444	47736988	545	SAT1_CF	blue	na
chrUn	47737030	47737808	779	SAT1_CF	blue	na
chrUn	47737820	47739484	1665	SAT1_CF	blue	na
chrUn	47739562	47740069	508	SAT1_CF	blue	na
chrUn	47740083	47740485	403	SAT1_CF	blue	na
chrUn	47740525	47741025	501	SAT1_CF	blue	na
chrUn	47741039	47741146	108	SAT1_CF	blue	na

chrUn	47741168	47741720	553	SAT1_CF	blue	na
chrUn	47741732	47742416	685	SAT1_CF	blue	na
chrUn	47742428	47743103	676	SAT1_CF	blue	na
chrUn	47743148	47743689	542	SAT1_CF	blue	na
chrUn	47743711	47744393	683	SAT1_CF	blue	na
chrUn	47744405	47745884	1480	SAT1_CF	blue	na
chrUn	47745926	47746484	559	SAT1_CF	blue	na
chrUn	47746532	47747074	543	SAT1_CF	blue	na
chrUn	47747088	47748573	1486	SAT1_CF	blue	na
chrUn	47748615	47749112	498	SAT1_CF	blue	na
chrUn	47749130	47749886	757	SAT1_CF	blue	na
chrUn	47749899	47751401	1503	SAT1_CF	blue	na
chrUn	47751437	47751938	502	SAT1_CF	blue	na
chrUn	47751965	47752633	669	SAT1_CF	blue	na
chrUn	47752675	47753526	852	SAT1_CF	blue	na
chrUn	47753596	47755512	1917	SAT1_CF	blue	na
chrUn	47755555	47756043	489	SAT1_CF	blue	na
chrUn	47756095	47756231	137	SAT1_CF	blue	na
chrUn	47756297	47756798	502	SAT1_CF	blue	na
chrUn	47756820	47758864	2045	SAT1_CF	blue	na
chrUn	47758897	47759603	707	SAT1_CF	blue	na
chrUn	47759615	47761211	1597	SAT1_CF	blue	na
chrUn	47761234	47761899	666	SAT1_CF	blue	na
chrUn	47761942	47762898	957	SAT1_CF	blue	na
chrUn	47762977	47763936	960	SAT1_CF	blue	na
chrUn	47763976	47764478	503	SAT1_CF	blue	na
chrUn	47764491	47764597	107	SAT1_CF	blue	na
chrUn	47764620	47765172	553	SAT1_CF	blue	na
chrUn	47765190	47765778	589	SAT1_CF	blue	na
chrUn	47765790	47765873	84	SAT1_CF	blue	na
chrUn	47765886	47767917	2032	SAT1_CF	blue	na
chrUn	47767929	47768598	670	SAT1_CF	blue	na
chrUn	47768640	47769271	632	SAT1_CF	blue	na
chrUn	47769299	47769864	566	SAT1_CF	blue	na
chrUn	47809873	47811022	1150	Sat2CF	purple	na
chrUn	47831533	47832248	716	Sat2CF	purple	na
chrUn	47851742	47852506	765	SAT1_CF	blue	na
chrUn	47852549	47853637	1089	SAT1_CF	blue	na
chrUn	47853628	47854864	1237	CarSat1	red	na
chrUn	47854888	47856479	1592	CarSat1	red	na
chrUn	47856500	47857286	787	CarSat1	red	na
chrUn	47857328	47858031	704	CarSat1	red	na

chrUn	47858049	47858766	718	CarSat1	red	na
chrUn	47858789	47859565	777	CarSat1	red	na
chrUn	47859586	47861045	1460	CarSat1	red	na
chrUn	47861246	47861888	643	CarSat1	red	na
chrUn	47884751	47885692	942	Sat2CF	purple	na
chrUn	47884768	47885876	1109	Sat2CF	purple	na
chrUn	47887552	47887966	415	CarSat1	red	na
chrUn	47888938	47889789	852	CarSat1	red	na
chrUn	47889810	47889952	143	CarSat1	red	na
chrUn	47978447	47978846	400	SAT1_CF	blue	na
chrUn	47978886	47979388	503	SAT1_CF	blue	na
chrUn	47979401	47980162	762	SAT1_CF	blue	na
chrUn	47980174	47981437	1264	SAT1_CF	blue	na
chrUn	47981450	47981995	546	SAT1_CF	blue	na
chrUn	47982033	47982380	348	SAT1_CF	blue	na
chrUn	47982422	47983007	586	SAT1_CF	blue	na
chrUn	47983019	47984357	1339	SAT1_CF	blue	na
chrUn	47984370	47984872	503	SAT1_CF	blue	na
chrUn	47984884	47985567	684	SAT1_CF	blue	na
chrUn	47985579	47986256	678	SAT1_CF	blue	na
chrUn	47986353	47986893	541	SAT1_CF	blue	na
chrUn	47986918	47987795	878	SAT1_CF	blue	na
chrUn	47987843	47988813	971	SAT1_CF	blue	na
chrUn	47988892	47989436	545	SAT1_CF	blue	na
chrUn	47989525	47990357	833	SAT1_CF	blue	na
chrUn	47990370	47990476	107	SAT1_CF	blue	na
chrUn	47990499	47991475	977	SAT1_CF	blue	na
chrUn	47991554	47992548	995	SAT1_CF	blue	na
chrUn	47992587	47993088	502	SAT1_CF	blue	na
chrUn	47993101	47994455	1355	SAT1_CF	blue	na
chrUn	47994497	47995035	539	SAT1_CF	blue	na
chrUn	47995081	47996919	1839	SAT1_CF	blue	na
chrUn	47996937	47997044	108	SAT1_CF	blue	na
chrUn	47997061	47997496	436	SAT1_CF	blue	na
chrUn	47997510	47997617	108	SAT1_CF	blue	na
chrUn	47997639	47997919	281	SAT1_CF	blue	na
chrUn	47998102	47998381	280	SAT1_CF	blue	na
chrUn	47998424	47999414	991	SAT1_CF	blue	na
chrUn	47999481	48000246	766	SAT1_CF	blue	na
chrUn	48000293	48000814	522	SAT1_CF	blue	na
chrUn	48000825	48001500	676	SAT1_CF	blue	na
chrUn	48001543	48003287	1745	SAT1_CF	blue	na

chrUn	48003311	48004513	1203	SAT1_CF	blue	na
chrUn	48004556	48005867	1312	SAT1_CF	blue	na
chrUn	48005893	48006381	489	SAT1_CF	blue	na
chrUn	48006432	48007132	701	SAT1_CF	blue	na
chrUn	48007145	48008520	1376	SAT1_CF	blue	na
chrUn	48008534	48008647	114	SAT1_CF	blue	na
chrUn	48008664	48009393	730	SAT1_CF	blue	na
chrUn	48009435	48010134	700	SAT1_CF	blue	na
chrUn	48010148	48010734	587	SAT1_CF	blue	na
chrUn	48012122	48013129	1008	SAT1_CF	blue	na
chrUn	48013152	48014124	973	SAT1_CF	blue	na
chrUn	48015142	48021983	6842	CarSat1	red	na
chrUn	48022976	48023266	291	Carsat2	orange	na
chrUn	48023008	48024106	1099	CarSat1	red	na
chrUn	48024136	48025577	1442	CarSat1	red	na
chrUn	48025605	48026089	485	CarSat1	red	na
chrUn	48026113	48028300	2188	CarSat1	red	na
chrUn	48028324	48029410	1087	CarSat1	red	na
chrUn	48042458	48045073	2616	CarSat1	red	na
chrUn	48045097	48046037	941	CarSat1	red	na
chrUn	48046067	48046650	584	CarSat1	red	na
chrUn	48048078	48048980	903	CarSat1	red	na
chrUn	48049010	48050234	1225	CarSat1	red	na
chrUn	48050267	48053176	2910	CarSat1	red	na
chrUn	48053200	48055598	2399	CarSat1	red	na
chrUn	48055619	48055746	128	Carsat2	orange	na
chrUn	48055634	48056345	712	CarSat1	red	na
chrUn	48056360	48056486	127	Carsat2	orange	na
chrUn	48056375	48057080	706	CarSat1	red	na
chrUn	48057095	48057222	128	Carsat2	orange	na
chrUn	48057110	48058554	1445	CarSat1	red	na
chrUn	48058569	48058696	128	Carsat2	orange	na
chrUn	48058584	48059290	707	CarSat1	red	na
chrUn	48059305	48059431	127	Carsat2	orange	na
chrUn	48059320	48060027	708	CarSat1	red	na
chrUn	48060042	48060169	128	Carsat2	orange	na
chrUn	48060057	48060764	708	CarSat1	red	na
chrUn	48060794	48061501	708	CarSat1	red	na
chrUn	48061516	48061643	128	Carsat2	orange	na
chrUn	48061531	48063711	2181	CarSat1	red	na
chrUn	48063741	48066282	2542	CarSat1	red	na
chrUn	48077047	48078079	1033	Sat2CF	purple	na

chrUn	48077165	48078028	864	Sat2CF	purple	na
chrUn	48103807	48105096	1290	SAT1_CF	blue	na
chrUn	48105139	48105462	324	SAT1_CF	blue	na
chrUn	48105492	48106005	514	SAT1_CF	blue	na
chrUn	48106039	48106666	628	SAT1_CF	blue	na
chrUn	48106708	48107707	1000	SAT1_CF	blue	na
chrUn	48107751	48108668	918	SAT1_CF	blue	na
chrUn	48108711	48109212	502	SAT1_CF	blue	na
chrUn	48109229	48109907	679	SAT1_CF	blue	na
chrUn	48109920	48110029	110	SAT1_CF	blue	na
chrUn	48110049	48112568	2520	SAT1_CF	blue	na
chrUn	48112611	48113157	547	SAT1_CF	blue	na
chrUn	48113201	48114087	887	SAT1_CF	blue	na
chrUn	48114106	48114658	553	SAT1_CF	blue	na
chrUn	48114701	48116702	2002	SAT1_CF	blue	na
chrUn	48116742	48117243	502	SAT1_CF	blue	na
chrUn	48117256	48117940	685	SAT1_CF	blue	na
chrUn	48117951	48119395	1445	SAT1_CF	blue	na
chrUn	48119411	48119572	162	SAT1_CF	blue	na
chrUn	48119586	48120535	950	SAT1_CF	blue	na
chrUn	48120547	48121437	891	SAT1_CF	blue	na
chrUn	48121450	48122741	1292	SAT1_CF	blue	na
chrUn	48122783	48124526	1744	SAT1_CF	blue	na
chrUn	48124564	48126963	2400	SAT1_CF	blue	na
chrUn	48126983	48127183	201	SAT1_CF	blue	na
chrUn	48127196	48128737	1542	SAT1_CF	blue	na
chrUn	48128779	48131421	2643	SAT1_CF	blue	na
chrUn	48131435	48132124	690	SAT1_CF	blue	na
chrUn	48132136	48132816	681	SAT1_CF	blue	na
chrUn	48132865	48134470	1606	SAT1_CF	blue	na
chrUn	48134514	48135312	799	SAT1_CF	blue	na
chrUn	48135325	48136006	682	SAT1_CF	blue	na
chrUn	48136024	48136729	706	SAT1_CF	blue	na
chrUn	48136762	48137326	565	SAT1_CF	blue	na
chrUn	48137387	48137868	482	SAT1_CF	blue	na
chrUn	48137881	48138682	802	SAT1_CF	blue	na
chrUn	48138710	48139332	623	SAT1_CF	blue	na
chrUn	48252182	48252513	332	Sat2CF	purple	na
chrUn	48252213	48252387	175	Sat2CF	purple	na
chrUn	48252223	48252707	485	Sat2CF	purple	na
chrUn	48267670	48268468	799	Sat2CF	purple	na
chrUn	48268350	48268518	169	Sat2CF	purple	na

chrUn	48336757	48337131	375	SAT1_CF	blue	na
chrUn	48337171	48337670	500	SAT1_CF	blue	na
chrUn	48337683	48338443	761	SAT1_CF	blue	na
chrUn	48338456	48338527	72	SAT1_CF	blue	na
chrUn	48338550	48339100	551	SAT1_CF	blue	na
chrUn	48339118	48340334	1217	SAT1_CF	blue	na
chrUn	48340367	48340523	157	SAT1_CF	blue	na
chrUn	48340568	48341070	503	SAT1_CF	blue	na
chrUn	48341083	48342222	1140	SAT1_CF	blue	na
chrUn	48342264	48343045	782	SAT1_CF	blue	na
chrUn	48343088	48344293	1206	SAT1_CF	blue	na
chrUn	48344318	48344678	361	SAT1_CF	blue	na
chrUn	48344757	48346081	1325	SAT1_CF	blue	na
chrUn	48346093	48347424	1332	SAT1_CF	blue	na
chrUn	48347468	48347774	307	SAT1_CF	blue	na
chrUn	48347840	48348775	936	SAT1_CF	blue	na
chrUn	48348796	48349316	521	SAT1_CF	blue	na
chrUn	48349328	48350003	676	SAT1_CF	blue	na
chrUn	48350045	48351137	1093	SAT1_CF	blue	na
chrUn	48351149	48351752	604	SAT1_CF	blue	na
chrUn	48351779	48352366	588	SAT1_CF	blue	na
chrUn	48352393	48353064	672	SAT1_CF	blue	na
chrUn	48353108	48354451	1344	SAT1_CF	blue	na
chrUn	48354511	48355442	932	SAT1_CF	blue	na
chrUn	48355454	48356129	676	SAT1_CF	blue	na
chrUn	48356177	48357134	958	SAT1_CF	blue	na
chrUn	48357213	48358126	914	SAT1_CF	blue	na
chrUn	48358211	48358694	484	SAT1_CF	blue	na
chrUn	48358240	48359562	1323	SAT1_CF	blue	na
chrUn	48359575	48360249	675	SAT1_CF	blue	na
chrUn	48360261	48360905	645	SAT1_CF	blue	na
chrUn	48360949	48361415	467	SAT1_CF	blue	na
chrUn	48361431	48361950	520	SAT1_CF	blue	na
chrUn	48361980	48362645	666	SAT1_CF	blue	na
chrUn	48362665	48363516	852	SAT1_CF	blue	na
chrUn	48363558	48364354	797	SAT1_CF	blue	na
chrUn	48364398	48364942	545	SAT1_CF	blue	na
chrUn	48364955	48365357	403	SAT1_CF	blue	na
chrUn	48365397	48366174	778	SAT1_CF	blue	na
chrUn	48366202	48367786	1585	SAT1_CF	blue	na
chrUn	48367838	48368396	559	SAT1_CF	blue	na
chrUn	48368408	48369740	1333	SAT1_CF	blue	na

chrUn	48369765	48370090	326	SAT1_CF	blue	na
chrUn	48370134	48371247	1114	SAT1_CF	blue	na
chrUn	48371276	48371797	522	SAT1_CF	blue	na
chrUn	48371815	48372097	283	SAT1_CF	blue	na
chrUn	48400839	48400988	150	Sat2CF	purple	na
chrUn	48400864	48401950	1087	Sat2CF	purple	na
chrUn	48400949	48402084	1136	Sat2CF	purple	na
chrUn	48409401	48409936	536	SAT1_CF	blue	na
chrUn	48410004	48410525	522	SAT1_CF	blue	na
chrUn	48410538	48410641	104	SAT1_CF	blue	na
chrUn	48410665	48412898	2234	SAT1_CF	blue	na
chrUn	48412914	48413602	689	SAT1_CF	blue	na
chrUn	48413623	48414154	532	SAT1_CF	blue	na
chrUn	48415816	48416668	853	SAT1_CF	blue	na
chrUn	48416680	48417223	544	SAT1_CF	blue	na
chrUn	48417291	48417811	521	SAT1_CF	blue	na
chrUn	48417823	48419154	1332	SAT1_CF	blue	na
chrUn	48419198	48420154	957	SAT1_CF	blue	na
chrUn	48420234	48420815	582	SAT1_CF	blue	na
chrUn	48420827	48422014	1188	SAT1_CF	blue	na
chrUn	48422041	48422544	504	SAT1_CF	blue	na
chrUn	48422564	48423570	1007	SAT1_CF	blue	na
chrUn	48423623	48424124	502	SAT1_CF	blue	na
chrUn	48424137	48425690	1554	SAT1_CF	blue	na
chrUn	48425732	48426275	544	SAT1_CF	blue	na
chrUn	48426297	48427087	791	SAT1_CF	blue	na
chrUn	48427130	48427640	511	SAT1_CF	blue	na
chrUn	48427667	48427800	134	SAT1_CF	blue	na
chrUn	48427858	48428359	502	SAT1_CF	blue	na
chrUn	48428372	48429739	1368	SAT1_CF	blue	na
chrUn	48429781	48430410	630	SAT1_CF	blue	na
chrUn	48430438	48433657	3220	SAT1_CF	blue	na
chrUn	48433677	48434160	484	SAT1_CF	blue	na
chrUn	48434172	48436926	2755	SAT1_CF	blue	na
chrUn	48436961	48437462	502	SAT1_CF	blue	na
chrUn	48437484	48438158	675	SAT1_CF	blue	na
chrUn	48438170	48439062	893	SAT1_CF	blue	na
chrUn	48439131	48439676	546	SAT1_CF	blue	na
chrUn	48439718	48440907	1190	SAT1_CF	blue	na
chrUn	48440953	48442270	1318	SAT1_CF	blue	na
chrUn	48442292	48443918	1627	SAT1_CF	blue	na
chrUn	48443950	48444639	690	SAT1_CF	blue	na

chrUn	48444686	48445199	514	SAT1_CF	blue	na
chrUn	48445221	48446007	787	SAT1_CF	blue	na
chrUn	48484229	48484899	671	CarSat1	red	na
chrUn	48484920	48486126	1207	CarSat1	red	na
chrUn	48486151	48489070	2920	CarSat1	red	na
chrUn	48489095	48490314	1220	CarSat1	red	na
chrUn	48490344	48490784	441	CarSat1	red	na
chrUn	48490811	48491526	716	CarSat1	red	na
chrUn	48491547	48494223	2677	CarSat1	red	na
chrUn	48494248	48496433	2186	CarSat1	red	na
chrUn	48496458	48497410	953	CarSat1	red	na
chrUn	48497435	48498152	718	CarSat1	red	na
chrUn	48498178	48499365	1188	CarSat1	red	na
chrUn	48501619	48502171	553	CarSat1	red	na
chrUn	48502200	48502911	712	CarSat1	red	na
chrUn	48502938	48503917	980	CarSat1	red	na
chrUn	48503947	48504655	709	CarSat1	red	na
chrUn	48504685	48505125	441	CarSat1	red	na
chrUn	48505152	48505867	716	CarSat1	red	na
chrUn	48505888	48506598	711	CarSat1	red	na
chrUn	48506625	48507085	461	CarSat1	red	na
chrUn	48507110	48508809	1700	CarSat1	red	na
chrUn	48508834	48509584	751	CarSat1	red	na
chrUn	48509928	48511289	1362	CarSat1	red	na
chrUn	48511317	48512253	937	CarSat1	red	na
chrUn	48512269	48513231	963	CarSat1	red	na
chrUn	48513258	48516447	3190	CarSat1	red	na
chrUn	48516477	48516917	441	CarSat1	red	na
chrUn	48516944	48519624	2681	CarSat1	red	na
chrUn	48519640	48520352	713	CarSat1	red	na
chrUn	48520377	48520885	509	CarSat1	red	na
chrUn	48520900	48521382	483	CarSat1	red	na
chrUn	48522452	48523069	618	CarSat1	red	na
chrUn	48523096	48524167	1072	CarSat1	red	na
chrUn	48524181	48524970	790	CarSat1	red	na
chrUn	48524986	48525561	576	CarSat1	red	na
chrUn	48526407	48527283	877	CarSat1	red	na
chrUn	48527310	48528019	710	CarSat1	red	na
chrUn	48532974	48534039	1066	CarSat1	red	na
chrUn	48536980	48538764	1785	CarSat1	red	na
chrUn	48543488	48544138	651	CarSat1	red	na
chrUn	48544154	48545116	963	CarSat1	red	na

chrUn	48545143	48546120	978	CarSat1	red	na
chrUn	48546266	48547272	1007	CarSat1	red	na
chrUn	48547299	48547847	549	CarSat1	red	na
chrUn	48548477	48549571	1095	CarSat1	red	na
chrUn	48549598	48550489	892	CarSat1	red	na
chrUn	48551643	48552649	1007	CarSat1	red	na
chrUn	48552676	48553387	712	CarSat1	red	na
chrUn	48553414	48554124	711	CarSat1	red	na
chrUn	48554151	48554674	524	CarSat1	red	na
chrUn	48555614	48557665	2052	CarSat1	red	na
chrUn	48557681	48558355	675	CarSat1	red	na
chrUn	48559977	48560940	964	CarSat1	red	na
chrUn	48563799	48564863	1065	CarSat1	red	na
chrUn	48565706	48566490	785	CarSat1	red	na
chrUn	48566517	48567103	587	CarSat1	red	na
chrUn	48567374	48567974	601	CarSat1	red	na
chrUn	48567991	48568919	929	CarSat1	red	na
chrUn	48568946	48569415	470	CarSat1	red	na
chrUn	48569431	48570007	577	CarSat1	red	na
chrUn	48571211	48572041	831	CarSat1	red	na
chrUn	48572068	48572779	712	CarSat1	red	na
chrUn	48572806	48574000	1195	CarSat1	red	na
chrUn	48574027	48575457	1431	CarSat1	red	na
chrUn	48575474	48576194	721	CarSat1	red	na
chrUn	48576211	48577522	1312	CarSat1	red	na
chrUn	48577549	48578259	711	CarSat1	red	na
chrUn	48578286	48578995	710	CarSat1	red	na
chrUn	48580162	48581202	1041	CarSat1	red	na
chrUn	48586498	48587376	879	CarSat1	red	na
chrUn	48587404	48588252	849	CarSat1	red	na
chrUn	48588274	48588619	346	CarSat1	red	na
chrUn	48589501	48590964	1464	CarSat1	red	na
chrUn	48593915	48594999	1085	Sat2CF	purple	na
chrUn	48594000	48595133	1134	Sat2CF	purple	na
chrUn	48607686	48608231	546	Sat2CF	purple	na
chrUn	48607761	48608608	848	Sat2CF	purple	na
chrUn	48607783	48608506	724	Sat2CF	purple	na
chrUn	48623345	48624527	1183	Sat2CF	purple	na
chrUn	48627092	48628219	1128	CarSat1	red	na
chrUn	48628237	48628693	457	CarSat1	red	na
chrUn	48630727	48632508	1782	SAT1_CF	blue	na
chrUn	48632542	48633670	1129	SAT1_CF	blue	na

chrUn	48778129	48778721	593	SAT1_CF	blue	na
chrUn	48778767	48779557	791	SAT1_CF	blue	na
chrUn	48779612	48780458	847	SAT1_CF	blue	na
chrUn	48780470	48781138	669	SAT1_CF	blue	na
chrUn	48781165	48781666	502	SAT1_CF	blue	na
chrUn	48781753	48782939	1187	SAT1_CF	blue	na
chrUn	48782981	48783598	618	SAT1_CF	blue	na
chrUn	48783611	48784364	754	SAT1_CF	blue	na
chrUn	48784810	48786307	1498	SAT1_CF	blue	na
chrUn	48786319	48786820	502	SAT1_CF	blue	na
chrUn	48786860	48789413	2554	SAT1_CF	blue	na
chrUn	48789439	48790124	686	SAT1_CF	blue	na
chrUn	48790627	48791089	463	SAT1_CF	blue	na
chrUn	48791102	48791862	761	SAT1_CF	blue	na
chrUn	48791875	48792734	860	SAT1_CF	blue	na
chrUn	48792747	48793248	502	SAT1_CF	blue	na
chrUn	48793336	48793687	352	SAT1_CF	blue	na
chrUn	48793699	48796566	2868	SAT1_CF	blue	na
chrUn	48796568	48798537	1970	CarSat1	red	na
chrUn	48798559	48799262	704	CarSat1	red	na
chrUn	48838328	48840011	1684	SAT1_CF	blue	na
chrUn	48840022	48840408	387	SAT1_CF	blue	na
chrUn	48840411	48841851	1441	CarSat1	red	na
chrUn	48841868	48843007	1140	CarSat1	red	na
chrUn	48843219	48844004	786	CarSat1	red	na
chrUn	48844025	48844880	856	CarSat1	red	na
chrUn	48844984	48845633	650	CarSat1	red	na
chrUn	48845846	48846194	349	CarSat1	red	na
chrUn	48846377	48846536	160	CarSat1	red	na
chrUn	48846552	48846846	295	CarSat1	red	na
chrUn	48846873	48847595	723	CarSat1	red	na
chrUn	48847744	48848726	983	CarSat1	red	na
chrUn	48888200	48888994	795	Sat2CF	purple	na
chrUn	48888739	48889037	299	Sat2CF	purple	na
chrUn	48890714	48891124	411	CarSat1	red	na
chrUn	48891931	48892073	143	CarSat1	red	na
chrUn	48892094	48892803	710	CarSat1	red	na
chrUn	48892968	48893767	800	CarSat1	red	na
chrUn	48893791	48894559	769	CarSat1	red	na
chrUn	48894580	48895423	844	CarSat1	red	na
chrUn	48895453	48896639	1187	CarSat1	red	na
chrUn	48896630	48897348	719	SAT1_CF	blue	na

chrUn	48897428	48897622	195	SAT1_CF	blue	na
chrUn	48897650	48898381	732	SAT1_CF	blue	na
chrUn	48898423	48900287	1865	SAT1_CF	blue	na
chrUn	48900311	48900853	543	SAT1_CF	blue	na
chrUn	48900866	48901549	684	SAT1_CF	blue	na
chrUn	48901562	48903059	1498	SAT1_CF	blue	na
chrUn	48903102	48904174	1073	SAT1_CF	blue	na
chrUn	48904206	48905147	942	SAT1_CF	blue	na
chrUn	48905187	48905688	502	SAT1_CF	blue	na
chrUn	48905701	48906384	684	SAT1_CF	blue	na
chrUn	48906397	48907205	809	SAT1_CF	blue	na
chrUn	48907219	48908336	1118	SAT1_CF	blue	na
chrUn	48908376	48909282	907	SAT1_CF	blue	na
chrUn	48950615	48951734	1120	CarSat1	red	na
chrUn	48959858	48960840	983	CarSat1	red	na
chrUn	48960867	48961718	852	CarSat1	red	na
chrUn	48961739	48962593	855	CarSat1	red	na
chrUn	48962783	48963463	681	CarSat1	red	na
chrUn	48963663	48963939	277	CarSat1	red	na
chrUn	48964130	48964534	405	CarSat1	red	na
chrUn	48964685	48965408	724	CarSat1	red	na
chrUn	48965427	48966279	853	CarSat1	red	na
chrUn	48966429	48967078	650	CarSat1	red	na
chrUn	48967092	48967567	476	CarSat1	red	na
chrUn	48967584	48968278	695	CarSat1	red	na
chrUn	48968283	48968668	386	SAT1_CF	blue	na
chrUn	48968680	48971285	2606	SAT1_CF	blue	na
chrUn	48971458	48975558	4101	SAT1_CF	blue	na
chrUn	48975595	48976157	563	SAT1_CF	blue	na
chrUn	48976201	48976748	548	SAT1_CF	blue	na
chrUn	48976839	48977719	881	SAT1_CF	blue	na
chrUn	48977712	48978439	728	CarSat1	red	na
chrUn	48978457	48980427	1971	CarSat1	red	na
chrUn	48980441	48980977	537	SAT1_CF	blue	na
chrUn	48981003	48981530	528	SAT1_CF	blue	na
chrUn	48981542	48982339	798	SAT1_CF	blue	na
chrUn	48982350	48984379	2030	SAT1_CF	blue	na
chrUn	48984399	48985550	1152	SAT1_CF	blue	na
chrUn	48985545	48986264	720	CarSat1	red	na
chrUn	48986283	48986706	424	CarSat1	red	na
chrUn	48987742	48988062	321	SAT1_CF	blue	na
chrUn	48988075	48988576	502	SAT1_CF	blue	na

chrUn	48988590	48989832	1243	SAT1_CF	blue	na
chrUn	48989885	48989961	77	SAT1_CF	blue	na
chrUn	48989974	48991037	1064	SAT1_CF	blue	na
chrUn	48991051	48991928	878	SAT1_CF	blue	na
chrUn	48991971	48992648	678	SAT1_CF	blue	na
chrUn	48992660	48993342	683	SAT1_CF	blue	na
chrUn	48993355	48994055	701	SAT1_CF	blue	na
chrUn	48994083	48994593	511	SAT1_CF	blue	na
chrUn	48994662	48995385	724	SAT1_CF	blue	na
chrUn	48995397	48995880	484	SAT1_CF	blue	na
chrUn	48995933	48996120	188	SAT1_CF	blue	na
chrUn	48996131	48996679	549	SAT1_CF	blue	na
chrUn	48996758	48997722	965	SAT1_CF	blue	na
chrUn	48997765	48998310	546	SAT1_CF	blue	na
chrUn	48998331	48998439	109	SAT1_CF	blue	na
chrUn	48998452	48998953	502	SAT1_CF	blue	na
chrUn	48998967	49000209	1243	SAT1_CF	blue	na
chrUn	49000262	49000338	77	SAT1_CF	blue	na
chrUn	49000351	49000853	503	SAT1_CF	blue	na
chrUn	49000898	49001055	158	SAT1_CF	blue	na
chrUn	49001084	49002272	1189	SAT1_CF	blue	na
chrUn	49002300	49002931	632	SAT1_CF	blue	na
chrUn	49002974	49003648	675	SAT1_CF	blue	na
chrUn	49003661	49006076	2416	SAT1_CF	blue	na
chrUn	49006098	49006664	567	SAT1_CF	blue	na
chrUn	49006719	49007091	373	SAT1_CF	blue	na
chrUn	49007138	49007645	508	SAT1_CF	blue	na
chrUn	49007689	49008506	818	SAT1_CF	blue	na
chrUn	49008548	49009408	861	SAT1_CF	blue	na
chrUn	49009420	49010477	1058	SAT1_CF	blue	na
chrUn	49010520	49011816	1297	SAT1_CF	blue	na
chrUn	49011834	49012354	521	SAT1_CF	blue	na
chrUn	49012375	49014338	1964	SAT1_CF	blue	na
chrUn	49014380	49015057	678	SAT1_CF	blue	na
chrUn	49015069	49016305	1237	SAT1_CF	blue	na
chrUn	49016340	49016880	541	SAT1_CF	blue	na
chrUn	49016912	49018998	2087	CarSat1	red	na
chrUn	49019028	49021478	2451	CarSat1	red	na
chrUn	49042618	49043468	851	Sat2CF	purple	na
chrUn	49043020	49043518	499	Sat2CF	purple	na
chrUn	49059762	49061207	1446	CarSat1	red	na
chrUn	49061228	49062830	1603	CarSat1	red	na

chrUn	49062984	49064447	1464	CarSat1	red	na
chrUn	49064468	49066070	1603	CarSat1	red	na
chrUn	49083047	49083085	39	HSATII	na	na
chrUn	49083268	49083329	62	HSATII	na	na
chrUn	49084516	49085392	877	CarSat1	red	na
chrUn	49085638	49086282	645	CarSat1	red	na
chrUn	49086363	49086838	476	CarSat1	red	na
chrUn	49087025	49087373	349	CarSat1	red	na
chrUn	49087457	49087932	476	CarSat1	red	na
chrUn	49088119	49088468	350	CarSat1	red	na
chrUn	49088552	49089327	776	CarSat1	red	na
chrUn	49089555	49092144	2590	CarSat1	red	na
chrUn	49092147	49092676	530	SAT1_CF	blue	na
chrUn	49110883	49112061	1179	Sat2CF	purple	na
chrUn	49110914	49111933	1020	Sat2CF	purple	na
chrUn	49126774	49127957	1184	Sat2CF	purple	na
chrUn	49129402	49129984	583	SAT1_CF	blue	na
chrUn	49130026	49131536	1511	SAT1_CF	blue	na
chrUn	49131550	49132167	618	SAT1_CF	blue	na
chrUn	49132253	49133839	1587	SAT1_CF	blue	na
chrUn	49133853	49134478	626	SAT1_CF	blue	na
chrUn	49134490	49136824	2335	SAT1_CF	blue	na
chrUn	49136882	49137513	632	SAT1_CF	blue	na
chrUn	49137525	49138410	886	SAT1_CF	blue	na
chrUn	49138423	49139126	704	SAT1_CF	blue	na
chrUn	49139138	49139797	660	SAT1_CF	blue	na
chrUn	49139845	49140378	534	SAT1_CF	blue	na
chrUn	49140407	49141072	666	SAT1_CF	blue	na
chrUn	49141116	49142481	1366	SAT1_CF	blue	na
chrUn	49142691	49143965	1275	SAT1_CF	blue	na
chrUn	49143977	49144652	676	SAT1_CF	blue	na
chrUn	49144695	49148098	3404	SAT1_CF	blue	na
chrUn	49148140	49148918	779	SAT1_CF	blue	na
chrUn	49148984	49149505	522	SAT1_CF	blue	na
chrUn	49149518	49149920	403	SAT1_CF	blue	na
chrUn	49149941	49151420	1480	SAT1_CF	blue	na
chrUn	49151840	49152066	227	SAT1_CF	blue	na
chrUn	49152079	49152475	397	SAT1_CF	blue	na
chrUn	49152520	49153107	588	SAT1_CF	blue	na
chrUn	49153163	49153717	555	SAT1_CF	blue	na
chrUn	49153729	49155774	2046	SAT1_CF	blue	na
chrUn	49155954	49156138	185	SAT1_CF	blue	na

chrUn	49156198	49156678	481	SAT1_CF	blue	na
chrUn	49156691	49156796	106	SAT1_CF	blue	na
chrUn	49156819	49158516	1698	SAT1_CF	blue	na
chrUn	49158531	49158602	72	SAT1_CF	blue	na
chrUn	49158658	49159953	1296	SAT1_CF	blue	na
chrUn	49159996	49160608	613	SAT1_CF	blue	na
chrUn	49160624	49161026	403	SAT1_CF	blue	na
chrUn	49161066	49161567	502	SAT1_CF	blue	na
chrUn	49161579	49161688	110	SAT1_CF	blue	na
chrUn	49161709	49162339	631	SAT1_CF	blue	na
chrUn	49162351	49162906	556	SAT1_CF	blue	na
chrUn	49162917	49162965	49	SAT1_CF	blue	na
chrUn	49175056	49175822	767	Sat2CF	purple	na
chrUn	49175077	49175740	664	Sat2CF	purple	na
chrUn	49196115	49196848	734	Sat2CF	purple	na
chrUn	49196189	49196846	658	Sat2CF	purple	na
chrUn	49218407	49218588	182	SAT1_CF	blue	na
chrUn	49218600	49219842	1243	SAT1_CF	blue	na
chrUn	49221815	49222143	329	SAT1_CF	blue	na
chrUn	49222213	49222757	545	SAT1_CF	blue	na
chrUn	49222800	49223758	959	SAT1_CF	blue	na
chrUn	49223771	49224430	660	SAT1_CF	blue	na
chrUn	49224442	49225050	609	SAT1_CF	blue	na
chrUn	49227406	49227807	402	SAT1_CF	blue	na
chrUn	49227877	49228531	655	SAT1_CF	blue	na
chrUn	49228543	49229023	481	SAT1_CF	blue	na
chrUn	49229233	49229912	680	SAT1_CF	blue	na
chrUn	49230012	49231237	1226	SAT1_CF	blue	na
chrUn	49231286	49231827	542	SAT1_CF	blue	na
chrUn	49231865	49233140	1276	SAT1_CF	blue	na
chrUn	49233180	49233682	503	SAT1_CF	blue	na
chrUn	49233694	49234379	686	SAT1_CF	blue	na
chrUn	49234391	49235067	677	SAT1_CF	blue	na
chrUn	49235110	49236645	1536	SAT1_CF	blue	na
chrUn	49238045	49239024	980	SAT1_CF	blue	na
chrUn	49239052	49239536	485	SAT1_CF	blue	na
chrUn	49240281	49240902	622	SAT1_CF	blue	na
chrUn	49240930	49241087	158	SAT1_CF	blue	na
chrUn	49241132	49242332	1201	SAT1_CF	blue	na
chrUn	49242352	49243024	673	SAT1_CF	blue	na
chrUn	49243104	49245304	2201	SAT1_CF	blue	na
chrUn	49245320	49246829	1510	SAT1_CF	blue	na

chrUn	49261525	49262309	785	SAT1_CF	blue	na
chrUn	49262383	49262902	520	SAT1_CF	blue	na
chrUn	49262958	49263359	402	SAT1_CF	blue	na
chrUn	49264364	49264519	156	SAT1_CF	blue	na
chrUn	49264564	49265064	501	SAT1_CF	blue	na
chrUn	49265081	49265668	588	SAT1_CF	blue	na
chrUn	49265680	49266322	643	SAT1_CF	blue	na
chrUn	49266514	49266889	376	SAT1_CF	blue	na
chrUn	49267765	49268733	969	SAT1_CF	blue	na
chrUn	49268914	49269097	184	SAT1_CF	blue	na
chrUn	49269118	49269637	520	SAT1_CF	blue	na
chrUn	49269650	49271766	2117	SAT1_CF	blue	na
chrUn	49271779	49273004	1226	SAT1_CF	blue	na
chrUn	49273053	49273145	93	SAT1_CF	blue	na
chrUn	49273202	49273760	559	SAT1_CF	blue	na
chrUn	49273771	49274201	431	SAT1_CF	blue	na
chrUn	49274280	49275438	1159	SAT1_CF	blue	na
chrUn	49275651	49276137	487	SAT1_CF	blue	na
chrUn	49276189	49276346	158	SAT1_CF	blue	na
chrUn	49276391	49276892	502	SAT1_CF	blue	na
chrUn	49276906	49277663	758	SAT1_CF	blue	na
chrUn	49277713	49278622	910	SAT1_CF	blue	na
chrUn	49278676	49279492	817	SAT1_CF	blue	na
chrUn	49279541	49279990	450	SAT1_CF	blue	na
chrUn	49280003	49280979	977	SAT1_CF	blue	na
chrUn	49281005	49281507	503	SAT1_CF	blue	na
chrUn	49281535	49281692	158	SAT1_CF	blue	na
chrUn	49281718	49282238	521	SAT1_CF	blue	na
chrUn	49282250	49282924	675	SAT1_CF	blue	na
chrUn	49282966	49284133	1168	SAT1_CF	blue	na
chrUn	49284203	49284676	474	SAT1_CF	blue	na
chrUn	49284689	49285284	596	SAT1_CF	blue	na
chrUn	49285318	49287055	1738	SAT1_CF	blue	na
chrUn	49287172	49287270	99	SAT1_CF	blue	na
chrUn	49287330	49287879	550	SAT1_CF	blue	na
chrUn	49287921	49288605	685	SAT1_CF	blue	na
chrUn	49289499	49290170	672	SAT1_CF	blue	na
chrUn	49290215	49290847	633	SAT1_CF	blue	na
chrUn	49290875	49292110	1236	SAT1_CF	blue	na
chrUn	49292122	49293372	1251	SAT1_CF	blue	na
chrUn	49293384	49294001	618	SAT1_CF	blue	na
chrUn	49294043	49294593	551	SAT1_CF	blue	na

chrUn	49295111	49295428	318	SAT1_CF	blue	na
chrUn	49295452	49296830	1379	SAT1_CF	blue	na
chrUn	49331716	49332617	902	SAT1_CF	blue	na
chrUn	49332645	49332913	269	SAT1_CF	blue	na
chrUn	49332957	49333500	544	SAT1_CF	blue	na
chrUn	49333518	49334204	687	SAT1_CF	blue	na
chrUn	49334216	49336133	1918	SAT1_CF	blue	na
chrUn	49336176	49336683	508	SAT1_CF	blue	na
chrUn	49336716	49336872	157	SAT1_CF	blue	na
chrUn	49336898	49337418	521	SAT1_CF	blue	na
chrUn	49337430	49338027	598	SAT1_CF	blue	na
chrUn	49338047	49338959	913	SAT1_CF	blue	na
chrUn	49338987	49339255	269	SAT1_CF	blue	na
chrUn	49339299	49339842	544	SAT1_CF	blue	na
chrUn	49339860	49340546	687	SAT1_CF	blue	na
chrUn	49340558	49342901	2344	SAT1_CF	blue	na
chrUn	49342946	49343901	956	SAT1_CF	blue	na
chrUn	49343958	49344504	547	SAT1_CF	blue	na
chrUn	49344546	49346358	1813	SAT1_CF	blue	na
chrUn	49346403	49346904	502	SAT1_CF	blue	na
chrUn	49346922	49347029	108	SAT1_CF	blue	na
chrUn	49347046	49347678	633	SAT1_CF	blue	na
chrUn	49347722	49347768	47	SAT1_CF	blue	na
chrUn	49347785	49348303	519	SAT1_CF	blue	na
chrUn	49348315	49349167	853	SAT1_CF	blue	na
chrUn	49349218	49349841	624	SAT1_CF	blue	na
chrUn	49349928	49350590	663	SAT1_CF	blue	na
chrUn	49350602	49351462	861	SAT1_CF	blue	na
chrUn	49351504	49353361	1858	SAT1_CF	blue	na
chrUn	49353401	49353902	502	SAT1_CF	blue	na
chrUn	49353915	49354018	104	SAT1_CF	blue	na
chrUn	49354043	49354602	560	SAT1_CF	blue	na
chrUn	49354614	49355286	673	SAT1_CF	blue	na
chrUn	49355329	49356331	1003	SAT1_CF	blue	na
chrUn	49356398	49357333	936	SAT1_CF	blue	na
chrUn	49357442	49357938	497	SAT1_CF	blue	na
chrUn	49357956	49358058	103	SAT1_CF	blue	na
chrUn	49358079	49358634	556	SAT1_CF	blue	na
chrUn	49358647	49359253	607	SAT1_CF	blue	na
chrUn	49359265	49360124	860	SAT1_CF	blue	na
chrUn	49360135	49360736	602	SAT1_CF	blue	na
chrUn	49360758	49361160	403	SAT1_CF	blue	na

chrUn	49361200	49362289	1090	SAT1_CF	blue	na
chrUn	49362307	49362976	670	SAT1_CF	blue	na
chrUn	49363019	49363557	539	SAT1_CF	blue	na
chrUn	49363787	49364409	623	SAT1_CF	blue	na
chrUn	49365427	49366101	675	SAT1_CF	blue	na
chrUn	49366113	49367627	1515	SAT1_CF	blue	na
chrUn	49367639	49368102	464	SAT1_CF	blue	na
chrUn	49368172	49368717	546	SAT1_CF	blue	na
chrUn	49368739	49368845	107	SAT1_CF	blue	na
chrUn	49368858	49369360	503	SAT1_CF	blue	na
chrUn	49369426	49369561	136	SAT1_CF	blue	na
chrUn	49369595	49370099	505	SAT1_CF	blue	na
chrUn	49370179	49370734	556	SAT1_CF	blue	na
chrUn	49370848	49371821	974	SAT1_CF	blue	na
chrUn	49371864	49372539	676	SAT1_CF	blue	na
chrUn	49372551	49373237	687	SAT1_CF	blue	na
chrUn	49373255	49373799	545	SAT1_CF	blue	na
chrUn	49373842	49374105	264	SAT1_CF	blue	na
chrUn	49374133	49374759	627	SAT1_CF	blue	na
chrUn	49374783	49374889	107	SAT1_CF	blue	na
chrUn	49374901	49376257	1357	SAT1_CF	blue	na
chrUn	49376849	49377325	477	SAT1_CF	blue	na
chrUn	49377338	49377840	503	SAT1_CF	blue	na
chrUn	49377876	49378061	186	SAT1_CF	blue	na
chrUn	49378241	49379077	837	SAT1_CF	blue	na
chrUn	49379089	49379995	907	SAT1_CF	blue	na
chrUn	49380053	49380182	130	SAT1_CF	blue	na
chrUn	49381499	49381839	341	SAT1_CF	blue	na
chrUn	49381864	49383972	2109	SAT1_CF	blue	na
chrUn	49383992	49384588	597	SAT1_CF	blue	na
chrUn	49384600	49385120	521	SAT1_CF	blue	na
chrUn	49385141	49386351	1211	SAT1_CF	blue	na
chrUn	49386405	49387008	604	SAT1_CF	blue	na
chrUn	49387050	49387868	819	SAT1_CF	blue	na
chrUn	49387888	49388727	840	SAT1_CF	blue	na
chrUn	49388740	49389241	502	SAT1_CF	blue	na
chrUn	49389328	49389672	345	SAT1_CF	blue	na
chrUn	49389689	49390176	488	SAT1_CF	blue	na
chrUn	49390208	49390543	336	SAT1_CF	blue	na
chrUn	49390591	49391924	1334	SAT1_CF	blue	na
chrUn	49391937	49392438	502	SAT1_CF	blue	na
chrUn	49392524	49392698	175	SAT1_CF	blue	na

chrUn	49392779	49394025	1247	SAT1_CF	blue	na
chrUn	49394037	49394589	553	SAT1_CF	blue	na
chrUn	49394611	49394683	73	SAT1_CF	blue	na
chrUn	49394696	49395624	929	SAT1_CF	blue	na
chrUn	49395636	49396804	1169	SAT1_CF	blue	na
chrUn	49396816	49397887	1072	SAT1_CF	blue	na
chrUn	49397901	49399722	1822	SAT1_CF	blue	na
chrUn	49399735	49399952	218	SAT1_CF	blue	na
chrUn	49400952	49401527	576	SAT1_CF	blue	na
chrUn	49401573	49402081	509	SAT1_CF	blue	na
chrUn	49402160	49402485	326	SAT1_CF	blue	na
chrUn	49402510	49403405	896	SAT1_CF	blue	na
chrUn	49403475	49404444	970	SAT1_CF	blue	na
chrUn	49404488	49405819	1332	SAT1_CF	blue	na
chrUn	49405833	49406375	543	SAT1_CF	blue	na
chrUn	49406492	49407624	1133	SAT1_CF	blue	na
chrUn	49407677	49407753	77	SAT1_CF	blue	na
chrUn	49407766	49409555	1790	SAT1_CF	blue	na
chrUn	49409569	49410452	884	SAT1_CF	blue	na
chrUn	49410495	49411169	675	SAT1_CF	blue	na
chrUn	49411182	49411849	668	SAT1_CF	blue	na
chrUn	49411877	49414798	2922	SAT1_CF	blue	na
chrUn	49414841	49415508	668	SAT1_CF	blue	na
chrUn	49415530	49417664	2135	SAT1_CF	blue	na
chrUn	49417694	49418237	544	SAT1_CF	blue	na
chrUn	49418279	49418691	413	SAT1_CF	blue	na
chrUn	49418743	49419371	629	SAT1_CF	blue	na
chrUn	49419395	49419501	107	SAT1_CF	blue	na
chrUn	49419513	49420196	684	SAT1_CF	blue	na
chrUn	49420209	49420709	501	SAT1_CF	blue	na
chrUn	49420749	49422439	1691	SAT1_CF	blue	na
chrUn	49428613	49428979	367	SAT1_CF	blue	na
chrUn	49429096	49430190	1095	SAT1_CF	blue	na
chrUn	49431613	49432295	683	SAT1_CF	blue	na
chrUn	49432340	49432496	157	SAT1_CF	blue	na
chrUn	49432511	49433035	525	SAT1_CF	blue	na
chrUn	49433116	49433668	553	SAT1_CF	blue	na
chrUn	49433737	49434039	303	SAT1_CF	blue	na
chrUn	49438043	49438601	559	SAT1_CF	blue	na
chrUn	49440694	49441414	721	SAT1_CF	blue	na
chrUn	49441426	49441947	522	SAT1_CF	blue	na
chrUn	49441979	49442343	365	SAT1_CF	blue	na

chrUn	49444069	49444587	519	SAT1_CF	blue	na
chrUn	49444621	49444789	169	SAT1_CF	blue	na
chrUn	49444971	49445855	885	SAT1_CF	blue	na
chrUn	49447945	49448245	301	SAT1_CF	blue	na
chrUn	49448257	49448808	552	SAT1_CF	blue	na
chrUn	49448912	49449166	255	SAT1_CF	blue	na
chrUn	49451830	49452179	350	SAT1_CF	blue	na
chrUn	49452196	49452857	662	SAT1_CF	blue	na
chrUn	49458531	49459293	763	SAT1_CF	blue	na
chrUn	49459315	49461393	2079	SAT1_CF	blue	na
chrUn	49461472	49462201	730	SAT1_CF	blue	na
chrUn	49462214	49462705	492	SAT1_CF	blue	na
chrUn	49462722	49463397	676	SAT1_CF	blue	na
chrUn	49463422	49465658	2237	SAT1_CF	blue	na
chrUn	49465674	49465835	162	SAT1_CF	blue	na
chrUn	49465849	49466992	1144	SAT1_CF	blue	na
chrUn	49467032	49468222	1191	SAT1_CF	blue	na
chrUn	49468265	49468896	632	SAT1_CF	blue	na
chrUn	49468961	49469525	565	SAT1_CF	blue	na
chrUn	49469605	49470129	525	SAT1_CF	blue	na
chrUn	49470144	49470280	137	SAT1_CF	blue	na
chrUn	49470346	49471504	1159	SAT1_CF	blue	na
chrUn	49471517	49472585	1069	SAT1_CF	blue	na
chrUn	49472664	49473465	802	SAT1_CF	blue	na
chrUn	49473537	49474017	481	SAT1_CF	blue	na
chrUn	49474029	49475250	1222	SAT1_CF	blue	na
chrUn	49475337	49475839	503	SAT1_CF	blue	na
chrUn	49475852	49475924	73	SAT1_CF	blue	na
chrUn	49475946	49476460	515	SAT1_CF	blue	na
chrUn	49476472	49477331	860	SAT1_CF	blue	na
chrUn	49477373	49478154	782	SAT1_CF	blue	na
chrUn	49478233	49478776	544	SAT1_CF	blue	na
chrUn	49478791	49478948	158	SAT1_CF	blue	na
chrUn	49479015	49479515	501	SAT1_CF	blue	na
chrUn	49479528	49481269	1742	SAT1_CF	blue	na
chrUn	49481320	49481870	551	SAT1_CF	blue	na
chrUn	49483252	49483832	581	SAT1_CF	blue	na
chrUn	49483892	49484366	475	SAT1_CF	blue	na
chrUn	49484386	49485068	683	SAT1_CF	blue	na
chrUn	49485080	49485189	110	SAT1_CF	blue	na
chrUn	49485220	49486089	870	SAT1_CF	blue	na
chrUn	49487131	49487774	644	CarSat1	red	na

chrUn	49487801	49488761	961	CarSat1	red	na
chrUn	49488777	49489712	936	CarSat1	red	na
chrUn	49489728	49489855	128	Carsat2	orange	na
chrUn	49489744	49490234	491	CarSat1	red	na
chrUn	49490252	49490964	713	CarSat1	red	na
chrUn	49490989	49493924	2936	CarSat1	red	na
chrUn	49493940	49494409	470	CarSat1	red	na
chrUn	49494436	49495687	1252	CarSat1	red	na
chrUn	49496761	49497651	891	CarSat1	red	na
chrUn	49497667	49499607	1941	CarSat1	red	na
chrUn	49499634	49501414	1781	CarSat1	red	na
chrUn	49501596	49503267	1672	CarSat1	red	na
chrUn	49503294	49504258	965	CarSat1	red	na
chrUn	49504479	49508155	3677	CarSat1	red	na
chrUn	49508185	49508892	708	CarSat1	red	na
chrUn	49508907	49509032	126	Carsat2	orange	na
chrUn	49508922	49509897	976	CarSat1	red	na
chrUn	49509924	49510634	711	CarSat1	red	na
chrUn	49510655	49512106	1452	CarSat1	red	na
chrUn	49512133	49512841	709	CarSat1	red	na
chrUn	49512868	49513831	964	CarSat1	red	na
chrUn	49513847	49514316	470	CarSat1	red	na
chrUn	49514345	49515053	709	CarSat1	red	na
chrUn	49515080	49516528	1449	CarSat1	red	na
chrUn	49516549	49518990	2442	CarSat1	red	na
chrUn	49519015	49519474	460	CarSat1	red	na
chrUn	49519501	49520640	1140	CarSat1	red	na
chrUn	49569150	49570024	875	CarSat1	red	na
chrUn	49570122	49570977	856	CarSat1	red	na
chrUn	49570998	49571850	853	CarSat1	red	na
chrUn	49571999	49572660	662	CarSat1	red	na
chrUn	49572738	49573195	458	CarSat1	red	na
chrUn	49573379	49573788	410	CarSat1	red	na
chrUn	49573810	49574663	854	CarSat1	red	na
chrUn	49574690	49575491	802	CarSat1	red	na
chrUn	49575686	49578060	2375	CarSat1	red	na
chrUn	49578063	49578450	388	SAT1_CF	blue	na
chrUn	49578462	49579140	679	SAT1_CF	blue	na
chrUn	49579190	49580850	1661	SAT1_CF	blue	na
chrUn	49580892	49582219	1328	SAT1_CF	blue	na
chrUn	49582242	49583380	1139	SAT1_CF	blue	na
chrUn	49583400	49586352	2953	SAT1_CF	blue	na

chrUn	49586405	49587234	830	SAT1_CF	blue	na
chrUn	49587277	49588002	726	CarSat1	red	na
chrUn	49588023	49589612	1590	CarSat1	red	na
chrUn	49589759	49591590	1832	CarSat1	red	na
chrUn	49591592	49591985	394	SAT1_CF	blue	na
chrUn	49591997	49593188	1192	SAT1_CF	blue	na
chrUn	49593269	49595341	2073	SAT1_CF	blue	na
chrUn	49595361	49596510	1150	SAT1_CF	blue	na
chrUn	49596505	49597129	625	CarSat1	red	na
chrUn	49598140	49599493	1354	CarSat1	red	na
chrUn	49600072	49601589	1518	CarSat1	red	na
chrUn	49603379	49604307	929	CarSat1	red	na
chrUn	49604337	49605093	757	CarSat1	red	na
chrUn	49605246	49606690	1445	CarSat1	red	na
chrUn	49606993	49609312	2320	CarSat1	red	na
chrUn	49609660	49610145	486	CarSat1	red	na
chrUn	49610169	49611063	895	CarSat1	red	na
chrUn	49611635	49612693	1059	CarSat1	red	na
chrUn	49613477	49614599	1123	CarSat1	red	na
chrUn	49615072	49615645	574	CarSat1	red	na
chrUn	49615675	49616387	713	CarSat1	red	na
chrUn	49616417	49617124	708	CarSat1	red	na
chrUn	49617139	49617265	127	Carsat2	orange	na
chrUn	49617154	49618232	1079	CarSat1	red	na
chrUn	49618247	49619040	794	CarSat1	red	na
chrUn	49619051	49620396	1346	CarSat1	red	na
chrUn	49620930	49622643	1714	CarSat1	red	na
chrUn	49623496	49624283	788	CarSat1	red	na
chrUn	49624299	49624423	125	Carsat2	orange	na
chrUn	49624312	49624798	487	CarSat1	red	na
chrUn	49624822	49625295	474	CarSat1	red	na
chrUn	49625326	49626404	1079	CarSat1	red	na
chrUn	49626431	49628610	2180	CarSat1	red	na
chrUn	49628626	49628750	125	Carsat2	orange	na
chrUn	49628639	49629351	713	CarSat1	red	na
chrUn	49629381	49629867	487	CarSat1	red	na
chrUn	49629900	49631415	1516	CarSat1	red	na
chrUn	49633108	49634269	1162	CarSat1	red	na
chrUn	49634303	49635619	1317	CarSat1	red	na
chrUn	49635649	49636222	574	CarSat1	red	na
chrUn	49637619	49639249	1631	CarSat1	red	na
chrUn	49652711	49656055	3345	CarSat1	red	na

chrUn	49656066	49657577	1512	CarSat1	red	na
chrUn	49661244	49662237	994	CarSat1	red	na
chrUn	49662422	49662998	577	CarSat1	red	na
chrUn	49664290	49667949	3660	CarSat1	red	na
chrUn	49668392	49669933	1542	CarSat1	red	na
chrUn	49670650	49671806	1157	CarSat1	red	na
chrUn	49671836	49674024	2189	CarSat1	red	na
chrUn	49674054	49676235	2182	CarSat1	red	na
chrUn	49676259	49676759	501	CarSat1	red	na
chrUn	49676774	49677865	1092	CarSat1	red	na
chrUn	49680393	49681414	1022	CarSat1	red	na
chrUn	49681721	49682793	1073	CarSat1	red	na
chrUn	49683821	49685801	1981	CarSat1	red	na
chrUn	49687467	49688587	1121	CarSat1	red	na
chrUn	49689542	49690445	904	CarSat1	red	na
chrUn	49690479	49691512	1034	CarSat1	red	na
chrUn	49725045	49725959	915	CarSat1	red	na
chrUn	49725980	49726833	854	CarSat1	red	na
chrUn	49726854	49728071	1218	CarSat1	red	na
chrUn	49728257	49728657	401	CarSat1	red	na
chrUn	49728678	49730276	1599	CarSat1	red	na
chrUn	49730297	49731514	1218	CarSat1	red	na
chrUn	49731700	49732100	401	CarSat1	red	na
chrUn	49732121	49733719	1599	CarSat1	red	na
chrUn	49733740	49735337	1598	CarSat1	red	na
chrUn	49735368	49736957	1590	CarSat1	red	na
chrUn	49736978	49737477	500	CarSat1	red	na
chrUn	49749482	49750125	644	SAT1_CF	blue	na
chrUn	49750168	49751280	1113	SAT1_CF	blue	na
chrUn	49751312	49752166	855	SAT1_CF	blue	na
chrUn	49752210	49755384	3175	SAT1_CF	blue	na
chrUn	49755428	49756568	1141	SAT1_CF	blue	na
chrUn	49756554	49757276	723	CarSat1	red	na
chrUn	49758288	49760854	2567	CarSat1	red	na
chrUn	49760884	49761370	487	CarSat1	red	na
chrUn	49761394	49764533	3140	CarSat1	red	na
chrUn	49764563	49765783	1221	CarSat1	red	na
chrUn	49765807	49767268	1462	CarSat1	red	na
chrUn	49767309	49768226	918	CarSat1	red	na
chrUn	49768241	49768367	127	Carsat2	orange	na
chrUn	49768256	49768740	485	CarSat1	red	na
chrUn	49768764	49770443	1680	CarSat1	red	na

chrUn	49770473	49774114	3642	CarSat1	red	na
chrUn	49774140	49774263	124	Carsat2	orange	na
chrUn	49774155	49774641	487	CarSat1	red	na
chrUn	49774665	49779089	4425	CarSat1	red	na
chrUn	49779113	49779849	737	CarSat1	red	na
chrUn	49779873	49781536	1664	CarSat1	red	na
chrUn	49781579	49789739	8161	CarSat1	red	na
chrUn	49790739	49791291	553	SAT1_CF	blue	na
chrUn	49791331	49793716	2386	SAT1_CF	blue	na
chrUn	49793728	49794200	473	SAT1_CF	blue	na
chrUn	49794226	49794329	104	SAT1_CF	blue	na
chrUn	49794342	49794842	501	SAT1_CF	blue	na
chrUn	49794885	49795283	399	SAT1_CF	blue	na
chrUn	49795294	49796389	1096	SAT1_CF	blue	na
chrUn	49796404	49797235	832	SAT1_CF	blue	na
chrUn	49797249	49797564	316	SAT1_CF	blue	na
chrUn	49797606	49798288	683	SAT1_CF	blue	na
chrUn	49798302	49798795	494	SAT1_CF	blue	na
chrUn	49798806	49799824	1019	SAT1_CF	blue	na
chrUn	49799862	49802288	2427	SAT1_CF	blue	na
chrUn	49802308	49804608	2301	SAT1_CF	blue	na
chrUn	49804631	49805436	806	SAT1_CF	blue	na
chrUn	49805447	49805945	499	SAT1_CF	blue	na
chrUn	49805974	49806338	365	SAT1_CF	blue	na
chrUn	49806445	49807009	565	SAT1_CF	blue	na
chrUn	49807052	49807595	544	SAT1_CF	blue	na
chrUn	49807618	49808952	1335	SAT1_CF	blue	na
chrUn	49808973	49809932	960	SAT1_CF	blue	na
chrUn	49810047	49810536	490	SAT1_CF	blue	na
chrUn	49810559	49812335	1777	SAT1_CF	blue	na
chrUn	49812450	49813414	965	SAT1_CF	blue	na
chrUn	49813457	49814003	547	SAT1_CF	blue	na
chrUn	49814024	49814414	391	SAT1_CF	blue	na
chrUn	49817722	49818686	965	SAT1_CF	blue	na
chrUn	49818730	49819274	545	SAT1_CF	blue	na
chrUn	49819330	49819405	76	SAT1_CF	blue	na
chrUn	49819416	49819915	500	SAT1_CF	blue	na
chrUn	49819968	49821993	2026	SAT1_CF	blue	na
chrUn	49822037	49822711	675	SAT1_CF	blue	na
chrUn	49822723	49823354	632	SAT1_CF	blue	na
chrUn	49823375	49823484	110	SAT1_CF	blue	na
chrUn	49823496	49824015	520	SAT1_CF	blue	na

chrUn	49824036	49824417	382	SAT1_CF	blue	na
chrUn	49824445	49825176	732	SAT1_CF	blue	na
chrUn	49880037	49881091	1055	CarSat1	red	na
chrUn	49881128	49882105	978	CarSat1	red	na
chrUn	49882130	49882563	434	CarSat1	red	na
chrUn	49882600	49885051	2452	CarSat1	red	na
chrUn	49885080	49885789	710	CarSat1	red	na
chrUn	49885816	49886527	712	CarSat1	red	na
chrUn	49886552	49888734	2183	CarSat1	red	na
chrUn	49888755	49889469	715	CarSat1	red	na
chrUn	49889496	49889936	441	CarSat1	red	na
chrUn	49889966	49891195	1230	CarSat1	red	na
chrUn	49891220	49891680	461	CarSat1	red	na
chrUn	49894093	49895841	1749	CarSat1	red	na
chrUn	49895871	49896574	704	CarSat1	red	na
chrUn	49896604	49902481	5878	CarSat1	red	na
chrUn	49903369	49904115	747	CarSat1	red	na
chrUn	49904136	49905834	1699	CarSat1	red	na
chrUn	49905850	49906570	721	CarSat1	red	na
chrUn	49906586	49907102	517	CarSat1	red	na
chrUn	49908582	49909839	1258	CarSat1	red	na
chrUn	49909866	49910306	441	CarSat1	red	na
chrUn	49910336	49911036	701	CarSat1	red	na
chrUn	49911070	49911774	705	CarSat1	red	na
chrUn	49911804	49915538	3735	CarSat1	red	na
chrUn	49917835	49918074	240	SAT1_CF	blue	na
chrUn	49919652	49921187	1536	CarSat1	red	na
chrUn	49921403	49921453	51	CarSat1	red	na
chrUn	49921644	49923846	2203	CarSat1	red	na
chrUn	49924011	49925005	995	CarSat1	red	na
chrUn	49925289	49925602	314	CarSat1	red	na
chrUn	49925789	49926789	1001	CarSat1	red	na
chrUn	49926971	49929345	2375	CarSat1	red	na
chrUn	49929260	49929693	434	Carsat2	orange	na
chrUn	49929859	49930120	262	Carsat2	orange	na
chrUn	49930310	49931123	814	Carsat2	orange	na
chrUn	49930715	49933805	3091	CarSat1	red	na
chrUn	49934008	49935194	1187	CarSat1	red	na
chrUn	49941309	49943068	1760	Carsat2	orange	na
chrUn	49943250	49944241	992	Carsat2	orange	na
chrUn	50196669	50196905	237	SAT1_CF	blue	na
chrUn	50196932	50197535	604	SAT1_CF	blue	na

chrUn	50197547	50200057	2511	SAT1_CF	blue	na
chrUn	50200069	50200570	502	SAT1_CF	blue	na
chrUn	50200610	50201533	924	SAT1_CF	blue	na
chrUn	50201611	50201917	307	SAT1_CF	blue	na
chrUn	50201961	50203291	1331	SAT1_CF	blue	na
chrUn	50203304	50203805	502	SAT1_CF	blue	na
chrUn	50203845	50204237	393	SAT1_CF	blue	na
chrUn	50204254	50204802	549	SAT1_CF	blue	na
chrUn	50204828	50205035	208	SAT1_CF	blue	na
chrUn	50205083	50206417	1335	SAT1_CF	blue	na
chrUn	50206430	50206932	503	SAT1_CF	blue	na
chrUn	50207018	50208681	1664	SAT1_CF	blue	na
chrUn	50209894	50210357	464	SAT1_CF	blue	na
chrUn	50210402	50210558	157	SAT1_CF	blue	na
chrUn	50210624	50211714	1091	SAT1_CF	blue	na
chrUn	50213207	50213678	472	SAT1_CF	blue	na
chrUn	50213691	50214192	502	SAT1_CF	blue	na
chrUn	50214205	50216016	1812	SAT1_CF	blue	na
chrUn	50216096	50216770	675	SAT1_CF	blue	na
chrUn	50216782	50217485	704	SAT1_CF	blue	na
chrUn	50217498	50218333	836	SAT1_CF	blue	na
chrUn	50218346	50219663	1318	SAT1_CF	blue	na
chrUn	50219675	50220188	514	SAT1_CF	blue	na
chrUn	50220214	50220746	533	SAT1_CF	blue	na
chrUn	50220796	50221522	727	SAT1_CF	blue	na
chrUn	50221565	50222241	677	SAT1_CF	blue	na
chrUn	50222253	50222854	602	SAT1_CF	blue	na
chrUn	50222865	50223543	679	SAT1_CF	blue	na
chrUn	50223555	50224243	689	SAT1_CF	blue	na
chrUn	50224257	50225317	1061	SAT1_CF	blue	na
chrUn	50225350	50225897	548	SAT1_CF	blue	na
chrUn	50225911	50226611	701	SAT1_CF	blue	na
chrUn	50226631	50227734	1104	SAT1_CF	blue	na
chrUn	50227746	50228301	556	SAT1_CF	blue	na
chrUn	50228356	50228949	594	SAT1_CF	blue	na
chrUn	50229060	50229155	96	SAT1_CF	blue	na
chrUn	50230195	50230892	698	SAT1_CF	blue	na
chrUn	50230935	50231832	898	SAT1_CF	blue	na
chrUn	50231867	50232681	815	SAT1_CF	blue	na
chrUn	50232708	50233227	520	SAT1_CF	blue	na
chrUn	50233846	50234497	652	SAT1_CF	blue	na
chrUn	50234525	50235307	783	SAT1_CF	blue	na

chrUn	50235325	50235824	500	SAT1_CF	blue	na
chrUn	50235877	50236920	1044	SAT1_CF	blue	na
chrUn	50236967	50238020	1054	SAT1_CF	blue	na
chrUn	50238033	50238215	183	SAT1_CF	blue	na
chrUn	50238255	50238801	547	SAT1_CF	blue	na
chrUn	50238814	50238930	117	SAT1_CF	blue	na
chrUn	50238942	50239613	672	SAT1_CF	blue	na
chrUn	50239636	50240136	501	SAT1_CF	blue	na
chrUn	50240189	50240615	427	SAT1_CF	blue	na
chrUn	50240649	50241427	779	SAT1_CF	blue	na
chrUn	50241440	50241556	117	SAT1_CF	blue	na
chrUn	50241568	50242121	554	SAT1_CF	blue	na
chrUn	50242191	50242522	332	SAT1_CF	blue	na
chrUn	50244915	50245449	535	SAT1_CF	blue	na
chrUn	50245502	50246025	524	SAT1_CF	blue	na
chrUn	50247969	50248508	540	SAT1_CF	blue	na
chrUn	50248529	50248638	110	SAT1_CF	blue	na
chrUn	50248650	50249408	759	SAT1_CF	blue	na
chrUn	50249422	50249924	503	SAT1_CF	blue	na
chrUn	50249969	50250066	98	SAT1_CF	blue	na
chrUn	50250876	50251673	798	SAT1_CF	blue	na
chrUn	50251740	50252522	783	SAT1_CF	blue	na
chrUn	50252557	50253621	1065	SAT1_CF	blue	na
chrUn	50253643	50253715	73	SAT1_CF	blue	na
chrUn	50253728	50254458	731	SAT1_CF	blue	na
chrUn	50255492	50256094	603	SAT1_CF	blue	na
chrUn	50256107	50257801	1695	SAT1_CF	blue	na
chrUn	50257964	50258777	814	SAT1_CF	blue	na
chrUn	50258804	50259305	502	SAT1_CF	blue	na
chrUn	50259351	50260473	1123	SAT1_CF	blue	na
chrUn	50263540	50264205	666	SAT1_CF	blue	na
chrUn	50264249	50264922	674	SAT1_CF	blue	na
chrUn	50264934	50265625	692	SAT1_CF	blue	na
chrUn	50265638	50266181	544	SAT1_CF	blue	na
chrUn	50266224	50266767	544	SAT1_CF	blue	na
chrUn	50266793	50267591	799	SAT1_CF	blue	na
chrUn	50267604	50269228	1625	SAT1_CF	blue	na
chrUn	50270980	50271104	125	SAT1_CF	blue	na
chrUn	50271116	50271674	559	SAT1_CF	blue	na
chrUn	50271696	50271802	107	SAT1_CF	blue	na
chrUn	50271815	50272317	503	SAT1_CF	blue	na
chrUn	50273440	50274265	826	CarSat1	red	na

chrUn	50274295	50275003	709	CarSat1	red	na
chrUn	50275033	50282933	7901	CarSat1	red	na
chrUn	50282966	50285882	2917	CarSat1	red	na
chrUn	50285914	50287579	1666	CarSat1	red	na
chrUn	50287594	50287721	128	Carsat2	orange	na
chrUn	50287609	50291777	4169	CarSat1	red	na
chrUn	50291789	50293982	2194	CarSat1	red	na
chrUn	50294015	50294475	461	CarSat1	red	na
chrUn	50294492	50300080	5589	CarSat1	red	na
chrUn	50300121	50300851	731	CarSat1	red	na
chrUn	50301134	50301767	634	CarSat1	red	na
chrUn	50301800	50302499	700	CarSat1	red	na
chrUn	50302524	50303886	1363	CarSat1	red	na
chrUn	50327121	50328337	1217	Sat2CF	purple	na
chrUn	50327249	50328332	1084	Sat2CF	purple	na
chrUn	50328208	50328357	150	Sat2CF	purple	na
chrUn	50355719	50357275	1557	CarSat1	red	na
chrUn	50357296	50358893	1598	CarSat1	red	na
chrUn	50358914	50360508	1595	CarSat1	red	na
chrUn	50368092	50368197	106	SAT1_CF	blue	na
chrUn	50369585	50370310	726	SAT1_CF	blue	na
chrUn	50370353	50372168	1816	SAT1_CF	blue	na
chrUn	50372200	50372981	782	SAT1_CF	blue	na
chrUn	50372995	50373941	947	SAT1_CF	blue	na
chrUn	50373954	50374436	483	SAT1_CF	blue	na
chrUn	50374495	50375802	1308	SAT1_CF	blue	na
chrUn	50375815	50377807	1993	SAT1_CF	blue	na
chrUn	50377834	50379073	1240	SAT1_CF	blue	na
chrUn	50379188	50380692	1505	SAT1_CF	blue	na
chrUn	50380704	50381335	632	SAT1_CF	blue	na
chrUn	50381356	50381465	110	SAT1_CF	blue	na
chrUn	50381477	50381978	502	SAT1_CF	blue	na
chrUn	50382023	50382180	158	SAT1_CF	blue	na
chrUn	50382208	50384161	1954	SAT1_CF	blue	na
chrUn	50384196	50384676	481	SAT1_CF	blue	na
chrUn	50384702	50385765	1064	SAT1_CF	blue	na
chrUn	50385783	50386263	481	SAT1_CF	blue	na
chrUn	50386330	50388806	2477	SAT1_CF	blue	na
chrUn	50388841	50391344	2504	SAT1_CF	blue	na
chrUn	50391356	50391858	503	SAT1_CF	blue	na
chrUn	50391913	50393933	2021	SAT1_CF	blue	na
chrUn	50393976	50394651	676	SAT1_CF	blue	na

chrUn	50394663	50395356	694	SAT1_CF	blue	na
chrUn	50395378	50396176	799	SAT1_CF	blue	na
chrUn	50396219	50397911	1693	SAT1_CF	blue	na
chrUn	50397924	50398567	644	SAT1_CF	blue	na
chrUn	50399572	50400641	1070	CarSat1	red	na
chrUn	50400665	50401601	937	CarSat1	red	na
chrUn	50401631	50403343	1713	CarSat1	red	na
chrUn	50403370	50405810	2441	CarSat1	red	na
chrUn	50405826	50406759	934	CarSat1	red	na
chrUn	50406774	50406901	128	Carsat2	orange	na
chrUn	50406789	50407497	709	CarSat1	red	na
chrUn	50407512	50407639	128	Carsat2	orange	na
chrUn	50407527	50408504	978	CarSat1	red	na
chrUn	50408531	50410733	2203	CarSat1	red	na
chrUn	50410760	50411471	712	CarSat1	red	na
chrUn	50411498	50413684	2187	CarSat1	red	na
chrUn	50413711	50414407	697	CarSat1	red	na
chrUn	50414449	50415413	965	CarSat1	red	na
chrUn	50415429	50417373	1945	CarSat1	red	na
chrUn	50417398	50418360	963	CarSat1	red	na
chrUn	50418385	50418845	461	CarSat1	red	na
chrUn	50418872	50419566	695	CarSat1	red	na
chrUn	50419607	50420298	692	CarSat1	red	na
chrUn	50420340	50421050	711	CarSat1	red	na
chrUn	50421077	50423262	2186	CarSat1	red	na
chrUn	50423289	50423996	708	CarSat1	red	na
chrUn	50424027	50426681	2655	CarSat1	red	na
chrUn	50426711	50427689	979	CarSat1	red	na
chrUn	50427716	50428677	962	CarSat1	red	na
chrUn	50428693	50430208	1516	CarSat1	red	na
chrUn	50464023	50464904	882	CarSat1	red	na
chrUn	50464931	50469075	4145	CarSat1	red	na
chrUn	50469099	50480852	11754	CarSat1	red	na
chrUn	50480883	50481814	932	CarSat1	red	na
chrUn	50482253	50485968	3716	CarSat1	red	na
chrUn	50486001	50487699	1699	CarSat1	red	na
chrUn	50487720	50489182	1463	CarSat1	red	na
chrUn	50489211	50489337	127	CarSat1	red	na
chrUn	50489414	50493464	4051	CarSat1	red	na
chrUn	50493488	50494708	1221	CarSat1	red	na
chrUn	50495744	50496769	1026	CarSat1	red	na
chrUn	50496784	50498498	1715	CarSat1	red	na

chrUn	50499379	50499968	590	CarSat1	red	na
chrUn	50500001	50500714	714	CarSat1	red	na
chrUn	50500738	50501510	773	CarSat1	red	na
chrUn	50503755	50504886	1132	CarSat1	red	na
chrUn	50507048	50508116	1069	CarSat1	red	na
chrUn	50508146	50508869	724	CarSat1	red	na
chrUn	50508884	50509449	566	CarSat1	red	na
chrUn	50512535	50514108	1574	CarSat1	red	na
chrUn	50514138	50516093	1956	CarSat1	red	na
chrUn	50516225	50517502	1278	CarSat1	red	na
chrUn	50518143	50518782	640	CarSat1	red	na
chrUn	50518797	50519508	712	CarSat1	red	na
chrUn	50519538	50520192	655	CarSat1	red	na
chrUn	50521834	50523610	1777	CarSat1	red	na
chrUn	50523625	50524187	563	CarSat1	red	na
chrUn	50526766	50528240	1475	CarSat1	red	na
chrUn	50528270	50532381	4112	CarSat1	red	na
chrUn	50533016	50533269	254	SAT1_CF	blue	na
chrUn	50533281	50534409	1129	SAT1_CF	blue	na
chrUn	50536910	50538558	1649	SAT1_CF	blue	na
chrUn	50538598	50539957	1360	SAT1_CF	blue	na
chrUn	50542837	50544575	1739	SAT1_CF	blue	na
chrUn	50544618	50545208	591	SAT1_CF	blue	na
chrUn	50546233	50547636	1404	CarSat1	red	na
chrUn	50547660	50552330	4671	CarSat1	red	na
chrUn	50552360	50554318	1959	CarSat1	red	na
chrUn	50554330	50555549	1220	CarSat1	red	na
chrUn	50555704	50557232	1529	CarSat1	red	na
chrUn	50557244	50563125	5882	CarSat1	red	na
chrUn	50563149	50566946	3798	CarSat1	red	na
chrUn	50568044	50568536	493	CarSat1	red	na
chrUn	50568552	50569021	470	CarSat1	red	na
chrUn	50569048	50570984	1937	CarSat1	red	na
chrUn	50570997	50573449	2453	CarSat1	red	na
chrUn	50573476	50574185	710	CarSat1	red	na
chrUn	50574212	50575902	1691	CarSat1	red	na
chrUn	50575927	50576855	929	CarSat1	red	na
chrUn	50576892	50577588	697	CarSat1	red	na
chrUn	50616412	50617627	1216	Sat2CF	purple	na
chrUn	50616540	50617622	1083	Sat2CF	purple	na
chrUn	50678695	50679225	531	SAT1_CF	blue	na
chrUn	50679343	50679862	520	SAT1_CF	blue	na

chrUn	50679919	50680545	627	SAT1_CF	blue	na
chrUn	50680580	50681743	1164	SAT1_CF	blue	na
chrUn	50681758	50681894	137	SAT1_CF	blue	na
chrUn	50681990	50682509	520	SAT1_CF	blue	na
chrUn	50682566	50683192	627	SAT1_CF	blue	na
chrUn	50683227	50684390	1164	SAT1_CF	blue	na
chrUn	50684405	50684541	137	SAT1_CF	blue	na
chrUn	50684637	50685156	520	SAT1_CF	blue	na
chrUn	50685213	50686381	1169	SAT1_CF	blue	na
chrUn	50686467	50688159	1693	SAT1_CF	blue	na
chrUn	50688176	50688855	680	SAT1_CF	blue	na
chrUn	50688868	50688943	76	SAT1_CF	blue	na
chrUn	50688961	50689480	520	SAT1_CF	blue	na
chrUn	50689492	50690351	860	SAT1_CF	blue	na
chrUn	50690394	50691173	780	SAT1_CF	blue	na
chrUn	50691251	50692159	909	SAT1_CF	blue	na
chrUn	50692246	50692747	502	SAT1_CF	blue	na
chrUn	50692760	50693606	847	SAT1_CF	blue	na
chrUn	50693628	50694166	539	SAT1_CF	blue	na
chrUn	50694214	50695939	1726	SAT1_CF	blue	na
chrUn	50695969	50698234	2266	SAT1_CF	blue	na
chrUn	50698264	50698646	383	SAT1_CF	blue	na
chrUn	50698684	50699181	498	SAT1_CF	blue	na
chrUn	50699199	50699302	104	SAT1_CF	blue	na
chrUn	50699323	50699897	575	SAT1_CF	blue	na
chrUn	50699909	50700569	661	SAT1_CF	blue	na
chrUn	50700617	50701150	534	SAT1_CF	blue	na
chrUn	50701179	50701844	666	SAT1_CF	blue	na
chrUn	50701888	50702390	503	SAT1_CF	blue	na
chrUn	50702417	50703260	844	SAT1_CF	blue	na
chrUn	50703449	50704647	1199	SAT1_CF	blue	na
chrUn	50704665	50705335	671	SAT1_CF	blue	na
chrUn	50705378	50706993	1616	SAT1_CF	blue	na
chrUn	50707014	50707409	396	SAT1_CF	blue	na
chrUn	50707449	50707949	501	SAT1_CF	blue	na
chrUn	50707967	50708720	754	SAT1_CF	blue	na
chrUn	50709735	50710228	494	CarSat1	red	na
chrUn	50710244	50711178	935	CarSat1	red	na
chrUn	50711193	50711320	128	Carsat2	orange	na
chrUn	50711208	50714121	2914	CarSat1	red	na
chrUn	50714136	50714263	128	Carsat2	orange	na
chrUn	50714151	50716113	1963	CarSat1	red	na

chrUn	50716137	50718327	2191	CarSat1	red	na
chrUn	50718360	50719556	1197	CarSat1	red	na
chrUn	50719576	50720763	1188	CarSat1	red	na
chrUn	50720793	50723245	2453	CarSat1	red	na
chrUn	50723265	50724974	1710	CarSat1	red	na
chrUn	50724999	50725925	927	CarSat1	red	na
chrUn	50725940	50726066	127	Carsat2	orange	na
chrUn	50725955	50726659	705	CarSat1	red	na
chrUn	50726689	50727911	1223	CarSat1	red	na
chrUn	50727935	50730856	2922	CarSat1	red	na
chrUn	50730880	50731596	717	CarSat1	red	na
chrUn	50731620	50732230	611	CarSat1	red	na
chrUn	50732608	50733435	828	CarSat1	red	na
chrUn	50733465	50733951	487	CarSat1	red	na
chrUn	50733975	50736383	2409	CarSat1	red	na
chrUn	50736398	50736524	127	Carsat2	orange	na
chrUn	50736413	50738596	2184	CarSat1	red	na
chrUn	50738626	50739111	486	CarSat1	red	na
chrUn	50739135	50740194	1060	CarSat1	red	na
chrUn	50742711	50743783	1073	Sat2CF	purple	na
chrUn	50764200	50764714	515	Sat2CF	purple	na
chrUn	50764262	50765110	849	Sat2CF	purple	na
chrUn	50764486	50765185	700	Sat2CF	purple	na
chrUn	50791958	50793141	1184	Sat2CF	purple	na
chrUn	50799935	50801184	1250	Sat2CF	purple	na
chrUn	50803815	50804749	935	CarSat1	red	na
chrUn	50804938	50805507	570	CarSat1	red	na
chrUn	50812885	50815112	2228	CarSat1	red	na
chrUn	50815131	50815533	403	CarSat1	red	na
chrUn	50815562	50816937	1376	SAT1_CF	blue	na
chrUn	50817132	50817292	161	Sat2CF	purple	na
chrUn	50817165	50818113	949	Sat2CF	purple	na
chrUn	50817797	50818174	378	Sat2CF	purple	na
chrUn	50824190	50826417	2228	CarSat1	red	na
chrUn	50826436	50826838	403	CarSat1	red	na
chrUn	50826867	50828242	1376	SAT1_CF	blue	na
chrUn	50855835	50856695	861	CarSat1	red	na
chrUn	50856716	50856984	269	CarSat1	red	na
chrUn	50912100	50913270	1171	SAT1_CF	blue	na
chrUn	50913309	50914644	1336	SAT1_CF	blue	na
chrUn	50914711	50915231	521	SAT1_CF	blue	na
chrUn	50915244	50915646	403	SAT1_CF	blue	na

chrUn	50915686	50916188	503	SAT1_CF	blue	na
chrUn	50916201	50918082	1882	SAT1_CF	blue	na
chrUn	50918199	50918762	564	SAT1_CF	blue	na
chrUn	50918804	50919583	780	SAT1_CF	blue	na
chrUn	50919627	50920171	545	SAT1_CF	blue	na
chrUn	50920184	50920587	404	SAT1_CF	blue	na
chrUn	50920625	50921127	503	SAT1_CF	blue	na
chrUn	50921154	50922562	1409	SAT1_CF	blue	na
chrUn	50922600	50923828	1229	SAT1_CF	blue	na
chrUn	50923945	50924465	521	SAT1_CF	blue	na
chrUn	50924496	50924689	194	SAT1_CF	blue	na
chrUn	50924717	50926502	1786	SAT1_CF	blue	na
chrUn	50926555	50927056	502	SAT1_CF	blue	na
chrUn	50927067	50928176	1110	SAT1_CF	blue	na
chrUn	50928216	50928717	502	SAT1_CF	blue	na
chrUn	50928730	50929575	846	SAT1_CF	blue	na
chrUn	50929598	50930748	1151	SAT1_CF	blue	na
chrUn	50930778	50931338	561	SAT1_CF	blue	na
chrUn	50931425	50931925	501	SAT1_CF	blue	na
chrUn	50931938	50933240	1303	SAT1_CF	blue	na
chrUn	50933282	50934687	1406	SAT1_CF	blue	na
chrUn	50934698	50935388	691	SAT1_CF	blue	na
chrUn	50935432	50936128	697	SAT1_CF	blue	na
chrUn	50936142	50936918	777	SAT1_CF	blue	na
chrUn	50936971	50937492	522	SAT1_CF	blue	na
chrUn	50937519	50938836	1318	SAT1_CF	blue	na
chrUn	50938854	50939455	602	SAT1_CF	blue	na
chrUn	50939467	50940967	1501	SAT1_CF	blue	na
chrUn	50941053	50941548	496	SAT1_CF	blue	na
chrUn	50941568	50941903	336	SAT1_CF	blue	na
chrUn	50942943	50944718	1776	CarSat1	red	na
chrUn	50944748	50945460	713	CarSat1	red	na
chrUn	50945490	50947672	2183	CarSat1	red	na
chrUn	50947703	50949159	1457	CarSat1	red	na
chrUn	50949174	50952345	3172	CarSat1	red	na
chrUn	50952378	50955966	3589	CarSat1	red	na
chrUn	50959048	50960465	1418	CarSat1	red	na
chrUn	50960489	50961205	717	CarSat1	red	na
chrUn	50961229	50961942	714	CarSat1	red	na
chrUn	50961966	50962680	715	CarSat1	red	na
chrUn	50962704	50964818	2115	CarSat1	red	na
chrUn	50965292	50966350	1059	CarSat1	red	na

chrUn	50966374	50967338	965	CarSat1	red	na
chrUn	50967363	50969092	1730	CarSat1	red	na
chrUn	50969107	50970570	1464	CarSat1	red	na
chrUn	50970585	50971307	723	CarSat1	red	na
chrUn	50971322	50972044	723	CarSat1	red	na
chrUn	50972059	50972781	723	CarSat1	red	na
chrUn	50972796	50973731	936	CarSat1	red	na
chrUn	50973747	50975465	1719	CarSat1	red	na
chrUn	50975476	50976207	732	CarSat1	red	na
chrUn	50977225	50978726	1502	CarSat1	red	na
chrUn	50980616	50981517	902	CarSat1	red	na
chrUn	50981547	50982874	1328	CarSat1	red	na
chrUn	50983897	50985063	1167	CarSat1	red	na
chrUn	50985090	50985678	589	CarSat1	red	na
chrUn	50985810	50986427	618	CarSat1	red	na
chrUn	50986457	50986943	487	CarSat1	red	na
chrUn	50986967	50987998	1032	CarSat1	red	na
chrUn	50988034	50989534	1501	CarSat1	red	na
chrUn	50989564	50990271	708	CarSat1	red	na
chrUn	50990286	50990412	127	Carsat2	orange	na
chrUn	50990301	50991012	712	CarSat1	red	na
chrUn	50991042	50992859	1818	CarSat1	red	na
chrUn	50993251	50993736	486	CarSat1	red	na
chrUn	50993760	50994478	719	CarSat1	red	na
chrUn	50994502	50996165	1664	CarSat1	red	na
chrUn	50996994	50999074	2081	CarSat1	red	na
chrUn	51000629	51002146	1518	CarSat1	red	na
chrUn	51004454	51005747	1294	CarSat1	red	na
chrUn	51007065	51007548	484	CarSat1	red	na
chrUn	51007578	51008940	1363	CarSat1	red	na
chrUn	51012023	51013090	1068	CarSat1	red	na
chrUn	51013114	51014411	1298	CarSat1	red	na
chrUn	51015696	51017792	2097	CarSat1	red	na
chrUn	51018163	51018866	704	CarSat1	red	na
chrUn	51018900	51020775	1876	CarSat1	red	na
chrUn	51056451	51059302	2852	CarSat1	red	na
chrUn	51059327	51060777	1451	CarSat1	red	na
chrUn	51060802	51061999	1198	CarSat1	red	na
chrUn	51062026	51062459	434	CarSat1	red	na
chrUn	51062725	51064208	1484	CarSat1	red	na
chrUn	51064235	51066671	2437	CarSat1	red	na
chrUn	51066696	51069364	2669	CarSat1	red	na

chrUn	51069391	51071090	1700	CarSat1	red	na
chrUn	51071115	51071823	709	CarSat1	red	na
chrUn	51071848	51073032	1185	CarSat1	red	na
chrUn	51073059	51074022	964	CarSat1	red	na
chrUn	51074038	51076224	2187	CarSat1	red	na
chrUn	51077400	51079430	2031	CarSat1	red	na
chrUn	51081007	51082793	1787	CarSat1	red	na
chrUn	51082809	51083277	469	CarSat1	red	na
chrUn	51083304	51084014	711	CarSat1	red	na
chrUn	51084044	51084751	708	CarSat1	red	na
chrUn	51084776	51085487	712	CarSat1	red	na
chrUn	51085514	51086960	1447	CarSat1	red	na
chrUn	51086987	51088190	1204	CarSat1	red	na
chrUn	51088217	51088972	756	CarSat1	red	na
chrUn	51098841	51099222	382	Sat2CF	purple	na
chrUn	51098900	51099744	845	Sat2CF	purple	na
chrUn	51098976	51099830	855	Sat2CF	purple	na
chrUn	51099705	51099865	161	Sat2CF	purple	na
chrUn	51100059	51101437	1379	SAT1_CF	blue	na
chrUn	51101466	51103339	1874	CarSat1	red	na
chrUn	51103360	51104217	858	CarSat1	red	na
chrUn	51104238	51105737	1500	CarSat1	red	na
chrUn	51105757	51106430	674	CarSat1	red	na
chrUn	51112324	51112381	58	CarSat1	red	na
chrUn	51112395	51112796	402	CarSat1	red	na
chrUn	51112826	51115200	2375	CarSat1	red	na
chrUn	51121637	51123083	1447	CarSat1	red	na
chrUn	51123435	51124679	1245	CarSat1	red	na
chrUn	51124711	51125590	880	CarSat1	red	na
chrUn	51125585	51127129	1545	SAT1_CF	blue	na
chrUn	51127171	51128220	1050	SAT1_CF	blue	na
chrUn	51134295	51134972	678	SAT1_CF	blue	na
chrUn	51134990	51135780	791	SAT1_CF	blue	na
chrUn	51135809	51136322	514	SAT1_CF	blue	na
chrUn	51136400	51137976	1577	SAT1_CF	blue	na
chrUn	51138020	51139379	1360	SAT1_CF	blue	na
chrUn	51139394	51141538	2145	SAT1_CF	blue	na
chrUn	51141557	51142559	1003	SAT1_CF	blue	na
chrUn	51142564	51143749	1186	CarSat1	red	na
chrUn	51143764	51145276	1513	CarSat1	red	na
chrUn	51145297	51146283	987	CarSat1	red	na
chrUn	51151027	51151468	442	SAT1_CF	blue	na

chrUn	51151545	51152140	596	SAT1_CF	blue	na
chrUn	51153593	51154772	1180	SAT1_CF	blue	na
chrUn	51154798	51155463	666	SAT1_CF	blue	na
chrUn	51155481	51155978	498	SAT1_CF	blue	na
chrUn	51156022	51156145	124	SAT1_CF	blue	na
chrUn	51156859	51157015	157	SAT1_CF	blue	na
chrUn	51157029	51158462	1434	SAT1_CF	blue	na
chrUn	51158504	51159362	859	SAT1_CF	blue	na
chrUn	51159374	51160173	800	SAT1_CF	blue	na
chrUn	51160185	51160860	676	SAT1_CF	blue	na
chrUn	51160872	51161371	500	SAT1_CF	blue	na
chrUn	51161411	51161592	182	SAT1_CF	blue	na
chrUn	51161774	51162163	390	SAT1_CF	blue	na
chrUn	51163301	51164025	725	SAT1_CF	blue	na
chrUn	51164037	51164146	110	SAT1_CF	blue	na
chrUn	51164166	51164435	270	SAT1_CF	blue	na
chrUn	51165153	51165779	627	SAT1_CF	blue	na
chrUn	51165801	51165939	139	SAT1_CF	blue	na
chrUn	51165952	51166453	502	SAT1_CF	blue	na
chrUn	51166466	51167148	683	SAT1_CF	blue	na
chrUn	51167160	51169326	2167	SAT1_CF	blue	na
chrUn	51169371	51169872	502	SAT1_CF	blue	na
chrUn	51169884	51169992	109	SAT1_CF	blue	na
chrUn	51170015	51170891	877	SAT1_CF	blue	na
chrUn	51172055	51172619	565	SAT1_CF	blue	na
chrUn	51173463	51173611	149	SAT1_CF	blue	na
chrUn	51173624	51174229	606	SAT1_CF	blue	na
chrUn	51175796	51176382	587	SAT1_CF	blue	na
chrUn	51176397	51178671	2275	SAT1_CF	blue	na
chrUn	51178714	51179382	669	SAT1_CF	blue	na
chrUn	51179394	51180591	1198	SAT1_CF	blue	na
chrUn	51184540	51187042	2503	SAT1_CF	blue	na
chrUn	51187095	51187385	291	SAT1_CF	blue	na
chrUn	51194162	51195421	1260	SAT1_CF	blue	na
chrUn	51196938	51197259	322	SAT1_CF	blue	na
chrUn	51197351	51197859	509	SAT1_CF	blue	na
chrUn	51199049	51200790	1742	CarSat1	red	na
chrUn	51200805	51200931	127	Carsat2	orange	na
chrUn	51200820	51210662	9843	CarSat1	red	na
chrUn	51210683	51212323	1641	CarSat1	red	na
chrUn	51236947	51237637	691	CarSat1	red	na
chrUn	51237661	51238868	1208	CarSat1	red	na

chrUn	51238879	51241548	2670	CarSat1	red	na
chrUn	51241578	51244026	2449	CarSat1	red	na
chrUn	51244037	51245503	1467	CarSat1	red	na
chrUn	51245524	51246978	1455	CarSat1	red	na
chrUn	51246999	51248453	1455	CarSat1	red	na
chrUn	51248464	51249198	735	CarSat1	red	na
chrUn	51249214	51253043	3830	CarSat1	red	na
chrUn	51254045	51255655	1611	SAT1_CF	blue	na
chrUn	51255668	51257090	1423	SAT1_CF	blue	na
chrUn	51263360	51265993	2634	SAT1_CF	blue	na
chrUn	51266036	51267251	1216	SAT1_CF	blue	na
chrUn	51267272	51269058	1787	SAT1_CF	blue	na
chrUn	51269071	51270320	1250	SAT1_CF	blue	na
chrUn	51270325	51271506	1182	CarSat1	red	na
chrUn	51271525	51273033	1509	CarSat1	red	na
chrUn	51273059	51274014	956	CarSat1	red	na
chrUn	51284783	51285702	920	CarSat1	red	na
chrUn	51285723	51286700	978	CarSat1	red	na
chrUn	51286730	51287300	571	CarSat1	red	na
chrUn	51287900	51288546	647	CarSat1	red	na
chrUn	51288571	51290571	2001	CarSat1	red	na
chrUn	51290598	51291444	847	CarSat1	red	na
chrUn	51291877	51292947	1071	CarSat1	red	na
chrUn	51293383	51293970	588	CarSat1	red	na
chrUn	51293997	51294933	937	CarSat1	red	na
chrUn	51297046	51298915	1870	CarSat1	red	na
chrUn	51298940	51299683	744	CarSat1	red	na
chrUn	51299710	51300421	712	CarSat1	red	na
chrUn	51300448	51302537	2090	CarSat1	red	na
chrUn	51302556	51303695	1140	CarSat1	red	na
chrUn	51303879	51304731	853	CarSat1	red	na
chrUn	51304747	51306205	1459	CarSat1	red	na
chrUn	51306221	51307182	962	CarSat1	red	na
chrUn	51307209	51307919	711	CarSat1	red	na
chrUn	51307946	51308790	845	CarSat1	red	na
chrUn	51310862	51313339	2478	CarSat1	red	na
chrUn	51313355	51314812	1458	CarSat1	red	na
chrUn	51314828	51315396	569	CarSat1	red	na
chrUn	51316078	51317175	1098	CarSat1	red	na
chrUn	51317780	51319010	1231	CarSat1	red	na
chrUn	51319053	51319608	556	CarSat1	red	na
chrUn	51320255	51321241	987	CarSat1	red	na

chrUn	51322268	51322935	668	CarSat1	red	na
chrUn	51322962	51323671	710	CarSat1	red	na
chrUn	51323698	51324674	977	CarSat1	red	na
chrUn	51324704	51327114	2411	CarSat1	red	na
chrUn	51327130	51328092	963	CarSat1	red	na
chrUn	51328119	51330304	2186	CarSat1	red	na
chrUn	51330331	51333521	3191	CarSat1	red	na
chrUn	51333558	51334491	934	CarSat1	red	na
chrUn	51334515	51335737	1223	CarSat1	red	na
chrUn	51335774	51337677	1904	CarSat1	red	na
chrUn	51339488	51340627	1140	CarSat1	red	na
chrUn	51340654	51341828	1175	CarSat1	red	na
chrUn	51341828	51342145	318	SAT1_CF	blue	na
chrUn	51342172	51342368	197	SAT1_CF	blue	na
chrUn	51343332	51344296	965	CarSat1	red	na
chrUn	51344324	51345531	1208	CarSat1	red	na
chrUn	51345556	51350691	5136	CarSat1	red	na
chrUn	51350707	51351941	1235	CarSat1	red	na
chrUn	51351971	51352649	679	CarSat1	red	na
chrUn	51352688	51353157	470	CarSat1	red	na
chrUn	51353173	51354232	1060	CarSat1	red	na
chrUn	51359624	51359670	47	Sat111CF	na	na
chrUn	51359960	51360200	241	Sat5CF	stalk	na
chrUn	51359987	51360200	214	Sat111CF	na	na
chrUn	51441067	51441771	705	CarSat1	red	na
chrUn	51441801	51443383	1583	CarSat1	red	na
chrUn	51443404	51444195	792	CarSat1	red	na
chrUn	51444277	51445133	857	CarSat1	red	na
chrUn	51445154	51446370	1217	CarSat1	red	na
chrUn	51446442	51447456	1015	SAT1_CF	blue	na
chrUn	51447451	51448175	725	CarSat1	red	na
chrUn	51448207	51448633	427	CarSat1	red	na
chrUn	51450519	51450681	163	Sat2CF	purple	na
chrUn	51450547	51450906	360	Sat2CF	purple	na
chrUn	51450586	51451370	785	Sat2CF	purple	na
chrUn	51466803	51467928	1126	Sat2CF	purple	na
chrUn	51467478	51468009	532	Sat2CF	purple	na
chrUn	51503906	51504163	258	Sat11CF	na	na
chrUn	51504215	51504360	146	Sat11CF	na	na
chrUn	51504411	51504791	381	Sat11CF	na	na
chrUn	51514138	51515058	921	Sat2CF	purple	na
chrUn	51514951	51515129	179	Sat2CF	purple	na

chrUn	51529948	51530112	165	Sat2CF	purple	na
chrUn	51529968	51530806	839	Sat2CF	purple	na
chrUn	51530019	51530745	727	Sat2CF	purple	na
chrUn	51590345	51591056	712	CarSat1	red	na
chrUn	51591251	51592868	1618	CarSat1	red	na
chrUn	51592901	51593601	701	CarSat1	red	na
chrUn	51593615	51595552	1938	CarSat1	red	na
chrUn	51595573	51596190	618	CarSat1	red	na
chrUn	51596633	51599759	3127	CarSat1	red	na
chrUn	51602272	51603023	752	CarSat1	red	na
chrUn	51603044	51603757	714	CarSat1	red	na
chrUn	51603778	51604489	712	CarSat1	red	na
chrUn	51604516	51605226	711	CarSat1	red	na
chrUn	51605253	51605963	711	CarSat1	red	na
chrUn	51605990	51606701	712	CarSat1	red	na
chrUn	51606723	51607441	719	CarSat1	red	na
chrUn	51607468	51607908	441	CarSat1	red	na
chrUn	51608389	51610135	1747	CarSat1	red	na
chrUn	51611390	51613380	1991	CarSat1	red	na
chrUn	51613407	51614348	942	CarSat1	red	na
chrUn	51615439	51617910	2472	CarSat1	red	na
chrUn	51619089	51620564	1476	CarSat1	red	na
chrUn	51621676	51622814	1139	SAT1_CF	blue	na
chrUn	51622825	51623428	604	SAT1_CF	blue	na
chrUn	51623450	51624640	1191	SAT1_CF	blue	na
chrUn	51624654	51626938	2285	SAT1_CF	blue	na
chrUn	51626949	51628450	1502	SAT1_CF	blue	na
chrUn	51638294	51638872	579	SAT1_CF	blue	na
chrUn	51638888	51639055	168	SAT1_CF	blue	na
chrUn	51639107	51639593	487	SAT1_CF	blue	na
chrUn	51639608	51639802	195	SAT1_CF	blue	na
chrUn	51639819	51642657	2839	SAT1_CF	blue	na
chrUn	51642700	51643905	1206	SAT1_CF	blue	na
chrUn	51643948	51645092	1145	SAT1_CF	blue	na
chrUn	51645108	51647118	2011	SAT1_CF	blue	na
chrUn	51647200	51647943	744	SAT1_CF	blue	na
chrUn	51651379	51652017	639	CarSat1	red	na
chrUn	51652032	51652159	128	Carsat2	orange	na
chrUn	51652047	51652752	706	CarSat1	red	na
chrUn	51652782	51653487	706	CarSat1	red	na
chrUn	51653502	51653625	124	Carsat2	orange	na
chrUn	51653517	51654224	708	CarSat1	red	na

chrUn	51654239	51654364	126	Carsat2	orange	na
chrUn	51654254	51654959	706	CarSat1	red	na
chrUn	51654989	51655696	708	CarSat1	red	na
chrUn	51655711	51655837	127	Carsat2	orange	na
chrUn	51655726	51657908	2183	CarSat1	red	na
chrUn	51657923	51658049	127	Carsat2	orange	na
chrUn	51657938	51658644	707	CarSat1	red	na
chrUn	51658674	51659380	707	CarSat1	red	na
chrUn	51659395	51659522	128	Carsat2	orange	na
chrUn	51659410	51660119	710	CarSat1	red	na
chrUn	51660134	51660260	127	Carsat2	orange	na
chrUn	51660149	51661376	1228	CarSat1	red	na
chrUn	51661409	51662855	1447	CarSat1	red	na
chrUn	51662888	51664827	1940	CarSat1	red	na
chrUn	51664848	51667025	2178	CarSat1	red	na
chrUn	51667046	51669233	2188	CarSat1	red	na
chrUn	51669254	51670224	971	CarSat1	red	na
chrUn	51670249	51671910	1662	CarSat1	red	na
chrUn	51671924	51672050	127	Carsat2	orange	na
chrUn	51671939	51674869	2931	CarSat1	red	na
chrUn	51674884	51675011	128	Carsat2	orange	na
chrUn	51674899	51675606	708	CarSat1	red	na
chrUn	51675621	51675748	128	Carsat2	orange	na
chrUn	51675636	51676346	711	CarSat1	red	na
chrUn	51676361	51676488	128	Carsat2	orange	na
chrUn	51676376	51677821	1446	CarSat1	red	na
chrUn	51677836	51677963	128	Carsat2	orange	na
chrUn	51677851	51678557	707	CarSat1	red	na
chrUn	51678587	51680240	1654	CarSat1	red	na
chrUn	51728528	51729162	635	SAT1_CF	blue	na
chrUn	51729206	51730670	1465	SAT1_CF	blue	na
chrUn	51730682	51731256	575	SAT1_CF	blue	na
chrUn	51731278	51731374	97	SAT1_CF	blue	na
chrUn	51731398	51732229	832	SAT1_CF	blue	na
chrUn	51732243	51733115	873	SAT1_CF	blue	na
chrUn	51733145	51733688	544	SAT1_CF	blue	na
chrUn	51733730	51735155	1426	SAT1_CF	blue	na
chrUn	51735177	51735284	108	SAT1_CF	blue	na
chrUn	51735297	51735796	500	SAT1_CF	blue	na
chrUn	51735864	51736001	138	SAT1_CF	blue	na
chrUn	51736034	51736540	507	SAT1_CF	blue	na
chrUn	51736571	51738031	1461	SAT1_CF	blue	na

chrUn	51738043	51738665	623	SAT1_CF	blue	na
chrUn	51738678	51739678	1001	SAT1_CF	blue	na
chrUn	51739721	51740395	675	SAT1_CF	blue	na
chrUn	51740408	51740890	483	SAT1_CF	blue	na
chrUn	51740950	51741405	456	SAT1_CF	blue	na
chrUn	51741416	51742279	864	SAT1_CF	blue	na
chrUn	51742317	51742861	545	SAT1_CF	blue	na
chrUn	51742902	51743764	863	SAT1_CF	blue	na
chrUn	51743776	51744444	669	SAT1_CF	blue	na
chrUn	51744456	51745088	633	SAT1_CF	blue	na
chrUn	51745159	51745702	544	SAT1_CF	blue	na
chrUn	51745745	51746291	547	SAT1_CF	blue	na
chrUn	51746335	51747332	998	SAT1_CF	blue	na
chrUn	51747375	51748054	680	SAT1_CF	blue	na
chrUn	51748066	51748873	808	SAT1_CF	blue	na
chrUn	51748900	51749420	521	SAT1_CF	blue	na
chrUn	51749441	51751523	2083	SAT1_CF	blue	na
chrUn	51751540	51751652	113	SAT1_CF	blue	na
chrUn	51751665	51752347	683	SAT1_CF	blue	na
chrUn	51752367	51752860	494	SAT1_CF	blue	na
chrUn	51752902	51753304	403	SAT1_CF	blue	na
chrUn	51753315	51753822	508	SAT1_CF	blue	na
chrUn	51753869	51754942	1074	SAT1_CF	blue	na
chrUn	51754991	51755536	546	SAT1_CF	blue	na
chrUn	51755549	51755665	117	SAT1_CF	blue	na
chrUn	51755677	51756348	672	SAT1_CF	blue	na
chrUn	51756371	51756871	501	SAT1_CF	blue	na
chrUn	51756918	51757297	380	SAT1_CF	blue	na
chrUn	51758302	51758827	526	SAT1_CF	blue	na
chrUn	51758856	51760356	1501	SAT1_CF	blue	na
chrUn	51760399	51761714	1316	SAT1_CF	blue	na
chrUn	51761727	51762404	678	SAT1_CF	blue	na
chrUn	51762446	51763939	1494	SAT1_CF	blue	na
chrUn	51764423	51765480	1058	SAT1_CF	blue	na
chrUn	51765508	51765643	136	SAT1_CF	blue	na
chrUn	51765710	51766208	499	SAT1_CF	blue	na
chrUn	51766251	51766434	184	SAT1_CF	blue	na
chrUn	51766473	51767565	1093	SAT1_CF	blue	na
chrUn	51767609	51768237	629	SAT1_CF	blue	na
chrUn	51768275	51768773	499	SAT1_CF	blue	na
chrUn	51768915	51769458	544	SAT1_CF	blue	na
chrUn	51769529	51770002	474	SAT1_CF	blue	na

chrUn	51770016	51770131	116	SAT1_CF	blue	na
chrUn	51770144	51770415	272	SAT1_CF	blue	na
chrUn	51771426	51773144	1719	SAT1_CF	blue	na
chrUn	51773187	51773611	425	SAT1_CF	blue	na
chrUn	51773624	51774252	629	SAT1_CF	blue	na
chrUn	51774268	51774809	542	SAT1_CF	blue	na
chrUn	51774822	51776019	1198	SAT1_CF	blue	na
chrUn	51776032	51776882	851	SAT1_CF	blue	na
chrUn	51776924	51777621	698	SAT1_CF	blue	na
chrUn	51777635	51778179	545	SAT1_CF	blue	na
chrUn	51778216	51778728	513	SAT1_CF	blue	na
chrUn	51778834	51779463	630	SAT1_CF	blue	na
chrUn	51779475	51780315	841	SAT1_CF	blue	na
chrUn	51780327	51781866	1540	SAT1_CF	blue	na
chrUn	51781892	51782596	705	SAT1_CF	blue	na
chrUn	51782609	51783725	1117	SAT1_CF	blue	na
chrUn	51783788	51784344	557	SAT1_CF	blue	na
chrUn	51784356	51785030	675	SAT1_CF	blue	na
chrUn	51785073	51786038	966	SAT1_CF	blue	na
chrUn	51786093	51787119	1027	SAT1_CF	blue	na
chrUn	51787155	51787649	495	SAT1_CF	blue	na
chrUn	51787669	51788404	736	SAT1_CF	blue	na
chrUn	51789432	51790578	1147	SAT1_CF	blue	na
chrUn	51790601	51790706	106	SAT1_CF	blue	na
chrUn	51790719	51791465	747	SAT1_CF	blue	na
chrUn	51791492	51791993	502	SAT1_CF	blue	na
chrUn	51792039	51792967	929	SAT1_CF	blue	na
chrUn	51792984	51793266	283	SAT1_CF	blue	na
chrUn	51793308	51793852	545	SAT1_CF	blue	na
chrUn	51793894	51794441	548	SAT1_CF	blue	na
chrUn	51794498	51795452	955	SAT1_CF	blue	na
chrUn	51795497	51796021	525	SAT1_CF	blue	na
chrUn	51796042	51796320	279	SAT1_CF	blue	na
chrUn	51796362	51797842	1481	SAT1_CF	blue	na
chrUn	51797854	51798544	691	SAT1_CF	blue	na
chrUn	51798558	51799459	902	SAT1_CF	blue	na
chrUn	51799511	51800139	629	SAT1_CF	blue	na
chrUn	51800163	51800269	107	SAT1_CF	blue	na
chrUn	51800281	51800783	503	SAT1_CF	blue	na
chrUn	51800849	51800984	136	SAT1_CF	blue	na
chrUn	51801012	51801977	966	SAT1_CF	blue	na
chrUn	51802029	51802201	173	SAT1_CF	blue	na

chrUn	51802245	51803579	1335	SAT1_CF	blue	na
chrUn	51803591	51804261	671	SAT1_CF	blue	na
chrUn	51804286	51804788	503	SAT1_CF	blue	na
chrUn	51804854	51804990	137	SAT1_CF	blue	na
chrUn	51805005	51805527	523	SAT1_CF	blue	na
chrUn	51805607	51806848	1242	SAT1_CF	blue	na
chrUn	51806879	51807398	520	SAT1_CF	blue	na
chrUn	51807515	51807868	354	SAT1_CF	blue	na
chrUn	51807880	51808849	970	SAT1_CF	blue	na
chrUn	51808892	51809434	543	SAT1_CF	blue	na
chrUn	51809530	51810203	674	SAT1_CF	blue	na
chrUn	51810216	51810718	503	SAT1_CF	blue	na
chrUn	51810805	51811159	355	SAT1_CF	blue	na
chrUn	51811172	51811715	544	SAT1_CF	blue	na
chrUn	51811740	51812060	321	SAT1_CF	blue	na
chrUn	51812071	51812613	543	SAT1_CF	blue	na
chrUn	51812655	51813513	859	SAT1_CF	blue	na
chrUn	51813525	51814171	647	SAT1_CF	blue	na
chrUn	51814184	51814946	763	SAT1_CF	blue	na
chrUn	51814958	51815531	574	SAT1_CF	blue	na
chrUn	51815554	51815660	107	SAT1_CF	blue	na
chrUn	51815673	51816218	546	SAT1_CF	blue	na
chrUn	51816295	51817351	1057	SAT1_CF	blue	na
chrUn	51817464	51818028	565	SAT1_CF	blue	na
chrUn	51852512	51853698	1187	CarSat1	red	na
chrUn	51879695	51880870	1176	Sat2CF	purple	na
chrUn	51881257	51882343	1087	Sat2CF	purple	na
chrUn	51884040	51887407	3368	CarSat1	red	na
chrUn	51887440	51888143	704	CarSat1	red	na
chrUn	51888176	51888881	706	CarSat1	red	na
chrUn	51888914	51889414	501	CarSat1	red	na
chrUn	51889429	51890359	931	CarSat1	red	na
chrUn	51890395	51892570	2176	CarSat1	red	na
chrUn	51892603	51893088	486	CarSat1	red	na
chrUn	51893122	51894575	1454	CarSat1	red	na
chrUn	51894590	51896992	2403	CarSat1	red	na
chrUn	51897025	51899737	2713	CarSat1	red	na
chrUn	51899752	51900473	722	CarSat1	red	na
chrUn	51900488	51901424	937	CarSat1	red	na
chrUn	51901446	51903004	1559	CarSat1	red	na
chrUn	51903037	51903523	487	CarSat1	red	na
chrUn	51903553	51904275	723	CarSat1	red	na

chrUn	51904290	51905754	1465	CarSat1	red	na
chrUn	51905769	51906722	954	CarSat1	red	na
chrUn	51907305	51907975	671	CarSat1	red	na
chrUn	51907990	51908916	927	CarSat1	red	na
chrUn	51908949	51910648	1700	CarSat1	red	na
chrUn	51910669	51911137	469	CarSat1	red	na
chrUn	51911162	51911864	703	CarSat1	red	na
chrUn	51911897	51913277	1381	CarSat1	red	na
chrUn	51942409	51943184	776	Sat10CF	na	na
chrUn	51962549	51963491	943	CarSat1	red	na
chrUn	51963516	51963985	470	CarSat1	red	na
chrUn	51964006	51964451	446	CarSat1	red	na
chrUn	51964477	51965699	1223	CarSat1	red	na
chrUn	51965732	51967661	1930	CarSat1	red	na
chrUn	51967686	51968131	446	CarSat1	red	na
chrUn	51968146	51968273	128	Carsat2	orange	na
chrUn	51968161	51968868	708	CarSat1	red	na
chrUn	51968883	51969009	127	Carsat2	orange	na
chrUn	51968898	51970353	1456	CarSat1	red	na
chrUn	51970368	51970491	124	Carsat2	orange	na
chrUn	51970383	51971611	1229	CarSat1	red	na
chrUn	51971644	51972570	927	CarSat1	red	na
chrUn	51972585	51972711	127	Carsat2	orange	na
chrUn	51972600	51977992	5393	CarSat1	red	na
chrUn	51978015	51980205	2191	CarSat1	red	na
chrUn	51980216	51985835	5620	CarSat1	red	na
chrUn	51985865	51986571	707	CarSat1	red	na
chrUn	51986601	51987308	708	CarSat1	red	na
chrUn	51987338	51987823	486	CarSat1	red	na
chrUn	51987847	51990262	2416	CarSat1	red	na
chrUn	51990277	51990403	127	Carsat2	orange	na
chrUn	51990292	51990979	688	CarSat1	red	na
chrUn	52044367	52045262	896	CarSat1	red	na
chrUn	52045278	52046000	723	CarSat1	red	na
chrUn	52046016	52046737	722	CarSat1	red	na
chrUn	52046753	52054573	7821	CarSat1	red	na
chrUn	52064580	52066164	1585	CarSat1	red	na
chrUn	52066189	52069533	3345	CarSat1	red	na
chrUn	52069560	52072733	3174	CarSat1	red	na
chrUn	52072760	52074205	1446	CarSat1	red	na
chrUn	52074230	52077575	3346	CarSat1	red	na
chrUn	52077602	52079791	2190	CarSat1	red	na

chrUn	52079818	52082763	2946	CarSat1	red	na
chrUn	52087024	52087822	799	Sat2CF	purple	na
chrUn	52087714	52087838	125	Sat2CF	purple	na
chrUn	52132343	52133519	1177	Sat2CF	purple	na
chrUn	52179515	52180137	623	SAT1_CF	blue	na
chrUn	52180171	52180837	667	SAT1_CF	blue	na
chrUn	52180882	52182140	1259	SAT1_CF	blue	na
chrUn	52182220	52182537	318	SAT1_CF	blue	na
chrUn	52182592	52183094	503	SAT1_CF	blue	na
chrUn	52183107	52183182	76	SAT1_CF	blue	na
chrUn	52183201	52184410	1210	SAT1_CF	blue	na
chrUn	52184453	52185050	598	SAT1_CF	blue	na
chrUn	52185066	52186815	1750	SAT1_CF	blue	na
chrUn	52186902	52187404	503	SAT1_CF	blue	na
chrUn	52187416	52187521	106	SAT1_CF	blue	na
chrUn	52187545	52188347	803	SAT1_CF	blue	na
chrUn	52188360	52188468	109	SAT1_CF	blue	na
chrUn	52188489	52189238	750	SAT1_CF	blue	na
chrUn	52189260	52189911	652	SAT1_CF	blue	na
chrUn	52189923	52190465	543	SAT1_CF	blue	na
chrUn	52190509	52191515	1007	SAT1_CF	blue	na
chrUn	52191608	52192518	911	SAT1_CF	blue	na
chrUn	52192635	52193176	542	SAT1_CF	blue	na
chrUn	52193188	52193301	114	SAT1_CF	blue	na
chrUn	52193317	52193864	548	SAT1_CF	blue	na
chrUn	52193907	52195535	1629	SAT1_CF	blue	na
chrUn	52195546	52195947	402	SAT1_CF	blue	na
chrUn	52195968	52196488	521	SAT1_CF	blue	na
chrUn	52196501	52197175	675	SAT1_CF	blue	na
chrUn	52197195	52197860	666	SAT1_CF	blue	na
chrUn	52197903	52198534	632	SAT1_CF	blue	na
chrUn	52198599	52199159	561	SAT1_CF	blue	na
chrUn	52199265	52199747	483	SAT1_CF	blue	na
chrUn	52199760	52199840	81	SAT1_CF	blue	na
chrUn	52199854	52200368	515	SAT1_CF	blue	na
chrUn	52200380	52201265	886	SAT1_CF	blue	na
chrUn	52201287	52202137	851	SAT1_CF	blue	na
chrUn	52202150	52204047	1898	SAT1_CF	blue	na
chrUn	52204062	52205005	944	SAT1_CF	blue	na
chrUn	52205018	52205865	848	SAT1_CF	blue	na
chrUn	52205907	52206992	1086	SAT1_CF	blue	na
chrUn	52207014	52207113	100	SAT1_CF	blue	na

chrUn	52207134	52207596	463	SAT1_CF	blue	na
chrUn	52247740	52248403	664	SAT1_CF	blue	na
chrUn	52248415	52248922	508	SAT1_CF	blue	na
chrUn	52251761	52252874	1114	SAT1_CF	blue	na
chrUn	52253597	52254098	502	SAT1_CF	blue	na
chrUn	52254111	52254784	674	SAT1_CF	blue	na
chrUn	52254828	52255338	511	SAT1_CF	blue	na
chrUn	52255408	52256037	630	SAT1_CF	blue	na
chrUn	52256060	52256913	854	SAT1_CF	blue	na
chrUn	52256992	52257958	967	SAT1_CF	blue	na
chrUn	52258675	52259086	412	SAT1_CF	blue	na
chrUn	52259129	52259682	554	SAT1_CF	blue	na
chrUn	52259694	52260010	317	SAT1_CF	blue	na
chrUn	52262352	52264422	2071	SAT1_CF	blue	na
chrUn	52264435	52265116	682	SAT1_CF	blue	na
chrUn	52265128	52265811	684	SAT1_CF	blue	na
chrUn	52265822	52266314	493	SAT1_CF	blue	na
chrUn	52266328	52267102	775	SAT1_CF	blue	na
chrUn	52268002	52268859	858	SAT1_CF	blue	na
chrUn	52268903	52269428	526	SAT1_CF	blue	na
chrUn	52270031	52270631	601	SAT1_CF	blue	na
chrUn	52270644	52271146	503	SAT1_CF	blue	na
chrUn	52271159	52271757	599	SAT1_CF	blue	na
chrUn	52271769	52272999	1231	SAT1_CF	blue	na
chrUn	52273042	52273553	512	SAT1_CF	blue	na
chrUn	52273581	52273737	157	SAT1_CF	blue	na
chrUn	52273763	52274395	633	SAT1_CF	blue	na
chrUn	52325497	52327023	1527	SAT1_CF	blue	na
chrUn	52327068	52327746	679	SAT1_CF	blue	na
chrUn	52327758	52328959	1202	SAT1_CF	blue	na
chrUn	52328997	52329716	720	SAT1_CF	blue	na
chrUn	52329758	52330300	543	SAT1_CF	blue	na
chrUn	52330342	52330889	548	SAT1_CF	blue	na
chrUn	52330921	52331465	545	SAT1_CF	blue	na
chrUn	52331581	52331933	353	SAT1_CF	blue	na
chrUn	52331946	52333303	1358	SAT1_CF	blue	na
chrUn	52333315	52334518	1204	SAT1_CF	blue	na
chrUn	52334563	52335788	1226	SAT1_CF	blue	na
chrUn	52335800	52336743	944	SAT1_CF	blue	na
chrUn	52336800	52337320	521	SAT1_CF	blue	na
chrUn	52337388	52339428	2041	SAT1_CF	blue	na
chrUn	52339470	52340142	673	SAT1_CF	blue	na

chrUn	52340155	52340655	501	SAT1_CF	blue	na
chrUn	52340721	52340856	136	SAT1_CF	blue	na
chrUn	52340871	52341395	525	SAT1_CF	blue	na
chrUn	52341476	52342262	787	SAT1_CF	blue	na
chrUn	52342285	52342390	106	SAT1_CF	blue	na
chrUn	52342403	52342922	520	SAT1_CF	blue	na
chrUn	52342941	52343124	184	SAT1_CF	blue	na
chrUn	52343306	52345100	1795	SAT1_CF	blue	na
chrUn	52345112	52345670	559	SAT1_CF	blue	na
chrUn	52345735	52346253	519	SAT1_CF	blue	na
chrUn	52346324	52347104	781	SAT1_CF	blue	na
chrUn	52347146	52348007	862	SAT1_CF	blue	na
chrUn	52348019	52349255	1237	SAT1_CF	blue	na
chrUn	52349298	52349844	547	SAT1_CF	blue	na
chrUn	52349901	52350568	668	SAT1_CF	blue	na
chrUn	52350580	52351211	632	SAT1_CF	blue	na
chrUn	52351234	52351854	621	SAT1_CF	blue	na
chrUn	52351894	52352853	960	SAT1_CF	blue	na
chrUn	52352897	52353494	598	SAT1_CF	blue	na
chrUn	52372226	52372364	139	Carsat3	green	na
chrUn	52372391	52373177	787	Carsat3	green	na
chrUn	52373476	52373552	77	Carsat3	green	na
chrUn	52464557	52465997	1441	SAT1_CF	blue	na
chrUn	52466040	52466720	681	SAT1_CF	blue	na
chrUn	52466734	52467416	683	SAT1_CF	blue	na
chrUn	52467430	52469238	1809	SAT1_CF	blue	na
chrUn	52469283	52469830	548	SAT1_CF	blue	na
chrUn	52469850	52469959	110	SAT1_CF	blue	na
chrUn	52469971	52470690	720	SAT1_CF	blue	na
chrUn	52470706	52471381	676	SAT1_CF	blue	na
chrUn	52471393	52472063	671	SAT1_CF	blue	na
chrUn	52472132	52472757	626	SAT1_CF	blue	na
chrUn	52472770	52473766	997	SAT1_CF	blue	na
chrUn	52475355	52476049	695	SAT1_CF	blue	na
chrUn	52476071	52476209	139	SAT1_CF	blue	na
chrUn	52476222	52476722	501	SAT1_CF	blue	na
chrUn	52479341	52479693	353	SAT1_CF	blue	na
chrUn	52479717	52480956	1240	SAT1_CF	blue	na
chrUn	52480999	52481339	341	SAT1_CF	blue	na
chrUn	52487643	52488204	562	SAT1_CF	blue	na
chrUn	52488219	52488604	386	SAT1_CF	blue	na
chrUn	52488631	52489536	906	SAT1_CF	blue	na

chrUn	52490180	52490577	398	SAT1_CF	blue	na
chrUn	52490590	52491091	502	SAT1_CF	blue	na
chrUn	52491131	52492172	1042	SAT1_CF	blue	na
chrUn	52492208	52493170	963	SAT1_CF	blue	na
chrUn	52493213	52493885	673	SAT1_CF	blue	na
chrUn	52493897	52494659	763	SAT1_CF	blue	na
chrUn	52494677	52495273	597	SAT1_CF	blue	na
chrUn	52496618	52497912	1295	SAT1_CF	blue	na
chrUn	52497960	52498503	544	SAT1_CF	blue	na
chrUn	52498560	52499224	665	SAT1_CF	blue	na
chrUn	52499241	52499737	497	SAT1_CF	blue	na
chrUn	52499777	52500707	931	SAT1_CF	blue	na
chrUn	52500741	52501813	1073	SAT1_CF	blue	na
chrUn	52501856	52502403	548	SAT1_CF	blue	na
chrUn	52502426	52503012	587	SAT1_CF	blue	na
chrUn	52504286	52504441	156	SAT1_CF	blue	na
chrUn	52504459	52505166	708	SAT1_CF	blue	na
chrUn	52505177	52505397	221	SAT1_CF	blue	na
chrUn	52506398	52506581	184	SAT1_CF	blue	na
chrUn	52506625	52507580	956	SAT1_CF	blue	na
chrUn	52507594	52508302	709	SAT1_CF	blue	na
chrUn	52508334	52508688	355	SAT1_CF	blue	na
chrUn	52508794	52509274	481	SAT1_CF	blue	na
chrUn	52509287	52509360	74	SAT1_CF	blue	na
chrUn	52509416	52509969	554	SAT1_CF	blue	na
chrUn	52509991	52511154	1164	SAT1_CF	blue	na
chrUn	52511235	52511783	549	SAT1_CF	blue	na
chrUn	52511826	52512027	202	SAT1_CF	blue	na
chrUn	52512207	52512393	187	SAT1_CF	blue	na
chrUn	52512451	52512931	481	SAT1_CF	blue	na
chrUn	52512944	52513704	761	SAT1_CF	blue	na
chrUn	52513716	52514392	677	SAT1_CF	blue	na
chrUn	52514435	52516392	1958	SAT1_CF	blue	na
chrUn	52516404	52517991	1588	SAT1_CF	blue	na
chrUn	52518022	52519375	1354	SAT1_CF	blue	na
chrUn	52519388	52520088	701	SAT1_CF	blue	na
chrUn	52520100	52521740	1641	SAT1_CF	blue	na
chrUn	52521752	52521836	85	SAT1_CF	blue	na
chrUn	52521849	52522372	524	SAT1_CF	blue	na
chrUn	52522384	52522782	399	SAT1_CF	blue	na
chrUn	52522805	52523323	519	SAT1_CF	blue	na
chrUn	52523351	52524020	670	SAT1_CF	blue	na

chrUn	52524032	52524714	683	SAT1_CF	blue	na
chrUn	52524725	52525217	493	SAT1_CF	blue	na
chrUn	52525245	52526263	1019	SAT1_CF	blue	na
chrUn	52526328	52526889	562	SAT1_CF	blue	na
chrUn	52528305	52528878	574	SAT1_CF	blue	na
chrUn	52528913	52529420	508	SAT1_CF	blue	na
chrUn	52529433	52530108	676	SAT1_CF	blue	na
chrUn	52530151	52531137	987	SAT1_CF	blue	na
chrUn	52531158	52532191	1034	SAT1_CF	blue	na
chrUn	52532211	52532729	519	SAT1_CF	blue	na
chrUn	52532742	52533414	673	SAT1_CF	blue	na
chrUn	52533443	52533964	522	SAT1_CF	blue	na
chrUn	52533991	52534189	199	SAT1_CF	blue	na
chrUn	52573275	52574322	1048	CarSat1	red	na
chrUn	52576345	52577547	1203	CarSat1	red	na
chrUn	52577572	52578497	926	CarSat1	red	na
chrUn	52578512	52578638	127	Carsat2	orange	na
chrUn	52578527	52579134	608	CarSat1	red	na
chrUn	52579547	52580727	1181	CarSat1	red	na
chrUn	52580828	52581492	665	CarSat1	red	na
chrUn	52581507	52581634	128	Carsat2	orange	na
chrUn	52581522	52583856	2335	CarSat1	red	na
chrUn	52584256	52586328	2073	CarSat1	red	na
chrUn	52586353	52589009	2657	CarSat1	red	na
chrUn	52589024	52589148	125	Carsat2	orange	na
chrUn	52589039	52589832	794	CarSat1	red	na
chrUn	52590735	52592096	1362	CarSat1	red	na
chrUn	52592111	52592234	124	Carsat2	orange	na
chrUn	52592126	52592946	821	CarSat1	red	na
chrUn	52592981	52595156	2176	CarSat1	red	na
chrUn	52595183	52596569	1387	CarSat1	red	na
chrUn	52596588	52598900	2313	CarSat1	red	na
chrUn	52598929	52601108	2180	CarSat1	red	na
chrUn	52601963	52603137	1175	CarSat1	red	na
chrUn	52603174	52604267	1094	CarSat1	red	na
chrUn	52605530	52605898	369	Carsat2	orange	na
chrUn	52605568	52606863	1296	CarSat1	red	na
chrUn	52682905	52683668	764	SAT1_CF	blue	na
chrUn	52683680	52684866	1187	SAT1_CF	blue	na
chrUn	52684893	52685395	503	SAT1_CF	blue	na
chrUn	52685439	52686289	851	SAT1_CF	blue	na
chrUn	52688370	52688447	78	SAT1_CF	blue	na

chrUn	52688485	52688854	370	SAT1_CF	blue	na
chrUn	52688882	52689391	510	SAT1_CF	blue	na
chrUn	52689456	52689613	158	SAT1_CF	blue	na
chrUn	52689639	52690157	519	SAT1_CF	blue	na
chrUn	52690177	52690775	599	SAT1_CF	blue	na
chrUn	52690797	52691871	1075	SAT1_CF	blue	na
chrUn	52691889	52692558	670	SAT1_CF	blue	na
chrUn	52692601	52693557	957	SAT1_CF	blue	na
chrUn	52693673	52694616	944	SAT1_CF	blue	na
chrUn	52694654	52695174	521	SAT1_CF	blue	na
chrUn	52695187	52695295	109	SAT1_CF	blue	na
chrUn	52695316	52697681	2366	SAT1_CF	blue	na
chrUn	52697751	52698909	1159	SAT1_CF	blue	na
chrUn	52698922	52701174	2253	SAT1_CF	blue	na
chrUn	52701187	52701294	108	SAT1_CF	blue	na
chrUn	52701316	52702749	1434	SAT1_CF	blue	na
chrUn	52702761	52703875	1115	SAT1_CF	blue	na
chrUn	52703887	52705331	1445	SAT1_CF	blue	na
chrUn	52705377	52706930	1554	SAT1_CF	blue	na
chrUn	52710259	52711130	872	Sat2CF	purple	na
chrUn	52710354	52711132	779	Sat2CF	purple	na
chrUn	52710846	52711191	346	Sat2CF	purple	na
chrUn	52743103	52744270	1168	Sat2CF	purple	na
chrUn	52744162	52744341	180	Sat2CF	purple	na
chrUn	52759451	52760292	842	Sat2CF	purple	na
chrUn	52861495	52862398	904	CarSat1	red	na
chrUn	52862419	52863275	857	CarSat1	red	na
chrUn	52863429	52864468	1040	CarSat1	red	na
chrUn	52868359	52869205	847	Sat2CF	purple	na
chrUn	52868443	52869167	725	Sat2CF	purple	na
chrUn	52869058	52869226	169	Sat2CF	purple	na
chrUn	52880154	52881328	1175	Sat2CF	purple	na
chrUn	52935367	52935925	559	Sat2CF	purple	na
chrUn	52935376	52935851	476	Sat2CF	purple	na
chrUn	52966473	52967087	615	SAT1_CF	blue	na
chrUn	52967132	52967526	395	SAT1_CF	blue	na
chrUn	52967541	52968175	635	SAT1_CF	blue	na
chrUn	52968201	52969323	1123	SAT1_CF	blue	na
chrUn	52969336	52970434	1099	SAT1_CF	blue	na
chrUn	52970678	52970819	142	SAT1_CF	blue	na
chrUn	52970863	52972195	1333	SAT1_CF	blue	na
chrUn	52972207	52973684	1478	SAT1_CF	blue	na

chrUn	52973697	52974198	502	SAT1_CF	blue	na
chrUn	52974237	52974418	182	SAT1_CF	blue	na
chrUn	52974599	52975462	864	SAT1_CF	blue	na
chrUn	52975477	52976634	1158	SAT1_CF	blue	na
chrUn	52976648	52977408	761	SAT1_CF	blue	na
chrUn	52977421	52978580	1160	SAT1_CF	blue	na
chrUn	52978624	52979300	677	SAT1_CF	blue	na
chrUn	52979314	52979995	682	SAT1_CF	blue	na
chrUn	52980007	52980690	684	SAT1_CF	blue	na
chrUn	52980703	52981219	517	SAT1_CF	blue	na
chrUn	52981236	52981609	374	SAT1_CF	blue	na
chrUn	52981652	52982622	971	SAT1_CF	blue	na
chrUn	52982666	52984771	2106	SAT1_CF	blue	na
chrUn	52984786	52986081	1296	SAT1_CF	blue	na
chrUn	52986093	52986788	696	SAT1_CF	blue	na
chrUn	52986799	52987685	887	SAT1_CF	blue	na
chrUn	52987699	52988588	890	SAT1_CF	blue	na
chrUn	52988631	52989296	666	SAT1_CF	blue	na
chrUn	52989318	52990441	1124	SAT1_CF	blue	na
chrUn	52990541	52991902	1362	SAT1_CF	blue	na
chrUn	52991916	52992408	493	SAT1_CF	blue	na
chrUn	52992419	52993609	1191	SAT1_CF	blue	na
chrUn	53064394	53064849	456	SAT1_CF	blue	na
chrUn	53064862	53065355	494	SAT1_CF	blue	na
chrUn	53065366	53066048	683	SAT1_CF	blue	na
chrUn	53066060	53067257	1198	SAT1_CF	blue	na
chrUn	53067344	53069330	1987	SAT1_CF	blue	na
chrUn	53069373	53069918	546	SAT1_CF	blue	na
chrUn	53070265	53070798	534	SAT1_CF	blue	na
chrUn	53070811	53071331	521	SAT1_CF	blue	na
chrUn	53071357	53071513	157	SAT1_CF	blue	na
chrUn	53071541	53072983	1443	SAT1_CF	blue	na
chrUn	53072996	53073755	760	SAT1_CF	blue	na
chrUn	53073768	53074267	500	SAT1_CF	blue	na
chrUn	53074302	53074684	383	SAT1_CF	blue	na
chrUn	53074719	53075680	962	SAT1_CF	blue	na
chrUn	53075723	53076927	1205	SAT1_CF	blue	na
chrUn	53076997	53077673	677	SAT1_CF	blue	na
chrUn	53077685	53078190	506	SAT1_CF	blue	na
chrUn	53078244	53078886	643	SAT1_CF	blue	na
chrUn	53078973	53080259	1287	SAT1_CF	blue	na
chrUn	53080304	53080461	158	SAT1_CF	blue	na

chrUn	53080476	53080999	524	SAT1_CF	blue	na
chrUn	53081079	53081643	565	SAT1_CF	blue	na
chrUn	53081671	53082301	631	SAT1_CF	blue	na
chrUn	53082344	53083021	678	SAT1_CF	blue	na
chrUn	53083033	53083318	286	SAT1_CF	blue	na
chrUn	53083329	53083834	506	SAT1_CF	blue	na
chrUn	53083854	53083963	110	SAT1_CF	blue	na
chrUn	53083975	53084660	686	SAT1_CF	blue	na
chrUn	53084685	53085186	502	SAT1_CF	blue	na
chrUn	53085215	53085663	449	SAT1_CF	blue	na
chrUn	53085674	53086949	1276	SAT1_CF	blue	na
chrUn	53086961	53088438	1478	SAT1_CF	blue	na
chrUn	53088456	53090168	1713	SAT1_CF	blue	na
chrUn	53090238	53091702	1465	SAT1_CF	blue	na
chrUn	53120784	53121981	1198	CarSat1	red	na
chrUn	53123920	53125364	1445	CarSat1	red	na
chrUn	53126614	53128057	1444	CarSat1	red	na
chrUn	53128072	53128198	127	Carsat2	orange	na
chrUn	53128087	53128624	538	CarSat1	red	na
chrUn	53131631	53132377	747	CarSat1	red	na
chrUn	53132404	53135053	2650	CarSat1	red	na
chrUn	53135068	53135194	127	Carsat2	orange	na
chrUn	53135083	53136229	1147	CarSat1	red	na
chrUn	53136937	53139108	2172	CarSat1	red	na
chrUn	53139935	53140459	525	CarSat1	red	na
chrUn	53140470	53140600	131	Carsat2	orange	na
chrUn	53140489	53141526	1038	CarSat1	red	na
chrUn	53142382	53143399	1018	CarSat1	red	na
chrUn	53143414	53143541	128	Carsat2	orange	na
chrUn	53143429	53144137	709	CarSat1	red	na
chrUn	53144168	53144878	711	CarSat1	red	na
chrUn	53144908	53145614	707	CarSat1	red	na
chrUn	53145644	53148400	2757	CarSat1	red	na
chrUn	53148421	53149136	716	CarSat1	red	na
chrUn	53149157	53149875	719	CarSat1	red	na
chrUn	53149896	53150340	445	CarSat1	red	na
chrUn	53150370	53152087	1718	CarSat1	red	na
chrUn	53152108	53152826	719	CarSat1	red	na
chrUn	53152847	53154843	1997	CarSat1	red	na
chrUn	53156277	53157577	1301	CarSat1	red	na
chrUn	53158587	53159322	736	SAT1_CF	blue	na
chrUn	53159335	53159816	482	SAT1_CF	blue	na

chrUn	53160124	53160289	166	SAT1_CF	blue	na
chrUn	53160322	53162120	1799	SAT1_CF	blue	na
chrUn	53162134	53162763	630	SAT1_CF	blue	na
chrUn	53162784	53162892	109	SAT1_CF	blue	na
chrUn	53162904	53163587	684	SAT1_CF	blue	na
chrUn	53163600	53164119	520	SAT1_CF	blue	na
chrUn	53164187	53165085	899	SAT1_CF	blue	na
chrUn	53165140	53166138	999	SAT1_CF	blue	na
chrUn	53166181	53166849	669	SAT1_CF	blue	na
chrUn	53166867	53167417	551	SAT1_CF	blue	na
chrUn	53167440	53167511	72	SAT1_CF	blue	na
chrUn	53167524	53168285	762	SAT1_CF	blue	na
chrUn	53168298	53168799	502	SAT1_CF	blue	na
chrUn	53168886	53170879	1994	SAT1_CF	blue	na
chrUn	53170922	53171596	675	SAT1_CF	blue	na
chrUn	53171608	53172182	575	SAT1_CF	blue	na
chrUn	53172235	53172311	77	SAT1_CF	blue	na
chrUn	53172324	53173159	836	SAT1_CF	blue	na
chrUn	53173172	53174485	1314	SAT1_CF	blue	na
chrUn	53174497	53175011	515	SAT1_CF	blue	na
chrUn	53175037	53175572	536	SAT1_CF	blue	na
chrUn	53175617	53176343	727	SAT1_CF	blue	na
chrUn	53176386	53177062	677	SAT1_CF	blue	na
chrUn	53177074	53177676	603	SAT1_CF	blue	na
chrUn	53177687	53178367	681	SAT1_CF	blue	na
chrUn	53178379	53179068	690	SAT1_CF	blue	na
chrUn	53179082	53180951	1870	SAT1_CF	blue	na
chrUn	53180974	53181079	106	SAT1_CF	blue	na
chrUn	53181092	53181592	501	SAT1_CF	blue	na
chrUn	53181637	53183488	1852	SAT1_CF	blue	na
chrUn	53183530	53184588	1059	SAT1_CF	blue	na
chrUn	53184611	53184682	72	SAT1_CF	blue	na
chrUn	53184695	53185550	856	SAT1_CF	blue	na
chrUn	53215661	53216498	838	Sat2CF	purple	na
chrUn	53215735	53216461	727	Sat2CF	purple	na
chrUn	53216351	53216519	169	Sat2CF	purple	na
chrUn	53231488	53232708	1221	Sat2CF	purple	na
chrUn	53242486	53242899	414	Sat2CF	purple	na
chrUn	53247069	53249294	2226	CarSat1	red	na
chrUn	53249327	53254023	4697	CarSat1	red	na
chrUn	53254211	53259316	5106	CarSat1	red	na
chrUn	53259331	53259457	127	Carsat2	orange	na

chrUn	53259346	53262977	3632	CarSat1	red	na
chrUn	53263286	53269673	6388	CarSat1	red	na
chrUn	53269689	53271145	1457	CarSat1	red	na
chrUn	53271161	53271882	722	CarSat1	red	na
chrUn	53271898	53272619	722	CarSat1	red	na
chrUn	53272644	53274173	1530	CarSat1	red	na
chrUn	53289136	53290213	1078	Sat2CF	purple	na
chrUn	53306693	53307233	541	CarSat1	red	na
chrUn	53307254	53308434	1181	CarSat1	red	na
chrUn	53308464	53309171	708	CarSat1	red	na
chrUn	53309201	53310431	1231	CarSat1	red	na
chrUn	53310447	53311899	1453	CarSat1	red	na
chrUn	53311923	53312859	937	CarSat1	red	na
chrUn	53312889	53313868	980	CarSat1	red	na
chrUn	53313895	53321466	7572	CarSat1	red	na
chrUn	53321480	53324884	3405	CarSat1	red	na
chrUn	53324905	53327804	2900	CarSat1	red	na
chrUn	53327986	53330464	2479	CarSat1	red	na
chrUn	53330497	53330934	438	CarSat1	red	na
chrUn	53331165	53332470	1306	CarSat1	red	na
chrUn	53332659	53333453	795	CarSat1	red	na
chrUn	53334494	53335377	884	SAT1_CF	blue	na
chrUn	53335391	53335899	509	SAT1_CF	blue	na
chrUn	53335912	53335993	82	SAT1_CF	blue	na
chrUn	53336041	53337364	1324	SAT1_CF	blue	na
chrUn	53337379	53338062	684	SAT1_CF	blue	na
chrUn	53338074	53339001	928	SAT1_CF	blue	na
chrUn	53339014	53340058	1045	SAT1_CF	blue	na
chrUn	53340098	53340598	501	SAT1_CF	blue	na
chrUn	53340616	53342096	1481	SAT1_CF	blue	na
chrUn	53342107	53342509	403	SAT1_CF	blue	na
chrUn	53342549	53343637	1089	SAT1_CF	blue	na
chrUn	53343655	53344980	1326	SAT1_CF	blue	na
chrUn	53345005	53345329	325	SAT1_CF	blue	na
chrUn	53345407	53345915	509	SAT1_CF	blue	na
chrUn	53345998	53346579	582	SAT1_CF	blue	na
chrUn	53346601	53347352	752	SAT1_CF	blue	na
chrUn	53347365	53347981	617	SAT1_CF	blue	na
chrUn	53348194	53348349	156	SAT1_CF	blue	na
chrUn	53348375	53351265	2891	SAT1_CF	blue	na
chrUn	53352344	53352942	599	SAT1_CF	blue	na
chrUn	53352954	53353629	676	SAT1_CF	blue	na

chrUn	53353671	53354884	1214	SAT1_CF	blue	na
chrUn	53355228	53355679	452	SAT1_CF	blue	na
chrUn	53355693	53356798	1106	SAT1_CF	blue	na
chrUn	53356842	53357801	960	SAT1_CF	blue	na
chrUn	53357861	53358795	935	SAT1_CF	blue	na
chrUn	53358808	53361000	2193	SAT1_CF	blue	na
chrUn	53361225	53361382	158	SAT1_CF	blue	na
chrUn	53361408	53361927	520	SAT1_CF	blue	na
chrUn	53361947	53362722	776	SAT1_CF	blue	na
chrUn	53367859	53368185	327	SAT1_CF	blue	na
chrUn	53384438	53385921	1484	CarSat1	red	na
chrUn	53385949	53386788	840	CarSat1	red	na
chrUn	53386809	53388401	1593	CarSat1	red	na
chrUn	53439800	53440906	1107	Sat7CF	lime	na
chrUn	53486353	53487751	1399	CarSat1	red	na
chrUn	53487772	53488402	631	CarSat1	red	na
chrUn	53488683	53489700	1018	CarSat1	red	na
chrUn	53499904	53502056	2153	CarSat1	red	na
chrUn	53502142	53502632	491	CarSat1	red	na
chrUn	53502663	53503997	1335	CarSat1	red	na
chrUn	53504024	53504537	514	CarSat1	red	na
chrUn	53504568	53506012	1445	CarSat1	red	na
chrUn	53506235	53507124	890	CarSat1	red	na
chrUn	53517121	53517555	435	SAT1_CF	blue	na
chrUn	53517567	53518250	684	SAT1_CF	blue	na
chrUn	53518262	53519190	929	SAT1_CF	blue	na
chrUn	53519203	53520675	1473	SAT1_CF	blue	na
chrUn	53520705	53521230	526	SAT1_CF	blue	na
chrUn	53521293	53521947	655	SAT1_CF	blue	na
chrUn	53521960	53523294	1335	SAT1_CF	blue	na
chrUn	53523319	53524597	1279	SAT1_CF	blue	na
chrUn	53524646	53525146	501	SAT1_CF	blue	na
chrUn	53525159	53525843	685	SAT1_CF	blue	na
chrUn	53525855	53528962	3108	SAT1_CF	blue	na
chrUn	53528974	53529659	686	SAT1_CF	blue	na
chrUn	53529671	53532816	3146	SAT1_CF	blue	na
chrUn	53532832	53532936	105	SAT1_CF	blue	na
chrUn	53532957	53533512	556	SAT1_CF	blue	na
chrUn	53533524	53534444	921	SAT1_CF	blue	na
chrUn	53534486	53535536	1051	SAT1_CF	blue	na
chrUn	53535875	53537653	1779	SAT1_CF	blue	na
chrUn	53538165	53538470	306	SAT1_CF	blue	na

chrUn	53538488	53539003	516	SAT1_CF	blue	na
chrUn	53539029	53540133	1105	SAT1_CF	blue	na
chrUn	53540184	53541963	1780	SAT1_CF	blue	na
chrUn	53541977	53542379	403	SAT1_CF	blue	na
chrUn	53542419	53543785	1367	SAT1_CF	blue	na
chrUn	53543802	53544306	505	SAT1_CF	blue	na
chrUn	53581180	53582348	1169	CarSat1	red	na
chrUn	53587957	53589208	1252	CarSat1	red	na
chrUn	53589243	53591918	2676	CarSat1	red	na
chrUn	53591932	53592059	128	Carsat2	orange	na
chrUn	53591948	53592654	707	CarSat1	red	na
chrUn	53592669	53592791	123	Carsat2	orange	na
chrUn	53592684	53593569	886	CarSat1	red	na
chrUn	53593908	53594366	459	CarSat1	red	na
chrUn	53594381	53594507	127	Carsat2	orange	na
chrUn	53594396	53595103	708	CarSat1	red	na
chrUn	53595118	53595245	128	Carsat2	orange	na
chrUn	53595133	53595803	671	CarSat1	red	na
chrUn	53596952	53598716	1765	CarSat1	red	na
chrUn	53600512	53601197	686	CarSat1	red	na
chrUn	53601218	53602277	1060	CarSat1	red	na
chrUn	53604999	53605761	763	CarSat1	red	na
chrUn	53605794	53606546	753	CarSat1	red	na
chrUn	53608331	53609871	1541	CarSat1	red	na
chrUn	53616428	53617144	717	CarSat1	red	na
chrUn	53652639	53653144	506	SAT1_CF	blue	na
chrUn	53653191	53653688	498	SAT1_CF	blue	na
chrUn	53653741	53654167	427	SAT1_CF	blue	na
chrUn	53654201	53654979	779	SAT1_CF	blue	na
chrUn	53654992	53655108	117	SAT1_CF	blue	na
chrUn	53655120	53655791	672	SAT1_CF	blue	na
chrUn	53655814	53656314	501	SAT1_CF	blue	na
chrUn	53656380	53656516	137	SAT1_CF	blue	na
chrUn	53656544	53657773	1230	SAT1_CF	blue	na
chrUn	53657819	53658838	1020	SAT1_CF	blue	na
chrUn	53658899	53659443	545	SAT1_CF	blue	na
chrUn	53659464	53659573	110	SAT1_CF	blue	na
chrUn	53659585	53660343	759	SAT1_CF	blue	na
chrUn	53660357	53660857	501	SAT1_CF	blue	na
chrUn	53660944	53661833	890	SAT1_CF	blue	na
chrUn	53661900	53662682	783	SAT1_CF	blue	na
chrUn	53662724	53663884	1161	SAT1_CF	blue	na

chrUn	53663897	53664824	928	SAT1_CF	blue	na
chrUn	53664836	53665507	672	SAT1_CF	blue	na
chrUn	53665531	53666291	761	SAT1_CF	blue	na
chrUn	53666304	53669060	2757	SAT1_CF	blue	na
chrUn	53669083	53669188	106	SAT1_CF	blue	na
chrUn	53669201	53669947	747	SAT1_CF	blue	na
chrUn	53669974	53670475	502	SAT1_CF	blue	na
chrUn	53670521	53671449	929	SAT1_CF	blue	na
chrUn	53671466	53672923	1458	SAT1_CF	blue	na
chrUn	53672980	53673586	607	SAT1_CF	blue	na
chrUn	53673657	53674200	544	SAT1_CF	blue	na
chrUn	53674243	53674789	547	SAT1_CF	blue	na
chrUn	53674833	53675830	998	SAT1_CF	blue	na
chrUn	53675873	53676552	680	SAT1_CF	blue	na
chrUn	53676564	53677371	808	SAT1_CF	blue	na
chrUn	53677398	53677918	521	SAT1_CF	blue	na
chrUn	53677939	53678887	949	SAT1_CF	blue	na
chrUn	53680047	53680831	785	SAT1_CF	blue	na
chrUn	53680876	53681421	546	SAT1_CF	blue	na
chrUn	53683038	53683458	421	SAT1_CF	blue	na
chrUn	53683471	53684155	685	SAT1_CF	blue	na
chrUn	53684168	53685971	1804	SAT1_CF	blue	na
chrUn	53686050	53686215	166	SAT1_CF	blue	na
chrUn	53686396	53686581	186	SAT1_CF	blue	na
chrUn	53686598	53687118	521	SAT1_CF	blue	na
chrUn	53687131	53687237	107	SAT1_CF	blue	na
chrUn	53687259	53687816	558	SAT1_CF	blue	na
chrUn	53687828	53690538	2711	SAT1_CF	blue	na
chrUn	53690625	53691123	499	SAT1_CF	blue	na
chrUn	53691136	53691241	106	SAT1_CF	blue	na
chrUn	53691264	53692439	1176	SAT1_CF	blue	na
chrUn	53692472	53692628	157	SAT1_CF	blue	na
chrUn	53692673	53693173	501	SAT1_CF	blue	na
chrUn	53693186	53693549	364	SAT1_CF	blue	na
chrUn	53694275	53694685	411	SAT1_CF	blue	na
chrUn	53694765	53695289	525	SAT1_CF	blue	na
chrUn	53695304	53695418	115	SAT1_CF	blue	na
chrUn	53695866	53697565	1700	SAT1_CF	blue	na
chrUn	53697608	53699231	1624	SAT1_CF	blue	na
chrUn	53699312	53699891	580	SAT1_CF	blue	na
chrUn	53699904	53701238	1335	SAT1_CF	blue	na
chrUn	53701281	53702245	965	SAT1_CF	blue	na

chrUn	53702257	53702410	154	SAT1_CF	blue	na
chrUn	53703413	53704127	715	SAT1_CF	blue	na
chrUn	53704170	53705623	1454	SAT1_CF	blue	na
chrUn	53705644	53706163	520	SAT1_CF	blue	na
chrUn	53706176	53706429	254	SAT1_CF	blue	na
chrUn	53706441	53707165	725	SAT1_CF	blue	na
chrUn	53707176	53707668	493	SAT1_CF	blue	na
chrUn	53707682	53709231	1550	SAT1_CF	blue	na
chrUn	53803343	53804552	1210	Carsat3	green	na
chrUn	53804936	53805176	241	Carsat3	green	na
chrUn	53821916	53822215	300	Carsat3	green	na
chrUn	53840492	53841281	790	SAT1_CF	blue	na
chrUn	53841324	53841954	631	SAT1_CF	blue	na
chrUn	53841995	53842500	506	SAT1_CF	blue	na
chrUn	53844185	53845214	1030	SAT1_CF	blue	na
chrUn	53848286	53849220	935	SAT1_CF	blue	na
chrUn	53849233	53849677	445	SAT1_CF	blue	na
chrUn	53852001	53852197	197	SAT1_CF	blue	na
chrUn	53852210	53853646	1437	SAT1_CF	blue	na
chrUn	53853661	53853903	243	SAT1_CF	blue	na
chrUn	53855612	53855903	292	SAT1_CF	blue	na
chrUn	53855917	53856026	110	SAT1_CF	blue	na
chrUn	53856047	53856593	547	SAT1_CF	blue	na
chrUn	53856636	53857114	479	SAT1_CF	blue	na
chrUn	53859946	53860292	347	SAT1_CF	blue	na
chrUn	53860306	53860636	331	SAT1_CF	blue	na
chrUn	53860675	53861314	640	SAT1_CF	blue	na
chrUn	53867453	53868160	708	SAT1_CF	blue	na
chrUn	53868204	53868749	546	SAT1_CF	blue	na
chrUn	53868762	53869461	700	SAT1_CF	blue	na
chrUn	53869479	53870157	679	SAT1_CF	blue	na
chrUn	53870184	53871250	1067	SAT1_CF	blue	na
chrUn	53871271	53872706	1436	SAT1_CF	blue	na
chrUn	53872748	53872913	166	SAT1_CF	blue	na
chrUn	53873096	53873281	186	SAT1_CF	blue	na
chrUn	53873298	53873811	514	SAT1_CF	blue	na
chrUn	53873831	53873939	109	SAT1_CF	blue	na
chrUn	53873959	53874564	606	SAT1_CF	blue	na
chrUn	53875003	53876517	1515	SAT1_CF	blue	na
chrUn	53876543	53877062	520	SAT1_CF	blue	na
chrUn	53877075	53877757	683	SAT1_CF	blue	na
chrUn	53877769	53878718	950	SAT1_CF	blue	na

chrUn	53878732	53879352	621	SAT1_CF	blue	na
chrUn	53880109	53880612	504	SAT1_CF	blue	na
chrUn	53880838	53881351	514	SAT1_CF	blue	na
chrUn	53881534	53881784	251	SAT1_CF	blue	na
chrUn	53881835	53883020	1186	SAT1_CF	blue	na
chrUn	53883063	53883626	564	SAT1_CF	blue	na
chrUn	53884333	53884492	160	SAT1_CF	blue	na
chrUn	53884505	53885830	1326	SAT1_CF	blue	na
chrUn	53885876	53886182	307	SAT1_CF	blue	na
chrUn	53892104	53892270	167	Sat2CF	purple	na
chrUn	53892132	53893129	998	Sat2CF	purple	na
chrUn	53892176	53893040	865	Sat2CF	purple	na
chrUn	53892997	53893164	168	Sat2CF	purple	na
chrUn	53962502	53963171	670	Sat7CF	lime	na
chrUn	53997002	53998106	1105	Sat2CF	purple	na
chrUn	54072346	54073266	921	CarSat1	red	na
chrUn	54073280	54074728	1449	CarSat1	red	na
chrUn	54074764	54075480	717	CarSat1	red	na
chrUn	54075495	54076205	711	CarSat1	red	na
chrUn	54076235	54077165	931	CarSat1	red	na
chrUn	54077198	54078436	1239	CarSat1	red	na
chrUn	54078451	54079158	708	CarSat1	red	na
chrUn	54079188	54080636	1449	CarSat1	red	na
chrUn	54080666	54082109	1444	CarSat1	red	na
chrUn	54082139	54084058	1920	CarSat1	red	na
chrUn	54084079	54085083	1005	CarSat1	red	na
chrUn	54085098	54085824	727	CarSat1	red	na
chrUn	54085839	54086769	931	CarSat1	red	na
chrUn	54086802	54087288	487	CarSat1	red	na
chrUn	54087318	54088025	708	CarSat1	red	na
chrUn	54088055	54088766	712	CarSat1	red	na
chrUn	54088796	54089502	707	CarSat1	red	na
chrUn	54089532	54090253	722	CarSat1	red	na
chrUn	54090268	54091198	931	CarSat1	red	na
chrUn	54091231	54093946	2716	CarSat1	red	na
chrUn	54093961	54094668	708	CarSat1	red	na
chrUn	54094698	54095409	712	CarSat1	red	na
chrUn	54095439	54096163	725	CarSat1	red	na
chrUn	54096178	54096884	707	CarSat1	red	na
chrUn	54096914	54097635	722	CarSat1	red	na
chrUn	54097650	54098217	568	CarSat1	red	na
chrUn	54188157	54189066	910	CarSat1	red	na

chrUn	54189097	54190686	1590	CarSat1	red	na
chrUn	54190717	54192304	1588	CarSat1	red	na
chrUn	54192325	54193922	1598	CarSat1	red	na
chrUn	54193943	54195540	1598	CarSat1	red	na
chrUn	54195561	54197158	1598	CarSat1	red	na
chrUn	54197189	54198772	1584	CarSat1	red	na
chrUn	54198793	54200390	1598	CarSat1	red	na
chrUn	54200421	54202012	1592	CarSat1	red	na
chrUn	54202033	54203631	1599	CarSat1	red	na
chrUn	54203652	54205253	1602	CarSat1	red	na
chrUn	54205284	54206978	1695	CarSat1	red	na
chrUn	54207276	54208456	1181	CarSat1	red	na
chrUn	54208487	54210076	1590	CarSat1	red	na
chrUn	54210097	54211697	1601	CarSat1	red	na
chrUn	54211718	54213310	1593	CarSat1	red	na
chrUn	54213341	54214230	890	CarSat1	red	na
chrUn	54317952	54319771	1820	CarSat1	red	na
chrUn	54319944	54321305	1362	CarSat1	red	na
chrUn	54321409	54327626	6218	CarSat1	red	na
chrUn	54328464	54329255	792	CarSat1	red	na
chrUn	54329282	54331467	2186	CarSat1	red	na
chrUn	54331494	54332371	878	CarSat1	red	na
chrUn	54332905	54335671	2767	CarSat1	red	na
chrUn	54335697	54336205	509	CarSat1	red	na
chrUn	54336244	54336943	700	CarSat1	red	na
chrUn	54336970	54338064	1095	CarSat1	red	na
chrUn	54338561	54339746	1186	CarSat1	red	na
chrUn	54342155	54343368	1214	CarSat1	red	na
chrUn	54343744	54344793	1050	CarSat1	red	na
chrUn	54345350	54347471	2122	CarSat1	red	na
chrUn	54348431	54349658	1228	CarSat1	red	na
chrUn	54361272	54362009	738	Sat2CF	purple	na
chrUn	54361346	54362007	662	Sat2CF	purple	na
chrUn	54380555	54381224	670	Sat7CF	lime	na
chrUn	54401270	54403630	2361	CarSat1	red	na
chrUn	54403661	54405112	1452	CarSat1	red	na
chrUn	54417569	54419350	1782	CarSat1	red	na
chrUn	54427355	54427926	572	CarSat1	red	na
chrUn	54427947	54428987	1041	CarSat1	red	na
chrUn	54432031	54433281	1251	Sat2CF	purple	na
chrUn	54432067	54433151	1085	Sat2CF	purple	na
chrUn	54444243	54446844	2602	CarSat1	red	na

chrUn	54446877	54447362	486	CarSat1	red	na
chrUn	54447399	54448116	718	CarSat1	red	na
chrUn	54448131	54448852	722	CarSat1	red	na
chrUn	54448867	54450329	1463	CarSat1	red	na
chrUn	54450344	54451806	1463	CarSat1	red	na
chrUn	54451821	54452279	459	CarSat1	red	na
chrUn	54452291	54453814	1524	CarSat1	red	na
chrUn	54453965	54454596	632	CarSat1	red	na
chrUn	54454617	54461226	6610	CarSat1	red	na
chrUn	54461247	54465390	4144	CarSat1	red	na
chrUn	54465424	54469799	4376	CarSat1	red	na
chrUn	54485536	54486671	1136	Sat2CF	purple	na
chrUn	54536264	54536709	446	SAT1_CF	blue	na
chrUn	54536722	54537241	520	SAT1_CF	blue	na
chrUn	54537309	54539301	1993	SAT1_CF	blue	na
chrUn	54539344	54540536	1193	SAT1_CF	blue	na
chrUn	54540604	54540959	356	SAT1_CF	blue	na
chrUn	54540975	54541514	540	SAT1_CF	blue	na
chrUn	54541593	54542556	964	SAT1_CF	blue	na
chrUn	54542601	54543278	678	SAT1_CF	blue	na
chrUn	54543290	54544000	711	SAT1_CF	blue	na
chrUn	54544021	54544129	109	SAT1_CF	blue	na
chrUn	54544141	54544660	520	SAT1_CF	blue	na
chrUn	54544728	54545586	859	SAT1_CF	blue	na
chrUn	54545624	54546167	544	SAT1_CF	blue	na
chrUn	54546209	54547061	853	SAT1_CF	blue	na
chrUn	54547079	54547688	610	SAT1_CF	blue	na
chrUn	54547701	54548404	704	SAT1_CF	blue	na
chrUn	54548419	54550919	2501	SAT1_CF	blue	na
chrUn	54550942	54551047	106	SAT1_CF	blue	na
chrUn	54551060	54551561	502	SAT1_CF	blue	na
chrUn	54551597	54551782	186	SAT1_CF	blue	na
chrUn	54551963	54552169	207	SAT1_CF	blue	na
chrUn	54552212	54554011	1800	SAT1_CF	blue	na
chrUn	54554025	54554705	681	SAT1_CF	blue	na
chrUn	54554717	54555400	684	SAT1_CF	blue	na
chrUn	54555413	54555931	519	SAT1_CF	blue	na
chrUn	54555999	54556514	516	SAT1_CF	blue	na
chrUn	54556533	54557941	1409	SAT1_CF	blue	na
chrUn	54557968	54558488	521	SAT1_CF	blue	na
chrUn	54558505	54558690	186	SAT1_CF	blue	na
chrUn	54558872	54559037	166	SAT1_CF	blue	na

chrUn	54559079	54561734	2656	SAT1_CF	blue	na
chrUn	54562736	54564258	1523	CarSat1	red	na
chrUn	54564279	54565485	1207	CarSat1	red	na
chrUn	54565509	54568419	2911	CarSat1	red	na
chrUn	54568452	54572100	3649	CarSat1	red	na
chrUn	54572133	54573573	1441	CarSat1	red	na
chrUn	54573606	54574844	1239	CarSat1	red	na
chrUn	54574866	54575696	831	CarSat1	red	na
chrUn	54576243	54578513	2271	CarSat1	red	na
chrUn	54578550	54581470	2921	CarSat1	red	na
chrUn	54581485	54582191	707	CarSat1	red	na
chrUn	54582220	54584626	2407	CarSat1	red	na
chrUn	54584651	54585872	1222	CarSat1	red	na
chrUn	54585902	54586838	937	CarSat1	red	na
chrUn	54586863	54587823	961	CarSat1	red	na
chrUn	54587844	54588736	893	CarSat1	red	na
chrUn	54629661	54630177	517	CarSat1	red	na
chrUn	54630204	54632386	2183	CarSat1	red	na
chrUn	54632413	54637057	4645	CarSat1	red	na
chrUn	54637082	54637782	701	CarSat1	red	na
chrUn	54637807	54639990	2184	CarSat1	red	na
chrUn	54640015	54640733	719	CarSat1	red	na
chrUn	54640749	54642042	1294	CarSat1	red	na
chrUn	54642416	54645854	3439	CarSat1	red	na
chrUn	54645870	54646589	720	CarSat1	red	na
chrUn	54646605	54652483	5879	CarSat1	red	na
chrUn	54652499	54653219	721	CarSat1	red	na
chrUn	54653235	54653957	723	CarSat1	red	na
chrUn	54653973	54654693	721	CarSat1	red	na
chrUn	54654709	54655248	540	CarSat1	red	na
chrUn	54687829	54688314	486	CarSat1	red	na
chrUn	54688344	54690740	2397	CarSat1	red	na
chrUn	54690773	54691487	715	CarSat1	red	na
chrUn	54691511	54692009	499	CarSat1	red	na
chrUn	54692026	54693275	1250	CarSat1	red	na
chrUn	54693458	54695625	2168	CarSat1	red	na
chrUn	54695647	54697586	1940	CarSat1	red	na
chrUn	54697610	54702992	5383	CarSat1	red	na
chrUn	54703014	54704596	1583	CarSat1	red	na
chrUn	54707038	54708411	1374	CarSat1	red	na
chrUn	54708426	54710831	2406	CarSat1	red	na
chrUn	54710855	54713287	2433	CarSat1	red	na

chrUn	54713306	54714285	980	CarSat1	red	na
chrUn	54714315	54715293	979	CarSat1	red	na
chrUn	54716293	54718478	2186	SAT1_CF	blue	na
chrUn	54718496	54719016	521	SAT1_CF	blue	na
chrUn	54719033	54719406	374	SAT1_CF	blue	na
chrUn	54719449	54719992	544	SAT1_CF	blue	na
chrUn	54720073	54721862	1790	SAT1_CF	blue	na
chrUn	54721874	54722430	557	SAT1_CF	blue	na
chrUn	54722451	54723389	939	SAT1_CF	blue	na
chrUn	54723401	54723864	464	SAT1_CF	blue	na
chrUn	54723934	54724479	546	SAT1_CF	blue	na
chrUn	54724501	54724606	106	SAT1_CF	blue	na
chrUn	54724620	54725122	503	SAT1_CF	blue	na
chrUn	54725207	54726480	1274	SAT1_CF	blue	na
chrUn	54726493	54727048	556	SAT1_CF	blue	na
chrUn	54727069	54727171	103	SAT1_CF	blue	na
chrUn	54727189	54727686	498	SAT1_CF	blue	na
chrUn	54727773	54729000	1228	SAT1_CF	blue	na
chrUn	54729038	54729581	544	SAT1_CF	blue	na
chrUn	54729623	54730186	564	SAT1_CF	blue	na
chrUn	54730303	54732175	1873	SAT1_CF	blue	na
chrUn	54732202	54732722	521	SAT1_CF	blue	na
chrUn	54732739	54732924	186	SAT1_CF	blue	na
chrUn	54733104	54733269	166	SAT1_CF	blue	na
chrUn	54733311	54733858	548	SAT1_CF	blue	na
chrUn	54733902	54734709	808	SAT1_CF	blue	na
chrUn	54734721	54734857	137	SAT1_CF	blue	na
chrUn	54734868	54735880	1013	SAT1_CF	blue	na
chrUn	54735924	54736597	674	SAT1_CF	blue	na
chrUn	54736609	54737292	684	SAT1_CF	blue	na
chrUn	54737306	54737806	501	SAT1_CF	blue	na
chrUn	54737846	54739122	1277	SAT1_CF	blue	na
chrUn	54739161	54739704	544	SAT1_CF	blue	na
chrUn	54739746	54740606	861	SAT1_CF	blue	na
chrUn	54740620	54741382	763	SAT1_CF	blue	na
chrUn	54742794	54743927	1134	Sat2CF	purple	na
chrUn	54759711	54760296	586	Sat2CF	purple	na
chrUn	54779643	54780979	1337	CarSat1	red	na
chrUn	54781006	54781711	706	CarSat1	red	na
chrUn	54781736	54782453	718	CarSat1	red	na
chrUn	54782480	54785651	3172	CarSat1	red	na
chrUn	54785676	54786602	927	CarSat1	red	na

chrUn	54786630	54789509	2880	CarSat1	red	na
chrUn	54789852	54793011	3160	CarSat1	red	na
chrUn	54793045	54793747	703	CarSat1	red	na
chrUn	54793772	54794697	926	CarSat1	red	na
chrUn	54794735	54798413	3679	CarSat1	red	na
chrUn	54798450	54799393	944	CarSat1	red	na
chrUn	54799418	54800624	1207	CarSat1	red	na
chrUn	54800645	54804886	4242	CarSat1	red	na
chrUn	54832566	54833504	939	CarSat1	red	na
chrUn	54833531	54834494	964	CarSat1	red	na
chrUn	54834519	54835230	712	CarSat1	red	na
chrUn	54835246	54837199	1954	CarSat1	red	na
chrUn	54837215	54837684	470	CarSat1	red	na
chrUn	54837711	54838422	712	CarSat1	red	na
chrUn	54838449	54839897	1449	CarSat1	red	na
chrUn	54839924	54840634	711	CarSat1	red	na
chrUn	54840661	54841624	964	CarSat1	red	na
chrUn	54841649	54842276	628	CarSat1	red	na
chrUn	54842728	54844820	2093	CarSat1	red	na
chrUn	54844835	54844962	128	Carsat2	orange	na
chrUn	54844850	54846081	1232	CarSat1	red	na
chrUn	54846097	54846566	470	CarSat1	red	na
chrUn	54846593	54849755	3163	CarSat1	red	na
chrUn	54849780	54850500	721	CarSat1	red	na
chrUn	54850525	54851452	928	CarSat1	red	na
chrUn	54851482	54851968	487	CarSat1	red	na
chrUn	54852001	54852787	787	CarSat1	red	na
chrUn	54852968	54853122	155	CarSat1	red	na
chrUn	54853152	54854598	1447	CarSat1	red	na
chrUn	54854613	54854740	128	Carsat2	orange	na
chrUn	54854628	54856585	1958	CarSat1	red	na
chrUn	54856618	54857323	706	CarSat1	red	na
chrUn	54884157	54885800	1644	CarSat1	red	na
chrUn	54886879	54889366	2488	CarSat1	red	na
chrUn	54890631	54891311	681	CarSat1	red	na
chrUn	54891341	54892798	1458	CarSat1	red	na
chrUn	54892813	54894752	1940	CarSat1	red	na
chrUn	54894777	54895891	1115	CarSat1	red	na
chrUn	54895924	54896770	847	CarSat1	red	na
chrUn	54901073	54902092	1020	CarSat1	red	na
chrUn	54904087	54906409	2323	CarSat1	red	na
chrUn	54907396	54908395	1000	CarSat1	red	na

chrUn	54908428	54909389	962	CarSat1	red	na
chrUn	54909410	54910045	636	CarSat1	red	na
chrUn	54911294	54912175	882	CarSat1	red	na
chrUn	54912196	54912796	601	CarSat1	red	na
chrUn	54914959	54916936	1978	CarSat1	red	na
chrUn	54916951	54917396	446	CarSat1	red	na
chrUn	54917417	54919132	1716	CarSat1	red	na
chrUn	54919928	54920512	585	CarSat1	red	na
chrUn	54920542	54922388	1847	CarSat1	red	na
chrUn	54930461	54930623	163	Sat2CF	purple	na
chrUn	54930487	54931337	851	Sat2CF	purple	na
chrUn	54930736	54931408	673	Sat2CF	purple	na
chrUn	54951690	54952686	997	SAT1_CF	blue	na
chrUn	54952738	54953264	527	SAT1_CF	blue	na
chrUn	54953302	54954573	1272	SAT1_CF	blue	na
chrUn	54954594	54955742	1149	SAT1_CF	blue	na
chrUn	54955763	54956305	543	SAT1_CF	blue	na
chrUn	54956347	54957612	1266	SAT1_CF	blue	na
chrUn	54957638	54958123	486	SAT1_CF	blue	na
chrUn	54958150	54958897	748	SAT1_CF	blue	na
chrUn	54958909	54959763	855	SAT1_CF	blue	na
chrUn	54959848	54960392	545	SAT1_CF	blue	na
chrUn	54960429	54961627	1199	SAT1_CF	blue	na
chrUn	54961727	54962298	572	SAT1_CF	blue	na
chrUn	54962325	54963342	1018	SAT1_CF	blue	na
chrUn	54963363	54963857	495	SAT1_CF	blue	na
chrUn	54963869	54964545	677	SAT1_CF	blue	na
chrUn	54964606	54966242	1637	SAT1_CF	blue	na
chrUn	54966359	54967520	1162	SAT1_CF	blue	na
chrUn	54967532	54968216	685	SAT1_CF	blue	na
chrUn	54968229	54969068	840	SAT1_CF	blue	na
chrUn	54969081	54970869	1789	SAT1_CF	blue	na
chrUn	54970902	54971233	332	SAT1_CF	blue	na
chrUn	54971320	54972436	1117	SAT1_CF	blue	na
chrUn	54972526	54974479	1954	SAT1_CF	blue	na
chrUn	54974491	54975848	1358	SAT1_CF	blue	na
chrUn	54975891	54976369	479	SAT1_CF	blue	na
chrUn	55108109	55108365	257	Sat15CF	na	na
chrUn	55108109	55108280	172	Sat15CF	na	na
chrUn	55108109	55108365	257	Sat15CF	na	na
chrUn	55108505	55108833	329	Sat15CF	na	na
chrUn	55130462	55130639	178	Sat2CF	purple	na

chrUn	55130531	55131700	1170	Sat2CF	purple	na
chrUn	55132099	55132535	437	CarSat1	red	na
chrUn	55132713	55132835	123	CarSat1	red	na
chrUn	55132856	55133712	857	CarSat1	red	na
chrUn	55133733	55135276	1544	CarSat1	red	na
chrUn	55135472	55138082	2611	CarSat1	red	na
chrUn	55138087	55139087	1001	SAT1_CF	blue	na
chrUn	55139119	55140161	1043	SAT1_CF	blue	na
chrUn	55140215	55141281	1067	SAT1_CF	blue	na
chrUn	55141296	55142119	824	SAT1_CF	blue	na
chrUn	55142139	55143382	1244	SAT1_CF	blue	na
chrUn	55143407	55145254	1848	SAT1_CF	blue	na
chrUn	55146302	55148863	2562	CarSat1	red	na
chrUn	55148897	55150798	1902	CarSat1	red	na
chrUn	55150832	55151805	974	CarSat1	red	na
chrUn	55151838	55152542	705	CarSat1	red	na
chrUn	55152566	55155710	3145	CarSat1	red	na
chrUn	55155740	55156448	709	CarSat1	red	na
chrUn	55156478	55158657	2180	CarSat1	red	na
chrUn	55158687	55160128	1442	CarSat1	red	na
chrUn	55160158	55163080	2923	CarSat1	red	na
chrUn	55163110	55166026	2917	CarSat1	red	na
chrUn	55166056	55167500	1445	CarSat1	red	na
chrUn	55167530	55168236	707	CarSat1	red	na
chrUn	55168266	55169702	1437	CarSat1	red	na
chrUn	55169717	55169843	127	Carsat2	orange	na
chrUn	55169732	55170622	891	CarSat1	red	na
chrUn	55199985	55200178	194	Sat10CF	na	na
chrUn	55200099	55200181	83	Sat10CF	na	na
chrUn	55226159	55226343	185	Sat2CF	purple	na
chrUn	55226184	55226354	171	Sat2CF	purple	na
chrUn	55339762	55340327	566	Sat2CF	purple	na
chrUn	55340203	55340347	145	Sat2CF	purple	na
chrUn	55388731	55389843	1113	Sat2CF	purple	na
chrUn	55408354	55408605	252	Sat2CF	purple	na
chrUn	55408379	55408634	256	Sat2CF	purple	na
chrUn	55408478	55408655	178	Sat2CF	purple	na
chrUn	55415015	55415425	411	CarSat1	red	na
chrUn	55415457	55416181	725	CarSat1	red	na
chrUn	55416224	55417054	831	SAT1_CF	blue	na
chrUn	55417107	55418298	1192	SAT1_CF	blue	na
chrUn	55418335	55420053	1719	SAT1_CF	blue	na

chrUn	55420072	55423201	3130	SAT1_CF	blue	na
chrUn	55423249	55425012	1764	SAT1_CF	blue	na
chrUn	55425027	55426218	1192	CarSat1	red	na
chrUn	55426232	55426872	641	CarSat1	red	na
chrUn	55427029	55427879	851	CarSat1	red	na
chrUn	55427900	55428754	855	CarSat1	red	na
chrUn	55428774	55429626	853	CarSat1	red	na
chrUn	55429647	55430495	849	CarSat1	red	na
chrUn	55430522	55431206	685	CarSat1	red	na
chrUn	55431456	55432228	773	CarSat1	red	na
chrUn	55432328	55432964	637	CarSat1	red	na
chrUn	55433202	55434078	877	CarSat1	red	na
chrUn	55470290	55471127	838	CarSat1	red	na
chrUn	55471142	55473283	2142	CarSat1	red	na
chrUn	55473471	55473532	62	CarSat1	red	na
chrUn	55473562	55475175	1614	CarSat1	red	na
chrUn	55475318	55475970	653	CarSat1	red	na
chrUn	55476000	55478351	2352	CarSat1	red	na
chrUn	55478558	55479888	1331	CarSat1	red	na
chrUn	55479905	55480612	708	CarSat1	red	na
chrUn	55480642	55483583	2942	CarSat1	red	na
chrUn	55483598	55484816	1219	CarSat1	red	na
chrUn	55486539	55488117	1579	CarSat1	red	na
chrUn	55488147	55489611	1465	CarSat1	red	na
chrUn	55489626	55491093	1468	CarSat1	red	na
chrUn	55491123	55492061	939	CarSat1	red	na
chrUn	55492085	55493535	1451	CarSat1	red	na
chrUn	55493559	55494045	487	CarSat1	red	na
chrUn	55494075	55495807	1733	CarSat1	red	na
chrUn	55496863	55498537	1675	CarSat1	red	na
chrUn	55498567	55500749	2183	CarSat1	red	na
chrUn	55500779	55501487	709	CarSat1	red	na
chrUn	55501517	55502225	709	CarSat1	red	na
chrUn	55502255	55505438	3184	CarSat1	red	na
chrUn	55505463	55506175	713	CarSat1	red	na
chrUn	55506200	55507084	885	CarSat1	red	na
chrUn	55507287	55508002	716	CarSat1	red	na
chrUn	55513190	55514296	1107	Sat2CF	purple	na
chrUn	55514499	55515853	1355	SAT1_CF	blue	na
chrUn	55515903	55516305	403	CarSat1	red	na
chrUn	55516324	55517843	1520	CarSat1	red	na
chrUn	55517861	55518543	683	CarSat1	red	na

chrUn	55558997	55560142	1146	CarSat1	red	na
chrUn	55560167	55562055	1889	CarSat1	red	na
chrUn	55562250	55562315	66	CarSat1	red	na
chrUn	55562348	55564783	2436	CarSat1	red	na
chrUn	55564808	55566527	1720	CarSat1	red	na
chrUn	55566557	55567002	446	CarSat1	red	na
chrUn	55567027	55568958	1932	CarSat1	red	na
chrUn	55568991	55570951	1961	CarSat1	red	na
chrUn	55570992	55574357	3366	CarSat1	red	na
chrUn	55574378	55575585	1208	CarSat1	red	na
chrUn	55575610	55576562	953	CarSat1	red	na
chrUn	55576587	55578774	2188	CarSat1	red	na
chrUn	55578801	55579510	710	CarSat1	red	na
chrUn	55579537	55580246	710	CarSat1	red	na
chrUn	55580273	55580984	712	CarSat1	red	na
chrUn	55581011	55581721	711	CarSat1	red	na
chrUn	55581748	55582805	1058	CarSat1	red	na
chrUn	55583810	55584889	1080	SAT1_CF	blue	na
chrUn	55586942	55587692	751	SAT1_CF	blue	na
chrUn	55587750	55588717	968	SAT1_CF	blue	na
chrUn	55590037	55590762	726	SAT1_CF	blue	na
chrUn	55590774	55591270	497	SAT1_CF	blue	na
chrUn	55591333	55592981	1649	SAT1_CF	blue	na
chrUn	55592995	55593695	701	SAT1_CF	blue	na
chrUn	55593737	55594520	784	SAT1_CF	blue	na
chrUn	55594550	55596185	1636	SAT1_CF	blue	na
chrUn	55596375	55596482	108	SAT1_CF	blue	na
chrUn	55596525	55597201	677	SAT1_CF	blue	na
chrUn	55597213	55597896	684	SAT1_CF	blue	na
chrUn	55597909	55598451	543	SAT1_CF	blue	na
chrUn	55598495	55599455	961	SAT1_CF	blue	na
chrUn	55599503	55600905	1403	SAT1_CF	blue	na
chrUn	55602101	55602237	137	SAT1_CF	blue	na
chrUn	55602250	55602793	544	SAT1_CF	blue	na
chrUn	55602850	55603369	520	SAT1_CF	blue	na
chrUn	55603487	55603802	316	SAT1_CF	blue	na
chrUn	55605026	55605601	576	SAT1_CF	blue	na
chrUn	55605686	55605822	137	SAT1_CF	blue	na
chrUn	55605850	55606907	1058	SAT1_CF	blue	na
chrUn	55606962	55607550	589	SAT1_CF	blue	na
chrUn	55607636	55608936	1301	SAT1_CF	blue	na
chrUn	55608948	55609686	739	SAT1_CF	blue	na

chrUn	55609935	55610747	813	SAT1_CF	blue	na
chrUn	55610759	55611441	683	SAT1_CF	blue	na
chrUn	55611454	55611770	317	SAT1_CF	blue	na
chrUn	55616081	55616788	708	SAT1_CF	blue	na
chrUn	55616819	55617715	897	SAT1_CF	blue	na
chrUn	55618742	55619259	518	CarSat1	red	na
chrUn	55619286	55620730	1445	CarSat1	red	na
chrUn	55620751	55622665	1915	CarSat1	red	na
chrUn	55622695	55623674	980	CarSat1	red	na
chrUn	55623701	55624662	962	CarSat1	red	na
chrUn	55624678	55625398	721	CarSat1	red	na
chrUn	55625414	55627608	2195	CarSat1	red	na
chrUn	55627624	55629078	1455	CarSat1	red	na
chrUn	55629094	55630301	1208	CarSat1	red	na
chrUn	55630328	55631369	1042	CarSat1	red	na
chrUn	55632252	55633085	834	CarSat1	red	na
chrUn	55633112	55633541	430	CarSat1	red	na
chrUn	55633581	55636287	2707	CarSat1	red	na
chrUn	55636303	55637971	1669	CarSat1	red	na
chrUn	55638002	55638713	712	CarSat1	red	na
chrUn	55638728	55638855	128	Carsat2	orange	na
chrUn	55638743	55639967	1225	CarSat1	red	na
chrUn	55639991	55641455	1465	CarSat1	red	na
chrUn	55641471	55642409	939	CarSat1	red	na
chrUn	55642424	55642551	128	Carsat2	orange	na
chrUn	55642440	55643276	837	CarSat1	red	na
chrUn	55644323	55645120	798	CarSat1	red	na
chrUn	55645136	55645605	470	CarSat1	red	na
chrUn	55645632	55646594	963	CarSat1	red	na
chrUn	55646610	55647813	1204	CarSat1	red	na
chrUn	55647829	55650028	2200	CarSat1	red	na
chrUn	55650055	55651095	1041	CarSat1	red	na
chrUn	55652681	55655499	2819	CarSat1	red	na
chrUn	55656021	55657249	1229	CarSat1	red	na
chrUn	55657265	55658398	1134	CarSat1	red	na
chrUn	55658830	55660055	1226	CarSat1	red	na
chrUn	55660071	55660540	470	CarSat1	red	na
chrUn	55660567	55661278	712	CarSat1	red	na
chrUn	55661305	55662015	711	CarSat1	red	na
chrUn	55662042	55663487	1446	CarSat1	red	na
chrUn	55663514	55664223	710	CarSat1	red	na
chrUn	55664250	55665698	1449	CarSat1	red	na

chrUn	55665725	55667173	1449	CarSat1	red	na
chrUn	55667200	55667910	711	CarSat1	red	na
chrUn	55667937	55669637	1701	CarSat1	red	na
chrUn	55669653	55670454	802	CarSat1	red	na
chrUn	55672132	55673382	1251	Sat2CF	purple	na
chrUn	55672262	55673346	1085	Sat2CF	purple	na
chrUn	55673222	55673440	219	Sat2CF	purple	na
chrUn	55675853	55675993	141	CarSat1	red	na
chrUn	55676014	55677171	1158	CarSat1	red	na
chrUn	55677344	55677477	134	CarSat1	red	na
chrUn	55677493	55677793	301	CarSat1	red	na
chrUn	55677814	55678748	935	CarSat1	red	na
chrUn	55678937	55679504	568	CarSat1	red	na
chrUn	55688569	55688976	408	CarSat1	red	na
chrUn	55689010	55690782	1773	CarSat1	red	na
chrUn	55690808	55691210	403	CarSat1	red	na
chrUn	55691242	55691645	404	SAT1_CF	blue	na
chrUn	55691675	55692604	930	SAT1_CF	blue	na
chrUn	55692799	55692959	161	Sat2CF	purple	na
chrUn	55692834	55693843	1010	Sat2CF	purple	na
chrUn	55692920	55693780	861	Sat2CF	purple	na
chrUn	55759090	55759856	767	Sat2CF	purple	na
chrUn	55762193	55763058	866	CarSat1	red	na
chrUn	55763080	55764794	1715	CarSat1	red	na
chrUn	55764824	55766138	1315	CarSat1	red	na
chrUn	55766115	55766291	177	Carsat2	orange	na
chrUn	55766302	55767231	930	CarSat1	red	na
chrUn	55767265	55767748	484	CarSat1	red	na
chrUn	55767778	55768500	723	CarSat1	red	na
chrUn	55768515	55769237	723	CarSat1	red	na
chrUn	55769252	55769974	723	CarSat1	red	na
chrUn	55769989	55772930	2942	CarSat1	red	na
chrUn	55772945	55774142	1198	CarSat1	red	na
chrUn	55774176	55775346	1171	CarSat1	red	na
chrUn	55775379	55777354	1976	CarSat1	red	na
chrUn	55777369	55778076	708	CarSat1	red	na
chrUn	55778106	55778814	709	CarSat1	red	na
chrUn	55778844	55779565	722	CarSat1	red	na
chrUn	55779580	55780301	722	CarSat1	red	na
chrUn	55780316	55781042	727	CarSat1	red	na
chrUn	55781057	55782515	1459	CarSat1	red	na
chrUn	55782530	55783256	727	CarSat1	red	na

chrUn	55783271	55783978	708	CarSat1	red	na
chrUn	55784008	55784730	723	CarSat1	red	na
chrUn	55784745	55785756	1012	CarSat1	red	na
chrUn	55800322	55801310	989	CarSat1	red	na
chrUn	55801331	55802195	865	CarSat1	red	na
chrUn	55802345	55803804	1460	CarSat1	red	na
chrUn	55803964	55804355	392	CarSat1	red	na
chrUn	55804370	55808433	4064	SAT1_CF	blue	na
chrUn	55808445	55809930	1486	SAT1_CF	blue	na
chrUn	55809965	55810316	352	SAT1_CF	blue	na
chrUn	55847547	55848439	893	CarSat1	red	na
chrUn	55848662	55850111	1450	CarSat1	red	na
chrUn	55850132	55850914	783	CarSat1	red	na
chrUn	55850978	55851885	908	CarSat1	red	na
chrUn	55851906	55853437	1532	CarSat1	red	na
chrUn	55853521	55855117	1597	CarSat1	red	na
chrUn	55855148	55856730	1583	CarSat1	red	na
chrUn	55856761	55858343	1583	CarSat1	red	na
chrUn	55863489	55864482	994	SAT1_CF	blue	na
chrUn	55864503	55865041	539	SAT1_CF	blue	na
chrUn	55865090	55866116	1027	SAT1_CF	blue	na
chrUn	55866159	55867204	1046	SAT1_CF	blue	na
chrUn	55867232	55867903	672	SAT1_CF	blue	na
chrUn	55867946	55868815	870	SAT1_CF	blue	na
chrUn	55878221	55882034	3814	SAT1_CF	blue	na
chrUn	55882052	55883144	1093	SAT1_CF	blue	na
chrUn	55883157	55883661	505	SAT1_CF	blue	na
chrUn	55883674	55885784	2111	SAT1_CF	blue	na
chrUn	55885835	55886381	547	SAT1_CF	blue	na
chrUn	55886423	55887827	1405	SAT1_CF	blue	na
chrUn	55887846	55888447	602	SAT1_CF	blue	na
chrUn	55895555	55896340	786	Sat2CF	purple	na
chrUn	55896019	55896399	381	Sat2CF	purple	na
chrUn	55911540	55912125	586	Sat2CF	purple	na
chrUn	55911569	55912084	516	Sat2CF	purple	na
chrUn	55913849	55915594	1746	CarSat1	red	na
chrUn	55915610	55918325	2716	CarSat1	red	na
chrUn	55918341	55918777	437	CarSat1	red	na
chrUn	55918804	55919515	712	CarSat1	red	na
chrUn	55919536	55920981	1446	CarSat1	red	na
chrUn	55921001	55922456	1456	CarSat1	red	na
chrUn	55922724	55923883	1160	CarSat1	red	na

chrUn	55923911	55924054	144	CarSat1	red	na
chrUn	55924248	55924958	711	CarSat1	red	na
chrUn	55933511	55934100	590	CarSat1	red	na
chrUn	55934301	55935845	1545	CarSat1	red	na
chrUn	55938247	55939929	1683	CarSat1	red	na
chrUn	55939956	55941401	1446	CarSat1	red	na
chrUn	55941428	55946800	5373	CarSat1	red	na
chrUn	55946824	55947546	723	CarSat1	red	na
chrUn	55947562	55948274	713	CarSat1	red	na
chrUn	55948298	55949012	715	CarSat1	red	na
chrUn	55949036	55949750	715	CarSat1	red	na
chrUn	55949774	55953189	3416	CarSat1	red	na
chrUn	55953216	55954665	1450	CarSat1	red	na
chrUn	55954691	55955654	964	CarSat1	red	na
chrUn	55955670	55956391	722	CarSat1	red	na
chrUn	55956407	55959331	2925	CarSat1	red	na
chrUn	55959356	55960563	1208	CarSat1	red	na
chrUn	55960590	55961728	1139	CarSat1	red	na
chrUn	55962729	55963339	611	CarSat1	red	na
chrUn	55963366	55964076	711	CarSat1	red	na
chrUn	55964103	55966287	2185	CarSat1	red	na
chrUn	55966314	55966911	598	CarSat1	red	na
chrUn	55967137	55967265	129	Carsat2	orange	na
chrUn	55967150	55971453	4304	CarSat1	red	na
chrUn	55971469	55972182	714	CarSat1	red	na
chrUn	55972207	55973167	961	CarSat1	red	na
chrUn	55974059	55975377	1319	CarSat1	red	na
chrUn	55975631	55976114	484	CarSat1	red	na
chrUn	55976141	55976851	711	CarSat1	red	na
chrUn	55977545	55978324	780	CarSat1	red	na
chrUn	55978351	55979061	711	CarSat1	red	na
chrUn	55979092	55979555	464	CarSat1	red	na
chrUn	55979571	55980862	1292	CarSat1	red	na
chrUn	55983742	55984211	470	CarSat1	red	na
chrUn	55984238	55984947	710	CarSat1	red	na
chrUn	55984974	55986421	1448	CarSat1	red	na
chrUn	55986448	55987057	610	CarSat1	red	na
chrUn	55987252	55987951	700	CarSat1	red	na
chrUn	55991648	55992238	591	CarSat1	red	na
chrUn	55992259	55993045	787	CarSat1	red	na
chrUn	55993072	55993840	769	CarSat1	red	na
chrUn	55993870	55994571	702	CarSat1	red	na

chrUn	56016910	56017214	305	Sat7CF	lime	na
chrUn	56017008	56017245	238	Sat7CF	lime	na
chrUn	56016910	56017214	305	Sat7CF	lime	na
chrUn	56017008	56017245	238	Sat7CF	lime	na
chrUn	56053039	56054237	1199	CarSat1	red	na
chrUn	56054264	56054974	711	CarSat1	red	na
chrUn	56055001	56055711	711	CarSat1	red	na
chrUn	56055738	56056448	711	CarSat1	red	na
chrUn	56056475	56057923	1449	CarSat1	red	na
chrUn	56057950	56058660	711	CarSat1	red	na
chrUn	56058687	56060386	1700	CarSat1	red	na
chrUn	56060402	56060866	465	CarSat1	red	na
chrUn	56060897	56062050	1154	CarSat1	red	na
chrUn	56062154	56064854	2701	CarSat1	red	na
chrUn	56064870	56066324	1455	CarSat1	red	na
chrUn	56066340	56067547	1208	CarSat1	red	na
chrUn	56067574	56068284	711	CarSat1	red	na
chrUn	56068311	56069021	711	CarSat1	red	na
chrUn	56069048	56069758	711	CarSat1	red	na
chrUn	56069785	56071486	1702	CarSat1	red	na
chrUn	56071511	56075624	4114	CarSat1	red	na
chrUn	56075640	56076258	619	CarSat1	red	na
chrUn	56159225	56159970	746	SAT1_CF	blue	na
chrUn	56162953	56163445	493	SAT1_CF	blue	na
chrUn	56163469	56164069	601	SAT1_CF	blue	na
chrUn	56164082	56164583	502	SAT1_CF	blue	na
chrUn	56164623	56165025	403	SAT1_CF	blue	na
chrUn	56165038	56166698	1661	SAT1_CF	blue	na
chrUn	56166741	56168111	1371	SAT1_CF	blue	na
chrUn	56168124	56168853	730	SAT1_CF	blue	na
chrUn	56171649	56172419	771	SAT1_CF	blue	na
chrUn	56172432	56172953	522	SAT1_CF	blue	na
chrUn	56172979	56173085	107	SAT1_CF	blue	na
chrUn	56178954	56180763	1810	SAT1_CF	blue	na
chrUn	56180783	56181257	475	SAT1_CF	blue	na
chrUn	56181317	56181899	583	SAT1_CF	blue	na
chrUn	56182626	56184177	1552	SAT1_CF	blue	na
chrUn	56184217	56184720	504	SAT1_CF	blue	na
chrUn	56184758	56184815	58	SAT1_CF	blue	na
chrUn	56185821	56186607	787	Sat8CF	dgreen	na
chrUn	56189092	56189469	378	Sat8CF	dgreen	na
chrUn	56197212	56197493	282	Sat8CF	dgreen	na

chrUn	56197279	56198708	1430	Sat8CF	dgreen	na
chrUn	56197886	56199038	1153	Sat8CF	dgreen	na
chrUn	56200912	56202183	1272	Sat8CF	dgreen	na
chrUn	56201061	56202489	1429	Sat8CF	dgreen	na
chrUn	56201667	56203096	1430	Sat8CF	dgreen	na
chrUn	56203190	56204624	1435	Sat8CF	dgreen	na
chrUn	56207549	56208956	1408	Sat8CF	dgreen	na
chrUn	56208134	56209564	1431	Sat8CF	dgreen	na
chrUn	56209658	56211092	1435	Sat8CF	dgreen	na
chrUn	56210271	56211229	959	Sat8CF	dgreen	na
chrUn	56211923	56212546	624	Sat8CF	dgreen	na
chrUn	56212584	56213680	1097	Sat8CF	dgreen	na
chrUn	56212857	56214287	1431	Sat8CF	dgreen	na
chrUn	56214381	56215811	1431	Sat8CF	dgreen	na
chrUn	56215905	56217339	1435	Sat8CF	dgreen	na
chrUn	56221690	56222203	514	Sat8CF	dgreen	na
chrUn	56223203	56226603	3401	CarSat1	red	na
chrUn	56226627	56228818	2192	CarSat1	red	na
chrUn	56228842	56231540	2699	CarSat1	red	na
chrUn	56231561	56232522	962	CarSat1	red	na
chrUn	56232546	56235468	2923	CarSat1	red	na
chrUn	56235492	56236433	942	CarSat1	red	na
chrUn	56236463	56236915	453	CarSat1	red	na
chrUn	56237182	56240614	3433	CarSat1	red	na
chrUn	56240638	56241349	712	CarSat1	red	na
chrUn	56241373	56244295	2923	CarSat1	red	na
chrUn	56244319	56245031	713	CarSat1	red	na
chrUn	56245064	56245769	706	CarSat1	red	na
chrUn	56245793	56246567	775	CarSat1	red	na
chrUn	56284167	56284805	639	CarSat1	red	na
chrUn	56284830	56286061	1232	CarSat1	red	na
chrUn	56286098	56287534	1437	CarSat1	red	na
chrUn	56287571	56288745	1175	CarSat1	red	na
chrUn	56288766	56289235	470	CarSat1	red	na
chrUn	56289260	56289963	704	CarSat1	red	na
chrUn	56289997	56291911	1915	CarSat1	red	na
chrUn	56291948	56293667	1720	CarSat1	red	na
chrUn	56294106	56295092	987	CarSat1	red	na
chrUn	56295336	56296974	1639	CarSat1	red	na
chrUn	56297908	56298637	730	CarSat1	red	na
chrUn	56298658	56299372	715	CarSat1	red	na
chrUn	56299393	56300362	970	CarSat1	red	na

chrUn	56302498	56305021	2524	CarSat1	red	na
chrUn	56305046	56306011	966	CarSat1	red	na
chrUn	56306032	56307482	1451	CarSat1	red	na
chrUn	56307509	56308220	712	CarSat1	red	na
chrUn	56308247	56308957	711	CarSat1	red	na
chrUn	56308984	56310964	1981	CarSat1	red	na
chrUn	56360966	56362053	1088	CarSat1	red	na
chrUn	56362068	56362995	928	CarSat1	red	na
chrUn	56363029	56364256	1228	CarSat1	red	na
chrUn	56364293	56365221	929	CarSat1	red	na
chrUn	56365246	56369417	4172	CarSat1	red	na
chrUn	56369447	56370630	1184	CarSat1	red	na
chrUn	56370651	56371643	993	CarSat1	red	na
chrUn	56371658	56372583	926	CarSat1	red	na
chrUn	56373352	56374729	1378	CarSat1	red	na
chrUn	56374787	56376586	1800	CarSat1	red	na
chrUn	56376606	56378793	2188	CarSat1	red	na
chrUn	56378814	56380745	1932	CarSat1	red	na
chrUn	56380778	56381490	713	CarSat1	red	na
chrUn	56381514	56382218	705	CarSat1	red	na
chrUn	56382251	56384753	2503	CarSat1	red	na
chrUn	56436669	56437098	430	SAT1_CF	blue	na
chrUn	56439858	56440152	295	Sat2CF	purple	na
chrUn	56442939	56445348	2410	SAT1_CF	blue	na
chrUn	56445359	56445998	640	SAT1_CF	blue	na
chrUn	56446043	56447403	1361	SAT1_CF	blue	na
chrUn	56448418	56449036	619	SAT1_CF	blue	na
chrUn	56449116	56449711	596	SAT1_CF	blue	na
chrUn	56449766	56451447	1682	SAT1_CF	blue	na
chrUn	56451459	56451979	521	SAT1_CF	blue	na
chrUn	56452026	56452159	134	SAT1_CF	blue	na
chrUn	56452177	56453199	1023	SAT1_CF	blue	na
chrUn	56453241	56453789	549	SAT1_CF	blue	na
chrUn	56453833	56454486	654	SAT1_CF	blue	na
chrUn	56454498	56457639	3142	SAT1_CF	blue	na
chrUn	56457652	56458049	398	SAT1_CF	blue	na
chrUn	56458095	56458723	629	SAT1_CF	blue	na
chrUn	56458788	56459363	576	SAT1_CF	blue	na
chrUn	56459374	56460098	725	SAT1_CF	blue	na
chrUn	56460112	56460604	493	SAT1_CF	blue	na
chrUn	56460615	56461297	683	SAT1_CF	blue	na
chrUn	56461309	56461992	684	SAT1_CF	blue	na

chrUn	56462005	56462637	633	SAT1_CF	blue	na
chrUn	56462658	56462767	110	SAT1_CF	blue	na
chrUn	56462779	56463280	502	SAT1_CF	blue	na
chrUn	56463320	56464280	961	SAT1_CF	blue	na
chrUn	56464358	56464664	307	SAT1_CF	blue	na
chrUn	56464708	56465354	647	SAT1_CF	blue	na
chrUn	56465366	56465910	545	SAT1_CF	blue	na
chrUn	56465924	56467537	1614	SAT1_CF	blue	na
chrUn	56467580	56468256	677	SAT1_CF	blue	na
chrUn	56468268	56469254	987	SAT1_CF	blue	na
chrUn	56469275	56470233	959	SAT1_CF	blue	na
chrUn	56470277	56470746	470	SAT1_CF	blue	na
chrUn	56470762	56471442	681	SAT1_CF	blue	na
chrUn	56510482	56511358	877	CarSat1	red	na
chrUn	56511373	56511496	124	Carsat2	orange	na
chrUn	56511388	56511872	485	CarSat1	red	na
chrUn	56511905	56513753	1849	CarSat1	red	na
chrUn	56516452	56517210	759	CarSat1	red	na
chrUn	56517235	56517956	722	CarSat1	red	na
chrUn	56517981	56518689	709	CarSat1	red	na
chrUn	56518714	56520419	1706	CarSat1	red	na
chrUn	56520702	56522154	1453	CarSat1	red	na
chrUn	56522175	56522621	447	CarSat1	red	na
chrUn	56522651	56523606	956	CarSat1	red	na
chrUn	56523652	56526311	2660	CarSat1	red	na
chrUn	56526341	56527049	709	CarSat1	red	na
chrUn	56527079	56527562	484	CarSat1	red	na
chrUn	56528205	56529325	1121	CarSat1	red	na
chrUn	56529355	56530456	1102	CarSat1	red	na
chrUn	56530584	56531592	1009	CarSat1	red	na
chrUn	56531622	56532657	1036	CarSat1	red	na
chrUn	56533199	56534106	908	CarSat1	red	na
chrUn	56534137	56535698	1562	CarSat1	red	na
chrUn	56535737	56536994	1258	CarSat1	red	na
chrUn	56537024	56537651	628	CarSat1	red	na
chrUn	56544362	56545446	1085	Sat2CF	purple	na
chrUn	56573841	56574156	316	SAT1_CF	blue	na
chrUn	56581679	56582929	1251	Sat2CF	purple	na
chrUn	56581809	56582893	1085	Sat2CF	purple	na
chrUn	56586239	56587372	1134	CarSat1	red	na
chrUn	56589040	56590078	1039	CarSat1	red	na
chrUn	56607507	56607782	276	Carsat3	green	na

chrUn	56623306	56623634	329	Carsat3	green	na
chrUn	56662305	56663015	711	CarSat1	red	na
chrUn	56663045	56663753	709	CarSat1	red	na
chrUn	56663783	56665226	1444	CarSat1	red	na
chrUn	56665257	56667435	2179	CarSat1	red	na
chrUn	56667465	56669632	2168	CarSat1	red	na
chrUn	56669669	56670154	486	CarSat1	red	na
chrUn	56670187	56673313	3127	CarSat1	red	na
chrUn	56673353	56674794	1442	CarSat1	red	na
chrUn	56674824	56677516	2693	CarSat1	red	na
chrUn	56677549	56679216	1668	CarSat1	red	na
chrUn	56679231	56679358	128	Carsat2	orange	na
chrUn	56679246	56679953	708	CarSat1	red	na
chrUn	56679968	56680094	127	Carsat2	orange	na
chrUn	56679983	56680689	707	CarSat1	red	na
chrUn	56680704	56680830	127	Carsat2	orange	na
chrUn	56680719	56681425	707	CarSat1	red	na
chrUn	56681440	56681567	128	Carsat2	orange	na
chrUn	56681455	56681941	487	CarSat1	red	na
chrUn	56681974	56682898	925	CarSat1	red	na
chrUn	56682928	56683635	708	CarSat1	red	na
chrUn	56683650	56683777	128	Carsat2	orange	na
chrUn	56683666	56684321	656	CarSat1	red	na
chrUn	56684531	56684956	426	CarSat1	red	na
chrUn	56709631	56710631	1001	SAT1_CF	blue	na
chrUn	56710643	56713564	2922	SAT1_CF	blue	na
chrUn	56713609	56715080	1472	SAT1_CF	blue	na
chrUn	56715170	56716995	1826	SAT1_CF	blue	na
chrUn	56717037	56717691	655	SAT1_CF	blue	na
chrUn	56717763	56719400	1638	SAT1_CF	blue	na
chrUn	56719413	56720093	681	SAT1_CF	blue	na
chrUn	56720105	56721400	1296	SAT1_CF	blue	na
chrUn	56721412	56722635	1224	SAT1_CF	blue	na
chrUn	56722648	56723754	1107	SAT1_CF	blue	na
chrUn	56723774	56724607	834	SAT1_CF	blue	na
chrUn	56724677	56725221	545	SAT1_CF	blue	na
chrUn	56725266	56726253	988	SAT1_CF	blue	na
chrUn	56726310	56727268	959	SAT1_CF	blue	na
chrUn	56727308	56727808	501	SAT1_CF	blue	na
chrUn	56727835	56728583	749	SAT1_CF	blue	na
chrUn	56728601	56729258	658	SAT1_CF	blue	na
chrUn	56729309	56730116	808	SAT1_CF	blue	na

chrUn	56730148	56730653	506	SAT1_CF	blue	na
chrUn	56730665	56731341	677	SAT1_CF	blue	na
chrUn	56731411	56732266	856	SAT1_CF	blue	na
chrUn	56733306	56735407	2102	CarSat1	red	na
chrUn	56735437	56736145	709	CarSat1	red	na
chrUn	56736175	56736613	439	CarSat1	red	na
chrUn	56736642	56738582	1941	CarSat1	red	na
chrUn	56738598	56740045	1448	CarSat1	red	na
chrUn	56740070	56741030	961	CarSat1	red	na
chrUn	56741057	56742035	979	CarSat1	red	na
chrUn	56742065	56742506	442	CarSat1	red	na
chrUn	56742533	56745458	2926	CarSat1	red	na
chrUn	56745485	56746192	708	CarSat1	red	na
chrUn	56746221	56747153	933	CarSat1	red	na
chrUn	56747934	56748608	675	CarSat1	red	na
chrUn	56748635	56749345	711	CarSat1	red	na
chrUn	56749378	56750352	975	CarSat1	red	na
chrUn	56750382	56750822	441	CarSat1	red	na
chrUn	56750849	56752296	1448	CarSat1	red	na
chrUn	56752323	56754267	1945	CarSat1	red	na
chrUn	56754292	56755510	1219	CarSat1	red	na
chrUn	56755540	56756649	1110	CarSat1	red	na
chrUn	56762222	56763763	1542	CarSat1	red	na
chrUn	56763790	56765274	1485	CarSat1	red	na
chrUn	56774674	56775357	684	CarSat1	red	na
chrUn	56775375	56776891	1517	CarSat1	red	na
chrUn	56776910	56777312	403	CarSat1	red	na
chrUn	56777309	56778644	1336	SAT1_CF	blue	na
chrUn	56778872	56779884	1013	Sat2CF	purple	na
chrUn	56887379	56888078	700	CarSat1	red	na
chrUn	56888278	56888890	613	CarSat1	red	na
chrUn	56888907	56890614	1708	CarSat1	red	na
chrUn	56890639	56891352	714	CarSat1	red	na
chrUn	56891377	56892820	1444	CarSat1	red	na
chrUn	56892831	56893770	940	CarSat1	red	na
chrUn	56893800	56894504	705	CarSat1	red	na
chrUn	56894539	56895763	1225	CarSat1	red	na
chrUn	56895789	56897721	1933	CarSat1	red	na
chrUn	56897742	56899439	1698	CarSat1	red	na
chrUn	56899464	56900670	1207	CarSat1	red	na
chrUn	56900691	56901408	718	CarSat1	red	na
chrUn	56901433	56902386	954	CarSat1	red	na

chrUn	56902419	56904436	2018	CarSat1	red	na
chrUn	56937050	56937923	874	SAT1_CF	blue	na
chrUn	56937965	56938640	676	SAT1_CF	blue	na
chrUn	56938652	56939771	1120	SAT1_CF	blue	na
chrUn	56939837	56941633	1797	SAT1_CF	blue	na
chrUn	56941676	56942349	674	SAT1_CF	blue	na
chrUn	56942362	56943252	891	SAT1_CF	blue	na
chrUn	56943266	56943758	493	SAT1_CF	blue	na
chrUn	56943769	56945059	1291	SAT1_CF	blue	na
chrUn	56945129	56945462	334	SAT1_CF	blue	na
chrUn	56945907	56948391	2485	SAT1_CF	blue	na
chrUn	56948404	56948913	510	SAT1_CF	blue	na
chrUn	56948945	56949805	861	SAT1_CF	blue	na
chrUn	56949853	56950534	682	SAT1_CF	blue	na
chrUn	56950552	56951040	489	SAT1_CF	blue	na
chrUn	56951106	56951241	136	SAT1_CF	blue	na
chrUn	56951269	56951780	512	SAT1_CF	blue	na
chrUn	56951824	56952897	1074	SAT1_CF	blue	na
chrUn	56952940	56953800	861	SAT1_CF	blue	na
chrUn	56953812	56954571	760	SAT1_CF	blue	na
chrUn	56954584	56955085	502	SAT1_CF	blue	na
chrUn	56955172	56955661	490	SAT1_CF	blue	na
chrUn	56955701	56956622	922	SAT1_CF	blue	na
chrUn	56956653	56958108	1456	SAT1_CF	blue	na
chrUn	56958164	56959151	988	SAT1_CF	blue	na
chrUn	56959164	56959933	770	SAT1_CF	blue	na
chrUn	57027424	57030456	3033	SAT1_CF	blue	na
chrUn	57030499	57031040	542	SAT1_CF	blue	na
chrUn	57031057	57034592	3536	SAT1_CF	blue	na
chrUn	57034609	57034803	195	SAT1_CF	blue	na
chrUn	57034818	57035329	512	SAT1_CF	blue	na
chrUn	57035356	57036051	696	SAT1_CF	blue	na
chrUn	57036063	57036761	699	SAT1_CF	blue	na
chrUn	57036804	57038153	1350	SAT1_CF	blue	na
chrUn	57038146	57038870	725	CarSat1	red	na
chrUn	57038932	57039321	390	CarSat1	red	na
chrUn	57075344	57076308	965	CarSat1	red	na
chrUn	57076333	57078520	2188	CarSat1	red	na
chrUn	57078536	57079258	723	CarSat1	red	na
chrUn	57079283	57080943	1661	CarSat1	red	na
chrUn	57080973	57082942	1970	CarSat1	red	na
chrUn	57082958	57083679	722	CarSat1	red	na

chrUn	57083704	57084416	713	CarSat1	red	na
chrUn	57084441	57085143	703	CarSat1	red	na
chrUn	57085417	57087148	1732	CarSat1	red	na
chrUn	57087173	57087892	720	CarSat1	red	na
chrUn	57087908	57088376	469	CarSat1	red	na
chrUn	57088403	57089115	713	CarSat1	red	na
chrUn	57089140	57089850	711	CarSat1	red	na
chrUn	57089877	57090839	963	CarSat1	red	na
chrUn	57090855	57092795	1941	CarSat1	red	na
chrUn	57092822	57094272	1451	CarSat1	red	na
chrUn	57094291	57095260	970	CarSat1	red	na
chrUn	57095285	57096211	927	CarSat1	red	na
chrUn	57096226	57096353	128	Carsat2	orange	na
chrUn	57096241	57096949	709	CarSat1	red	na
chrUn	57096977	57097815	839	CarSat1	red	na
chrUn	57098831	57099553	723	CarSat1	red	na
chrUn	57099569	57100290	722	CarSat1	red	na
chrUn	57100315	57101763	1449	CarSat1	red	na
chrUn	57101790	57104484	2695	CarSat1	red	na
chrUn	57104514	57106926	2413	CarSat1	red	na
chrUn	57106951	57107908	958	CarSat1	red	na
chrUn	57107933	57108398	466	CarSat1	red	na
chrUn	57108422	57109135	714	CarSat1	red	na
chrUn	57109160	57110115	956	CarSat1	red	na
chrUn	57110136	57111588	1453	CarSat1	red	na
chrUn	57111609	57112328	720	CarSat1	red	na
chrUn	57112349	57113555	1207	CarSat1	red	na
chrUn	57113580	57115752	2173	CarSat1	red	na
chrUn	57115777	57118221	2445	CarSat1	red	na
chrUn	57118242	57118710	469	CarSat1	red	na
chrUn	57118735	57121062	2328	CarSat1	red	na
chrUn	57145287	57145738	452	SAT1_CF	blue	na
chrUn	57145782	57146330	549	SAT1_CF	blue	na
chrUn	57146343	57147004	662	SAT1_CF	blue	na
chrUn	57147016	57148941	1926	SAT1_CF	blue	na
chrUn	57148953	57149627	675	SAT1_CF	blue	na
chrUn	57149697	57150173	477	SAT1_CF	blue	na
chrUn	57150185	57150850	666	SAT1_CF	blue	na
chrUn	57150893	57151446	554	SAT1_CF	blue	na
chrUn	57151459	57151961	503	SAT1_CF	blue	na
chrUn	57152044	57152784	741	SAT1_CF	blue	na
chrUn	57152797	57153201	405	SAT1_CF	blue	na

chrUn	57153239	57153740	502	SAT1_CF	blue	na
chrUn	57153753	57154672	920	SAT1_CF	blue	na
chrUn	57154714	57155496	783	SAT1_CF	blue	na
chrUn	57155574	57156495	922	SAT1_CF	blue	na
chrUn	57156517	57157723	1207	SAT1_CF	blue	na
chrUn	57157734	57158436	703	SAT1_CF	blue	na
chrUn	57158464	57159127	664	SAT1_CF	blue	na
chrUn	57159145	57159658	514	SAT1_CF	blue	na
chrUn	57159881	57160576	696	SAT1_CF	blue	na
chrUn	57160589	57160695	107	SAT1_CF	blue	na
chrUn	57160718	57161772	1055	SAT1_CF	blue	na
chrUn	57161785	57162285	501	SAT1_CF	blue	na
chrUn	57162303	57163784	1482	SAT1_CF	blue	na
chrUn	57163795	57164197	403	SAT1_CF	blue	na
chrUn	57164237	57165350	1114	SAT1_CF	blue	na
chrUn	57165393	57167484	2092	SAT1_CF	blue	na
chrUn	57168495	57171234	2740	CarSat1	red	na
chrUn	57171257	57174457	3201	CarSat1	red	na
chrUn	57174494	57176148	1655	CarSat1	red	na
chrUn	57176181	57177135	955	CarSat1	red	na
chrUn	57177162	57177873	712	CarSat1	red	na
chrUn	57177900	57178609	710	CarSat1	red	na
chrUn	57178636	57180078	1443	CarSat1	red	na
chrUn	57180105	57180565	461	CarSat1	red	na
chrUn	57180590	57181553	964	CarSat1	red	na
chrUn	57181580	57182573	994	CarSat1	red	na
chrUn	57182589	57183027	439	CarSat1	red	na
chrUn	57183054	57183764	711	CarSat1	red	na
chrUn	57183791	57184501	711	CarSat1	red	na
chrUn	57184528	57185522	995	CarSat1	red	na
chrUn	57185537	57186463	927	CarSat1	red	na
chrUn	57186488	57187452	965	CarSat1	red	na
chrUn	57187479	57188188	710	CarSat1	red	na
chrUn	57188215	57189208	994	CarSat1	red	na
chrUn	57189224	57189932	709	CarSat1	red	na
chrUn	57189962	57190644	683	CarSat1	red	na
chrUn	57218871	57219949	1079	CarSat1	red	na
chrUn	57220124	57220257	134	CarSat1	red	na
chrUn	57220273	57220567	295	CarSat1	red	na
chrUn	57220594	57221528	935	CarSat1	red	na
chrUn	57221717	57222284	568	CarSat1	red	na
chrUn	57231589	57231996	408	CarSat1	red	na

chrUn	57232030	57233809	1780	CarSat1	red	na
chrUn	57233828	57234230	403	CarSat1	red	na
chrUn	57234262	57234664	403	SAT1_CF	blue	na
chrUn	57234694	57235623	930	SAT1_CF	blue	na
chrUn	57243462	57243969	508	CarSat1	red	na
chrUn	57244002	57245696	1695	CarSat1	red	na
chrUn	57245723	57246434	712	CarSat1	red	na
chrUn	57246461	57248914	2454	CarSat1	red	na
chrUn	57248944	57250384	1441	CarSat1	red	na
chrUn	57250414	57255537	5124	CarSat1	red	na
chrUn	57255552	57258473	2922	CarSat1	red	na
chrUn	57258503	57260679	2177	CarSat1	red	na
chrUn	57260709	57261417	709	CarSat1	red	na
chrUn	57261447	57265577	4131	CarSat1	red	na
chrUn	57314183	57315757	1575	CarSat1	red	na
chrUn	57316737	57317401	665	CarSat1	red	na
chrUn	57317431	57317924	494	CarSat1	red	na
chrUn	57317940	57318658	719	CarSat1	red	na
chrUn	57318674	57319310	637	CarSat1	red	na
chrUn	57319580	57320916	1337	CarSat1	red	na
chrUn	57320932	57321402	471	CarSat1	red	na
chrUn	57321423	57322302	880	CarSat1	red	na
chrUn	57323203	57324804	1602	CarSat1	red	na
chrUn	57324834	57325540	707	CarSat1	red	na
chrUn	57325570	57326502	933	CarSat1	red	na
chrUn	57326519	57327647	1129	CarSat1	red	na
chrUn	57327672	57329176	1505	CarSat1	red	na
chrUn	57329336	57331866	2531	CarSat1	red	na
chrUn	57331896	57332600	705	CarSat1	red	na
chrUn	57332612	57332742	131	Carsat2	orange	na
chrUn	57332630	57333123	494	CarSat1	red	na
chrUn	57333148	57333858	711	CarSat1	red	na
chrUn	57333883	57334595	713	CarSat1	red	na
chrUn	57334611	57335247	637	CarSat1	red	na
chrUn	57336892	57338605	1714	CarSat1	red	na
chrUn	57341254	57342776	1523	CarSat1	red	na
chrUn	57375513	57376285	773	Sat2CF	purple	na
chrUn	57393730	57394874	1145	Sat2CF	purple	na
chrUn	57394207	57394953	747	Sat2CF	purple	na
chrUn	57459489	57460621	1133	Sat2CF	purple	na
chrUn	57460510	57460655	146	Sat2CF	purple	na
chrUn	57513908	57514671	764	SAT1_CF	blue	na

chrUn	57514693	57514797	105	SAT1_CF	blue	na
chrUn	57514814	57515362	549	SAT1_CF	blue	na
chrUn	57515405	57516398	994	SAT1_CF	blue	na
chrUn	57516418	57517429	1012	SAT1_CF	blue	na
chrUn	57517482	57517984	503	SAT1_CF	blue	na
chrUn	57517996	57518680	685	SAT1_CF	blue	na
chrUn	57518723	57519271	549	SAT1_CF	blue	na
chrUn	57519282	57519469	188	SAT1_CF	blue	na
chrUn	57519499	57520589	1091	SAT1_CF	blue	na
chrUn	57520632	57521171	540	SAT1_CF	blue	na
chrUn	57521250	57522038	789	SAT1_CF	blue	na
chrUn	57522082	57522629	548	SAT1_CF	blue	na
chrUn	57522640	57523039	400	SAT1_CF	blue	na
chrUn	57523082	57523583	502	SAT1_CF	blue	na
chrUn	57523610	57524200	591	SAT1_CF	blue	na
chrUn	57524212	57524321	110	SAT1_CF	blue	na
chrUn	57524341	57524850	510	SAT1_CF	blue	na
chrUn	57524887	57525400	514	SAT1_CF	blue	na
chrUn	57525487	57525988	502	SAT1_CF	blue	na
chrUn	57526001	57527548	1548	SAT1_CF	blue	na
chrUn	57527590	57529446	1857	SAT1_CF	blue	na
chrUn	57529486	57529987	502	SAT1_CF	blue	na
chrUn	57530000	57530684	685	SAT1_CF	blue	na
chrUn	57530707	57531372	666	SAT1_CF	blue	na
chrUn	57531415	57532045	631	SAT1_CF	blue	na
chrUn	57532072	57532626	555	SAT1_CF	blue	na
chrUn	57533707	57535057	1351	SAT1_CF	blue	na
chrUn	57535125	57535638	514	SAT1_CF	blue	na
chrUn	57535723	57536038	316	SAT1_CF	blue	na
chrUn	57536640	57536969	330	SAT1_CF	blue	na
chrUn	57537049	57537556	508	SAT1_CF	blue	na
chrUn	57537569	57537971	403	SAT1_CF	blue	na
chrUn	57538011	57538513	503	SAT1_CF	blue	na
chrUn	57538526	57539260	735	SAT1_CF	blue	na
chrUn	57540571	57540737	167	SAT1_CF	blue	na
chrUn	57540781	57541737	957	SAT1_CF	blue	na
chrUn	57541751	57542295	545	SAT1_CF	blue	na
chrUn	57545687	57545991	305	SAT1_CF	blue	na
chrUn	57546005	57546110	106	SAT1_CF	blue	na
chrUn	57546132	57546676	545	SAT1_CF	blue	na
chrUn	57548647	57550252	1606	SAT1_CF	blue	na
chrUn	57550280	57551391	1112	SAT1_CF	blue	na

chrUn	57557519	57557789	271	SAT1_CF	blue	na
chrUn	57557833	57558382	550	SAT1_CF	blue	na
chrUn	57558425	57558962	538	SAT1_CF	blue	na
chrUn	57560009	57560473	465	SAT1_CF	blue	na
chrUn	57560486	57560562	77	SAT1_CF	blue	na
chrUn	57560615	57561143	529	SAT1_CF	blue	na
chrUn	57561155	57561622	468	SAT1_CF	blue	na
chrUn	57568187	57568986	800	SAT1_CF	blue	na
chrUn	57569020	57569421	402	SAT1_CF	blue	na
chrUn	57569463	57569963	501	SAT1_CF	blue	na
chrUn	57569976	57570659	684	SAT1_CF	blue	na
chrUn	57570671	57571354	684	SAT1_CF	blue	na
chrUn	57571366	57571816	451	SAT1_CF	blue	na
chrUn	57573852	57574752	901	SAT1_CF	blue	na
chrUn	57574764	57576170	1407	SAT1_CF	blue	na
chrUn	57576397	57576478	82	SAT1_CF	blue	na
chrUn	57576517	57577792	1276	SAT1_CF	blue	na
chrUn	57577820	57578383	564	SAT1_CF	blue	na
chrUn	57579433	57580290	858	CarSat1	red	na
chrUn	57580320	57584709	4390	CarSat1	red	na
chrUn	57584724	57584851	128	Carsat2	orange	na
chrUn	57584739	57587654	2916	CarSat1	red	na
chrUn	57587669	57587795	127	Carsat2	orange	na
chrUn	57587684	57588395	712	CarSat1	red	na
chrUn	57588425	57588909	485	CarSat1	red	na
chrUn	57588942	57589867	926	CarSat1	red	na
chrUn	57589882	57590008	127	Carsat2	orange	na
chrUn	57589897	57590604	708	CarSat1	red	na
chrUn	57590619	57590745	127	Carsat2	orange	na
chrUn	57590634	57591339	706	CarSat1	red	na
chrUn	57591354	57591479	126	Carsat2	orange	na
chrUn	57591369	57596491	5123	CarSat1	red	na
chrUn	57596521	57597971	1451	CarSat1	red	na
chrUn	57598003	57598707	705	CarSat1	red	na
chrUn	57598737	57599445	709	CarSat1	red	na
chrUn	57599460	57599586	127	Carsat2	orange	na
chrUn	57599475	57601120	1646	CarSat1	red	na
chrUn	57632096	57632339	244	Sat7CF	lime	na
chrUn	57632135	57632488	354	Sat7CF	lime	na
chrUn	57647427	57648622	1196	CarSat1	red	na
chrUn	57648649	57650094	1446	CarSat1	red	na
chrUn	57650121	57650830	710	CarSat1	red	na

chrUn	57650857	57652312	1456	CarSat1	red	na
chrUn	57652328	57653049	722	CarSat1	red	na
chrUn	57653065	57653534	470	CarSat1	red	na
chrUn	57653561	57655252	1692	CarSat1	red	na
chrUn	57655277	57656231	955	CarSat1	red	na
chrUn	57656653	57657235	583	CarSat1	red	na
chrUn	57657262	57658041	780	CarSat1	red	na
chrUn	57659007	57659685	679	CarSat1	red	na
chrUn	57659710	57660179	470	CarSat1	red	na
chrUn	57660206	57663593	3388	CarSat1	red	na
chrUn	57663623	57664602	980	CarSat1	red	na
chrUn	57664629	57665338	710	CarSat1	red	na
chrUn	57665365	57666782	1418	CarSat1	red	na
chrUn	57666798	57669208	2411	CarSat1	red	na
chrUn	57669238	57669945	708	CarSat1	red	na
chrUn	57669975	57670575	601	CarSat1	red	na
chrUn	57671613	57672188	576	CarSat1	red	na
chrUn	57672218	57672702	485	CarSat1	red	na
chrUn	57672726	57673661	936	CarSat1	red	na
chrUn	57673691	57675131	1441	CarSat1	red	na
chrUn	57675161	57675873	713	CarSat1	red	na
chrUn	57675903	57676389	487	CarSat1	red	na
chrUn	57676413	57677775	1363	CarSat1	red	na
chrUn	57677859	57680711	2853	CarSat1	red	na
chrUn	57680728	57681448	721	CarSat1	red	na
chrUn	57681473	57682567	1095	CarSat1	red	na
chrUn	57682592	57683768	1177	CarSat1	red	na
chrUn	57683797	57684286	490	CarSat1	red	na
chrUn	57684303	57686605	2303	CarSat1	red	na
chrUn	57686635	57688597	1963	CarSat1	red	na
chrUn	57688614	57689330	717	CarSat1	red	na
chrUn	57689346	57690067	722	CarSat1	red	na
chrUn	57690092	57691432	1341	CarSat1	red	na
chrUn	57691449	57692169	721	CarSat1	red	na
chrUn	57692194	57693150	957	CarSat1	red	na
chrUn	57694163	57694712	550	CarSat1	red	na
chrUn	57694742	57696702	1961	CarSat1	red	na
chrUn	57696726	57697661	936	CarSat1	red	na
chrUn	57697691	57698399	709	CarSat1	red	na
chrUn	57698429	57699874	1446	CarSat1	red	na
chrUn	57699904	57700529	626	CarSat1	red	na
chrUn	57702447	57704926	2480	CarSat1	red	na

chrUn	57704956	57705442	487	CarSat1	red	na
chrUn	57705475	57707138	1664	CarSat1	red	na
chrUn	57707168	57707875	708	CarSat1	red	na
chrUn	57707905	57708613	709	CarSat1	red	na
chrUn	57708643	57709351	709	CarSat1	red	na
chrUn	57709366	57709492	127	Carsat2	orange	na
chrUn	57709381	57710092	712	CarSat1	red	na
chrUn	57710122	57711566	1445	CarSat1	red	na
chrUn	57711596	57712817	1222	CarSat1	red	na
chrUn	57712841	57713559	719	CarSat1	red	na
chrUn	57713592	57715996	2405	CarSat1	red	na
chrUn	57716026	57716733	708	CarSat1	red	na
chrUn	57716763	57717506	744	CarSat1	red	na
chrUn	57718507	57719182	676	CarSat1	red	na
chrUn	57719206	57730451	11246	CarSat1	red	na
chrUn	57730466	57730592	127	Carsat2	orange	na
chrUn	57730481	57730966	486	CarSat1	red	na
chrUn	57730990	57733181	2192	CarSat1	red	na
chrUn	57733205	57735145	1941	CarSat1	red	na
chrUn	57735172	57736131	960	CarSat1	red	na
chrUn	57736147	57737083	937	CarSat1	red	na
chrUn	57737113	57739299	2187	CarSat1	red	na
chrUn	57739314	57739440	127	Carsat2	orange	na
chrUn	57739329	57739958	630	CarSat1	red	na
chrUn	57770225	57771145	921	CarSat1	red	na
chrUn	57771160	57771866	707	CarSat1	red	na
chrUn	57771896	57772618	723	CarSat1	red	na
chrUn	57772633	57774816	2184	CarSat1	red	na
chrUn	57774846	57776706	1861	CarSat1	red	na
chrUn	57776736	57779660	2925	CarSat1	red	na
chrUn	57779690	57781357	1668	CarSat1	red	na
chrUn	57781390	57784090	2701	CarSat1	red	na
chrUn	57784120	57784874	755	CarSat1	red	na
chrUn	57785673	57787049	1377	CarSat1	red	na
chrUn	57787070	57789984	2915	CarSat1	red	na
chrUn	57790021	57792384	2364	CarSat1	red	na
chrUn	57793388	57795221	1834	SAT1_CF	blue	na
chrUn	57795235	57795637	403	SAT1_CF	blue	na
chrUn	57795677	57796178	502	SAT1_CF	blue	na
chrUn	57796205	57796949	745	SAT1_CF	blue	na
chrUn	57796963	57797575	613	SAT1_CF	blue	na
chrUn	57797587	57798439	853	SAT1_CF	blue	na

chrUn	57798490	57799566	1077	SAT1_CF	blue	na
chrUn	57799609	57800120	512	SAT1_CF	blue	na
chrUn	57800147	57800289	143	SAT1_CF	blue	na
chrUn	57800344	57800850	507	SAT1_CF	blue	na
chrUn	57800872	57801530	659	SAT1_CF	blue	na
chrUn	57801573	57801735	163	SAT1_CF	blue	na
chrUn	57802840	57803068	229	SAT1_CF	blue	na
chrUn	57803155	57803656	502	SAT1_CF	blue	na
chrUn	57803679	57804351	673	SAT1_CF	blue	na
chrUn	57804363	57805036	674	SAT1_CF	blue	na
chrUn	57805085	57805585	501	SAT1_CF	blue	na
chrUn	57805596	57805689	94	SAT1_CF	blue	na
chrUn	57805719	57806970	1252	SAT1_CF	blue	na
chrUn	57806982	57807585	604	SAT1_CF	blue	na
chrUn	57807604	57807700	97	SAT1_CF	blue	na
chrUn	57807724	57808277	554	SAT1_CF	blue	na
chrUn	57808289	57809154	866	SAT1_CF	blue	na
chrUn	57809196	57811354	2159	SAT1_CF	blue	na
chrUn	57811366	57812021	656	SAT1_CF	blue	na
chrUn	57812049	57812178	130	SAT1_CF	blue	na
chrUn	57812250	57812751	502	SAT1_CF	blue	na
chrUn	57812764	57815250	2487	SAT1_CF	blue	na
chrUn	57815329	57815494	166	SAT1_CF	blue	na
chrUn	57815675	57815860	186	SAT1_CF	blue	na
chrUn	57835402	57836045	644	Sat2CF	purple	na
chrUn	57843517	57844197	681	CarSat1	red	na
chrUn	57844192	57844847	656	SAT1_CF	blue	na
chrUn	57846061	57846626	566	SAT1_CF	blue	na
chrUn	57846654	57847165	512	SAT1_CF	blue	na
chrUn	57847220	57847847	628	SAT1_CF	blue	na
chrUn	57847860	57848402	543	SAT1_CF	blue	na
chrUn	57918401	57918647	247	Sat19CF	na	na
chrUn	57950045	57950601	557	SAT1_CF	blue	na
chrUn	57950644	57951322	679	SAT1_CF	blue	na
chrUn	57951334	57951748	415	SAT1_CF	blue	na
chrUn	57951722	57954423	2702	CarSat1	red	na
chrUn	57954453	57955307	855	CarSat1	red	na
chrUn	57955329	57956909	1581	CarSat1	red	na
chrUn	57956939	57957638	700	CarSat1	red	na
chrUn	58042992	58043481	490	SAT1_CF	blue	na
chrUn	58043503	58043609	107	SAT1_CF	blue	na
chrUn	58043622	58044123	502	SAT1_CF	blue	na

chrUn	58044208	58046048	1841	SAT1_CF	blue	na
chrUn	58046069	58046169	101	SAT1_CF	blue	na
chrUn	58046187	58046684	498	SAT1_CF	blue	na
chrUn	58046697	58049476	2780	SAT1_CF	blue	na
chrUn	58049488	58050167	680	SAT1_CF	blue	na
chrUn	58050184	58054553	4370	SAT1_CF	blue	na
chrUn	58054595	58055460	866	SAT1_CF	blue	na
chrUn	58055472	58056027	556	SAT1_CF	blue	na
chrUn	58056048	58056154	107	SAT1_CF	blue	na
chrUn	58056167	58056849	683	SAT1_CF	blue	na
chrUn	58056869	58057363	495	SAT1_CF	blue	na
chrUn	58057399	58057735	337	SAT1_CF	blue	na
chrUn	58057746	58058284	539	SAT1_CF	blue	na
chrUn	58058364	58059324	961	SAT1_CF	blue	na
chrUn	58059369	58060762	1394	SAT1_CF	blue	na
chrUn	58060774	58061457	684	SAT1_CF	blue	na
chrUn	58061469	58062229	761	SAT1_CF	blue	na
chrUn	58062242	58064051	1810	SAT1_CF	blue	na
chrUn	58068434	58069520	1087	Sat2CF	purple	na
chrUn	58069391	58069592	202	Sat2CF	purple	na
chrUn	58071211	58071620	410	CarSat1	red	na
chrUn	58072426	58072568	143	CarSat1	red	na
chrUn	58072618	58073442	825	CarSat1	red	na
chrUn	58073463	58075044	1582	CarSat1	red	na
chrUn	58075065	58075907	843	CarSat1	red	na
chrUn	58075928	58077362	1435	CarSat1	red	na
chrUn	58077521	58078376	856	CarSat1	red	na
chrUn	58078395	58079343	949	CarSat1	red	na
chrUn	58115126	58115350	225	Sat43CF	na	na
chrUn	58132149	58132691	543	SAT1_CF	blue	na
chrUn	58132735	58133395	661	SAT1_CF	blue	na
chrUn	58133435	58133923	489	SAT1_CF	blue	na
chrUn	58133951	58134087	137	SAT1_CF	blue	na
chrUn	58134153	58134653	501	SAT1_CF	blue	na
chrUn	58134668	58135350	683	SAT1_CF	blue	na
chrUn	58135362	58136036	675	SAT1_CF	blue	na
chrUn	58136080	58136818	739	SAT1_CF	blue	na
chrUn	58137073	58138677	1605	SAT1_CF	blue	na
chrUn	58138719	58138884	166	SAT1_CF	blue	na
chrUn	58139066	58139251	186	SAT1_CF	blue	na
chrUn	58139268	58139788	521	SAT1_CF	blue	na
chrUn	58139801	58139909	109	SAT1_CF	blue	na

chrUn	58139929	58141172	1244	SAT1_CF	blue	na
chrUn	58141216	58142098	883	SAT1_CF	blue	na
chrUn	58142112	58142657	546	SAT1_CF	blue	na
chrUn	58142680	58142839	160	SAT1_CF	blue	na
chrUn	58142852	58143236	385	SAT1_CF	blue	na
chrUn	58143274	58143787	514	SAT1_CF	blue	na
chrUn	58143807	58145619	1813	SAT1_CF	blue	na
chrUn	58145632	58146034	403	SAT1_CF	blue	na
chrUn	58146074	58146576	503	SAT1_CF	blue	na
chrUn	58146589	58148468	1880	SAT1_CF	blue	na
chrUn	58148585	58149247	663	SAT1_CF	blue	na
chrUn	58149262	58149526	265	SAT1_CF	blue	na
chrUn	58149562	58150071	510	SAT1_CF	blue	na
chrUn	58150084	58150155	72	SAT1_CF	blue	na
chrUn	58150178	58150729	552	SAT1_CF	blue	na
chrUn	58150772	58151313	542	SAT1_CF	blue	na
chrUn	58151359	58151918	560	SAT1_CF	blue	na
chrUn	58152005	58152505	501	SAT1_CF	blue	na
chrUn	58152523	58153276	754	SAT1_CF	blue	na
chrUn	58208290	58211204	2915	CarSat1	red	na
chrUn	58211219	58211346	128	Carsat2	orange	na
chrUn	58211234	58212922	1689	CarSat1	red	na
chrUn	58212961	58213685	725	CarSat1	red	na
chrUn	58213706	58214150	445	CarSat1	red	na
chrUn	58214165	58214280	116	Carsat2	orange	na
chrUn	58214180	58214885	706	CarSat1	red	na
chrUn	58214900	58215026	127	Carsat2	orange	na
chrUn	58214915	58215625	711	CarSat1	red	na
chrUn	58215640	58215767	128	Carsat2	orange	na
chrUn	58215655	58217835	2181	CarSat1	red	na
chrUn	58217865	58218947	1083	CarSat1	red	na
chrUn	58219050	58219856	807	CarSat1	red	na
chrUn	58219886	58219944	59	CarSat1	red	na
chrUn	58220135	58220802	668	CarSat1	red	na
chrUn	58220817	58220944	128	Carsat2	orange	na
chrUn	58220832	58222281	1450	CarSat1	red	na
chrUn	58222296	58222421	126	Carsat2	orange	na
chrUn	58222311	58223017	707	CarSat1	red	na
chrUn	58223032	58223159	128	Carsat2	orange	na
chrUn	58223047	58225226	2180	CarSat1	red	na
chrUn	58225256	58226699	1444	CarSat1	red	na
chrUn	58226714	58226841	128	Carsat2	orange	na

chrUn	58226729	58227435	707	CarSat1	red	na
chrUn	58227450	58227576	127	Carsat2	orange	na
chrUn	58227465	58228176	712	CarSat1	red	na
chrUn	58228191	58228318	128	Carsat2	orange	na
chrUn	58228206	58229191	986	CarSat1	red	na
chrUn	58301110	58302898	1789	CarSat1	red	na
chrUn	58304427	58306424	1998	CarSat1	red	na
chrUn	58306445	58308609	2165	CarSat1	red	na
chrUn	58308789	58310778	1990	CarSat1	red	na
chrUn	58311489	58312939	1451	CarSat1	red	na
chrUn	58312960	58313389	430	CarSat1	red	na
chrUn	58313430	58314258	829	CarSat1	red	na
chrUn	58314447	58315078	632	CarSat1	red	na
chrUn	58315111	58315802	692	CarSat1	red	na
chrUn	58315831	58316543	713	CarSat1	red	na
chrUn	58316576	58317276	701	CarSat1	red	na
chrUn	58317301	58318569	1269	CarSat1	red	na
chrUn	58325166	58325744	579	Sat2CF	purple	na
chrUn	58325231	58325752	522	Sat2CF	purple	na
chrUn	58417324	58417993	670	Sat7CF	lime	na
chrUn	58441583	58442532	950	CarSat1	red	na
chrUn	58442683	58444129	1447	CarSat1	red	na
chrUn	58444289	58444932	644	CarSat1	red	na
chrUn	58444996	58445902	907	CarSat1	red	na
chrUn	58445923	58446158	236	CarSat1	red	na
chrUn	58446346	58447171	826	CarSat1	red	na
chrUn	58447208	58447909	702	CarSat1	red	na
chrUn	58447940	58451072	3133	CarSat1	red	na
chrUn	58451097	58453925	2829	CarSat1	red	na
chrUn	58453961	58455130	1170	CarSat1	red	na
chrUn	58455157	58456459	1303	CarSat1	red	na
chrUn	58457501	58465144	7644	CarSat1	red	na
chrUn	58465338	58466700	1363	CarSat1	red	na
chrUn	58466730	58467242	513	CarSat1	red	na
chrUn	58467598	58469433	1836	CarSat1	red	na
chrUn	58469449	58470169	721	CarSat1	red	na
chrUn	58470185	58471118	934	CarSat1	red	na
chrUn	58471133	58471260	128	Carsat2	orange	na
chrUn	58471148	58472378	1231	CarSat1	red	na
chrUn	58472394	58473850	1457	CarSat1	red	na
chrUn	58473875	58474587	713	CarSat1	red	na
chrUn	58474603	58475324	722	CarSat1	red	na

chrUn	58475349	58476546	1198	CarSat1	red	na
chrUn	58476573	58477535	963	CarSat1	red	na
chrUn	58477560	58478600	1041	CarSat1	red	na
chrUn	58482605	58483997	1393	CarSat1	red	na
chrUn	58484026	58484758	733	CarSat1	red	na
chrUn	58484776	58485831	1056	CarSat1	red	na
chrUn	58485872	58487233	1362	SAT1_CF	blue	na
chrUn	58487471	58488368	898	Sat2CF	purple	na
chrUn	58488234	58488396	163	Sat2CF	purple	na
chrUn	58499475	58499710	236	Sat2CF	purple	na
chrUn	58499516	58500125	610	Sat2CF	purple	na
chrUn	58499804	58500194	391	Sat2CF	purple	na
chrUn	58501216	58502915	1700	CarSat1	red	na
chrUn	58502942	58503652	711	CarSat1	red	na
chrUn	58503679	58504651	973	CarSat1	red	na
chrUn	58504665	58505126	462	CarSat1	red	na
chrUn	58505153	58505864	712	CarSat1	red	na
chrUn	58505891	58506885	995	CarSat1	red	na
chrUn	58506900	58507622	723	CarSat1	red	na
chrUn	58507644	58510286	2643	CarSat1	red	na
chrUn	58510313	58511024	712	CarSat1	red	na
chrUn	58511051	58511761	711	CarSat1	red	na
chrUn	58511788	58513235	1448	CarSat1	red	na
chrUn	58513262	58513972	711	CarSat1	red	na
chrUn	58513999	58515448	1450	CarSat1	red	na
chrUn	58515475	58516672	1198	CarSat1	red	na
chrUn	58516697	58517660	964	CarSat1	red	na
chrUn	58517687	58520141	2455	CarSat1	red	na
chrUn	58520169	58521704	1536	CarSat1	red	na
chrUn	58577157	58578781	1625	CarSat1	red	na
chrUn	58578802	58580387	1586	CarSat1	red	na
chrUn	58580418	58582013	1596	CarSat1	red	na
chrUn	58582034	58582343	310	CarSat1	red	na
chrUn	58582358	58582916	559	CarSat1	red	na
chrUn	58582979	58583767	789	CarSat1	red	na
chrUn	58583784	58585231	1448	CarSat1	red	na
chrUn	58585391	58586347	957	CarSat1	red	na
chrUn	58608504	58608754	251	SAT1_CF	blue	na
chrUn	58608745	58610718	1974	CarSat1	red	na
chrUn	58610745	58612332	1588	CarSat1	red	na
chrUn	58612353	58613935	1583	CarSat1	red	na
chrUn	58613956	58614872	917	CarSat1	red	na

chrUn	58620085	58621610	1526	CarSat1	red	na
chrUn	58621625	58621751	127	Carsat2	orange	na
chrUn	58621640	58622346	707	CarSat1	red	na
chrUn	58622376	58623087	712	CarSat1	red	na
chrUn	58623103	58623229	127	Carsat2	orange	na
chrUn	58623117	58623824	708	CarSat1	red	na
chrUn	58623854	58624339	486	CarSat1	red	na
chrUn	58624372	58625302	931	CarSat1	red	na
chrUn	58625317	58625443	127	Carsat2	orange	na
chrUn	58625332	58626043	712	CarSat1	red	na
chrUn	58626058	58626185	128	Carsat2	orange	na
chrUn	58626073	58626780	708	CarSat1	red	na
chrUn	58626795	58626922	128	Carsat2	orange	na
chrUn	58626810	58627604	795	CarSat1	red	na
chrUn	58627634	58627760	127	Carsat2	orange	na
chrUn	58627649	58628355	707	CarSat1	red	na
chrUn	58628370	58628497	128	Carsat2	orange	na
chrUn	58628385	58629092	708	CarSat1	red	na
chrUn	58629122	58630100	979	CarSat1	red	na
chrUn	58630121	58630565	445	CarSat1	red	na
chrUn	58630580	58630707	128	Carsat2	orange	na
chrUn	58630595	58631302	708	CarSat1	red	na
chrUn	58631317	58631443	127	Carsat2	orange	na
chrUn	58631332	58632038	707	CarSat1	red	na
chrUn	58632053	58632180	128	Carsat2	orange	na
chrUn	58632068	58632775	708	CarSat1	red	na
chrUn	58632790	58632916	127	Carsat2	orange	na
chrUn	58632805	58633512	708	CarSat1	red	na
chrUn	58633527	58633654	128	Carsat2	orange	na
chrUn	58633542	58634249	708	CarSat1	red	na
chrUn	58634278	58634982	705	CarSat1	red	na
chrUn	58635012	58635718	707	CarSat1	red	na
chrUn	58635748	58636450	703	CarSat1	red	na
chrUn	58636465	58636588	124	Carsat2	orange	na
chrUn	58636480	58637925	1446	CarSat1	red	na
chrUn	58637940	58638067	128	Carsat2	orange	na
chrUn	58637955	58638662	708	CarSat1	red	na
chrUn	58638677	58638803	127	Carsat2	orange	na
chrUn	58638692	58640508	1817	CarSat1	red	na
chrUn	58658729	58659466	738	Sat2CF	purple	na
chrUn	58658731	58659392	662	Sat2CF	purple	na
chrUn	58668681	58670283	1603	SAT1_CF	blue	na

chrUn	58670295	58671717	1423	SAT1_CF	blue	na
chrUn	58671750	58672266	517	SAT1_CF	blue	na
chrUn	58673673	58674252	580	SAT1_CF	blue	na
chrUn	58674266	58675218	953	SAT1_CF	blue	na
chrUn	58675240	58675347	108	SAT1_CF	blue	na
chrUn	58675359	58676040	682	SAT1_CF	blue	na
chrUn	58676054	58676572	519	SAT1_CF	blue	na
chrUn	58676610	58677502	893	SAT1_CF	blue	na
chrUn	58677583	58678107	525	SAT1_CF	blue	na
chrUn	58678129	58678237	109	SAT1_CF	blue	na
chrUn	58678249	58679481	1233	SAT1_CF	blue	na
chrUn	58679499	58680023	525	SAT1_CF	blue	na
chrUn	58680045	58680153	109	SAT1_CF	blue	na
chrUn	58680165	58681760	1596	SAT1_CF	blue	na
chrUn	58681772	58682479	708	SAT1_CF	blue	na
chrUn	58682547	58684258	1712	SAT1_CF	blue	na
chrUn	58684272	58684792	521	SAT1_CF	blue	na
chrUn	58684849	58685970	1122	SAT1_CF	blue	na
chrUn	58685984	58686966	983	SAT1_CF	blue	na
chrUn	58686994	58688554	1561	SAT1_CF	blue	na
chrUn	58688567	58689057	491	SAT1_CF	blue	na
chrUn	58703914	58705081	1168	Sat10CF	na	na
chrUn	58704546	58705765	1220	Sat10CF	na	na
chrUn	58704773	58705945	1173	Sat10CF	na	na
chrUn	58705416	58706605	1190	Sat10CF	na	na
chrUn	58705583	58706785	1203	Sat10CF	na	na
chrUn	58705943	58707135	1193	Sat10CF	na	na
chrUn	58706063	58707258	1196	Sat10CF	na	na
chrUn	58732812	58735499	2688	CarSat1	red	na
chrUn	58735523	58736473	951	CarSat1	red	na
chrUn	58736495	58736620	126	Carsat2	orange	na
chrUn	58736510	58737221	712	CarSat1	red	na
chrUn	58737251	58737958	708	CarSat1	red	na
chrUn	58737988	58738694	707	CarSat1	red	na
chrUn	58738712	58738837	126	Carsat2	orange	na
chrUn	58738726	58740169	1444	CarSat1	red	na
chrUn	58740199	58741110	912	CarSat1	red	na
chrUn	58741874	58743421	1548	CarSat1	red	na
chrUn	58743436	58743563	128	Carsat2	orange	na
chrUn	58743451	58743937	487	CarSat1	red	na
chrUn	58743958	58747381	3424	CarSat1	red	na
chrUn	58747410	58748360	951	CarSat1	red	na

chrUn	58748387	58749098	712	CarSat1	red	na
chrUn	58749124	58751386	2263	CarSat1	red	na
chrUn	58751573	58752464	892	CarSat1	red	na
chrUn	58752496	58753199	704	CarSat1	red	na
chrUn	58753228	58753823	596	CarSat1	red	na
chrUn	58754837	58756162	1326	CarSat1	red	na
chrUn	58756183	58759834	3652	CarSat1	red	na
chrUn	58759855	58760833	979	CarSat1	red	na
chrUn	58760863	58765709	4847	CarSat1	red	na
chrUn	58765730	58767442	1713	CarSat1	red	na
chrUn	58767472	58770089	2618	CarSat1	red	na
chrUn	58770110	58771825	1716	CarSat1	red	na
chrUn	58771859	58773766	1908	CarSat1	red	na
chrUn	58773787	58775038	1252	CarSat1	red	na
chrUn	58840508	58841459	952	CarSat1	red	na
chrUn	58841608	58842330	723	CarSat1	red	na
chrUn	58842479	58843107	629	CarSat1	red	na
chrUn	58843362	58843697	336	CarSat1	red	na
chrUn	58843880	58844228	349	CarSat1	red	na
chrUn	58844441	58845090	650	CarSat1	red	na
chrUn	58845359	58846049	691	CarSat1	red	na
chrUn	58846209	58846855	647	CarSat1	red	na
chrUn	58892364	58893036	673	Sat2CF	purple	na
chrUn	58892435	58893285	851	Sat2CF	purple	na
chrUn	58893149	58893311	163	Sat2CF	purple	na
chrUn	58909330	58909710	381	CarSat1	red	na
chrUn	58911348	58912303	956	Sat2CF	purple	na
chrUn	58916159	58916738	580	SAT1_CF	blue	na
chrUn	58916776	58917276	501	SAT1_CF	blue	na
chrUn	58917289	58917965	677	SAT1_CF	blue	na
chrUn	58918008	58918974	967	SAT1_CF	blue	na
chrUn	58919054	58919964	911	SAT1_CF	blue	na
chrUn	58920051	58920639	589	SAT1_CF	blue	na
chrUn	58920660	58921330	671	SAT1_CF	blue	na
chrUn	58921344	58922873	1530	SAT1_CF	blue	na
chrUn	58922954	58923119	166	SAT1_CF	blue	na
chrUn	58923301	58923486	186	SAT1_CF	blue	na
chrUn	58923522	58924022	501	SAT1_CF	blue	na
chrUn	58924035	58924783	749	SAT1_CF	blue	na
chrUn	58924800	58925659	860	SAT1_CF	blue	na
chrUn	58925712	58926212	501	SAT1_CF	blue	na
chrUn	58926225	58926332	108	SAT1_CF	blue	na

chrUn	58926354	58928254	1901	SAT1_CF	blue	na
chrUn	58928298	58929630	1333	SAT1_CF	blue	na
chrUn	58929717	58930218	502	SAT1_CF	blue	na
chrUn	58930230	58930339	110	SAT1_CF	blue	na
chrUn	58930360	58931420	1061	SAT1_CF	blue	na
chrUn	58931433	58932594	1162	SAT1_CF	blue	na
chrUn	58932636	58935554	2919	SAT1_CF	blue	na
chrUn	58935590	58936131	542	SAT1_CF	blue	na
chrUn	58958153	58961135	2983	CarSat1	red	na
chrUn	58961161	58962357	1197	CarSat1	red	na
chrUn	58962384	58963094	711	CarSat1	red	na
chrUn	58963121	58964084	964	CarSat1	red	na
chrUn	58964100	58964822	723	CarSat1	red	na
chrUn	58964847	58965558	712	CarSat1	red	na
chrUn	58965584	58966294	711	CarSat1	red	na
chrUn	58966310	58967517	1208	CarSat1	red	na
chrUn	58967544	58968497	954	CarSat1	red	na
chrUn	58968521	58969244	724	CarSat1	red	na
chrUn	58969260	58969729	470	CarSat1	red	na
chrUn	58969756	58970462	707	CarSat1	red	na
chrUn	58970489	58973876	3388	CarSat1	red	na
chrUn	58973906	58976606	2701	CarSat1	red	na
chrUn	58976622	58977341	720	CarSat1	red	na
chrUn	58977357	58978118	762	CarSat1	red	na
chrUn	58979121	58980371	1251	CarSat1	red	na
chrUn	58980399	58980859	461	CarSat1	red	na
chrUn	58980884	58982583	1700	CarSat1	red	na
chrUn	58982610	58983666	1057	CarSat1	red	na
chrUn	58983843	58984994	1152	CarSat1	red	na
chrUn	58985021	58985732	712	CarSat1	red	na
chrUn	58985759	58987206	1448	CarSat1	red	na
chrUn	58987233	58987943	711	CarSat1	red	na
chrUn	58987970	58990155	2186	CarSat1	red	na
chrUn	58990182	58991630	1449	CarSat1	red	na
chrUn	58991647	58992257	611	CarSat1	red	na
chrUn	58992456	58992555	100	CarSat1	red	na
chrUn	58992569	58993155	587	CarSat1	red	na
chrUn	59000703	59001871	1169	Sat2CF	purple	na
chrUn	59001693	59001902	210	Sat2CF	purple	na
chrUn	59016604	59017391	788	Sat2CF	purple	na
chrUn	59024650	59025523	874	CarSat1	red	na
chrUn	59025629	59026478	850	CarSat1	red	na

chrUn	59026512	59026973	462	CarSat1	red	na
chrUn	59027154	59027489	336	CarSat1	red	na
chrUn	59027730	59028335	606	CarSat1	red	na
chrUn	59030543	59031258	716	CarSat1	red	na
chrUn	59031408	59031801	394	CarSat1	red	na
chrUn	59031841	59032517	677	CarSat1	red	na
chrUn	59032582	59034434	1853	SAT1_CF	blue	na
chrUn	59034477	59034652	176	SAT1_CF	blue	na
chrUn	59034680	59035224	545	SAT1_CF	blue	na
chrUn	59035304	59035816	513	SAT1_CF	blue	na
chrUn	59035844	59036640	797	SAT1_CF	blue	na
chrUn	59036654	59038172	1519	SAT1_CF	blue	na
chrUn	59038187	59039888	1702	SAT1_CF	blue	na
chrUn	59039878	59040592	715	CarSat1	red	na
chrUn	59040624	59040985	362	CarSat1	red	na
chrUn	59041986	59044094	2109	CarSat1	red	na
chrUn	59045029	59048592	3564	CarSat1	red	na
chrUn	59048626	59049063	438	CarSat1	red	na
chrUn	59049084	59049634	551	CarSat1	red	na
chrUn	59049872	59051556	1685	CarSat1	red	na
chrUn	59053462	59056859	3398	CarSat1	red	na
chrUn	59056875	59058410	1536	CarSat1	red	na
chrUn	59058577	59059011	435	CarSat1	red	na
chrUn	59059036	59059755	720	CarSat1	red	na
chrUn	59059771	59060493	723	CarSat1	red	na
chrUn	59060509	59061372	864	CarSat1	red	na
chrUn	59063198	59064425	1228	CarSat1	red	na
chrUn	59065265	59066424	1160	CarSat1	red	na
chrUn	59074489	59075672	1184	CarSat1	red	na
chrUn	59076678	59077043	366	SAT1_CF	blue	na
chrUn	59077055	59078388	1334	SAT1_CF	blue	na
chrUn	59078432	59078996	565	SAT1_CF	blue	na
chrUn	59079076	59079601	526	SAT1_CF	blue	na
chrUn	59079616	59079773	158	SAT1_CF	blue	na
chrUn	59079818	59080320	503	SAT1_CF	blue	na
chrUn	59080342	59081016	675	SAT1_CF	blue	na
chrUn	59081028	59081700	673	SAT1_CF	blue	na
chrUn	59081747	59082121	375	SAT1_CF	blue	na
chrUn	59082177	59083776	1600	SAT1_CF	blue	na
chrUn	59083855	59084118	264	SAT1_CF	blue	na
chrUn	59084148	59084672	525	SAT1_CF	blue	na
chrUn	59084742	59084840	99	SAT1_CF	blue	na

chrUn	59084887	59085389	503	SAT1_CF	blue	na
chrUn	59085402	59086162	761	SAT1_CF	blue	na
chrUn	59086174	59086848	675	SAT1_CF	blue	na
chrUn	59086891	59088050	1160	SAT1_CF	blue	na
chrUn	59089036	59089565	530	SAT1_CF	blue	na
chrUn	59089681	59090641	961	SAT1_CF	blue	na
chrUn	59090701	59091182	482	SAT1_CF	blue	na
chrUn	59091194	59091879	686	SAT1_CF	blue	na
chrUn	59091901	59092567	667	SAT1_CF	blue	na
chrUn	59092612	59093832	1221	SAT1_CF	blue	na
chrUn	59093912	59094436	525	SAT1_CF	blue	na
chrUn	59094451	59094608	158	SAT1_CF	blue	na
chrUn	59094634	59095809	1176	SAT1_CF	blue	na
chrUn	59095822	59097217	1396	SAT1_CF	blue	na
chrUn	59097232	59097538	307	SAT1_CF	blue	na
chrUn	59098570	59099132	563	CarSat1	red	na
chrUn	59099162	59099604	443	CarSat1	red	na
chrUn	59099626	59100603	978	CarSat1	red	na
chrUn	59100633	59101359	727	CarSat1	red	na
chrUn	59101374	59102833	1460	CarSat1	red	na
chrUn	59102847	59103555	709	CarSat1	red	na
chrUn	59103585	59104307	723	CarSat1	red	na
chrUn	59104322	59105783	1462	CarSat1	red	na
chrUn	59105798	59106520	723	CarSat1	red	na
chrUn	59106535	59108732	2198	CarSat1	red	na
chrUn	59108747	59110660	1914	CarSat1	red	na
chrUn	59110687	59112864	2178	CarSat1	red	na
chrUn	59112891	59116547	3657	CarSat1	red	na
chrUn	59116568	59117544	977	CarSat1	red	na
chrUn	59117574	59118496	923	CarSat1	red	na
chrUn	59146953	59148306	1354	CarSat1	red	na
chrUn	59148327	59148795	469	CarSat1	red	na
chrUn	59148820	59151500	2681	CarSat1	red	na
chrUn	59151522	59156122	4601	CarSat1	red	na
chrUn	59156147	59156861	715	CarSat1	red	na
chrUn	59156888	59157353	466	CarSat1	red	na
chrUn	59157369	59158086	718	CarSat1	red	na
chrUn	59158102	59160281	2180	CarSat1	red	na
chrUn	59160306	59161014	709	CarSat1	red	na
chrUn	59161039	59162001	963	CarSat1	red	na
chrUn	59162028	59162739	712	CarSat1	red	na
chrUn	59162766	59164209	1444	CarSat1	red	na

chrUn	59164237	59166622	2386	CarSat1	red	na
chrUn	59218285	59220363	2079	SAT1_CF	blue	na
chrUn	59220392	59221851	1460	SAT1_CF	blue	na
chrUn	59221873	59222351	479	SAT1_CF	blue	na
chrUn	59222417	59222813	397	SAT1_CF	blue	na
chrUn	59222826	59224801	1976	SAT1_CF	blue	na
chrUn	59224918	59226800	1883	SAT1_CF	blue	na
chrUn	59226813	59227295	483	SAT1_CF	blue	na
chrUn	59227355	59227756	402	SAT1_CF	blue	na
chrUn	59227769	59228276	508	SAT1_CF	blue	na
chrUn	59229037	59229147	111	SAT1_CF	blue	na
chrUn	59229169	59230473	1305	SAT1_CF	blue	na
chrUn	59230516	59231182	667	SAT1_CF	blue	na
chrUn	59231204	59232496	1293	SAT1_CF	blue	na
chrUn	59232508	59233061	554	SAT1_CF	blue	na
chrUn	59233082	59233191	110	SAT1_CF	blue	na
chrUn	59233203	59233705	503	SAT1_CF	blue	na
chrUn	59233771	59233907	137	SAT1_CF	blue	na
chrUn	59233935	59235114	1180	SAT1_CF	blue	na
chrUn	59235136	59235243	108	SAT1_CF	blue	na
chrUn	59235256	59235775	520	SAT1_CF	blue	na
chrUn	59235796	59236188	393	SAT1_CF	blue	na
chrUn	59236199	59236745	547	SAT1_CF	blue	na
chrUn	59236791	59237433	643	SAT1_CF	blue	na
chrUn	59237446	59237946	501	SAT1_CF	blue	na
chrUn	59238013	59238146	134	SAT1_CF	blue	na
chrUn	59238363	59238849	487	SAT1_CF	blue	na
chrUn	59239851	59240402	552	CarSat1	red	na
chrUn	59240429	59241139	711	CarSat1	red	na
chrUn	59241166	59242127	962	CarSat1	red	na
chrUn	59242143	59242611	469	CarSat1	red	na
chrUn	59242638	59243351	714	CarSat1	red	na
chrUn	59243376	59244086	711	CarSat1	red	na
chrUn	59244113	59245072	960	CarSat1	red	na
chrUn	59245088	59246289	1202	CarSat1	red	na
chrUn	59246316	59247026	711	CarSat1	red	na
chrUn	59247053	59248010	958	CarSat1	red	na
chrUn	59248026	59249233	1208	CarSat1	red	na
chrUn	59249258	59249966	709	CarSat1	red	na
chrUn	59249993	59250955	963	CarSat1	red	na
chrUn	59250971	59251693	723	CarSat1	red	na
chrUn	59251709	59252909	1201	CarSat1	red	na

chrUn	59252936	59253647	712	CarSat1	red	na
chrUn	59253674	59254636	963	CarSat1	red	na
chrUn	59254652	59255857	1206	CarSat1	red	na
chrUn	59255884	59256591	708	CarSat1	red	na
chrUn	59256618	59257328	711	CarSat1	red	na
chrUn	59257355	59258062	708	CarSat1	red	na
chrUn	59258093	59259708	1616	CarSat1	red	na
chrUn	59260716	59260822	107	Carsat2	orange	na
chrUn	59260741	59261854	1114	CarSat1	red	na
chrUn	59261884	59262863	980	CarSat1	red	na
chrUn	59262890	59263602	713	CarSat1	red	na
chrUn	59263629	59265078	1450	CarSat1	red	na
chrUn	59265105	59266315	1211	CarSat1	red	na
chrUn	59267206	59267758	553	CarSat1	red	na
chrUn	59267788	59269750	1963	CarSat1	red	na
chrUn	59269774	59271228	1455	CarSat1	red	na
chrUn	59271252	59272932	1681	CarSat1	red	na
chrUn	59272962	59273673	712	CarSat1	red	na
chrUn	59273688	59273814	127	Carsat2	orange	na
chrUn	59273703	59274414	712	CarSat1	red	na
chrUn	59274429	59274555	127	Carsat2	orange	na
chrUn	59274444	59274997	554	CarSat1	red	na
chrUn	59294186	59295534	1349	CarSat1	red	na
chrUn	59295564	59297746	2183	CarSat1	red	na
chrUn	59297776	59298998	1223	CarSat1	red	na
chrUn	59299022	59299661	640	CarSat1	red	na
chrUn	59299871	59300601	731	CarSat1	red	na
chrUn	59301632	59302800	1169	SAT1_CF	blue	na
chrUn	59309625	59310341	717	SAT1_CF	blue	na
chrUn	59310419	59310927	509	SAT1_CF	blue	na
chrUn	59310941	59311592	652	SAT1_CF	blue	na
chrUn	59313340	59313947	608	SAT1_CF	blue	na
chrUn	59313961	59314646	686	SAT1_CF	blue	na
chrUn	59315021	59315365	345	SAT1_CF	blue	na
chrUn	59315391	59315955	565	SAT1_CF	blue	na
chrUn	59316035	59316301	267	SAT1_CF	blue	na
chrUn	59317503	59317734	232	SAT1_CF	blue	na
chrUn	59317747	59318419	673	SAT1_CF	blue	na
chrUn	59318464	59319096	633	SAT1_CF	blue	na
chrUn	59319124	59320359	1236	SAT1_CF	blue	na
chrUn	59320371	59321595	1225	SAT1_CF	blue	na
chrUn	59321629	59322088	460	SAT1_CF	blue	na

chrUn	59322489	59323116	628	SAT1_CF	blue	na
chrUn	59323163	59324254	1092	SAT1_CF	blue	na
chrUn	59330142	59330241	100	SAT1_CF	blue	na
chrUn	59330253	59332177	1925	SAT1_CF	blue	na
chrUn	59332204	59332724	521	SAT1_CF	blue	na
chrUn	59332766	59333078	313	SAT1_CF	blue	na
chrUn	59333089	59333275	187	SAT1_CF	blue	na
chrUn	59336029	59336602	574	SAT1_CF	blue	na
chrUn	59336642	59337136	495	SAT1_CF	blue	na
chrUn	59337156	59337839	684	SAT1_CF	blue	na
chrUn	59337862	59338213	352	SAT1_CF	blue	na
chrUn	59338779	59339952	1174	SAT1_CF	blue	na
chrUn	59340983	59342032	1050	CarSat1	red	na
chrUn	59342063	59343651	1589	CarSat1	red	na
chrUn	59367405	59367505	101	Sat7CF	lime	na
chrUn	59367452	59368214	763	Sat7CF	lime	na
chrUn	59367531	59368214	684	Sat7CF	lime	na
chrUn	59384986	59385963	978	CarSat1	red	na
chrUn	59385988	59386708	721	CarSat1	red	na
chrUn	59386724	59387436	713	CarSat1	red	na
chrUn	59387461	59389899	2439	CarSat1	red	na
chrUn	59389926	59390394	469	CarSat1	red	na
chrUn	59390410	59391082	673	CarSat1	red	na
chrUn	59391469	59392000	532	CarSat1	red	na
chrUn	59392016	59393475	1460	CarSat1	red	na
chrUn	59393491	59395933	2443	CarSat1	red	na
chrUn	59395955	59396665	711	CarSat1	red	na
chrUn	59396692	59397672	981	CarSat1	red	na
chrUn	59397702	59398879	1178	CarSat1	red	na
chrUn	59398906	59399450	545	CarSat1	red	na
chrUn	59401157	59402266	1110	CarSat1	red	na
chrUn	59402295	59403257	963	CarSat1	red	na
chrUn	59403284	59404257	974	CarSat1	red	na
chrUn	59404287	59405462	1176	CarSat1	red	na
chrUn	59405488	59406745	1258	CarSat1	red	na
chrUn	59407802	59408238	437	SAT1_CF	blue	na
chrUn	59408251	59409919	1669	SAT1_CF	blue	na
chrUn	59409962	59411513	1552	SAT1_CF	blue	na
chrUn	59411526	59411881	356	SAT1_CF	blue	na
chrUn	59411968	59412469	502	SAT1_CF	blue	na
chrUn	59412496	59413156	661	SAT1_CF	blue	na
chrUn	59413203	59413320	118	SAT1_CF	blue	na

chrUn	59413362	59413903	542	SAT1_CF	blue	na
chrUn	59413941	59415170	1230	SAT1_CF	blue	na
chrUn	59415287	59415807	521	SAT1_CF	blue	na
chrUn	59415838	59417845	2008	SAT1_CF	blue	na
chrUn	59417898	59418398	501	SAT1_CF	blue	na
chrUn	59418423	59419093	671	SAT1_CF	blue	na
chrUn	59419105	59420439	1335	SAT1_CF	blue	na
chrUn	59420483	59421046	564	SAT1_CF	blue	na
chrUn	59421126	59421392	267	SAT1_CF	blue	na
chrUn	59421495	59423781	2287	SAT1_CF	blue	na
chrUn	59423808	59425181	1374	SAT1_CF	blue	na
chrUn	59425193	59425868	676	SAT1_CF	blue	na
chrUn	59425880	59426053	174	SAT1_CF	blue	na
chrUn	59426096	59426886	791	SAT1_CF	blue	na
chrUn	59426930	59427542	613	SAT1_CF	blue	na
chrUn	59428544	59430384	1841	CarSat1	red	na
chrUn	59430399	59432810	2412	CarSat1	red	na
chrUn	59432834	59437009	4176	CarSat1	red	na
chrUn	59437025	59438483	1459	CarSat1	red	na
chrUn	59438499	59439204	706	CarSat1	red	na
chrUn	59439234	59446058	6825	CarSat1	red	na
chrUn	59446091	59447784	1694	CarSat1	red	na
chrUn	59447805	59448263	459	CarSat1	red	na
chrUn	59476653	59477198	546	Sat2CF	purple	na
chrUn	59496147	59496936	790	CarSat1	red	na
chrUn	59496966	59497674	709	CarSat1	red	na
chrUn	59497689	59497816	128	Carsat2	orange	na
chrUn	59497704	59499300	1597	CarSat1	red	na
chrUn	59500333	59501242	910	CarSat1	red	na
chrUn	59501253	59503920	2668	CarSat1	red	na
chrUn	59503935	59504062	128	Carsat2	orange	na
chrUn	59503950	59507610	3661	CarSat1	red	na
chrUn	59507625	59507752	128	Carsat2	orange	na
chrUn	59507640	59508617	978	CarSat1	red	na
chrUn	59508638	59509356	719	CarSat1	red	na
chrUn	59509381	59510342	962	CarSat1	red	na
chrUn	59510363	59511300	938	CarSat1	red	na
chrUn	59511315	59511441	127	Carsat2	orange	na
chrUn	59511330	59512775	1446	CarSat1	red	na
chrUn	59512790	59512916	127	Carsat2	orange	na
chrUn	59512805	59516835	4031	CarSat1	red	na
chrUn	59545227	59545927	701	CarSat1	red	na

chrUn	59546123	59546732	610	CarSat1	red	na
chrUn	59546749	59549671	2923	CarSat1	red	na
chrUn	59549692	59551141	1450	CarSat1	red	na
chrUn	59551168	59551864	697	CarSat1	red	na
chrUn	59551906	59553605	1700	CarSat1	red	na
chrUn	59553621	59555077	1457	CarSat1	red	na
chrUn	59555093	59555815	723	CarSat1	red	na
chrUn	59555831	59556551	721	CarSat1	red	na
chrUn	59556567	59557500	934	CarSat1	red	na
chrUn	59557530	59558841	1312	CarSat1	red	na
chrUn	59607614	59608518	905	CarSat1	red	na
chrUn	59608704	59608850	147	CarSat1	red	na
chrUn	59608888	59609369	482	CarSat1	red	na
chrUn	59609394	59610330	937	CarSat1	red	na
chrUn	59610360	59611066	707	CarSat1	red	na
chrUn	59611096	59611802	707	CarSat1	red	na
chrUn	59611832	59612394	563	CarSat1	red	na
chrUn	59612581	59612727	147	CarSat1	red	na
chrUn	59612765	59614063	1299	CarSat1	red	na
chrUn	59614249	59614403	155	CarSat1	red	na
chrUn	59614433	59614995	563	CarSat1	red	na
chrUn	59615181	59615424	244	CarSat1	red	na
chrUn	59616878	59617022	145	CarSat1	red	na
chrUn	59617220	59617413	194	CarSat1	red	na
chrUn	59617430	59617880	451	CarSat1	red	na
chrUn	59617910	59618472	563	CarSat1	red	na
chrUn	59618658	59618812	155	CarSat1	red	na
chrUn	59618842	59619820	979	CarSat1	red	na
chrUn	59619847	59621763	1917	CarSat1	red	na
chrUn	59621793	59622355	563	CarSat1	red	na
chrUn	59622538	59622692	155	CarSat1	red	na
chrUn	59622722	59624689	1968	CarSat1	red	na
chrUn	59624705	59625427	723	CarSat1	red	na
chrUn	59625443	59625903	461	CarSat1	red	na
chrUn	59625930	59626643	714	CarSat1	red	na
chrUn	59626670	59627380	711	CarSat1	red	na
chrUn	59627407	59628077	671	CarSat1	red	na
chrUn	59670147	59671362	1216	CarSat1	red	na
chrUn	59671387	59672107	721	CarSat1	red	na
chrUn	59672124	59672840	717	CarSat1	red	na
chrUn	59672856	59673564	709	CarSat1	red	na
chrUn	59673589	59674308	720	CarSat1	red	na

chrUn	59674325	59674859	535	CarSat1	red	na
chrUn	59675033	59675604	572	CarSat1	red	na
chrUn	59675648	59676217	570	SAT1_CF	blue	na
chrUn	59676257	59677631	1375	SAT1_CF	blue	na
chrUn	59677644	59678991	1348	SAT1_CF	blue	na
chrUn	59679077	59681293	2217	SAT1_CF	blue	na
chrUn	59681326	59681879	554	SAT1_CF	blue	na
chrUn	59681958	59682778	821	SAT1_CF	blue	na
chrUn	59682821	59682929	109	SAT1_CF	blue	na
chrUn	59682955	59684170	1216	SAT1_CF	blue	na
chrUn	59684182	59684291	110	SAT1_CF	blue	na
chrUn	59684311	59685514	1204	SAT1_CF	blue	na
chrUn	59685558	59686384	827	SAT1_CF	blue	na
chrUn	59687781	59689572	1792	SAT1_CF	blue	na
chrUn	59690589	59691458	870	CarSat1	red	na
chrUn	59691473	59691600	128	Carsat2	orange	na
chrUn	59691488	59692936	1449	CarSat1	red	na
chrUn	59692951	59693078	128	Carsat2	orange	na
chrUn	59692966	59693671	706	CarSat1	red	na
chrUn	59693686	59693809	124	Carsat2	orange	na
chrUn	59693701	59694412	712	CarSat1	red	na
chrUn	59694427	59694554	128	Carsat2	orange	na
chrUn	59694442	59695148	707	CarSat1	red	na
chrUn	59695163	59695289	127	Carsat2	orange	na
chrUn	59695178	59695884	707	CarSat1	red	na
chrUn	59695899	59696022	124	Carsat2	orange	na
chrUn	59695914	59696399	486	CarSat1	red	na
chrUn	59696432	59697366	935	CarSat1	red	na
chrUn	59697381	59697508	128	Carsat2	orange	na
chrUn	59697396	59698103	708	CarSat1	red	na
chrUn	59698118	59698244	127	Carsat2	orange	na
chrUn	59698133	59698840	708	CarSat1	red	na
chrUn	59699339	59700315	977	CarSat1	red	na
chrUn	59700330	59700457	128	Carsat2	orange	na
chrUn	59700345	59701052	708	CarSat1	red	na
chrUn	59701067	59701193	127	Carsat2	orange	na
chrUn	59701082	59701793	712	CarSat1	red	na
chrUn	59701823	59702530	708	CarSat1	red	na
chrUn	59702545	59702670	126	Carsat2	orange	na
chrUn	59702560	59703270	711	CarSat1	red	na
chrUn	59703300	59703786	487	CarSat1	red	na
chrUn	59703821	59704749	929	CarSat1	red	na

chrUn	59704764	59704890	127	Carsat2	orange	na
chrUn	59704779	59705486	708	CarSat1	red	na
chrUn	59705501	59705628	128	Carsat2	orange	na
chrUn	59705516	59706223	708	CarSat1	red	na
chrUn	59706238	59706364	127	Carsat2	orange	na
chrUn	59706253	59706960	708	CarSat1	red	na
chrUn	59706975	59707102	128	Carsat2	orange	na
chrUn	59706990	59707697	708	CarSat1	red	na
chrUn	59707727	59708433	707	CarSat1	red	na
chrUn	59708448	59708574	127	Carsat2	orange	na
chrUn	59708463	59708948	486	CarSat1	red	na
chrUn	59708982	59709911	930	CarSat1	red	na
chrUn	59709941	59710532	592	CarSat1	red	na
chrUn	59770720	59772253	1534	CarSat1	red	na
chrUn	59772268	59772391	124	Carsat2	orange	na
chrUn	59772283	59772989	707	CarSat1	red	na
chrUn	59773019	59773504	486	CarSat1	red	na
chrUn	59773537	59774468	932	CarSat1	red	na
chrUn	59774483	59774610	128	Carsat2	orange	na
chrUn	59774498	59775205	708	CarSat1	red	na
chrUn	59775220	59775347	128	Carsat2	orange	na
chrUn	59775235	59776216	982	CarSat1	red	na
chrUn	59776241	59777427	1187	CarSat1	red	na
chrUn	59777442	59777568	127	Carsat2	orange	na
chrUn	59777457	59778164	708	CarSat1	red	na
chrUn	59778194	59778895	702	CarSat1	red	na
chrUn	59778925	59779630	706	CarSat1	red	na
chrUn	59779675	59780367	693	CarSat1	red	na
chrUn	59780401	59781105	705	CarSat1	red	na
chrUn	59781135	59781839	705	CarSat1	red	na
chrUn	59781869	59782573	705	CarSat1	red	na
chrUn	59782603	59783307	705	CarSat1	red	na
chrUn	59783339	59784775	1437	CarSat1	red	na
chrUn	59784805	59785509	705	CarSat1	red	na
chrUn	59785539	59786517	979	CarSat1	red	na
chrUn	59786538	59786975	438	CarSat1	red	na
chrUn	59787009	59787710	702	CarSat1	red	na
chrUn	59787744	59789183	1440	CarSat1	red	na
chrUn	59789213	59790066	854	CarSat1	red	na
chrUn	59866046	59866590	545	CarSat1	red	na
chrUn	59866615	59869049	2435	CarSat1	red	na
chrUn	59869076	59871015	1940	CarSat1	red	na

chrUn	59871031	59871989	959	CarSat1	red	na
chrUn	59872016	59873728	1713	CarSat1	red	na
chrUn	59873758	59874469	712	CarSat1	red	na
chrUn	59874499	59878156	3658	CarSat1	red	na
chrUn	59878173	59880381	2209	CarSat1	red	na
chrUn	59880397	59881104	708	CarSat1	red	na
chrUn	59881134	59882580	1447	CarSat1	red	na
chrUn	59882610	59884066	1457	CarSat1	red	na
chrUn	59884081	59885375	1295	CarSat1	red	na
chrUn	59886380	59887026	647	SAT1_CF	blue	na
chrUn	59887044	59888808	1765	SAT1_CF	blue	na
chrUn	59888829	59890058	1230	SAT1_CF	blue	na
chrUn	59890099	59891793	1695	SAT1_CF	blue	na
chrUn	59891972	59892156	185	SAT1_CF	blue	na
chrUn	59892177	59892696	520	SAT1_CF	blue	na
chrUn	59892709	59892814	106	SAT1_CF	blue	na
chrUn	59892837	59894109	1273	SAT1_CF	blue	na
chrUn	59894124	59894260	137	SAT1_CF	blue	na
chrUn	59894326	59894828	503	SAT1_CF	blue	na
chrUn	59894841	59894947	107	SAT1_CF	blue	na
chrUn	59894969	59895525	557	SAT1_CF	blue	na
chrUn	59895539	59896906	1368	SAT1_CF	blue	na
chrUn	59896949	59897917	969	SAT1_CF	blue	na
chrUn	59897987	59898700	714	SAT1_CF	blue	na
chrUn	59898714	59899235	522	SAT1_CF	blue	na
chrUn	59899269	59899648	380	SAT1_CF	blue	na
chrUn	59899688	59900189	502	SAT1_CF	blue	na
chrUn	59900202	59900308	107	SAT1_CF	blue	na
chrUn	59900330	59900884	555	SAT1_CF	blue	na
chrUn	59900896	59902344	1449	SAT1_CF	blue	na
chrUn	59902390	59903018	629	SAT1_CF	blue	na
chrUn	59903045	59903608	564	SAT1_CF	blue	na
chrUn	59903688	59903882	195	SAT1_CF	blue	na
chrUn	59903908	59904903	996	SAT1_CF	blue	na
chrUn	59904923	59905689	767	SAT1_CF	blue	na
chrUn	59936154	59936828	675	CarSat1	red	na
chrUn	59936849	59938045	1197	CarSat1	red	na
chrUn	59938070	59939037	968	CarSat1	red	na
chrUn	59939058	59939527	470	CarSat1	red	na
chrUn	59939552	59940254	703	CarSat1	red	na
chrUn	59940279	59941731	1453	CarSat1	red	na
chrUn	59941756	59942723	968	CarSat1	red	na

chrUn	59942744	59944460	1717	CarSat1	red	na
chrUn	59944490	59944929	440	CarSat1	red	na
chrUn	59944956	59945934	979	CarSat1	red	na
chrUn	59945964	59946409	446	CarSat1	red	na
chrUn	59946430	59948161	1732	CarSat1	red	na
chrUn	59948176	59948881	706	CarSat1	red	na
chrUn	59948911	59951561	2651	CarSat1	red	na
chrUn	59951589	59952568	980	CarSat1	red	na
chrUn	59952598	59954512	1915	CarSat1	red	na
chrUn	59954539	59955424	886	CarSat1	red	na
chrUn	59956427	59956557	131	SAT1_CF	blue	na
chrUn	59956580	59956686	107	SAT1_CF	blue	na
chrUn	59956699	59957200	502	SAT1_CF	blue	na
chrUn	59957240	59958264	1025	SAT1_CF	blue	na
chrUn	59959298	59959909	612	SAT1_CF	blue	na
chrUn	59959922	59961232	1311	SAT1_CF	blue	na
chrUn	59961276	59962784	1509	SAT1_CF	blue	na
chrUn	59964936	59965750	815	SAT1_CF	blue	na
chrUn	59965792	59966408	617	SAT1_CF	blue	na
chrUn	59968551	59969110	560	SAT1_CF	blue	na
chrUn	59969142	59969648	507	SAT1_CF	blue	na
chrUn	59969696	59970433	738	SAT1_CF	blue	na
chrUn	59972170	59973090	921	SAT1_CF	blue	na
chrUn	59973132	59973656	525	SAT1_CF	blue	na
chrUn	59973670	59973743	74	SAT1_CF	blue	na
chrUn	59975759	59976250	492	SAT1_CF	blue	na
chrUn	59976303	59977568	1266	SAT1_CF	blue	na
chrUn	59977605	59978148	544	SAT1_CF	blue	na
chrUn	59978190	59979621	1432	SAT1_CF	blue	na
chrUn	59979641	59979749	109	SAT1_CF	blue	na
chrUn	59979762	59980282	521	SAT1_CF	blue	na
chrUn	59980308	59980463	156	SAT1_CF	blue	na
chrUn	59982113	59983641	1529	SAT1_CF	blue	na
chrUn	59983653	59984205	553	SAT1_CF	blue	na
chrUn	59984224	59984442	219	SAT1_CF	blue	na
chrUn	59984486	59985076	591	SAT1_CF	blue	na
chrUn	59987600	59988308	709	SAT1_CF	blue	na
chrUn	59988360	59988598	239	SAT1_CF	blue	na
chrUn	60009868	60011205	1338	CarSat1	red	na
chrUn	60011235	60011681	447	CarSat1	red	na
chrUn	60011702	60012418	717	CarSat1	red	na
chrUn	60012439	60013417	979	CarSat1	red	na

chrUn	60013447	60013887	441	CarSat1	red	na
chrUn	60013914	60015118	1205	CarSat1	red	na
chrUn	60015143	60016095	953	CarSat1	red	na
chrUn	60016125	60017329	1205	CarSat1	red	na
chrUn	60017354	60019530	2177	CarSat1	red	na
chrUn	60019555	60021005	1451	CarSat1	red	na
chrUn	60021039	60021992	954	CarSat1	red	na
chrUn	60022019	60024472	2454	CarSat1	red	na
chrUn	60024502	60025438	937	CarSat1	red	na
chrUn	60025463	60026683	1221	CarSat1	red	na
chrUn	60026713	60027159	447	CarSat1	red	na
chrUn	60027178	60027784	607	CarSat1	red	na
chrUn	60027970	60029187	1218	CarSat1	red	na
chrUn	60033357	60034120	764	SAT1_CF	blue	na
chrUn	60034132	60036270	2139	SAT1_CF	blue	na
chrUn	60036300	60036788	489	SAT1_CF	blue	na
chrUn	60036840	60036997	158	SAT1_CF	blue	na
chrUn	60037042	60037533	492	SAT1_CF	blue	na
chrUn	60037550	60038235	686	SAT1_CF	blue	na
chrUn	60038246	60039517	1272	SAT1_CF	blue	na
chrUn	60039559	60040339	781	SAT1_CF	blue	na
chrUn	60040383	60042250	1868	SAT1_CF	blue	na
chrUn	60042267	60043601	1335	SAT1_CF	blue	na
chrUn	60043664	60044134	471	SAT1_CF	blue	na
chrUn	60044154	60044260	107	SAT1_CF	blue	na
chrUn	60044283	60044836	554	SAT1_CF	blue	na
chrUn	60044848	60044970	123	SAT1_CF	blue	na
chrUn	60044991	60045386	396	SAT1_CF	blue	na
chrUn	60045400	60046543	1144	SAT1_CF	blue	na
chrUn	60046583	60048319	1737	SAT1_CF	blue	na
chrUn	60048384	60048520	137	SAT1_CF	blue	na
chrUn	60048587	60049396	810	SAT1_CF	blue	na
chrUn	60108808	60109986	1179	Sat2CF	purple	na
chrUn	60138578	60139247	670	Sat54CF	na	na
chrUn	60139493	60140215	723	Sat54CF	na	na
chrUn	60140301	60140840	540	Sat54CF	na	na
chrUn	60141091	60141283	193	Sat54CF	na	na
chrUn	60156253	60157217	965	Sat90CF	na	na
chrUn	60156365	60157365	1001	Sat90CF	na	na
chrUn	60156489	60157514	1026	Sat90CF	na	na
chrUn	60156715	60157497	783	Sat90CF	na	na
chrUn	60158608	60162439	3832	CarSat1	red	na

chrUn	60162463	60163667	1205	CarSat1	red	na
chrUn	60163688	60164649	962	CarSat1	red	na
chrUn	60164673	60165385	713	CarSat1	red	na
chrUn	60165409	60166343	935	CarSat1	red	na
chrUn	60166355	60166484	130	Carsat2	orange	na
chrUn	60166373	60167592	1220	CarSat1	red	na
chrUn	60167616	60168328	713	CarSat1	red	na
chrUn	60168352	60169286	935	CarSat1	red	na
chrUn	60169316	60170024	709	CarSat1	red	na
chrUn	60170054	60170761	708	CarSat1	red	na
chrUn	60170791	60171501	711	CarSat1	red	na
chrUn	60171533	60173504	1972	CarSat1	red	na
chrUn	60173520	60175195	1676	CarSat1	red	na
chrUn	60175225	60175711	487	CarSat1	red	na
chrUn	60175735	60177761	2027	CarSat1	red	na
chrUn	60178925	60179208	284	CarSat1	red	na
chrUn	60319001	60319284	284	Sat2CF	purple	na
chrUn	60319011	60319264	254	Sat2CF	purple	na
chrUn	60319130	60319292	163	Sat2CF	purple	na
chrUn	60327547	60328712	1166	CarSat1	red	na
chrUn	60328739	60329208	470	CarSat1	red	na
chrUn	60329224	60330187	964	CarSat1	red	na
chrUn	60330214	60330925	712	CarSat1	red	na
chrUn	60330952	60331662	711	CarSat1	red	na
chrUn	60331689	60332397	709	CarSat1	red	na
chrUn	60332424	60333134	711	CarSat1	red	na
chrUn	60333161	60335345	2185	CarSat1	red	na
chrUn	60335372	60336083	712	CarSat1	red	na
chrUn	60336110	60336820	711	CarSat1	red	na
chrUn	60336847	60337557	711	CarSat1	red	na
chrUn	60337584	60338294	711	CarSat1	red	na
chrUn	60338321	60339515	1195	CarSat1	red	na
chrUn	60339540	60340503	964	CarSat1	red	na
chrUn	60340530	60342716	2187	CarSat1	red	na
chrUn	60342743	60346577	3835	CarSat1	red	na
chrUn	60415103	60415175	73	Sat10CF	na	na
chrUn	60415113	60415175	63	Sat10CF	na	na
chrUn	60416932	60417219	288	Sat10CF	na	na
chrUn	60416956	60417136	181	Sat10CF	na	na
chrUn	60418553	60419154	602	Sat10CF	na	na
chrUn	60418815	60419174	360	Sat10CF	na	na
chrUn	60418553	60419154	602	Sat10CF	na	na

chrUn	60418815	60419174	360	Sat10CF	na	na
chrUn	60492185	60493094	910	CarSat1	red	na
chrUn	60493159	60494678	1520	CarSat1	red	na
chrUn	60494697	60495876	1180	CarSat1	red	na
chrUn	60495881	60496881	1001	SAT1_CF	blue	na
chrUn	60496893	60497115	223	SAT1_CF	blue	na
chrUn	60497131	60497654	524	SAT1_CF	blue	na
chrUn	60497676	60500837	3162	SAT1_CF	blue	na
chrUn	60500832	60501713	882	CarSat1	red	na
chrUn	60501745	60502991	1247	CarSat1	red	na
chrUn	60503012	60503513	502	CarSat1	red	na
chrUn	60503703	60504800	1098	CarSat1	red	na
chrUn	60505767	60506180	414	CarSat1	red	na
chrUn	60508329	60508966	638	CarSat1	red	na
chrUn	60508978	60510175	1198	CarSat1	red	na
chrUn	60510190	60510317	128	Carsat2	orange	na
chrUn	60510205	60510917	713	CarSat1	red	na
chrUn	60510947	60511920	974	CarSat1	red	na
chrUn	60511950	60512389	440	CarSat1	red	na
chrUn	60512404	60512530	127	Carsat2	orange	na
chrUn	60512419	60513866	1448	CarSat1	red	na
chrUn	60513881	60514007	127	Carsat2	orange	na
chrUn	60513896	60514607	712	CarSat1	red	na
chrUn	60514622	60514749	128	Carsat2	orange	na
chrUn	60514638	60516827	2190	CarSat1	red	na
chrUn	60516858	60517568	711	CarSat1	red	na
chrUn	60517598	60518310	713	CarSat1	red	na
chrUn	60518340	60519047	708	CarSat1	red	na
chrUn	60519062	60519188	127	Carsat2	orange	na
chrUn	60519077	60519788	712	CarSat1	red	na
chrUn	60519818	60521716	1899	CarSat1	red	na
chrUn	60521850	60522866	1017	CarSat1	red	na
chrUn	60522903	60523388	486	CarSat1	red	na
chrUn	60523422	60524346	925	CarSat1	red	na
chrUn	60524361	60524487	127	Carsat2	orange	na
chrUn	60524376	60524860	485	CarSat1	red	na
chrUn	60524894	60525819	926	CarSat1	red	na
chrUn	60525834	60525949	116	Carsat2	orange	na
chrUn	60525849	60527220	1372	CarSat1	red	na
chrUn	60550234	60553487	3254	CarSat1	red	na
chrUn	60553520	60554019	500	CarSat1	red	na
chrUn	60554034	60554756	723	CarSat1	red	na

chrUn	60554771	60556432	1662	CarSat1	red	na
chrUn	60556465	60559923	3459	CarSat1	red	na
chrUn	60559938	60560645	708	CarSat1	red	na
chrUn	60560675	60561398	724	CarSat1	red	na
chrUn	60561413	60563596	2184	CarSat1	red	na
chrUn	60563626	60564334	709	CarSat1	red	na
chrUn	60564364	60565069	706	CarSat1	red	na
chrUn	60565099	60566558	1460	CarSat1	red	na
chrUn	60566573	60569151	2579	CarSat1	red	na
chrUn	60570156	60573571	3416	CarSat1	red	na
chrUn	60575146	60584424	9279	CarSat1	red	na
chrUn	60584457	60587370	2914	CarSat1	red	na
chrUn	60587394	60588107	714	CarSat1	red	na
chrUn	60588131	60588766	636	CarSat1	red	na
chrUn	60589130	60590987	1858	CarSat1	red	na
chrUn	60591999	60592624	626	CarSat1	red	na
chrUn	60592640	60594838	2199	CarSat1	red	na
chrUn	60594854	60597050	2197	CarSat1	red	na
chrUn	60597066	60597535	470	CarSat1	red	na
chrUn	60597562	60598272	711	CarSat1	red	na
chrUn	60598299	60599748	1450	CarSat1	red	na
chrUn	60599775	60600485	711	CarSat1	red	na
chrUn	60600512	60601475	964	CarSat1	red	na
chrUn	60601491	60602213	723	CarSat1	red	na
chrUn	60602229	60603687	1459	CarSat1	red	na
chrUn	60603712	60604905	1194	CarSat1	red	na
chrUn	60604932	60605894	963	CarSat1	red	na
chrUn	60605910	60607113	1204	CarSat1	red	na
chrUn	60607141	60608103	963	CarSat1	red	na
chrUn	60608119	60609577	1459	CarSat1	red	na
chrUn	60609593	60610315	723	CarSat1	red	na
chrUn	60610331	60610895	565	CarSat1	red	na
chrUn	60611912	60612414	503	SAT1_CF	blue	na
chrUn	60612454	60612948	495	SAT1_CF	blue	na
chrUn	60612968	60615321	2354	SAT1_CF	blue	na
chrUn	60615338	60616018	681	SAT1_CF	blue	na
chrUn	60616030	60617487	1458	SAT1_CF	blue	na
chrUn	60617515	60618660	1146	SAT1_CF	blue	na
chrUn	60618693	60618851	159	SAT1_CF	blue	na
chrUn	60618876	60619396	521	SAT1_CF	blue	na
chrUn	60619414	60620104	691	SAT1_CF	blue	na
chrUn	60620613	60622056	1444	SAT1_CF	blue	na

chrUn	60622068	60623231	1164	SAT1_CF	blue	na
chrUn	60623282	60623476	195	SAT1_CF	blue	na
chrUn	60623732	60624244	513	SAT1_CF	blue	na
chrUn	60624468	60624602	135	SAT1_CF	blue	na
chrUn	60624689	60626891	2203	SAT1_CF	blue	na
chrUn	60627785	60628321	537	SAT1_CF	blue	na
chrUn	60628338	60629016	679	SAT1_CF	blue	na
chrUn	60629414	60629824	411	SAT1_CF	blue	na
chrUn	60630006	60630468	463	SAT1_CF	blue	na
chrUn	60630963	60631172	210	SAT1_CF	blue	na
chrUn	60631185	60631290	106	SAT1_CF	blue	na
chrUn	60631314	60632132	819	SAT1_CF	blue	na
chrUn	60635322	60635700	379	SAT1_CF	blue	na
chrUn	60635718	60636416	699	SAT1_CF	blue	na
chrUn	60640994	60641873	880	Sat2CF	purple	na
chrUn	60641083	60642159	1077	Sat2CF	purple	na
chrUn	60642036	60642184	149	Sat2CF	purple	na
chrUn	60690945	60691902	958	CarSat1	red	na
chrUn	60692062	60693501	1440	CarSat1	red	na
chrUn	60699410	60701811	2402	SAT1_CF	blue	na
chrUn	60701823	60702140	318	SAT1_CF	blue	na
chrUn	60702215	60702782	568	SAT1_CF	blue	na
chrUn	60702801	60703318	518	SAT1_CF	blue	na
chrUn	60703388	60703905	518	SAT1_CF	blue	na
chrUn	60703992	60705623	1632	SAT1_CF	blue	na
chrUn	60705637	60707004	1368	SAT1_CF	blue	na
chrUn	60707050	60708703	1654	SAT1_CF	blue	na
chrUn	60708714	60709031	318	SAT1_CF	blue	na
chrUn	60709104	60710282	1179	SAT1_CF	blue	na
chrUn	60710294	60710969	676	SAT1_CF	blue	na
chrUn	60711014	60711662	649	SAT1_CF	blue	na
chrUn	60711698	60712233	536	SAT1_CF	blue	na
chrUn	60712314	60712837	524	SAT1_CF	blue	na
chrUn	60712901	60713447	547	SAT1_CF	blue	na
chrUn	60713460	60715333	1874	SAT1_CF	blue	na
chrUn	60715361	60715518	158	SAT1_CF	blue	na
chrUn	60715563	60716164	602	SAT1_CF	blue	na
chrUn	60716191	60716736	546	SAT1_CF	blue	na
chrUn	60716780	60718199	1420	SAT1_CF	blue	na
chrUn	60755410	60756222	813	Sat2CF	purple	na
chrUn	60755909	60756296	388	Sat2CF	purple	na
chrUn	60758832	60760357	1526	CarSat1	red	na

chrUn	60760387	60761833	1447	CarSat1	red	na
chrUn	60761863	60762571	709	CarSat1	red	na
chrUn	60762601	60763821	1221	CarSat1	red	na
chrUn	60763846	60765508	1663	CarSat1	red	na
chrUn	60765545	60766525	981	CarSat1	red	na
chrUn	60767552	60768703	1152	CarSat1	red	na
chrUn	60768773	60769044	272	CarSat1	red	na
chrUn	60770282	60772683	2402	CarSat1	red	na
chrUn	60772713	60773421	709	CarSat1	red	na
chrUn	60773451	60776373	2923	CarSat1	red	na
chrUn	60776403	60777111	709	CarSat1	red	na
chrUn	60777141	60779849	2709	CarSat1	red	na
chrUn	60802675	60803305	631	CarSat1	red	na
chrUn	60803338	60803838	501	CarSat1	red	na
chrUn	60803853	60805314	1462	CarSat1	red	na
chrUn	60805329	60806772	1444	CarSat1	red	na
chrUn	60806803	60807523	721	CarSat1	red	na
chrUn	60807539	60809203	1665	CarSat1	red	na
chrUn	60809236	60809721	486	CarSat1	red	na
chrUn	60809751	60810472	722	CarSat1	red	na
chrUn	60810487	60811687	1201	CarSat1	red	na
chrUn	60811730	60813628	1899	CarSat1	red	na
chrUn	60813661	60814375	715	CarSat1	red	na
chrUn	60814399	60815101	703	CarSat1	red	na
chrUn	60815134	60816575	1442	CarSat1	red	na
chrUn	60816608	60818795	2188	CarSat1	red	na
chrUn	60818819	60821441	2623	CarSat1	red	na
chrUn	60832235	60833073	839	Sat2CF	purple	na
chrUn	60832296	60833022	727	Sat2CF	purple	na
chrUn	60844134	60844647	514	Sat2CF	purple	na
chrUn	60845670	60846929	1260	CarSat1	red	na
chrUn	60846956	60847914	959	CarSat1	red	na
chrUn	60847930	60848398	469	CarSat1	red	na
chrUn	60848425	60849136	712	CarSat1	red	na
chrUn	60849163	60850220	1058	CarSat1	red	na
chrUn	60850407	60851433	1027	CarSat1	red	na
chrUn	60852350	60853686	1337	CarSat1	red	na
chrUn	60853708	60854421	714	CarSat1	red	na
chrUn	60854448	60855411	964	CarSat1	red	na
chrUn	60855437	60855895	459	CarSat1	red	na
chrUn	60855922	60856632	711	CarSat1	red	na
chrUn	60856659	60857366	708	CarSat1	red	na

chrUn	60857393	60858839	1447	CarSat1	red	na
chrUn	60858866	60859575	710	CarSat1	red	na
chrUn	60859602	60860314	713	CarSat1	red	na
chrUn	60860339	60861300	962	CarSat1	red	na
chrUn	60861316	60862522	1207	CarSat1	red	na
chrUn	60862549	60863998	1450	CarSat1	red	na
chrUn	60864025	60864734	710	CarSat1	red	na
chrUn	60864761	60865306	546	CarSat1	red	na
chrUn	60866400	60868100	1701	CarSat1	red	na
chrUn	60868116	60868836	721	CarSat1	red	na
chrUn	60868852	60870059	1208	CarSat1	red	na
chrUn	60870086	60871047	962	CarSat1	red	na
chrUn	60871063	60871531	469	CarSat1	red	na
chrUn	60871558	60873258	1701	CarSat1	red	na
chrUn	60873274	60873741	468	CarSat1	red	na
chrUn	60873768	60874945	1178	CarSat1	red	na
chrUn	60874975	60876937	1963	CarSat1	red	na
chrUn	60876961	60877674	714	CarSat1	red	na
chrUn	60877698	60880399	2702	CarSat1	red	na
chrUn	60880420	60881136	717	CarSat1	red	na
chrUn	60881163	60882127	965	CarSat1	red	na
chrUn	60882143	60882864	722	CarSat1	red	na
chrUn	60882880	60883599	720	CarSat1	red	na
chrUn	60883615	60885024	1410	CarSat1	red	na
chrUn	60886033	60887105	1073	SAT1_CF	blue	na
chrUn	60887117	60887800	684	SAT1_CF	blue	na
chrUn	60887811	60888313	503	SAT1_CF	blue	na
chrUn	60888356	60888778	423	SAT1_CF	blue	na
chrUn	60888801	60888907	107	SAT1_CF	blue	na
chrUn	60888920	60890591	1672	SAT1_CF	blue	na
chrUn	60890654	60891433	780	SAT1_CF	blue	na
chrUn	60891481	60892157	677	SAT1_CF	blue	na
chrUn	60892169	60892852	684	SAT1_CF	blue	na
chrUn	60892864	60893908	1045	SAT1_CF	blue	na
chrUn	60893930	60895415	1486	SAT1_CF	blue	na
chrUn	60895427	60896516	1090	SAT1_CF	blue	na
chrUn	60896560	60897104	545	SAT1_CF	blue	na
chrUn	60897154	60897596	443	SAT1_CF	blue	na
chrUn	60897626	60898301	676	SAT1_CF	blue	na
chrUn	60898313	60899074	762	SAT1_CF	blue	na
chrUn	60899095	60899589	495	SAT1_CF	blue	na
chrUn	60899634	60899790	157	SAT1_CF	blue	na

chrUn	60899805	60902388	2584	SAT1_CF	blue	na
chrUn	60902400	60903030	631	SAT1_CF	blue	na
chrUn	60903042	60903652	611	SAT1_CF	blue	na
chrUn	60903712	60904840	1129	SAT1_CF	blue	na
chrUn	60905847	60906378	532	CarSat1	red	na
chrUn	60906591	60906939	349	CarSat1	red	na
chrUn	60907122	60907281	160	CarSat1	red	na
chrUn	60907297	60907591	295	CarSat1	red	na
chrUn	60907618	60908339	722	CarSat1	red	na
chrUn	60908488	60909439	952	CarSat1	red	na
chrUn	60925521	60927149	1629	SAT1_CF	blue	na
chrUn	60927161	60927812	652	SAT1_CF	blue	na
chrUn	60927857	60928357	501	SAT1_CF	blue	na
chrUn	60928397	60931017	2621	SAT1_CF	blue	na
chrUn	60931028	60931146	119	SAT1_CF	blue	na
chrUn	60931164	60931812	649	SAT1_CF	blue	na
chrUn	60931823	60932397	575	SAT1_CF	blue	na
chrUn	60932420	60932526	107	SAT1_CF	blue	na
chrUn	60932539	60934219	1681	SAT1_CF	blue	na
chrUn	60934273	60934400	128	SAT1_CF	blue	na
chrUn	60934444	60935088	645	SAT1_CF	blue	na
chrUn	60935101	60935776	676	SAT1_CF	blue	na
chrUn	60935788	60936472	685	SAT1_CF	blue	na
chrUn	60936485	60937063	579	SAT1_CF	blue	na
chrUn	60937384	60937621	238	SAT1_CF	blue	na
chrUn	60937654	60938807	1154	SAT1_CF	blue	na
chrUn	60938820	60940084	1265	SAT1_CF	blue	na
chrUn	60940114	60941088	975	SAT1_CF	blue	na
chrUn	60941100	60942299	1200	SAT1_CF	blue	na
chrUn	60942352	60942901	550	SAT1_CF	blue	na
chrUn	60942924	60944465	1542	SAT1_CF	blue	na
chrUn	60958952	60960390	1439	CarSat1	red	na
chrUn	60960411	60961191	781	CarSat1	red	na
chrUn	60961205	60961648	444	CarSat1	red	na
chrUn	60961651	60962038	388	SAT1_CF	blue	na
chrUn	60962050	60962666	617	SAT1_CF	blue	na
chrUn	60962661	60963379	719	CarSat1	red	na
chrUn	60963410	60963832	423	CarSat1	red	na
chrUn	60965153	60967992	2840	CarSat1	red	na
chrUn	60968235	60970483	2249	CarSat1	red	na
chrUn	60970513	60972000	1488	CarSat1	red	na
chrUn	60972210	60972684	475	CarSat1	red	na

chrUn	60972717	60973504	788	CarSat1	red	na
chrUn	60973517	60974786	1270	CarSat1	red	na
chrUn	60974810	60975526	717	CarSat1	red	na
chrUn	60975559	60976494	936	CarSat1	red	na
chrUn	60976524	60978482	1959	CarSat1	red	na
chrUn	60978505	60980072	1568	CarSat1	red	na
chrUn	60980097	60980629	533	CarSat1	red	na
chrUn	60980653	60981918	1266	CarSat1	red	na
chrUn	60984778	60985843	1066	CarSat1	red	na
chrUn	60985873	60986359	487	CarSat1	red	na
chrUn	60986383	60987083	701	CarSat1	red	na
chrUn	60988262	60988978	717	Sat2CF	purple	na
chrUn	60988844	60989006	163	Sat2CF	purple	na
chrUn	61007716	61008872	1157	Sat2CF	purple	na
chrUn	61031794	61032233	440	CarSat1	red	na
chrUn	61076696	61077246	551	Sat2CF	purple	na
chrUn	61092525	61093148	624	Sat2CF	purple	na
chrUn	61092598	61093148	551	Sat2CF	purple	na
chrUn	61092611	61093073	463	Sat2CF	purple	na
chrUn	61093880	61094030	151	Sat2CF	purple	na
chrUn	61093884	61094136	253	Sat2CF	purple	na
chrUn	61110654	61111769	1116	Sat2CF	purple	na
chrUn	61130604	61131486	883	SAT1_CF	blue	na
chrUn	61131504	61132188	685	SAT1_CF	blue	na
chrUn	61132201	61134443	2243	SAT1_CF	blue	na
chrUn	61134483	61134977	495	SAT1_CF	blue	na
chrUn	61134991	61135757	767	SAT1_CF	blue	na
chrUn	61135769	61136444	676	SAT1_CF	blue	na
chrUn	61136492	61138053	1562	SAT1_CF	blue	na
chrUn	61140006	61141518	1513	SAT1_CF	blue	na
chrUn	61141530	61142413	884	SAT1_CF	blue	na
chrUn	61142441	61143415	975	SAT1_CF	blue	na
chrUn	61143433	61146210	2778	SAT1_CF	blue	na
chrUn	61146222	61146824	603	SAT1_CF	blue	na
chrUn	61146844	61146939	96	SAT1_CF	blue	na
chrUn	61146963	61147516	554	SAT1_CF	blue	na
chrUn	61147528	61147614	87	SAT1_CF	blue	na
chrUn	61148903	61149146	244	SAT1_CF	blue	na
chrUn	61149188	61149887	700	SAT1_CF	blue	na
chrUn	61149901	61151030	1130	SAT1_CF	blue	na
chrUn	61151083	61151584	502	SAT1_CF	blue	na
chrUn	61151597	61152317	721	SAT1_CF	blue	na

chrUn	61153326	61154594	1269	CarSat1	red	na
chrUn	61154624	61155336	713	CarSat1	red	na
chrUn	61155366	61156550	1185	CarSat1	red	na
chrUn	61156571	61158016	1446	CarSat1	red	na
chrUn	61158043	61158509	467	CarSat1	red	na
chrUn	61158525	61160623	2099	CarSat1	red	na
chrUn	61160873	61164184	3312	CarSat1	red	na
chrUn	61164214	61166390	2177	CarSat1	red	na
chrUn	61166420	61167127	708	CarSat1	red	na
chrUn	61167157	61167879	723	CarSat1	red	na
chrUn	61167894	61170075	2182	CarSat1	red	na
chrUn	61170105	61170826	722	CarSat1	red	na
chrUn	61170841	61172153	1313	CarSat1	red	na
chrUn	61239988	61240601	614	SAT1_CF	blue	na
chrUn	61240645	61242390	1746	SAT1_CF	blue	na
chrUn	61242432	61244942	2511	SAT1_CF	blue	na
chrUn	61244954	61245632	679	SAT1_CF	blue	na
chrUn	61245675	61246323	649	SAT1_CF	blue	na
chrUn	61276296	61277042	747	Sat2CF	purple	na
chrUn	61276375	61277518	1144	Sat2CF	purple	na
chrUn	61302058	61302932	875	Sat36CF	na	na
chrUn	61323924	61325166	1243	CarSat1	red	na
chrUn	61325193	61325903	711	CarSat1	red	na
chrUn	61325930	61326639	710	CarSat1	red	na
chrUn	61326666	61327377	712	CarSat1	red	na
chrUn	61327404	61328113	710	CarSat1	red	na
chrUn	61328140	61330575	2436	CarSat1	red	na
chrUn	61330591	61332785	2195	CarSat1	red	na
chrUn	61332801	61333419	619	CarSat1	red	na
chrUn	61333833	61334535	703	CarSat1	red	na
chrUn	61334551	61335019	469	CarSat1	red	na
chrUn	61335046	61336008	963	CarSat1	red	na
chrUn	61336024	61336745	722	CarSat1	red	na
chrUn	61336761	61339440	2680	CarSat1	red	na
chrUn	61339467	61340178	712	CarSat1	red	na
chrUn	61340205	61341166	962	CarSat1	red	na
chrUn	61341182	61342693	1512	CarSat1	red	na
chrUn	61379537	61380490	954	Sat2CF	purple	na
chrUn	61379600	61380410	811	Sat2CF	purple	na
chrUn	61404097	61405051	955	CarSat1	red	na
chrUn	61405070	61405923	854	CarSat1	red	na
chrUn	61405951	61407933	1983	CarSat1	red	na

chrUn	61407948	61408310	363	SAT1_CF	blue	na
chrUn	61408381	61409890	1510	SAT1_CF	blue	na
chrUn	61409931	61411068	1138	SAT1_CF	blue	na
chrUn	61411063	61411737	675	CarSat1	red	na
chrUn	61412130	61412367	238	CarSat1	red	na
chrUn	61413401	61413560	160	Carsat2	orange	na
chrUn	61413423	61414603	1181	CarSat1	red	na
chrUn	61414633	61416350	1718	CarSat1	red	na
chrUn	61416371	61419041	2671	CarSat1	red	na
chrUn	61419071	61419779	709	CarSat1	red	na
chrUn	61419809	61420517	709	CarSat1	red	na
chrUn	61420547	61423001	2455	CarSat1	red	na
chrUn	61423022	61423468	447	CarSat1	red	na
chrUn	61423498	61424434	937	CarSat1	red	na
chrUn	61426084	61428994	2911	CarSat1	red	na
chrUn	61429032	61429522	491	CarSat1	red	na
chrUn	61429539	61430808	1270	CarSat1	red	na
chrUn	61430837	61431280	444	CarSat1	red	na
chrUn	61431310	61432289	980	CarSat1	red	na
chrUn	61432310	61433089	780	CarSat1	red	na
chrUn	61474711	61476789	2079	SAT1_CF	blue	na
chrUn	61476863	61477345	483	SAT1_CF	blue	na
chrUn	61477363	61477529	167	SAT1_CF	blue	na
chrUn	61477541	61478214	674	SAT1_CF	blue	na
chrUn	61478257	61479848	1592	SAT1_CF	blue	na
chrUn	61479883	61482408	2526	SAT1_CF	blue	na
chrUn	61482448	61484375	1928	SAT1_CF	blue	na
chrUn	61484400	61485268	869	SAT1_CF	blue	na
chrUn	61485313	61485709	397	SAT1_CF	blue	na
chrUn	61485742	61486283	542	SAT1_CF	blue	na
chrUn	61486326	61488362	2037	SAT1_CF	blue	na
chrUn	61488402	61488902	501	SAT1_CF	blue	na
chrUn	61488915	61489825	911	SAT1_CF	blue	na
chrUn	61489839	61491400	1562	SAT1_CF	blue	na
chrUn	61491418	61492797	1380	SAT1_CF	blue	na
chrUn	61492809	61492933	125	SAT1_CF	blue	na
chrUn	61493943	61495135	1193	CarSat1	red	na
chrUn	61495151	61501026	5876	CarSat1	red	na
chrUn	61501042	61502743	1702	CarSat1	red	na
chrUn	61502770	61504750	1981	CarSat1	red	na
chrUn	61507597	61508644	1048	CarSat1	red	na
chrUn	61508671	61509140	470	CarSat1	red	na

chrUn	61509156	61510387	1232	CarSat1	red	na
chrUn	61510417	61511336	920	CarSat1	red	na
chrUn	61511369	61512332	964	CarSat1	red	na
chrUn	61512359	61514075	1717	CarSat1	red	na
chrUn	61514105	61514972	868	CarSat1	red	na
chrUn	61515979	61516440	462	SAT1_CF	blue	na
chrUn	61516483	61517158	676	SAT1_CF	blue	na
chrUn	61517170	61517774	605	SAT1_CF	blue	na
chrUn	61517786	61518398	613	SAT1_CF	blue	na
chrUn	61518411	61519090	680	SAT1_CF	blue	na
chrUn	61519107	61519605	499	SAT1_CF	blue	na
chrUn	61519647	61520321	675	SAT1_CF	blue	na
chrUn	61520349	61520850	502	SAT1_CF	blue	na
chrUn	61520863	61521257	395	SAT1_CF	blue	na
chrUn	61521301	61522041	741	SAT1_CF	blue	na
chrUn	61522626	61522779	154	SAT1_CF	blue	na
chrUn	61522836	61523355	520	SAT1_CF	blue	na
chrUn	61523451	61523587	137	SAT1_CF	blue	na
chrUn	61523602	61524127	526	SAT1_CF	blue	na
chrUn	61524157	61524312	156	SAT1_CF	blue	na
chrUn	61524329	61524776	448	SAT1_CF	blue	na
chrUn	61524800	61525428	629	SAT1_CF	blue	na
chrUn	61525452	61525558	107	SAT1_CF	blue	na
chrUn	61525570	61526072	503	SAT1_CF	blue	na
chrUn	61526159	61528848	2690	SAT1_CF	blue	na
chrUn	61528860	61529379	520	SAT1_CF	blue	na
chrUn	61529394	61529474	81	SAT1_CF	blue	na
chrUn	61529487	61530170	684	SAT1_CF	blue	na
chrUn	61530195	61530347	153	SAT1_CF	blue	na
chrUn	61530371	61530868	498	SAT1_CF	blue	na
chrUn	61530910	61531869	960	SAT1_CF	blue	na
chrUn	61532263	61532743	481	SAT1_CF	blue	na
chrUn	61532786	61533330	545	SAT1_CF	blue	na
chrUn	61533387	61534051	665	SAT1_CF	blue	na
chrUn	61534108	61534627	520	SAT1_CF	blue	na
chrUn	61534745	61535077	333	SAT1_CF	blue	na
chrUn	61580429	61581086	658	Sat29CF	na	na
chrUn	61605454	61605529	76	Sat2CF	purple	na
chrUn	61609322	61610364	1043	CarSat1	red	na
chrUn	61610391	61611350	960	CarSat1	red	na
chrUn	61611367	61612301	935	CarSat1	red	na
chrUn	61612316	61612443	128	Carsat2	orange	na

chrUn	61612331	61614293	1963	CarSat1	red	na
chrUn	61614310	61615030	721	CarSat1	red	na
chrUn	61615055	61616252	1198	CarSat1	red	na
chrUn	61616279	61617454	1176	CarSat1	red	na
chrUn	61617484	61618982	1499	CarSat1	red	na
chrUn	61619308	61620406	1099	CarSat1	red	na
chrUn	61620421	61620548	128	Carsat2	orange	na
chrUn	61620436	61621144	709	CarSat1	red	na
chrUn	61621174	61622395	1222	CarSat1	red	na
chrUn	61622420	61624093	1674	CarSat1	red	na
chrUn	61624108	61624235	128	Carsat2	orange	na
chrUn	61624123	61625342	1220	CarSat1	red	na
chrUn	61625367	61626303	937	CarSat1	red	na
chrUn	61626333	61627311	979	CarSat1	red	na
chrUn	61627338	61627726	389	CarSat1	red	na
chrUn	61643307	61643931	625	CarSat1	red	na
chrUn	61644032	61645622	1591	CarSat1	red	na
chrUn	61645648	61647594	1947	CarSat1	red	na
chrUn	61647596	61647815	220	SAT1_CF	blue	na
chrUn	61648836	61650043	1208	CarSat1	red	na
chrUn	61650070	61651518	1449	CarSat1	red	na
chrUn	61651545	61652721	1177	CarSat1	red	na
chrUn	61652751	61653457	707	CarSat1	red	na
chrUn	61653487	61654199	713	CarSat1	red	na
chrUn	61654214	61654341	128	Carsat2	orange	na
chrUn	61654229	61655669	1441	CarSat1	red	na
chrUn	61655684	61655810	127	Carsat2	orange	na
chrUn	61655699	61656410	712	CarSat1	red	na
chrUn	61656425	61656552	128	Carsat2	orange	na
chrUn	61656440	61659137	2698	CarSat1	red	na
chrUn	61659170	61660105	936	CarSat1	red	na
chrUn	61660135	61660845	711	CarSat1	red	na
chrUn	61660860	61660986	127	Carsat2	orange	na
chrUn	61660875	61661586	712	CarSat1	red	na
chrUn	61661616	61662825	1210	CarSat1	red	na
chrUn	61664046	61665850	1805	CarSat1	red	na
chrUn	61665880	61666588	709	CarSat1	red	na
chrUn	61666618	61667299	682	CarSat1	red	na
chrUn	61693425	61699130	5706	CarSat1	red	na
chrUn	61699319	61699520	202	CarSat1	red	na
chrUn	61699556	61700993	1438	CarSat1	red	na
chrUn	61701020	61702196	1177	CarSat1	red	na

chrUn	61702226	61702287	62	CarSat1	red	na
chrUn	61702475	61703662	1188	CarSat1	red	na
chrUn	61703678	61704147	470	CarSat1	red	na
chrUn	61704174	61705616	1443	CarSat1	red	na
chrUn	61705643	61708561	2919	CarSat1	red	na
chrUn	61708588	61709028	441	CarSat1	red	na
chrUn	61709058	61709766	709	CarSat1	red	na
chrUn	61709796	61710280	485	CarSat1	red	na
chrUn	61710304	61711383	1080	CarSat1	red	na
chrUn	61712440	61713314	875	CarSat1	red	na
chrUn	61713335	61713884	550	CarSat1	red	na
chrUn	61715380	61715910	531	CarSat1	red	na
chrUn	61715931	61716645	715	CarSat1	red	na
chrUn	61716666	61717381	716	CarSat1	red	na
chrUn	61717402	61718116	715	CarSat1	red	na
chrUn	61718137	61718851	715	CarSat1	red	na
chrUn	61718872	61719586	715	CarSat1	red	na
chrUn	61719607	61720321	715	CarSat1	red	na
chrUn	61720342	61721056	715	CarSat1	red	na
chrUn	61721077	61721791	715	CarSat1	red	na
chrUn	61721812	61722526	715	CarSat1	red	na
chrUn	61722547	61724095	1549	CarSat1	red	na
chrUn	61733530	61734025	496	CarSat1	red	na
chrUn	61734230	61734778	549	CarSat1	red	na
chrUn	61737894	61738354	461	CarSat1	red	na
chrUn	61738381	61739562	1182	CarSat1	red	na
chrUn	61739592	61740076	485	CarSat1	red	na
chrUn	61740100	61742282	2183	CarSat1	red	na
chrUn	61742306	61743023	718	CarSat1	red	na
chrUn	61743047	61745456	2410	CarSat1	red	na
chrUn	61745486	61746933	1448	CarSat1	red	na
chrUn	61746948	61747072	125	Carsat2	orange	na
chrUn	61746963	61749143	2181	CarSat1	red	na
chrUn	61749173	61751356	2184	CarSat1	red	na
chrUn	61751386	61752094	709	CarSat1	red	na
chrUn	61752109	61752235	127	Carsat2	orange	na
chrUn	61752124	61753349	1226	CarSat1	red	na
chrUn	61753373	61754090	718	CarSat1	red	na
chrUn	61754114	61755058	945	CarSat1	red	na
chrUn	61755091	61755793	703	CarSat1	red	na
chrUn	61756817	61757078	262	SAT1_CF	blue	na
chrUn	61757089	61758214	1126	SAT1_CF	blue	na

chrUn	61760001	61760536	536	SAT1_CF	blue	na
chrUn	61760549	61761068	520	SAT1_CF	blue	na
chrUn	61761087	61761238	152	SAT1_CF	blue	na
chrUn	61761278	61761789	512	SAT1_CF	blue	na
chrUn	61763277	61763854	578	SAT1_CF	blue	na
chrUn	61763876	61765440	1565	SAT1_CF	blue	na
chrUn	61765468	61765566	99	SAT1_CF	blue	na
chrUn	61765675	61766426	752	SAT1_CF	blue	na
chrUn	61766443	61767057	615	SAT1_CF	blue	na
chrUn	61767886	61769527	1642	SAT1_CF	blue	na
chrUn	61769545	61770065	521	SAT1_CF	blue	na
chrUn	61770086	61772049	1964	SAT1_CF	blue	na
chrUn	61772092	61772758	667	SAT1_CF	blue	na
chrUn	61772781	61773461	681	SAT1_CF	blue	na
chrUn	61773481	61773975	495	SAT1_CF	blue	na
chrUn	61774015	61774567	553	SAT1_CF	blue	na
chrUn	61774585	61775121	537	CarSat1	red	na
chrUn	61775161	61776802	1642	CarSat1	red	na
chrUn	61780955	61781647	693	CarSat1	red	na
chrUn	61781659	61783001	1343	CarSat1	red	na
chrUn	61798658	61799741	1084	Sat2CF	purple	na
chrUn	61814574	61815830	1257	CarSat1	red	na
chrUn	61815860	61817308	1449	CarSat1	red	na
chrUn	61817338	61820259	2922	CarSat1	red	na
chrUn	61820289	61822249	1961	CarSat1	red	na
chrUn	61822273	61824743	2471	CarSat1	red	na
chrUn	61824758	61824884	127	Carsat2	orange	na
chrUn	61824773	61826217	1445	CarSat1	red	na
chrUn	61826248	61829911	3664	CarSat1	red	na
chrUn	61829941	61830427	487	CarSat1	red	na
chrUn	61830451	61831387	937	CarSat1	red	na
chrUn	61831417	61832124	708	CarSat1	red	na
chrUn	61832139	61832264	126	Carsat2	orange	na
chrUn	61832154	61832988	835	CarSat1	red	na
chrUn	61833999	61834853	855	CarSat1	red	na
chrUn	61834869	61837058	2190	CarSat1	red	na
chrUn	61837074	61840731	3658	CarSat1	red	na
chrUn	61840765	61841477	713	CarSat1	red	na
chrUn	61841494	61844199	2706	CarSat1	red	na
chrUn	61844241	61846132	1892	CarSat1	red	na
chrUn	61846159	61848347	2189	CarSat1	red	na
chrUn	61848374	61849351	978	CarSat1	red	na

chrUn	61849381	61850087	707	CarSat1	red	na
chrUn	61850117	61851293	1177	CarSat1	red	na
chrUn	61851320	61851867	548	CarSat1	red	na
chrUn	61880881	61881735	855	SAT1_CF	blue	na
chrUn	61881762	61882434	673	SAT1_CF	blue	na
chrUn	61882446	61883129	684	SAT1_CF	blue	na
chrUn	61883142	61883642	501	SAT1_CF	blue	na
chrUn	61883655	61884143	489	SAT1_CF	blue	na
chrUn	61884187	61886073	1887	SAT1_CF	blue	na
chrUn	61886116	61886282	167	SAT1_CF	blue	na
chrUn	61886301	61887014	714	SAT1_CF	blue	na
chrUn	61887026	61887610	585	SAT1_CF	blue	na
chrUn	61887653	61888765	1113	SAT1_CF	blue	na
chrUn	61888808	61890163	1356	SAT1_CF	blue	na
chrUn	61890190	61890690	501	SAT1_CF	blue	na
chrUn	61890756	61890892	137	SAT1_CF	blue	na
chrUn	61890920	61892290	1371	SAT1_CF	blue	na
chrUn	61892308	61892632	325	SAT1_CF	blue	na
chrUn	61892675	61894311	1637	SAT1_CF	blue	na
chrUn	61894356	61894510	155	SAT1_CF	blue	na
chrUn	61894527	61895665	1139	SAT1_CF	blue	na
chrUn	61895737	61896329	593	SAT1_CF	blue	na
chrUn	61896360	61897587	1228	SAT1_CF	blue	na
chrUn	61897615	61898115	501	SAT1_CF	blue	na
chrUn	61898202	61898651	450	SAT1_CF	blue	na
chrUn	61899711	61901214	1504	CarSat1	red	na
chrUn	61901239	61901961	723	CarSat1	red	na
chrUn	61901977	61902939	963	CarSat1	red	na
chrUn	61902966	61903676	711	CarSat1	red	na
chrUn	61903703	61904163	461	CarSat1	red	na
chrUn	61904188	61905887	1700	CarSat1	red	na
chrUn	61905914	61906624	711	CarSat1	red	na
chrUn	61906651	61907111	461	CarSat1	red	na
chrUn	61907136	61907847	712	CarSat1	red	na
chrUn	61907872	61908641	770	CarSat1	red	na
chrUn	61909502	61910625	1124	CarSat1	red	na
chrUn	61910650	61911361	712	CarSat1	red	na
chrUn	61911386	61913826	2441	CarSat1	red	na
chrUn	61913853	61915819	1967	CarSat1	red	na
chrUn	61915844	61916805	962	CarSat1	red	na
chrUn	61916832	61918375	1544	CarSat1	red	na
chrUn	61919390	61920062	673	CarSat1	red	na

chrUn	61920099	61921323	1225	CarSat1	red	na
chrUn	61921348	61926215	4868	CarSat1	red	na
chrUn	61926248	61927856	1609	CarSat1	red	na
chrUn	61928031	61928998	968	CarSat1	red	na
chrUn	61929301	61930262	962	CarSat1	red	na
chrUn	61930278	61931214	937	CarSat1	red	na
chrUn	61931244	61932238	995	CarSat1	red	na
chrUn	61932265	61932972	708	CarSat1	red	na
chrUn	61932999	61933439	441	CarSat1	red	na
chrUn	61933469	61935921	2453	CarSat1	red	na
chrUn	61935948	61936656	709	CarSat1	red	na
chrUn	61936685	61937505	821	CarSat1	red	na
chrUn	61961354	61961928	575	CarSat1	red	na
chrUn	61961958	61962681	724	CarSat1	red	na
chrUn	61962696	61963625	930	CarSat1	red	na
chrUn	61963651	61964882	1232	CarSat1	red	na
chrUn	61964912	61965635	724	CarSat1	red	na
chrUn	61965650	61966361	712	CarSat1	red	na
chrUn	61966391	61967846	1456	CarSat1	red	na
chrUn	61967861	61969322	1462	CarSat1	red	na
chrUn	61969337	61970795	1459	CarSat1	red	na
chrUn	61970810	61972075	1266	CarSat1	red	na
chrUn	61972227	61972531	305	CarSat1	red	na
chrUn	61972589	61973291	703	CarSat1	red	na
chrUn	61973306	61974753	1448	CarSat1	red	na
chrUn	61974783	61975474	692	CarSat1	red	na
chrUn	61975517	61976239	723	CarSat1	red	na
chrUn	61976254	61976974	721	CarSat1	red	na
chrUn	61976990	61977717	728	CarSat1	red	na
chrUn	61977732	61978439	708	CarSat1	red	na
chrUn	61978476	61979110	635	CarSat1	red	na
chrUn	62020148	62021923	1776	CarSat1	red	na
chrUn	62021938	62026069	4132	CarSat1	red	na
chrUn	62026096	62026802	707	CarSat1	red	na
chrUn	62026833	62028277	1445	CarSat1	red	na
chrUn	62028304	62029751	1448	CarSat1	red	na
chrUn	62029782	62030489	708	CarSat1	red	na
chrUn	62030516	62031226	711	CarSat1	red	na
chrUn	62031253	62032701	1449	CarSat1	red	na
chrUn	62032732	62033706	975	CarSat1	red	na
chrUn	62033751	62034921	1171	CarSat1	red	na
chrUn	62034952	62035657	706	CarSat1	red	na

chrUn	62035684	62036394	711	CarSat1	red	na
chrUn	62036421	62037136	716	CarSat1	red	na
chrUn	62037162	62037869	708	CarSat1	red	na
chrUn	62080715	62080839	125	Sat2CF	purple	na
chrUn	62080731	62081529	799	Sat2CF	purple	na
chrUn	62082953	62083808	856	CarSat1	red	na
chrUn	62083838	62084546	709	CarSat1	red	na
chrUn	62084574	62085097	524	CarSat1	red	na
chrUn	62085116	62085888	773	CarSat1	red	na
chrUn	62085915	62086625	711	CarSat1	red	na
chrUn	62086652	62089102	2451	CarSat1	red	na
chrUn	62089145	62089833	689	CarSat1	red	na
chrUn	62089863	62091775	1913	CarSat1	red	na
chrUn	62091802	62092511	710	CarSat1	red	na
chrUn	62092537	62093006	470	CarSat1	red	na
chrUn	62093022	62093987	966	CarSat1	red	na
chrUn	62094014	62096210	2197	CarSat1	red	na
chrUn	62096237	62097033	797	CarSat1	red	na
chrUn	62097212	62100534	3323	CarSat1	red	na
chrUn	62141114	62142772	1659	CarSat1	red	na
chrUn	62142797	62143266	470	CarSat1	red	na
chrUn	62143293	62144468	1176	CarSat1	red	na
chrUn	62144498	62145477	980	CarSat1	red	na
chrUn	62145498	62146672	1175	CarSat1	red	na
chrUn	62146709	62147193	485	CarSat1	red	na
chrUn	62147218	62148155	938	CarSat1	red	na
chrUn	62148170	62148297	128	Carsat2	orange	na
chrUn	62148185	62149162	978	CarSat1	red	na
chrUn	62149183	62151697	2515	CarSat1	red	na
chrUn	62152599	62154327	1729	CarSat1	red	na
chrUn	62154352	62155558	1207	CarSat1	red	na
chrUn	62155579	62158014	2436	CarSat1	red	na
chrUn	62158039	62159556	1518	CarSat1	red	na
chrUn	62185092	62186314	1223	CarSat1	red	na
chrUn	62186345	62187057	713	CarSat1	red	na
chrUn	62187087	62188250	1164	CarSat1	red	na
chrUn	62188353	62188527	175	CarSat1	red	na
chrUn	62188604	62189769	1166	CarSat1	red	na
chrUn	62189802	62190507	706	CarSat1	red	na
chrUn	62190540	62191777	1238	CarSat1	red	na
chrUn	62191792	62192738	947	CarSat1	red	na
chrUn	62192750	62193979	1230	CarSat1	red	na

chrUn	62194009	62195670	1662	CarSat1	red	na
chrUn	62195703	62197143	1441	CarSat1	red	na
chrUn	62197176	62200088	2913	CarSat1	red	na
chrUn	62200121	62201364	1244	CarSat1	red	na
chrUn	62201379	62202575	1197	CarSat1	red	na
chrUn	62214144	62215095	952	CarSat1	red	na
chrUn	62215114	62215967	854	CarSat1	red	na
chrUn	62215986	62216765	780	CarSat1	red	na
chrUn	62216867	62218820	1954	CarSat1	red	na
chrUn	62218825	62219584	760	SAT1_CF	blue	na
chrUn	62219579	62220302	724	CarSat1	red	na
chrUn	62220334	62220759	426	CarSat1	red	na
chrUn	62222092	62225210	3119	SAT1_CF	blue	na
chrUn	62225253	62225751	499	SAT1_CF	blue	na
chrUn	62225794	62226587	794	SAT1_CF	blue	na
chrUn	62226769	62226950	182	SAT1_CF	blue	na
chrUn	62226990	62227489	500	SAT1_CF	blue	na
chrUn	62227503	62228115	613	SAT1_CF	blue	na
chrUn	62228161	62228486	326	SAT1_CF	blue	na
chrUn	62228547	62229888	1342	SAT1_CF	blue	na
chrUn	62229902	62230014	113	SAT1_CF	blue	na
chrUn	62230035	62230589	555	SAT1_CF	blue	na
chrUn	62230601	62231371	771	SAT1_CF	blue	na
chrUn	62231391	62231526	136	SAT1_CF	blue	na
chrUn	62231787	62232168	382	SAT1_CF	blue	na
chrUn	62232181	62232864	684	SAT1_CF	blue	na
chrUn	62232876	62234540	1665	SAT1_CF	blue	na
chrUn	62234566	62235141	576	SAT1_CF	blue	na
chrUn	62235171	62235680	510	SAT1_CF	blue	na
chrUn	62235718	62236588	871	SAT1_CF	blue	na
chrUn	62236600	62238060	1461	SAT1_CF	blue	na
chrUn	62238091	62238597	507	SAT1_CF	blue	na
chrUn	62238630	62238787	158	SAT1_CF	blue	na
chrUn	62238832	62239333	502	SAT1_CF	blue	na
chrUn	62239345	62239831	487	SAT1_CF	blue	na
chrUn	62254124	62255075	952	CarSat1	red	na
chrUn	62255233	62255945	713	CarSat1	red	na
chrUn	62255972	62256835	864	CarSat1	red	na
chrUn	62256856	62257641	786	CarSat1	red	na
chrUn	62257853	62258306	454	CarSat1	red	na
chrUn	62296250	62296818	569	CarSat1	red	na
chrUn	62296833	62296959	127	Carsat2	orange	na

chrUn	62296848	62298811	1964	CarSat1	red	na
chrUn	62298844	62300518	1675	CarSat1	red	na
chrUn	62300548	62304211	3664	CarSat1	red	na
chrUn	62304241	62305684	1444	CarSat1	red	na
chrUn	62305699	62305826	128	Carsat2	orange	na
chrUn	62305714	62307158	1445	CarSat1	red	na
chrUn	62307173	62307299	127	Carsat2	orange	na
chrUn	62307188	62307893	706	CarSat1	red	na
chrUn	62307909	62308034	126	Carsat2	orange	na
chrUn	62307923	62308628	706	CarSat1	red	na
chrUn	62308643	62308770	128	Carsat2	orange	na
chrUn	62308658	62309879	1222	CarSat1	red	na
chrUn	62309912	62311583	1672	CarSat1	red	na
chrUn	62311598	62311704	107	Carsat2	orange	na
chrUn	62311613	62312320	708	CarSat1	red	na
chrUn	62312350	62313057	708	CarSat1	red	na
chrUn	62313072	62313198	127	Carsat2	orange	na
chrUn	62313087	62313844	758	CarSat1	red	na
chrUn	62343738	62344063	326	SAT1_CF	blue	na
chrUn	62344141	62344648	508	SAT1_CF	blue	na
chrUn	62344661	62345016	356	SAT1_CF	blue	na
chrUn	62345103	62345605	503	SAT1_CF	blue	na
chrUn	62345618	62347405	1788	SAT1_CF	blue	na
chrUn	62347439	62348371	933	SAT1_CF	blue	na
chrUn	62348444	62348964	521	SAT1_CF	blue	na
chrUn	62348995	62349257	263	SAT1_CF	blue	na
chrUn	62350805	62350902	98	SAT1_CF	blue	na
chrUn	62350947	62351447	501	SAT1_CF	blue	na
chrUn	62351461	62352220	760	SAT1_CF	blue	na
chrUn	62352232	62353598	1367	SAT1_CF	blue	na
chrUn	62353663	62354821	1159	SAT1_CF	blue	na
chrUn	62354832	62355443	612	SAT1_CF	blue	na
chrUn	62355466	62356132	667	SAT1_CF	blue	na
chrUn	62356175	62356701	527	SAT1_CF	blue	na
chrUn	62356723	62358217	1495	SAT1_CF	blue	na
chrUn	62358257	62358759	503	SAT1_CF	blue	na
chrUn	62358772	62360114	1343	SAT1_CF	blue	na
chrUn	62360130	62360278	149	SAT1_CF	blue	na
chrUn	62360292	62361728	1437	SAT1_CF	blue	na
chrUn	62361740	62362393	654	SAT1_CF	blue	na
chrUn	62362477	62362616	140	SAT1_CF	blue	na
chrUn	62363627	62366357	2731	CarSat1	red	na

chrUn	62366384	62367586	1203	CarSat1	red	na
chrUn	62367619	62368839	1221	CarSat1	red	na
chrUn	62368875	62369575	701	CarSat1	red	na
chrUn	62369605	62371068	1464	CarSat1	red	na
chrUn	62371083	62374755	3673	CarSat1	red	na
chrUn	62374770	62379925	5156	CarSat1	red	na
chrUn	62379940	62380998	1059	CarSat1	red	na
chrUn	62381999	62383134	1136	CarSat1	red	na
chrUn	62383161	62383872	712	CarSat1	red	na
chrUn	62383899	62384610	712	CarSat1	red	na
chrUn	62384637	62385347	711	CarSat1	red	na
chrUn	62385368	62386075	708	CarSat1	red	na
chrUn	62386102	62386813	712	CarSat1	red	na
chrUn	62386840	62387803	964	CarSat1	red	na
chrUn	62387819	62388410	592	CarSat1	red	na
chrUn	62388702	62389409	708	CarSat1	red	na
chrUn	62389436	62390399	964	CarSat1	red	na
chrUn	62390415	62391136	722	CarSat1	red	na
chrUn	62391152	62391874	723	CarSat1	red	na
chrUn	62391890	62392610	721	CarSat1	red	na
chrUn	62392626	62394568	1943	CarSat1	red	na
chrUn	62394595	62396294	1700	CarSat1	red	na
chrUn	62396310	62397030	721	CarSat1	red	na
chrUn	62397046	62398505	1460	CarSat1	red	na
chrUn	62398521	62399654	1134	CarSat1	red	na
chrUn	62400665	62401360	696	CarSat1	red	na
chrUn	62401387	62402227	841	CarSat1	red	na
chrUn	62403704	62404290	587	CarSat1	red	na
chrUn	62404317	62405195	879	CarSat1	red	na
chrUn	62406175	62407246	1072	CarSat1	red	na
chrUn	62407273	62407742	470	CarSat1	red	na
chrUn	62407758	62408716	959	CarSat1	red	na
chrUn	62408743	62409722	980	CarSat1	red	na
chrUn	62410391	62411378	988	CarSat1	red	na
chrUn	62411394	62412355	962	CarSat1	red	na
chrUn	62412382	62413631	1250	CarSat1	red	na
chrUn	62413647	62414364	718	CarSat1	red	na
chrUn	62414380	62414998	619	CarSat1	red	na
chrUn	62415017	62415745	729	CarSat1	red	na
chrUn	62415775	62416873	1099	CarSat1	red	na
chrUn	62417066	62417779	714	CarSat1	red	na
chrUn	62423098	62423260	163	Sat2CF	purple	na

chrUn	62423119	62423469	351	Sat2CF	purple	na
chrUn	62423166	62423466	301	Sat2CF	purple	na
chrUn	62433929	62436165	2237	CarSat1	red	na
chrUn	62436186	62437782	1597	CarSat1	red	na
chrUn	62437803	62439312	1510	CarSat1	red	na
chrUn	62439412	62440895	1484	CarSat1	red	na
chrUn	62440897	62441798	902	SAT1_CF	blue	na
chrUn	62446641	62447594	954	CarSat1	red	na
chrUn	62447745	62449183	1439	CarSat1	red	na
chrUn	62449343	62449986	644	CarSat1	red	na
chrUn	62450050	62450956	907	CarSat1	red	na
chrUn	62450977	62452019	1043	CarSat1	red	na
chrUn	62452056	62453963	1908	CarSat1	red	na
chrUn	62453990	62455055	1066	CarSat1	red	na
chrUn	62455136	62458260	3125	CarSat1	red	na
chrUn	62458439	62458921	483	CarSat1	red	na
chrUn	62458946	62460134	1189	CarSat1	red	na
chrUn	62465419	62466330	912	CarSat1	red	na
chrUn	62466351	62467935	1585	CarSat1	red	na
chrUn	62467956	62468811	856	CarSat1	red	na
chrUn	62468832	62470422	1591	CarSat1	red	na
chrUn	62470443	62472034	1592	CarSat1	red	na
chrUn	62497598	62498210	613	SAT1_CF	blue	na
chrUn	62498228	62498887	660	SAT1_CF	blue	na
chrUn	62498939	62499220	282	SAT1_CF	blue	na
chrUn	62499400	62499676	277	SAT1_CF	blue	na
chrUn	62499689	62500805	1117	SAT1_CF	blue	na
chrUn	62500823	62501514	692	SAT1_CF	blue	na
chrUn	62501561	62502080	520	SAT1_CF	blue	na
chrUn	62502093	62502768	676	SAT1_CF	blue	na
chrUn	62502811	62503531	721	SAT1_CF	blue	na
chrUn	62503582	62504117	536	SAT1_CF	blue	na
chrUn	62504139	62504657	519	SAT1_CF	blue	na
chrUn	62504669	62505985	1317	SAT1_CF	blue	na
chrUn	62505998	62506830	833	SAT1_CF	blue	na
chrUn	62506843	62507546	704	SAT1_CF	blue	na
chrUn	62507558	62509145	1588	SAT1_CF	blue	na
chrUn	62509176	62510097	922	SAT1_CF	blue	na
chrUn	62510137	62511153	1017	SAT1_CF	blue	na
chrUn	62511201	62512481	1281	SAT1_CF	blue	na
chrUn	62512569	62513070	502	SAT1_CF	blue	na
chrUn	62513083	62514269	1187	SAT1_CF	blue	na

chrUn	62514283	62514789	507	SAT1_CF	blue	na
chrUn	62515811	62517077	1267	CarSat1	red	na
chrUn	62517104	62517814	711	CarSat1	red	na
chrUn	62517841	62518550	710	CarSat1	red	na
chrUn	62518576	62519773	1198	CarSat1	red	na
chrUn	62519798	62521496	1699	CarSat1	red	na
chrUn	62521523	62521990	468	CarSat1	red	na
chrUn	62522006	62522966	961	CarSat1	red	na
chrUn	62522993	62524186	1194	CarSat1	red	na
chrUn	62524211	62525911	1701	CarSat1	red	na
chrUn	62525943	62528854	2912	CarSat1	red	na
chrUn	62528881	62529590	710	CarSat1	red	na
chrUn	62529617	62530326	710	CarSat1	red	na
chrUn	62530353	62532970	2618	CarSat1	red	na
chrUn	62533970	62534935	966	CarSat1	red	na
chrUn	62534965	62535900	936	CarSat1	red	na
chrUn	62535916	62537144	1229	CarSat1	red	na
chrUn	62537174	62539088	1915	CarSat1	red	na
chrUn	62539115	62539830	716	CarSat1	red	na
chrUn	62539852	62540562	711	CarSat1	red	na
chrUn	62540589	62541299	711	CarSat1	red	na
chrUn	62541326	62542305	980	CarSat1	red	na
chrUn	62542335	62544250	1916	CarSat1	red	na
chrUn	62544277	62545722	1446	CarSat1	red	na
chrUn	62545749	62546218	470	CarSat1	red	na
chrUn	62546234	62547196	963	CarSat1	red	na
chrUn	62547223	62547921	699	CarSat1	red	na
chrUn	62547948	62548658	711	CarSat1	red	na
chrUn	62548685	62549144	460	CarSat1	red	na
chrUn	62549169	62550132	964	CarSat1	red	na
chrUn	62550159	62551138	980	CarSat1	red	na
chrUn	62552141	62553219	1079	CarSat1	red	na
chrUn	62553250	62554450	1201	CarSat1	red	na
chrUn	62554466	62555428	963	CarSat1	red	na
chrUn	62555455	62556165	711	CarSat1	red	na
chrUn	62556192	62556900	709	CarSat1	red	na
chrUn	62556927	62558134	1208	CarSat1	red	na
chrUn	62558150	62559112	963	CarSat1	red	na
chrUn	62559139	62559849	711	CarSat1	red	na
chrUn	62559876	62561083	1208	CarSat1	red	na
chrUn	62561099	62561821	723	CarSat1	red	na
chrUn	62561837	62562557	721	CarSat1	red	na

chrUn	62562574	62563295	722	CarSat1	red	na
chrUn	62563311	62564032	722	CarSat1	red	na
chrUn	62564048	62565750	1703	CarSat1	red	na
chrUn	62565777	62569303	3527	CarSat1	red	na
chrUn	62570310	62570959	650	SAT1_CF	blue	na
chrUn	62570971	62573317	2347	SAT1_CF	blue	na
chrUn	62573359	62573524	166	SAT1_CF	blue	na
chrUn	62573706	62573891	186	SAT1_CF	blue	na
chrUn	62573908	62574428	521	SAT1_CF	blue	na
chrUn	62574441	62574549	109	SAT1_CF	blue	na
chrUn	62574569	62577650	3082	SAT1_CF	blue	na
chrUn	62577662	62578140	479	SAT1_CF	blue	na
chrUn	62578320	62578544	225	SAT1_CF	blue	na
chrUn	62578556	62580514	1959	SAT1_CF	blue	na
chrUn	62580525	62582201	1677	SAT1_CF	blue	na
chrUn	62582254	62582755	502	SAT1_CF	blue	na
chrUn	62582782	62583526	745	SAT1_CF	blue	na
chrUn	62583538	62584399	862	SAT1_CF	blue	na
chrUn	62584442	62585516	1075	SAT1_CF	blue	na
chrUn	62585559	62586082	524	SAT1_CF	blue	na
chrUn	62586097	62586233	137	SAT1_CF	blue	na
chrUn	62586299	62587433	1135	SAT1_CF	blue	na
chrUn	62588539	62589941	1403	CarSat1	red	na
chrUn	62589968	62591414	1447	CarSat1	red	na
chrUn	62591441	62591911	471	CarSat1	red	na
chrUn	62591927	62592877	951	CarSat1	red	na
chrUn	62592916	62594365	1450	CarSat1	red	na
chrUn	62594392	62595840	1449	CarSat1	red	na
chrUn	62595867	62596577	711	CarSat1	red	na
chrUn	62596604	62597313	710	CarSat1	red	na
chrUn	62597340	62597800	461	CarSat1	red	na
chrUn	62597825	62598787	963	CarSat1	red	na
chrUn	62598814	62599792	979	CarSat1	red	na
chrUn	62599822	62600262	441	CarSat1	red	na
chrUn	62600289	62600749	461	CarSat1	red	na
chrUn	62600774	62601733	960	CarSat1	red	na
chrUn	62601760	62602966	1207	CarSat1	red	na
chrUn	62602982	62604438	1457	CarSat1	red	na
chrUn	62604453	62605550	1098	CarSat1	red	na
chrUn	62620894	62621967	1074	Sat2CF	purple	na
chrUn	62626289	62626493	205	SAT1_CF	blue	na
chrUn	62626601	62627894	1294	SAT1_CF	blue	na

chrUn	62627907	62628408	502	SAT1_CF	blue	na
chrUn	62628461	62631065	2605	SAT1_CF	blue	na
chrUn	62631134	62631932	799	SAT1_CF	blue	na
chrUn	62631978	62633308	1331	SAT1_CF	blue	na
chrUn	62633321	62633823	503	SAT1_CF	blue	na
chrUn	62633863	62635235	1373	SAT1_CF	blue	na
chrUn	62635259	62636626	1368	SAT1_CF	blue	na
chrUn	62636638	62637085	448	SAT1_CF	blue	na
chrUn	62638091	62638578	488	SAT1_CF	blue	na
chrUn	62638592	62638821	230	SAT1_CF	blue	na
chrUn	62639215	62639326	112	SAT1_CF	blue	na
chrUn	62639348	62639453	106	SAT1_CF	blue	na
chrUn	62639466	62639965	500	SAT1_CF	blue	na
chrUn	62640969	62641960	992	SAT1_CF	blue	na
chrUn	62642401	62644521	2121	SAT1_CF	blue	na
chrUn	62644546	62645566	1021	SAT1_CF	blue	na
chrUn	62646571	62647295	725	CarSat1	red	na
chrUn	62647476	62649640	2165	CarSat1	red	na
chrUn	62649661	62652839	3179	CarSat1	red	na
chrUn	62652869	62655512	2644	CarSat1	red	na
chrUn	62655560	62656511	952	CarSat1	red	na
chrUn	62656541	62659473	2933	CarSat1	red	na
chrUn	62659488	62660931	1444	CarSat1	red	na
chrUn	62660961	62661682	722	CarSat1	red	na
chrUn	62661697	62662419	723	CarSat1	red	na
chrUn	62662434	62663140	707	CarSat1	red	na
chrUn	62663170	62663615	446	CarSat1	red	na
chrUn	62669026	62669903	878	CarSat1	red	na
chrUn	62670337	62671062	726	CarSat1	red	na
chrUn	62671083	62671852	770	CarSat1	red	na
chrUn	62671878	62672673	796	CarSat1	red	na
chrUn	62672694	62673469	776	CarSat1	red	na
chrUn	62673487	62674284	798	CarSat1	red	na
chrUn	62674303	62676266	1964	CarSat1	red	na
chrUn	62676270	62677345	1076	SAT1_CF	blue	na
chrUn	62677387	62678699	1313	SAT1_CF	blue	na
chrUn	62678728	62679860	1133	SAT1_CF	blue	na
chrUn	62679904	62680390	487	SAT1_CF	blue	na
chrUn	62680385	62681109	725	CarSat1	red	na
chrUn	62681141	62681567	427	CarSat1	red	na
chrUn	62719483	62720532	1050	CarSat1	red	na
chrUn	62720553	62721545	993	CarSat1	red	na

chrUn	62721561	62722488	928	CarSat1	red	na
chrUn	62722521	62723743	1223	CarSat1	red	na
chrUn	62723773	62724496	724	CarSat1	red	na
chrUn	62724518	62726924	2407	CarSat1	red	na
chrUn	62726948	62729658	2711	CarSat1	red	na
chrUn	62729673	62732075	2403	CarSat1	red	na
chrUn	62732099	62734290	2192	CarSat1	red	na
chrUn	62734314	62735027	714	CarSat1	red	na
chrUn	62735051	62735536	486	CarSat1	red	na
chrUn	62735566	62736342	777	CarSat1	red	na
chrUn	62737349	62738481	1133	CarSat1	red	na
chrUn	62738505	62739740	1236	CarSat1	red	na
chrUn	62739755	62741669	1915	CarSat1	red	na
chrUn	62741690	62742160	471	CarSat1	red	na
chrUn	62742184	62743913	1730	CarSat1	red	na
chrUn	62744136	62744830	695	CarSat1	red	na
chrUn	62744857	62745326	470	CarSat1	red	na
chrUn	62745342	62746579	1238	CarSat1	red	na
chrUn	62746609	62747321	713	CarSat1	red	na
chrUn	62747351	62748799	1449	CarSat1	red	na
chrUn	62748829	62750502	1674	CarSat1	red	na
chrUn	62750526	62751026	501	CarSat1	red	na
chrUn	62751041	62751768	728	CarSat1	red	na
chrUn	62751783	62754136	2354	CarSat1	red	na
chrUn	62772954	62773595	642	CarSat1	red	na
chrUn	62773622	62774316	695	CarSat1	red	na
chrUn	62774359	62775068	710	CarSat1	red	na
chrUn	62775095	62775805	711	CarSat1	red	na
chrUn	62775832	62776525	694	CarSat1	red	na
chrUn	62776567	62778013	1447	CarSat1	red	na
chrUn	62778040	62778749	710	CarSat1	red	na
chrUn	62778776	62779486	711	CarSat1	red	na
chrUn	62779513	62780804	1292	CarSat1	red	na
chrUn	62784354	62785041	688	CarSat1	red	na
chrUn	62785068	62786513	1446	CarSat1	red	na
chrUn	62786540	62786979	440	CarSat1	red	na
chrUn	62786995	62787117	123	Carsat2	orange	na
chrUn	62787009	62787983	975	CarSat1	red	na
chrUn	62788010	62789710	1701	CarSat1	red	na
chrUn	62789752	62792060	2309	CarSat1	red	na
chrUn	62792085	62793144	1060	CarSat1	red	na
chrUn	62794162	62800381	6220	CarSat1	red	na

chrUn	62800402	62807504	7103	CarSat1	red	na
chrUn	62807521	62808228	708	CarSat1	red	na
chrUn	62808253	62808963	711	CarSat1	red	na
chrUn	62808988	62810897	1910	CarSat1	red	na
chrUn	62818352	62819081	730	Sat79CF	na	na
chrUn	62818390	62819081	692	Sat79CF	na	na
chrUn	62818422	62819168	747	Sat18CF	na	na
chrUn	62818549	62819168	620	Sat18CF	na	na
chrUn	62832932	62834976	2045	SAT1_CF	blue	na
chrUn	62834987	62835306	320	SAT1_CF	blue	na
chrUn	62835379	62836146	768	SAT1_CF	blue	na
chrUn	62836164	62836832	669	SAT1_CF	blue	na
chrUn	62836875	62837893	1019	SAT1_CF	blue	na
chrUn	62837990	62838538	549	SAT1_CF	blue	na
chrUn	62838617	62839157	541	SAT1_CF	blue	na
chrUn	62839537	62841569	2033	SAT1_CF	blue	na
chrUn	62841582	62841922	341	SAT1_CF	blue	na
chrUn	62842058	62842955	898	SAT1_CF	blue	na
chrUn	62842982	62843161	180	SAT1_CF	blue	na
chrUn	62844156	62845392	1237	SAT1_CF	blue	na
chrUn	62846794	62847061	268	SAT1_CF	blue	na
chrUn	62847074	62847757	684	SAT1_CF	blue	na
chrUn	62847769	62848452	684	SAT1_CF	blue	na
chrUn	62848464	62849121	658	SAT1_CF	blue	na
chrUn	62849165	62850234	1070	SAT1_CF	blue	na
chrUn	62850257	62851510	1254	SAT1_CF	blue	na
chrUn	62851523	62851732	210	SAT1_CF	blue	na
chrUn	62851746	62852292	547	SAT1_CF	blue	na
chrUn	62852303	62852415	113	SAT1_CF	blue	na
chrUn	62853442	62854976	1535	CarSat1	red	na
chrUn	62855006	62857183	2178	CarSat1	red	na
chrUn	62857213	62857919	707	CarSat1	red	na
chrUn	62857949	62859458	1510	CarSat1	red	na
chrUn	62860719	62860803	85	CarSat1	red	na
chrUn	62860985	62862620	1636	CarSat1	red	na
chrUn	62862645	62863357	713	CarSat1	red	na
chrUn	62863382	62864342	961	CarSat1	red	na
chrUn	62864369	62865079	711	CarSat1	red	na
chrUn	62865106	62866083	978	CarSat1	red	na
chrUn	62866113	62869045	2933	CarSat1	red	na
chrUn	62869060	62869787	728	CarSat1	red	na
chrUn	62869802	62871332	1531	CarSat1	red	na

chrUn	62883119	62883839	721	SAT1_CF	blue	na
chrUn	62883882	62885584	1703	SAT1_CF	blue	na
chrUn	62885596	62885913	318	SAT1_CF	blue	na
chrUn	62885926	62887042	1117	SAT1_CF	blue	na
chrUn	62887088	62887581	494	SAT1_CF	blue	na
chrUn	62887567	62888290	724	CarSat1	red	na
chrUn	62888449	62888748	300	CarSat1	red	na
chrUn	62889987	62890093	107	Carsat2	orange	na
chrUn	62890012	62891118	1107	CarSat1	red	na
chrUn	62891133	62891260	128	Carsat2	orange	na
chrUn	62891148	62891634	487	CarSat1	red	na
chrUn	62891667	62892370	704	CarSat1	red	na
chrUn	62892403	62893322	920	CarSat1	red	na
chrUn	62893359	62894068	710	CarSat1	red	na
chrUn	62894083	62894210	128	Carsat2	orange	na
chrUn	62894099	62894585	487	CarSat1	red	na
chrUn	62894609	62896268	1660	CarSat1	red	na
chrUn	62896293	62896420	128	Carsat2	orange	na
chrUn	62896308	62897014	707	CarSat1	red	na
chrUn	62897029	62897155	127	Carsat2	orange	na
chrUn	62897044	62897749	706	CarSat1	red	na
chrUn	62897763	62897889	127	Carsat2	orange	na
chrUn	62897778	62898262	485	CarSat1	red	na
chrUn	62898295	62899226	932	CarSat1	red	na
chrUn	62899256	62899963	708	CarSat1	red	na
chrUn	62899978	62900104	127	Carsat2	orange	na
chrUn	62899993	62900697	705	CarSat1	red	na
chrUn	62900712	62900827	116	Carsat2	orange	na
chrUn	62900727	62901212	486	CarSat1	red	na
chrUn	62901245	62902169	925	CarSat1	red	na
chrUn	62902184	62902310	127	Carsat2	orange	na
chrUn	62902199	62902910	712	CarSat1	red	na
chrUn	62902925	62903052	128	Carsat2	orange	na
chrUn	62902940	62903648	709	CarSat1	red	na
chrUn	62903678	62905641	1964	CarSat1	red	na
chrUn	62905660	62906606	947	CarSat1	red	na
chrUn	62931958	62934068	2111	CarSat1	red	na
chrUn	62934093	62935783	1691	CarSat1	red	na
chrUn	62935810	62937008	1199	CarSat1	red	na
chrUn	62937033	62937526	494	CarSat1	red	na
chrUn	62937563	62938730	1168	CarSat1	red	na
chrUn	62938757	62942175	3419	CarSat1	red	na

chrUn	62942200	62947325	5126	CarSat1	red	na
chrUn	62947350	62948514	1165	CarSat1	red	na
chrUn	62953869	62954616	748	CarSat1	red	na
chrUn	62954645	62956138	1494	CarSat1	red	na
chrUn	62965694	62966009	316	SAT1_CF	blue	na
chrUn	62978407	62978786	380	Sat2CF	purple	na
chrUn	62978461	62979251	791	Sat2CF	purple	na
chrUn	62984629	62985745	1117	CarSat1	red	na
chrUn	62985770	62986473	704	CarSat1	red	na
chrUn	62986498	62987460	963	CarSat1	red	na
chrUn	62987487	62988935	1449	CarSat1	red	na
chrUn	62988962	62989673	712	CarSat1	red	na
chrUn	62989700	62990740	1041	CarSat1	red	na
chrUn	62990987	62993345	2359	CarSat1	red	na
chrUn	62993371	62994616	1246	CarSat1	red	na
chrUn	62994635	62995336	702	CarSat1	red	na
chrUn	62995366	62998512	3147	CarSat1	red	na
chrUn	62998536	62999386	851	CarSat1	red	na
chrUn	62999586	63000285	700	CarSat1	red	na
chrUn	63052061	63052327	267	Sat2CF	purple	na
chrUn	63052062	63052291	230	Sat2CF	purple	na
chrUn	63115323	63117728	2406	CarSat1	red	na
chrUn	63117758	63118462	705	CarSat1	red	na
chrUn	63118492	63119196	705	CarSat1	red	na
chrUn	63119230	63122404	3175	CarSat1	red	na
chrUn	63124241	63125016	776	CarSat1	red	na
chrUn	63125050	63125751	702	CarSat1	red	na
chrUn	63125788	63126222	435	CarSat1	red	na
chrUn	63126243	63127221	979	CarSat1	red	na
chrUn	63127255	63128690	1436	CarSat1	red	na
chrUn	63128724	63129161	438	CarSat1	red	na
chrUn	63129209	63130160	952	CarSat1	red	na
chrUn	63130197	63130895	699	CarSat1	red	na
chrUn	63130925	63131629	705	CarSat1	red	na
chrUn	63131663	63133094	1432	CarSat1	red	na
chrUn	63133136	63133571	436	CarSat1	red	na
chrUn	63144625	63145502	878	CarSat1	red	na
chrUn	63145741	63147184	1444	CarSat1	red	na
chrUn	63147215	63148803	1589	CarSat1	red	na
chrUn	63148834	63150418	1585	CarSat1	red	na
chrUn	63150449	63151055	607	CarSat1	red	na
chrUn	63180631	63181585	955	CarSat1	red	na

chrUn	63181604	63182394	791	CarSat1	red	na
chrUn	63182544	63184005	1462	CarSat1	red	na
chrUn	63184166	63184871	706	CarSat1	red	na
chrUn	63184892	63185726	835	CarSat1	red	na
chrUn	63185747	63187730	1984	CarSat1	red	na
chrUn	63187780	63188107	328	SAT1_CF	blue	na
chrUn	63188177	63190289	2113	SAT1_CF	blue	na
chrUn	63190302	63190915	614	SAT1_CF	blue	na
chrUn	63190910	63191634	725	CarSat1	red	na
chrUn	63210273	63210856	584	CarSat1	red	na
chrUn	63210883	63211592	710	CarSat1	red	na
chrUn	63211619	63212825	1207	CarSat1	red	na
chrUn	63212841	63215037	2197	CarSat1	red	na
chrUn	63215053	63216014	962	CarSat1	red	na
chrUn	63216041	63217984	1944	CarSat1	red	na
chrUn	63218000	63221669	3670	CarSat1	red	na
chrUn	63221685	63222406	722	CarSat1	red	na
chrUn	63222422	63223142	721	CarSat1	red	na
chrUn	63223158	63223878	721	CarSat1	red	na
chrUn	63223894	63224606	713	CarSat1	red	na
chrUn	63224639	63226592	1954	CarSat1	red	na
chrUn	63227912	63229282	1371	CarSat1	red	na
chrUn	63229476	63229742	267	CarSat1	red	na
chrUn	63231367	63232133	767	CarSat1	red	na
chrUn	63232158	63232710	553	CarSat1	red	na
chrUn	63233128	63237762	4635	CarSat1	red	na
chrUn	63239577	63240527	951	CarSat1	red	na
chrUn	63241850	63242382	533	CarSat1	red	na
chrUn	63242415	63243376	962	CarSat1	red	na
chrUn	63243413	63244330	918	CarSat1	red	na
chrUn	63244356	63245075	720	CarSat1	red	na
chrUn	63245099	63246304	1206	CarSat1	red	na
chrUn	63246582	63248498	1917	CarSat1	red	na
chrUn	63248535	63249338	804	CarSat1	red	na
chrUn	63250362	63252233	1872	CarSat1	red	na
chrUn	63254233	63255512	1280	CarSat1	red	na
chrUn	63255527	63255653	127	Carsat2	orange	na
chrUn	63255542	63256247	706	CarSat1	red	na
chrUn	63256262	63256388	127	Carsat2	orange	na
chrUn	63256277	63256761	485	CarSat1	red	na
chrUn	63257025	63258946	1922	CarSat1	red	na
chrUn	63260724	63262107	1384	CarSat1	red	na

chrUn	63262133	63262547	415	CarSat1	red	na
chrUn	63263945	63264783	839	CarSat1	red	na
chrUn	63264809	63267465	2657	CarSat1	red	na
chrUn	63267495	63268201	707	CarSat1	red	na
chrUn	63268231	63271661	3431	CarSat1	red	na
chrUn	63271695	63272272	578	CarSat1	red	na
chrUn	63297485	63297994	510	SAT1_CF	blue	na
chrUn	63298007	63298409	403	SAT1_CF	blue	na
chrUn	63298469	63299228	760	SAT1_CF	blue	na
chrUn	63301674	63302208	535	SAT1_CF	blue	na
chrUn	63302220	63302328	109	SAT1_CF	blue	na
chrUn	63302349	63302978	630	SAT1_CF	blue	na
chrUn	63302992	63304323	1332	SAT1_CF	blue	na
chrUn	63304367	63304706	340	SAT1_CF	blue	na
chrUn	63304750	63305291	542	SAT1_CF	blue	na
chrUn	63305311	63306148	838	SAT1_CF	blue	na
chrUn	63306161	63306779	619	SAT1_CF	blue	na
chrUn	63306820	63307870	1051	SAT1_CF	blue	na
chrUn	63307882	63309036	1155	SAT1_CF	blue	na
chrUn	63309048	63309713	666	SAT1_CF	blue	na
chrUn	63309756	63310526	771	SAT1_CF	blue	na
chrUn	63312683	63314112	1430	SAT1_CF	blue	na
chrUn	63315037	63315691	655	SAT1_CF	blue	na
chrUn	63315773	63316315	543	SAT1_CF	blue	na
chrUn	63316329	63317329	1001	SAT1_CF	blue	na
chrUn	63317357	63318564	1208	SAT1_CF	blue	na
chrUn	63318775	63318917	143	SAT1_CF	blue	na
chrUn	63318977	63319408	432	SAT1_CF	blue	na
chrUn	63354997	63355323	327	SAT1_CF	blue	na
chrUn	63355336	63355443	108	SAT1_CF	blue	na
chrUn	63355465	63356038	574	SAT1_CF	blue	na
chrUn	63361563	63361727	165	SAT1_CF	blue	na
chrUn	63361906	63362061	156	SAT1_CF	blue	na
chrUn	63362105	63362651	547	SAT1_CF	blue	na
chrUn	63362709	63363976	1268	SAT1_CF	blue	na
chrUn	63364476	63364553	78	SAT1_CF	blue	na
chrUn	63364636	63365156	521	SAT1_CF	blue	na
chrUn	63365187	63365970	784	SAT1_CF	blue	na
chrUn	63371016	63371239	224	SAT1_CF	blue	na
chrUn	63371310	63371941	632	SAT1_CF	blue	na
chrUn	63371959	63373609	1651	SAT1_CF	blue	na
chrUn	63373621	63374987	1367	SAT1_CF	blue	na

chrUn	63375003	63375164	162	SAT1_CF	blue	na
chrUn	63379490	63380182	693	SAT1_CF	blue	na
chrUn	63380195	63382150	1956	SAT1_CF	blue	na
chrUn	63384694	63385815	1122	SAT1_CF	blue	na
chrUn	63387757	63389086	1330	SAT1_CF	blue	na
chrUn	63389098	63389207	110	SAT1_CF	blue	na
chrUn	63389228	63389690	463	SAT1_CF	blue	na
chrUn	63391953	63392568	616	SAT1_CF	blue	na
chrUn	63392581	63393187	607	SAT1_CF	blue	na
chrUn	63432066	63433100	1035	SAT1_CF	blue	na
chrUn	63433121	63433228	108	SAT1_CF	blue	na
chrUn	63433241	63433721	481	SAT1_CF	blue	na
chrUn	63433790	63433962	173	SAT1_CF	blue	na
chrUn	63434143	63434931	789	SAT1_CF	blue	na
chrUn	63434973	63435833	861	SAT1_CF	blue	na
chrUn	63435847	63436528	682	SAT1_CF	blue	na
chrUn	63436540	63437223	684	SAT1_CF	blue	na
chrUn	63437236	63437752	517	SAT1_CF	blue	na
chrUn	63437769	63438142	374	SAT1_CF	blue	na
chrUn	63438188	63439599	1412	SAT1_CF	blue	na
chrUn	63439668	63440511	844	SAT1_CF	blue	na
chrUn	63440523	63441205	683	SAT1_CF	blue	na
chrUn	63441218	63441737	520	SAT1_CF	blue	na
chrUn	63441763	63441918	156	SAT1_CF	blue	na
chrUn	63441933	63444052	2120	SAT1_CF	blue	na
chrUn	63444064	63444766	703	SAT1_CF	blue	na
chrUn	63444779	63445614	836	SAT1_CF	blue	na
chrUn	63445627	63447474	1848	SAT1_CF	blue	na
chrUn	63447496	63448233	738	SAT1_CF	blue	na
chrUn	63449914	63450706	793	CarSat1	red	na
chrUn	63450701	63451319	619	SAT1_CF	blue	na
chrUn	63451332	63452037	706	SAT1_CF	blue	na
chrUn	63452096	63452643	548	SAT1_CF	blue	na
chrUn	63452657	63453358	702	SAT1_CF	blue	na
chrUn	63453390	63454158	769	SAT1_CF	blue	na
chrUn	63466474	63467434	961	CarSat1	red	na
chrUn	63467459	63468171	713	CarSat1	red	na
chrUn	63468196	63468901	706	CarSat1	red	na
chrUn	63468931	63469653	723	CarSat1	red	na
chrUn	63469678	63472076	2399	CarSat1	red	na
chrUn	63472091	63472217	127	Carsat2	orange	na
chrUn	63472106	63472802	697	CarSat1	red	na

chrUn	63472839	63474592	1754	CarSat1	red	na
chrUn	63474656	63475438	783	CarSat1	red	na
chrUn	63475459	63477230	1772	CarSat1	red	na
chrUn	63520156	63520838	683	SAT1_CF	blue	na
chrUn	63520850	63522164	1315	SAT1_CF	blue	na
chrUn	63522215	63522417	203	SAT1_CF	blue	na
chrUn	63524950	63525542	593	SAT1_CF	blue	na
chrUn	63525575	63525842	268	SAT1_CF	blue	na
chrUn	63525922	63526485	564	SAT1_CF	blue	na
chrUn	63526513	63527144	632	SAT1_CF	blue	na
chrUn	63527186	63528048	863	SAT1_CF	blue	na
chrUn	63528060	63528615	556	SAT1_CF	blue	na
chrUn	63528636	63528734	99	SAT1_CF	blue	na
chrUn	63528756	63529333	578	SAT1_CF	blue	na
chrUn	63529393	63529535	143	SAT1_CF	blue	na
chrUn	63529550	63530822	1273	SAT1_CF	blue	na
chrUn	63530884	63531463	580	SAT1_CF	blue	na
chrUn	63531499	63531684	186	SAT1_CF	blue	na
chrUn	63531866	63532030	165	SAT1_CF	blue	na
chrUn	63532110	63532719	610	SAT1_CF	blue	na
chrUn	63532739	63533898	1160	SAT1_CF	blue	na
chrUn	63533910	63535102	1193	SAT1_CF	blue	na
chrUn	63535115	63535826	712	SAT1_CF	blue	na
chrUn	63536135	63537140	1006	SAT1_CF	blue	na
chrUn	63539859	63540043	185	SAT1_CF	blue	na
chrUn	63540056	63540740	685	SAT1_CF	blue	na
chrUn	63540752	63541529	778	SAT1_CF	blue	na
chrUn	63542533	63545621	3089	CarSat1	red	na
chrUn	63545664	63547693	2030	CarSat1	red	na
chrUn	63548217	63550066	1850	CarSat1	red	na
chrUn	63550093	63550803	711	CarSat1	red	na
chrUn	63550830	63552277	1448	CarSat1	red	na
chrUn	63552304	63552866	563	CarSat1	red	na
chrUn	63553893	63556205	2313	CarSat1	red	na
chrUn	63556239	63557435	1197	CarSat1	red	na
chrUn	63557462	63560163	2702	CarSat1	red	na
chrUn	63561172	63561273	102	SAT1_CF	blue	na
chrUn	63561290	63564156	2867	SAT1_CF	blue	na
chrUn	63564191	63565111	921	SAT1_CF	blue	na
chrUn	63565164	63565666	503	SAT1_CF	blue	na
chrUn	63565683	63567032	1350	SAT1_CF	blue	na
chrUn	63567044	63568149	1106	SAT1_CF	blue	na

chrUn	63568142	63568866	725	CarSat1	red	na
chrUn	63568887	63570870	1984	CarSat1	red	na
chrUn	63570861	63572650	1790	SAT1_CF	blue	na
chrUn	63572731	63573993	1263	SAT1_CF	blue	na
chrUn	63574005	63574799	795	SAT1_CF	blue	na
chrUn	63574819	63575342	524	SAT1_CF	blue	na
chrUn	63575354	63575972	619	SAT1_CF	blue	na
chrUn	63575967	63576691	725	CarSat1	red	na
chrUn	63576723	63577133	411	CarSat1	red	na
chrUn	63578275	63580156	1882	CarSat1	red	na
chrUn	63580186	63584062	3877	CarSat1	red	na
chrUn	63584096	63584598	503	CarSat1	red	na
chrUn	63584613	63585321	709	CarSat1	red	na
chrUn	63585351	63586063	713	CarSat1	red	na
chrUn	63586093	63587537	1445	CarSat1	red	na
chrUn	63587567	63588275	709	CarSat1	red	na
chrUn	63588305	63590711	2407	CarSat1	red	na
chrUn	63590744	63591458	715	CarSat1	red	na
chrUn	63591482	63591969	488	CarSat1	red	na
chrUn	63591999	63592707	709	CarSat1	red	na
chrUn	63592737	63593445	709	CarSat1	red	na
chrUn	63593475	63594353	879	CarSat1	red	na
chrUn	63649687	63650570	884	Sat2CF	purple	na
chrUn	63649823	63650559	737	Sat2CF	purple	na
chrUn	63654422	63654966	545	CarSat1	red	na
chrUn	63654996	63657701	2706	CarSat1	red	na
chrUn	63657726	63658439	714	CarSat1	red	na
chrUn	63658455	63659391	937	CarSat1	red	na
chrUn	63659421	63660399	979	CarSat1	red	na
chrUn	63660426	63661387	962	CarSat1	red	na
chrUn	63661403	63662335	933	CarSat1	red	na
chrUn	63662365	63663597	1233	CarSat1	red	na
chrUn	63663622	63664082	461	CarSat1	red	na
chrUn	63664109	63665070	962	CarSat1	red	na
chrUn	63665086	63666545	1460	CarSat1	red	na
chrUn	63666561	63667029	469	CarSat1	red	na
chrUn	63667056	63668017	962	CarSat1	red	na
chrUn	63668033	63669707	1675	CarSat1	red	na
chrUn	63669737	63670444	708	CarSat1	red	na
chrUn	63689413	63690482	1070	CarSat1	red	na
chrUn	63690512	63691446	935	CarSat1	red	na
chrUn	63691470	63699338	7869	CarSat1	red	na

chrUn	63699353	63699493	141	CarSat1	red	na
chrUn	63699678	63702471	2794	CarSat1	red	na
chrUn	63702501	63705421	2921	CarSat1	red	na
chrUn	63726509	63736932	10424	Carsat2	orange	na
chrUn	63737450	63742896	5447	Carsat2	orange	na
chrUn	63760894	63763557	2664	CarSat1	red	na
chrUn	63763581	63764295	715	CarSat1	red	na
chrUn	63764319	63765037	719	CarSat1	red	na
chrUn	63765061	63766499	1439	CarSat1	red	na
chrUn	63766532	63767984	1453	CarSat1	red	na
chrUn	63768008	63768948	941	CarSat1	red	na
chrUn	63768984	63770929	1946	CarSat1	red	na
chrUn	63770953	63773875	2923	CarSat1	red	na
chrUn	63773899	63774617	719	CarSat1	red	na
chrUn	63774641	63776085	1445	CarSat1	red	na
chrUn	63776118	63776870	753	CarSat1	red	na
chrUn	63792366	63792793	428	SAT1_CF	blue	na
chrUn	63792840	63793834	995	SAT1_CF	blue	na
chrUn	63816253	63817133	881	CarSat1	red	na
chrUn	63817231	63818084	854	CarSat1	red	na
chrUn	63818273	63819051	779	CarSat1	red	na
chrUn	63819234	63819318	85	CarSat1	red	na
chrUn	63819335	63819642	308	CarSat1	red	na
chrUn	63819793	63820441	649	CarSat1	red	na
chrUn	63820459	63820932	474	CarSat1	red	na
chrUn	63820949	63822389	1441	CarSat1	red	na
chrUn	63822389	63824058	1670	SAT1_CF	blue	na
chrUn	63824078	63829338	5261	SAT1_CF	blue	na
chrUn	63829333	63830055	723	CarSat1	red	na
chrUn	63830208	63830449	242	CarSat1	red	na
chrUn	63859012	63859892	881	CarSat1	red	na
chrUn	63859913	63860512	600	CarSat1	red	na
chrUn	63860710	63861345	636	CarSat1	red	na
chrUn	63861370	63862071	702	CarSat1	red	na
chrUn	63864675	63865955	1281	CarSat1	red	na
chrUn	63866291	63869201	2911	CarSat1	red	na
chrUn	63869228	63869696	469	CarSat1	red	na
chrUn	63869712	63870673	962	CarSat1	red	na
chrUn	63870700	63873022	2323	CarSat1	red	na
chrUn	63875711	63876232	522	CarSat1	red	na
chrUn	63876257	63877826	1570	CarSat1	red	na
chrUn	63878955	63880134	1180	CarSat1	red	na

chrUn	63882799	63884215	1417	CarSat1	red	na
chrUn	63885218	63885957	740	SAT1_CF	blue	na
chrUn	63886001	63887588	1588	SAT1_CF	blue	na
chrUn	63887668	63889037	1370	SAT1_CF	blue	na
chrUn	63889050	63889799	750	SAT1_CF	blue	na
chrUn	63889811	63890413	603	SAT1_CF	blue	na
chrUn	63890424	63891506	1083	SAT1_CF	blue	na
chrUn	63891551	63891708	158	SAT1_CF	blue	na
chrUn	63891723	63892762	1040	SAT1_CF	blue	na
chrUn	63892775	63893399	625	SAT1_CF	blue	na
chrUn	63893441	63894601	1161	SAT1_CF	blue	na
chrUn	63894614	63895542	929	SAT1_CF	blue	na
chrUn	63895554	63896225	672	SAT1_CF	blue	na
chrUn	63896249	63897447	1199	SAT1_CF	blue	na
chrUn	63897460	63899070	1611	SAT1_CF	blue	na
chrUn	63899384	63900249	866	SAT1_CF	blue	na
chrUn	63904279	63904937	659	SAT1_CF	blue	na
chrUn	63904981	63905391	411	SAT1_CF	blue	na
chrUn	63906395	63907204	810	SAT1_CF	blue	na
chrUn	63907247	63907787	541	SAT1_CF	blue	na
chrUn	63907814	63907949	136	SAT1_CF	blue	na
chrUn	63907977	63908540	564	SAT1_CF	blue	na
chrUn	63908641	63908878	238	SAT1_CF	blue	na
chrUn	63908916	63909456	541	SAT1_CF	blue	na
chrUn	63909474	63909537	64	SAT1_CF	blue	na
chrUn	63910129	63910597	469	SAT1_CF	blue	na
chrUn	63910677	63911225	549	SAT1_CF	blue	na
chrUn	63911236	63911619	384	SAT1_CF	blue	na
chrUn	63914214	63914743	530	SAT1_CF	blue	na
chrUn	63914755	63915428	674	SAT1_CF	blue	na
chrUn	63915471	63915997	527	SAT1_CF	blue	na
chrUn	63916018	63916307	290	SAT1_CF	blue	na
chrUn	63916597	63917230	634	SAT1_CF	blue	na
chrUn	63917289	63917394	106	SAT1_CF	blue	na
chrUn	63917441	63917960	520	SAT1_CF	blue	na
chrUn	63917987	63918247	261	SAT1_CF	blue	na
chrUn	63918317	63920217	1901	SAT1_CF	blue	na
chrUn	63920261	63921594	1334	SAT1_CF	blue	na
chrUn	63921681	63922182	502	SAT1_CF	blue	na
chrUn	63922194	63922303	110	SAT1_CF	blue	na
chrUn	63922324	63923213	890	SAT1_CF	blue	na
chrUn	63924446	63925096	651	SAT1_CF	blue	na

chrUn	63925121	63925459	339	SAT1_CF	blue	na
chrUn	63927958	63928423	466	SAT1_CF	blue	na
chrUn	63928451	63929116	666	SAT1_CF	blue	na
chrUn	63929130	63929370	241	SAT1_CF	blue	na
chrUn	63930384	63931553	1170	CarSat1	red	na
chrUn	63931569	63933029	1461	CarSat1	red	na
chrUn	63933054	63933767	714	CarSat1	red	na
chrUn	63933792	63935721	1930	CarSat1	red	na
chrUn	63935748	63936460	713	CarSat1	red	na
chrUn	63936487	63937449	963	CarSat1	red	na
chrUn	63937474	63938670	1197	CarSat1	red	na
chrUn	63938697	63939658	962	CarSat1	red	na
chrUn	63939674	63940395	722	CarSat1	red	na
chrUn	63940411	63941618	1208	CarSat1	red	na
chrUn	63941645	63942606	962	CarSat1	red	na
chrUn	63942622	63945303	2682	CarSat1	red	na
chrUn	63945330	63946207	878	CarSat1	red	na
chrUn	63957543	63957884	342	Sat19CF	na	na
chrUn	63970694	63971584	891	CarSat1	red	na
chrUn	63971805	63973251	1447	CarSat1	red	na
chrUn	63973272	63974054	783	CarSat1	red	na
chrUn	63974119	63975026	908	CarSat1	red	na
chrUn	63984134	63985726	1593	CarSat1	red	na
chrUn	63985757	63987359	1603	CarSat1	red	na
chrUn	63987380	63988982	1603	CarSat1	red	na
chrUn	63989003	63990444	1442	CarSat1	red	na
chrUn	63990603	63991560	958	CarSat1	red	na
chrUn	63999663	63999752	90	SAT1_CF	blue	na
chrUn	64000206	64000260	55	SAT1_CF	blue	na
chrUn	64048556	64049583	1028	CarSat1	red	na
chrUn	64051297	64053444	2148	CarSat1	red	na
chrUn	64054908	64057002	2095	CarSat1	red	na
chrUn	64058344	64059489	1146	CarSat1	red	na
chrUn	64059519	64060979	1461	CarSat1	red	na
chrUn	64060994	64063173	2180	CarSat1	red	na
chrUn	64063405	64064833	1429	CarSat1	red	na
chrUn	64065039	64066044	1006	CarSat1	red	na
chrUn	64067081	64068487	1407	CarSat1	red	na
chrUn	64070403	64071250	848	CarSat1	red	na
chrUn	64071271	64072104	834	CarSat1	red	na
chrUn	64092108	64092621	514	CarSat1	red	na
chrUn	64092642	64093359	718	CarSat1	red	na

chrUn	64093380	64094052	673	CarSat1	red	na
chrUn	64100852	64101356	505	CarSat1	red	na
chrUn	64101371	64101497	127	Carsat2	orange	na
chrUn	64101386	64102747	1362	CarSat1	red	na
chrUn	64102835	64103634	800	CarSat1	red	na
chrUn	64103660	64104363	704	CarSat1	red	na
chrUn	64104387	64106058	1672	CarSat1	red	na
chrUn	64106088	64106795	708	CarSat1	red	na
chrUn	64106825	64108044	1220	CarSat1	red	na
chrUn	64109152	64110333	1182	CarSat1	red	na
chrUn	64110987	64111833	847	CarSat1	red	na
chrUn	64119167	64120744	1578	CarSat1	red	na
chrUn	64120954	64121087	134	CarSat1	red	na
chrUn	64122536	64123804	1269	CarSat1	red	na
chrUn	64131301	64132711	1411	CarSat1	red	na
chrUn	64147758	64148933	1176	Sat2CF	purple	na
chrUn	64157426	64158291	866	Sat2CF	purple	na
chrUn	64158168	64158317	150	Sat2CF	purple	na
chrUn	64174201	64175297	1097	CarSat1	red	na
chrUn	64175312	64175439	128	Carsat2	orange	na
chrUn	64175327	64176558	1232	CarSat1	red	na
chrUn	64176583	64177295	713	CarSat1	red	na
chrUn	64177320	64178882	1563	CarSat1	red	na
chrUn	64178897	64179023	127	Carsat2	orange	na
chrUn	64178912	64179619	708	CarSat1	red	na
chrUn	64179634	64179761	128	Carsat2	orange	na
chrUn	64179649	64180355	707	CarSat1	red	na
chrUn	64180385	64181091	707	CarSat1	red	na
chrUn	64181106	64181232	127	Carsat2	orange	na
chrUn	64181121	64181828	708	CarSat1	red	na
chrUn	64181843	64181966	124	Carsat2	orange	na
chrUn	64181858	64184559	2702	CarSat1	red	na
chrUn	64184592	64185295	704	CarSat1	red	na
chrUn	64185328	64186048	721	CarSat1	red	na
chrUn	64186060	64186787	728	CarSat1	red	na
chrUn	64186820	64187744	925	CarSat1	red	na
chrUn	64187759	64187886	128	Carsat2	orange	na
chrUn	64187774	64188996	1223	CarSat1	red	na
chrUn	64189021	64189850	830	CarSat1	red	na
chrUn	64200278	64200559	282	Sat15CF	na	na
chrUn	64200278	64200559	282	Sat15CF	na	na
chrUn	64200691	64201013	323	Sat15CF	na	na

chrUn	64211940	64212689	750	CarSat1	red	na
chrUn	64212719	64213653	935	CarSat1	red	na
chrUn	64213669	64215367	1699	CarSat1	red	na
chrUn	64215394	64216105	712	CarSat1	red	na
chrUn	64216130	64216600	471	CarSat1	red	na
chrUn	64216616	64217847	1232	CarSat1	red	na
chrUn	64217877	64219053	1177	CarSat1	red	na
chrUn	64219080	64220530	1451	CarSat1	red	na
chrUn	64220557	64222288	1732	CarSat1	red	na
chrUn	64222303	64223747	1445	CarSat1	red	na
chrUn	64223777	64224485	709	CarSat1	red	na
chrUn	64224515	64226667	2153	CarSat1	red	na
chrUn	64226697	64227575	879	CarSat1	red	na
chrUn	64228612	64232844	4233	CarSat1	red	na
chrUn	64232871	64234569	1699	CarSat1	red	na
chrUn	64234585	64236778	2194	CarSat1	red	na
chrUn	64236794	64237333	540	CarSat1	red	na
chrUn	64238540	64240712	2173	CarSat1	red	na
chrUn	64240734	64240862	129	Carsat2	orange	na
chrUn	64240750	64242463	1714	CarSat1	red	na
chrUn	64242490	64243199	710	CarSat1	red	na
chrUn	64243226	64243935	710	CarSat1	red	na
chrUn	64243962	64244922	961	CarSat1	red	na
chrUn	64262784	64263763	980	CarSat1	red	na
chrUn	64263785	64264753	969	CarSat1	red	na
chrUn	64264769	64266226	1458	CarSat1	red	na
chrUn	64266242	64266711	470	CarSat1	red	na
chrUn	64266740	64267695	956	CarSat1	red	na
chrUn	64267720	64268422	703	CarSat1	red	na
chrUn	64268444	64269904	1461	CarSat1	red	na
chrUn	64269920	64271377	1458	CarSat1	red	na
chrUn	64271393	64272329	937	CarSat1	red	na
chrUn	64272359	64275063	2705	CarSat1	red	na
chrUn	64275079	64275798	720	CarSat1	red	na
chrUn	64275814	64277754	1941	CarSat1	red	na
chrUn	64277781	64278366	586	CarSat1	red	na
chrUn	64279397	64280077	681	CarSat1	red	na
chrUn	64280107	64281559	1453	CarSat1	red	na
chrUn	64281574	64281689	116	Carsat2	orange	na
chrUn	64281589	64282556	968	CarSat1	red	na
chrUn	64282594	64283311	718	CarSat1	red	na
chrUn	64283322	64286002	2681	CarSat1	red	na

chrUn	64286032	64288728	2697	CarSat1	red	na
chrUn	64288761	64291904	3144	CarSat1	red	na
chrUn	64291919	64292045	127	Carsat2	orange	na
chrUn	64291934	64293379	1446	CarSat1	red	na
chrUn	64293394	64293520	127	Carsat2	orange	na
chrUn	64293409	64294122	714	CarSat1	red	na
chrUn	64294137	64294260	124	Carsat2	orange	na
chrUn	64294153	64294948	796	CarSat1	red	na
chrUn	64295980	64296943	964	CarSat1	red	na
chrUn	64296970	64297439	470	CarSat1	red	na
chrUn	64297455	64298679	1225	CarSat1	red	na
chrUn	64298711	64299885	1175	CarSat1	red	na
chrUn	64299912	64300623	712	CarSat1	red	na
chrUn	64300650	64301110	461	CarSat1	red	na
chrUn	64301135	64302838	1704	CarSat1	red	na
chrUn	64302865	64304072	1208	CarSat1	red	na
chrUn	64304088	64305048	961	CarSat1	red	na
chrUn	64305075	64309225	4151	CarSat1	red	na
chrUn	64309241	64311516	2276	CarSat1	red	na
chrUn	64338664	64340310	1647	CarSat1	red	na
chrUn	64340331	64341047	717	CarSat1	red	na
chrUn	64341074	64341772	699	CarSat1	red	na
chrUn	64341799	64344236	2438	CarSat1	red	na
chrUn	64344261	64344973	713	CarSat1	red	na
chrUn	64344989	64345711	723	CarSat1	red	na
chrUn	64345736	64347183	1448	CarSat1	red	na
chrUn	64347199	64347920	722	CarSat1	red	na
chrUn	64347936	64348642	707	CarSat1	red	na
chrUn	64348673	64349602	930	CarSat1	red	na
chrUn	64349623	64349750	128	Carsat2	orange	na
chrUn	64349638	64351605	1968	CarSat1	red	na
chrUn	64351630	64352090	461	CarSat1	red	na
chrUn	64352117	64354211	2095	CarSat1	red	na
chrUn	64361654	64362761	1108	Sat7CF	lime	na
chrUn	64388724	64390619	1896	CarSat1	red	na
chrUn	64390640	64391355	716	CarSat1	red	na
chrUn	64391376	64392336	961	CarSat1	red	na
chrUn	64392361	64393072	712	CarSat1	red	na
chrUn	64393097	64393817	721	CarSat1	red	na
chrUn	64393842	64394761	920	CarSat1	red	na
chrUn	64394798	64395282	485	CarSat1	red	na
chrUn	64395307	64397252	1946	CarSat1	red	na

chrUn	64397279	64404243	6965	CarSat1	red	na
chrUn	64440295	64440648	354	SAT1_CF	blue	na
chrUn	64440690	64441373	684	SAT1_CF	blue	na
chrUn	64441386	64442321	936	SAT1_CF	blue	na
chrUn	64442342	64443725	1384	SAT1_CF	blue	na
chrUn	64443764	64444067	304	SAT1_CF	blue	na
chrUn	64444079	64445519	1441	SAT1_CF	blue	na
chrUn	64445562	64446421	860	SAT1_CF	blue	na
chrUn	64446433	64447037	605	SAT1_CF	blue	na
chrUn	64447050	64447551	502	SAT1_CF	blue	na
chrUn	64448060	64449134	1075	SAT1_CF	blue	na
chrUn	64449214	64449356	143	SAT1_CF	blue	na
chrUn	64449384	64449893	510	SAT1_CF	blue	na
chrUn	64449936	64450506	571	SAT1_CF	blue	na
chrUn	64450522	64451058	537	SAT1_CF	blue	na
chrUn	64451102	64451776	675	SAT1_CF	blue	na
chrUn	64451788	64453073	1286	SAT1_CF	blue	na
chrUn	64453096	64453640	545	SAT1_CF	blue	na
chrUn	64453690	64455168	1479	SAT1_CF	blue	na
chrUn	64455180	64455941	762	SAT1_CF	blue	na
chrUn	64455954	64456178	225	SAT1_CF	blue	na
chrUn	64462589	64464181	1593	CarSat1	red	na
chrUn	64464202	64465795	1594	CarSat1	red	na
chrUn	64465816	64466721	906	CarSat1	red	na
chrUn	64466785	64467568	784	CarSat1	red	na
[truncated: 452,730 more chars]
